# Mixed heavy metal stress induces global iron starvation response

**DOI:** 10.1038/s41396-022-01351-3

**Published:** 2022-12-26

**Authors:** Jennifer L. Goff, Yan Chen, Michael P. Thorgersen, Linh T. Hoang, Farris L. Poole, Elizabeth G. Szink, Gary Siuzdak, Christopher J. Petzold, Michael W. W. Adams

**Affiliations:** 1 Department of Biochemistry and Molecular Biology, University of Georgia, Athens, GA USA; 2 Biological Systems and Engineering, Lawrence Berkeley National Laboratory, Berkeley, CA USA; 3 Scripps Center for Metabolomics, Scripps Research, La Jolla, CA USA

**Keywords:** Soil microbiology, Bacterial genomics

## Abstract

Multiple heavy metal contamination is an increasingly common global problem. Heavy metals have the potential to disrupt microbially mediated biogeochemical cycling. However, systems-level studies on the effects of combinations of heavy metals on bacteria are lacking. For this study, we focused on the Oak Ridge Reservation (ORR; Oak Ridge, TN, USA) subsurface which is contaminated with several heavy metals and high concentrations of nitrate. Using a native *Bacillus cereus* isolate that represents a dominant species at this site, we assessed the combined impact of eight metal contaminants, all at site-relevant concentrations, on cell processes through an integrated multi-omics approach that included discovery proteomics, targeted metabolomics, and targeted gene-expression profiling. The combination of eight metals impacted cell physiology in a manner that could not have been predicted from summing phenotypic responses to the individual metals. Exposure to the metal mixture elicited a global iron starvation response not observed during individual metal exposures. This disruption of iron homeostasis resulted in decreased activity of the iron-cofactor-containing nitrate and nitrite reductases, both of which are important in biological nitrate removal at the site. We propose that the combinatorial effects of simultaneous exposure to multiple heavy metals is an underappreciated yet significant form of cell stress in the environment with the potential to disrupt global nutrient cycles and to impede bioremediation efforts at mixed waste sites. Our work underscores the need to shift from single- to multi-metal studies for assessing and predicting the impacts of complex contaminants on microbial systems.

## Introduction

Beginning in the 20th century, increasing levels of heavy metal contamination occurred in both aquatic [1, 2] and terrestrial [3, 4] environments due to greater anthropogenic inputs from urbanization, industrial processes, and agricultural activities. Regardless of specific input source, a common theme that emerges at contaminated sites is the simultaneous presence of multiple heavy metals at elevated concentrations [2, 5–8]. A recent meta-analysis by Zhou et al. [2] compiled heavy metal concentrations for global surface water bodies and compared these values to the limits set by WHO and the US EPA. From 1972 to 2017, heavy metal pollution in surface waters shifted from single metals to multiple metals.

Heavy metal pollution is not only detrimental to human [9], animal [10, 11], and plant health [12], but also disrupts the natural cycling of elements via impacts on microbial activity. Aponte et al. [13] found that individual heavy metal contaminants linearly decreased the activities of key soil microbial enzymes, particularly those involved in carbon and sulfur cycling. In soil systems, individual heavy metal contaminants inhibit multiple steps of the denitrification pathways, resulting in accumulation of toxic intermediates, including nitrite and the greenhouse gas nitrous oxide [14–16]. However, studies investigating the impacts of environmentally relevant combinations of heavy metals on environmental microorganisms are scarce. A single report by Dey et al. [17] explored the proteomic response of the eukaryotic microorganism *Aspergillus fumigatus* to multi-metal stress and suggested a role for protein turnover pathways and antioxidant proteins in this response. However, this study did not examine if this response was due to synergistic or additive interactive effects of the metals in the mixture. In prokaryotic systems, the limited studies on this form of stress have exclusively focused on determinations of IC_50_ values for binary combinations of metals [18–24]. For example, Fulladosa et al. [18] explored the synergistic/antagonistic toxicity of binary metal pairings in *Vibrio fisheri* by determining IC_50_ values for individual metals and binary combinations of metals. They found that Co-Cu and Zn-Pb pairings and Co-Cd, Cd-Zn, Cd-Pb, and Cu-Pb pairs were synergistic and antagonistic in their impacts on *V. fisheri* growth, respectively. [18]. However, the mechanism for these interactive effects remains unknown, and the impact of multiple metal exposure on bacterial gene regulation and metabolism is still unexplored.

**Fig. 1:**
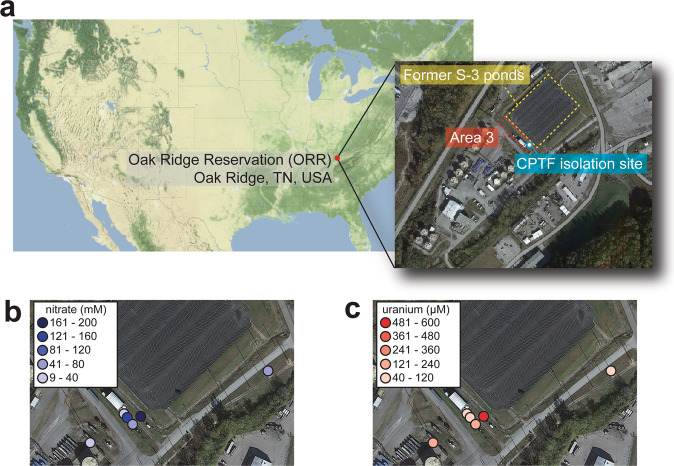
Map and geochemical parameters of the contaminated Oak Ridge Reservation (ORR) study site. **a** Map showing the location of the ORR in the United States (US). The US map was generated using the *get_stamenmap* function in the *ggmap* R package implemented in RStudio [88]. The map insert shows the study site: the subsurface regions immediately adjacent to the former S-3 ponds (indicated by yellow, dashed box). Area 3 is marked with a red, dashed box. The CPTF isolation site described in Goff et al. (2022) is also marked. Distribution of (**b**) nitrate (mM) and (**c**) uranium (µM) in the Area 3 groundwater and in groundwater samples taken from two sites in close vicinity to Area 3 and the former S-3 ponds. All satellite maps were generated in Google My Maps. Imagery: ©2022 Maxar Technologies. Map data: ©2022 US Geological Survey.

The subsurface of the US Department of Energy (DOE) Oak Ridge Reservation (ORR) in Oak Ridge, Tennessee is contaminated with nitric acid and multiple heavy metals, making it ideal for investigating the impacts of multi-metal contamination on native microbial communities [25]. The contamination at this site is the result of liquid waste discharge from uranium processing operations at the Y-12 National Security Complex into unlined waste ponds (referred to as the S-3 ponds) from 1951 until 1983 [26]. The subsurface of the region immediately adjacent to the former S-3 ponds (Fig. 1a) is contaminated with high levels of uranium (U) and nitrate (Fig. 1b,c), as well as elevated concentrations of other metals, such as nickel (Ni), cadmium (Cd), copper (Cu), aluminum (Al), manganese (Mn), and iron (Fe) (Fig. S1) [26, 27]. As nitrate is a major co-contaminant, the impact of these metal contaminants on nitrogen cycling by microorganisms at the site is a significant point of concern [28, 29]. Previously, we isolated *Bacillus cereus* strain CPT56D-587-MTF (referred to as strain CPTF) from the subsurface sediments of the area immediately adjacent to the former S-3 ponds, referred to as Area 3 (Fig. 1a). Strain CPTF carries out dissimilatory nitrate reduction to ammonium (DNRA) via NarGHI and NasDE [30] and is a representative isolate of a highly abundant amplicon sequence variant (ASV) found in the subsurface sediments of Area 3. In a recent study, this CPTF-matching ASV had the highest relative abundance across all Area 3 samples, up to 10–40% of total reads in several samples, suggesting that strain CPTF represents a dominant species in this region [30]. Thus, this isolate is ideal for detailed studies of physiological responses to site-relevant stressors.

We assessed the impact of a mixture of eight major metal contaminants of the ORR subsurface (Al, U, Mn, Fe, Cd, Cu, Co, and Ni) on *B. cereus* strain CPTF. We compared the growth of strain CPTF in the presence of individual metals to that when metals are added in combination. Using a high-throughput mass spectrometry (MS)-based proteomics approach, we compared the system-level responses of cells exposed to the metal mixture to those exposed to individual metals. We further validated and expanded upon these results with a combination of targeted MS-based metabolomics and targeted gene expression profiling. Finally, to explore the potential impact on nitrogen cycling at ORR, we examined the effects of the metal mixture on nitrate and nitrite reduction by strain CPTF.

## Materials and methods

### Media and culture conditions

*Bacillus cereus* str. CPT56D-587-MTF (referred to as strain CPTF) [30, 31] was routinely streaked out on LB plates and grown overnight at 30 °C. A single isolated colony was inoculated into LB broth and grown overnight shaking at 200 rpm at 30 °C. The overnight culture was diluted 100-fold into an anoxic defined medium supplemented with nitrate (named *B. cereus* experimental medium or BCE medium) for further experimentation (Supporting Information). For metals exposure, a contaminated ORR environmental metal mix (COMM) [32] was used that contains: 500 µM AlK(SO_4_)_2_·12H_2_O, 50 µM UO_2_(CH_3_COO)_2_, 50 µM MnCl_2_·4H_2_O, 50 µM NiSO_4_·6H_2_O, 15 µM CoCl_2_·6 H_2_O, 5 µM (NH_4_)_2_Fe(SO_4_)_2_·6H_2_O, 5 µM CuCl_2_·2H_2_O, and 2.5 µM Cd(CH_3_COO)_2_·2H_2_O. The same metal salts and concentrations were used for the individual metal treatments.

### Growth curves

Strain CPTF was grown in 10 mL BCE medium amended with COMM or the individual metal concentrations described above. Growth was determined by taking optical density measurements at 600 nm (OD600) on a spectrophotometer.

### Proteomic analysis

Strain CPTF was grown in triplicate 50 mL cultures in anoxic BCE medium amended with COMM or the individual metal concentrations described above. Control cultures had no amendments. Following 10 h of growth at 30 °C, samples were collected for proteomic analysis. Protein was extracted, and tryptic peptides were prepared by following established proteomic sample preparation procedures. Peptides were analyzed using an Agilent 1290 UHPLC system (Santa Clara, CA) coupled to a Thermo Scientific Obitrap Exploris 480 mass spectrometer (Waltham, MA) [33]. The software suite DIA-NN (data-independent acquisition with neural networks) was used for peptide identification and quantification (Supporting Information) [34]. Briefly, 20 µg of trypsin-digested peptides were injected onto Agilent InfinityLab Poroshell 120 EC-C18 (2.1 mm ID, 100 mm length, 1.9 µm particle size, 120 Å pore size) column operating at 60 °C. The peptides were eluted from the column by using a 10 min gradient from 98% buffer A (99.9% H2O, 0.1% formic acid) and 2% buffer B (99.9% acetonitrile, 0.1% formic acid) to 65% buffer A and 35% buffer B. The mass spectrometer is operated in data independent mode with a duty cycle of 3 MS1 survey scans and 45 MS2 scans. For data analysis, the raw data was analyzed by searching against a FASTA sequence database (library-free mode) containing the proteins from the genome of interest [31, 35] and common proteomic contaminants. DIA-NN automatically generates a set of decoy precursors out of the sequence database for negative controls and subsequently utilizes deep neural networks to obtain q-values to distinguish between target and decoy precursors [34]. It reports integrated ion chromatographic peak area of identified precursors as the quantitative peptide abundance values. Recently, DIA-NN was benchmarked against five other DIA workflows (Spectronaut, Skyline, DIA-Umpire, ScaffoldDIA) on complex samples [36]. Quantitative matrices on the protein level were extracted from the main DIA-NN reports and processed by an automated python script described in an established protocol [37]. For quantitative analysis, the peptide abundance values were summed to a protein abundance. Next, the protein abundances of the sample replicates were averaged, and the standard deviation and percent coefficient of variation were calculated. Missing values imputation was achieved by replacing missing values with a quantitative value of 1000, a value ~90% of the lowest value in the dataset (LLOD). For comparative analysis, adjusted *p* values were calculated by Welch’s *t*-test with Benjamini–Hochberg adjustment and adjusted *p* values lower than 0.05 were considered statistically significant.

### Metabolomic analysis

Quantitative MS-based metabolomics was used to validate proteomic observations of differentially regulated metabolic pathways. Strain CPTF was grown in 200 mL BCE medium (*n* = 5 replicates) amended with COMM. Control cultures had no further amendments to the medium. Following 10 h of growth at 30 °C, the cells were sampled for analysis. Intracellular metabolites were quantified using an Agilent 6495 triple quadrupole mass spectrometer with a jet stream source, coupled to an Agilent 1290 UPLC stack (Supporting Information). MS transition states for the target compounds are given in Table S1. Data were processed using Agilent Quantitative software. Limits of quantification are given in Table S2.

### Iron uptake

Strain CPTF was grown in triplicate in 500 mL BCE medium. Control cultures had no further amendments to the medium. A second set of control cultures was amended with 5 µM (NH_4_)_2_Fe(SO_4_)_2_·6H_2_O—the same amount of iron (Fe) present in the COMM. COMM-treated cultures were amended with metals as described above. Prior to inoculation, a medium sample was collected from each culture bottle for determination of initial Fe concentrations. Cultures were then grown for 10 h at 30 °C and sampled for analysis. Inductively coupled plasma/mass spectroscopy analysis was performed using an Agilent 7900 single quadruple mass spectrometer to quantify the total iron content of the uninoculated culture medium, spent medium, and whole cell extracts (Supporting Information).

### Enzyme activity assays

For both nitrate and nitrite reductase activity assays, strain CPTF was grown in 500 mL of BCE medium with or without COMM addition at 30 °C. After 10 h of growth, cells were harvested by centrifugation at 6600 × *g* at 4 °C for 15 min. Cells were washed once in pre-chilled 50 mM potassium phosphate buffer (pH 7.0). Nitrate and nitrite reductase assays were performed using a modified version of the procedure described by Thorgersen and Adams [38] (Supporting Information).

### Quantitative reverse transcriptase PCR (qRT-PCR)

Strain CPTF was grown in triplicate 50 mL cultures in BCE medium. One set of cultures was left untreated as a control. One set was treated with the COMM described above. Following 10 h of growth at 30 °C, RNA was extracted, and cDNA was prepared (Supporting Information). Quantitative qRT-PCR was performed with the Brilliant II SYBR Green QPCR Master Mix (Agilent). Primers were designed to amplify ~150 bp product within the target genes (Table S3). The *recA* gene (UIJ64731.1) was used as the reference gene. Statistical comparisons were performed using a Student’s *t* test.

## Results and discussion

### Growth of strain CPTF with a synthetic mixture of metals mimicking a contaminated site

**Fig. 2:**
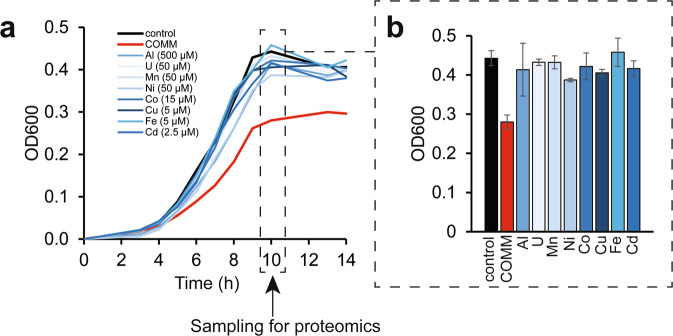
Growth of strain CPTF with field-relevant concentrations of individual metals or COMM. **a** Growth assessment was performed under anoxic nitrate-respiring conditions with glucose (20 mM) as the carbon source. The added concentrations of the individual metals are indicated in the legend. The COMM is a mixture of all the metals at the same concentrations as they are present individually (Table S4). Each time point represents an average of three replicates. For clarity, error bars are not shown but can be viewed in Table S5. The dashed box indicates the 10 h time point where samples were collected for the proteomic analysis described later in the manuscript. **b** Growth at the 10 h time point. Error bars represent ±SD. Data are the average of three replicates.

In the contaminated ORR subsurface, multiple metals co-exist at elevated concentrations along with high levels of nitrate [26]. To explore the toxicity of these metals, we exposed the ORR isolate *B. cereus* strain CPTF to acontaminated ORR environmental metal mix (COMM) containing eight metals, Al^3+^ (500 µM), U^6+^ (50 µM), Mn^2+^ (50 µM), Fe^2+^ (5 µM), Cd^2+^ (2.5 µM), Co^2+^ (15 µM), Cu^2+^ (5 µM), and Ni^2+^ (50 µM), where the concentrations reflect those typically found in contaminated ORR groundwater (Fig. 1, Fig. S1, Table S4). Growth experiments were performed under anoxic, nitrate-respiring conditions. Compared to the control, strain CPTF cultures grown with COMM had a slower growth rate and lower growth yield (Fig. 2a). To determine if the individual metals also inhibit growth, the cells were grown with the individual COMM components at their COMM concentrations. For all the above-mentioned metal exposed cultures, we compared growth at the transition point between exponential and stationary phase (10 h) (Fig. 2b). There was no significant difference (*p* > 0.05) between control culture growth and growth during Al, U, Mn, Co, Fe, or Cd. However, growth with Ni and Cu was 87 ± 3.9% and 92 ± 4.4% of the control, respectively (*p* < 0.05). We considered that the toxicity of the COMM may be the result of the additive or synergistic effects of the mildly toxic Ni and Cu. However, the growth defect of CPTF grown with Ni and Cu at their COMM concentrations was not to the extent of that observed in the presence of COMM (Fig. S2). These data suggest that the combined toxicity of the COMM metals is greater than what would be predicted from the sum of the individual parts.

### MS-based proteomics analysis of metals-treated CPTF cultures: global response

**Fig. 3:**
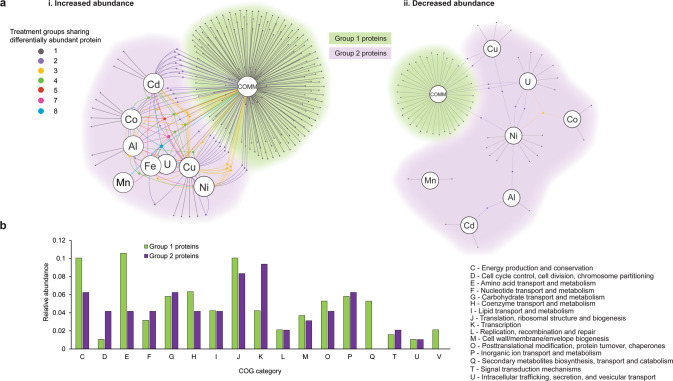
Comparison of the global response of the strain CPTF proteome between metals mix and individual metal treatments. **a** Network diagrams of differentially abundant proteins at 10 h of growth under anoxic nitrate-respiring conditions (*n* = 3 replicates). Large nodes represent the metal treatment condition. Small nodes represent individual differentially abundant proteins (relative to the untreated control). Edges indicate conditions under which the proteins are differentially abundant. Network maps display proteins that were increased (i) or (ii) decreased in abundance in response to metal treatments. Small node/edge colors (legend is shown on the image) represent the number of treatment groups sharing the differentially abundant proteins. Background cloud colors distinguish the Group 1 proteins (green: only differentially abundant in the COMM treatment) from the Group 2 proteins (purple: all other proteins). **b** Functional comparisons of Group 1 and Group 2 proteins using COG categories. Category S (unknown function) is excluded from the visualization for clarity. All other categories were not represented in the differentially abundant proteomes.

We further examined the interactions of the COMM metals within the cellular system of strain CPTF using a proteomics-based approach. We compared the proteomic response of cells grown with COMM to cells grown with the individual metal treatments. Triplicate CPTF cultures were grown with either the COMM or individual metals at the same concentrations that they are present at in the COMM. Control cultures had no added metals other than those in the standard medium. All cultures were grown under anoxic, nitrate-respiring conditions. Samples were collected for MS-based proteomics analysis at 10 h (Fig. 2). Across all ten conditions a total of 1303 unique proteins were identified (Table S6). Differentially abundant proteins in metal-treated cultures were identified through comparison to control cultures without metal exposure. In sum, 295 differentially abundant proteins (*p* < 0.05) were identified across the nine metal treatments (COMM and eight individual metals; Fig. 3a, Table S7). Of these 295 proteins, 191 (65%, termed Group 1) were differentially abundant only in the COMM-treated cultures. The remainder (35%, Group 2) were differentially abundant in the COMM and one or more individual metal treatments or only in the individual metal treatments.

### Enzymes of competing siderophore and tryptophan biosynthesis pathways are differentially abundant only during COMM exposure

A total of 276 out of the 295 differentially abundant proteins were assigned to Clusters of Orthologous Genes (COG) categories [39] (Fig. 3b). Functional comparison of Group 1 to Group 2 proteins revealed a ~2.5-fold enrichment of COG Category E (amino acid metabolism and transport) among the Group 1 proteins. A more detailed analysis revealed that a number of these Group 1 Category E proteins were involved in biosynthesis of the essential amino acid tryptophan. In addition, none of the Group 2 proteins belonged to Category Q (secondary metabolites biosynthesis, transport, and catabolism). In contrast, ten (5%) of the Group 1 proteins fell into COG Category Q. These included several involved in the biosynthesis of the siderophore bacillibactin. Bacillibactin is a tripeptide catecholate-type siderophore secreted by members of the genus *Bacillus* for chelation of ferric iron (Fe^3+^) from the environment during periods of iron limitation [40]. These two observations are interesting as the tryptophan and bacillibactin biosynthetic pathways of strain CPTF are predicted to share chorismite as a common intermediate [41, 42]. Chorismate is the branch point in the biosynthesis of aromatic amino acids and other aromatic metabolites [43].

**Fig. 4:**
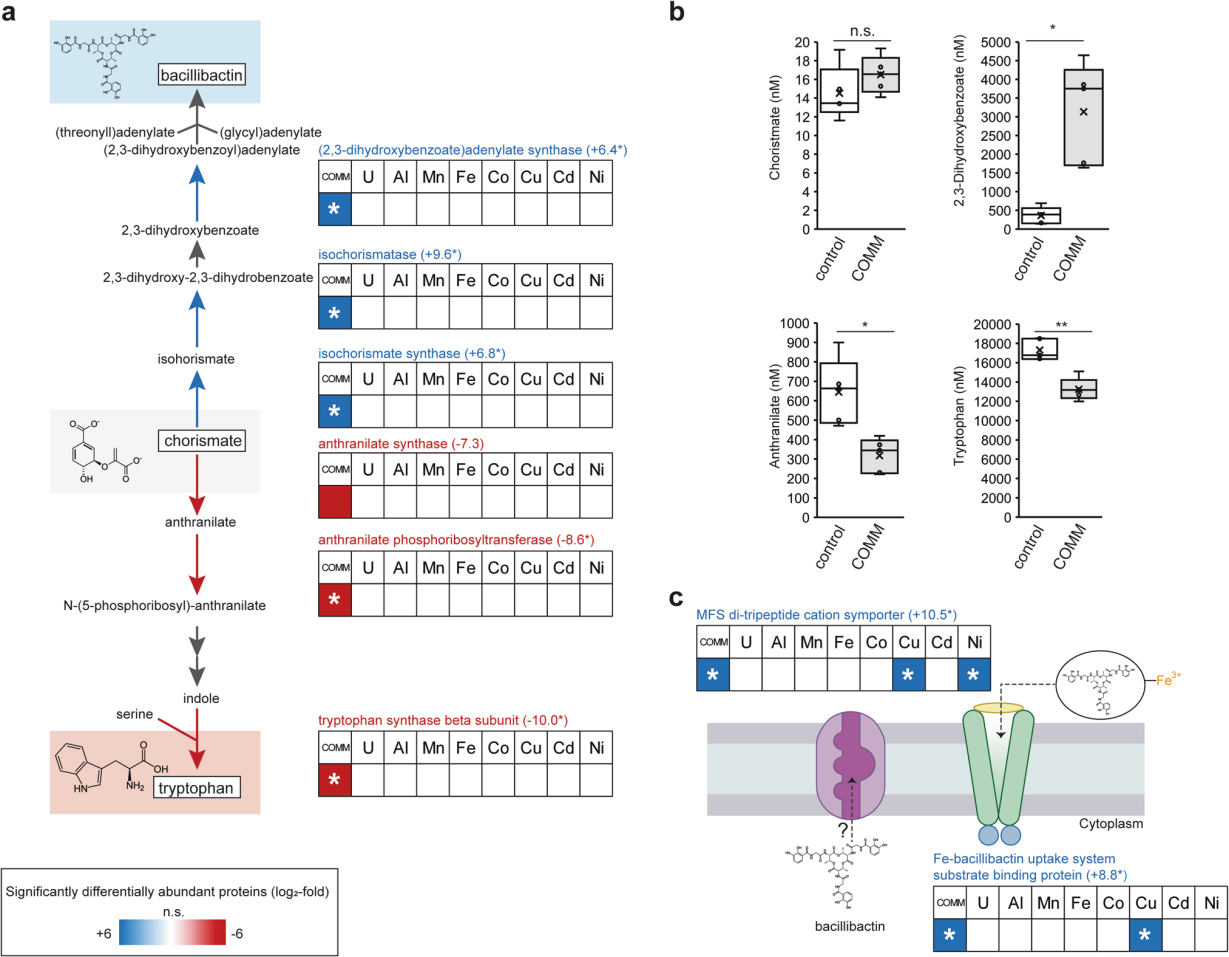
COMM exposure dysregulates bacillibactin and tryptophan biosynthetic pathways. For all panels, asterisks indicate imputed log_2_-fold changes due to the non-detectability of the peptide under the compared conditions. This fold-change was imputed from a quantitative value of 1000, a value ~90% of the lowest value in the dataset (LLOD). **a** Patterns of protein abundance across the enzymes of the bacillibactin and tryptophan biosynthetic pathway. Heat maps display average (*n* = 3 replicates) log_2_-fold abundance changes of individual proteins across the different treatment conditions relative to the control (left-to-right: COMM, U, Al, Mn, Fe, Co, Cu, Cd, Ni). Numbers in parentheses to the right of protein names indicate the specific log_2_-fold change of the protein in the COMM-exposed cultures. The heat map scale (displayed at log_2_-fold abundance changes relative to the control) is at the bottom of the image. Blue boxes indicate increased protein abundance (*p* < 0.05), red boxes indicate proteins that were significantly decreased in abundance (*p* < 0.05) and white boxes indicate no significant difference in abundance relative to the control. Pathway proteins lacking a heat map had no significant change in abundance patterns in any of the tested conditions. **b** Plots of intracellular concentrations of select bacillibactin/tryptophan biosynthetic pathway metabolites (*n* = 5 replicates). Center lines represent median values. Interquartile ranges and maximum/minimum values are represented by box ranges and whisker ranges, respectively. Mean values are indicated with an “x”. Internal points are marked with dots. * *p* < 0.01, ** *p* < 0.001. **c** Differential protein abundance patterns of putative bacillibactin transporters. Heat map details are the same as (**a**).

Further analysis of specific protein abundance patterns revealed that the bacillibactin biosynthesis enzymes isochorismate synthase (DhbC), 2,3-dihydrozybenzoate dehydrogenase (DhbB), and (2,3-dihydrozybenzoate)adenylate synthase (DhbE) were only detected in COMM-exposed cultures treatment but not in control or individual metal exposure cultures (Fig. 4a). Where commercial standards were available, we performed targeted MS-based analysis of intracellular metabolites to confirm changes in metabolic pathway flux suggested by the proteomics data. DhbC (ON, imputed +6.8 log_2_FC) catalyzes the conversion of chorismate to isochorismate. Likely due to its role as a branch point intermediate, chorismate concentrations were low in all samples and no difference was observed in its concentrations between control and COMM cultures (Fig. 4b). Isochorismate is then converted to 2,3-dihydroxy-2,3-dihydroxybenzoate by DhbB (ON, imputed +9.6 log_2_FC) and then to 2,3-dihydroxybenzoate by an enzyme that was not differentially abundant. We found that intracellular 2,3-dihydroxybenzoate levels increased 8.7-fold in response to COMM exposure (Fig. 4b), likely due to greater metabolic flux through the pathway. In bacteria such as *Bacillus subtilis*, 2,3-dihydroxybenzoate is secreted as a siderophore in addition to its role as a biosynthetic intermediate [44, 45]. Adenylation of 2,3-dihydrozybenzoate to (2,3-dihydrozybenzoate)adenylate is catalyzed by DhbE (ON, imputed +6.4 log_2_FC). The final step is the formation of the tripeptide bacillibactin mediated by a non-ribosomal peptide synthase not differentially abundant in our dataset.

In contrast to the bacillibactin biosynthetic pathway, the abundance of three enzymes in the tryptophan biosynthetic pathway were significantly decreased in response to COMM treatment (Fig. 4a), although there was also no change in their abundance with any of the individual metal treatments. These included anthranilate synthase (TrpE, −7.3 log_2_FC), which catalyzes chorismate conversion to anthranilate. Supporting this observation, intracellular anthranilate concentrations decreased 2.0-fold in response to COMM exposure (Fig. 4b). Anthranilate phosphoribosyltransferase (TrpD), which catalyzes conversion of anthranilate to N-(5-phosphoribosyl)-anthranilate, was detectable under all conditions except for COMM exposure (OFF, imputed −8.6 log_2_FC). Similarly, the tryptophan synthase beta subunit was detectable under all conditions except for COMM exposure (TrpB, OFF, imputed −10.0 log_2_FC). TrpB catalyzes the conversion of the downstream intermediate indole to tryptophan. Consistent with the decreased levels of TrpB and lower metabolic flux through the pathway, intracellular tryptophan concentrations decreased 1.3-fold during COMM exposure (Fig. 4).

We also detected of two putative siderophore transporters during COMM exposure that were not detectable under control conditions. However, we note that these transporters were part of the Group 2 proteins as they were also detected during some individual metal treatments (Fig. 4c). The substrate-binding subunit (FeuA, ON, imputed +8.8 log_2_FC) of the FeuABC bacillibactin-Fe^3+^ ATP-type transporter [46] was significantly increased during COMM exposure. In addition, while there is no homolog of a characterized bacillibactin exporter [47] in the strain CPTF genome, we detected (ON, imputed +10.5 log_2_FC) a member of a major facilitator superfamily (MFS) type di/tripeptide cation symporter during growth with COMM that was not detectable under control conditions. This transporter was also detected during Cu and Ni exposure (Fig. 4c). The previously characterized bacillibactin transporter of *B. subtilis* (YmfE) is also of the MFS-type [47]. Further supporting this proposed function, the gene encoding this transporter (LW858_01095) is located immediately adjacent to a second ABC-type siderophore-Fe^3+^ transport system (Fig. S3) and is downstream of a metal-responsive ArsR-type transcriptional regulator [48].

Our proteomic and metabolomic data suggest that increased bacillibactin and/or 2,3-dihydrozybenzoate production, export and re-import occurs during COMM exposure of *B. cereus* str. CPTF. However, the biosynthesis and membrane transport of siderophores are energetically costly processes [49, 50]. In addition, iron uptake is typically tightly regulated by bacterial cells to prevent mismetallation of non-iron-containing metalloproteins [51] and oxidative damage triggered by excessive intracellular iron [52]. In *B. subtilis* str. CU1065 and *B. cereus* str. 569, the genes encoding siderophore biosynthetic enzymes and transporters are regulated by a canonical ferric uptake regulator (FUR) that represses its regulon under iron-replete conditions [53, 54]. During periods of iron starvation, the FUR regulon is de-repressed. We hypothesize that COMM exposure induces a global iron starvation response involving increased siderophore biosynthesis and increased abundance of siderophore transporters. In addition, we suggest that chorismate is shunted from tryptophan to bacillibactin biosynthesis for prioritization of siderophore-mediated iron acquisition. We note, though, that such an iron acquisition/starvation response is puzzling in this system as the preferred source of iron for microorganisms, ferrous iron (Fe^2+^), is a component of the COMM.

### COMM exposure results in a canonical global iron starvation response

We examined the proteomic data and conducted targeted gene expression analyses to determine if the alterations observed in the bacillibactin and tryptophan pathways were indicative of a more global iron starvation response to COMM exposure, suggestive of a physiological need for iron acquisition (Table 1). For these analyses, we compared our data to prior studies of cellular responses to iron limitation conditions. We confirmed the presence of a FUR homolog in the strain CPTF genome with 83% sequence identity (full length) to *B. subtilis* FUR. In *B. subtilis*, as well as other model microorganisms, this FUR-regulated response includes: (1) increased expression of transporters for iron uptake (e.g., siderophore-Fe^3+^ transporters) and iron scavenging proteins, (2) increased siderophore biosynthesis, and (3) an iron-sparing response including increased expression of flavodoxins (Flds) [53, 55]. We have presented above evidence for increased iron transport in strain CPTF. Related to iron-scavenging, we note the increased abundance of two heme monooxygenases (HmoA and HmoB) exclusively during COMM exposure (Table 1). HmoA and B catalyze heme porphyrin ring opening to release free Fe^2+^ [56]. Heme monooxygenases are produced by *Bacillus* and *Staphylococcus* species during periods of iron limitation [57–59].

**Table 1:** Changes in expression levels of proteins proposed to comprise the strain CPTF global iron starvation response.

Protein (GenBank ID)	Log_2_-fold expression change during metal exposure^a^									Reference organism
	COMM	U	Al	Mn	Fe	Co	Cu	Cd	Ni	
*(i) Siderophore biosynthesis and transport*										
Isochorismate synthase DhbC (UIJ68570.1)	+6.8^b^	n.s.	n.s.	n.s.	n.s.	n.s.	n.s.	n.s.	n.s.	*Bacillus subtilis* [53]
Isochorismatase DhbB (UIJ68572.1)	+9.6^b^	n.s.	n.s.	n.s.	n.s.	n.s.	n.s.	n.s.	n.s.	*Bacillus subtilis* [53]
2,3-dihydroxybenzoate adenylate synthase DhbE (UIJ68571.1)	+6.4^b^	n.s.	n.s.	n.s.	n.s.	n.s.	n.s.	n.s.	n.s.	*Bacillus subtilis* [53]
MFS di-tripeptide cation symporter (UIJ66957.1)	+8.8^b^	n.s.	n.s.	n.s.	n.s.	n.s.	+7.6^b^	n.s.	+6.8^b^	This work
Fe-bacillibactin uptake system substrate-binding protein FeuA (UIJ66957.1)	+ 10.5^b^	n.s.	n.s.	n.s.	n.s.	n.s.	+5.9^b^	n.s.	n.s.	*Bacillus subtilis* [53]
*(ii) Aromatic amino acid biosynthesis*										
Tryptophan synthase subunit beta TrpB (UIJ67623.1)	−10.9^b^	n.s.	n.s.	n.s.	n.s.	n.s.	n.s.	n.s.	n.s.	This work
Anthranilate phosphoribosyltransferase TrpD (UIJ67620.1)	−8.6^b^	n.s.	n.s.	n.s.	n.s.	n.s.	n.s.	n.s.	n.s.	This work
Anthranilate synthase TrpE (UIJ67618.1)	−7.3	n.s.	n.s.	n.s.	n.s.	n.s.	n.s.	n.s.	n.s.	This work
Prephenate dehydrogenase TyrA (UIJ69055.1)	−1.1	n.s.	n.s.	n.s.	n.s.	n.s.	n.s.	n.s.	n.s.	This work
*(iii) Iron scavenging*										
Heme monooxygenase A (UIJ69081.1)	+4.6	n.s.	n.s.	n.s.	n.s.	n.s.	n.s.	n.s.	n.s.	*Bacillus subtilis* [56]
Heme monooxygenase B (UIJ67406.1)	+1.6	n.s.	n.s.	n.s.	n.s.	n.s.	n.s.	n.s.	n.s.	*Bacillus anthracis* str. Sterne [56]
*(iv) Iron sparing*										
Flavodoxin Fld (UIJ64511.1)	+8.3^b^	n.s.	n.s.	n.s.	n.s.	n.s.	n.s.	n.s.	n.s.	*Bacillus subtilis* [53]
Ferredoxin-dependent assimilatory sulfite reductase Sir (CysI) (UIJ67790.1)	−2.3	n.s.	n.s.	n.s.	n.s.	n.s.	n.s.	n.s.	n.s.	*Pseudomonas aeruginosa* [64]
Glutamate synthase GltB (UIJ66883.1)	−1.0	n.s.	n.s.	n.s.	n.s.	n.s.	n.s.	n.s.	n.s.	*Bacillus subtilis* [63]
Nitrate reductase subunit beta NarH, (UIJ68393.1)	−2.1	n.s.	n.s.	n.s.	n.s.	n.s.	n.s.	n.s.	−1.1	*Escherichia coli* [61]
*(v) Sulfur assimilation*										
Sulfate adenylyltransferase CysN (UIJ67788.1)	−1.2	n.s.	n.s.	n.s.	n.s.	n.s.	n.s.	n.s.	n.s.	*Bacillus subtilis* [62]
Adenylyl-sulfate kinase CysC (UIJ67789.1	−1.4	n.s.	n.s.	n.s.	n.s.	n.s.	n.s.	n.s.	n.s.	*Bacillus subtilis* [62]
Fmnh2-dependent alkanesulfonate monooxygenase SsuD (UIJ69025.1)	−6.7^b^	n.s.	n.s.	n.s.	n.s.	n.s.	n.s.	n.s.	n.s.	*Pseudomonas aeruginosa* [64]

^a^Only significant values (*p* < 0.05) are reported. Values are the average of three replicates.

^b^Imputed log2-fold changes due to the non-detectability of the peptide under the compared conditions. This fold-change was imputed from a quantitative value of 1000, a value ~90% of the lowest value in the dataset (LLOD).

In response to COMM exposure, we detected increased abundance of enzymes involved in bacillibactin biosynthesis, consistent with what has been observed with other *Bacillus* species during periods of iron starvation [53, 54]. We further suggest that the parallel decreases in abundance of the enzymes in the tryptophan biosynthetic pathway are to conserve the intermediate chorismate for siderophore biosynthesis. In support of this model, we observed decreased abundance of the prephenate dehydrogenase (TyrA) exclusively during COMM exposure (Table 1). TyrA catalyzes the oxidative decarboxylation of prephenate to 4-hydroxyphenylpyruvate in the tyrosine biosynthetic pathway which also utilizes chorismate as an intermediate [60]. We were unable to find any reported instance of tryptophan or tyrosine biosynthesis repression in the global iron starvation response of other bacterial species. This novel finding highlights the strength of discovery proteomics coupled with detailed pathway analysis for uncovering non-obvious cellular responses to environmental stress. Future studies should determine how this response is regulated and if it occurs in other bacterial systems.

To adjust to iron-limited conditions, bacteria typically have an iron-sparing response for minimizing the synthesis of abundant, non-essential, iron-bearing enzymes and enzymes involved in iron cofactor biosynthesis [61]. The iron-sparing response of *B. subtilis* is under post-transcriptional regulatory control of the sRNA FsrA (analogous to RhyB in gram-negative bacteria) and three small, basic proteins (FbpA, FbpB, and FbpC) [62]. This response includes decreased expression of iron-containing enzymes of the TCA cycle, cytochrome and porphyrin biogenesis enzymes, cysteine biosynthesis enzymes, iron-containing enzymes of the isoleucine biosynthetic pathways, and iron-containing glutamate synthase [62, 63]. During COMM exposure, we similarly observed decreased abundance of the CPTF sulfate adenylyltransferase (CysN) and the adenylyl-sulfate kinase (CysC) as well as the ferredoxin-dependent sulfite reductase (Sir/CysI) (Table 1). We speculate that the decreased abundance of the non-iron containing enzymes of the cysteine biosynthetic pathway is due to the role of cysteine as the sulfur donor in iron-sulfur cluster biosynthesis [64]. We also observed decreased glutamate synthase (GltB) levels during COMM exposure (Table 1). Finally, we note decreased abundance of the NarH subunit of the respiratory iron-sulfur cluster-containing nitrate reductase. Prior studies with *B. subtilis* during iron-limitation were conducted under oxic conditions when respiratory nitrate reductase is not produced. However, decreased levels of this enzyme have been shown in *Escherichia coli* during iron limitation under anoxic conditions [65].

Connected to the iron-sparing response, the protein Fld is up-regulated across all three domains of life during periods of iron limitation [66–68]. Flavodoxin is a small electron transfer protein that contains a flavin mononucleotide cofactor. During low-iron conditions, organisms will increase the production of Fld which can replace the iron-sulfur containing ferredoxin as the physiological electron donor for various oxidoreductase reactions [66, 68]. Accordingly, we observed increased production of Fld in the COMM-exposed proteome of strain CPTF relative to the control (Table 1). Levels of Fld were not altered by any of the individual metal treatments.

We used qRT-PCR to validate representative protein abundance changes observed during COMM exposure. We selected 11 transcripts to represent the various parts of the proposed global iron starvation response of strain CPTF. Of these 11 transcripts, seven (*dhbB, dhbC, dhbE, mfs, sir, fld*, and *hmoA*) were consistent with abundance patterns of their respective protein products during COMM exposure (Fig. S4). However, four transcripts (*feuA, trpB, trpD*, and *trpE*) were not differentially expressed despite large changes in their protein product abundances observed under the same conditions. The discrepancy between the *feuA* transcript expression levels (n.s.) and FeuA protein levels (+8.8-log_2_FC) is likely the result of temporal changes in gene expression during the transition into stationary phase that begins at our sampling time-point (10 h) and subsequent lag in protein-level changes [69]. In contrast, TrpBDE protein levels decreased during COMM exposure while its transcript abundance is unchanged, suggesting post-transcriptional or post-translational regulation of this peptide product. These results emphasize the utility of combined proteomic and transcriptomic approaches.

While increased abundance of proteins involved in siderophore biosynthesis and transport has been observed previously during individual metal exposure experiments [70, 71], there are no reports of an iron-starvation response occurring to the extent observed here with strain CPTF during COMM exposure. In addition, these prior studies have either utilized high heavy metal concentrations that are not relevant even to contaminated environments, or the heavy metal exposure is performed under iron-deficient conditions [72]. For example, in *B. subtilis*, copper exposure (500 µM) induces expression of bacillibactin biosynthetic enzymes as well as the bacillibactin-Fe^3+^ transporter. However, no expression changes were observed for the heme monooxygenases or Fld. Furthermore, several iron-containing enzymes were actually up-regulated during copper exposure and there was no evidence of an iron-sparing response [73]. Thus, exposure of strain CPTF to COMM appears to induce a physiological state of iron starvation more comparable to that induced by iron chelators or what is observed in Δ*fur* mutant strains [53].

### Proposed mechanism for iron starvation response in COMM-exposed cells

Soluble iron concentrations are elevated in the contaminated ORR groundwater ([Fe]_AVG_ = 5.6 µM) relative to non-contaminated ORR groundwater ([Fe]_AVG_ = 0.35 µM) [29, 32]. In our experiments, soluble [Fe] was 3.7 and 5.3 µM Fe in the control and COMM-amended cultures, respectively. The COMM iron is added in the ferrous (Fe^2+^) form. Due to the insolubility of Fe^3+^ at neutral pH, this total concentration of soluble iron in the medium reflects [Fe^2+^]. Yet, we observed that COMM exposure induces a physiological state of iron starvation that is not observed in the individual metal treatments or the control cultures. We considered that COMM components may compete with Fe^2+^ for transmembrane transport, limiting Fe^2+^ uptake. However, compared to control cultures, COMM-exposed cultures over-imported iron with total intracellular iron concentrations of 22.0 and 151.1 µM, respectively (Fig. S5A). As an additional control, we amended CPTF cultures with 5 µM ferrous iron (the same as that present in the COMM) and measured intracellular iron concentrations. While these cultures do take up about 50% more iron than the unamended control (33.6 vs. 22.0 µM, respectively), these values are still less than that measured for the COMM-exposed cultures. Following incubation and cell growth, [Fe]_aq_ was decreased but still >1 µM in all spent media samples (Table S8).

Individual metals within the COMM may also disrupt intracellular iron homeostasis by displacing iron in the metal-binding sites of enzymes, a process known as mismetallation, which is a known mechanism of toxicity for many heavy metals [74]. We propose that individual metals in the COMM may target different stages of the iron cofactor assembly and insertion processes for different enzymes. During individual metal exposure, the cells appear to manage this stress by up-regulating proteins for iron cofactor repair and biosynthesis with minimal or no growth defect (Fig. 2). For example, we observed increased abundance of iron-sulfur cluster repair and assembly proteins (IscA and Ric) as well as heme biosynthesis proteins (HemFH) during exposure of strain CPTF to Cd and Cu (Fig. S5B). However, in combination, that stress response is seemingly overwhelmed, perhaps as multiple metals target a broader range of iron-bearing enzymes, resulting in significant disruption of intracellular iron homeostasis. The levels of two heme biosynthesis proteins (HemFH) are increased during COMM exposure relative to the control while the iron-sulfur cluster repair/assembly proteins are not, suggesting that iron may be prioritized for heme biosynthesis under these conditions. Increased rates of iron cofactor synthesis required to overcome the displacement of iron by other metals could lead to a significant shift in the intracellular Fe^2+^ equilibrium, leading to FUR de-repression.

### Potential impacts of COMM-disrupted iron homeostasis on nitrogen cycling

**Fig. 5:**
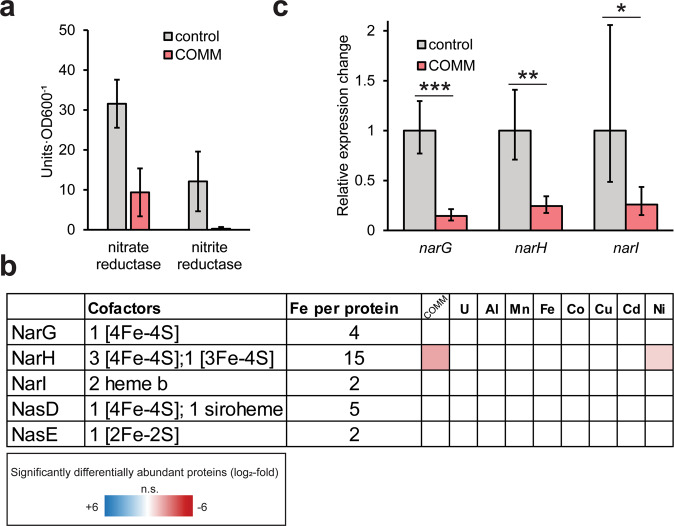
COMM treatment impacts nitrogen oxide reduction activity. **a** Activity of nitrate and nitrite reductases with (red bars) or without (gray bars) COMM. One unit of activity represents 1 nmol nitrate/nitrate reduced per minute. Experiments were performed in triplicate and error bars represent SD. **b** NarGHI and NasDE differential abundance. The heat map displays average (*n* = 3 replicates) log_2_-fold abundance changes of individual proteins across the different treatment conditions relative to the control (left-to-right: COMM, U, Al, Mn, Fe, Co, Cu, Cd, and Ni). The heat map scale (displayed at log_2_-fold abundance changes relative to the control) is at the bottom of the image. The number of Fe atoms per protein was determined from their *Bacillus subtilis* (UP000001570) homologs (**c**) Relative changes in expression of *narG, narH, and narI* with (red bars) and without (gray bars) COMM exposure. Experiments were performed in triplicate and error bars represent ±SD. * *p* < 0.05, ** *p* < 0.01, *** *p* < 0.001.

The nitrate-respiring conditions used for our experiments mimic the contaminated ORR subsurface due to the high concentrations of anthropogenic nitrate. However, nitrogen cycling at the site may be influenced by heavy metal co-contaminants. Nitrite accumulation has been measured in the porewaters of contaminated ORR sediment cores, suggestive of inhibition of nitrite reduction in situ [75]. Strain CPTF carries out DNRA via the NarGHI respiratory nitrate reductase and NasDE nitrite reductase [30]. Both NarGHI and NasDE contain iron cofactors [76, 77]. Thus, we sought to determine if the dysregulation of iron homeostasis in strain CPTF induced by COMM impacts the activity of these two enzymes. We found that the activity of the NasDE nitrite reductase was near-absent (0.25 ± 0.44 units·OD600^−1^) in COMM-exposed CPTF cultures relative to the control cultures (12.11 ± 7.48 units·OD600^−1^) (Fig. 5a). However, levels of both protein subunits were unchanged between control and COMM-exposed cultures, (Fig. 5b), suggesting that the loss of activity is due to direct inhibition of the enzyme by the metals. We ruled out direct inhibition of the nitrite reductase holoenzyme by either allosteric inhibition or displacement of metal centers (Table S9). Instead, we propose that the COMM metals are mis-incorporated during iron cofactor biosynthesis, resulting in mismetallation of the nitrite reductase apoenzyme which yields an inactive nitrite reductase.

**Fig. 6:**
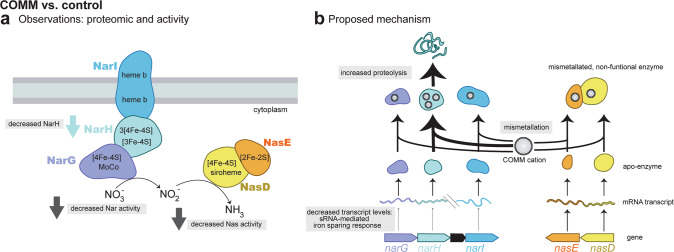
Model for decreased nitrate/nitrite reductase activity during COMM exposure. **a** Observed changes in enzyme levels and functions in COMM-exposed CPTF cells relative to the control. **b** Proposed mechanisms for changes described in (**a**).

We also observed decreased nitrate reductase activity in the COMM-exposed cultures relative to the control (9.38 ± 6.00 v. 31.57 ± 6.03 units·OD600^−1^) (Fig. 5a). As noted above, COMM exposure resulted in decreased levels of the NarH subunit of the nitrate reductase compared to the control (Fig. 5b), suggesting that the decrease in activity is, in part, due to lower protein levels. However, no changes were observed in NarG and NarI abundance (Fig. 5b). This difference in relative abundance changes between the three subunits is puzzling as all three are present in the same operon and should be co-regulated. Indeed, gene expression analysis confirmed the decreased expression of *narH* as well as *narI* and *narG* (Fig. 5c). This discrepancy between the protein-level and transcript-level fold-changes is likely due to different post-translational controls on the three protein products. Decreased *narGHI* expression is likely part of the iron-sparing response described above. At a later time point, all three protein subunits would likely have decreased in the COMM-exposed cultures relative to the control. However, at the time point where all measurements were conducted, NarH levels may have already been subject to post-translational regulation, possibly via enhanced proteolytic degradation. Mismetallation of metal cofactor centers can result in misfolding, targeting proteins for cleavage by proteases [78]. NarH contains a greater number of iron atoms than either NarG or NarI: NarH contains a predicted 15 iron atoms per protein molecule compared to 4 and 2, respectively (Fig. 5b). We speculate that this may make NarH more vulnerable to mismetallation and subsequent turnover by proteases than the other subunits. We observed increased abundance of two CPTF proteases exclusively during COMM treatment: Clp and Hls. Similar to the nitrite reductase, we excluded the possibility of holoenzyme inhibition either allosterically or through displacement of metal centers by the COMM metals (Table S9). An integrated model for the impacts of COMM on nitrate and nitrite reduction is presented in Fig. 6. Our data suggest that the inhibition of microbial nitrite and nitrate reduction frequently observed at heavy metal contaminated sites [15, 79] may occur at multiple regulatory levels.

## Ecological implications

Environmental stressors select for microbial community functions that facilitate resilience within that ecological niche. There is significant enrichment for genes involved in nitrate respiration and heavy metal tolerance in metagenomes originating from contaminated ORR groundwater [80, 81]. Our data suggest that multiple metal stress induces a physiological state of iron starvation even in iron-replete environments like the contaminated ORR subsurface. Mining of published ORR metagenome data [80] revealed a significant overabundance of the high-affinity ferrous iron transporter gene *efeU* in a contaminated groundwater metagenome relative to a metagenome from a pristine background well. EfeU is important for iron acquisition under iron-depleted conditions [82]. Thus, microbial communities in the contaminated regions of ORR may experience this induced iron-starvation state, selecting for community members capable of coping with this stress. Like the ORR, sites impacted by industrial and agricultural activities are frequently co-contaminated with nitrate and multiple heavy metals [83–85]. Examining the interplay between these co-contaminants is essential for site management and remediation efforts. While nitrate reduction can be desirable in such sites, accumulation of intermediates like nitrite and nitrous oxide may compound existing problems [25]. For example, nitrite can inhibit bioremediation processes due to the high toxicity of nitrite to microorganisms even at low millimolar concentrations. In addition, at uranium-contaminated sites like the ORR, nitrite accumulation can lead to the oxidative re-mobilization of uraninite to UO_2_^2+^, counteracting bioremediation efforts. The inhibition of enzymatic activity due to the multiple metal-induced disruption of iron homeostasis could lead to the accumulation of these undesirable intermediates.

Our findings call into question the transferability of single metal toxicity studies to contaminated environments. In these environments, metals rarely exist as singular entities. Yet, studies attempting to use laboratory experimentation to constrain biological activity in these environments typically handle co-existing metal contaminants as independent parameters [86], with minimal consideration given to their interdependent, combinatorial effects. The construction of biological regulatory and metabolic models for contaminated environments may have limited predictive power if exclusively derived from single metal perturbation experiments. While single metal exposure studies have been instrumental for developing a detailed mechanistic understanding of heavy metal toxicity in microbial systems, we propose that it is time for a shift to multi-metal perturbation studies to better reflect the stressors microorganisms face in anthropogenically-disturbed environments.

## Supplementary information

Tables S1-S9 and Figures S1-S5

Table S6

Table S7

## Data Availability

The generated mass spectrometry proteomics data have been deposited to the ProteomeXchange Consortium via the PRIDE [87] partner repository with the dataset identifier PXD035730. DIA-NN is freely available for download from https://github.com/vdemichev/DiaNN.
