# Supplementary material for: Mixed heavy metal stress induces global iron starvation response
Source: ISME J. 2022 Dec 26;17(3):382–92. doi: 10.1038/s41396-022-01351-3 (PMC9938188; doi:10.1038/s41396-022-01351-3)
Supplement: Supplementary file 2 — Table S6 [file 41396_2022_1351_MOESM2_ESM.pdf]

GenBank	Protein Annotation (NCBI)	Sample	Counts (mean)	Counts (SD)	CV%
UJJ64229.1	SMI1/KNR4_family_protein_[Bacillus_cereus]	CPTF_Al	899524.6667	17883.5373	1.988109716
UJJ64229.1	SMI1/KNR4_family_protein_[Bacillus_cereus]	CPTF_Cd	890235	55576.76125	6.242931501
UJJ64229.1	SMI1/KNR4_family_protein_[Bacillus_cereus]	CPTF_Co	937271	81179.59406	8.661272359
UJJ64229.1	SMI1/KNR4_family_protein_[Bacillus_cereus]	CPTF_Cu	942984.6667	90831.30399	9.632320355
UJJ64229.1	SMI1/KNR4_family_protein_[Bacillus_cereus]	CPTF_Fe	957257.9667	81659.93698	8.530609284
UJJ64229.1	SMI1/KNR4_family_protein_[Bacillus_cereus]	CPTF_Mn	832944	126024.3302	15.12998835
UJJ64229.1	SMI1/KNR4_family_protein_[Bacillus_cereus]	CPTF_Ni	940350.3333	97071.51397	10.32290951
UJJ64229.1	SMI1/KNR4_family_protein_[Bacillus_cereus]	CPTF_U	803177.6667	297527.4208	37.0437866
UJJ64229.1	SMI1/KNR4_family_protein_[Bacillus_cereus]	CPTF_metals_mix	1107099	182904.9306	16.5210998
UJJ64229.1	SMI1/KNR4_family_protein_[Bacillus_cereus]	CPTF_zcontrol	951413.3333	53065.9328	5.577589776
UJJ64230.1	HNH_endonuclease_[Bacillus_cereus]	CPTF_Al	330031.1333	31374.26281	9.506455496
UJJ64230.1	HNH_endonuclease_[Bacillus_cereus]	CPTF_Cd	53502.66667	92669.33701	173.2050808
UJJ64230.1	HNH_endonuclease_[Bacillus_cereus]	CPTF_Co	243520.9667	225297.7952	92.51679571
UJJ64230.1	HNH_endonuclease_[Bacillus_cereus]	CPTF_Cu	255097.0667	185803.8362	72.83652402
UJJ64230.1	HNH_endonuclease_[Bacillus_cereus]	CPTF_Fe	314353.7	126924.9182	40.3764671
UJJ64230.1	HNH_endonuclease_[Bacillus_cereus]	CPTF_Mn	140404.6667	122818.2544	87.47448168
UJJ64230.1	HNH_endonuclease_[Bacillus_cereus]	CPTF_Ni	287154.7333	98128.29738	34.17262054
UJJ64230.1	HNH_endonuclease_[Bacillus_cereus]	CPTF_U	206738.1333	168100.6731	81.31091754
UJJ64230.1	HNH_endonuclease_[Bacillus_cereus]	CPTF_metals_mix	15839.6	14204.06825	89.67441257
UJJ64230.1	HNH_endonuclease_[Bacillus_cereus]	CPTF_zcontrol	379976.7667	76308.82592	20.08249783
UJJ64234.1	hypothetical_protein_LW858_14490_[Bacillus_cereus]	CPTF_Al	50901.5	48734.30521	95.7423754
UJJ64234.1	hypothetical_protein_LW858_14490_[Bacillus_cereus]	CPTF_Cd	0	0	0
UJJ64234.1	hypothetical_protein_LW858_14490_[Bacillus_cereus]	CPTF_Co	16585.16667	28726.35132	173.2050808
UJJ64234.1	hypothetical_protein_LW858_14490_[Bacillus_cereus]	CPTF_Cu	0	0	0
UJJ64234.1	hypothetical_protein_LW858_14490_[Bacillus_cereus]	CPTF_Fe	45219.03333	39516.22565	87.38847944
UJJ64234.1	hypothetical_protein_LW858_14490_[Bacillus_cereus]	CPTF_Mn	0	0	0
UJJ64234.1	hypothetical_protein_LW858_14490_[Bacillus_cereus]	CPTF_Ni	0	0	0
UJJ64234.1	hypothetical_protein_LW858_14490_[Bacillus_cereus]	CPTF_U	0	0	0
UJJ64234.1	hypothetical_protein_LW858_14490_[Bacillus_cereus]	CPTF_metals_mix	0	0	0
UJJ64234.1	hypothetical_protein_LW858_14490_[Bacillus_cereus]	CPTF_zcontrol	0	0	0
UJJ64240.1	alpha/beta_hydrolase_[Bacillus_cereus]	CPTF_Al	1093803.133	98187.95131	8.976748038
UJJ64240.1	alpha/beta_hydrolase_[Bacillus_cereus]	CPTF_Cd	1182657.333	14702.74486	1.243195678
UJJ64240.1	alpha/beta_hydrolase_[Bacillus_cereus]	CPTF_Co	777539.6667	196457.1658	25.26651363
UJJ64240.1	alpha/beta_hydrolase_[Bacillus_cereus]	CPTF_Cu	869966	182051.7761	20.92630931
UJJ64240.1	alpha/beta_hydrolase_[Bacillus_cereus]	CPTF_Fe	1104437.667	229769.391	20.80419728
UJJ64240.1	alpha/beta_hydrolase_[Bacillus_cereus]	CPTF_Mn	759781.3333	140917.8122	18.54715376
UJJ64240.1	alpha/beta_hydrolase_[Bacillus_cereus]	CPTF_Ni	726139.3	152532.4966	21.00595528
UJJ64240.1	alpha/beta_hydrolase_[Bacillus_cereus]	CPTF_U	723781	247503.4321	34.1959007
UJJ64240.1	alpha/beta_hydrolase_[Bacillus_cereus]	CPTF_metals_mix	693420.4667	44146.93161	6.366545803
UJJ64240.1	alpha/beta_hydrolase_[Bacillus_cereus]	CPTF_zcontrol	791938.6667	218457.0319	27.58509479
UJJ64246.1	ABC_transporter_ATP-binding_protein_[Bacillus_cereus]	CPTF_Al	359692.7333	137397.4965	38.19857444
UJJ64246.1	ABC_transporter_ATP-binding_protein_[Bacillus_cereus]	CPTF_Cd	155337.9667	146615.6439	94.38493824
UJJ64246.1	ABC_transporter_ATP-binding_protein_[Bacillus_cereus]	CPTF_Co	465027.4667	227339.696	48.88736952
UJJ64246.1	ABC_transporter_ATP-binding_protein_[Bacillus_cereus]	CPTF_Cu	420862.1	115669.8589	27.48402836
UJJ64246.1	ABC_transporter_ATP-binding_protein_[Bacillus_cereus]	CPTF_Fe	345491.1	158203.6921	45.79096019
UJJ64246.1	ABC_transporter_ATP-binding_protein_[Bacillus_cereus]	CPTF_Mn	354748.1667	68663.7378	19.35562865
UJJ64246.1	ABC_transporter_ATP-binding_protein_[Bacillus_cereus]	CPTF_Ni	533113.5333	107104.838	20.0904369
UJJ64246.1	ABC_transporter_ATP-binding_protein_[Bacillus_cereus]	CPTF_U	472544	311626.9673	65.94665625
UJJ64246.1	ABC_transporter_ATP-binding_protein_[Bacillus_cereus]	CPTF_metals_mix	752348.2	89381.30262	11.88031056
UJJ64246.1	ABC_transporter_ATP-binding_protein_[Bacillus_cereus]	CPTF_zcontrol	306734.4667	130507.3455	42.54733644
UJJ64281.1	ABC_transporter_ATP-binding_protein_[Bacillus_cereus]	CPTF_Al	297091.6667	107552.564	36.20181112
UJJ64281.1	ABC_transporter_ATP-binding_protein_[Bacillus_cereus]	CPTF_Cd	618782.5	127047.7771	20.53189564
UJJ64281.1	ABC_transporter_ATP-binding_protein_[Bacillus_cereus]	CPTF_Co	240456.4667	240539.7931	100.0346534
UJJ64281.1	ABC_transporter_ATP-binding_protein_[Bacillus_cereus]	CPTF_Cu	113282.5667	108587.8178	95.85571815
UJJ64281.1	ABC_transporter_ATP-binding_protein_[Bacillus_cereus]	CPTF_Fe	434783	202242.2358	46.51567237
UJJ64281.1	ABC_transporter_ATP-binding_protein_[Bacillus_cereus]	CPTF_Mn	356405.3333	199694.1984	56.03008141
UJJ64281.1	ABC_transporter_ATP-binding_protein_[Bacillus_cereus]	CPTF_Ni	0	0	0
UJJ64281.1	ABC_transporter_ATP-binding_protein_[Bacillus_cereus]	CPTF_U	86443.26667	40896.04407	47.30969298
UJJ64281.1	ABC_transporter_ATP-binding_protein_[Bacillus_cereus]	CPTF_metals_mix	357481.3333	46658.98253	13.05214515
UJJ64281.1	ABC_transporter_ATP-binding_protein_[Bacillus_cereus]	CPTF_zcontrol	277643	140167.2971	50.48472213

UIJ64288.1	tyrosine-protein_phosphatase_[Bacillus_cereus]	CPTF_Al	10940.2	18948.98224	173.2050808
UIJ64288.1	tyrosine-protein_phosphatase_[Bacillus_cereus]	CPTF_Cd	16454.4	28499.85681	173.2050808
UIJ64288.1	tyrosine-protein_phosphatase_[Bacillus_cereus]	CPTF_Co	9608.733333	16642.81433	173.2050808
UIJ64288.1	tyrosine-protein_phosphatase_[Bacillus_cereus]	CPTF_Cu	56305.06667	5048.791222	8.966850624
UIJ64288.1	tyrosine-protein_phosphatase_[Bacillus_cereus]	CPTF_Fe	0	0	0
UIJ64288.1	tyrosine-protein_phosphatase_[Bacillus_cereus]	CPTF_Mn	0	0	0
UIJ64288.1	tyrosine-protein_phosphatase_[Bacillus_cereus]	CPTF_Ni	13222.96667	22902.85009	173.2050808
UIJ64288.1	tyrosine-protein_phosphatase_[Bacillus_cereus]	CPTF_U	0	0	0
UIJ64288.1	tyrosine-protein_phosphatase_[Bacillus_cereus]	CPTF_metals_mix	99034.16667	17352.15088	17.52137819
UIJ64288.1	tyrosine-protein_phosphatase_[Bacillus_cereus]	CPTF_zcontrol	27371.23333	24606.35556	89.89859997
UIJ64311.1	lactonase_family_protein_[Bacillus_cereus]	CPTF_Al	360487.6667	145935.4939	40.48279799
UIJ64311.1	lactonase_family_protein_[Bacillus_cereus]	CPTF_Cd	222952	48048.92383	21.55124145
UIJ64311.1	lactonase_family_protein_[Bacillus_cereus]	CPTF_Co	222232.6667	51528.76131	23.18685281
UIJ64311.1	lactonase_family_protein_[Bacillus_cereus]	CPTF_Cu	303139.3333	27183.05432	8.967181535
UIJ64311.1	lactonase_family_protein_[Bacillus_cereus]	CPTF_Fe	293689.3333	41638.76588	14.1778271
UIJ64311.1	lactonase_family_protein_[Bacillus_cereus]	CPTF_Mn	204506.3333	179121.578	87.58730115
UIJ64311.1	lactonase_family_protein_[Bacillus_cereus]	CPTF_Ni	350408.3333	180702.4369	51.56910372
UIJ64311.1	lactonase_family_protein_[Bacillus_cereus]	CPTF_U	337753.3333	3030.113089	0.897137878
UIJ64311.1	lactonase_family_protein_[Bacillus_cereus]	CPTF_metals_mix	554701.6667	91067.9655	16.41746744
UIJ64311.1	lactonase_family_protein_[Bacillus_cereus]	CPTF_zcontrol	266715.3333	36396.24305	13.64610073
UIJ64338.1	oxidoreductase_[Bacillus_cereus]	CPTF_Al	23984.13333	41541.73751	173.2050808
UIJ64338.1	oxidoreductase_[Bacillus_cereus]	CPTF_Cd	57407.66667	35079.57671	61.10608347
UIJ64338.1	oxidoreductase_[Bacillus_cereus]	CPTF_Co	6465.433333	11198.45903	173.2050808
UIJ64338.1	oxidoreductase_[Bacillus_cereus]	CPTF_Cu	61913.76667	69545.18244	112.3258787
UIJ64338.1	oxidoreductase_[Bacillus_cereus]	CPTF_Fe	27269.7	34951.63424	128.1702191
UIJ64338.1	oxidoreductase_[Bacillus_cereus]	CPTF_Mn	38119.33333	66024.62208	173.2050808
UIJ64338.1	oxidoreductase_[Bacillus_cereus]	CPTF_Ni	0	0	0
UIJ64338.1	oxidoreductase_[Bacillus_cereus]	CPTF_U	38359.66667	66440.89163	173.2050808
UIJ64338.1	oxidoreductase_[Bacillus_cereus]	CPTF_metals_mix	120183.7667	89793.55845	74.71354987
UIJ64338.1	oxidoreductase_[Bacillus_cereus]	CPTF_zcontrol	0	0	0
UIJ64342.1	aldo/keto_reductase_[Bacillus_cereus]	CPTF_Al	0	0	0
UIJ64342.1	aldo/keto_reductase_[Bacillus_cereus]	CPTF_Cd	23664.76667	40988.57822	173.2050808
UIJ64342.1	aldo/keto_reductase_[Bacillus_cereus]	CPTF_Co	12439.63333	21546.07696	173.2050808
UIJ64342.1	aldo/keto_reductase_[Bacillus_cereus]	CPTF_Cu	0	0	0
UIJ64342.1	aldo/keto_reductase_[Bacillus_cereus]	CPTF_Fe	33176.5	28738.80003	86.62396584
UIJ64342.1	aldo/keto_reductase_[Bacillus_cereus]	CPTF_Mn	11567.66667	20035.78639	173.2050808
UIJ64342.1	aldo/keto_reductase_[Bacillus_cereus]	CPTF_Ni	13441.73333	23281.76508	173.2050808
UIJ64342.1	aldo/keto_reductase_[Bacillus_cereus]	CPTF_U	6587.9	11410.57752	173.2050808
UIJ64342.1	aldo/keto_reductase_[Bacillus_cereus]	CPTF_metals_mix	0	0	0
UIJ64342.1	aldo/keto_reductase_[Bacillus_cereus]	CPTF_zcontrol	0	0	0
UIJ64363.1	hypothetical_protein_LW858_15275_[Bacillus_cereus]	CPTF_Al	117777.3333	122060.0558	103.6362875
UIJ64363.1	hypothetical_protein_LW858_15275_[Bacillus_cereus]	CPTF_Cd	0	0	0
UIJ64363.1	hypothetical_protein_LW858_15275_[Bacillus_cereus]	CPTF_Co	0	0	0
UIJ64363.1	hypothetical_protein_LW858_15275_[Bacillus_cereus]	CPTF_Cu	51871.33333	89843.78479	173.2050808
UIJ64363.1	hypothetical_protein_LW858_15275_[Bacillus_cereus]	CPTF_Fe	69129.66667	119736.095	173.2050808
UIJ64363.1	hypothetical_protein_LW858_15275_[Bacillus_cereus]	CPTF_Mn	0	0	0
UIJ64363.1	hypothetical_protein_LW858_15275_[Bacillus_cereus]	CPTF_Ni	52287	90563.74058	173.2050808
UIJ64363.1	hypothetical_protein_LW858_15275_[Bacillus_cereus]	CPTF_U	83223.33333	144147.0417	173.2050808
UIJ64363.1	hypothetical_protein_LW858_15275_[Bacillus_cereus]	CPTF_metals_mix	0	0	0
UIJ64363.1	hypothetical_protein_LW858_15275_[Bacillus_cereus]	CPTF_zcontrol	176503.6667	161353.9655	91.4167782
UIJ64365.1	hypothetical_protein_LW858_15285_[Bacillus_cereus]	CPTF_Al	1182913.667	178324.1332	15.07499137
UIJ64365.1	hypothetical_protein_LW858_15285_[Bacillus_cereus]	CPTF_Cd	1079352.467	58869.01638	5.454104956
UIJ64365.1	hypothetical_protein_LW858_15285_[Bacillus_cereus]	CPTF_Co	982797.7333	164751.3017	16.76350037
UIJ64365.1	hypothetical_protein_LW858_15285_[Bacillus_cereus]	CPTF_Cu	1091244.333	58118.50807	5.32589323
UIJ64365.1	hypothetical_protein_LW858_15285_[Bacillus_cereus]	CPTF_Fe	1208055.467	119982.2913	9.931852846
UIJ64365.1	hypothetical_protein_LW858_15285_[Bacillus_cereus]	CPTF_Mn	1179490.3	159098.4092	13.48874248
UIJ64365.1	hypothetical_protein_LW858_15285_[Bacillus_cereus]	CPTF_Ni	742530.6333	84431.00306	11.37070974
UIJ64365.1	hypothetical_protein_LW858_15285_[Bacillus_cereus]	CPTF_U	815994.3333	174086.5513	21.33428434
UIJ64365.1	hypothetical_protein_LW858_15285_[Bacillus_cereus]	CPTF_metals_mix	675245.2667	70582.74813	10.45290528
UIJ64365.1	hypothetical_protein_LW858_15285_[Bacillus_cereus]	CPTF_zcontrol	1292069.367	186522.6376	14.4359616
UIJ64366.1	hydroxylamine_reductase_[Bacillus_cereus]	CPTF_Al	1764617.233	740775.023	41.97936012

UIJ64366.1	hydroxylamine_reductase_[Bacillus_cereus]	CPTF_Cd	2282335.033	468598.3709	20.53153301
UIJ64366.1	hydroxylamine_reductase_[Bacillus_cereus]	CPTF_Co	1070715.167	814878.5849	76.10600935
UIJ64366.1	hydroxylamine_reductase_[Bacillus_cereus]	CPTF_Cu	1073086.5	55868.4754	5.206334755
UIJ64366.1	hydroxylamine_reductase_[Bacillus_cereus]	CPTF_Fe	2336912.6	1353403.081	57.91415055
UIJ64366.1	hydroxylamine_reductase_[Bacillus_cereus]	CPTF_Mn	2999703.233	3217172.29	107.2496857
UIJ64366.1	hydroxylamine_reductase_[Bacillus_cereus]	CPTF_Ni	588605.3	120278.5525	20.43450042
UIJ64366.1	hydroxylamine_reductase_[Bacillus_cereus]	CPTF_U	568295.2333	40157.48571	7.066306974
UIJ64366.1	hydroxylamine_reductase_[Bacillus_cereus]	CPTF_metals_mix	2833817.1	373251.8075	13.17134431
UIJ64366.1	hydroxylamine_reductase_[Bacillus_cereus]	CPTF_zcontrol	1217357.833	311842.3362	25.61632477
UIJ64381.1	SDR_family_oxidoreductase_[Bacillus_cereus]	CPTF_Al	0	0	0
UIJ64381.1	SDR_family_oxidoreductase_[Bacillus_cereus]	CPTF_Cd	0	0	0
UIJ64381.1	SDR_family_oxidoreductase_[Bacillus_cereus]	CPTF_Co	0	0	0
UIJ64381.1	SDR_family_oxidoreductase_[Bacillus_cereus]	CPTF_Cu	0	0	0
UIJ64381.1	SDR_family_oxidoreductase_[Bacillus_cereus]	CPTF_Fe	0	0	0
UIJ64381.1	SDR_family_oxidoreductase_[Bacillus_cereus]	CPTF_Mn	20999.3	36371.85452	173.2050808
UIJ64381.1	SDR_family_oxidoreductase_[Bacillus_cereus]	CPTF_Ni	0	0	0
UIJ64381.1	SDR_family_oxidoreductase_[Bacillus_cereus]	CPTF_U	0	0	0
UIJ64381.1	SDR_family_oxidoreductase_[Bacillus_cereus]	CPTF_metals_mix	1015951.867	171876.5708	16.9177868
UIJ64381.1	SDR_family_oxidoreductase_[Bacillus_cereus]	CPTF_zcontrol	0	0	0
UIJ64382.1	bacillithiol_biosynthesis_deacetylase_BshB2_[Bacillus_cereus]	CPTF_Al	694539.3333	230666.3769	33.21142027
UIJ64382.1	bacillithiol_biosynthesis_deacetylase_BshB2_[Bacillus_cereus]	CPTF_Cd	816071.3333	10641.34307	1.30397217
UIJ64382.1	bacillithiol_biosynthesis_deacetylase_BshB2_[Bacillus_cereus]	CPTF_Co	647503.6667	156505.2245	24.17055416
UIJ64382.1	bacillithiol_biosynthesis_deacetylase_BshB2_[Bacillus_cereus]	CPTF_Cu	805047.3333	60006.39969	7.453772867
UIJ64382.1	bacillithiol_biosynthesis_deacetylase_BshB2_[Bacillus_cereus]	CPTF_Fe	775344.1333	70607.27057	9.106571848
UIJ64382.1	bacillithiol_biosynthesis_deacetylase_BshB2_[Bacillus_cereus]	CPTF_Mn	584965.6	56183.63932	9.604605693
UIJ64382.1	bacillithiol_biosynthesis_deacetylase_BshB2_[Bacillus_cereus]	CPTF_Ni	730467.8333	44674.57623	6.115885491
UIJ64382.1	bacillithiol_biosynthesis_deacetylase_BshB2_[Bacillus_cereus]	CPTF_U	793740.3333	40935.82375	5.157331943
UIJ64382.1	bacillithiol_biosynthesis_deacetylase_BshB2_[Bacillus_cereus]	CPTF_metals_mix	1237876.567	145442.6489	11.74936604
UIJ64382.1	bacillithiol_biosynthesis_deacetylase_BshB2_[Bacillus_cereus]	CPTF_zcontrol	677694.6667	87170.65502	12.86282146
UIJ64383.1	YojF_family_protein_[Bacillus_cereus]	CPTF_Al	0	0	0
UIJ64383.1	YojF_family_protein_[Bacillus_cereus]	CPTF_Cd	0	0	0
UIJ64383.1	YojF_family_protein_[Bacillus_cereus]	CPTF_Co	0	0	0
UIJ64383.1	YojF_family_protein_[Bacillus_cereus]	CPTF_Cu	166298.3333	172808.1372	103.9145334
UIJ64383.1	YojF_family_protein_[Bacillus_cereus]	CPTF_Fe	0	0	0
UIJ64383.1	YojF_family_protein_[Bacillus_cereus]	CPTF_Mn	0	0	0
UIJ64383.1	YojF_family_protein_[Bacillus_cereus]	CPTF_Ni	103406.6667	179105.6005	173.2050808
UIJ64383.1	YojF_family_protein_[Bacillus_cereus]	CPTF_U	66804	115707.9221	173.2050808
UIJ64383.1	YojF_family_protein_[Bacillus_cereus]	CPTF_metals_mix	511305.6667	92305.82753	18.05296392
UIJ64383.1	YojF_family_protein_[Bacillus_cereus]	CPTF_zcontrol	92795	160725.6547	173.2050808
UIJ64393.1	DinB_family_protein_[Bacillus_cereus]	CPTF_Al	27722.73333	30855.89102	111.3017633
UIJ64393.1	DinB_family_protein_[Bacillus_cereus]	CPTF_Cd	15867.33333	27483.02751	173.2050808
UIJ64393.1	DinB_family_protein_[Bacillus_cereus]	CPTF_Co	36796.3	32000.11148	86.96556851
UIJ64393.1	DinB_family_protein_[Bacillus_cereus]	CPTF_Cu	0	0	0
UIJ64393.1	DinB_family_protein_[Bacillus_cereus]	CPTF_Fe	0	0	0
UIJ64393.1	DinB_family_protein_[Bacillus_cereus]	CPTF_Mn	0	0	0
UIJ64393.1	DinB_family_protein_[Bacillus_cereus]	CPTF_Ni	41661.83333	36082.20339	86.60733459
UIJ64393.1	DinB_family_protein_[Bacillus_cereus]	CPTF_U	0	0	0
UIJ64393.1	DinB_family_protein_[Bacillus_cereus]	CPTF_metals_mix	0	0	0
UIJ64393.1	DinB_family_protein_[Bacillus_cereus]	CPTF_zcontrol	10025.53333	17364.73311	173.2050808
UIJ64399.1	phosphoglycerate_mutase_family_protein_[Bacillus_cereus]	CPTF_Al	348553.3667	47764.52354	13.70364716
UIJ64399.1	phosphoglycerate_mutase_family_protein_[Bacillus_cereus]	CPTF_Cd	532927	234539.2703	44.00964302
UIJ64399.1	phosphoglycerate_mutase_family_protein_[Bacillus_cereus]	CPTF_Co	252556.6667	17586.72716	6.9634777
UIJ64399.1	phosphoglycerate_mutase_family_protein_[Bacillus_cereus]	CPTF_Cu	373926.9	34770.33767	9.298699202
UIJ64399.1	phosphoglycerate_mutase_family_protein_[Bacillus_cereus]	CPTF_Fe	229241	216790.5858	94.56885363
UIJ64399.1	phosphoglycerate_mutase_family_protein_[Bacillus_cereus]	CPTF_Mn	334154.6333	17653.63392	5.283073213
UIJ64399.1	phosphoglycerate_mutase_family_protein_[Bacillus_cereus]	CPTF_Ni	279286	65835.36639	23.57274134
UIJ64399.1	phosphoglycerate_mutase_family_protein_[Bacillus_cereus]	CPTF_U	278635.3333	148923.0506	53.44729574
UIJ64399.1	phosphoglycerate_mutase_family_protein_[Bacillus_cereus]	CPTF_metals_mix	960550.6667	127101.2035	13.23211861
UIJ64399.1	phosphoglycerate_mutase_family_protein_[Bacillus_cereus]	CPTF_zcontrol	230136.8333	154000.03	66.91672417
UIJ64405.1	oligoendopeptidase_F_[Bacillus_cereus]	CPTF_Al	984207.6	131616.4248	13.37283159
UIJ64405.1	oligoendopeptidase_F_[Bacillus_cereus]	CPTF_Cd	1179075.333	162315.3419	13.76632496

UIJ64405.1	oligoendopeptidase_F_[Bacillus_cereus]	CPTF_Co	1023598.467	127855.295	12.49076656
UIJ64405.1	oligoendopeptidase_F_[Bacillus_cereus]	CPTF_Cu	940582.4667	187914.562	19.97853124
UIJ64405.1	oligoendopeptidase_F_[Bacillus_cereus]	CPTF_Fe	875738.9	43086.195	4.919981858
UIJ64405.1	oligoendopeptidase_F_[Bacillus_cereus]	CPTF_Mn	791840.9	264202.0455	33.36554673
UIJ64405.1	oligoendopeptidase_F_[Bacillus_cereus]	CPTF_Ni	689061.1	137425.7566	19.9439145
UIJ64405.1	oligoendopeptidase_F_[Bacillus_cereus]	CPTF_U	628052.3333	210158.2144	33.46189533
UIJ64405.1	oligoendopeptidase_F_[Bacillus_cereus]	CPTF_metals_mix	1042496.333	34944.80763	3.352031706
UIJ64405.1	oligoendopeptidase_F_[Bacillus_cereus]	CPTF_zcontrol	897834.1	36247.60456	4.03722743
UIJ64422.1	YdiU_family_protein_[Bacillus_cereus]	CPTF_Al	0	0	0
UIJ64422.1	YdiU_family_protein_[Bacillus_cereus]	CPTF_Cd	0	0	0
UIJ64422.1	YdiU_family_protein_[Bacillus_cereus]	CPTF_Co	0	0	0
UIJ64422.1	YdiU_family_protein_[Bacillus_cereus]	CPTF_Cu	0	0	0
UIJ64422.1	YdiU_family_protein_[Bacillus_cereus]	CPTF_Fe	0	0	0
UIJ64422.1	YdiU_family_protein_[Bacillus_cereus]	CPTF_Mn	0	0	0
UIJ64422.1	YdiU_family_protein_[Bacillus_cereus]	CPTF_Ni	0	0	0
UIJ64422.1	YdiU_family_protein_[Bacillus_cereus]	CPTF_U	0	0	0
UIJ64422.1	YdiU_family_protein_[Bacillus_cereus]	CPTF_metals_mix	149558.6333	97347.18165	65.08964376
UIJ64422.1	YdiU_family_protein_[Bacillus_cereus]	CPTF_zcontrol	0	0	0
UIJ64423.1	hypothetical_protein_LW858_15595_[Bacillus_cereus]	CPTF_Al	1115090.667	292251.9741	26.2088082
UIJ64423.1	hypothetical_protein_LW858_15595_[Bacillus_cereus]	CPTF_Cd	1005931.367	360543.1899	35.84172855
UIJ64423.1	hypothetical_protein_LW858_15595_[Bacillus_cereus]	CPTF_Co	1284420	578838.2094	45.06611618
UIJ64423.1	hypothetical_protein_LW858_15595_[Bacillus_cereus]	CPTF_Cu	480339.4333	169247.9988	35.23508317
UIJ64423.1	hypothetical_protein_LW858_15595_[Bacillus_cereus]	CPTF_Fe	900165.3333	242266.3724	26.91354171
UIJ64423.1	hypothetical_protein_LW858_15595_[Bacillus_cereus]	CPTF_Mn	669727	438029.0964	65.40412681
UIJ64423.1	hypothetical_protein_LW858_15595_[Bacillus_cereus]	CPTF_Ni	815544.0333	711088.6345	87.19193635
UIJ64423.1	hypothetical_protein_LW858_15595_[Bacillus_cereus]	CPTF_U	546698.6	370856.6104	67.83566126
UIJ64423.1	hypothetical_protein_LW858_15595_[Bacillus_cereus]	CPTF_metals_mix	178003.7	41978.1691	23.58275086
UIJ64423.1	hypothetical_protein_LW858_15595_[Bacillus_cereus]	CPTF_zcontrol	1327790.333	429948.0974	32.38072206
UIJ64425.1	FMN-binding_negative_transcriptional_regulator_[Bacillus_cereus]	CPTF_Al	0	0	0
UIJ64425.1	FMN-binding_negative_transcriptional_regulator_[Bacillus_cereus]	CPTF_Cd	0	0	0
UIJ64425.1	FMN-binding_negative_transcriptional_regulator_[Bacillus_cereus]	CPTF_Co	0	0	0
UIJ64425.1	FMN-binding_negative_transcriptional_regulator_[Bacillus_cereus]	CPTF_Cu	0	0	0
UIJ64425.1	FMN-binding_negative_transcriptional_regulator_[Bacillus_cereus]	CPTF_Fe	0	0	0
UIJ64425.1	FMN-binding_negative_transcriptional_regulator_[Bacillus_cereus]	CPTF_Mn	0	0	0
UIJ64425.1	FMN-binding_negative_transcriptional_regulator_[Bacillus_cereus]	CPTF_Ni	0	0	0
UIJ64425.1	FMN-binding_negative_transcriptional_regulator_[Bacillus_cereus]	CPTF_U	0	0	0
UIJ64425.1	FMN-binding_negative_transcriptional_regulator_[Bacillus_cereus]	CPTF_metals_mix	158173.6667	12218.96208	7.725029291
UIJ64425.1	FMN-binding_negative_transcriptional_regulator_[Bacillus_cereus]	CPTF_zcontrol	0	0	0
UIJ64437.1	VOC_family_protein_[Bacillus_cereus]	CPTF_Al	1339750	128334.603	9.578996305
UIJ64437.1	VOC_family_protein_[Bacillus_cereus]	CPTF_Cd	1279607.7	143122.1273	11.18484418
UIJ64437.1	VOC_family_protein_[Bacillus_cereus]	CPTF_Co	1444624.867	169885.3172	11.7598223
UIJ64437.1	VOC_family_protein_[Bacillus_cereus]	CPTF_Cu	1373777.3	80904.87071	5.889227512
UIJ64437.1	VOC_family_protein_[Bacillus_cereus]	CPTF_Fe	1134008.333	260548.3058	22.97587224
UIJ64437.1	VOC_family_protein_[Bacillus_cereus]	CPTF_Mn	1169533.4	86090.39326	7.361088898
UIJ64437.1	VOC_family_protein_[Bacillus_cereus]	CPTF_Ni	1212888.167	191458.7322	15.78535742
UIJ64437.1	VOC_family_protein_[Bacillus_cereus]	CPTF_U	1093360.833	200591.5724	18.34632871
UIJ64437.1	VOC_family_protein_[Bacillus_cereus]	CPTF_metals_mix	1692366.5	207723.5346	12.27414597
UIJ64437.1	VOC_family_protein_[Bacillus_cereus]	CPTF_zcontrol	1175805.3	130753.3235	11.12032099
UIJ64438.1	VanW_family_protein_[Bacillus_cereus]	CPTF_Al	41403.33333	71712.67694	173.2050808
UIJ64438.1	VanW_family_protein_[Bacillus_cereus]	CPTF_Cd	345122	111859.0102	32.41144007
UIJ64438.1	VanW_family_protein_[Bacillus_cereus]	CPTF_Co	130800	120554.0958	92.16673989
UIJ64438.1	VanW_family_protein_[Bacillus_cereus]	CPTF_Cu	33432.33333	57906.49995	173.2050808
UIJ64438.1	VanW_family_protein_[Bacillus_cereus]	CPTF_Fe	169277	158375.9186	93.56021115
UIJ64438.1	VanW_family_protein_[Bacillus_cereus]	CPTF_Mn	0	0	0
UIJ64438.1	VanW_family_protein_[Bacillus_cereus]	CPTF_Ni	282152.0333	194302.1496	68.86434498
UIJ64438.1	VanW_family_protein_[Bacillus_cereus]	CPTF_U	163912.3333	179011.9739	109.2120222
UIJ64438.1	VanW_family_protein_[Bacillus_cereus]	CPTF_metals_mix	188249	91767.80859	48.74809884
UIJ64438.1	VanW_family_protein_[Bacillus_cereus]	CPTF_zcontrol	150522.0667	130565.6293	86.74185264
UIJ64444.1	hypothetical_protein_LW858_15720_[Bacillus_cereus]	CPTF_Al	0	0	0
UIJ64444.1	hypothetical_protein_LW858_15720_[Bacillus_cereus]	CPTF_Cd	0	0	0
UIJ64444.1	hypothetical_protein_LW858_15720_[Bacillus_cereus]	CPTF_Co	0	0	0

UIJ64444.1	hypothetical_protein_LW858_15720_[Bacillus_cereus]	CPTF_Cu	0	0	0
UIJ64444.1	hypothetical_protein_LW858_15720_[Bacillus_cereus]	CPTF_Fe	0	0	0
UIJ64444.1	hypothetical_protein_LW858_15720_[Bacillus_cereus]	CPTF_Mn	0	0	0
UIJ64444.1	hypothetical_protein_LW858_15720_[Bacillus_cereus]	CPTF_Ni	0	0	0
UIJ64444.1	hypothetical_protein_LW858_15720_[Bacillus_cereus]	CPTF_U	0	0	0
UIJ64444.1	hypothetical_protein_LW858_15720_[Bacillus_cereus]	CPTF_metals_mix	30438.26667	52720.62436	173.2050808
UIJ64444.1	hypothetical_protein_LW858_15720_[Bacillus_cereus]	CPTF_zcontrol	0	0	0
UIJ64472.1	hypothetical_protein_LW858_15860_[Bacillus_cereus]	CPTF_Al	336666.6667	583123.7719	173.2050808
UIJ64472.1	hypothetical_protein_LW858_15860_[Bacillus_cereus]	CPTF_Cd	708604.6667	619819.0227	87.47035573
UIJ64472.1	hypothetical_protein_LW858_15860_[Bacillus_cereus]	CPTF_Co	0	0	0
UIJ64472.1	hypothetical_protein_LW858_15860_[Bacillus_cereus]	CPTF_Cu	0	0	0
UIJ64472.1	hypothetical_protein_LW858_15860_[Bacillus_cereus]	CPTF_Fe	0	0	0
UIJ64472.1	hypothetical_protein_LW858_15860_[Bacillus_cereus]	CPTF_Mn	756666.6667	656226.5869	86.72598065
UIJ64472.1	hypothetical_protein_LW858_15860_[Bacillus_cereus]	CPTF_Ni	257810	446540.0187	173.2050808
UIJ64472.1	hypothetical_protein_LW858_15860_[Bacillus_cereus]	CPTF_U	0	0	0
UIJ64472.1	hypothetical_protein_LW858_15860_[Bacillus_cereus]	CPTF_metals_mix	585287	593520.9896	101.4068294
UIJ64472.1	hypothetical_protein_LW858_15860_[Bacillus_cereus]	CPTF_zcontrol	743333.3333	645316.4598	86.81387352
UIJ64495.1	helix-turn-helix_transcriptional_regulator_[Bacillus_cereus]	CPTF_Al	0	0	0
UIJ64495.1	helix-turn-helix_transcriptional_regulator_[Bacillus_cereus]	CPTF_Cd	0	0	0
UIJ64495.1	helix-turn-helix_transcriptional_regulator_[Bacillus_cereus]	CPTF_Co	0	0	0
UIJ64495.1	helix-turn-helix_transcriptional_regulator_[Bacillus_cereus]	CPTF_Cu	0	0	0
UIJ64495.1	helix-turn-helix_transcriptional_regulator_[Bacillus_cereus]	CPTF_Fe	0	0	0
UIJ64495.1	helix-turn-helix_transcriptional_regulator_[Bacillus_cereus]	CPTF_Mn	0	0	0
UIJ64495.1	helix-turn-helix_transcriptional_regulator_[Bacillus_cereus]	CPTF_Ni	0	0	0
UIJ64495.1	helix-turn-helix_transcriptional_regulator_[Bacillus_cereus]	CPTF_U	0	0	0
UIJ64495.1	helix-turn-helix_transcriptional_regulator_[Bacillus_cereus]	CPTF_metals_mix	7956.2	13780.54264	173.2050808
UIJ64495.1	helix-turn-helix_transcriptional_regulator_[Bacillus_cereus]	CPTF_zcontrol	0	0	0
UIJ64509.1	cold_shock-like_protein_CspB_[Bacillus_cereus]	CPTF_Al	7523009.333	1322552.533	17.58009959
UIJ64509.1	cold_shock-like_protein_CspB_[Bacillus_cereus]	CPTF_Cd	15280000	1211321.592	7.927497332
UIJ64509.1	cold_shock-like_protein_CspB_[Bacillus_cereus]	CPTF_Co	10254293.67	711991.3028	6.943348084
UIJ64509.1	cold_shock-like_protein_CspB_[Bacillus_cereus]	CPTF_Cu	8823523	1740513.215	19.72583077
UIJ64509.1	cold_shock-like_protein_CspB_[Bacillus_cereus]	CPTF_Fe	10446172	4239067.921	40.5801084
UIJ64509.1	cold_shock-like_protein_CspB_[Bacillus_cereus]	CPTF_Mn	10584570	7189211.421	67.92162007
UIJ64509.1	cold_shock-like_protein_CspB_[Bacillus_cereus]	CPTF_Ni	9351878.333	2874909.938	30.74152417
UIJ64509.1	cold_shock-like_protein_CspB_[Bacillus_cereus]	CPTF_U	13430000	3635945.544	27.07331008
UIJ64509.1	cold_shock-like_protein_CspB_[Bacillus_cereus]	CPTF_metals_mix	33017893.87	3459589.3	10.47792241
UIJ64509.1	cold_shock-like_protein_CspB_[Bacillus_cereus]	CPTF_zcontrol	5780326	979261.9867	16.94129339
UIJ64511.1	flavodoxin_[Bacillus_cereus]	CPTF_Al	0	0	0
UIJ64511.1	flavodoxin_[Bacillus_cereus]	CPTF_Cd	0	0	0
UIJ64511.1	flavodoxin_[Bacillus_cereus]	CPTF_Co	0	0	0
UIJ64511.1	flavodoxin_[Bacillus_cereus]	CPTF_Cu	107975.6667	93642.21177	86.72529159
UIJ64511.1	flavodoxin_[Bacillus_cereus]	CPTF_Fe	0	0	0
UIJ64511.1	flavodoxin_[Bacillus_cereus]	CPTF_Mn	0	0	0
UIJ64511.1	flavodoxin_[Bacillus_cereus]	CPTF_Ni	0	0	0
UIJ64511.1	flavodoxin_[Bacillus_cereus]	CPTF_U	0	0	0
UIJ64511.1	flavodoxin_[Bacillus_cereus]	CPTF_metals_mix	324219.6667	41900.28337	12.92342436
UIJ64511.1	flavodoxin_[Bacillus_cereus]	CPTF_zcontrol	0	0	0
UIJ64516.1	(S)-benzoin_forming_benzil_reductase_[Bacillus_cereus]	CPTF_Al	86558.33333	75776.51753	87.54387314
UIJ64516.1	(S)-benzoin_forming_benzil_reductase_[Bacillus_cereus]	CPTF_Cd	26512.1	45920.30422	173.2050808
UIJ64516.1	(S)-benzoin_forming_benzil_reductase_[Bacillus_cereus]	CPTF_Co	0	0	0
UIJ64516.1	(S)-benzoin_forming_benzil_reductase_[Bacillus_cereus]	CPTF_Cu	160981.5667	90657.21377	56.31527612
UIJ64516.1	(S)-benzoin_forming_benzil_reductase_[Bacillus_cereus]	CPTF_Fe	129872.4333	56870.15427	43.78924211
UIJ64516.1	(S)-benzoin_forming_benzil_reductase_[Bacillus_cereus]	CPTF_Mn	30234.26667	52367.286	173.2050808
UIJ64516.1	(S)-benzoin_forming_benzil_reductase_[Bacillus_cereus]	CPTF_Ni	78676.3	89017.4386	113.1439056
UIJ64516.1	(S)-benzoin_forming_benzil_reductase_[Bacillus_cereus]	CPTF_U	27740.86667	48048.59051	173.2050808
UIJ64516.1	(S)-benzoin_forming_benzil_reductase_[Bacillus_cereus]	CPTF_metals_mix	173938.3333	112028.2957	64.40690419
UIJ64516.1	(S)-benzoin_forming_benzil_reductase_[Bacillus_cereus]	CPTF_zcontrol	32195.33333	55763.9531	173.2050808
UIJ64517.1	GNAT_family_N-acetyltransferase_[Bacillus_cereus]	CPTF_Al	380023.7	12791.20831	3.365897523
UIJ64517.1	GNAT_family_N-acetyltransferase_[Bacillus_cereus]	CPTF_Cd	434857.0333	197961.4891	45.52334996
UIJ64517.1	GNAT_family_N-acetyltransferase_[Bacillus_cereus]	CPTF_Co	418747.3333	21583.06119	5.15419669
UIJ64517.1	GNAT_family_N-acetyltransferase_[Bacillus_cereus]	CPTF_Cu	406181.1	192129.4957	47.30143664

UIJ64517.1	GNAT_family_N-acetyltransferase_[Bacillus_cereus]	CPTF_Fe	512801.0333	48994.29351	9.554250153
UIJ64517.1	GNAT_family_N-acetyltransferase_[Bacillus_cereus]	CPTF_Mn	265915.7	246584.4104	92.73029401
UIJ64517.1	GNAT_family_N-acetyltransferase_[Bacillus_cereus]	CPTF_Ni	439718.3667	52267.05954	11.88648542
UIJ64517.1	GNAT_family_N-acetyltransferase_[Bacillus_cereus]	CPTF_U	329197.5	205547.1927	62.43886807
UIJ64517.1	GNAT_family_N-acetyltransferase_[Bacillus_cereus]	CPTF_metals_mix	538525.1	59045.48684	10.96429616
UIJ64517.1	GNAT_family_N-acetyltransferase_[Bacillus_cereus]	CPTF_zcontrol	405843.4	46863.40462	11.5471644
UIJ64523.1	aldehyde_dehydrogenase_DhaS_[Bacillus_cereus]	CPTF_Al	9214149.333	615625.0418	6.681300894
UIJ64523.1	aldehyde_dehydrogenase_DhaS_[Bacillus_cereus]	CPTF_Cd	8681086.4	583054.8073	6.716380651
UIJ64523.1	aldehyde_dehydrogenase_DhaS_[Bacillus_cereus]	CPTF_Co	8497890.333	574464.3798	6.760082295
UIJ64523.1	aldehyde_dehydrogenase_DhaS_[Bacillus_cereus]	CPTF_Cu	6698906.1	518157.5294	7.734957345
UIJ64523.1	aldehyde_dehydrogenase_DhaS_[Bacillus_cereus]	CPTF_Fe	8366797.9	415906.386	4.970914691
UIJ64523.1	aldehyde_dehydrogenase_DhaS_[Bacillus_cereus]	CPTF_Mn	8818396.8	394571.8235	4.47441675
UIJ64523.1	aldehyde_dehydrogenase_DhaS_[Bacillus_cereus]	CPTF_Ni	8963762.033	895532.6014	9.990588751
UIJ64523.1	aldehyde_dehydrogenase_DhaS_[Bacillus_cereus]	CPTF_U	8847571.667	1360734.893	15.37975554
UIJ64523.1	aldehyde_dehydrogenase_DhaS_[Bacillus_cereus]	CPTF_metals_mix	6256393.7	75973.31073	1.214330721
UIJ64523.1	aldehyde_dehydrogenase_DhaS_[Bacillus_cereus]	CPTF_zcontrol	8766672.233	19015.14985	0.216902712
UIJ64524.1	3-oxoacyl-ACP_reductase_FabG_[Bacillus_cereus]	CPTF_Al	29508	51109.35523	173.2050808
UIJ64524.1	3-oxoacyl-ACP_reductase_FabG_[Bacillus_cereus]	CPTF_Cd	0	0	0
UIJ64524.1	3-oxoacyl-ACP_reductase_FabG_[Bacillus_cereus]	CPTF_Co	32600.7	56466.06876	173.2050808
UIJ64524.1	3-oxoacyl-ACP_reductase_FabG_[Bacillus_cereus]	CPTF_Cu	68573.5	59490.54781	86.75442818
UIJ64524.1	3-oxoacyl-ACP_reductase_FabG_[Bacillus_cereus]	CPTF_Fe	0	0	0
UIJ64524.1	3-oxoacyl-ACP_reductase_FabG_[Bacillus_cereus]	CPTF_Mn	0	0	0
UIJ64524.1	3-oxoacyl-ACP_reductase_FabG_[Bacillus_cereus]	CPTF_Ni	73654.06667	65733.26689	89.24594373
UIJ64524.1	3-oxoacyl-ACP_reductase_FabG_[Bacillus_cereus]	CPTF_U	28320.46667	49052.48716	173.2050808
UIJ64524.1	3-oxoacyl-ACP_reductase_FabG_[Bacillus_cereus]	CPTF_metals_mix	135611.4333	69557.84147	51.2920185
UIJ64524.1	3-oxoacyl-ACP_reductase_FabG_[Bacillus_cereus]	CPTF_zcontrol	0	0	0
UIJ64548.1	tubulin-like_doman-containing_protein_[Bacillus_cereus]	CPTF_Al	295299.6667	20695.82244	7.008413748
UIJ64548.1	tubulin-like_doman-containing_protein_[Bacillus_cereus]	CPTF_Cd	167314.6667	148068.4031	88.49696564
UIJ64548.1	tubulin-like_doman-containing_protein_[Bacillus_cereus]	CPTF_Co	109654.6667	189927.454	173.2050808
UIJ64548.1	tubulin-like_doman-containing_protein_[Bacillus_cereus]	CPTF_Cu	187892.6667	162905.8015	86.70152188
UIJ64548.1	tubulin-like_doman-containing_protein_[Bacillus_cereus]	CPTF_Fe	187352	162514.481	86.74285889
UIJ64548.1	tubulin-like_doman-containing_protein_[Bacillus_cereus]	CPTF_Mn	261277	40470.20915	15.48938833
UIJ64548.1	tubulin-like_doman-containing_protein_[Bacillus_cereus]	CPTF_Ni	85280.66667	147710.4476	173.2050808
UIJ64548.1	tubulin-like_doman-containing_protein_[Bacillus_cereus]	CPTF_U	0	0	0
UIJ64548.1	tubulin-like_doman-containing_protein_[Bacillus_cereus]	CPTF_metals_mix	0	0	0
UIJ64548.1	tubulin-like_doman-containing_protein_[Bacillus_cereus]	CPTF_zcontrol	111082.3333	192400.2452	173.2050808
UIJ64554.1	peptide_ABC_transporter_substrate-binding_protein_[Bacillus_cereus]	CPTF_Al	3005104.567	516118.8393	17.17473811
UIJ64554.1	peptide_ABC_transporter_substrate-binding_protein_[Bacillus_cereus]	CPTF_Cd	3367667.233	694917.0225	20.63496701
UIJ64554.1	peptide_ABC_transporter_substrate-binding_protein_[Bacillus_cereus]	CPTF_Co	2732070.633	177085.6922	6.481739163
UIJ64554.1	peptide_ABC_transporter_substrate-binding_protein_[Bacillus_cereus]	CPTF_Cu	1790578.533	173518.3449	9.690630246
UIJ64554.1	peptide_ABC_transporter_substrate-binding_protein_[Bacillus_cereus]	CPTF_Fe	3647711.767	617690.3203	16.9336384
UIJ64554.1	peptide_ABC_transporter_substrate-binding_protein_[Bacillus_cereus]	CPTF_Mn	2445839.533	586044.5749	23.96087589
UIJ64554.1	peptide_ABC_transporter_substrate-binding_protein_[Bacillus_cereus]	CPTF_Ni	2420756.4	495618.1986	20.47369156
UIJ64554.1	peptide_ABC_transporter_substrate-binding_protein_[Bacillus_cereus]	CPTF_U	2258283.067	917221.2967	40.61586921
UIJ64554.1	peptide_ABC_transporter_substrate-binding_protein_[Bacillus_cereus]	CPTF_metals_mix	988863.4333	59312.57523	5.998055265
UIJ64554.1	peptide_ABC_transporter_substrate-binding_protein_[Bacillus_cereus]	CPTF_zcontrol	1749014.967	130605.5604	7.467378089
UIJ64556.1	peptide_ABC_transporter_substrate-binding_protein_[Bacillus_cereus]	CPTF_Al	41751144.53	2687344.397	6.436576595
UIJ64556.1	peptide_ABC_transporter_substrate-binding_protein_[Bacillus_cereus]	CPTF_Cd	42282861.63	2953185.979	6.984356936
UIJ64556.1	peptide_ABC_transporter_substrate-binding_protein_[Bacillus_cereus]	CPTF_Co	40236166.9	3099185.602	7.702487192
UIJ64556.1	peptide_ABC_transporter_substrate-binding_protein_[Bacillus_cereus]	CPTF_Cu	31084547.17	1938010.799	6.23464382
UIJ64556.1	peptide_ABC_transporter_substrate-binding_protein_[Bacillus_cereus]	CPTF_Fe	39641977.97	1895625.786	4.781864789
UIJ64556.1	peptide_ABC_transporter_substrate-binding_protein_[Bacillus_cereus]	CPTF_Mn	39996484.57	1608999.751	4.022852928
UIJ64556.1	peptide_ABC_transporter_substrate-binding_protein_[Bacillus_cereus]	CPTF_Ni	38294524.07	2049672.231	5.352389882
UIJ64556.1	peptide_ABC_transporter_substrate-binding_protein_[Bacillus_cereus]	CPTF_U	36244222.1	1774710.59	4.896533813
UIJ64556.1	peptide_ABC_transporter_substrate-binding_protein_[Bacillus_cereus]	CPTF_metals_mix	22631518.13	670840.3252	2.964186146
UIJ64556.1	peptide_ABC_transporter_substrate-binding_protein_[Bacillus_cereus]	CPTF_zcontrol	41030474.2	727831.0269	1.77387915
UIJ64566.1	oxidoreductase_[Bacillus_cereus]	CPTF_Al	91540.76667	93273.265	101.8925976
UIJ64566.1	oxidoreductase_[Bacillus_cereus]	CPTF_Cd	148743	45841.57707	30.81931726
UIJ64566.1	oxidoreductase_[Bacillus_cereus]	CPTF_Co	54531.2	48641.24433	89.19892526
UIJ64566.1	oxidoreductase_[Bacillus_cereus]	CPTF_Cu	162715	32318.71823	19.86216282
UIJ64566.1	oxidoreductase_[Bacillus_cereus]	CPTF_Fe	52598	91102.40838	173.2050808

UIJ64566.1	oxidoreductase_[Bacillus_cereus]	CPTF_Mn	36436.66667	63110.15793	173.2050808
UIJ64566.1	oxidoreductase_[Bacillus_cereus]	CPTF_Ni	100188.3333	93149.57108	92.97446916
UIJ64566.1	oxidoreductase_[Bacillus_cereus]	CPTF_U	47573.33333	82399.43042	173.2050808
UIJ64566.1	oxidoreductase_[Bacillus_cereus]	CPTF_metals_mix	104948.1	32215.96848	30.69704785
UIJ64566.1	oxidoreductase_[Bacillus_cereus]	CPTF_zcontrol	36888.26667	32441.43604	87.94513532
UIJ64567.1	DNA_topoisomerase_IV_subunit_A_[Bacillus_cereus]	CPTF_Al	68439.43333	59585.19208	87.06266136
UIJ64567.1	DNA_topoisomerase_IV_subunit_A_[Bacillus_cereus]	CPTF_Cd	37845.33333	65550.04016	173.2050808
UIJ64567.1	DNA_topoisomerase_IV_subunit_A_[Bacillus_cereus]	CPTF_Co	121671.9	112503.6244	92.46475514
UIJ64567.1	DNA_topoisomerase_IV_subunit_A_[Bacillus_cereus]	CPTF_Cu	60694.7	56662.40764	93.35643416
UIJ64567.1	DNA_topoisomerase_IV_subunit_A_[Bacillus_cereus]	CPTF_Fe	9364.833333	16220.36714	173.2050808
UIJ64567.1	DNA_topoisomerase_IV_subunit_A_[Bacillus_cereus]	CPTF_Mn	28300.7	49018.25029	173.2050808
UIJ64567.1	DNA_topoisomerase_IV_subunit_A_[Bacillus_cereus]	CPTF_Ni	36699	63564.53259	173.2050808
UIJ64567.1	DNA_topoisomerase_IV_subunit_A_[Bacillus_cereus]	CPTF_U	33060.13333	57261.83064	173.2050808
UIJ64567.1	DNA_topoisomerase_IV_subunit_A_[Bacillus_cereus]	CPTF_metals_mix	86380.1	37024.33582	42.86211271
UIJ64567.1	DNA_topoisomerase_IV_subunit_A_[Bacillus_cereus]	CPTF_zcontrol	102820.4333	16315.26098	15.86772244
UIJ64568.1	DNA_topoisomerase_IV_subunit_B_[Bacillus_cereus]	CPTF_Al	184826.3667	57487.65924	31.10360295
UIJ64568.1	DNA_topoisomerase_IV_subunit_B_[Bacillus_cereus]	CPTF_Cd	166610.8333	113422.8487	68.07651484
UIJ64568.1	DNA_topoisomerase_IV_subunit_B_[Bacillus_cereus]	CPTF_Co	170319.8333	71416.74761	41.93096377
UIJ64568.1	DNA_topoisomerase_IV_subunit_B_[Bacillus_cereus]	CPTF_Cu	167461.4	63368.34366	37.84056723
UIJ64568.1	DNA_topoisomerase_IV_subunit_B_[Bacillus_cereus]	CPTF_Fe	156670.7667	90884.17349	58.00965644
UIJ64568.1	DNA_topoisomerase_IV_subunit_B_[Bacillus_cereus]	CPTF_Mn	69423.26667	76304.37646	109.9118208
UIJ64568.1	DNA_topoisomerase_IV_subunit_B_[Bacillus_cereus]	CPTF_Ni	141803.1667	104671.7042	73.81478613
UIJ64568.1	DNA_topoisomerase_IV_subunit_B_[Bacillus_cereus]	CPTF_U	20935.13333	36260.7146	173.2050808
UIJ64568.1	DNA_topoisomerase_IV_subunit_B_[Bacillus_cereus]	CPTF_metals_mix	173044.3667	48936.48382	28.2797324
UIJ64568.1	DNA_topoisomerase_IV_subunit_B_[Bacillus_cereus]	CPTF_zcontrol	114911.2	63987.67679	55.68445617
UIJ64569.1	CoA-binding_protein_[Bacillus_cereus]	CPTF_Al	3460746.667	180902.0653	5.227255352
UIJ64569.1	CoA-binding_protein_[Bacillus_cereus]	CPTF_Cd	4493574.667	1170480.717	26.0478751
UIJ64569.1	CoA-binding_protein_[Bacillus_cereus]	CPTF_Co	3026263.667	470505.6146	15.54740982
UIJ64569.1	CoA-binding_protein_[Bacillus_cereus]	CPTF_Cu	3597813.667	241800.4796	6.720761608
UIJ64569.1	CoA-binding_protein_[Bacillus_cereus]	CPTF_Fe	3220778.6	31066.82897	0.964575118
UIJ64569.1	CoA-binding_protein_[Bacillus_cereus]	CPTF_Mn	3686432.233	554169.2533	15.03267165
UIJ64569.1	CoA-binding_protein_[Bacillus_cereus]	CPTF_Ni	2316620.333	170342.0483	7.353041234
UIJ64569.1	CoA-binding_protein_[Bacillus_cereus]	CPTF_U	2964198.3	257587.8691	8.689967506
UIJ64569.1	CoA-binding_protein_[Bacillus_cereus]	CPTF_metals_mix	3758785	256423.8145	6.821986747
UIJ64569.1	CoA-binding_protein_[Bacillus_cereus]	CPTF_zcontrol	4407768.667	1075971.492	24.41079768
UIJ64570.1	trypsin-like_peptidase_domain-containing_protein_[Bacillus_cereus]	CPTF_Al	1329737.533	193397.95	14.54406942
UIJ64570.1	trypsin-like_peptidase_domain-containing_protein_[Bacillus_cereus]	CPTF_Cd	1369271.567	143463.6126	10.47736739
UIJ64570.1	trypsin-like_peptidase_domain-containing_protein_[Bacillus_cereus]	CPTF_Co	1150578.533	191657.2066	16.65746414
UIJ64570.1	trypsin-like_peptidase_domain-containing_protein_[Bacillus_cereus]	CPTF_Cu	2834554.733	85968.57756	3.03287767
UIJ64570.1	trypsin-like_peptidase_domain-containing_protein_[Bacillus_cereus]	CPTF_Fe	1202968.167	199896.1565	16.61691157
UIJ64570.1	trypsin-like_peptidase_domain-containing_protein_[Bacillus_cereus]	CPTF_Mn	1088446.5	117340.7199	10.78056845
UIJ64570.1	trypsin-like_peptidase_domain-containing_protein_[Bacillus_cereus]	CPTF_Ni	872215.7667	363781.9253	41.70779057
UIJ64570.1	trypsin-like_peptidase_domain-containing_protein_[Bacillus_cereus]	CPTF_U	838462.1333	338581.265	40.38122314
UIJ64570.1	trypsin-like_peptidase_domain-containing_protein_[Bacillus_cereus]	CPTF_metals_mix	5154305.767	305232.4067	5.921891725
UIJ64570.1	trypsin-like_peptidase_domain-containing_protein_[Bacillus_cereus]	CPTF_zcontrol	1310315.333	109622.1587	8.366089893
UIJ64571.1	response_regulator_transcription_factor_[Bacillus_cereus]	CPTF_Al	1515865.167	300003.4664	19.79090707
UIJ64571.1	response_regulator_transcription_factor_[Bacillus_cereus]	CPTF_Cd	1336619.033	810670.3795	60.65081817
UIJ64571.1	response_regulator_transcription_factor_[Bacillus_cereus]	CPTF_Co	1385876.9	105206.774	7.591350574
UIJ64571.1	response_regulator_transcription_factor_[Bacillus_cereus]	CPTF_Cu	1389118.567	109417.8071	7.876779545
UIJ64571.1	response_regulator_transcription_factor_[Bacillus_cereus]	CPTF_Fe	1605016.533	202170.4978	12.59616294
UIJ64571.1	response_regulator_transcription_factor_[Bacillus_cereus]	CPTF_Mn	1633243.9	92289.37438	5.650679263
UIJ64571.1	response_regulator_transcription_factor_[Bacillus_cereus]	CPTF_Ni	1392852.2	61807.25392	4.437459619
UIJ64571.1	response_regulator_transcription_factor_[Bacillus_cereus]	CPTF_U	1642602.033	277849.1797	16.91518543
UIJ64571.1	response_regulator_transcription_factor_[Bacillus_cereus]	CPTF_metals_mix	1689981.567	53057.49212	3.139530819
UIJ64571.1	response_regulator_transcription_factor_[Bacillus_cereus]	CPTF_zcontrol	1521205.5	260640.542	17.13381538
UIJ64572.1	anaerobic_ribonucleoside-triphosphate_reductase_activating_protein_[Bacillus_cereus]	CPTF_Al	81426.36667	3953.618122	4.855451967
UIJ64572.1	anaerobic_ribonucleoside-triphosphate_reductase_activating_protein_[Bacillus_cereus]	CPTF_Cd	27415.93333	23753.73957	86.64209708
UIJ64572.1	anaerobic_ribonucleoside-triphosphate_reductase_activating_protein_[Bacillus_cereus]	CPTF_Co	36977.06667	23165.44193	62.64813307
UIJ64572.1	anaerobic_ribonucleoside-triphosphate_reductase_activating_protein_[Bacillus_cereus]	CPTF_Cu	27626.66667	26321.72975	95.27653142
UIJ64572.1	anaerobic_ribonucleoside-triphosphate_reductase_activating_protein_[Bacillus_cereus]	CPTF_Fe	50706.26667	44698.5246	88.15187459
UIJ64572.1	anaerobic_ribonucleoside-triphosphate_reductase_activating_protein_[Bacillus_cereus]	CPTF_Mn	65393.73333	13918.88249	21.28473445

UIJ64572.1	anaerobic_ribonucleoside-triphosphate_reductase_activating_protein_[Bacillus_cereus]	CPTF_Ni	45168.46667	15763.12905	34.89852592
UIJ64572.1	anaerobic_ribonucleoside-triphosphate_reductase_activating_protein_[Bacillus_cereus]	CPTF_U	0	0	0
UIJ64572.1	anaerobic_ribonucleoside-triphosphate_reductase_activating_protein_[Bacillus_cereus]	CPTF_metals_mix	4716.366667	8168.986694	173.2050808
UIJ64572.1	anaerobic_ribonucleoside-triphosphate_reductase_activating_protein_[Bacillus_cereus]	CPTF_zcontrol	62231.5	54302.86217	87.25944605
UIJ64573.1	anaerobic_ribonucleoside_triphosphate_reductase_[Bacillus_cereus]	CPTF_Al	776882.0333	128691.7645	16.56516163
UIJ64573.1	anaerobic_ribonucleoside_triphosphate_reductase_[Bacillus_cereus]	CPTF_Cd	599666.4333	133721.3582	22.29929021
UIJ64573.1	anaerobic_ribonucleoside_triphosphate_reductase_[Bacillus_cereus]	CPTF_Co	798002.4667	71300.01836	8.934811775
UIJ64573.1	anaerobic_ribonucleoside_triphosphate_reductase_[Bacillus_cereus]	CPTF_Cu	1025893.6	105388.8479	10.27288287
UIJ64573.1	anaerobic_ribonucleoside_triphosphate_reductase_[Bacillus_cereus]	CPTF_Fe	706971.7667	76119.36122	10.76695914
UIJ64573.1	anaerobic_ribonucleoside_triphosphate_reductase_[Bacillus_cereus]	CPTF_Mn	607789.8667	120703.1784	19.85936012
UIJ64573.1	anaerobic_ribonucleoside_triphosphate_reductase_[Bacillus_cereus]	CPTF_Ni	853489.8333	173434.7401	20.32065683
UIJ64573.1	anaerobic_ribonucleoside_triphosphate_reductase_[Bacillus_cereus]	CPTF_U	669010.4667	276912.6056	41.3913712
UIJ64573.1	anaerobic_ribonucleoside_triphosphate_reductase_[Bacillus_cereus]	CPTF_metals_mix	1277694.033	170742.1201	13.36330261
UIJ64573.1	anaerobic_ribonucleoside_triphosphate_reductase_[Bacillus_cereus]	CPTF_zcontrol	755222.5333	22959.56131	3.040105438
UIJ64574.1	glycerol-3-phosphate_1-O-acyltransferase_PlsY_[Bacillus_cereus]	CPTF_Al	56156.13333	8178.868486	14.56451504
UIJ64574.1	glycerol-3-phosphate_1-O-acyltransferase_PlsY_[Bacillus_cereus]	CPTF_Cd	48594.26667	12844.35246	26.43182692
UIJ64574.1	glycerol-3-phosphate_1-O-acyltransferase_PlsY_[Bacillus_cereus]	CPTF_Co	51435.73333	11122.16219	21.62341522
UIJ64574.1	glycerol-3-phosphate_1-O-acyltransferase_PlsY_[Bacillus_cereus]	CPTF_Cu	52984.2	4759.162066	8.982228789
UIJ64574.1	glycerol-3-phosphate_1-O-acyltransferase_PlsY_[Bacillus_cereus]	CPTF_Fe	48418.53333	7413.551407	15.31139193
UIJ64574.1	glycerol-3-phosphate_1-O-acyltransferase_PlsY_[Bacillus_cereus]	CPTF_Mn	39819.66667	35786.4859	89.87138492
UIJ64574.1	glycerol-3-phosphate_1-O-acyltransferase_PlsY_[Bacillus_cereus]	CPTF_Ni	38063.36667	33610.52718	88.30150911
UIJ64574.1	glycerol-3-phosphate_1-O-acyltransferase_PlsY_[Bacillus_cereus]	CPTF_U	27735.66667	27053.10971	97.53906418
UIJ64574.1	glycerol-3-phosphate_1-O-acyltransferase_PlsY_[Bacillus_cereus]	CPTF_metals_mix	37631.43333	6569.299284	17.45694677
UIJ64574.1	glycerol-3-phosphate_1-O-acyltransferase_PlsY_[Bacillus_cereus]	CPTF_zcontrol	45045.43333	6318.664382	14.02731401
UIJ64576.1	acyl-CoA_thioesterase_[Bacillus_cereus]	CPTF_Al	522920.3333	75715.09679	14.47927953
UIJ64576.1	acyl-CoA_thioesterase_[Bacillus_cereus]	CPTF_Cd	702549	21835.97424	3.108106942
UIJ64576.1	acyl-CoA_thioesterase_[Bacillus_cereus]	CPTF_Co	529457	86698.91797	16.37506312
UIJ64576.1	acyl-CoA_thioesterase_[Bacillus_cereus]	CPTF_Cu	531496.9667	28558.56652	5.373232268
UIJ64576.1	acyl-CoA_thioesterase_[Bacillus_cereus]	CPTF_Fe	572968.3333	82882.59306	14.46547536
UIJ64576.1	acyl-CoA_thioesterase_[Bacillus_cereus]	CPTF_Mn	349176.6667	325203.7309	93.13443936
UIJ64576.1	acyl-CoA_thioesterase_[Bacillus_cereus]	CPTF_Ni	106266	184058.1111	173.2050808
UIJ64576.1	acyl-CoA_thioesterase_[Bacillus_cereus]	CPTF_U	283534.6667	292439.5652	103.1406736
UIJ64576.1	acyl-CoA_thioesterase_[Bacillus_cereus]	CPTF_metals_mix	731348.9333	27833.95056	3.805837308
UIJ64576.1	acyl-CoA_thioesterase_[Bacillus_cereus]	CPTF_zcontrol	548042.6667	130171.1726	23.7520143
UIJ64582.1	aconitate_hydratase_AcnA_[Bacillus_cereus]	CPTF_Al	39958779.93	1664556.081	4.165682945
UIJ64582.1	aconitate_hydratase_AcnA_[Bacillus_cereus]	CPTF_Cd	40686447.13	1063517.89	2.613936495
UIJ64582.1	aconitate_hydratase_AcnA_[Bacillus_cereus]	CPTF_Co	38545995.9	4121095.327	10.69137074
UIJ64582.1	aconitate_hydratase_AcnA_[Bacillus_cereus]	CPTF_Cu	37427698.67	2439913.539	6.519004976
UIJ64582.1	aconitate_hydratase_AcnA_[Bacillus_cereus]	CPTF_Fe	39785849.93	724611.56	1.821279579
UIJ64582.1	aconitate_hydratase_AcnA_[Bacillus_cereus]	CPTF_Mn	40522366.23	6697633.426	16.52823872
UIJ64582.1	aconitate_hydratase_AcnA_[Bacillus_cereus]	CPTF_Ni	35669836.93	1519809.636	4.260769789
UIJ64582.1	aconitate_hydratase_AcnA_[Bacillus_cereus]	CPTF_U	31364576.43	1454259.475	4.636630366
UIJ64582.1	aconitate_hydratase_AcnA_[Bacillus_cereus]	CPTF_metals_mix	38944800.3	2729631.402	7.008975219
UIJ64582.1	aconitate_hydratase_AcnA_[Bacillus_cereus]	CPTF_zcontrol	37674479.4	1861130.013	4.940028482
UIJ64600.1	CsbD_family_protein_[Bacillus_cereus]	CPTF_Al	1268153.267	114653.8671	9.041010269
UIJ64600.1	CsbD_family_protein_[Bacillus_cereus]	CPTF_Cd	1197722.733	149337.1742	12.46842613
UIJ64600.1	CsbD_family_protein_[Bacillus_cereus]	CPTF_Co	1117003.167	134916.9723	12.07847715
UIJ64600.1	CsbD_family_protein_[Bacillus_cereus]	CPTF_Cu	1406322.467	96441.05495	6.857677185
UIJ64600.1	CsbD_family_protein_[Bacillus_cereus]	CPTF_Fe	1249709.667	111167.3855	8.895456959
UIJ64600.1	CsbD_family_protein_[Bacillus_cereus]	CPTF_Mn	1042794	93988.32757	9.013125082
UIJ64600.1	CsbD_family_protein_[Bacillus_cereus]	CPTF_Ni	1299550	160636.2077	12.36091014
UIJ64600.1	CsbD_family_protein_[Bacillus_cereus]	CPTF_U	1382498.333	563572.4483	40.7647832
UIJ64600.1	CsbD_family_protein_[Bacillus_cereus]	CPTF_metals_mix	1395382.333	21802.15369	1.562450173
UIJ64600.1	CsbD_family_protein_[Bacillus_cereus]	CPTF_zcontrol	939306	316372.4121	33.68150657
UIJ64612.1	alkene_reductase_[Bacillus_cereus]	CPTF_Al	0	0	0
UIJ64612.1	alkene_reductase_[Bacillus_cereus]	CPTF_Cd	30775.16667	53304.15228	173.2050808
UIJ64612.1	alkene_reductase_[Bacillus_cereus]	CPTF_Co	0	0	0
UIJ64612.1	alkene_reductase_[Bacillus_cereus]	CPTF_Cu	10039.16667	17388.34673	173.2050808
UIJ64612.1	alkene_reductase_[Bacillus_cereus]	CPTF_Fe	0	0	0
UIJ64612.1	alkene_reductase_[Bacillus_cereus]	CPTF_Mn	13521.4	23419.75179	173.2050808
UIJ64612.1	alkene_reductase_[Bacillus_cereus]	CPTF_Ni	11662.76667	20200.50442	173.2050808

UIJ64612.1	alkene_reductase_[Bacillus_cereus]	CPTF_U	0	0	0
UIJ64612.1	alkene_reductase_[Bacillus_cereus]	CPTF_metals_mix	92939.16667	38064.76386	40.95664425
UIJ64612.1	alkene_reductase_[Bacillus_cereus]	CPTF_zcontrol	0	0	0
UIJ64615.1	imidazolonepropionase_[Bacillus_cereus]	CPTF_Al	194052	20323.24991	10.4730948
UIJ64615.1	imidazolonepropionase_[Bacillus_cereus]	CPTF_Cd	234702	49062.17391	20.9040289
UIJ64615.1	imidazolonepropionase_[Bacillus_cereus]	CPTF_Co	157506.0333	88696.4257	56.31303374
UIJ64615.1	imidazolonepropionase_[Bacillus_cereus]	CPTF_Cu	67510.56667	62326.62322	92.32128584
UIJ64615.1	imidazolonepropionase_[Bacillus_cereus]	CPTF_Fe	200483.7667	41260.60044	20.58051937
UIJ64615.1	imidazolonepropionase_[Bacillus_cereus]	CPTF_Mn	122590.1333	109601.8979	89.40515429
UIJ64615.1	imidazolonepropionase_[Bacillus_cereus]	CPTF_Ni	128522.6667	111987.6226	87.13453084
UIJ64615.1	imidazolonepropionase_[Bacillus_cereus]	CPTF_U	202937.9333	177799.7469	87.61286958
UIJ64615.1	imidazolonepropionase_[Bacillus_cereus]	CPTF_metals_mix	0	0	0
UIJ64615.1	imidazolonepropionase_[Bacillus_cereus]	CPTF_zcontrol	292685.6667	89238.04996	30.48938165
UIJ64616.1	urocanate_hydratase_[Bacillus_cereus]	CPTF_Al	1786848.233	249846.4121	13.98252003
UIJ64616.1	urocanate_hydratase_[Bacillus_cereus]	CPTF_Cd	1595063.233	102676.0167	6.43711262
UIJ64616.1	urocanate_hydratase_[Bacillus_cereus]	CPTF_Co	1717457.033	103686.9275	6.037235603
UIJ64616.1	urocanate_hydratase_[Bacillus_cereus]	CPTF_Cu	1396961.9	188340.3526	13.48213954
UIJ64616.1	urocanate_hydratase_[Bacillus_cereus]	CPTF_Fe	1544906.333	373020.5593	24.14518934
UIJ64616.1	urocanate_hydratase_[Bacillus_cereus]	CPTF_Mn	1549544.233	249052.4851	16.07262831
UIJ64616.1	urocanate_hydratase_[Bacillus_cereus]	CPTF_Ni	1693523.667	229089.4486	13.52738395
UIJ64616.1	urocanate_hydratase_[Bacillus_cereus]	CPTF_U	1369393.667	156087.8635	11.39831937
UIJ64616.1	urocanate_hydratase_[Bacillus_cereus]	CPTF_metals_mix	831814.3667	119508.6021	14.36722025
UIJ64616.1	urocanate_hydratase_[Bacillus_cereus]	CPTF_zcontrol	1893386.7	102499.0519	5.413529727
UIJ64617.1	hut_operon_transcriptional_regulator_HutP_[Bacillus_cereus]	CPTF_Al	0	0	0
UIJ64617.1	hut_operon_transcriptional_regulator_HutP_[Bacillus_cereus]	CPTF_Cd	21665.53333	37525.80451	173.2050808
UIJ64617.1	hut_operon_transcriptional_regulator_HutP_[Bacillus_cereus]	CPTF_Co	0	0	0
UIJ64617.1	hut_operon_transcriptional_regulator_HutP_[Bacillus_cereus]	CPTF_Cu	19502.2	33778.80126	173.2050808
UIJ64617.1	hut_operon_transcriptional_regulator_HutP_[Bacillus_cereus]	CPTF_Fe	28202.1	48847.47008	173.2050808
UIJ64617.1	hut_operon_transcriptional_regulator_HutP_[Bacillus_cereus]	CPTF_Mn	0	0	0
UIJ64617.1	hut_operon_transcriptional_regulator_HutP_[Bacillus_cereus]	CPTF_Ni	0	0	0
UIJ64617.1	hut_operon_transcriptional_regulator_HutP_[Bacillus_cereus]	CPTF_U	17136.83333	29681.86601	173.2050808
UIJ64617.1	hut_operon_transcriptional_regulator_HutP_[Bacillus_cereus]	CPTF_metals_mix	91670.66667	86139.02032	93.96574002
UIJ64617.1	hut_operon_transcriptional_regulator_HutP_[Bacillus_cereus]	CPTF_zcontrol	23897.86667	41392.31926	173.2050808
UIJ64621.1	hypothetical_protein_LW858_16650_[Bacillus_cereus]	CPTF_Al	0	0	0
UIJ64621.1	hypothetical_protein_LW858_16650_[Bacillus_cereus]	CPTF_Cd	0	0	0
UIJ64621.1	hypothetical_protein_LW858_16650_[Bacillus_cereus]	CPTF_Co	41818.1	43323.08213	103.5988774
UIJ64621.1	hypothetical_protein_LW858_16650_[Bacillus_cereus]	CPTF_Cu	38019.96667	35228.54451	92.65801
UIJ64621.1	hypothetical_protein_LW858_16650_[Bacillus_cereus]	CPTF_Fe	36034.66667	62413.8735	173.2050808
UIJ64621.1	hypothetical_protein_LW858_16650_[Bacillus_cereus]	CPTF_Mn	16052.56667	27803.86106	173.2050808
UIJ64621.1	hypothetical_protein_LW858_16650_[Bacillus_cereus]	CPTF_Ni	19450.66667	19147.89512	98.44338729
UIJ64621.1	hypothetical_protein_LW858_16650_[Bacillus_cereus]	CPTF_U	24555.5	42531.37361	173.2050808
UIJ64621.1	hypothetical_protein_LW858_16650_[Bacillus_cereus]	CPTF_metals_mix	18006.6	31188.34607	173.2050808
UIJ64621.1	hypothetical_protein_LW858_16650_[Bacillus_cereus]	CPTF_zcontrol	10510.7	18205.06642	173.2050808
UIJ64640.1	YneF_family_protein_[Bacillus_cereus]	CPTF_Al	454476.3333	49653.66482	10.92546766
UIJ64640.1	YneF_family_protein_[Bacillus_cereus]	CPTF_Cd	467124.3333	61669.17634	13.20187623
UIJ64640.1	YneF_family_protein_[Bacillus_cereus]	CPTF_Co	630861.3333	50686.79381	8.03453804
UIJ64640.1	YneF_family_protein_[Bacillus_cereus]	CPTF_Cu	669211	22534.94901	3.367390705
UIJ64640.1	YneF_family_protein_[Bacillus_cereus]	CPTF_Fe	474947	95060.11167	20.01488833
UIJ64640.1	YneF_family_protein_[Bacillus_cereus]	CPTF_Mn	482793.3333	26127.69619	5.411776507
UIJ64640.1	YneF_family_protein_[Bacillus_cereus]	CPTF_Ni	572691.3333	58109.06293	10.14666358
UIJ64640.1	YneF_family_protein_[Bacillus_cereus]	CPTF_U	423433	110898.3715	26.19029963
UIJ64640.1	YneF_family_protein_[Bacillus_cereus]	CPTF_metals_mix	807052.6667	38426.9617	4.761394551
UIJ64640.1	YneF_family_protein_[Bacillus_cereus]	CPTF_zcontrol	526838.6667	38566.447	7.320352404
UIJ64642.1	transketolase_[Bacillus_cereus]	CPTF_Al	17802388.17	540430.8648	3.035721162
UIJ64642.1	transketolase_[Bacillus_cereus]	CPTF_Cd	18368080.17	68985.68638	0.375573744
UIJ64642.1	transketolase_[Bacillus_cereus]	CPTF_Co	18476804.33	465119.3471	2.517314892
UIJ64642.1	transketolase_[Bacillus_cereus]	CPTF_Cu	18433281.03	250727.2751	1.360187992
UIJ64642.1	transketolase_[Bacillus_cereus]	CPTF_Fe	18053903.8	1033778.011	5.726063583
UIJ64642.1	transketolase_[Bacillus_cereus]	CPTF_Mn	18621564.87	1994030.907	10.7081812
UIJ64642.1	transketolase_[Bacillus_cereus]	CPTF_Ni	15351756.37	721455.2482	4.699496468
UIJ64642.1	transketolase_[Bacillus_cereus]	CPTF_U	14832030.43	1371136.101	9.244426155

UIJ64642.1	transketolase_[Bacillus_cereus]	CPTF_metals_mix	22715530.27	527801.5864	2.323527473
UIJ64642.1	transketolase_[Bacillus_cereus]	CPTF_zcontrol	17508398.87	912227.822	5.210229838
UIJ64646.1	DUF896_domain-containing_protein_[Bacillus_cereus]	CPTF_Al	105951.3333	92079.39722	86.90725668
UIJ64646.1	DUF896_domain-containing_protein_[Bacillus_cereus]	CPTF_Cd	205583.6667	24288.12914	11.81423093
UIJ64646.1	DUF896_domain-containing_protein_[Bacillus_cereus]	CPTF_Co	137865.1	44806.7362	32.50041976
UIJ64646.1	DUF896_domain-containing_protein_[Bacillus_cereus]	CPTF_Cu	104001.6667	90105.70578	86.63871327
UIJ64646.1	DUF896_domain-containing_protein_[Bacillus_cereus]	CPTF_Fe	116386	111594.3662	95.88298093
UIJ64646.1	DUF896_domain-containing_protein_[Bacillus_cereus]	CPTF_Mn	169325.6667	43598.01058	25.7480224
UIJ64646.1	DUF896_domain-containing_protein_[Bacillus_cereus]	CPTF_Ni	0	0	0
UIJ64646.1	DUF896_domain-containing_protein_[Bacillus_cereus]	CPTF_U	0	0	0
UIJ64646.1	DUF896_domain-containing_protein_[Bacillus_cereus]	CPTF_metals_mix	589479	84180.39766	14.2804744
UIJ64646.1	DUF896_domain-containing_protein_[Bacillus_cereus]	CPTF_zcontrol	103606.3333	90814.06574	87.65300616
UIJ64648.1	transcriptional_repressor_LexA_[Bacillus_cereus]	CPTF_Al	1012747.767	13585.90863	1.341489863
UIJ64648.1	transcriptional_repressor_LexA_[Bacillus_cereus]	CPTF_Cd	1139826.067	104305.0246	9.150959754
UIJ64648.1	transcriptional_repressor_LexA_[Bacillus_cereus]	CPTF_Co	936839.3333	58544.59826	6.249161001
UIJ64648.1	transcriptional_repressor_LexA_[Bacillus_cereus]	CPTF_Cu	899175.7333	122363.1937	13.60837367
UIJ64648.1	transcriptional_repressor_LexA_[Bacillus_cereus]	CPTF_Fe	1160924.8	71280.23986	6.139953239
UIJ64648.1	transcriptional_repressor_LexA_[Bacillus_cereus]	CPTF_Mn	805463.1667	577918.1493	71.74979233
UIJ64648.1	transcriptional_repressor_LexA_[Bacillus_cereus]	CPTF_Ni	984651.7667	117530.223	11.93622222
UIJ64648.1	transcriptional_repressor_LexA_[Bacillus_cereus]	CPTF_U	1228110.667	115862.4923	9.434206172
UIJ64648.1	transcriptional_repressor_LexA_[Bacillus_cereus]	CPTF_metals_mix	818416	92760.59865	11.33416241
UIJ64648.1	transcriptional_repressor_LexA_[Bacillus_cereus]	CPTF_zcontrol	1083721.667	63567.79668	5.865693991
UIJ64659.1	type_I_glutamate--ammonia_ligase_[Bacillus_cereus]	CPTF_Al	9770089.233	1012860.162	10.36694894
UIJ64659.1	type_I_glutamate--ammonia_ligase_[Bacillus_cereus]	CPTF_Cd	10120730.43	755439.9663	7.46428305
UIJ64659.1	type_I_glutamate--ammonia_ligase_[Bacillus_cereus]	CPTF_Co	10209888.77	728952.4735	7.13967106
UIJ64659.1	type_I_glutamate--ammonia_ligase_[Bacillus_cereus]	CPTF_Cu	10046681.8	673557.7681	6.704280891
UIJ64659.1	type_I_glutamate--ammonia_ligase_[Bacillus_cereus]	CPTF_Fe	10350908.87	625448.4991	6.04245006
UIJ64659.1	type_I_glutamate--ammonia_ligase_[Bacillus_cereus]	CPTF_Mn	10806140.67	437919.7351	4.052508186
UIJ64659.1	type_I_glutamate--ammonia_ligase_[Bacillus_cereus]	CPTF_Ni	10164206.63	548273.3742	5.394158088
UIJ64659.1	type_I_glutamate--ammonia_ligase_[Bacillus_cereus]	CPTF_U	10670999	1542142.676	14.45171793
UIJ64659.1	type_I_glutamate--ammonia_ligase_[Bacillus_cereus]	CPTF_metals_mix	11343743.83	1465801.656	12.92167452
UIJ64659.1	type_I_glutamate--ammonia_ligase_[Bacillus_cereus]	CPTF_zcontrol	10421341.23	95264.89228	0.914132741
UIJ64665.1	tyrosine-type_recombinase/integrase_[Bacillus_cereus]	CPTF_Al	44235.96667	42901.15429	96.9825179
UIJ64665.1	tyrosine-type_recombinase/integrase_[Bacillus_cereus]	CPTF_Cd	92917.13333	16819.03701	18.10111484
UIJ64665.1	tyrosine-type_recombinase/integrase_[Bacillus_cereus]	CPTF_Co	99036.26667	90805.19646	91.6888323
UIJ64665.1	tyrosine-type_recombinase/integrase_[Bacillus_cereus]	CPTF_Cu	161971.6333	44794.19586	27.65558076
UIJ64665.1	tyrosine-type_recombinase/integrase_[Bacillus_cereus]	CPTF_Fe	73869.73333	19970.62971	27.03492866
UIJ64665.1	tyrosine-type_recombinase/integrase_[Bacillus_cereus]	CPTF_Mn	19630.16667	34000.44603	173.2050808
UIJ64665.1	tyrosine-type_recombinase/integrase_[Bacillus_cereus]	CPTF_Ni	38859.8	33882.85782	87.19256872
UIJ64665.1	tyrosine-type_recombinase/integrase_[Bacillus_cereus]	CPTF_U	0	0	0
UIJ64665.1	tyrosine-type_recombinase/integrase_[Bacillus_cereus]	CPTF_metals_mix	275987.9667	79322.81893	28.74140489
UIJ64665.1	tyrosine-type_recombinase/integrase_[Bacillus_cereus]	CPTF_zcontrol	33644.8	29143.88547	86.62225804
UIJ64666.1	RNA_chaperone_Hfq_[Bacillus_cereus]	CPTF_Al	332772.3333	292575.5316	87.9206299
UIJ64666.1	RNA_chaperone_Hfq_[Bacillus_cereus]	CPTF_Cd	65528.43333	73829.28869	112.6675627
UIJ64666.1	RNA_chaperone_Hfq_[Bacillus_cereus]	CPTF_Co	401518.3333	107680.8766	26.81842089
UIJ64666.1	RNA_chaperone_Hfq_[Bacillus_cereus]	CPTF_Cu	474937	70363.43071	14.81531881
UIJ64666.1	RNA_chaperone_Hfq_[Bacillus_cereus]	CPTF_Fe	278800.6	264270.1822	94.78824012
UIJ64666.1	RNA_chaperone_Hfq_[Bacillus_cereus]	CPTF_Mn	114503	198325.0136	173.2050808
UIJ64666.1	RNA_chaperone_Hfq_[Bacillus_cereus]	CPTF_Ni	183898.2667	318521.1413	173.2050808
UIJ64666.1	RNA_chaperone_Hfq_[Bacillus_cereus]	CPTF_U	265409.5333	289827.9858	109.2002921
UIJ64666.1	RNA_chaperone_Hfq_[Bacillus_cereus]	CPTF_metals_mix	844777.2	103498.2645	12.25154567
UIJ64666.1	RNA_chaperone_Hfq_[Bacillus_cereus]	CPTF_zcontrol	118191.8333	140774.0901	119.1064442
UIJ64667.1	tRNA_(adenosine(37)-N6)-dimethylallyltransferase_MiaA_[Bacillus_cereus]	CPTF_Al	0	0	0
UIJ64667.1	tRNA_(adenosine(37)-N6)-dimethylallyltransferase_MiaA_[Bacillus_cereus]	CPTF_Cd	0	0	0
UIJ64667.1	tRNA_(adenosine(37)-N6)-dimethylallyltransferase_MiaA_[Bacillus_cereus]	CPTF_Co	0	0	0
UIJ64667.1	tRNA_(adenosine(37)-N6)-dimethylallyltransferase_MiaA_[Bacillus_cereus]	CPTF_Cu	9903.733333	17153.76932	173.2050808
UIJ64667.1	tRNA_(adenosine(37)-N6)-dimethylallyltransferase_MiaA_[Bacillus_cereus]	CPTF_Fe	0	0	0
UIJ64667.1	tRNA_(adenosine(37)-N6)-dimethylallyltransferase_MiaA_[Bacillus_cereus]	CPTF_Mn	0	0	0
UIJ64667.1	tRNA_(adenosine(37)-N6)-dimethylallyltransferase_MiaA_[Bacillus_cereus]	CPTF_Ni	0	0	0
UIJ64667.1	tRNA_(adenosine(37)-N6)-dimethylallyltransferase_MiaA_[Bacillus_cereus]	CPTF_U	0	0	0
UIJ64667.1	tRNA_(adenosine(37)-N6)-dimethylallyltransferase_MiaA_[Bacillus_cereus]	CPTF_metals_mix	35142.53333	10571.44607	30.08162779

UIJ64667.1	tRNA_(adenosine(37)-N6)-dimethylallyltransferase_MiaA_[Bacillus_cereus]	CPTF_zcontrol	0	0	0
UIJ64673.1	iron-containing_alcohol_dehydrogenase_[Bacillus_cereus]	CPTF_Al	513799.3667	107650.995	20.95195168
UIJ64673.1	iron-containing_alcohol_dehydrogenase_[Bacillus_cereus]	CPTF_Cd	482779.6667	102210.1025	21.1711697
UIJ64673.1	iron-containing_alcohol_dehydrogenase_[Bacillus_cereus]	CPTF_Co	442968	31078.95087	7.016071334
UIJ64673.1	iron-containing_alcohol_dehydrogenase_[Bacillus_cereus]	CPTF_Cu	670792.4333	127788.6066	19.05039476
UIJ64673.1	iron-containing_alcohol_dehydrogenase_[Bacillus_cereus]	CPTF_Fe	523483.3333	103019.5797	19.67962934
UIJ64673.1	iron-containing_alcohol_dehydrogenase_[Bacillus_cereus]	CPTF_Mn	420698.2667	99840.42855	23.73207509
UIJ64673.1	iron-containing_alcohol_dehydrogenase_[Bacillus_cereus]	CPTF_Ni	514565.3333	207674.4964	40.35920863
UIJ64673.1	iron-containing_alcohol_dehydrogenase_[Bacillus_cereus]	CPTF_U	650359.6667	69032.39657	10.61449535
UIJ64673.1	iron-containing_alcohol_dehydrogenase_[Bacillus_cereus]	CPTF_metals_mix	963853.4	67344.4503	6.987001374
UIJ64673.1	iron-containing_alcohol_dehydrogenase_[Bacillus_cereus]	CPTF_zcontrol	577938	65181.41108	11.27827052
UIJ64680.1	HU_family_DNA-binding_protein_[Bacillus_cereus]	CPTF_Al	644481.4067	35779.22147	5.551629745
UIJ64680.1	HU_family_DNA-binding_protein_[Bacillus_cereus]	CPTF_Cd	597502.6667	162760.4076	27.240114
UIJ64680.1	HU_family_DNA-binding_protein_[Bacillus_cereus]	CPTF_Co	716137.6	82130.09917	11.46848024
UIJ64680.1	HU_family_DNA-binding_protein_[Bacillus_cereus]	CPTF_Cu	847873.3333	67878.41967	8.005726445
UIJ64680.1	HU_family_DNA-binding_protein_[Bacillus_cereus]	CPTF_Fe	521652.9	143918.0143	27.58884583
UIJ64680.1	HU_family_DNA-binding_protein_[Bacillus_cereus]	CPTF_Mn	519474.4333	67341.58532	12.96340705
UIJ64680.1	HU_family_DNA-binding_protein_[Bacillus_cereus]	CPTF_Ni	671826.9667	55620.39114	8.278975674
UIJ64680.1	HU_family_DNA-binding_protein_[Bacillus_cereus]	CPTF_U	358094.6667	42308.6534	11.81493536
UIJ64680.1	HU_family_DNA-binding_protein_[Bacillus_cereus]	CPTF_metals_mix	899542.0333	121301.7692	13.48483614
UIJ64680.1	HU_family_DNA-binding_protein_[Bacillus_cereus]	CPTF_zcontrol	503635.1667	99825.459	19.82098662
UIJ64682.1	heavy_metal_translocating_P-type_ATPase_[Bacillus_cereus]	CPTF_Al	0	0	0
UIJ64682.1	heavy_metal_translocating_P-type_ATPase_[Bacillus_cereus]	CPTF_Cd	0	0	0
UIJ64682.1	heavy_metal_translocating_P-type_ATPase_[Bacillus_cereus]	CPTF_Co	0	0	0
UIJ64682.1	heavy_metal_translocating_P-type_ATPase_[Bacillus_cereus]	CPTF_Cu	0	0	0
UIJ64682.1	heavy_metal_translocating_P-type_ATPase_[Bacillus_cereus]	CPTF_Fe	0	0	0
UIJ64682.1	heavy_metal_translocating_P-type_ATPase_[Bacillus_cereus]	CPTF_Mn	0	0	0
UIJ64682.1	heavy_metal_translocating_P-type_ATPase_[Bacillus_cereus]	CPTF_Ni	0	0	0
UIJ64682.1	heavy_metal_translocating_P-type_ATPase_[Bacillus_cereus]	CPTF_U	0	0	0
UIJ64682.1	heavy_metal_translocating_P-type_ATPase_[Bacillus_cereus]	CPTF_metals_mix	61160.86667	22309.70835	36.47709649
UIJ64682.1	heavy_metal_translocating_P-type_ATPase_[Bacillus_cereus]	CPTF_zcontrol	0	0	0
UIJ64683.1	copper_chaperone_CopZ_[Bacillus_cereus]	CPTF_Al	0	0	0
UIJ64683.1	copper_chaperone_CopZ_[Bacillus_cereus]	CPTF_Cd	7578.4	13126.17384	173.2050808
UIJ64683.1	copper_chaperone_CopZ_[Bacillus_cereus]	CPTF_Co	18677.4	16361.82117	87.60224214
UIJ64683.1	copper_chaperone_CopZ_[Bacillus_cereus]	CPTF_Cu	126007.6667	109587.139	86.96862806
UIJ64683.1	copper_chaperone_CopZ_[Bacillus_cereus]	CPTF_Fe	0	0	0
UIJ64683.1	copper_chaperone_CopZ_[Bacillus_cereus]	CPTF_Mn	0	0	0
UIJ64683.1	copper_chaperone_CopZ_[Bacillus_cereus]	CPTF_Ni	57373.86667	22999.73536	40.08747658
UIJ64683.1	copper_chaperone_CopZ_[Bacillus_cereus]	CPTF_U	0	0	0
UIJ64683.1	copper_chaperone_CopZ_[Bacillus_cereus]	CPTF_metals_mix	418628.3333	49950.09496	11.93184765
UIJ64683.1	copper_chaperone_CopZ_[Bacillus_cereus]	CPTF_zcontrol	0	0	0
UIJ64690.1	iron-hydroxamate_ABC_transporter_substrate-binding_protein_[Bacillus_cereus]	CPTF_Al	0	0	0
UIJ64690.1	iron-hydroxamate_ABC_transporter_substrate-binding_protein_[Bacillus_cereus]	CPTF_Cd	52392.96667	53902.62358	102.8814114
UIJ64690.1	iron-hydroxamate_ABC_transporter_substrate-binding_protein_[Bacillus_cereus]	CPTF_Co	52560.33333	91037.1678	173.2050808
UIJ64690.1	iron-hydroxamate_ABC_transporter_substrate-binding_protein_[Bacillus_cereus]	CPTF_Cu	1057220.4	121993.1932	11.53905025
UIJ64690.1	iron-hydroxamate_ABC_transporter_substrate-binding_protein_[Bacillus_cereus]	CPTF_Fe	0	0	0
UIJ64690.1	iron-hydroxamate_ABC_transporter_substrate-binding_protein_[Bacillus_cereus]	CPTF_Mn	54598.03333	60980.08811	111.6891661
UIJ64690.1	iron-hydroxamate_ABC_transporter_substrate-binding_protein_[Bacillus_cereus]	CPTF_Ni	0	0	0
UIJ64690.1	iron-hydroxamate_ABC_transporter_substrate-binding_protein_[Bacillus_cereus]	CPTF_U	0	0	0
UIJ64690.1	iron-hydroxamate_ABC_transporter_substrate-binding_protein_[Bacillus_cereus]	CPTF_metals_mix	1934260.533	72555.35151	3.751064051
UIJ64690.1	iron-hydroxamate_ABC_transporter_substrate-binding_protein_[Bacillus_cereus]	CPTF_zcontrol	0	0	0
UIJ64695.1	peptidase_T_[Bacillus_cereus]	CPTF_Al	801034.3667	70908.13567	8.852071599
UIJ64695.1	peptidase_T_[Bacillus_cereus]	CPTF_Cd	816874.1333	28898.31492	3.537670461
UIJ64695.1	peptidase_T_[Bacillus_cereus]	CPTF_Co	540809.6667	128404.5316	23.74301709
UIJ64695.1	peptidase_T_[Bacillus_cereus]	CPTF_Cu	574867.0667	84783.40978	14.74835048
UIJ64695.1	peptidase_T_[Bacillus_cereus]	CPTF_Fe	670649.3667	83946.04083	12.51712818
UIJ64695.1	peptidase_T_[Bacillus_cereus]	CPTF_Mn	516760.8333	106977.8154	20.7016106
UIJ64695.1	peptidase_T_[Bacillus_cereus]	CPTF_Ni	467366.3333	104177.9581	22.29042845
UIJ64695.1	peptidase_T_[Bacillus_cereus]	CPTF_U	419871.9667	188236.6296	44.83191175
UIJ64695.1	peptidase_T_[Bacillus_cereus]	CPTF_metals_mix	1006478.733	153796.986	15.28069902
UIJ64695.1	peptidase_T_[Bacillus_cereus]	CPTF_zcontrol	536448.1333	79030.92727	14.73225879

UIJ64720.1	DNA_mismatch_repair_endonuclease_MutL_[Bacillus_cereus]	CPTF_Al	0	0	0
UIJ64720.1	DNA_mismatch_repair_endonuclease_MutL_[Bacillus_cereus]	CPTF_Cd	7343.466667	12719.25737	173.2050808
UIJ64720.1	DNA_mismatch_repair_endonuclease_MutL_[Bacillus_cereus]	CPTF_Co	0	0	0
UIJ64720.1	DNA_mismatch_repair_endonuclease_MutL_[Bacillus_cereus]	CPTF_Cu	0	0	0
UIJ64720.1	DNA_mismatch_repair_endonuclease_MutL_[Bacillus_cereus]	CPTF_Fe	0	0	0
UIJ64720.1	DNA_mismatch_repair_endonuclease_MutL_[Bacillus_cereus]	CPTF_Mn	0	0	0
UIJ64720.1	DNA_mismatch_repair_endonuclease_MutL_[Bacillus_cereus]	CPTF_Ni	0	0	0
UIJ64720.1	DNA_mismatch_repair_endonuclease_MutL_[Bacillus_cereus]	CPTF_U	0	0	0
UIJ64720.1	DNA_mismatch_repair_endonuclease_MutL_[Bacillus_cereus]	CPTF_metals_mix	94162.6	24601.46896	26.12658206
UIJ64720.1	DNA_mismatch_repair_endonuclease_MutL_[Bacillus_cereus]	CPTF_zcontrol	0	0	0
UIJ64721.1	DNA_mismatch_repair_protein_MutS_[Bacillus_cereus]	CPTF_Al	444210.3333	46165.70775	10.39275863
UIJ64721.1	DNA_mismatch_repair_protein_MutS_[Bacillus_cereus]	CPTF_Cd	434400.3667	10225.08625	2.353839231
UIJ64721.1	DNA_mismatch_repair_protein_MutS_[Bacillus_cereus]	CPTF_Co	390446.9	31142.56863	7.976134176
UIJ64721.1	DNA_mismatch_repair_protein_MutS_[Bacillus_cereus]	CPTF_Cu	437118.6667	103565.5661	23.69278048
UIJ64721.1	DNA_mismatch_repair_protein_MutS_[Bacillus_cereus]	CPTF_Fe	437727.3333	25898.38652	5.916556849
UIJ64721.1	DNA_mismatch_repair_protein_MutS_[Bacillus_cereus]	CPTF_Mn	388991.5667	25817.43894	6.637017651
UIJ64721.1	DNA_mismatch_repair_protein_MutS_[Bacillus_cereus]	CPTF_Ni	321889.4667	38389.59018	11.92632694
UIJ64721.1	DNA_mismatch_repair_protein_MutS_[Bacillus_cereus]	CPTF_U	388346.7667	26024.51241	6.701359364
UIJ64721.1	DNA_mismatch_repair_protein_MutS_[Bacillus_cereus]	CPTF_metals_mix	284748.9	22911.67868	8.046274693
UIJ64721.1	DNA_mismatch_repair_protein_MutS_[Bacillus_cereus]	CPTF_zcontrol	418895.5333	28895.55887	6.89803461
UIJ64724.1	tRNA_(N6-isopentenyl_adenosine(37)-C2)-methylthiotransferase_MiaB_[Bacillus_cereus]	CPTF_Al	1158247.133	280156.3317	24.18795814
UIJ64724.1	tRNA_(N6-isopentenyl_adenosine(37)-C2)-methylthiotransferase_MiaB_[Bacillus_cereus]	CPTF_Cd	1391208.633	241654.6319	17.37012164
UIJ64724.1	tRNA_(N6-isopentenyl_adenosine(37)-C2)-methylthiotransferase_MiaB_[Bacillus_cereus]	CPTF_Co	1274054.667	268468.3695	21.07196626
UIJ64724.1	tRNA_(N6-isopentenyl_adenosine(37)-C2)-methylthiotransferase_MiaB_[Bacillus_cereus]	CPTF_Cu	1170559.733	368328.732	31.46603471
UIJ64724.1	tRNA_(N6-isopentenyl_adenosine(37)-C2)-methylthiotransferase_MiaB_[Bacillus_cereus]	CPTF_Fe	1079778	400194.1175	37.06262931
UIJ64724.1	tRNA_(N6-isopentenyl_adenosine(37)-C2)-methylthiotransferase_MiaB_[Bacillus_cereus]	CPTF_Mn	1147376.5	501821.4129	43.73642069
UIJ64724.1	tRNA_(N6-isopentenyl_adenosine(37)-C2)-methylthiotransferase_MiaB_[Bacillus_cereus]	CPTF_Ni	1115628.133	179320.4713	16.0734985
UIJ64724.1	tRNA_(N6-isopentenyl_adenosine(37)-C2)-methylthiotransferase_MiaB_[Bacillus_cereus]	CPTF_U	917977.6667	222836.4597	24.27471471
UIJ64724.1	tRNA_(N6-isopentenyl_adenosine(37)-C2)-methylthiotransferase_MiaB_[Bacillus_cereus]	CPTF_metals_mix	1563639.1	231535.832	14.80749823
UIJ64724.1	tRNA_(N6-isopentenyl_adenosine(37)-C2)-methylthiotransferase_MiaB_[Bacillus_cereus]	CPTF_zcontrol	787007.8	575402.0197	73.11261968
UIJ64725.1	2-oxoacid:ferredoxin_oxidoreductase_subunit_beta_[Bacillus_cereus]	CPTF_Al	1326086.1	424392.0062	32.00335229
UIJ64725.1	2-oxoacid:ferredoxin_oxidoreductase_subunit_beta_[Bacillus_cereus]	CPTF_Cd	905682.9667	142361.5095	15.71869128
UIJ64725.1	2-oxoacid:ferredoxin_oxidoreductase_subunit_beta_[Bacillus_cereus]	CPTF_Co	1160003.4	610016.7375	52.58749565
UIJ64725.1	2-oxoacid:ferredoxin_oxidoreductase_subunit_beta_[Bacillus_cereus]	CPTF_Cu	1461016.433	947793.7447	64.87221657
UIJ64725.1	2-oxoacid:ferredoxin_oxidoreductase_subunit_beta_[Bacillus_cereus]	CPTF_Fe	945402.7667	549948.6438	58.17083081
UIJ64725.1	2-oxoacid:ferredoxin_oxidoreductase_subunit_beta_[Bacillus_cereus]	CPTF_Mn	1266174.433	541463.6867	42.76375138
UIJ64725.1	2-oxoacid:ferredoxin_oxidoreductase_subunit_beta_[Bacillus_cereus]	CPTF_Ni	961134.8333	360386.8546	37.49597268
UIJ64725.1	2-oxoacid:ferredoxin_oxidoreductase_subunit_beta_[Bacillus_cereus]	CPTF_U	807310.0667	48633.49146	6.024140348
UIJ64725.1	2-oxoacid:ferredoxin_oxidoreductase_subunit_beta_[Bacillus_cereus]	CPTF_metals_mix	1434029.967	607414.2151	42.35714938
UIJ64725.1	2-oxoacid:ferredoxin_oxidoreductase_subunit_beta_[Bacillus_cereus]	CPTF_zcontrol	1124649.433	485177.0804	43.14029474
UIJ64726.1	2-oxoacid:acceptor_oxidoreductase_subunit_alpha_[Bacillus_cereus]	CPTF_Al	6318594.333	203246.987	3.216648771
UIJ64726.1	2-oxoacid:acceptor_oxidoreductase_subunit_alpha_[Bacillus_cereus]	CPTF_Cd	6133859	175975.6623	2.868922522
UIJ64726.1	2-oxoacid:acceptor_oxidoreductase_subunit_alpha_[Bacillus_cereus]	CPTF_Co	5779206.233	406583.4244	7.035281455
UIJ64726.1	2-oxoacid:acceptor_oxidoreductase_subunit_alpha_[Bacillus_cereus]	CPTF_Cu	6506998.667	295617.2434	4.54306599
UIJ64726.1	2-oxoacid:acceptor_oxidoreductase_subunit_alpha_[Bacillus_cereus]	CPTF_Fe	5960237.2	319819.5308	5.365885955
UIJ64726.1	2-oxoacid:acceptor_oxidoreductase_subunit_alpha_[Bacillus_cereus]	CPTF_Mn	5884803.333	630615.372	10.7159974
UIJ64726.1	2-oxoacid:acceptor_oxidoreductase_subunit_alpha_[Bacillus_cereus]	CPTF_Ni	6372356.333	317640.0895	4.984656741
UIJ64726.1	2-oxoacid:acceptor_oxidoreductase_subunit_alpha_[Bacillus_cereus]	CPTF_U	6006466.367	748374.5118	12.4594806
UIJ64726.1	2-oxoacid:acceptor_oxidoreductase_subunit_alpha_[Bacillus_cereus]	CPTF_metals_mix	6784586.367	292984.0511	4.318377499
UIJ64726.1	2-oxoacid:acceptor_oxidoreductase_subunit_alpha_[Bacillus_cereus]	CPTF_zcontrol	6096010.667	226974.759	3.723332707
UIJ64728.1	stage_V_sporulation_protein_SpoVS_[Bacillus_cereus]	CPTF_Al	3392491.667	503974.5744	14.85558769
UIJ64728.1	stage_V_sporulation_protein_SpoVS_[Bacillus_cereus]	CPTF_Cd	4081393.667	450277.4638	11.03244383
UIJ64728.1	stage_V_sporulation_protein_SpoVS_[Bacillus_cereus]	CPTF_Co	3415782	282656.1134	8.275004476
UIJ64728.1	stage_V_sporulation_protein_SpoVS_[Bacillus_cereus]	CPTF_Cu	2772034.233	391577.3332	14.12599197
UIJ64728.1	stage_V_sporulation_protein_SpoVS_[Bacillus_cereus]	CPTF_Fe	3458490.333	110390.7794	3.191877632
UIJ64728.1	stage_V_sporulation_protein_SpoVS_[Bacillus_cereus]	CPTF_Mn	2989079.333	810698.4696	27.12201247
UIJ64728.1	stage_V_sporulation_protein_SpoVS_[Bacillus_cereus]	CPTF_Ni	2082183.367	528315.6649	25.37315749
UIJ64728.1	stage_V_sporulation_protein_SpoVS_[Bacillus_cereus]	CPTF_U	1798703.533	524727.4476	29.17253666
UIJ64728.1	stage_V_sporulation_protein_SpoVS_[Bacillus_cereus]	CPTF_metals_mix	2255307.567	52318.52053	2.319795371
UIJ64728.1	stage_V_sporulation_protein_SpoVS_[Bacillus_cereus]	CPTF_zcontrol	2612351.933	215787.646	8.260282363
UIJ64729.1	TIGR00282_family_metallophosphoesterase_[Bacillus_cereus]	CPTF_Al	488543	239310.9492	48.98462349

UIJ64729.1	TIGR00282_family_metallophosphoesterase_[Bacillus_cereus]	CPTF_Cd	258476.9667	208356.6434	80.60936573
UIJ64729.1	TIGR00282_family_metallophosphoesterase_[Bacillus_cereus]	CPTF_Co	353455.5667	228020.0123	64.51164836
UIJ64729.1	TIGR00282_family_metallophosphoesterase_[Bacillus_cereus]	CPTF_Cu	335373.8333	239933.6457	71.54214844
UIJ64729.1	TIGR00282_family_metallophosphoesterase_[Bacillus_cereus]	CPTF_Fe	402456.1333	26023.65561	6.466209222
UIJ64729.1	TIGR00282_family_metallophosphoesterase_[Bacillus_cereus]	CPTF_Mn	544148.4	54367.2449	9.991253287
UIJ64729.1	TIGR00282_family_metallophosphoesterase_[Bacillus_cereus]	CPTF_Ni	296033.3333	227341.8359	76.79602611
UIJ64729.1	TIGR00282_family_metallophosphoesterase_[Bacillus_cereus]	CPTF_U	475250.7667	112912.441	23.75849739
UIJ64729.1	TIGR00282_family_metallophosphoesterase_[Bacillus_cereus]	CPTF_metals_mix	353306.9	175650.4614	49.71611406
UIJ64729.1	TIGR00282_family_metallophosphoesterase_[Bacillus_cereus]	CPTF_zcontrol	577532.6667	227369.8073	39.36916825
UIJ64730.1	ribonuclease_Y_[Bacillus_cereus]	CPTF_Al	1243994	282608.0769	22.71780064
UIJ64730.1	ribonuclease_Y_[Bacillus_cereus]	CPTF_Cd	1172162.6	351119.9322	29.95488272
UIJ64730.1	ribonuclease_Y_[Bacillus_cereus]	CPTF_Co	1460615.567	160748.8027	11.0055518
UIJ64730.1	ribonuclease_Y_[Bacillus_cereus]	CPTF_Cu	1273865.533	414375.0283	32.52894575
UIJ64730.1	ribonuclease_Y_[Bacillus_cereus]	CPTF_Fe	1054593.333	240494.7788	22.804504
UIJ64730.1	ribonuclease_Y_[Bacillus_cereus]	CPTF_Mn	1234357.767	218619.9339	17.71122925
UIJ64730.1	ribonuclease_Y_[Bacillus_cereus]	CPTF_Ni	1011060.067	255898.9046	25.3099606
UIJ64730.1	ribonuclease_Y_[Bacillus_cereus]	CPTF_U	1020794.667	301924.6709	29.57741462
UIJ64730.1	ribonuclease_Y_[Bacillus_cereus]	CPTF_metals_mix	1855680.833	219373.1956	11.8217094
UIJ64730.1	ribonuclease_Y_[Bacillus_cereus]	CPTF_zcontrol	1227129.933	219312.3073	17.87197112
UIJ64731.1	recombinase_RecA_[Bacillus_cereus]	CPTF_Al	2478987.367	140388.1999	5.663126881
UIJ64731.1	recombinase_RecA_[Bacillus_cereus]	CPTF_Cd	2515762.667	94121.78581	3.741282398
UIJ64731.1	recombinase_RecA_[Bacillus_cereus]	CPTF_Co	2484120.367	426161.3956	17.15542457
UIJ64731.1	recombinase_RecA_[Bacillus_cereus]	CPTF_Cu	2410754.633	112515.0453	4.667212653
UIJ64731.1	recombinase_RecA_[Bacillus_cereus]	CPTF_Fe	2519044.7	120275.8433	4.774660937
UIJ64731.1	recombinase_RecA_[Bacillus_cereus]	CPTF_Mn	2428024.6	287313.8235	11.83323363
UIJ64731.1	recombinase_RecA_[Bacillus_cereus]	CPTF_Ni	2132141.2	231910.5022	10.87688293
UIJ64731.1	recombinase_RecA_[Bacillus_cereus]	CPTF_U	2348814.867	318471.0475	13.55879733
UIJ64731.1	recombinase_RecA_[Bacillus_cereus]	CPTF_metals_mix	2733129.467	268768.0153	9.833709622
UIJ64731.1	recombinase_RecA_[Bacillus_cereus]	CPTF_zcontrol	2156543.217	189558.1727	8.789908369
UIJ64732.1	competence/damage-inducible_protein_CinA_[Bacillus_cereus]	CPTF_Al	474877.3667	114189.2309	24.04604619
UIJ64732.1	competence/damage-inducible_protein_CinA_[Bacillus_cereus]	CPTF_Cd	425017.3333	158896.6789	37.38592909
UIJ64732.1	competence/damage-inducible_protein_CinA_[Bacillus_cereus]	CPTF_Co	734118.6	69046.94662	9.405421224
UIJ64732.1	competence/damage-inducible_protein_CinA_[Bacillus_cereus]	CPTF_Cu	427887.7667	269927.8623	63.08379985
UIJ64732.1	competence/damage-inducible_protein_CinA_[Bacillus_cereus]	CPTF_Fe	579361.5	262719.0012	45.34629954
UIJ64732.1	competence/damage-inducible_protein_CinA_[Bacillus_cereus]	CPTF_Mn	452028.1333	55248.21282	12.2222952
UIJ64732.1	competence/damage-inducible_protein_CinA_[Bacillus_cereus]	CPTF_Ni	529470.5667	276375.6519	52.1984921
UIJ64732.1	competence/damage-inducible_protein_CinA_[Bacillus_cereus]	CPTF_U	282957.7	82523.61166	29.16464605
UIJ64732.1	competence/damage-inducible_protein_CinA_[Bacillus_cereus]	CPTF_metals_mix	830420.0667	150177.709	18.08454721
UIJ64732.1	competence/damage-inducible_protein_CinA_[Bacillus_cereus]	CPTF_zcontrol	236837.0667	165872.4839	70.03653872
UIJ64734.1	DUF4115_domain-containing_protein_[Bacillus_cereus]	CPTF_Al	160364.0667	28367.82179	17.68963733
UIJ64734.1	DUF4115_domain-containing_protein_[Bacillus_cereus]	CPTF_Cd	172521.2	66598.28385	38.60295653
UIJ64734.1	DUF4115_domain-containing_protein_[Bacillus_cereus]	CPTF_Co	141361.8667	66605.74545	47.11719434
UIJ64734.1	DUF4115_domain-containing_protein_[Bacillus_cereus]	CPTF_Cu	170679.1333	20044.28491	11.74384034
UIJ64734.1	DUF4115_domain-containing_protein_[Bacillus_cereus]	CPTF_Fe	159851.9667	73496.00773	45.97754364
UIJ64734.1	DUF4115_domain-containing_protein_[Bacillus_cereus]	CPTF_Mn	100833.7667	98020.56332	97.21005825
UIJ64734.1	DUF4115_domain-containing_protein_[Bacillus_cereus]	CPTF_Ni	143693.8333	50863.88528	35.39740301
UIJ64734.1	DUF4115_domain-containing_protein_[Bacillus_cereus]	CPTF_U	51383.1	57941.06642	112.7628859
UIJ64734.1	DUF4115_domain-containing_protein_[Bacillus_cereus]	CPTF_metals_mix	162322.3667	21415.12594	13.19296063
UIJ64734.1	DUF4115_domain-containing_protein_[Bacillus_cereus]	CPTF_zcontrol	140583.9	28641.06313	20.37293255
UIJ64735.1	DUF3388_domain-containing_protein_[Bacillus_cereus]	CPTF_Al	0	0	0
UIJ64735.1	DUF3388_domain-containing_protein_[Bacillus_cereus]	CPTF_Cd	0	0	0
UIJ64735.1	DUF3388_domain-containing_protein_[Bacillus_cereus]	CPTF_Co	0	0	0
UIJ64735.1	DUF3388_domain-containing_protein_[Bacillus_cereus]	CPTF_Cu	0	0	0
UIJ64735.1	DUF3388_domain-containing_protein_[Bacillus_cereus]	CPTF_Fe	0	0	0
UIJ64735.1	DUF3388_domain-containing_protein_[Bacillus_cereus]	CPTF_Mn	0	0	0
UIJ64735.1	DUF3388_domain-containing_protein_[Bacillus_cereus]	CPTF_Ni	0	0	0
UIJ64735.1	DUF3388_domain-containing_protein_[Bacillus_cereus]	CPTF_U	17706.23333	30668.09574	173.2050808
UIJ64735.1	DUF3388_domain-containing_protein_[Bacillus_cereus]	CPTF_metals_mix	0	0	0
UIJ64735.1	DUF3388_domain-containing_protein_[Bacillus_cereus]	CPTF_zcontrol	0	0	0
UIJ64738.1	insulinase_family_protein_[Bacillus_cereus]	CPTF_Al	0	0	0
UIJ64738.1	insulinase_family_protein_[Bacillus_cereus]	CPTF_Cd	64473.9	17645.24589	27.36804488

UIJ64738.1	insulinase_family_protein_[Bacillus_cereus]	CPTF_Co	16866.6	29213.80815	173.2050808
UIJ64738.1	insulinase_family_protein_[Bacillus_cereus]	CPTF_Cu	22268.6	38570.34661	173.2050808
UIJ64738.1	insulinase_family_protein_[Bacillus_cereus]	CPTF_Fe	23307.3	40369.42779	173.2050808
UIJ64738.1	insulinase_family_protein_[Bacillus_cereus]	CPTF_Mn	20532.2	35562.81359	173.2050808
UIJ64738.1	insulinase_family_protein_[Bacillus_cereus]	CPTF_Ni	0	0	0
UIJ64738.1	insulinase_family_protein_[Bacillus_cereus]	CPTF_U	0	0	0
UIJ64738.1	insulinase_family_protein_[Bacillus_cereus]	CPTF_metals_mix	116756.8	39926.48816	34.19628506
UIJ64738.1	insulinase_family_protein_[Bacillus_cereus]	CPTF_zcontrol	0	0	0
UIJ64739.1	insulinase_family_protein_[Bacillus_cereus]	CPTF_Al	104797.3333	119123.9959	113.6708274
UIJ64739.1	insulinase_family_protein_[Bacillus_cereus]	CPTF_Cd	166077.3333	37065.27804	22.31808357
UIJ64739.1	insulinase_family_protein_[Bacillus_cereus]	CPTF_Co	79420	70079.8002	88.23948653
UIJ64739.1	insulinase_family_protein_[Bacillus_cereus]	CPTF_Cu	258541	61084.00268	23.62642779
UIJ64739.1	insulinase_family_protein_[Bacillus_cereus]	CPTF_Fe	121350.6667	107416.7117	88.51761153
UIJ64739.1	insulinase_family_protein_[Bacillus_cereus]	CPTF_Mn	115231	115908.3355	100.5878067
UIJ64739.1	insulinase_family_protein_[Bacillus_cereus]	CPTF_Ni	29473.23333	51049.1376	173.2050808
UIJ64739.1	insulinase_family_protein_[Bacillus_cereus]	CPTF_U	82244	78942.47536	95.98569544
UIJ64739.1	insulinase_family_protein_[Bacillus_cereus]	CPTF_metals_mix	778431.3	151720.4437	19.4905374
UIJ64739.1	insulinase_family_protein_[Bacillus_cereus]	CPTF_zcontrol	126791.3333	26009.17723	20.5133715
UIJ64740.1	ABC_transporter_permease_[Bacillus_cereus]	CPTF_Al	109473.6667	94812.50088	86.60758679
UIJ64740.1	ABC_transporter_permease_[Bacillus_cereus]	CPTF_Cd	55991	96979.25677	173.2050808
UIJ64740.1	ABC_transporter_permease_[Bacillus_cereus]	CPTF_Co	58755.33333	51798.01271	88.15882707
UIJ64740.1	ABC_transporter_permease_[Bacillus_cereus]	CPTF_Cu	85783.86667	94436.44454	110.0864862
UIJ64740.1	ABC_transporter_permease_[Bacillus_cereus]	CPTF_Fe	120947.8333	54403.91722	44.98130783
UIJ64740.1	ABC_transporter_permease_[Bacillus_cereus]	CPTF_Mn	0	0	0
UIJ64740.1	ABC_transporter_permease_[Bacillus_cereus]	CPTF_Ni	32717.63333	56668.60324	173.2050808
UIJ64740.1	ABC_transporter_permease_[Bacillus_cereus]	CPTF_U	0	0	0
UIJ64740.1	ABC_transporter_permease_[Bacillus_cereus]	CPTF_metals_mix	286570.1	84552.51541	29.50500258
UIJ64740.1	ABC_transporter_permease_[Bacillus_cereus]	CPTF_zcontrol	0	0	0
UIJ64742.1	ABC_transporter_ATP-binding_protein_[Bacillus_cereus]	CPTF_Al	2476971.5	12654.24067	0.510875506
UIJ64742.1	ABC_transporter_ATP-binding_protein_[Bacillus_cereus]	CPTF_Cd	2041827.3	443656.3188	21.72839587
UIJ64742.1	ABC_transporter_ATP-binding_protein_[Bacillus_cereus]	CPTF_Co	2377713.033	213748.7648	8.989678815
UIJ64742.1	ABC_transporter_ATP-binding_protein_[Bacillus_cereus]	CPTF_Cu	1382610.567	20842.40606	1.507467581
UIJ64742.1	ABC_transporter_ATP-binding_protein_[Bacillus_cereus]	CPTF_Fe	2181099.467	728127.2594	33.38349628
UIJ64742.1	ABC_transporter_ATP-binding_protein_[Bacillus_cereus]	CPTF_Mn	2472354.1	179307.7658	7.252511516
UIJ64742.1	ABC_transporter_ATP-binding_protein_[Bacillus_cereus]	CPTF_Ni	1754699.333	538533.387	30.69092105
UIJ64742.1	ABC_transporter_ATP-binding_protein_[Bacillus_cereus]	CPTF_U	2187496.233	437767.1705	20.01224797
UIJ64742.1	ABC_transporter_ATP-binding_protein_[Bacillus_cereus]	CPTF_metals_mix	1880504.067	201105.7161	10.69424521
UIJ64742.1	ABC_transporter_ATP-binding_protein_[Bacillus_cereus]	CPTF_zcontrol	2769467.733	251526.6865	9.082130962
UIJ64743.1	BMP_family_ABC_transporter_substrate-binding_protein_[Bacillus_cereus]	CPTF_Al	24907376.17	998137.2777	4.007396327
UIJ64743.1	BMP_family_ABC_transporter_substrate-binding_protein_[Bacillus_cereus]	CPTF_Cd	24232182.73	784817.5847	3.238740783
UIJ64743.1	BMP_family_ABC_transporter_substrate-binding_protein_[Bacillus_cereus]	CPTF_Co	24154036.87	901317.1237	3.731538246
UIJ64743.1	BMP_family_ABC_transporter_substrate-binding_protein_[Bacillus_cereus]	CPTF_Cu	20721274.7	271912.7706	1.312239592
UIJ64743.1	BMP_family_ABC_transporter_substrate-binding_protein_[Bacillus_cereus]	CPTF_Fe	23321289.4	1111848.54	4.76752602
UIJ64743.1	BMP_family_ABC_transporter_substrate-binding_protein_[Bacillus_cereus]	CPTF_Mn	24487990.1	1702841.829	6.953783557
UIJ64743.1	BMP_family_ABC_transporter_substrate-binding_protein_[Bacillus_cereus]	CPTF_Ni	22154201.93	1371416.598	6.190322732
UIJ64743.1	BMP_family_ABC_transporter_substrate-binding_protein_[Bacillus_cereus]	CPTF_U	20111793.33	2476531.23	12.31382597
UIJ64743.1	BMP_family_ABC_transporter_substrate-binding_protein_[Bacillus_cereus]	CPTF_metals_mix	21831958.13	722710.8124	3.310334364
UIJ64743.1	BMP_family_ABC_transporter_substrate-binding_protein_[Bacillus_cereus]	CPTF_zcontrol	23921152	425374.9078	1.778237552
UIJ64744.1	GntR_family_transcriptional_regulator_[Bacillus_cereus]	CPTF_Al	2124076.167	159081.8345	7.489459984
UIJ64744.1	GntR_family_transcriptional_regulator_[Bacillus_cereus]	CPTF_Cd	1838380.6	236145.9726	12.84532553
UIJ64744.1	GntR_family_transcriptional_regulator_[Bacillus_cereus]	CPTF_Co	1935513.667	156548.4011	8.088209544
UIJ64744.1	GntR_family_transcriptional_regulator_[Bacillus_cereus]	CPTF_Cu	1688941.867	35668.87862	2.111906829
UIJ64744.1	GntR_family_transcriptional_regulator_[Bacillus_cereus]	CPTF_Fe	1990934.967	72321.60324	3.632544732
UIJ64744.1	GntR_family_transcriptional_regulator_[Bacillus_cereus]	CPTF_Mn	1942736.333	291105.2855	14.98429203
UIJ64744.1	GntR_family_transcriptional_regulator_[Bacillus_cereus]	CPTF_Ni	1888848.167	273932.7602	14.50263526
UIJ64744.1	GntR_family_transcriptional_regulator_[Bacillus_cereus]	CPTF_U	1925368.133	426610.1223	22.15732747
UIJ64744.1	GntR_family_transcriptional_regulator_[Bacillus_cereus]	CPTF_metals_mix	1688815.633	193622.1996	11.46496964
UIJ64744.1	GntR_family_transcriptional_regulator_[Bacillus_cereus]	CPTF_zcontrol	2227803.267	74530.15988	3.345455184
UIJ64748.1	ribonuclease_J_[Bacillus_cereus]	CPTF_Al	742152	77511.34964	10.44413404
UIJ64748.1	ribonuclease_J_[Bacillus_cereus]	CPTF_Cd	754866.7	148781.8427	19.70968419
UIJ64748.1	ribonuclease_J_[Bacillus_cereus]	CPTF_Co	949265.6667	53558.98031	5.642148683

UIJ64748.1	ribonuclease_J_[Bacillus_cereus]	CPTF_Cu	787665.7333	124773.5474	15.84092618
UIJ64748.1	ribonuclease_J_[Bacillus_cereus]	CPTF_Fe	704494.3333	21181.86548	3.006676488
UIJ64748.1	ribonuclease_J_[Bacillus_cereus]	CPTF_Mn	670838.4	236823.6234	35.30263375
UIJ64748.1	ribonuclease_J_[Bacillus_cereus]	CPTF_Ni	638614	5208.544998	0.815601443
UIJ64748.1	ribonuclease_J_[Bacillus_cereus]	CPTF_U	596295.3333	178730.949	29.97356158
UIJ64748.1	ribonuclease_J_[Bacillus_cereus]	CPTF_metals_mix	1274150.9	132334.3307	10.38607991
UIJ64748.1	ribonuclease_J_[Bacillus_cereus]	CPTF_zcontrol	664866	119834.6086	18.02387377
UIJ64749.1	4-hydroxy-tetrahydrodipicolinate_synthase_[Bacillus_cereus]	CPTF_Al	3908374.667	32952.31061	0.843120566
UIJ64749.1	4-hydroxy-tetrahydrodipicolinate_synthase_[Bacillus_cereus]	CPTF_Cd	3644310.667	325039.2411	8.919087061
UIJ64749.1	4-hydroxy-tetrahydrodipicolinate_synthase_[Bacillus_cereus]	CPTF_Co	4184430.033	144229.4567	3.446812483
UIJ64749.1	4-hydroxy-tetrahydrodipicolinate_synthase_[Bacillus_cereus]	CPTF_Cu	4907276.633	251294.7094	5.120858843
UIJ64749.1	4-hydroxy-tetrahydrodipicolinate_synthase_[Bacillus_cereus]	CPTF_Fe	3380518.1	418995.4897	12.3944164
UIJ64749.1	4-hydroxy-tetrahydrodipicolinate_synthase_[Bacillus_cereus]	CPTF_Mn	3666854	902131.5169	24.60232987
UIJ64749.1	4-hydroxy-tetrahydrodipicolinate_synthase_[Bacillus_cereus]	CPTF_Ni	3833327.233	291698.3177	7.60953344
UIJ64749.1	4-hydroxy-tetrahydrodipicolinate_synthase_[Bacillus_cereus]	CPTF_U	2634687.667	391254.6981	14.85013587
UIJ64749.1	4-hydroxy-tetrahydrodipicolinate_synthase_[Bacillus_cereus]	CPTF_metals_mix	6472858.8	250087.4179	3.863631598
UIJ64749.1	4-hydroxy-tetrahydrodipicolinate_synthase_[Bacillus_cereus]	CPTF_zcontrol	3562197.333	164111.7527	4.6070371
UIJ64750.1	aspartate_kinase_[Bacillus_cereus]	CPTF_Al	140301.3333	16574.92095	11.81380145
UIJ64750.1	aspartate_kinase_[Bacillus_cereus]	CPTF_Cd	108921.1	32107.86723	29.47809674
UIJ64750.1	aspartate_kinase_[Bacillus_cereus]	CPTF_Co	151206.3333	15464.34655	10.22731404
UIJ64750.1	aspartate_kinase_[Bacillus_cereus]	CPTF_Cu	167954.3333	9183.056372	5.467591214
UIJ64750.1	aspartate_kinase_[Bacillus_cereus]	CPTF_Fe	118975.6667	14111.43367	11.86077294
UIJ64750.1	aspartate_kinase_[Bacillus_cereus]	CPTF_Mn	144088.1333	31933.2698	22.16231764
UIJ64750.1	aspartate_kinase_[Bacillus_cereus]	CPTF_Ni	186811.4	22924.47107	12.27145188
UIJ64750.1	aspartate_kinase_[Bacillus_cereus]	CPTF_U	96521.66667	83620.47807	86.63389367
UIJ64750.1	aspartate_kinase_[Bacillus_cereus]	CPTF_metals_mix	517212.2333	331336.8824	64.06207377
UIJ64750.1	aspartate_kinase_[Bacillus_cereus]	CPTF_zcontrol	134270.8333	65366.94129	48.68290429
UIJ64751.1	aspartate-semialdehyde_dehydrogenase_[Bacillus_cereus]	CPTF_Al	10132207.83	513547.4388	5.068465307
UIJ64751.1	aspartate-semialdehyde_dehydrogenase_[Bacillus_cereus]	CPTF_Cd	9602751	681427.5002	7.096169631
UIJ64751.1	aspartate-semialdehyde_dehydrogenase_[Bacillus_cereus]	CPTF_Co	10303161.07	250432.6872	2.43063935
UIJ64751.1	aspartate-semialdehyde_dehydrogenase_[Bacillus_cereus]	CPTF_Cu	10805509.23	953175.9881	8.821203772
UIJ64751.1	aspartate-semialdehyde_dehydrogenase_[Bacillus_cereus]	CPTF_Fe	9481475.4	699257.762	7.374988939
UIJ64751.1	aspartate-semialdehyde_dehydrogenase_[Bacillus_cereus]	CPTF_Mn	9319967.633	1128865.795	12.11233601
UIJ64751.1	aspartate-semialdehyde_dehydrogenase_[Bacillus_cereus]	CPTF_Ni	9394970.2	1033730.528	11.00302083
UIJ64751.1	aspartate-semialdehyde_dehydrogenase_[Bacillus_cereus]	CPTF_U	8134977.2	926111.979	11.38432175
UIJ64751.1	aspartate-semialdehyde_dehydrogenase_[Bacillus_cereus]	CPTF_metals_mix	12745299.33	165592.5372	1.299244003
UIJ64751.1	aspartate-semialdehyde_dehydrogenase_[Bacillus_cereus]	CPTF_zcontrol	9351700.8	389231.7598	4.162149411
UIJ64755.1	insulinase_family_protein_[Bacillus_cereus]	CPTF_Al	289798.6667	150413.4555	51.90274241
UIJ64755.1	insulinase_family_protein_[Bacillus_cereus]	CPTF_Cd	378498.5	20959.7344	5.537600386
UIJ64755.1	insulinase_family_protein_[Bacillus_cereus]	CPTF_Co	405816.4333	105225.5045	25.92933549
UIJ64755.1	insulinase_family_protein_[Bacillus_cereus]	CPTF_Cu	502060.3	144885.7718	28.85824109
UIJ64755.1	insulinase_family_protein_[Bacillus_cereus]	CPTF_Fe	440684.6667	39660.49022	8.99974363
UIJ64755.1	insulinase_family_protein_[Bacillus_cereus]	CPTF_Mn	308259.2	73665.46684	23.89724843
UIJ64755.1	insulinase_family_protein_[Bacillus_cereus]	CPTF_Ni	200293.8667	77997.64896	38.94160628
UIJ64755.1	insulinase_family_protein_[Bacillus_cereus]	CPTF_U	190033.9333	79932.74175	42.06235189
UIJ64755.1	insulinase_family_protein_[Bacillus_cereus]	CPTF_metals_mix	776127.5	66371.18202	8.551582313
UIJ64755.1	insulinase_family_protein_[Bacillus_cereus]	CPTF_zcontrol	267118.7667	51908.43899	19.43271887
UIJ64757.1	polyribonucleotide_nucleotidyltransferase_[Bacillus_cereus]	CPTF_Al	6115733.1	444527.6717	7.268591753
UIJ64757.1	polyribonucleotide_nucleotidyltransferase_[Bacillus_cereus]	CPTF_Cd	5756523	718280.0847	12.47767245
UIJ64757.1	polyribonucleotide_nucleotidyltransferase_[Bacillus_cereus]	CPTF_Co	6810878.267	438464.8937	6.43771444
UIJ64757.1	polyribonucleotide_nucleotidyltransferase_[Bacillus_cereus]	CPTF_Cu	6000840.1	761439.3614	12.68887937
UIJ64757.1	polyribonucleotide_nucleotidyltransferase_[Bacillus_cereus]	CPTF_Fe	6137957.067	139971.8759	2.280431003
UIJ64757.1	polyribonucleotide_nucleotidyltransferase_[Bacillus_cereus]	CPTF_Mn	5710970.6	763160.5729	13.36306254
UIJ64757.1	polyribonucleotide_nucleotidyltransferase_[Bacillus_cereus]	CPTF_Ni	5622925	990762.9176	17.62006282
UIJ64757.1	polyribonucleotide_nucleotidyltransferase_[Bacillus_cereus]	CPTF_U	4652272.567	533861.5936	11.47528624
UIJ64757.1	polyribonucleotide_nucleotidyltransferase_[Bacillus_cereus]	CPTF_metals_mix	5550606.7	188163.2821	3.389958834
UIJ64757.1	polyribonucleotide_nucleotidyltransferase_[Bacillus_cereus]	CPTF_zcontrol	5602904.467	370843.4394	6.618771418
UIJ64758.1	30S_ribosomal_protein_S15_[Bacillus_cereus]	CPTF_Al	6870144.633	858439.0958	12.49521141
UIJ64758.1	30S_ribosomal_protein_S15_[Bacillus_cereus]	CPTF_Cd	7545554.867	455182.2305	6.03245538
UIJ64758.1	30S_ribosomal_protein_S15_[Bacillus_cereus]	CPTF_Co	7832340.867	243312.9637	3.10651653
UIJ64758.1	30S_ribosomal_protein_S15_[Bacillus_cereus]	CPTF_Cu	6589557.233	507205.621	7.69711231

UIJ64758.1	30S_ribosomal_protein_S15_[Bacillus_cereus]	CPTF_Fe	7277651	881312.8966	12.10985381
UIJ64758.1	30S_ribosomal_protein_S15_[Bacillus_cereus]	CPTF_Mn	7655760.333	1655184.203	21.6201152
UIJ64758.1	30S_ribosomal_protein_S15_[Bacillus_cereus]	CPTF_Ni	5661642.333	538739.1428	9.51559832
UIJ64758.1	30S_ribosomal_protein_S15_[Bacillus_cereus]	CPTF_U	4518644.233	2467695.835	54.61142121
UIJ64758.1	30S_ribosomal_protein_S15_[Bacillus_cereus]	CPTF_metals_mix	5963192.667	341767.9993	5.731292252
UIJ64758.1	30S_ribosomal_protein_S15_[Bacillus_cereus]	CPTF_zcontrol	6708049	313503.3958	4.673540635
UIJ64759.1	bifunctional_riboflavin_kinase/FAD_synthetase_[Bacillus_cereus]	CPTF_Al	0	0	0
UIJ64759.1	bifunctional_riboflavin_kinase/FAD_synthetase_[Bacillus_cereus]	CPTF_Cd	18695.43333	32381.4404	173.2050808
UIJ64759.1	bifunctional_riboflavin_kinase/FAD_synthetase_[Bacillus_cereus]	CPTF_Co	0	0	0
UIJ64759.1	bifunctional_riboflavin_kinase/FAD_synthetase_[Bacillus_cereus]	CPTF_Cu	0	0	0
UIJ64759.1	bifunctional_riboflavin_kinase/FAD_synthetase_[Bacillus_cereus]	CPTF_Fe	0	0	0
UIJ64759.1	bifunctional_riboflavin_kinase/FAD_synthetase_[Bacillus_cereus]	CPTF_Mn	12956.3	22440.96988	173.2050808
UIJ64759.1	bifunctional_riboflavin_kinase/FAD_synthetase_[Bacillus_cereus]	CPTF_Ni	0	0	0
UIJ64759.1	bifunctional_riboflavin_kinase/FAD_synthetase_[Bacillus_cereus]	CPTF_U	0	0	0
UIJ64759.1	bifunctional_riboflavin_kinase/FAD_synthetase_[Bacillus_cereus]	CPTF_metals_mix	74575.03333	23376.42846	31.34618574
UIJ64759.1	bifunctional_riboflavin_kinase/FAD_synthetase_[Bacillus_cereus]	CPTF_zcontrol	0	0	0
UIJ64761.1	30S_ribosome-binding_factor_RbfA_[Bacillus_cereus]	CPTF_Al	8857.5	15341.64003	173.2050808
UIJ64761.1	30S_ribosome-binding_factor_RbfA_[Bacillus_cereus]	CPTF_Cd	45378.6	19645.20467	43.29178219
UIJ64761.1	30S_ribosome-binding_factor_RbfA_[Bacillus_cereus]	CPTF_Co	396973.9333	653634.7492	164.6543247
UIJ64761.1	30S_ribosome-binding_factor_RbfA_[Bacillus_cereus]	CPTF_Cu	670110.6667	598784.8289	89.35611067
UIJ64761.1	30S_ribosome-binding_factor_RbfA_[Bacillus_cereus]	CPTF_Fe	23807.43333	23552.28696	98.92829113
UIJ64761.1	30S_ribosome-binding_factor_RbfA_[Bacillus_cereus]	CPTF_Mn	506719.2667	851679.5898	168.0772068
UIJ64761.1	30S_ribosome-binding_factor_RbfA_[Bacillus_cereus]	CPTF_Ni	513195	559703.4698	109.0625337
UIJ64761.1	30S_ribosome-binding_factor_RbfA_[Bacillus_cereus]	CPTF_U	869235.3333	606570.9441	69.78213158
UIJ64761.1	30S_ribosome-binding_factor_RbfA_[Bacillus_cereus]	CPTF_metals_mix	239462.1667	78458.30827	32.76438586
UIJ64761.1	30S_ribosome-binding_factor_RbfA_[Bacillus_cereus]	CPTF_zcontrol	399339.3333	544827.8163	136.4322948
UIJ64763.1	translation_initiation_factor_IF-2_[Bacillus_cereus]	CPTF_Al	6018703.3	865852.6431	14.386033
UIJ64763.1	translation_initiation_factor_IF-2_[Bacillus_cereus]	CPTF_Cd	7294290.2	264930.913	3.632031434
UIJ64763.1	translation_initiation_factor_IF-2_[Bacillus_cereus]	CPTF_Co	5792196.8	521323.0555	9.000437545
UIJ64763.1	translation_initiation_factor_IF-2_[Bacillus_cereus]	CPTF_Cu	4918057.1	24250.55272	0.493092134
UIJ64763.1	translation_initiation_factor_IF-2_[Bacillus_cereus]	CPTF_Fe	6106343.033	1519128.709	24.87788028
UIJ64763.1	translation_initiation_factor_IF-2_[Bacillus_cereus]	CPTF_Mn	6943817.4	2925183.327	42.12644369
UIJ64763.1	translation_initiation_factor_IF-2_[Bacillus_cereus]	CPTF_Ni	3463053.567	755725.1084	21.82250704
UIJ64763.1	translation_initiation_factor_IF-2_[Bacillus_cereus]	CPTF_U	3780558.667	1182652.306	31.28247463
UIJ64763.1	translation_initiation_factor_IF-2_[Bacillus_cereus]	CPTF_metals_mix	13507608.8	1198386.371	8.871935726
UIJ64763.1	translation_initiation_factor_IF-2_[Bacillus_cereus]	CPTF_zcontrol	5628891.467	648519.6509	11.52126764
UIJ64765.1	YlxR_family_protein_[Bacillus_cereus]	CPTF_Al	101913.0667	146468.6672	143.7192227
UIJ64765.1	YlxR_family_protein_[Bacillus_cereus]	CPTF_Cd	0	0	0
UIJ64765.1	YlxR_family_protein_[Bacillus_cereus]	CPTF_Co	77374	134015.6992	173.2050808
UIJ64765.1	YlxR_family_protein_[Bacillus_cereus]	CPTF_Cu	45217.66667	78319.29607	173.2050808
UIJ64765.1	YlxR_family_protein_[Bacillus_cereus]	CPTF_Fe	58424.66667	101194.4911	173.2050808
UIJ64765.1	YlxR_family_protein_[Bacillus_cereus]	CPTF_Mn	181060.3333	162800.078	89.91482289
UIJ64765.1	YlxR_family_protein_[Bacillus_cereus]	CPTF_Ni	115482	111841.2864	96.84737568
UIJ64765.1	YlxR_family_protein_[Bacillus_cereus]	CPTF_U	193162.6667	168829.5835	87.40280224
UIJ64765.1	YlxR_family_protein_[Bacillus_cereus]	CPTF_metals_mix	12332.2	21359.99697	173.2050808
UIJ64765.1	YlxR_family_protein_[Bacillus_cereus]	CPTF_zcontrol	0	0	0
UIJ64766.1	transcription_termination_factor_NusA_[Bacillus_cereus]	CPTF_Al	897097.2333	421209.8593	46.9525313
UIJ64766.1	transcription_termination_factor_NusA_[Bacillus_cereus]	CPTF_Cd	969186.8667	476188.8052	49.13281655
UIJ64766.1	transcription_termination_factor_NusA_[Bacillus_cereus]	CPTF_Co	2200699.967	1787589.823	81.22823872
UIJ64766.1	transcription_termination_factor_NusA_[Bacillus_cereus]	CPTF_Cu	1749959.767	863432.753	49.34014881
UIJ64766.1	transcription_termination_factor_NusA_[Bacillus_cereus]	CPTF_Fe	1163953.467	771056.392	66.24460634
UIJ64766.1	transcription_termination_factor_NusA_[Bacillus_cereus]	CPTF_Mn	2321275.533	1502675.045	64.73488492
UIJ64766.1	transcription_termination_factor_NusA_[Bacillus_cereus]	CPTF_Ni	2314462.833	2895385.137	125.0996601
UIJ64766.1	transcription_termination_factor_NusA_[Bacillus_cereus]	CPTF_U	2920896.833	2131459.583	72.97277874
UIJ64766.1	transcription_termination_factor_NusA_[Bacillus_cereus]	CPTF_metals_mix	1562712.387	109585.4945	7.012518456
UIJ64766.1	transcription_termination_factor_NusA_[Bacillus_cereus]	CPTF_zcontrol	1081042.167	879514.1425	81.35798673
UIJ64767.1	ribosome_maturation_factor_RimP_[Bacillus_cereus]	CPTF_Al	178558.1333	276161.8295	154.6621397
UIJ64767.1	ribosome_maturation_factor_RimP_[Bacillus_cereus]	CPTF_Cd	293890.0667	257854.6507	87.73847094
UIJ64767.1	ribosome_maturation_factor_RimP_[Bacillus_cereus]	CPTF_Co	175217.9	257271.2948	146.8293449
UIJ64767.1	ribosome_maturation_factor_RimP_[Bacillus_cereus]	CPTF_Cu	254801.5333	207245.8484	81.33618572
UIJ64767.1	ribosome_maturation_factor_RimP_[Bacillus_cereus]	CPTF_Fe	307060.3667	217941.9695	70.97691305

UIJ64767.1	ribosome_maturation_factor_RimP_[Bacillus_cereus]	CPTF_Mn	306163	265268.6469	86.64294735
UIJ64767.1	ribosome_maturation_factor_RimP_[Bacillus_cereus]	CPTF_Ni	190987.7	274923.2867	143.9481635
UIJ64767.1	ribosome_maturation_factor_RimP_[Bacillus_cereus]	CPTF_U	356897.3333	309125.1929	86.61459865
UIJ64767.1	ribosome_maturation_factor_RimP_[Bacillus_cereus]	CPTF_metals_mix	294717.2	27644.46213	9.37999619
UIJ64767.1	ribosome_maturation_factor_RimP_[Bacillus_cereus]	CPTF_zcontrol	493346.3333	23062.75127	4.674758829
UIJ64768.1	PolC-type_DNA_polymerase_III_[Bacillus_cereus]	CPTF_Al	98641.83333	58132.71226	58.93312228
UIJ64768.1	PolC-type_DNA_polymerase_III_[Bacillus_cereus]	CPTF_Cd	129342.2633	95108.55143	73.53246261
UIJ64768.1	PolC-type_DNA_polymerase_III_[Bacillus_cereus]	CPTF_Co	79156.14333	28164.90919	35.58145711
UIJ64768.1	PolC-type_DNA_polymerase_III_[Bacillus_cereus]	CPTF_Cu	247607.6	168696.648	68.13064218
UIJ64768.1	PolC-type_DNA_polymerase_III_[Bacillus_cereus]	CPTF_Fe	184695.8667	55216.83432	29.89608556
UIJ64768.1	PolC-type_DNA_polymerase_III_[Bacillus_cereus]	CPTF_Mn	172618.3333	227442.0941	131.7601032
UIJ64768.1	PolC-type_DNA_polymerase_III_[Bacillus_cereus]	CPTF_Ni	111631.0667	59622.6365	53.41043338
UIJ64768.1	PolC-type_DNA_polymerase_III_[Bacillus_cereus]	CPTF_U	165083.3	149431.9671	90.5191301
UIJ64768.1	PolC-type_DNA_polymerase_III_[Bacillus_cereus]	CPTF_metals_mix	151409.7033	58511.23202	38.64430795
UIJ64768.1	PolC-type_DNA_polymerase_III_[Bacillus_cereus]	CPTF_zcontrol	185719.6	197464.777	106.3241451
UIJ64769.1	proline--tRNA_ligase_[Bacillus_cereus]	CPTF_Al	480696.8333	35672.56834	7.421011719
UIJ64769.1	proline--tRNA_ligase_[Bacillus_cereus]	CPTF_Cd	716076.6667	527865.5351	73.71634346
UIJ64769.1	proline--tRNA_ligase_[Bacillus_cereus]	CPTF_Co	540412.6333	68359.26049	12.64945641
UIJ64769.1	proline--tRNA_ligase_[Bacillus_cereus]	CPTF_Cu	691467.6667	267044.1084	38.61989813
UIJ64769.1	proline--tRNA_ligase_[Bacillus_cereus]	CPTF_Fe	581362.4	351608.3533	60.4800643
UIJ64769.1	proline--tRNA_ligase_[Bacillus_cereus]	CPTF_Mn	1070798.633	685055.6161	63.97613845
UIJ64769.1	proline--tRNA_ligase_[Bacillus_cereus]	CPTF_Ni	495529.5667	59454.64959	11.99820426
UIJ64769.1	proline--tRNA_ligase_[Bacillus_cereus]	CPTF_U	346090.0667	337906.4449	97.63540693
UIJ64769.1	proline--tRNA_ligase_[Bacillus_cereus]	CPTF_metals_mix	1041779.667	678240.3962	65.10401555
UIJ64769.1	proline--tRNA_ligase_[Bacillus_cereus]	CPTF_zcontrol	419564.9333	8899.412527	2.12110494
UIJ64770.1	RIP_metalloprotease_RseP_[Bacillus_cereus]	CPTF_Al	282131	92097.14185	32.64339681
UIJ64770.1	RIP_metalloprotease_RseP_[Bacillus_cereus]	CPTF_Cd	139486.9	90918.1955	65.18045458
UIJ64770.1	RIP_metalloprotease_RseP_[Bacillus_cereus]	CPTF_Co	365604.6667	97156.38518	26.5741644
UIJ64770.1	RIP_metalloprotease_RseP_[Bacillus_cereus]	CPTF_Cu	350230.6667	104462.2964	29.82671318
UIJ64770.1	RIP_metalloprotease_RseP_[Bacillus_cereus]	CPTF_Fe	226696.6667	21840.64775	9.634304763
UIJ64770.1	RIP_metalloprotease_RseP_[Bacillus_cereus]	CPTF_Mn	179650.2667	95774.45119	53.31161093
UIJ64770.1	RIP_metalloprotease_RseP_[Bacillus_cereus]	CPTF_Ni	315431	87843.77387	27.8488081
UIJ64770.1	RIP_metalloprotease_RseP_[Bacillus_cereus]	CPTF_U	248929.3333	62530.29324	25.11969658
UIJ64770.1	RIP_metalloprotease_RseP_[Bacillus_cereus]	CPTF_metals_mix	316201	112618.2148	35.61602109
UIJ64770.1	RIP_metalloprotease_RseP_[Bacillus_cereus]	CPTF_zcontrol	325694.3333	130347.1698	40.02131953
UIJ64771.1	1-deoxy-D-xylulose-5-phosphate_reductoisomerase_[Bacillus_cereus]	CPTF_Al	1116666.667	72341.78138	6.478368482
UIJ64771.1	1-deoxy-D-xylulose-5-phosphate_reductoisomerase_[Bacillus_cereus]	CPTF_Cd	558282.3333	483493.1647	86.60370136
UIJ64771.1	1-deoxy-D-xylulose-5-phosphate_reductoisomerase_[Bacillus_cereus]	CPTF_Co	634405.3333	556476.1818	87.71618909
UIJ64771.1	1-deoxy-D-xylulose-5-phosphate_reductoisomerase_[Bacillus_cereus]	CPTF_Cu	961555	199035.6953	20.69935628
UIJ64771.1	1-deoxy-D-xylulose-5-phosphate_reductoisomerase_[Bacillus_cereus]	CPTF_Fe	685501.3333	604087.4721	88.12345692
UIJ64771.1	1-deoxy-D-xylulose-5-phosphate_reductoisomerase_[Bacillus_cereus]	CPTF_Mn	299709.3333	519111.7928	173.2050808
UIJ64771.1	1-deoxy-D-xylulose-5-phosphate_reductoisomerase_[Bacillus_cereus]	CPTF_Ni	583964.6667	516023.6652	88.36556296
UIJ64771.1	1-deoxy-D-xylulose-5-phosphate_reductoisomerase_[Bacillus_cereus]	CPTF_U	694502	621925.9474	89.54991452
UIJ64771.1	1-deoxy-D-xylulose-5-phosphate_reductoisomerase_[Bacillus_cereus]	CPTF_metals_mix	0	0	0
UIJ64771.1	1-deoxy-D-xylulose-5-phosphate_reductoisomerase_[Bacillus_cereus]	CPTF_zcontrol	941635.6667	115519.4091	12.26795174
UIJ64774.1	ribosome_recycling_factor_[Bacillus_cereus]	CPTF_Al	5540425	1012554.447	18.27575407
UIJ64774.1	ribosome_recycling_factor_[Bacillus_cereus]	CPTF_Cd	7341615.333	454383.0575	6.189142809
UIJ64774.1	ribosome_recycling_factor_[Bacillus_cereus]	CPTF_Co	4152785	1563103.751	37.63989109
UIJ64774.1	ribosome_recycling_factor_[Bacillus_cereus]	CPTF_Cu	3956389.667	308216.5979	7.790349886
UIJ64774.1	ribosome_recycling_factor_[Bacillus_cereus]	CPTF_Fe	5639539.133	1518921.31	26.93342973
UIJ64774.1	ribosome_recycling_factor_[Bacillus_cereus]	CPTF_Mn	6304880	2081650.446	33.01649588
UIJ64774.1	ribosome_recycling_factor_[Bacillus_cereus]	CPTF_Ni	2468949.667	68999.2393	2.794679869
UIJ64774.1	ribosome_recycling_factor_[Bacillus_cereus]	CPTF_U	3734296.667	162220.0172	4.344058111
UIJ64774.1	ribosome_recycling_factor_[Bacillus_cereus]	CPTF_metals_mix	7881660	1268376.681	16.09276068
UIJ64774.1	ribosome_recycling_factor_[Bacillus_cereus]	CPTF_zcontrol	4700891.667	1796385.407	38.21371634
UIJ64775.1	UMP_kinase_[Bacillus_cereus]	CPTF_Al	2059809	385097.0838	18.69576663
UIJ64775.1	UMP_kinase_[Bacillus_cereus]	CPTF_Cd	2546488.9	72371.27476	2.842002365
UIJ64775.1	UMP_kinase_[Bacillus_cereus]	CPTF_Co	1997008.333	244107.4452	12.22365681
UIJ64775.1	UMP_kinase_[Bacillus_cereus]	CPTF_Cu	1704528.367	135124.3361	7.927373854
UIJ64775.1	UMP_kinase_[Bacillus_cereus]	CPTF_Fe	2132648.333	362607.42	17.00268227
UIJ64775.1	UMP_kinase_[Bacillus_cereus]	CPTF_Mn	1996973	671130.8085	33.60740523

UIJ64775.1	UMP_kinase_[Bacillus_cereus]	CPTF_Ni	686900.8333	295645.1785	43.04044546
UIJ64775.1	UMP_kinase_[Bacillus_cereus]	CPTF_U	1230245.333	343979.6832	27.96025101
UIJ64775.1	UMP_kinase_[Bacillus_cereus]	CPTF_metals_mix	2001135	37959.36463	1.896891745
UIJ64775.1	UMP_kinase_[Bacillus_cereus]	CPTF_zcontrol	1873539.667	571123.2555	30.48364898
UIJ64776.1	translation_elongation_factor_Ts_[Bacillus_cereus]	CPTF_Al	17015798.63	978993.4957	5.753438418
UIJ64776.1	translation_elongation_factor_Ts_[Bacillus_cereus]	CPTF_Cd	16560167.1	1265614.967	7.642525338
UIJ64776.1	translation_elongation_factor_Ts_[Bacillus_cereus]	CPTF_Co	18286523.3	738742.2297	4.039817835
UIJ64776.1	translation_elongation_factor_Ts_[Bacillus_cereus]	CPTF_Cu	16541666.03	673793.0674	4.073308372
UIJ64776.1	translation_elongation_factor_Ts_[Bacillus_cereus]	CPTF_Fe	15648478.43	596586.0104	3.812421846
UIJ64776.1	translation_elongation_factor_Ts_[Bacillus_cereus]	CPTF_Mn	16837043.57	1865738.79	11.08115438
UIJ64776.1	translation_elongation_factor_Ts_[Bacillus_cereus]	CPTF_Ni	16169523.1	563411.4856	3.484403851
UIJ64776.1	translation_elongation_factor_Ts_[Bacillus_cereus]	CPTF_U	13456077.9	789054.1444	5.863923725
UIJ64776.1	translation_elongation_factor_Ts_[Bacillus_cereus]	CPTF_metals_mix	16841332.17	391273.0821	2.32329057
UIJ64776.1	translation_elongation_factor_Ts_[Bacillus_cereus]	CPTF_zcontrol	15318670.87	506989.8371	3.309620277
UIJ64777.1	30S_ribosomal_protein_S2_[Bacillus_cereus]	CPTF_Al	9959371.167	942316.9684	9.461611106
UIJ64777.1	30S_ribosomal_protein_S2_[Bacillus_cereus]	CPTF_Cd	10404130.13	963070.2324	9.256614633
UIJ64777.1	30S_ribosomal_protein_S2_[Bacillus_cereus]	CPTF_Co	11377116.07	85343.6956	0.750134701
UIJ64777.1	30S_ribosomal_protein_S2_[Bacillus_cereus]	CPTF_Cu	11200485.9	553977.4286	4.946012464
UIJ64777.1	30S_ribosomal_protein_S2_[Bacillus_cereus]	CPTF_Fe	9753693.867	577277.6077	5.918553684
UIJ64777.1	30S_ribosomal_protein_S2_[Bacillus_cereus]	CPTF_Mn	9890232.433	2274474.208	22.99717649
UIJ64777.1	30S_ribosomal_protein_S2_[Bacillus_cereus]	CPTF_Ni	9116967.167	1191341.571	13.06730132
UIJ64777.1	30S_ribosomal_protein_S2_[Bacillus_cereus]	CPTF_U	7540704.967	898521.33	11.91561444
UIJ64777.1	30S_ribosomal_protein_S2_[Bacillus_cereus]	CPTF_metals_mix	11119053.37	1114248.341	10.02107197
UIJ64777.1	30S_ribosomal_protein_S2_[Bacillus_cereus]	CPTF_zcontrol	9799585.167	456188.2519	4.655179215
UIJ64778.1	GTP-sensing_pleiotropic_transcriptional_regulator_CodY_[Bacillus_cereus]	CPTF_Al	6657470.333	453061.0158	6.805302813
UIJ64778.1	GTP-sensing_pleiotropic_transcriptional_regulator_CodY_[Bacillus_cereus]	CPTF_Cd	7688845.667	190237.2918	2.47419834
UIJ64778.1	GTP-sensing_pleiotropic_transcriptional_regulator_CodY_[Bacillus_cereus]	CPTF_Co	7205657.333	616118.7457	8.550486336
UIJ64778.1	GTP-sensing_pleiotropic_transcriptional_regulator_CodY_[Bacillus_cereus]	CPTF_Cu	7207018	580424.6377	8.053603276
UIJ64778.1	GTP-sensing_pleiotropic_transcriptional_regulator_CodY_[Bacillus_cereus]	CPTF_Fe	7277652	932506.6571	12.81329002
UIJ64778.1	GTP-sensing_pleiotropic_transcriptional_regulator_CodY_[Bacillus_cereus]	CPTF_Mn	7195192.7	923521.5923	12.83525863
UIJ64778.1	GTP-sensing_pleiotropic_transcriptional_regulator_CodY_[Bacillus_cereus]	CPTF_Ni	6154961.333	324371.0565	5.270074643
UIJ64778.1	GTP-sensing_pleiotropic_transcriptional_regulator_CodY_[Bacillus_cereus]	CPTF_U	6385201.033	1360665.454	21.3096729
UIJ64778.1	GTP-sensing_pleiotropic_transcriptional_regulator_CodY_[Bacillus_cereus]	CPTF_metals_mix	10671316	528025.9015	4.94808608
UIJ64778.1	GTP-sensing_pleiotropic_transcriptional_regulator_CodY_[Bacillus_cereus]	CPTF_zcontrol	7024979	500818.5447	7.129110916
UIJ64779.1	ATP-dependent_protease_ATPase_subunit_HslU_[Bacillus_cereus]	CPTF_Al	3323256.633	367525.0588	11.05918379
UIJ64779.1	ATP-dependent_protease_ATPase_subunit_HslU_[Bacillus_cereus]	CPTF_Cd	4069565.6	490373.4547	12.04977393
UIJ64779.1	ATP-dependent_protease_ATPase_subunit_HslU_[Bacillus_cereus]	CPTF_Co	3697872.733	544877.737	14.73489696
UIJ64779.1	ATP-dependent_protease_ATPase_subunit_HslU_[Bacillus_cereus]	CPTF_Cu	3359687.267	381884.6132	11.36667145
UIJ64779.1	ATP-dependent_protease_ATPase_subunit_HslU_[Bacillus_cereus]	CPTF_Fe	3481026.567	387225.4193	11.12388578
UIJ64779.1	ATP-dependent_protease_ATPase_subunit_HslU_[Bacillus_cereus]	CPTF_Mn	3735714.167	767459.6289	20.54385305
UIJ64779.1	ATP-dependent_protease_ATPase_subunit_HslU_[Bacillus_cereus]	CPTF_Ni	3750483.033	888390.4425	23.68736066
UIJ64779.1	ATP-dependent_protease_ATPase_subunit_HslU_[Bacillus_cereus]	CPTF_U	3294428.667	288554.5245	8.75886394
UIJ64779.1	ATP-dependent_protease_ATPase_subunit_HslU_[Bacillus_cereus]	CPTF_metals_mix	7186197.233	831265.1659	11.56752506
UIJ64779.1	ATP-dependent_protease_ATPase_subunit_HslU_[Bacillus_cereus]	CPTF_zcontrol	3031733	142050.6649	4.68546092
UIJ64780.1	ATP-dependent_protease_proteolytic_subunit_HslV_[Bacillus_cereus]	CPTF_Al	0	0	0
UIJ64780.1	ATP-dependent_protease_proteolytic_subunit_HslV_[Bacillus_cereus]	CPTF_Cd	0	0	0
UIJ64780.1	ATP-dependent_protease_proteolytic_subunit_HslV_[Bacillus_cereus]	CPTF_Co	0	0	0
UIJ64780.1	ATP-dependent_protease_proteolytic_subunit_HslV_[Bacillus_cereus]	CPTF_Cu	0	0	0
UIJ64780.1	ATP-dependent_protease_proteolytic_subunit_HslV_[Bacillus_cereus]	CPTF_Fe	0	0	0
UIJ64780.1	ATP-dependent_protease_proteolytic_subunit_HslV_[Bacillus_cereus]	CPTF_Mn	55986.66667	96971.75121	173.2050808
UIJ64780.1	ATP-dependent_protease_proteolytic_subunit_HslV_[Bacillus_cereus]	CPTF_Ni	0	0	0
UIJ64780.1	ATP-dependent_protease_proteolytic_subunit_HslV_[Bacillus_cereus]	CPTF_U	0	0	0
UIJ64780.1	ATP-dependent_protease_proteolytic_subunit_HslV_[Bacillus_cereus]	CPTF_metals_mix	140261.6667	242940.333	173.2050808
UIJ64780.1	ATP-dependent_protease_proteolytic_subunit_HslV_[Bacillus_cereus]	CPTF_zcontrol	0	0	0
UIJ64782.1	FADH(2)-oxidizing_methylenetetrahydrofolate--tRNA-(uracil(54)-C(5))-methyltransferase_TrmFO_[Bacillus_cereus]	CPTF_Al	305539.7	83685.56388	27.38942399
UIJ64782.1	FADH(2)-oxidizing_methylenetetrahydrofolate--tRNA-(uracil(54)-C(5))-methyltransferase_TrmFO_[Bacillus_cereus]	CPTF_Cd	331763.3333	145651.0916	43.90210641
UIJ64782.1	FADH(2)-oxidizing_methylenetetrahydrofolate--tRNA-(uracil(54)-C(5))-methyltransferase_TrmFO_[Bacillus_cereus]	CPTF_Co	420358.9333	114840.0541	27.31952267
UIJ64782.1	FADH(2)-oxidizing_methylenetetrahydrofolate--tRNA-(uracil(54)-C(5))-methyltransferase_TrmFO_[Bacillus_cereus]	CPTF_Cu	424904.5333	110889.4858	26.09750595
UIJ64782.1	FADH(2)-oxidizing_methylenetetrahydrofolate--tRNA-(uracil(54)-C(5))-methyltransferase_TrmFO_[Bacillus_cereus]	CPTF_Fe	267629.3333	232088.4435	86.72010673
UIJ64782.1	FADH(2)-oxidizing_methylenetetrahydrofolate--tRNA-(uracil(54)-C(5))-methyltransferase_TrmFO_[Bacillus_cereus]	CPTF_Mn	340512	66428.54237	19.50842918
UIJ64782.1	FADH(2)-oxidizing_methylenetetrahydrofolate--tRNA-(uracil(54)-C(5))-methyltransferase_TrmFO_[Bacillus_cereus]	CPTF_Ni	437708.4667	22900.4145	5.231887488

UIJ64782.1	FADH(2)-oxidizing_methylenetetrahydrofolate--tRNA-(uracil(54)-C(5))-methyltransferase_TrmFO_[Bacillus_cereus]	CPTF_U	261097	226184.8716	86.62867501
UIJ64782.1	FADH(2)-oxidizing_methylenetetrahydrofolate--tRNA-(uracil(54)-C(5))-methyltransferase_TrmFO_[Bacillus_cereus]	CPTF_metals_mix	447438.0667	49666.54335	11.10020516
UIJ64782.1	FADH(2)-oxidizing_methylenetetrahydrofolate--tRNA-(uracil(54)-C(5))-methyltransferase_TrmFO_[Bacillus_cereus]	CPTF_zcontrol	261399	227016.2262	86.84663148
UIJ64783.1	type_I_DNA_topoisomerase_[Bacillus_cereus]	CPTF_Al	641905.5	245504.4071	38.24619155
UIJ64783.1	type_I_DNA_topoisomerase_[Bacillus_cereus]	CPTF_Cd	524232.8667	99363.71479	18.95411774
UIJ64783.1	type_I_DNA_topoisomerase_[Bacillus_cereus]	CPTF_Co	1008372	83166.42657	8.247593801
UIJ64783.1	type_I_DNA_topoisomerase_[Bacillus_cereus]	CPTF_Cu	1411964.433	252755.5473	17.90098542
UIJ64783.1	type_I_DNA_topoisomerase_[Bacillus_cereus]	CPTF_Fe	704660.1333	189891.888	26.94801068
UIJ64783.1	type_I_DNA_topoisomerase_[Bacillus_cereus]	CPTF_Mn	608014.2333	476908.2346	78.43701816
UIJ64783.1	type_I_DNA_topoisomerase_[Bacillus_cereus]	CPTF_Ni	716196.5	108619.917	15.16621723
UIJ64783.1	type_I_DNA_topoisomerase_[Bacillus_cereus]	CPTF_U	205247.4667	167036.9838	81.3832134
UIJ64783.1	type_I_DNA_topoisomerase_[Bacillus_cereus]	CPTF_metals_mix	925828.1	212057.2062	22.90459819
UIJ64783.1	type_I_DNA_topoisomerase_[Bacillus_cereus]	CPTF_zcontrol	689024.3667	103202.7967	14.97810553
UIJ64785.1	succinate--CoA_ligase_subunit_alpha_[Bacillus_cereus]	CPTF_Al	5434155.333	810842.1201	14.9212172
UIJ64785.1	succinate--CoA_ligase_subunit_alpha_[Bacillus_cereus]	CPTF_Cd	7058864.067	963872.8201	13.65478654
UIJ64785.1	succinate--CoA_ligase_subunit_alpha_[Bacillus_cereus]	CPTF_Co	4581536.633	883269.6034	19.27889427
UIJ64785.1	succinate--CoA_ligase_subunit_alpha_[Bacillus_cereus]	CPTF_Cu	4889389.933	278334.5699	5.692623695
UIJ64785.1	succinate--CoA_ligase_subunit_alpha_[Bacillus_cereus]	CPTF_Fe	5190152.667	611758.0226	11.78689842
UIJ64785.1	succinate--CoA_ligase_subunit_alpha_[Bacillus_cereus]	CPTF_Mn	5706681.8	1848514.696	32.39211087
UIJ64785.1	succinate--CoA_ligase_subunit_alpha_[Bacillus_cereus]	CPTF_Ni	3630817.6	451157.8402	12.42579193
UIJ64785.1	succinate--CoA_ligase_subunit_alpha_[Bacillus_cereus]	CPTF_U	3310879.767	351478.041	10.61585034
UIJ64785.1	succinate--CoA_ligase_subunit_alpha_[Bacillus_cereus]	CPTF_metals_mix	8330408.9	551104.3385	6.615573679
UIJ64785.1	succinate--CoA_ligase_subunit_alpha_[Bacillus_cereus]	CPTF_zcontrol	4744039.333	325741.2774	6.8663275
UIJ64786.1	ADP-forming_succinate--CoA_ligase_subunit_beta_[Bacillus_cereus]	CPTF_Al	10421510.4	281935.2782	2.705320701
UIJ64786.1	ADP-forming_succinate--CoA_ligase_subunit_beta_[Bacillus_cereus]	CPTF_Cd	11014600.9	310990.4232	2.823437962
UIJ64786.1	ADP-forming_succinate--CoA_ligase_subunit_beta_[Bacillus_cereus]	CPTF_Co	11182954.4	369079.0204	3.300371326
UIJ64786.1	ADP-forming_succinate--CoA_ligase_subunit_beta_[Bacillus_cereus]	CPTF_Cu	11152597.63	1191652.922	10.68498085
UIJ64786.1	ADP-forming_succinate--CoA_ligase_subunit_beta_[Bacillus_cereus]	CPTF_Fe	9616329.7	1044416.263	10.86086163
UIJ64786.1	ADP-forming_succinate--CoA_ligase_subunit_beta_[Bacillus_cereus]	CPTF_Mn	10504297.77	1749669.235	16.65669875
UIJ64786.1	ADP-forming_succinate--CoA_ligase_subunit_beta_[Bacillus_cereus]	CPTF_Ni	10116553.6	1179400.768	11.658128
UIJ64786.1	ADP-forming_succinate--CoA_ligase_subunit_beta_[Bacillus_cereus]	CPTF_U	8411011.333	307353.4805	3.65417984
UIJ64786.1	ADP-forming_succinate--CoA_ligase_subunit_beta_[Bacillus_cereus]	CPTF_metals_mix	12298596.33	1435139.682	11.66913397
UIJ64786.1	ADP-forming_succinate--CoA_ligase_subunit_beta_[Bacillus_cereus]	CPTF_zcontrol	10207836.57	364573.3116	3.571504199
UIJ64788.1	ribosome_biogenesis_GTPase_YlqF_[Bacillus_cereus]	CPTF_Al	240452	127304.5423	52.94384838
UIJ64788.1	ribosome_biogenesis_GTPase_YlqF_[Bacillus_cereus]	CPTF_Cd	527604.7333	323910.8309	61.39270755
UIJ64788.1	ribosome_biogenesis_GTPase_YlqF_[Bacillus_cereus]	CPTF_Co	295797	12771.00157	4.317488536
UIJ64788.1	ribosome_biogenesis_GTPase_YlqF_[Bacillus_cereus]	CPTF_Cu	249969.6	83647.53081	33.46308143
UIJ64788.1	ribosome_biogenesis_GTPase_YlqF_[Bacillus_cereus]	CPTF_Fe	439184.3333	108289.66	24.6569952
UIJ64788.1	ribosome_biogenesis_GTPase_YlqF_[Bacillus_cereus]	CPTF_Mn	352165	173253.4093	49.19665762
UIJ64788.1	ribosome_biogenesis_GTPase_YlqF_[Bacillus_cereus]	CPTF_Ni	200858.7667	25175.50356	12.53393316
UIJ64788.1	ribosome_biogenesis_GTPase_YlqF_[Bacillus_cereus]	CPTF_U	245433.1	109298.2698	44.5328156
UIJ64788.1	ribosome_biogenesis_GTPase_YlqF_[Bacillus_cereus]	CPTF_metals_mix	540980.6	134435.4425	24.85032596
UIJ64788.1	ribosome_biogenesis_GTPase_YlqF_[Bacillus_cereus]	CPTF_zcontrol	202240.1	122219.5119	60.43287749
UIJ64789.1	signal_peptidase_I_[Bacillus_cereus]	CPTF_Al	0	0	0
UIJ64789.1	signal_peptidase_I_[Bacillus_cereus]	CPTF_Cd	143326.1	33741.26788	23.54160748
UIJ64789.1	signal_peptidase_I_[Bacillus_cereus]	CPTF_Co	59863.63333	52961.00958	88.46942063
UIJ64789.1	signal_peptidase_I_[Bacillus_cereus]	CPTF_Cu	66034.1	58152.67048	88.06460674
UIJ64789.1	signal_peptidase_I_[Bacillus_cereus]	CPTF_Fe	0	0	0
UIJ64789.1	signal_peptidase_I_[Bacillus_cereus]	CPTF_Mn	0	0	0
UIJ64789.1	signal_peptidase_I_[Bacillus_cereus]	CPTF_Ni	124549.6	24557.47432	19.71702384
UIJ64789.1	signal_peptidase_I_[Bacillus_cereus]	CPTF_U	108074.3333	95325.30862	88.20346671
UIJ64789.1	signal_peptidase_I_[Bacillus_cereus]	CPTF_metals_mix	22856.66667	39588.90796	173.2050808
UIJ64789.1	signal_peptidase_I_[Bacillus_cereus]	CPTF_zcontrol	0	0	0
UIJ64790.1	50S_ribosomal_protein_L19_[Bacillus_cereus]	CPTF_Al	19221701.67	562410.8792	2.92591618
UIJ64790.1	50S_ribosomal_protein_L19_[Bacillus_cereus]	CPTF_Cd	18786139	870253.6097	4.632423989
UIJ64790.1	50S_ribosomal_protein_L19_[Bacillus_cereus]	CPTF_Co	20095652.33	1695776.995	8.438526734
UIJ64790.1	50S_ribosomal_protein_L19_[Bacillus_cereus]	CPTF_Cu	19444093	1840235.374	9.464238696
UIJ64790.1	50S_ribosomal_protein_L19_[Bacillus_cereus]	CPTF_Fe	20240171.33	798912.3411	3.947161948
UIJ64790.1	50S_ribosomal_protein_L19_[Bacillus_cereus]	CPTF_Mn	19931834.33	588161.097	2.950862862
UIJ64790.1	50S_ribosomal_protein_L19_[Bacillus_cereus]	CPTF_Ni	20199591.33	1262251.341	6.248895439
UIJ64790.1	50S_ribosomal_protein_L19_[Bacillus_cereus]	CPTF_U	21471021.33	1700308.86	7.919087002

UIJ64790.1	50S_ribosomal_protein_L19_[Bacillus_cereus]	CPTF_metals_mix	18353894	1484620.357	8.088857639
UIJ64790.1	50S_ribosomal_protein_L19_[Bacillus_cereus]	CPTF_zcontrol	20303365.33	974547.8641	4.799932662
UIJ64792.1	ribosome_maturation_factor_RimM_[Bacillus_cereus]	CPTF_Al	54623	94609.81126	173.2050808
UIJ64792.1	ribosome_maturation_factor_RimM_[Bacillus_cereus]	CPTF_Cd	34572	59880.46052	173.2050808
UIJ64792.1	ribosome_maturation_factor_RimM_[Bacillus_cereus]	CPTF_Co	73463.63333	65739.88282	89.48629387
UIJ64792.1	ribosome_maturation_factor_RimM_[Bacillus_cereus]	CPTF_Cu	11723	20304.83162	173.2050808
UIJ64792.1	ribosome_maturation_factor_RimM_[Bacillus_cereus]	CPTF_Fe	75175.56667	68597.287	91.24944452
UIJ64792.1	ribosome_maturation_factor_RimM_[Bacillus_cereus]	CPTF_Mn	60802.33333	105312.7306	173.2050808
UIJ64792.1	ribosome_maturation_factor_RimM_[Bacillus_cereus]	CPTF_Ni	8627.966667	14944.07663	173.2050808
UIJ64792.1	ribosome_maturation_factor_RimM_[Bacillus_cereus]	CPTF_U	67826.66667	117479.2328	173.2050808
UIJ64792.1	ribosome_maturation_factor_RimM_[Bacillus_cereus]	CPTF_metals_mix	26711.76667	23147.26442	86.65568515
UIJ64792.1	ribosome_maturation_factor_RimM_[Bacillus_cereus]	CPTF_zcontrol	0	0	0
UIJ64794.1	30S_ribosomal_protein_S16_[Bacillus_cereus]	CPTF_Al	2143333.333	275741.4248	12.86507425
UIJ64794.1	30S_ribosomal_protein_S16_[Bacillus_cereus]	CPTF_Cd	2083333.333	143643.0762	6.894867656
UIJ64794.1	30S_ribosomal_protein_S16_[Bacillus_cereus]	CPTF_Co	2200000	112694.2767	5.122467123
UIJ64794.1	30S_ribosomal_protein_S16_[Bacillus_cereus]	CPTF_Cu	2090000	43588.98944	2.085597581
UIJ64794.1	30S_ribosomal_protein_S16_[Bacillus_cereus]	CPTF_Fe	2203333.333	190875.1774	8.66301864
UIJ64794.1	30S_ribosomal_protein_S16_[Bacillus_cereus]	CPTF_Mn	2301260.333	723078.8306	31.42099223
UIJ64794.1	30S_ribosomal_protein_S16_[Bacillus_cereus]	CPTF_Ni	2100000	130766.9683	6.226998491
UIJ64794.1	30S_ribosomal_protein_S16_[Bacillus_cereus]	CPTF_U	2120000	345976.8778	16.31966405
UIJ64794.1	30S_ribosomal_protein_S16_[Bacillus_cereus]	CPTF_metals_mix	4009634	651951.6055	16.25962882
UIJ64794.1	30S_ribosomal_protein_S16_[Bacillus_cereus]	CPTF_zcontrol	1976666.667	68068.59286	3.443605035
UIJ64795.1	signal_recognition_particle_protein_[Bacillus_cereus]	CPTF_Al	852981.4667	151934.2314	17.81213746
UIJ64795.1	signal_recognition_particle_protein_[Bacillus_cereus]	CPTF_Cd	1153844.533	68772.62961	5.960302937
UIJ64795.1	signal_recognition_particle_protein_[Bacillus_cereus]	CPTF_Co	1027278.6	168069.3577	16.36064041
UIJ64795.1	signal_recognition_particle_protein_[Bacillus_cereus]	CPTF_Cu	819167.2	96306.60764	11.75664842
UIJ64795.1	signal_recognition_particle_protein_[Bacillus_cereus]	CPTF_Fe	953154.3	163957.9481	17.20161658
UIJ64795.1	signal_recognition_particle_protein_[Bacillus_cereus]	CPTF_Mn	947883.5667	326216.9167	34.41529405
UIJ64795.1	signal_recognition_particle_protein_[Bacillus_cereus]	CPTF_Ni	543729.4667	74616.74264	13.72313756
UIJ64795.1	signal_recognition_particle_protein_[Bacillus_cereus]	CPTF_U	702904.6	104505.1442	14.86761421
UIJ64795.1	signal_recognition_particle_protein_[Bacillus_cereus]	CPTF_metals_mix	1866639.367	46579.68541	2.495376785
UIJ64795.1	signal_recognition_particle_protein_[Bacillus_cereus]	CPTF_zcontrol	718240.5333	106800.4676	14.86973551
UIJ64797.1	signal_recognition_particle-docking_protein_FtsY_[Bacillus_cereus]	CPTF_Al	1231185.667	212384.3302	17.25039008
UIJ64797.1	signal_recognition_particle-docking_protein_FtsY_[Bacillus_cereus]	CPTF_Cd	1458253.667	163764.7393	11.23019562
UIJ64797.1	signal_recognition_particle-docking_protein_FtsY_[Bacillus_cereus]	CPTF_Co	1267437.667	204919.0452	16.16797817
UIJ64797.1	signal_recognition_particle-docking_protein_FtsY_[Bacillus_cereus]	CPTF_Cu	1369387.6	32362.42766	2.3632774
UIJ64797.1	signal_recognition_particle-docking_protein_FtsY_[Bacillus_cereus]	CPTF_Fe	1265303	265142.0264	20.95482476
UIJ64797.1	signal_recognition_particle-docking_protein_FtsY_[Bacillus_cereus]	CPTF_Mn	1132652.667	325025.3106	28.69593832
UIJ64797.1	signal_recognition_particle-docking_protein_FtsY_[Bacillus_cereus]	CPTF_Ni	908221.6667	68574.04047	7.550363858
UIJ64797.1	signal_recognition_particle-docking_protein_FtsY_[Bacillus_cereus]	CPTF_U	1089409	276857.5547	25.41355494
UIJ64797.1	signal_recognition_particle-docking_protein_FtsY_[Bacillus_cereus]	CPTF_metals_mix	1331108	148983.969	11.19247792
UIJ64797.1	signal_recognition_particle-docking_protein_FtsY_[Bacillus_cereus]	CPTF_zcontrol	1106900	182313.4123	16.47063079
UIJ64799.1	ribonuclease_III_[Bacillus_cereus]	CPTF_Al	632548.5667	303368.9543	47.95978844
UIJ64799.1	ribonuclease_III_[Bacillus_cereus]	CPTF_Cd	484325.0333	52885.5448	10.91943244
UIJ64799.1	ribonuclease_III_[Bacillus_cereus]	CPTF_Co	816561.6333	284975.429	34.89943899
UIJ64799.1	ribonuclease_III_[Bacillus_cereus]	CPTF_Cu	929067.9	353118.8251	38.00785982
UIJ64799.1	ribonuclease_III_[Bacillus_cereus]	CPTF_Fe	850119.7333	432815.0164	50.91224206
UIJ64799.1	ribonuclease_III_[Bacillus_cereus]	CPTF_Mn	795275.1333	646935.3759	81.34736631
UIJ64799.1	ribonuclease_III_[Bacillus_cereus]	CPTF_Ni	858386.4333	280510.3443	32.67879517
UIJ64799.1	ribonuclease_III_[Bacillus_cereus]	CPTF_U	618605	183121.8335	29.60238497
UIJ64799.1	ribonuclease_III_[Bacillus_cereus]	CPTF_metals_mix	288965.3333	81980.49818	28.37035752
UIJ64799.1	ribonuclease_III_[Bacillus_cereus]	CPTF_zcontrol	854468.9667	406183.7997	47.53640162
UIJ64800.1	3-oxoacyl-[acyl-carrier-protein]_reductase_[Bacillus_cereus]	CPTF_Al	1355530.3	480882.0991	35.4755699
UIJ64800.1	3-oxoacyl-[acyl-carrier-protein]_reductase_[Bacillus_cereus]	CPTF_Cd	1999720.9	464533.6025	23.22992186
UIJ64800.1	3-oxoacyl-[acyl-carrier-protein]_reductase_[Bacillus_cereus]	CPTF_Co	1399460	253993.2226	18.14937351
UIJ64800.1	3-oxoacyl-[acyl-carrier-protein]_reductase_[Bacillus_cereus]	CPTF_Cu	1359902.7	195723.3963	14.3924559
UIJ64800.1	3-oxoacyl-[acyl-carrier-protein]_reductase_[Bacillus_cereus]	CPTF_Fe	1324659.6	335847.7534	25.35351372
UIJ64800.1	3-oxoacyl-[acyl-carrier-protein]_reductase_[Bacillus_cereus]	CPTF_Mn	1493732	875604.7674	58.61859874
UIJ64800.1	3-oxoacyl-[acyl-carrier-protein]_reductase_[Bacillus_cereus]	CPTF_Ni	988429.3333	32157.04903	3.253348312
UIJ64800.1	3-oxoacyl-[acyl-carrier-protein]_reductase_[Bacillus_cereus]	CPTF_U	701106.3667	145476.8574	20.74961295
UIJ64800.1	3-oxoacyl-[acyl-carrier-protein]_reductase_[Bacillus_cereus]	CPTF_metals_mix	4093701	724851.2031	17.70650087

UIJ64800.1	3-oxoacyl-[acyl-carrier-protein]_reductase_[Bacillus_cereus]	CPTF_zcontrol	1119395.733	189798.1501	16.95541125
UIJ64801.1	ACP_S-malonyltransferase_[Bacillus_cereus]	CPTF_Al	743104.6333	78740.06066	10.59609335
UIJ64801.1	ACP_S-malonyltransferase_[Bacillus_cereus]	CPTF_Cd	673766.3333	37893.3287	5.624105395
UIJ64801.1	ACP_S-malonyltransferase_[Bacillus_cereus]	CPTF_Co	667452.4	40647.14676	6.089894464
UIJ64801.1	ACP_S-malonyltransferase_[Bacillus_cereus]	CPTF_Cu	630396.6667	46519.51726	7.379404068
UIJ64801.1	ACP_S-malonyltransferase_[Bacillus_cereus]	CPTF_Fe	765781.2	73340.63497	9.577231064
UIJ64801.1	ACP_S-malonyltransferase_[Bacillus_cereus]	CPTF_Mn	767017.6667	240827.5951	31.39792022
UIJ64801.1	ACP_S-malonyltransferase_[Bacillus_cereus]	CPTF_Ni	696562.3333	75949.70765	10.90350483
UIJ64801.1	ACP_S-malonyltransferase_[Bacillus_cereus]	CPTF_U	908006	113539.3269	12.50424853
UIJ64801.1	ACP_S-malonyltransferase_[Bacillus_cereus]	CPTF_metals_mix	930655.5	171257.2903	18.40179211
UIJ64801.1	ACP_S-malonyltransferase_[Bacillus_cereus]	CPTF_zcontrol	803490.3333	86703.95493	10.79091451
UIJ64802.1	phosphate_acyltransferase_PlsX_[Bacillus_cereus]	CPTF_Al	111268.6667	109697.3034	98.58777561
UIJ64802.1	phosphate_acyltransferase_PlsX_[Bacillus_cereus]	CPTF_Cd	0	0	0
UIJ64802.1	phosphate_acyltransferase_PlsX_[Bacillus_cereus]	CPTF_Co	0	0	0
UIJ64802.1	phosphate_acyltransferase_PlsX_[Bacillus_cereus]	CPTF_Cu	0	0	0
UIJ64802.1	phosphate_acyltransferase_PlsX_[Bacillus_cereus]	CPTF_Fe	0	0	0
UIJ64802.1	phosphate_acyltransferase_PlsX_[Bacillus_cereus]	CPTF_Mn	0	0	0
UIJ64802.1	phosphate_acyltransferase_PlsX_[Bacillus_cereus]	CPTF_Ni	0	0	0
UIJ64802.1	phosphate_acyltransferase_PlsX_[Bacillus_cereus]	CPTF_U	93588.33333	162099.7483	173.2050808
UIJ64802.1	phosphate_acyltransferase_PlsX_[Bacillus_cereus]	CPTF_metals_mix	352249.6667	277353.4307	78.73774114
UIJ64802.1	phosphate_acyltransferase_PlsX_[Bacillus_cereus]	CPTF_zcontrol	0	0	0
UIJ64803.1	transcription_factor_FapR_[Bacillus_cereus]	CPTF_Al	72038.36667	63582.10524	88.2614476
UIJ64803.1	transcription_factor_FapR_[Bacillus_cereus]	CPTF_Cd	102475.1667	7426.569943	7.247189914
UIJ64803.1	transcription_factor_FapR_[Bacillus_cereus]	CPTF_Co	50831.16667	44024.71309	86.60968451
UIJ64803.1	transcription_factor_FapR_[Bacillus_cereus]	CPTF_Cu	96726.66667	35552.4995	36.75563391
UIJ64803.1	transcription_factor_FapR_[Bacillus_cereus]	CPTF_Fe	29483.33333	35136.36702	119.1736586
UIJ64803.1	transcription_factor_FapR_[Bacillus_cereus]	CPTF_Mn	24458.96667	22128.53709	90.47208491
UIJ64803.1	transcription_factor_FapR_[Bacillus_cereus]	CPTF_Ni	406767.6667	510818.0838	125.5798151
UIJ64803.1	transcription_factor_FapR_[Bacillus_cereus]	CPTF_U	0	0	0
UIJ64803.1	transcription_factor_FapR_[Bacillus_cereus]	CPTF_metals_mix	13342.5	23109.8879	173.2050808
UIJ64803.1	transcription_factor_FapR_[Bacillus_cereus]	CPTF_zcontrol	56219.46667	55657.77122	99.00088799
UIJ64805.1	DAK2_domain-containing_protein_[Bacillus_cereus]	CPTF_Al	5159093.8	385957.3196	7.481106849
UIJ64805.1	DAK2_domain-containing_protein_[Bacillus_cereus]	CPTF_Cd	5160938.967	603413.2366	11.69192739
UIJ64805.1	DAK2_domain-containing_protein_[Bacillus_cereus]	CPTF_Co	5836244.433	266290.8301	4.562708659
UIJ64805.1	DAK2_domain-containing_protein_[Bacillus_cereus]	CPTF_Cu	5808418.733	293459.9517	5.052320867
UIJ64805.1	DAK2_domain-containing_protein_[Bacillus_cereus]	CPTF_Fe	5095996.333	266365.9683	5.226965462
UIJ64805.1	DAK2_domain-containing_protein_[Bacillus_cereus]	CPTF_Mn	5024605.5	281290.1438	5.598253312
UIJ64805.1	DAK2_domain-containing_protein_[Bacillus_cereus]	CPTF_Ni	4720814.8	755358.034	16.00058604
UIJ64805.1	DAK2_domain-containing_protein_[Bacillus_cereus]	CPTF_U	4953821.833	369099.7273	7.450807471
UIJ64805.1	DAK2_domain-containing_protein_[Bacillus_cereus]	CPTF_metals_mix	5928711.433	43969.41911	0.741635338
UIJ64805.1	DAK2_domain-containing_protein_[Bacillus_cereus]	CPTF_zcontrol	5352763.7	146395.1632	2.734945375
UIJ64806.1	Asp23/Gls24_family_envelope_stress_response_protein_[Bacillus_cereus]	CPTF_Al	3116666.667	479200.7234	15.37542428
UIJ64806.1	Asp23/Gls24_family_envelope_stress_response_protein_[Bacillus_cereus]	CPTF_Cd	2963333.333	142243.922	4.800132349
UIJ64806.1	Asp23/Gls24_family_envelope_stress_response_protein_[Bacillus_cereus]	CPTF_Co	2973333.333	239652.5262	8.060062542
UIJ64806.1	Asp23/Gls24_family_envelope_stress_response_protein_[Bacillus_cereus]	CPTF_Cu	2863333.333	284487.8439	9.935547517
UIJ64806.1	Asp23/Gls24_family_envelope_stress_response_protein_[Bacillus_cereus]	CPTF_Fe	3053333.333	202072.5942	6.618098064
UIJ64806.1	Asp23/Gls24_family_envelope_stress_response_protein_[Bacillus_cereus]	CPTF_Mn	3323333.333	267644.0422	8.053481712
UIJ64806.1	Asp23/Gls24_family_envelope_stress_response_protein_[Bacillus_cereus]	CPTF_Ni	3200000	160000	5
UIJ64806.1	Asp23/Gls24_family_envelope_stress_response_protein_[Bacillus_cereus]	CPTF_U	3390000	205182.8453	6.052591306
UIJ64806.1	Asp23/Gls24_family_envelope_stress_response_protein_[Bacillus_cereus]	CPTF_metals_mix	2077626.233	100499.9866	4.837250559
UIJ64806.1	Asp23/Gls24_family_envelope_stress_response_protein_[Bacillus_cereus]	CPTF_zcontrol	3720000	235796.5225	6.338616195
UIJ64807.1	50S_ribosomal_protein_L28_[Bacillus_cereus]	CPTF_Al	1425548.833	182254.6956	12.78487915
UIJ64807.1	50S_ribosomal_protein_L28_[Bacillus_cereus]	CPTF_Cd	1394773.233	196869.0096	14.11476825
UIJ64807.1	50S_ribosomal_protein_L28_[Bacillus_cereus]	CPTF_Co	1563201.867	274948.0618	17.58877517
UIJ64807.1	50S_ribosomal_protein_L28_[Bacillus_cereus]	CPTF_Cu	1485864.867	261994.7319	17.6324737
UIJ64807.1	50S_ribosomal_protein_L28_[Bacillus_cereus]	CPTF_Fe	1496077.567	159919.781	10.6892707
UIJ64807.1	50S_ribosomal_protein_L28_[Bacillus_cereus]	CPTF_Mn	1614540.6	198305.3881	12.28246525
UIJ64807.1	50S_ribosomal_protein_L28_[Bacillus_cereus]	CPTF_Ni	1516262.6	142527.2233	9.399903637
UIJ64807.1	50S_ribosomal_protein_L28_[Bacillus_cereus]	CPTF_U	1683968.333	353401.7394	20.98624615
UIJ64807.1	50S_ribosomal_protein_L28_[Bacillus_cereus]	CPTF_metals_mix	1107483.5	228955.5363	20.67349413
UIJ64807.1	50S_ribosomal_protein_L28_[Bacillus_cereus]	CPTF_zcontrol	1749033	309022.3913	17.66818529

UIJ64809.1	ribulose-phosphate_3-epimerase_[Bacillus_cereus]	CPTF_Al	85130.26667	44860.28154	52.69604254
UIJ64809.1	ribulose-phosphate_3-epimerase_[Bacillus_cereus]	CPTF_Cd	98995.96667	34318.0584	34.66611778
UIJ64809.1	ribulose-phosphate_3-epimerase_[Bacillus_cereus]	CPTF_Co	61532.03333	10991.78029	17.86350896
UIJ64809.1	ribulose-phosphate_3-epimerase_[Bacillus_cereus]	CPTF_Cu	112744	20991.64579	18.61885847
UIJ64809.1	ribulose-phosphate_3-epimerase_[Bacillus_cereus]	CPTF_Fe	32442.23333	28665.85431	88.35968232
UIJ64809.1	ribulose-phosphate_3-epimerase_[Bacillus_cereus]	CPTF_Mn	35695.26667	31184.33275	87.36265522
UIJ64809.1	ribulose-phosphate_3-epimerase_[Bacillus_cereus]	CPTF_Ni	55961.86667	55835.92899	99.77495804
UIJ64809.1	ribulose-phosphate_3-epimerase_[Bacillus_cereus]	CPTF_U	58579.13333	51107.50444	87.24523826
UIJ64809.1	ribulose-phosphate_3-epimerase_[Bacillus_cereus]	CPTF_metals_mix	111345.8333	15701.4218	14.10149022
UIJ64809.1	ribulose-phosphate_3-epimerase_[Bacillus_cereus]	CPTF_zcontrol	22151.33333	38367.23479	173.2050808
UIJ64812.1	Stp1/IreP_family_PP2C-type_Ser/Thr_phosphatase_[Bacillus_cereus]	CPTF_Al	0	0	0
UIJ64812.1	Stp1/IreP_family_PP2C-type_Ser/Thr_phosphatase_[Bacillus_cereus]	CPTF_Cd	6969.233333	12071.06622	173.2050808
UIJ64812.1	Stp1/IreP_family_PP2C-type_Ser/Thr_phosphatase_[Bacillus_cereus]	CPTF_Co	0	0	0
UIJ64812.1	Stp1/IreP_family_PP2C-type_Ser/Thr_phosphatase_[Bacillus_cereus]	CPTF_Cu	36243.8	31706.02649	87.47986273
UIJ64812.1	Stp1/IreP_family_PP2C-type_Ser/Thr_phosphatase_[Bacillus_cereus]	CPTF_Fe	21868.03333	37876.54479	173.2050808
UIJ64812.1	Stp1/IreP_family_PP2C-type_Ser/Thr_phosphatase_[Bacillus_cereus]	CPTF_Mn	25174.6	43603.68626	173.2050808
UIJ64812.1	Stp1/IreP_family_PP2C-type_Ser/Thr_phosphatase_[Bacillus_cereus]	CPTF_Ni	0	0	0
UIJ64812.1	Stp1/IreP_family_PP2C-type_Ser/Thr_phosphatase_[Bacillus_cereus]	CPTF_U	26919.6	46626.11492	173.2050808
UIJ64812.1	Stp1/IreP_family_PP2C-type_Ser/Thr_phosphatase_[Bacillus_cereus]	CPTF_metals_mix	11215.46667	19425.7581	173.2050808
UIJ64812.1	Stp1/IreP_family_PP2C-type_Ser/Thr_phosphatase_[Bacillus_cereus]	CPTF_zcontrol	34553.13333	32770.93547	94.84215267
UIJ64813.1	23S_rRNA_(adenine(2503)-C(2))-methyltransferase_RlmN_[Bacillus_cereus]	CPTF_Al	186211.5	66485.48629	35.70428587
UIJ64813.1	23S_rRNA_(adenine(2503)-C(2))-methyltransferase_RlmN_[Bacillus_cereus]	CPTF_Cd	251269.2333	63341.77185	25.20872572
UIJ64813.1	23S_rRNA_(adenine(2503)-C(2))-methyltransferase_RlmN_[Bacillus_cereus]	CPTF_Co	223714.9	36128.92246	16.14953785
UIJ64813.1	23S_rRNA_(adenine(2503)-C(2))-methyltransferase_RlmN_[Bacillus_cereus]	CPTF_Cu	171775.4333	10682.2587	6.21873483
UIJ64813.1	23S_rRNA_(adenine(2503)-C(2))-methyltransferase_RlmN_[Bacillus_cereus]	CPTF_Fe	250885.8333	12685.84576	5.056421716
UIJ64813.1	23S_rRNA_(adenine(2503)-C(2))-methyltransferase_RlmN_[Bacillus_cereus]	CPTF_Mn	129974.4333	63985.45267	49.22926074
UIJ64813.1	23S_rRNA_(adenine(2503)-C(2))-methyltransferase_RlmN_[Bacillus_cereus]	CPTF_Ni	8439.6	14617.816	173.2050808
UIJ64813.1	23S_rRNA_(adenine(2503)-C(2))-methyltransferase_RlmN_[Bacillus_cereus]	CPTF_U	58275.23333	100935.665	173.2050808
UIJ64813.1	23S_rRNA_(adenine(2503)-C(2))-methyltransferase_RlmN_[Bacillus_cereus]	CPTF_metals_mix	134098.7667	28176.16528	21.01150218
UIJ64813.1	23S_rRNA_(adenine(2503)-C(2))-methyltransferase_RlmN_[Bacillus_cereus]	CPTF_zcontrol	185272.9667	11552.3136	6.23529369
UIJ64815.1	methionyl-tRNA_formyltransferase_[Bacillus_cereus]	CPTF_Al	0	0	0
UIJ64815.1	methionyl-tRNA_formyltransferase_[Bacillus_cereus]	CPTF_Cd	0	0	0
UIJ64815.1	methionyl-tRNA_formyltransferase_[Bacillus_cereus]	CPTF_Co	0	0	0
UIJ64815.1	methionyl-tRNA_formyltransferase_[Bacillus_cereus]	CPTF_Cu	0	0	0
UIJ64815.1	methionyl-tRNA_formyltransferase_[Bacillus_cereus]	CPTF_Fe	0	0	0
UIJ64815.1	methionyl-tRNA_formyltransferase_[Bacillus_cereus]	CPTF_Mn	0	0	0
UIJ64815.1	methionyl-tRNA_formyltransferase_[Bacillus_cereus]	CPTF_Ni	0	0	0
UIJ64815.1	methionyl-tRNA_formyltransferase_[Bacillus_cereus]	CPTF_U	0	0	0
UIJ64815.1	methionyl-tRNA_formyltransferase_[Bacillus_cereus]	CPTF_metals_mix	514220.6	139313.8235	27.09222919
UIJ64815.1	methionyl-tRNA_formyltransferase_[Bacillus_cereus]	CPTF_zcontrol	0	0	0
UIJ64819.1	DNA-directed_RNA_polymerase_subunit_omega_[Bacillus_cereus]	CPTF_Al	0	0	0
UIJ64819.1	DNA-directed_RNA_polymerase_subunit_omega_[Bacillus_cereus]	CPTF_Cd	274477	41939.58587	15.27981793
UIJ64819.1	DNA-directed_RNA_polymerase_subunit_omega_[Bacillus_cereus]	CPTF_Co	0	0	0
UIJ64819.1	DNA-directed_RNA_polymerase_subunit_omega_[Bacillus_cereus]	CPTF_Cu	0	0	0
UIJ64819.1	DNA-directed_RNA_polymerase_subunit_omega_[Bacillus_cereus]	CPTF_Fe	55113	95458.51616	173.2050808
UIJ64819.1	DNA-directed_RNA_polymerase_subunit_omega_[Bacillus_cereus]	CPTF_Mn	129310	223971.4899	173.2050808
UIJ64819.1	DNA-directed_RNA_polymerase_subunit_omega_[Bacillus_cereus]	CPTF_Ni	0	0	0
UIJ64819.1	DNA-directed_RNA_polymerase_subunit_omega_[Bacillus_cereus]	CPTF_U	0	0	0
UIJ64819.1	DNA-directed_RNA_polymerase_subunit_omega_[Bacillus_cereus]	CPTF_metals_mix	825148.3333	48940.76507	5.931147539
UIJ64819.1	DNA-directed_RNA_polymerase_subunit_omega_[Bacillus_cereus]	CPTF_zcontrol	0	0	0
UIJ64820.1	guanylate_kinase_[Bacillus_cereus]	CPTF_Al	759177.6667	137248.6635	18.07859603
UIJ64820.1	guanylate_kinase_[Bacillus_cereus]	CPTF_Cd	701080.6667	88496.72622	12.6229021
UIJ64820.1	guanylate_kinase_[Bacillus_cereus]	CPTF_Co	822386.1667	97880.14237	11.90196848
UIJ64820.1	guanylate_kinase_[Bacillus_cereus]	CPTF_Cu	707451	106613.1118	15.07003478
UIJ64820.1	guanylate_kinase_[Bacillus_cereus]	CPTF_Fe	661395.3333	97570.36013	14.75219966
UIJ64820.1	guanylate_kinase_[Bacillus_cereus]	CPTF_Mn	752701.1333	174433.2662	23.1743063
UIJ64820.1	guanylate_kinase_[Bacillus_cereus]	CPTF_Ni	780569.5333	120578.3383	15.44748202
UIJ64820.1	guanylate_kinase_[Bacillus_cereus]	CPTF_U	706703.3333	64523.55381	9.130217839
UIJ64820.1	guanylate_kinase_[Bacillus_cereus]	CPTF_metals_mix	412014.5333	78368.36858	19.02077772
UIJ64820.1	guanylate_kinase_[Bacillus_cereus]	CPTF_zcontrol	811299.0333	63762.64243	7.859326809
UIJ64824.1	NFACT_RNA_binding_domain-containing_protein_[Bacillus_cereus]	CPTF_Al	159015.3	244982.5448	154.0622473

UIJ64824.1	NFACT_RNA_binding_domain-containing_protein_[Bacillus_cereus]	CPTF_Cd	133934.3	156484.6342	116.8368627
UIJ64824.1	NFACT_RNA_binding_domain-containing_protein_[Bacillus_cereus]	CPTF_Co	57215.43333	72477.3325	126.6744448
UIJ64824.1	NFACT_RNA_binding_domain-containing_protein_[Bacillus_cereus]	CPTF_Cu	66796.93333	67104.58717	100.4605808
UIJ64824.1	NFACT_RNA_binding_domain-containing_protein_[Bacillus_cereus]	CPTF_Fe	175273.6667	303582.8959	173.2050808
UIJ64824.1	NFACT_RNA_binding_domain-containing_protein_[Bacillus_cereus]	CPTF_Mn	12170.26667	21079.52021	173.2050808
UIJ64824.1	NFACT_RNA_binding_domain-containing_protein_[Bacillus_cereus]	CPTF_Ni	0	0	0
UIJ64824.1	NFACT_RNA_binding_domain-containing_protein_[Bacillus_cereus]	CPTF_U	633231.4333	274631.0923	43.36978202
UIJ64824.1	NFACT_RNA_binding_domain-containing_protein_[Bacillus_cereus]	CPTF_metals_mix	515663.2333	107669.694	20.87984697
UIJ64824.1	NFACT_RNA_binding_domain-containing_protein_[Bacillus_cereus]	CPTF_zcontrol	198082.6667	343089.2428	173.2050808
UIJ64826.1	DinB_family_protein_[Bacillus_cereus]	CPTF_Al	42451.7	37141.26027	87.49063117
UIJ64826.1	DinB_family_protein_[Bacillus_cereus]	CPTF_Cd	44103.66667	3938.591203	8.930303307
UIJ64826.1	DinB_family_protein_[Bacillus_cereus]	CPTF_Co	35383.56667	32858.98568	92.86510315
UIJ64826.1	DinB_family_protein_[Bacillus_cereus]	CPTF_Cu	26290.46667	22905.45769	87.12457629
UIJ64826.1	DinB_family_protein_[Bacillus_cereus]	CPTF_Fe	37260.33333	42908.81152	115.1594945
UIJ64826.1	DinB_family_protein_[Bacillus_cereus]	CPTF_Mn	0	0	0
UIJ64826.1	DinB_family_protein_[Bacillus_cereus]	CPTF_Ni	0	0	0
UIJ64826.1	DinB_family_protein_[Bacillus_cereus]	CPTF_U	0	0	0
UIJ64826.1	DinB_family_protein_[Bacillus_cereus]	CPTF_metals_mix	169691.1667	69292.19002	40.83429408
UIJ64826.1	DinB_family_protein_[Bacillus_cereus]	CPTF_zcontrol	0	0	0
UIJ64828.1	orotate_phosphoribosyltransferase_[Bacillus_cereus]	CPTF_Al	2028936.667	379466.9699	18.70275086
UIJ64828.1	orotate_phosphoribosyltransferase_[Bacillus_cereus]	CPTF_Cd	2029221	549008.7048	27.05514603
UIJ64828.1	orotate_phosphoribosyltransferase_[Bacillus_cereus]	CPTF_Co	1988486	541549.9093	27.23428324
UIJ64828.1	orotate_phosphoribosyltransferase_[Bacillus_cereus]	CPTF_Cu	2067062.667	429319.2867	20.76953416
UIJ64828.1	orotate_phosphoribosyltransferase_[Bacillus_cereus]	CPTF_Fe	1944768.333	189624.5964	9.750497946
UIJ64828.1	orotate_phosphoribosyltransferase_[Bacillus_cereus]	CPTF_Mn	1927747.667	330352.0358	17.13668451
UIJ64828.1	orotate_phosphoribosyltransferase_[Bacillus_cereus]	CPTF_Ni	2110568.333	268057.0876	12.70070641
UIJ64828.1	orotate_phosphoribosyltransferase_[Bacillus_cereus]	CPTF_U	2112867.333	419465.0207	19.85288021
UIJ64828.1	orotate_phosphoribosyltransferase_[Bacillus_cereus]	CPTF_metals_mix	1698089.667	266381.5394	15.68713034
UIJ64828.1	orotate_phosphoribosyltransferase_[Bacillus_cereus]	CPTF_zcontrol	1756452.333	146102.3235	8.31803521
UIJ64829.1	orotidine-5'-phosphate_decarboxylase_[Bacillus_cereus]	CPTF_Al	1272964.5	269470.1065	21.16870553
UIJ64829.1	orotidine-5'-phosphate_decarboxylase_[Bacillus_cereus]	CPTF_Cd	1330100.133	151669.7185	11.40287973
UIJ64829.1	orotidine-5'-phosphate_decarboxylase_[Bacillus_cereus]	CPTF_Co	1335130.6	216196.5133	16.19291126
UIJ64829.1	orotidine-5'-phosphate_decarboxylase_[Bacillus_cereus]	CPTF_Cu	1443383.033	42275.47929	2.928916186
UIJ64829.1	orotidine-5'-phosphate_decarboxylase_[Bacillus_cereus]	CPTF_Fe	1382784.333	78970.36949	5.710967906
UIJ64829.1	orotidine-5'-phosphate_decarboxylase_[Bacillus_cereus]	CPTF_Mn	1236085.967	196932.3464	15.93192963
UIJ64829.1	orotidine-5'-phosphate_decarboxylase_[Bacillus_cereus]	CPTF_Ni	1011592	78426.5727	7.752786963
UIJ64829.1	orotidine-5'-phosphate_decarboxylase_[Bacillus_cereus]	CPTF_U	1016689.433	259756.874	25.54928433
UIJ64829.1	orotidine-5'-phosphate_decarboxylase_[Bacillus_cereus]	CPTF_metals_mix	1028806.167	53047.33362	5.156202921
UIJ64829.1	orotidine-5'-phosphate_decarboxylase_[Bacillus_cereus]	CPTF_zcontrol	1388381.5	58093.46232	4.18425788
UIJ64830.1	dihydroorotate_oxidase_B_catalytic_subunit_[Bacillus_cereus]	CPTF_Al	736045.6667	124492.6678	16.9137152
UIJ64830.1	dihydroorotate_oxidase_B_catalytic_subunit_[Bacillus_cereus]	CPTF_Cd	725014	24103.98164	3.324622923
UIJ64830.1	dihydroorotate_oxidase_B_catalytic_subunit_[Bacillus_cereus]	CPTF_Co	809415.3333	336248.8076	41.54218406
UIJ64830.1	dihydroorotate_oxidase_B_catalytic_subunit_[Bacillus_cereus]	CPTF_Cu	687252.3667	231514.1626	33.68692111
UIJ64830.1	dihydroorotate_oxidase_B_catalytic_subunit_[Bacillus_cereus]	CPTF_Fe	672274.3333	76421.37192	11.36758733
UIJ64830.1	dihydroorotate_oxidase_B_catalytic_subunit_[Bacillus_cereus]	CPTF_Mn	462476.2667	233494.4845	50.48788474
UIJ64830.1	dihydroorotate_oxidase_B_catalytic_subunit_[Bacillus_cereus]	CPTF_Ni	588855	148233.8706	25.173238
UIJ64830.1	dihydroorotate_oxidase_B_catalytic_subunit_[Bacillus_cereus]	CPTF_U	496317.6667	87522.91994	17.63445588
UIJ64830.1	dihydroorotate_oxidase_B_catalytic_subunit_[Bacillus_cereus]	CPTF_metals_mix	559516.3333	92173.01994	16.47369602
UIJ64830.1	dihydroorotate_oxidase_B_catalytic_subunit_[Bacillus_cereus]	CPTF_zcontrol	653555	141039.4441	21.58034811
UIJ64831.1	dihydroorotate_oxidase_B_electron_transfer_subunit_[Bacillus_cereus]	CPTF_Al	66779.66667	115665.7756	173.2050808
UIJ64831.1	dihydroorotate_oxidase_B_electron_transfer_subunit_[Bacillus_cereus]	CPTF_Cd	112046.6	30503.36556	27.22382076
UIJ64831.1	dihydroorotate_oxidase_B_electron_transfer_subunit_[Bacillus_cereus]	CPTF_Co	134659.6667	118283.0128	87.83848629
UIJ64831.1	dihydroorotate_oxidase_B_electron_transfer_subunit_[Bacillus_cereus]	CPTF_Cu	113078.3333	103568.4793	91.59002992
UIJ64831.1	dihydroorotate_oxidase_B_electron_transfer_subunit_[Bacillus_cereus]	CPTF_Fe	160760.3333	139233.8919	86.60960635
UIJ64831.1	dihydroorotate_oxidase_B_electron_transfer_subunit_[Bacillus_cereus]	CPTF_Mn	32980.56667	57124.01713	173.2050808
UIJ64831.1	dihydroorotate_oxidase_B_electron_transfer_subunit_[Bacillus_cereus]	CPTF_Ni	185501	53767.39505	28.98496237
UIJ64831.1	dihydroorotate_oxidase_B_electron_transfer_subunit_[Bacillus_cereus]	CPTF_U	99187.06667	120669.0368	121.6580355
UIJ64831.1	dihydroorotate_oxidase_B_electron_transfer_subunit_[Bacillus_cereus]	CPTF_metals_mix	0	0	0
UIJ64831.1	dihydroorotate_oxidase_B_electron_transfer_subunit_[Bacillus_cereus]	CPTF_zcontrol	46098	79844.07813	173.2050808
UIJ64832.1	carbamoyl-phosphate_synthase_large_subunit_[Bacillus_cereus]	CPTF_Al	5315128	471926.7076	8.878934009
UIJ64832.1	carbamoyl-phosphate_synthase_large_subunit_[Bacillus_cereus]	CPTF_Cd	6236827.067	610316.3235	9.785686167

UIJ64832.1	carbamoyl-phosphate_synthase_large_subunit_[Bacillus_cereus]	CPTF_Co	5191094.933	1020913.786	19.66663678
UIJ64832.1	carbamoyl-phosphate_synthase_large_subunit_[Bacillus_cereus]	CPTF_Cu	5823878.7	537422.7645	9.227918234
UIJ64832.1	carbamoyl-phosphate_synthase_large_subunit_[Bacillus_cereus]	CPTF_Fe	5362665.5	221026.2675	4.121574756
UIJ64832.1	carbamoyl-phosphate_synthase_large_subunit_[Bacillus_cereus]	CPTF_Mn	5367710.5	1047897.361	19.52224066
UIJ64832.1	carbamoyl-phosphate_synthase_large_subunit_[Bacillus_cereus]	CPTF_Ni	4478675.8	519392.1237	11.59700204
UIJ64832.1	carbamoyl-phosphate_synthase_large_subunit_[Bacillus_cereus]	CPTF_U	5064647.167	392783.7995	7.755403024
UIJ64832.1	carbamoyl-phosphate_synthase_large_subunit_[Bacillus_cereus]	CPTF_metals_mix	10218621.7	1228481.763	12.02199083
UIJ64832.1	carbamoyl-phosphate_synthase_large_subunit_[Bacillus_cereus]	CPTF_zcontrol	5019167.933	632966.3625	12.61098196
UIJ64833.1	carbamoyl_phosphate_synthase_small_subunit_[Bacillus_cereus]	CPTF_Al	6187521.133	48459.50775	0.783181289
UIJ64833.1	carbamoyl_phosphate_synthase_small_subunit_[Bacillus_cereus]	CPTF_Cd	6030741.333	304330.44	5.046318904
UIJ64833.1	carbamoyl_phosphate_synthase_small_subunit_[Bacillus_cereus]	CPTF_Co	5563649.033	533589.0028	9.590630171
UIJ64833.1	carbamoyl_phosphate_synthase_small_subunit_[Bacillus_cereus]	CPTF_Cu	5892843.467	199861.424	3.391595672
UIJ64833.1	carbamoyl_phosphate_synthase_small_subunit_[Bacillus_cereus]	CPTF_Fe	6168462	754571.4892	12.23273304
UIJ64833.1	carbamoyl_phosphate_synthase_small_subunit_[Bacillus_cereus]	CPTF_Mn	6168298.933	516669.9831	8.376215041
UIJ64833.1	carbamoyl_phosphate_synthase_small_subunit_[Bacillus_cereus]	CPTF_Ni	6257011.167	377254.0214	6.029300754
UIJ64833.1	carbamoyl_phosphate_synthase_small_subunit_[Bacillus_cereus]	CPTF_U	7109972.4	696853.6025	9.801073243
UIJ64833.1	carbamoyl_phosphate_synthase_small_subunit_[Bacillus_cereus]	CPTF_metals_mix	4950460.067	83402.63975	1.684745228
UIJ64833.1	carbamoyl_phosphate_synthase_small_subunit_[Bacillus_cereus]	CPTF_zcontrol	6612801.333	474473.9993	7.175083227
UIJ64834.1	dihydroorotase_[Bacillus_cereus]	CPTF_Al	818817	146621.0202	17.90644554
UIJ64834.1	dihydroorotase_[Bacillus_cereus]	CPTF_Cd	712665.3333	28124.22517	3.946343936
UIJ64834.1	dihydroorotase_[Bacillus_cereus]	CPTF_Co	804382.0667	384635.9209	47.81756541
UIJ64834.1	dihydroorotase_[Bacillus_cereus]	CPTF_Cu	806273.6667	307844.3233	38.18112087
UIJ64834.1	dihydroorotase_[Bacillus_cereus]	CPTF_Fe	814432.6667	88453.81218	10.86078884
UIJ64834.1	dihydroorotase_[Bacillus_cereus]	CPTF_Mn	521845.6667	101550.2925	19.45983248
UIJ64834.1	dihydroorotase_[Bacillus_cereus]	CPTF_Ni	677271	206974.233	30.5600318
UIJ64834.1	dihydroorotase_[Bacillus_cereus]	CPTF_U	731846.6667	285541.4947	39.01657378
UIJ64834.1	dihydroorotase_[Bacillus_cereus]	CPTF_metals_mix	1186692.6	365851.3267	30.82949423
UIJ64834.1	dihydroorotase_[Bacillus_cereus]	CPTF_zcontrol	724478.3333	66041.57385	9.115741743
UIJ64835.1	aspartate_carbamoyltransferase_[Bacillus_cereus]	CPTF_Al	1052799.767	103869.5215	9.866028161
UIJ64835.1	aspartate_carbamoyltransferase_[Bacillus_cereus]	CPTF_Cd	1260024.533	125449.4303	9.95611014
UIJ64835.1	aspartate_carbamoyltransferase_[Bacillus_cereus]	CPTF_Co	1128460.567	131654.6305	11.66674622
UIJ64835.1	aspartate_carbamoyltransferase_[Bacillus_cereus]	CPTF_Cu	1144277.7	70011.53061	6.118403829
UIJ64835.1	aspartate_carbamoyltransferase_[Bacillus_cereus]	CPTF_Fe	1285470.933	75734.69095	5.891591088
UIJ64835.1	aspartate_carbamoyltransferase_[Bacillus_cereus]	CPTF_Mn	1003503.633	50193.87949	5.001863254
UIJ64835.1	aspartate_carbamoyltransferase_[Bacillus_cereus]	CPTF_Ni	1101181.2	269821.371	24.50290388
UIJ64835.1	aspartate_carbamoyltransferase_[Bacillus_cereus]	CPTF_U	1165947.567	164524.4327	14.11079172
UIJ64835.1	aspartate_carbamoyltransferase_[Bacillus_cereus]	CPTF_metals_mix	455661.4	288255.5444	63.26090916
UIJ64835.1	aspartate_carbamoyltransferase_[Bacillus_cereus]	CPTF_zcontrol	1157651.233	134103.3638	11.58409026
UIJ64837.1	bifunctional_pyrimidine_operon_transcriptional_regulator/uracil_phosphoribosyltransferase_[Bacillus_cereus	CPTF_Al	1197868.8	418512.1314	34.93806094
UIJ64837.1	bifunctional_pyrimidine_operon_transcriptional_regulator/uracil_phosphoribosyltransferase_[Bacillus_cereus	CPTF_Cd	1473841.433	265071.0967	17.98504851
UIJ64837.1	bifunctional_pyrimidine_operon_transcriptional_regulator/uracil_phosphoribosyltransferase_[Bacillus_cereus	CPTF_Co	1209402.2	198161.9729	16.38511762
UIJ64837.1	bifunctional_pyrimidine_operon_transcriptional_regulator/uracil_phosphoribosyltransferase_[Bacillus_cereus	CPTF_Cu	1299690.467	346038.6948	26.62470055
UIJ64837.1	bifunctional_pyrimidine_operon_transcriptional_regulator/uracil_phosphoribosyltransferase_[Bacillus_cereus	CPTF_Fe	1029095.133	238754.5113	23.20043148
UIJ64837.1	bifunctional_pyrimidine_operon_transcriptional_regulator/uracil_phosphoribosyltransferase_[Bacillus_cereus	CPTF_Mn	1198555.8	425687.6684	35.51671674
UIJ64837.1	bifunctional_pyrimidine_operon_transcriptional_regulator/uracil_phosphoribosyltransferase_[Bacillus_cereus	CPTF_Ni	949038.3333	224109.9342	23.61442382
UIJ64837.1	bifunctional_pyrimidine_operon_transcriptional_regulator/uracil_phosphoribosyltransferase_[Bacillus_cereus	CPTF_U	1216494.8	279197.2239	22.95095909
UIJ64837.1	bifunctional_pyrimidine_operon_transcriptional_regulator/uracil_phosphoribosyltransferase_[Bacillus_cereus	CPTF_metals_mix	2754353.133	340817.1948	12.37376539
UIJ64837.1	bifunctional_pyrimidine_operon_transcriptional_regulator/uracil_phosphoribosyltransferase_[Bacillus_cereus	CPTF_zcontrol	1442996.333	132030.5653	9.149750577
UIJ64841.1	isoleucine--tRNA_ligase_[Bacillus_cereus]	CPTF_Al	4775857.333	180259.9502	3.774399812
UIJ64841.1	isoleucine--tRNA_ligase_[Bacillus_cereus]	CPTF_Cd	5503162.6	876449.2719	15.9262834
UIJ64841.1	isoleucine--tRNA_ligase_[Bacillus_cereus]	CPTF_Co	5321381.5	363482.7582	6.830608897
UIJ64841.1	isoleucine--tRNA_ligase_[Bacillus_cereus]	CPTF_Cu	5768534.567	497131.3451	8.617983291
UIJ64841.1	isoleucine--tRNA_ligase_[Bacillus_cereus]	CPTF_Fe	4628706.667	415704.2453	8.981002152
UIJ64841.1	isoleucine--tRNA_ligase_[Bacillus_cereus]	CPTF_Mn	4645805.867	290634.1728	6.255839807
UIJ64841.1	isoleucine--tRNA_ligase_[Bacillus_cereus]	CPTF_Ni	4827173.533	583906.6271	12.09624272
UIJ64841.1	isoleucine--tRNA_ligase_[Bacillus_cereus]	CPTF_U	4001202.267	778911.0732	19.46692572
UIJ64841.1	isoleucine--tRNA_ligase_[Bacillus_cereus]	CPTF_metals_mix	6291215.267	472733.5329	7.514184666
UIJ64841.1	isoleucine--tRNA_ligase_[Bacillus_cereus]	CPTF_zcontrol	4191451.9	270055.6639	6.443009973
UIJ64842.1	septum_site-determining_protein_DivIVA_[Bacillus_cereus]	CPTF_Al	3954242.467	122735.7804	3.103901227
UIJ64842.1	septum_site-determining_protein_DivIVA_[Bacillus_cereus]	CPTF_Cd	4203469.7	142736.9502	3.395693568
UIJ64842.1	septum_site-determining_protein_DivIVA_[Bacillus_cereus]	CPTF_Co	4074188.733	149980.0748	3.681225505

UIJ64842.1	septum_site-determining_protein_DivIVA_[Bacillus_cereus]	CPTF_Cu	3853851.233	130434.513	3.384523822
UIJ64842.1	septum_site-determining_protein_DivIVA_[Bacillus_cereus]	CPTF_Fe	3969082.967	385399.9901	9.710051247
UIJ64842.1	septum_site-determining_protein_DivIVA_[Bacillus_cereus]	CPTF_Mn	4293759.667	286610.2488	6.675041712
UIJ64842.1	septum_site-determining_protein_DivIVA_[Bacillus_cereus]	CPTF_Ni	3264578.667	162626.4135	4.981543719
UIJ64842.1	septum_site-determining_protein_DivIVA_[Bacillus_cereus]	CPTF_U	3676457	1326831.547	36.08995147
UIJ64842.1	septum_site-determining_protein_DivIVA_[Bacillus_cereus]	CPTF_metals_mix	5738662.167	777066.4848	13.54089964
UIJ64842.1	septum_site-determining_protein_DivIVA_[Bacillus_cereus]	CPTF_zcontrol	4166130.433	275055.3332	6.60217767
UIJ64844.1	YggT_family_protein_[Bacillus_cereus]	CPTF_Al	637390	63011.0392	9.885790364
UIJ64844.1	YggT_family_protein_[Bacillus_cereus]	CPTF_Cd	596343.6667	57627.70726	9.66350621
UIJ64844.1	YggT_family_protein_[Bacillus_cereus]	CPTF_Co	667175.3333	26112.70013	3.9139187
UIJ64844.1	YggT_family_protein_[Bacillus_cereus]	CPTF_Cu	672058	69131.92528	10.28660105
UIJ64844.1	YggT_family_protein_[Bacillus_cereus]	CPTF_Fe	658898.6667	100411.8047	15.23933949
UIJ64844.1	YggT_family_protein_[Bacillus_cereus]	CPTF_Mn	651369.3333	160284.353	24.60729187
UIJ64844.1	YggT_family_protein_[Bacillus_cereus]	CPTF_Ni	705312.6667	57416.24182	8.140537457
UIJ64844.1	YggT_family_protein_[Bacillus_cereus]	CPTF_U	730161.6667	74845.64397	10.25055784
UIJ64844.1	YggT_family_protein_[Bacillus_cereus]	CPTF_metals_mix	368858.6667	30928.36532	8.384882372
UIJ64844.1	YggT_family_protein_[Bacillus_cereus]	CPTF_zcontrol	705058.3333	4473.740754	0.634520655
UIJ64845.1	cell_division_protein_SepF_[Bacillus_cereus]	CPTF_Al	150321.6667	15457.65378	10.28305109
UIJ64845.1	cell_division_protein_SepF_[Bacillus_cereus]	CPTF_Cd	91523	79435.28767	86.7927053
UIJ64845.1	cell_division_protein_SepF_[Bacillus_cereus]	CPTF_Co	213078.8	116031.9845	54.4549643
UIJ64845.1	cell_division_protein_SepF_[Bacillus_cereus]	CPTF_Cu	59024.66667	102233.7216	173.2050808
UIJ64845.1	cell_division_protein_SepF_[Bacillus_cereus]	CPTF_Fe	101283	88674.49413	87.55121208
UIJ64845.1	cell_division_protein_SepF_[Bacillus_cereus]	CPTF_Mn	65516	113477.0407	173.2050808
UIJ64845.1	cell_division_protein_SepF_[Bacillus_cereus]	CPTF_Ni	67719.33333	117293.326	173.2050808
UIJ64845.1	cell_division_protein_SepF_[Bacillus_cereus]	CPTF_U	33698.33333	58367.22546	173.2050808
UIJ64845.1	cell_division_protein_SepF_[Bacillus_cereus]	CPTF_metals_mix	220626.6333	41842.46124	18.96528112
UIJ64845.1	cell_division_protein_SepF_[Bacillus_cereus]	CPTF_zcontrol	260366.7	46349.11208	17.80147464
UIJ64846.1	YggS_family_pyridoxal_phosphate-dependent_enzyme_[Bacillus_cereus]	CPTF_Al	334423.7667	188202.8491	56.27675657
UIJ64846.1	YggS_family_pyridoxal_phosphate-dependent_enzyme_[Bacillus_cereus]	CPTF_Cd	394592.6667	63022.865	15.97162601
UIJ64846.1	YggS_family_pyridoxal_phosphate-dependent_enzyme_[Bacillus_cereus]	CPTF_Co	512393.3	37923.22128	7.401193825
UIJ64846.1	YggS_family_pyridoxal_phosphate-dependent_enzyme_[Bacillus_cereus]	CPTF_Cu	455805.7	13818.72523	3.031714003
UIJ64846.1	YggS_family_pyridoxal_phosphate-dependent_enzyme_[Bacillus_cereus]	CPTF_Fe	318081.7333	197761.2117	62.17308038
UIJ64846.1	YggS_family_pyridoxal_phosphate-dependent_enzyme_[Bacillus_cereus]	CPTF_Mn	278473.2	231179.4717	83.01677565
UIJ64846.1	YggS_family_pyridoxal_phosphate-dependent_enzyme_[Bacillus_cereus]	CPTF_Ni	216977.5667	94691.73634	43.64125647
UIJ64846.1	YggS_family_pyridoxal_phosphate-dependent_enzyme_[Bacillus_cereus]	CPTF_U	211450.7667	229783.2814	108.6698739
UIJ64846.1	YggS_family_pyridoxal_phosphate-dependent_enzyme_[Bacillus_cereus]	CPTF_metals_mix	823747.2667	60621.37661	7.359220365
UIJ64846.1	YggS_family_pyridoxal_phosphate-dependent_enzyme_[Bacillus_cereus]	CPTF_zcontrol	316505.1	215198.89	67.99223456
UIJ64847.1	peptidoglycan_editing_factor_PgeF_[Bacillus_cereus]	CPTF_Al	619027	179678.3815	29.02593611
UIJ64847.1	peptidoglycan_editing_factor_PgeF_[Bacillus_cereus]	CPTF_Cd	755393.6667	46833.96377	6.19994128
UIJ64847.1	peptidoglycan_editing_factor_PgeF_[Bacillus_cereus]	CPTF_Co	744711.1667	136529.6445	18.3332345
UIJ64847.1	peptidoglycan_editing_factor_PgeF_[Bacillus_cereus]	CPTF_Cu	828316.6667	22197.79571	2.679868292
UIJ64847.1	peptidoglycan_editing_factor_PgeF_[Bacillus_cereus]	CPTF_Fe	542766	209573.1613	38.61206511
UIJ64847.1	peptidoglycan_editing_factor_PgeF_[Bacillus_cereus]	CPTF_Mn	494029.3333	162596.2026	32.91225675
UIJ64847.1	peptidoglycan_editing_factor_PgeF_[Bacillus_cereus]	CPTF_Ni	770869.3333	75446.19031	9.787156791
UIJ64847.1	peptidoglycan_editing_factor_PgeF_[Bacillus_cereus]	CPTF_U	634662.3333	188334.877	29.67481558
UIJ64847.1	peptidoglycan_editing_factor_PgeF_[Bacillus_cereus]	CPTF_metals_mix	866822.9667	65855.81542	7.597377775
UIJ64847.1	peptidoglycan_editing_factor_PgeF_[Bacillus_cereus]	CPTF_zcontrol	716701	37126.5094	5.180195004
UIJ64852.1	cell_division_protein_FtsZ_[Bacillus_cereus]	CPTF_Al	12321092.43	139933.9705	1.135726976
UIJ64852.1	cell_division_protein_FtsZ_[Bacillus_cereus]	CPTF_Cd	13635674.37	1504685.012	11.03491453
UIJ64852.1	cell_division_protein_FtsZ_[Bacillus_cereus]	CPTF_Co	12178427.9	96444.46492	0.791928693
UIJ64852.1	cell_division_protein_FtsZ_[Bacillus_cereus]	CPTF_Cu	12287759	1284232.518	10.45131597
UIJ64852.1	cell_division_protein_FtsZ_[Bacillus_cereus]	CPTF_Fe	12701357.87	1221227.228	9.614934406
UIJ64852.1	cell_division_protein_FtsZ_[Bacillus_cereus]	CPTF_Mn	12845000.13	1088697.441	8.475651461
UIJ64852.1	cell_division_protein_FtsZ_[Bacillus_cereus]	CPTF_Ni	13266264.67	1216152.436	9.167255945
UIJ64852.1	cell_division_protein_FtsZ_[Bacillus_cereus]	CPTF_U	12314575.5	999893.4079	8.11959298
UIJ64852.1	cell_division_protein_FtsZ_[Bacillus_cereus]	CPTF_metals_mix	14301724.47	1223448.597	8.554552986
UIJ64852.1	cell_division_protein_FtsZ_[Bacillus_cereus]	CPTF_zcontrol	12838838.8	893685.1542	6.96079426
UIJ64853.1	cell_division_protein_FtsA_[Bacillus_cereus]	CPTF_Al	2765151.933	134756.6699	4.87339116
UIJ64853.1	cell_division_protein_FtsA_[Bacillus_cereus]	CPTF_Cd	2773678.067	28252.16093	1.018581113
UIJ64853.1	cell_division_protein_FtsA_[Bacillus_cereus]	CPTF_Co	2647844.167	111988.6141	4.22942617
UIJ64853.1	cell_division_protein_FtsA_[Bacillus_cereus]	CPTF_Cu	2537146.767	81947.33938	3.229901417

UIJ64853.1	cell_division_protein_FtsA_[Bacillus_cereus]	CPTF_Fe	2561054.1	259562.2543	10.1349774
UIJ64853.1	cell_division_protein_FtsA_[Bacillus_cereus]	CPTF_Mn	2366264.5	460016.095	19.44060332
UIJ64853.1	cell_division_protein_FtsA_[Bacillus_cereus]	CPTF_Ni	2714052.933	149695.8939	5.515584903
UIJ64853.1	cell_division_protein_FtsA_[Bacillus_cereus]	CPTF_U	2896095	68866.75753	2.377917766
UIJ64853.1	cell_division_protein_FtsA_[Bacillus_cereus]	CPTF_metals_mix	2746559.8	487287.4989	17.74174001
UIJ64853.1	cell_division_protein_FtsA_[Bacillus_cereus]	CPTF_zcontrol	2760321.867	65776.31893	2.382922069
UIJ64859.1	UDP-N-acetylmuramoyl-L-alanyl-D-glutamate--2,6-diaminopimelate_ligase_[Bacillus_cereus]	CPTF_Al	214667.3333	30072.76569	14.00900883
UIJ64859.1	UDP-N-acetylmuramoyl-L-alanyl-D-glutamate--2,6-diaminopimelate_ligase_[Bacillus_cereus]	CPTF_Cd	225276.6	7634.593105	3.388986297
UIJ64859.1	UDP-N-acetylmuramoyl-L-alanyl-D-glutamate--2,6-diaminopimelate_ligase_[Bacillus_cereus]	CPTF_Co	221396.7	7080.165935	3.197954592
UIJ64859.1	UDP-N-acetylmuramoyl-L-alanyl-D-glutamate--2,6-diaminopimelate_ligase_[Bacillus_cereus]	CPTF_Cu	208093.3333	34823.3223	16.73447282
UIJ64859.1	UDP-N-acetylmuramoyl-L-alanyl-D-glutamate--2,6-diaminopimelate_ligase_[Bacillus_cereus]	CPTF_Fe	212972.3333	21544.68494	10.11618956
UIJ64859.1	UDP-N-acetylmuramoyl-L-alanyl-D-glutamate--2,6-diaminopimelate_ligase_[Bacillus_cereus]	CPTF_Mn	218360.6667	36669.25416	16.79297591
UIJ64859.1	UDP-N-acetylmuramoyl-L-alanyl-D-glutamate--2,6-diaminopimelate_ligase_[Bacillus_cereus]	CPTF_Ni	218644.6667	6221.989821	2.84570848
UIJ64859.1	UDP-N-acetylmuramoyl-L-alanyl-D-glutamate--2,6-diaminopimelate_ligase_[Bacillus_cereus]	CPTF_U	240137	28594.42423	11.9075462
UIJ64859.1	UDP-N-acetylmuramoyl-L-alanyl-D-glutamate--2,6-diaminopimelate_ligase_[Bacillus_cereus]	CPTF_metals_mix	143901.6667	21111.62737	14.67087065
UIJ64859.1	UDP-N-acetylmuramoyl-L-alanyl-D-glutamate--2,6-diaminopimelate_ligase_[Bacillus_cereus]	CPTF_zcontrol	222219.3333	16774.98901	7.54884319
UIJ64863.1	16S_rRNA_(cytosine(1402)-N(4))-methyltransferase_RsmH_[Bacillus_cereus]	CPTF_Al	0	0	0
UIJ64863.1	16S_rRNA_(cytosine(1402)-N(4))-methyltransferase_RsmH_[Bacillus_cereus]	CPTF_Cd	0	0	0
UIJ64863.1	16S_rRNA_(cytosine(1402)-N(4))-methyltransferase_RsmH_[Bacillus_cereus]	CPTF_Co	0	0	0
UIJ64863.1	16S_rRNA_(cytosine(1402)-N(4))-methyltransferase_RsmH_[Bacillus_cereus]	CPTF_Cu	103774.7667	90283.26098	86.99924257
UIJ64863.1	16S_rRNA_(cytosine(1402)-N(4))-methyltransferase_RsmH_[Bacillus_cereus]	CPTF_Fe	71106	123159.2047	173.2050808
UIJ64863.1	16S_rRNA_(cytosine(1402)-N(4))-methyltransferase_RsmH_[Bacillus_cereus]	CPTF_Mn	0	0	0
UIJ64863.1	16S_rRNA_(cytosine(1402)-N(4))-methyltransferase_RsmH_[Bacillus_cereus]	CPTF_Ni	59585	103204.2474	173.2050808
UIJ64863.1	16S_rRNA_(cytosine(1402)-N(4))-methyltransferase_RsmH_[Bacillus_cereus]	CPTF_U	0	0	0
UIJ64863.1	16S_rRNA_(cytosine(1402)-N(4))-methyltransferase_RsmH_[Bacillus_cereus]	CPTF_metals_mix	129890.7	37535.42382	28.89769924
UIJ64863.1	16S_rRNA_(cytosine(1402)-N(4))-methyltransferase_RsmH_[Bacillus_cereus]	CPTF_zcontrol	33863.66667	58653.5912	173.2050808
UIJ64864.1	bacillithiol_biosynthesis_cysteine-adding_enzyme_BshC_[Bacillus_cereus]	CPTF_Al	0	0	0
UIJ64864.1	bacillithiol_biosynthesis_cysteine-adding_enzyme_BshC_[Bacillus_cereus]	CPTF_Cd	0	0	0
UIJ64864.1	bacillithiol_biosynthesis_cysteine-adding_enzyme_BshC_[Bacillus_cereus]	CPTF_Co	0	0	0
UIJ64864.1	bacillithiol_biosynthesis_cysteine-adding_enzyme_BshC_[Bacillus_cereus]	CPTF_Cu	17265.23333	29904.26134	173.2050808
UIJ64864.1	bacillithiol_biosynthesis_cysteine-adding_enzyme_BshC_[Bacillus_cereus]	CPTF_Fe	0	0	0
UIJ64864.1	bacillithiol_biosynthesis_cysteine-adding_enzyme_BshC_[Bacillus_cereus]	CPTF_Mn	0	0	0
UIJ64864.1	bacillithiol_biosynthesis_cysteine-adding_enzyme_BshC_[Bacillus_cereus]	CPTF_Ni	0	0	0
UIJ64864.1	bacillithiol_biosynthesis_cysteine-adding_enzyme_BshC_[Bacillus_cereus]	CPTF_U	0	0	0
UIJ64864.1	bacillithiol_biosynthesis_cysteine-adding_enzyme_BshC_[Bacillus_cereus]	CPTF_metals_mix	102175.8	53261.95325	52.1277575
UIJ64864.1	bacillithiol_biosynthesis_cysteine-adding_enzyme_BshC_[Bacillus_cereus]	CPTF_zcontrol	0	0	0
UIJ64865.1	N-acetyltransferase_[Bacillus_cereus]	CPTF_Al	19208.43333	33269.98247	173.2050808
UIJ64865.1	N-acetyltransferase_[Bacillus_cereus]	CPTF_Cd	77451.96667	33095.05539	42.72978055
UIJ64865.1	N-acetyltransferase_[Bacillus_cereus]	CPTF_Co	6897.733333	11947.22459	173.2050808
UIJ64865.1	N-acetyltransferase_[Bacillus_cereus]	CPTF_Cu	9001	15590.18932	173.2050808
UIJ64865.1	N-acetyltransferase_[Bacillus_cereus]	CPTF_Fe	212401.0333	153124.631	72.09222505
UIJ64865.1	N-acetyltransferase_[Bacillus_cereus]	CPTF_Mn	30453.93333	52747.75982	173.2050808
UIJ64865.1	N-acetyltransferase_[Bacillus_cereus]	CPTF_Ni	0	0	0
UIJ64865.1	N-acetyltransferase_[Bacillus_cereus]	CPTF_U	69748.33333	120807.6571	173.2050808
UIJ64865.1	N-acetyltransferase_[Bacillus_cereus]	CPTF_metals_mix	0	0	0
UIJ64865.1	N-acetyltransferase_[Bacillus_cereus]	CPTF_zcontrol	92775.66667	160692.1684	173.2050808
UIJ64867.1	50S_ribosomal_protein_L32_[Bacillus_cereus]	CPTF_Al	487280.6667	154292.1626	31.66392043
UIJ64867.1	50S_ribosomal_protein_L32_[Bacillus_cereus]	CPTF_Cd	113625.6667	196805.4277	173.2050808
UIJ64867.1	50S_ribosomal_protein_L32_[Bacillus_cereus]	CPTF_Co	320973.3333	278141.4062	86.65561194
UIJ64867.1	50S_ribosomal_protein_L32_[Bacillus_cereus]	CPTF_Cu	296977.3333	257253.0485	86.62379908
UIJ64867.1	50S_ribosomal_protein_L32_[Bacillus_cereus]	CPTF_Fe	278180	251917.7527	90.55926114
UIJ64867.1	50S_ribosomal_protein_L32_[Bacillus_cereus]	CPTF_Mn	435235.6667	69144.52971	15.88668738
UIJ64867.1	50S_ribosomal_protein_L32_[Bacillus_cereus]	CPTF_Ni	151628.3333	262627.9772	173.2050808
UIJ64867.1	50S_ribosomal_protein_L32_[Bacillus_cereus]	CPTF_U	232269.3333	402302.2864	173.2050808
UIJ64867.1	50S_ribosomal_protein_L32_[Bacillus_cereus]	CPTF_metals_mix	555768.3333	145972.7115	26.26502857
UIJ64867.1	50S_ribosomal_protein_L32_[Bacillus_cereus]	CPTF_zcontrol	183365.6667	317598.651	173.2050808
UIJ64868.1	DUF177_domain-containing_protein_[Bacillus_cereus]	CPTF_Al	82464.43333	4384.390266	5.316704534
UIJ64868.1	DUF177_domain-containing_protein_[Bacillus_cereus]	CPTF_Cd	36513.66667	63243.52584	173.2050808
UIJ64868.1	DUF177_domain-containing_protein_[Bacillus_cereus]	CPTF_Co	75412.13333	37028.51196	49.10153091
UIJ64868.1	DUF177_domain-containing_protein_[Bacillus_cereus]	CPTF_Cu	83171.36667	76525.96745	92.00999156
UIJ64868.1	DUF177_domain-containing_protein_[Bacillus_cereus]	CPTF_Fe	56276.46667	48834.9161	86.77679853

UIJ64868.1	DUF177_domain-containing_protein_[Bacillus_cereus]	CPTF_Mn	0	0	0
UIJ64868.1	DUF177_domain-containing_protein_[Bacillus_cereus]	CPTF_Ni	27428.63333	47507.78652	173.2050808
UIJ64868.1	DUF177_domain-containing_protein_[Bacillus_cereus]	CPTF_U	0	0	0
UIJ64868.1	DUF177_domain-containing_protein_[Bacillus_cereus]	CPTF_metals_mix	209028	19506.9554	9.332221234
UIJ64868.1	DUF177_domain-containing_protein_[Bacillus_cereus]	CPTF_zcontrol	0	0	0
UIJ64870.1	PDZ_domain-containing_protein_[Bacillus_cereus]	CPTF_Al	85874.66667	78027.70727	90.86231167
UIJ64870.1	PDZ_domain-containing_protein_[Bacillus_cereus]	CPTF_Cd	17141.76667	29690.4108	173.2050808
UIJ64870.1	PDZ_domain-containing_protein_[Bacillus_cereus]	CPTF_Co	0	0	0
UIJ64870.1	PDZ_domain-containing_protein_[Bacillus_cereus]	CPTF_Cu	0	0	0
UIJ64870.1	PDZ_domain-containing_protein_[Bacillus_cereus]	CPTF_Fe	21056.7	36471.27424	173.2050808
UIJ64870.1	PDZ_domain-containing_protein_[Bacillus_cereus]	CPTF_Mn	32902.3	56988.45529	173.2050808
UIJ64870.1	PDZ_domain-containing_protein_[Bacillus_cereus]	CPTF_Ni	0	0	0
UIJ64870.1	PDZ_domain-containing_protein_[Bacillus_cereus]	CPTF_U	0	0	0
UIJ64870.1	PDZ_domain-containing_protein_[Bacillus_cereus]	CPTF_metals_mix	87452.66667	80073.52057	91.56212569
UIJ64870.1	PDZ_domain-containing_protein_[Bacillus_cereus]	CPTF_zcontrol	0	0	0
UIJ64873.1	pantetheine-phosphate_adenylyltransferase_[Bacillus_cereus]	CPTF_Al	847074.6667	40680.15685	4.802428694
UIJ64873.1	pantetheine-phosphate_adenylyltransferase_[Bacillus_cereus]	CPTF_Cd	979026.3333	76534.12165	7.8173711
UIJ64873.1	pantetheine-phosphate_adenylyltransferase_[Bacillus_cereus]	CPTF_Co	1040308	131367.6719	12.62776716
UIJ64873.1	pantetheine-phosphate_adenylyltransferase_[Bacillus_cereus]	CPTF_Cu	947842.6667	65685.81784	6.930033871
UIJ64873.1	pantetheine-phosphate_adenylyltransferase_[Bacillus_cereus]	CPTF_Fe	960581	103401.4153	10.76446601
UIJ64873.1	pantetheine-phosphate_adenylyltransferase_[Bacillus_cereus]	CPTF_Mn	863435.6667	240308.5353	27.83166651
UIJ64873.1	pantetheine-phosphate_adenylyltransferase_[Bacillus_cereus]	CPTF_Ni	1076986.267	179171.9949	16.63642337
UIJ64873.1	pantetheine-phosphate_adenylyltransferase_[Bacillus_cereus]	CPTF_U	1152712.667	56599.07845	4.910076907
UIJ64873.1	pantetheine-phosphate_adenylyltransferase_[Bacillus_cereus]	CPTF_metals_mix	788741.3333	63138.89074	8.005018639
UIJ64873.1	pantetheine-phosphate_adenylyltransferase_[Bacillus_cereus]	CPTF_zcontrol	911106.3333	225257.0352	24.72346278
UIJ64875.1	methylthioribose_kinase_[Bacillus_cereus]	CPTF_Al	50882.1	67895.95934	133.4378089
UIJ64875.1	methylthioribose_kinase_[Bacillus_cereus]	CPTF_Cd	81126.7	79449.30085	97.93237103
UIJ64875.1	methylthioribose_kinase_[Bacillus_cereus]	CPTF_Co	46257.36667	61776.16836	133.5488222
UIJ64875.1	methylthioribose_kinase_[Bacillus_cereus]	CPTF_Cu	136339.3667	91133.14792	66.84287169
UIJ64875.1	methylthioribose_kinase_[Bacillus_cereus]	CPTF_Fe	51559.4	51054.22961	99.02021671
UIJ64875.1	methylthioribose_kinase_[Bacillus_cereus]	CPTF_Mn	89746.66667	80656.2028	89.87097325
UIJ64875.1	methylthioribose_kinase_[Bacillus_cereus]	CPTF_Ni	98378.3	86736.6691	88.16646466
UIJ64875.1	methylthioribose_kinase_[Bacillus_cereus]	CPTF_U	32800.06667	56811.38196	173.2050808
UIJ64875.1	methylthioribose_kinase_[Bacillus_cereus]	CPTF_metals_mix	153605.9	113522.554	73.90507394
UIJ64875.1	methylthioribose_kinase_[Bacillus_cereus]	CPTF_zcontrol	37074.33333	64214.62899	173.2050808
UIJ64881.1	YugN-like_family_protein_[Bacillus_cereus]	CPTF_Al	215950.7	156221.3922	72.34122983
UIJ64881.1	YugN-like_family_protein_[Bacillus_cereus]	CPTF_Cd	159698.3333	140974.4828	88.27548781
UIJ64881.1	YugN-like_family_protein_[Bacillus_cereus]	CPTF_Co	254835.1333	222407.1679	87.27492359
UIJ64881.1	YugN-like_family_protein_[Bacillus_cereus]	CPTF_Cu	157789	140948.2812	89.32706409
UIJ64881.1	YugN-like_family_protein_[Bacillus_cereus]	CPTF_Fe	113674.7	196890.3559	173.2050808
UIJ64881.1	YugN-like_family_protein_[Bacillus_cereus]	CPTF_Mn	246333	261532.1742	106.1701738
UIJ64881.1	YugN-like_family_protein_[Bacillus_cereus]	CPTF_Ni	183804	160276.7399	87.19981059
UIJ64881.1	YugN-like_family_protein_[Bacillus_cereus]	CPTF_U	0	0	0
UIJ64881.1	YugN-like_family_protein_[Bacillus_cereus]	CPTF_metals_mix	792876.6667	101689.8784	12.82543461
UIJ64881.1	YugN-like_family_protein_[Bacillus_cereus]	CPTF_zcontrol	10919.66667	18913.41747	173.2050808
UIJ64882.1	formamidase_[Bacillus_cereus]	CPTF_Al	167435.4	135040.8647	80.65251713
UIJ64882.1	formamidase_[Bacillus_cereus]	CPTF_Cd	296589.1	179692.4918	60.58634382
UIJ64882.1	formamidase_[Bacillus_cereus]	CPTF_Co	208862.7667	55892.0436	26.76017583
UIJ64882.1	formamidase_[Bacillus_cereus]	CPTF_Cu	159375.3	108334.2372	67.97429541
UIJ64882.1	formamidase_[Bacillus_cereus]	CPTF_Fe	193390.2333	13947.47294	7.212087551
UIJ64882.1	formamidase_[Bacillus_cereus]	CPTF_Mn	215884.7333	30086.55385	13.93639716
UIJ64882.1	formamidase_[Bacillus_cereus]	CPTF_Ni	114035.3	104281.1925	91.44641392
UIJ64882.1	formamidase_[Bacillus_cereus]	CPTF_U	101902.1667	138789.9012	136.199166
UIJ64882.1	formamidase_[Bacillus_cereus]	CPTF_metals_mix	351914.9	63794.10627	18.12770822
UIJ64882.1	formamidase_[Bacillus_cereus]	CPTF_zcontrol	104862.9333	126757.7158	120.8794297
UIJ64890.1	pyruvate_carboxylase_[Bacillus_cereus]	CPTF_Al	8602699.7	439706.7449	5.111264605
UIJ64890.1	pyruvate_carboxylase_[Bacillus_cereus]	CPTF_Cd	10154783.83	960779.4678	9.461348302
UIJ64890.1	pyruvate_carboxylase_[Bacillus_cereus]	CPTF_Co	8319890.4	1299514.698	15.6193728
UIJ64890.1	pyruvate_carboxylase_[Bacillus_cereus]	CPTF_Cu	9775350.167	496610.6867	5.080234244
UIJ64890.1	pyruvate_carboxylase_[Bacillus_cereus]	CPTF_Fe	8419561	1336355.089	15.87202811
UIJ64890.1	pyruvate_carboxylase_[Bacillus_cereus]	CPTF_Mn	8305753.5	848285.7756	10.21323081

UIJ64890.1	pyruvate_carboxylase_[Bacillus_cereus]	CPTF_Ni	7028979.267	1910157.742	27.17546417
UIJ64890.1	pyruvate_carboxylase_[Bacillus_cereus]	CPTF_U	7694832	359862.7389	4.676680907
UIJ64890.1	pyruvate_carboxylase_[Bacillus_cereus]	CPTF_metals_mix	17325812.57	1075077.68	6.205063549
UIJ64890.1	pyruvate_carboxylase_[Bacillus_cereus]	CPTF_zcontrol	8002208.5	49657.82149	0.620551458
UIJ64892.1	YlaN_family_protein_[Bacillus_cereus]	CPTF_Al	725852	103008.0458	14.19132907
UIJ64892.1	YlaN_family_protein_[Bacillus_cereus]	CPTF_Cd	736215.4	116669.3266	15.8471728
UIJ64892.1	YlaN_family_protein_[Bacillus_cereus]	CPTF_Co	294704.3333	335715.5623	113.916059
UIJ64892.1	YlaN_family_protein_[Bacillus_cereus]	CPTF_Cu	229483	397476.2155	173.2050808
UIJ64892.1	YlaN_family_protein_[Bacillus_cereus]	CPTF_Fe	335303	419243.0316	125.0340831
UIJ64892.1	YlaN_family_protein_[Bacillus_cereus]	CPTF_Mn	595491.3333	544314.5733	91.40596056
UIJ64892.1	YlaN_family_protein_[Bacillus_cereus]	CPTF_Ni	477847	413912.2067	86.62023759
UIJ64892.1	YlaN_family_protein_[Bacillus_cereus]	CPTF_U	295226.3333	511347.0091	173.2050808
UIJ64892.1	YlaN_family_protein_[Bacillus_cereus]	CPTF_metals_mix	2157207.167	425291.31	19.71490344
UIJ64892.1	YlaN_family_protein_[Bacillus_cereus]	CPTF_zcontrol	430269.3333	372669.2475	86.61301622
UIJ64893.1	peptidyl-prolyl_cis-trans_isomerase_[Bacillus_cereus]	CPTF_Al	131839.4	37631.34779	28.54332452
UIJ64893.1	peptidyl-prolyl_cis-trans_isomerase_[Bacillus_cereus]	CPTF_Cd	85125	69462.45839	81.60053849
UIJ64893.1	peptidyl-prolyl_cis-trans_isomerase_[Bacillus_cereus]	CPTF_Co	93748.9	38534.09772	41.10351985
UIJ64893.1	peptidyl-prolyl_cis-trans_isomerase_[Bacillus_cereus]	CPTF_Cu	81305.86667	60962.98991	74.97981684
UIJ64893.1	peptidyl-prolyl_cis-trans_isomerase_[Bacillus_cereus]	CPTF_Fe	86148.6	16914.99058	19.63466682
UIJ64893.1	peptidyl-prolyl_cis-trans_isomerase_[Bacillus_cereus]	CPTF_Mn	34451.66667	59672.03707	173.2050808
UIJ64893.1	peptidyl-prolyl_cis-trans_isomerase_[Bacillus_cereus]	CPTF_Ni	8636.666667	14959.14547	173.2050808
UIJ64893.1	peptidyl-prolyl_cis-trans_isomerase_[Bacillus_cereus]	CPTF_U	0	0	0
UIJ64893.1	peptidyl-prolyl_cis-trans_isomerase_[Bacillus_cereus]	CPTF_metals_mix	270570.7667	206665.1055	76.38116567
UIJ64893.1	peptidyl-prolyl_cis-trans_isomerase_[Bacillus_cereus]	CPTF_zcontrol	8232.5	14259.10827	173.2050808
UIJ64894.1	PhoH_family_protein_[Bacillus_cereus]	CPTF_Al	69800.6	77938.61552	111.6589478
UIJ64894.1	PhoH_family_protein_[Bacillus_cereus]	CPTF_Cd	15245.46667	26405.92285	173.2050808
UIJ64894.1	PhoH_family_protein_[Bacillus_cereus]	CPTF_Co	80810.76667	76135.64457	94.21472869
UIJ64894.1	PhoH_family_protein_[Bacillus_cereus]	CPTF_Cu	111765.4	102822.67	91.99865974
UIJ64894.1	PhoH_family_protein_[Bacillus_cereus]	CPTF_Fe	53339.9	92387.41687	173.2050808
UIJ64894.1	PhoH_family_protein_[Bacillus_cereus]	CPTF_Mn	46507.63333	45744.18823	98.35845205
UIJ64894.1	PhoH_family_protein_[Bacillus_cereus]	CPTF_Ni	80047.73333	38751.77252	48.41083052
UIJ64894.1	PhoH_family_protein_[Bacillus_cereus]	CPTF_U	17055.96667	29541.80084	173.2050808
UIJ64894.1	PhoH_family_protein_[Bacillus_cereus]	CPTF_metals_mix	65479.8	50778.63965	77.54855643
UIJ64894.1	PhoH_family_protein_[Bacillus_cereus]	CPTF_zcontrol	34023.53333	58930.48839	173.2050808
UIJ64895.1	pyridoxamine_5'-phosphate_oxidase_family_protein_[Bacillus_cereus]	CPTF_Al	156437.5667	138268.0489	88.38545101
UIJ64895.1	pyridoxamine_5'-phosphate_oxidase_family_protein_[Bacillus_cereus]	CPTF_Cd	254926.6667	182432.7146	71.56282119
UIJ64895.1	pyridoxamine_5'-phosphate_oxidase_family_protein_[Bacillus_cereus]	CPTF_Co	0	0	0
UIJ64895.1	pyridoxamine_5'-phosphate_oxidase_family_protein_[Bacillus_cereus]	CPTF_Cu	14918.46667	25839.54224	173.2050808
UIJ64895.1	pyridoxamine_5'-phosphate_oxidase_family_protein_[Bacillus_cereus]	CPTF_Fe	73163.06667	107901.5483	147.4808988
UIJ64895.1	pyridoxamine_5'-phosphate_oxidase_family_protein_[Bacillus_cereus]	CPTF_Mn	82001.26667	127418.7875	155.3863649
UIJ64895.1	pyridoxamine_5'-phosphate_oxidase_family_protein_[Bacillus_cereus]	CPTF_Ni	0	0	0
UIJ64895.1	pyridoxamine_5'-phosphate_oxidase_family_protein_[Bacillus_cereus]	CPTF_U	9047.933333	15671.48024	173.2050808
UIJ64895.1	pyridoxamine_5'-phosphate_oxidase_family_protein_[Bacillus_cereus]	CPTF_metals_mix	356556.8333	334764.5904	93.88814323
UIJ64895.1	pyridoxamine_5'-phosphate_oxidase_family_protein_[Bacillus_cereus]	CPTF_zcontrol	0	0	0
UIJ64899.1	translational_GTPase_TypA_[Bacillus_cereus]	CPTF_Al	2299978.333	84726.65983	3.683802521
UIJ64899.1	translational_GTPase_TypA_[Bacillus_cereus]	CPTF_Cd	2551840.167	90414.76472	3.543120212
UIJ64899.1	translational_GTPase_TypA_[Bacillus_cereus]	CPTF_Co	2575244.633	105237.4485	4.086502973
UIJ64899.1	translational_GTPase_TypA_[Bacillus_cereus]	CPTF_Cu	2642971.3	200838.3078	7.598959089
UIJ64899.1	translational_GTPase_TypA_[Bacillus_cereus]	CPTF_Fe	2236023.067	105212.5015	4.705340616
UIJ64899.1	translational_GTPase_TypA_[Bacillus_cereus]	CPTF_Mn	2452489.867	356004.1159	14.51602801
UIJ64899.1	translational_GTPase_TypA_[Bacillus_cereus]	CPTF_Ni	2189997.8	295486.9285	13.49256737
UIJ64899.1	translational_GTPase_TypA_[Bacillus_cereus]	CPTF_U	2070462.4	159007.9518	7.679828032
UIJ64899.1	translational_GTPase_TypA_[Bacillus_cereus]	CPTF_metals_mix	4154082.033	648709.0114	15.61618202
UIJ64899.1	translational_GTPase_TypA_[Bacillus_cereus]	CPTF_zcontrol	2204036.667	276270.3106	12.53474204
UIJ64901.1	inositol_monophosphatase_family_protein_[Bacillus_cereus]	CPTF_Al	12077.43333	20918.72816	173.2050808
UIJ64901.1	inositol_monophosphatase_family_protein_[Bacillus_cereus]	CPTF_Cd	31917.5	27641.40587	86.60266584
UIJ64901.1	inositol_monophosphatase_family_protein_[Bacillus_cereus]	CPTF_Co	35317.96667	33707.6334	95.44047005
UIJ64901.1	inositol_monophosphatase_family_protein_[Bacillus_cereus]	CPTF_Cu	19098.2	33079.05273	173.2050808
UIJ64901.1	inositol_monophosphatase_family_protein_[Bacillus_cereus]	CPTF_Fe	40201.46667	15046.82969	37.42855904
UIJ64901.1	inositol_monophosphatase_family_protein_[Bacillus_cereus]	CPTF_Mn	0	0	0
UIJ64901.1	inositol_monophosphatase_family_protein_[Bacillus_cereus]	CPTF_Ni	35133.53333	30531.62853	86.90167379

UIJ64901.1	inositol_monophosphatase_family_protein_[Bacillus_cereus]	CPTF_U	0	0	0
UIJ64901.1	inositol_monophosphatase_family_protein_[Bacillus_cereus]	CPTF_metals_mix	0	0	0
UIJ64901.1	inositol_monophosphatase_family_protein_[Bacillus_cereus]	CPTF_zcontrol	11124.3	19267.8528	173.2050808
UIJ64905.1	arginine_decarboxylase_[Bacillus_cereus]	CPTF_Al	128000.4667	48101.40477	37.57908547
UIJ64905.1	arginine_decarboxylase_[Bacillus_cereus]	CPTF_Cd	66368.36667	57712.1097	86.95725479
UIJ64905.1	arginine_decarboxylase_[Bacillus_cereus]	CPTF_Co	155770.5	136068.5358	87.35192852
UIJ64905.1	arginine_decarboxylase_[Bacillus_cereus]	CPTF_Cu	175665.6333	69216.92785	39.4026575
UIJ64905.1	arginine_decarboxylase_[Bacillus_cereus]	CPTF_Fe	144380.7	47662.33113	33.01156673
UIJ64905.1	arginine_decarboxylase_[Bacillus_cereus]	CPTF_Mn	155423.5667	134619.5672	86.61464289
UIJ64905.1	arginine_decarboxylase_[Bacillus_cereus]	CPTF_Ni	64340.26667	111440.6108	173.2050808
UIJ64905.1	arginine_decarboxylase_[Bacillus_cereus]	CPTF_U	193920.9	37475.50071	19.32514789
UIJ64905.1	arginine_decarboxylase_[Bacillus_cereus]	CPTF_metals_mix	182058.1333	83922.76049	46.09668294
UIJ64905.1	arginine_decarboxylase_[Bacillus_cereus]	CPTF_zcontrol	132110.5	26640.83961	20.16557322
UIJ64909.1	GapA-binding_peptide_SR1P_[Bacillus_cereus]	CPTF_Al	0	0	0
UIJ64909.1	GapA-binding_peptide_SR1P_[Bacillus_cereus]	CPTF_Cd	0	0	0
UIJ64909.1	GapA-binding_peptide_SR1P_[Bacillus_cereus]	CPTF_Co	0	0	0
UIJ64909.1	GapA-binding_peptide_SR1P_[Bacillus_cereus]	CPTF_Cu	0	0	0
UIJ64909.1	GapA-binding_peptide_SR1P_[Bacillus_cereus]	CPTF_Fe	0	0	0
UIJ64909.1	GapA-binding_peptide_SR1P_[Bacillus_cereus]	CPTF_Mn	0	0	0
UIJ64909.1	GapA-binding_peptide_SR1P_[Bacillus_cereus]	CPTF_Ni	0	0	0
UIJ64909.1	GapA-binding_peptide_SR1P_[Bacillus_cereus]	CPTF_U	0	0	0
UIJ64909.1	GapA-binding_peptide_SR1P_[Bacillus_cereus]	CPTF_metals_mix	218043.3333	52353.10043	24.01041097
UIJ64909.1	GapA-binding_peptide_SR1P_[Bacillus_cereus]	CPTF_zcontrol	0	0	0
UIJ64910.1	DUF1885_family_protein_[Bacillus_cereus]	CPTF_Al	17632.76667	30540.84774	173.2050808
UIJ64910.1	DUF1885_family_protein_[Bacillus_cereus]	CPTF_Cd	0	0	0
UIJ64910.1	DUF1885_family_protein_[Bacillus_cereus]	CPTF_Co	0	0	0
UIJ64910.1	DUF1885_family_protein_[Bacillus_cereus]	CPTF_Cu	0	0	0
UIJ64910.1	DUF1885_family_protein_[Bacillus_cereus]	CPTF_Fe	0	0	0
UIJ64910.1	DUF1885_family_protein_[Bacillus_cereus]	CPTF_Mn	0	0	0
UIJ64910.1	DUF1885_family_protein_[Bacillus_cereus]	CPTF_Ni	0	0	0
UIJ64910.1	DUF1885_family_protein_[Bacillus_cereus]	CPTF_U	0	0	0
UIJ64910.1	DUF1885_family_protein_[Bacillus_cereus]	CPTF_metals_mix	32291.86667	28332.37094	87.73841177
UIJ64910.1	DUF1885_family_protein_[Bacillus_cereus]	CPTF_zcontrol	0	0	0
UIJ64912.1	dihydrolipoyl_dehydrogenase_[Bacillus_cereus]	CPTF_Al	27403953.77	2660344.404	9.707885319
UIJ64912.1	dihydrolipoyl_dehydrogenase_[Bacillus_cereus]	CPTF_Cd	29339017.67	2628742.646	8.959886374
UIJ64912.1	dihydrolipoyl_dehydrogenase_[Bacillus_cereus]	CPTF_Co	25307364.07	2587381.17	10.2238272
UIJ64912.1	dihydrolipoyl_dehydrogenase_[Bacillus_cereus]	CPTF_Cu	26058653.1	863542.0589	3.313839958
UIJ64912.1	dihydrolipoyl_dehydrogenase_[Bacillus_cereus]	CPTF_Fe	26395398.3	521046.2952	1.974004292
UIJ64912.1	dihydrolipoyl_dehydrogenase_[Bacillus_cereus]	CPTF_Mn	26517649.87	2465044.174	9.295862139
UIJ64912.1	dihydrolipoyl_dehydrogenase_[Bacillus_cereus]	CPTF_Ni	22502936.03	1524502.792	6.774683935
UIJ64912.1	dihydrolipoyl_dehydrogenase_[Bacillus_cereus]	CPTF_U	22587884.3	1764821.693	7.813134112
UIJ64912.1	dihydrolipoyl_dehydrogenase_[Bacillus_cereus]	CPTF_metals_mix	26655826.6	1570913.077	5.893319687
UIJ64912.1	dihydrolipoyl_dehydrogenase_[Bacillus_cereus]	CPTF_zcontrol	25151592.83	348377.8556	1.385112497
UIJ64913.1	pyruvate_dehydrogenase_complex_dihydrolipoyllysine-residue_acetyltransferase_[Bacillus_cereus]	CPTF_Al	28477194.8	2379379.341	8.355385275
UIJ64913.1	pyruvate_dehydrogenase_complex_dihydrolipoyllysine-residue_acetyltransferase_[Bacillus_cereus]	CPTF_Cd	28273088.27	663491.2612	2.346723693
UIJ64913.1	pyruvate_dehydrogenase_complex_dihydrolipoyllysine-residue_acetyltransferase_[Bacillus_cereus]	CPTF_Co	26814894.13	1113671.208	4.153181446
UIJ64913.1	pyruvate_dehydrogenase_complex_dihydrolipoyllysine-residue_acetyltransferase_[Bacillus_cereus]	CPTF_Cu	26920938.9	769870.268	2.859745237
UIJ64913.1	pyruvate_dehydrogenase_complex_dihydrolipoyllysine-residue_acetyltransferase_[Bacillus_cereus]	CPTF_Fe	26658844.53	325586.3511	1.221307063
UIJ64913.1	pyruvate_dehydrogenase_complex_dihydrolipoyllysine-residue_acetyltransferase_[Bacillus_cereus]	CPTF_Mn	28484515.93	1576694.911	5.535270162
UIJ64913.1	pyruvate_dehydrogenase_complex_dihydrolipoyllysine-residue_acetyltransferase_[Bacillus_cereus]	CPTF_Ni	26338389	1082265.953	4.109081817
UIJ64913.1	pyruvate_dehydrogenase_complex_dihydrolipoyllysine-residue_acetyltransferase_[Bacillus_cereus]	CPTF_U	28294660	1570504.509	5.550533241
UIJ64913.1	pyruvate_dehydrogenase_complex_dihydrolipoyllysine-residue_acetyltransferase_[Bacillus_cereus]	CPTF_metals_mix	20746746.97	752421.6446	3.626696975
UIJ64913.1	pyruvate_dehydrogenase_complex_dihydrolipoyllysine-residue_acetyltransferase_[Bacillus_cereus]	CPTF_zcontrol	29067075.4	1712393.301	5.891178514
UIJ64914.1	pyruvate_dehydrogenase_complex_E1_component_subunit_beta_[Bacillus_cereus]	CPTF_Al	30685309.1	2859199.338	9.317811753
UIJ64914.1	pyruvate_dehydrogenase_complex_E1_component_subunit_beta_[Bacillus_cereus]	CPTF_Cd	33039879.7	1274609.758	3.857791764
UIJ64914.1	pyruvate_dehydrogenase_complex_E1_component_subunit_beta_[Bacillus_cereus]	CPTF_Co	28900476.67	1607976.055	5.563839219
UIJ64914.1	pyruvate_dehydrogenase_complex_E1_component_subunit_beta_[Bacillus_cereus]	CPTF_Cu	29008544.97	441750.8094	1.522829945
UIJ64914.1	pyruvate_dehydrogenase_complex_E1_component_subunit_beta_[Bacillus_cereus]	CPTF_Fe	29491482.7	2097828.936	7.113338307
UIJ64914.1	pyruvate_dehydrogenase_complex_E1_component_subunit_beta_[Bacillus_cereus]	CPTF_Mn	31992732.23	6876778.262	21.49481392
UIJ64914.1	pyruvate_dehydrogenase_complex_E1_component_subunit_beta_[Bacillus_cereus]	CPTF_Ni	24575282.37	1993558.267	8.112046231
UIJ64914.1	pyruvate_dehydrogenase_complex_E1_component_subunit_beta_[Bacillus_cereus]	CPTF_U	25744059.9	1446827.489	5.620043981

UIJ64914.1	pyruvate_dehydrogenase_complex_E1_component_subunit_beta_[Bacillus_cereus]	CPTF_metals_mix	25307399.27	20253.75753	0.080030972
UIJ64914.1	pyruvate_dehydrogenase_complex_E1_component_subunit_beta_[Bacillus_cereus]	CPTF_zcontrol	30123512.7	1275216.745	4.233293631
UIJ64915.1	pyruvate_dehydrogenase_E1_component_subunit_alpha_[Bacillus_cereus]	CPTF_Al	17963264.93	1988348.233	11.06896904
UIJ64915.1	pyruvate_dehydrogenase_E1_component_subunit_alpha_[Bacillus_cereus]	CPTF_Cd	19342189.17	406286.4872	2.100519665
UIJ64915.1	pyruvate_dehydrogenase_E1_component_subunit_alpha_[Bacillus_cereus]	CPTF_Co	16122913.67	1209084.847	7.499170882
UIJ64915.1	pyruvate_dehydrogenase_E1_component_subunit_alpha_[Bacillus_cereus]	CPTF_Cu	16055031	1800567.609	11.21497435
UIJ64915.1	pyruvate_dehydrogenase_E1_component_subunit_alpha_[Bacillus_cereus]	CPTF_Fe	16810451.33	1381496.688	8.218082075
UIJ64915.1	pyruvate_dehydrogenase_E1_component_subunit_alpha_[Bacillus_cereus]	CPTF_Mn	17743054.67	2587686.92	14.58422447
UIJ64915.1	pyruvate_dehydrogenase_E1_component_subunit_alpha_[Bacillus_cereus]	CPTF_Ni	13172349	689897.7447	5.237469374
UIJ64915.1	pyruvate_dehydrogenase_E1_component_subunit_alpha_[Bacillus_cereus]	CPTF_U	13877051	769556.8459	5.545535906
UIJ64915.1	pyruvate_dehydrogenase_E1_component_subunit_alpha_[Bacillus_cereus]	CPTF_metals_mix	12579862.6	747067.5574	5.938598705
UIJ64915.1	pyruvate_dehydrogenase_E1_component_subunit_alpha_[Bacillus_cereus]	CPTF_zcontrol	17498232.5	353764.5282	2.021715783
UIJ64917.1	peptide_deformylase_[Bacillus_cereus]	CPTF_Al	752890.5333	30035.18539	3.989316383
UIJ64917.1	peptide_deformylase_[Bacillus_cereus]	CPTF_Cd	645701.6667	156213.4678	24.192824
UIJ64917.1	peptide_deformylase_[Bacillus_cereus]	CPTF_Co	758789.3333	78829.4987	10.38885172
UIJ64917.1	peptide_deformylase_[Bacillus_cereus]	CPTF_Cu	916496.6	9741.136878	1.062866668
UIJ64917.1	peptide_deformylase_[Bacillus_cereus]	CPTF_Fe	640423.6667	196737.6441	30.71992094
UIJ64917.1	peptide_deformylase_[Bacillus_cereus]	CPTF_Mn	702667.3	58487.33385	8.323616859
UIJ64917.1	peptide_deformylase_[Bacillus_cereus]	CPTF_Ni	631278.3	203467.1441	32.2309739
UIJ64917.1	peptide_deformylase_[Bacillus_cereus]	CPTF_U	534155	147522.4686	27.61791401
UIJ64917.1	peptide_deformylase_[Bacillus_cereus]	CPTF_metals_mix	577359.7333	152311.9247	26.38076677
UIJ64917.1	peptide_deformylase_[Bacillus_cereus]	CPTF_zcontrol	706659.5333	45831.00647	6.485585251
UIJ64918.1	Cof-type_HAD-IIB_family_hydrolase_[Bacillus_cereus]	CPTF_Al	25261.93333	43754.95203	173.2050808
UIJ64918.1	Cof-type_HAD-IIB_family_hydrolase_[Bacillus_cereus]	CPTF_Cd	0	0	0
UIJ64918.1	Cof-type_HAD-IIB_family_hydrolase_[Bacillus_cereus]	CPTF_Co	27842.6	24960.8976	89.65002406
UIJ64918.1	Cof-type_HAD-IIB_family_hydrolase_[Bacillus_cereus]	CPTF_Cu	17850.86667	16724.1452	93.68814137
UIJ64918.1	Cof-type_HAD-IIB_family_hydrolase_[Bacillus_cereus]	CPTF_Fe	5671.766667	9823.788035	173.2050808
UIJ64918.1	Cof-type_HAD-IIB_family_hydrolase_[Bacillus_cereus]	CPTF_Mn	23266.46667	22861.90271	98.26117147
UIJ64918.1	Cof-type_HAD-IIB_family_hydrolase_[Bacillus_cereus]	CPTF_Ni	0	0	0
UIJ64918.1	Cof-type_HAD-IIB_family_hydrolase_[Bacillus_cereus]	CPTF_U	34717	60131.60789	173.2050808
UIJ64918.1	Cof-type_HAD-IIB_family_hydrolase_[Bacillus_cereus]	CPTF_metals_mix	162252.6333	37465.0034	23.09053642
UIJ64918.1	Cof-type_HAD-IIB_family_hydrolase_[Bacillus_cereus]	CPTF_zcontrol	28998.36667	50226.6444	173.2050808
UIJ64919.1	DNA-dependent_RNA_polymerase_auxiliary_subunit_epsilon_family_protein_[Bacillus_cereus]	CPTF_Al	0	0	0
UIJ64919.1	DNA-dependent_RNA_polymerase_auxiliary_subunit_epsilon_family_protein_[Bacillus_cereus]	CPTF_Cd	0	0	0
UIJ64919.1	DNA-dependent_RNA_polymerase_auxiliary_subunit_epsilon_family_protein_[Bacillus_cereus]	CPTF_Co	0	0	0
UIJ64919.1	DNA-dependent_RNA_polymerase_auxiliary_subunit_epsilon_family_protein_[Bacillus_cereus]	CPTF_Cu	0	0	0
UIJ64919.1	DNA-dependent_RNA_polymerase_auxiliary_subunit_epsilon_family_protein_[Bacillus_cereus]	CPTF_Fe	0	0	0
UIJ64919.1	DNA-dependent_RNA_polymerase_auxiliary_subunit_epsilon_family_protein_[Bacillus_cereus]	CPTF_Mn	0	0	0
UIJ64919.1	DNA-dependent_RNA_polymerase_auxiliary_subunit_epsilon_family_protein_[Bacillus_cereus]	CPTF_Ni	0	0	0
UIJ64919.1	DNA-dependent_RNA_polymerase_auxiliary_subunit_epsilon_family_protein_[Bacillus_cereus]	CPTF_U	0	0	0
UIJ64919.1	DNA-dependent_RNA_polymerase_auxiliary_subunit_epsilon_family_protein_[Bacillus_cereus]	CPTF_metals_mix	158957	164518.7944	103.4989302
UIJ64919.1	DNA-dependent_RNA_polymerase_auxiliary_subunit_epsilon_family_protein_[Bacillus_cereus]	CPTF_zcontrol	0	0	0
UIJ64920.1	ribonuclease_J1_[Bacillus_cereus]	CPTF_Al	4051165.833	902441.0339	22.27608227
UIJ64920.1	ribonuclease_J1_[Bacillus_cereus]	CPTF_Cd	4247121.367	1030136.101	24.25492496
UIJ64920.1	ribonuclease_J1_[Bacillus_cereus]	CPTF_Co	4441857.4	536718.9409	12.08320963
UIJ64920.1	ribonuclease_J1_[Bacillus_cereus]	CPTF_Cu	4465074.933	443197.236	9.925863343
UIJ64920.1	ribonuclease_J1_[Bacillus_cereus]	CPTF_Fe	4611708.133	926771.2458	20.09605159
UIJ64920.1	ribonuclease_J1_[Bacillus_cereus]	CPTF_Mn	4029454.967	290497.867	7.20935882
UIJ64920.1	ribonuclease_J1_[Bacillus_cereus]	CPTF_Ni	3541748.233	221830.3951	6.263302202
UIJ64920.1	ribonuclease_J1_[Bacillus_cereus]	CPTF_U	3529498.433	493701.5599	13.98786738
UIJ64920.1	ribonuclease_J1_[Bacillus_cereus]	CPTF_metals_mix	6503246.167	592062.9957	9.104114783
UIJ64920.1	ribonuclease_J1_[Bacillus_cereus]	CPTF_zcontrol	4333252.567	727276.9137	16.78362621
UIJ64921.1	TrkA_family_potassium_uptake_protein_[Bacillus_cereus]	CPTF_Al	213972.3333	10000.4858	4.673728444
UIJ64921.1	TrkA_family_potassium_uptake_protein_[Bacillus_cereus]	CPTF_Cd	179110.2	76730.19241	42.83965537
UIJ64921.1	TrkA_family_potassium_uptake_protein_[Bacillus_cereus]	CPTF_Co	219473	6678.036613	3.042759981
UIJ64921.1	TrkA_family_potassium_uptake_protein_[Bacillus_cereus]	CPTF_Cu	207584.3333	22629.82723	10.90151018
UIJ64921.1	TrkA_family_potassium_uptake_protein_[Bacillus_cereus]	CPTF_Fe	225151.6667	36309.78601	16.12681201
UIJ64921.1	TrkA_family_potassium_uptake_protein_[Bacillus_cereus]	CPTF_Mn	224853	9291.105639	4.13207991
UIJ64921.1	TrkA_family_potassium_uptake_protein_[Bacillus_cereus]	CPTF_Ni	247344	25719.36914	10.39821833
UIJ64921.1	TrkA_family_potassium_uptake_protein_[Bacillus_cereus]	CPTF_U	283067.6667	87451.75295	30.89429251
UIJ64921.1	TrkA_family_potassium_uptake_protein_[Bacillus_cereus]	CPTF_metals_mix	205350.3	36031.34637	17.54628377

UIJ64921.1	TrkA_family_potassium_uptake_protein_[Bacillus_cereus]	CPTF_zcontrol	196585	45906.93008	23.35220392
UIJ64923.1	N-acetyldiaminopimelate_deacetylase_[Bacillus_cereus]	CPTF_Al	297293.6667	257564.9281	86.63653383
UIJ64923.1	N-acetyldiaminopimelate_deacetylase_[Bacillus_cereus]	CPTF_Cd	379441.6667	23308.64604	6.142879944
UIJ64923.1	N-acetyldiaminopimelate_deacetylase_[Bacillus_cereus]	CPTF_Co	181052.8333	185859.1728	102.6546613
UIJ64923.1	N-acetyldiaminopimelate_deacetylase_[Bacillus_cereus]	CPTF_Cu	401693.6333	30816.40883	7.67161993
UIJ64923.1	N-acetyldiaminopimelate_deacetylase_[Bacillus_cereus]	CPTF_Fe	437815.5333	26251.03373	5.995911915
UIJ64923.1	N-acetyldiaminopimelate_deacetylase_[Bacillus_cereus]	CPTF_Mn	161355.7333	279476.3282	173.2050808
UIJ64923.1	N-acetyldiaminopimelate_deacetylase_[Bacillus_cereus]	CPTF_Ni	397974.9667	141548.9284	35.56729449
UIJ64923.1	N-acetyldiaminopimelate_deacetylase_[Bacillus_cereus]	CPTF_U	481102.6667	51563.0593	10.71768312
UIJ64923.1	N-acetyldiaminopimelate_deacetylase_[Bacillus_cereus]	CPTF_metals_mix	122958.3333	212970.0805	173.2050808
UIJ64923.1	N-acetyldiaminopimelate_deacetylase_[Bacillus_cereus]	CPTF_zcontrol	499801.9667	42012.77565	8.40588442
UIJ64924.1	2,3,4,5-tetrahydropyridine-2,6-dicarboxylate_N-acetyltransferase_[Bacillus_cereus]	CPTF_Al	5995299	454360.9404	7.578620188
UIJ64924.1	2,3,4,5-tetrahydropyridine-2,6-dicarboxylate_N-acetyltransferase_[Bacillus_cereus]	CPTF_Cd	7158233.333	806085.218	11.26095198
UIJ64924.1	2,3,4,5-tetrahydropyridine-2,6-dicarboxylate_N-acetyltransferase_[Bacillus_cereus]	CPTF_Co	6079198.1	557852.1471	9.176410077
UIJ64924.1	2,3,4,5-tetrahydropyridine-2,6-dicarboxylate_N-acetyltransferase_[Bacillus_cereus]	CPTF_Cu	6133018.967	51676.48714	0.842594608
UIJ64924.1	2,3,4,5-tetrahydropyridine-2,6-dicarboxylate_N-acetyltransferase_[Bacillus_cereus]	CPTF_Fe	6412776.133	183703.5907	2.864649988
UIJ64924.1	2,3,4,5-tetrahydropyridine-2,6-dicarboxylate_N-acetyltransferase_[Bacillus_cereus]	CPTF_Mn	6614468.9	140958.8002	2.131067548
UIJ64924.1	2,3,4,5-tetrahydropyridine-2,6-dicarboxylate_N-acetyltransferase_[Bacillus_cereus]	CPTF_Ni	5331916.867	96660.92322	1.812873787
UIJ64924.1	2,3,4,5-tetrahydropyridine-2,6-dicarboxylate_N-acetyltransferase_[Bacillus_cereus]	CPTF_U	6604942	210270.5824	3.183534124
UIJ64924.1	2,3,4,5-tetrahydropyridine-2,6-dicarboxylate_N-acetyltransferase_[Bacillus_cereus]	CPTF_metals_mix	7989914.933	1458767.95	18.25761554
UIJ64924.1	2,3,4,5-tetrahydropyridine-2,6-dicarboxylate_N-acetyltransferase_[Bacillus_cereus]	CPTF_zcontrol	6813536.333	487463.4848	7.154338965
UIJ64930.1	YkuJ_family_protein_[Bacillus_cereus]	CPTF_Al	1614856.333	75426.92871	4.670813567
UIJ64930.1	YkuJ_family_protein_[Bacillus_cereus]	CPTF_Cd	1409777.333	439217.8407	31.15512147
UIJ64930.1	YkuJ_family_protein_[Bacillus_cereus]	CPTF_Co	1660717.667	183102.3252	11.02549391
UIJ64930.1	YkuJ_family_protein_[Bacillus_cereus]	CPTF_Cu	1701388.667	17151.7117	1.008100738
UIJ64930.1	YkuJ_family_protein_[Bacillus_cereus]	CPTF_Fe	1658959	142451.5955	8.586806277
UIJ64930.1	YkuJ_family_protein_[Bacillus_cereus]	CPTF_Mn	1628183	120464.2037	7.398689438
UIJ64930.1	YkuJ_family_protein_[Bacillus_cereus]	CPTF_Ni	1398787.333	343255.6211	24.53951454
UIJ64930.1	YkuJ_family_protein_[Bacillus_cereus]	CPTF_U	1132149.333	328720.2814	29.03506381
UIJ64930.1	YkuJ_family_protein_[Bacillus_cereus]	CPTF_metals_mix	1536180.133	13155.48239	0.856376287
UIJ64930.1	YkuJ_family_protein_[Bacillus_cereus]	CPTF_zcontrol	1628604.667	80196.3397	4.924236148
UIJ64934.1	2,4-dienoyl-CoA_reductase_[Bacillus_cereus]	CPTF_Al	2173495.567	295312.0474	13.58696341
UIJ64934.1	2,4-dienoyl-CoA_reductase_[Bacillus_cereus]	CPTF_Cd	3202785.033	480198.835	14.9931647
UIJ64934.1	2,4-dienoyl-CoA_reductase_[Bacillus_cereus]	CPTF_Co	1749347.367	159102.1293	9.094942053
UIJ64934.1	2,4-dienoyl-CoA_reductase_[Bacillus_cereus]	CPTF_Cu	1603373.6	161303.3301	10.0602461
UIJ64934.1	2,4-dienoyl-CoA_reductase_[Bacillus_cereus]	CPTF_Fe	2330555.2	658357.3013	28.24894691
UIJ64934.1	2,4-dienoyl-CoA_reductase_[Bacillus_cereus]	CPTF_Mn	2355857.367	1403082.824	59.55720596
UIJ64934.1	2,4-dienoyl-CoA_reductase_[Bacillus_cereus]	CPTF_Ni	965826.1333	415833.4324	43.05468842
UIJ64934.1	2,4-dienoyl-CoA_reductase_[Bacillus_cereus]	CPTF_U	729803.5667	371512.5334	50.90582594
UIJ64934.1	2,4-dienoyl-CoA_reductase_[Bacillus_cereus]	CPTF_metals_mix	2348219.667	238370.5749	10.15111909
UIJ64934.1	2,4-dienoyl-CoA_reductase_[Bacillus_cereus]	CPTF_zcontrol	1727834.767	435189.0242	25.18695842
UIJ64938.1	cytoplasmic_protein_[Bacillus_cereus]	CPTF_Al	13673.56667	23683.31219	173.2050808
UIJ64938.1	cytoplasmic_protein_[Bacillus_cereus]	CPTF_Cd	0	0	0
UIJ64938.1	cytoplasmic_protein_[Bacillus_cereus]	CPTF_Co	17389.1	30118.8047	173.2050808
UIJ64938.1	cytoplasmic_protein_[Bacillus_cereus]	CPTF_Cu	0	0	0
UIJ64938.1	cytoplasmic_protein_[Bacillus_cereus]	CPTF_Fe	13538.13333	23448.73477	173.2050808
UIJ64938.1	cytoplasmic_protein_[Bacillus_cereus]	CPTF_Mn	41688.83333	37379.80662	89.66383473
UIJ64938.1	cytoplasmic_protein_[Bacillus_cereus]	CPTF_Ni	0	0	0
UIJ64938.1	cytoplasmic_protein_[Bacillus_cereus]	CPTF_U	0	0	0
UIJ64938.1	cytoplasmic_protein_[Bacillus_cereus]	CPTF_metals_mix	73734	9989.28161	13.54772779
UIJ64938.1	cytoplasmic_protein_[Bacillus_cereus]	CPTF_zcontrol	21446.6	37146.60085	173.2050808
UIJ64939.1	YkyA_family_protein_[Bacillus_cereus]	CPTF_Al	0	0	0
UIJ64939.1	YkyA_family_protein_[Bacillus_cereus]	CPTF_Cd	0	0	0
UIJ64939.1	YkyA_family_protein_[Bacillus_cereus]	CPTF_Co	11027.43333	19100.07481	173.2050808
UIJ64939.1	YkyA_family_protein_[Bacillus_cereus]	CPTF_Cu	0	0	0
UIJ64939.1	YkyA_family_protein_[Bacillus_cereus]	CPTF_Fe	0	0	0
UIJ64939.1	YkyA_family_protein_[Bacillus_cereus]	CPTF_Mn	0	0	0
UIJ64939.1	YkyA_family_protein_[Bacillus_cereus]	CPTF_Ni	0	0	0
UIJ64939.1	YkyA_family_protein_[Bacillus_cereus]	CPTF_U	0	0	0
UIJ64939.1	YkyA_family_protein_[Bacillus_cereus]	CPTF_metals_mix	9746.9	16882.12602	173.2050808
UIJ64939.1	YkyA_family_protein_[Bacillus_cereus]	CPTF_zcontrol	0	0	0

UIJ64945.1	5-methyltetrahydropteroyltriglutamate--homocysteine_S-methyltransferase_[Bacillus_cereus]	CPTF_Al	1411097.867	274601.0399	19.46009886
UIJ64945.1	5-methyltetrahydropteroyltriglutamate--homocysteine_S-methyltransferase_[Bacillus_cereus]	CPTF_Cd	1796765.167	322136.354	17.92868428
UIJ64945.1	5-methyltetrahydropteroyltriglutamate--homocysteine_S-methyltransferase_[Bacillus_cereus]	CPTF_Co	1574693.1	120632.6092	7.660706027
UIJ64945.1	5-methyltetrahydropteroyltriglutamate--homocysteine_S-methyltransferase_[Bacillus_cereus]	CPTF_Cu	1179195.9	224296.8669	19.02117086
UIJ64945.1	5-methyltetrahydropteroyltriglutamate--homocysteine_S-methyltransferase_[Bacillus_cereus]	CPTF_Fe	1058855.667	374499.9933	35.36837031
UIJ64945.1	5-methyltetrahydropteroyltriglutamate--homocysteine_S-methyltransferase_[Bacillus_cereus]	CPTF_Mn	1074382.067	266670.9899	24.82087129
UIJ64945.1	5-methyltetrahydropteroyltriglutamate--homocysteine_S-methyltransferase_[Bacillus_cereus]	CPTF_Ni	1046316.6	210113.4537	20.08125014
UIJ64945.1	5-methyltetrahydropteroyltriglutamate--homocysteine_S-methyltransferase_[Bacillus_cereus]	CPTF_U	1284077.333	259974.8917	20.24604632
UIJ64945.1	5-methyltetrahydropteroyltriglutamate--homocysteine_S-methyltransferase_[Bacillus_cereus]	CPTF_metals_mix	2359848.733	34173.50556	1.448122716
UIJ64945.1	5-methyltetrahydropteroyltriglutamate--homocysteine_S-methyltransferase_[Bacillus_cereus]	CPTF_zcontrol	1396364	419085.9772	30.01265982
UIJ64949.1	hypothetical_protein_LW858_18470_[Bacillus_cereus]	CPTF_Al	131584.8	60761.4143	46.17662094
UIJ64949.1	hypothetical_protein_LW858_18470_[Bacillus_cereus]	CPTF_Cd	140497.6667	33531.59323	23.86629901
UIJ64949.1	hypothetical_protein_LW858_18470_[Bacillus_cereus]	CPTF_Co	124191.8667	56275.93118	45.31370104
UIJ64949.1	hypothetical_protein_LW858_18470_[Bacillus_cereus]	CPTF_Cu	123256.9	78503.69634	63.69111696
UIJ64949.1	hypothetical_protein_LW858_18470_[Bacillus_cereus]	CPTF_Fe	99657.7	62649.4638	62.86464949
UIJ64949.1	hypothetical_protein_LW858_18470_[Bacillus_cereus]	CPTF_Mn	91121.46667	43883.76027	48.15962898
UIJ64949.1	hypothetical_protein_LW858_18470_[Bacillus_cereus]	CPTF_Ni	255157.5333	32897.37437	12.89296614
UIJ64949.1	hypothetical_protein_LW858_18470_[Bacillus_cereus]	CPTF_U	39282.33333	68038.99717	173.2050808
UIJ64949.1	hypothetical_protein_LW858_18470_[Bacillus_cereus]	CPTF_metals_mix	114935.2667	39104.44525	34.02301694
UIJ64949.1	hypothetical_protein_LW858_18470_[Bacillus_cereus]	CPTF_zcontrol	152166.3333	42485.53218	27.92045471
UIJ64950.1	aminotransferase_A_[Bacillus_cereus]	CPTF_Al	74204	128525.0981	173.2050808
UIJ64950.1	aminotransferase_A_[Bacillus_cereus]	CPTF_Cd	78141	75264.77332	96.31918368
UIJ64950.1	aminotransferase_A_[Bacillus_cereus]	CPTF_Co	97566.66667	86754.76399	88.91844618
UIJ64950.1	aminotransferase_A_[Bacillus_cereus]	CPTF_Cu	157596.6667	146665.5802	93.06388475
UIJ64950.1	aminotransferase_A_[Bacillus_cereus]	CPTF_Fe	0	0	0
UIJ64950.1	aminotransferase_A_[Bacillus_cereus]	CPTF_Mn	166152.6667	287784.8605	173.2050808
UIJ64950.1	aminotransferase_A_[Bacillus_cereus]	CPTF_Ni	423172	374445.6214	88.48544361
UIJ64950.1	aminotransferase_A_[Bacillus_cereus]	CPTF_U	48931.33333	84751.55542	173.2050808
UIJ64950.1	aminotransferase_A_[Bacillus_cereus]	CPTF_metals_mix	191112.8333	149181.9274	78.05960739
UIJ64950.1	aminotransferase_A_[Bacillus_cereus]	CPTF_zcontrol	0	0	0
UIJ64954.1	extracellular_solute-binding_protein_[Bacillus_cereus]	CPTF_Al	92309.26667	31341.73633	33.9529686
UIJ64954.1	extracellular_solute-binding_protein_[Bacillus_cereus]	CPTF_Cd	90990.9	18145.95327	19.94260224
UIJ64954.1	extracellular_solute-binding_protein_[Bacillus_cereus]	CPTF_Co	93111.16667	47261.22767	50.75785146
UIJ64954.1	extracellular_solute-binding_protein_[Bacillus_cereus]	CPTF_Cu	131865.1667	29082.19795	22.05449603
UIJ64954.1	extracellular_solute-binding_protein_[Bacillus_cereus]	CPTF_Fe	128690.3333	17036.79378	13.23859636
UIJ64954.1	extracellular_solute-binding_protein_[Bacillus_cereus]	CPTF_Mn	130049.7	54141.69493	41.63154158
UIJ64954.1	extracellular_solute-binding_protein_[Bacillus_cereus]	CPTF_Ni	112232.8333	31292.23687	27.88153515
UIJ64954.1	extracellular_solute-binding_protein_[Bacillus_cereus]	CPTF_U	148600	35234.11916	23.71071276
UIJ64954.1	extracellular_solute-binding_protein_[Bacillus_cereus]	CPTF_metals_mix	16993.5	16016.82523	94.25265681
UIJ64954.1	extracellular_solute-binding_protein_[Bacillus_cereus]	CPTF_zcontrol	41975	11424.8645	27.21825967
UIJ64957.1	sn-glycerol-3-phosphate_ABC_transporter_ATP-binding_protein_UgpC_[Bacillus_cereus]	CPTF_Al	0	0	0
UIJ64957.1	sn-glycerol-3-phosphate_ABC_transporter_ATP-binding_protein_UgpC_[Bacillus_cereus]	CPTF_Cd	0	0	0
UIJ64957.1	sn-glycerol-3-phosphate_ABC_transporter_ATP-binding_protein_UgpC_[Bacillus_cereus]	CPTF_Co	0	0	0
UIJ64957.1	sn-glycerol-3-phosphate_ABC_transporter_ATP-binding_protein_UgpC_[Bacillus_cereus]	CPTF_Cu	0	0	0
UIJ64957.1	sn-glycerol-3-phosphate_ABC_transporter_ATP-binding_protein_UgpC_[Bacillus_cereus]	CPTF_Fe	0	0	0
UIJ64957.1	sn-glycerol-3-phosphate_ABC_transporter_ATP-binding_protein_UgpC_[Bacillus_cereus]	CPTF_Mn	0	0	0
UIJ64957.1	sn-glycerol-3-phosphate_ABC_transporter_ATP-binding_protein_UgpC_[Bacillus_cereus]	CPTF_Ni	0	0	0
UIJ64957.1	sn-glycerol-3-phosphate_ABC_transporter_ATP-binding_protein_UgpC_[Bacillus_cereus]	CPTF_U	0	0	0
UIJ64957.1	sn-glycerol-3-phosphate_ABC_transporter_ATP-binding_protein_UgpC_[Bacillus_cereus]	CPTF_metals_mix	593946	514439.3175	86.61381969
UIJ64957.1	sn-glycerol-3-phosphate_ABC_transporter_ATP-binding_protein_UgpC_[Bacillus_cereus]	CPTF_zcontrol	0	0	0
UIJ64964.1	acetyl-CoA_C-acetyltransferase_[Bacillus_cereus]	CPTF_Al	21007541.1	256463.3022	1.220815425
UIJ64964.1	acetyl-CoA_C-acetyltransferase_[Bacillus_cereus]	CPTF_Cd	20184219.4	1163617.14	5.764984602
UIJ64964.1	acetyl-CoA_C-acetyltransferase_[Bacillus_cereus]	CPTF_Co	18523445.7	886852.671	4.787730563
UIJ64964.1	acetyl-CoA_C-acetyltransferase_[Bacillus_cereus]	CPTF_Cu	21276864.5	360873.3539	1.696083339
UIJ64964.1	acetyl-CoA_C-acetyltransferase_[Bacillus_cereus]	CPTF_Fe	21138920.37	1015714.925	4.804951753
UIJ64964.1	acetyl-CoA_C-acetyltransferase_[Bacillus_cereus]	CPTF_Mn	21203532.37	1903067.251	8.975236851
UIJ64964.1	acetyl-CoA_C-acetyltransferase_[Bacillus_cereus]	CPTF_Ni	20907760.57	2205650.619	10.54943504
UIJ64964.1	acetyl-CoA_C-acetyltransferase_[Bacillus_cereus]	CPTF_U	19208323.67	1713712.208	8.921716635
UIJ64964.1	acetyl-CoA_C-acetyltransferase_[Bacillus_cereus]	CPTF_metals_mix	17194916.2	1832532.434	10.65740834
UIJ64964.1	acetyl-CoA_C-acetyltransferase_[Bacillus_cereus]	CPTF_zcontrol	20190460.13	696457.9459	3.449440683
UIJ64965.1	NUDIX_hydrolase_[Bacillus_cereus]	CPTF_Al	86620.33333	150030.8183	173.2050808

UIJ64965.1	NUDIX_hydrolase_[Bacillus_cereus]	CPTF_Cd	107185.0333	121280.5454	113.1506346
UIJ64965.1	NUDIX_hydrolase_[Bacillus_cereus]	CPTF_Co	112969.6667	195669.2024	173.2050808
UIJ64965.1	NUDIX_hydrolase_[Bacillus_cereus]	CPTF_Cu	91303.33333	158142.0122	173.2050808
UIJ64965.1	NUDIX_hydrolase_[Bacillus_cereus]	CPTF_Fe	0	0	0
UIJ64965.1	NUDIX_hydrolase_[Bacillus_cereus]	CPTF_Mn	64800	62558.18112	96.54040296
UIJ64965.1	NUDIX_hydrolase_[Bacillus_cereus]	CPTF_Ni	0	0	0
UIJ64965.1	NUDIX_hydrolase_[Bacillus_cereus]	CPTF_U	117174.9	101481.907	86.60720594
UIJ64965.1	NUDIX_hydrolase_[Bacillus_cereus]	CPTF_metals_mix	18381.53333	31837.74965	173.2050808
UIJ64965.1	NUDIX_hydrolase_[Bacillus_cereus]	CPTF_zcontrol	171679.3333	169335.7898	98.63492976
UIJ64966.1	DUF3928_family_protein_[Bacillus_cereus]	CPTF_Al	2073810.333	85996.66851	4.146795255
UIJ64966.1	DUF3928_family_protein_[Bacillus_cereus]	CPTF_Cd	2518728.667	535968.8085	21.27933888
UIJ64966.1	DUF3928_family_protein_[Bacillus_cereus]	CPTF_Co	1873408	132508.8907	7.073146413
UIJ64966.1	DUF3928_family_protein_[Bacillus_cereus]	CPTF_Cu	1774397	17786.37605	1.002389885
UIJ64966.1	DUF3928_family_protein_[Bacillus_cereus]	CPTF_Fe	2330711	619462.7832	26.57827518
UIJ64966.1	DUF3928_family_protein_[Bacillus_cereus]	CPTF_Mn	2409849	413371.6476	17.15342528
UIJ64966.1	DUF3928_family_protein_[Bacillus_cereus]	CPTF_Ni	2103405.667	99471.90856	4.72908817
UIJ64966.1	DUF3928_family_protein_[Bacillus_cereus]	CPTF_U	1320634.667	489475.221	37.06363564
UIJ64966.1	DUF3928_family_protein_[Bacillus_cereus]	CPTF_metals_mix	3126109.533	176167.1643	5.635348422
UIJ64966.1	DUF3928_family_protein_[Bacillus_cereus]	CPTF_zcontrol	2058928	320321.6	15.55768827
UIJ64970.1	3-hydroxybutyrate_dehydrogenase_[Bacillus_cereus]	CPTF_Al	2394020.867	1259116.508	52.59421609
UIJ64970.1	3-hydroxybutyrate_dehydrogenase_[Bacillus_cereus]	CPTF_Cd	2449560.033	1276807.334	52.12394537
UIJ64970.1	3-hydroxybutyrate_dehydrogenase_[Bacillus_cereus]	CPTF_Co	2498885.733	1108612.531	44.36427468
UIJ64970.1	3-hydroxybutyrate_dehydrogenase_[Bacillus_cereus]	CPTF_Cu	2092487.2	1119276.269	53.49023252
UIJ64970.1	3-hydroxybutyrate_dehydrogenase_[Bacillus_cereus]	CPTF_Fe	1437792.367	736835.1692	51.24767569
UIJ64970.1	3-hydroxybutyrate_dehydrogenase_[Bacillus_cereus]	CPTF_Mn	1588182.967	909983.3629	57.29713654
UIJ64970.1	3-hydroxybutyrate_dehydrogenase_[Bacillus_cereus]	CPTF_Ni	1583687.967	957596.5772	60.46624066
UIJ64970.1	3-hydroxybutyrate_dehydrogenase_[Bacillus_cereus]	CPTF_U	917593.0667	63829.91414	6.956233265
UIJ64970.1	3-hydroxybutyrate_dehydrogenase_[Bacillus_cereus]	CPTF_metals_mix	640404.3	21098.24047	3.294518864
UIJ64970.1	3-hydroxybutyrate_dehydrogenase_[Bacillus_cereus]	CPTF_zcontrol	891895.7333	8447.983371	0.947194056
UIJ64978.1	cupin_domain-containing_protein_[Bacillus_cereus]	CPTF_Al	159613.1333	142520.6359	89.29129635
UIJ64978.1	cupin_domain-containing_protein_[Bacillus_cereus]	CPTF_Cd	167307.8	152290.6354	91.02422924
UIJ64978.1	cupin_domain-containing_protein_[Bacillus_cereus]	CPTF_Co	141215.3333	122966.4877	87.0772917
UIJ64978.1	cupin_domain-containing_protein_[Bacillus_cereus]	CPTF_Cu	102483	93776.56002	91.5045032
UIJ64978.1	cupin_domain-containing_protein_[Bacillus_cereus]	CPTF_Fe	181352.3333	177190.0812	97.70488083
UIJ64978.1	cupin_domain-containing_protein_[Bacillus_cereus]	CPTF_Mn	221817.8	105861.9655	47.72473874
UIJ64978.1	cupin_domain-containing_protein_[Bacillus_cereus]	CPTF_Ni	13500.23333	23383.09005	173.2050808
UIJ64978.1	cupin_domain-containing_protein_[Bacillus_cereus]	CPTF_U	79417.66667	137555.4337	173.2050808
UIJ64978.1	cupin_domain-containing_protein_[Bacillus_cereus]	CPTF_metals_mix	288182.4667	37638.05307	13.06049376
UIJ64978.1	cupin_domain-containing_protein_[Bacillus_cereus]	CPTF_zcontrol	262419	79860.24033	30.43233925
UIJ64980.1	DUF3909_family_protein_[Bacillus_cereus]	CPTF_Al	603884	43638.45395	7.226297426
UIJ64980.1	DUF3909_family_protein_[Bacillus_cereus]	CPTF_Cd	614389.6667	31724.65981	5.163605694
UIJ64980.1	DUF3909_family_protein_[Bacillus_cereus]	CPTF_Co	567334.3333	57408.74909	10.11903312
UIJ64980.1	DUF3909_family_protein_[Bacillus_cereus]	CPTF_Cu	639669.6667	42452.23303	6.636586858
UIJ64980.1	DUF3909_family_protein_[Bacillus_cereus]	CPTF_Fe	615563	23585.77921	3.831578443
UIJ64980.1	DUF3909_family_protein_[Bacillus_cereus]	CPTF_Mn	566898	26042.80632	4.593913953
UIJ64980.1	DUF3909_family_protein_[Bacillus_cereus]	CPTF_Ni	611190.6667	73913.24764	12.09332074
UIJ64980.1	DUF3909_family_protein_[Bacillus_cereus]	CPTF_U	649963.3333	113152.277	17.40902467
UIJ64980.1	DUF3909_family_protein_[Bacillus_cereus]	CPTF_metals_mix	512923.6667	21468.52061	4.185519602
UIJ64980.1	DUF3909_family_protein_[Bacillus_cereus]	CPTF_zcontrol	675747.3333	60394.03229	8.937368941
UIJ64981.1	NAD(P)-dependent_oxidoreductase_[Bacillus_cereus]	CPTF_Al	713580.0333	311171.4299	43.60708195
UIJ64981.1	NAD(P)-dependent_oxidoreductase_[Bacillus_cereus]	CPTF_Cd	911088.6667	385484.8994	42.31036051
UIJ64981.1	NAD(P)-dependent_oxidoreductase_[Bacillus_cereus]	CPTF_Co	968731.5	199123.5183	20.5550783
UIJ64981.1	NAD(P)-dependent_oxidoreductase_[Bacillus_cereus]	CPTF_Cu	823441.7667	309728.9052	37.61394159
UIJ64981.1	NAD(P)-dependent_oxidoreductase_[Bacillus_cereus]	CPTF_Fe	693141.2667	335096.3567	48.34459767
UIJ64981.1	NAD(P)-dependent_oxidoreductase_[Bacillus_cereus]	CPTF_Mn	429588.8	22000.95955	5.121399707
UIJ64981.1	NAD(P)-dependent_oxidoreductase_[Bacillus_cereus]	CPTF_Ni	1213368.133	205555.1379	16.94087163
UIJ64981.1	NAD(P)-dependent_oxidoreductase_[Bacillus_cereus]	CPTF_U	503078.2667	33542.90933	6.667532976
UIJ64981.1	NAD(P)-dependent_oxidoreductase_[Bacillus_cereus]	CPTF_metals_mix	1096676.59	238230.3746	21.7229379
UIJ64981.1	NAD(P)-dependent_oxidoreductase_[Bacillus_cereus]	CPTF_zcontrol	668781.8333	468025.2518	69.98175316
UIJ64984.1	nitroreductase_family_protein_[Bacillus_cereus]	CPTF_Al	0	0	0
UIJ64984.1	nitroreductase_family_protein_[Bacillus_cereus]	CPTF_Cd	0	0	0

UIJ64984.1	nitroreductase_family_protein_[Bacillus_cereus]	CPTF_Co	0	0	0
UIJ64984.1	nitroreductase_family_protein_[Bacillus_cereus]	CPTF_Cu	18963.43333	32845.63002	173.2050808
UIJ64984.1	nitroreductase_family_protein_[Bacillus_cereus]	CPTF_Fe	0	0	0
UIJ64984.1	nitroreductase_family_protein_[Bacillus_cereus]	CPTF_Mn	0	0	0
UIJ64984.1	nitroreductase_family_protein_[Bacillus_cereus]	CPTF_Ni	20578.33333	35642.71887	173.2050808
UIJ64984.1	nitroreductase_family_protein_[Bacillus_cereus]	CPTF_U	0	0	0
UIJ64984.1	nitroreductase_family_protein_[Bacillus_cereus]	CPTF_metals_mix	263063.3	20664.13769	7.85519595
UIJ64984.1	nitroreductase_family_protein_[Bacillus_cereus]	CPTF_zcontrol	0	0	0
UIJ64987.1	phosphoenolpyruvate--protein_phosphotransferase_[Bacillus_cereus]	CPTF_Al	6323533.593	529172.6174	8.368305625
UIJ64987.1	phosphoenolpyruvate--protein_phosphotransferase_[Bacillus_cereus]	CPTF_Cd	7323222.6	298619.7795	4.07770999
UIJ64987.1	phosphoenolpyruvate--protein_phosphotransferase_[Bacillus_cereus]	CPTF_Co	7436929.5	671402.3308	9.027950726
UIJ64987.1	phosphoenolpyruvate--protein_phosphotransferase_[Bacillus_cereus]	CPTF_Cu	6967903.167	529813.4075	7.603627588
UIJ64987.1	phosphoenolpyruvate--protein_phosphotransferase_[Bacillus_cereus]	CPTF_Fe	6403866.867	869472.6188	13.57730629
UIJ64987.1	phosphoenolpyruvate--protein_phosphotransferase_[Bacillus_cereus]	CPTF_Mn	6996408.667	1767093.332	25.25714858
UIJ64987.1	phosphoenolpyruvate--protein_phosphotransferase_[Bacillus_cereus]	CPTF_Ni	5833394.4	1056698.947	18.11464946
UIJ64987.1	phosphoenolpyruvate--protein_phosphotransferase_[Bacillus_cereus]	CPTF_U	6176632.533	248946.0656	4.030449671
UIJ64987.1	phosphoenolpyruvate--protein_phosphotransferase_[Bacillus_cereus]	CPTF_metals_mix	12326848.6	619989.6294	5.029587444
UIJ64987.1	phosphoenolpyruvate--protein_phosphotransferase_[Bacillus_cereus]	CPTF_zcontrol	6534101.867	1408498.87	21.55612047
UIJ64988.1	phosphocarrier_protein_HPr_[Bacillus_cereus]	CPTF_Al	1407996	248889.9017	17.67688983
UIJ64988.1	phosphocarrier_protein_HPr_[Bacillus_cereus]	CPTF_Cd	1813084.667	153566.5984	8.469907734
UIJ64988.1	phosphocarrier_protein_HPr_[Bacillus_cereus]	CPTF_Co	1827132.667	248575.1577	13.60465839
UIJ64988.1	phosphocarrier_protein_HPr_[Bacillus_cereus]	CPTF_Cu	2082582.667	266464.966	12.79492864
UIJ64988.1	phosphocarrier_protein_HPr_[Bacillus_cereus]	CPTF_Fe	1742016.667	344136.3291	19.75505377
UIJ64988.1	phosphocarrier_protein_HPr_[Bacillus_cereus]	CPTF_Mn	1536683	946822.5409	61.61469483
UIJ64988.1	phosphocarrier_protein_HPr_[Bacillus_cereus]	CPTF_Ni	1176853.333	455439.9933	38.69980909
UIJ64988.1	phosphocarrier_protein_HPr_[Bacillus_cereus]	CPTF_U	634546.6667	70979.86536	11.1859173
UIJ64988.1	phosphocarrier_protein_HPr_[Bacillus_cereus]	CPTF_metals_mix	3122279.2	182479.8027	5.844442184
UIJ64988.1	phosphocarrier_protein_HPr_[Bacillus_cereus]	CPTF_zcontrol	1422975	66575.44225	4.67860941
UIJ64989.1	PTS_glucose_transporter_subunit_IIABC_[Bacillus_cereus]	CPTF_Al	9325593.767	910385.6367	9.762227044
UIJ64989.1	PTS_glucose_transporter_subunit_IIABC_[Bacillus_cereus]	CPTF_Cd	10904922.53	803150.1927	7.365024283
UIJ64989.1	PTS_glucose_transporter_subunit_IIABC_[Bacillus_cereus]	CPTF_Co	9877523.133	334083.1845	3.382256665
UIJ64989.1	PTS_glucose_transporter_subunit_IIABC_[Bacillus_cereus]	CPTF_Cu	8528470.933	537930.0278	6.307461583
UIJ64989.1	PTS_glucose_transporter_subunit_IIABC_[Bacillus_cereus]	CPTF_Fe	9782024.367	1190096.244	12.16615498
UIJ64989.1	PTS_glucose_transporter_subunit_IIABC_[Bacillus_cereus]	CPTF_Mn	9486456.433	1804275.003	19.01948337
UIJ64989.1	PTS_glucose_transporter_subunit_IIABC_[Bacillus_cereus]	CPTF_Ni	6378524.333	337354.5455	5.288912104
UIJ64989.1	PTS_glucose_transporter_subunit_IIABC_[Bacillus_cereus]	CPTF_U	7753150.433	775925.5071	10.00787375
UIJ64989.1	PTS_glucose_transporter_subunit_IIABC_[Bacillus_cereus]	CPTF_metals_mix	11442120.1	403258.4151	3.524333005
UIJ64989.1	PTS_glucose_transporter_subunit_IIABC_[Bacillus_cereus]	CPTF_zcontrol	9544188.4	1625079.31	17.02689891
UIJ64992.1	glucosamine-6-phosphate_deaminase_[Bacillus_cereus]	CPTF_Al	187909.4333	88149.69918	46.91073653
UIJ64992.1	glucosamine-6-phosphate_deaminase_[Bacillus_cereus]	CPTF_Cd	171894.7667	40478.81748	23.54860376
UIJ64992.1	glucosamine-6-phosphate_deaminase_[Bacillus_cereus]	CPTF_Co	221300.6667	65167.96365	29.44770327
UIJ64992.1	glucosamine-6-phosphate_deaminase_[Bacillus_cereus]	CPTF_Cu	225719.2333	29056.15363	12.87269729
UIJ64992.1	glucosamine-6-phosphate_deaminase_[Bacillus_cereus]	CPTF_Fe	160812.7	27514.61126	17.10972533
UIJ64992.1	glucosamine-6-phosphate_deaminase_[Bacillus_cereus]	CPTF_Mn	132909.5667	66228.46847	49.82972267
UIJ64992.1	glucosamine-6-phosphate_deaminase_[Bacillus_cereus]	CPTF_Ni	92669.53333	99502.26464	107.373223
UIJ64992.1	glucosamine-6-phosphate_deaminase_[Bacillus_cereus]	CPTF_U	183489.4333	62058.99817	33.82156511
UIJ64992.1	glucosamine-6-phosphate_deaminase_[Bacillus_cereus]	CPTF_metals_mix	260514.4	81141.81122	31.14676625
UIJ64992.1	glucosamine-6-phosphate_deaminase_[Bacillus_cereus]	CPTF_zcontrol	135642.0667	48170.4252	35.51289536
UIJ64998.1	GNAT_family_N-acetyltransferase_RibT_[Bacillus_cereus]	CPTF_Al	60779.86667	54225.01345	89.21542021
UIJ64998.1	GNAT_family_N-acetyltransferase_RibT_[Bacillus_cereus]	CPTF_Cd	31433.66667	54444.70773	173.2050808
UIJ64998.1	GNAT_family_N-acetyltransferase_RibT_[Bacillus_cereus]	CPTF_Co	61473.16667	7262.835513	11.81464354
UIJ64998.1	GNAT_family_N-acetyltransferase_RibT_[Bacillus_cereus]	CPTF_Cu	44895.66667	77761.57571	173.2050808
UIJ64998.1	GNAT_family_N-acetyltransferase_RibT_[Bacillus_cereus]	CPTF_Fe	26459.6	45829.37155	173.2050808
UIJ64998.1	GNAT_family_N-acetyltransferase_RibT_[Bacillus_cereus]	CPTF_Mn	35967.9	34487.30169	95.88355642
UIJ64998.1	GNAT_family_N-acetyltransferase_RibT_[Bacillus_cereus]	CPTF_Ni	29879.93333	51753.56266	173.2050808
UIJ64998.1	GNAT_family_N-acetyltransferase_RibT_[Bacillus_cereus]	CPTF_U	0	0	0
UIJ64998.1	GNAT_family_N-acetyltransferase_RibT_[Bacillus_cereus]	CPTF_metals_mix	29221.26667	27270.79675	93.32516987
UIJ64998.1	GNAT_family_N-acetyltransferase_RibT_[Bacillus_cereus]	CPTF_zcontrol	0	0	0
UIJ64999.1	peptidylprolyl_isomerase_[Bacillus_cereus]	CPTF_Al	1914831.067	341897.0683	17.85520792
UIJ64999.1	peptidylprolyl_isomerase_[Bacillus_cereus]	CPTF_Cd	2472164.967	154982.6521	6.269106399
UIJ64999.1	peptidylprolyl_isomerase_[Bacillus_cereus]	CPTF_Co	2202249	174250.5343	7.912390211

UIJ64999.1	peptidylprolyl_isomerase_[Bacillus_cereus]	CPTF_Cu	2132898.333	526237.1609	24.67239778
UIJ64999.1	peptidylprolyl_isomerase_[Bacillus_cereus]	CPTF_Fe	2202513.233	312920.4939	14.20742855
UIJ64999.1	peptidylprolyl_isomerase_[Bacillus_cereus]	CPTF_Mn	1933267	748945.8845	38.73990941
UIJ64999.1	peptidylprolyl_isomerase_[Bacillus_cereus]	CPTF_Ni	1007322.333	315662.6564	31.3368071
UIJ64999.1	peptidylprolyl_isomerase_[Bacillus_cereus]	CPTF_U	904537.0667	347189.8696	38.38315559
UIJ64999.1	peptidylprolyl_isomerase_[Bacillus_cereus]	CPTF_metals_mix	2591227.267	88318.5747	3.40836853
UIJ64999.1	peptidylprolyl_isomerase_[Bacillus_cereus]	CPTF_zcontrol	1604709.567	388381.0458	24.20257559
UIJ65000.1	DUF1002_domain-containing_protein_[Bacillus_cereus]	CPTF_Al	0	0	0
UIJ65000.1	DUF1002_domain-containing_protein_[Bacillus_cereus]	CPTF_Cd	0	0	0
UIJ65000.1	DUF1002_domain-containing_protein_[Bacillus_cereus]	CPTF_Co	0	0	0
UIJ65000.1	DUF1002_domain-containing_protein_[Bacillus_cereus]	CPTF_Cu	0	0	0
UIJ65000.1	DUF1002_domain-containing_protein_[Bacillus_cereus]	CPTF_Fe	0	0	0
UIJ65000.1	DUF1002_domain-containing_protein_[Bacillus_cereus]	CPTF_Mn	0	0	0
UIJ65000.1	DUF1002_domain-containing_protein_[Bacillus_cereus]	CPTF_Ni	0	0	0
UIJ65000.1	DUF1002_domain-containing_protein_[Bacillus_cereus]	CPTF_U	0	0	0
UIJ65000.1	DUF1002_domain-containing_protein_[Bacillus_cereus]	CPTF_metals_mix	152435.6667	37456.63482	24.57209369
UIJ65000.1	DUF1002_domain-containing_protein_[Bacillus_cereus]	CPTF_zcontrol	0	0	0
UIJ65009.1	RNA_polymerase_sporulation_sigma_factor_SigF_[Bacillus_cereus]	CPTF_Al	81606.53333	44664.74912	54.73183003
UIJ65009.1	RNA_polymerase_sporulation_sigma_factor_SigF_[Bacillus_cereus]	CPTF_Cd	142774.8333	95227.86708	66.69793608
UIJ65009.1	RNA_polymerase_sporulation_sigma_factor_SigF_[Bacillus_cereus]	CPTF_Co	99210.3	112559.4942	113.4554519
UIJ65009.1	RNA_polymerase_sporulation_sigma_factor_SigF_[Bacillus_cereus]	CPTF_Cu	47395.76667	32626.19878	68.83779096
UIJ65009.1	RNA_polymerase_sporulation_sigma_factor_SigF_[Bacillus_cereus]	CPTF_Fe	120899.2333	95006.82118	78.58347697
UIJ65009.1	RNA_polymerase_sporulation_sigma_factor_SigF_[Bacillus_cereus]	CPTF_Mn	71344.33333	123572.0102	173.2050808
UIJ65009.1	RNA_polymerase_sporulation_sigma_factor_SigF_[Bacillus_cereus]	CPTF_Ni	48173.46667	32446.00203	67.35243335
UIJ65009.1	RNA_polymerase_sporulation_sigma_factor_SigF_[Bacillus_cereus]	CPTF_U	89466.33333	96504.70018	107.8670563
UIJ65009.1	RNA_polymerase_sporulation_sigma_factor_SigF_[Bacillus_cereus]	CPTF_metals_mix	39659.26667	55724.26771	140.5075595
UIJ65009.1	RNA_polymerase_sporulation_sigma_factor_SigF_[Bacillus_cereus]	CPTF_zcontrol	14486.4	25091.18082	173.2050808
UIJ65018.1	MarR_family_transcriptional_regulator_[Bacillus_cereus]	CPTF_Al	0	0	0
UIJ65018.1	MarR_family_transcriptional_regulator_[Bacillus_cereus]	CPTF_Cd	0	0	0
UIJ65018.1	MarR_family_transcriptional_regulator_[Bacillus_cereus]	CPTF_Co	0	0	0
UIJ65018.1	MarR_family_transcriptional_regulator_[Bacillus_cereus]	CPTF_Cu	0	0	0
UIJ65018.1	MarR_family_transcriptional_regulator_[Bacillus_cereus]	CPTF_Fe	0	0	0
UIJ65018.1	MarR_family_transcriptional_regulator_[Bacillus_cereus]	CPTF_Mn	0	0	0
UIJ65018.1	MarR_family_transcriptional_regulator_[Bacillus_cereus]	CPTF_Ni	0	0	0
UIJ65018.1	MarR_family_transcriptional_regulator_[Bacillus_cereus]	CPTF_U	0	0	0
UIJ65018.1	MarR_family_transcriptional_regulator_[Bacillus_cereus]	CPTF_metals_mix	33480.66667	57990.21574	173.2050808
UIJ65018.1	MarR_family_transcriptional_regulator_[Bacillus_cereus]	CPTF_zcontrol	0	0	0
UIJ65022.1	pyrimidine-nucleoside_phosphorylase_[Bacillus_cereus]	CPTF_Al	101911.0333	81535.73059	80.00677446
UIJ65022.1	pyrimidine-nucleoside_phosphorylase_[Bacillus_cereus]	CPTF_Cd	97065.6	63628.10603	65.55165376
UIJ65022.1	pyrimidine-nucleoside_phosphorylase_[Bacillus_cereus]	CPTF_Co	52619.1	51036.00445	96.99140511
UIJ65022.1	pyrimidine-nucleoside_phosphorylase_[Bacillus_cereus]	CPTF_Cu	83276.86667	40581.41954	48.73072339
UIJ65022.1	pyrimidine-nucleoside_phosphorylase_[Bacillus_cereus]	CPTF_Fe	71659.5	48637.15774	67.87258876
UIJ65022.1	pyrimidine-nucleoside_phosphorylase_[Bacillus_cereus]	CPTF_Mn	14820.23333	12874.97165	86.87428438
UIJ65022.1	pyrimidine-nucleoside_phosphorylase_[Bacillus_cereus]	CPTF_Ni	86742.9	86299.36843	99.48868257
UIJ65022.1	pyrimidine-nucleoside_phosphorylase_[Bacillus_cereus]	CPTF_U	42008.86667	36830.74752	87.6737471
UIJ65022.1	pyrimidine-nucleoside_phosphorylase_[Bacillus_cereus]	CPTF_metals_mix	449706.4333	173138.939	38.50043633
UIJ65022.1	pyrimidine-nucleoside_phosphorylase_[Bacillus_cereus]	CPTF_zcontrol	28075.2	27955.03496	99.57198867
UIJ65023.1	purine-nucleoside_phosphorylase_[Bacillus_cereus]	CPTF_Al	14354.6	24862.89652	173.2050808
UIJ65023.1	purine-nucleoside_phosphorylase_[Bacillus_cereus]	CPTF_Cd	48044	83214.649	173.2050808
UIJ65023.1	purine-nucleoside_phosphorylase_[Bacillus_cereus]	CPTF_Co	29770.5	51564.01857	173.2050808
UIJ65023.1	purine-nucleoside_phosphorylase_[Bacillus_cereus]	CPTF_Cu	44374.66667	76859.17724	173.2050808
UIJ65023.1	purine-nucleoside_phosphorylase_[Bacillus_cereus]	CPTF_Fe	0	0	0
UIJ65023.1	purine-nucleoside_phosphorylase_[Bacillus_cereus]	CPTF_Mn	46024.76667	42010.07086	91.27709688
UIJ65023.1	purine-nucleoside_phosphorylase_[Bacillus_cereus]	CPTF_Ni	0	0	0
UIJ65023.1	purine-nucleoside_phosphorylase_[Bacillus_cereus]	CPTF_U	0	0	0
UIJ65023.1	purine-nucleoside_phosphorylase_[Bacillus_cereus]	CPTF_metals_mix	347870.3333	27157.62104	7.806822955
UIJ65023.1	purine-nucleoside_phosphorylase_[Bacillus_cereus]	CPTF_zcontrol	10266.93333	17782.85017	173.2050808
UIJ65024.1	phosphopentomutase_[Bacillus_cereus]	CPTF_Al	1495075.233	202244.254	13.527363
UIJ65024.1	phosphopentomutase_[Bacillus_cereus]	CPTF_Cd	1688566.9	304443.7288	18.0297108
UIJ65024.1	phosphopentomutase_[Bacillus_cereus]	CPTF_Co	1471028.667	413663.2954	28.12068213
UIJ65024.1	phosphopentomutase_[Bacillus_cereus]	CPTF_Cu	1104910.867	302144.018	27.34555584

UIJ65024.1	phosphopentomutase_[Bacillus_cereus]	CPTF_Fe	1353938.133	133230.3559	9.840210021
UIJ65024.1	phosphopentomutase_[Bacillus_cereus]	CPTF_Mn	1429903	211423.2556	14.785846
UIJ65024.1	phosphopentomutase_[Bacillus_cereus]	CPTF_Ni	1409123.867	24333.30053	1.726839003
UIJ65024.1	phosphopentomutase_[Bacillus_cereus]	CPTF_U	1775822.933	59645.92203	3.358776425
UIJ65024.1	phosphopentomutase_[Bacillus_cereus]	CPTF_metals_mix	1740534.133	113049.537	6.495106005
UIJ65024.1	phosphopentomutase_[Bacillus_cereus]	CPTF_zcontrol	1535224.533	185664.0862	12.09361121
UIJ65025.1	FixH_family_protein_[Bacillus_cereus]	CPTF_Al	501451.3333	119342.087	23.79933587
UIJ65025.1	FixH_family_protein_[Bacillus_cereus]	CPTF_Cd	338214.6667	328762.9055	97.20539584
UIJ65025.1	FixH_family_protein_[Bacillus_cereus]	CPTF_Co	147718.3333	128305.3645	86.85811816
UIJ65025.1	FixH_family_protein_[Bacillus_cereus]	CPTF_Cu	327050.6667	63922.29051	19.54507268
UIJ65025.1	FixH_family_protein_[Bacillus_cereus]	CPTF_Fe	0	0	0
UIJ65025.1	FixH_family_protein_[Bacillus_cereus]	CPTF_Mn	142086.6667	135552.2464	95.40110245
UIJ65025.1	FixH_family_protein_[Bacillus_cereus]	CPTF_Ni	233322.6667	35638.74805	15.27444742
UIJ65025.1	FixH_family_protein_[Bacillus_cereus]	CPTF_U	0	0	0
UIJ65025.1	FixH_family_protein_[Bacillus_cereus]	CPTF_metals_mix	1039380.967	344004.5534	33.09706108
UIJ65025.1	FixH_family_protein_[Bacillus_cereus]	CPTF_zcontrol	0	0	0
UIJ65028.1	transcriptional_repressor_[Bacillus_cereus]	CPTF_Al	441360.3	48982.15813	11.09799819
UIJ65028.1	transcriptional_repressor_[Bacillus_cereus]	CPTF_Cd	529725.6667	121644.5714	22.96369215
UIJ65028.1	transcriptional_repressor_[Bacillus_cereus]	CPTF_Co	460740.6667	80961.17356	17.57196172
UIJ65028.1	transcriptional_repressor_[Bacillus_cereus]	CPTF_Cu	433360.7	82753.3972	19.09573185
UIJ65028.1	transcriptional_repressor_[Bacillus_cereus]	CPTF_Fe	456114	80234.54663	17.59089759
UIJ65028.1	transcriptional_repressor_[Bacillus_cereus]	CPTF_Mn	315035.3333	172519.142	54.76183898
UIJ65028.1	transcriptional_repressor_[Bacillus_cereus]	CPTF_Ni	436370.6667	50078.8631	11.47622123
UIJ65028.1	transcriptional_repressor_[Bacillus_cereus]	CPTF_U	328480.6667	99195.17698	30.19817817
UIJ65028.1	transcriptional_repressor_[Bacillus_cereus]	CPTF_metals_mix	1069407.333	309620.1394	28.95249824
UIJ65028.1	transcriptional_repressor_[Bacillus_cereus]	CPTF_zcontrol	390484.0667	63003.37838	16.13468609
UIJ65030.1	GNAT_family_N-acetyltransferase_[Bacillus_cereus]	CPTF_Al	202924.2667	137961.1558	67.98652427
UIJ65030.1	GNAT_family_N-acetyltransferase_[Bacillus_cereus]	CPTF_Cd	5972.9	10345.36627	173.2050808
UIJ65030.1	GNAT_family_N-acetyltransferase_[Bacillus_cereus]	CPTF_Co	443683.7333	55710.26842	12.55630176
UIJ65030.1	GNAT_family_N-acetyltransferase_[Bacillus_cereus]	CPTF_Cu	452565.5333	85823.19229	18.96370492
UIJ65030.1	GNAT_family_N-acetyltransferase_[Bacillus_cereus]	CPTF_Fe	127213.1	104702.5988	82.30488749
UIJ65030.1	GNAT_family_N-acetyltransferase_[Bacillus_cereus]	CPTF_Mn	99145.96667	47971.38797	48.38460865
UIJ65030.1	GNAT_family_N-acetyltransferase_[Bacillus_cereus]	CPTF_Ni	519077.6667	82316.75362	15.85827303
UIJ65030.1	GNAT_family_N-acetyltransferase_[Bacillus_cereus]	CPTF_U	86027.7	113109.9108	131.480803
UIJ65030.1	GNAT_family_N-acetyltransferase_[Bacillus_cereus]	CPTF_metals_mix	588680.4667	104378.9244	17.73099845
UIJ65030.1	GNAT_family_N-acetyltransferase_[Bacillus_cereus]	CPTF_zcontrol	100561.6667	174177.916	173.2050808
UIJ65031.1	NUDIX_hydrolase_[Bacillus_cereus]	CPTF_Al	1289518.5	391367.0028	30.34985561
UIJ65031.1	NUDIX_hydrolase_[Bacillus_cereus]	CPTF_Cd	1402269.667	484540.947	34.55404894
UIJ65031.1	NUDIX_hydrolase_[Bacillus_cereus]	CPTF_Co	1451931.1	218063.8147	15.01888173
UIJ65031.1	NUDIX_hydrolase_[Bacillus_cereus]	CPTF_Cu	1575502.333	11808.76714	0.749523938
UIJ65031.1	NUDIX_hydrolase_[Bacillus_cereus]	CPTF_Fe	1532855.6	86144.6774	5.619882094
UIJ65031.1	NUDIX_hydrolase_[Bacillus_cereus]	CPTF_Mn	1473247.667	415420.4523	28.19759785
UIJ65031.1	NUDIX_hydrolase_[Bacillus_cereus]	CPTF_Ni	1238918.267	210092.7546	16.95775744
UIJ65031.1	NUDIX_hydrolase_[Bacillus_cereus]	CPTF_U	1149311.667	404276.8667	35.17556451
UIJ65031.1	NUDIX_hydrolase_[Bacillus_cereus]	CPTF_metals_mix	1559763.333	24300.21941	1.557942727
UIJ65031.1	NUDIX_hydrolase_[Bacillus_cereus]	CPTF_zcontrol	1441934.9	341342.7977	23.6725526
UIJ65033.1	aldo/keto_reductase_[Bacillus_cereus]	CPTF_Al	0	0	0
UIJ65033.1	aldo/keto_reductase_[Bacillus_cereus]	CPTF_Cd	0	0	0
UIJ65033.1	aldo/keto_reductase_[Bacillus_cereus]	CPTF_Co	0	0	0
UIJ65033.1	aldo/keto_reductase_[Bacillus_cereus]	CPTF_Cu	30955.26667	53616.09463	173.2050808
UIJ65033.1	aldo/keto_reductase_[Bacillus_cereus]	CPTF_Fe	0	0	0
UIJ65033.1	aldo/keto_reductase_[Bacillus_cereus]	CPTF_Mn	92171	88479.8471	95.99532076
UIJ65033.1	aldo/keto_reductase_[Bacillus_cereus]	CPTF_Ni	0	0	0
UIJ65033.1	aldo/keto_reductase_[Bacillus_cereus]	CPTF_U	0	0	0
UIJ65033.1	aldo/keto_reductase_[Bacillus_cereus]	CPTF_metals_mix	195590	21104.72504	10.79028838
UIJ65033.1	aldo/keto_reductase_[Bacillus_cereus]	CPTF_zcontrol	0	0	0
UIJ65034.1	aldo/keto_reductase_[Bacillus_cereus]	CPTF_Al	166069	15651.97036	9.424980192
UIJ65034.1	aldo/keto_reductase_[Bacillus_cereus]	CPTF_Cd	129213.6667	16339.40851	12.64526341
UIJ65034.1	aldo/keto_reductase_[Bacillus_cereus]	CPTF_Co	141552.3333	14854.71162	10.49414819
UIJ65034.1	aldo/keto_reductase_[Bacillus_cereus]	CPTF_Cu	195926.3333	6487.230251	3.311055814
UIJ65034.1	aldo/keto_reductase_[Bacillus_cereus]	CPTF_Fe	92590.93333	28406.26821	30.67931944

UIJ65034.1	aldo/keto_reductase_[Bacillus_cereus]	CPTF_Mn	117217	12981.59039	11.07483589
UIJ65034.1	aldo/keto_reductase_[Bacillus_cereus]	CPTF_Ni	62044.93333	54908.98748	88.49874523
UIJ65034.1	aldo/keto_reductase_[Bacillus_cereus]	CPTF_U	193673.3333	140461.9775	72.52520266
UIJ65034.1	aldo/keto_reductase_[Bacillus_cereus]	CPTF_metals_mix	166201.6667	82946.13443	49.90692097
UIJ65034.1	aldo/keto_reductase_[Bacillus_cereus]	CPTF_zcontrol	100643.9667	43291.3641	43.01436592
UIJ65035.1	YqkE_family_protein_[Bacillus_cereus]	CPTF_Al	0	0	0
UIJ65035.1	YqkE_family_protein_[Bacillus_cereus]	CPTF_Cd	6551.4	11347.35766	173.2050808
UIJ65035.1	YqkE_family_protein_[Bacillus_cereus]	CPTF_Co	11169.56667	19346.25697	173.2050808
UIJ65035.1	YqkE_family_protein_[Bacillus_cereus]	CPTF_Cu	0	0	0
UIJ65035.1	YqkE_family_protein_[Bacillus_cereus]	CPTF_Fe	0	0	0
UIJ65035.1	YqkE_family_protein_[Bacillus_cereus]	CPTF_Mn	0	0	0
UIJ65035.1	YqkE_family_protein_[Bacillus_cereus]	CPTF_Ni	0	0	0
UIJ65035.1	YqkE_family_protein_[Bacillus_cereus]	CPTF_U	0	0	0
UIJ65035.1	YqkE_family_protein_[Bacillus_cereus]	CPTF_metals_mix	29337.83333	35175.10564	119.8967396
UIJ65035.1	YqkE_family_protein_[Bacillus_cereus]	CPTF_zcontrol	0	0	0
UIJ65036.1	5'-nucleotidase_C-terminal_domain-containing_protein_[Bacillus_cereus]	CPTF_Al	855897.2	186854.0216	21.83136265
UIJ65036.1	5'-nucleotidase_C-terminal_domain-containing_protein_[Bacillus_cereus]	CPTF_Cd	856333.8333	145644.2365	17.00788067
UIJ65036.1	5'-nucleotidase_C-terminal_domain-containing_protein_[Bacillus_cereus]	CPTF_Co	856825.7667	152505.6438	17.79890962
UIJ65036.1	5'-nucleotidase_C-terminal_domain-containing_protein_[Bacillus_cereus]	CPTF_Cu	160633.0667	259399.9183	161.4860027
UIJ65036.1	5'-nucleotidase_C-terminal_domain-containing_protein_[Bacillus_cereus]	CPTF_Fe	392821	503555.2005	128.1894808
UIJ65036.1	5'-nucleotidase_C-terminal_domain-containing_protein_[Bacillus_cereus]	CPTF_Mn	447020.0667	388576.2781	86.92591386
UIJ65036.1	5'-nucleotidase_C-terminal_domain-containing_protein_[Bacillus_cereus]	CPTF_Ni	609425.1	380931.4849	62.50669441
UIJ65036.1	5'-nucleotidase_C-terminal_domain-containing_protein_[Bacillus_cereus]	CPTF_U	634829.5333	330200.467	52.01403679
UIJ65036.1	5'-nucleotidase_C-terminal_domain-containing_protein_[Bacillus_cereus]	CPTF_metals_mix	142681.5667	162478.152	113.8746622
UIJ65036.1	5'-nucleotidase_C-terminal_domain-containing_protein_[Bacillus_cereus]	CPTF_zcontrol	1022879.933	163095.0242	15.944689
UIJ65044.1	CDGSH_iron-sulfur_domain-containing_protein_[Bacillus_cereus]	CPTF_Al	40524.26667	41704.36464	102.9120773
UIJ65044.1	CDGSH_iron-sulfur_domain-containing_protein_[Bacillus_cereus]	CPTF_Cd	0	0	0
UIJ65044.1	CDGSH_iron-sulfur_domain-containing_protein_[Bacillus_cereus]	CPTF_Co	56506.2	65716.43196	116.2995069
UIJ65044.1	CDGSH_iron-sulfur_domain-containing_protein_[Bacillus_cereus]	CPTF_Cu	0	0	0
UIJ65044.1	CDGSH_iron-sulfur_domain-containing_protein_[Bacillus_cereus]	CPTF_Fe	92821	80643.65803	86.88083304
UIJ65044.1	CDGSH_iron-sulfur_domain-containing_protein_[Bacillus_cereus]	CPTF_Mn	53322.3	46944.32476	88.03882196
UIJ65044.1	CDGSH_iron-sulfur_domain-containing_protein_[Bacillus_cereus]	CPTF_Ni	0	0	0
UIJ65044.1	CDGSH_iron-sulfur_domain-containing_protein_[Bacillus_cereus]	CPTF_U	0	0	0
UIJ65044.1	CDGSH_iron-sulfur_domain-containing_protein_[Bacillus_cereus]	CPTF_metals_mix	19066.93333	20686.83054	108.4958455
UIJ65044.1	CDGSH_iron-sulfur_domain-containing_protein_[Bacillus_cereus]	CPTF_zcontrol	38374.2	38267.00425	99.72065672
UIJ65045.1	bifunctional_diaminohydroxyphosphoribosylaminopyrimidine_deaminase/5-amino-6-(5-phosphoribosylamino)uracil_reductase_RibD_[Bacillus_cereus]	CPTF_Al	4564.866667	7906.580996	173.2050808
UIJ65045.1	bifunctional_diaminohydroxyphosphoribosylaminopyrimidine_deaminase/5-amino-6-(5-phosphoribosylamino)uracil_reductase_RibD_[Bacillus_cereus]	CPTF_Cd	3357.2	5814.840971	173.2050808
UIJ65045.1	bifunctional_diaminohydroxyphosphoribosylaminopyrimidine_deaminase/5-amino-6-(5-phosphoribosylamino)uracil_reductase_RibD_[Bacillus_cereus]	CPTF_Co	0	0	0
UIJ65045.1	bifunctional_diaminohydroxyphosphoribosylaminopyrimidine_deaminase/5-amino-6-(5-phosphoribosylamino)uracil_reductase_RibD_[Bacillus_cereus]	CPTF_Cu	10957.33333	18978.65805	173.2050808
UIJ65045.1	bifunctional_diaminohydroxyphosphoribosylaminopyrimidine_deaminase/5-amino-6-(5-phosphoribosylamino)uracil_reductase_RibD_[Bacillus_cereus]	CPTF_Fe	5989.733333	10374.52246	173.2050808
UIJ65045.1	bifunctional_diaminohydroxyphosphoribosylaminopyrimidine_deaminase/5-amino-6-(5-phosphoribosylamino)uracil_reductase_RibD_[Bacillus_cereus]	CPTF_Mn	5919.7	10253.22117	173.2050808
UIJ65045.1	bifunctional_diaminohydroxyphosphoribosylaminopyrimidine_deaminase/5-amino-6-(5-phosphoribosylamino)uracil_reductase_RibD_[Bacillus_cereus]	CPTF_Ni	5509.5	9542.733924	173.2050808
UIJ65045.1	bifunctional_diaminohydroxyphosphoribosylaminopyrimidine_deaminase/5-amino-6-(5-phosphoribosylamino)uracil_reductase_RibD_[Bacillus_cereus]	CPTF_U	0	0	0
UIJ65045.1	bifunctional_diaminohydroxyphosphoribosylaminopyrimidine_deaminase/5-amino-6-(5-phosphoribosylamino)uracil_reductase_RibD_[Bacillus_cereus]	CPTF_metals_mix	22429.46667	12966.24605	57.80898067
UIJ65045.1	bifunctional_diaminohydroxyphosphoribosylaminopyrimidine_deaminase/5-amino-6-(5-phosphoribosylamino)uracil_reductase_RibD_[Bacillus_cereus]	CPTF_zcontrol	0	0	0
UIJ65047.1	bifunctional_3,4-dihydroxy-2-butanone_4-phosphate_synthase/GTP_cyclohydrolase_II_[Bacillus_cereus]	CPTF_Al	376997.3333	236833.8472	62.82109349
UIJ65047.1	bifunctional_3,4-dihydroxy-2-butanone_4-phosphate_synthase/GTP_cyclohydrolase_II_[Bacillus_cereus]	CPTF_Cd	351552.3333	176563.353	50.22391725
UIJ65047.1	bifunctional_3,4-dihydroxy-2-butanone_4-phosphate_synthase/GTP_cyclohydrolase_II_[Bacillus_cereus]	CPTF_Co	318859.4333	30270.7054	9.493432603
UIJ65047.1	bifunctional_3,4-dihydroxy-2-butanone_4-phosphate_synthase/GTP_cyclohydrolase_II_[Bacillus_cereus]	CPTF_Cu	272149	10231.40171	3.759485323
UIJ65047.1	bifunctional_3,4-dihydroxy-2-butanone_4-phosphate_synthase/GTP_cyclohydrolase_II_[Bacillus_cereus]	CPTF_Fe	271603.5667	220359.708	81.13284765
UIJ65047.1	bifunctional_3,4-dihydroxy-2-butanone_4-phosphate_synthase/GTP_cyclohydrolase_II_[Bacillus_cereus]	CPTF_Mn	183861.8	143177.5069	77.87235133
UIJ65047.1	bifunctional_3,4-dihydroxy-2-butanone_4-phosphate_synthase/GTP_cyclohydrolase_II_[Bacillus_cereus]	CPTF_Ni	350694	414286.713	118.1333907
UIJ65047.1	bifunctional_3,4-dihydroxy-2-butanone_4-phosphate_synthase/GTP_cyclohydrolase_II_[Bacillus_cereus]	CPTF_U	534281.8333	226359.0844	42.3669813
UIJ65047.1	bifunctional_3,4-dihydroxy-2-butanone_4-phosphate_synthase/GTP_cyclohydrolase_II_[Bacillus_cereus]	CPTF_metals_mix	568401.2	142217.7649	25.02066585
UIJ65047.1	bifunctional_3,4-dihydroxy-2-butanone_4-phosphate_synthase/GTP_cyclohydrolase_II_[Bacillus_cereus]	CPTF_zcontrol	76103.8	68507.52328	90.01853163
UIJ65048.1	6,7-dimethyl-8-ribityllumazine_synthase_[Bacillus_cereus]	CPTF_Al	637020.6	17900.60005	2.810050421
UIJ65048.1	6,7-dimethyl-8-ribityllumazine_synthase_[Bacillus_cereus]	CPTF_Cd	726122.9	69121.63451	9.519274837
UIJ65048.1	6,7-dimethyl-8-ribityllumazine_synthase_[Bacillus_cereus]	CPTF_Co	541374.6667	115314.0884	21.30023724
UIJ65048.1	6,7-dimethyl-8-ribityllumazine_synthase_[Bacillus_cereus]	CPTF_Cu	576377.6667	80525.81038	13.97101502
UIJ65048.1	6,7-dimethyl-8-ribityllumazine_synthase_[Bacillus_cereus]	CPTF_Fe	479535.3333	90517.90684	18.87617044
UIJ65048.1	6,7-dimethyl-8-ribityllumazine_synthase_[Bacillus_cereus]	CPTF_Mn	514556.9333	134082.6209	26.05787858

UIJ65048.1	6,7-dimethyl-8-ribityllumazine_synthase_[Bacillus_cereus]	CPTF_Ni	455639.1667	215776.1044	47.35679462
UIJ65048.1	6,7-dimethyl-8-ribityllumazine_synthase_[Bacillus_cereus]	CPTF_U	248273.9667	123048.5024	49.56158073
UIJ65048.1	6,7-dimethyl-8-ribityllumazine_synthase_[Bacillus_cereus]	CPTF_metals_mix	1069919.9	162028.1184	15.14394847
UIJ65048.1	6,7-dimethyl-8-ribityllumazine_synthase_[Bacillus_cereus]	CPTF_zcontrol	484154.5	27950.88688	5.773133757
UIJ65056.1	hypothetical_protein_LW858_19100_[Bacillus_cereus]	CPTF_Al	910000	1576166.235	173.2050808
UIJ65056.1	hypothetical_protein_LW858_19100_[Bacillus_cereus]	CPTF_Cd	0	0	0
UIJ65056.1	hypothetical_protein_LW858_19100_[Bacillus_cereus]	CPTF_Co	900000	780448.589	86.71650988
UIJ65056.1	hypothetical_protein_LW858_19100_[Bacillus_cereus]	CPTF_Cu	0	0	0
UIJ65056.1	hypothetical_protein_LW858_19100_[Bacillus_cereus]	CPTF_Fe	0	0	0
UIJ65056.1	hypothetical_protein_LW858_19100_[Bacillus_cereus]	CPTF_Mn	590000	1021909.976	173.2050808
UIJ65056.1	hypothetical_protein_LW858_19100_[Bacillus_cereus]	CPTF_Ni	251418	435468.7499	173.2050808
UIJ65056.1	hypothetical_protein_LW858_19100_[Bacillus_cereus]	CPTF_U	533333.3333	923760.4307	173.2050808
UIJ65056.1	hypothetical_protein_LW858_19100_[Bacillus_cereus]	CPTF_metals_mix	640055.3333	554304.885	86.60265076
UIJ65056.1	hypothetical_protein_LW858_19100_[Bacillus_cereus]	CPTF_zcontrol	933333.3333	873059.7536	93.54211646
UIJ65058.1	bifunctional_2',3'-cyclic-nucleotide_2'-phosphodiesterase/3'-nucleotidase_[Bacillus_cereus]	CPTF_Al	1066831.167	370539.7426	34.73274443
UIJ65058.1	bifunctional_2',3'-cyclic-nucleotide_2'-phosphodiesterase/3'-nucleotidase_[Bacillus_cereus]	CPTF_Cd	849313.7667	442355.5589	52.08387951
UIJ65058.1	bifunctional_2',3'-cyclic-nucleotide_2'-phosphodiesterase/3'-nucleotidase_[Bacillus_cereus]	CPTF_Co	779506.8667	214222.4881	27.48179615
UIJ65058.1	bifunctional_2',3'-cyclic-nucleotide_2'-phosphodiesterase/3'-nucleotidase_[Bacillus_cereus]	CPTF_Cu	526670.9333	173339.5082	32.91229821
UIJ65058.1	bifunctional_2',3'-cyclic-nucleotide_2'-phosphodiesterase/3'-nucleotidase_[Bacillus_cereus]	CPTF_Fe	1254838.6	236516.1989	18.84833626
UIJ65058.1	bifunctional_2',3'-cyclic-nucleotide_2'-phosphodiesterase/3'-nucleotidase_[Bacillus_cereus]	CPTF_Mn	609596.4	322558.963	52.9135282
UIJ65058.1	bifunctional_2',3'-cyclic-nucleotide_2'-phosphodiesterase/3'-nucleotidase_[Bacillus_cereus]	CPTF_Ni	935073.3	602280.2633	64.40995196
UIJ65058.1	bifunctional_2',3'-cyclic-nucleotide_2'-phosphodiesterase/3'-nucleotidase_[Bacillus_cereus]	CPTF_U	1486053.5	213594.8723	14.37329627
UIJ65058.1	bifunctional_2',3'-cyclic-nucleotide_2'-phosphodiesterase/3'-nucleotidase_[Bacillus_cereus]	CPTF_metals_mix	342871.4	319301.2815	93.12566797
UIJ65058.1	bifunctional_2',3'-cyclic-nucleotide_2'-phosphodiesterase/3'-nucleotidase_[Bacillus_cereus]	CPTF_zcontrol	1088830.3	300740.523	27.62051377
UIJ65063.1	ornithine_carbamoyltransferase_[Bacillus_cereus]	CPTF_Al	1699119.133	467478.7875	27.51300826
UIJ65063.1	ornithine_carbamoyltransferase_[Bacillus_cereus]	CPTF_Cd	1456915.133	257379.6425	17.66606967
UIJ65063.1	ornithine_carbamoyltransferase_[Bacillus_cereus]	CPTF_Co	1841539.5	363577.2928	19.74311672
UIJ65063.1	ornithine_carbamoyltransferase_[Bacillus_cereus]	CPTF_Cu	1931936.867	527942.8347	27.32712667
UIJ65063.1	ornithine_carbamoyltransferase_[Bacillus_cereus]	CPTF_Fe	1330329.9	422019.5521	31.72292468
UIJ65063.1	ornithine_carbamoyltransferase_[Bacillus_cereus]	CPTF_Mn	1610683.433	255139.7057	15.84046253
UIJ65063.1	ornithine_carbamoyltransferase_[Bacillus_cereus]	CPTF_Ni	1430159.667	140211.0982	9.803877248
UIJ65063.1	ornithine_carbamoyltransferase_[Bacillus_cereus]	CPTF_U	1393870.333	908566.1501	65.18297494
UIJ65063.1	ornithine_carbamoyltransferase_[Bacillus_cereus]	CPTF_metals_mix	4000971.933	305616.3209	7.638551982
UIJ65063.1	ornithine_carbamoyltransferase_[Bacillus_cereus]	CPTF_zcontrol	1836622	725421.6866	39.49760411
UIJ65064.1	acetylornithine_transaminase_[Bacillus_cereus]	CPTF_Al	449474.8	85981.31857	19.12928568
UIJ65064.1	acetylornithine_transaminase_[Bacillus_cereus]	CPTF_Cd	606852.4667	143325.1006	23.61778331
UIJ65064.1	acetylornithine_transaminase_[Bacillus_cereus]	CPTF_Co	713817.6333	107608.2876	15.07503914
UIJ65064.1	acetylornithine_transaminase_[Bacillus_cereus]	CPTF_Cu	808384.5667	25789.07516	3.190198851
UIJ65064.1	acetylornithine_transaminase_[Bacillus_cereus]	CPTF_Fe	527301.4333	71357.36403	13.53255643
UIJ65064.1	acetylornithine_transaminase_[Bacillus_cereus]	CPTF_Mn	400628.6333	76673.56639	19.13831414
UIJ65064.1	acetylornithine_transaminase_[Bacillus_cereus]	CPTF_Ni	401317.4	188819.0341	47.04980002
UIJ65064.1	acetylornithine_transaminase_[Bacillus_cereus]	CPTF_U	495174.6667	99088.69635	20.01085738
UIJ65064.1	acetylornithine_transaminase_[Bacillus_cereus]	CPTF_metals_mix	1250662.2	96958.99995	7.752612972
UIJ65064.1	acetylornithine_transaminase_[Bacillus_cereus]	CPTF_zcontrol	400800.6667	45544.87629	11.36347319
UIJ65065.1	acetylglutamate_kinase_[Bacillus_cereus]	CPTF_Al	0	0	0
UIJ65065.1	acetylglutamate_kinase_[Bacillus_cereus]	CPTF_Cd	0	0	0
UIJ65065.1	acetylglutamate_kinase_[Bacillus_cereus]	CPTF_Co	4863.9	8424.521923	173.2050808
UIJ65065.1	acetylglutamate_kinase_[Bacillus_cereus]	CPTF_Cu	0	0	0
UIJ65065.1	acetylglutamate_kinase_[Bacillus_cereus]	CPTF_Fe	0	0	0
UIJ65065.1	acetylglutamate_kinase_[Bacillus_cereus]	CPTF_Mn	0	0	0
UIJ65065.1	acetylglutamate_kinase_[Bacillus_cereus]	CPTF_Ni	0	0	0
UIJ65065.1	acetylglutamate_kinase_[Bacillus_cereus]	CPTF_U	0	0	0
UIJ65065.1	acetylglutamate_kinase_[Bacillus_cereus]	CPTF_metals_mix	66757.3	69478.71077	104.0765741
UIJ65065.1	acetylglutamate_kinase_[Bacillus_cereus]	CPTF_zcontrol	0	0	0
UIJ65066.1	bifunctional_glutamate_N-acetyltransferase/amino-acid_acetyltransferase_ArgJ_[Bacillus_cereus]	CPTF_Al	0	0	0
UIJ65066.1	bifunctional_glutamate_N-acetyltransferase/amino-acid_acetyltransferase_ArgJ_[Bacillus_cereus]	CPTF_Cd	0	0	0
UIJ65066.1	bifunctional_glutamate_N-acetyltransferase/amino-acid_acetyltransferase_ArgJ_[Bacillus_cereus]	CPTF_Co	0	0	0
UIJ65066.1	bifunctional_glutamate_N-acetyltransferase/amino-acid_acetyltransferase_ArgJ_[Bacillus_cereus]	CPTF_Cu	0	0	0
UIJ65066.1	bifunctional_glutamate_N-acetyltransferase/amino-acid_acetyltransferase_ArgJ_[Bacillus_cereus]	CPTF_Fe	0	0	0
UIJ65066.1	bifunctional_glutamate_N-acetyltransferase/amino-acid_acetyltransferase_ArgJ_[Bacillus_cereus]	CPTF_Mn	0	0	0
UIJ65066.1	bifunctional_glutamate_N-acetyltransferase/amino-acid_acetyltransferase_ArgJ_[Bacillus_cereus]	CPTF_Ni	0	0	0

UIJ65066.1	bifunctional_glutamate_N-acetyltransferase/amino-acid_acetyltransferase_ArgJ_[Bacillus_cereus]	CPTF_U	0	0	0
UIJ65066.1	bifunctional_glutamate_N-acetyltransferase/amino-acid_acetyltransferase_ArgJ_[Bacillus_cereus]	CPTF_metals_mix	58994.4	61607.01709	104.4285849
UIJ65066.1	bifunctional_glutamate_N-acetyltransferase/amino-acid_acetyltransferase_ArgJ_[Bacillus_cereus]	CPTF_zcontrol	0	0	0
UIJ65067.1	N-acetyl-gamma-glutamyl-phosphate_reductase_[Bacillus_cereus]	CPTF_Al	251842.6667	18021.86035	7.15599965
UIJ65067.1	N-acetyl-gamma-glutamyl-phosphate_reductase_[Bacillus_cereus]	CPTF_Cd	281660	72054.36279	25.58203607
UIJ65067.1	N-acetyl-gamma-glutamyl-phosphate_reductase_[Bacillus_cereus]	CPTF_Co	222702	50219.44254	22.55006356
UIJ65067.1	N-acetyl-gamma-glutamyl-phosphate_reductase_[Bacillus_cereus]	CPTF_Cu	250449.6667	12118.01074	4.838501445
UIJ65067.1	N-acetyl-gamma-glutamyl-phosphate_reductase_[Bacillus_cereus]	CPTF_Fe	238398.3333	64493.13692	27.05267944
UIJ65067.1	N-acetyl-gamma-glutamyl-phosphate_reductase_[Bacillus_cereus]	CPTF_Mn	185246.1333	98322.19382	53.07651612
UIJ65067.1	N-acetyl-gamma-glutamyl-phosphate_reductase_[Bacillus_cereus]	CPTF_Ni	227859.6667	36583.09722	16.05509995
UIJ65067.1	N-acetyl-gamma-glutamyl-phosphate_reductase_[Bacillus_cereus]	CPTF_U	294239.6667	38222.77411	12.99035393
UIJ65067.1	N-acetyl-gamma-glutamyl-phosphate_reductase_[Bacillus_cereus]	CPTF_metals_mix	792417.4667	122776.0163	15.49385538
UIJ65067.1	N-acetyl-gamma-glutamyl-phosphate_reductase_[Bacillus_cereus]	CPTF_zcontrol	210707.3333	29913.16435	14.1965464
UIJ65072.1	L-serine_ammonia-lyase,_iron-sulfur-dependent,_subunit_alpha_[Bacillus_cereus]	CPTF_Al	375751.1333	501346.49	133.4251438
UIJ65072.1	L-serine_ammonia-lyase,_iron-sulfur-dependent,_subunit_alpha_[Bacillus_cereus]	CPTF_Cd	44670.93333	41355.41145	92.57789878
UIJ65072.1	L-serine_ammonia-lyase,_iron-sulfur-dependent,_subunit_alpha_[Bacillus_cereus]	CPTF_Co	121918.6	70113.26705	57.50826129
UIJ65072.1	L-serine_ammonia-lyase,_iron-sulfur-dependent,_subunit_alpha_[Bacillus_cereus]	CPTF_Cu	640853.3333	276809.0192	43.19381749
UIJ65072.1	L-serine_ammonia-lyase,_iron-sulfur-dependent,_subunit_alpha_[Bacillus_cereus]	CPTF_Fe	282388.9667	259876.1366	92.02772322
UIJ65072.1	L-serine_ammonia-lyase,_iron-sulfur-dependent,_subunit_alpha_[Bacillus_cereus]	CPTF_Mn	355133.3	501204.1845	141.1312835
UIJ65072.1	L-serine_ammonia-lyase,_iron-sulfur-dependent,_subunit_alpha_[Bacillus_cereus]	CPTF_Ni	69582.7	84669.59691	121.6819654
UIJ65072.1	L-serine_ammonia-lyase,_iron-sulfur-dependent,_subunit_alpha_[Bacillus_cereus]	CPTF_U	119300.8333	23926.04875	20.05522349
UIJ65072.1	L-serine_ammonia-lyase,_iron-sulfur-dependent,_subunit_alpha_[Bacillus_cereus]	CPTF_metals_mix	743001	179380.4606	24.14269437
UIJ65072.1	L-serine_ammonia-lyase,_iron-sulfur-dependent,_subunit_alpha_[Bacillus_cereus]	CPTF_zcontrol	42892.8	39800.89446	92.79155117
UIJ65076.1	tripeptidase_T_[Bacillus_cereus]	CPTF_Al	10692651	465972.0103	4.357871685
UIJ65076.1	tripeptidase_T_[Bacillus_cereus]	CPTF_Cd	11426905	711301.4862	6.224795657
UIJ65076.1	tripeptidase_T_[Bacillus_cereus]	CPTF_Co	9672606.667	913960.2907	9.448955408
UIJ65076.1	tripeptidase_T_[Bacillus_cereus]	CPTF_Cu	10389730.23	484594.4294	4.664167582
UIJ65076.1	tripeptidase_T_[Bacillus_cereus]	CPTF_Fe	10528603.43	1199827.797	11.39588745
UIJ65076.1	tripeptidase_T_[Bacillus_cereus]	CPTF_Mn	10890185.57	1758445.217	16.14706385
UIJ65076.1	tripeptidase_T_[Bacillus_cereus]	CPTF_Ni	8414434.033	1017043.149	12.08688718
UIJ65076.1	tripeptidase_T_[Bacillus_cereus]	CPTF_U	9160753.833	232019.1269	2.532751465
UIJ65076.1	tripeptidase_T_[Bacillus_cereus]	CPTF_metals_mix	12489657.2	706659.5219	5.657957705
UIJ65076.1	tripeptidase_T_[Bacillus_cereus]	CPTF_zcontrol	10445166.33	368437.9334	3.527353434
UIJ65077.1	GNAT_family_N-acetyltransferase_[Bacillus_cereus]	CPTF_Al	0	0	0
UIJ65077.1	GNAT_family_N-acetyltransferase_[Bacillus_cereus]	CPTF_Cd	0	0	0
UIJ65077.1	GNAT_family_N-acetyltransferase_[Bacillus_cereus]	CPTF_Co	0	0	0
UIJ65077.1	GNAT_family_N-acetyltransferase_[Bacillus_cereus]	CPTF_Cu	0	0	0
UIJ65077.1	GNAT_family_N-acetyltransferase_[Bacillus_cereus]	CPTF_Fe	0	0	0
UIJ65077.1	GNAT_family_N-acetyltransferase_[Bacillus_cereus]	CPTF_Mn	0	0	0
UIJ65077.1	GNAT_family_N-acetyltransferase_[Bacillus_cereus]	CPTF_Ni	0	0	0
UIJ65077.1	GNAT_family_N-acetyltransferase_[Bacillus_cereus]	CPTF_U	0	0	0
UIJ65077.1	GNAT_family_N-acetyltransferase_[Bacillus_cereus]	CPTF_metals_mix	475867.1667	100048.9965	21.02456389
UIJ65077.1	GNAT_family_N-acetyltransferase_[Bacillus_cereus]	CPTF_zcontrol	0	0	0
UIJ65082.1	amino_acid_ABC_transporter_ATP-binding_protein_[Bacillus_cereus]	CPTF_Al	2690707.667	128139.3556	4.762291988
UIJ65082.1	amino_acid_ABC_transporter_ATP-binding_protein_[Bacillus_cereus]	CPTF_Cd	2341525.333	82165.6112	3.509063517
UIJ65082.1	amino_acid_ABC_transporter_ATP-binding_protein_[Bacillus_cereus]	CPTF_Co	2376387	567690.0921	23.88878967
UIJ65082.1	amino_acid_ABC_transporter_ATP-binding_protein_[Bacillus_cereus]	CPTF_Cu	2423014.333	122015.7859	5.035702191
UIJ65082.1	amino_acid_ABC_transporter_ATP-binding_protein_[Bacillus_cereus]	CPTF_Fe	2365560	484948.13	20.50035213
UIJ65082.1	amino_acid_ABC_transporter_ATP-binding_protein_[Bacillus_cereus]	CPTF_Mn	2371432.333	328146.3025	13.83747273
UIJ65082.1	amino_acid_ABC_transporter_ATP-binding_protein_[Bacillus_cereus]	CPTF_Ni	2256298.667	124510.6225	5.518357315
UIJ65082.1	amino_acid_ABC_transporter_ATP-binding_protein_[Bacillus_cereus]	CPTF_U	2661109.667	502084.739	18.86749521
UIJ65082.1	amino_acid_ABC_transporter_ATP-binding_protein_[Bacillus_cereus]	CPTF_metals_mix	2951206	427513.8633	14.48607326
UIJ65082.1	amino_acid_ABC_transporter_ATP-binding_protein_[Bacillus_cereus]	CPTF_zcontrol	2757831.333	268478.7953	9.73514196
UIJ65083.1	amino_acid_ABC_transporter_permease_[Bacillus_cereus]	CPTF_Al	633777.3	99284.99829	15.66559709
UIJ65083.1	amino_acid_ABC_transporter_permease_[Bacillus_cereus]	CPTF_Cd	508147.4333	122998.1684	24.20521297
UIJ65083.1	amino_acid_ABC_transporter_permease_[Bacillus_cereus]	CPTF_Co	674703.6667	83368.34657	12.35629072
UIJ65083.1	amino_acid_ABC_transporter_permease_[Bacillus_cereus]	CPTF_Cu	639345.5333	47378.96999	7.410542112
UIJ65083.1	amino_acid_ABC_transporter_permease_[Bacillus_cereus]	CPTF_Fe	583944.1667	120401.2158	20.61861778
UIJ65083.1	amino_acid_ABC_transporter_permease_[Bacillus_cereus]	CPTF_Mn	559430.4333	69091.27878	12.35028963
UIJ65083.1	amino_acid_ABC_transporter_permease_[Bacillus_cereus]	CPTF_Ni	685899.0333	190530.9197	27.77827499
UIJ65083.1	amino_acid_ABC_transporter_permease_[Bacillus_cereus]	CPTF_U	558809.3333	50554.00008	9.046735097

UIJ65083.1	amino_acid_ABC_transporter_permease_[Bacillus_cereus]	CPTF_metals_mix	750627.0333	63513.72583	8.461422652
UIJ65083.1	amino_acid_ABC_transporter_permease_[Bacillus_cereus]	CPTF_zcontrol	590405.3667	87291.86538	14.78507316
UIJ65084.1	transporter_substrate-binding_domain-containing_protein_[Bacillus_cereus]	CPTF_Al	5491414.433	443942.989	8.084310415
UIJ65084.1	transporter_substrate-binding_domain-containing_protein_[Bacillus_cereus]	CPTF_Cd	5515083.067	548760.649	9.950179215
UIJ65084.1	transporter_substrate-binding_domain-containing_protein_[Bacillus_cereus]	CPTF_Co	5953983.9	401670.8636	6.746253774
UIJ65084.1	transporter_substrate-binding_domain-containing_protein_[Bacillus_cereus]	CPTF_Cu	5759478.833	631221.2984	10.95969473
UIJ65084.1	transporter_substrate-binding_domain-containing_protein_[Bacillus_cereus]	CPTF_Fe	5304894.667	133598.9567	2.518409225
UIJ65084.1	transporter_substrate-binding_domain-containing_protein_[Bacillus_cereus]	CPTF_Mn	6003704.833	1361762.301	22.68203282
UIJ65084.1	transporter_substrate-binding_domain-containing_protein_[Bacillus_cereus]	CPTF_Ni	4197955.033	747143.5572	17.79779801
UIJ65084.1	transporter_substrate-binding_domain-containing_protein_[Bacillus_cereus]	CPTF_U	4691230.667	468359.6491	9.983726711
UIJ65084.1	transporter_substrate-binding_domain-containing_protein_[Bacillus_cereus]	CPTF_metals_mix	8488736.067	606472.4457	7.144437534
UIJ65084.1	transporter_substrate-binding_domain-containing_protein_[Bacillus_cereus]	CPTF_zcontrol	4960983.4	525756.8086	10.59783447
UIJ65088.1	2-oxo_acid_dehydrogenase_subunit_E2_[Bacillus_cereus]	CPTF_Al	2358691.633	179169.9924	7.596160087
UIJ65088.1	2-oxo_acid_dehydrogenase_subunit_E2_[Bacillus_cereus]	CPTF_Cd	2082684.333	769473.0284	36.94621485
UIJ65088.1	2-oxo_acid_dehydrogenase_subunit_E2_[Bacillus_cereus]	CPTF_Co	2257827	194018.107	8.593134326
UIJ65088.1	2-oxo_acid_dehydrogenase_subunit_E2_[Bacillus_cereus]	CPTF_Cu	2454080.933	111015.4122	4.523706236
UIJ65088.1	2-oxo_acid_dehydrogenase_subunit_E2_[Bacillus_cereus]	CPTF_Fe	1907240.333	572617.2517	30.02334009
UIJ65088.1	2-oxo_acid_dehydrogenase_subunit_E2_[Bacillus_cereus]	CPTF_Mn	2473872.633	115137.7651	4.654150889
UIJ65088.1	2-oxo_acid_dehydrogenase_subunit_E2_[Bacillus_cereus]	CPTF_Ni	2350050.067	243314.4608	10.35358626
UIJ65088.1	2-oxo_acid_dehydrogenase_subunit_E2_[Bacillus_cereus]	CPTF_U	1932419.667	849064.9095	43.93791495
UIJ65088.1	2-oxo_acid_dehydrogenase_subunit_E2_[Bacillus_cereus]	CPTF_metals_mix	3527558.167	592937.1064	16.80871238
UIJ65088.1	2-oxo_acid_dehydrogenase_subunit_E2_[Bacillus_cereus]	CPTF_zcontrol	2115954.667	498011.3518	23.53601236
UIJ65089.1	3-methyl-2-oxobutanoate_dehydrogenase_subunit_beta_[Bacillus_cereus]	CPTF_Al	1353412.767	339406.9325	25.07785806
UIJ65089.1	3-methyl-2-oxobutanoate_dehydrogenase_subunit_beta_[Bacillus_cereus]	CPTF_Cd	1842177	133417.3748	7.242375449
UIJ65089.1	3-methyl-2-oxobutanoate_dehydrogenase_subunit_beta_[Bacillus_cereus]	CPTF_Co	1289303.167	247160.6967	19.17009925
UIJ65089.1	3-methyl-2-oxobutanoate_dehydrogenase_subunit_beta_[Bacillus_cereus]	CPTF_Cu	1278471.667	104959.7744	8.209784946
UIJ65089.1	3-methyl-2-oxobutanoate_dehydrogenase_subunit_beta_[Bacillus_cereus]	CPTF_Fe	1493936.067	255415.2351	17.09679824
UIJ65089.1	3-methyl-2-oxobutanoate_dehydrogenase_subunit_beta_[Bacillus_cereus]	CPTF_Mn	1446840.7	201346.5963	13.91629337
UIJ65089.1	3-methyl-2-oxobutanoate_dehydrogenase_subunit_beta_[Bacillus_cereus]	CPTF_Ni	959210.0333	152158.5099	15.86289808
UIJ65089.1	3-methyl-2-oxobutanoate_dehydrogenase_subunit_beta_[Bacillus_cereus]	CPTF_U	1061656	203037.8317	19.12463469
UIJ65089.1	3-methyl-2-oxobutanoate_dehydrogenase_subunit_beta_[Bacillus_cereus]	CPTF_metals_mix	3902791.667	605812.5128	15.52254295
UIJ65089.1	3-methyl-2-oxobutanoate_dehydrogenase_subunit_beta_[Bacillus_cereus]	CPTF_zcontrol	1203508.667	194612.0814	16.17039301
UIJ65090.1	3-methyl-2-oxobutanoate_dehydrogenase_subunit_alpha_[Bacillus_cereus]	CPTF_Al	1961571.833	219669.0488	11.19862373
UIJ65090.1	3-methyl-2-oxobutanoate_dehydrogenase_subunit_alpha_[Bacillus_cereus]	CPTF_Cd	2098761.867	58589.95079	2.791643574
UIJ65090.1	3-methyl-2-oxobutanoate_dehydrogenase_subunit_alpha_[Bacillus_cereus]	CPTF_Co	1998405.167	147173.8568	7.364565467
UIJ65090.1	3-methyl-2-oxobutanoate_dehydrogenase_subunit_alpha_[Bacillus_cereus]	CPTF_Cu	2218667.6	217076.9453	9.784113011
UIJ65090.1	3-methyl-2-oxobutanoate_dehydrogenase_subunit_alpha_[Bacillus_cereus]	CPTF_Fe	2137977.633	143299.7247	6.702582967
UIJ65090.1	3-methyl-2-oxobutanoate_dehydrogenase_subunit_alpha_[Bacillus_cereus]	CPTF_Mn	2065590.733	201145.6843	9.73792538
UIJ65090.1	3-methyl-2-oxobutanoate_dehydrogenase_subunit_alpha_[Bacillus_cereus]	CPTF_Ni	1715567.167	757556.6658	44.15779694
UIJ65090.1	3-methyl-2-oxobutanoate_dehydrogenase_subunit_alpha_[Bacillus_cereus]	CPTF_U	1908702.6	430576.9616	22.55861975
UIJ65090.1	3-methyl-2-oxobutanoate_dehydrogenase_subunit_alpha_[Bacillus_cereus]	CPTF_metals_mix	2267418.067	35455.3194	1.563686905
UIJ65090.1	3-methyl-2-oxobutanoate_dehydrogenase_subunit_alpha_[Bacillus_cereus]	CPTF_zcontrol	2100997.567	70089.91988	3.336030512
UIJ65091.1	dihydrolipoyl_dehydrogenase_[Bacillus_cereus]	CPTF_Al	1293733.5	631675.0107	48.82574431
UIJ65091.1	dihydrolipoyl_dehydrogenase_[Bacillus_cereus]	CPTF_Cd	2273100.333	113432.7732	4.990222892
UIJ65091.1	dihydrolipoyl_dehydrogenase_[Bacillus_cereus]	CPTF_Co	1346673.8	443498.3695	32.93287279
UIJ65091.1	dihydrolipoyl_dehydrogenase_[Bacillus_cereus]	CPTF_Cu	1218026.867	216405.3852	17.7668811
UIJ65091.1	dihydrolipoyl_dehydrogenase_[Bacillus_cereus]	CPTF_Fe	1657838	394840.6433	23.81659989
UIJ65091.1	dihydrolipoyl_dehydrogenase_[Bacillus_cereus]	CPTF_Mn	1465803.333	994709.7988	67.86106814
UIJ65091.1	dihydrolipoyl_dehydrogenase_[Bacillus_cereus]	CPTF_Ni	207496.7	222557.6053	107.258383
UIJ65091.1	dihydrolipoyl_dehydrogenase_[Bacillus_cereus]	CPTF_U	1035835.8	511152.4488	49.34686065
UIJ65091.1	dihydrolipoyl_dehydrogenase_[Bacillus_cereus]	CPTF_metals_mix	2991661.1	165896.4145	5.545294369
UIJ65091.1	dihydrolipoyl_dehydrogenase_[Bacillus_cereus]	CPTF_zcontrol	1125754	560303.7707	49.7714217
UIJ65092.1	butyrate_kinase_[Bacillus_cereus]	CPTF_Al	141320	14104.72655	9.980700926
UIJ65092.1	butyrate_kinase_[Bacillus_cereus]	CPTF_Cd	129286.8	33965.25488	26.27124724
UIJ65092.1	butyrate_kinase_[Bacillus_cereus]	CPTF_Co	146355.0333	45908.1944	31.36769085
UIJ65092.1	butyrate_kinase_[Bacillus_cereus]	CPTF_Cu	132639.1667	30222.27908	22.78533547
UIJ65092.1	butyrate_kinase_[Bacillus_cereus]	CPTF_Fe	133483.1667	27789.62856	20.81882627
UIJ65092.1	butyrate_kinase_[Bacillus_cereus]	CPTF_Mn	194981.6	127903.4655	65.59771051
UIJ65092.1	butyrate_kinase_[Bacillus_cereus]	CPTF_Ni	122420.3333	18620.12396	15.20999286
UIJ65092.1	butyrate_kinase_[Bacillus_cereus]	CPTF_U	148801	41044.33325	27.58337192
UIJ65092.1	butyrate_kinase_[Bacillus_cereus]	CPTF_metals_mix	1004244.233	242776.0406	24.17499972

UIJ65092.1	butyrate_kinase_[Bacillus_cereus]	CPTF_zcontrol	117132.0333	30075.40061	25.67649494
UIJ65093.1	leucine_dehydrogenase_[Bacillus_cereus]	CPTF_Al	7410487.7	512563.0717	6.91672522
UIJ65093.1	leucine_dehydrogenase_[Bacillus_cereus]	CPTF_Cd	7925406	309656.8482	3.90714177
UIJ65093.1	leucine_dehydrogenase_[Bacillus_cereus]	CPTF_Co	7548645.8	728162.2573	9.646263404
UIJ65093.1	leucine_dehydrogenase_[Bacillus_cereus]	CPTF_Cu	7758026.733	485819.2881	6.26215022
UIJ65093.1	leucine_dehydrogenase_[Bacillus_cereus]	CPTF_Fe	7615900	798714.0245	10.4874542
UIJ65093.1	leucine_dehydrogenase_[Bacillus_cereus]	CPTF_Mn	7300114.467	873181.9113	11.96120849
UIJ65093.1	leucine_dehydrogenase_[Bacillus_cereus]	CPTF_Ni	6914912.967	501172.3748	7.247703294
UIJ65093.1	leucine_dehydrogenase_[Bacillus_cereus]	CPTF_U	6925508	1122541.19	16.20879205
UIJ65093.1	leucine_dehydrogenase_[Bacillus_cereus]	CPTF_metals_mix	12550386.4	1995854.991	15.90273739
UIJ65093.1	leucine_dehydrogenase_[Bacillus_cereus]	CPTF_zcontrol	6812733.5	216111.2272	3.172166168
UIJ65097.1	glycerophosphodiester_phosphodiesterase_[Bacillus_cereus]	CPTF_Al	0	0	0
UIJ65097.1	glycerophosphodiester_phosphodiesterase_[Bacillus_cereus]	CPTF_Cd	0	0	0
UIJ65097.1	glycerophosphodiester_phosphodiesterase_[Bacillus_cereus]	CPTF_Co	48137.33333	83376.30707	173.2050808
UIJ65097.1	glycerophosphodiester_phosphodiesterase_[Bacillus_cereus]	CPTF_Cu	0	0	0
UIJ65097.1	glycerophosphodiester_phosphodiesterase_[Bacillus_cereus]	CPTF_Fe	64225.33333	111241.5405	173.2050808
UIJ65097.1	glycerophosphodiester_phosphodiesterase_[Bacillus_cereus]	CPTF_Mn	0	0	0
UIJ65097.1	glycerophosphodiester_phosphodiesterase_[Bacillus_cereus]	CPTF_Ni	0	0	0
UIJ65097.1	glycerophosphodiester_phosphodiesterase_[Bacillus_cereus]	CPTF_U	0	0	0
UIJ65097.1	glycerophosphodiester_phosphodiesterase_[Bacillus_cereus]	CPTF_metals_mix	210416.4333	98305.89242	46.71968385
UIJ65097.1	glycerophosphodiester_phosphodiesterase_[Bacillus_cereus]	CPTF_zcontrol	0	0	0
UIJ65100.1	sporulation_transcription_factor_Spo0A_[Bacillus_cereus]	CPTF_Al	167373.63	101945.9812	60.90922517
UIJ65100.1	sporulation_transcription_factor_Spo0A_[Bacillus_cereus]	CPTF_Cd	105973.1667	39548.87091	37.31970286
UIJ65100.1	sporulation_transcription_factor_Spo0A_[Bacillus_cereus]	CPTF_Co	389183.3	169865.4025	43.64663194
UIJ65100.1	sporulation_transcription_factor_Spo0A_[Bacillus_cereus]	CPTF_Cu	171669.8333	28023.18419	16.32388385
UIJ65100.1	sporulation_transcription_factor_Spo0A_[Bacillus_cereus]	CPTF_Fe	249750.2333	91091.70712	36.47312193
UIJ65100.1	sporulation_transcription_factor_Spo0A_[Bacillus_cereus]	CPTF_Mn	211599.1333	245274.4722	115.9146866
UIJ65100.1	sporulation_transcription_factor_Spo0A_[Bacillus_cereus]	CPTF_Ni	89536.16667	5737.064565	6.40753874
UIJ65100.1	sporulation_transcription_factor_Spo0A_[Bacillus_cereus]	CPTF_U	152520	8687.199606	5.695777345
UIJ65100.1	sporulation_transcription_factor_Spo0A_[Bacillus_cereus]	CPTF_metals_mix	574747.7667	82115.27252	14.28718427
UIJ65100.1	sporulation_transcription_factor_Spo0A_[Bacillus_cereus]	CPTF_zcontrol	291459.5333	228535.9439	78.41086592
UIJ65103.1	arginine_repressor_ArgR_[Bacillus_cereus]	CPTF_Al	2717748.767	176356.5144	6.489066118
UIJ65103.1	arginine_repressor_ArgR_[Bacillus_cereus]	CPTF_Cd	2700143	60088.94224	2.225398515
UIJ65103.1	arginine_repressor_ArgR_[Bacillus_cereus]	CPTF_Co	2723340.333	165894.2451	6.09157229
UIJ65103.1	arginine_repressor_ArgR_[Bacillus_cereus]	CPTF_Cu	2922667.333	84912.72468	2.905316103
UIJ65103.1	arginine_repressor_ArgR_[Bacillus_cereus]	CPTF_Fe	2702711.667	81102.32007	3.00077589
UIJ65103.1	arginine_repressor_ArgR_[Bacillus_cereus]	CPTF_Mn	2667040.667	440680.6808	16.52320815
UIJ65103.1	arginine_repressor_ArgR_[Bacillus_cereus]	CPTF_Ni	2737883.333	40322.50156	1.472761862
UIJ65103.1	arginine_repressor_ArgR_[Bacillus_cereus]	CPTF_U	2914282	186113.6133	6.386259574
UIJ65103.1	arginine_repressor_ArgR_[Bacillus_cereus]	CPTF_metals_mix	2260062	79241.09652	3.506147022
UIJ65103.1	arginine_repressor_ArgR_[Bacillus_cereus]	CPTF_zcontrol	3002704.233	201332.2999	6.705032671
UIJ65105.1	1-deoxy-D-xylulose-5-phosphate_synthase_[Bacillus_cereus]	CPTF_Al	143437.2667	95499.0355	66.57895658
UIJ65105.1	1-deoxy-D-xylulose-5-phosphate_synthase_[Bacillus_cereus]	CPTF_Cd	183459.6	6925.46477	3.774926343
UIJ65105.1	1-deoxy-D-xylulose-5-phosphate_synthase_[Bacillus_cereus]	CPTF_Co	204815.7333	120914.9205	59.03595323
UIJ65105.1	1-deoxy-D-xylulose-5-phosphate_synthase_[Bacillus_cereus]	CPTF_Cu	274902.3333	105775.7759	38.47758389
UIJ65105.1	1-deoxy-D-xylulose-5-phosphate_synthase_[Bacillus_cereus]	CPTF_Fe	252309.3	59679.40102	23.65327042
UIJ65105.1	1-deoxy-D-xylulose-5-phosphate_synthase_[Bacillus_cereus]	CPTF_Mn	130175.6667	104465.8455	80.24990242
UIJ65105.1	1-deoxy-D-xylulose-5-phosphate_synthase_[Bacillus_cereus]	CPTF_Ni	228513.1667	51108.46858	22.36565592
UIJ65105.1	1-deoxy-D-xylulose-5-phosphate_synthase_[Bacillus_cereus]	CPTF_U	209183.4333	111178.5804	53.14884577
UIJ65105.1	1-deoxy-D-xylulose-5-phosphate_synthase_[Bacillus_cereus]	CPTF_metals_mix	276016.6	107155.5695	38.82214675
UIJ65105.1	1-deoxy-D-xylulose-5-phosphate_synthase_[Bacillus_cereus]	CPTF_zcontrol	229303.1667	82600.41939	36.02236314
UIJ65106.1	(2E,6E)-farnesyl_diphosphate_synthase_[Bacillus_cereus]	CPTF_Al	104156.3333	91742.67075	88.08170162
UIJ65106.1	(2E,6E)-farnesyl_diphosphate_synthase_[Bacillus_cereus]	CPTF_Cd	106343.9	44913.22671	42.23394732
UIJ65106.1	(2E,6E)-farnesyl_diphosphate_synthase_[Bacillus_cereus]	CPTF_Co	124460	10998.37006	8.836871333
UIJ65106.1	(2E,6E)-farnesyl_diphosphate_synthase_[Bacillus_cereus]	CPTF_Cu	109249.7667	17822.96825	16.31396459
UIJ65106.1	(2E,6E)-farnesyl_diphosphate_synthase_[Bacillus_cereus]	CPTF_Fe	84872.63333	36562.32659	43.07905287
UIJ65106.1	(2E,6E)-farnesyl_diphosphate_synthase_[Bacillus_cereus]	CPTF_Mn	87240	86879.27507	99.58651429
UIJ65106.1	(2E,6E)-farnesyl_diphosphate_synthase_[Bacillus_cereus]	CPTF_Ni	97143	85240.76864	87.74772103
UIJ65106.1	(2E,6E)-farnesyl_diphosphate_synthase_[Bacillus_cereus]	CPTF_U	114430.3333	109460.1966	95.6566265
UIJ65106.1	(2E,6E)-farnesyl_diphosphate_synthase_[Bacillus_cereus]	CPTF_metals_mix	88123.66667	8277.49419	9.393043325
UIJ65106.1	(2E,6E)-farnesyl_diphosphate_synthase_[Bacillus_cereus]	CPTF_zcontrol	132598	12989.26815	9.795975918

UIJ65109.1	bifunctional_methylenetetrahydrofolate_dehydrogenase/methenyltetrahydrofolate_cyclohydrolase_FolD_[Bacillus_cereus]	CPTF_Al	13369616.27	921043.5965	6.889080271
UIJ65109.1	bifunctional_methylenetetrahydrofolate_dehydrogenase/methenyltetrahydrofolate_cyclohydrolase_FolD_[Bacillus_cereus]	CPTF_Cd	12298507.97	509675.5935	4.144206719
UIJ65109.1	bifunctional_methylenetetrahydrofolate_dehydrogenase/methenyltetrahydrofolate_cyclohydrolase_FolD_[Bacillus_cereus]	CPTF_Co	12640814.7	443666.9972	3.509797491
UIJ65109.1	bifunctional_methylenetetrahydrofolate_dehydrogenase/methenyltetrahydrofolate_cyclohydrolase_FolD_[Bacillus_cereus]	CPTF_Cu	10987489.3	1273693.309	11.59221432
UIJ65109.1	bifunctional_methylenetetrahydrofolate_dehydrogenase/methenyltetrahydrofolate_cyclohydrolase_FolD_[Bacillus_cereus]	CPTF_Fe	11894022	524441.3369	4.409285075
UIJ65109.1	bifunctional_methylenetetrahydrofolate_dehydrogenase/methenyltetrahydrofolate_cyclohydrolase_FolD_[Bacillus_cereus]	CPTF_Mn	13096390	754623.3467	5.762071431
UIJ65109.1	bifunctional_methylenetetrahydrofolate_dehydrogenase/methenyltetrahydrofolate_cyclohydrolase_FolD_[Bacillus_cereus]	CPTF_Ni	12591498.1	627294.6374	4.981890418
UIJ65109.1	bifunctional_methylenetetrahydrofolate_dehydrogenase/methenyltetrahydrofolate_cyclohydrolase_FolD_[Bacillus_cereus]	CPTF_U	12425802.67	706785.5157	5.68804716
UIJ65109.1	bifunctional_methylenetetrahydrofolate_dehydrogenase/methenyltetrahydrofolate_cyclohydrolase_FolD_[Bacillus_cereus]	CPTF_metals_mix	12064657.43	555680.6595	4.605855264
UIJ65109.1	bifunctional_methylenetetrahydrofolate_dehydrogenase/methenyltetrahydrofolate_cyclohydrolase_FolD_[Bacillus_cereus]	CPTF_zcontrol	13997078.33	623807.8875	4.456700696
UIJ65110.1	N_utilization_substance_protein_NusB_[Bacillus_cereus]	CPTF_Al	274714.3333	475819.1829	173.2050808
UIJ65110.1	N_utilization_substance_protein_NusB_[Bacillus_cereus]	CPTF_Cd	95052.66667	82362.25884	86.64907754
UIJ65110.1	N_utilization_substance_protein_NusB_[Bacillus_cereus]	CPTF_Co	251104.3333	434925.4633	173.2050808
UIJ65110.1	N_utilization_substance_protein_NusB_[Bacillus_cereus]	CPTF_Cu	841047.3333	29620.61507	3.521872539
UIJ65110.1	N_utilization_substance_protein_NusB_[Bacillus_cereus]	CPTF_Fe	0	0	0
UIJ65110.1	N_utilization_substance_protein_NusB_[Bacillus_cereus]	CPTF_Mn	615308	543516.4069	88.33241351
UIJ65110.1	N_utilization_substance_protein_NusB_[Bacillus_cereus]	CPTF_Ni	777994.3333	162518.7468	20.88945122
UIJ65110.1	N_utilization_substance_protein_NusB_[Bacillus_cereus]	CPTF_U	691544.3333	105224.2135	15.21583048
UIJ65110.1	N_utilization_substance_protein_NusB_[Bacillus_cereus]	CPTF_metals_mix	934292.9	133914.0453	14.33319737
UIJ65110.1	N_utilization_substance_protein_NusB_[Bacillus_cereus]	CPTF_zcontrol	526477.3333	480469.1282	91.26112328
UIJ65111.1	Asp23/Gls24_family_envelope_stress_response_protein_[Bacillus_cereus]	CPTF_Al	6012000	110161.547	1.832361061
UIJ65111.1	Asp23/Gls24_family_envelope_stress_response_protein_[Bacillus_cereus]	CPTF_Cd	6255089.467	109695.0846	1.753693295
UIJ65111.1	Asp23/Gls24_family_envelope_stress_response_protein_[Bacillus_cereus]	CPTF_Co	5904095.933	168109.7225	2.847340632
UIJ65111.1	Asp23/Gls24_family_envelope_stress_response_protein_[Bacillus_cereus]	CPTF_Cu	6129713.233	250293.5885	4.083283818
UIJ65111.1	Asp23/Gls24_family_envelope_stress_response_protein_[Bacillus_cereus]	CPTF_Fe	6119514.867	184466.2063	3.014392649
UIJ65111.1	Asp23/Gls24_family_envelope_stress_response_protein_[Bacillus_cereus]	CPTF_Mn	6090818.2	646448.1083	10.61348553
UIJ65111.1	Asp23/Gls24_family_envelope_stress_response_protein_[Bacillus_cereus]	CPTF_Ni	5872906.1	96702.20741	1.646581876
UIJ65111.1	Asp23/Gls24_family_envelope_stress_response_protein_[Bacillus_cereus]	CPTF_U	5990617.667	248423.8839	4.146882638
UIJ65111.1	Asp23/Gls24_family_envelope_stress_response_protein_[Bacillus_cereus]	CPTF_metals_mix	4398678.3	200705.9535	4.562869568
UIJ65111.1	Asp23/Gls24_family_envelope_stress_response_protein_[Bacillus_cereus]	CPTF_zcontrol	6252367.567	97315.73937	1.556462225
UIJ65112.1	acetyl-CoA_carboxylase_biotin_carboxylase_subunit_[Bacillus_cereus]	CPTF_Al	5239871.333	711273.0835	13.57424712
UIJ65112.1	acetyl-CoA_carboxylase_biotin_carboxylase_subunit_[Bacillus_cereus]	CPTF_Cd	6364316.667	326650.0814	5.132524017
UIJ65112.1	acetyl-CoA_carboxylase_biotin_carboxylase_subunit_[Bacillus_cereus]	CPTF_Co	5025513.333	233519.3708	4.646676971
UIJ65112.1	acetyl-CoA_carboxylase_biotin_carboxylase_subunit_[Bacillus_cereus]	CPTF_Cu	4663252.8	148737.9413	3.189574911
UIJ65112.1	acetyl-CoA_carboxylase_biotin_carboxylase_subunit_[Bacillus_cereus]	CPTF_Fe	5407863.033	591432.7365	10.93653321
UIJ65112.1	acetyl-CoA_carboxylase_biotin_carboxylase_subunit_[Bacillus_cereus]	CPTF_Mn	5373015.333	758220.9813	14.11164745
UIJ65112.1	acetyl-CoA_carboxylase_biotin_carboxylase_subunit_[Bacillus_cereus]	CPTF_Ni	4336390.267	306808.0854	7.075195417
UIJ65112.1	acetyl-CoA_carboxylase_biotin_carboxylase_subunit_[Bacillus_cereus]	CPTF_U	4783734.667	664891.035	13.89899485
UIJ65112.1	acetyl-CoA_carboxylase_biotin_carboxylase_subunit_[Bacillus_cereus]	CPTF_metals_mix	7030516.133	320405.686	4.557356528
UIJ65112.1	acetyl-CoA_carboxylase_biotin_carboxylase_subunit_[Bacillus_cereus]	CPTF_zcontrol	4882935.667	359797.2919	7.368462673
UIJ65113.1	acetyl-CoA_carboxylase_biotin_carboxyl_carrier_protein_[Bacillus_cereus]	CPTF_Al	462559.6667	47858.13948	10.34637106
UIJ65113.1	acetyl-CoA_carboxylase_biotin_carboxyl_carrier_protein_[Bacillus_cereus]	CPTF_Cd	485048	63228.7791	13.03557155
UIJ65113.1	acetyl-CoA_carboxylase_biotin_carboxyl_carrier_protein_[Bacillus_cereus]	CPTF_Co	555818	101413.9662	18.24589456
UIJ65113.1	acetyl-CoA_carboxylase_biotin_carboxyl_carrier_protein_[Bacillus_cereus]	CPTF_Cu	427154.6667	177724.2054	41.60652319
UIJ65113.1	acetyl-CoA_carboxylase_biotin_carboxyl_carrier_protein_[Bacillus_cereus]	CPTF_Fe	526083.6667	127663.7098	24.26680733
UIJ65113.1	acetyl-CoA_carboxylase_biotin_carboxyl_carrier_protein_[Bacillus_cereus]	CPTF_Mn	433015.3333	82315.08279	19.00973856
UIJ65113.1	acetyl-CoA_carboxylase_biotin_carboxyl_carrier_protein_[Bacillus_cereus]	CPTF_Ni	419142	206458.66	49.25744974
UIJ65113.1	acetyl-CoA_carboxylase_biotin_carboxyl_carrier_protein_[Bacillus_cereus]	CPTF_U	65414.5	60428.52045	92.37786799
UIJ65113.1	acetyl-CoA_carboxylase_biotin_carboxyl_carrier_protein_[Bacillus_cereus]	CPTF_metals_mix	393321.1333	107492.5337	27.32945795
UIJ65113.1	acetyl-CoA_carboxylase_biotin_carboxyl_carrier_protein_[Bacillus_cereus]	CPTF_zcontrol	408417.6667	171640.9337	42.02583475
UIJ65126.1	elongation_factor_P_[Bacillus_cereus]	CPTF_Al	0	0	0
UIJ65126.1	elongation_factor_P_[Bacillus_cereus]	CPTF_Cd	0	0	0
UIJ65126.1	elongation_factor_P_[Bacillus_cereus]	CPTF_Co	0	0	0
UIJ65126.1	elongation_factor_P_[Bacillus_cereus]	CPTF_Cu	0	0	0
UIJ65126.1	elongation_factor_P_[Bacillus_cereus]	CPTF_Fe	0	0	0
UIJ65126.1	elongation_factor_P_[Bacillus_cereus]	CPTF_Mn	0	0	0
UIJ65126.1	elongation_factor_P_[Bacillus_cereus]	CPTF_Ni	0	0	0
UIJ65126.1	elongation_factor_P_[Bacillus_cereus]	CPTF_U	0	0	0
UIJ65126.1	elongation_factor_P_[Bacillus_cereus]	CPTF_metals_mix	532754.3333	260197.7912	48.84010789
UIJ65126.1	elongation_factor_P_[Bacillus_cereus]	CPTF_zcontrol	0	0	0
UIJ65131.1	HAD_family_hydrolase_[Bacillus_cereus]	CPTF_Al	266679	2351.995111	0.881957376

UIJ65131.1	HAD_family_hydrolase_[Bacillus_cereus]	CPTF_Cd	136631.3333	119569.7743	87.51270402
UIJ65131.1	HAD_family_hydrolase_[Bacillus_cereus]	CPTF_Co	92643.66667	160463.5377	173.2050808
UIJ65131.1	HAD_family_hydrolase_[Bacillus_cereus]	CPTF_Cu	159733	163802.3519	102.5475962
UIJ65131.1	HAD_family_hydrolase_[Bacillus_cereus]	CPTF_Fe	218937.6667	190228.1365	86.88689315
UIJ65131.1	HAD_family_hydrolase_[Bacillus_cereus]	CPTF_Mn	223807	199050.5656	88.93848967
UIJ65131.1	HAD_family_hydrolase_[Bacillus_cereus]	CPTF_Ni	186028	162386.147	87.29123951
UIJ65131.1	HAD_family_hydrolase_[Bacillus_cereus]	CPTF_U	244775.3333	246026.4	100.5111081
UIJ65131.1	HAD_family_hydrolase_[Bacillus_cereus]	CPTF_metals_mix	0	0	0
UIJ65131.1	HAD_family_hydrolase_[Bacillus_cereus]	CPTF_zcontrol	223274.6667	199372.4634	89.29470878
UIJ65132.1	transcriptional_regulator_MntR_[Bacillus_cereus]	CPTF_Al	117367.5667	31503.53924	26.84177591
UIJ65132.1	transcriptional_regulator_MntR_[Bacillus_cereus]	CPTF_Cd	131114.2	56019.96188	42.72608297
UIJ65132.1	transcriptional_regulator_MntR_[Bacillus_cereus]	CPTF_Co	44465.6	39254.72876	88.28111789
UIJ65132.1	transcriptional_regulator_MntR_[Bacillus_cereus]	CPTF_Cu	116988.0667	63400.09744	54.19364492
UIJ65132.1	transcriptional_regulator_MntR_[Bacillus_cereus]	CPTF_Fe	79936.03333	7874.747349	9.85131113
UIJ65132.1	transcriptional_regulator_MntR_[Bacillus_cereus]	CPTF_Mn	69803.76667	5873.697478	8.414585285
UIJ65132.1	transcriptional_regulator_MntR_[Bacillus_cereus]	CPTF_Ni	78475.5	70689.03924	90.07784498
UIJ65132.1	transcriptional_regulator_MntR_[Bacillus_cereus]	CPTF_U	158287.7333	118867.8839	75.09608066
UIJ65132.1	transcriptional_regulator_MntR_[Bacillus_cereus]	CPTF_metals_mix	60104.56667	53356.99301	88.77360901
UIJ65132.1	transcriptional_regulator_MntR_[Bacillus_cereus]	CPTF_zcontrol	113633.0333	33629.54053	29.59486299
UIJ65137.1	lipoate--protein_ligase_family_protein_[Bacillus_cereus]	CPTF_Al	0	0	0
UIJ65137.1	lipoate--protein_ligase_family_protein_[Bacillus_cereus]	CPTF_Cd	23096.5	40004.31148	173.2050808
UIJ65137.1	lipoate--protein_ligase_family_protein_[Bacillus_cereus]	CPTF_Co	0	0	0
UIJ65137.1	lipoate--protein_ligase_family_protein_[Bacillus_cereus]	CPTF_Cu	0	0	0
UIJ65137.1	lipoate--protein_ligase_family_protein_[Bacillus_cereus]	CPTF_Fe	0	0	0
UIJ65137.1	lipoate--protein_ligase_family_protein_[Bacillus_cereus]	CPTF_Mn	0	0	0
UIJ65137.1	lipoate--protein_ligase_family_protein_[Bacillus_cereus]	CPTF_Ni	0	0	0
UIJ65137.1	lipoate--protein_ligase_family_protein_[Bacillus_cereus]	CPTF_U	0	0	0
UIJ65137.1	lipoate--protein_ligase_family_protein_[Bacillus_cereus]	CPTF_metals_mix	95203.03333	19194.38527	20.161527
UIJ65137.1	lipoate--protein_ligase_family_protein_[Bacillus_cereus]	CPTF_zcontrol	0	0	0
UIJ65138.1	rhodanese-like_domain-containing_protein_[Bacillus_cereus]	CPTF_Al	237928.6667	38807.54566	16.31058006
UIJ65138.1	rhodanese-like_domain-containing_protein_[Bacillus_cereus]	CPTF_Cd	239006.6667	55197.19001	23.09441439
UIJ65138.1	rhodanese-like_domain-containing_protein_[Bacillus_cereus]	CPTF_Co	303921.4333	42704.92719	14.05130488
UIJ65138.1	rhodanese-like_domain-containing_protein_[Bacillus_cereus]	CPTF_Cu	279946.9667	41850.18785	14.94932713
UIJ65138.1	rhodanese-like_domain-containing_protein_[Bacillus_cereus]	CPTF_Fe	226160.6667	32354.9032	14.3061584
UIJ65138.1	rhodanese-like_domain-containing_protein_[Bacillus_cereus]	CPTF_Mn	261537.3333	138906.3201	53.11146916
UIJ65138.1	rhodanese-like_domain-containing_protein_[Bacillus_cereus]	CPTF_Ni	245014.3333	21224.0892	8.662386772
UIJ65138.1	rhodanese-like_domain-containing_protein_[Bacillus_cereus]	CPTF_U	208698	31857.41936	15.26484171
UIJ65138.1	rhodanese-like_domain-containing_protein_[Bacillus_cereus]	CPTF_metals_mix	962645.9667	85801.90634	8.913132066
UIJ65138.1	rhodanese-like_domain-containing_protein_[Bacillus_cereus]	CPTF_zcontrol	251367.3333	36120.47909	14.3695995
UIJ65145.1	aminomethyl-transferring_glycine_dehydrogenase_subunit_2_[Bacillus_cereus]	CPTF_Al	16317418.67	301464.7349	1.847502605
UIJ65145.1	aminomethyl-transferring_glycine_dehydrogenase_subunit_2_[Bacillus_cereus]	CPTF_Cd	17230979.97	543323.5845	3.153178667
UIJ65145.1	aminomethyl-transferring_glycine_dehydrogenase_subunit_2_[Bacillus_cereus]	CPTF_Co	16626565.2	1968062.933	11.83685812
UIJ65145.1	aminomethyl-transferring_glycine_dehydrogenase_subunit_2_[Bacillus_cereus]	CPTF_Cu	15485017.1	2281087.802	14.73093499
UIJ65145.1	aminomethyl-transferring_glycine_dehydrogenase_subunit_2_[Bacillus_cereus]	CPTF_Fe	16055899.87	1461800.968	9.104447465
UIJ65145.1	aminomethyl-transferring_glycine_dehydrogenase_subunit_2_[Bacillus_cereus]	CPTF_Mn	16387025.3	2154409.113	13.14704209
UIJ65145.1	aminomethyl-transferring_glycine_dehydrogenase_subunit_2_[Bacillus_cereus]	CPTF_Ni	15191822.33	1163762.599	7.660454248
UIJ65145.1	aminomethyl-transferring_glycine_dehydrogenase_subunit_2_[Bacillus_cereus]	CPTF_U	16921429.8	2797101.825	16.52993782
UIJ65145.1	aminomethyl-transferring_glycine_dehydrogenase_subunit_2_[Bacillus_cereus]	CPTF_metals_mix	19591188.9	1010486.535	5.157862241
UIJ65145.1	aminomethyl-transferring_glycine_dehydrogenase_subunit_2_[Bacillus_cereus]	CPTF_zcontrol	16549490.17	630388.9096	3.809113775
UIJ65146.1	aminomethyl-transferring_glycine_dehydrogenase_subunit_1_[Bacillus_cereus]	CPTF_Al	7341053.933	444548.7471	6.055652924
UIJ65146.1	aminomethyl-transferring_glycine_dehydrogenase_subunit_1_[Bacillus_cereus]	CPTF_Cd	7459886.333	227269.1161	3.046549317
UIJ65146.1	aminomethyl-transferring_glycine_dehydrogenase_subunit_1_[Bacillus_cereus]	CPTF_Co	6711940.467	668933.4187	9.966319309
UIJ65146.1	aminomethyl-transferring_glycine_dehydrogenase_subunit_1_[Bacillus_cereus]	CPTF_Cu	6513840.3	89985.78624	1.381455211
UIJ65146.1	aminomethyl-transferring_glycine_dehydrogenase_subunit_1_[Bacillus_cereus]	CPTF_Fe	6977311.9	351507.8621	5.037869413
UIJ65146.1	aminomethyl-transferring_glycine_dehydrogenase_subunit_1_[Bacillus_cereus]	CPTF_Mn	6485650	818088.5755	12.61382553
UIJ65146.1	aminomethyl-transferring_glycine_dehydrogenase_subunit_1_[Bacillus_cereus]	CPTF_Ni	5332707.2	785659.8569	14.73285195
UIJ65146.1	aminomethyl-transferring_glycine_dehydrogenase_subunit_1_[Bacillus_cereus]	CPTF_U	5538932.667	224779.2964	4.058169867
UIJ65146.1	aminomethyl-transferring_glycine_dehydrogenase_subunit_1_[Bacillus_cereus]	CPTF_metals_mix	5695902.467	372256.9635	6.535522082
UIJ65146.1	aminomethyl-transferring_glycine_dehydrogenase_subunit_1_[Bacillus_cereus]	CPTF_zcontrol	6931869	471708.0456	6.804918639
UIJ65147.1	glycine_cleavage_system_aminomethyltransferase_GcvT_[Bacillus_cereus]	CPTF_Al	8253857.267	684281.8875	8.290449731
UIJ65147.1	glycine_cleavage_system_aminomethyltransferase_GcvT_[Bacillus_cereus]	CPTF_Cd	9065086.067	752303.9422	8.298916709

UIJ65147.1	glycine_cleavage_system_aminomethyltransferase_GcvT_[Bacillus_cereus]	CPTF_Co	7961615.8	467364.0071	5.87021553
UIJ65147.1	glycine_cleavage_system_aminomethyltransferase_GcvT_[Bacillus_cereus]	CPTF_Cu	7566758.133	698526.879	9.23152117
UIJ65147.1	glycine_cleavage_system_aminomethyltransferase_GcvT_[Bacillus_cereus]	CPTF_Fe	7531270.567	920680.9375	12.22477574
UIJ65147.1	glycine_cleavage_system_aminomethyltransferase_GcvT_[Bacillus_cereus]	CPTF_Mn	8130290.933	2498517.308	30.73097049
UIJ65147.1	glycine_cleavage_system_aminomethyltransferase_GcvT_[Bacillus_cereus]	CPTF_Ni	6314941.867	1101427.157	17.44160405
UIJ65147.1	glycine_cleavage_system_aminomethyltransferase_GcvT_[Bacillus_cereus]	CPTF_U	5580850.467	337396.5464	6.045611657
UIJ65147.1	glycine_cleavage_system_aminomethyltransferase_GcvT_[Bacillus_cereus]	CPTF_metals_mix	11703525.63	888820.7148	7.594469757
UIJ65147.1	glycine_cleavage_system_aminomethyltransferase_GcvT_[Bacillus_cereus]	CPTF_zcontrol	7640444.267	469360.7795	6.143108479
UIJ65168.1	2OG-Fe(II)_oxygenase_[Bacillus_cereus]	CPTF_Al	12259.96667	21234.88517	173.2050808
UIJ65168.1	2OG-Fe(II)_oxygenase_[Bacillus_cereus]	CPTF_Cd	74988.9	22560.14337	30.08464368
UIJ65168.1	2OG-Fe(II)_oxygenase_[Bacillus_cereus]	CPTF_Co	23643.76667	40952.20515	173.2050808
UIJ65168.1	2OG-Fe(II)_oxygenase_[Bacillus_cereus]	CPTF_Cu	22820.56667	21584.53727	94.58370418
UIJ65168.1	2OG-Fe(II)_oxygenase_[Bacillus_cereus]	CPTF_Fe	0	0	0
UIJ65168.1	2OG-Fe(II)_oxygenase_[Bacillus_cereus]	CPTF_Mn	20290.76667	35144.63879	173.2050808
UIJ65168.1	2OG-Fe(II)_oxygenase_[Bacillus_cereus]	CPTF_Ni	0	0	0
UIJ65168.1	2OG-Fe(II)_oxygenase_[Bacillus_cereus]	CPTF_U	0	0	0
UIJ65168.1	2OG-Fe(II)_oxygenase_[Bacillus_cereus]	CPTF_metals_mix	117096.9667	34025.31814	29.05738646
UIJ65168.1	2OG-Fe(II)_oxygenase_[Bacillus_cereus]	CPTF_zcontrol	16288.7	28212.85599	173.2050808
UIJ65175.1	helix-turn-helix_domain-containing_protein_[Bacillus_cereus]	CPTF_Al	7215.3	12497.26619	173.2050808
UIJ65175.1	helix-turn-helix_domain-containing_protein_[Bacillus_cereus]	CPTF_Cd	20543.1	35581.69294	173.2050808
UIJ65175.1	helix-turn-helix_domain-containing_protein_[Bacillus_cereus]	CPTF_Co	40115.66667	35598.30118	88.73914892
UIJ65175.1	helix-turn-helix_domain-containing_protein_[Bacillus_cereus]	CPTF_Cu	88133.4	21936.63709	24.89026532
UIJ65175.1	helix-turn-helix_domain-containing_protein_[Bacillus_cereus]	CPTF_Fe	23426.13333	40575.25316	173.2050808
UIJ65175.1	helix-turn-helix_domain-containing_protein_[Bacillus_cereus]	CPTF_Mn	6216.166667	10766.71649	173.2050808
UIJ65175.1	helix-turn-helix_domain-containing_protein_[Bacillus_cereus]	CPTF_Ni	26780.2	25931.57639	96.83115283
UIJ65175.1	helix-turn-helix_domain-containing_protein_[Bacillus_cereus]	CPTF_U	0	0	0
UIJ65175.1	helix-turn-helix_domain-containing_protein_[Bacillus_cereus]	CPTF_metals_mix	1858073.3	75336.44755	4.054546586
UIJ65175.1	helix-turn-helix_domain-containing_protein_[Bacillus_cereus]	CPTF_zcontrol	15868.96667	27485.85653	173.2050808
UIJ65177.1	L-cystine_transporter_[Bacillus_cereus]	CPTF_Al	28297.2	30239.25244	106.8630552
UIJ65177.1	L-cystine_transporter_[Bacillus_cereus]	CPTF_Cd	71171.33333	53305.13983	74.89692456
UIJ65177.1	L-cystine_transporter_[Bacillus_cereus]	CPTF_Co	52393.33333	47228.47865	90.14215291
UIJ65177.1	L-cystine_transporter_[Bacillus_cereus]	CPTF_Cu	48831.46667	14901.76419	30.51672458
UIJ65177.1	L-cystine_transporter_[Bacillus_cereus]	CPTF_Fe	47922	23459.24403	48.95297364
UIJ65177.1	L-cystine_transporter_[Bacillus_cereus]	CPTF_Mn	27264.7	27095.17168	99.37821316
UIJ65177.1	L-cystine_transporter_[Bacillus_cereus]	CPTF_Ni	21831.8	37813.78682	173.2050808
UIJ65177.1	L-cystine_transporter_[Bacillus_cereus]	CPTF_U	20810.5	36044.84333	173.2050808
UIJ65177.1	L-cystine_transporter_[Bacillus_cereus]	CPTF_metals_mix	113167.5333	33533.54817	29.6317744
UIJ65177.1	L-cystine_transporter_[Bacillus_cereus]	CPTF_zcontrol	15971.9	27664.14229	173.2050808
UIJ65181.1	hypothetical_protein_LW858_19795_[Bacillus_cereus]	CPTF_Al	120627.2667	64544.62505	53.50749199
UIJ65181.1	hypothetical_protein_LW858_19795_[Bacillus_cereus]	CPTF_Cd	145237	60264.34141	41.49379387
UIJ65181.1	hypothetical_protein_LW858_19795_[Bacillus_cereus]	CPTF_Co	129414.9667	79594.58712	61.50338649
UIJ65181.1	hypothetical_protein_LW858_19795_[Bacillus_cereus]	CPTF_Cu	92828.8	71164.86348	76.66248349
UIJ65181.1	hypothetical_protein_LW858_19795_[Bacillus_cereus]	CPTF_Fe	49286.96667	43225.26709	87.70121192
UIJ65181.1	hypothetical_protein_LW858_19795_[Bacillus_cereus]	CPTF_Mn	89204.46667	39708.72864	44.51428289
UIJ65181.1	hypothetical_protein_LW858_19795_[Bacillus_cereus]	CPTF_Ni	78043.7	9536.113389	12.21894066
UIJ65181.1	hypothetical_protein_LW858_19795_[Bacillus_cereus]	CPTF_U	19301.3	22577.19582	116.9724102
UIJ65181.1	hypothetical_protein_LW858_19795_[Bacillus_cereus]	CPTF_metals_mix	104943.5667	64991.13767	61.92960629
UIJ65181.1	hypothetical_protein_LW858_19795_[Bacillus_cereus]	CPTF_zcontrol	89141.46667	7799.416976	8.749482443
UIJ65186.1	bifunctional_homocysteine_S-methyltransferase/methylenetetrahydrofolate_reductase_[Bacillus_cereus]	CPTF_Al	273830	237163.6849	86.60982541
UIJ65186.1	bifunctional_homocysteine_S-methyltransferase/methylenetetrahydrofolate_reductase_[Bacillus_cereus]	CPTF_Cd	246959.6667	427746.6901	173.2050808
UIJ65186.1	bifunctional_homocysteine_S-methyltransferase/methylenetetrahydrofolate_reductase_[Bacillus_cereus]	CPTF_Co	361826.6667	626702.1702	173.2050808
UIJ65186.1	bifunctional_homocysteine_S-methyltransferase/methylenetetrahydrofolate_reductase_[Bacillus_cereus]	CPTF_Cu	698944.3333	694315.7353	99.33777301
UIJ65186.1	bifunctional_homocysteine_S-methyltransferase/methylenetetrahydrofolate_reductase_[Bacillus_cereus]	CPTF_Fe	292758.3333	507072.3077	173.2050808
UIJ65186.1	bifunctional_homocysteine_S-methyltransferase/methylenetetrahydrofolate_reductase_[Bacillus_cereus]	CPTF_Mn	711700.6667	689888.1097	96.93515013
UIJ65186.1	bifunctional_homocysteine_S-methyltransferase/methylenetetrahydrofolate_reductase_[Bacillus_cereus]	CPTF_Ni	394472.6667	347679.5865	88.13781433
UIJ65186.1	bifunctional_homocysteine_S-methyltransferase/methylenetetrahydrofolate_reductase_[Bacillus_cereus]	CPTF_U	785573	774670.4617	98.61215466
UIJ65186.1	bifunctional_homocysteine_S-methyltransferase/methylenetetrahydrofolate_reductase_[Bacillus_cereus]	CPTF_metals_mix	0	0	0
UIJ65186.1	bifunctional_homocysteine_S-methyltransferase/methylenetetrahydrofolate_reductase_[Bacillus_cereus]	CPTF_zcontrol	570784	17937.39839	3.142589559
UIJ65187.1	cystathionine_gamma-synthase/O-acetylhomoserine_thiolyase_[Bacillus_cereus]	CPTF_Al	29074.56667	50358.62667	173.2050808
UIJ65187.1	cystathionine_gamma-synthase/O-acetylhomoserine_thiolyase_[Bacillus_cereus]	CPTF_Cd	21105.5	36555.79832	173.2050808
UIJ65187.1	cystathionine_gamma-synthase/O-acetylhomoserine_thiolyase_[Bacillus_cereus]	CPTF_Co	48121.73333	44985.46526	93.48263693

UIJ65187.1	cystathionine_gamma-synthase/O-acetylhomoserine_thiolyase_[Bacillus_cereus]	CPTF_Cu	0	0	0
UIJ65187.1	cystathionine_gamma-synthase/O-acetylhomoserine_thiolyase_[Bacillus_cereus]	CPTF_Fe	59003.4	16267.90324	27.57112852
UIJ65187.1	cystathionine_gamma-synthase/O-acetylhomoserine_thiolyase_[Bacillus_cereus]	CPTF_Mn	28268.4	48962.30505	173.2050808
UIJ65187.1	cystathionine_gamma-synthase/O-acetylhomoserine_thiolyase_[Bacillus_cereus]	CPTF_Ni	19092.63333	33069.41098	173.2050808
UIJ65187.1	cystathionine_gamma-synthase/O-acetylhomoserine_thiolyase_[Bacillus_cereus]	CPTF_U	56720.9	54890.63892	96.77321574
UIJ65187.1	cystathionine_gamma-synthase/O-acetylhomoserine_thiolyase_[Bacillus_cereus]	CPTF_metals_mix	3694.8	6399.581324	173.2050808
UIJ65187.1	cystathionine_gamma-synthase/O-acetylhomoserine_thiolyase_[Bacillus_cereus]	CPTF_zcontrol	25105.93333	43484.7521	173.2050808
UIJ65190.1	metalloregulator_ArsR/SmtB_family_transcription_factor_[Bacillus_cereus]	CPTF_Al	2040767.167	113686.4076	5.570768165
UIJ65190.1	metalloregulator_ArsR/SmtB_family_transcription_factor_[Bacillus_cereus]	CPTF_Cd	2088054.1	316371.0555	15.1514779
UIJ65190.1	metalloregulator_ArsR/SmtB_family_transcription_factor_[Bacillus_cereus]	CPTF_Co	2271279.667	131324.1024	5.781943296
UIJ65190.1	metalloregulator_ArsR/SmtB_family_transcription_factor_[Bacillus_cereus]	CPTF_Cu	1758968	101966.505	5.796950543
UIJ65190.1	metalloregulator_ArsR/SmtB_family_transcription_factor_[Bacillus_cereus]	CPTF_Fe	2133331.133	370310.2526	17.35831099
UIJ65190.1	metalloregulator_ArsR/SmtB_family_transcription_factor_[Bacillus_cereus]	CPTF_Mn	2325280.033	485515.1958	20.87985915
UIJ65190.1	metalloregulator_ArsR/SmtB_family_transcription_factor_[Bacillus_cereus]	CPTF_Ni	2206890.667	386583.0961	17.51709325
UIJ65190.1	metalloregulator_ArsR/SmtB_family_transcription_factor_[Bacillus_cereus]	CPTF_U	863333	9097.38979	1.05375212
UIJ65190.1	metalloregulator_ArsR/SmtB_family_transcription_factor_[Bacillus_cereus]	CPTF_metals_mix	1126068	94458.12356	8.388314344
UIJ65190.1	metalloregulator_ArsR/SmtB_family_transcription_factor_[Bacillus_cereus]	CPTF_zcontrol	2028286	242126.5626	11.93749612
UIJ65192.1	MBL_fold_metallo-hydrolase_[Bacillus_cereus]	CPTF_Al	186874.3333	24094.86174	12.8936175
UIJ65192.1	MBL_fold_metallo-hydrolase_[Bacillus_cereus]	CPTF_Cd	193808.3333	16402.44132	8.463228098
UIJ65192.1	MBL_fold_metallo-hydrolase_[Bacillus_cereus]	CPTF_Co	219019.6667	19509.86895	8.907816017
UIJ65192.1	MBL_fold_metallo-hydrolase_[Bacillus_cereus]	CPTF_Cu	202516	3820.176305	1.886357772
UIJ65192.1	MBL_fold_metallo-hydrolase_[Bacillus_cereus]	CPTF_Fe	219196.6667	8898.713915	4.059693995
UIJ65192.1	MBL_fold_metallo-hydrolase_[Bacillus_cereus]	CPTF_Mn	226768	52847.80655	23.30479016
UIJ65192.1	MBL_fold_metallo-hydrolase_[Bacillus_cereus]	CPTF_Ni	193587	37764.80212	19.5079226
UIJ65192.1	MBL_fold_metallo-hydrolase_[Bacillus_cereus]	CPTF_U	224034.6667	20662.2305	9.22278271
UIJ65192.1	MBL_fold_metallo-hydrolase_[Bacillus_cereus]	CPTF_metals_mix	146600.6667	26015.27217	17.74567113
UIJ65192.1	MBL_fold_metallo-hydrolase_[Bacillus_cereus]	CPTF_zcontrol	201268.6667	40763.32883	20.25319167
UIJ65194.1	glucokinase_[Bacillus_cereus]	CPTF_Al	2800016.767	95690.64805	3.41750268
UIJ65194.1	glucokinase_[Bacillus_cereus]	CPTF_Cd	2984120.833	74021.83044	2.480523899
UIJ65194.1	glucokinase_[Bacillus_cereus]	CPTF_Co	2666500.333	387312.7231	14.52513312
UIJ65194.1	glucokinase_[Bacillus_cereus]	CPTF_Cu	2468267.5	390938.0692	15.83856163
UIJ65194.1	glucokinase_[Bacillus_cereus]	CPTF_Fe	2743596.133	151362.5825	5.516941091
UIJ65194.1	glucokinase_[Bacillus_cereus]	CPTF_Mn	2530180	512996.3589	20.27509343
UIJ65194.1	glucokinase_[Bacillus_cereus]	CPTF_Ni	2100987.167	390650.9605	18.59368618
UIJ65194.1	glucokinase_[Bacillus_cereus]	CPTF_U	2216078.4	586542.1521	26.46757227
UIJ65194.1	glucokinase_[Bacillus_cereus]	CPTF_metals_mix	2997928.467	251895.2353	8.402309732
UIJ65194.1	glucokinase_[Bacillus_cereus]	CPTF_zcontrol	2819426.8	165767.327	5.879469083
UIJ65199.1	phosphate_signaling_complex_protein_PhoU_[Bacillus_cereus]	CPTF_Al	163302.6667	26811.2279	16.41812007
UIJ65199.1	phosphate_signaling_complex_protein_PhoU_[Bacillus_cereus]	CPTF_Cd	0	0	0
UIJ65199.1	phosphate_signaling_complex_protein_PhoU_[Bacillus_cereus]	CPTF_Co	61619	106727.2387	173.2050808
UIJ65199.1	phosphate_signaling_complex_protein_PhoU_[Bacillus_cereus]	CPTF_Cu	116450.5	23608.91977	20.27378137
UIJ65199.1	phosphate_signaling_complex_protein_PhoU_[Bacillus_cereus]	CPTF_Fe	0	0	0
UIJ65199.1	phosphate_signaling_complex_protein_PhoU_[Bacillus_cereus]	CPTF_Mn	0	0	0
UIJ65199.1	phosphate_signaling_complex_protein_PhoU_[Bacillus_cereus]	CPTF_Ni	27689.33333	47959.33216	173.2050808
UIJ65199.1	phosphate_signaling_complex_protein_PhoU_[Bacillus_cereus]	CPTF_U	65309	113118.5062	173.2050808
UIJ65199.1	phosphate_signaling_complex_protein_PhoU_[Bacillus_cereus]	CPTF_metals_mix	0	0	0
UIJ65199.1	phosphate_signaling_complex_protein_PhoU_[Bacillus_cereus]	CPTF_zcontrol	47747.33333	82700.80726	173.2050808
UIJ65204.1	penicillin-binding_protein_2_[Bacillus_cereus]	CPTF_Al	62533.8	68503.17348	109.5458352
UIJ65204.1	penicillin-binding_protein_2_[Bacillus_cereus]	CPTF_Cd	26366	45667.25159	173.2050808
UIJ65204.1	penicillin-binding_protein_2_[Bacillus_cereus]	CPTF_Co	24705.66667	42791.4699	173.2050808
UIJ65204.1	penicillin-binding_protein_2_[Bacillus_cereus]	CPTF_Cu	47942.33333	83038.55717	173.2050808
UIJ65204.1	penicillin-binding_protein_2_[Bacillus_cereus]	CPTF_Fe	36499.33333	63218.69978	173.2050808
UIJ65204.1	penicillin-binding_protein_2_[Bacillus_cereus]	CPTF_Mn	0	0	0
UIJ65204.1	penicillin-binding_protein_2_[Bacillus_cereus]	CPTF_Ni	0	0	0
UIJ65204.1	penicillin-binding_protein_2_[Bacillus_cereus]	CPTF_U	22591.06667	39128.87526	173.2050808
UIJ65204.1	penicillin-binding_protein_2_[Bacillus_cereus]	CPTF_metals_mix	74370.26667	43659.98612	58.70623849
UIJ65204.1	penicillin-binding_protein_2_[Bacillus_cereus]	CPTF_zcontrol	0	0	0
UIJ65206.1	superoxide_dismutase_[Mn]_[Bacillus_cereus]	CPTF_Al	18108266.07	1076546.45	5.945055402
UIJ65206.1	superoxide_dismutase_[Mn]_[Bacillus_cereus]	CPTF_Cd	21652885.6	1251987.577	5.782081891
UIJ65206.1	superoxide_dismutase_[Mn]_[Bacillus_cereus]	CPTF_Co	16051875.17	738660.3675	4.60170765
UIJ65206.1	superoxide_dismutase_[Mn]_[Bacillus_cereus]	CPTF_Cu	16467061.07	449196.2553	2.727847145

UIJ65206.1	superoxide_dismutase_[Mn]_[Bacillus_cereus]	CPTF_Fe	17376375.47	1137645.421	6.547081256
UIJ65206.1	superoxide_dismutase_[Mn]_[Bacillus_cereus]	CPTF_Mn	16691867.47	874796.1334	5.24085238
UIJ65206.1	superoxide_dismutase_[Mn]_[Bacillus_cereus]	CPTF_Ni	13343695	706774.8244	5.296694989
UIJ65206.1	superoxide_dismutase_[Mn]_[Bacillus_cereus]	CPTF_U	15316712.1	658719.2524	4.300657009
UIJ65206.1	superoxide_dismutase_[Mn]_[Bacillus_cereus]	CPTF_metals_mix	19106882.67	212031.5337	1.109712858
UIJ65206.1	superoxide_dismutase_[Mn]_[Bacillus_cereus]	CPTF_zcontrol	16502492.9	1154581.58	6.996407071
UIJ65207.1	DUF1189_domain-containing_protein_[Bacillus_cereus]	CPTF_Al	81470.36667	30507.32174	37.44591192
UIJ65207.1	DUF1189_domain-containing_protein_[Bacillus_cereus]	CPTF_Cd	103731.3	19984.77599	19.26590719
UIJ65207.1	DUF1189_domain-containing_protein_[Bacillus_cereus]	CPTF_Co	55495.73333	28704.40798	51.72363037
UIJ65207.1	DUF1189_domain-containing_protein_[Bacillus_cereus]	CPTF_Cu	81496.1	6961.914526	8.542635201
UIJ65207.1	DUF1189_domain-containing_protein_[Bacillus_cereus]	CPTF_Fe	77399.93333	12291.66694	15.88072032
UIJ65207.1	DUF1189_domain-containing_protein_[Bacillus_cereus]	CPTF_Mn	57870.4	51141.08447	88.37174872
UIJ65207.1	DUF1189_domain-containing_protein_[Bacillus_cereus]	CPTF_Ni	18003.96667	31183.785	173.2050808
UIJ65207.1	DUF1189_domain-containing_protein_[Bacillus_cereus]	CPTF_U	16633.56667	28810.18258	173.2050808
UIJ65207.1	DUF1189_domain-containing_protein_[Bacillus_cereus]	CPTF_metals_mix	110213	2716.168809	2.464472258
UIJ65207.1	DUF1189_domain-containing_protein_[Bacillus_cereus]	CPTF_zcontrol	46329.93333	49384.29944	106.5926408
UIJ65211.1	metal_ABC_transporter_ATP-binding_protein_[Bacillus_cereus]	CPTF_Al	0	0	0
UIJ65211.1	metal_ABC_transporter_ATP-binding_protein_[Bacillus_cereus]	CPTF_Cd	0	0	0
UIJ65211.1	metal_ABC_transporter_ATP-binding_protein_[Bacillus_cereus]	CPTF_Co	68586.66667	59417.4977	86.63126609
UIJ65211.1	metal_ABC_transporter_ATP-binding_protein_[Bacillus_cereus]	CPTF_Cu	104885.6667	90839.77254	86.60837598
UIJ65211.1	metal_ABC_transporter_ATP-binding_protein_[Bacillus_cereus]	CPTF_Fe	0	0	0
UIJ65211.1	metal_ABC_transporter_ATP-binding_protein_[Bacillus_cereus]	CPTF_Mn	47495.93333	42071.5051	88.57917331
UIJ65211.1	metal_ABC_transporter_ATP-binding_protein_[Bacillus_cereus]	CPTF_Ni	0	0	0
UIJ65211.1	metal_ABC_transporter_ATP-binding_protein_[Bacillus_cereus]	CPTF_U	90523.33333	81495.37764	90.02692968
UIJ65211.1	metal_ABC_transporter_ATP-binding_protein_[Bacillus_cereus]	CPTF_metals_mix	0	0	0
UIJ65211.1	metal_ABC_transporter_ATP-binding_protein_[Bacillus_cereus]	CPTF_zcontrol	20568.66667	35625.97571	173.2050808
UIJ65214.1	deoxyribonuclease_IV_[Bacillus_cereus]	CPTF_Al	107238	185741.6645	173.2050808
UIJ65214.1	deoxyribonuclease_IV_[Bacillus_cereus]	CPTF_Cd	0	0	0
UIJ65214.1	deoxyribonuclease_IV_[Bacillus_cereus]	CPTF_Co	0	0	0
UIJ65214.1	deoxyribonuclease_IV_[Bacillus_cereus]	CPTF_Cu	137164.6667	237576.1717	173.2050808
UIJ65214.1	deoxyribonuclease_IV_[Bacillus_cereus]	CPTF_Fe	0	0	0
UIJ65214.1	deoxyribonuclease_IV_[Bacillus_cereus]	CPTF_Mn	0	0	0
UIJ65214.1	deoxyribonuclease_IV_[Bacillus_cereus]	CPTF_Ni	0	0	0
UIJ65214.1	deoxyribonuclease_IV_[Bacillus_cereus]	CPTF_U	0	0	0
UIJ65214.1	deoxyribonuclease_IV_[Bacillus_cereus]	CPTF_metals_mix	375320.3333	32246.06925	8.591612655
UIJ65214.1	deoxyribonuclease_IV_[Bacillus_cereus]	CPTF_zcontrol	105135.3333	182099.739	173.2050808
UIJ65215.1	DEAD/DEAH_box_helicase_[Bacillus_cereus]	CPTF_Al	568600.4333	91187.47137	16.03717937
UIJ65215.1	DEAD/DEAH_box_helicase_[Bacillus_cereus]	CPTF_Cd	560331.5333	117190.7937	20.91454554
UIJ65215.1	DEAD/DEAH_box_helicase_[Bacillus_cereus]	CPTF_Co	661659.6	67117.56532	10.14382098
UIJ65215.1	DEAD/DEAH_box_helicase_[Bacillus_cereus]	CPTF_Cu	610068.9333	64118.56821	10.510053
UIJ65215.1	DEAD/DEAH_box_helicase_[Bacillus_cereus]	CPTF_Fe	583030.1667	60882.1281	10.44236329
UIJ65215.1	DEAD/DEAH_box_helicase_[Bacillus_cereus]	CPTF_Mn	582703.3	46803.59581	8.032148746
UIJ65215.1	DEAD/DEAH_box_helicase_[Bacillus_cereus]	CPTF_Ni	541878	45105.2507	8.323875614
UIJ65215.1	DEAD/DEAH_box_helicase_[Bacillus_cereus]	CPTF_U	642663.3333	245211.813	38.15556299
UIJ65215.1	DEAD/DEAH_box_helicase_[Bacillus_cereus]	CPTF_metals_mix	469674.5	141884.096	30.20902689
UIJ65215.1	DEAD/DEAH_box_helicase_[Bacillus_cereus]	CPTF_zcontrol	684958.5333	114000.0778	16.64335464
UIJ65217.1	4-hydroxy-3-methylbut-2-enyl_diphosphate_reductase_[Bacillus_cereus]	CPTF_Al	0	0	0
UIJ65217.1	4-hydroxy-3-methylbut-2-enyl_diphosphate_reductase_[Bacillus_cereus]	CPTF_Cd	0	0	0
UIJ65217.1	4-hydroxy-3-methylbut-2-enyl_diphosphate_reductase_[Bacillus_cereus]	CPTF_Co	43943.26667	38700.96533	88.07029669
UIJ65217.1	4-hydroxy-3-methylbut-2-enyl_diphosphate_reductase_[Bacillus_cereus]	CPTF_Cu	0	0	0
UIJ65217.1	4-hydroxy-3-methylbut-2-enyl_diphosphate_reductase_[Bacillus_cereus]	CPTF_Fe	6195.833333	10731.49813	173.2050808
UIJ65217.1	4-hydroxy-3-methylbut-2-enyl_diphosphate_reductase_[Bacillus_cereus]	CPTF_Mn	0	0	0
UIJ65217.1	4-hydroxy-3-methylbut-2-enyl_diphosphate_reductase_[Bacillus_cereus]	CPTF_Ni	0	0	0
UIJ65217.1	4-hydroxy-3-methylbut-2-enyl_diphosphate_reductase_[Bacillus_cereus]	CPTF_U	0	0	0
UIJ65217.1	4-hydroxy-3-methylbut-2-enyl_diphosphate_reductase_[Bacillus_cereus]	CPTF_metals_mix	49107.3	44674.16194	90.97254775
UIJ65217.1	4-hydroxy-3-methylbut-2-enyl_diphosphate_reductase_[Bacillus_cereus]	CPTF_zcontrol	0	0	0
UIJ65218.1	Nif3-like_dinuclear_metal_center_hexameric_protein_[Bacillus_cereus]	CPTF_Al	14605.4	25297.29486	173.2050808
UIJ65218.1	Nif3-like_dinuclear_metal_center_hexameric_protein_[Bacillus_cereus]	CPTF_Cd	0	0	0
UIJ65218.1	Nif3-like_dinuclear_metal_center_hexameric_protein_[Bacillus_cereus]	CPTF_Co	0	0	0
UIJ65218.1	Nif3-like_dinuclear_metal_center_hexameric_protein_[Bacillus_cereus]	CPTF_Cu	41535	71940.73029	173.2050808
UIJ65218.1	Nif3-like_dinuclear_metal_center_hexameric_protein_[Bacillus_cereus]	CPTF_Fe	41787	72377.2071	173.2050808

UIJ65218.1	Nif3-like_dinuclear_metal_center_hexameric_protein_[Bacillus_cereus]	CPTF_Mn	47517.66667	82303.01292	173.2050808
UIJ65218.1	Nif3-like_dinuclear_metal_center_hexameric_protein_[Bacillus_cereus]	CPTF_Ni	87450.66667	75927.05408	86.82272757
UIJ65218.1	Nif3-like_dinuclear_metal_center_hexameric_protein_[Bacillus_cereus]	CPTF_U	43602	75520.87931	173.2050808
UIJ65218.1	Nif3-like_dinuclear_metal_center_hexameric_protein_[Bacillus_cereus]	CPTF_metals_mix	0	0	0
UIJ65218.1	Nif3-like_dinuclear_metal_center_hexameric_protein_[Bacillus_cereus]	CPTF_zcontrol	0	0	0
UIJ65219.1	tRNA_(adenine(22)-N(1))-methyltransferase_TrmK_[Bacillus_cereus]	CPTF_Al	0	0	0
UIJ65219.1	tRNA_(adenine(22)-N(1))-methyltransferase_TrmK_[Bacillus_cereus]	CPTF_Cd	55332.1	49045.29318	88.63804768
UIJ65219.1	tRNA_(adenine(22)-N(1))-methyltransferase_TrmK_[Bacillus_cereus]	CPTF_Co	0	0	0
UIJ65219.1	tRNA_(adenine(22)-N(1))-methyltransferase_TrmK_[Bacillus_cereus]	CPTF_Cu	8639.2	14963.53334	173.2050808
UIJ65219.1	tRNA_(adenine(22)-N(1))-methyltransferase_TrmK_[Bacillus_cereus]	CPTF_Fe	9071.533333	15712.35664	173.2050808
UIJ65219.1	tRNA_(adenine(22)-N(1))-methyltransferase_TrmK_[Bacillus_cereus]	CPTF_Mn	0	0	0
UIJ65219.1	tRNA_(adenine(22)-N(1))-methyltransferase_TrmK_[Bacillus_cereus]	CPTF_Ni	0	0	0
UIJ65219.1	tRNA_(adenine(22)-N(1))-methyltransferase_TrmK_[Bacillus_cereus]	CPTF_U	0	0	0
UIJ65219.1	tRNA_(adenine(22)-N(1))-methyltransferase_TrmK_[Bacillus_cereus]	CPTF_metals_mix	77737.23333	32567.40697	41.89421925
UIJ65219.1	tRNA_(adenine(22)-N(1))-methyltransferase_TrmK_[Bacillus_cereus]	CPTF_zcontrol	0	0	0
UIJ65221.1	RNA_polymerase_sigma_factor_RpoD_[Bacillus_cereus]	CPTF_Al	5809411.3	1089493.867	18.75394615
UIJ65221.1	RNA_polymerase_sigma_factor_RpoD_[Bacillus_cereus]	CPTF_Cd	5752770.1	913078.8809	15.87198628
UIJ65221.1	RNA_polymerase_sigma_factor_RpoD_[Bacillus_cereus]	CPTF_Co	5698618.833	231683.5755	4.065609268
UIJ65221.1	RNA_polymerase_sigma_factor_RpoD_[Bacillus_cereus]	CPTF_Cu	4700498.167	385762.6698	8.206846511
UIJ65221.1	RNA_polymerase_sigma_factor_RpoD_[Bacillus_cereus]	CPTF_Fe	5248125.2	501614.9635	9.557983935
UIJ65221.1	RNA_polymerase_sigma_factor_RpoD_[Bacillus_cereus]	CPTF_Mn	6092524.3	1129022.796	18.53128097
UIJ65221.1	RNA_polymerase_sigma_factor_RpoD_[Bacillus_cereus]	CPTF_Ni	4646947.167	327280.393	7.042911857
UIJ65221.1	RNA_polymerase_sigma_factor_RpoD_[Bacillus_cereus]	CPTF_U	5988340	579681.0563	9.680162721
UIJ65221.1	RNA_polymerase_sigma_factor_RpoD_[Bacillus_cereus]	CPTF_metals_mix	6722283.2	1116028.488	16.60192609
UIJ65221.1	RNA_polymerase_sigma_factor_RpoD_[Bacillus_cereus]	CPTF_zcontrol	5631991.87	322301.221	5.722686191
UIJ65224.1	kinase/pyrophosphorylase_[Bacillus_cereus]	CPTF_Al	1889894.367	246892.6618	13.06383395
UIJ65224.1	kinase/pyrophosphorylase_[Bacillus_cereus]	CPTF_Cd	2169798.333	102168.9406	4.708683706
UIJ65224.1	kinase/pyrophosphorylase_[Bacillus_cereus]	CPTF_Co	1704914.333	61260.96905	3.593199251
UIJ65224.1	kinase/pyrophosphorylase_[Bacillus_cereus]	CPTF_Cu	1695216	125903.8887	7.427011585
UIJ65224.1	kinase/pyrophosphorylase_[Bacillus_cereus]	CPTF_Fe	1752124.567	138646.3256	7.913040447
UIJ65224.1	kinase/pyrophosphorylase_[Bacillus_cereus]	CPTF_Mn	1795408.1	173375.7911	9.656622977
UIJ65224.1	kinase/pyrophosphorylase_[Bacillus_cereus]	CPTF_Ni	1404194.133	116927.4906	8.327017458
UIJ65224.1	kinase/pyrophosphorylase_[Bacillus_cereus]	CPTF_U	1391925.033	51188.26736	3.677516111
UIJ65224.1	kinase/pyrophosphorylase_[Bacillus_cereus]	CPTF_metals_mix	2172898.033	58409.56798	2.688095211
UIJ65224.1	kinase/pyrophosphorylase_[Bacillus_cereus]	CPTF_zcontrol	1623402.533	267778.0222	16.4948629
UIJ65227.1	GTPase_Era_[Bacillus_cereus]	CPTF_Al	20772.13333	22772.09111	109.6280808
UIJ65227.1	GTPase_Era_[Bacillus_cereus]	CPTF_Cd	52015.16667	26962.26952	51.83539965
UIJ65227.1	GTPase_Era_[Bacillus_cereus]	CPTF_Co	0	0	0
UIJ65227.1	GTPase_Era_[Bacillus_cereus]	CPTF_Cu	0	0	0
UIJ65227.1	GTPase_Era_[Bacillus_cereus]	CPTF_Fe	0	0	0
UIJ65227.1	GTPase_Era_[Bacillus_cereus]	CPTF_Mn	19838.5	34361.28995	173.2050808
UIJ65227.1	GTPase_Era_[Bacillus_cereus]	CPTF_Ni	0	0	0
UIJ65227.1	GTPase_Era_[Bacillus_cereus]	CPTF_U	14110.86667	24440.73801	173.2050808
UIJ65227.1	GTPase_Era_[Bacillus_cereus]	CPTF_metals_mix	207995.4667	29201.45273	14.03946596
UIJ65227.1	GTPase_Era_[Bacillus_cereus]	CPTF_zcontrol	13139.63333	22758.51253	173.2050808
UIJ65230.1	rRNA_maturation_RNase_YbeY_[Bacillus_cereus]	CPTF_Al	28444.33333	49267.03052	173.2050808
UIJ65230.1	rRNA_maturation_RNase_YbeY_[Bacillus_cereus]	CPTF_Cd	0	0	0
UIJ65230.1	rRNA_maturation_RNase_YbeY_[Bacillus_cereus]	CPTF_Co	0	0	0
UIJ65230.1	rRNA_maturation_RNase_YbeY_[Bacillus_cereus]	CPTF_Cu	33968	58834.30183	173.2050808
UIJ65230.1	rRNA_maturation_RNase_YbeY_[Bacillus_cereus]	CPTF_Fe	34404.66667	59590.63068	173.2050808
UIJ65230.1	rRNA_maturation_RNase_YbeY_[Bacillus_cereus]	CPTF_Mn	0	0	0
UIJ65230.1	rRNA_maturation_RNase_YbeY_[Bacillus_cereus]	CPTF_Ni	41117.33333	71217.31041	173.2050808
UIJ65230.1	rRNA_maturation_RNase_YbeY_[Bacillus_cereus]	CPTF_U	35824.33333	62049.56548	173.2050808
UIJ65230.1	rRNA_maturation_RNase_YbeY_[Bacillus_cereus]	CPTF_metals_mix	14115.7	24449.10958	173.2050808
UIJ65230.1	rRNA_maturation_RNase_YbeY_[Bacillus_cereus]	CPTF_zcontrol	0	0	0
UIJ65232.1	PhoH_family_protein_[Bacillus_cereus]	CPTF_Al	48947.06667	10368.58037	21.18325178
UIJ65232.1	PhoH_family_protein_[Bacillus_cereus]	CPTF_Cd	0	0	0
UIJ65232.1	PhoH_family_protein_[Bacillus_cereus]	CPTF_Co	37662.63333	33660.56704	89.37390739
UIJ65232.1	PhoH_family_protein_[Bacillus_cereus]	CPTF_Cu	78984.13333	21802.5387	27.60369428
UIJ65232.1	PhoH_family_protein_[Bacillus_cereus]	CPTF_Fe	44762.96667	40601.81603	90.70403295
UIJ65232.1	PhoH_family_protein_[Bacillus_cereus]	CPTF_Mn	0	0	0

UIJ65232.1	PhoH_family_protein_[Bacillus_cereus]	CPTF_Ni	114868.4333	17423.17503	15.16793998
UIJ65232.1	PhoH_family_protein_[Bacillus_cereus]	CPTF_U	71633	62355.93147	87.04916933
UIJ65232.1	PhoH_family_protein_[Bacillus_cereus]	CPTF_metals_mix	361690.9	76831.95055	21.24243395
UIJ65232.1	PhoH_family_protein_[Bacillus_cereus]	CPTF_zcontrol	10474.5	18142.36618	173.2050808
UIJ65235.1	GatB/YqeY_domain-containing_protein_[Bacillus_cereus]	CPTF_Al	1772463.667	317092.884	17.88995115
UIJ65235.1	GatB/YqeY_domain-containing_protein_[Bacillus_cereus]	CPTF_Cd	1963301.333	126282.1672	6.432133726
UIJ65235.1	GatB/YqeY_domain-containing_protein_[Bacillus_cereus]	CPTF_Co	1902168.667	365889.4951	19.23538651
UIJ65235.1	GatB/YqeY_domain-containing_protein_[Bacillus_cereus]	CPTF_Cu	1656880.333	260850.4605	15.74347014
UIJ65235.1	GatB/YqeY_domain-containing_protein_[Bacillus_cereus]	CPTF_Fe	1906416.3	570886.877	29.94555161
UIJ65235.1	GatB/YqeY_domain-containing_protein_[Bacillus_cereus]	CPTF_Mn	2037514	270050.3044	13.2539116
UIJ65235.1	GatB/YqeY_domain-containing_protein_[Bacillus_cereus]	CPTF_Ni	1549168	536509.2216	34.63208778
UIJ65235.1	GatB/YqeY_domain-containing_protein_[Bacillus_cereus]	CPTF_U	1205651	321654.1868	26.67888027
UIJ65235.1	GatB/YqeY_domain-containing_protein_[Bacillus_cereus]	CPTF_metals_mix	1901716.667	152574.3057	8.022977784
UIJ65235.1	GatB/YqeY_domain-containing_protein_[Bacillus_cereus]	CPTF_zcontrol	1816576.667	268179.5357	14.76290765
UIJ65236.1	30S_ribosomal_protein_S21_[Bacillus_cereus]	CPTF_Al	8922037	162792.3466	1.824609633
UIJ65236.1	30S_ribosomal_protein_S21_[Bacillus_cereus]	CPTF_Cd	9146369	237851.8499	2.600505729
UIJ65236.1	30S_ribosomal_protein_S21_[Bacillus_cereus]	CPTF_Co	8937360.667	567565.0256	6.350476911
UIJ65236.1	30S_ribosomal_protein_S21_[Bacillus_cereus]	CPTF_Cu	8125509.333	412784.0575	5.080100713
UIJ65236.1	30S_ribosomal_protein_S21_[Bacillus_cereus]	CPTF_Fe	9166312.333	238978.8205	2.607142456
UIJ65236.1	30S_ribosomal_protein_S21_[Bacillus_cereus]	CPTF_Mn	9153692	277182.0392	3.028090077
UIJ65236.1	30S_ribosomal_protein_S21_[Bacillus_cereus]	CPTF_Ni	8431969.667	479585.7575	5.687707338
UIJ65236.1	30S_ribosomal_protein_S21_[Bacillus_cereus]	CPTF_U	9147564.333	450252.1749	4.922099025
UIJ65236.1	30S_ribosomal_protein_S21_[Bacillus_cereus]	CPTF_metals_mix	5997651	170344.5629	2.840187982
UIJ65236.1	30S_ribosomal_protein_S21_[Bacillus_cereus]	CPTF_zcontrol	9622176.667	414945.555	4.312387617
UIJ65237.1	tRNA_(N(6)-L-threonylcarbamoyladenosine(37)-C(2))-methylthiotransferase_MtaB_[Bacillus_cereus]	CPTF_Al	9394.9	16272.44413	173.2050808
UIJ65237.1	tRNA_(N(6)-L-threonylcarbamoyladenosine(37)-C(2))-methylthiotransferase_MtaB_[Bacillus_cereus]	CPTF_Cd	51177.46667	21433.78848	41.88130026
UIJ65237.1	tRNA_(N(6)-L-threonylcarbamoyladenosine(37)-C(2))-methylthiotransferase_MtaB_[Bacillus_cereus]	CPTF_Co	25114.6	24741.59152	98.51477435
UIJ65237.1	tRNA_(N(6)-L-threonylcarbamoyladenosine(37)-C(2))-methylthiotransferase_MtaB_[Bacillus_cereus]	CPTF_Cu	19525.13333	17400.8077	89.12004545
UIJ65237.1	tRNA_(N(6)-L-threonylcarbamoyladenosine(37)-C(2))-methylthiotransferase_MtaB_[Bacillus_cereus]	CPTF_Fe	61770	27254.41449	44.12241296
UIJ65237.1	tRNA_(N(6)-L-threonylcarbamoyladenosine(37)-C(2))-methylthiotransferase_MtaB_[Bacillus_cereus]	CPTF_Mn	0	0	0
UIJ65237.1	tRNA_(N(6)-L-threonylcarbamoyladenosine(37)-C(2))-methylthiotransferase_MtaB_[Bacillus_cereus]	CPTF_Ni	13817.86667	23933.24712	173.2050808
UIJ65237.1	tRNA_(N(6)-L-threonylcarbamoyladenosine(37)-C(2))-methylthiotransferase_MtaB_[Bacillus_cereus]	CPTF_U	56477.46667	33065.21307	58.54585027
UIJ65237.1	tRNA_(N(6)-L-threonylcarbamoyladenosine(37)-C(2))-methylthiotransferase_MtaB_[Bacillus_cereus]	CPTF_metals_mix	0	0	0
UIJ65237.1	tRNA_(N(6)-L-threonylcarbamoyladenosine(37)-C(2))-methylthiotransferase_MtaB_[Bacillus_cereus]	CPTF_zcontrol	27834.43333	26648.28564	95.73855994
UIJ65239.1	50S_ribosomal_protein_L11_methyltransferase_[Bacillus_cereus]	CPTF_Al	0	0	0
UIJ65239.1	50S_ribosomal_protein_L11_methyltransferase_[Bacillus_cereus]	CPTF_Cd	0	0	0
UIJ65239.1	50S_ribosomal_protein_L11_methyltransferase_[Bacillus_cereus]	CPTF_Co	0	0	0
UIJ65239.1	50S_ribosomal_protein_L11_methyltransferase_[Bacillus_cereus]	CPTF_Cu	0	0	0
UIJ65239.1	50S_ribosomal_protein_L11_methyltransferase_[Bacillus_cereus]	CPTF_Fe	0	0	0
UIJ65239.1	50S_ribosomal_protein_L11_methyltransferase_[Bacillus_cereus]	CPTF_Mn	49009.33333	84886.65538	173.2050808
UIJ65239.1	50S_ribosomal_protein_L11_methyltransferase_[Bacillus_cereus]	CPTF_Ni	0	0	0
UIJ65239.1	50S_ribosomal_protein_L11_methyltransferase_[Bacillus_cereus]	CPTF_U	0	0	0
UIJ65239.1	50S_ribosomal_protein_L11_methyltransferase_[Bacillus_cereus]	CPTF_metals_mix	117016.3333	23296.29499	19.90858398
UIJ65239.1	50S_ribosomal_protein_L11_methyltransferase_[Bacillus_cereus]	CPTF_zcontrol	0	0	0
UIJ65240.1	chaperone_protein_DnaJ_[Bacillus_cereus]	CPTF_Al	2285746.333	26584.35597	1.163049267
UIJ65240.1	chaperone_protein_DnaJ_[Bacillus_cereus]	CPTF_Cd	2325172	225867.9384	9.714031411
UIJ65240.1	chaperone_protein_DnaJ_[Bacillus_cereus]	CPTF_Co	2052845.333	13425.55181	0.653997239
UIJ65240.1	chaperone_protein_DnaJ_[Bacillus_cereus]	CPTF_Cu	2036923.633	135255.9142	6.640205455
UIJ65240.1	chaperone_protein_DnaJ_[Bacillus_cereus]	CPTF_Fe	2257810.333	185322.6751	8.208070997
UIJ65240.1	chaperone_protein_DnaJ_[Bacillus_cereus]	CPTF_Mn	2044022.333	512950.8889	25.09517047
UIJ65240.1	chaperone_protein_DnaJ_[Bacillus_cereus]	CPTF_Ni	2054831	220394.5003	10.72567527
UIJ65240.1	chaperone_protein_DnaJ_[Bacillus_cereus]	CPTF_U	2022784.267	256401.1431	12.67565441
UIJ65240.1	chaperone_protein_DnaJ_[Bacillus_cereus]	CPTF_metals_mix	1680214.333	260807.0017	15.52224598
UIJ65240.1	chaperone_protein_DnaJ_[Bacillus_cereus]	CPTF_zcontrol	2166435	172687.6827	7.971053029
UIJ65241.1	chaperone_protein_DnaK_[Bacillus_cereus]	CPTF_Al	46540033.63	4576838.995	9.834197867
UIJ65241.1	chaperone_protein_DnaK_[Bacillus_cereus]	CPTF_Cd	52763692.08	933556.231	1.769315592
UIJ65241.1	chaperone_protein_DnaK_[Bacillus_cereus]	CPTF_Co	45654191.73	2849664.356	6.241846035
UIJ65241.1	chaperone_protein_DnaK_[Bacillus_cereus]	CPTF_Cu	47569740.1	1177929.552	2.476216076
UIJ65241.1	chaperone_protein_DnaK_[Bacillus_cereus]	CPTF_Fe	48010709.2	5953502.099	12.40036275
UIJ65241.1	chaperone_protein_DnaK_[Bacillus_cereus]	CPTF_Mn	48147657	8321907.829	17.28413873
UIJ65241.1	chaperone_protein_DnaK_[Bacillus_cereus]	CPTF_Ni	32419016.33	2738386.598	8.446852829

UIJ65241.1	chaperone_protein_DnaK_[Bacillus_cereus]	CPTF_U	35075218.27	3197200.431	9.11526881
UIJ65241.1	chaperone_protein_DnaK_[Bacillus_cereus]	CPTF_metals_mix	59126879.33	2436534.815	4.120858131
UIJ65241.1	chaperone_protein_DnaK_[Bacillus_cereus]	CPTF_zcontrol	44522689.2	6552401.348	14.71699366
UIJ65242.1	nucleotide_exchange_factor_GrpE_[Bacillus_cereus]	CPTF_Al	2379626.2	521951.0495	21.93416132
UIJ65242.1	nucleotide_exchange_factor_GrpE_[Bacillus_cereus]	CPTF_Cd	2973078.3	163654.7803	5.504556683
UIJ65242.1	nucleotide_exchange_factor_GrpE_[Bacillus_cereus]	CPTF_Co	2398233.333	168568.5764	7.02886471
UIJ65242.1	nucleotide_exchange_factor_GrpE_[Bacillus_cereus]	CPTF_Cu	2846713.467	282960.5482	9.939902682
UIJ65242.1	nucleotide_exchange_factor_GrpE_[Bacillus_cereus]	CPTF_Fe	2234086.433	363507.2517	16.27095739
UIJ65242.1	nucleotide_exchange_factor_GrpE_[Bacillus_cereus]	CPTF_Mn	2240917.1	560706.7653	25.02130781
UIJ65242.1	nucleotide_exchange_factor_GrpE_[Bacillus_cereus]	CPTF_Ni	1704493	243790.1264	14.30279422
UIJ65242.1	nucleotide_exchange_factor_GrpE_[Bacillus_cereus]	CPTF_U	1708232	558868.2744	32.71618108
UIJ65242.1	nucleotide_exchange_factor_GrpE_[Bacillus_cereus]	CPTF_metals_mix	3917632.767	198046.6868	5.055264202
UIJ65242.1	nucleotide_exchange_factor_GrpE_[Bacillus_cereus]	CPTF_zcontrol	1876215.2	444897.5413	23.71250064
UIJ65247.1	elongation_factor_4_[Bacillus_cereus]	CPTF_Al	294675.3667	176767.6531	59.98725142
UIJ65247.1	elongation_factor_4_[Bacillus_cereus]	CPTF_Cd	336265.5333	166478.4093	49.50802054
UIJ65247.1	elongation_factor_4_[Bacillus_cereus]	CPTF_Co	439132.7	233931.3002	53.2712094
UIJ65247.1	elongation_factor_4_[Bacillus_cereus]	CPTF_Cu	306532.9333	164443.019	53.64611796
UIJ65247.1	elongation_factor_4_[Bacillus_cereus]	CPTF_Fe	228088.9333	123615.0815	54.1960014
UIJ65247.1	elongation_factor_4_[Bacillus_cereus]	CPTF_Mn	255491.4667	153590.0016	60.11551133
UIJ65247.1	elongation_factor_4_[Bacillus_cereus]	CPTF_Ni	444956.3	163736.611	36.79835772
UIJ65247.1	elongation_factor_4_[Bacillus_cereus]	CPTF_U	146371.4333	77796.03167	53.14973687
UIJ65247.1	elongation_factor_4_[Bacillus_cereus]	CPTF_metals_mix	1047381.967	46147.17226	4.405954439
UIJ65247.1	elongation_factor_4_[Bacillus_cereus]	CPTF_zcontrol	180899.7333	110286.8981	60.96576044
UIJ65250.1	30S_ribosomal_protein_S20_[Bacillus_cereus]	CPTF_Al	937489.4667	309819.7135	33.04780742
UIJ65250.1	30S_ribosomal_protein_S20_[Bacillus_cereus]	CPTF_Cd	1129239.533	172515.7814	15.27716453
UIJ65250.1	30S_ribosomal_protein_S20_[Bacillus_cereus]	CPTF_Co	998336.7667	72284.93485	7.240536187
UIJ65250.1	30S_ribosomal_protein_S20_[Bacillus_cereus]	CPTF_Cu	862159.3	170894.9754	19.82174006
UIJ65250.1	30S_ribosomal_protein_S20_[Bacillus_cereus]	CPTF_Fe	913129.3	382540.2807	41.89333107
UIJ65250.1	30S_ribosomal_protein_S20_[Bacillus_cereus]	CPTF_Mn	978077.5	633074.3739	64.72640193
UIJ65250.1	30S_ribosomal_protein_S20_[Bacillus_cereus]	CPTF_Ni	735783.5333	103914.1435	14.12292322
UIJ65250.1	30S_ribosomal_protein_S20_[Bacillus_cereus]	CPTF_U	425512.8	153568.5118	36.09022144
UIJ65250.1	30S_ribosomal_protein_S20_[Bacillus_cereus]	CPTF_metals_mix	1971380.2	113323.96	5.748457856
UIJ65250.1	30S_ribosomal_protein_S20_[Bacillus_cereus]	CPTF_zcontrol	488987.8	198451.732	40.58418882
UIJ65263.1	ribosome_biogenesis_GTPase_YqeH_[Bacillus_cereus]	CPTF_Al	125143.3333	22393.82811	17.89454341
UIJ65263.1	ribosome_biogenesis_GTPase_YqeH_[Bacillus_cereus]	CPTF_Cd	154692.3333	47689.57216	30.82865914
UIJ65263.1	ribosome_biogenesis_GTPase_YqeH_[Bacillus_cereus]	CPTF_Co	163834	9460.906246	5.774690386
UIJ65263.1	ribosome_biogenesis_GTPase_YqeH_[Bacillus_cereus]	CPTF_Cu	162110.0333	19176.40449	11.82925208
UIJ65263.1	ribosome_biogenesis_GTPase_YqeH_[Bacillus_cereus]	CPTF_Fe	169656.6667	34870.54761	20.55359703
UIJ65263.1	ribosome_biogenesis_GTPase_YqeH_[Bacillus_cereus]	CPTF_Mn	120979.3667	50321.85706	41.59540461
UIJ65263.1	ribosome_biogenesis_GTPase_YqeH_[Bacillus_cereus]	CPTF_Ni	121598.3333	15541.35877	12.78089785
UIJ65263.1	ribosome_biogenesis_GTPase_YqeH_[Bacillus_cereus]	CPTF_U	102088.6667	90053.68563	88.21124673
UIJ65263.1	ribosome_biogenesis_GTPase_YqeH_[Bacillus_cereus]	CPTF_metals_mix	186237	3712.09523	1.993210388
UIJ65263.1	ribosome_biogenesis_GTPase_YqeH_[Bacillus_cereus]	CPTF_zcontrol	128192	12746.28491	9.943120405
UIJ65266.1	phosphatidylserine_decarboxylase_[Bacillus_cereus]	CPTF_Al	4562.833333	7903.05916	173.2050808
UIJ65266.1	phosphatidylserine_decarboxylase_[Bacillus_cereus]	CPTF_Cd	0	0	0
UIJ65266.1	phosphatidylserine_decarboxylase_[Bacillus_cereus]	CPTF_Co	11785.13333	20412.44971	173.2050808
UIJ65266.1	phosphatidylserine_decarboxylase_[Bacillus_cereus]	CPTF_Cu	7747.233333	13418.60175	173.2050808
UIJ65266.1	phosphatidylserine_decarboxylase_[Bacillus_cereus]	CPTF_Fe	0	0	0
UIJ65266.1	phosphatidylserine_decarboxylase_[Bacillus_cereus]	CPTF_Mn	0	0	0
UIJ65266.1	phosphatidylserine_decarboxylase_[Bacillus_cereus]	CPTF_Ni	0	0	0
UIJ65266.1	phosphatidylserine_decarboxylase_[Bacillus_cereus]	CPTF_U	0	0	0
UIJ65266.1	phosphatidylserine_decarboxylase_[Bacillus_cereus]	CPTF_metals_mix	0	0	0
UIJ65266.1	phosphatidylserine_decarboxylase_[Bacillus_cereus]	CPTF_zcontrol	0	0	0
UIJ65271.1	GTP_pyrophosphokinase_family_protein_[Bacillus_cereus]	CPTF_Al	0	0	0
UIJ65271.1	GTP_pyrophosphokinase_family_protein_[Bacillus_cereus]	CPTF_Cd	0	0	0
UIJ65271.1	GTP_pyrophosphokinase_family_protein_[Bacillus_cereus]	CPTF_Co	0	0	0
UIJ65271.1	GTP_pyrophosphokinase_family_protein_[Bacillus_cereus]	CPTF_Cu	0	0	0
UIJ65271.1	GTP_pyrophosphokinase_family_protein_[Bacillus_cereus]	CPTF_Fe	0	0	0
UIJ65271.1	GTP_pyrophosphokinase_family_protein_[Bacillus_cereus]	CPTF_Mn	0	0	0
UIJ65271.1	GTP_pyrophosphokinase_family_protein_[Bacillus_cereus]	CPTF_Ni	0	0	0
UIJ65271.1	GTP_pyrophosphokinase_family_protein_[Bacillus_cereus]	CPTF_U	0	0	0

UIJ65271.1	GTP_pyrophosphokinase_family_protein_[Bacillus_cereus]	CPTF_metals_mix	11058.5	19153.88386	173.2050808
UIJ65271.1	GTP_pyrophosphokinase_family_protein_[Bacillus_cereus]	CPTF_zcontrol	0	0	0
UIJ65281.1	aromatic_amino_acid_hydroxylase_[Bacillus_cereus]	CPTF_Al	0	0	0
UIJ65281.1	aromatic_amino_acid_hydroxylase_[Bacillus_cereus]	CPTF_Cd	0	0	0
UIJ65281.1	aromatic_amino_acid_hydroxylase_[Bacillus_cereus]	CPTF_Co	0	0	0
UIJ65281.1	aromatic_amino_acid_hydroxylase_[Bacillus_cereus]	CPTF_Cu	12725.33333	22040.92388	173.2050808
UIJ65281.1	aromatic_amino_acid_hydroxylase_[Bacillus_cereus]	CPTF_Fe	0	0	0
UIJ65281.1	aromatic_amino_acid_hydroxylase_[Bacillus_cereus]	CPTF_Mn	0	0	0
UIJ65281.1	aromatic_amino_acid_hydroxylase_[Bacillus_cereus]	CPTF_Ni	0	0	0
UIJ65281.1	aromatic_amino_acid_hydroxylase_[Bacillus_cereus]	CPTF_U	0	0	0
UIJ65281.1	aromatic_amino_acid_hydroxylase_[Bacillus_cereus]	CPTF_metals_mix	30776.33333	33183.32668	107.820923
UIJ65281.1	aromatic_amino_acid_hydroxylase_[Bacillus_cereus]	CPTF_zcontrol	0	0	0
UIJ65286.1	enoyl-CoA_hydratase_[Bacillus_cereus]	CPTF_Al	346025.3333	44244.95456	12.78662291
UIJ65286.1	enoyl-CoA_hydratase_[Bacillus_cereus]	CPTF_Cd	387899.6667	31247.71746	8.055618538
UIJ65286.1	enoyl-CoA_hydratase_[Bacillus_cereus]	CPTF_Co	301067.6667	40767.75331	13.54105998
UIJ65286.1	enoyl-CoA_hydratase_[Bacillus_cereus]	CPTF_Cu	273361.6667	29774.31504	10.89191305
UIJ65286.1	enoyl-CoA_hydratase_[Bacillus_cereus]	CPTF_Fe	437898.3333	39495.86095	9.019413399
UIJ65286.1	enoyl-CoA_hydratase_[Bacillus_cereus]	CPTF_Mn	386770.6333	43488.81818	11.24408485
UIJ65286.1	enoyl-CoA_hydratase_[Bacillus_cereus]	CPTF_Ni	384704	23857.65204	6.201560692
UIJ65286.1	enoyl-CoA_hydratase_[Bacillus_cereus]	CPTF_U	373719.3333	18040.59313	4.827310636
UIJ65286.1	enoyl-CoA_hydratase_[Bacillus_cereus]	CPTF_metals_mix	206961.3667	18145.61619	8.767634501
UIJ65286.1	enoyl-CoA_hydratase_[Bacillus_cereus]	CPTF_zcontrol	329045.6667	27187.63234	8.262571155
UIJ65292.1	ABC_transporter_substrate-binding_protein_[Bacillus_cereus]	CPTF_Al	0	0	0
UIJ65292.1	ABC_transporter_substrate-binding_protein_[Bacillus_cereus]	CPTF_Cd	0	0	0
UIJ65292.1	ABC_transporter_substrate-binding_protein_[Bacillus_cereus]	CPTF_Co	0	0	0
UIJ65292.1	ABC_transporter_substrate-binding_protein_[Bacillus_cereus]	CPTF_Cu	0	0	0
UIJ65292.1	ABC_transporter_substrate-binding_protein_[Bacillus_cereus]	CPTF_Fe	0	0	0
UIJ65292.1	ABC_transporter_substrate-binding_protein_[Bacillus_cereus]	CPTF_Mn	0	0	0
UIJ65292.1	ABC_transporter_substrate-binding_protein_[Bacillus_cereus]	CPTF_Ni	0	0	0
UIJ65292.1	ABC_transporter_substrate-binding_protein_[Bacillus_cereus]	CPTF_U	0	0	0
UIJ65292.1	ABC_transporter_substrate-binding_protein_[Bacillus_cereus]	CPTF_metals_mix	293182	21365.87309	7.287580101
UIJ65292.1	ABC_transporter_substrate-binding_protein_[Bacillus_cereus]	CPTF_zcontrol	0	0	0
UIJ65294.1	bifunctional_acetaldehyde-CoA/alcohol_dehydrogenase_[Bacillus_cereus]	CPTF_Al	426765	265852.036	62.29471395
UIJ65294.1	bifunctional_acetaldehyde-CoA/alcohol_dehydrogenase_[Bacillus_cereus]	CPTF_Cd	513871.7333	195443.8959	38.0335954
UIJ65294.1	bifunctional_acetaldehyde-CoA/alcohol_dehydrogenase_[Bacillus_cereus]	CPTF_Co	1568106.133	774033.0061	49.36100878
UIJ65294.1	bifunctional_acetaldehyde-CoA/alcohol_dehydrogenase_[Bacillus_cereus]	CPTF_Cu	698586.1667	174994.8245	25.04985539
UIJ65294.1	bifunctional_acetaldehyde-CoA/alcohol_dehydrogenase_[Bacillus_cereus]	CPTF_Fe	737162.2	165317.9067	22.42625933
UIJ65294.1	bifunctional_acetaldehyde-CoA/alcohol_dehydrogenase_[Bacillus_cereus]	CPTF_Mn	557400.7	93130.48564	16.70799582
UIJ65294.1	bifunctional_acetaldehyde-CoA/alcohol_dehydrogenase_[Bacillus_cereus]	CPTF_Ni	769991.0667	332646.5327	43.20134961
UIJ65294.1	bifunctional_acetaldehyde-CoA/alcohol_dehydrogenase_[Bacillus_cereus]	CPTF_U	128768	223032.7184	173.2050808
UIJ65294.1	bifunctional_acetaldehyde-CoA/alcohol_dehydrogenase_[Bacillus_cereus]	CPTF_metals_mix	2220225.7	561938.0614	25.30995211
UIJ65294.1	bifunctional_acetaldehyde-CoA/alcohol_dehydrogenase_[Bacillus_cereus]	CPTF_zcontrol	50276.7	45477.61413	90.45465222
UIJ65295.1	bifunctional_cystathionine_gamma-lyase/homocysteine_desulfhydrase_[Bacillus_cereus]	CPTF_Al	11131145.13	618105.4154	5.552936449
UIJ65295.1	bifunctional_cystathionine_gamma-lyase/homocysteine_desulfhydrase_[Bacillus_cereus]	CPTF_Cd	12896233.67	233580.4342	1.811229854
UIJ65295.1	bifunctional_cystathionine_gamma-lyase/homocysteine_desulfhydrase_[Bacillus_cereus]	CPTF_Co	12407176.9	313786.8503	2.529075331
UIJ65295.1	bifunctional_cystathionine_gamma-lyase/homocysteine_desulfhydrase_[Bacillus_cereus]	CPTF_Cu	14388282	396582.383	2.756287255
UIJ65295.1	bifunctional_cystathionine_gamma-lyase/homocysteine_desulfhydrase_[Bacillus_cereus]	CPTF_Fe	11470448.33	205772.3125	1.793934348
UIJ65295.1	bifunctional_cystathionine_gamma-lyase/homocysteine_desulfhydrase_[Bacillus_cereus]	CPTF_Mn	11463119.1	705329.9038	6.153036514
UIJ65295.1	bifunctional_cystathionine_gamma-lyase/homocysteine_desulfhydrase_[Bacillus_cereus]	CPTF_Ni	11401895.77	209846.9011	1.840456231
UIJ65295.1	bifunctional_cystathionine_gamma-lyase/homocysteine_desulfhydrase_[Bacillus_cereus]	CPTF_U	10861802.6	313828.0138	2.889281138
UIJ65295.1	bifunctional_cystathionine_gamma-lyase/homocysteine_desulfhydrase_[Bacillus_cereus]	CPTF_metals_mix	20826819.33	1001274.17	4.807619224
UIJ65295.1	bifunctional_cystathionine_gamma-lyase/homocysteine_desulfhydrase_[Bacillus_cereus]	CPTF_zcontrol	11118628.93	984791.788	8.85713332
UIJ65296.1	O-acetylserine_dependent_cystathionine_beta-synthase_[Bacillus_cereus]	CPTF_Al	1183520.133	305245.2107	25.79129853
UIJ65296.1	O-acetylserine_dependent_cystathionine_beta-synthase_[Bacillus_cereus]	CPTF_Cd	1540118.033	168478.3843	10.93931638
UIJ65296.1	O-acetylserine_dependent_cystathionine_beta-synthase_[Bacillus_cereus]	CPTF_Co	1588462.833	459691.0828	28.93936661
UIJ65296.1	O-acetylserine_dependent_cystathionine_beta-synthase_[Bacillus_cereus]	CPTF_Cu	2088464.867	257084.6041	12.30974043
UIJ65296.1	O-acetylserine_dependent_cystathionine_beta-synthase_[Bacillus_cereus]	CPTF_Fe	1162685.933	330820.4256	28.45312015
UIJ65296.1	O-acetylserine_dependent_cystathionine_beta-synthase_[Bacillus_cereus]	CPTF_Mn	1109436.5	343651.5892	30.97532749
UIJ65296.1	O-acetylserine_dependent_cystathionine_beta-synthase_[Bacillus_cereus]	CPTF_Ni	938855.3333	423439.1405	45.10163872
UIJ65296.1	O-acetylserine_dependent_cystathionine_beta-synthase_[Bacillus_cereus]	CPTF_U	1199423.333	344933.1932	28.7582527
UIJ65296.1	O-acetylserine_dependent_cystathionine_beta-synthase_[Bacillus_cereus]	CPTF_metals_mix	4735573.133	107199.0653	2.263697809

UIJ65296.1	O-acetylserine_dependent_cystathionine_beta-synthase_[Bacillus_cereus]	CPTF_zcontrol	1144537.567	213330.1164	18.63897897
UIJ65297.1	5'-methylthioadenosine/S-adenosylhomocysteine_nucleosidase_[Bacillus_cereus]	CPTF_Al	1771810.7	240032.0766	13.54727548
UIJ65297.1	5'-methylthioadenosine/S-adenosylhomocysteine_nucleosidase_[Bacillus_cereus]	CPTF_Cd	1861553.867	20296.92672	1.090321751
UIJ65297.1	5'-methylthioadenosine/S-adenosylhomocysteine_nucleosidase_[Bacillus_cereus]	CPTF_Co	2043524.333	76621.65862	3.749485992
UIJ65297.1	5'-methylthioadenosine/S-adenosylhomocysteine_nucleosidase_[Bacillus_cereus]	CPTF_Cu	2422183.467	37921.43194	1.565588753
UIJ65297.1	5'-methylthioadenosine/S-adenosylhomocysteine_nucleosidase_[Bacillus_cereus]	CPTF_Fe	1679756.333	189910.9172	11.30586106
UIJ65297.1	5'-methylthioadenosine/S-adenosylhomocysteine_nucleosidase_[Bacillus_cereus]	CPTF_Mn	1579830.4	258553.5135	16.36590317
UIJ65297.1	5'-methylthioadenosine/S-adenosylhomocysteine_nucleosidase_[Bacillus_cereus]	CPTF_Ni	1726677.667	195282.845	11.30974523
UIJ65297.1	5'-methylthioadenosine/S-adenosylhomocysteine_nucleosidase_[Bacillus_cereus]	CPTF_U	1359430.333	30705.27734	2.258687083
UIJ65297.1	5'-methylthioadenosine/S-adenosylhomocysteine_nucleosidase_[Bacillus_cereus]	CPTF_metals_mix	4875573.667	1095341.34	22.46589662
UIJ65297.1	5'-methylthioadenosine/S-adenosylhomocysteine_nucleosidase_[Bacillus_cereus]	CPTF_zcontrol	1541846.067	85696.13235	5.558021271
UIJ65298.1	class_I_SAM-dependent_methyltransferase_[Bacillus_cereus]	CPTF_Al	2938626.633	316596.1182	10.77360814
UIJ65298.1	class_I_SAM-dependent_methyltransferase_[Bacillus_cereus]	CPTF_Cd	2331125.9	1168625.289	50.13136736
UIJ65298.1	class_I_SAM-dependent_methyltransferase_[Bacillus_cereus]	CPTF_Co	2339025.8	1177883.5495	50.35787055
UIJ65298.1	class_I_SAM-dependent_methyltransferase_[Bacillus_cereus]	CPTF_Cu	3437923.267	1223180.267	35.57904502
UIJ65298.1	class_I_SAM-dependent_methyltransferase_[Bacillus_cereus]	CPTF_Fe	2567504.767	1005711.067	39.17075755
UIJ65298.1	class_I_SAM-dependent_methyltransferase_[Bacillus_cereus]	CPTF_Mn	2425887.2	1090707.734	44.96118919
UIJ65298.1	class_I_SAM-dependent_methyltransferase_[Bacillus_cereus]	CPTF_Ni	2767655.467	1246273.202	45.02992577
UIJ65298.1	class_I_SAM-dependent_methyltransferase_[Bacillus_cereus]	CPTF_U	2669148.6	217266.4435	8.13991561
UIJ65298.1	class_I_SAM-dependent_methyltransferase_[Bacillus_cereus]	CPTF_metals_mix	7291032.367	330593.1537	4.534243398
UIJ65298.1	class_I_SAM-dependent_methyltransferase_[Bacillus_cereus]	CPTF_zcontrol	2694032.233	1060678.625	39.37141552
UIJ65300.1	YrrS_family_protein_[Bacillus_cereus]	CPTF_Al	16280	28197.78715	173.2050808
UIJ65300.1	YrrS_family_protein_[Bacillus_cereus]	CPTF_Cd	39594.06667	38237.61109	96.57409381
UIJ65300.1	YrrS_family_protein_[Bacillus_cereus]	CPTF_Co	0	0	0
UIJ65300.1	YrrS_family_protein_[Bacillus_cereus]	CPTF_Cu	5826.833333	10092.37138	173.2050808
UIJ65300.1	YrrS_family_protein_[Bacillus_cereus]	CPTF_Fe	0	0	0
UIJ65300.1	YrrS_family_protein_[Bacillus_cereus]	CPTF_Mn	10444.9	18091.09748	173.2050808
UIJ65300.1	YrrS_family_protein_[Bacillus_cereus]	CPTF_Ni	0	0	0
UIJ65300.1	YrrS_family_protein_[Bacillus_cereus]	CPTF_U	14402.23333	24945.39988	173.2050808
UIJ65300.1	YrrS_family_protein_[Bacillus_cereus]	CPTF_metals_mix	44057.5	28366.82968	64.38592675
UIJ65300.1	YrrS_family_protein_[Bacillus_cereus]	CPTF_zcontrol	0	0	0
UIJ65303.1	transcription_elongation_factor_GreA_[Bacillus_cereus]	CPTF_Al	6227721.667	72158.66878	1.158668814
UIJ65303.1	transcription_elongation_factor_GreA_[Bacillus_cereus]	CPTF_Cd	6928071.667	301538.1749	4.352411312
UIJ65303.1	transcription_elongation_factor_GreA_[Bacillus_cereus]	CPTF_Co	6315609.667	165240.5495	2.616383187
UIJ65303.1	transcription_elongation_factor_GreA_[Bacillus_cereus]	CPTF_Cu	5880147.333	515187.318	8.761469547
UIJ65303.1	transcription_elongation_factor_GreA_[Bacillus_cereus]	CPTF_Fe	6153553.333	225766.8292	3.668885552
UIJ65303.1	transcription_elongation_factor_GreA_[Bacillus_cereus]	CPTF_Mn	6708636	204175.753	3.043476394
UIJ65303.1	transcription_elongation_factor_GreA_[Bacillus_cereus]	CPTF_Ni	5676956.667	223233.1234	3.932267526
UIJ65303.1	transcription_elongation_factor_GreA_[Bacillus_cereus]	CPTF_U	5575316.667	145052.0836	2.601683317
UIJ65303.1	transcription_elongation_factor_GreA_[Bacillus_cereus]	CPTF_metals_mix	5390419	257790.803	4.782388956
UIJ65303.1	transcription_elongation_factor_GreA_[Bacillus_cereus]	CPTF_zcontrol	6236112.667	187396.9261	3.005027909
UIJ65304.1	uridine_kinase_[Bacillus_cereus]	CPTF_Al	0	0	0
UIJ65304.1	uridine_kinase_[Bacillus_cereus]	CPTF_Cd	59213.66667	102561.0792	173.2050808
UIJ65304.1	uridine_kinase_[Bacillus_cereus]	CPTF_Co	147892	22760.79401	15.39014552
UIJ65304.1	uridine_kinase_[Bacillus_cereus]	CPTF_Cu	193433.7333	111969.2896	57.88508949
UIJ65304.1	uridine_kinase_[Bacillus_cereus]	CPTF_Fe	187925.9667	289285.4839	153.9358765
UIJ65304.1	uridine_kinase_[Bacillus_cereus]	CPTF_Mn	0	0	0
UIJ65304.1	uridine_kinase_[Bacillus_cereus]	CPTF_Ni	10199.63333	17666.28315	173.2050808
UIJ65304.1	uridine_kinase_[Bacillus_cereus]	CPTF_U	171999.4667	237889.3872	138.3082121
UIJ65304.1	uridine_kinase_[Bacillus_cereus]	CPTF_metals_mix	103919.8667	110557.0656	106.3868432
UIJ65304.1	uridine_kinase_[Bacillus_cereus]	CPTF_zcontrol	102955.4	111705.2398	108.4986701
UIJ65306.1	U32_family_peptidase_[Bacillus_cereus]	CPTF_Al	116522	79056.19731	67.8465846
UIJ65306.1	U32_family_peptidase_[Bacillus_cereus]	CPTF_Cd	20092.96667	17464.33323	86.91764397
UIJ65306.1	U32_family_peptidase_[Bacillus_cereus]	CPTF_Co	97600.7	103906.9089	106.4612333
UIJ65306.1	U32_family_peptidase_[Bacillus_cereus]	CPTF_Cu	215374.2333	108264.9	50.26826948
UIJ65306.1	U32_family_peptidase_[Bacillus_cereus]	CPTF_Fe	117651.9667	166202.645	141.2663551
UIJ65306.1	U32_family_peptidase_[Bacillus_cereus]	CPTF_Mn	44733.23333	38778.14071	86.68754263
UIJ65306.1	U32_family_peptidase_[Bacillus_cereus]	CPTF_Ni	174375.4	198287.0702	113.7127543
UIJ65306.1	U32_family_peptidase_[Bacillus_cereus]	CPTF_U	202229.3	179018.8373	88.52270036
UIJ65306.1	U32_family_peptidase_[Bacillus_cereus]	CPTF_metals_mix	0	0	0
UIJ65306.1	U32_family_peptidase_[Bacillus_cereus]	CPTF_zcontrol	138329.7	76042.65401	54.97203711

UIJ65309.1	DUF1292_domain-containing_protein_[Bacillus_cereus]	CPTF_Al	1081606.533	127574.6512	11.79492239
UIJ65309.1	DUF1292_domain-containing_protein_[Bacillus_cereus]	CPTF_Cd	1173535.7	47170.59943	4.019528288
UIJ65309.1	DUF1292_domain-containing_protein_[Bacillus_cereus]	CPTF_Co	1042881.633	48837.22689	4.682911783
UIJ65309.1	DUF1292_domain-containing_protein_[Bacillus_cereus]	CPTF_Cu	906868	17831.14306	1.966233571
UIJ65309.1	DUF1292_domain-containing_protein_[Bacillus_cereus]	CPTF_Fe	1017825.867	111236.3586	10.92882016
UIJ65309.1	DUF1292_domain-containing_protein_[Bacillus_cereus]	CPTF_Mn	1149637.333	60455.14801	5.258627766
UIJ65309.1	DUF1292_domain-containing_protein_[Bacillus_cereus]	CPTF_Ni	896784.3	78059.7106	8.704402006
UIJ65309.1	DUF1292_domain-containing_protein_[Bacillus_cereus]	CPTF_U	896891.3333	107615.872	11.99876373
UIJ65309.1	DUF1292_domain-containing_protein_[Bacillus_cereus]	CPTF_metals_mix	922375.6667	62950.7691	6.824851454
UIJ65309.1	DUF1292_domain-containing_protein_[Bacillus_cereus]	CPTF_zcontrol	1145747.033	129235.3445	11.27957051
UIJ65310.1	Holliday_junction_resolvase_RuvX_[Bacillus_cereus]	CPTF_Al	179049	51708.30781	28.87941726
UIJ65310.1	Holliday_junction_resolvase_RuvX_[Bacillus_cereus]	CPTF_Cd	161317.6667	35682.22911	22.1192321
UIJ65310.1	Holliday_junction_resolvase_RuvX_[Bacillus_cereus]	CPTF_Co	160900.3667	91398.94835	56.80468618
UIJ65310.1	Holliday_junction_resolvase_RuvX_[Bacillus_cereus]	CPTF_Cu	137097.6667	56586.92883	41.27490293
UIJ65310.1	Holliday_junction_resolvase_RuvX_[Bacillus_cereus]	CPTF_Fe	247864.3333	20835.28273	8.405922081
UIJ65310.1	Holliday_junction_resolvase_RuvX_[Bacillus_cereus]	CPTF_Mn	144484.6667	128418.9959	88.88070881
UIJ65310.1	Holliday_junction_resolvase_RuvX_[Bacillus_cereus]	CPTF_Ni	73533.26667	79691.49052	108.3747454
UIJ65310.1	Holliday_junction_resolvase_RuvX_[Bacillus_cereus]	CPTF_U	108315	130174.2686	120.1812017
UIJ65310.1	Holliday_junction_resolvase_RuvX_[Bacillus_cereus]	CPTF_metals_mix	178482.6667	55805.61737	31.26668736
UIJ65310.1	Holliday_junction_resolvase_RuvX_[Bacillus_cereus]	CPTF_zcontrol	149646.3333	129685.6661	86.66143914
UIJ65311.1	IreB_family_regulatory_phosphoprotein_[Bacillus_cereus]	CPTF_Al	749121.3333	61525.10127	8.212968785
UIJ65311.1	IreB_family_regulatory_phosphoprotein_[Bacillus_cereus]	CPTF_Cd	801524.3333	29169.06101	3.639198437
UIJ65311.1	IreB_family_regulatory_phosphoprotein_[Bacillus_cereus]	CPTF_Co	697888.6667	64788.88675	9.283556224
UIJ65311.1	IreB_family_regulatory_phosphoprotein_[Bacillus_cereus]	CPTF_Cu	772716.6667	22079.55834	2.857393827
UIJ65311.1	IreB_family_regulatory_phosphoprotein_[Bacillus_cereus]	CPTF_Fe	734435.6667	55052.21896	7.495853137
UIJ65311.1	IreB_family_regulatory_phosphoprotein_[Bacillus_cereus]	CPTF_Mn	739467	62799.77567	8.492573119
UIJ65311.1	IreB_family_regulatory_phosphoprotein_[Bacillus_cereus]	CPTF_Ni	745417.3333	20528.94974	2.754020979
UIJ65311.1	IreB_family_regulatory_phosphoprotein_[Bacillus_cereus]	CPTF_U	626881.6667	96316.25688	15.36434418
UIJ65311.1	IreB_family_regulatory_phosphoprotein_[Bacillus_cereus]	CPTF_metals_mix	846654	83631.41676	9.877874168
UIJ65311.1	IreB_family_regulatory_phosphoprotein_[Bacillus_cereus]	CPTF_zcontrol	756536.3333	109058.9705	14.41556284
UIJ65312.1	alanine--tRNA_ligase_[Bacillus_cereus]	CPTF_Al	5290794.8	160097.9532	3.025971698
UIJ65312.1	alanine--tRNA_ligase_[Bacillus_cereus]	CPTF_Cd	5442521.133	828854.9445	15.22924623
UIJ65312.1	alanine--tRNA_ligase_[Bacillus_cereus]	CPTF_Co	5380544.567	551393.0084	10.24790338
UIJ65312.1	alanine--tRNA_ligase_[Bacillus_cereus]	CPTF_Cu	5388584.233	82828.16309	1.537104358
UIJ65312.1	alanine--tRNA_ligase_[Bacillus_cereus]	CPTF_Fe	4654344.333	769808.351	16.53956596
UIJ65312.1	alanine--tRNA_ligase_[Bacillus_cereus]	CPTF_Mn	5318801.067	1218901.456	22.91684612
UIJ65312.1	alanine--tRNA_ligase_[Bacillus_cereus]	CPTF_Ni	4182384.433	800812.5901	19.14727359
UIJ65312.1	alanine--tRNA_ligase_[Bacillus_cereus]	CPTF_U	3257159.9	1131290.857	34.73243229
UIJ65312.1	alanine--tRNA_ligase_[Bacillus_cereus]	CPTF_metals_mix	7705813.867	469546.265	6.093402633
UIJ65312.1	alanine--tRNA_ligase_[Bacillus_cereus]	CPTF_zcontrol	4959323.3	220001.7845	4.436125075
UIJ65313.1	PH_domain-containing_protein_[Bacillus_cereus]	CPTF_Al	0	0	0
UIJ65313.1	PH_domain-containing_protein_[Bacillus_cereus]	CPTF_Cd	0	0	0
UIJ65313.1	PH_domain-containing_protein_[Bacillus_cereus]	CPTF_Co	0	0	0
UIJ65313.1	PH_domain-containing_protein_[Bacillus_cereus]	CPTF_Cu	5776.833333	10005.76884	173.2050808
UIJ65313.1	PH_domain-containing_protein_[Bacillus_cereus]	CPTF_Fe	0	0	0
UIJ65313.1	PH_domain-containing_protein_[Bacillus_cereus]	CPTF_Mn	0	0	0
UIJ65313.1	PH_domain-containing_protein_[Bacillus_cereus]	CPTF_Ni	0	0	0
UIJ65313.1	PH_domain-containing_protein_[Bacillus_cereus]	CPTF_U	0	0	0
UIJ65313.1	PH_domain-containing_protein_[Bacillus_cereus]	CPTF_metals_mix	4988.4	8640.162248	173.2050808
UIJ65313.1	PH_domain-containing_protein_[Bacillus_cereus]	CPTF_zcontrol	0	0	0
UIJ65320.1	cysteine_desulfurase_[Bacillus_cereus]	CPTF_Al	200935.3333	30400.40862	15.12944892
UIJ65320.1	cysteine_desulfurase_[Bacillus_cereus]	CPTF_Cd	187003.3333	33953.03495	18.15637954
UIJ65320.1	cysteine_desulfurase_[Bacillus_cereus]	CPTF_Co	120646.3333	121119.7435	100.392395
UIJ65320.1	cysteine_desulfurase_[Bacillus_cereus]	CPTF_Cu	157748	53059.35903	33.63551933
UIJ65320.1	cysteine_desulfurase_[Bacillus_cereus]	CPTF_Fe	196031	45145.39774	23.02972374
UIJ65320.1	cysteine_desulfurase_[Bacillus_cereus]	CPTF_Mn	129091.3333	14939.77785	11.57302932
UIJ65320.1	cysteine_desulfurase_[Bacillus_cereus]	CPTF_Ni	128509	23133.92589	18.00179434
UIJ65320.1	cysteine_desulfurase_[Bacillus_cereus]	CPTF_U	65579.43333	57289.39505	87.35878329
UIJ65320.1	cysteine_desulfurase_[Bacillus_cereus]	CPTF_metals_mix	395809	125139.5724	31.61615132
UIJ65320.1	cysteine_desulfurase_[Bacillus_cereus]	CPTF_zcontrol	193920	54160.64035	27.92937312
UIJ65321.1	Rrf2_family_transcriptional_regulator_[Bacillus_cereus]	CPTF_Al	1415020.5	103301.0241	7.300319968

UIJ65321.1	Rrf2_family_transcriptional_regulator_[Bacillus_cereus]	CPTF_Cd	1695675.6	57271.42258	3.377498773
UIJ65321.1	Rrf2_family_transcriptional_regulator_[Bacillus_cereus]	CPTF_Co	1754364.6	273223.0526	15.57390366
UIJ65321.1	Rrf2_family_transcriptional_regulator_[Bacillus_cereus]	CPTF_Cu	1637439.333	70152.50634	4.28428125
UIJ65321.1	Rrf2_family_transcriptional_regulator_[Bacillus_cereus]	CPTF_Fe	1400665.867	112419.2043	8.026125789
UIJ65321.1	Rrf2_family_transcriptional_regulator_[Bacillus_cereus]	CPTF_Mn	1285074.7	218019.6432	16.96552295
UIJ65321.1	Rrf2_family_transcriptional_regulator_[Bacillus_cereus]	CPTF_Ni	1428427.267	412572.0871	28.88296077
UIJ65321.1	Rrf2_family_transcriptional_regulator_[Bacillus_cereus]	CPTF_U	997784.4667	342742.6327	34.35036765
UIJ65321.1	Rrf2_family_transcriptional_regulator_[Bacillus_cereus]	CPTF_metals_mix	2416394.667	260189.2448	10.76766343
UIJ65321.1	Rrf2_family_transcriptional_regulator_[Bacillus_cereus]	CPTF_zcontrol	1384483.233	110370.399	7.97195635
UIJ65324.1	tRNA_threonylcarbamoyladenosine_dehydratase_[Bacillus_cereus]	CPTF_Al	0	0	0
UIJ65324.1	tRNA_threonylcarbamoyladenosine_dehydratase_[Bacillus_cereus]	CPTF_Cd	0	0	0
UIJ65324.1	tRNA_threonylcarbamoyladenosine_dehydratase_[Bacillus_cereus]	CPTF_Co	0	0	0
UIJ65324.1	tRNA_threonylcarbamoyladenosine_dehydratase_[Bacillus_cereus]	CPTF_Cu	60702.66667	105140.1028	173.2050808
UIJ65324.1	tRNA_threonylcarbamoyladenosine_dehydratase_[Bacillus_cereus]	CPTF_Fe	0	0	0
UIJ65324.1	tRNA_threonylcarbamoyladenosine_dehydratase_[Bacillus_cereus]	CPTF_Mn	0	0	0
UIJ65324.1	tRNA_threonylcarbamoyladenosine_dehydratase_[Bacillus_cereus]	CPTF_Ni	92752.66667	160652.3312	173.2050808
UIJ65324.1	tRNA_threonylcarbamoyladenosine_dehydratase_[Bacillus_cereus]	CPTF_U	0	0	0
UIJ65324.1	tRNA_threonylcarbamoyladenosine_dehydratase_[Bacillus_cereus]	CPTF_metals_mix	716508	224727.9215	31.36432831
UIJ65324.1	tRNA_threonylcarbamoyladenosine_dehydratase_[Bacillus_cereus]	CPTF_zcontrol	0	0	0
UIJ65325.1	aspartate--tRNA_ligase_[Bacillus_cereus]	CPTF_Al	5600435.5	484641.5801	8.653640956
UIJ65325.1	aspartate--tRNA_ligase_[Bacillus_cereus]	CPTF_Cd	5850997.867	254325.9659	4.346710966
UIJ65325.1	aspartate--tRNA_ligase_[Bacillus_cereus]	CPTF_Co	6313616.767	320084.3156	5.069745717
UIJ65325.1	aspartate--tRNA_ligase_[Bacillus_cereus]	CPTF_Cu	5902015.033	476808.1638	8.078735163
UIJ65325.1	aspartate--tRNA_ligase_[Bacillus_cereus]	CPTF_Fe	5662055.267	507234.5052	8.958487357
UIJ65325.1	aspartate--tRNA_ligase_[Bacillus_cereus]	CPTF_Mn	6102347.033	1078196.791	17.66855908
UIJ65325.1	aspartate--tRNA_ligase_[Bacillus_cereus]	CPTF_Ni	5853452.467	1091098.492	18.64025545
UIJ65325.1	aspartate--tRNA_ligase_[Bacillus_cereus]	CPTF_U	6204584.9	207394.149	3.34259507
UIJ65325.1	aspartate--tRNA_ligase_[Bacillus_cereus]	CPTF_metals_mix	4720818.3	124630.7441	2.64002417
UIJ65325.1	aspartate--tRNA_ligase_[Bacillus_cereus]	CPTF_zcontrol	5982533.3	582417.3768	9.735296864
UIJ65326.1	histidine--tRNA_ligase_[Bacillus_cereus]	CPTF_Al	784488.5	170752.5823	21.76610394
UIJ65326.1	histidine--tRNA_ligase_[Bacillus_cereus]	CPTF_Cd	866032.5667	92700.91311	10.70409089
UIJ65326.1	histidine--tRNA_ligase_[Bacillus_cereus]	CPTF_Co	816740.9	200016.1322	24.48954525
UIJ65326.1	histidine--tRNA_ligase_[Bacillus_cereus]	CPTF_Cu	953023.2667	58628.49954	6.151843464
UIJ65326.1	histidine--tRNA_ligase_[Bacillus_cereus]	CPTF_Fe	706861.4333	183008.5359	25.89029861
UIJ65326.1	histidine--tRNA_ligase_[Bacillus_cereus]	CPTF_Mn	635635.3	56353.34719	8.865673003
UIJ65326.1	histidine--tRNA_ligase_[Bacillus_cereus]	CPTF_Ni	986614.9	160911.5725	16.30946102
UIJ65326.1	histidine--tRNA_ligase_[Bacillus_cereus]	CPTF_U	699666.5667	273269.4864	39.05710226
UIJ65326.1	histidine--tRNA_ligase_[Bacillus_cereus]	CPTF_metals_mix	2179473.067	244747.3461	11.22965683
UIJ65326.1	histidine--tRNA_ligase_[Bacillus_cereus]	CPTF_zcontrol	873960.8	183939.6678	21.04667255
UIJ65330.1	adenine_phosphoribosyltransferase_[Bacillus_cereus]	CPTF_Al	773425	385861.4738	49.88996655
UIJ65330.1	adenine_phosphoribosyltransferase_[Bacillus_cereus]	CPTF_Cd	925052.3333	145486.9859	15.727433
UIJ65330.1	adenine_phosphoribosyltransferase_[Bacillus_cereus]	CPTF_Co	1209598.167	243849.9039	20.15957949
UIJ65330.1	adenine_phosphoribosyltransferase_[Bacillus_cereus]	CPTF_Cu	1205931.6	193543.4422	16.04928855
UIJ65330.1	adenine_phosphoribosyltransferase_[Bacillus_cereus]	CPTF_Fe	835736.3333	284688.8913	34.06443874
UIJ65330.1	adenine_phosphoribosyltransferase_[Bacillus_cereus]	CPTF_Mn	1048076	45277.98334	4.320104967
UIJ65330.1	adenine_phosphoribosyltransferase_[Bacillus_cereus]	CPTF_Ni	1015889.667	477599.9089	47.01297046
UIJ65330.1	adenine_phosphoribosyltransferase_[Bacillus_cereus]	CPTF_U	569003	492815.4309	86.61033964
UIJ65330.1	adenine_phosphoribosyltransferase_[Bacillus_cereus]	CPTF_metals_mix	755217.5333	230675.7663	30.54428109
UIJ65330.1	adenine_phosphoribosyltransferase_[Bacillus_cereus]	CPTF_zcontrol	757519.6667	445608.2432	58.82464348
UIJ65333.1	protein_translocase_subunit_SecDF_[Bacillus_cereus]	CPTF_Al	2328870.733	182886.794	7.853024703
UIJ65333.1	protein_translocase_subunit_SecDF_[Bacillus_cereus]	CPTF_Cd	2260837.7	423943.426	18.75160813
UIJ65333.1	protein_translocase_subunit_SecDF_[Bacillus_cereus]	CPTF_Co	2411490.533	43461.26962	1.802257526
UIJ65333.1	protein_translocase_subunit_SecDF_[Bacillus_cereus]	CPTF_Cu	2481080.9	374371.9481	15.08906655
UIJ65333.1	protein_translocase_subunit_SecDF_[Bacillus_cereus]	CPTF_Fe	2557839.167	287525.8174	11.24096547
UIJ65333.1	protein_translocase_subunit_SecDF_[Bacillus_cereus]	CPTF_Mn	2192395.2	642471.7727	29.30456027
UIJ65333.1	protein_translocase_subunit_SecDF_[Bacillus_cereus]	CPTF_Ni	2251269.933	261364.4808	11.60964649
UIJ65333.1	protein_translocase_subunit_SecDF_[Bacillus_cereus]	CPTF_U	2233338.467	690785.6587	30.93063004
UIJ65333.1	protein_translocase_subunit_SecDF_[Bacillus_cereus]	CPTF_metals_mix	2274022.7	239732.0147	10.54219972
UIJ65333.1	protein_translocase_subunit_SecDF_[Bacillus_cereus]	CPTF_zcontrol	2514270.3	102399.1043	4.072716615
UIJ65338.1	preprotein_translocase_subunit_YajC_[Bacillus_cereus]	CPTF_Al	515155.8667	108943.6521	21.14770677
UIJ65338.1	preprotein_translocase_subunit_YajC_[Bacillus_cereus]	CPTF_Cd	525818	97160.97718	18.47806222

UIJ65338.1	preprotein_translocase_subunit_YajC_[Bacillus_cereus]	CPTF_Co	509354.6667	130331.6074	25.58759463
UIJ65338.1	preprotein_translocase_subunit_YajC_[Bacillus_cereus]	CPTF_Cu	374676.6667	109622.6958	29.25794574
UIJ65338.1	preprotein_translocase_subunit_YajC_[Bacillus_cereus]	CPTF_Fe	340596.3333	61877.09541	18.16728172
UIJ65338.1	preprotein_translocase_subunit_YajC_[Bacillus_cereus]	CPTF_Mn	598347.6667	178864.4826	29.89306929
UIJ65338.1	preprotein_translocase_subunit_YajC_[Bacillus_cereus]	CPTF_Ni	556114.3333	197407.3926	35.49762716
UIJ65338.1	preprotein_translocase_subunit_YajC_[Bacillus_cereus]	CPTF_U	325455.3333	47364.64517	14.55334736
UIJ65338.1	preprotein_translocase_subunit_YajC_[Bacillus_cereus]	CPTF_metals_mix	636564.3333	39314.01191	6.175968374
UIJ65338.1	preprotein_translocase_subunit_YajC_[Bacillus_cereus]	CPTF_zcontrol	391161.9	60325.37638	15.42209923
UIJ65339.1	tRNA_guanosine(34)_transglycosylase_Tgt_[Bacillus_cereus]	CPTF_Al	905969.8333	20070.69379	2.21538213
UIJ65339.1	tRNA_guanosine(34)_transglycosylase_Tgt_[Bacillus_cereus]	CPTF_Cd	956692.5	35636.48822	3.724967868
UIJ65339.1	tRNA_guanosine(34)_transglycosylase_Tgt_[Bacillus_cereus]	CPTF_Co	895374.8333	24472.63298	2.733227702
UIJ65339.1	tRNA_guanosine(34)_transglycosylase_Tgt_[Bacillus_cereus]	CPTF_Cu	858166.8333	112680.1318	13.13032938
UIJ65339.1	tRNA_guanosine(34)_transglycosylase_Tgt_[Bacillus_cereus]	CPTF_Fe	932873	116913.8813	12.53266857
UIJ65339.1	tRNA_guanosine(34)_transglycosylase_Tgt_[Bacillus_cereus]	CPTF_Mn	859970	245219.7637	28.51492072
UIJ65339.1	tRNA_guanosine(34)_transglycosylase_Tgt_[Bacillus_cereus]	CPTF_Ni	955987.8	90267.80462	9.442359476
UIJ65339.1	tRNA_guanosine(34)_transglycosylase_Tgt_[Bacillus_cereus]	CPTF_U	973281.3333	51923.86058	5.334928227
UIJ65339.1	tRNA_guanosine(34)_transglycosylase_Tgt_[Bacillus_cereus]	CPTF_metals_mix	1226228.067	183685.4366	14.97971231
UIJ65339.1	tRNA_guanosine(34)_transglycosylase_Tgt_[Bacillus_cereus]	CPTF_zcontrol	918488.4	43880.53919	4.777473421
UIJ65340.1	tRNA_preQ1(34)_S-adenosylmethionine_ribosyltransferase-isomerase_QueA_[Bacillus_cereus]	CPTF_Al	0	0	0
UIJ65340.1	tRNA_preQ1(34)_S-adenosylmethionine_ribosyltransferase-isomerase_QueA_[Bacillus_cereus]	CPTF_Cd	0	0	0
UIJ65340.1	tRNA_preQ1(34)_S-adenosylmethionine_ribosyltransferase-isomerase_QueA_[Bacillus_cereus]	CPTF_Co	0	0	0
UIJ65340.1	tRNA_preQ1(34)_S-adenosylmethionine_ribosyltransferase-isomerase_QueA_[Bacillus_cereus]	CPTF_Cu	0	0	0
UIJ65340.1	tRNA_preQ1(34)_S-adenosylmethionine_ribosyltransferase-isomerase_QueA_[Bacillus_cereus]	CPTF_Fe	0	0	0
UIJ65340.1	tRNA_preQ1(34)_S-adenosylmethionine_ribosyltransferase-isomerase_QueA_[Bacillus_cereus]	CPTF_Mn	0	0	0
UIJ65340.1	tRNA_preQ1(34)_S-adenosylmethionine_ribosyltransferase-isomerase_QueA_[Bacillus_cereus]	CPTF_Ni	0	0	0
UIJ65340.1	tRNA_preQ1(34)_S-adenosylmethionine_ribosyltransferase-isomerase_QueA_[Bacillus_cereus]	CPTF_U	0	0	0
UIJ65340.1	tRNA_preQ1(34)_S-adenosylmethionine_ribosyltransferase-isomerase_QueA_[Bacillus_cereus]	CPTF_metals_mix	111179.8	56000.31192	50.36914253
UIJ65340.1	tRNA_preQ1(34)_S-adenosylmethionine_ribosyltransferase-isomerase_QueA_[Bacillus_cereus]	CPTF_zcontrol	0	0	0
UIJ65350.1	carboxylating_nicotinate-nucleotide_diphosphorylase_[Bacillus_cereus]	CPTF_Al	33522.1	29296.07815	87.39332604
UIJ65350.1	carboxylating_nicotinate-nucleotide_diphosphorylase_[Bacillus_cereus]	CPTF_Cd	66022.26667	29727.08774	45.02585149
UIJ65350.1	carboxylating_nicotinate-nucleotide_diphosphorylase_[Bacillus_cereus]	CPTF_Co	31225.63333	7557.088211	24.20155303
UIJ65350.1	carboxylating_nicotinate-nucleotide_diphosphorylase_[Bacillus_cereus]	CPTF_Cu	0	0	0
UIJ65350.1	carboxylating_nicotinate-nucleotide_diphosphorylase_[Bacillus_cereus]	CPTF_Fe	9748.6	16885.0705	173.2050808
UIJ65350.1	carboxylating_nicotinate-nucleotide_diphosphorylase_[Bacillus_cereus]	CPTF_Mn	25899.03333	44858.4416	173.2050808
UIJ65350.1	carboxylating_nicotinate-nucleotide_diphosphorylase_[Bacillus_cereus]	CPTF_Ni	15441.7	26745.80896	173.2050808
UIJ65350.1	carboxylating_nicotinate-nucleotide_diphosphorylase_[Bacillus_cereus]	CPTF_U	0	0	0
UIJ65350.1	carboxylating_nicotinate-nucleotide_diphosphorylase_[Bacillus_cereus]	CPTF_metals_mix	0	0	0
UIJ65350.1	carboxylating_nicotinate-nucleotide_diphosphorylase_[Bacillus_cereus]	CPTF_zcontrol	12843.56667	22245.71002	173.2050808
UIJ65354.1	MOSC_domain-containing_protein_[Bacillus_cereus]	CPTF_Al	501703	4041.792548	0.805614586
UIJ65354.1	MOSC_domain-containing_protein_[Bacillus_cereus]	CPTF_Cd	480818.6667	135070.6676	28.09181028
UIJ65354.1	MOSC_domain-containing_protein_[Bacillus_cereus]	CPTF_Co	508385.5667	36467.19601	7.173137555
UIJ65354.1	MOSC_domain-containing_protein_[Bacillus_cereus]	CPTF_Cu	498462.3333	73248.66338	14.69492447
UIJ65354.1	MOSC_domain-containing_protein_[Bacillus_cereus]	CPTF_Fe	495516.3333	68990.69506	13.92299112
UIJ65354.1	MOSC_domain-containing_protein_[Bacillus_cereus]	CPTF_Mn	432458.3	95080.75115	21.98610852
UIJ65354.1	MOSC_domain-containing_protein_[Bacillus_cereus]	CPTF_Ni	535381.4333	17967.94279	3.356101216
UIJ65354.1	MOSC_domain-containing_protein_[Bacillus_cereus]	CPTF_U	613197.4333	59783.60631	9.749487369
UIJ65354.1	MOSC_domain-containing_protein_[Bacillus_cereus]	CPTF_metals_mix	529195.3	93623.41823	17.69165717
UIJ65354.1	MOSC_domain-containing_protein_[Bacillus_cereus]	CPTF_zcontrol	638179.3333	9275.939324	1.453500425
UIJ65355.1	prephenate_dehydratase_[Bacillus_cereus]	CPTF_Al	0	0	0
UIJ65355.1	prephenate_dehydratase_[Bacillus_cereus]	CPTF_Cd	0	0	0
UIJ65355.1	prephenate_dehydratase_[Bacillus_cereus]	CPTF_Co	0	0	0
UIJ65355.1	prephenate_dehydratase_[Bacillus_cereus]	CPTF_Cu	14692.63333	25448.38743	173.2050808
UIJ65355.1	prephenate_dehydratase_[Bacillus_cereus]	CPTF_Fe	16931.93333	14832.9859	87.60361626
UIJ65355.1	prephenate_dehydratase_[Bacillus_cereus]	CPTF_Mn	0	0	0
UIJ65355.1	prephenate_dehydratase_[Bacillus_cereus]	CPTF_Ni	0	0	0
UIJ65355.1	prephenate_dehydratase_[Bacillus_cereus]	CPTF_U	0	0	0
UIJ65355.1	prephenate_dehydratase_[Bacillus_cereus]	CPTF_metals_mix	102543.8667	31512.32325	30.73057832
UIJ65355.1	prephenate_dehydratase_[Bacillus_cereus]	CPTF_zcontrol	0	0	0
UIJ65361.1	GTPase_ObgE_[Bacillus_cereus]	CPTF_Al	696982.8	196098.9111	28.13540177
UIJ65361.1	GTPase_ObgE_[Bacillus_cereus]	CPTF_Cd	946417	198012.7272	20.92235528
UIJ65361.1	GTPase_ObgE_[Bacillus_cereus]	CPTF_Co	683674.7	2652.81482	0.388022962

UIJ65361.1	GTPase_ObgE_[Bacillus_cereus]	CPTF_Cu	806165.7333	106405.1291	13.1989149
UIJ65361.1	GTPase_ObgE_[Bacillus_cereus]	CPTF_Fe	723349.1667	86748.50621	11.99261853
UIJ65361.1	GTPase_ObgE_[Bacillus_cereus]	CPTF_Mn	684756.4333	210667.9139	30.76537928
UIJ65361.1	GTPase_ObgE_[Bacillus_cereus]	CPTF_Ni	625970.7	10685.9201	1.707095891
UIJ65361.1	GTPase_ObgE_[Bacillus_cereus]	CPTF_U	887726.9	475065.1838	53.51478972
UIJ65361.1	GTPase_ObgE_[Bacillus_cereus]	CPTF_metals_mix	718111.9333	98105.11293	13.66153497
UIJ65361.1	GTPase_ObgE_[Bacillus_cereus]	CPTF_zcontrol	578690.7333	178224.2219	30.79783581
UIJ65362.1	sporulation_initiation_phosphotransferase_B_[Bacillus_cereus]	CPTF_Al	83705.63333	16897.98218	20.18738943
UIJ65362.1	sporulation_initiation_phosphotransferase_B_[Bacillus_cereus]	CPTF_Cd	71065.73333	16916.93046	23.80462379
UIJ65362.1	sporulation_initiation_phosphotransferase_B_[Bacillus_cereus]	CPTF_Co	55860.26667	31701.45448	56.75134827
UIJ65362.1	sporulation_initiation_phosphotransferase_B_[Bacillus_cereus]	CPTF_Cu	76841.4	55544.27526	72.28430932
UIJ65362.1	sporulation_initiation_phosphotransferase_B_[Bacillus_cereus]	CPTF_Fe	69944.9	38235.24322	54.66480505
UIJ65362.1	sporulation_initiation_phosphotransferase_B_[Bacillus_cereus]	CPTF_Mn	42223.76667	12774.83866	30.25509013
UIJ65362.1	sporulation_initiation_phosphotransferase_B_[Bacillus_cereus]	CPTF_Ni	46586.43333	40675.49294	87.31188466
UIJ65362.1	sporulation_initiation_phosphotransferase_B_[Bacillus_cereus]	CPTF_U	61232.73333	9109.220499	14.87639046
UIJ65362.1	sporulation_initiation_phosphotransferase_B_[Bacillus_cereus]	CPTF_metals_mix	27794.3	15077.05065	54.24511734
UIJ65362.1	sporulation_initiation_phosphotransferase_B_[Bacillus_cereus]	CPTF_zcontrol	44868.76667	23264.43647	51.84995755
UIJ65363.1	50S_ribosomal_protein_L27_[Bacillus_cereus]	CPTF_Al	9214890.9	475394.345	5.158979636
UIJ65363.1	50S_ribosomal_protein_L27_[Bacillus_cereus]	CPTF_Cd	10663938.53	145360.0199	1.363098816
UIJ65363.1	50S_ribosomal_protein_L27_[Bacillus_cereus]	CPTF_Co	9979157	96214.98286	0.964159426
UIJ65363.1	50S_ribosomal_protein_L27_[Bacillus_cereus]	CPTF_Cu	8735191.333	675297.8484	7.730773405
UIJ65363.1	50S_ribosomal_protein_L27_[Bacillus_cereus]	CPTF_Fe	9533952	1650922.17	17.31624168
UIJ65363.1	50S_ribosomal_protein_L27_[Bacillus_cereus]	CPTF_Mn	9602592.633	1867705.605	19.45001393
UIJ65363.1	50S_ribosomal_protein_L27_[Bacillus_cereus]	CPTF_Ni	7343801	1042827.759	14.20010916
UIJ65363.1	50S_ribosomal_protein_L27_[Bacillus_cereus]	CPTF_U	7090949.533	563900.1497	7.952392653
UIJ65363.1	50S_ribosomal_protein_L27_[Bacillus_cereus]	CPTF_metals_mix	9187013.733	82247.64621	0.895259859
UIJ65363.1	50S_ribosomal_protein_L27_[Bacillus_cereus]	CPTF_zcontrol	10162447.93	876543.4558	8.625318049
UIJ65365.1	50S_ribosomal_protein_L21_[Bacillus_cereus]	CPTF_Al	2561516.333	291700.8604	11.38781965
UIJ65365.1	50S_ribosomal_protein_L21_[Bacillus_cereus]	CPTF_Cd	3512478	171574.847	4.884723748
UIJ65365.1	50S_ribosomal_protein_L21_[Bacillus_cereus]	CPTF_Co	2726666.667	334713.8081	12.27556753
UIJ65365.1	50S_ribosomal_protein_L21_[Bacillus_cereus]	CPTF_Cu	2583333.333	130511.813	5.052070181
UIJ65365.1	50S_ribosomal_protein_L21_[Bacillus_cereus]	CPTF_Fe	2881496.333	522132.4475	18.12018435
UIJ65365.1	50S_ribosomal_protein_L21_[Bacillus_cereus]	CPTF_Mn	4167748	2907702.301	69.76674936
UIJ65365.1	50S_ribosomal_protein_L21_[Bacillus_cereus]	CPTF_Ni	2430000	167032.9309	6.873783164
UIJ65365.1	50S_ribosomal_protein_L21_[Bacillus_cereus]	CPTF_U	2426385.667	356098.285	14.67607932
UIJ65365.1	50S_ribosomal_protein_L21_[Bacillus_cereus]	CPTF_metals_mix	11630655	1105613.93	9.506033233
UIJ65365.1	50S_ribosomal_protein_L21_[Bacillus_cereus]	CPTF_zcontrol	2430305.367	103640.2793	4.26449617
UIJ65369.1	septum_site-determining_protein_MinD_[Bacillus_cereus]	CPTF_Al	2509139.933	70440.26683	2.807347087
UIJ65369.1	septum_site-determining_protein_MinD_[Bacillus_cereus]	CPTF_Cd	2434987.567	88638.37799	3.640198381
UIJ65369.1	septum_site-determining_protein_MinD_[Bacillus_cereus]	CPTF_Co	2673383.667	169525.98	6.341251429
UIJ65369.1	septum_site-determining_protein_MinD_[Bacillus_cereus]	CPTF_Cu	2610357.933	150736.6145	5.774557296
UIJ65369.1	septum_site-determining_protein_MinD_[Bacillus_cereus]	CPTF_Fe	2218606.833	205288.7914	9.253049631
UIJ65369.1	septum_site-determining_protein_MinD_[Bacillus_cereus]	CPTF_Mn	2341831.8	551755.3073	23.56084273
UIJ65369.1	septum_site-determining_protein_MinD_[Bacillus_cereus]	CPTF_Ni	1680120.2	358773.4194	21.35403285
UIJ65369.1	septum_site-determining_protein_MinD_[Bacillus_cereus]	CPTF_U	1569300	241621.3195	15.39675776
UIJ65369.1	septum_site-determining_protein_MinD_[Bacillus_cereus]	CPTF_metals_mix	3387382.3	476788.1596	14.07541628
UIJ65369.1	septum_site-determining_protein_MinD_[Bacillus_cereus]	CPTF_zcontrol	2008318.467	397426.8929	19.78903742
UIJ65370.1	septum_site-determining_protein_MinC_[Bacillus_cereus]	CPTF_Al	229137	26335.07553	11.49315716
UIJ65370.1	septum_site-determining_protein_MinC_[Bacillus_cereus]	CPTF_Cd	206870.6667	41161.4724	19.89720102
UIJ65370.1	septum_site-determining_protein_MinC_[Bacillus_cereus]	CPTF_Co	223132.3333	40283.51238	18.05364188
UIJ65370.1	septum_site-determining_protein_MinC_[Bacillus_cereus]	CPTF_Cu	259339	14427.82322	5.563306415
UIJ65370.1	septum_site-determining_protein_MinC_[Bacillus_cereus]	CPTF_Fe	264563	35780.26047	13.5242874
UIJ65370.1	septum_site-determining_protein_MinC_[Bacillus_cereus]	CPTF_Mn	188313.2667	101785.2174	54.05100726
UIJ65370.1	septum_site-determining_protein_MinC_[Bacillus_cereus]	CPTF_Ni	235547.3333	38579.24901	16.37855478
UIJ65370.1	septum_site-determining_protein_MinC_[Bacillus_cereus]	CPTF_U	156014.3	132435.2194	84.88659016
UIJ65370.1	septum_site-determining_protein_MinC_[Bacillus_cereus]	CPTF_metals_mix	209879.6667	6070.615894	2.89242688
UIJ65370.1	septum_site-determining_protein_MinC_[Bacillus_cereus]	CPTF_zcontrol	150951.8333	92540.88222	61.30490778
UIJ65372.1	rod_shape-determining_protein_MreC_[Bacillus_cereus]	CPTF_Al	129848.1667	31445.99011	24.21750797
UIJ65372.1	rod_shape-determining_protein_MreC_[Bacillus_cereus]	CPTF_Cd	46607.3	62509.46684	134.1194766
UIJ65372.1	rod_shape-determining_protein_MreC_[Bacillus_cereus]	CPTF_Co	150531.2	132186.7751	87.81353971
UIJ65372.1	rod_shape-determining_protein_MreC_[Bacillus_cereus]	CPTF_Cu	197079.6333	38277.37283	19.42228742

UIJ65372.1	rod_shape-determining_protein_MreC_[Bacillus_cereus]	CPTF_Fe	43605.33333	75526.65281	173.2050808
UIJ65372.1	rod_shape-determining_protein_MreC_[Bacillus_cereus]	CPTF_Mn	38386.66667	66487.657	173.2050808
UIJ65372.1	rod_shape-determining_protein_MreC_[Bacillus_cereus]	CPTF_Ni	142014.6667	127174.2653	89.55009247
UIJ65372.1	rod_shape-determining_protein_MreC_[Bacillus_cereus]	CPTF_U	49265.4	85330.17586	173.2050808
UIJ65372.1	rod_shape-determining_protein_MreC_[Bacillus_cereus]	CPTF_metals_mix	398654.4333	35224.51944	8.835852932
UIJ65372.1	rod_shape-determining_protein_MreC_[Bacillus_cereus]	CPTF_zcontrol	59150	102450.8053	173.2050808
UIJ65373.1	cell_shape-determining_protein_MreB_[Bacillus_cereus]	CPTF_Al	5524383.133	331490.821	6.000503822
UIJ65373.1	cell_shape-determining_protein_MreB_[Bacillus_cereus]	CPTF_Cd	5794978.167	322962.4369	5.573143291
UIJ65373.1	cell_shape-determining_protein_MreB_[Bacillus_cereus]	CPTF_Co	6247830.7	349549.5254	5.594734272
UIJ65373.1	cell_shape-determining_protein_MreB_[Bacillus_cereus]	CPTF_Cu	7275973.733	396851.8357	5.454278015
UIJ65373.1	cell_shape-determining_protein_MreB_[Bacillus_cereus]	CPTF_Fe	5458163	496616.2842	9.098597535
UIJ65373.1	cell_shape-determining_protein_MreB_[Bacillus_cereus]	CPTF_Mn	5363647.033	798943.043	14.89551863
UIJ65373.1	cell_shape-determining_protein_MreB_[Bacillus_cereus]	CPTF_Ni	5238701.433	197237.8083	3.76501335
UIJ65373.1	cell_shape-determining_protein_MreB_[Bacillus_cereus]	CPTF_U	4587526.333	205018.3766	4.469039777
UIJ65373.1	cell_shape-determining_protein_MreB_[Bacillus_cereus]	CPTF_metals_mix	8703264.8	460503.5491	5.291158659
UIJ65373.1	cell_shape-determining_protein_MreB_[Bacillus_cereus]	CPTF_zcontrol	5233422.167	696845.0873	13.3152852
UIJ65378.1	SMI1/KNR4_family_protein_[Bacillus_cereus]	CPTF_Al	580094.2	120763.05	20.81783441
UIJ65378.1	SMI1/KNR4_family_protein_[Bacillus_cereus]	CPTF_Cd	493630.1333	287954.1566	58.3339908
UIJ65378.1	SMI1/KNR4_family_protein_[Bacillus_cereus]	CPTF_Co	734091.7667	251913.4711	34.31634607
UIJ65378.1	SMI1/KNR4_family_protein_[Bacillus_cereus]	CPTF_Cu	556967.1	173452.7601	31.1423709
UIJ65378.1	SMI1/KNR4_family_protein_[Bacillus_cereus]	CPTF_Fe	546302.1667	33859.26418	6.197900402
UIJ65378.1	SMI1/KNR4_family_protein_[Bacillus_cereus]	CPTF_Mn	421010.4	92607.48142	21.99648309
UIJ65378.1	SMI1/KNR4_family_protein_[Bacillus_cereus]	CPTF_Ni	918912.3333	165326.1695	17.99150621
UIJ65378.1	SMI1/KNR4_family_protein_[Bacillus_cereus]	CPTF_U	629543.7333	291662.1991	46.32914024
UIJ65378.1	SMI1/KNR4_family_protein_[Bacillus_cereus]	CPTF_metals_mix	688796.3	27078.46295	3.931272998
UIJ65378.1	SMI1/KNR4_family_protein_[Bacillus_cereus]	CPTF_zcontrol	553103.3333	107835.8213	19.49650541
UIJ65379.1	tetratricopeptide_repeat_protein_[Bacillus_cereus]	CPTF_Al	194637.3667	65040.51639	33.41625378
UIJ65379.1	tetratricopeptide_repeat_protein_[Bacillus_cereus]	CPTF_Cd	152120.3333	83072.73607	54.60988301
UIJ65379.1	tetratricopeptide_repeat_protein_[Bacillus_cereus]	CPTF_Co	187720.9	53777.81271	28.64774924
UIJ65379.1	tetratricopeptide_repeat_protein_[Bacillus_cereus]	CPTF_Cu	233580.5333	2619.357985	1.121393957
UIJ65379.1	tetratricopeptide_repeat_protein_[Bacillus_cereus]	CPTF_Fe	159452.6	62172.11514	38.99096982
UIJ65379.1	tetratricopeptide_repeat_protein_[Bacillus_cereus]	CPTF_Mn	6608.833333	11446.83511	173.2050808
UIJ65379.1	tetratricopeptide_repeat_protein_[Bacillus_cereus]	CPTF_Ni	122011	45400.12376	37.20986121
UIJ65379.1	tetratricopeptide_repeat_protein_[Bacillus_cereus]	CPTF_U	154051.3	109962.0808	71.38017064
UIJ65379.1	tetratricopeptide_repeat_protein_[Bacillus_cereus]	CPTF_metals_mix	330315.1333	79452.69851	24.05360533
UIJ65379.1	tetratricopeptide_repeat_protein_[Bacillus_cereus]	CPTF_zcontrol	35975.33333	38872.10212	108.0520971
UIJ65383.1	hypothetical_protein_LW858_20905_[Bacillus_cereus]	CPTF_Al	226398.6667	60601.72833	26.76770549
UIJ65383.1	hypothetical_protein_LW858_20905_[Bacillus_cereus]	CPTF_Cd	318719	46730.41446	14.66194813
UIJ65383.1	hypothetical_protein_LW858_20905_[Bacillus_cereus]	CPTF_Co	271965.6667	4537.86308	1.668542627
UIJ65383.1	hypothetical_protein_LW858_20905_[Bacillus_cereus]	CPTF_Cu	461435	78850.5392	17.08811408
UIJ65383.1	hypothetical_protein_LW858_20905_[Bacillus_cereus]	CPTF_Fe	280444	49329.91811	17.58993529
UIJ65383.1	hypothetical_protein_LW858_20905_[Bacillus_cereus]	CPTF_Mn	275702.3333	28203.89665	10.22983604
UIJ65383.1	hypothetical_protein_LW858_20905_[Bacillus_cereus]	CPTF_Ni	304806.6667	51735.36127	16.97317248
UIJ65383.1	hypothetical_protein_LW858_20905_[Bacillus_cereus]	CPTF_U	334118	35724.13543	10.69207149
UIJ65383.1	hypothetical_protein_LW858_20905_[Bacillus_cereus]	CPTF_metals_mix	517232.3333	45480.39689	8.793030513
UIJ65383.1	hypothetical_protein_LW858_20905_[Bacillus_cereus]	CPTF_zcontrol	271981.6667	124908.8814	45.92547832
UIJ65389.1	hypothetical_protein_LW858_20935_[Bacillus_cereus]	CPTF_Al	0	0	0
UIJ65389.1	hypothetical_protein_LW858_20935_[Bacillus_cereus]	CPTF_Cd	6010.7	10410.83779	173.2050808
UIJ65389.1	hypothetical_protein_LW858_20935_[Bacillus_cereus]	CPTF_Co	0	0	0
UIJ65389.1	hypothetical_protein_LW858_20935_[Bacillus_cereus]	CPTF_Cu	0	0	0
UIJ65389.1	hypothetical_protein_LW858_20935_[Bacillus_cereus]	CPTF_Fe	0	0	0
UIJ65389.1	hypothetical_protein_LW858_20935_[Bacillus_cereus]	CPTF_Mn	0	0	0
UIJ65389.1	hypothetical_protein_LW858_20935_[Bacillus_cereus]	CPTF_Ni	0	0	0
UIJ65389.1	hypothetical_protein_LW858_20935_[Bacillus_cereus]	CPTF_U	0	0	0
UIJ65389.1	hypothetical_protein_LW858_20935_[Bacillus_cereus]	CPTF_metals_mix	1020653.5	730690.59	71.59046533
UIJ65389.1	hypothetical_protein_LW858_20935_[Bacillus_cereus]	CPTF_zcontrol	0	0	0
UIJ65395.1	XRE_family_transcriptional_regulator_[Bacillus_cereus]	CPTF_Al	312612	198483.2308	63.49187838
UIJ65395.1	XRE_family_transcriptional_regulator_[Bacillus_cereus]	CPTF_Cd	70956.66667	122900.5518	173.2050808
UIJ65395.1	XRE_family_transcriptional_regulator_[Bacillus_cereus]	CPTF_Co	0	0	0
UIJ65395.1	XRE_family_transcriptional_regulator_[Bacillus_cereus]	CPTF_Cu	113582.0333	56101.77507	49.39317727
UIJ65395.1	XRE_family_transcriptional_regulator_[Bacillus_cereus]	CPTF_Fe	80094.66667	138728.0321	173.2050808

UIJ65395.1	XRE_family_transcriptional_regulator_[Bacillus_cereus]	CPTF_Mn	136800.9333	79112.78443	57.83058822
UIJ65395.1	XRE_family_transcriptional_regulator_[Bacillus_cereus]	CPTF_Ni	113589	102549.341	90.28104923
UIJ65395.1	XRE_family_transcriptional_regulator_[Bacillus_cereus]	CPTF_U	64769.66667	112184.3535	173.2050808
UIJ65395.1	XRE_family_transcriptional_regulator_[Bacillus_cereus]	CPTF_metals_mix	152277	158908.4573	104.3548647
UIJ65395.1	XRE_family_transcriptional_regulator_[Bacillus_cereus]	CPTF_zcontrol	255796.2333	200679.5022	78.45287619
UIJ65399.1	bifunctional_tetrahydrofolate_synthase/dihydrofolate_synthase_[Bacillus_cereus]	CPTF_Al	0	0	0
UIJ65399.1	bifunctional_tetrahydrofolate_synthase/dihydrofolate_synthase_[Bacillus_cereus]	CPTF_Cd	0	0	0
UIJ65399.1	bifunctional_tetrahydrofolate_synthase/dihydrofolate_synthase_[Bacillus_cereus]	CPTF_Co	0	0	0
UIJ65399.1	bifunctional_tetrahydrofolate_synthase/dihydrofolate_synthase_[Bacillus_cereus]	CPTF_Cu	0	0	0
UIJ65399.1	bifunctional_tetrahydrofolate_synthase/dihydrofolate_synthase_[Bacillus_cereus]	CPTF_Fe	0	0	0
UIJ65399.1	bifunctional_tetrahydrofolate_synthase/dihydrofolate_synthase_[Bacillus_cereus]	CPTF_Mn	0	0	0
UIJ65399.1	bifunctional_tetrahydrofolate_synthase/dihydrofolate_synthase_[Bacillus_cereus]	CPTF_Ni	0	0	0
UIJ65399.1	bifunctional_tetrahydrofolate_synthase/dihydrofolate_synthase_[Bacillus_cereus]	CPTF_U	0	0	0
UIJ65399.1	bifunctional_tetrahydrofolate_synthase/dihydrofolate_synthase_[Bacillus_cereus]	CPTF_metals_mix	10825.46667	18750.25828	173.2050808
UIJ65399.1	bifunctional_tetrahydrofolate_synthase/dihydrofolate_synthase_[Bacillus_cereus]	CPTF_zcontrol	0	0	0
UIJ65400.1	valine--tRNA_ligase_[Bacillus_cereus]	CPTF_Al	5514184.6	1184611.966	21.48299435
UIJ65400.1	valine--tRNA_ligase_[Bacillus_cereus]	CPTF_Cd	6648240.367	149261.2078	2.245123515
UIJ65400.1	valine--tRNA_ligase_[Bacillus_cereus]	CPTF_Co	6422738.1	318814.2722	4.963837342
UIJ65400.1	valine--tRNA_ligase_[Bacillus_cereus]	CPTF_Cu	6004330.8	407434.9042	6.78568383
UIJ65400.1	valine--tRNA_ligase_[Bacillus_cereus]	CPTF_Fe	5732825.733	245057.1153	4.274630465
UIJ65400.1	valine--tRNA_ligase_[Bacillus_cereus]	CPTF_Mn	6248461.7	258279.7114	4.133492751
UIJ65400.1	valine--tRNA_ligase_[Bacillus_cereus]	CPTF_Ni	5435926.567	357094.9449	6.569164254
UIJ65400.1	valine--tRNA_ligase_[Bacillus_cereus]	CPTF_U	5960278.033	803553.3747	13.48181025
UIJ65400.1	valine--tRNA_ligase_[Bacillus_cereus]	CPTF_metals_mix	9150733.467	117072.8481	1.279382123
UIJ65400.1	valine--tRNA_ligase_[Bacillus_cereus]	CPTF_zcontrol	5457505.533	1634653.961	29.95240135
UIJ65403.1	glutamate-1-semialdehyde_2,1-aminomutase_[Bacillus_cereus]	CPTF_Al	2820036	441524.6826	15.65670377
UIJ65403.1	glutamate-1-semialdehyde_2,1-aminomutase_[Bacillus_cereus]	CPTF_Cd	3620104	180093.7138	4.974821547
UIJ65403.1	glutamate-1-semialdehyde_2,1-aminomutase_[Bacillus_cereus]	CPTF_Co	2875001.667	549247.9305	19.10426477
UIJ65403.1	glutamate-1-semialdehyde_2,1-aminomutase_[Bacillus_cereus]	CPTF_Cu	3020409.867	303775.3384	10.05742107
UIJ65403.1	glutamate-1-semialdehyde_2,1-aminomutase_[Bacillus_cereus]	CPTF_Fe	2933202.667	476025.3407	16.22885954
UIJ65403.1	glutamate-1-semialdehyde_2,1-aminomutase_[Bacillus_cereus]	CPTF_Mn	2596088.267	682146.5134	26.27593684
UIJ65403.1	glutamate-1-semialdehyde_2,1-aminomutase_[Bacillus_cereus]	CPTF_Ni	2688877.667	209870.9058	7.805148905
UIJ65403.1	glutamate-1-semialdehyde_2,1-aminomutase_[Bacillus_cereus]	CPTF_U	1973271.333	516581.4244	26.17893524
UIJ65403.1	glutamate-1-semialdehyde_2,1-aminomutase_[Bacillus_cereus]	CPTF_metals_mix	3806854.567	161763.4646	4.249268307
UIJ65403.1	glutamate-1-semialdehyde_2,1-aminomutase_[Bacillus_cereus]	CPTF_zcontrol	2853840.1	463371.872	16.23678468
UIJ65404.1	porphobilinogen_synthase_[Bacillus_cereus]	CPTF_Al	3264148.2	243729.13	7.466852455
UIJ65404.1	porphobilinogen_synthase_[Bacillus_cereus]	CPTF_Cd	3030944.067	418810.7915	13.81783307
UIJ65404.1	porphobilinogen_synthase_[Bacillus_cereus]	CPTF_Co	2845590.1	529674.4065	18.61386875
UIJ65404.1	porphobilinogen_synthase_[Bacillus_cereus]	CPTF_Cu	3110266.6	119309.919	3.836002965
UIJ65404.1	porphobilinogen_synthase_[Bacillus_cereus]	CPTF_Fe	2417538.833	41750.38797	1.726979
UIJ65404.1	porphobilinogen_synthase_[Bacillus_cereus]	CPTF_Mn	3213942.5	181928.5089	5.660602482
UIJ65404.1	porphobilinogen_synthase_[Bacillus_cereus]	CPTF_Ni	2461647.2	254299.707	10.33046925
UIJ65404.1	porphobilinogen_synthase_[Bacillus_cereus]	CPTF_U	2697806.967	321356.7373	11.91177654
UIJ65404.1	porphobilinogen_synthase_[Bacillus_cereus]	CPTF_metals_mix	3173961.967	249576.2534	7.863240202
UIJ65404.1	porphobilinogen_synthase_[Bacillus_cereus]	CPTF_zcontrol	3162155.2	180416.0179	5.705476377
UIJ65406.1	hydroxymethylbilane_synthase_[Bacillus_cereus]	CPTF_Al	1768090.5	225653.003	12.76252562
UIJ65406.1	hydroxymethylbilane_synthase_[Bacillus_cereus]	CPTF_Cd	1555966.2	219627.3111	14.11517237
UIJ65406.1	hydroxymethylbilane_synthase_[Bacillus_cereus]	CPTF_Co	1555219.9	95001.10668	6.108532091
UIJ65406.1	hydroxymethylbilane_synthase_[Bacillus_cereus]	CPTF_Cu	1699174.667	354703.6686	20.87505632
UIJ65406.1	hydroxymethylbilane_synthase_[Bacillus_cereus]	CPTF_Fe	1626128.4	190011.9345	11.68492811
UIJ65406.1	hydroxymethylbilane_synthase_[Bacillus_cereus]	CPTF_Mn	1578089.167	332682.5512	21.08135321
UIJ65406.1	hydroxymethylbilane_synthase_[Bacillus_cereus]	CPTF_Ni	1922313.967	151650.8739	7.888975296
UIJ65406.1	hydroxymethylbilane_synthase_[Bacillus_cereus]	CPTF_U	1159097.933	290680.5878	25.07817325
UIJ65406.1	hydroxymethylbilane_synthase_[Bacillus_cereus]	CPTF_metals_mix	2720863.9	142964.5976	5.254382536
UIJ65406.1	hydroxymethylbilane_synthase_[Bacillus_cereus]	CPTF_zcontrol	1667854.867	191619.5899	11.48898467
UIJ65410.1	organic_hydroperoxide_resistance_protein_[Bacillus_cereus]	CPTF_Al	763921.6667	89988.79917	11.77984643
UIJ65410.1	organic_hydroperoxide_resistance_protein_[Bacillus_cereus]	CPTF_Cd	960157	81382.3709	8.475944132
UIJ65410.1	organic_hydroperoxide_resistance_protein_[Bacillus_cereus]	CPTF_Co	720883	117177.7661	16.25475509
UIJ65410.1	organic_hydroperoxide_resistance_protein_[Bacillus_cereus]	CPTF_Cu	758531.3333	83911.66839	11.06238658
UIJ65410.1	organic_hydroperoxide_resistance_protein_[Bacillus_cereus]	CPTF_Fe	786915	51728.46216	6.573576836
UIJ65410.1	organic_hydroperoxide_resistance_protein_[Bacillus_cereus]	CPTF_Mn	709235.6667	171100.3283	24.12460855

UIJ65410.1	organic_hydroperoxide_resistance_protein_[Bacillus_cereus]	CPTF_Ni	830108.6667	88289.45849	10.63589167
UIJ65410.1	organic_hydroperoxide_resistance_protein_[Bacillus_cereus]	CPTF_U	543237.3333	68845.22642	12.67313975
UIJ65410.1	organic_hydroperoxide_resistance_protein_[Bacillus_cereus]	CPTF_metals_mix	1003669.667	118588.6505	11.81550608
UIJ65410.1	organic_hydroperoxide_resistance_protein_[Bacillus_cereus]	CPTF_zcontrol	586758.6667	120036.5651	20.45757002
UIJ65412.1	endopeptidase_La_[Bacillus_cereus]	CPTF_Al	1296680.967	372586.1739	28.73383534
UIJ65412.1	endopeptidase_La_[Bacillus_cereus]	CPTF_Cd	1832781.233	246962.2132	13.47472402
UIJ65412.1	endopeptidase_La_[Bacillus_cereus]	CPTF_Co	1399017.433	130388.0563	9.319973662
UIJ65412.1	endopeptidase_La_[Bacillus_cereus]	CPTF_Cu	1345290.433	244183.9672	18.15102235
UIJ65412.1	endopeptidase_La_[Bacillus_cereus]	CPTF_Fe	1497231.3	422188.7732	28.19796602
UIJ65412.1	endopeptidase_La_[Bacillus_cereus]	CPTF_Mn	1515996.433	530970.9506	35.02455144
UIJ65412.1	endopeptidase_La_[Bacillus_cereus]	CPTF_Ni	875619.1333	18090.36554	2.06600848
UIJ65412.1	endopeptidase_La_[Bacillus_cereus]	CPTF_U	1090547.7	197355.091	18.09687839
UIJ65412.1	endopeptidase_La_[Bacillus_cereus]	CPTF_metals_mix	3199197.167	421400.8624	13.17208163
UIJ65412.1	endopeptidase_La_[Bacillus_cereus]	CPTF_zcontrol	1325965.433	503371.2162	37.96261981
UIJ65414.1	ATP-dependent_Clp_protease_ATP-binding_subunit_ClpX_[Bacillus_cereus]	CPTF_Al	7866944.867	466124.2241	5.925098396
UIJ65414.1	ATP-dependent_Clp_protease_ATP-binding_subunit_ClpX_[Bacillus_cereus]	CPTF_Cd	6590724.133	471843.5467	7.159206442
UIJ65414.1	ATP-dependent_Clp_protease_ATP-binding_subunit_ClpX_[Bacillus_cereus]	CPTF_Co	8397783.333	456025.7078	5.430310473
UIJ65414.1	ATP-dependent_Clp_protease_ATP-binding_subunit_ClpX_[Bacillus_cereus]	CPTF_Cu	7861386.4	189926.6992	2.415944078
UIJ65414.1	ATP-dependent_Clp_protease_ATP-binding_subunit_ClpX_[Bacillus_cereus]	CPTF_Fe	7505894.033	335309.5972	4.46728392
UIJ65414.1	ATP-dependent_Clp_protease_ATP-binding_subunit_ClpX_[Bacillus_cereus]	CPTF_Mn	8026331.267	85224.40898	1.061810261
UIJ65414.1	ATP-dependent_Clp_protease_ATP-binding_subunit_ClpX_[Bacillus_cereus]	CPTF_Ni	7827084.6	228845.356	2.923762393
UIJ65414.1	ATP-dependent_Clp_protease_ATP-binding_subunit_ClpX_[Bacillus_cereus]	CPTF_U	7718686.233	458254.8929	5.936954541
UIJ65414.1	ATP-dependent_Clp_protease_ATP-binding_subunit_ClpX_[Bacillus_cereus]	CPTF_metals_mix	7229791.9	348252.0417	4.816902706
UIJ65414.1	ATP-dependent_Clp_protease_ATP-binding_subunit_ClpX_[Bacillus_cereus]	CPTF_zcontrol	8537090.1	454479.7631	5.323591033
UIJ65415.1	trigger_factor_[Bacillus_cereus]	CPTF_Al	24935176.57	702835.3803	2.818650104
UIJ65415.1	trigger_factor_[Bacillus_cereus]	CPTF_Cd	26613254.97	1159149.217	4.355533429
UIJ65415.1	trigger_factor_[Bacillus_cereus]	CPTF_Co	24673175.23	558349.3477	2.262981325
UIJ65415.1	trigger_factor_[Bacillus_cereus]	CPTF_Cu	22648716	753989.1607	3.329059187
UIJ65415.1	trigger_factor_[Bacillus_cereus]	CPTF_Fe	25168992.57	1909384.465	7.586257017
UIJ65415.1	trigger_factor_[Bacillus_cereus]	CPTF_Mn	25760286.47	2290941.011	8.893305648
UIJ65415.1	trigger_factor_[Bacillus_cereus]	CPTF_Ni	21353037.13	193434.0341	0.905885345
UIJ65415.1	trigger_factor_[Bacillus_cereus]	CPTF_U	23187462.2	1399130.381	6.033995307
UIJ65415.1	trigger_factor_[Bacillus_cereus]	CPTF_metals_mix	26051116.87	1980723.928	7.60322077
UIJ65415.1	trigger_factor_[Bacillus_cereus]	CPTF_zcontrol	25499265.7	1350367.3	5.295710534
UIJ65416.1	hypothetical_protein_LW858_21075_[Bacillus_cereus]	CPTF_Al	87119.26667	51595.3576	59.22381991
UIJ65416.1	hypothetical_protein_LW858_21075_[Bacillus_cereus]	CPTF_Cd	84366.9	43432.51765	51.4805186
UIJ65416.1	hypothetical_protein_LW858_21075_[Bacillus_cereus]	CPTF_Co	82313.73333	14916.26839	18.12123905
UIJ65416.1	hypothetical_protein_LW858_21075_[Bacillus_cereus]	CPTF_Cu	101244.0333	51346.81457	50.71589197
UIJ65416.1	hypothetical_protein_LW858_21075_[Bacillus_cereus]	CPTF_Fe	64637.93333	41820.799	64.70008685
UIJ65416.1	hypothetical_protein_LW858_21075_[Bacillus_cereus]	CPTF_Mn	53974.4	62129.38772	115.1089919
UIJ65416.1	hypothetical_protein_LW858_21075_[Bacillus_cereus]	CPTF_Ni	84761.66667	60399.4153	71.25793731
UIJ65416.1	hypothetical_protein_LW858_21075_[Bacillus_cereus]	CPTF_U	60033	56763.98354	94.55463418
UIJ65416.1	hypothetical_protein_LW858_21075_[Bacillus_cereus]	CPTF_metals_mix	56089.93333	19208.41849	34.24575027
UIJ65416.1	hypothetical_protein_LW858_21075_[Bacillus_cereus]	CPTF_zcontrol	68535.4	26322.61177	38.40732201
UIJ65418.1	PH_domain-containing_protein_[Bacillus_cereus]	CPTF_Al	2066834.133	265405.415	12.84115695
UIJ65418.1	PH_domain-containing_protein_[Bacillus_cereus]	CPTF_Cd	2191189	402037.1603	18.34789971
UIJ65418.1	PH_domain-containing_protein_[Bacillus_cereus]	CPTF_Co	2020607.733	562034.4114	27.81511731
UIJ65418.1	PH_domain-containing_protein_[Bacillus_cereus]	CPTF_Cu	1980244.9	91286.82001	4.609875274
UIJ65418.1	PH_domain-containing_protein_[Bacillus_cereus]	CPTF_Fe	2247263	251130.2353	11.17493748
UIJ65418.1	PH_domain-containing_protein_[Bacillus_cereus]	CPTF_Mn	1948836	201957.9128	10.36300196
UIJ65418.1	PH_domain-containing_protein_[Bacillus_cereus]	CPTF_Ni	1983084.333	867271.0695	43.73344365
UIJ65418.1	PH_domain-containing_protein_[Bacillus_cereus]	CPTF_U	2008817.167	240184.58	11.9565177
UIJ65418.1	PH_domain-containing_protein_[Bacillus_cereus]	CPTF_metals_mix	1930457.333	106558.561	5.519860977
UIJ65418.1	PH_domain-containing_protein_[Bacillus_cereus]	CPTF_zcontrol	1901909.933	310381.56	16.31946679
UIJ65421.1	XTP/dITP_diphosphatase_[Bacillus_cereus]	CPTF_Al	166822.0333	39588.54585	23.73100546
UIJ65421.1	XTP/dITP_diphosphatase_[Bacillus_cereus]	CPTF_Cd	197447.7	82564.03242	41.81564659
UIJ65421.1	XTP/dITP_diphosphatase_[Bacillus_cereus]	CPTF_Co	264385.7667	12694.22782	4.801403638
UIJ65421.1	XTP/dITP_diphosphatase_[Bacillus_cereus]	CPTF_Cu	194084.4667	42384.46482	21.83815405
UIJ65421.1	XTP/dITP_diphosphatase_[Bacillus_cereus]	CPTF_Fe	224632.1	74519.14046	33.17386093
UIJ65421.1	XTP/dITP_diphosphatase_[Bacillus_cereus]	CPTF_Mn	208169.5667	19736.30298	9.480878161
UIJ65421.1	XTP/dITP_diphosphatase_[Bacillus_cereus]	CPTF_Ni	37214.33333	64457.1161	173.2050808

UIJ65421.1	XTP/dITP_diphosphatase_[Bacillus_cereus]	CPTF_U	139836.3333	127276.3653	91.01809401
UIJ65421.1	XTP/dITP_diphosphatase_[Bacillus_cereus]	CPTF_metals_mix	303682.6567	95410.85445	31.41794645
UIJ65421.1	XTP/dITP_diphosphatase_[Bacillus_cereus]	CPTF_zcontrol	175171.1667	79331.66165	45.28808203
UIJ65422.1	ribonuclease_PH_[Bacillus_cereus]	CPTF_Al	89863.5	65281.66418	72.64536122
UIJ65422.1	ribonuclease_PH_[Bacillus_cereus]	CPTF_Cd	41914.76667	6364.813074	15.18513302
UIJ65422.1	ribonuclease_PH_[Bacillus_cereus]	CPTF_Co	117884.6	67877.87526	57.57993433
UIJ65422.1	ribonuclease_PH_[Bacillus_cereus]	CPTF_Cu	103732.2333	25283.41993	24.37373526
UIJ65422.1	ribonuclease_PH_[Bacillus_cereus]	CPTF_Fe	63455.46667	4615.212244	7.273151528
UIJ65422.1	ribonuclease_PH_[Bacillus_cereus]	CPTF_Mn	87065.3	124372.7926	142.8500132
UIJ65422.1	ribonuclease_PH_[Bacillus_cereus]	CPTF_Ni	63683.03333	8919.259986	14.00570846
UIJ65422.1	ribonuclease_PH_[Bacillus_cereus]	CPTF_U	47049.23333	42661.85747	90.67492591
UIJ65422.1	ribonuclease_PH_[Bacillus_cereus]	CPTF_metals_mix	486039.7667	32022.43526	6.588439353
UIJ65422.1	ribonuclease_PH_[Bacillus_cereus]	CPTF_zcontrol	0	0	0
UIJ65424.1	glutamate_racemase_[Bacillus_cereus]	CPTF_Al	0	0	0
UIJ65424.1	glutamate_racemase_[Bacillus_cereus]	CPTF_Cd	0	0	0
UIJ65424.1	glutamate_racemase_[Bacillus_cereus]	CPTF_Co	0	0	0
UIJ65424.1	glutamate_racemase_[Bacillus_cereus]	CPTF_Cu	0	0	0
UIJ65424.1	glutamate_racemase_[Bacillus_cereus]	CPTF_Fe	0	0	0
UIJ65424.1	glutamate_racemase_[Bacillus_cereus]	CPTF_Mn	15742.73333	27267.21398	173.2050808
UIJ65424.1	glutamate_racemase_[Bacillus_cereus]	CPTF_Ni	0	0	0
UIJ65424.1	glutamate_racemase_[Bacillus_cereus]	CPTF_U	0	0	0
UIJ65424.1	glutamate_racemase_[Bacillus_cereus]	CPTF_metals_mix	232183.6667	24767.78973	10.66732647
UIJ65424.1	glutamate_racemase_[Bacillus_cereus]	CPTF_zcontrol	0	0	0
UIJ65438.1	5'-nucleotidase,_lipoprotein_e(P4)_family_[Bacillus_cereus]	CPTF_Al	160397	20453.2538	12.75164361
UIJ65438.1	5'-nucleotidase,_lipoprotein_e(P4)_family_[Bacillus_cereus]	CPTF_Cd	103055.3333	19859.14929	19.27037509
UIJ65438.1	5'-nucleotidase,_lipoprotein_e(P4)_family_[Bacillus_cereus]	CPTF_Co	142346	19922.8575	13.99607822
UIJ65438.1	5'-nucleotidase,_lipoprotein_e(P4)_family_[Bacillus_cereus]	CPTF_Cu	58742.16667	14505.58837	24.69365567
UIJ65438.1	5'-nucleotidase,_lipoprotein_e(P4)_family_[Bacillus_cereus]	CPTF_Fe	152971.6667	9490.838231	6.204311189
UIJ65438.1	5'-nucleotidase,_lipoprotein_e(P4)_family_[Bacillus_cereus]	CPTF_Mn	144084.6	51276.713	35.58792057
UIJ65438.1	5'-nucleotidase,_lipoprotein_e(P4)_family_[Bacillus_cereus]	CPTF_Ni	135576.6667	15510.13173	11.4401188
UIJ65438.1	5'-nucleotidase,_lipoprotein_e(P4)_family_[Bacillus_cereus]	CPTF_U	126139.3333	109918.6762	87.14068265
UIJ65438.1	5'-nucleotidase,_lipoprotein_e(P4)_family_[Bacillus_cereus]	CPTF_metals_mix	74602.23333	57443.37358	76.99953609
UIJ65438.1	5'-nucleotidase,_lipoprotein_e(P4)_family_[Bacillus_cereus]	CPTF_zcontrol	140594.4	69661.10857	49.54756987
UIJ65442.1	acyl-CoA_thioesterase_[Bacillus_cereus]	CPTF_Al	0	0	0
UIJ65442.1	acyl-CoA_thioesterase_[Bacillus_cereus]	CPTF_Cd	0	0	0
UIJ65442.1	acyl-CoA_thioesterase_[Bacillus_cereus]	CPTF_Co	0	0	0
UIJ65442.1	acyl-CoA_thioesterase_[Bacillus_cereus]	CPTF_Cu	0	0	0
UIJ65442.1	acyl-CoA_thioesterase_[Bacillus_cereus]	CPTF_Fe	0	0	0
UIJ65442.1	acyl-CoA_thioesterase_[Bacillus_cereus]	CPTF_Mn	81935.66667	141916.7376	173.2050808
UIJ65442.1	acyl-CoA_thioesterase_[Bacillus_cereus]	CPTF_Ni	0	0	0
UIJ65442.1	acyl-CoA_thioesterase_[Bacillus_cereus]	CPTF_U	0	0	0
UIJ65442.1	acyl-CoA_thioesterase_[Bacillus_cereus]	CPTF_metals_mix	0	0	0
UIJ65442.1	acyl-CoA_thioesterase_[Bacillus_cereus]	CPTF_zcontrol	0	0	0
UIJ65443.1	succinate_dehydrogenase_iron-sulfur_subunit_[Bacillus_cereus]	CPTF_Al	1230138	234178.4286	19.0367608
UIJ65443.1	succinate_dehydrogenase_iron-sulfur_subunit_[Bacillus_cereus]	CPTF_Cd	1929577.867	228747.7441	11.85480762
UIJ65443.1	succinate_dehydrogenase_iron-sulfur_subunit_[Bacillus_cereus]	CPTF_Co	1518501	254656.9694	16.77028658
UIJ65443.1	succinate_dehydrogenase_iron-sulfur_subunit_[Bacillus_cereus]	CPTF_Cu	1339234	67369.09647	5.030420111
UIJ65443.1	succinate_dehydrogenase_iron-sulfur_subunit_[Bacillus_cereus]	CPTF_Fe	1496876.333	197596.9215	13.20061765
UIJ65443.1	succinate_dehydrogenase_iron-sulfur_subunit_[Bacillus_cereus]	CPTF_Mn	1766916.5	487531.227	27.59220524
UIJ65443.1	succinate_dehydrogenase_iron-sulfur_subunit_[Bacillus_cereus]	CPTF_Ni	1104529.333	474887.1913	42.99452961
UIJ65443.1	succinate_dehydrogenase_iron-sulfur_subunit_[Bacillus_cereus]	CPTF_U	943898.8667	448672.8911	47.5340004
UIJ65443.1	succinate_dehydrogenase_iron-sulfur_subunit_[Bacillus_cereus]	CPTF_metals_mix	1645922.133	44929.9131	2.729771488
UIJ65443.1	succinate_dehydrogenase_iron-sulfur_subunit_[Bacillus_cereus]	CPTF_zcontrol	1269104.1	127807.3291	10.0706734
UIJ65444.1	succinate_dehydrogenase_flavoprotein_subunit_[Bacillus_cereus]	CPTF_Al	14215126.87	1001048.027	7.042132205
UIJ65444.1	succinate_dehydrogenase_flavoprotein_subunit_[Bacillus_cereus]	CPTF_Cd	15557476.6	593614.9022	3.815624586
UIJ65444.1	succinate_dehydrogenase_flavoprotein_subunit_[Bacillus_cereus]	CPTF_Co	13810507.03	1243109.891	9.00118937
UIJ65444.1	succinate_dehydrogenase_flavoprotein_subunit_[Bacillus_cereus]	CPTF_Cu	12477118.47	529295.3729	4.242128295
UIJ65444.1	succinate_dehydrogenase_flavoprotein_subunit_[Bacillus_cereus]	CPTF_Fe	13865064.07	122719.3023	0.885097261
UIJ65444.1	succinate_dehydrogenase_flavoprotein_subunit_[Bacillus_cereus]	CPTF_Mn	14040972.97	2251588.1	16.035841
UIJ65444.1	succinate_dehydrogenase_flavoprotein_subunit_[Bacillus_cereus]	CPTF_Ni	13856363.6	831459.8854	6.000563419
UIJ65444.1	succinate_dehydrogenase_flavoprotein_subunit_[Bacillus_cereus]	CPTF_U	14128591.4	645299.867	4.567333351

UIJ65444.1	succinate_dehydrogenase_flavoprotein_subunit_[Bacillus_cereus]	CPTF_metals_mix	12178374.07	1682027.183	13.81159072
UIJ65444.1	succinate_dehydrogenase_flavoprotein_subunit_[Bacillus_cereus]	CPTF_zcontrol	15450807.07	368589.609	2.385568646
UIJ65445.1	succinate_dehydrogenase_cytochrome_B558_[Bacillus_cereus]	CPTF_Al	0	0	0
UIJ65445.1	succinate_dehydrogenase_cytochrome_B558_[Bacillus_cereus]	CPTF_Cd	43340.86667	39096.12056	90.20613468
UIJ65445.1	succinate_dehydrogenase_cytochrome_B558_[Bacillus_cereus]	CPTF_Co	23468.73333	9388.070913	40.00246106
UIJ65445.1	succinate_dehydrogenase_cytochrome_B558_[Bacillus_cereus]	CPTF_Cu	0	0	0
UIJ65445.1	succinate_dehydrogenase_cytochrome_B558_[Bacillus_cereus]	CPTF_Fe	0	0	0
UIJ65445.1	succinate_dehydrogenase_cytochrome_B558_[Bacillus_cereus]	CPTF_Mn	0	0	0
UIJ65445.1	succinate_dehydrogenase_cytochrome_B558_[Bacillus_cereus]	CPTF_Ni	0	0	0
UIJ65445.1	succinate_dehydrogenase_cytochrome_B558_[Bacillus_cereus]	CPTF_U	0	0	0
UIJ65445.1	succinate_dehydrogenase_cytochrome_B558_[Bacillus_cereus]	CPTF_metals_mix	158096.6667	13426.77721	8.492764267
UIJ65445.1	succinate_dehydrogenase_cytochrome_B558_[Bacillus_cereus]	CPTF_zcontrol	0	0	0
UIJ65448.1	thioredoxin_[Bacillus_cereus]	CPTF_Al	1747094.333	182712.5621	10.45808224
UIJ65448.1	thioredoxin_[Bacillus_cereus]	CPTF_Cd	2210711	303879.6963	13.74579022
UIJ65448.1	thioredoxin_[Bacillus_cereus]	CPTF_Co	1705838.333	206117.4072	12.08305636
UIJ65448.1	thioredoxin_[Bacillus_cereus]	CPTF_Cu	1744356	173342.7308	9.937348272
UIJ65448.1	thioredoxin_[Bacillus_cereus]	CPTF_Fe	1725380.667	308415.7599	17.87522985
UIJ65448.1	thioredoxin_[Bacillus_cereus]	CPTF_Mn	1883862.667	770132.3607	40.8804938
UIJ65448.1	thioredoxin_[Bacillus_cereus]	CPTF_Ni	1373172	189506.0649	13.80060654
UIJ65448.1	thioredoxin_[Bacillus_cereus]	CPTF_U	1352917.4	590298.4769	43.63152376
UIJ65448.1	thioredoxin_[Bacillus_cereus]	CPTF_metals_mix	4824637.133	682882.7734	14.15407531
UIJ65448.1	thioredoxin_[Bacillus_cereus]	CPTF_zcontrol	1458873.667	167150.4731	11.45750156
UIJ65449.1	electron_transfer_flavoprotein_subunit_alpha_[Bacillus_cereus]	CPTF_Al	12335881.33	448272.2291	3.633888953
UIJ65449.1	electron_transfer_flavoprotein_subunit_alpha_[Bacillus_cereus]	CPTF_Cd	12339237	2393211.295	19.39513193
UIJ65449.1	electron_transfer_flavoprotein_subunit_alpha_[Bacillus_cereus]	CPTF_Co	8971462.267	2178966.707	24.28775424
UIJ65449.1	electron_transfer_flavoprotein_subunit_alpha_[Bacillus_cereus]	CPTF_Cu	9305619.867	1404396.629	15.09191917
UIJ65449.1	electron_transfer_flavoprotein_subunit_alpha_[Bacillus_cereus]	CPTF_Fe	10897411.87	1664193.856	15.27145965
UIJ65449.1	electron_transfer_flavoprotein_subunit_alpha_[Bacillus_cereus]	CPTF_Mn	10949211.77	3004039.994	27.43613018
UIJ65449.1	electron_transfer_flavoprotein_subunit_alpha_[Bacillus_cereus]	CPTF_Ni	8283102.433	1116094.713	13.47435604
UIJ65449.1	electron_transfer_flavoprotein_subunit_alpha_[Bacillus_cereus]	CPTF_U	9354237.133	1394935.076	14.91233391
UIJ65449.1	electron_transfer_flavoprotein_subunit_alpha_[Bacillus_cereus]	CPTF_metals_mix	14319852.1	2441527.254	17.04994742
UIJ65449.1	electron_transfer_flavoprotein_subunit_alpha_[Bacillus_cereus]	CPTF_zcontrol	10979316.2	121752.3239	1.108924469
UIJ65450.1	electron_transfer_flavoprotein_subunit_beta_[Bacillus_cereus]	CPTF_Al	13121130.47	185424.1453	1.413172026
UIJ65450.1	electron_transfer_flavoprotein_subunit_beta_[Bacillus_cereus]	CPTF_Cd	13383796.97	298013.7599	2.226675738
UIJ65450.1	electron_transfer_flavoprotein_subunit_beta_[Bacillus_cereus]	CPTF_Co	10704323.17	621667.3126	5.807628403
UIJ65450.1	electron_transfer_flavoprotein_subunit_beta_[Bacillus_cereus]	CPTF_Cu	10850340	877791.1485	8.089987489
UIJ65450.1	electron_transfer_flavoprotein_subunit_beta_[Bacillus_cereus]	CPTF_Fe	12634567.3	582473.2871	4.610156195
UIJ65450.1	electron_transfer_flavoprotein_subunit_beta_[Bacillus_cereus]	CPTF_Mn	13112645.93	908614.3503	6.929298289
UIJ65450.1	electron_transfer_flavoprotein_subunit_beta_[Bacillus_cereus]	CPTF_Ni	11708088.67	289609.44	2.473584274
UIJ65450.1	electron_transfer_flavoprotein_subunit_beta_[Bacillus_cereus]	CPTF_U	11643923.67	350638.0218	3.011339062
UIJ65450.1	electron_transfer_flavoprotein_subunit_beta_[Bacillus_cereus]	CPTF_metals_mix	12844005.6	788708.2566	6.140672008
UIJ65450.1	electron_transfer_flavoprotein_subunit_beta_[Bacillus_cereus]	CPTF_zcontrol	10922278.33	401273.1488	3.673896018
UIJ65451.1	enoyl-CoA_hydratase_[Bacillus_cereus]	CPTF_Al	5724897.333	234424.0897	4.094817358
UIJ65451.1	enoyl-CoA_hydratase_[Bacillus_cereus]	CPTF_Cd	5686955	363699.4079	6.395327692
UIJ65451.1	enoyl-CoA_hydratase_[Bacillus_cereus]	CPTF_Co	5425479.333	213872.691	3.94200545
UIJ65451.1	enoyl-CoA_hydratase_[Bacillus_cereus]	CPTF_Cu	5729802.667	62994.76359	1.099422917
UIJ65451.1	enoyl-CoA_hydratase_[Bacillus_cereus]	CPTF_Fe	5529952	296575.0874	5.363068023
UIJ65451.1	enoyl-CoA_hydratase_[Bacillus_cereus]	CPTF_Mn	5293575.2	568313.8187	10.7359166
UIJ65451.1	enoyl-CoA_hydratase_[Bacillus_cereus]	CPTF_Ni	5309612	558153.8604	10.51214025
UIJ65451.1	enoyl-CoA_hydratase_[Bacillus_cereus]	CPTF_U	4662293.333	277383.545	5.949508648
UIJ65451.1	enoyl-CoA_hydratase_[Bacillus_cereus]	CPTF_metals_mix	5559654.833	625879.0012	11.25751544
UIJ65451.1	enoyl-CoA_hydratase_[Bacillus_cereus]	CPTF_zcontrol	4851384.667	90325.63093	1.861852587
UIJ65452.1	TetR_family_transcriptional_regulator_[Bacillus_cereus]	CPTF_Al	10321.43333	17877.24694	173.2050808
UIJ65452.1	TetR_family_transcriptional_regulator_[Bacillus_cereus]	CPTF_Cd	0	0	0
UIJ65452.1	TetR_family_transcriptional_regulator_[Bacillus_cereus]	CPTF_Co	31133.3	53924.45741	173.2050808
UIJ65452.1	TetR_family_transcriptional_regulator_[Bacillus_cereus]	CPTF_Cu	98524.63333	13790.77285	13.99728411
UIJ65452.1	TetR_family_transcriptional_regulator_[Bacillus_cereus]	CPTF_Fe	0	0	0
UIJ65452.1	TetR_family_transcriptional_regulator_[Bacillus_cereus]	CPTF_Mn	0	0	0
UIJ65452.1	TetR_family_transcriptional_regulator_[Bacillus_cereus]	CPTF_Ni	193511.6667	15227.32203	7.868942631
UIJ65452.1	TetR_family_transcriptional_regulator_[Bacillus_cereus]	CPTF_U	67308.83333	61962.85134	92.05753282
UIJ65452.1	TetR_family_transcriptional_regulator_[Bacillus_cereus]	CPTF_metals_mix	17039.3	15620.76093	91.67489819

UIJ65452.1	TetR_family_transcriptional_regulator_[Bacillus_cereus]	CPTF_zcontrol	0	0	0
UIJ65455.1	iron-siderophore_ABC_transporter_substrate-binding_protein_[Bacillus_cereus]	CPTF_Al	131950.6667	14331.05371	10.860918
UIJ65455.1	iron-siderophore_ABC_transporter_substrate-binding_protein_[Bacillus_cereus]	CPTF_Cd	114449.6667	99193.78574	86.67022686
UIJ65455.1	iron-siderophore_ABC_transporter_substrate-binding_protein_[Bacillus_cereus]	CPTF_Co	206356.3333	76810.48024	37.22225482
UIJ65455.1	iron-siderophore_ABC_transporter_substrate-binding_protein_[Bacillus_cereus]	CPTF_Cu	419421.3333	82755.10262	19.73078049
UIJ65455.1	iron-siderophore_ABC_transporter_substrate-binding_protein_[Bacillus_cereus]	CPTF_Fe	0	0	0
UIJ65455.1	iron-siderophore_ABC_transporter_substrate-binding_protein_[Bacillus_cereus]	CPTF_Mn	49047.66667	84953.05066	173.2050808
UIJ65455.1	iron-siderophore_ABC_transporter_substrate-binding_protein_[Bacillus_cereus]	CPTF_Ni	100113	93430.65621	93.32519873
UIJ65455.1	iron-siderophore_ABC_transporter_substrate-binding_protein_[Bacillus_cereus]	CPTF_U	137236.6667	127669.1462	93.02845171
UIJ65455.1	iron-siderophore_ABC_transporter_substrate-binding_protein_[Bacillus_cereus]	CPTF_metals_mix	294670.6667	42631.5433	14.46752192
UIJ65455.1	iron-siderophore_ABC_transporter_substrate-binding_protein_[Bacillus_cereus]	CPTF_zcontrol	81229.46667	75117.67761	92.47589661
UIJ65458.1	DinB_family_protein_[Bacillus_cereus]	CPTF_Al	202033	190130.5464	94.1086587
UIJ65458.1	DinB_family_protein_[Bacillus_cereus]	CPTF_Cd	143142	247929.2167	173.2050808
UIJ65458.1	DinB_family_protein_[Bacillus_cereus]	CPTF_Co	211501.4667	145144.372	68.6257047
UIJ65458.1	DinB_family_protein_[Bacillus_cereus]	CPTF_Cu	279447.0667	147979.6502	52.9544475
UIJ65458.1	DinB_family_protein_[Bacillus_cereus]	CPTF_Fe	132502.6667	119770.1699	90.39076189
UIJ65458.1	DinB_family_protein_[Bacillus_cereus]	CPTF_Mn	147127.0333	193077.0772	131.2315438
UIJ65458.1	DinB_family_protein_[Bacillus_cereus]	CPTF_Ni	267121.9333	185457.6675	69.42809421
UIJ65458.1	DinB_family_protein_[Bacillus_cereus]	CPTF_U	208257.8667	291569.6464	140.0041454
UIJ65458.1	DinB_family_protein_[Bacillus_cereus]	CPTF_metals_mix	105457	182656.882	173.2050808
UIJ65458.1	DinB_family_protein_[Bacillus_cereus]	CPTF_zcontrol	98484.56667	90296.19268	91.68562724
UIJ65464.1	hypothetical_protein_LW858_21360_[Bacillus_cereus]	CPTF_Al	0	0	0
UIJ65464.1	hypothetical_protein_LW858_21360_[Bacillus_cereus]	CPTF_Cd	0	0	0
UIJ65464.1	hypothetical_protein_LW858_21360_[Bacillus_cereus]	CPTF_Co	0	0	0
UIJ65464.1	hypothetical_protein_LW858_21360_[Bacillus_cereus]	CPTF_Cu	0	0	0
UIJ65464.1	hypothetical_protein_LW858_21360_[Bacillus_cereus]	CPTF_Fe	0	0	0
UIJ65464.1	hypothetical_protein_LW858_21360_[Bacillus_cereus]	CPTF_Mn	0	0	0
UIJ65464.1	hypothetical_protein_LW858_21360_[Bacillus_cereus]	CPTF_Ni	0	0	0
UIJ65464.1	hypothetical_protein_LW858_21360_[Bacillus_cereus]	CPTF_U	0	0	0
UIJ65464.1	hypothetical_protein_LW858_21360_[Bacillus_cereus]	CPTF_metals_mix	277440.6667	109781.8656	39.56949314
UIJ65464.1	hypothetical_protein_LW858_21360_[Bacillus_cereus]	CPTF_zcontrol	0	0	0
UIJ65466.1	class_I_SAM-dependent_rRNA_methyltransferase_[Bacillus_cereus]	CPTF_Al	34480.33333	59721.6892	173.2050808
UIJ65466.1	class_I_SAM-dependent_rRNA_methyltransferase_[Bacillus_cereus]	CPTF_Cd	713333.3333	1235529.576	173.2050808
UIJ65466.1	class_I_SAM-dependent_rRNA_methyltransferase_[Bacillus_cereus]	CPTF_Co	1413333.333	1227286.981	86.83634298
UIJ65466.1	class_I_SAM-dependent_rRNA_methyltransferase_[Bacillus_cereus]	CPTF_Cu	619158.9333	867460.064	140.1029715
UIJ65466.1	class_I_SAM-dependent_rRNA_methyltransferase_[Bacillus_cereus]	CPTF_Fe	1120000	1178855.377	105.2549444
UIJ65466.1	class_I_SAM-dependent_rRNA_methyltransferase_[Bacillus_cereus]	CPTF_Mn	23254.86667	40278.61059	173.2050808
UIJ65466.1	class_I_SAM-dependent_rRNA_methyltransferase_[Bacillus_cereus]	CPTF_Ni	130445.3333	120940.0725	92.7132228
UIJ65466.1	class_I_SAM-dependent_rRNA_methyltransferase_[Bacillus_cereus]	CPTF_U	0	0	0
UIJ65466.1	class_I_SAM-dependent_rRNA_methyltransferase_[Bacillus_cereus]	CPTF_metals_mix	72746.7	68527.03551	94.19951078
UIJ65466.1	class_I_SAM-dependent_rRNA_methyltransferase_[Bacillus_cereus]	CPTF_zcontrol	0	0	0
UIJ65477.1	endonuclease_MutS2_[Bacillus_cereus]	CPTF_Al	364327.0667	149181.3319	40.94709001
UIJ65477.1	endonuclease_MutS2_[Bacillus_cereus]	CPTF_Cd	450560	79778.39657	17.70649782
UIJ65477.1	endonuclease_MutS2_[Bacillus_cereus]	CPTF_Co	466655.3333	29981.18944	6.424696623
UIJ65477.1	endonuclease_MutS2_[Bacillus_cereus]	CPTF_Cu	385243.6667	219892.1806	57.07872696
UIJ65477.1	endonuclease_MutS2_[Bacillus_cereus]	CPTF_Fe	495117.6667	40580.521	8.196136743
UIJ65477.1	endonuclease_MutS2_[Bacillus_cereus]	CPTF_Mn	152604.4333	132175.8939	86.61340367
UIJ65477.1	endonuclease_MutS2_[Bacillus_cereus]	CPTF_Ni	512212.7	203581.6108	39.74552188
UIJ65477.1	endonuclease_MutS2_[Bacillus_cereus]	CPTF_U	412582.3333	102236.4188	24.77964046
UIJ65477.1	endonuclease_MutS2_[Bacillus_cereus]	CPTF_metals_mix	227368.8333	90619.0055	39.85550885
UIJ65477.1	endonuclease_MutS2_[Bacillus_cereus]	CPTF_zcontrol	332301.1	90882.71813	27.34950866
UIJ65479.1	cell_division_protein_ZapA_[Bacillus_cereus]	CPTF_Al	103630.3333	46533.322	44.90318665
UIJ65479.1	cell_division_protein_ZapA_[Bacillus_cereus]	CPTF_Cd	38154.33333	66085.24386	173.2050808
UIJ65479.1	cell_division_protein_ZapA_[Bacillus_cereus]	CPTF_Co	72817.5	21789.33701	29.9232149
UIJ65479.1	cell_division_protein_ZapA_[Bacillus_cereus]	CPTF_Cu	101699.5	40672.6794	39.99299839
UIJ65479.1	cell_division_protein_ZapA_[Bacillus_cereus]	CPTF_Fe	111450.1	72487.89372	65.04067176
UIJ65479.1	cell_division_protein_ZapA_[Bacillus_cereus]	CPTF_Mn	199008	9621.478681	4.834719549
UIJ65479.1	cell_division_protein_ZapA_[Bacillus_cereus]	CPTF_Ni	96477.1	100537.2318	104.2083892
UIJ65479.1	cell_division_protein_ZapA_[Bacillus_cereus]	CPTF_U	112380.2333	48505.25593	43.16173271
UIJ65479.1	cell_division_protein_ZapA_[Bacillus_cereus]	CPTF_metals_mix	89653	78065.48734	87.07515346
UIJ65479.1	cell_division_protein_ZapA_[Bacillus_cereus]	CPTF_zcontrol	136721	33594.28172	24.57141311

UIJ65483.1	asparagine--tRNA_ligase_[Bacillus_cereus]	CPTF_Al	20975481.67	307453.1926	1.465774172
UIJ65483.1	asparagine--tRNA_ligase_[Bacillus_cereus]	CPTF_Cd	21308053.97	1328124.418	6.232969093
UIJ65483.1	asparagine--tRNA_ligase_[Bacillus_cereus]	CPTF_Co	20601671.13	1154672.762	5.604752906
UIJ65483.1	asparagine--tRNA_ligase_[Bacillus_cereus]	CPTF_Cu	21735641.87	1084491.287	4.989460601
UIJ65483.1	asparagine--tRNA_ligase_[Bacillus_cereus]	CPTF_Fe	20663032.8	1066182.99	5.159857222
UIJ65483.1	asparagine--tRNA_ligase_[Bacillus_cereus]	CPTF_Mn	21016509.23	612946.7631	2.916501291
UIJ65483.1	asparagine--tRNA_ligase_[Bacillus_cereus]	CPTF_Ni	18787546.83	1108434.91	5.899838442
UIJ65483.1	asparagine--tRNA_ligase_[Bacillus_cereus]	CPTF_U	17615898.67	1258280.382	7.142867963
UIJ65483.1	asparagine--tRNA_ligase_[Bacillus_cereus]	CPTF_metals_mix	24957541.67	2498578.522	10.01131664
UIJ65483.1	asparagine--tRNA_ligase_[Bacillus_cereus]	CPTF_zcontrol	20565718.9	1037684.633	5.045700752
UIJ65484.1	phenylalanine--tRNA_ligase_subunit_beta_[Bacillus_cereus]	CPTF_Al	2873545.1	175275.8308	6.09963737
UIJ65484.1	phenylalanine--tRNA_ligase_subunit_beta_[Bacillus_cereus]	CPTF_Cd	2741684.8	87852.91087	3.204340297
UIJ65484.1	phenylalanine--tRNA_ligase_subunit_beta_[Bacillus_cereus]	CPTF_Co	2888565.967	57711.69928	1.997935998
UIJ65484.1	phenylalanine--tRNA_ligase_subunit_beta_[Bacillus_cereus]	CPTF_Cu	2864690.667	58302.12373	2.03519788
UIJ65484.1	phenylalanine--tRNA_ligase_subunit_beta_[Bacillus_cereus]	CPTF_Fe	2778171.5	248914.3577	8.959646935
UIJ65484.1	phenylalanine--tRNA_ligase_subunit_beta_[Bacillus_cereus]	CPTF_Mn	2674435.433	200241.5379	7.487245174
UIJ65484.1	phenylalanine--tRNA_ligase_subunit_beta_[Bacillus_cereus]	CPTF_Ni	2903449.167	187993.4571	6.474832046
UIJ65484.1	phenylalanine--tRNA_ligase_subunit_beta_[Bacillus_cereus]	CPTF_U	2508639.3	779450.0077	31.07062891
UIJ65484.1	phenylalanine--tRNA_ligase_subunit_beta_[Bacillus_cereus]	CPTF_metals_mix	3100556.367	333897.7094	10.76896111
UIJ65484.1	phenylalanine--tRNA_ligase_subunit_beta_[Bacillus_cereus]	CPTF_zcontrol	2694584.267	87021.21416	3.229485722
UIJ65485.1	phenylalanine--tRNA_ligase_subunit_alpha_[Bacillus_cereus]	CPTF_Al	285635.2667	133725.5815	46.81690151
UIJ65485.1	phenylalanine--tRNA_ligase_subunit_alpha_[Bacillus_cereus]	CPTF_Cd	240695.3333	9181.306788	3.814493061
UIJ65485.1	phenylalanine--tRNA_ligase_subunit_alpha_[Bacillus_cereus]	CPTF_Co	283806.1	56793.60329	20.01141036
UIJ65485.1	phenylalanine--tRNA_ligase_subunit_alpha_[Bacillus_cereus]	CPTF_Cu	264251.6667	67878.21061	25.68695648
UIJ65485.1	phenylalanine--tRNA_ligase_subunit_alpha_[Bacillus_cereus]	CPTF_Fe	264132.9333	62604.4235	23.70186205
UIJ65485.1	phenylalanine--tRNA_ligase_subunit_alpha_[Bacillus_cereus]	CPTF_Mn	404170.6333	129062.1569	31.93259139
UIJ65485.1	phenylalanine--tRNA_ligase_subunit_alpha_[Bacillus_cereus]	CPTF_Ni	265294.3333	11747.27893	4.428017284
UIJ65485.1	phenylalanine--tRNA_ligase_subunit_alpha_[Bacillus_cereus]	CPTF_U	278197.8333	35047.32656	12.59798689
UIJ65485.1	phenylalanine--tRNA_ligase_subunit_alpha_[Bacillus_cereus]	CPTF_metals_mix	688551.5333	58490.18969	8.49467133
UIJ65485.1	phenylalanine--tRNA_ligase_subunit_alpha_[Bacillus_cereus]	CPTF_zcontrol	192251.3333	21931.33558	11.4076377
UIJ65486.1	RNA_methyltransferase_[Bacillus_cereus]	CPTF_Al	170717.6	219190.7946	128.3937887
UIJ65486.1	RNA_methyltransferase_[Bacillus_cereus]	CPTF_Cd	0	0	0
UIJ65486.1	RNA_methyltransferase_[Bacillus_cereus]	CPTF_Co	103954	180053.6097	173.2050808
UIJ65486.1	RNA_methyltransferase_[Bacillus_cereus]	CPTF_Cu	126995.3333	219962.3697	173.2050808
UIJ65486.1	RNA_methyltransferase_[Bacillus_cereus]	CPTF_Fe	0	0	0
UIJ65486.1	RNA_methyltransferase_[Bacillus_cereus]	CPTF_Mn	123642.3333	214154.8033	173.2050808
UIJ65486.1	RNA_methyltransferase_[Bacillus_cereus]	CPTF_Ni	0	0	0
UIJ65486.1	RNA_methyltransferase_[Bacillus_cereus]	CPTF_U	0	0	0
UIJ65486.1	RNA_methyltransferase_[Bacillus_cereus]	CPTF_metals_mix	422440.8	95227.72865	22.54226596
UIJ65486.1	RNA_methyltransferase_[Bacillus_cereus]	CPTF_zcontrol	233129.3333	206397.4011	88.53343256
UIJ65492.1	M42_family_metallopeptidase_[Bacillus_cereus]	CPTF_Al	1920997.5	244319.3665	12.71835942
UIJ65492.1	M42_family_metallopeptidase_[Bacillus_cereus]	CPTF_Cd	2058575.2	211720.2616	10.28479609
UIJ65492.1	M42_family_metallopeptidase_[Bacillus_cereus]	CPTF_Co	1956787.833	343379.1711	17.54810436
UIJ65492.1	M42_family_metallopeptidase_[Bacillus_cereus]	CPTF_Cu	2460012.067	288539.1003	11.72917418
UIJ65492.1	M42_family_metallopeptidase_[Bacillus_cereus]	CPTF_Fe	1813232.833	433100.6534	23.88555102
UIJ65492.1	M42_family_metallopeptidase_[Bacillus_cereus]	CPTF_Mn	1801222.9	187759.5735	10.42400546
UIJ65492.1	M42_family_metallopeptidase_[Bacillus_cereus]	CPTF_Ni	1808028.367	338542.8325	18.72442041
UIJ65492.1	M42_family_metallopeptidase_[Bacillus_cereus]	CPTF_U	1605504.1	179131.5243	11.15733833
UIJ65492.1	M42_family_metallopeptidase_[Bacillus_cereus]	CPTF_metals_mix	3217256.667	73167.00544	2.274204797
UIJ65492.1	M42_family_metallopeptidase_[Bacillus_cereus]	CPTF_zcontrol	1552025.633	216419.5971	13.94433136
UIJ65493.1	dUTP_diphosphatase_[Bacillus_cereus]	CPTF_Al	143465.3	55697.59639	38.8230439
UIJ65493.1	dUTP_diphosphatase_[Bacillus_cereus]	CPTF_Cd	226207.3333	59171.52154	26.15809164
UIJ65493.1	dUTP_diphosphatase_[Bacillus_cereus]	CPTF_Co	153881.8667	45674.4596	29.68150867
UIJ65493.1	dUTP_diphosphatase_[Bacillus_cereus]	CPTF_Cu	229861.5	102333.4872	44.51962909
UIJ65493.1	dUTP_diphosphatase_[Bacillus_cereus]	CPTF_Fe	144388.1	37187.8096	25.75545325
UIJ65493.1	dUTP_diphosphatase_[Bacillus_cereus]	CPTF_Mn	85934.33333	77133.04409	89.75812239
UIJ65493.1	dUTP_diphosphatase_[Bacillus_cereus]	CPTF_Ni	138066.4	93566.64563	67.76930928
UIJ65493.1	dUTP_diphosphatase_[Bacillus_cereus]	CPTF_U	35678.7	35634.53242	99.87620744
UIJ65493.1	dUTP_diphosphatase_[Bacillus_cereus]	CPTF_metals_mix	322229	11083.84694	3.439742215
UIJ65493.1	dUTP_diphosphatase_[Bacillus_cereus]	CPTF_zcontrol	138609.3333	70440.73255	50.81961716
UIJ65494.1	50S_ribosomal_protein_L20_[Bacillus_cereus]	CPTF_Al	6555812.667	239461.8362	3.652664412

UIJ65494.1	50S_ribosomal_protein_L20_[Bacillus_cereus]	CPTF_Cd	6752593.667	628753.8947	9.311294677
UIJ65494.1	50S_ribosomal_protein_L20_[Bacillus_cereus]	CPTF_Co	6684522.667	198663.6204	2.971994117
UIJ65494.1	50S_ribosomal_protein_L20_[Bacillus_cereus]	CPTF_Cu	6522724.333	219758.7139	3.369124658
UIJ65494.1	50S_ribosomal_protein_L20_[Bacillus_cereus]	CPTF_Fe	6658417	428708.6901	6.438597795
UIJ65494.1	50S_ribosomal_protein_L20_[Bacillus_cereus]	CPTF_Mn	6298133.667	891681.4406	14.15786783
UIJ65494.1	50S_ribosomal_protein_L20_[Bacillus_cereus]	CPTF_Ni	5674345.533	325402.8534	5.734632328
UIJ65494.1	50S_ribosomal_protein_L20_[Bacillus_cereus]	CPTF_U	5511305	862070.2229	15.64185293
UIJ65494.1	50S_ribosomal_protein_L20_[Bacillus_cereus]	CPTF_metals_mix	7009044.233	881600.6714	12.57804405
UIJ65494.1	50S_ribosomal_protein_L20_[Bacillus_cereus]	CPTF_zcontrol	6075775	493772.4001	8.126903977
UIJ65495.1	50S_ribosomal_protein_L35_[Bacillus_cereus]	CPTF_Al	7973816	440709.566	5.526959313
UIJ65495.1	50S_ribosomal_protein_L35_[Bacillus_cereus]	CPTF_Cd	9297187.133	433834.8186	4.666301887
UIJ65495.1	50S_ribosomal_protein_L35_[Bacillus_cereus]	CPTF_Co	8316119	172666.5044	2.076287081
UIJ65495.1	50S_ribosomal_protein_L35_[Bacillus_cereus]	CPTF_Cu	7514655.333	203841.1291	2.712581217
UIJ65495.1	50S_ribosomal_protein_L35_[Bacillus_cereus]	CPTF_Fe	8618125	507442.2613	5.888081936
UIJ65495.1	50S_ribosomal_protein_L35_[Bacillus_cereus]	CPTF_Mn	8614201.133	566058.9424	6.57122969
UIJ65495.1	50S_ribosomal_protein_L35_[Bacillus_cereus]	CPTF_Ni	6848311.333	227216.9615	3.317853854
UIJ65495.1	50S_ribosomal_protein_L35_[Bacillus_cereus]	CPTF_U	7009884.167	857327.4771	12.23026596
UIJ65495.1	50S_ribosomal_protein_L35_[Bacillus_cereus]	CPTF_metals_mix	6290597	511372.3436	8.129154412
UIJ65495.1	50S_ribosomal_protein_L35_[Bacillus_cereus]	CPTF_zcontrol	8943804.433	846065.4874	9.459794137
UIJ65496.1	translation_initiation_factor_IF-3_[Bacillus_cereus]	CPTF_Al	673313.6667	70182.66812	10.42347298
UIJ65496.1	translation_initiation_factor_IF-3_[Bacillus_cereus]	CPTF_Cd	760216.3333	40613.8748	5.342410182
UIJ65496.1	translation_initiation_factor_IF-3_[Bacillus_cereus]	CPTF_Co	724682.6667	36500.8639	5.036806533
UIJ65496.1	translation_initiation_factor_IF-3_[Bacillus_cereus]	CPTF_Cu	665272	82870.90274	12.45669482
UIJ65496.1	translation_initiation_factor_IF-3_[Bacillus_cereus]	CPTF_Fe	769576.6667	54542.40837	7.087326153
UIJ65496.1	translation_initiation_factor_IF-3_[Bacillus_cereus]	CPTF_Mn	723906.6667	102991.8563	14.2272286
UIJ65496.1	translation_initiation_factor_IF-3_[Bacillus_cereus]	CPTF_Ni	572440	14430.71381	2.520912901
UIJ65496.1	translation_initiation_factor_IF-3_[Bacillus_cereus]	CPTF_U	642674.3333	65327.11257	10.16488588
UIJ65496.1	translation_initiation_factor_IF-3_[Bacillus_cereus]	CPTF_metals_mix	629856.7333	103134.0986	16.37421547
UIJ65496.1	translation_initiation_factor_IF-3_[Bacillus_cereus]	CPTF_zcontrol	694212.6667	2946.28789	0.424407106
UIJ65497.1	threonine--tRNA_ligase_[Bacillus_cereus]	CPTF_Al	7495966.8	169904.3883	2.266610737
UIJ65497.1	threonine--tRNA_ligase_[Bacillus_cereus]	CPTF_Cd	7052529.567	414149.4756	5.872353624
UIJ65497.1	threonine--tRNA_ligase_[Bacillus_cereus]	CPTF_Co	7292993.4	552907.0142	7.581345326
UIJ65497.1	threonine--tRNA_ligase_[Bacillus_cereus]	CPTF_Cu	6968080.867	778174.6536	11.16770411
UIJ65497.1	threonine--tRNA_ligase_[Bacillus_cereus]	CPTF_Fe	6726103.967	476151.8466	7.079162751
UIJ65497.1	threonine--tRNA_ligase_[Bacillus_cereus]	CPTF_Mn	7158006.7	1473789.684	20.58938676
UIJ65497.1	threonine--tRNA_ligase_[Bacillus_cereus]	CPTF_Ni	6993622.333	750101.9707	10.7255144
UIJ65497.1	threonine--tRNA_ligase_[Bacillus_cereus]	CPTF_U	6413831.867	808065.2367	12.59879045
UIJ65497.1	threonine--tRNA_ligase_[Bacillus_cereus]	CPTF_metals_mix	7079360.603	964631.0926	13.62596351
UIJ65497.1	threonine--tRNA_ligase_[Bacillus_cereus]	CPTF_zcontrol	6733233.62	359689.8396	5.34200742
UIJ65502.1	transcriptional_regulator_NrdR_[Bacillus_cereus]	CPTF_Al	692701.6667	137991.8319	19.92081707
UIJ65502.1	transcriptional_regulator_NrdR_[Bacillus_cereus]	CPTF_Cd	708346.8	206659.2507	29.17486896
UIJ65502.1	transcriptional_regulator_NrdR_[Bacillus_cereus]	CPTF_Co	755061.6667	51381.60054	6.804954193
UIJ65502.1	transcriptional_regulator_NrdR_[Bacillus_cereus]	CPTF_Cu	799291.3333	161112.8442	20.15696123
UIJ65502.1	transcriptional_regulator_NrdR_[Bacillus_cereus]	CPTF_Fe	531771.6667	184384.4482	34.6736127
UIJ65502.1	transcriptional_regulator_NrdR_[Bacillus_cereus]	CPTF_Mn	651114.7333	59425.00674	9.126656747
UIJ65502.1	transcriptional_regulator_NrdR_[Bacillus_cereus]	CPTF_Ni	652517.3333	71772.70401	10.99935593
UIJ65502.1	transcriptional_regulator_NrdR_[Bacillus_cereus]	CPTF_U	551506.8667	69468.13554	12.59605995
UIJ65502.1	transcriptional_regulator_NrdR_[Bacillus_cereus]	CPTF_metals_mix	957492.5667	15978.2877	1.668763629
UIJ65502.1	transcriptional_regulator_NrdR_[Bacillus_cereus]	CPTF_zcontrol	636967.3333	176240.3329	27.66866113
UIJ65503.1	glyceraldehyde-3-phosphate_dehydrogenase_[Bacillus_cereus]	CPTF_Al	548689.8333	205986.9362	37.54159886
UIJ65503.1	glyceraldehyde-3-phosphate_dehydrogenase_[Bacillus_cereus]	CPTF_Cd	985769.3667	147052.7553	14.91756188
UIJ65503.1	glyceraldehyde-3-phosphate_dehydrogenase_[Bacillus_cereus]	CPTF_Co	477617.2667	116529.3669	24.3980641
UIJ65503.1	glyceraldehyde-3-phosphate_dehydrogenase_[Bacillus_cereus]	CPTF_Cu	666296.7333	49550.48709	7.436699688
UIJ65503.1	glyceraldehyde-3-phosphate_dehydrogenase_[Bacillus_cereus]	CPTF_Fe	799174.9	120577.7034	15.08777407
UIJ65503.1	glyceraldehyde-3-phosphate_dehydrogenase_[Bacillus_cereus]	CPTF_Mn	630855.2333	349957.1579	55.47344927
UIJ65503.1	glyceraldehyde-3-phosphate_dehydrogenase_[Bacillus_cereus]	CPTF_Ni	507992.5667	29221.4925	5.752346474
UIJ65503.1	glyceraldehyde-3-phosphate_dehydrogenase_[Bacillus_cereus]	CPTF_U	515834.0333	102883.4827	19.94507458
UIJ65503.1	glyceraldehyde-3-phosphate_dehydrogenase_[Bacillus_cereus]	CPTF_metals_mix	2379239.733	123111.2207	5.174393272
UIJ65503.1	glyceraldehyde-3-phosphate_dehydrogenase_[Bacillus_cereus]	CPTF_zcontrol	397959.5	142742.847	35.86868688
UIJ65504.1	dephospho-CoA_kinase_[Bacillus_cereus]	CPTF_Al	266406.6	236217.1448	88.66790267
UIJ65504.1	dephospho-CoA_kinase_[Bacillus_cereus]	CPTF_Cd	201032.3333	165365.6497	82.25823525

UIJ65504.1	dephospho-CoA_kinase_[Bacillus_cereus]	CPTF_Co	234204.0333	162025.3543	69.18128265
UIJ65504.1	dephospho-CoA_kinase_[Bacillus_cereus]	CPTF_Cu	353333.8333	128626.5129	36.40367854
UIJ65504.1	dephospho-CoA_kinase_[Bacillus_cereus]	CPTF_Fe	276816.7	252727.8656	91.29791145
UIJ65504.1	dephospho-CoA_kinase_[Bacillus_cereus]	CPTF_Mn	163701.6667	283539.604	173.2050808
UIJ65504.1	dephospho-CoA_kinase_[Bacillus_cereus]	CPTF_Ni	396158.1667	148552.1411	37.49818975
UIJ65504.1	dephospho-CoA_kinase_[Bacillus_cereus]	CPTF_U	145095.6	170700.6677	117.6470325
UIJ65504.1	dephospho-CoA_kinase_[Bacillus_cereus]	CPTF_metals_mix	367803.1333	43993.03862	11.96102878
UIJ65504.1	dephospho-CoA_kinase_[Bacillus_cereus]	CPTF_zcontrol	14910.1	25825.05075	173.2050808
UIJ65506.1	DNA-formamidopyrimidine_glycosylase_[Bacillus_cereus]	CPTF_Al	1086666.667	1882161.878	173.2050808
UIJ65506.1	DNA-formamidopyrimidine_glycosylase_[Bacillus_cereus]	CPTF_Cd	0	0	0
UIJ65506.1	DNA-formamidopyrimidine_glycosylase_[Bacillus_cereus]	CPTF_Co	3296666.667	225018.5178	6.825637546
UIJ65506.1	DNA-formamidopyrimidine_glycosylase_[Bacillus_cereus]	CPTF_Cu	1940000	1712191.578	88.25729784
UIJ65506.1	DNA-formamidopyrimidine_glycosylase_[Bacillus_cereus]	CPTF_Fe	736666.6667	1275944.095	173.2050808
UIJ65506.1	DNA-formamidopyrimidine_glycosylase_[Bacillus_cereus]	CPTF_Mn	2710000	2423654.266	89.43373674
UIJ65506.1	DNA-formamidopyrimidine_glycosylase_[Bacillus_cereus]	CPTF_Ni	1633333.333	1459600.402	89.36328991
UIJ65506.1	DNA-formamidopyrimidine_glycosylase_[Bacillus_cereus]	CPTF_U	0	0	0
UIJ65506.1	DNA-formamidopyrimidine_glycosylase_[Bacillus_cereus]	CPTF_metals_mix	1843333.333	3192746.989	173.2050808
UIJ65506.1	DNA-formamidopyrimidine_glycosylase_[Bacillus_cereus]	CPTF_zcontrol	1500000	2598076.211	173.2050808
UIJ65507.1	DNA_polymerase_I_[Bacillus_cereus]	CPTF_Al	1525882.9	530783.3781	34.78532842
UIJ65507.1	DNA_polymerase_I_[Bacillus_cereus]	CPTF_Cd	2015456.267	876269.5566	43.47747808
UIJ65507.1	DNA_polymerase_I_[Bacillus_cereus]	CPTF_Co	1567278.867	668274.6133	42.63916445
UIJ65507.1	DNA_polymerase_I_[Bacillus_cereus]	CPTF_Cu	1826006.433	198023.0418	10.84459716
UIJ65507.1	DNA_polymerase_I_[Bacillus_cereus]	CPTF_Fe	1207430	534772.5566	44.29014987
UIJ65507.1	DNA_polymerase_I_[Bacillus_cereus]	CPTF_Mn	1577527.967	194709.2651	12.3426823
UIJ65507.1	DNA_polymerase_I_[Bacillus_cereus]	CPTF_Ni	1516523.467	582423.2063	38.40515621
UIJ65507.1	DNA_polymerase_I_[Bacillus_cereus]	CPTF_U	1587319.867	115585.9053	7.281828178
UIJ65507.1	DNA_polymerase_I_[Bacillus_cereus]	CPTF_metals_mix	3331341.667	401921.7169	12.06486026
UIJ65507.1	DNA_polymerase_I_[Bacillus_cereus]	CPTF_zcontrol	1604593.4	893034.9092	55.65490356
UIJ65511.1	MaoC_family_dehydratase_N-terminal_domain-containing_protein_[Bacillus_cereus]	CPTF_Al	199092.6333	95506.55287	47.97091247
UIJ65511.1	MaoC_family_dehydratase_N-terminal_domain-containing_protein_[Bacillus_cereus]	CPTF_Cd	114400.9	103651.1856	90.60347046
UIJ65511.1	MaoC_family_dehydratase_N-terminal_domain-containing_protein_[Bacillus_cereus]	CPTF_Co	159844.6	143247.6888	89.61684586
UIJ65511.1	MaoC_family_dehydratase_N-terminal_domain-containing_protein_[Bacillus_cereus]	CPTF_Cu	177748.8333	52156.54592	29.34283446
UIJ65511.1	MaoC_family_dehydratase_N-terminal_domain-containing_protein_[Bacillus_cereus]	CPTF_Fe	189112.6	166069.0733	87.81491731
UIJ65511.1	MaoC_family_dehydratase_N-terminal_domain-containing_protein_[Bacillus_cereus]	CPTF_Mn	37743.56667	33902.79224	89.82402894
UIJ65511.1	MaoC_family_dehydratase_N-terminal_domain-containing_protein_[Bacillus_cereus]	CPTF_Ni	173708.3333	162265.8118	93.4127964
UIJ65511.1	MaoC_family_dehydratase_N-terminal_domain-containing_protein_[Bacillus_cereus]	CPTF_U	179246	163901.2092	91.43925623
UIJ65511.1	MaoC_family_dehydratase_N-terminal_domain-containing_protein_[Bacillus_cereus]	CPTF_metals_mix	86350.63333	44106.41646	51.07827789
UIJ65511.1	MaoC_family_dehydratase_N-terminal_domain-containing_protein_[Bacillus_cereus]	CPTF_zcontrol	284379.1333	62282.49045	21.90121677
UIJ65512.1	malate_dehydrogenase_[Bacillus_cereus]	CPTF_Al	1685545.867	297205.5358	17.63259854
UIJ65512.1	malate_dehydrogenase_[Bacillus_cereus]	CPTF_Cd	2322062.533	376519.9117	16.21489113
UIJ65512.1	malate_dehydrogenase_[Bacillus_cereus]	CPTF_Co	1409652.8	291019.9156	20.64479392
UIJ65512.1	malate_dehydrogenase_[Bacillus_cereus]	CPTF_Cu	1384662.033	97821.89285	7.06467647
UIJ65512.1	malate_dehydrogenase_[Bacillus_cereus]	CPTF_Fe	1838636.667	331021.1591	18.00362002
UIJ65512.1	malate_dehydrogenase_[Bacillus_cereus]	CPTF_Mn	1867302.467	834674.5038	44.6994806
UIJ65512.1	malate_dehydrogenase_[Bacillus_cereus]	CPTF_Ni	1291564.267	119836.5835	9.278406551
UIJ65512.1	malate_dehydrogenase_[Bacillus_cereus]	CPTF_U	1021334.167	363764.4246	35.61659214
UIJ65512.1	malate_dehydrogenase_[Bacillus_cereus]	CPTF_metals_mix	3084902.4	183893.8666	5.961091883
UIJ65512.1	malate_dehydrogenase_[Bacillus_cereus]	CPTF_zcontrol	1406685.367	136684.9975	9.716813774
UIJ65513.1	NADP-dependent_isocitrate_dehydrogenase_[Bacillus_cereus]	CPTF_Al	24669873.7	1409209.159	5.712267423
UIJ65513.1	NADP-dependent_isocitrate_dehydrogenase_[Bacillus_cereus]	CPTF_Cd	26194398.03	696427.4417	2.658688475
UIJ65513.1	NADP-dependent_isocitrate_dehydrogenase_[Bacillus_cereus]	CPTF_Co	23976865.7	1740776.282	7.260232857
UIJ65513.1	NADP-dependent_isocitrate_dehydrogenase_[Bacillus_cereus]	CPTF_Cu	23338453.5	256651.1141	1.099692034
UIJ65513.1	NADP-dependent_isocitrate_dehydrogenase_[Bacillus_cereus]	CPTF_Fe	24168477.93	214967.5259	0.889454133
UIJ65513.1	NADP-dependent_isocitrate_dehydrogenase_[Bacillus_cereus]	CPTF_Mn	24942123.63	1096951.106	4.397986002
UIJ65513.1	NADP-dependent_isocitrate_dehydrogenase_[Bacillus_cereus]	CPTF_Ni	21346918	1841796.817	8.627928475
UIJ65513.1	NADP-dependent_isocitrate_dehydrogenase_[Bacillus_cereus]	CPTF_U	21332034.97	4689910.415	21.98529312
UIJ65513.1	NADP-dependent_isocitrate_dehydrogenase_[Bacillus_cereus]	CPTF_metals_mix	25332583.07	2259657.999	8.9199668
UIJ65513.1	NADP-dependent_isocitrate_dehydrogenase_[Bacillus_cereus]	CPTF_zcontrol	25465996	945823.8104	3.714065652
UIJ65514.1	citrate_synthase_[Bacillus_cereus]	CPTF_Al	7198699	221246.1585	3.073418662
UIJ65514.1	citrate_synthase_[Bacillus_cereus]	CPTF_Cd	7228439.133	849133.844	11.74712588
UIJ65514.1	citrate_synthase_[Bacillus_cereus]	CPTF_Co	6636397	372604.042	5.614553228

UIJ65514.1	citrate_synthase_[Bacillus_cereus]	CPTF_Cu	7188977	433577.3539	6.031141202
UIJ65514.1	citrate_synthase_[Bacillus_cereus]	CPTF_Fe	7119240.667	346581.824	4.868241436
UIJ65514.1	citrate_synthase_[Bacillus_cereus]	CPTF_Mn	7171387.633	207206.7404	2.889353511
UIJ65514.1	citrate_synthase_[Bacillus_cereus]	CPTF_Ni	6968180.667	1002148.755	14.3817849
UIJ65514.1	citrate_synthase_[Bacillus_cereus]	CPTF_U	5769574.6	700239.4256	12.13676006
UIJ65514.1	citrate_synthase_[Bacillus_cereus]	CPTF_metals_mix	7876439.267	944748.4935	11.99461408
UIJ65514.1	citrate_synthase_[Bacillus_cereus]	CPTF_zcontrol	7097112.267	484580.3541	6.827852455
UIJ65518.1	pyruvate_kinase_[Bacillus_cereus]	CPTF_Al	21750888.7	2522314.826	11.59637595
UIJ65518.1	pyruvate_kinase_[Bacillus_cereus]	CPTF_Cd	23672202.33	988214.6868	4.174578575
UIJ65518.1	pyruvate_kinase_[Bacillus_cereus]	CPTF_Co	22554029.67	1697227.038	7.525160973
UIJ65518.1	pyruvate_kinase_[Bacillus_cereus]	CPTF_Cu	21506696.07	310039.275	1.441594162
UIJ65518.1	pyruvate_kinase_[Bacillus_cereus]	CPTF_Fe	22100979.13	1354716.215	6.129666053
UIJ65518.1	pyruvate_kinase_[Bacillus_cereus]	CPTF_Mn	22471174.8	4631663.995	20.61157922
UIJ65518.1	pyruvate_kinase_[Bacillus_cereus]	CPTF_Ni	16892253.63	1500330.802	8.881768143
UIJ65518.1	pyruvate_kinase_[Bacillus_cereus]	CPTF_U	18230719.47	860529.1697	4.720215081
UIJ65518.1	pyruvate_kinase_[Bacillus_cereus]	CPTF_metals_mix	40624510.4	2838568.654	6.987330126
UIJ65518.1	pyruvate_kinase_[Bacillus_cereus]	CPTF_zcontrol	19923163.2	1140209.06	5.723032272
UIJ65519.1	6-phosphofructokinase_[Bacillus_cereus]	CPTF_Al	4582467.3	336426.2917	7.341597215
UIJ65519.1	6-phosphofructokinase_[Bacillus_cereus]	CPTF_Cd	5003871.467	426511.5695	8.523631599
UIJ65519.1	6-phosphofructokinase_[Bacillus_cereus]	CPTF_Co	4523176.667	419399.4019	9.272231283
UIJ65519.1	6-phosphofructokinase_[Bacillus_cereus]	CPTF_Cu	4599243.3	268502.1182	5.837962914
UIJ65519.1	6-phosphofructokinase_[Bacillus_cereus]	CPTF_Fe	4390605.633	251800.7283	5.734988503
UIJ65519.1	6-phosphofructokinase_[Bacillus_cereus]	CPTF_Mn	4800220.633	1180531.124	24.59326799
UIJ65519.1	6-phosphofructokinase_[Bacillus_cereus]	CPTF_Ni	4563591.4	467345.3318	10.24073566
UIJ65519.1	6-phosphofructokinase_[Bacillus_cereus]	CPTF_U	4073090.333	265662.5864	6.522383857
UIJ65519.1	6-phosphofructokinase_[Bacillus_cereus]	CPTF_metals_mix	10467948.93	1547145.484	14.77983408
UIJ65519.1	6-phosphofructokinase_[Bacillus_cereus]	CPTF_zcontrol	3785022.667	171856.2448	4.540428419
UIJ65520.1	acetyl-CoA_carboxylase_carboxyl_transferase_subunit_alpha_[Bacillus_cereus]	CPTF_Al	890887.3333	304093.6801	34.1337977
UIJ65520.1	acetyl-CoA_carboxylase_carboxyl_transferase_subunit_alpha_[Bacillus_cereus]	CPTF_Cd	489706.1667	54490.12375	11.1271059
UIJ65520.1	acetyl-CoA_carboxylase_carboxyl_transferase_subunit_alpha_[Bacillus_cereus]	CPTF_Co	910217.4333	219763.6216	24.14407959
UIJ65520.1	acetyl-CoA_carboxylase_carboxyl_transferase_subunit_alpha_[Bacillus_cereus]	CPTF_Cu	801044.2	297622.2293	37.15428304
UIJ65520.1	acetyl-CoA_carboxylase_carboxyl_transferase_subunit_alpha_[Bacillus_cereus]	CPTF_Fe	805150.0667	95476.39536	11.8582112
UIJ65520.1	acetyl-CoA_carboxylase_carboxyl_transferase_subunit_alpha_[Bacillus_cereus]	CPTF_Mn	610497.6333	110445.3704	18.09103989
UIJ65520.1	acetyl-CoA_carboxylase_carboxyl_transferase_subunit_alpha_[Bacillus_cereus]	CPTF_Ni	731573.6667	248018.5425	33.90205987
UIJ65520.1	acetyl-CoA_carboxylase_carboxyl_transferase_subunit_alpha_[Bacillus_cereus]	CPTF_U	606871.4333	100698.2983	16.59302
UIJ65520.1	acetyl-CoA_carboxylase_carboxyl_transferase_subunit_alpha_[Bacillus_cereus]	CPTF_metals_mix	581422.8667	232475.7926	39.98394386
UIJ65520.1	acetyl-CoA_carboxylase_carboxyl_transferase_subunit_alpha_[Bacillus_cereus]	CPTF_zcontrol	615425.3333	189206.0927	30.74395583
UIJ65521.1	acetyl-CoA_carboxylase,_carboxyltransferase_subunit_beta_[Bacillus_cereus]	CPTF_Al	68010	24319.33337	35.75846694
UIJ65521.1	acetyl-CoA_carboxylase,_carboxyltransferase_subunit_beta_[Bacillus_cereus]	CPTF_Cd	42348.26667	28298.51738	66.82331914
UIJ65521.1	acetyl-CoA_carboxylase,_carboxyltransferase_subunit_beta_[Bacillus_cereus]	CPTF_Co	404064.9667	624390.6416	154.5272897
UIJ65521.1	acetyl-CoA_carboxylase,_carboxyltransferase_subunit_beta_[Bacillus_cereus]	CPTF_Cu	961508.4	111517.4543	11.59817785
UIJ65521.1	acetyl-CoA_carboxylase,_carboxyltransferase_subunit_beta_[Bacillus_cereus]	CPTF_Fe	59145.3	32046.02733	54.18186623
UIJ65521.1	acetyl-CoA_carboxylase,_carboxyltransferase_subunit_beta_[Bacillus_cereus]	CPTF_Mn	79619.46667	85759.54791	107.7117839
UIJ65521.1	acetyl-CoA_carboxylase,_carboxyltransferase_subunit_beta_[Bacillus_cereus]	CPTF_Ni	597630.7	389254.1404	65.132889
UIJ65521.1	acetyl-CoA_carboxylase,_carboxyltransferase_subunit_beta_[Bacillus_cereus]	CPTF_U	20584.6	35653.57305	173.2050808
UIJ65521.1	acetyl-CoA_carboxylase,_carboxyltransferase_subunit_beta_[Bacillus_cereus]	CPTF_metals_mix	1127488.3	610783.206	54.17202165
UIJ65521.1	acetyl-CoA_carboxylase,_carboxyltransferase_subunit_beta_[Bacillus_cereus]	CPTF_zcontrol	14376.93333	24901.57899	173.2050808
UIJ65522.1	GntR_family_transcriptional_regulator_[Bacillus_cereus]	CPTF_Al	96567.66667	167260.105	173.2050808
UIJ65522.1	GntR_family_transcriptional_regulator_[Bacillus_cereus]	CPTF_Cd	0	0	0
UIJ65522.1	GntR_family_transcriptional_regulator_[Bacillus_cereus]	CPTF_Co	95982.66667	166246.8553	173.2050808
UIJ65522.1	GntR_family_transcriptional_regulator_[Bacillus_cereus]	CPTF_Cu	0	0	0
UIJ65522.1	GntR_family_transcriptional_regulator_[Bacillus_cereus]	CPTF_Fe	0	0	0
UIJ65522.1	GntR_family_transcriptional_regulator_[Bacillus_cereus]	CPTF_Mn	0	0	0
UIJ65522.1	GntR_family_transcriptional_regulator_[Bacillus_cereus]	CPTF_Ni	92116.33333	159550.1695	173.2050808
UIJ65522.1	GntR_family_transcriptional_regulator_[Bacillus_cereus]	CPTF_U	0	0	0
UIJ65522.1	GntR_family_transcriptional_regulator_[Bacillus_cereus]	CPTF_metals_mix	11731.66667	20319.84272	173.2050808
UIJ65522.1	GntR_family_transcriptional_regulator_[Bacillus_cereus]	CPTF_zcontrol	0	0	0
UIJ65526.1	bifunctional_oligoribonuclease/PAP_phosphatase_NrnA_[Bacillus_cereus]	CPTF_Al	257439.9	55731.40477	21.64831666
UIJ65526.1	bifunctional_oligoribonuclease/PAP_phosphatase_NrnA_[Bacillus_cereus]	CPTF_Cd	381348.4667	166265.8478	43.59945361
UIJ65526.1	bifunctional_oligoribonuclease/PAP_phosphatase_NrnA_[Bacillus_cereus]	CPTF_Co	402189	111124.4991	27.62992005
UIJ65526.1	bifunctional_oligoribonuclease/PAP_phosphatase_NrnA_[Bacillus_cereus]	CPTF_Cu	410471.8	187857.739	45.76629601

UIJ65526.1	bifunctional_oligoribonuclease/PAP_phosphatase_NrnA_[Bacillus_cereus]	CPTF_Fe	293627.5	128146.6157	43.64257969
UIJ65526.1	bifunctional_oligoribonuclease/PAP_phosphatase_NrnA_[Bacillus_cereus]	CPTF_Mn	215646	101710.2308	47.16536864
UIJ65526.1	bifunctional_oligoribonuclease/PAP_phosphatase_NrnA_[Bacillus_cereus]	CPTF_Ni	228788.4667	235167.6879	102.7882617
UIJ65526.1	bifunctional_oligoribonuclease/PAP_phosphatase_NrnA_[Bacillus_cereus]	CPTF_U	269377	47614.13168	17.67564851
UIJ65526.1	bifunctional_oligoribonuclease/PAP_phosphatase_NrnA_[Bacillus_cereus]	CPTF_metals_mix	489533.9	130520.7791	26.66225549
UIJ65526.1	bifunctional_oligoribonuclease/PAP_phosphatase_NrnA_[Bacillus_cereus]	CPTF_zcontrol	195227	7408.957889	3.795047759
UIJ65529.1	CBS_domain-containing_protein_[Bacillus_cereus]	CPTF_Al	94046.3	31542.72783	33.53957342
UIJ65529.1	CBS_domain-containing_protein_[Bacillus_cereus]	CPTF_Cd	101051.1	41953.38129	41.51699615
UIJ65529.1	CBS_domain-containing_protein_[Bacillus_cereus]	CPTF_Co	105037.5333	11982.34542	11.40767975
UIJ65529.1	CBS_domain-containing_protein_[Bacillus_cereus]	CPTF_Cu	224062.9	164188.1525	73.27770573
UIJ65529.1	CBS_domain-containing_protein_[Bacillus_cereus]	CPTF_Fe	107291	17455.23139	16.26905462
UIJ65529.1	CBS_domain-containing_protein_[Bacillus_cereus]	CPTF_Mn	178915	206977.2793	115.684699
UIJ65529.1	CBS_domain-containing_protein_[Bacillus_cereus]	CPTF_Ni	156029.3333	145621.1136	93.32931861
UIJ65529.1	CBS_domain-containing_protein_[Bacillus_cereus]	CPTF_U	147940.8333	167047.3936	112.9150011
UIJ65529.1	CBS_domain-containing_protein_[Bacillus_cereus]	CPTF_metals_mix	37130.66667	64312.20119	173.2050808
UIJ65529.1	CBS_domain-containing_protein_[Bacillus_cereus]	CPTF_zcontrol	274649.1	162077.6982	59.01264493
UIJ65531.1	metal-dependent_hydrolase_[Bacillus_cereus]	CPTF_Al	31499.93333	27960.56388	88.76388273
UIJ65531.1	metal-dependent_hydrolase_[Bacillus_cereus]	CPTF_Cd	41749.33333	18569.58686	44.47876261
UIJ65531.1	metal-dependent_hydrolase_[Bacillus_cereus]	CPTF_Co	6709.233333	11620.73301	173.2050808
UIJ65531.1	metal-dependent_hydrolase_[Bacillus_cereus]	CPTF_Cu	21503.06667	21565.11551	100.2885581
UIJ65531.1	metal-dependent_hydrolase_[Bacillus_cereus]	CPTF_Fe	21827.76667	16794.75521	76.94216026
UIJ65531.1	metal-dependent_hydrolase_[Bacillus_cereus]	CPTF_Mn	30600.56667	35916.26159	117.3712304
UIJ65531.1	metal-dependent_hydrolase_[Bacillus_cereus]	CPTF_Ni	0	0	0
UIJ65531.1	metal-dependent_hydrolase_[Bacillus_cereus]	CPTF_U	0	0	0
UIJ65531.1	metal-dependent_hydrolase_[Bacillus_cereus]	CPTF_metals_mix	47364.23333	23086.65476	48.7428026
UIJ65531.1	metal-dependent_hydrolase_[Bacillus_cereus]	CPTF_zcontrol	11714.4	20289.93598	173.2050808
UIJ65532.1	Xaa-Pro_dipeptidase_[Bacillus_cereus]	CPTF_Al	3633556.867	400938.2076	11.03431768
UIJ65532.1	Xaa-Pro_dipeptidase_[Bacillus_cereus]	CPTF_Cd	3946635.433	136007.8424	3.446171927
UIJ65532.1	Xaa-Pro_dipeptidase_[Bacillus_cereus]	CPTF_Co	3288928.567	556335.4103	16.91539962
UIJ65532.1	Xaa-Pro_dipeptidase_[Bacillus_cereus]	CPTF_Cu	3662686.133	145519.0003	3.97301311
UIJ65532.1	Xaa-Pro_dipeptidase_[Bacillus_cereus]	CPTF_Fe	3368772.267	65675.39928	1.949535145
UIJ65532.1	Xaa-Pro_dipeptidase_[Bacillus_cereus]	CPTF_Mn	3716473.867	1191925.818	32.07141664
UIJ65532.1	Xaa-Pro_dipeptidase_[Bacillus_cereus]	CPTF_Ni	2530331.733	155280.3537	6.136758738
UIJ65532.1	Xaa-Pro_dipeptidase_[Bacillus_cereus]	CPTF_U	3337039.267	543728.0451	16.29372631
UIJ65532.1	Xaa-Pro_dipeptidase_[Bacillus_cereus]	CPTF_metals_mix	5962836.833	448507.0342	7.521705636
UIJ65532.1	Xaa-Pro_dipeptidase_[Bacillus_cereus]	CPTF_zcontrol	3182392.633	524091.18	16.46846384
UIJ65540.1	alanine_dehydrogenase_[Bacillus_cereus]	CPTF_Al	1810224.8	136299.2196	7.529408479
UIJ65540.1	alanine_dehydrogenase_[Bacillus_cereus]	CPTF_Cd	1879126.933	148631.8728	7.909623889
UIJ65540.1	alanine_dehydrogenase_[Bacillus_cereus]	CPTF_Co	1649910.467	130692.4592	7.921184927
UIJ65540.1	alanine_dehydrogenase_[Bacillus_cereus]	CPTF_Cu	1678860.633	68068.29966	4.054434198
UIJ65540.1	alanine_dehydrogenase_[Bacillus_cereus]	CPTF_Fe	1607551.7	166168.1031	10.33671907
UIJ65540.1	alanine_dehydrogenase_[Bacillus_cereus]	CPTF_Mn	1938612.633	462489.6022	23.85673106
UIJ65540.1	alanine_dehydrogenase_[Bacillus_cereus]	CPTF_Ni	1736625.067	361725.3603	20.8292145
UIJ65540.1	alanine_dehydrogenase_[Bacillus_cereus]	CPTF_U	2070903	1069769.259	51.65713988
UIJ65540.1	alanine_dehydrogenase_[Bacillus_cereus]	CPTF_metals_mix	5949043.133	925986.6222	15.56530355
UIJ65540.1	alanine_dehydrogenase_[Bacillus_cereus]	CPTF_zcontrol	1500337.333	226066.1128	15.06768563
UIJ65541.1	universal_stress_protein_[Bacillus_cereus]	CPTF_Al	10818435.6	3513871.667	32.48040472
UIJ65541.1	universal_stress_protein_[Bacillus_cereus]	CPTF_Cd	19134570.67	2975747.469	15.55168141
UIJ65541.1	universal_stress_protein_[Bacillus_cereus]	CPTF_Co	9169707.867	3895534.474	42.48264537
UIJ65541.1	universal_stress_protein_[Bacillus_cereus]	CPTF_Cu	8754616.333	1534511.653	17.52802858
UIJ65541.1	universal_stress_protein_[Bacillus_cereus]	CPTF_Fe	11990262.07	4682567.701	39.05308887
UIJ65541.1	universal_stress_protein_[Bacillus_cereus]	CPTF_Mn	14459024.8	11964224.22	82.74572029
UIJ65541.1	universal_stress_protein_[Bacillus_cereus]	CPTF_Ni	3465831.433	1199887.208	34.62047222
UIJ65541.1	universal_stress_protein_[Bacillus_cereus]	CPTF_U	3471222.503	1276811.418	36.78275931
UIJ65541.1	universal_stress_protein_[Bacillus_cereus]	CPTF_metals_mix	44198973.63	2700573.134	6.110035849
UIJ65541.1	universal_stress_protein_[Bacillus_cereus]	CPTF_zcontrol	7619857.333	2483806.02	32.59648981
UIJ65545.1	argininosuccinate_lyase_[Bacillus_cereus]	CPTF_Al	4314462.533	597772.2572	13.8550805
UIJ65545.1	argininosuccinate_lyase_[Bacillus_cereus]	CPTF_Cd	3723043.8	225085.5141	6.045739082
UIJ65545.1	argininosuccinate_lyase_[Bacillus_cereus]	CPTF_Co	3706926.067	493374.8264	13.30954051
UIJ65545.1	argininosuccinate_lyase_[Bacillus_cereus]	CPTF_Cu	4373267.467	788607.9115	18.03246468
UIJ65545.1	argininosuccinate_lyase_[Bacillus_cereus]	CPTF_Fe	3794338.467	441869.9194	11.64550615

UIJ65545.1	argininosuccinate_lyase_[Bacillus_cereus]	CPTF_Mn	3830276.667	1267583.122	33.09377448
UIJ65545.1	argininosuccinate_lyase_[Bacillus_cereus]	CPTF_Ni	3570668.933	244232.8605	6.839974948
UIJ65545.1	argininosuccinate_lyase_[Bacillus_cereus]	CPTF_U	3799171.067	747791.7932	19.68302506
UIJ65545.1	argininosuccinate_lyase_[Bacillus_cereus]	CPTF_metals_mix	4213585.767	556502.2037	13.20733063
UIJ65545.1	argininosuccinate_lyase_[Bacillus_cereus]	CPTF_zcontrol	4422377.867	901362.982	20.38186264
UIJ65546.1	argininosuccinate_synthase_[Bacillus_cereus]	CPTF_Al	6098531.267	439159.7014	7.201073213
UIJ65546.1	argininosuccinate_synthase_[Bacillus_cereus]	CPTF_Cd	6268593.267	451047.5733	7.195355547
UIJ65546.1	argininosuccinate_synthase_[Bacillus_cereus]	CPTF_Co	6589863.933	902912.0854	13.70152851
UIJ65546.1	argininosuccinate_synthase_[Bacillus_cereus]	CPTF_Cu	6882738.633	493454.7991	7.169454274
UIJ65546.1	argininosuccinate_synthase_[Bacillus_cereus]	CPTF_Fe	6033359.067	338586.8123	5.611912179
UIJ65546.1	argininosuccinate_synthase_[Bacillus_cereus]	CPTF_Mn	6342948.1	728385.1491	11.48338498
UIJ65546.1	argininosuccinate_synthase_[Bacillus_cereus]	CPTF_Ni	6209075.5	696967.7163	11.22498376
UIJ65546.1	argininosuccinate_synthase_[Bacillus_cereus]	CPTF_U	4995463.8	335149.9861	6.709086474
UIJ65546.1	argininosuccinate_synthase_[Bacillus_cereus]	CPTF_metals_mix	8240924.2	711655.7953	8.635630883
UIJ65546.1	argininosuccinate_synthase_[Bacillus_cereus]	CPTF_zcontrol	6419328.6	289315.9638	4.506950522
UIJ65549.1	EcsC_family_protein_[Bacillus_cereus]	CPTF_Al	270319	235840.8843	87.24539686
UIJ65549.1	EcsC_family_protein_[Bacillus_cereus]	CPTF_Cd	171534.3333	149626.2646	87.22817276
UIJ65549.1	EcsC_family_protein_[Bacillus_cereus]	CPTF_Co	131911.3333	228477.1314	173.2050808
UIJ65549.1	EcsC_family_protein_[Bacillus_cereus]	CPTF_Cu	417030.6667	91123.77196	21.85061657
UIJ65549.1	EcsC_family_protein_[Bacillus_cereus]	CPTF_Fe	214842	56258.12056	26.18581123
UIJ65549.1	EcsC_family_protein_[Bacillus_cereus]	CPTF_Mn	266744	258785.807	97.01654282
UIJ65549.1	EcsC_family_protein_[Bacillus_cereus]	CPTF_Ni	645701	168178.6678	26.04590481
UIJ65549.1	EcsC_family_protein_[Bacillus_cereus]	CPTF_U	382758.6667	336038.0121	87.79370432
UIJ65549.1	EcsC_family_protein_[Bacillus_cereus]	CPTF_metals_mix	326620.3667	256122.0579	78.41582583
UIJ65549.1	EcsC_family_protein_[Bacillus_cereus]	CPTF_zcontrol	174667.3333	151926.3549	86.98040556
UIJ65552.1	acetate_kinase_[Bacillus_cereus]	CPTF_Al	4932416.033	547640.3329	11.10288202
UIJ65552.1	acetate_kinase_[Bacillus_cereus]	CPTF_Cd	5396190.8	559564.7457	10.36962492
UIJ65552.1	acetate_kinase_[Bacillus_cereus]	CPTF_Co	4487397.267	409427.595	9.123943585
UIJ65552.1	acetate_kinase_[Bacillus_cereus]	CPTF_Cu	4783559.187	185959.3845	3.887469084
UIJ65552.1	acetate_kinase_[Bacillus_cereus]	CPTF_Fe	4993037.3	419936.781	8.410447504
UIJ65552.1	acetate_kinase_[Bacillus_cereus]	CPTF_Mn	4758927.3	559544.0919	11.75777768
UIJ65552.1	acetate_kinase_[Bacillus_cereus]	CPTF_Ni	2426345.4	634992.882	26.17075384
UIJ65552.1	acetate_kinase_[Bacillus_cereus]	CPTF_U	3527680.333	269782.0421	7.647576215
UIJ65552.1	acetate_kinase_[Bacillus_cereus]	CPTF_metals_mix	6451670.1	808550.8482	12.53242704
UIJ65552.1	acetate_kinase_[Bacillus_cereus]	CPTF_zcontrol	4623293.833	554976.9818	12.00393057
UIJ65554.1	thiol_peroxidase_[Bacillus_cereus]	CPTF_Al	3595371.567	1111337.445	30.91022511
UIJ65554.1	thiol_peroxidase_[Bacillus_cereus]	CPTF_Cd	5874150.067	523161.161	8.906159276
UIJ65554.1	thiol_peroxidase_[Bacillus_cereus]	CPTF_Co	3125439.967	916155.4857	29.31284861
UIJ65554.1	thiol_peroxidase_[Bacillus_cereus]	CPTF_Cu	2804817.267	391930.775	13.97348696
UIJ65554.1	thiol_peroxidase_[Bacillus_cereus]	CPTF_Fe	3838040.8	1333286.15	34.73871747
UIJ65554.1	thiol_peroxidase_[Bacillus_cereus]	CPTF_Mn	4710625.6	3211256.227	68.17048306
UIJ65554.1	thiol_peroxidase_[Bacillus_cereus]	CPTF_Ni	1156076.1	353788.3625	30.60251505
UIJ65554.1	thiol_peroxidase_[Bacillus_cereus]	CPTF_U	1564672.333	892784.0255	57.05884909
UIJ65554.1	thiol_peroxidase_[Bacillus_cereus]	CPTF_metals_mix	10291251.2	954179.5475	9.271754513
UIJ65554.1	thiol_peroxidase_[Bacillus_cereus]	CPTF_zcontrol	3216674.767	1154471.06	35.89020166
UIJ65557.1	NAD_kinase_[Bacillus_cereus]	CPTF_Al	2084720.267	45348.90938	2.175299492
UIJ65557.1	NAD_kinase_[Bacillus_cereus]	CPTF_Cd	2286746.7	82591.15844	3.61173183
UIJ65557.1	NAD_kinase_[Bacillus_cereus]	CPTF_Co	1970841.933	105780.0997	5.367254368
UIJ65557.1	NAD_kinase_[Bacillus_cereus]	CPTF_Cu	2174319.767	164639.33	7.571992516
UIJ65557.1	NAD_kinase_[Bacillus_cereus]	CPTF_Fe	2205926.733	104456.4637	4.735264419
UIJ65557.1	NAD_kinase_[Bacillus_cereus]	CPTF_Mn	1974611.567	356136.0205	18.03575075
UIJ65557.1	NAD_kinase_[Bacillus_cereus]	CPTF_Ni	1867194	82356.42945	4.410705553
UIJ65557.1	NAD_kinase_[Bacillus_cereus]	CPTF_U	2130444.967	113487.1465	5.32692223
UIJ65557.1	NAD_kinase_[Bacillus_cereus]	CPTF_metals_mix	2008011.267	133183.3112	6.632597805
UIJ65557.1	NAD_kinase_[Bacillus_cereus]	CPTF_zcontrol	2019869.1	20856.90709	1.032587067
UIJ65559.1	VOC_family_protein_[Bacillus_cereus]	CPTF_Al	0	0	0
UIJ65559.1	VOC_family_protein_[Bacillus_cereus]	CPTF_Cd	0	0	0
UIJ65559.1	VOC_family_protein_[Bacillus_cereus]	CPTF_Co	0	0	0
UIJ65559.1	VOC_family_protein_[Bacillus_cereus]	CPTF_Cu	0	0	0
UIJ65559.1	VOC_family_protein_[Bacillus_cereus]	CPTF_Fe	0	0	0
UIJ65559.1	VOC_family_protein_[Bacillus_cereus]	CPTF_Mn	37876.33333	65603.73374	173.2050808

UIJ65559.1	VOC_family_protein_[Bacillus_cereus]	CPTF_Ni	0	0	0
UIJ65559.1	VOC_family_protein_[Bacillus_cereus]	CPTF_U	0	0	0
UIJ65559.1	VOC_family_protein_[Bacillus_cereus]	CPTF_metals_mix	138794.6667	33722.1309	24.29641694
UIJ65559.1	VOC_family_protein_[Bacillus_cereus]	CPTF_zcontrol	0	0	0
UIJ65563.1	cysteine_desulfurase_[Bacillus_cereus]	CPTF_Al	0	0	0
UIJ65563.1	cysteine_desulfurase_[Bacillus_cereus]	CPTF_Cd	0	0	0
UIJ65563.1	cysteine_desulfurase_[Bacillus_cereus]	CPTF_Co	0	0	0
UIJ65563.1	cysteine_desulfurase_[Bacillus_cereus]	CPTF_Cu	0	0	0
UIJ65563.1	cysteine_desulfurase_[Bacillus_cereus]	CPTF_Fe	0	0	0
UIJ65563.1	cysteine_desulfurase_[Bacillus_cereus]	CPTF_Mn	59603.33333	103236.0016	173.2050808
UIJ65563.1	cysteine_desulfurase_[Bacillus_cereus]	CPTF_Ni	0	0	0
UIJ65563.1	cysteine_desulfurase_[Bacillus_cereus]	CPTF_U	0	0	0
UIJ65563.1	cysteine_desulfurase_[Bacillus_cereus]	CPTF_metals_mix	107218	95344.3257	88.92567079
UIJ65563.1	cysteine_desulfurase_[Bacillus_cereus]	CPTF_zcontrol	0	0	0
UIJ65564.1	septation_ring_formation_regulator_EzrA_[Bacillus_cereus]	CPTF_Al	208890.7667	112051.439	53.64116411
UIJ65564.1	septation_ring_formation_regulator_EzrA_[Bacillus_cereus]	CPTF_Cd	279827.0667	250952.3608	89.681232
UIJ65564.1	septation_ring_formation_regulator_EzrA_[Bacillus_cereus]	CPTF_Co	219747.7667	149440.8297	68.00561933
UIJ65564.1	septation_ring_formation_regulator_EzrA_[Bacillus_cereus]	CPTF_Cu	223498.6667	156891.1976	70.19782261
UIJ65564.1	septation_ring_formation_regulator_EzrA_[Bacillus_cereus]	CPTF_Fe	210731.4667	121426.8097	57.62158427
UIJ65564.1	septation_ring_formation_regulator_EzrA_[Bacillus_cereus]	CPTF_Mn	74709.13333	9331.924625	12.49100908
UIJ65564.1	septation_ring_formation_regulator_EzrA_[Bacillus_cereus]	CPTF_Ni	186557.7667	126222.5226	67.65868012
UIJ65564.1	septation_ring_formation_regulator_EzrA_[Bacillus_cereus]	CPTF_U	207740.7667	113309.024	54.54347059
UIJ65564.1	septation_ring_formation_regulator_EzrA_[Bacillus_cereus]	CPTF_metals_mix	305117.9667	185531.0379	60.80633006
UIJ65564.1	septation_ring_formation_regulator_EzrA_[Bacillus_cereus]	CPTF_zcontrol	114334.1667	141952.5395	124.1558352
UIJ65570.1	30S_ribosomal_protein_S4_[Bacillus_cereus]	CPTF_Al	29169147.2	1493859.95	5.121369988
UIJ65570.1	30S_ribosomal_protein_S4_[Bacillus_cereus]	CPTF_Cd	31509823.53	1653692.039	5.248179309
UIJ65570.1	30S_ribosomal_protein_S4_[Bacillus_cereus]	CPTF_Co	31400028.13	43694.39602	0.139154003
UIJ65570.1	30S_ribosomal_protein_S4_[Bacillus_cereus]	CPTF_Cu	27995163.7	1743263.667	6.22701723
UIJ65570.1	30S_ribosomal_protein_S4_[Bacillus_cereus]	CPTF_Fe	30289500.97	2414833.645	7.972510502
UIJ65570.1	30S_ribosomal_protein_S4_[Bacillus_cereus]	CPTF_Mn	31102267.57	4049064.223	13.0185499
UIJ65570.1	30S_ribosomal_protein_S4_[Bacillus_cereus]	CPTF_Ni	26109719.83	753778.881	2.886966562
UIJ65570.1	30S_ribosomal_protein_S4_[Bacillus_cereus]	CPTF_U	24917814.33	2279483.928	9.148009122
UIJ65570.1	30S_ribosomal_protein_S4_[Bacillus_cereus]	CPTF_metals_mix	30752937	1537326.649	4.998958795
UIJ65570.1	30S_ribosomal_protein_S4_[Bacillus_cereus]	CPTF_zcontrol	28860709.93	1562152.373	5.412730237
UIJ65575.1	tyrosine--tRNA_ligase_[Bacillus_cereus]	CPTF_Al	3233078.667	315713.3893	9.765100756
UIJ65575.1	tyrosine--tRNA_ligase_[Bacillus_cereus]	CPTF_Cd	3432988.333	84624.13346	2.465028286
UIJ65575.1	tyrosine--tRNA_ligase_[Bacillus_cereus]	CPTF_Co	3536844.867	273767.0136	7.740430354
UIJ65575.1	tyrosine--tRNA_ligase_[Bacillus_cereus]	CPTF_Cu	3383854.967	215987.2868	6.382876597
UIJ65575.1	tyrosine--tRNA_ligase_[Bacillus_cereus]	CPTF_Fe	3115961	290890.4574	9.335497376
UIJ65575.1	tyrosine--tRNA_ligase_[Bacillus_cereus]	CPTF_Mn	3337113.067	330069.7139	9.890875954
UIJ65575.1	tyrosine--tRNA_ligase_[Bacillus_cereus]	CPTF_Ni	2923926.667	71648.19427	2.450410097
UIJ65575.1	tyrosine--tRNA_ligase_[Bacillus_cereus]	CPTF_U	3753913.533	39099.77037	1.041573548
UIJ65575.1	tyrosine--tRNA_ligase_[Bacillus_cereus]	CPTF_metals_mix	3262239.2	209274.6271	6.415060768
UIJ65575.1	tyrosine--tRNA_ligase_[Bacillus_cereus]	CPTF_zcontrol	3139217.933	421219.9724	13.41799076
UIJ65576.1	acetate--CoA_ligase_[Bacillus_cereus]	CPTF_Al	8447313.1	236323.3703	2.797615851
UIJ65576.1	acetate--CoA_ligase_[Bacillus_cereus]	CPTF_Cd	10003761.73	689381.3223	6.891220929
UIJ65576.1	acetate--CoA_ligase_[Bacillus_cereus]	CPTF_Co	7645226.533	218799.397	2.861908617
UIJ65576.1	acetate--CoA_ligase_[Bacillus_cereus]	CPTF_Cu	5503015	169037.8858	3.071732237
UIJ65576.1	acetate--CoA_ligase_[Bacillus_cereus]	CPTF_Fe	9040551.967	619632.478	6.853923082
UIJ65576.1	acetate--CoA_ligase_[Bacillus_cereus]	CPTF_Mn	8428907.133	415032.9873	4.923924071
UIJ65576.1	acetate--CoA_ligase_[Bacillus_cereus]	CPTF_Ni	6418644.5	72227.77936	1.125280881
UIJ65576.1	acetate--CoA_ligase_[Bacillus_cereus]	CPTF_U	6755440.967	1648285.289	24.39937374
UIJ65576.1	acetate--CoA_ligase_[Bacillus_cereus]	CPTF_metals_mix	4389606	746910.6574	17.01543732
UIJ65576.1	acetate--CoA_ligase_[Bacillus_cereus]	CPTF_zcontrol	7974049.9	823163.5124	10.32302936
UIJ65577.1	acetoin_utilization_protein_acetyltransferase_AcuA_[Bacillus_cereus]	CPTF_Al	217166.7	44977.13525	20.71088028
UIJ65577.1	acetoin_utilization_protein_acetyltransferase_AcuA_[Bacillus_cereus]	CPTF_Cd	107036.3	135319.8108	126.4242232
UIJ65577.1	acetoin_utilization_protein_acetyltransferase_AcuA_[Bacillus_cereus]	CPTF_Co	264162.9667	59118.34562	22.37949792
UIJ65577.1	acetoin_utilization_protein_acetyltransferase_AcuA_[Bacillus_cereus]	CPTF_Cu	235886.6667	11643.54934	4.936077782
UIJ65577.1	acetoin_utilization_protein_acetyltransferase_AcuA_[Bacillus_cereus]	CPTF_Fe	209275.3333	43999.39611	21.0246451
UIJ65577.1	acetoin_utilization_protein_acetyltransferase_AcuA_[Bacillus_cereus]	CPTF_Mn	120070.9	43121.53378	35.91339265
UIJ65577.1	acetoin_utilization_protein_acetyltransferase_AcuA_[Bacillus_cereus]	CPTF_Ni	201277.6	42507.06756	21.11862798

UIJ65577.1	acetoin_utilization_protein_acetyltransferase_AcuA_[Bacillus_cereus]	CPTF_U	163442.1333	84998.03849	52.00497372
UIJ65577.1	acetoin_utilization_protein_acetyltransferase_AcuA_[Bacillus_cereus]	CPTF_metals_mix	108967.2333	97882.95916	89.82788326
UIJ65577.1	acetoin_utilization_protein_acetyltransferase_AcuA_[Bacillus_cereus]	CPTF_zcontrol	76466.96667	100227.9166	131.073483
UIJ65578.1	acetoin_utilization_AcuB_family_protein_[Bacillus_cereus]	CPTF_Al	377714.2	360141.0664	95.34750517
UIJ65578.1	acetoin_utilization_AcuB_family_protein_[Bacillus_cereus]	CPTF_Cd	30689.9	26578.25297	86.60260532
UIJ65578.1	acetoin_utilization_AcuB_family_protein_[Bacillus_cereus]	CPTF_Co	76502.63333	71020.34942	92.83386248
UIJ65578.1	acetoin_utilization_AcuB_family_protein_[Bacillus_cereus]	CPTF_Cu	0	0	0
UIJ65578.1	acetoin_utilization_AcuB_family_protein_[Bacillus_cereus]	CPTF_Fe	239433.4667	345792.3513	144.4210603
UIJ65578.1	acetoin_utilization_AcuB_family_protein_[Bacillus_cereus]	CPTF_Mn	261629.6667	453155.8754	173.2050808
UIJ65578.1	acetoin_utilization_AcuB_family_protein_[Bacillus_cereus]	CPTF_Ni	0	0	0
UIJ65578.1	acetoin_utilization_AcuB_family_protein_[Bacillus_cereus]	CPTF_U	648639.6667	50346.42948	7.761848691
UIJ65578.1	acetoin_utilization_AcuB_family_protein_[Bacillus_cereus]	CPTF_metals_mix	496316.6333	51546.53659	10.38581686
UIJ65578.1	acetoin_utilization_AcuB_family_protein_[Bacillus_cereus]	CPTF_zcontrol	201418	348866.2096	173.2050808
UIJ65579.1	acetoin_utilization_protein_AcuC_[Bacillus_cereus]	CPTF_Al	629351.3333	139313.5254	22.13604994
UIJ65579.1	acetoin_utilization_protein_AcuC_[Bacillus_cereus]	CPTF_Cd	653656.2667	170646.0411	26.10638799
UIJ65579.1	acetoin_utilization_protein_AcuC_[Bacillus_cereus]	CPTF_Co	512202.2333	141930.8886	27.70993162
UIJ65579.1	acetoin_utilization_protein_AcuC_[Bacillus_cereus]	CPTF_Cu	494001.9333	107855.6075	21.83303348
UIJ65579.1	acetoin_utilization_protein_AcuC_[Bacillus_cereus]	CPTF_Fe	654829.7333	263835.1436	40.29064811
UIJ65579.1	acetoin_utilization_protein_AcuC_[Bacillus_cereus]	CPTF_Mn	492626.1333	298680.2363	60.63020535
UIJ65579.1	acetoin_utilization_protein_AcuC_[Bacillus_cereus]	CPTF_Ni	751222.4333	158590.6773	21.11101456
UIJ65579.1	acetoin_utilization_protein_AcuC_[Bacillus_cereus]	CPTF_U	475398.5667	186178.6228	39.16263865
UIJ65579.1	acetoin_utilization_protein_AcuC_[Bacillus_cereus]	CPTF_metals_mix	223739.9333	62377.71727	27.87956371
UIJ65579.1	acetoin_utilization_protein_AcuC_[Bacillus_cereus]	CPTF_zcontrol	409586.6333	16885.43883	4.122556122
UIJ65584.1	Gfo/Idh/MocA_family_oxidoreductase_[Bacillus_cereus]	CPTF_Al	0	0	0
UIJ65584.1	Gfo/Idh/MocA_family_oxidoreductase_[Bacillus_cereus]	CPTF_Cd	19462.1	18209.57513	93.56428714
UIJ65584.1	Gfo/Idh/MocA_family_oxidoreductase_[Bacillus_cereus]	CPTF_Co	0	0	0
UIJ65584.1	Gfo/Idh/MocA_family_oxidoreductase_[Bacillus_cereus]	CPTF_Cu	0	0	0
UIJ65584.1	Gfo/Idh/MocA_family_oxidoreductase_[Bacillus_cereus]	CPTF_Fe	0	0	0
UIJ65584.1	Gfo/Idh/MocA_family_oxidoreductase_[Bacillus_cereus]	CPTF_Mn	0	0	0
UIJ65584.1	Gfo/Idh/MocA_family_oxidoreductase_[Bacillus_cereus]	CPTF_Ni	3807.133333	6594.148365	173.2050808
UIJ65584.1	Gfo/Idh/MocA_family_oxidoreductase_[Bacillus_cereus]	CPTF_U	0	0	0
UIJ65584.1	Gfo/Idh/MocA_family_oxidoreductase_[Bacillus_cereus]	CPTF_metals_mix	56577.63333	18767.48783	33.17121399
UIJ65584.1	Gfo/Idh/MocA_family_oxidoreductase_[Bacillus_cereus]	CPTF_zcontrol	0	0	0
UIJ65589.1	catabolite_control_protein_A_[Bacillus_cereus]	CPTF_Al	331883.6	125921.4226	37.94144169
UIJ65589.1	catabolite_control_protein_A_[Bacillus_cereus]	CPTF_Cd	327554.7333	123911.7832	37.82933678
UIJ65589.1	catabolite_control_protein_A_[Bacillus_cereus]	CPTF_Co	620221.3333	232730.1528	37.52372585
UIJ65589.1	catabolite_control_protein_A_[Bacillus_cereus]	CPTF_Cu	546549.7333	92485.18484	16.9216412
UIJ65589.1	catabolite_control_protein_A_[Bacillus_cereus]	CPTF_Fe	289693.4667	217715.7689	75.15384154
UIJ65589.1	catabolite_control_protein_A_[Bacillus_cereus]	CPTF_Mn	381517.3667	141001.6124	36.95811113
UIJ65589.1	catabolite_control_protein_A_[Bacillus_cereus]	CPTF_Ni	231310.5	156494.7163	67.65569066
UIJ65589.1	catabolite_control_protein_A_[Bacillus_cereus]	CPTF_U	85634.56667	148323.4204	173.2050808
UIJ65589.1	catabolite_control_protein_A_[Bacillus_cereus]	CPTF_metals_mix	1637574.867	475649.0067	29.0459396
UIJ65589.1	catabolite_control_protein_A_[Bacillus_cereus]	CPTF_zcontrol	351098.3333	72622.50383	20.68437726
UIJ65591.1	CamS_family_sex_pheromone_protein_[Bacillus_cereus]	CPTF_Al	0	0	0
UIJ65591.1	CamS_family_sex_pheromone_protein_[Bacillus_cereus]	CPTF_Cd	0	0	0
UIJ65591.1	CamS_family_sex_pheromone_protein_[Bacillus_cereus]	CPTF_Co	27104.7	23476.30388	86.6134061
UIJ65591.1	CamS_family_sex_pheromone_protein_[Bacillus_cereus]	CPTF_Cu	65412.46667	13513.41738	20.65877969
UIJ65591.1	CamS_family_sex_pheromone_protein_[Bacillus_cereus]	CPTF_Fe	0	0	0
UIJ65591.1	CamS_family_sex_pheromone_protein_[Bacillus_cereus]	CPTF_Mn	0	0	0
UIJ65591.1	CamS_family_sex_pheromone_protein_[Bacillus_cereus]	CPTF_Ni	35949.73333	35483.28078	98.70248675
UIJ65591.1	CamS_family_sex_pheromone_protein_[Bacillus_cereus]	CPTF_U	0	0	0
UIJ65591.1	CamS_family_sex_pheromone_protein_[Bacillus_cereus]	CPTF_metals_mix	58212.9	20696.64825	35.5533709
UIJ65591.1	CamS_family_sex_pheromone_protein_[Bacillus_cereus]	CPTF_zcontrol	15423.46667	26714.2279	173.2050808
UIJ65593.1	DUF948_domain-containing_protein_[Bacillus_cereus]	CPTF_Al	634464.3333	298057.4302	46.97780704
UIJ65593.1	DUF948_domain-containing_protein_[Bacillus_cereus]	CPTF_Cd	529545	293085.2611	55.34662042
UIJ65593.1	DUF948_domain-containing_protein_[Bacillus_cereus]	CPTF_Co	520738.6667	278819.412	53.54305909
UIJ65593.1	DUF948_domain-containing_protein_[Bacillus_cereus]	CPTF_Cu	844828.3333	17910.57259	2.120025084
UIJ65593.1	DUF948_domain-containing_protein_[Bacillus_cereus]	CPTF_Fe	578561.6667	226204.7767	39.09778157
UIJ65593.1	DUF948_domain-containing_protein_[Bacillus_cereus]	CPTF_Mn	523255.3333	117015.6274	22.36300711
UIJ65593.1	DUF948_domain-containing_protein_[Bacillus_cereus]	CPTF_Ni	387614.3333	157922.3487	40.74213338
UIJ65593.1	DUF948_domain-containing_protein_[Bacillus_cereus]	CPTF_U	496139.4	129779.8028	26.15793119

UIJ65593.1	DUF948_domain-containing_protein_[Bacillus_cereus]	CPTF_metals_mix	872283.3333	64166.01201	7.356097447
UIJ65593.1	DUF948_domain-containing_protein_[Bacillus_cereus]	CPTF_zcontrol	675369.4	243181.1239	36.00712793
UIJ65594.1	aminopeptidase_[Bacillus_cereus]	CPTF_Al	3149467.333	109941.1665	3.490786057
UIJ65594.1	aminopeptidase_[Bacillus_cereus]	CPTF_Cd	2770696	199184.3289	7.188963673
UIJ65594.1	aminopeptidase_[Bacillus_cereus]	CPTF_Co	2949944	226702.7342	7.684984333
UIJ65594.1	aminopeptidase_[Bacillus_cereus]	CPTF_Cu	3126203.7	31049.81476	0.993211503
UIJ65594.1	aminopeptidase_[Bacillus_cereus]	CPTF_Fe	2902847.333	178921.9752	6.163671551
UIJ65594.1	aminopeptidase_[Bacillus_cereus]	CPTF_Mn	3100523.333	238261.5183	7.684558144
UIJ65594.1	aminopeptidase_[Bacillus_cereus]	CPTF_Ni	2941252.567	158783.7553	5.398508005
UIJ65594.1	aminopeptidase_[Bacillus_cereus]	CPTF_U	2771779.3	103006.1842	3.71624769
UIJ65594.1	aminopeptidase_[Bacillus_cereus]	CPTF_metals_mix	2903156.167	309379.0739	10.65664594
UIJ65594.1	aminopeptidase_[Bacillus_cereus]	CPTF_zcontrol	2963318	198287.8787	6.691414106
UIJ65601.1	UDP-N-acetylmuramate--L-alanine_ligase_[Bacillus_cereus]	CPTF_Al	510359.0667	261941.4111	51.32492556
UIJ65601.1	UDP-N-acetylmuramate--L-alanine_ligase_[Bacillus_cereus]	CPTF_Cd	484989.9667	309741.0417	63.86545352
UIJ65601.1	UDP-N-acetylmuramate--L-alanine_ligase_[Bacillus_cereus]	CPTF_Co	604350.4667	101979.9003	16.87429826
UIJ65601.1	UDP-N-acetylmuramate--L-alanine_ligase_[Bacillus_cereus]	CPTF_Cu	778836.4	86510.29634	11.10763394
UIJ65601.1	UDP-N-acetylmuramate--L-alanine_ligase_[Bacillus_cereus]	CPTF_Fe	426828.1	290617.8868	68.08780555
UIJ65601.1	UDP-N-acetylmuramate--L-alanine_ligase_[Bacillus_cereus]	CPTF_Mn	278896.7333	81792.03923	29.32699794
UIJ65601.1	UDP-N-acetylmuramate--L-alanine_ligase_[Bacillus_cereus]	CPTF_Ni	414509.8333	230581.2572	55.62745166
UIJ65601.1	UDP-N-acetylmuramate--L-alanine_ligase_[Bacillus_cereus]	CPTF_U	304385.3333	69274.1937	22.75871605
UIJ65601.1	UDP-N-acetylmuramate--L-alanine_ligase_[Bacillus_cereus]	CPTF_metals_mix	526866.3	54663.53545	10.37521957
UIJ65601.1	UDP-N-acetylmuramate--L-alanine_ligase_[Bacillus_cereus]	CPTF_zcontrol	445779.1667	191271.3395	42.90719571
UIJ65602.1	nicotinate_phosphoribosyltransferase_[Bacillus_cereus]	CPTF_Al	10998.4	19049.7876	173.2050808
UIJ65602.1	nicotinate_phosphoribosyltransferase_[Bacillus_cereus]	CPTF_Cd	6241.766667	10811.057	173.2050808
UIJ65602.1	nicotinate_phosphoribosyltransferase_[Bacillus_cereus]	CPTF_Co	6006.033333	10402.75489	173.2050808
UIJ65602.1	nicotinate_phosphoribosyltransferase_[Bacillus_cereus]	CPTF_Cu	5182.666667	8976.641985	173.2050808
UIJ65602.1	nicotinate_phosphoribosyltransferase_[Bacillus_cereus]	CPTF_Fe	8522.333333	14761.11433	173.2050808
UIJ65602.1	nicotinate_phosphoribosyltransferase_[Bacillus_cereus]	CPTF_Mn	75455.76667	73277.02974	97.11256406
UIJ65602.1	nicotinate_phosphoribosyltransferase_[Bacillus_cereus]	CPTF_Ni	7776.966667	13470.1014	173.2050808
UIJ65602.1	nicotinate_phosphoribosyltransferase_[Bacillus_cereus]	CPTF_U	0	0	0
UIJ65602.1	nicotinate_phosphoribosyltransferase_[Bacillus_cereus]	CPTF_metals_mix	49482.86667	58004.29636	117.2209701
UIJ65602.1	nicotinate_phosphoribosyltransferase_[Bacillus_cereus]	CPTF_zcontrol	32133.33333	55656.56595	173.2050808
UIJ65603.1	DNA_translocase_FtsK_[Bacillus_cereus]	CPTF_Al	0	0	0
UIJ65603.1	DNA_translocase_FtsK_[Bacillus_cereus]	CPTF_Cd	0	0	0
UIJ65603.1	DNA_translocase_FtsK_[Bacillus_cereus]	CPTF_Co	14303.16667	24773.81138	173.2050808
UIJ65603.1	DNA_translocase_FtsK_[Bacillus_cereus]	CPTF_Cu	0	0	0
UIJ65603.1	DNA_translocase_FtsK_[Bacillus_cereus]	CPTF_Fe	0	0	0
UIJ65603.1	DNA_translocase_FtsK_[Bacillus_cereus]	CPTF_Mn	0	0	0
UIJ65603.1	DNA_translocase_FtsK_[Bacillus_cereus]	CPTF_Ni	0	0	0
UIJ65603.1	DNA_translocase_FtsK_[Bacillus_cereus]	CPTF_U	0	0	0
UIJ65603.1	DNA_translocase_FtsK_[Bacillus_cereus]	CPTF_metals_mix	14838.26667	25700.63176	173.2050808
UIJ65603.1	DNA_translocase_FtsK_[Bacillus_cereus]	CPTF_zcontrol	0	0	0
UIJ65606.1	DUF4479_domain-containing_protein_[Bacillus_cereus]	CPTF_Al	2221640.233	45949.34062	2.068261995
UIJ65606.1	DUF4479_domain-containing_protein_[Bacillus_cereus]	CPTF_Cd	2303617.533	74496.8279	3.233906099
UIJ65606.1	DUF4479_domain-containing_protein_[Bacillus_cereus]	CPTF_Co	2268289.333	85690.25651	3.77774807
UIJ65606.1	DUF4479_domain-containing_protein_[Bacillus_cereus]	CPTF_Cu	2168150	79381.42932	3.661251727
UIJ65606.1	DUF4479_domain-containing_protein_[Bacillus_cereus]	CPTF_Fe	2214168.333	122383.9592	5.52731052
UIJ65606.1	DUF4479_domain-containing_protein_[Bacillus_cereus]	CPTF_Mn	2120632.667	286107.1988	13.49159632
UIJ65606.1	DUF4479_domain-containing_protein_[Bacillus_cereus]	CPTF_Ni	2296879.333	129085.7916	5.620051072
UIJ65606.1	DUF4479_domain-containing_protein_[Bacillus_cereus]	CPTF_U	2556196.4	45029.24055	1.76157202
UIJ65606.1	DUF4479_domain-containing_protein_[Bacillus_cereus]	CPTF_metals_mix	1897373.5	75435.68221	3.975795077
UIJ65606.1	DUF4479_domain-containing_protein_[Bacillus_cereus]	CPTF_zcontrol	2124695.3	89181.05564	4.197357411
UIJ65608.1	thioredoxin_family_protein_[Bacillus_cereus]	CPTF_Al	1001775.333	208450.0954	20.80806828
UIJ65608.1	thioredoxin_family_protein_[Bacillus_cereus]	CPTF_Cd	1386162.3	73006.23883	5.26678866
UIJ65608.1	thioredoxin_family_protein_[Bacillus_cereus]	CPTF_Co	983968	141936.0353	14.42486294
UIJ65608.1	thioredoxin_family_protein_[Bacillus_cereus]	CPTF_Cu	876273.6667	88417.31715	10.09014883
UIJ65608.1	thioredoxin_family_protein_[Bacillus_cereus]	CPTF_Fe	1122662.667	97466.55171	8.681730907
UIJ65608.1	thioredoxin_family_protein_[Bacillus_cereus]	CPTF_Mn	925620.3333	172790.7316	18.66756005
UIJ65608.1	thioredoxin_family_protein_[Bacillus_cereus]	CPTF_Ni	581101.3333	66928.1387	11.51746431
UIJ65608.1	thioredoxin_family_protein_[Bacillus_cereus]	CPTF_U	902601.3333	132247.0662	14.65176943
UIJ65608.1	thioredoxin_family_protein_[Bacillus_cereus]	CPTF_metals_mix	2116633.533	212358.7336	10.03285312

UJJ65608.1	thioredoxin_family_protein_[Bacillus_cereus]	CPTF_zcontrol	789459	158606.2243	20.09049543
UJJ65610.1	M42_family_metallopeptidase_[Bacillus_cereus]	CPTF_Al	3879212.333	246960.8687	6.366263238
UJJ65610.1	M42_family_metallopeptidase_[Bacillus_cereus]	CPTF_Cd	4025105	531809.5039	13.21231381
UJJ65610.1	M42_family_metallopeptidase_[Bacillus_cereus]	CPTF_Co	4064262.667	442779.2915	10.89445559
UJJ65610.1	M42_family_metallopeptidase_[Bacillus_cereus]	CPTF_Cu	3247545	591476.828	18.21304487
UJJ65610.1	M42_family_metallopeptidase_[Bacillus_cereus]	CPTF_Fe	3618176	480473.4296	13.27943775
UJJ65610.1	M42_family_metallopeptidase_[Bacillus_cereus]	CPTF_Mn	4542523	645815.0337	14.21709992
UJJ65610.1	M42_family_metallopeptidase_[Bacillus_cereus]	CPTF_Ni	3175724.333	646945.7298	20.37159595
UJJ65610.1	M42_family_metallopeptidase_[Bacillus_cereus]	CPTF_U	3008096	234660.2973	7.800957725
UJJ65610.1	M42_family_metallopeptidase_[Bacillus_cereus]	CPTF_metals_mix	3813132.667	431653.8504	11.32018968
UJJ65610.1	M42_family_metallopeptidase_[Bacillus_cereus]	CPTF_zcontrol	3602597.333	596492.5133	16.5572907
UJJ65611.1	PepSY_domain-containing_protein_[Bacillus_cereus]	CPTF_Al	750152.5	62235.87617	8.296429883
UJJ65611.1	PepSY_domain-containing_protein_[Bacillus_cereus]	CPTF_Cd	817439.3333	43319.87655	5.299460741
UJJ65611.1	PepSY_domain-containing_protein_[Bacillus_cereus]	CPTF_Co	667361.3333	94700.21184	14.19024554
UJJ65611.1	PepSY_domain-containing_protein_[Bacillus_cereus]	CPTF_Cu	726402.9333	124946.6612	17.20073742
UJJ65611.1	PepSY_domain-containing_protein_[Bacillus_cereus]	CPTF_Fe	728703.8	52204.04928	7.163960073
UJJ65611.1	PepSY_domain-containing_protein_[Bacillus_cereus]	CPTF_Mn	650292.1333	124095.0403	19.08296808
UJJ65611.1	PepSY_domain-containing_protein_[Bacillus_cereus]	CPTF_Ni	652711.5667	73109.04904	11.20082021
UJJ65611.1	PepSY_domain-containing_protein_[Bacillus_cereus]	CPTF_U	759239.3667	55890.65411	7.361400971
UJJ65611.1	PepSY_domain-containing_protein_[Bacillus_cereus]	CPTF_metals_mix	776104.3333	11270.29903	1.452162879
UJJ65611.1	PepSY_domain-containing_protein_[Bacillus_cereus]	CPTF_zcontrol	698069.4333	53140.39099	7.612479282
UJJ65612.1	MBL_fold_metallo-hydrolase_[Bacillus_cereus]	CPTF_Al	170607.6	47584.12534	27.89097633
UJJ65612.1	MBL_fold_metallo-hydrolase_[Bacillus_cereus]	CPTF_Cd	159127.4	21993.94775	13.82159688
UJJ65612.1	MBL_fold_metallo-hydrolase_[Bacillus_cereus]	CPTF_Co	134353.3667	40673.56497	30.27357332
UJJ65612.1	MBL_fold_metallo-hydrolase_[Bacillus_cereus]	CPTF_Cu	130398	10021.96528	7.685674072
UJJ65612.1	MBL_fold_metallo-hydrolase_[Bacillus_cereus]	CPTF_Fe	128162.3333	7107.393357	5.545617946
UJJ65612.1	MBL_fold_metallo-hydrolase_[Bacillus_cereus]	CPTF_Mn	128892	2702.160432	2.096453179
UJJ65612.1	MBL_fold_metallo-hydrolase_[Bacillus_cereus]	CPTF_Ni	131393.3333	26949.9445	20.51089185
UJJ65612.1	MBL_fold_metallo-hydrolase_[Bacillus_cereus]	CPTF_U	37807.33333	65484.22223	173.2050808
UJJ65612.1	MBL_fold_metallo-hydrolase_[Bacillus_cereus]	CPTF_metals_mix	93340.13333	39185.27293	41.98116237
UJJ65612.1	MBL_fold_metallo-hydrolase_[Bacillus_cereus]	CPTF_zcontrol	88121.03333	40441.55993	45.89319757
UJJ65618.1	dipeptidase_PepV_[Bacillus_cereus]	CPTF_Al	2378710.567	661834.287	27.82323736
UJJ65618.1	dipeptidase_PepV_[Bacillus_cereus]	CPTF_Cd	2315217.933	397988.9849	17.19013054
UJJ65618.1	dipeptidase_PepV_[Bacillus_cereus]	CPTF_Co	2888929.133	203273.7668	7.036301599
UJJ65618.1	dipeptidase_PepV_[Bacillus_cereus]	CPTF_Cu	2838338.933	406238.9498	14.31255954
UJJ65618.1	dipeptidase_PepV_[Bacillus_cereus]	CPTF_Fe	2660158.733	204671.2875	7.693950173
UJJ65618.1	dipeptidase_PepV_[Bacillus_cereus]	CPTF_Mn	2239711.567	208823.1021	9.323660473
UJJ65618.1	dipeptidase_PepV_[Bacillus_cereus]	CPTF_Ni	2265892.567	408427.2136	18.02500346
UJJ65618.1	dipeptidase_PepV_[Bacillus_cereus]	CPTF_U	2240905.7	124973.1055	5.576901585
UJJ65618.1	dipeptidase_PepV_[Bacillus_cereus]	CPTF_metals_mix	2930176.467	277581.0777	9.473186372
UJJ65618.1	dipeptidase_PepV_[Bacillus_cereus]	CPTF_zcontrol	2340020.533	184215.0559	7.872369206
UJJ65626.1	SRPBCC_domain-containing_protein_[Bacillus_cereus]	CPTF_Al	26775	23258.36556	86.86597783
UJJ65626.1	SRPBCC_domain-containing_protein_[Bacillus_cereus]	CPTF_Cd	49204.16667	21361.86968	43.41475759
UJJ65626.1	SRPBCC_domain-containing_protein_[Bacillus_cereus]	CPTF_Co	46454.6	40254.15636	86.65268103
UJJ65626.1	SRPBCC_domain-containing_protein_[Bacillus_cereus]	CPTF_Cu	78888.7	9272.871578	11.7543724
UJJ65626.1	SRPBCC_domain-containing_protein_[Bacillus_cereus]	CPTF_Fe	43211.53333	37527.16607	86.84525444
UJJ65626.1	SRPBCC_domain-containing_protein_[Bacillus_cereus]	CPTF_Mn	27626.26667	47850.09749	173.2050808
UJJ65626.1	SRPBCC_domain-containing_protein_[Bacillus_cereus]	CPTF_Ni	41479.13333	36144.53347	87.13907588
UJJ65626.1	SRPBCC_domain-containing_protein_[Bacillus_cereus]	CPTF_U	21672.63333	37538.10207	173.2050808
UJJ65626.1	SRPBCC_domain-containing_protein_[Bacillus_cereus]	CPTF_metals_mix	83620.36667	40930.71269	48.94825785
UJJ65626.1	SRPBCC_domain-containing_protein_[Bacillus_cereus]	CPTF_zcontrol	0	0	0
UJJ65630.1	transcription_factor_YdeB_[Bacillus_cereus]	CPTF_Al	0	0	0
UJJ65630.1	transcription_factor_YdeB_[Bacillus_cereus]	CPTF_Cd	0	0	0
UJJ65630.1	transcription_factor_YdeB_[Bacillus_cereus]	CPTF_Co	0	0	0
UJJ65630.1	transcription_factor_YdeB_[Bacillus_cereus]	CPTF_Cu	0	0	0
UJJ65630.1	transcription_factor_YdeB_[Bacillus_cereus]	CPTF_Fe	0	0	0
UJJ65630.1	transcription_factor_YdeB_[Bacillus_cereus]	CPTF_Mn	0	0	0
UJJ65630.1	transcription_factor_YdeB_[Bacillus_cereus]	CPTF_Ni	13882.76667	24045.65722	173.2050808
UJJ65630.1	transcription_factor_YdeB_[Bacillus_cereus]	CPTF_U	0	0	0
UJJ65630.1	transcription_factor_YdeB_[Bacillus_cereus]	CPTF_metals_mix	0	0	0
UJJ65630.1	transcription_factor_YdeB_[Bacillus_cereus]	CPTF_zcontrol	0	0	0

UIJ65634.1	molybdopterin_synthase_catalytic_subunit_MoaE_[Bacillus_cereus]	CPTF_Al	24028.63333	26038.47984	108.3643813
UIJ65634.1	molybdopterin_synthase_catalytic_subunit_MoaE_[Bacillus_cereus]	CPTF_Cd	14579.73333	25252.83889	173.2050808
UIJ65634.1	molybdopterin_synthase_catalytic_subunit_MoaE_[Bacillus_cereus]	CPTF_Co	0	0	0
UIJ65634.1	molybdopterin_synthase_catalytic_subunit_MoaE_[Bacillus_cereus]	CPTF_Cu	9981.2	17287.94552	173.2050808
UIJ65634.1	molybdopterin_synthase_catalytic_subunit_MoaE_[Bacillus_cereus]	CPTF_Fe	0	0	0
UIJ65634.1	molybdopterin_synthase_catalytic_subunit_MoaE_[Bacillus_cereus]	CPTF_Mn	0	0	0
UIJ65634.1	molybdopterin_synthase_catalytic_subunit_MoaE_[Bacillus_cereus]	CPTF_Ni	38140.16667	44037.10876	115.4612384
UIJ65634.1	molybdopterin_synthase_catalytic_subunit_MoaE_[Bacillus_cereus]	CPTF_U	6586.733333	11408.55679	173.2050808
UIJ65634.1	molybdopterin_synthase_catalytic_subunit_MoaE_[Bacillus_cereus]	CPTF_metals_mix	35240.16667	34029.92013	96.56571846
UIJ65634.1	molybdopterin_synthase_catalytic_subunit_MoaE_[Bacillus_cereus]	CPTF_zcontrol	28211.4	25797.23272	91.4425825
UIJ65636.1	molybdopterin_molybdotransferase_MoeA_[Bacillus_cereus]	CPTF_Al	87788.56667	22782.69897	25.95178374
UIJ65636.1	molybdopterin_molybdotransferase_MoeA_[Bacillus_cereus]	CPTF_Cd	74083.53333	40672.95168	54.90147385
UIJ65636.1	molybdopterin_molybdotransferase_MoeA_[Bacillus_cereus]	CPTF_Co	56518.8	50036.21747	88.5302191
UIJ65636.1	molybdopterin_molybdotransferase_MoeA_[Bacillus_cereus]	CPTF_Cu	54670.8	47581.87443	87.03343363
UIJ65636.1	molybdopterin_molybdotransferase_MoeA_[Bacillus_cereus]	CPTF_Fe	75007.33333	17567.49699	23.42103927
UIJ65636.1	molybdopterin_molybdotransferase_MoeA_[Bacillus_cereus]	CPTF_Mn	25602.46667	44344.77307	173.2050808
UIJ65636.1	molybdopterin_molybdotransferase_MoeA_[Bacillus_cereus]	CPTF_Ni	104567.5333	24304.64035	23.2430082
UIJ65636.1	molybdopterin_molybdotransferase_MoeA_[Bacillus_cereus]	CPTF_U	104443.7333	39752.43072	38.06109706
UIJ65636.1	molybdopterin_molybdotransferase_MoeA_[Bacillus_cereus]	CPTF_metals_mix	45209.63333	21606.31997	47.79140722
UIJ65636.1	molybdopterin_molybdotransferase_MoeA_[Bacillus_cereus]	CPTF_zcontrol	0	0	0
UIJ65637.1	cyclic_pyranopterin_monophosphate_synthase_MoaC_[Bacillus_cereus]	CPTF_Al	69393.36667	68519.39585	98.74055568
UIJ65637.1	cyclic_pyranopterin_monophosphate_synthase_MoaC_[Bacillus_cereus]	CPTF_Cd	43104.33333	74658.89536	173.2050808
UIJ65637.1	cyclic_pyranopterin_monophosphate_synthase_MoaC_[Bacillus_cereus]	CPTF_Co	38579	66820.78811	173.2050808
UIJ65637.1	cyclic_pyranopterin_monophosphate_synthase_MoaC_[Bacillus_cereus]	CPTF_Cu	115126	5350.41746	4.647444939
UIJ65637.1	cyclic_pyranopterin_monophosphate_synthase_MoaC_[Bacillus_cereus]	CPTF_Fe	56608.8	49027.40494	86.60739133
UIJ65637.1	cyclic_pyranopterin_monophosphate_synthase_MoaC_[Bacillus_cereus]	CPTF_Mn	21013.83333	36397.027	173.2050808
UIJ65637.1	cyclic_pyranopterin_monophosphate_synthase_MoaC_[Bacillus_cereus]	CPTF_Ni	48522.5	48577.24222	100.1128182
UIJ65637.1	cyclic_pyranopterin_monophosphate_synthase_MoaC_[Bacillus_cereus]	CPTF_U	56088	66099.36995	117.8493973
UIJ65637.1	cyclic_pyranopterin_monophosphate_synthase_MoaC_[Bacillus_cereus]	CPTF_metals_mix	164878	23637.39721	14.33629545
UIJ65637.1	cyclic_pyranopterin_monophosphate_synthase_MoaC_[Bacillus_cereus]	CPTF_zcontrol	0	0	0
UIJ65638.1	molybdopterin-synthase_adenylyltransferase_MoeB_[Bacillus_cereus]	CPTF_Al	43860	75967.74842	173.2050808
UIJ65638.1	molybdopterin-synthase_adenylyltransferase_MoeB_[Bacillus_cereus]	CPTF_Cd	65244	62280.55444	95.45790331
UIJ65638.1	molybdopterin-synthase_adenylyltransferase_MoeB_[Bacillus_cereus]	CPTF_Co	87574.53333	20843.58846	23.80097006
UIJ65638.1	molybdopterin-synthase_adenylyltransferase_MoeB_[Bacillus_cereus]	CPTF_Cu	58168.5	55393.10139	95.22869146
UIJ65638.1	molybdopterin-synthase_adenylyltransferase_MoeB_[Bacillus_cereus]	CPTF_Fe	64774.26667	65396.1262	100.960041
UIJ65638.1	molybdopterin-synthase_adenylyltransferase_MoeB_[Bacillus_cereus]	CPTF_Mn	26432.96667	45783.24126	173.2050808
UIJ65638.1	molybdopterin-synthase_adenylyltransferase_MoeB_[Bacillus_cereus]	CPTF_Ni	77321.7	69250.98471	89.56216005
UIJ65638.1	molybdopterin-synthase_adenylyltransferase_MoeB_[Bacillus_cereus]	CPTF_U	26302.9	45557.95919	173.2050808
UIJ65638.1	molybdopterin-synthase_adenylyltransferase_MoeB_[Bacillus_cereus]	CPTF_metals_mix	57433.96667	6574.584635	11.44720627
UIJ65638.1	molybdopterin-synthase_adenylyltransferase_MoeB_[Bacillus_cereus]	CPTF_zcontrol	44114.23333	45461.14547	103.0532371
UIJ65643.1	rhodanese-like_domain-containing_protein_[Bacillus_cereus]	CPTF_Al	37661.43333	34077.74102	90.48445056
UIJ65643.1	rhodanese-like_domain-containing_protein_[Bacillus_cereus]	CPTF_Cd	204895	109504.1866	53.44405019
UIJ65643.1	rhodanese-like_domain-containing_protein_[Bacillus_cereus]	CPTF_Co	38190.73333	33307.85538	87.21449543
UIJ65643.1	rhodanese-like_domain-containing_protein_[Bacillus_cereus]	CPTF_Cu	112403.3333	99954.0338	88.92443919
UIJ65643.1	rhodanese-like_domain-containing_protein_[Bacillus_cereus]	CPTF_Fe	180390	41697.57795	23.11523807
UIJ65643.1	rhodanese-like_domain-containing_protein_[Bacillus_cereus]	CPTF_Mn	87037	150752.5061	173.2050808
UIJ65643.1	rhodanese-like_domain-containing_protein_[Bacillus_cereus]	CPTF_Ni	46887.33333	81211.24356	173.2050808
UIJ65643.1	rhodanese-like_domain-containing_protein_[Bacillus_cereus]	CPTF_U	0	0	0
UIJ65643.1	rhodanese-like_domain-containing_protein_[Bacillus_cereus]	CPTF_metals_mix	269029.1	37415.68808	13.90767321
UIJ65643.1	rhodanese-like_domain-containing_protein_[Bacillus_cereus]	CPTF_zcontrol	30330.13333	52533.33193	173.2050808
UIJ65651.1	leucine--tRNA_ligase_[Bacillus_cereus]	CPTF_Al	1027036.2	183492.4322	17.86620883
UIJ65651.1	leucine--tRNA_ligase_[Bacillus_cereus]	CPTF_Cd	1599289.567	218318.5424	13.65097021
UIJ65651.1	leucine--tRNA_ligase_[Bacillus_cereus]	CPTF_Co	1398511.967	311596.7437	22.28059188
UIJ65651.1	leucine--tRNA_ligase_[Bacillus_cereus]	CPTF_Cu	1292486.333	185292.5911	14.33613542
UIJ65651.1	leucine--tRNA_ligase_[Bacillus_cereus]	CPTF_Fe	1083353.233	174170.5257	16.07698397
UIJ65651.1	leucine--tRNA_ligase_[Bacillus_cereus]	CPTF_Mn	996340.1667	230145.3699	23.09907576
UIJ65651.1	leucine--tRNA_ligase_[Bacillus_cereus]	CPTF_Ni	773823.6667	181588.893	23.46644343
UIJ65651.1	leucine--tRNA_ligase_[Bacillus_cereus]	CPTF_U	879657.8333	554131.0297	62.99392885
UIJ65651.1	leucine--tRNA_ligase_[Bacillus_cereus]	CPTF_metals_mix	1593277.6	170752.9532	10.71708742
UIJ65651.1	leucine--tRNA_ligase_[Bacillus_cereus]	CPTF_zcontrol	616782.84	233633.341	37.87935166
UIJ65657.1	ABC_transporter_ATP-binding_protein_[Bacillus_cereus]	CPTF_Al	65089.26667	57521.71357	88.37357757

UIJ65657.1	ABC_transporter_ATP-binding_protein_[Bacillus_cereus]	CPTF_Cd	88143.46667	49406.84314	56.05275696
UIJ65657.1	ABC_transporter_ATP-binding_protein_[Bacillus_cereus]	CPTF_Co	105392	44272.26034	42.00723048
UIJ65657.1	ABC_transporter_ATP-binding_protein_[Bacillus_cereus]	CPTF_Cu	167348.1	116558.8227	69.6505205
UIJ65657.1	ABC_transporter_ATP-binding_protein_[Bacillus_cereus]	CPTF_Fe	39037.7	41348.15896	105.9185325
UIJ65657.1	ABC_transporter_ATP-binding_protein_[Bacillus_cereus]	CPTF_Mn	42143.8	37097.08519	88.02501244
UIJ65657.1	ABC_transporter_ATP-binding_protein_[Bacillus_cereus]	CPTF_Ni	52703.1	39815.32838	75.54646382
UIJ65657.1	ABC_transporter_ATP-binding_protein_[Bacillus_cereus]	CPTF_U	0	0	0
UIJ65657.1	ABC_transporter_ATP-binding_protein_[Bacillus_cereus]	CPTF_metals_mix	475969.1	59751.9355	12.55374256
UIJ65657.1	ABC_transporter_ATP-binding_protein_[Bacillus_cereus]	CPTF_zcontrol	98606.86667	117765.2647	119.4290709
UIJ65672.1	molybdenum_cofactor_biosynthesis_protein_MoaB_[Bacillus_cereus]	CPTF_Al	144054.5667	72347.89883	50.22256532
UIJ65672.1	molybdenum_cofactor_biosynthesis_protein_MoaB_[Bacillus_cereus]	CPTF_Cd	109159.5667	40370.0311	36.98258644
UIJ65672.1	molybdenum_cofactor_biosynthesis_protein_MoaB_[Bacillus_cereus]	CPTF_Co	144141.6667	31010.33045	21.51378651
UIJ65672.1	molybdenum_cofactor_biosynthesis_protein_MoaB_[Bacillus_cereus]	CPTF_Cu	170224	19258.14602	11.3134141
UIJ65672.1	molybdenum_cofactor_biosynthesis_protein_MoaB_[Bacillus_cereus]	CPTF_Fe	127910.3333	36733.49175	28.7181581
UIJ65672.1	molybdenum_cofactor_biosynthesis_protein_MoaB_[Bacillus_cereus]	CPTF_Mn	120966.6667	105977.569	87.60890242
UIJ65672.1	molybdenum_cofactor_biosynthesis_protein_MoaB_[Bacillus_cereus]	CPTF_Ni	113585.8333	45150.24735	39.74989312
UIJ65672.1	molybdenum_cofactor_biosynthesis_protein_MoaB_[Bacillus_cereus]	CPTF_U	117966.3333	105409.1706	89.35529966
UIJ65672.1	molybdenum_cofactor_biosynthesis_protein_MoaB_[Bacillus_cereus]	CPTF_metals_mix	103593.3	31088.99092	30.01061933
UIJ65672.1	molybdenum_cofactor_biosynthesis_protein_MoaB_[Bacillus_cereus]	CPTF_zcontrol	141516	24134.9907	17.05460209
UIJ65675.1	methionine_adenosyltransferase_[Bacillus_cereus]	CPTF_Al	6014257.6	797071.3258	13.25302936
UIJ65675.1	methionine_adenosyltransferase_[Bacillus_cereus]	CPTF_Cd	7798594.633	215971.7989	2.769368188
UIJ65675.1	methionine_adenosyltransferase_[Bacillus_cereus]	CPTF_Co	6250976.833	672413.4727	10.75693433
UIJ65675.1	methionine_adenosyltransferase_[Bacillus_cereus]	CPTF_Cu	5750815.633	323133.2176	5.618911093
UIJ65675.1	methionine_adenosyltransferase_[Bacillus_cereus]	CPTF_Fe	6677554.833	1041324.37	15.5943964
UIJ65675.1	methionine_adenosyltransferase_[Bacillus_cereus]	CPTF_Mn	7038053.467	2316924.091	32.91995581
UIJ65675.1	methionine_adenosyltransferase_[Bacillus_cereus]	CPTF_Ni	3867378.267	505975.879	13.08317532
UIJ65675.1	methionine_adenosyltransferase_[Bacillus_cereus]	CPTF_U	4451556.933	705100.0543	15.83940327
UIJ65675.1	methionine_adenosyltransferase_[Bacillus_cereus]	CPTF_metals_mix	11498491	619930.557	5.391407942
UIJ65675.1	methionine_adenosyltransferase_[Bacillus_cereus]	CPTF_zcontrol	5711958.367	679046.1904	11.88815021
UIJ65676.1	phosphoenolpyruvate_carboxykinase_(ATP)_[Bacillus_cereus]	CPTF_Al	747222.9333	208795.9818	27.94293007
UIJ65676.1	phosphoenolpyruvate_carboxykinase_(ATP)_[Bacillus_cereus]	CPTF_Cd	1440472.633	457199.1122	31.73952088
UIJ65676.1	phosphoenolpyruvate_carboxykinase_(ATP)_[Bacillus_cereus]	CPTF_Co	1136217.733	203781.6675	17.93508951
UIJ65676.1	phosphoenolpyruvate_carboxykinase_(ATP)_[Bacillus_cereus]	CPTF_Cu	1283366.067	241585.3919	18.82435559
UIJ65676.1	phosphoenolpyruvate_carboxykinase_(ATP)_[Bacillus_cereus]	CPTF_Fe	1116353.667	185437.0836	16.61096202
UIJ65676.1	phosphoenolpyruvate_carboxykinase_(ATP)_[Bacillus_cereus]	CPTF_Mn	803810.9333	248708.3343	30.94114847
UIJ65676.1	phosphoenolpyruvate_carboxykinase_(ATP)_[Bacillus_cereus]	CPTF_Ni	356211.6667	310153.7118	87.07005998
UIJ65676.1	phosphoenolpyruvate_carboxykinase_(ATP)_[Bacillus_cereus]	CPTF_U	408046.4667	134713.1478	33.01416845
UIJ65676.1	phosphoenolpyruvate_carboxykinase_(ATP)_[Bacillus_cereus]	CPTF_metals_mix	2610053.5	201975.1038	7.738351104
UIJ65676.1	phosphoenolpyruvate_carboxykinase_(ATP)_[Bacillus_cereus]	CPTF_zcontrol	775725.3667	71029.78944	9.156563972
UIJ65699.1	antimutator_8-oxo-(dGTP/GTP)ase_[Bacillus_cereus]	CPTF_Al	336997.6667	91738.31666	27.2222409
UIJ65699.1	antimutator_8-oxo-(dGTP/GTP)ase_[Bacillus_cereus]	CPTF_Cd	197790.5	179605.2081	90.80578093
UIJ65699.1	antimutator_8-oxo-(dGTP/GTP)ase_[Bacillus_cereus]	CPTF_Co	267883.1333	18133.85399	6.769315323
UIJ65699.1	antimutator_8-oxo-(dGTP/GTP)ase_[Bacillus_cereus]	CPTF_Cu	207132.5	120641.6984	58.24373211
UIJ65699.1	antimutator_8-oxo-(dGTP/GTP)ase_[Bacillus_cereus]	CPTF_Fe	282394	130658.08	46.26800853
UIJ65699.1	antimutator_8-oxo-(dGTP/GTP)ase_[Bacillus_cereus]	CPTF_Mn	294825.6667	255589.0026	86.69157117
UIJ65699.1	antimutator_8-oxo-(dGTP/GTP)ase_[Bacillus_cereus]	CPTF_Ni	197691.3333	185873.8923	94.02227663
UIJ65699.1	antimutator_8-oxo-(dGTP/GTP)ase_[Bacillus_cereus]	CPTF_U	335196.3333	183792.7647	54.83137684
UIJ65699.1	antimutator_8-oxo-(dGTP/GTP)ase_[Bacillus_cereus]	CPTF_metals_mix	709753.8	304201.269	42.86011136
UIJ65699.1	antimutator_8-oxo-(dGTP/GTP)ase_[Bacillus_cereus]	CPTF_zcontrol	518360.3667	170689.5916	32.92875045
UIJ65705.1	S-ribosylhomocysteine_lyase_LuxS_[Bacillus_cereus]	CPTF_Al	428678.6333	72631.57635	16.94312959
UIJ65705.1	S-ribosylhomocysteine_lyase_LuxS_[Bacillus_cereus]	CPTF_Cd	542086.6333	107723.1256	19.87193908
UIJ65705.1	S-ribosylhomocysteine_lyase_LuxS_[Bacillus_cereus]	CPTF_Co	662063.0667	105122.1967	15.87797327
UIJ65705.1	S-ribosylhomocysteine_lyase_LuxS_[Bacillus_cereus]	CPTF_Cu	511118.9	38461.50177	7.524961759
UIJ65705.1	S-ribosylhomocysteine_lyase_LuxS_[Bacillus_cereus]	CPTF_Fe	384146.7	74634.1402	19.4285517
UIJ65705.1	S-ribosylhomocysteine_lyase_LuxS_[Bacillus_cereus]	CPTF_Mn	475981.6667	129758.705	27.26128211
UIJ65705.1	S-ribosylhomocysteine_lyase_LuxS_[Bacillus_cereus]	CPTF_Ni	592958.0667	94055.2684	15.86204383
UIJ65705.1	S-ribosylhomocysteine_lyase_LuxS_[Bacillus_cereus]	CPTF_U	182775.5	99949.53173	54.68431586
UIJ65705.1	S-ribosylhomocysteine_lyase_LuxS_[Bacillus_cereus]	CPTF_metals_mix	2962216.633	948938.4963	32.03474336
UIJ65705.1	S-ribosylhomocysteine_lyase_LuxS_[Bacillus_cereus]	CPTF_zcontrol	406447.4333	22130.7742	5.444929007
UIJ65707.1	carbonic_anhydrase_[Bacillus_cereus]	CPTF_Al	1546066	104468.34	6.757042711
UIJ65707.1	carbonic_anhydrase_[Bacillus_cereus]	CPTF_Cd	1601019.867	136627.7091	8.533792237

UIJ65707.1	carbonic_anhydrase_[Bacillus_cereus]	CPTF_Co	1391177.567	139133.711	10.00114682
UIJ65707.1	carbonic_anhydrase_[Bacillus_cereus]	CPTF_Cu	1679426.3	121037.6098	7.207080763
UIJ65707.1	carbonic_anhydrase_[Bacillus_cereus]	CPTF_Fe	1549080.633	137811.6994	8.896354159
UIJ65707.1	carbonic_anhydrase_[Bacillus_cereus]	CPTF_Mn	1608558.567	115227.4148	7.163395673
UIJ65707.1	carbonic_anhydrase_[Bacillus_cereus]	CPTF_Ni	1512121.4	105229.5989	6.959070807
UIJ65707.1	carbonic_anhydrase_[Bacillus_cereus]	CPTF_U	1513221.867	203188.994	13.42757453
UIJ65707.1	carbonic_anhydrase_[Bacillus_cereus]	CPTF_metals_mix	1715648.333	31385.14761	1.829346201
UIJ65707.1	carbonic_anhydrase_[Bacillus_cereus]	CPTF_zcontrol	1623263.4	93182.49727	5.740442203
UIJ65712.1	YitT_family_protein_[Bacillus_cereus]	CPTF_Al	11616.53333	20120.42594	173.2050808
UIJ65712.1	YitT_family_protein_[Bacillus_cereus]	CPTF_Cd	20084.2	18666.33178	92.94037992
UIJ65712.1	YitT_family_protein_[Bacillus_cereus]	CPTF_Co	38769.16667	4922.902007	12.69798252
UIJ65712.1	YitT_family_protein_[Bacillus_cereus]	CPTF_Cu	26122.96667	25632.22085	98.12140089
UIJ65712.1	YitT_family_protein_[Bacillus_cereus]	CPTF_Fe	0	0	0
UIJ65712.1	YitT_family_protein_[Bacillus_cereus]	CPTF_Mn	0	0	0
UIJ65712.1	YitT_family_protein_[Bacillus_cereus]	CPTF_Ni	8445	14627.16907	173.2050808
UIJ65712.1	YitT_family_protein_[Bacillus_cereus]	CPTF_U	0	0	0
UIJ65712.1	YitT_family_protein_[Bacillus_cereus]	CPTF_metals_mix	36014.86667	21163.33228	58.76276727
UIJ65712.1	YitT_family_protein_[Bacillus_cereus]	CPTF_zcontrol	0	0	0
UIJ65716.1	PspA/IM30_family_protein_[Bacillus_cereus]	CPTF_Al	1330422.5	96898.19667	7.283265029
UIJ65716.1	PspA/IM30_family_protein_[Bacillus_cereus]	CPTF_Cd	1510402	168182.2641	11.13493389
UIJ65716.1	PspA/IM30_family_protein_[Bacillus_cereus]	CPTF_Co	1268927.9	86681.36575	6.831070997
UIJ65716.1	PspA/IM30_family_protein_[Bacillus_cereus]	CPTF_Cu	1762695.2	119045.3176	6.753596287
UIJ65716.1	PspA/IM30_family_protein_[Bacillus_cereus]	CPTF_Fe	831513.3333	721378.313	86.7548702
UIJ65716.1	PspA/IM30_family_protein_[Bacillus_cereus]	CPTF_Mn	1307880	124490.3649	9.518485251
UIJ65716.1	PspA/IM30_family_protein_[Bacillus_cereus]	CPTF_Ni	1091353.967	168266.9853	15.41818607
UIJ65716.1	PspA/IM30_family_protein_[Bacillus_cereus]	CPTF_U	892477.3333	99314.06326	11.12790875
UIJ65716.1	PspA/IM30_family_protein_[Bacillus_cereus]	CPTF_metals_mix	3565373.2	228834.3864	6.418244979
UIJ65716.1	PspA/IM30_family_protein_[Bacillus_cereus]	CPTF_zcontrol	717662.1	621523.5034	86.60391895
UIJ65728.1	hypoxanthine_phosphoribosyltransferase_[Bacillus_cereus]	CPTF_Al	313825.4667	121656.8324	38.76576164
UIJ65728.1	hypoxanthine_phosphoribosyltransferase_[Bacillus_cereus]	CPTF_Cd	307913.0333	61725.38524	20.04636977
UIJ65728.1	hypoxanthine_phosphoribosyltransferase_[Bacillus_cereus]	CPTF_Co	421831.6667	17404.55484	4.125947912
UIJ65728.1	hypoxanthine_phosphoribosyltransferase_[Bacillus_cereus]	CPTF_Cu	551909.6667	162084.0857	29.36786497
UIJ65728.1	hypoxanthine_phosphoribosyltransferase_[Bacillus_cereus]	CPTF_Fe	328897.4	45148.36572	13.72718839
UIJ65728.1	hypoxanthine_phosphoribosyltransferase_[Bacillus_cereus]	CPTF_Mn	198362.1333	21666.23397	10.92256551
UIJ65728.1	hypoxanthine_phosphoribosyltransferase_[Bacillus_cereus]	CPTF_Ni	234209	184643.7833	78.83718527
UIJ65728.1	hypoxanthine_phosphoribosyltransferase_[Bacillus_cereus]	CPTF_U	137371.2667	237934.0134	173.2050808
UIJ65728.1	hypoxanthine_phosphoribosyltransferase_[Bacillus_cereus]	CPTF_metals_mix	492560.1667	49441.73766	10.03770524
UIJ65728.1	hypoxanthine_phosphoribosyltransferase_[Bacillus_cereus]	CPTF_zcontrol	250018.3	10065.77406	4.026014919
UIJ65729.1	YegS/Rv2252/BmrU_family_lipid_kinase_[Bacillus_cereus]	CPTF_Al	0	0	0
UIJ65729.1	YegS/Rv2252/BmrU_family_lipid_kinase_[Bacillus_cereus]	CPTF_Cd	0	0	0
UIJ65729.1	YegS/Rv2252/BmrU_family_lipid_kinase_[Bacillus_cereus]	CPTF_Co	0	0	0
UIJ65729.1	YegS/Rv2252/BmrU_family_lipid_kinase_[Bacillus_cereus]	CPTF_Cu	0	0	0
UIJ65729.1	YegS/Rv2252/BmrU_family_lipid_kinase_[Bacillus_cereus]	CPTF_Fe	0	0	0
UIJ65729.1	YegS/Rv2252/BmrU_family_lipid_kinase_[Bacillus_cereus]	CPTF_Mn	14108.2	24436.1192	173.2050808
UIJ65729.1	YegS/Rv2252/BmrU_family_lipid_kinase_[Bacillus_cereus]	CPTF_Ni	0	0	0
UIJ65729.1	YegS/Rv2252/BmrU_family_lipid_kinase_[Bacillus_cereus]	CPTF_U	0	0	0
UIJ65729.1	YegS/Rv2252/BmrU_family_lipid_kinase_[Bacillus_cereus]	CPTF_metals_mix	183205.4333	59276.34447	32.35512364
UIJ65729.1	YegS/Rv2252/BmrU_family_lipid_kinase_[Bacillus_cereus]	CPTF_zcontrol	0	0	0
UIJ65744.1	response_regulator_transcription_factor_[Bacillus_cereus]	CPTF_Al	220993.8	19238.94307	8.705648336
UIJ65744.1	response_regulator_transcription_factor_[Bacillus_cereus]	CPTF_Cd	277267.2667	55253.01048	19.92770771
UIJ65744.1	response_regulator_transcription_factor_[Bacillus_cereus]	CPTF_Co	229651.9	26924.38823	11.72399977
UIJ65744.1	response_regulator_transcription_factor_[Bacillus_cereus]	CPTF_Cu	190746	18142.61858	9.511401855
UIJ65744.1	response_regulator_transcription_factor_[Bacillus_cereus]	CPTF_Fe	197874	36539.19779	18.46589132
UIJ65744.1	response_regulator_transcription_factor_[Bacillus_cereus]	CPTF_Mn	260739.6667	70431.93377	27.0123586
UIJ65744.1	response_regulator_transcription_factor_[Bacillus_cereus]	CPTF_Ni	244224	68424.63247	28.01716149
UIJ65744.1	response_regulator_transcription_factor_[Bacillus_cereus]	CPTF_U	232616.2333	34459.61072	14.81393204
UIJ65744.1	response_regulator_transcription_factor_[Bacillus_cereus]	CPTF_metals_mix	396235.3	221421.781	55.88138688
UIJ65744.1	response_regulator_transcription_factor_[Bacillus_cereus]	CPTF_zcontrol	84387.8	54061.42693	64.06308369
UIJ65748.1	ABC_transporter_ATP-binding_protein_[Bacillus_cereus]	CPTF_Al	0	0	0
UIJ65748.1	ABC_transporter_ATP-binding_protein_[Bacillus_cereus]	CPTF_Cd	0	0	0
UIJ65748.1	ABC_transporter_ATP-binding_protein_[Bacillus_cereus]	CPTF_Co	29491.63333	51081.00733	173.2050808

UIJ65748.1	ABC_transporter_ATP-binding_protein_[Bacillus_cereus]	CPTF_Cu	0	0	0
UIJ65748.1	ABC_transporter_ATP-binding_protein_[Bacillus_cereus]	CPTF_Fe	47069.33333	81526.47681	173.2050808
UIJ65748.1	ABC_transporter_ATP-binding_protein_[Bacillus_cereus]	CPTF_Mn	0	0	0
UIJ65748.1	ABC_transporter_ATP-binding_protein_[Bacillus_cereus]	CPTF_Ni	0	0	0
UIJ65748.1	ABC_transporter_ATP-binding_protein_[Bacillus_cereus]	CPTF_U	57033	98784.05371	173.2050808
UIJ65748.1	ABC_transporter_ATP-binding_protein_[Bacillus_cereus]	CPTF_metals_mix	14148.56667	24506.03632	173.2050808
UIJ65748.1	ABC_transporter_ATP-binding_protein_[Bacillus_cereus]	CPTF_zcontrol	0	0	0
UIJ65763.1	1,4-dihydroxy-2-naphthoyl-CoA_synthase_[Bacillus_cereus]	CPTF_Al	3836326.233	281524.5931	7.338390325
UIJ65763.1	1,4-dihydroxy-2-naphthoyl-CoA_synthase_[Bacillus_cereus]	CPTF_Cd	4128573.033	342981.5497	8.307508355
UIJ65763.1	1,4-dihydroxy-2-naphthoyl-CoA_synthase_[Bacillus_cereus]	CPTF_Co	3716986.333	263552.6263	7.090492207
UIJ65763.1	1,4-dihydroxy-2-naphthoyl-CoA_synthase_[Bacillus_cereus]	CPTF_Cu	4164463	402339.3945	9.661255114
UIJ65763.1	1,4-dihydroxy-2-naphthoyl-CoA_synthase_[Bacillus_cereus]	CPTF_Fe	4119217.467	209623.4972	5.088915527
UIJ65763.1	1,4-dihydroxy-2-naphthoyl-CoA_synthase_[Bacillus_cereus]	CPTF_Mn	3920565.233	788689.6973	20.11673446
UIJ65763.1	1,4-dihydroxy-2-naphthoyl-CoA_synthase_[Bacillus_cereus]	CPTF_Ni	4352458	612579.2985	14.07432992
UIJ65763.1	1,4-dihydroxy-2-naphthoyl-CoA_synthase_[Bacillus_cereus]	CPTF_U	5032066.667	228669.4054	4.544244354
UIJ65763.1	1,4-dihydroxy-2-naphthoyl-CoA_synthase_[Bacillus_cereus]	CPTF_metals_mix	4191234.933	634287.8753	15.13367505
UIJ65763.1	1,4-dihydroxy-2-naphthoyl-CoA_synthase_[Bacillus_cereus]	CPTF_zcontrol	4455204.667	411451.3136	9.235295444
UIJ65769.1	cold-shock_protein_CspD_[Bacillus_cereus]	CPTF_Al	2237795	471215.4878	21.05713382
UIJ65769.1	cold-shock_protein_CspD_[Bacillus_cereus]	CPTF_Cd	3938351.667	295996.2955	7.515740608
UIJ65769.1	cold-shock_protein_CspD_[Bacillus_cereus]	CPTF_Co	3191880.733	495082.7054	15.51068936
UIJ65769.1	cold-shock_protein_CspD_[Bacillus_cereus]	CPTF_Cu	3287072.733	173432.2227	5.276190603
UIJ65769.1	cold-shock_protein_CspD_[Bacillus_cereus]	CPTF_Fe	3012218.2	692864.1652	23.00179201
UIJ65769.1	cold-shock_protein_CspD_[Bacillus_cereus]	CPTF_Mn	2713657.933	1503302.652	55.39764733
UIJ65769.1	cold-shock_protein_CspD_[Bacillus_cereus]	CPTF_Ni	2905153.667	271473.5475	9.344550361
UIJ65769.1	cold-shock_protein_CspD_[Bacillus_cereus]	CPTF_U	3501001.667	501032.8909	14.31112974
UIJ65769.1	cold-shock_protein_CspD_[Bacillus_cereus]	CPTF_metals_mix	8086359.667	514344.6393	6.360645093
UIJ65769.1	cold-shock_protein_CspD_[Bacillus_cereus]	CPTF_zcontrol	1660551.7	58818.29324	3.542093464
UIJ65770.1	TIGR00266_family_protein_[Bacillus_cereus]	CPTF_Al	984540	57718.02807	5.862436068
UIJ65770.1	TIGR00266_family_protein_[Bacillus_cereus]	CPTF_Cd	1005486.667	59778.84687	5.945265
UIJ65770.1	TIGR00266_family_protein_[Bacillus_cereus]	CPTF_Co	957887	125108.5223	13.0608853
UIJ65770.1	TIGR00266_family_protein_[Bacillus_cereus]	CPTF_Cu	814097.3333	9208.809605	1.131168133
UIJ65770.1	TIGR00266_family_protein_[Bacillus_cereus]	CPTF_Fe	1012992.333	117771.313	11.62608138
UIJ65770.1	TIGR00266_family_protein_[Bacillus_cereus]	CPTF_Mn	920797	204589.6112	22.21875301
UIJ65770.1	TIGR00266_family_protein_[Bacillus_cereus]	CPTF_Ni	554676.3333	118425.223	21.35032917
UIJ65770.1	TIGR00266_family_protein_[Bacillus_cereus]	CPTF_U	780062	96664.77155	12.39193443
UIJ65770.1	TIGR00266_family_protein_[Bacillus_cereus]	CPTF_metals_mix	1001441.567	27127.9818	2.70889313
UIJ65770.1	TIGR00266_family_protein_[Bacillus_cereus]	CPTF_zcontrol	1014055	172649.0511	17.02561016
UIJ65773.1	glycogen_phosphorylase_[Bacillus_cereus]	CPTF_Al	1919681.967	339459.9335	17.68313394
UIJ65773.1	glycogen_phosphorylase_[Bacillus_cereus]	CPTF_Cd	1554204.333	468093.9474	30.11791547
UIJ65773.1	glycogen_phosphorylase_[Bacillus_cereus]	CPTF_Co	1827515.4	199019.837	10.89018659
UIJ65773.1	glycogen_phosphorylase_[Bacillus_cereus]	CPTF_Cu	886169.8	306707.4585	34.61046162
UIJ65773.1	glycogen_phosphorylase_[Bacillus_cereus]	CPTF_Fe	1437957.1	689546.6329	47.95321313
UIJ65773.1	glycogen_phosphorylase_[Bacillus_cereus]	CPTF_Mn	1564786.667	631498.5444	40.35684595
UIJ65773.1	glycogen_phosphorylase_[Bacillus_cereus]	CPTF_Ni	1936291.5	248025.2311	12.80929194
UIJ65773.1	glycogen_phosphorylase_[Bacillus_cereus]	CPTF_U	1169693.8	700876.8269	59.91968384
UIJ65773.1	glycogen_phosphorylase_[Bacillus_cereus]	CPTF_metals_mix	768608.1	137464.9993	17.88492722
UIJ65773.1	glycogen_phosphorylase_[Bacillus_cereus]	CPTF_zcontrol	2163059.767	155248.5933	7.177267855
UIJ65774.1	glucose-1-phosphate_adenylyltransferase_subunit_GlgD_[Bacillus_cereus]	CPTF_Al	5692313.733	190393.8165	3.344752686
UIJ65774.1	glucose-1-phosphate_adenylyltransferase_subunit_GlgD_[Bacillus_cereus]	CPTF_Cd	5114320.967	1864733.03	36.46100905
UIJ65774.1	glucose-1-phosphate_adenylyltransferase_subunit_GlgD_[Bacillus_cereus]	CPTF_Co	6071127.267	520964.4974	8.581017569
UIJ65774.1	glucose-1-phosphate_adenylyltransferase_subunit_GlgD_[Bacillus_cereus]	CPTF_Cu	4609707.633	493463.3219	10.70487244
UIJ65774.1	glucose-1-phosphate_adenylyltransferase_subunit_GlgD_[Bacillus_cereus]	CPTF_Fe	4944108.967	1893167.256	38.29137401
UIJ65774.1	glucose-1-phosphate_adenylyltransferase_subunit_GlgD_[Bacillus_cereus]	CPTF_Mn	5641758.933	851280.3696	15.08891783
UIJ65774.1	glucose-1-phosphate_adenylyltransferase_subunit_GlgD_[Bacillus_cereus]	CPTF_Ni	5647592.8	633791.1403	11.22232361
UIJ65774.1	glucose-1-phosphate_adenylyltransferase_subunit_GlgD_[Bacillus_cereus]	CPTF_U	5705800.833	517304.9316	9.066298434
UIJ65774.1	glucose-1-phosphate_adenylyltransferase_subunit_GlgD_[Bacillus_cereus]	CPTF_metals_mix	2914083.767	458384.589	15.72997298
UIJ65774.1	glucose-1-phosphate_adenylyltransferase_subunit_GlgD_[Bacillus_cereus]	CPTF_zcontrol	5399932.633	847109.8201	15.6874146
UIJ65775.1	glucose-1-phosphate_adenylyltransferase_[Bacillus_cereus]	CPTF_Al	6828725.333	201878.8088	2.956317598
UIJ65775.1	glucose-1-phosphate_adenylyltransferase_[Bacillus_cereus]	CPTF_Cd	7513536.167	368923.6682	4.910120349
UIJ65775.1	glucose-1-phosphate_adenylyltransferase_[Bacillus_cereus]	CPTF_Co	6397839.667	765927.1136	11.97165221
UIJ65775.1	glucose-1-phosphate_adenylyltransferase_[Bacillus_cereus]	CPTF_Cu	4960447.367	59885.32114	1.207256457

UIJ65775.1	glucose-1-phosphate_adenylyltransferase_[Bacillus_cereus]	CPTF_Fe	6374882.2	187284.4996	2.93785036
UIJ65775.1	glucose-1-phosphate_adenylyltransferase_[Bacillus_cereus]	CPTF_Mn	7372247.967	714476.4859	9.691433185
UIJ65775.1	glucose-1-phosphate_adenylyltransferase_[Bacillus_cereus]	CPTF_Ni	5145902.533	231098.3099	4.490918908
UIJ65775.1	glucose-1-phosphate_adenylyltransferase_[Bacillus_cereus]	CPTF_U	5706485.333	418854.6965	7.339976747
UIJ65775.1	glucose-1-phosphate_adenylyltransferase_[Bacillus_cereus]	CPTF_metals_mix	5070889.6	489303.2613	9.649258806
UIJ65775.1	glucose-1-phosphate_adenylyltransferase_[Bacillus_cereus]	CPTF_zcontrol	6576873.9	663336.0249	10.08588632
UIJ65776.1	1,4-alpha-glucan_branching_protein_GlgB_[Bacillus_cereus]	CPTF_Al	1094035	142047.8672	12.98385035
UIJ65776.1	1,4-alpha-glucan_branching_protein_GlgB_[Bacillus_cereus]	CPTF_Cd	1047301.067	46510.19345	4.440957327
UIJ65776.1	1,4-alpha-glucan_branching_protein_GlgB_[Bacillus_cereus]	CPTF_Co	1078362.167	106869.5282	9.910355863
UIJ65776.1	1,4-alpha-glucan_branching_protein_GlgB_[Bacillus_cereus]	CPTF_Cu	813431.9	22401.34836	2.753930398
UIJ65776.1	1,4-alpha-glucan_branching_protein_GlgB_[Bacillus_cereus]	CPTF_Fe	1086330.4	45840.51093	4.219757721
UIJ65776.1	1,4-alpha-glucan_branching_protein_GlgB_[Bacillus_cereus]	CPTF_Mn	906270.2	370554.0929	40.88781612
UIJ65776.1	1,4-alpha-glucan_branching_protein_GlgB_[Bacillus_cereus]	CPTF_Ni	1034298.467	279962.1463	27.06782958
UIJ65776.1	1,4-alpha-glucan_branching_protein_GlgB_[Bacillus_cereus]	CPTF_U	994952.3333	119330.8842	11.99362826
UIJ65776.1	1,4-alpha-glucan_branching_protein_GlgB_[Bacillus_cereus]	CPTF_metals_mix	430857.1333	110843.2601	25.72622141
UIJ65776.1	1,4-alpha-glucan_branching_protein_GlgB_[Bacillus_cereus]	CPTF_zcontrol	1007879.233	232711.9674	23.08927099
UIJ65777.1	CalY_family_protein_[Bacillus_cereus]	CPTF_Al	0	0	0
UIJ65777.1	CalY_family_protein_[Bacillus_cereus]	CPTF_Cd	15606.56667	27031.3664	173.2050808
UIJ65777.1	CalY_family_protein_[Bacillus_cereus]	CPTF_Co	80092.26667	110707.2415	138.2246328
UIJ65777.1	CalY_family_protein_[Bacillus_cereus]	CPTF_Cu	0	0	0
UIJ65777.1	CalY_family_protein_[Bacillus_cereus]	CPTF_Fe	69919.2	87913.51344	125.7358686
UIJ65777.1	CalY_family_protein_[Bacillus_cereus]	CPTF_Mn	30202.5	26539.82322	87.87293509
UIJ65777.1	CalY_family_protein_[Bacillus_cereus]	CPTF_Ni	0	0	0
UIJ65777.1	CalY_family_protein_[Bacillus_cereus]	CPTF_U	0	0	0
UIJ65777.1	CalY_family_protein_[Bacillus_cereus]	CPTF_metals_mix	0	0	0
UIJ65777.1	CalY_family_protein_[Bacillus_cereus]	CPTF_zcontrol	0	0	0
UIJ65779.1	L-lactate_dehydrogenase_[Bacillus_cereus]	CPTF_Al	3342654	379727.3523	11.36005558
UIJ65779.1	L-lactate_dehydrogenase_[Bacillus_cereus]	CPTF_Cd	4727064.6	399383.6529	8.448872327
UIJ65779.1	L-lactate_dehydrogenase_[Bacillus_cereus]	CPTF_Co	3615560.667	632455.1755	17.49258922
UIJ65779.1	L-lactate_dehydrogenase_[Bacillus_cereus]	CPTF_Cu	3368806.767	647366.3749	19.2164888
UIJ65779.1	L-lactate_dehydrogenase_[Bacillus_cereus]	CPTF_Fe	4353760	747488.983	17.16881461
UIJ65779.1	L-lactate_dehydrogenase_[Bacillus_cereus]	CPTF_Mn	3337236.333	424127.5572	12.70894581
UIJ65779.1	L-lactate_dehydrogenase_[Bacillus_cereus]	CPTF_Ni	2386022.7	411486.4008	17.24570352
UIJ65779.1	L-lactate_dehydrogenase_[Bacillus_cereus]	CPTF_U	2620610.333	779213.9139	29.73406248
UIJ65779.1	L-lactate_dehydrogenase_[Bacillus_cereus]	CPTF_metals_mix	4587443.267	347497.109	7.57496254
UIJ65779.1	L-lactate_dehydrogenase_[Bacillus_cereus]	CPTF_zcontrol	2322801	62847.98122	2.705698044
UIJ65783.1	glucose-6-phosphate_isomerase_[Bacillus_cereus]	CPTF_Al	33590478.63	684707.3029	2.038396982
UIJ65783.1	glucose-6-phosphate_isomerase_[Bacillus_cereus]	CPTF_Cd	34794131.93	337551.6436	0.970139575
UIJ65783.1	glucose-6-phosphate_isomerase_[Bacillus_cereus]	CPTF_Co	34672155.47	332951.8571	0.960286007
UIJ65783.1	glucose-6-phosphate_isomerase_[Bacillus_cereus]	CPTF_Cu	34088236.27	423907.4359	1.243559311
UIJ65783.1	glucose-6-phosphate_isomerase_[Bacillus_cereus]	CPTF_Fe	33604816.53	1066741.498	3.174370844
UIJ65783.1	glucose-6-phosphate_isomerase_[Bacillus_cereus]	CPTF_Mn	34013773.37	1417004.691	4.165973225
UIJ65783.1	glucose-6-phosphate_isomerase_[Bacillus_cereus]	CPTF_Ni	31507382.6	741987.0044	2.354962371
UIJ65783.1	glucose-6-phosphate_isomerase_[Bacillus_cereus]	CPTF_U	32711085.8	1222559.086	3.737445748
UIJ65783.1	glucose-6-phosphate_isomerase_[Bacillus_cereus]	CPTF_metals_mix	36540613.5	1253344.808	3.430004829
UIJ65783.1	glucose-6-phosphate_isomerase_[Bacillus_cereus]	CPTF_zcontrol	34015856.67	717525.93	2.109386622
UIJ65786.1	aminotransferase_[Bacillus_cereus]	CPTF_Al	0	0	0
UIJ65786.1	aminotransferase_[Bacillus_cereus]	CPTF_Cd	0	0	0
UIJ65786.1	aminotransferase_[Bacillus_cereus]	CPTF_Co	0	0	0
UIJ65786.1	aminotransferase_[Bacillus_cereus]	CPTF_Cu	0	0	0
UIJ65786.1	aminotransferase_[Bacillus_cereus]	CPTF_Fe	0	0	0
UIJ65786.1	aminotransferase_[Bacillus_cereus]	CPTF_Mn	0	0	0
UIJ65786.1	aminotransferase_[Bacillus_cereus]	CPTF_Ni	0	0	0
UIJ65786.1	aminotransferase_[Bacillus_cereus]	CPTF_U	0	0	0
UIJ65786.1	aminotransferase_[Bacillus_cereus]	CPTF_metals_mix	61819.46667	34842.24138	56.36127787
UIJ65786.1	aminotransferase_[Bacillus_cereus]	CPTF_zcontrol	0	0	0
UIJ65788.1	D-glycerate_dehydrogenase_[Bacillus_cereus]	CPTF_Al	333103	27370.29779	8.216767122
UIJ65788.1	D-glycerate_dehydrogenase_[Bacillus_cereus]	CPTF_Cd	201606.6667	175133.636	86.86897062
UIJ65788.1	D-glycerate_dehydrogenase_[Bacillus_cereus]	CPTF_Co	276785	15006.06451	5.421559878
UIJ65788.1	D-glycerate_dehydrogenase_[Bacillus_cereus]	CPTF_Cu	290857	17608.82367	6.054117202
UIJ65788.1	D-glycerate_dehydrogenase_[Bacillus_cereus]	CPTF_Fe	335043	35160.29532	10.49426352

UIJ65788.1	D-glycerate_dehydrogenase_[Bacillus_cereus]	CPTF_Mn	191839.6667	173095.9264	90.22947624
UIJ65788.1	D-glycerate_dehydrogenase_[Bacillus_cereus]	CPTF_Ni	327235	34759.31333	10.62212579
UIJ65788.1	D-glycerate_dehydrogenase_[Bacillus_cereus]	CPTF_U	335527.3333	14946.035	4.454491041
UIJ65788.1	D-glycerate_dehydrogenase_[Bacillus_cereus]	CPTF_metals_mix	247830.6667	37630.75094	15.1840575
UIJ65788.1	D-glycerate_dehydrogenase_[Bacillus_cereus]	CPTF_zcontrol	314181.3333	23514.61253	7.484407899
UIJ65791.1	pyridoxal_phosphate-dependent_aminotransferase_[Bacillus_cereus]	CPTF_Al	0	0	0
UIJ65791.1	pyridoxal_phosphate-dependent_aminotransferase_[Bacillus_cereus]	CPTF_Cd	41738.66667	72293.49131	173.2050808
UIJ65791.1	pyridoxal_phosphate-dependent_aminotransferase_[Bacillus_cereus]	CPTF_Co	73140.53333	68744.46664	93.98956161
UIJ65791.1	pyridoxal_phosphate-dependent_aminotransferase_[Bacillus_cereus]	CPTF_Cu	0	0	0
UIJ65791.1	pyridoxal_phosphate-dependent_aminotransferase_[Bacillus_cereus]	CPTF_Fe	0	0	0
UIJ65791.1	pyridoxal_phosphate-dependent_aminotransferase_[Bacillus_cereus]	CPTF_Mn	0	0	0
UIJ65791.1	pyridoxal_phosphate-dependent_aminotransferase_[Bacillus_cereus]	CPTF_Ni	0	0	0
UIJ65791.1	pyridoxal_phosphate-dependent_aminotransferase_[Bacillus_cereus]	CPTF_U	0	0	0
UIJ65791.1	pyridoxal_phosphate-dependent_aminotransferase_[Bacillus_cereus]	CPTF_metals_mix	70522.43333	72098.03221	102.2341811
UIJ65791.1	pyridoxal_phosphate-dependent_aminotransferase_[Bacillus_cereus]	CPTF_zcontrol	118286.3333	204877.9392	173.2050808
UIJ65794.1	kinase-associated_protein_B_[Bacillus_cereus]	CPTF_Al	134989.1667	63828.58951	47.28423109
UIJ65794.1	kinase-associated_protein_B_[Bacillus_cereus]	CPTF_Cd	132364.8333	30740.7435	23.22425279
UIJ65794.1	kinase-associated_protein_B_[Bacillus_cereus]	CPTF_Co	113034.2	21023.25945	18.5990253
UIJ65794.1	kinase-associated_protein_B_[Bacillus_cereus]	CPTF_Cu	193118.3	95525.56724	49.46479295
UIJ65794.1	kinase-associated_protein_B_[Bacillus_cereus]	CPTF_Fe	88776.53333	10400.98781	11.71592021
UIJ65794.1	kinase-associated_protein_B_[Bacillus_cereus]	CPTF_Mn	67779.46667	22038.17933	32.51453635
UIJ65794.1	kinase-associated_protein_B_[Bacillus_cereus]	CPTF_Ni	138909.9333	45127.20063	32.48666208
UIJ65794.1	kinase-associated_protein_B_[Bacillus_cereus]	CPTF_U	59291.1	51351.60776	86.60930183
UIJ65794.1	kinase-associated_protein_B_[Bacillus_cereus]	CPTF_metals_mix	80661.06667	49504.20606	61.3731111
UIJ65794.1	kinase-associated_protein_B_[Bacillus_cereus]	CPTF_zcontrol	96382.33333	54241.06604	56.27697957
UIJ65798.1	DNA_alkylation_repair_protein_[Bacillus_cereus]	CPTF_Al	205673.3333	196536.0849	95.55739759
UIJ65798.1	DNA_alkylation_repair_protein_[Bacillus_cereus]	CPTF_Cd	110954.1333	121430.6597	109.4422137
UIJ65798.1	DNA_alkylation_repair_protein_[Bacillus_cereus]	CPTF_Co	244523	235604.0014	96.35249095
UIJ65798.1	DNA_alkylation_repair_protein_[Bacillus_cereus]	CPTF_Cu	493998	285005.217	57.69359735
UIJ65798.1	DNA_alkylation_repair_protein_[Bacillus_cereus]	CPTF_Fe	75967	131578.7037	173.2050808
UIJ65798.1	DNA_alkylation_repair_protein_[Bacillus_cereus]	CPTF_Mn	190502.3333	235356.0713	123.5449809
UIJ65798.1	DNA_alkylation_repair_protein_[Bacillus_cereus]	CPTF_Ni	67723	117299.6768	173.2050808
UIJ65798.1	DNA_alkylation_repair_protein_[Bacillus_cereus]	CPTF_U	251266.8667	277769.919	110.5477704
UIJ65798.1	DNA_alkylation_repair_protein_[Bacillus_cereus]	CPTF_metals_mix	254283.3333	93733.71376	36.86191797
UIJ65798.1	DNA_alkylation_repair_protein_[Bacillus_cereus]	CPTF_zcontrol	412672	262033.3789	63.49676713
UIJ65799.1	glycine--tRNA_ligase_[Bacillus_cereus]	CPTF_Al	5545101.233	298454.2897	5.382305518
UIJ65799.1	glycine--tRNA_ligase_[Bacillus_cereus]	CPTF_Cd	4711303.2	202680.952	4.302014609
UIJ65799.1	glycine--tRNA_ligase_[Bacillus_cereus]	CPTF_Co	6106091.133	588387.5811	9.636075981
UIJ65799.1	glycine--tRNA_ligase_[Bacillus_cereus]	CPTF_Cu	4889258.167	726650.3098	14.86217919
UIJ65799.1	glycine--tRNA_ligase_[Bacillus_cereus]	CPTF_Fe	5561986.467	886428.9259	15.93727225
UIJ65799.1	glycine--tRNA_ligase_[Bacillus_cereus]	CPTF_Mn	5007652.1	486381.3021	9.712761438
UIJ65799.1	glycine--tRNA_ligase_[Bacillus_cereus]	CPTF_Ni	5428976	300859.3748	5.541733373
UIJ65799.1	glycine--tRNA_ligase_[Bacillus_cereus]	CPTF_U	4485071.033	1138549.077	25.38530759
UIJ65799.1	glycine--tRNA_ligase_[Bacillus_cereus]	CPTF_metals_mix	4117342.133	105388.7683	2.559631065
UIJ65799.1	glycine--tRNA_ligase_[Bacillus_cereus]	CPTF_zcontrol	4344798.167	373786.6425	8.603084151
UIJ65800.1	hotdog_fold_thioesterase_[Bacillus_cereus]	CPTF_Al	52655.33333	91201.71262	173.2050808
UIJ65800.1	hotdog_fold_thioesterase_[Bacillus_cereus]	CPTF_Cd	170411.3333	55991.42199	32.85663042
UIJ65800.1	hotdog_fold_thioesterase_[Bacillus_cereus]	CPTF_Co	122454.6333	63791.4173	52.09391884
UIJ65800.1	hotdog_fold_thioesterase_[Bacillus_cereus]	CPTF_Cu	389672.3333	47981.36072	12.31325825
UIJ65800.1	hotdog_fold_thioesterase_[Bacillus_cereus]	CPTF_Fe	88991	77965.30019	87.61032035
UIJ65800.1	hotdog_fold_thioesterase_[Bacillus_cereus]	CPTF_Mn	130595.2333	47833.55593	36.62733678
UIJ65800.1	hotdog_fold_thioesterase_[Bacillus_cereus]	CPTF_Ni	192918.6667	167091.3797	86.61234423
UIJ65800.1	hotdog_fold_thioesterase_[Bacillus_cereus]	CPTF_U	176750.3333	57970.59872	32.7980138
UIJ65800.1	hotdog_fold_thioesterase_[Bacillus_cereus]	CPTF_metals_mix	713424.9	25997.50633	3.644042467
UIJ65800.1	hotdog_fold_thioesterase_[Bacillus_cereus]	CPTF_zcontrol	237055	71497.31531	30.16064428
UIJ65803.1	phospho-sugar_mutase_[Bacillus_cereus]	CPTF_Al	5173791.167	467055.0273	9.02732662
UIJ65803.1	phospho-sugar_mutase_[Bacillus_cereus]	CPTF_Cd	5098952.633	1118527.728	21.93642123
UIJ65803.1	phospho-sugar_mutase_[Bacillus_cereus]	CPTF_Co	5445048.067	324248.9319	5.954932408
UIJ65803.1	phospho-sugar_mutase_[Bacillus_cereus]	CPTF_Cu	6040567.567	150393.7154	2.48972822
UIJ65803.1	phospho-sugar_mutase_[Bacillus_cereus]	CPTF_Fe	4953257.967	793628.2243	16.02234791
UIJ65803.1	phospho-sugar_mutase_[Bacillus_cereus]	CPTF_Mn	5295248.5	586501.167	11.07598948

UIJ65803.1	phospho-sugar_mutase_[Bacillus_cereus]	CPTF_Ni	4785199.2	53850.40344	1.125353432
UIJ65803.1	phospho-sugar_mutase_[Bacillus_cereus]	CPTF_U	5378440.067	729723.7098	13.56757165
UIJ65803.1	phospho-sugar_mutase_[Bacillus_cereus]	CPTF_metals_mix	6464716.833	102077.1524	1.578988764
UIJ65803.1	phospho-sugar_mutase_[Bacillus_cereus]	CPTF_zcontrol	4728471.767	582856.8625	12.32653786
UIJ65809.1	cytosol_aminopeptidase_[Bacillus_cereus]	CPTF_Al	6716226.533	897905.2554	13.36919252
UIJ65809.1	cytosol_aminopeptidase_[Bacillus_cereus]	CPTF_Cd	8403714.833	582023.0049	6.925782424
UIJ65809.1	cytosol_aminopeptidase_[Bacillus_cereus]	CPTF_Co	6254440.733	551237.9057	8.813544315
UIJ65809.1	cytosol_aminopeptidase_[Bacillus_cereus]	CPTF_Cu	6599838.833	409663.0722	6.207167819
UIJ65809.1	cytosol_aminopeptidase_[Bacillus_cereus]	CPTF_Fe	6363566.333	378227.4775	5.943640055
UIJ65809.1	cytosol_aminopeptidase_[Bacillus_cereus]	CPTF_Mn	6687566.633	1944157.303	29.07122141
UIJ65809.1	cytosol_aminopeptidase_[Bacillus_cereus]	CPTF_Ni	5897138.167	452481.1144	7.672893217
UIJ65809.1	cytosol_aminopeptidase_[Bacillus_cereus]	CPTF_U	5649515	441707.8163	7.818508603
UIJ65809.1	cytosol_aminopeptidase_[Bacillus_cereus]	CPTF_metals_mix	10095359.3	595004.5435	5.893842168
UIJ65809.1	cytosol_aminopeptidase_[Bacillus_cereus]	CPTF_zcontrol	6054345.667	194123.9462	3.206357168
UIJ65814.1	NAD(P)/FAD-dependent_oxidoreductase_[Bacillus_cereus]	CPTF_Al	1881183.667	74127.41721	3.940466767
UIJ65814.1	NAD(P)/FAD-dependent_oxidoreductase_[Bacillus_cereus]	CPTF_Cd	1863630.667	154306.2143	8.279870957
UIJ65814.1	NAD(P)/FAD-dependent_oxidoreductase_[Bacillus_cereus]	CPTF_Co	1960315	126886.6715	6.472769503
UIJ65814.1	NAD(P)/FAD-dependent_oxidoreductase_[Bacillus_cereus]	CPTF_Cu	1810042.833	72980.3225	4.031966601
UIJ65814.1	NAD(P)/FAD-dependent_oxidoreductase_[Bacillus_cereus]	CPTF_Fe	2044256.667	250139.5233	12.23620925
UIJ65814.1	NAD(P)/FAD-dependent_oxidoreductase_[Bacillus_cereus]	CPTF_Mn	1869123	109217.7371	5.843261099
UIJ65814.1	NAD(P)/FAD-dependent_oxidoreductase_[Bacillus_cereus]	CPTF_Ni	1867913.333	158679.0804	8.494991582
UIJ65814.1	NAD(P)/FAD-dependent_oxidoreductase_[Bacillus_cereus]	CPTF_U	2173987.2	475638.0207	21.87860263
UIJ65814.1	NAD(P)/FAD-dependent_oxidoreductase_[Bacillus_cereus]	CPTF_metals_mix	1891606.7	78422.79439	4.145829806
UIJ65814.1	NAD(P)/FAD-dependent_oxidoreductase_[Bacillus_cereus]	CPTF_zcontrol	1844051.233	97793.76066	5.303201934
UIJ65816.1	GyrI-like_domain-containing_protein_[Bacillus_cereus]	CPTF_Al	423294	71209.1607	16.82262463
UIJ65816.1	GyrI-like_domain-containing_protein_[Bacillus_cereus]	CPTF_Cd	387256.6667	36230.6414	9.355717931
UIJ65816.1	GyrI-like_domain-containing_protein_[Bacillus_cereus]	CPTF_Co	348531	24928.15621	7.152349779
UIJ65816.1	GyrI-like_domain-containing_protein_[Bacillus_cereus]	CPTF_Cu	425522.3333	41808.07626	9.825119149
UIJ65816.1	GyrI-like_domain-containing_protein_[Bacillus_cereus]	CPTF_Fe	332141.3333	46956.75556	14.13758266
UIJ65816.1	GyrI-like_domain-containing_protein_[Bacillus_cereus]	CPTF_Mn	366486.3333	120135.8352	32.78044072
UIJ65816.1	GyrI-like_domain-containing_protein_[Bacillus_cereus]	CPTF_Ni	440619.6667	31991.5744	7.260587036
UIJ65816.1	GyrI-like_domain-containing_protein_[Bacillus_cereus]	CPTF_U	405076.6667	217998.8962	53.81670042
UIJ65816.1	GyrI-like_domain-containing_protein_[Bacillus_cereus]	CPTF_metals_mix	378026.2667	60685.46475	16.05324024
UIJ65816.1	GyrI-like_domain-containing_protein_[Bacillus_cereus]	CPTF_zcontrol	464816.6667	24103.20233	5.185528845
UIJ65823.1	iron-sulfur_cluster_assembly_accessory_protein_[Bacillus_cereus]	CPTF_Al	65774.53333	57868.30399	87.9798017
UIJ65823.1	iron-sulfur_cluster_assembly_accessory_protein_[Bacillus_cereus]	CPTF_Cd	110511	13322.26606	12.05514932
UIJ65823.1	iron-sulfur_cluster_assembly_accessory_protein_[Bacillus_cereus]	CPTF_Co	65652.16667	36191.40119	55.12598141
UIJ65823.1	iron-sulfur_cluster_assembly_accessory_protein_[Bacillus_cereus]	CPTF_Cu	81844.2	21961.99709	26.8339077
UIJ65823.1	iron-sulfur_cluster_assembly_accessory_protein_[Bacillus_cereus]	CPTF_Fe	94960.56667	29480.98191	31.04549914
UIJ65823.1	iron-sulfur_cluster_assembly_accessory_protein_[Bacillus_cereus]	CPTF_Mn	15482.23333	26816.01475	173.2050808
UIJ65823.1	iron-sulfur_cluster_assembly_accessory_protein_[Bacillus_cereus]	CPTF_Ni	13802.36667	23906.40033	173.2050808
UIJ65823.1	iron-sulfur_cluster_assembly_accessory_protein_[Bacillus_cereus]	CPTF_U	111122.8333	122218.2106	109.9847861
UIJ65823.1	iron-sulfur_cluster_assembly_accessory_protein_[Bacillus_cereus]	CPTF_metals_mix	63443.56667	57502.5748	90.63578519
UIJ65823.1	iron-sulfur_cluster_assembly_accessory_protein_[Bacillus_cereus]	CPTF_zcontrol	53286.66667	5810.997148	10.90516167
UIJ65824.1	diaminopimelate_epimerase_[Bacillus_cereus]	CPTF_Al	576408.5667	26535.49068	4.603590614
UIJ65824.1	diaminopimelate_epimerase_[Bacillus_cereus]	CPTF_Cd	615144.3667	45513.53477	7.398837937
UIJ65824.1	diaminopimelate_epimerase_[Bacillus_cereus]	CPTF_Co	566226.9667	36437.29266	6.435103731
UIJ65824.1	diaminopimelate_epimerase_[Bacillus_cereus]	CPTF_Cu	564838.4667	93200.89759	16.50045156
UIJ65824.1	diaminopimelate_epimerase_[Bacillus_cereus]	CPTF_Fe	551864.8333	67294.84229	12.19408055
UIJ65824.1	diaminopimelate_epimerase_[Bacillus_cereus]	CPTF_Mn	507331.6667	79212.97258	15.61364641
UIJ65824.1	diaminopimelate_epimerase_[Bacillus_cereus]	CPTF_Ni	476971.8	54941.84683	11.51888787
UIJ65824.1	diaminopimelate_epimerase_[Bacillus_cereus]	CPTF_U	464385.4333	154565.324	33.28384417
UIJ65824.1	diaminopimelate_epimerase_[Bacillus_cereus]	CPTF_metals_mix	696846.9333	47027.90673	6.748670975
UIJ65824.1	diaminopimelate_epimerase_[Bacillus_cereus]	CPTF_zcontrol	481634.6667	46932.26488	9.744370188
UIJ65826.1	NAD(P)/FAD-dependent_oxidoreductase_[Bacillus_cereus]	CPTF_Al	2314460.333	46933.79966	2.027850682
UIJ65826.1	NAD(P)/FAD-dependent_oxidoreductase_[Bacillus_cereus]	CPTF_Cd	2682091	101042.558	3.767305358
UIJ65826.1	NAD(P)/FAD-dependent_oxidoreductase_[Bacillus_cereus]	CPTF_Co	1990890.333	414937.7694	20.84181948
UIJ65826.1	NAD(P)/FAD-dependent_oxidoreductase_[Bacillus_cereus]	CPTF_Cu	2202792.167	431660.8802	19.59607841
UIJ65826.1	NAD(P)/FAD-dependent_oxidoreductase_[Bacillus_cereus]	CPTF_Fe	2640222.1	399000.1775	15.1123717
UIJ65826.1	NAD(P)/FAD-dependent_oxidoreductase_[Bacillus_cereus]	CPTF_Mn	2227323.633	489018.4921	21.95543049
UIJ65826.1	NAD(P)/FAD-dependent_oxidoreductase_[Bacillus_cereus]	CPTF_Ni	2398010.733	424651.376	17.70848521

UIJ65826.1	NAD(P)/FAD-dependent_oxidoreductase_[Bacillus_cereus]	CPTF_U	2579780.967	357740.6469	13.86709382
UIJ65826.1	NAD(P)/FAD-dependent_oxidoreductase_[Bacillus_cereus]	CPTF_metals_mix	1237809.367	135193.6855	10.92201183
UIJ65826.1	NAD(P)/FAD-dependent_oxidoreductase_[Bacillus_cereus]	CPTF_zcontrol	2497185.167	129568.8312	5.188595262
UIJ65830.1	alkylphosphonate_utilization_operon_protein_PhnA_[Bacillus_cereus]	CPTF_Al	337609.6667	19542.29419	5.788428509
UIJ65830.1	alkylphosphonate_utilization_operon_protein_PhnA_[Bacillus_cereus]	CPTF_Cd	328549	26211.61996	7.977994138
UIJ65830.1	alkylphosphonate_utilization_operon_protein_PhnA_[Bacillus_cereus]	CPTF_Co	372826	25289.11108	6.783086769
UIJ65830.1	alkylphosphonate_utilization_operon_protein_PhnA_[Bacillus_cereus]	CPTF_Cu	331606.6667	43014.44783	12.97152686
UIJ65830.1	alkylphosphonate_utilization_operon_protein_PhnA_[Bacillus_cereus]	CPTF_Fe	365435.3333	31569.695	8.638927909
UIJ65830.1	alkylphosphonate_utilization_operon_protein_PhnA_[Bacillus_cereus]	CPTF_Mn	312370.6667	64210.78277	20.55595791
UIJ65830.1	alkylphosphonate_utilization_operon_protein_PhnA_[Bacillus_cereus]	CPTF_Ni	343298.6667	40479.75021	11.7914091
UIJ65830.1	alkylphosphonate_utilization_operon_protein_PhnA_[Bacillus_cereus]	CPTF_U	331963.3333	71388.03251	21.50479446
UIJ65830.1	alkylphosphonate_utilization_operon_protein_PhnA_[Bacillus_cereus]	CPTF_metals_mix	327616.6667	38039.21936	11.61089262
UIJ65830.1	alkylphosphonate_utilization_operon_protein_PhnA_[Bacillus_cereus]	CPTF_zcontrol	314376	52827.65794	16.80397293
UIJ65837.1	nucleotidyltransferase_domain-containing_protein_[Bacillus_cereus]	CPTF_Al	18659.4	20500.7499	109.868216
UIJ65837.1	nucleotidyltransferase_domain-containing_protein_[Bacillus_cereus]	CPTF_Cd	44083.26667	4345.626565	9.857768931
UIJ65837.1	nucleotidyltransferase_domain-containing_protein_[Bacillus_cereus]	CPTF_Co	0	0	0
UIJ65837.1	nucleotidyltransferase_domain-containing_protein_[Bacillus_cereus]	CPTF_Cu	0	0	0
UIJ65837.1	nucleotidyltransferase_domain-containing_protein_[Bacillus_cereus]	CPTF_Fe	22684.36667	20726.45255	91.36888348
UIJ65837.1	nucleotidyltransferase_domain-containing_protein_[Bacillus_cereus]	CPTF_Mn	10003.86667	17327.20534	173.2050808
UIJ65837.1	nucleotidyltransferase_domain-containing_protein_[Bacillus_cereus]	CPTF_Ni	0	0	0
UIJ65837.1	nucleotidyltransferase_domain-containing_protein_[Bacillus_cereus]	CPTF_U	6601.8	11434.65302	173.2050808
UIJ65837.1	nucleotidyltransferase_domain-containing_protein_[Bacillus_cereus]	CPTF_metals_mix	7203.466667	12476.77026	173.2050808
UIJ65837.1	nucleotidyltransferase_domain-containing_protein_[Bacillus_cereus]	CPTF_zcontrol	0	0	0
UIJ65839.1	NifU_family_protein_[Bacillus_cereus]	CPTF_Al	2509446.2	36195.71552	1.442378622
UIJ65839.1	NifU_family_protein_[Bacillus_cereus]	CPTF_Cd	3215852.967	135482.6154	4.212960505
UIJ65839.1	NifU_family_protein_[Bacillus_cereus]	CPTF_Co	2659530.033	257349.4864	9.676502358
UIJ65839.1	NifU_family_protein_[Bacillus_cereus]	CPTF_Cu	3401513.867	287762.5118	8.459836505
UIJ65839.1	NifU_family_protein_[Bacillus_cereus]	CPTF_Fe	3052752.533	340560.9824	11.15586601
UIJ65839.1	NifU_family_protein_[Bacillus_cereus]	CPTF_Mn	2331514.733	265085.2556	11.36965818
UIJ65839.1	NifU_family_protein_[Bacillus_cereus]	CPTF_Ni	3330001.333	26287.41681	0.7894116
UIJ65839.1	NifU_family_protein_[Bacillus_cereus]	CPTF_U	2585479.4	475563.9672	18.39364751
UIJ65839.1	NifU_family_protein_[Bacillus_cereus]	CPTF_metals_mix	3684704.7	109192.1953	2.963390669
UIJ65839.1	NifU_family_protein_[Bacillus_cereus]	CPTF_zcontrol	2587051.367	308504.6003	11.92495071
UIJ65842.1	phosphatidylglycerophosphatase_A_[Bacillus_cereus]	CPTF_Al	263527.3333	293676.3096	111.44055
UIJ65842.1	phosphatidylglycerophosphatase_A_[Bacillus_cereus]	CPTF_Cd	698712.5333	106777.2052	15.28199369
UIJ65842.1	phosphatidylglycerophosphatase_A_[Bacillus_cereus]	CPTF_Co	510608.3	54702.89021	10.71327869
UIJ65842.1	phosphatidylglycerophosphatase_A_[Bacillus_cereus]	CPTF_Cu	360426.1333	242306.5357	67.22779324
UIJ65842.1	phosphatidylglycerophosphatase_A_[Bacillus_cereus]	CPTF_Fe	459420.6333	289994.0061	63.12167653
UIJ65842.1	phosphatidylglycerophosphatase_A_[Bacillus_cereus]	CPTF_Mn	578554.1	90880.73437	15.70825172
UIJ65842.1	phosphatidylglycerophosphatase_A_[Bacillus_cereus]	CPTF_Ni	253106.9	280626.2567	110.8726221
UIJ65842.1	phosphatidylglycerophosphatase_A_[Bacillus_cereus]	CPTF_U	301198.5	198240.5938	65.817258
UIJ65842.1	phosphatidylglycerophosphatase_A_[Bacillus_cereus]	CPTF_metals_mix	785324.2667	52746.20503	6.716487351
UIJ65842.1	phosphatidylglycerophosphatase_A_[Bacillus_cereus]	CPTF_zcontrol	345647.8333	232990.3855	67.40686994
UIJ65843.1	GNAT_family_N-acetyltransferase_[Bacillus_cereus]	CPTF_Al	59293.4	21316.41195	35.95073304
UIJ65843.1	GNAT_family_N-acetyltransferase_[Bacillus_cereus]	CPTF_Cd	83027.8	11362.01587	13.68459223
UIJ65843.1	GNAT_family_N-acetyltransferase_[Bacillus_cereus]	CPTF_Co	57274.46667	51985.7753	90.76605742
UIJ65843.1	GNAT_family_N-acetyltransferase_[Bacillus_cereus]	CPTF_Cu	101165.1	41373.41785	40.89692775
UIJ65843.1	GNAT_family_N-acetyltransferase_[Bacillus_cereus]	CPTF_Fe	73040.96667	36815.43981	50.40382335
UIJ65843.1	GNAT_family_N-acetyltransferase_[Bacillus_cereus]	CPTF_Mn	49972.66667	47658.91439	95.36996436
UIJ65843.1	GNAT_family_N-acetyltransferase_[Bacillus_cereus]	CPTF_Ni	89279.23333	12462.10488	13.95857067
UIJ65843.1	GNAT_family_N-acetyltransferase_[Bacillus_cereus]	CPTF_U	95853	89832.68956	93.71922585
UIJ65843.1	GNAT_family_N-acetyltransferase_[Bacillus_cereus]	CPTF_metals_mix	37777.96667	9472.930349	25.07527849
UIJ65843.1	GNAT_family_N-acetyltransferase_[Bacillus_cereus]	CPTF_zcontrol	53482.16667	51667.81947	96.60756601
UIJ65844.1	NAD(P)H-dependent_oxidoreductase_[Bacillus_cereus]	CPTF_Al	69309.86667	90585.47724	130.6963663
UIJ65844.1	NAD(P)H-dependent_oxidoreductase_[Bacillus_cereus]	CPTF_Cd	94740	84556.6704	89.25128816
UIJ65844.1	NAD(P)H-dependent_oxidoreductase_[Bacillus_cereus]	CPTF_Co	136715	8931.444284	6.532892721
UIJ65844.1	NAD(P)H-dependent_oxidoreductase_[Bacillus_cereus]	CPTF_Cu	79292.63333	97740.05341	123.264986
UIJ65844.1	NAD(P)H-dependent_oxidoreductase_[Bacillus_cereus]	CPTF_Fe	50314.66667	87147.55903	173.2050808
UIJ65844.1	NAD(P)H-dependent_oxidoreductase_[Bacillus_cereus]	CPTF_Mn	36397.66667	63042.60794	173.2050808
UIJ65844.1	NAD(P)H-dependent_oxidoreductase_[Bacillus_cereus]	CPTF_Ni	0	0	0
UIJ65844.1	NAD(P)H-dependent_oxidoreductase_[Bacillus_cereus]	CPTF_U	0	0	0

UIJ65844.1	NAD(P)H-dependent_oxidoreductase_[Bacillus_cereus]	CPTF_metals_mix	116251.3667	104903.4824	90.23849389
UIJ65844.1	NAD(P)H-dependent_oxidoreductase_[Bacillus_cereus]	CPTF_zcontrol	0	0	0
UIJ65846.1	TIGR01457_family_HAD-type_hydrolase_[Bacillus_cereus]	CPTF_Al	997512.2	88407.66301	8.862815213
UIJ65846.1	TIGR01457_family_HAD-type_hydrolase_[Bacillus_cereus]	CPTF_Cd	1060083.333	81379.21052	7.676680499
UIJ65846.1	TIGR01457_family_HAD-type_hydrolase_[Bacillus_cereus]	CPTF_Co	1075641.333	67371.48929	6.263378619
UIJ65846.1	TIGR01457_family_HAD-type_hydrolase_[Bacillus_cereus]	CPTF_Cu	1079562.767	112179.8014	10.39122549
UIJ65846.1	TIGR01457_family_HAD-type_hydrolase_[Bacillus_cereus]	CPTF_Fe	986670.3	29412.1784	2.98095305
UIJ65846.1	TIGR01457_family_HAD-type_hydrolase_[Bacillus_cereus]	CPTF_Mn	1043786.667	107439.7681	10.29326888
UIJ65846.1	TIGR01457_family_HAD-type_hydrolase_[Bacillus_cereus]	CPTF_Ni	749207	188589.1891	25.17184024
UIJ65846.1	TIGR01457_family_HAD-type_hydrolase_[Bacillus_cereus]	CPTF_U	863385	248053.1422	28.73030481
UIJ65846.1	TIGR01457_family_HAD-type_hydrolase_[Bacillus_cereus]	CPTF_metals_mix	1055378.567	44307.08628	4.198217367
UIJ65846.1	TIGR01457_family_HAD-type_hydrolase_[Bacillus_cereus]	CPTF_zcontrol	938783.4333	39472.13886	4.204605392
UIJ65847.1	transcriptional_regulator_[Bacillus_cereus]	CPTF_Al	0	0	0
UIJ65847.1	transcriptional_regulator_[Bacillus_cereus]	CPTF_Cd	0	0	0
UIJ65847.1	transcriptional_regulator_[Bacillus_cereus]	CPTF_Co	14142.1	24494.83573	173.2050808
UIJ65847.1	transcriptional_regulator_[Bacillus_cereus]	CPTF_Cu	0	0	0
UIJ65847.1	transcriptional_regulator_[Bacillus_cereus]	CPTF_Fe	0	0	0
UIJ65847.1	transcriptional_regulator_[Bacillus_cereus]	CPTF_Mn	0	0	0
UIJ65847.1	transcriptional_regulator_[Bacillus_cereus]	CPTF_Ni	0	0	0
UIJ65847.1	transcriptional_regulator_[Bacillus_cereus]	CPTF_U	0	0	0
UIJ65847.1	transcriptional_regulator_[Bacillus_cereus]	CPTF_metals_mix	0	0	0
UIJ65847.1	transcriptional_regulator_[Bacillus_cereus]	CPTF_zcontrol	0	0	0
UIJ65848.1	DUF86_domain-containing_protein_[Bacillus_cereus]	CPTF_Al	131650.7	87156.60296	66.20291648
UIJ65848.1	DUF86_domain-containing_protein_[Bacillus_cereus]	CPTF_Cd	66231.26667	41254.76592	62.28895807
UIJ65848.1	DUF86_domain-containing_protein_[Bacillus_cereus]	CPTF_Co	136573.9667	101486.4407	74.30877432
UIJ65848.1	DUF86_domain-containing_protein_[Bacillus_cereus]	CPTF_Cu	141831.7667	82491.97985	58.16185033
UIJ65848.1	DUF86_domain-containing_protein_[Bacillus_cereus]	CPTF_Fe	46786.83333	10570.67605	22.59327101
UIJ65848.1	DUF86_domain-containing_protein_[Bacillus_cereus]	CPTF_Mn	38073.13333	3101.506496	8.146181373
UIJ65848.1	DUF86_domain-containing_protein_[Bacillus_cereus]	CPTF_Ni	137218.8	104150.0375	75.90070563
UIJ65848.1	DUF86_domain-containing_protein_[Bacillus_cereus]	CPTF_U	7759.8	13440.36786	173.2050808
UIJ65848.1	DUF86_domain-containing_protein_[Bacillus_cereus]	CPTF_metals_mix	224078.8667	16710.87483	7.457586287
UIJ65848.1	DUF86_domain-containing_protein_[Bacillus_cereus]	CPTF_zcontrol	109334.4333	95590.32588	87.42929649
UIJ65849.1	class_II_fructose-bisphosphatase_[Bacillus_cereus]	CPTF_Al	860975.3	444061.2862	51.57654188
UIJ65849.1	class_II_fructose-bisphosphatase_[Bacillus_cereus]	CPTF_Cd	1073538.833	51772.90506	4.822639243
UIJ65849.1	class_II_fructose-bisphosphatase_[Bacillus_cereus]	CPTF_Co	1198108.833	139377.7471	11.63314577
UIJ65849.1	class_II_fructose-bisphosphatase_[Bacillus_cereus]	CPTF_Cu	1005770.7	81907.141	8.14371914
UIJ65849.1	class_II_fructose-bisphosphatase_[Bacillus_cereus]	CPTF_Fe	994289.3	138945.4892	13.97435225
UIJ65849.1	class_II_fructose-bisphosphatase_[Bacillus_cereus]	CPTF_Mn	1068251.8	84861.16741	7.943929269
UIJ65849.1	class_II_fructose-bisphosphatase_[Bacillus_cereus]	CPTF_Ni	1215377.967	173324.128	14.26092399
UIJ65849.1	class_II_fructose-bisphosphatase_[Bacillus_cereus]	CPTF_U	1025891.567	126644.5875	12.34483171
UIJ65849.1	class_II_fructose-bisphosphatase_[Bacillus_cereus]	CPTF_metals_mix	598152.7	172691.066	28.87073251
UIJ65849.1	class_II_fructose-bisphosphatase_[Bacillus_cereus]	CPTF_zcontrol	1097228.333	61378.54874	5.593963159
UIJ65850.1	DUF3055_domain-containing_protein_[Bacillus_cereus]	CPTF_Al	53836.33333	93247.26463	173.2050808
UIJ65850.1	DUF3055_domain-containing_protein_[Bacillus_cereus]	CPTF_Cd	81528.5	79349.77476	97.32765201
UIJ65850.1	DUF3055_domain-containing_protein_[Bacillus_cereus]	CPTF_Co	76640	132744.3739	173.2050808
UIJ65850.1	DUF3055_domain-containing_protein_[Bacillus_cereus]	CPTF_Cu	114255	106847.5385	93.51672883
UIJ65850.1	DUF3055_domain-containing_protein_[Bacillus_cereus]	CPTF_Fe	147744	127999.0739	86.63571711
UIJ65850.1	DUF3055_domain-containing_protein_[Bacillus_cereus]	CPTF_Mn	0	0	0
UIJ65850.1	DUF3055_domain-containing_protein_[Bacillus_cereus]	CPTF_Ni	142167.6667	123410.4053	86.80623955
UIJ65850.1	DUF3055_domain-containing_protein_[Bacillus_cereus]	CPTF_U	0	0	0
UIJ65850.1	DUF3055_domain-containing_protein_[Bacillus_cereus]	CPTF_metals_mix	65363	113212.0369	173.2050808
UIJ65850.1	DUF3055_domain-containing_protein_[Bacillus_cereus]	CPTF_zcontrol	64637.66667	111955.7227	173.2050808
UIJ65860.1	lipoyl_synthase_[Bacillus_cereus]	CPTF_Al	9050051.667	5486779.153	60.62704784
UIJ65860.1	lipoyl_synthase_[Bacillus_cereus]	CPTF_Cd	6687250	4646081.765	69.47671712
UIJ65860.1	lipoyl_synthase_[Bacillus_cereus]	CPTF_Co	12213703.33	871064.4265	7.13186167
UIJ65860.1	lipoyl_synthase_[Bacillus_cereus]	CPTF_Cu	10744627	2949272.212	27.44880964
UIJ65860.1	lipoyl_synthase_[Bacillus_cereus]	CPTF_Fe	10804589	1700279.561	15.73664265
UIJ65860.1	lipoyl_synthase_[Bacillus_cereus]	CPTF_Mn	10597803	5796190.049	54.69237396
UIJ65860.1	lipoyl_synthase_[Bacillus_cereus]	CPTF_Ni	5888798.733	3726307.989	63.27789687
UIJ65860.1	lipoyl_synthase_[Bacillus_cereus]	CPTF_U	5610013.667	2279741.703	40.63700801
UIJ65860.1	lipoyl_synthase_[Bacillus_cereus]	CPTF_metals_mix	2061532.667	134097.4503	6.504745351

UIJ65860.1	lipoyl_synthase_[Bacillus_cereus]	CPTF_zcontrol	14305297.33	3474568.808	24.2886864
UIJ65864.1	Fe-S_cluster_assembly_protein_SufB_[Bacillus_cereus]	CPTF_Al	11065956.13	5570156.549	50.33597171
UIJ65864.1	Fe-S_cluster_assembly_protein_SufB_[Bacillus_cereus]	CPTF_Cd	10098610.77	3800112.482	37.63005199
UIJ65864.1	Fe-S_cluster_assembly_protein_SufB_[Bacillus_cereus]	CPTF_Co	15580728.83	825295.4456	5.296898845
UIJ65864.1	Fe-S_cluster_assembly_protein_SufB_[Bacillus_cereus]	CPTF_Cu	12997437.77	1071816.424	8.246367039
UIJ65864.1	Fe-S_cluster_assembly_protein_SufB_[Bacillus_cereus]	CPTF_Fe	14132481.03	118584.9439	0.839095016
UIJ65864.1	Fe-S_cluster_assembly_protein_SufB_[Bacillus_cereus]	CPTF_Mn	10834860.57	6191228.255	57.1417437
UIJ65864.1	Fe-S_cluster_assembly_protein_SufB_[Bacillus_cereus]	CPTF_Ni	6551662.033	3217458.254	49.10903885
UIJ65864.1	Fe-S_cluster_assembly_protein_SufB_[Bacillus_cereus]	CPTF_U	10875804.53	5898667.912	54.23661205
UIJ65864.1	Fe-S_cluster_assembly_protein_SufB_[Bacillus_cereus]	CPTF_metals_mix	7776322.3	311181.371	4.001652182
UIJ65864.1	Fe-S_cluster_assembly_protein_SufB_[Bacillus_cereus]	CPTF_zcontrol	14375788.67	2800641.253	19.48165292
UIJ65865.1	iron-sulfur_cluster_assembly_scaffold_protein_SufU_[Bacillus_cereus]	CPTF_Al	184666.6667	181983.6256	98.54708967
UIJ65865.1	iron-sulfur_cluster_assembly_scaffold_protein_SufU_[Bacillus_cereus]	CPTF_Cd	258716	225156.4023	87.02840269
UIJ65865.1	iron-sulfur_cluster_assembly_scaffold_protein_SufU_[Bacillus_cereus]	CPTF_Co	338660.6667	33192.80483	9.801198691
UIJ65865.1	iron-sulfur_cluster_assembly_scaffold_protein_SufU_[Bacillus_cereus]	CPTF_Cu	91805	159010.9244	173.2050808
UIJ65865.1	iron-sulfur_cluster_assembly_scaffold_protein_SufU_[Bacillus_cereus]	CPTF_Fe	0	0	0
UIJ65865.1	iron-sulfur_cluster_assembly_scaffold_protein_SufU_[Bacillus_cereus]	CPTF_Mn	104362.6667	92149.26877	88.29715808
UIJ65865.1	iron-sulfur_cluster_assembly_scaffold_protein_SufU_[Bacillus_cereus]	CPTF_Ni	0	0	0
UIJ65865.1	iron-sulfur_cluster_assembly_scaffold_protein_SufU_[Bacillus_cereus]	CPTF_U	139884.5	167596.1333	119.8103673
UIJ65865.1	iron-sulfur_cluster_assembly_scaffold_protein_SufU_[Bacillus_cereus]	CPTF_metals_mix	364441.6333	56296.67893	15.44737861
UIJ65865.1	iron-sulfur_cluster_assembly_scaffold_protein_SufU_[Bacillus_cereus]	CPTF_zcontrol	74076	128303.3956	173.2050808
UIJ65866.1	cysteine_desulfurase_SufS_[Bacillus_cereus]	CPTF_Al	2210609.733	337157.9704	15.25180882
UIJ65866.1	cysteine_desulfurase_SufS_[Bacillus_cereus]	CPTF_Cd	2643139.367	211740.1842	8.010935286
UIJ65866.1	cysteine_desulfurase_SufS_[Bacillus_cereus]	CPTF_Co	2430488.867	111770.797	4.598696112
UIJ65866.1	cysteine_desulfurase_SufS_[Bacillus_cereus]	CPTF_Cu	2611412.833	200547.1852	7.679643091
UIJ65866.1	cysteine_desulfurase_SufS_[Bacillus_cereus]	CPTF_Fe	2176006.4	241784.0608	11.11136717
UIJ65866.1	cysteine_desulfurase_SufS_[Bacillus_cereus]	CPTF_Mn	2085732.8	180078.113	8.63380549
UIJ65866.1	cysteine_desulfurase_SufS_[Bacillus_cereus]	CPTF_Ni	1633411.267	305869.9246	18.72583658
UIJ65866.1	cysteine_desulfurase_SufS_[Bacillus_cereus]	CPTF_U	1697316.233	266295.4954	15.68920925
UIJ65866.1	cysteine_desulfurase_SufS_[Bacillus_cereus]	CPTF_metals_mix	2921547.567	156276.4912	5.349099669
UIJ65866.1	cysteine_desulfurase_SufS_[Bacillus_cereus]	CPTF_zcontrol	1823760.4	244046.5663	13.38150375
UIJ65867.1	Fe-S_cluster_assembly_protein_SufD_[Bacillus_cereus]	CPTF_Al	5445600.3	603030.4271	11.07371812
UIJ65867.1	Fe-S_cluster_assembly_protein_SufD_[Bacillus_cereus]	CPTF_Cd	6544970.133	564188.186	8.620179687
UIJ65867.1	Fe-S_cluster_assembly_protein_SufD_[Bacillus_cereus]	CPTF_Co	6506596.1	325678.7418	5.005362817
UIJ65867.1	Fe-S_cluster_assembly_protein_SufD_[Bacillus_cereus]	CPTF_Cu	6805684	384800.3908	5.654103112
UIJ65867.1	Fe-S_cluster_assembly_protein_SufD_[Bacillus_cereus]	CPTF_Fe	5463584.467	644627.8573	11.79862526
UIJ65867.1	Fe-S_cluster_assembly_protein_SufD_[Bacillus_cereus]	CPTF_Mn	5221508.8	856643.0314	16.406044
UIJ65867.1	Fe-S_cluster_assembly_protein_SufD_[Bacillus_cereus]	CPTF_Ni	4280535.333	867200.5029	20.25916002
UIJ65867.1	Fe-S_cluster_assembly_protein_SufD_[Bacillus_cereus]	CPTF_U	4874268.567	761712.0761	15.62720777
UIJ65867.1	Fe-S_cluster_assembly_protein_SufD_[Bacillus_cereus]	CPTF_metals_mix	7085443.2	636965.3921	8.989774868
UIJ65867.1	Fe-S_cluster_assembly_protein_SufD_[Bacillus_cereus]	CPTF_zcontrol	5462815.033	482745.6589	8.83693949
UIJ65868.1	Fe-S_cluster_assembly_ATPase_SufC_[Bacillus_cereus]	CPTF_Al	6840228.467	58305.47806	0.852390799
UIJ65868.1	Fe-S_cluster_assembly_ATPase_SufC_[Bacillus_cereus]	CPTF_Cd	7928665.967	147908.2695	1.865487462
UIJ65868.1	Fe-S_cluster_assembly_ATPase_SufC_[Bacillus_cereus]	CPTF_Co	7820849.067	150845.4769	1.928760875
UIJ65868.1	Fe-S_cluster_assembly_ATPase_SufC_[Bacillus_cereus]	CPTF_Cu	8255327.867	188337.5879	2.281406516
UIJ65868.1	Fe-S_cluster_assembly_ATPase_SufC_[Bacillus_cereus]	CPTF_Fe	6731302.967	112968.246	1.678252287
UIJ65868.1	Fe-S_cluster_assembly_ATPase_SufC_[Bacillus_cereus]	CPTF_Mn	6989165.833	281254.0309	4.024143047
UIJ65868.1	Fe-S_cluster_assembly_ATPase_SufC_[Bacillus_cereus]	CPTF_Ni	6714580.133	324067.9214	4.826331877
UIJ65868.1	Fe-S_cluster_assembly_ATPase_SufC_[Bacillus_cereus]	CPTF_U	6423147.8	519592.0631	8.089368006
UIJ65868.1	Fe-S_cluster_assembly_ATPase_SufC_[Bacillus_cereus]	CPTF_metals_mix	7107648.167	134766.0832	1.896071388
UIJ65868.1	Fe-S_cluster_assembly_ATPase_SufC_[Bacillus_cereus]	CPTF_zcontrol	6611706.133	63763.09975	0.964397063
UIJ65870.1	methionine_ABC_transporter_substrate-binding_lipoprotein_MetQ_[Bacillus_cereus]	CPTF_Al	14154.56667	24516.42863	173.2050808
UIJ65870.1	methionine_ABC_transporter_substrate-binding_lipoprotein_MetQ_[Bacillus_cereus]	CPTF_Cd	27035.8	23414.22677	86.60452722
UIJ65870.1	methionine_ABC_transporter_substrate-binding_lipoprotein_MetQ_[Bacillus_cereus]	CPTF_Co	86299.68	117188.0913	135.7920345
UIJ65870.1	methionine_ABC_transporter_substrate-binding_lipoprotein_MetQ_[Bacillus_cereus]	CPTF_Cu	16317.43333	28262.62358	173.2050808
UIJ65870.1	methionine_ABC_transporter_substrate-binding_lipoprotein_MetQ_[Bacillus_cereus]	CPTF_Fe	85763.53333	148546.7972	173.2050808
UIJ65870.1	methionine_ABC_transporter_substrate-binding_lipoprotein_MetQ_[Bacillus_cereus]	CPTF_Mn	183621.9333	318042.5179	173.2050808
UIJ65870.1	methionine_ABC_transporter_substrate-binding_lipoprotein_MetQ_[Bacillus_cereus]	CPTF_Ni	60431.36667	74107.00598	122.6300348
UIJ65870.1	methionine_ABC_transporter_substrate-binding_lipoprotein_MetQ_[Bacillus_cereus]	CPTF_U	84552.26667	78358.01713	92.67405856
UIJ65870.1	methionine_ABC_transporter_substrate-binding_lipoprotein_MetQ_[Bacillus_cereus]	CPTF_metals_mix	210961.3667	151069.998	71.61026704
UIJ65870.1	methionine_ABC_transporter_substrate-binding_lipoprotein_MetQ_[Bacillus_cereus]	CPTF_zcontrol	135225.1333	117315.3745	86.75559907

UIJ65872.1	methionine_ABC_transporter_ATP-binding_protein_[Bacillus_cereus]	CPTF_Al	63254.83333	59528.7184	94.10935934
UIJ65872.1	methionine_ABC_transporter_ATP-binding_protein_[Bacillus_cereus]	CPTF_Cd	101439.2667	111639.7209	110.0557255
UIJ65872.1	methionine_ABC_transporter_ATP-binding_protein_[Bacillus_cereus]	CPTF_Co	92594.2	43507.65231	46.98744879
UIJ65872.1	methionine_ABC_transporter_ATP-binding_protein_[Bacillus_cereus]	CPTF_Cu	122201.8333	53519.26533	43.79579575
UIJ65872.1	methionine_ABC_transporter_ATP-binding_protein_[Bacillus_cereus]	CPTF_Fe	55503.43333	48280.52595	86.98655749
UIJ65872.1	methionine_ABC_transporter_ATP-binding_protein_[Bacillus_cereus]	CPTF_Mn	0	0	0
UIJ65872.1	methionine_ABC_transporter_ATP-binding_protein_[Bacillus_cereus]	CPTF_Ni	17500.73333	30312.1593	173.2050808
UIJ65872.1	methionine_ABC_transporter_ATP-binding_protein_[Bacillus_cereus]	CPTF_U	39878.7	38829.45163	97.36890027
UIJ65872.1	methionine_ABC_transporter_ATP-binding_protein_[Bacillus_cereus]	CPTF_metals_mix	17825.33333	30874.383	173.2050808
UIJ65872.1	methionine_ABC_transporter_ATP-binding_protein_[Bacillus_cereus]	CPTF_zcontrol	0	0	0
UIJ65876.1	glycine_cleavage_system_protein_GcvH_[Bacillus_cereus]	CPTF_Al	1958520.633	24761.39539	1.264290759
UIJ65876.1	glycine_cleavage_system_protein_GcvH_[Bacillus_cereus]	CPTF_Cd	1911637.3	139458.0458	7.295214724
UIJ65876.1	glycine_cleavage_system_protein_GcvH_[Bacillus_cereus]	CPTF_Co	1760157.333	123741.6885	7.030149301
UIJ65876.1	glycine_cleavage_system_protein_GcvH_[Bacillus_cereus]	CPTF_Cu	1701409	33513.82137	1.969768667
UIJ65876.1	glycine_cleavage_system_protein_GcvH_[Bacillus_cereus]	CPTF_Fe	1835497.433	294912.9255	16.06719357
UIJ65876.1	glycine_cleavage_system_protein_GcvH_[Bacillus_cereus]	CPTF_Mn	2042359.333	249870.9543	12.23442664
UIJ65876.1	glycine_cleavage_system_protein_GcvH_[Bacillus_cereus]	CPTF_Ni	1614444.167	173758.1978	10.76272574
UIJ65876.1	glycine_cleavage_system_protein_GcvH_[Bacillus_cereus]	CPTF_U	1505435.133	219916.1476	14.60814503
UIJ65876.1	glycine_cleavage_system_protein_GcvH_[Bacillus_cereus]	CPTF_metals_mix	3214948.767	638599.3276	19.86343715
UIJ65876.1	glycine_cleavage_system_protein_GcvH_[Bacillus_cereus]	CPTF_zcontrol	1717010	138824.0414	8.085220316
UIJ65877.1	arsenate_reductase_family_protein_[Bacillus_cereus]	CPTF_Al	283263	96386.39635	34.02717487
UIJ65877.1	arsenate_reductase_family_protein_[Bacillus_cereus]	CPTF_Cd	292922.3333	91028.02486	31.07582267
UIJ65877.1	arsenate_reductase_family_protein_[Bacillus_cereus]	CPTF_Co	249050.4333	46833.18994	18.80470125
UIJ65877.1	arsenate_reductase_family_protein_[Bacillus_cereus]	CPTF_Cu	260461.4667	35297.39903	13.55186987
UIJ65877.1	arsenate_reductase_family_protein_[Bacillus_cereus]	CPTF_Fe	356664.1	77873.23799	21.83377525
UIJ65877.1	arsenate_reductase_family_protein_[Bacillus_cereus]	CPTF_Mn	276235.1333	101954.2058	36.90848611
UIJ65877.1	arsenate_reductase_family_protein_[Bacillus_cereus]	CPTF_Ni	254032	59319.55479	23.35121355
UIJ65877.1	arsenate_reductase_family_protein_[Bacillus_cereus]	CPTF_U	194170.6667	24585.14865	12.66161829
UIJ65877.1	arsenate_reductase_family_protein_[Bacillus_cereus]	CPTF_metals_mix	638613.6667	55418.23023	8.677896063
UIJ65877.1	arsenate_reductase_family_protein_[Bacillus_cereus]	CPTF_zcontrol	222788.7333	18355.30858	8.23888547
UIJ65882.1	L-lactate_dehydrogenase_[Bacillus_cereus]	CPTF_Al	2211412.767	375046.7744	16.95960067
UIJ65882.1	L-lactate_dehydrogenase_[Bacillus_cereus]	CPTF_Cd	3157922	367807.5915	11.64713984
UIJ65882.1	L-lactate_dehydrogenase_[Bacillus_cereus]	CPTF_Co	2319286.667	361208.6522	15.57412705
UIJ65882.1	L-lactate_dehydrogenase_[Bacillus_cereus]	CPTF_Cu	2326439.333	225088.7429	9.675246618
UIJ65882.1	L-lactate_dehydrogenase_[Bacillus_cereus]	CPTF_Fe	2373092	813839.0215	34.29445725
UIJ65882.1	L-lactate_dehydrogenase_[Bacillus_cereus]	CPTF_Mn	2599023	1183765.078	45.54654106
UIJ65882.1	L-lactate_dehydrogenase_[Bacillus_cereus]	CPTF_Ni	1802202.633	245044.6501	13.59695328
UIJ65882.1	L-lactate_dehydrogenase_[Bacillus_cereus]	CPTF_U	1979758	200292.7401	10.11703148
UIJ65882.1	L-lactate_dehydrogenase_[Bacillus_cereus]	CPTF_metals_mix	7920583.433	300412.3807	3.792806215
UIJ65882.1	L-lactate_dehydrogenase_[Bacillus_cereus]	CPTF_zcontrol	1670981.333	126352.8308	7.561594393
UIJ65888.1	acyl-CoA_dehydrogenase_family_protein_[Bacillus_cereus]	CPTF_Al	11375227.3	520356.0915	4.574467637
UIJ65888.1	acyl-CoA_dehydrogenase_family_protein_[Bacillus_cereus]	CPTF_Cd	12977589.33	310537.9183	2.392878294
UIJ65888.1	acyl-CoA_dehydrogenase_family_protein_[Bacillus_cereus]	CPTF_Co	8960713	1547429.96	17.26904946
UIJ65888.1	acyl-CoA_dehydrogenase_family_protein_[Bacillus_cereus]	CPTF_Cu	10682971.97	837160.4039	7.836399894
UIJ65888.1	acyl-CoA_dehydrogenase_family_protein_[Bacillus_cereus]	CPTF_Fe	11496905	1366591.109	11.88659999
UIJ65888.1	acyl-CoA_dehydrogenase_family_protein_[Bacillus_cereus]	CPTF_Mn	10844719.03	1104186.78	10.18179241
UIJ65888.1	acyl-CoA_dehydrogenase_family_protein_[Bacillus_cereus]	CPTF_Ni	8196722.367	920841.0048	11.23425881
UIJ65888.1	acyl-CoA_dehydrogenase_family_protein_[Bacillus_cereus]	CPTF_U	8762683.2	1084984.083	12.38187046
UIJ65888.1	acyl-CoA_dehydrogenase_family_protein_[Bacillus_cereus]	CPTF_metals_mix	14266088.33	1264374.546	8.862797683
UIJ65888.1	acyl-CoA_dehydrogenase_family_protein_[Bacillus_cereus]	CPTF_zcontrol	10026575.6	496440.856	4.951250315
UIJ65889.1	acetyl-CoA_C-acetyltransferase_[Bacillus_cereus]	CPTF_Al	933646.5333	140103.922	15.00609888
UIJ65889.1	acetyl-CoA_C-acetyltransferase_[Bacillus_cereus]	CPTF_Cd	1118130.567	135677.2274	12.13429195
UIJ65889.1	acetyl-CoA_C-acetyltransferase_[Bacillus_cereus]	CPTF_Co	668106.2	144718.7807	21.66104441
UIJ65889.1	acetyl-CoA_C-acetyltransferase_[Bacillus_cereus]	CPTF_Cu	1067235.467	275667.3365	25.83003893
UIJ65889.1	acetyl-CoA_C-acetyltransferase_[Bacillus_cereus]	CPTF_Fe	760495.9	291649.2158	38.34987353
UIJ65889.1	acetyl-CoA_C-acetyltransferase_[Bacillus_cereus]	CPTF_Mn	930919.3667	190720.6399	20.48734259
UIJ65889.1	acetyl-CoA_C-acetyltransferase_[Bacillus_cereus]	CPTF_Ni	846156.8	397146.5595	46.93533864
UIJ65889.1	acetyl-CoA_C-acetyltransferase_[Bacillus_cereus]	CPTF_U	594107.3	437087.4912	73.57046298
UIJ65889.1	acetyl-CoA_C-acetyltransferase_[Bacillus_cereus]	CPTF_metals_mix	2793133.667	72213.71028	2.585401162
UIJ65889.1	acetyl-CoA_C-acetyltransferase_[Bacillus_cereus]	CPTF_zcontrol	763983.6333	55611.02414	7.279085796
UIJ65890.1	3-hydroxyacyl-CoA_dehydrogenase/enoyl-CoA_hydratase_family_protein_[Bacillus_cereus]	CPTF_Al	6152736.067	817461.0251	13.28613833

UIJ65890.1	3-hydroxyacyl-CoA_dehydrogenase/enoyl-CoA_hydratase_family_protein_[Bacillus_cereus]	CPTF_Cd	5980231.167	281759.9483	4.711522689
UIJ65890.1	3-hydroxyacyl-CoA_dehydrogenase/enoyl-CoA_hydratase_family_protein_[Bacillus_cereus]	CPTF_Co	4926086.267	855658.3337	17.36994213
UIJ65890.1	3-hydroxyacyl-CoA_dehydrogenase/enoyl-CoA_hydratase_family_protein_[Bacillus_cereus]	CPTF_Cu	6645817.3	80979.86238	1.218508706
UIJ65890.1	3-hydroxyacyl-CoA_dehydrogenase/enoyl-CoA_hydratase_family_protein_[Bacillus_cereus]	CPTF_Fe	5460130.767	793424.1567	14.53122994
UIJ65890.1	3-hydroxyacyl-CoA_dehydrogenase/enoyl-CoA_hydratase_family_protein_[Bacillus_cereus]	CPTF_Mn	5985538.8	452534.4654	7.560463319
UIJ65890.1	3-hydroxyacyl-CoA_dehydrogenase/enoyl-CoA_hydratase_family_protein_[Bacillus_cereus]	CPTF_Ni	5385905.967	1106350.25	20.54158126
UIJ65890.1	3-hydroxyacyl-CoA_dehydrogenase/enoyl-CoA_hydratase_family_protein_[Bacillus_cereus]	CPTF_U	4804635.633	227613.8409	4.737379861
UIJ65890.1	3-hydroxyacyl-CoA_dehydrogenase/enoyl-CoA_hydratase_family_protein_[Bacillus_cereus]	CPTF_metals_mix	6028620.267	323283.7441	5.362483118
UIJ65890.1	3-hydroxyacyl-CoA_dehydrogenase/enoyl-CoA_hydratase_family_protein_[Bacillus_cereus]	CPTF_zcontrol	5877658.833	301294.2307	5.126092535
UIJ65893.1	proline_dehydrogenase_[Bacillus_cereus]	CPTF_Al	441673.3333	132138.7896	29.91776493
UIJ65893.1	proline_dehydrogenase_[Bacillus_cereus]	CPTF_Cd	403474.7	34693.48299	8.598676197
UIJ65893.1	proline_dehydrogenase_[Bacillus_cereus]	CPTF_Co	961252.3667	72901.71537	7.584034942
UIJ65893.1	proline_dehydrogenase_[Bacillus_cereus]	CPTF_Cu	551919.8667	408672.1247	74.04555432
UIJ65893.1	proline_dehydrogenase_[Bacillus_cereus]	CPTF_Fe	492840.8	296423.626	60.14591852
UIJ65893.1	proline_dehydrogenase_[Bacillus_cereus]	CPTF_Mn	507838.3333	392163.6765	77.2221494
UIJ65893.1	proline_dehydrogenase_[Bacillus_cereus]	CPTF_Ni	733052.6667	356833.0273	48.67767945
UIJ65893.1	proline_dehydrogenase_[Bacillus_cereus]	CPTF_U	402699.3	297259.7083	73.81679291
UIJ65893.1	proline_dehydrogenase_[Bacillus_cereus]	CPTF_metals_mix	856010	103653.6178	12.10892604
UIJ65893.1	proline_dehydrogenase_[Bacillus_cereus]	CPTF_zcontrol	319542.2	279662.084	87.51960898
UIJ65894.1	YusU_family_protein_[Bacillus_cereus]	CPTF_Al	414877	83962.71734	20.23797833
UIJ65894.1	YusU_family_protein_[Bacillus_cereus]	CPTF_Cd	325673.6667	99510.98001	30.55542716
UIJ65894.1	YusU_family_protein_[Bacillus_cereus]	CPTF_Co	312593.3667	165031.3924	52.79427206
UIJ65894.1	YusU_family_protein_[Bacillus_cereus]	CPTF_Cu	257647.5667	227299.8022	88.22121053
UIJ65894.1	YusU_family_protein_[Bacillus_cereus]	CPTF_Fe	303666.3333	128034.7868	42.16298377
UIJ65894.1	YusU_family_protein_[Bacillus_cereus]	CPTF_Mn	377139.6667	83455.9007	22.12864572
UIJ65894.1	YusU_family_protein_[Bacillus_cereus]	CPTF_Ni	353959.6	161759.9738	45.70012336
UIJ65894.1	YusU_family_protein_[Bacillus_cereus]	CPTF_U	262334.6	141448.0606	53.91894956
UIJ65894.1	YusU_family_protein_[Bacillus_cereus]	CPTF_metals_mix	215497.6667	59232.09328	27.48618776
UIJ65894.1	YusU_family_protein_[Bacillus_cereus]	CPTF_zcontrol	389209.6667	117212.3079	30.11546679
UIJ65895.1	MTH1187_family_thiamine-binding_protein_[Bacillus_cereus]	CPTF_Al	54495.93333	60648.25455	111.2895052
UIJ65895.1	MTH1187_family_thiamine-binding_protein_[Bacillus_cereus]	CPTF_Cd	89265.56667	25206.52435	28.23767919
UIJ65895.1	MTH1187_family_thiamine-binding_protein_[Bacillus_cereus]	CPTF_Co	71003.9	12758.57913	17.96884274
UIJ65895.1	MTH1187_family_thiamine-binding_protein_[Bacillus_cereus]	CPTF_Cu	93972.96667	32285.98531	34.35667347
UIJ65895.1	MTH1187_family_thiamine-binding_protein_[Bacillus_cereus]	CPTF_Fe	49970.9	48191.90345	96.43993493
UIJ65895.1	MTH1187_family_thiamine-binding_protein_[Bacillus_cereus]	CPTF_Mn	33363.66667	57787.56579	173.2050808
UIJ65895.1	MTH1187_family_thiamine-binding_protein_[Bacillus_cereus]	CPTF_Ni	0	0	0
UIJ65895.1	MTH1187_family_thiamine-binding_protein_[Bacillus_cereus]	CPTF_U	0	0	0
UIJ65895.1	MTH1187_family_thiamine-binding_protein_[Bacillus_cereus]	CPTF_metals_mix	166994	53158.60859	31.83264584
UIJ65895.1	MTH1187_family_thiamine-binding_protein_[Bacillus_cereus]	CPTF_zcontrol	45982.73333	46823.03348	101.8274254
UIJ65926.1	neutral_protease_NprB_[Bacillus_cereus]	CPTF_Al	4333240.9	494373.7553	11.40886848
UIJ65926.1	neutral_protease_NprB_[Bacillus_cereus]	CPTF_Cd	5338455.267	787744.3369	14.75603517
UIJ65926.1	neutral_protease_NprB_[Bacillus_cereus]	CPTF_Co	4336420.033	42585.65571	0.982046374
UIJ65926.1	neutral_protease_NprB_[Bacillus_cereus]	CPTF_Cu	2850018.333	274461.2647	9.630157866
UIJ65926.1	neutral_protease_NprB_[Bacillus_cereus]	CPTF_Fe	4173619.333	1104332.319	26.45982373
UIJ65926.1	neutral_protease_NprB_[Bacillus_cereus]	CPTF_Mn	4406784.967	465349.7208	10.55984634
UIJ65926.1	neutral_protease_NprB_[Bacillus_cereus]	CPTF_Ni	3255501.333	453787.2082	13.93908838
UIJ65926.1	neutral_protease_NprB_[Bacillus_cereus]	CPTF_U	4296521.533	589725.8672	13.72565836
UIJ65926.1	neutral_protease_NprB_[Bacillus_cereus]	CPTF_metals_mix	1649877.7	214168.7234	12.98088479
UIJ65926.1	neutral_protease_NprB_[Bacillus_cereus]	CPTF_zcontrol	4512151.333	365048.0667	8.090332964
UIJ65933.1	DNA_starvation/stationary_phase_protection_protein_[Bacillus_cereus]	CPTF_Al	554565.8	111722.7211	20.14598108
UIJ65933.1	DNA_starvation/stationary_phase_protection_protein_[Bacillus_cereus]	CPTF_Cd	645730.5333	348771.0478	54.0118563
UIJ65933.1	DNA_starvation/stationary_phase_protection_protein_[Bacillus_cereus]	CPTF_Co	602776.5667	85799.08344	14.23397793
UIJ65933.1	DNA_starvation/stationary_phase_protection_protein_[Bacillus_cereus]	CPTF_Cu	697076.9	59283.03411	8.504518527
UIJ65933.1	DNA_starvation/stationary_phase_protection_protein_[Bacillus_cereus]	CPTF_Fe	504030.9667	66794.57804	13.25207824
UIJ65933.1	DNA_starvation/stationary_phase_protection_protein_[Bacillus_cereus]	CPTF_Mn	544182.3	103837.5492	19.08139041
UIJ65933.1	DNA_starvation/stationary_phase_protection_protein_[Bacillus_cereus]	CPTF_Ni	352465.1	45567.59556	12.92825745
UIJ65933.1	DNA_starvation/stationary_phase_protection_protein_[Bacillus_cereus]	CPTF_U	478503.6667	104863.1556	21.91480711
UIJ65933.1	DNA_starvation/stationary_phase_protection_protein_[Bacillus_cereus]	CPTF_metals_mix	753578.7	69791.47304	9.261338337
UIJ65933.1	DNA_starvation/stationary_phase_protection_protein_[Bacillus_cereus]	CPTF_zcontrol	336355.7	70308.55322	20.90303605
UIJ65936.1	MazG-like_family_protein_[Bacillus_cereus]	CPTF_Al	772874.9	225045.9612	29.11803207
UIJ65936.1	MazG-like_family_protein_[Bacillus_cereus]	CPTF_Cd	763645.6667	60656.54746	7.943022544

UIJ65936.1	MazG-like_family_protein_[Bacillus_cereus]	CPTF_Co	546228.6667	241752.3325	44.25844838
UIJ65936.1	MazG-like_family_protein_[Bacillus_cereus]	CPTF_Cu	482134.2667	77297.64533	16.03238987
UIJ65936.1	MazG-like_family_protein_[Bacillus_cereus]	CPTF_Fe	853624.2	213132.5115	24.96795563
UIJ65936.1	MazG-like_family_protein_[Bacillus_cereus]	CPTF_Mn	822245.5333	127320.1234	15.48444086
UIJ65936.1	MazG-like_family_protein_[Bacillus_cereus]	CPTF_Ni	559322.5333	94553.58556	16.9050199
UIJ65936.1	MazG-like_family_protein_[Bacillus_cereus]	CPTF_U	482952.6667	199600.3911	41.32918293
UIJ65936.1	MazG-like_family_protein_[Bacillus_cereus]	CPTF_metals_mix	247338.6667	71669.13812	28.97611566
UIJ65936.1	MazG-like_family_protein_[Bacillus_cereus]	CPTF_zcontrol	758592.9667	108473.4033	14.29928935
UIJ65937.1	ferritin_[Bacillus_cereus]	CPTF_Al	41646.09333	68351.86467	164.1255138
UIJ65937.1	ferritin_[Bacillus_cereus]	CPTF_Cd	0	0	0
UIJ65937.1	ferritin_[Bacillus_cereus]	CPTF_Co	0	0	0
UIJ65937.1	ferritin_[Bacillus_cereus]	CPTF_Cu	0	0	0
UIJ65937.1	ferritin_[Bacillus_cereus]	CPTF_Fe	4380.066667	7586.498007	173.2050808
UIJ65937.1	ferritin_[Bacillus_cereus]	CPTF_Mn	0	0	0
UIJ65937.1	ferritin_[Bacillus_cereus]	CPTF_Ni	0	0	0
UIJ65937.1	ferritin_[Bacillus_cereus]	CPTF_U	10407.93333	18027.06934	173.2050808
UIJ65937.1	ferritin_[Bacillus_cereus]	CPTF_metals_mix	715457.9667	275375.2956	38.48937441
UIJ65937.1	ferritin_[Bacillus_cereus]	CPTF_zcontrol	0	0	0
UIJ65945.1	aldo/keto_reductase_[Bacillus_cereus]	CPTF_Al	15735.43333	27254.57001	173.2050808
UIJ65945.1	aldo/keto_reductase_[Bacillus_cereus]	CPTF_Cd	74937.33333	34528.32177	46.07626164
UIJ65945.1	aldo/keto_reductase_[Bacillus_cereus]	CPTF_Co	0	0	0
UIJ65945.1	aldo/keto_reductase_[Bacillus_cereus]	CPTF_Cu	37054	34604.2523	93.38870917
UIJ65945.1	aldo/keto_reductase_[Bacillus_cereus]	CPTF_Fe	8742.533333	15142.51192	173.2050808
UIJ65945.1	aldo/keto_reductase_[Bacillus_cereus]	CPTF_Mn	12830.6	22223.25109	173.2050808
UIJ65945.1	aldo/keto_reductase_[Bacillus_cereus]	CPTF_Ni	0	0	0
UIJ65945.1	aldo/keto_reductase_[Bacillus_cereus]	CPTF_U	0	0	0
UIJ65945.1	aldo/keto_reductase_[Bacillus_cereus]	CPTF_metals_mix	0	0	0
UIJ65945.1	aldo/keto_reductase_[Bacillus_cereus]	CPTF_zcontrol	0	0	0
UIJ65947.1	DUF1641_domain-containing_protein_[Bacillus_cereus]	CPTF_Al	3535681.8	328236.4315	9.283539923
UIJ65947.1	DUF1641_domain-containing_protein_[Bacillus_cereus]	CPTF_Cd	3467132	200291.1083	5.776852692
UIJ65947.1	DUF1641_domain-containing_protein_[Bacillus_cereus]	CPTF_Co	3343860.333	155060.9853	4.637184866
UIJ65947.1	DUF1641_domain-containing_protein_[Bacillus_cereus]	CPTF_Cu	3260813.3	182118.8111	5.585073242
UIJ65947.1	DUF1641_domain-containing_protein_[Bacillus_cereus]	CPTF_Fe	3410185.667	199295.5607	5.844126397
UIJ65947.1	DUF1641_domain-containing_protein_[Bacillus_cereus]	CPTF_Mn	3322275.467	314426.3079	9.464185348
UIJ65947.1	DUF1641_domain-containing_protein_[Bacillus_cereus]	CPTF_Ni	3107970.6	226090.8372	7.274548775
UIJ65947.1	DUF1641_domain-containing_protein_[Bacillus_cereus]	CPTF_U	2985665.033	409585.8567	13.71841289
UIJ65947.1	DUF1641_domain-containing_protein_[Bacillus_cereus]	CPTF_metals_mix	3342539.6	237578.3041	7.107718456
UIJ65947.1	DUF1641_domain-containing_protein_[Bacillus_cereus]	CPTF_zcontrol	3232388.667	95711.89917	2.961026938
UIJ65948.1	NAD(P)/FAD-dependent_oxidoreductase_[Bacillus_cereus]	CPTF_Al	5413516.533	1235591.334	22.82419064
UIJ65948.1	NAD(P)/FAD-dependent_oxidoreductase_[Bacillus_cereus]	CPTF_Cd	8165914.233	742692.9717	9.095037622
UIJ65948.1	NAD(P)/FAD-dependent_oxidoreductase_[Bacillus_cereus]	CPTF_Co	5193850.767	1264541.843	24.34690367
UIJ65948.1	NAD(P)/FAD-dependent_oxidoreductase_[Bacillus_cereus]	CPTF_Cu	4756047.167	595639.5051	12.52383511
UIJ65948.1	NAD(P)/FAD-dependent_oxidoreductase_[Bacillus_cereus]	CPTF_Fe	6508982.267	1745426.525	26.81565956
UIJ65948.1	NAD(P)/FAD-dependent_oxidoreductase_[Bacillus_cereus]	CPTF_Mn	5984082.6	3165881.082	52.90503648
UIJ65948.1	NAD(P)/FAD-dependent_oxidoreductase_[Bacillus_cereus]	CPTF_Ni	2641299.733	353995.4298	13.40231952
UIJ65948.1	NAD(P)/FAD-dependent_oxidoreductase_[Bacillus_cereus]	CPTF_U	3948219.967	595382.1123	15.07976043
UIJ65948.1	NAD(P)/FAD-dependent_oxidoreductase_[Bacillus_cereus]	CPTF_metals_mix	9422154.067	269794.6169	2.863406977
UIJ65948.1	NAD(P)/FAD-dependent_oxidoreductase_[Bacillus_cereus]	CPTF_zcontrol	4839135.233	1678979.792	34.69586427
UIJ65949.1	tyrosine--tRNA_ligase_[Bacillus_cereus]	CPTF_Al	5825532.2	203283.8447	3.489532591
UIJ65949.1	tyrosine--tRNA_ligase_[Bacillus_cereus]	CPTF_Cd	6654576.267	55259.87542	0.830404119
UIJ65949.1	tyrosine--tRNA_ligase_[Bacillus_cereus]	CPTF_Co	6707758.5	167613.2719	2.498797056
UIJ65949.1	tyrosine--tRNA_ligase_[Bacillus_cereus]	CPTF_Cu	6813627.7	308292.0812	4.524639366
UIJ65949.1	tyrosine--tRNA_ligase_[Bacillus_cereus]	CPTF_Fe	6163925.033	91461.95296	1.483826498
UIJ65949.1	tyrosine--tRNA_ligase_[Bacillus_cereus]	CPTF_Mn	5716399.733	178563.7542	3.123710072
UIJ65949.1	tyrosine--tRNA_ligase_[Bacillus_cereus]	CPTF_Ni	5774732.133	438155.322	7.587457079
UIJ65949.1	tyrosine--tRNA_ligase_[Bacillus_cereus]	CPTF_U	6328929.833	1022929.717	16.16275965
UIJ65949.1	tyrosine--tRNA_ligase_[Bacillus_cereus]	CPTF_metals_mix	9772901.133	309783.8057	3.16982441
UIJ65949.1	tyrosine--tRNA_ligase_[Bacillus_cereus]	CPTF_zcontrol	5893769.467	254189.976	4.312859154
UIJ65950.1	UDP-N-acetylmuramate_dehydrogenase_[Bacillus_cereus]	CPTF_Al	113585	109958.8692	96.80756189
UIJ65950.1	UDP-N-acetylmuramate_dehydrogenase_[Bacillus_cereus]	CPTF_Cd	36146	62606.70849	173.2050808
UIJ65950.1	UDP-N-acetylmuramate_dehydrogenase_[Bacillus_cereus]	CPTF_Co	115213.3333	101645.5987	88.22381559

UIJ65950.1	UDP-N-acetylmuramate_dehydrogenase_[Bacillus_cereus]	CPTF_Cu	157015.4	52529.30746	33.45487605
UIJ65950.1	UDP-N-acetylmuramate_dehydrogenase_[Bacillus_cereus]	CPTF_Fe	41891.33333	72557.91773	173.2050808
UIJ65950.1	UDP-N-acetylmuramate_dehydrogenase_[Bacillus_cereus]	CPTF_Mn	61473.33333	106474.9366	173.2050808
UIJ65950.1	UDP-N-acetylmuramate_dehydrogenase_[Bacillus_cereus]	CPTF_Ni	178656.3333	50239.84731	28.12094392
UIJ65950.1	UDP-N-acetylmuramate_dehydrogenase_[Bacillus_cereus]	CPTF_U	143141	125178.1852	87.45096455
UIJ65950.1	UDP-N-acetylmuramate_dehydrogenase_[Bacillus_cereus]	CPTF_metals_mix	86284.33333	149448.8492	173.2050808
UIJ65950.1	UDP-N-acetylmuramate_dehydrogenase_[Bacillus_cereus]	CPTF_zcontrol	180953.6667	16555.28488	9.148908216
UIJ65965.1	SsrA-binding_protein_[Bacillus_cereus]	CPTF_Al	335799.3333	37539.49394	11.17914487
UIJ65965.1	SsrA-binding_protein_[Bacillus_cereus]	CPTF_Cd	330466.6667	31150.3079	9.426157323
UIJ65965.1	SsrA-binding_protein_[Bacillus_cereus]	CPTF_Co	345592.6667	55033.76888	15.92446084
UIJ65965.1	SsrA-binding_protein_[Bacillus_cereus]	CPTF_Cu	393669.6667	8627.886956	2.19165653
UIJ65965.1	SsrA-binding_protein_[Bacillus_cereus]	CPTF_Fe	341371	51952.59457	15.21880727
UIJ65965.1	SsrA-binding_protein_[Bacillus_cereus]	CPTF_Mn	326489.6667	59935.84609	18.35765484
UIJ65965.1	SsrA-binding_protein_[Bacillus_cereus]	CPTF_Ni	342428	45056.89856	13.15806493
UIJ65965.1	SsrA-binding_protein_[Bacillus_cereus]	CPTF_U	315106	93699.38591	29.73583045
UIJ65965.1	SsrA-binding_protein_[Bacillus_cereus]	CPTF_metals_mix	384148.4333	23751.00571	6.182767817
UIJ65965.1	SsrA-binding_protein_[Bacillus_cereus]	CPTF_zcontrol	324540.6667	5971.174619	1.839884869
UIJ65967.1	ribonuclease_R_[Bacillus_cereus]	CPTF_Al	679909.1667	252300.4284	37.107961
UIJ65967.1	ribonuclease_R_[Bacillus_cereus]	CPTF_Cd	1240050.267	235607.3681	18.99982399
UIJ65967.1	ribonuclease_R_[Bacillus_cereus]	CPTF_Co	876517.3	122271.0252	13.94964197
UIJ65967.1	ribonuclease_R_[Bacillus_cereus]	CPTF_Cu	835890.8333	214983.8272	25.71912726
UIJ65967.1	ribonuclease_R_[Bacillus_cereus]	CPTF_Fe	879665.3333	386218.8057	43.90519793
UIJ65967.1	ribonuclease_R_[Bacillus_cereus]	CPTF_Mn	737096.2333	311427.6591	42.25061057
UIJ65967.1	ribonuclease_R_[Bacillus_cereus]	CPTF_Ni	505876.6333	121730.9634	24.06336948
UIJ65967.1	ribonuclease_R_[Bacillus_cereus]	CPTF_U	373275	206389.1268	55.29144111
UIJ65967.1	ribonuclease_R_[Bacillus_cereus]	CPTF_metals_mix	1155876.1	271208.098	23.46342294
UIJ65967.1	ribonuclease_R_[Bacillus_cereus]	CPTF_zcontrol	620493.6667	181978.3288	29.32799134
UIJ65972.1	phosphopyruvate_hydratase_[Bacillus_cereus]	CPTF_Al	41976777.37	2964880.898	7.063145586
UIJ65972.1	phosphopyruvate_hydratase_[Bacillus_cereus]	CPTF_Cd	45951497.13	2698934.596	5.873442138
UIJ65972.1	phosphopyruvate_hydratase_[Bacillus_cereus]	CPTF_Co	37253089.2	4098039.523	11.00053609
UIJ65972.1	phosphopyruvate_hydratase_[Bacillus_cereus]	CPTF_Cu	38976855.63	1786684.947	4.583963787
UIJ65972.1	phosphopyruvate_hydratase_[Bacillus_cereus]	CPTF_Fe	41018479.4	3969026.447	9.676191086
UIJ65972.1	phosphopyruvate_hydratase_[Bacillus_cereus]	CPTF_Mn	44821263.9	10771731.03	24.03263562
UIJ65972.1	phosphopyruvate_hydratase_[Bacillus_cereus]	CPTF_Ni	36595130.77	2158561.626	5.898494091
UIJ65972.1	phosphopyruvate_hydratase_[Bacillus_cereus]	CPTF_U	34659806.33	409926.159	1.182713357
UIJ65972.1	phosphopyruvate_hydratase_[Bacillus_cereus]	CPTF_metals_mix	64133934.6	4018976.708	6.266536948
UIJ65972.1	phosphopyruvate_hydratase_[Bacillus_cereus]	CPTF_zcontrol	34676228.17	903719.0003	2.606162919
UIJ65973.1	2,3-bisphosphoglycerate-independent_phosphoglycerate_mutase_[Bacillus_cereus]	CPTF_Al	7745167.867	556093.8327	7.179880957
UIJ65973.1	2,3-bisphosphoglycerate-independent_phosphoglycerate_mutase_[Bacillus_cereus]	CPTF_Cd	7345658.633	278280.5921	3.78836815
UIJ65973.1	2,3-bisphosphoglycerate-independent_phosphoglycerate_mutase_[Bacillus_cereus]	CPTF_Co	6862882.2	474089.3819	6.908021558
UIJ65973.1	2,3-bisphosphoglycerate-independent_phosphoglycerate_mutase_[Bacillus_cereus]	CPTF_Cu	8194312.667	236162.2009	2.882025747
UIJ65973.1	2,3-bisphosphoglycerate-independent_phosphoglycerate_mutase_[Bacillus_cereus]	CPTF_Fe	7349719.7	498047.8009	6.776418982
UIJ65973.1	2,3-bisphosphoglycerate-independent_phosphoglycerate_mutase_[Bacillus_cereus]	CPTF_Mn	7314686.8	655629.1438	8.963188196
UIJ65973.1	2,3-bisphosphoglycerate-independent_phosphoglycerate_mutase_[Bacillus_cereus]	CPTF_Ni	7243829.367	246669.4818	3.40523595
UIJ65973.1	2,3-bisphosphoglycerate-independent_phosphoglycerate_mutase_[Bacillus_cereus]	CPTF_U	5782908.933	156433.5855	2.705102005
UIJ65973.1	2,3-bisphosphoglycerate-independent_phosphoglycerate_mutase_[Bacillus_cereus]	CPTF_metals_mix	12835850.7	948142.0789	7.386671137
UIJ65973.1	2,3-bisphosphoglycerate-independent_phosphoglycerate_mutase_[Bacillus_cereus]	CPTF_zcontrol	6900095.167	335657.503	4.8645344
UIJ65974.1	triose-phosphate_isomerase_[Bacillus_cereus]	CPTF_Al	11990365.9	3036392.758	25.32360383
UIJ65974.1	triose-phosphate_isomerase_[Bacillus_cereus]	CPTF_Cd	17242702.23	1673059.015	9.702997779
UIJ65974.1	triose-phosphate_isomerase_[Bacillus_cereus]	CPTF_Co	11023816.47	2385623.011	21.64062707
UIJ65974.1	triose-phosphate_isomerase_[Bacillus_cereus]	CPTF_Cu	11073127.03	1202886.231	10.86311235
UIJ65974.1	triose-phosphate_isomerase_[Bacillus_cereus]	CPTF_Fe	12235821.47	2786030.112	22.76945704
UIJ65974.1	triose-phosphate_isomerase_[Bacillus_cereus]	CPTF_Mn	14143850.47	9008077.308	63.68900272
UIJ65974.1	triose-phosphate_isomerase_[Bacillus_cereus]	CPTF_Ni	6331542.6	1506230.596	23.78931472
UIJ65974.1	triose-phosphate_isomerase_[Bacillus_cereus]	CPTF_U	7473765.333	1451255.115	19.41799147
UIJ65974.1	triose-phosphate_isomerase_[Bacillus_cereus]	CPTF_metals_mix	38199736.3	1630194.152	4.267553418
UIJ65974.1	triose-phosphate_isomerase_[Bacillus_cereus]	CPTF_zcontrol	9046136.367	1965348.197	21.72582987
UIJ65975.1	phosphoglycerate_kinase_[Bacillus_cereus]	CPTF_Al	24654229.87	2318654.565	9.404692734
UIJ65975.1	phosphoglycerate_kinase_[Bacillus_cereus]	CPTF_Cd	29200370.13	1332978.579	4.564937268
UIJ65975.1	phosphoglycerate_kinase_[Bacillus_cereus]	CPTF_Co	22104096.23	2309920.632	10.45019261
UIJ65975.1	phosphoglycerate_kinase_[Bacillus_cereus]	CPTF_Cu	23960930.8	1979717.508	8.262272966

UIJ65975.1	phosphoglycerate_kinase_[Bacillus_cereus]	CPTF_Fe	24738765.93	3255583.647	13.1598466
UIJ65975.1	phosphoglycerate_kinase_[Bacillus_cereus]	CPTF_Mn	26025355.23	7774041.516	29.87102941
UIJ65975.1	phosphoglycerate_kinase_[Bacillus_cereus]	CPTF_Ni	17273003.4	2805508.234	16.24215644
UIJ65975.1	phosphoglycerate_kinase_[Bacillus_cereus]	CPTF_U	17416654.67	2170912.744	12.46457937
UIJ65975.1	phosphoglycerate_kinase_[Bacillus_cereus]	CPTF_metals_mix	38975852.13	2470302.496	6.338033322
UIJ65975.1	phosphoglycerate_kinase_[Bacillus_cereus]	CPTF_zcontrol	22112348.43	1080757.269	4.887573438
UIJ65976.1	type_I_glyceraldehyde-3-phosphate_dehydrogenase_[Bacillus_cereus]	CPTF_Al	19691539.37	3471704.651	17.63043806
UIJ65976.1	type_I_glyceraldehyde-3-phosphate_dehydrogenase_[Bacillus_cereus]	CPTF_Cd	28924798.07	3886940.137	13.4380891
UIJ65976.1	type_I_glyceraldehyde-3-phosphate_dehydrogenase_[Bacillus_cereus]	CPTF_Co	17835397	3553626.234	19.92457041
UIJ65976.1	type_I_glyceraldehyde-3-phosphate_dehydrogenase_[Bacillus_cereus]	CPTF_Cu	16925945.67	3565192.534	21.06347618
UIJ65976.1	type_I_glyceraldehyde-3-phosphate_dehydrogenase_[Bacillus_cereus]	CPTF_Fe	18831842.4	4362976.843	23.16808282
UIJ65976.1	type_I_glyceraldehyde-3-phosphate_dehydrogenase_[Bacillus_cereus]	CPTF_Mn	27399244	21310628.07	77.77816085
UIJ65976.1	type_I_glyceraldehyde-3-phosphate_dehydrogenase_[Bacillus_cereus]	CPTF_Ni	10329489.67	3601372.046	34.86495619
UIJ65976.1	type_I_glyceraldehyde-3-phosphate_dehydrogenase_[Bacillus_cereus]	CPTF_U	9371748.833	2639560.414	28.16507848
UIJ65976.1	type_I_glyceraldehyde-3-phosphate_dehydrogenase_[Bacillus_cereus]	CPTF_metals_mix	62728493.4	4211140.222	6.713281307
UIJ65976.1	type_I_glyceraldehyde-3-phosphate_dehydrogenase_[Bacillus_cereus]	CPTF_zcontrol	15373643.67	2526750.398	16.43559882
UIJ65977.1	gapA_transcriptional_regulator_CggR_[Bacillus_cereus]	CPTF_Al	250004	32001.46195	12.80037997
UIJ65977.1	gapA_transcriptional_regulator_CggR_[Bacillus_cereus]	CPTF_Cd	447804	46925.2361	10.47896761
UIJ65977.1	gapA_transcriptional_regulator_CggR_[Bacillus_cereus]	CPTF_Co	281814.3333	90350.79723	32.06039812
UIJ65977.1	gapA_transcriptional_regulator_CggR_[Bacillus_cereus]	CPTF_Cu	402179	96155.8017	23.90870774
UIJ65977.1	gapA_transcriptional_regulator_CggR_[Bacillus_cereus]	CPTF_Fe	314874.6667	54717.36089	17.37750498
UIJ65977.1	gapA_transcriptional_regulator_CggR_[Bacillus_cereus]	CPTF_Mn	252283.0667	83381.27217	33.05068123
UIJ65977.1	gapA_transcriptional_regulator_CggR_[Bacillus_cereus]	CPTF_Ni	260218.6667	31622.53447	12.15229287
UIJ65977.1	gapA_transcriptional_regulator_CggR_[Bacillus_cereus]	CPTF_U	289454.6667	32947.43393	11.38258861
UIJ65977.1	gapA_transcriptional_regulator_CggR_[Bacillus_cereus]	CPTF_metals_mix	535309.6667	38241.23769	7.143759972
UIJ65977.1	gapA_transcriptional_regulator_CggR_[Bacillus_cereus]	CPTF_zcontrol	258773.3333	77508.54149	29.95229087
UIJ65991.1	ATP-dependent_Clp_endopeptidase_proteolytic_subunit_ClpP_[Bacillus_cereus]	CPTF_Al	10751371.67	487290.3617	4.532355283
UIJ65991.1	ATP-dependent_Clp_endopeptidase_proteolytic_subunit_ClpP_[Bacillus_cereus]	CPTF_Cd	12101723.67	443604.2535	3.66562868
UIJ65991.1	ATP-dependent_Clp_endopeptidase_proteolytic_subunit_ClpP_[Bacillus_cereus]	CPTF_Co	11244683.67	532241.093	4.733268705
UIJ65991.1	ATP-dependent_Clp_endopeptidase_proteolytic_subunit_ClpP_[Bacillus_cereus]	CPTF_Cu	11580137	1138271.636	9.829517874
UIJ65991.1	ATP-dependent_Clp_endopeptidase_proteolytic_subunit_ClpP_[Bacillus_cereus]	CPTF_Fe	11037713	214136.7883	1.940046714
UIJ65991.1	ATP-dependent_Clp_endopeptidase_proteolytic_subunit_ClpP_[Bacillus_cereus]	CPTF_Mn	11724736.97	4122076.402	35.15709063
UIJ65991.1	ATP-dependent_Clp_endopeptidase_proteolytic_subunit_ClpP_[Bacillus_cereus]	CPTF_Ni	10545205.67	1437355.899	13.63042072
UIJ65991.1	ATP-dependent_Clp_endopeptidase_proteolytic_subunit_ClpP_[Bacillus_cereus]	CPTF_U	8561287	195839.2619	2.287497919
UIJ65991.1	ATP-dependent_Clp_endopeptidase_proteolytic_subunit_ClpP_[Bacillus_cereus]	CPTF_metals_mix	16585429.37	1433261.497	8.641690639
UIJ65991.1	ATP-dependent_Clp_endopeptidase_proteolytic_subunit_ClpP_[Bacillus_cereus]	CPTF_zcontrol	9848393.333	121803.1665	1.236782106
UIJ65995.1	RNase_adapter_RapZ_[Bacillus_cereus]	CPTF_Al	3217837.267	85285.76616	2.65040644
UIJ65995.1	RNase_adapter_RapZ_[Bacillus_cereus]	CPTF_Cd	3127237.867	357220.173	11.42286542
UIJ65995.1	RNase_adapter_RapZ_[Bacillus_cereus]	CPTF_Co	2653020.9	315620.1302	11.89663188
UIJ65995.1	RNase_adapter_RapZ_[Bacillus_cereus]	CPTF_Cu	3051491.267	234544.3005	7.686218966
UIJ65995.1	RNase_adapter_RapZ_[Bacillus_cereus]	CPTF_Fe	2855902	305446.3348	10.69526667
UIJ65995.1	RNase_adapter_RapZ_[Bacillus_cereus]	CPTF_Mn	2505559.1	305643.5633	12.1986172
UIJ65995.1	RNase_adapter_RapZ_[Bacillus_cereus]	CPTF_Ni	2974382.333	104075.5277	3.499063538
UIJ65995.1	RNase_adapter_RapZ_[Bacillus_cereus]	CPTF_U	3417876.5	221448.097	6.47911348
UIJ65995.1	RNase_adapter_RapZ_[Bacillus_cereus]	CPTF_metals_mix	3429175.533	323776.5299	9.441818501
UIJ65995.1	RNase_adapter_RapZ_[Bacillus_cereus]	CPTF_zcontrol	2532298.6	102100.4619	4.031928223
UIJ65998.1	thioredoxin-disulfide_reductase_[Bacillus_cereus]	CPTF_Al	152040	56689.70447	37.28604609
UIJ65998.1	thioredoxin-disulfide_reductase_[Bacillus_cereus]	CPTF_Cd	123949.7667	28562.47621	23.04359014
UIJ65998.1	thioredoxin-disulfide_reductase_[Bacillus_cereus]	CPTF_Co	284561.4667	97757.39821	34.35370198
UIJ65998.1	thioredoxin-disulfide_reductase_[Bacillus_cereus]	CPTF_Cu	331559.6667	167837.3481	50.62055641
UIJ65998.1	thioredoxin-disulfide_reductase_[Bacillus_cereus]	CPTF_Fe	179566.6667	92469.76441	51.49606334
UIJ65998.1	thioredoxin-disulfide_reductase_[Bacillus_cereus]	CPTF_Mn	384854.6333	355422.9401	92.35251686
UIJ65998.1	thioredoxin-disulfide_reductase_[Bacillus_cereus]	CPTF_Ni	199893	92976.229	46.51299895
UIJ65998.1	thioredoxin-disulfide_reductase_[Bacillus_cereus]	CPTF_U	234865	145926.1628	62.13193232
UIJ65998.1	thioredoxin-disulfide_reductase_[Bacillus_cereus]	CPTF_metals_mix	3698566.667	719597.1153	19.45610773
UIJ65998.1	thioredoxin-disulfide_reductase_[Bacillus_cereus]	CPTF_zcontrol	130144.2	72833.25439	55.96350386
UIJ66001.1	pyrophosphatase_PpaX_[Bacillus_cereus]	CPTF_Al	22783.3	39461.83316	173.2050808
UIJ66001.1	pyrophosphatase_PpaX_[Bacillus_cereus]	CPTF_Cd	22651.26667	39233.14472	173.2050808
UIJ66001.1	pyrophosphatase_PpaX_[Bacillus_cereus]	CPTF_Co	30433.33333	41885.52541	137.630423
UIJ66001.1	pyrophosphatase_PpaX_[Bacillus_cereus]	CPTF_Cu	49115.43333	64605.82125	131.5387382
UIJ66001.1	pyrophosphatase_PpaX_[Bacillus_cereus]	CPTF_Fe	0	0	0

UIJ66001.1	pyrophosphatase_PpaX_[Bacillus_cereus]	CPTF_Mn	0	0	0
UIJ66001.1	pyrophosphatase_PpaX_[Bacillus_cereus]	CPTF_Ni	0	0	0
UIJ66001.1	pyrophosphatase_PpaX_[Bacillus_cereus]	CPTF_U	10101.93333	17497.06179	173.2050808
UIJ66001.1	pyrophosphatase_PpaX_[Bacillus_cereus]	CPTF_metals_mix	112246	24207.08916	21.56610406
UIJ66001.1	pyrophosphatase_PpaX_[Bacillus_cereus]	CPTF_zcontrol	0	0	0
UIJ66003.1	HPr(Ser)_kinase/phosphatase_[Bacillus_cereus]	CPTF_Al	679344.4667	99175.56529	14.5987154
UIJ66003.1	HPr(Ser)_kinase/phosphatase_[Bacillus_cereus]	CPTF_Cd	467449.8333	159283.9271	34.0750848
UIJ66003.1	HPr(Ser)_kinase/phosphatase_[Bacillus_cereus]	CPTF_Co	1030735.4	197905.3062	19.20039868
UIJ66003.1	HPr(Ser)_kinase/phosphatase_[Bacillus_cereus]	CPTF_Cu	899181.7667	34822.61276	3.872700054
UIJ66003.1	HPr(Ser)_kinase/phosphatase_[Bacillus_cereus]	CPTF_Fe	695170.0333	286537.1221	41.21827875
UIJ66003.1	HPr(Ser)_kinase/phosphatase_[Bacillus_cereus]	CPTF_Mn	619364.8333	260166.1239	42.00531091
UIJ66003.1	HPr(Ser)_kinase/phosphatase_[Bacillus_cereus]	CPTF_Ni	453586.6333	484434.5881	106.8008959
UIJ66003.1	HPr(Ser)_kinase/phosphatase_[Bacillus_cereus]	CPTF_U	394106	347696.9533	88.22422223
UIJ66003.1	HPr(Ser)_kinase/phosphatase_[Bacillus_cereus]	CPTF_metals_mix	1206237.4	85697.31191	7.104514576
UIJ66003.1	HPr(Ser)_kinase/phosphatase_[Bacillus_cereus]	CPTF_zcontrol	433562	250567.7863	57.79283847
UIJ66005.1	excinuclease_ABC_subunit_UvrA_[Bacillus_cereus]	CPTF_Al	831080	156735.793	18.85929068
UIJ66005.1	excinuclease_ABC_subunit_UvrA_[Bacillus_cereus]	CPTF_Cd	1353818.7	474017.8469	35.01339189
UIJ66005.1	excinuclease_ABC_subunit_UvrA_[Bacillus_cereus]	CPTF_Co	684196.8	120451.1092	17.60474607
UIJ66005.1	excinuclease_ABC_subunit_UvrA_[Bacillus_cereus]	CPTF_Cu	661491.5667	51542.01102	7.791786565
UIJ66005.1	excinuclease_ABC_subunit_UvrA_[Bacillus_cereus]	CPTF_Fe	763872.6667	71768.17528	9.395306104
UIJ66005.1	excinuclease_ABC_subunit_UvrA_[Bacillus_cereus]	CPTF_Mn	691707.8333	118466.3465	17.12664521
UIJ66005.1	excinuclease_ABC_subunit_UvrA_[Bacillus_cereus]	CPTF_Ni	654951.7	15499.19361	2.366463604
UIJ66005.1	excinuclease_ABC_subunit_UvrA_[Bacillus_cereus]	CPTF_U	590587.6667	281545.2166	47.67204472
UIJ66005.1	excinuclease_ABC_subunit_UvrA_[Bacillus_cereus]	CPTF_metals_mix	1801469.5	519577.5664	28.84187417
UIJ66005.1	excinuclease_ABC_subunit_UvrA_[Bacillus_cereus]	CPTF_zcontrol	1143438.767	813880.2938	71.17830159
UIJ66006.1	excinuclease_ABC_subunit_B_[Bacillus_cereus]	CPTF_Al	36303.6	32050.7452	88.28530836
UIJ66006.1	excinuclease_ABC_subunit_B_[Bacillus_cereus]	CPTF_Cd	56268.9	18196.69046	32.33880609
UIJ66006.1	excinuclease_ABC_subunit_B_[Bacillus_cereus]	CPTF_Co	43523.5	37896.16679	87.07058668
UIJ66006.1	excinuclease_ABC_subunit_B_[Bacillus_cereus]	CPTF_Cu	52650.9	42047.17876	79.86032292
UIJ66006.1	excinuclease_ABC_subunit_B_[Bacillus_cereus]	CPTF_Fe	85984.83333	28381.97572	33.00811855
UIJ66006.1	excinuclease_ABC_subunit_B_[Bacillus_cereus]	CPTF_Mn	47163.86667	45101.50487	95.62724191
UIJ66006.1	excinuclease_ABC_subunit_B_[Bacillus_cereus]	CPTF_Ni	62534.93333	55805.78643	89.23937942
UIJ66006.1	excinuclease_ABC_subunit_B_[Bacillus_cereus]	CPTF_U	12112.63333	20979.69635	173.2050808
UIJ66006.1	excinuclease_ABC_subunit_B_[Bacillus_cereus]	CPTF_metals_mix	47285.8	45651.72024	96.54424847
UIJ66006.1	excinuclease_ABC_subunit_B_[Bacillus_cereus]	CPTF_zcontrol	35426.76667	34983.80006	98.74962732
UIJ66020.1	permease-like_cell_division_protein_FtsX_[Bacillus_cereus]	CPTF_Al	137229	137059.3163	99.87634996
UIJ66020.1	permease-like_cell_division_protein_FtsX_[Bacillus_cereus]	CPTF_Cd	535491	20274.24911	3.786104549
UIJ66020.1	permease-like_cell_division_protein_FtsX_[Bacillus_cereus]	CPTF_Co	127725.5333	155747.6136	121.939294
UIJ66020.1	permease-like_cell_division_protein_FtsX_[Bacillus_cereus]	CPTF_Cu	0	0	0
UIJ66020.1	permease-like_cell_division_protein_FtsX_[Bacillus_cereus]	CPTF_Fe	269112.3333	249820.0494	92.83114093
UIJ66020.1	permease-like_cell_division_protein_FtsX_[Bacillus_cereus]	CPTF_Mn	275078.3333	254444.6623	92.49898354
UIJ66020.1	permease-like_cell_division_protein_FtsX_[Bacillus_cereus]	CPTF_Ni	0	0	0
UIJ66020.1	permease-like_cell_division_protein_FtsX_[Bacillus_cereus]	CPTF_U	0	0	0
UIJ66020.1	permease-like_cell_division_protein_FtsX_[Bacillus_cereus]	CPTF_metals_mix	561671	101907.7245	18.14366854
UIJ66020.1	permease-like_cell_division_protein_FtsX_[Bacillus_cereus]	CPTF_zcontrol	153964.6667	162737.0755	105.6976766
UIJ66021.1	cytochrome_c-551_[Bacillus_cereus]	CPTF_Al	287788.8333	85158.16624	29.590504
UIJ66021.1	cytochrome_c-551_[Bacillus_cereus]	CPTF_Cd	343430.4	165942.5382	48.3191174
UIJ66021.1	cytochrome_c-551_[Bacillus_cereus]	CPTF_Co	363990.9	71148.80491	19.54686365
UIJ66021.1	cytochrome_c-551_[Bacillus_cereus]	CPTF_Cu	242709.5333	90456.38682	37.2694
UIJ66021.1	cytochrome_c-551_[Bacillus_cereus]	CPTF_Fe	192895.1667	216818.2263	112.4021043
UIJ66021.1	cytochrome_c-551_[Bacillus_cereus]	CPTF_Mn	203034.9	181647.6454	89.4662176
UIJ66021.1	cytochrome_c-551_[Bacillus_cereus]	CPTF_Ni	391599.2	83815.84806	21.40347786
UIJ66021.1	cytochrome_c-551_[Bacillus_cereus]	CPTF_U	285616.2333	200686.6584	70.26444401
UIJ66021.1	cytochrome_c-551_[Bacillus_cereus]	CPTF_metals_mix	214935.7667	55936.73492	26.02486119
UIJ66021.1	cytochrome_c-551_[Bacillus_cereus]	CPTF_zcontrol	274593.9333	25121.22988	9.148501417
UIJ66022.1	peptide_chain_release_factor_2_[Bacillus_cereus]	CPTF_Al	2568346.433	220993.266	8.604495996
UIJ66022.1	peptide_chain_release_factor_2_[Bacillus_cereus]	CPTF_Cd	2582504.433	131748.8069	5.101590734
UIJ66022.1	peptide_chain_release_factor_2_[Bacillus_cereus]	CPTF_Co	1915984.933	668373.5497	34.88407127
UIJ66022.1	peptide_chain_release_factor_2_[Bacillus_cereus]	CPTF_Cu	2138831.6	496677.0863	23.22188836
UIJ66022.1	peptide_chain_release_factor_2_[Bacillus_cereus]	CPTF_Fe	2575651.967	137912.7906	5.354480823
UIJ66022.1	peptide_chain_release_factor_2_[Bacillus_cereus]	CPTF_Mn	2594557.5	433326.2479	16.70135458

UIJ66022.1	peptide_chain_release_factor_2_[Bacillus_cereus]	CPTF_Ni	2088762.433	726250.6341	34.76942243
UIJ66022.1	peptide_chain_release_factor_2_[Bacillus_cereus]	CPTF_U	2728367.233	71198.90403	2.609579208
UIJ66022.1	peptide_chain_release_factor_2_[Bacillus_cereus]	CPTF_metals_mix	3651471.233	298619.9419	8.178071875
UIJ66022.1	peptide_chain_release_factor_2_[Bacillus_cereus]	CPTF_zcontrol	2252430.333	641219.9557	28.46791513
UIJ66023.1	preprotein_translocase_subunit_SecA_[Bacillus_cereus]	CPTF_Al	9286654.367	899979.7026	9.691107982
UIJ66023.1	preprotein_translocase_subunit_SecA_[Bacillus_cereus]	CPTF_Cd	10656588.7	479137.0155	4.496157532
UIJ66023.1	preprotein_translocase_subunit_SecA_[Bacillus_cereus]	CPTF_Co	9499485.3	305283.5018	3.213684659
UIJ66023.1	preprotein_translocase_subunit_SecA_[Bacillus_cereus]	CPTF_Cu	9318542.2	270372.4434	2.901445715
UIJ66023.1	preprotein_translocase_subunit_SecA_[Bacillus_cereus]	CPTF_Fe	8925597.967	985664.9359	11.04312495
UIJ66023.1	preprotein_translocase_subunit_SecA_[Bacillus_cereus]	CPTF_Mn	9322346.3	1270128.573	13.62455902
UIJ66023.1	preprotein_translocase_subunit_SecA_[Bacillus_cereus]	CPTF_Ni	7540931.533	937132.4119	12.42727649
UIJ66023.1	preprotein_translocase_subunit_SecA_[Bacillus_cereus]	CPTF_U	7734005.1	885385.1747	11.44795178
UIJ66023.1	preprotein_translocase_subunit_SecA_[Bacillus_cereus]	CPTF_metals_mix	14132158.73	656553.5756	4.645812349
UIJ66023.1	preprotein_translocase_subunit_SecA_[Bacillus_cereus]	CPTF_zcontrol	8320913.133	622821.9714	7.48501951
UIJ66030.1	ribosome-associated_translation_inhibitor_RaiA_[Bacillus_cereus]	CPTF_Al	16982392.67	1311229.193	7.72110985
UIJ66030.1	ribosome-associated_translation_inhibitor_RaiA_[Bacillus_cereus]	CPTF_Cd	17815723	598762.1933	3.360863847
UIJ66030.1	ribosome-associated_translation_inhibitor_RaiA_[Bacillus_cereus]	CPTF_Co	14618831	1780256.171	12.17782852
UIJ66030.1	ribosome-associated_translation_inhibitor_RaiA_[Bacillus_cereus]	CPTF_Cu	17488419.67	1308636.025	7.48287181
UIJ66030.1	ribosome-associated_translation_inhibitor_RaiA_[Bacillus_cereus]	CPTF_Fe	16923418	1761043.452	10.40595613
UIJ66030.1	ribosome-associated_translation_inhibitor_RaiA_[Bacillus_cereus]	CPTF_Mn	16551655.8	1844166.076	11.14188271
UIJ66030.1	ribosome-associated_translation_inhibitor_RaiA_[Bacillus_cereus]	CPTF_Ni	14925627.93	1687077.962	11.30322938
UIJ66030.1	ribosome-associated_translation_inhibitor_RaiA_[Bacillus_cereus]	CPTF_U	12012462.27	1777525.017	14.79734111
UIJ66030.1	ribosome-associated_translation_inhibitor_RaiA_[Bacillus_cereus]	CPTF_metals_mix	16605523.27	915562.9587	5.513604985
UIJ66030.1	ribosome-associated_translation_inhibitor_RaiA_[Bacillus_cereus]	CPTF_zcontrol	15204680	948133.625	6.235801247
UIJ66034.1	helix-turn-helix_transcriptional_regulator_[Bacillus_cereus]	CPTF_Al	1281271.333	54363.49063	4.2429335
UIJ66034.1	helix-turn-helix_transcriptional_regulator_[Bacillus_cereus]	CPTF_Cd	1297830.667	18293.90626	1.409575743
UIJ66034.1	helix-turn-helix_transcriptional_regulator_[Bacillus_cereus]	CPTF_Co	1090903.667	96956.99996	8.887769188
UIJ66034.1	helix-turn-helix_transcriptional_regulator_[Bacillus_cereus]	CPTF_Cu	1215123	118247.4407	9.731314501
UIJ66034.1	helix-turn-helix_transcriptional_regulator_[Bacillus_cereus]	CPTF_Fe	1282198	80192.70805	6.254315484
UIJ66034.1	helix-turn-helix_transcriptional_regulator_[Bacillus_cereus]	CPTF_Mn	982449.8	311250.7419	31.68108354
UIJ66034.1	helix-turn-helix_transcriptional_regulator_[Bacillus_cereus]	CPTF_Ni	1134898.333	39833.3128	3.50985737
UIJ66034.1	helix-turn-helix_transcriptional_regulator_[Bacillus_cereus]	CPTF_U	1008504.833	230212.5449	22.82711369
UIJ66034.1	helix-turn-helix_transcriptional_regulator_[Bacillus_cereus]	CPTF_metals_mix	1412740.133	69255.31092	4.902197459
UIJ66034.1	helix-turn-helix_transcriptional_regulator_[Bacillus_cereus]	CPTF_zcontrol	1180251.333	89515.89218	7.584477107
UIJ66035.1	DegV_family_protein_[Bacillus_cereus]	CPTF_Al	0	0	0
UIJ66035.1	DegV_family_protein_[Bacillus_cereus]	CPTF_Cd	0	0	0
UIJ66035.1	DegV_family_protein_[Bacillus_cereus]	CPTF_Co	0	0	0
UIJ66035.1	DegV_family_protein_[Bacillus_cereus]	CPTF_Cu	0	0	0
UIJ66035.1	DegV_family_protein_[Bacillus_cereus]	CPTF_Fe	0	0	0
UIJ66035.1	DegV_family_protein_[Bacillus_cereus]	CPTF_Mn	0	0	0
UIJ66035.1	DegV_family_protein_[Bacillus_cereus]	CPTF_Ni	0	0	0
UIJ66035.1	DegV_family_protein_[Bacillus_cereus]	CPTF_U	0	0	0
UIJ66035.1	DegV_family_protein_[Bacillus_cereus]	CPTF_metals_mix	126049	25304.14346	20.07484665
UIJ66035.1	DegV_family_protein_[Bacillus_cereus]	CPTF_zcontrol	0	0	0
UIJ66038.1	UDP-N-acetylglucosamine_2-epimerase_(non-hydrolyzing)_[Bacillus_cereus]	CPTF_Al	2444176.867	296030.2096	12.11165254
UIJ66038.1	UDP-N-acetylglucosamine_2-epimerase_(non-hydrolyzing)_[Bacillus_cereus]	CPTF_Cd	2915180.033	399689.4515	13.71062668
UIJ66038.1	UDP-N-acetylglucosamine_2-epimerase_(non-hydrolyzing)_[Bacillus_cereus]	CPTF_Co	2797958.567	353282.8123	12.62644903
UIJ66038.1	UDP-N-acetylglucosamine_2-epimerase_(non-hydrolyzing)_[Bacillus_cereus]	CPTF_Cu	2796838.033	22695.35028	0.81146459
UIJ66038.1	UDP-N-acetylglucosamine_2-epimerase_(non-hydrolyzing)_[Bacillus_cereus]	CPTF_Fe	2411400.8	121302.2976	5.030366484
UIJ66038.1	UDP-N-acetylglucosamine_2-epimerase_(non-hydrolyzing)_[Bacillus_cereus]	CPTF_Mn	2490036.233	127813.8401	5.133011253
UIJ66038.1	UDP-N-acetylglucosamine_2-epimerase_(non-hydrolyzing)_[Bacillus_cereus]	CPTF_Ni	2277834.633	237204.3159	10.41358808
UIJ66038.1	UDP-N-acetylglucosamine_2-epimerase_(non-hydrolyzing)_[Bacillus_cereus]	CPTF_U	2103713.633	95041.22969	4.517783608
UIJ66038.1	UDP-N-acetylglucosamine_2-epimerase_(non-hydrolyzing)_[Bacillus_cereus]	CPTF_metals_mix	3755393.9	243052.6003	6.472093387
UIJ66038.1	UDP-N-acetylglucosamine_2-epimerase_(non-hydrolyzing)_[Bacillus_cereus]	CPTF_zcontrol	2512731.2	821158.5724	32.67992105
UIJ66071.1	D-amino-acid_transaminase_[Bacillus_cereus]	CPTF_Al	926751.3333	12998.29827	1.40256591
UIJ66071.1	D-amino-acid_transaminase_[Bacillus_cereus]	CPTF_Cd	1024969	70713.83049	6.899118948
UIJ66071.1	D-amino-acid_transaminase_[Bacillus_cereus]	CPTF_Co	988593.6667	118635.0204	12.0003824
UIJ66071.1	D-amino-acid_transaminase_[Bacillus_cereus]	CPTF_Cu	844623.9667	30110.69558	3.564982379
UIJ66071.1	D-amino-acid_transaminase_[Bacillus_cereus]	CPTF_Fe	1046365.067	80669.37027	7.709486186
UIJ66071.1	D-amino-acid_transaminase_[Bacillus_cereus]	CPTF_Mn	961191.4667	121469.7439	12.63741389
UIJ66071.1	D-amino-acid_transaminase_[Bacillus_cereus]	CPTF_Ni	997216.4667	65493.56437	6.567637675

UIJ66071.1	D-amino-acid_transaminase_[Bacillus_cereus]	CPTF_U	1096774.667	122929.8794	11.2083077
UIJ66071.1	D-amino-acid_transaminase_[Bacillus_cereus]	CPTF_metals_mix	989107	142445.4487	14.40141953
UIJ66071.1	D-amino-acid_transaminase_[Bacillus_cereus]	CPTF_zcontrol	768864	259144.2195	33.70481899
UIJ66072.1	SH3_domain-containing_protein_[Bacillus_cereus]	CPTF_Al	34302.63333	59413.90377	173.2050808
UIJ66072.1	SH3_domain-containing_protein_[Bacillus_cereus]	CPTF_Cd	51402.26667	25155.75782	48.93900493
UIJ66072.1	SH3_domain-containing_protein_[Bacillus_cereus]	CPTF_Co	26628.03333	25829.55352	97.00135641
UIJ66072.1	SH3_domain-containing_protein_[Bacillus_cereus]	CPTF_Cu	117528.8333	144547	122.9885432
UIJ66072.1	SH3_domain-containing_protein_[Bacillus_cereus]	CPTF_Fe	48955.91333	43647.20012	89.15613487
UIJ66072.1	SH3_domain-containing_protein_[Bacillus_cereus]	CPTF_Mn	27195.16667	47103.41039	173.2050808
UIJ66072.1	SH3_domain-containing_protein_[Bacillus_cereus]	CPTF_Ni	51487.16667	37144.43204	72.14308816
UIJ66072.1	SH3_domain-containing_protein_[Bacillus_cereus]	CPTF_U	46146.46667	47703.90205	103.374983
UIJ66072.1	SH3_domain-containing_protein_[Bacillus_cereus]	CPTF_metals_mix	377162.8333	98846.4198	26.20788982
UIJ66072.1	SH3_domain-containing_protein_[Bacillus_cereus]	CPTF_zcontrol	76362.3	72899.56837	95.46539113
UIJ66124.1	chaperonin_GroEL_[Bacillus_cereus]	CPTF_Al	89955192.63	4420767.117	4.91441015
UIJ66124.1	chaperonin_GroEL_[Bacillus_cereus]	CPTF_Cd	101780121	846136.7381	0.831337917
UIJ66124.1	chaperonin_GroEL_[Bacillus_cereus]	CPTF_Co	84622683.97	6612467.235	7.814059925
UIJ66124.1	chaperonin_GroEL_[Bacillus_cereus]	CPTF_Cu	87067757.87	3302074.084	3.792533729
UIJ66124.1	chaperonin_GroEL_[Bacillus_cereus]	CPTF_Fe	90731687.77	7897445.901	8.704176121
UIJ66124.1	chaperonin_GroEL_[Bacillus_cereus]	CPTF_Mn	98682439.67	24040906.77	24.36188936
UIJ66124.1	chaperonin_GroEL_[Bacillus_cereus]	CPTF_Ni	75083543.73	4942664	6.582885882
UIJ66124.1	chaperonin_GroEL_[Bacillus_cereus]	CPTF_U	78875619.23	5280549.472	6.694780369
UIJ66124.1	chaperonin_GroEL_[Bacillus_cereus]	CPTF_metals_mix	127718465.7	12312873.38	9.640636776
UIJ66124.1	chaperonin_GroEL_[Bacillus_cereus]	CPTF_zcontrol	80095832.47	2495525.076	3.115674061
UIJ66125.1	co-chaperone_GroES_[Bacillus_cereus]	CPTF_Al	30016260.33	790132.1767	2.632347161
UIJ66125.1	co-chaperone_GroES_[Bacillus_cereus]	CPTF_Cd	31775831.13	1107278.071	3.484654944
UIJ66125.1	co-chaperone_GroES_[Bacillus_cereus]	CPTF_Co	29520022.4	1060949.869	3.594000896
UIJ66125.1	co-chaperone_GroES_[Bacillus_cereus]	CPTF_Cu	30479457.33	1643375.801	5.391748886
UIJ66125.1	co-chaperone_GroES_[Bacillus_cereus]	CPTF_Fe	29910617.6	3064897.999	10.24685628
UIJ66125.1	co-chaperone_GroES_[Bacillus_cereus]	CPTF_Mn	31519638.83	1884454.125	5.978666617
UIJ66125.1	co-chaperone_GroES_[Bacillus_cereus]	CPTF_Ni	24721366.97	2340298.786	9.466704608
UIJ66125.1	co-chaperone_GroES_[Bacillus_cereus]	CPTF_U	22525862.33	1679207.042	7.454573847
UIJ66125.1	co-chaperone_GroES_[Bacillus_cereus]	CPTF_metals_mix	30879965.3	656015.2598	2.124404135
UIJ66125.1	co-chaperone_GroES_[Bacillus_cereus]	CPTF_zcontrol	30967874.1	1731866.397	5.592461372
UIJ66128.1	redox-sensing_transcriptional_repressor_Rex_[Bacillus_cereus]	CPTF_Al	304436.6667	81222.02562	26.67944913
UIJ66128.1	redox-sensing_transcriptional_repressor_Rex_[Bacillus_cereus]	CPTF_Cd	365468.2	222583.1872	60.9035717
UIJ66128.1	redox-sensing_transcriptional_repressor_Rex_[Bacillus_cereus]	CPTF_Co	337085.0667	198294.3542	58.82620555
UIJ66128.1	redox-sensing_transcriptional_repressor_Rex_[Bacillus_cereus]	CPTF_Cu	381204.6667	342859.8558	89.94114862
UIJ66128.1	redox-sensing_transcriptional_repressor_Rex_[Bacillus_cereus]	CPTF_Fe	171775.9333	181620.9785	105.7313298
UIJ66128.1	redox-sensing_transcriptional_repressor_Rex_[Bacillus_cereus]	CPTF_Mn	370125.6667	345677.4069	93.39460568
UIJ66128.1	redox-sensing_transcriptional_repressor_Rex_[Bacillus_cereus]	CPTF_Ni	338018.9333	296264.0668	87.64718113
UIJ66128.1	redox-sensing_transcriptional_repressor_Rex_[Bacillus_cereus]	CPTF_U	430078.3	120684.7094	28.06110176
UIJ66128.1	redox-sensing_transcriptional_repressor_Rex_[Bacillus_cereus]	CPTF_metals_mix	1424498	135947.4749	9.54353568
UIJ66128.1	redox-sensing_transcriptional_repressor_Rex_[Bacillus_cereus]	CPTF_zcontrol	386461.6667	45353.76053	11.73564274
UIJ66129.1	ATP-binding_cassette_domain-containing_protein_[Bacillus_cereus]	CPTF_Al	207772	219258.3784	105.5283572
UIJ66129.1	ATP-binding_cassette_domain-containing_protein_[Bacillus_cereus]	CPTF_Cd	31021.63333	29615.01345	95.46568078
UIJ66129.1	ATP-binding_cassette_domain-containing_protein_[Bacillus_cereus]	CPTF_Co	31015.16667	26951.40579	86.89750431
UIJ66129.1	ATP-binding_cassette_domain-containing_protein_[Bacillus_cereus]	CPTF_Cu	40713.63333	19248.96784	47.27892421
UIJ66129.1	ATP-binding_cassette_domain-containing_protein_[Bacillus_cereus]	CPTF_Fe	15219.73333	26361.35141	173.2050808
UIJ66129.1	ATP-binding_cassette_domain-containing_protein_[Bacillus_cereus]	CPTF_Mn	199558.8667	305239.6131	152.9571791
UIJ66129.1	ATP-binding_cassette_domain-containing_protein_[Bacillus_cereus]	CPTF_Ni	445354.3	391752.3263	87.96419532
UIJ66129.1	ATP-binding_cassette_domain-containing_protein_[Bacillus_cereus]	CPTF_U	741206.6667	417285.3101	56.29810536
UIJ66129.1	ATP-binding_cassette_domain-containing_protein_[Bacillus_cereus]	CPTF_metals_mix	55868	34556.95874	61.85465514
UIJ66129.1	ATP-binding_cassette_domain-containing_protein_[Bacillus_cereus]	CPTF_zcontrol	152001.3	218375.5742	143.6669122
UIJ66132.1	tRNA_(adenosine(37)-N6)-threonylcarbamoyltransferase_complex_dimerization_subunit_type_1_TsaB_[Bacillus_cereus]	CPTF_Al	15477.13333	26807.18129	173.2050808
UIJ66132.1	tRNA_(adenosine(37)-N6)-threonylcarbamoyltransferase_complex_dimerization_subunit_type_1_TsaB_[Bacillus_cereus]	CPTF_Cd	16104.4	27893.63903	173.2050808
UIJ66132.1	tRNA_(adenosine(37)-N6)-threonylcarbamoyltransferase_complex_dimerization_subunit_type_1_TsaB_[Bacillus_cereus]	CPTF_Co	0	0	0
UIJ66132.1	tRNA_(adenosine(37)-N6)-threonylcarbamoyltransferase_complex_dimerization_subunit_type_1_TsaB_[Bacillus_cereus]	CPTF_Cu	0	0	0
UIJ66132.1	tRNA_(adenosine(37)-N6)-threonylcarbamoyltransferase_complex_dimerization_subunit_type_1_TsaB_[Bacillus_cereus]	CPTF_Fe	0	0	0
UIJ66132.1	tRNA_(adenosine(37)-N6)-threonylcarbamoyltransferase_complex_dimerization_subunit_type_1_TsaB_[Bacillus_cereus]	CPTF_Mn	0	0	0
UIJ66132.1	tRNA_(adenosine(37)-N6)-threonylcarbamoyltransferase_complex_dimerization_subunit_type_1_TsaB_[Bacillus_cereus]	CPTF_Ni	0	0	0
UIJ66132.1	tRNA_(adenosine(37)-N6)-threonylcarbamoyltransferase_complex_dimerization_subunit_type_1_TsaB_[Bacillus_cereus]	CPTF_U	29643.23333	29781.29697	100.4657509

UIJ66132.1	tRNA_(adenosine(37)-N6)-threonylcarbamoyltransferase_complex_dimerization_subunit_type_1_TsaB_[Bacillus_cereus]	CPTF_metals_mix	194734.5667	14683.17078	7.540094719
UIJ66132.1	tRNA_(adenosine(37)-N6)-threonylcarbamoyltransferase_complex_dimerization_subunit_type_1_TsaB_[Bacillus_cereus]	CPTF_zcontrol	0	0	0
UIJ66133.1	tRNA_(adenosine(37)-N6)-threonylcarbamoyltransferase_complex_ATPase_subunit_type_1_TsaE_[Bacillus_cereus]	CPTF_Al	8292.966667	14363.83961	173.2050808
UIJ66133.1	tRNA_(adenosine(37)-N6)-threonylcarbamoyltransferase_complex_ATPase_subunit_type_1_TsaE_[Bacillus_cereus]	CPTF_Cd	0	0	0
UIJ66133.1	tRNA_(adenosine(37)-N6)-threonylcarbamoyltransferase_complex_ATPase_subunit_type_1_TsaE_[Bacillus_cereus]	CPTF_Co	43471.8	52811.32042	121.4840895
UIJ66133.1	tRNA_(adenosine(37)-N6)-threonylcarbamoyltransferase_complex_ATPase_subunit_type_1_TsaE_[Bacillus_cereus]	CPTF_Cu	13896.56667	12058.82553	86.77557428
UIJ66133.1	tRNA_(adenosine(37)-N6)-threonylcarbamoyltransferase_complex_ATPase_subunit_type_1_TsaE_[Bacillus_cereus]	CPTF_Fe	19585.93333	33923.83165	173.2050808
UIJ66133.1	tRNA_(adenosine(37)-N6)-threonylcarbamoyltransferase_complex_ATPase_subunit_type_1_TsaE_[Bacillus_cereus]	CPTF_Mn	22620.23333	39179.39341	173.2050808
UIJ66133.1	tRNA_(adenosine(37)-N6)-threonylcarbamoyltransferase_complex_ATPase_subunit_type_1_TsaE_[Bacillus_cereus]	CPTF_Ni	34194.66667	30301.07007	88.61343894
UIJ66133.1	tRNA_(adenosine(37)-N6)-threonylcarbamoyltransferase_complex_ATPase_subunit_type_1_TsaE_[Bacillus_cereus]	CPTF_U	0	0	0
UIJ66133.1	tRNA_(adenosine(37)-N6)-threonylcarbamoyltransferase_complex_ATPase_subunit_type_1_TsaE_[Bacillus_cereus]	CPTF_metals_mix	9920.433333	17182.69457	173.2050808
UIJ66133.1	tRNA_(adenosine(37)-N6)-threonylcarbamoyltransferase_complex_ATPase_subunit_type_1_TsaE_[Bacillus_cereus]	CPTF_zcontrol	17855.83333	30927.21054	173.2050808
UIJ66136.1	RNA-binding_transcriptional_accessory_protein_[Bacillus_cereus]	CPTF_Al	642841.8667	347465.503	54.05147378
UIJ66136.1	RNA-binding_transcriptional_accessory_protein_[Bacillus_cereus]	CPTF_Cd	597301.0667	194168.5048	32.50764407
UIJ66136.1	RNA-binding_transcriptional_accessory_protein_[Bacillus_cereus]	CPTF_Co	894327.6667	70859.30138	7.923192362
UIJ66136.1	RNA-binding_transcriptional_accessory_protein_[Bacillus_cereus]	CPTF_Cu	755304	259743.9035	34.3893192
UIJ66136.1	RNA-binding_transcriptional_accessory_protein_[Bacillus_cereus]	CPTF_Fe	527451.3	235815.5419	44.70849572
UIJ66136.1	RNA-binding_transcriptional_accessory_protein_[Bacillus_cereus]	CPTF_Mn	664986.2333	458361.2567	68.9279317
UIJ66136.1	RNA-binding_transcriptional_accessory_protein_[Bacillus_cereus]	CPTF_Ni	474223.8	76526.91697	16.13729993
UIJ66136.1	RNA-binding_transcriptional_accessory_protein_[Bacillus_cereus]	CPTF_U	455423.4	523548.3028	114.9585864
UIJ66136.1	RNA-binding_transcriptional_accessory_protein_[Bacillus_cereus]	CPTF_metals_mix	1818728.333	83375.20539	4.584258345
UIJ66136.1	RNA-binding_transcriptional_accessory_protein_[Bacillus_cereus]	CPTF_zcontrol	474173.5	177232.7772	37.37719994
UIJ66138.1	type_II_toxin-antitoxin_system_endoribonuclease_NdoA_[Bacillus_cereus]	CPTF_Al	0	0	0
UIJ66138.1	type_II_toxin-antitoxin_system_endoribonuclease_NdoA_[Bacillus_cereus]	CPTF_Cd	0	0	0
UIJ66138.1	type_II_toxin-antitoxin_system_endoribonuclease_NdoA_[Bacillus_cereus]	CPTF_Co	0	0	0
UIJ66138.1	type_II_toxin-antitoxin_system_endoribonuclease_NdoA_[Bacillus_cereus]	CPTF_Cu	0	0	0
UIJ66138.1	type_II_toxin-antitoxin_system_endoribonuclease_NdoA_[Bacillus_cereus]	CPTF_Fe	0	0	0
UIJ66138.1	type_II_toxin-antitoxin_system_endoribonuclease_NdoA_[Bacillus_cereus]	CPTF_Mn	0	0	0
UIJ66138.1	type_II_toxin-antitoxin_system_endoribonuclease_NdoA_[Bacillus_cereus]	CPTF_Ni	0	0	0
UIJ66138.1	type_II_toxin-antitoxin_system_endoribonuclease_NdoA_[Bacillus_cereus]	CPTF_U	0	0	0
UIJ66138.1	type_II_toxin-antitoxin_system_endoribonuclease_NdoA_[Bacillus_cereus]	CPTF_metals_mix	5257.3	9105.910711	173.2050808
UIJ66138.1	type_II_toxin-antitoxin_system_endoribonuclease_NdoA_[Bacillus_cereus]	CPTF_zcontrol	0	0	0
UIJ66146.1	DEAD/DEAH_box_helicase_[Bacillus_cereus]	CPTF_Al	5711002.733	1011037.944	17.70333497
UIJ66146.1	DEAD/DEAH_box_helicase_[Bacillus_cereus]	CPTF_Cd	6747243.033	595844.4149	8.830931566
UIJ66146.1	DEAD/DEAH_box_helicase_[Bacillus_cereus]	CPTF_Co	5914275.533	179715.7056	3.038676581
UIJ66146.1	DEAD/DEAH_box_helicase_[Bacillus_cereus]	CPTF_Cu	5512834.8	420765.5936	7.632472382
UIJ66146.1	DEAD/DEAH_box_helicase_[Bacillus_cereus]	CPTF_Fe	5741917.633	642812.5622	11.19508504
UIJ66146.1	DEAD/DEAH_box_helicase_[Bacillus_cereus]	CPTF_Mn	5745846.9	915516.3092	15.93353121
UIJ66146.1	DEAD/DEAH_box_helicase_[Bacillus_cereus]	CPTF_Ni	3813598.867	380134.7909	9.9678756
UIJ66146.1	DEAD/DEAH_box_helicase_[Bacillus_cereus]	CPTF_U	4298290.033	697186.7038	16.22009446
UIJ66146.1	DEAD/DEAH_box_helicase_[Bacillus_cereus]	CPTF_metals_mix	6231088.367	389921.5355	6.257679439
UIJ66146.1	DEAD/DEAH_box_helicase_[Bacillus_cereus]	CPTF_zcontrol	5319752.9	921833.5481	17.32850314
UIJ66147.1	UDP-N-acetylmuramoyl-tripeptide--D-alanyl-D-alanine_ligase_[Bacillus_cereus]	CPTF_Al	162340.2667	90237.81894	55.58560472
UIJ66147.1	UDP-N-acetylmuramoyl-tripeptide--D-alanyl-D-alanine_ligase_[Bacillus_cereus]	CPTF_Cd	220012.6667	22882.83475	10.4006897
UIJ66147.1	UDP-N-acetylmuramoyl-tripeptide--D-alanyl-D-alanine_ligase_[Bacillus_cereus]	CPTF_Co	262181	26526.33976	10.11756754
UIJ66147.1	UDP-N-acetylmuramoyl-tripeptide--D-alanyl-D-alanine_ligase_[Bacillus_cereus]	CPTF_Cu	285281	101414.3874	35.54894556
UIJ66147.1	UDP-N-acetylmuramoyl-tripeptide--D-alanyl-D-alanine_ligase_[Bacillus_cereus]	CPTF_Fe	231993	60659.9297	26.14731035
UIJ66147.1	UDP-N-acetylmuramoyl-tripeptide--D-alanyl-D-alanine_ligase_[Bacillus_cereus]	CPTF_Mn	143054.5667	130418.584	91.16701903
UIJ66147.1	UDP-N-acetylmuramoyl-tripeptide--D-alanyl-D-alanine_ligase_[Bacillus_cereus]	CPTF_Ni	277643.6667	110919.5348	39.95032055
UIJ66147.1	UDP-N-acetylmuramoyl-tripeptide--D-alanyl-D-alanine_ligase_[Bacillus_cereus]	CPTF_U	310186.6667	64895.40767	20.92140464
UIJ66147.1	UDP-N-acetylmuramoyl-tripeptide--D-alanyl-D-alanine_ligase_[Bacillus_cereus]	CPTF_metals_mix	201680.9	57420.41017	28.47092123
UIJ66147.1	UDP-N-acetylmuramoyl-tripeptide--D-alanyl-D-alanine_ligase_[Bacillus_cereus]	CPTF_zcontrol	298246.9667	108070.0672	36.23509349
UIJ66148.1	D-alanine--D-alanine_ligase_[Bacillus_cereus]	CPTF_Al	846812.0667	68398.30906	8.077153332
UIJ66148.1	D-alanine--D-alanine_ligase_[Bacillus_cereus]	CPTF_Cd	803908	84571.65859	10.5200668
UIJ66148.1	D-alanine--D-alanine_ligase_[Bacillus_cereus]	CPTF_Co	814699.9333	62160.65353	7.629883223
UIJ66148.1	D-alanine--D-alanine_ligase_[Bacillus_cereus]	CPTF_Cu	833398.8	191897.0142	23.0258328
UIJ66148.1	D-alanine--D-alanine_ligase_[Bacillus_cereus]	CPTF_Fe	806961.7667	58589.41999	7.260495157
UIJ66148.1	D-alanine--D-alanine_ligase_[Bacillus_cereus]	CPTF_Mn	837510.1667	220712.5354	26.35341566
UIJ66148.1	D-alanine--D-alanine_ligase_[Bacillus_cereus]	CPTF_Ni	785851.9	49940.52434	6.354953693
UIJ66148.1	D-alanine--D-alanine_ligase_[Bacillus_cereus]	CPTF_U	1033136.533	162555.5221	15.73417616
UIJ66148.1	D-alanine--D-alanine_ligase_[Bacillus_cereus]	CPTF_metals_mix	1683172.967	215826.1744	12.82257847

UIJ66148.1	D-alanine--D-alanine_ligase_[Bacillus_cereus]	CPTF_zcontrol	778800.2667	98055.82375	12.59062534
UIJ66150.1	homogentisate_1,2-dioxygenase_[Bacillus_cereus]	CPTF_Al	362453.4667	136085.8365	37.54574009
UIJ66150.1	homogentisate_1,2-dioxygenase_[Bacillus_cereus]	CPTF_Cd	438598.2333	44182.02329	10.0734613
UIJ66150.1	homogentisate_1,2-dioxygenase_[Bacillus_cereus]	CPTF_Co	333823.2667	79004.49415	23.66656313
UIJ66150.1	homogentisate_1,2-dioxygenase_[Bacillus_cereus]	CPTF_Cu	209354.1667	29214.44262	13.95455514
UIJ66150.1	homogentisate_1,2-dioxygenase_[Bacillus_cereus]	CPTF_Fe	464740.8	22586.5977	4.860041921
UIJ66150.1	homogentisate_1,2-dioxygenase_[Bacillus_cereus]	CPTF_Mn	271503.3333	138286.3438	50.93357127
UIJ66150.1	homogentisate_1,2-dioxygenase_[Bacillus_cereus]	CPTF_Ni	393332.4	159485.118	40.54716011
UIJ66150.1	homogentisate_1,2-dioxygenase_[Bacillus_cereus]	CPTF_U	317094.5667	136430.6983	43.02523998
UIJ66150.1	homogentisate_1,2-dioxygenase_[Bacillus_cereus]	CPTF_metals_mix	95264.73333	91907.23076	96.47560808
UIJ66150.1	homogentisate_1,2-dioxygenase_[Bacillus_cereus]	CPTF_zcontrol	310322.7333	65598.35472	21.13875255
UIJ66151.1	fumarylacetoacetate_hydrolase_family_protein_[Bacillus_cereus]	CPTF_Al	346536.3333	38767.32048	11.18708682
UIJ66151.1	fumarylacetoacetate_hydrolase_family_protein_[Bacillus_cereus]	CPTF_Cd	481663.3333	7030.373414	1.459603197
UIJ66151.1	fumarylacetoacetate_hydrolase_family_protein_[Bacillus_cereus]	CPTF_Co	292643.6667	39694.31856	13.56404498
UIJ66151.1	fumarylacetoacetate_hydrolase_family_protein_[Bacillus_cereus]	CPTF_Cu	254654.3333	15222.90959	5.977871803
UIJ66151.1	fumarylacetoacetate_hydrolase_family_protein_[Bacillus_cereus]	CPTF_Fe	290068.6667	51479.2653	17.74726857
UIJ66151.1	fumarylacetoacetate_hydrolase_family_protein_[Bacillus_cereus]	CPTF_Mn	342681.6667	28757.70301	8.391958428
UIJ66151.1	fumarylacetoacetate_hydrolase_family_protein_[Bacillus_cereus]	CPTF_Ni	243877	23533.41546	9.649706801
UIJ66151.1	fumarylacetoacetate_hydrolase_family_protein_[Bacillus_cereus]	CPTF_U	288337.6667	25623.82712	8.8867429
UIJ66151.1	fumarylacetoacetate_hydrolase_family_protein_[Bacillus_cereus]	CPTF_metals_mix	140746.6667	123652.1659	87.85441875
UIJ66151.1	fumarylacetoacetate_hydrolase_family_protein_[Bacillus_cereus]	CPTF_zcontrol	327336.3333	28054.84682	8.570648586
UIJ66152.1	4-hydroxyphenylpyruvate_dioxygenase_[Bacillus_cereus]	CPTF_Al	7208559.167	831598.6393	11.5362671
UIJ66152.1	4-hydroxyphenylpyruvate_dioxygenase_[Bacillus_cereus]	CPTF_Cd	8499086.3	705296.7129	8.298500427
UIJ66152.1	4-hydroxyphenylpyruvate_dioxygenase_[Bacillus_cereus]	CPTF_Co	6326920.267	562998.4488	8.898459678
UIJ66152.1	4-hydroxyphenylpyruvate_dioxygenase_[Bacillus_cereus]	CPTF_Cu	5134521.267	383896.8488	7.476779799
UIJ66152.1	4-hydroxyphenylpyruvate_dioxygenase_[Bacillus_cereus]	CPTF_Fe	7383599.1	507995.2998	6.880049864
UIJ66152.1	4-hydroxyphenylpyruvate_dioxygenase_[Bacillus_cereus]	CPTF_Mn	7370876.4	297068.8702	4.030305951
UIJ66152.1	4-hydroxyphenylpyruvate_dioxygenase_[Bacillus_cereus]	CPTF_Ni	7220006.333	934059.6008	12.93710224
UIJ66152.1	4-hydroxyphenylpyruvate_dioxygenase_[Bacillus_cereus]	CPTF_U	6248141	1488113.367	23.81689797
UIJ66152.1	4-hydroxyphenylpyruvate_dioxygenase_[Bacillus_cereus]	CPTF_metals_mix	3488437	394444.2035	11.30719011
UIJ66152.1	4-hydroxyphenylpyruvate_dioxygenase_[Bacillus_cereus]	CPTF_zcontrol	6442272.667	259204.6797	4.0234975
UIJ66163.1	ABC_transporter_substrate-binding_protein_[Bacillus_cereus]	CPTF_Al	445091.7667	88860.68934	19.96457719
UIJ66163.1	ABC_transporter_substrate-binding_protein_[Bacillus_cereus]	CPTF_Cd	574143.9667	271296.6954	47.25238114
UIJ66163.1	ABC_transporter_substrate-binding_protein_[Bacillus_cereus]	CPTF_Co	721391.5333	696376.7519	96.53242653
UIJ66163.1	ABC_transporter_substrate-binding_protein_[Bacillus_cereus]	CPTF_Cu	78869.06667	89701.93511	113.7352563
UIJ66163.1	ABC_transporter_substrate-binding_protein_[Bacillus_cereus]	CPTF_Fe	559684	264674.8986	47.29005986
UIJ66163.1	ABC_transporter_substrate-binding_protein_[Bacillus_cereus]	CPTF_Mn	1083128.467	384183.033	35.4697568
UIJ66163.1	ABC_transporter_substrate-binding_protein_[Bacillus_cereus]	CPTF_Ni	904567.1333	957376.9982	105.8381366
UIJ66163.1	ABC_transporter_substrate-binding_protein_[Bacillus_cereus]	CPTF_U	632436.1667	981907.245	155.2579212
UIJ66163.1	ABC_transporter_substrate-binding_protein_[Bacillus_cereus]	CPTF_metals_mix	59122.36667	36675.02912	62.03241038
UIJ66163.1	ABC_transporter_substrate-binding_protein_[Bacillus_cereus]	CPTF_zcontrol	991625.1667	807708.32	81.45298719
UIJ66176.1	1-acyl-sn-glycerol-3-phosphate_acyltransferase_[Bacillus_cereus]	CPTF_Al	134475.7667	41509.4951	30.86763967
UIJ66176.1	1-acyl-sn-glycerol-3-phosphate_acyltransferase_[Bacillus_cereus]	CPTF_Cd	116842.3333	112310.9958	96.12183579
UIJ66176.1	1-acyl-sn-glycerol-3-phosphate_acyltransferase_[Bacillus_cereus]	CPTF_Co	109285	105902.8624	96.90521333
UIJ66176.1	1-acyl-sn-glycerol-3-phosphate_acyltransferase_[Bacillus_cereus]	CPTF_Cu	56238.66667	97408.22802	173.2050808
UIJ66176.1	1-acyl-sn-glycerol-3-phosphate_acyltransferase_[Bacillus_cereus]	CPTF_Fe	135711.8667	72815.92365	53.65479485
UIJ66176.1	1-acyl-sn-glycerol-3-phosphate_acyltransferase_[Bacillus_cereus]	CPTF_Mn	108725.6667	94162.20423	86.60531328
UIJ66176.1	1-acyl-sn-glycerol-3-phosphate_acyltransferase_[Bacillus_cereus]	CPTF_Ni	85374.66667	74805.45906	87.6202063
UIJ66176.1	1-acyl-sn-glycerol-3-phosphate_acyltransferase_[Bacillus_cereus]	CPTF_U	125024.6667	108588.6157	86.85375344
UIJ66176.1	1-acyl-sn-glycerol-3-phosphate_acyltransferase_[Bacillus_cereus]	CPTF_metals_mix	243368	74557.69797	30.6357853
UIJ66176.1	1-acyl-sn-glycerol-3-phosphate_acyltransferase_[Bacillus_cereus]	CPTF_zcontrol	133326.3333	28407.65052	21.3068565
UIJ66187.1	aldo/keto_reductase_[Bacillus_cereus]	CPTF_Al	611580.8	25933.41056	4.240389915
UIJ66187.1	aldo/keto_reductase_[Bacillus_cereus]	CPTF_Cd	546455.4333	41760.49161	7.642067233
UIJ66187.1	aldo/keto_reductase_[Bacillus_cereus]	CPTF_Co	630072.2333	33211.05867	5.270992263
UIJ66187.1	aldo/keto_reductase_[Bacillus_cereus]	CPTF_Cu	588270.2333	185598.7356	31.5499111
UIJ66187.1	aldo/keto_reductase_[Bacillus_cereus]	CPTF_Fe	416101.6667	96467.04106	23.18352672
UIJ66187.1	aldo/keto_reductase_[Bacillus_cereus]	CPTF_Mn	462691	23920.47252	5.169859047
UIJ66187.1	aldo/keto_reductase_[Bacillus_cereus]	CPTF_Ni	513685.3667	104535.5786	20.35011806
UIJ66187.1	aldo/keto_reductase_[Bacillus_cereus]	CPTF_U	401509.7333	175187.7023	43.63224296
UIJ66187.1	aldo/keto_reductase_[Bacillus_cereus]	CPTF_metals_mix	1373567.167	236434.4907	17.21317286
UIJ66187.1	aldo/keto_reductase_[Bacillus_cereus]	CPTF_zcontrol	536041.6667	79467.05124	14.82478997

UIJ66189.1	peptide_ABC_transporter_substrate-binding_protein_[Bacillus_cereus]	CPTF_Al	0	0	0
UIJ66189.1	peptide_ABC_transporter_substrate-binding_protein_[Bacillus_cereus]	CPTF_Cd	22682.36667	39287.0115	173.2050808
UIJ66189.1	peptide_ABC_transporter_substrate-binding_protein_[Bacillus_cereus]	CPTF_Co	11377.46667	19706.35033	173.2050808
UIJ66189.1	peptide_ABC_transporter_substrate-binding_protein_[Bacillus_cereus]	CPTF_Cu	13010.86667	22535.48212	173.2050808
UIJ66189.1	peptide_ABC_transporter_substrate-binding_protein_[Bacillus_cereus]	CPTF_Fe	0	0	0
UIJ66189.1	peptide_ABC_transporter_substrate-binding_protein_[Bacillus_cereus]	CPTF_Mn	0	0	0
UIJ66189.1	peptide_ABC_transporter_substrate-binding_protein_[Bacillus_cereus]	CPTF_Ni	0	0	0
UIJ66189.1	peptide_ABC_transporter_substrate-binding_protein_[Bacillus_cereus]	CPTF_U	0	0	0
UIJ66189.1	peptide_ABC_transporter_substrate-binding_protein_[Bacillus_cereus]	CPTF_metals_mix	34170.63333	35318.90691	103.3604106
UIJ66189.1	peptide_ABC_transporter_substrate-binding_protein_[Bacillus_cereus]	CPTF_zcontrol	0	0	0
UIJ66194.1	peptide_ABC_transporter_substrate-binding_protein_[Bacillus_cereus]	CPTF_Al	211095	189265.1191	89.65874089
UIJ66194.1	peptide_ABC_transporter_substrate-binding_protein_[Bacillus_cereus]	CPTF_Cd	0	0	0
UIJ66194.1	peptide_ABC_transporter_substrate-binding_protein_[Bacillus_cereus]	CPTF_Co	274584.9	67167.332	24.46140775
UIJ66194.1	peptide_ABC_transporter_substrate-binding_protein_[Bacillus_cereus]	CPTF_Cu	134599.3333	125978.8228	93.59542852
UIJ66194.1	peptide_ABC_transporter_substrate-binding_protein_[Bacillus_cereus]	CPTF_Fe	0	0	0
UIJ66194.1	peptide_ABC_transporter_substrate-binding_protein_[Bacillus_cereus]	CPTF_Mn	38465.33333	66623.91166	173.2050808
UIJ66194.1	peptide_ABC_transporter_substrate-binding_protein_[Bacillus_cereus]	CPTF_Ni	0	0	0
UIJ66194.1	peptide_ABC_transporter_substrate-binding_protein_[Bacillus_cereus]	CPTF_U	69976.7	121203.1997	173.2050808
UIJ66194.1	peptide_ABC_transporter_substrate-binding_protein_[Bacillus_cereus]	CPTF_metals_mix	0	0	0
UIJ66194.1	peptide_ABC_transporter_substrate-binding_protein_[Bacillus_cereus]	CPTF_zcontrol	34642	60001.70408	173.2050808
UIJ66202.1	hypothetical_protein_LW858_25760_[Bacillus_cereus]	CPTF_Al	1549963.133	284699.1384	18.36812323
UIJ66202.1	hypothetical_protein_LW858_25760_[Bacillus_cereus]	CPTF_Cd	2370823.5	55199.71506	2.328292893
UIJ66202.1	hypothetical_protein_LW858_25760_[Bacillus_cereus]	CPTF_Co	1692280.4	398366.1636	23.5401984
UIJ66202.1	hypothetical_protein_LW858_25760_[Bacillus_cereus]	CPTF_Cu	1089505.367	48440.95949	4.446142348
UIJ66202.1	hypothetical_protein_LW858_25760_[Bacillus_cereus]	CPTF_Fe	1579934.833	180086.181	11.39832968
UIJ66202.1	hypothetical_protein_LW858_25760_[Bacillus_cereus]	CPTF_Mn	1867215.667	485757.0428	26.01504751
UIJ66202.1	hypothetical_protein_LW858_25760_[Bacillus_cereus]	CPTF_Ni	1407285.667	49587.60565	3.52363467
UIJ66202.1	hypothetical_protein_LW858_25760_[Bacillus_cereus]	CPTF_U	1173538.667	17016.08658	1.449980905
UIJ66202.1	hypothetical_protein_LW858_25760_[Bacillus_cereus]	CPTF_metals_mix	1868325.467	226969.217	12.14826972
UIJ66202.1	hypothetical_protein_LW858_25760_[Bacillus_cereus]	CPTF_zcontrol	1226280.867	41790.69913	3.407922301
UIJ66204.1	MetQ/NlpA_family_ABC_transporter_substrate-binding_protein_[Bacillus_cereus]	CPTF_Al	355645.9333	51774.42089	14.55785545
UIJ66204.1	MetQ/NlpA_family_ABC_transporter_substrate-binding_protein_[Bacillus_cereus]	CPTF_Cd	348375.6667	36422.5556	10.45496545
UIJ66204.1	MetQ/NlpA_family_ABC_transporter_substrate-binding_protein_[Bacillus_cereus]	CPTF_Co	333104	79912.76199	23.99033395
UIJ66204.1	MetQ/NlpA_family_ABC_transporter_substrate-binding_protein_[Bacillus_cereus]	CPTF_Cu	247246	90417.88909	36.57001088
UIJ66204.1	MetQ/NlpA_family_ABC_transporter_substrate-binding_protein_[Bacillus_cereus]	CPTF_Fe	293152	26494.24792	9.037716926
UIJ66204.1	MetQ/NlpA_family_ABC_transporter_substrate-binding_protein_[Bacillus_cereus]	CPTF_Mn	271961	70963.54269	26.09327907
UIJ66204.1	MetQ/NlpA_family_ABC_transporter_substrate-binding_protein_[Bacillus_cereus]	CPTF_Ni	313730.6667	81338.33009	25.92616494
UIJ66204.1	MetQ/NlpA_family_ABC_transporter_substrate-binding_protein_[Bacillus_cereus]	CPTF_U	358172.3333	87595.6741	24.45629267
UIJ66204.1	MetQ/NlpA_family_ABC_transporter_substrate-binding_protein_[Bacillus_cereus]	CPTF_metals_mix	50493.83333	45465.53208	90.04175179
UIJ66204.1	MetQ/NlpA_family_ABC_transporter_substrate-binding_protein_[Bacillus_cereus]	CPTF_zcontrol	314961	61022.72281	19.37469173
UIJ66211.1	DUF4256_domain-containing_protein_[Bacillus_cereus]	CPTF_Al	249163.1667	52886.32703	21.2255799
UIJ66211.1	DUF4256_domain-containing_protein_[Bacillus_cereus]	CPTF_Cd	363761.7333	176406.2079	48.4949877
UIJ66211.1	DUF4256_domain-containing_protein_[Bacillus_cereus]	CPTF_Co	327156.3333	86135.095	26.32842046
UIJ66211.1	DUF4256_domain-containing_protein_[Bacillus_cereus]	CPTF_Cu	236466.2333	92329.99719	39.04574276
UIJ66211.1	DUF4256_domain-containing_protein_[Bacillus_cereus]	CPTF_Fe	373947.1333	52772.09925	14.11218179
UIJ66211.1	DUF4256_domain-containing_protein_[Bacillus_cereus]	CPTF_Mn	448952.2667	190063.9516	42.33500211
UIJ66211.1	DUF4256_domain-containing_protein_[Bacillus_cereus]	CPTF_Ni	301221.1333	83121.45568	27.59482868
UIJ66211.1	DUF4256_domain-containing_protein_[Bacillus_cereus]	CPTF_U	287810.5667	199928.1204	69.4651773
UIJ66211.1	DUF4256_domain-containing_protein_[Bacillus_cereus]	CPTF_metals_mix	189993.1	129127.52	67.96432082
UIJ66211.1	DUF4256_domain-containing_protein_[Bacillus_cereus]	CPTF_zcontrol	260293.3667	32772.81231	12.59072128
UIJ66215.1	glutamine--fructose-6-phosphate_transaminase_(isomerizing)_[Bacillus_cereus]	CPTF_Al	8564919.167	605420.7219	7.068609873
UIJ66215.1	glutamine--fructose-6-phosphate_transaminase_(isomerizing)_[Bacillus_cereus]	CPTF_Cd	9613039.4	84600.46409	0.880059475
UIJ66215.1	glutamine--fructose-6-phosphate_transaminase_(isomerizing)_[Bacillus_cereus]	CPTF_Co	9811335.067	709891.0864	7.235417827
UIJ66215.1	glutamine--fructose-6-phosphate_transaminase_(isomerizing)_[Bacillus_cereus]	CPTF_Cu	8424587.633	432829.9327	5.13769874
UIJ66215.1	glutamine--fructose-6-phosphate_transaminase_(isomerizing)_[Bacillus_cereus]	CPTF_Fe	8300465.6	1129618.151	13.60909381
UIJ66215.1	glutamine--fructose-6-phosphate_transaminase_(isomerizing)_[Bacillus_cereus]	CPTF_Mn	8595454.933	1733372.602	20.1661531
UIJ66215.1	glutamine--fructose-6-phosphate_transaminase_(isomerizing)_[Bacillus_cereus]	CPTF_Ni	6748338.667	1377386.813	20.41075412
UIJ66215.1	glutamine--fructose-6-phosphate_transaminase_(isomerizing)_[Bacillus_cereus]	CPTF_U	6962403	640530.1296	9.199842778
UIJ66215.1	glutamine--fructose-6-phosphate_transaminase_(isomerizing)_[Bacillus_cereus]	CPTF_metals_mix	9280315.333	300255.5043	3.235401961
UIJ66215.1	glutamine--fructose-6-phosphate_transaminase_(isomerizing)_[Bacillus_cereus]	CPTF_zcontrol	8594483.2	690107.9101	8.029661518
UIJ66217.1	phosphoglucosamine_mutase_[Bacillus_cereus]	CPTF_Al	5387832.567	193363.0225	3.588883287

UIJ66217.1	phosphoglucosamine_mutase_[Bacillus_cereus]	CPTF_Cd	5512565.867	253484.5109	4.598303531
UIJ66217.1	phosphoglucosamine_mutase_[Bacillus_cereus]	CPTF_Co	5568207.533	256787.6696	4.611675626
UIJ66217.1	phosphoglucosamine_mutase_[Bacillus_cereus]	CPTF_Cu	5888148.633	261007.1751	4.432754528
UIJ66217.1	phosphoglucosamine_mutase_[Bacillus_cereus]	CPTF_Fe	5332108.333	387687.4532	7.270809762
UIJ66217.1	phosphoglucosamine_mutase_[Bacillus_cereus]	CPTF_Mn	5573031.433	470896.6363	8.449560027
UIJ66217.1	phosphoglucosamine_mutase_[Bacillus_cereus]	CPTF_Ni	4355806.667	1112653.321	25.54413927
UIJ66217.1	phosphoglucosamine_mutase_[Bacillus_cereus]	CPTF_U	4677105	1064698.024	22.76403937
UIJ66217.1	phosphoglucosamine_mutase_[Bacillus_cereus]	CPTF_metals_mix	9615527.5	1114351.548	11.58908388
UIJ66217.1	phosphoglucosamine_mutase_[Bacillus_cereus]	CPTF_zcontrol	5372271.667	178909.6144	3.330241386
UIJ66218.1	YbbR-like_domain-containing_protein_[Bacillus_cereus]	CPTF_Al	50409.83333	49080.80843	97.36356022
UIJ66218.1	YbbR-like_domain-containing_protein_[Bacillus_cereus]	CPTF_Cd	184978.3333	20463.61807	11.0627108
UIJ66218.1	YbbR-like_domain-containing_protein_[Bacillus_cereus]	CPTF_Co	48980.56667	49347.04414	100.74821
UIJ66218.1	YbbR-like_domain-containing_protein_[Bacillus_cereus]	CPTF_Cu	54589	94550.92153	173.2050808
UIJ66218.1	YbbR-like_domain-containing_protein_[Bacillus_cereus]	CPTF_Fe	0	0	0
UIJ66218.1	YbbR-like_domain-containing_protein_[Bacillus_cereus]	CPTF_Mn	44158	76483.89956	173.2050808
UIJ66218.1	YbbR-like_domain-containing_protein_[Bacillus_cereus]	CPTF_Ni	0	0	0
UIJ66218.1	YbbR-like_domain-containing_protein_[Bacillus_cereus]	CPTF_U	0	0	0
UIJ66218.1	YbbR-like_domain-containing_protein_[Bacillus_cereus]	CPTF_metals_mix	146888.3333	127215.5739	86.60699663
UIJ66218.1	YbbR-like_domain-containing_protein_[Bacillus_cereus]	CPTF_zcontrol	0	0	0
UIJ66220.1	arginase_[Bacillus_cereus]	CPTF_Al	17466.33333	30252.57676	173.2050808
UIJ66220.1	arginase_[Bacillus_cereus]	CPTF_Cd	30070.26667	26053.69205	86.64270369
UIJ66220.1	arginase_[Bacillus_cereus]	CPTF_Co	52174.83333	25423.98901	48.72845276
UIJ66220.1	arginase_[Bacillus_cereus]	CPTF_Cu	41040.9	11092.43965	27.02776901
UIJ66220.1	arginase_[Bacillus_cereus]	CPTF_Fe	16610.7	28770.57635	173.2050808
UIJ66220.1	arginase_[Bacillus_cereus]	CPTF_Mn	12683.33333	21968.17774	173.2050808
UIJ66220.1	arginase_[Bacillus_cereus]	CPTF_Ni	11381.3	19712.98986	173.2050808
UIJ66220.1	arginase_[Bacillus_cereus]	CPTF_U	17569.53333	30431.3244	173.2050808
UIJ66220.1	arginase_[Bacillus_cereus]	CPTF_metals_mix	0	0	0
UIJ66220.1	arginase_[Bacillus_cereus]	CPTF_zcontrol	12167.9	21075.42102	173.2050808
UIJ66226.1	Mrp/NBP35_family_ATP-binding_protein_[Bacillus_cereus]	CPTF_Al	458414.3667	232373.4063	50.69069016
UIJ66226.1	Mrp/NBP35_family_ATP-binding_protein_[Bacillus_cereus]	CPTF_Cd	376241.4667	103403.923	27.48339355
UIJ66226.1	Mrp/NBP35_family_ATP-binding_protein_[Bacillus_cereus]	CPTF_Co	403735.1333	115768.5621	28.67438391
UIJ66226.1	Mrp/NBP35_family_ATP-binding_protein_[Bacillus_cereus]	CPTF_Cu	497104.9667	85192.20281	17.13766881
UIJ66226.1	Mrp/NBP35_family_ATP-binding_protein_[Bacillus_cereus]	CPTF_Fe	344175.3	37161.06275	10.79713238
UIJ66226.1	Mrp/NBP35_family_ATP-binding_protein_[Bacillus_cereus]	CPTF_Mn	407750.8	37107.2844	9.100481077
UIJ66226.1	Mrp/NBP35_family_ATP-binding_protein_[Bacillus_cereus]	CPTF_Ni	412227.1	90589.64383	21.97566434
UIJ66226.1	Mrp/NBP35_family_ATP-binding_protein_[Bacillus_cereus]	CPTF_U	266483.9667	93573.00744	35.11393522
UIJ66226.1	Mrp/NBP35_family_ATP-binding_protein_[Bacillus_cereus]	CPTF_metals_mix	810320.5667	190952.0225	23.56499765
UIJ66226.1	Mrp/NBP35_family_ATP-binding_protein_[Bacillus_cereus]	CPTF_zcontrol	406094.8	192518.557	47.4072943
UIJ66229.1	30S_ribosomal_protein_S9_[Bacillus_cereus]	CPTF_Al	2835771	593108.6215	20.91525097
UIJ66229.1	30S_ribosomal_protein_S9_[Bacillus_cereus]	CPTF_Cd	2567413	386040.231	15.03615628
UIJ66229.1	30S_ribosomal_protein_S9_[Bacillus_cereus]	CPTF_Co	3032361.667	39719.71028	1.309860585
UIJ66229.1	30S_ribosomal_protein_S9_[Bacillus_cereus]	CPTF_Cu	2921758	242713.4854	8.307104334
UIJ66229.1	30S_ribosomal_protein_S9_[Bacillus_cereus]	CPTF_Fe	2764091	189639.8136	6.860838287
UIJ66229.1	30S_ribosomal_protein_S9_[Bacillus_cereus]	CPTF_Mn	2589310.333	288697.2632	11.1495814
UIJ66229.1	30S_ribosomal_protein_S9_[Bacillus_cereus]	CPTF_Ni	2622399	291634.3833	11.12090049
UIJ66229.1	30S_ribosomal_protein_S9_[Bacillus_cereus]	CPTF_U	2470840.333	432431.3732	17.50138879
UIJ66229.1	30S_ribosomal_protein_S9_[Bacillus_cereus]	CPTF_metals_mix	2223920.667	415166.5794	18.66822795
UIJ66229.1	30S_ribosomal_protein_S9_[Bacillus_cereus]	CPTF_zcontrol	2236052.667	125050.2879	5.592457179
UIJ66230.1	50S_ribosomal_protein_L13_[Bacillus_cereus]	CPTF_Al	2787736.067	342476.5271	12.28511304
UIJ66230.1	50S_ribosomal_protein_L13_[Bacillus_cereus]	CPTF_Cd	2689342.433	472416.7823	17.56625621
UIJ66230.1	50S_ribosomal_protein_L13_[Bacillus_cereus]	CPTF_Co	3317380.367	90878.41724	2.739463287
UIJ66230.1	50S_ribosomal_protein_L13_[Bacillus_cereus]	CPTF_Cu	2953612.433	263762.6215	8.930170341
UIJ66230.1	50S_ribosomal_protein_L13_[Bacillus_cereus]	CPTF_Fe	2469849.633	238861.3175	9.671087435
UIJ66230.1	50S_ribosomal_protein_L13_[Bacillus_cereus]	CPTF_Mn	3017176.6	1147106.76	38.01921172
UIJ66230.1	50S_ribosomal_protein_L13_[Bacillus_cereus]	CPTF_Ni	1984560.767	510106.7854	25.70376246
UIJ66230.1	50S_ribosomal_protein_L13_[Bacillus_cereus]	CPTF_U	1745936.9	531411.1597	30.43701979
UIJ66230.1	50S_ribosomal_protein_L13_[Bacillus_cereus]	CPTF_metals_mix	4226031.733	539302.3544	12.76143646
UIJ66230.1	50S_ribosomal_protein_L13_[Bacillus_cereus]	CPTF_zcontrol	2638433.9	46496.66124	1.762282589
UIJ66235.1	50S_ribosomal_protein_L17_[Bacillus_cereus]	CPTF_Al	4561072.333	45556.74902	0.998816631
UIJ66235.1	50S_ribosomal_protein_L17_[Bacillus_cereus]	CPTF_Cd	4773612	79307.29234	1.661368631

UJJ66235.1	50S_ribosomal_protein_L17_[Bacillus_cereus]	CPTF_Co	5764407.333	507010.9369	8.795543195
UJJ66235.1	50S_ribosomal_protein_L17_[Bacillus_cereus]	CPTF_Cu	4847043	393820.8924	8.12497212
UJJ66235.1	50S_ribosomal_protein_L17_[Bacillus_cereus]	CPTF_Fe	4727655.667	697965.5023	14.76345892
UJJ66235.1	50S_ribosomal_protein_L17_[Bacillus_cereus]	CPTF_Mn	4913449.667	986936.254	20.08642239
UJJ66235.1	50S_ribosomal_protein_L17_[Bacillus_cereus]	CPTF_Ni	4521085.333	743132.872	16.43704591
UJJ66235.1	50S_ribosomal_protein_L17_[Bacillus_cereus]	CPTF_U	3511506.333	515879.4926	14.69111668
UJJ66235.1	50S_ribosomal_protein_L17_[Bacillus_cereus]	CPTF_metals_mix	6286209.667	1141586.633	18.16017431
UJJ66235.1	50S_ribosomal_protein_L17_[Bacillus_cereus]	CPTF_zcontrol	4571957.667	281013.9215	6.146468142
UJJ66236.1	DNA-directed_RNA_polymerase_subunit_alpha_[Bacillus_cereus]	CPTF_Al	13304448.53	354053.7397	2.661168096
UJJ66236.1	DNA-directed_RNA_polymerase_subunit_alpha_[Bacillus_cereus]	CPTF_Cd	13275255.93	640777.6453	4.826857188
UJJ66236.1	DNA-directed_RNA_polymerase_subunit_alpha_[Bacillus_cereus]	CPTF_Co	13249584	680712.6293	5.137615108
UJJ66236.1	DNA-directed_RNA_polymerase_subunit_alpha_[Bacillus_cereus]	CPTF_Cu	12653701.67	276623.6632	2.186108623
UJJ66236.1	DNA-directed_RNA_polymerase_subunit_alpha_[Bacillus_cereus]	CPTF_Fe	14154679.67	705896.3899	4.987017767
UJJ66236.1	DNA-directed_RNA_polymerase_subunit_alpha_[Bacillus_cereus]	CPTF_Mn	13475512	683304.7424	5.070714511
UJJ66236.1	DNA-directed_RNA_polymerase_subunit_alpha_[Bacillus_cereus]	CPTF_Ni	12642827.33	834838.0528	6.603254405
UJJ66236.1	DNA-directed_RNA_polymerase_subunit_alpha_[Bacillus_cereus]	CPTF_U	13812123.27	2739755.252	19.83587316
UJJ66236.1	DNA-directed_RNA_polymerase_subunit_alpha_[Bacillus_cereus]	CPTF_metals_mix	12812197.8	1735148.298	13.54294029
UJJ66236.1	DNA-directed_RNA_polymerase_subunit_alpha_[Bacillus_cereus]	CPTF_zcontrol	13484605.5	525116.2796	3.894190895
UJJ66237.1	30S_ribosomal_protein_S11_[Bacillus_cereus]	CPTF_Al	6692464	1601214.543	23.92563551
UJJ66237.1	30S_ribosomal_protein_S11_[Bacillus_cereus]	CPTF_Cd	8576150.667	540752.4661	6.305305109
UJJ66237.1	30S_ribosomal_protein_S11_[Bacillus_cereus]	CPTF_Co	6906635	803021.0723	11.62680629
UJJ66237.1	30S_ribosomal_protein_S11_[Bacillus_cereus]	CPTF_Cu	5976553.4	825595.6091	13.81390835
UJJ66237.1	30S_ribosomal_protein_S11_[Bacillus_cereus]	CPTF_Fe	6734018.933	1491126.048	22.1431817
UJJ66237.1	30S_ribosomal_protein_S11_[Bacillus_cereus]	CPTF_Mn	6874818.067	3535022.408	51.41986848
UJJ66237.1	30S_ribosomal_protein_S11_[Bacillus_cereus]	CPTF_Ni	2785660.333	1083111.452	38.88167696
UJJ66237.1	30S_ribosomal_protein_S11_[Bacillus_cereus]	CPTF_U	3807383.633	817668.7947	21.47587093
UJJ66237.1	30S_ribosomal_protein_S11_[Bacillus_cereus]	CPTF_metals_mix	10327837.17	244161.028	2.364106095
UJJ66237.1	30S_ribosomal_protein_S11_[Bacillus_cereus]	CPTF_zcontrol	5428091.333	1579351.695	29.0958939
UJJ66238.1	30S_ribosomal_protein_S13_[Bacillus_cereus]	CPTF_Al	9531722.667	182556.7932	1.915254981
UJJ66238.1	30S_ribosomal_protein_S13_[Bacillus_cereus]	CPTF_Cd	9677118.667	457869.2122	4.731462204
UJJ66238.1	30S_ribosomal_protein_S13_[Bacillus_cereus]	CPTF_Co	9848150	646044.5297	6.560059805
UJJ66238.1	30S_ribosomal_protein_S13_[Bacillus_cereus]	CPTF_Cu	8784652	532797.2872	6.0650927
UJJ66238.1	30S_ribosomal_protein_S13_[Bacillus_cereus]	CPTF_Fe	9828283	840329.5807	8.550115831
UJJ66238.1	30S_ribosomal_protein_S13_[Bacillus_cereus]	CPTF_Mn	9706666.667	185022.5212	1.906138611
UJJ66238.1	30S_ribosomal_protein_S13_[Bacillus_cereus]	CPTF_Ni	9228196	110324.627	1.195516729
UJJ66238.1	30S_ribosomal_protein_S13_[Bacillus_cereus]	CPTF_U	10044903.9	900747.3167	8.96720691
UJJ66238.1	30S_ribosomal_protein_S13_[Bacillus_cereus]	CPTF_metals_mix	11873333.33	3480766.199	29.31582986
UJJ66238.1	30S_ribosomal_protein_S13_[Bacillus_cereus]	CPTF_zcontrol	10203333.33	443771.7131	4.349281736
UJJ66239.1	50S_ribosomal_protein_L36_[Bacillus_cereus]	CPTF_Al	0	0	0
UJJ66239.1	50S_ribosomal_protein_L36_[Bacillus_cereus]	CPTF_Cd	0	0	0
UJJ66239.1	50S_ribosomal_protein_L36_[Bacillus_cereus]	CPTF_Co	0	0	0
UJJ66239.1	50S_ribosomal_protein_L36_[Bacillus_cereus]	CPTF_Cu	0	0	0
UJJ66239.1	50S_ribosomal_protein_L36_[Bacillus_cereus]	CPTF_Fe	0	0	0
UJJ66239.1	50S_ribosomal_protein_L36_[Bacillus_cereus]	CPTF_Mn	0	0	0
UJJ66239.1	50S_ribosomal_protein_L36_[Bacillus_cereus]	CPTF_Ni	0	0	0
UJJ66239.1	50S_ribosomal_protein_L36_[Bacillus_cereus]	CPTF_U	0	0	0
UJJ66239.1	50S_ribosomal_protein_L36_[Bacillus_cereus]	CPTF_metals_mix	475906.6667	195461.5508	41.07140422
UJJ66239.1	50S_ribosomal_protein_L36_[Bacillus_cereus]	CPTF_zcontrol	0	0	0
UJJ66240.1	translation_initiation_factor_IF-1_[Bacillus_cereus]	CPTF_Al	0	0	0
UJJ66240.1	translation_initiation_factor_IF-1_[Bacillus_cereus]	CPTF_Cd	15678.56667	27156.07406	173.2050808
UJJ66240.1	translation_initiation_factor_IF-1_[Bacillus_cereus]	CPTF_Co	0	0	0
UJJ66240.1	translation_initiation_factor_IF-1_[Bacillus_cereus]	CPTF_Cu	0	0	0
UJJ66240.1	translation_initiation_factor_IF-1_[Bacillus_cereus]	CPTF_Fe	0	0	0
UJJ66240.1	translation_initiation_factor_IF-1_[Bacillus_cereus]	CPTF_Mn	144962.3333	251082.1265	173.2050808
UJJ66240.1	translation_initiation_factor_IF-1_[Bacillus_cereus]	CPTF_Ni	0	0	0
UJJ66240.1	translation_initiation_factor_IF-1_[Bacillus_cereus]	CPTF_U	0	0	0
UJJ66240.1	translation_initiation_factor_IF-1_[Bacillus_cereus]	CPTF_metals_mix	1066726	119464.8277	11.19920464
UJJ66240.1	translation_initiation_factor_IF-1_[Bacillus_cereus]	CPTF_zcontrol	0	0	0
UJJ66241.1	type_I_methionyl_aminopeptidase_[Bacillus_cereus]	CPTF_Al	203485.3333	37955.00244	18.65245117
UJJ66241.1	type_I_methionyl_aminopeptidase_[Bacillus_cereus]	CPTF_Cd	174256.3333	16710.47987	9.589596858
UJJ66241.1	type_I_methionyl_aminopeptidase_[Bacillus_cereus]	CPTF_Co	214060.3333	10651.22877	4.97580687

UIJ66241.1	type_I_methionyl_aminopeptidase_[Bacillus_cereus]	CPTF_Cu	164776.6667	15976.45972	9.695826507
UIJ66241.1	type_I_methionyl_aminopeptidase_[Bacillus_cereus]	CPTF_Fe	161145.8	54855.81309	34.04110631
UIJ66241.1	type_I_methionyl_aminopeptidase_[Bacillus_cereus]	CPTF_Mn	164639	34487.23921	20.947187
UIJ66241.1	type_I_methionyl_aminopeptidase_[Bacillus_cereus]	CPTF_Ni	147720.3333	128748.3987	87.15685633
UIJ66241.1	type_I_methionyl_aminopeptidase_[Bacillus_cereus]	CPTF_U	198545.3333	54690.5379	27.54561741
UIJ66241.1	type_I_methionyl_aminopeptidase_[Bacillus_cereus]	CPTF_metals_mix	103206.8333	128157.1342	124.1750474
UIJ66241.1	type_I_methionyl_aminopeptidase_[Bacillus_cereus]	CPTF_zcontrol	136310	25546.5794	18.74152989
UIJ66242.1	adenylate_kinase_[Bacillus_cereus]	CPTF_Al	10279422.73	379854.3837	3.69528906
UIJ66242.1	adenylate_kinase_[Bacillus_cereus]	CPTF_Cd	10526495.53	1012024.375	9.614067394
UIJ66242.1	adenylate_kinase_[Bacillus_cereus]	CPTF_Co	10463566.73	435365.0091	4.160770607
UIJ66242.1	adenylate_kinase_[Bacillus_cereus]	CPTF_Cu	9809265.433	635625.2649	6.479845706
UIJ66242.1	adenylate_kinase_[Bacillus_cereus]	CPTF_Fe	9950419.8	814864.748	8.189249945
UIJ66242.1	adenylate_kinase_[Bacillus_cereus]	CPTF_Mn	9600452.233	317812.036	3.310386097
UIJ66242.1	adenylate_kinase_[Bacillus_cereus]	CPTF_Ni	9816539.467	191015.1874	1.945850552
UIJ66242.1	adenylate_kinase_[Bacillus_cereus]	CPTF_U	8473423.233	513589.9364	6.061185926
UIJ66242.1	adenylate_kinase_[Bacillus_cereus]	CPTF_metals_mix	6911070.967	275866.5875	3.991661912
UIJ66242.1	adenylate_kinase_[Bacillus_cereus]	CPTF_zcontrol	10146791.2	660578.9116	6.510224746
UIJ66243.1	preprotein_translocase_subunit_SecY_[Bacillus_cereus]	CPTF_Al	320771	36512.59247	11.38275981
UIJ66243.1	preprotein_translocase_subunit_SecY_[Bacillus_cereus]	CPTF_Cd	314689	25765.39454	8.187573936
UIJ66243.1	preprotein_translocase_subunit_SecY_[Bacillus_cereus]	CPTF_Co	377810	34494.24069	9.130049679
UIJ66243.1	preprotein_translocase_subunit_SecY_[Bacillus_cereus]	CPTF_Cu	342254.6667	5020.500008	1.466890154
UIJ66243.1	preprotein_translocase_subunit_SecY_[Bacillus_cereus]	CPTF_Fe	297310.6667	59207.80345	19.91445652
UIJ66243.1	preprotein_translocase_subunit_SecY_[Bacillus_cereus]	CPTF_Mn	297348	114702.3156	38.57510917
UIJ66243.1	preprotein_translocase_subunit_SecY_[Bacillus_cereus]	CPTF_Ni	324535.6667	18360.58992	5.657495249
UIJ66243.1	preprotein_translocase_subunit_SecY_[Bacillus_cereus]	CPTF_U	307179.3333	101191.7835	32.94224987
UIJ66243.1	preprotein_translocase_subunit_SecY_[Bacillus_cereus]	CPTF_metals_mix	208974	30123.29247	14.41485183
UIJ66243.1	preprotein_translocase_subunit_SecY_[Bacillus_cereus]	CPTF_zcontrol	314443.6667	57772.96643	18.37307364
UIJ66244.1	50S_ribosomal_protein_L15_[Bacillus_cereus]	CPTF_Al	3390916.667	750681.7804	22.13801913
UIJ66244.1	50S_ribosomal_protein_L15_[Bacillus_cereus]	CPTF_Cd	3541680.733	728820.0573	20.57836694
UIJ66244.1	50S_ribosomal_protein_L15_[Bacillus_cereus]	CPTF_Co	3755081.733	1217479.944	32.42219559
UIJ66244.1	50S_ribosomal_protein_L15_[Bacillus_cereus]	CPTF_Cu	3414940.767	926383.6718	27.1273716
UIJ66244.1	50S_ribosomal_protein_L15_[Bacillus_cereus]	CPTF_Fe	3379032.567	1426797.383	42.2250261
UIJ66244.1	50S_ribosomal_protein_L15_[Bacillus_cereus]	CPTF_Mn	4069192.033	1188773.35	29.21398992
UIJ66244.1	50S_ribosomal_protein_L15_[Bacillus_cereus]	CPTF_Ni	4158300.133	428002.0298	10.29271616
UIJ66244.1	50S_ribosomal_protein_L15_[Bacillus_cereus]	CPTF_U	2236121.533	592727.615	26.50694992
UIJ66244.1	50S_ribosomal_protein_L15_[Bacillus_cereus]	CPTF_metals_mix	6062556.8	1451639.695	23.94434795
UIJ66244.1	50S_ribosomal_protein_L15_[Bacillus_cereus]	CPTF_zcontrol	4580799.9	659550.6925	14.39815549
UIJ66245.1	50S_ribosomal_protein_L30_[Bacillus_cereus]	CPTF_Al	5496815.933	1111315.794	20.21744602
UIJ66245.1	50S_ribosomal_protein_L30_[Bacillus_cereus]	CPTF_Cd	5739646.667	1207019.626	21.02951099
UIJ66245.1	50S_ribosomal_protein_L30_[Bacillus_cereus]	CPTF_Co	6174028.667	720085.066	11.66313124
UIJ66245.1	50S_ribosomal_protein_L30_[Bacillus_cereus]	CPTF_Cu	6681296	1342388.781	20.0917424
UIJ66245.1	50S_ribosomal_protein_L30_[Bacillus_cereus]	CPTF_Fe	6112350.667	957442.2632	15.66405979
UIJ66245.1	50S_ribosomal_protein_L30_[Bacillus_cereus]	CPTF_Mn	5403276.8	568718.3008	10.5254334
UIJ66245.1	50S_ribosomal_protein_L30_[Bacillus_cereus]	CPTF_Ni	5148165	774422.0618	15.04268146
UIJ66245.1	50S_ribosomal_protein_L30_[Bacillus_cereus]	CPTF_U	3795591.033	307660.8918	8.105743982
UIJ66245.1	50S_ribosomal_protein_L30_[Bacillus_cereus]	CPTF_metals_mix	6710102.067	848786.0647	12.64937636
UIJ66245.1	50S_ribosomal_protein_L30_[Bacillus_cereus]	CPTF_zcontrol	5266910.8	560071.1552	10.63376952
UIJ66246.1	30S_ribosomal_protein_S5_[Bacillus_cereus]	CPTF_Al	15046751.6	2449586.271	16.27983459
UIJ66246.1	30S_ribosomal_protein_S5_[Bacillus_cereus]	CPTF_Cd	19231315	959475.7604	4.989132362
UIJ66246.1	30S_ribosomal_protein_S5_[Bacillus_cereus]	CPTF_Co	15154555.83	1027471.064	6.779948389
UIJ66246.1	30S_ribosomal_protein_S5_[Bacillus_cereus]	CPTF_Cu	13125188.17	1060896.347	8.082903907
UIJ66246.1	30S_ribosomal_protein_S5_[Bacillus_cereus]	CPTF_Fe	17177427.33	2770136.849	16.12661079
UIJ66246.1	30S_ribosomal_protein_S5_[Bacillus_cereus]	CPTF_Mn	16295900	5276363.13	32.37847023
UIJ66246.1	30S_ribosomal_protein_S5_[Bacillus_cereus]	CPTF_Ni	9926829.333	456239.6359	4.596025786
UIJ66246.1	30S_ribosomal_protein_S5_[Bacillus_cereus]	CPTF_U	11506624.93	2358922.254	20.50055744
UIJ66246.1	30S_ribosomal_protein_S5_[Bacillus_cereus]	CPTF_metals_mix	16415123.7	710030.7707	4.325467073
UIJ66246.1	30S_ribosomal_protein_S5_[Bacillus_cereus]	CPTF_zcontrol	13765938.03	3228679.299	23.45411763
UIJ66247.1	50S_ribosomal_protein_L6_[Bacillus_cereus]	CPTF_Al	17634262.73	1271691.575	7.211481388
UIJ66247.1	50S_ribosomal_protein_L6_[Bacillus_cereus]	CPTF_Cd	19123026	483615.8968	2.528971601
UIJ66247.1	50S_ribosomal_protein_L6_[Bacillus_cereus]	CPTF_Co	18035209.63	1369907.719	7.595740482
UIJ66247.1	50S_ribosomal_protein_L6_[Bacillus_cereus]	CPTF_Cu	15553088.03	678111.8648	4.359982168

UIJ66247.1	50S_ribosomal_protein_L6_[Bacillus_cereus]	CPTF_Fe	18241243.73	2254587.105	12.35983214
UIJ66247.1	50S_ribosomal_protein_L6_[Bacillus_cereus]	CPTF_Mn	18769886.67	2238665.592	11.92689989
UIJ66247.1	50S_ribosomal_protein_L6_[Bacillus_cereus]	CPTF_Ni	15753513.67	489285.3342	3.10588066
UIJ66247.1	50S_ribosomal_protein_L6_[Bacillus_cereus]	CPTF_U	17793567.9	1572532.049	8.837643232
UIJ66247.1	50S_ribosomal_protein_L6_[Bacillus_cereus]	CPTF_metals_mix	19297001.4	2022141.439	10.47904489
UIJ66247.1	50S_ribosomal_protein_L6_[Bacillus_cereus]	CPTF_zcontrol	18096588.47	1508750.028	8.337206933
UIJ66248.1	30S_ribosomal_protein_S8_[Bacillus_cereus]	CPTF_Al	6030659.6	262159.6131	4.347113426
UIJ66248.1	30S_ribosomal_protein_S8_[Bacillus_cereus]	CPTF_Cd	6125695.6	547725.8347	8.941447151
UIJ66248.1	30S_ribosomal_protein_S8_[Bacillus_cereus]	CPTF_Co	5889397.667	255903.2694	4.345151811
UIJ66248.1	30S_ribosomal_protein_S8_[Bacillus_cereus]	CPTF_Cu	5518453.667	304647.3413	5.520520053
UIJ66248.1	30S_ribosomal_protein_S8_[Bacillus_cereus]	CPTF_Fe	6046504.033	511275.9457	8.455728184
UIJ66248.1	30S_ribosomal_protein_S8_[Bacillus_cereus]	CPTF_Mn	6473884.333	566360.1475	8.748382244
UIJ66248.1	30S_ribosomal_protein_S8_[Bacillus_cereus]	CPTF_Ni	5095202.333	1709936.866	33.55974414
UIJ66248.1	30S_ribosomal_protein_S8_[Bacillus_cereus]	CPTF_U	7240074	3164550.002	43.70880743
UIJ66248.1	30S_ribosomal_protein_S8_[Bacillus_cereus]	CPTF_metals_mix	4888903.667	995680.7132	20.36613485
UIJ66248.1	30S_ribosomal_protein_S8_[Bacillus_cereus]	CPTF_zcontrol	5903928	255764.1424	4.332101313
UIJ66250.1	50S_ribosomal_protein_L5_[Bacillus_cereus]	CPTF_Al	13815212.9	2014527.035	14.58194709
UIJ66250.1	50S_ribosomal_protein_L5_[Bacillus_cereus]	CPTF_Cd	13661305	2550226.056	18.66751424
UIJ66250.1	50S_ribosomal_protein_L5_[Bacillus_cereus]	CPTF_Co	15567902.93	1217616.32	7.821325233
UIJ66250.1	50S_ribosomal_protein_L5_[Bacillus_cereus]	CPTF_Cu	14630404.43	653815.2936	4.468880519
UIJ66250.1	50S_ribosomal_protein_L5_[Bacillus_cereus]	CPTF_Fe	12592713.67	1895645.574	15.05351129
UIJ66250.1	50S_ribosomal_protein_L5_[Bacillus_cereus]	CPTF_Mn	16958721.67	4577474.996	26.99186346
UIJ66250.1	50S_ribosomal_protein_L5_[Bacillus_cereus]	CPTF_Ni	13105495.33	1649132.611	12.58351989
UIJ66250.1	50S_ribosomal_protein_L5_[Bacillus_cereus]	CPTF_U	10913258.03	414068.1244	3.794175151
UIJ66250.1	50S_ribosomal_protein_L5_[Bacillus_cereus]	CPTF_metals_mix	18318739	2177862.513	11.88871414
UIJ66250.1	50S_ribosomal_protein_L5_[Bacillus_cereus]	CPTF_zcontrol	15203159.57	918772.6336	6.043300602
UIJ66251.1	50S_ribosomal_protein_L24_[Bacillus_cereus]	CPTF_Al	6779422.767	368753.2026	5.439300886
UIJ66251.1	50S_ribosomal_protein_L24_[Bacillus_cereus]	CPTF_Cd	7437298.6	415539.0352	5.587230762
UIJ66251.1	50S_ribosomal_protein_L24_[Bacillus_cereus]	CPTF_Co	7151616.1	240191.2528	3.358559093
UIJ66251.1	50S_ribosomal_protein_L24_[Bacillus_cereus]	CPTF_Cu	6212922.633	349616.1707	5.62724166
UIJ66251.1	50S_ribosomal_protein_L24_[Bacillus_cereus]	CPTF_Fe	6961923.167	778039.5118	11.1756406
UIJ66251.1	50S_ribosomal_protein_L24_[Bacillus_cereus]	CPTF_Mn	7287161.533	1656157.692	22.72706161
UIJ66251.1	50S_ribosomal_protein_L24_[Bacillus_cereus]	CPTF_Ni	6210039.4	137029.0422	2.206572831
UIJ66251.1	50S_ribosomal_protein_L24_[Bacillus_cereus]	CPTF_U	5358048.167	405917.6779	7.575849737
UIJ66251.1	50S_ribosomal_protein_L24_[Bacillus_cereus]	CPTF_metals_mix	6785998.933	345392.4801	5.089780937
UIJ66251.1	50S_ribosomal_protein_L24_[Bacillus_cereus]	CPTF_zcontrol	6513824.133	299541.4771	4.598550268
UIJ66252.1	50S_ribosomal_protein_L14_[Bacillus_cereus]	CPTF_Al	11175977.33	164929.3421	1.475748717
UIJ66252.1	50S_ribosomal_protein_L14_[Bacillus_cereus]	CPTF_Cd	10883763.33	212982.4488	1.956882397
UIJ66252.1	50S_ribosomal_protein_L14_[Bacillus_cereus]	CPTF_Co	10863659.67	433507.8597	3.990440358
UIJ66252.1	50S_ribosomal_protein_L14_[Bacillus_cereus]	CPTF_Cu	10012580.3	580929.7925	5.801998837
UIJ66252.1	50S_ribosomal_protein_L14_[Bacillus_cereus]	CPTF_Fe	11083826	891011.4881	8.03884406
UIJ66252.1	50S_ribosomal_protein_L14_[Bacillus_cereus]	CPTF_Mn	10851097	526847.8626	4.855249775
UIJ66252.1	50S_ribosomal_protein_L14_[Bacillus_cereus]	CPTF_Ni	10642708.67	144777.682	1.360346191
UIJ66252.1	50S_ribosomal_protein_L14_[Bacillus_cereus]	CPTF_U	10978115.33	634888.7154	5.783221401
UIJ66252.1	50S_ribosomal_protein_L14_[Bacillus_cereus]	CPTF_metals_mix	7610134.667	384032.3377	5.046327752
UIJ66252.1	50S_ribosomal_protein_L14_[Bacillus_cereus]	CPTF_zcontrol	11061120	372308.3304	3.365918916
UIJ66253.1	30S_ribosomal_protein_S17_[Bacillus_cereus]	CPTF_Al	1613187	99784.18476	6.185531173
UIJ66253.1	30S_ribosomal_protein_S17_[Bacillus_cereus]	CPTF_Cd	1625334.333	177694.0384	10.9327684
UIJ66253.1	30S_ribosomal_protein_S17_[Bacillus_cereus]	CPTF_Co	1739736.667	83976.935	4.826991154
UIJ66253.1	30S_ribosomal_protein_S17_[Bacillus_cereus]	CPTF_Cu	1568460.667	237250.8938	15.12635279
UIJ66253.1	30S_ribosomal_protein_S17_[Bacillus_cereus]	CPTF_Fe	1413766.333	149365.6436	10.56508704
UIJ66253.1	30S_ribosomal_protein_S17_[Bacillus_cereus]	CPTF_Mn	1484671	777833.2353	52.3909496
UIJ66253.1	30S_ribosomal_protein_S17_[Bacillus_cereus]	CPTF_Ni	659194	542404.0325	82.28291405
UIJ66253.1	30S_ribosomal_protein_S17_[Bacillus_cereus]	CPTF_U	977008.6667	532725.0468	54.52613318
UIJ66253.1	30S_ribosomal_protein_S17_[Bacillus_cereus]	CPTF_metals_mix	1755642.667	47311.965	2.694851629
UIJ66253.1	30S_ribosomal_protein_S17_[Bacillus_cereus]	CPTF_zcontrol	1540419	47608.51637	3.090621212
UIJ66254.1	50S_ribosomal_protein_L29_[Bacillus_cereus]	CPTF_Al	11826529.37	1971659.806	16.67149968
UIJ66254.1	50S_ribosomal_protein_L29_[Bacillus_cereus]	CPTF_Cd	14340509.73	1172021.53	8.172802443
UIJ66254.1	50S_ribosomal_protein_L29_[Bacillus_cereus]	CPTF_Co	12274042.07	1512356.094	12.32158148
UIJ66254.1	50S_ribosomal_protein_L29_[Bacillus_cereus]	CPTF_Cu	10336677.47	734480.5169	7.105576422
UIJ66254.1	50S_ribosomal_protein_L29_[Bacillus_cereus]	CPTF_Fe	12999959.47	3218243.163	24.75579383

UIJ66254.1	50S_ribosomal_protein_L29_[Bacillus_cereus]	CPTF_Mn	12699530.27	3984955.653	31.37876417
UIJ66254.1	50S_ribosomal_protein_L29_[Bacillus_cereus]	CPTF_Ni	4596720.233	1237235.343	26.91561115
UIJ66254.1	50S_ribosomal_protein_L29_[Bacillus_cereus]	CPTF_U	7110628.667	911352.6737	12.81676651
UIJ66254.1	50S_ribosomal_protein_L29_[Bacillus_cereus]	CPTF_metals_mix	11350002.33	363045.9234	3.198641839
UIJ66254.1	50S_ribosomal_protein_L29_[Bacillus_cereus]	CPTF_zcontrol	10792400.1	2638238.479	24.44533612
UIJ66255.1	50S_ribosomal_protein_L16_[Bacillus_cereus]	CPTF_Al	3189264.667	296145.3906	9.285695027
UIJ66255.1	50S_ribosomal_protein_L16_[Bacillus_cereus]	CPTF_Cd	3033333.333	187705.443	6.188091528
UIJ66255.1	50S_ribosomal_protein_L16_[Bacillus_cereus]	CPTF_Co	3776896.333	566246.7733	14.99238325
UIJ66255.1	50S_ribosomal_protein_L16_[Bacillus_cereus]	CPTF_Cu	3830434.2	299079.0381	7.807966996
UIJ66255.1	50S_ribosomal_protein_L16_[Bacillus_cereus]	CPTF_Fe	3270000	259422.4354	7.933407811
UIJ66255.1	50S_ribosomal_protein_L16_[Bacillus_cereus]	CPTF_Mn	3232524	437356.6044	13.52987957
UIJ66255.1	50S_ribosomal_protein_L16_[Bacillus_cereus]	CPTF_Ni	3226666.667	134288.2472	4.161825843
UIJ66255.1	50S_ribosomal_protein_L16_[Bacillus_cereus]	CPTF_U	2604816	405192.5747	15.55551619
UIJ66255.1	50S_ribosomal_protein_L16_[Bacillus_cereus]	CPTF_metals_mix	3931239.333	1080744.231	27.4911838
UIJ66255.1	50S_ribosomal_protein_L16_[Bacillus_cereus]	CPTF_zcontrol	3065021.367	111717.3683	3.644913196
UIJ66256.1	30S_ribosomal_protein_S3_[Bacillus_cereus]	CPTF_Al	22538773.43	1194505.59	5.299780814
UIJ66256.1	30S_ribosomal_protein_S3_[Bacillus_cereus]	CPTF_Cd	24172529.67	640106.1975	2.648072859
UIJ66256.1	30S_ribosomal_protein_S3_[Bacillus_cereus]	CPTF_Co	25268250.47	1974110.652	7.812613122
UIJ66256.1	30S_ribosomal_protein_S3_[Bacillus_cereus]	CPTF_Cu	22998825.67	403852.8273	1.755971514
UIJ66256.1	30S_ribosomal_protein_S3_[Bacillus_cereus]	CPTF_Fe	23981467.77	1105370.986	4.609271612
UIJ66256.1	30S_ribosomal_protein_S3_[Bacillus_cereus]	CPTF_Mn	24030673.3	2666606.997	11.09668033
UIJ66256.1	30S_ribosomal_protein_S3_[Bacillus_cereus]	CPTF_Ni	19488494	1231960.191	6.321474561
UIJ66256.1	30S_ribosomal_protein_S3_[Bacillus_cereus]	CPTF_U	20782696	1680164.921	8.0844416
UIJ66256.1	30S_ribosomal_protein_S3_[Bacillus_cereus]	CPTF_metals_mix	20841198.47	328146.1587	1.574507144
UIJ66256.1	30S_ribosomal_protein_S3_[Bacillus_cereus]	CPTF_zcontrol	24005780	2113310.103	8.803338623
UIJ66257.1	50S_ribosomal_protein_L22_[Bacillus_cereus]	CPTF_Al	4219483.333	560264.7763	13.27804217
UIJ66257.1	50S_ribosomal_protein_L22_[Bacillus_cereus]	CPTF_Cd	5459624.967	233806.605	4.282466404
UIJ66257.1	50S_ribosomal_protein_L22_[Bacillus_cereus]	CPTF_Co	4801885	101624.9541	2.116355434
UIJ66257.1	50S_ribosomal_protein_L22_[Bacillus_cereus]	CPTF_Cu	4416094.6	444990.2493	10.07655609
UIJ66257.1	50S_ribosomal_protein_L22_[Bacillus_cereus]	CPTF_Fe	4336123.633	727070.0678	16.76774302
UIJ66257.1	50S_ribosomal_protein_L22_[Bacillus_cereus]	CPTF_Mn	5144330.5	2457925.316	47.77930415
UIJ66257.1	50S_ribosomal_protein_L22_[Bacillus_cereus]	CPTF_Ni	3315116.333	463567.4255	13.98344368
UIJ66257.1	50S_ribosomal_protein_L22_[Bacillus_cereus]	CPTF_U	2848534.667	362559.1467	12.72791765
UIJ66257.1	50S_ribosomal_protein_L22_[Bacillus_cereus]	CPTF_metals_mix	9491052.467	2387228.681	25.15241265
UIJ66257.1	50S_ribosomal_protein_L22_[Bacillus_cereus]	CPTF_zcontrol	3848023.667	721244.7617	18.74325171
UIJ66258.1	30S_ribosomal_protein_S19_[Bacillus_cereus]	CPTF_Al	3091246.1	143864.75	4.653940363
UIJ66258.1	30S_ribosomal_protein_S19_[Bacillus_cereus]	CPTF_Cd	3075072.667	23307.50228	0.757949642
UIJ66258.1	30S_ribosomal_protein_S19_[Bacillus_cereus]	CPTF_Co	3320762.333	73130.83677	2.202230374
UIJ66258.1	30S_ribosomal_protein_S19_[Bacillus_cereus]	CPTF_Cu	2915579.333	214822.1693	7.36807834
UIJ66258.1	30S_ribosomal_protein_S19_[Bacillus_cereus]	CPTF_Fe	3221369.333	180790.7337	5.612232409
UIJ66258.1	30S_ribosomal_protein_S19_[Bacillus_cereus]	CPTF_Mn	3007175.767	94472.58192	3.141571669
UIJ66258.1	30S_ribosomal_protein_S19_[Bacillus_cereus]	CPTF_Ni	3060736.4	119589.928	3.907227294
UIJ66258.1	30S_ribosomal_protein_S19_[Bacillus_cereus]	CPTF_U	2857377.333	110994.468	3.884487593
UIJ66258.1	30S_ribosomal_protein_S19_[Bacillus_cereus]	CPTF_metals_mix	2409574.7	535839.6758	22.23793584
UIJ66258.1	30S_ribosomal_protein_S19_[Bacillus_cereus]	CPTF_zcontrol	3182602.867	120594.9887	3.789193743
UIJ66259.1	50S_ribosomal_protein_L2_[Bacillus_cereus]	CPTF_Al	14602382.1	712246.844	4.877607223
UIJ66259.1	50S_ribosomal_protein_L2_[Bacillus_cereus]	CPTF_Cd	15989194.3	668415.6177	4.180420884
UIJ66259.1	50S_ribosomal_protein_L2_[Bacillus_cereus]	CPTF_Co	16599309.5	394276.5026	2.375258457
UIJ66259.1	50S_ribosomal_protein_L2_[Bacillus_cereus]	CPTF_Cu	14453636.33	676296.216	4.679073144
UIJ66259.1	50S_ribosomal_protein_L2_[Bacillus_cereus]	CPTF_Fe	15046651.27	893219.2761	5.936332678
UIJ66259.1	50S_ribosomal_protein_L2_[Bacillus_cereus]	CPTF_Mn	15699618.37	1353422.728	8.620736484
UIJ66259.1	50S_ribosomal_protein_L2_[Bacillus_cereus]	CPTF_Ni	12558633.03	672883.9151	5.357939143
UIJ66259.1	50S_ribosomal_protein_L2_[Bacillus_cereus]	CPTF_U	14040662.87	1752290.945	12.48011552
UIJ66259.1	50S_ribosomal_protein_L2_[Bacillus_cereus]	CPTF_metals_mix	14690117.67	1014556.709	6.90638926
UIJ66259.1	50S_ribosomal_protein_L2_[Bacillus_cereus]	CPTF_zcontrol	14383011.07	1114552.071	7.749087209
UIJ66260.1	50S_ribosomal_protein_L23_[Bacillus_cereus]	CPTF_Al	17498783.73	463414.7496	2.648268341
UIJ66260.1	50S_ribosomal_protein_L23_[Bacillus_cereus]	CPTF_Cd	16778425.67	1814313.697	10.81337268
UIJ66260.1	50S_ribosomal_protein_L23_[Bacillus_cereus]	CPTF_Co	18031245.67	724686.896	4.019061741
UIJ66260.1	50S_ribosomal_protein_L23_[Bacillus_cereus]	CPTF_Cu	17289803.67	1699427.907	9.829075793
UIJ66260.1	50S_ribosomal_protein_L23_[Bacillus_cereus]	CPTF_Fe	17350000	857146.4286	4.940325237
UIJ66260.1	50S_ribosomal_protein_L23_[Bacillus_cereus]	CPTF_Mn	17499617	3390481.315	19.37460297

UIJ66260.1	50S_ribosomal_protein_L23_[Bacillus_cereus]	CPTF_Ni	18089033.33	1517554.122	8.389359973
UIJ66260.1	50S_ribosomal_protein_L23_[Bacillus_cereus]	CPTF_U	17634498.23	1251414.04	7.096397204
UIJ66260.1	50S_ribosomal_protein_L23_[Bacillus_cereus]	CPTF_metals_mix	12582264	699163.7114	5.556740118
UIJ66260.1	50S_ribosomal_protein_L23_[Bacillus_cereus]	CPTF_zcontrol	17923125.33	410048.7326	2.287819368
UIJ66261.1	50S_ribosomal_protein_L4_[Bacillus_cereus]	CPTF_Al	18409569	971936.0858	5.279515701
UIJ66261.1	50S_ribosomal_protein_L4_[Bacillus_cereus]	CPTF_Cd	19296564.8	1381300.798	7.158273052
UIJ66261.1	50S_ribosomal_protein_L4_[Bacillus_cereus]	CPTF_Co	20313929	1698733.952	8.362409616
UIJ66261.1	50S_ribosomal_protein_L4_[Bacillus_cereus]	CPTF_Cu	17378788.33	636660.2345	3.663432814
UIJ66261.1	50S_ribosomal_protein_L4_[Bacillus_cereus]	CPTF_Fe	19373293.43	708043.9941	3.654742527
UIJ66261.1	50S_ribosomal_protein_L4_[Bacillus_cereus]	CPTF_Mn	20650891.3	1122838.824	5.437241463
UIJ66261.1	50S_ribosomal_protein_L4_[Bacillus_cereus]	CPTF_Ni	19456161	1528860.31	7.857975218
UIJ66261.1	50S_ribosomal_protein_L4_[Bacillus_cereus]	CPTF_U	20748040.33	1194399.982	5.756688163
UIJ66261.1	50S_ribosomal_protein_L4_[Bacillus_cereus]	CPTF_metals_mix	12826227	1372139.369	10.69791895
UIJ66261.1	50S_ribosomal_protein_L4_[Bacillus_cereus]	CPTF_zcontrol	22158426	749463.6346	3.382296354
UIJ66262.1	50S_ribosomal_protein_L3_[Bacillus_cereus]	CPTF_Al	14136204.9	324112.4708	2.292782774
UIJ66262.1	50S_ribosomal_protein_L3_[Bacillus_cereus]	CPTF_Cd	14269250.07	372219.1499	2.60854038
UIJ66262.1	50S_ribosomal_protein_L3_[Bacillus_cereus]	CPTF_Co	15904913	1614309.006	10.14975062
UIJ66262.1	50S_ribosomal_protein_L3_[Bacillus_cereus]	CPTF_Cu	13454098.67	63272.48962	0.470284121
UIJ66262.1	50S_ribosomal_protein_L3_[Bacillus_cereus]	CPTF_Fe	14009918.57	940935.1732	6.716207298
UIJ66262.1	50S_ribosomal_protein_L3_[Bacillus_cereus]	CPTF_Mn	15129224.33	774868.0505	5.121664095
UIJ66262.1	50S_ribosomal_protein_L3_[Bacillus_cereus]	CPTF_Ni	13057318.33	771086.3064	5.905395631
UIJ66262.1	50S_ribosomal_protein_L3_[Bacillus_cereus]	CPTF_U	13571043.23	1275941.037	9.401937748
UIJ66262.1	50S_ribosomal_protein_L3_[Bacillus_cereus]	CPTF_metals_mix	11463571.17	428484.6695	3.73779395
UIJ66262.1	50S_ribosomal_protein_L3_[Bacillus_cereus]	CPTF_zcontrol	15345435.23	1056309.663	6.883543197
UIJ66263.1	30S_ribosomal_protein_S10_[Bacillus_cereus]	CPTF_Al	5650000	793032.1557	14.03596736
UIJ66263.1	30S_ribosomal_protein_S10_[Bacillus_cereus]	CPTF_Cd	6063333.333	354729.9442	5.850411395
UIJ66263.1	30S_ribosomal_protein_S10_[Bacillus_cereus]	CPTF_Co	5453333.333	212210.5872	3.891392186
UIJ66263.1	30S_ribosomal_protein_S10_[Bacillus_cereus]	CPTF_Cu	5106666.667	290229.794	5.683351058
UIJ66263.1	30S_ribosomal_protein_S10_[Bacillus_cereus]	CPTF_Fe	6230000	970412.2835	15.57644115
UIJ66263.1	30S_ribosomal_protein_S10_[Bacillus_cereus]	CPTF_Mn	5493998.133	922053.2757	16.78291935
UIJ66263.1	30S_ribosomal_protein_S10_[Bacillus_cereus]	CPTF_Ni	4993333.333	858739.3862	17.19771801
UIJ66263.1	30S_ribosomal_protein_S10_[Bacillus_cereus]	CPTF_U	7936666.667	232880.513	2.934235779
UIJ66263.1	30S_ribosomal_protein_S10_[Bacillus_cereus]	CPTF_metals_mix	7047058.367	400838.845	5.688030723
UIJ66263.1	30S_ribosomal_protein_S10_[Bacillus_cereus]	CPTF_zcontrol	5280000	320780.2986	6.075384444
UIJ66264.1	elongation_factor_Tu_[Bacillus_cereus]	CPTF_Al	121781494.6	3995433.11	3.280821213
UIJ66264.1	elongation_factor_Tu_[Bacillus_cereus]	CPTF_Cd	122811388.4	4541381.556	3.697850513
UIJ66264.1	elongation_factor_Tu_[Bacillus_cereus]	CPTF_Co	131319415	9354466.997	7.123445529
UIJ66264.1	elongation_factor_Tu_[Bacillus_cereus]	CPTF_Cu	124142878.5	6995184.665	5.634785298
UIJ66264.1	elongation_factor_Tu_[Bacillus_cereus]	CPTF_Fe	122335866.7	4846328.803	3.96149464
UIJ66264.1	elongation_factor_Tu_[Bacillus_cereus]	CPTF_Mn	122362542.5	12388100.7	10.12409554
UIJ66264.1	elongation_factor_Tu_[Bacillus_cereus]	CPTF_Ni	126965560.2	2889490.738	2.27580671
UIJ66264.1	elongation_factor_Tu_[Bacillus_cereus]	CPTF_U	137565883.1	8025315.829	5.833798065
UIJ66264.1	elongation_factor_Tu_[Bacillus_cereus]	CPTF_metals_mix	132580952.8	19842505.53	14.96633198
UIJ66264.1	elongation_factor_Tu_[Bacillus_cereus]	CPTF_zcontrol	131404039.8	3123084.453	2.376703531
UIJ66265.1	elongation_factor_G_[Bacillus_cereus]	CPTF_Al	36605334.87	2110760.438	5.766264522
UIJ66265.1	elongation_factor_G_[Bacillus_cereus]	CPTF_Cd	41650853.8	3214370.732	7.717418584
UIJ66265.1	elongation_factor_G_[Bacillus_cereus]	CPTF_Co	39070950.1	2528522.776	6.471618349
UIJ66265.1	elongation_factor_G_[Bacillus_cereus]	CPTF_Cu	36529706.53	1189833.33	3.257166408
UIJ66265.1	elongation_factor_G_[Bacillus_cereus]	CPTF_Fe	38278635.31	2427051.468	6.340485884
UIJ66265.1	elongation_factor_G_[Bacillus_cereus]	CPTF_Mn	44282197.7	10211536.47	23.0601393
UIJ66265.1	elongation_factor_G_[Bacillus_cereus]	CPTF_Ni	34401885.53	1758182.895	5.110716659
UIJ66265.1	elongation_factor_G_[Bacillus_cereus]	CPTF_U	33639025	4741371.16	14.09485311
UIJ66265.1	elongation_factor_G_[Bacillus_cereus]	CPTF_metals_mix	60703150.23	6185597.353	10.18991161
UIJ66265.1	elongation_factor_G_[Bacillus_cereus]	CPTF_zcontrol	36356011.37	3030993.728	8.336980911
UIJ66266.1	30S_ribosomal_protein_S7_[Bacillus_cereus]	CPTF_Al	17016970.77	1053349.878	6.1899964
UIJ66266.1	30S_ribosomal_protein_S7_[Bacillus_cereus]	CPTF_Cd	19273107.5	792502.1431	4.111958298
UIJ66266.1	30S_ribosomal_protein_S7_[Bacillus_cereus]	CPTF_Co	18179438.5	358736.0936	1.973306786
UIJ66266.1	30S_ribosomal_protein_S7_[Bacillus_cereus]	CPTF_Cu	15909420.8	696520.3467	4.378037111
UIJ66266.1	30S_ribosomal_protein_S7_[Bacillus_cereus]	CPTF_Fe	17734384.87	1566132.96	8.831053186
UIJ66266.1	30S_ribosomal_protein_S7_[Bacillus_cereus]	CPTF_Mn	18784597.93	2992456.038	15.93037045
UIJ66266.1	30S_ribosomal_protein_S7_[Bacillus_cereus]	CPTF_Ni	14641152.1	800538.245	5.467727126

UIJ66266.1	30S_ribosomal_protein_S7_[Bacillus_cereus]	CPTF_U	15951994.73	851096.167	5.335358877
UIJ66266.1	30S_ribosomal_protein_S7_[Bacillus_cereus]	CPTF_metals_mix	16957719	1506287.724	8.882608116
UIJ66266.1	30S_ribosomal_protein_S7_[Bacillus_cereus]	CPTF_zcontrol	17849936.87	2143949.804	12.01096575
UIJ66267.1	30S_ribosomal_protein_S12_[Bacillus_cereus]	CPTF_Al	10542054.63	631548.3208	5.990751735
UIJ66267.1	30S_ribosomal_protein_S12_[Bacillus_cereus]	CPTF_Cd	11200609.67	417780.9807	3.729984287
UIJ66267.1	30S_ribosomal_protein_S12_[Bacillus_cereus]	CPTF_Co	11382917.87	702908.3173	6.175115427
UIJ66267.1	30S_ribosomal_protein_S12_[Bacillus_cereus]	CPTF_Cu	10235728.93	831682.5231	8.125288668
UIJ66267.1	30S_ribosomal_protein_S12_[Bacillus_cereus]	CPTF_Fe	10675034.13	1053417.275	9.868045965
UIJ66267.1	30S_ribosomal_protein_S12_[Bacillus_cereus]	CPTF_Mn	11194275.5	2191167.802	19.57400282
UIJ66267.1	30S_ribosomal_protein_S12_[Bacillus_cereus]	CPTF_Ni	9267264.667	1134335.507	12.24024075
UIJ66267.1	30S_ribosomal_protein_S12_[Bacillus_cereus]	CPTF_U	9358788.667	1266777.985	13.53570457
UIJ66267.1	30S_ribosomal_protein_S12_[Bacillus_cereus]	CPTF_metals_mix	10679921.9	296844.7418	2.779465474
UIJ66267.1	30S_ribosomal_protein_S12_[Bacillus_cereus]	CPTF_zcontrol	10808654	896244.7775	8.291918471
UIJ66269.1	DNA-directed_RNA_polymerase_subunit_beta'_[Bacillus_cereus]	CPTF_Al	14844310.8	2445822.348	16.47649649
UIJ66269.1	DNA-directed_RNA_polymerase_subunit_beta'_[Bacillus_cereus]	CPTF_Cd	18480167.1	4848020.065	26.2336376
UIJ66269.1	DNA-directed_RNA_polymerase_subunit_beta'_[Bacillus_cereus]	CPTF_Co	15981474.6	910292.1263	5.69592074
UIJ66269.1	DNA-directed_RNA_polymerase_subunit_beta'_[Bacillus_cereus]	CPTF_Cu	16098681.4	987439.5113	6.133667018
UIJ66269.1	DNA-directed_RNA_polymerase_subunit_beta'_[Bacillus_cereus]	CPTF_Fe	15814432.53	1238150.15	7.829241721
UIJ66269.1	DNA-directed_RNA_polymerase_subunit_beta'_[Bacillus_cereus]	CPTF_Mn	17465516.93	4275655.369	24.48055437
UIJ66269.1	DNA-directed_RNA_polymerase_subunit_beta'_[Bacillus_cereus]	CPTF_Ni	14545290.17	388633.9612	2.671888678
UIJ66269.1	DNA-directed_RNA_polymerase_subunit_beta'_[Bacillus_cereus]	CPTF_U	12414800.67	1448346.057	11.66628524
UIJ66269.1	DNA-directed_RNA_polymerase_subunit_beta'_[Bacillus_cereus]	CPTF_metals_mix	24318274.53	3818327.761	15.70147485
UIJ66269.1	DNA-directed_RNA_polymerase_subunit_beta'_[Bacillus_cereus]	CPTF_zcontrol	15934658.47	1104728.027	6.932862911
UIJ66270.1	DNA-directed_RNA_polymerase_subunit_beta_[Bacillus_cereus]	CPTF_Al	25579069.33	2256865.522	8.823094744
UIJ66270.1	DNA-directed_RNA_polymerase_subunit_beta_[Bacillus_cereus]	CPTF_Cd	28352952.5	615799.1384	2.171904808
UIJ66270.1	DNA-directed_RNA_polymerase_subunit_beta_[Bacillus_cereus]	CPTF_Co	27218838.57	716032.3624	2.630649947
UIJ66270.1	DNA-directed_RNA_polymerase_subunit_beta_[Bacillus_cereus]	CPTF_Cu	25012000.77	1007761.443	4.029111674
UIJ66270.1	DNA-directed_RNA_polymerase_subunit_beta_[Bacillus_cereus]	CPTF_Fe	25585248.8	3086798.811	12.0647598
UIJ66270.1	DNA-directed_RNA_polymerase_subunit_beta_[Bacillus_cereus]	CPTF_Mn	26561357.8	6103355.462	22.97832629
UIJ66270.1	DNA-directed_RNA_polymerase_subunit_beta_[Bacillus_cereus]	CPTF_Ni	20818272.9	1553224.108	7.460869186
UIJ66270.1	DNA-directed_RNA_polymerase_subunit_beta_[Bacillus_cereus]	CPTF_U	20825408.93	1657898.693	7.96094184
UIJ66270.1	DNA-directed_RNA_polymerase_subunit_beta_[Bacillus_cereus]	CPTF_metals_mix	34446195.1	2398850.778	6.964051533
UIJ66270.1	DNA-directed_RNA_polymerase_subunit_beta_[Bacillus_cereus]	CPTF_zcontrol	24932197.03	2073565.623	8.316818689
UIJ66272.1	50S_ribosomal_protein_L7/L12_[Bacillus_cereus]	CPTF_Al	51199005.07	4568596.709	8.923213845
UIJ66272.1	50S_ribosomal_protein_L7/L12_[Bacillus_cereus]	CPTF_Cd	55425457.67	417803.4882	0.753811526
UIJ66272.1	50S_ribosomal_protein_L7/L12_[Bacillus_cereus]	CPTF_Co	57225897.33	3779879.806	6.605190974
UIJ66272.1	50S_ribosomal_protein_L7/L12_[Bacillus_cereus]	CPTF_Cu	46986608.67	3041930.041	6.474036172
UIJ66272.1	50S_ribosomal_protein_L7/L12_[Bacillus_cereus]	CPTF_Fe	51815231.2	8628820.675	16.65305833
UIJ66272.1	50S_ribosomal_protein_L7/L12_[Bacillus_cereus]	CPTF_Mn	56105974.83	10424179.96	18.57944719
UIJ66272.1	50S_ribosomal_protein_L7/L12_[Bacillus_cereus]	CPTF_Ni	42027951.53	3307168.668	7.868974212
UIJ66272.1	50S_ribosomal_protein_L7/L12_[Bacillus_cereus]	CPTF_U	46973418.87	2770258.902	5.897503244
UIJ66272.1	50S_ribosomal_protein_L7/L12_[Bacillus_cereus]	CPTF_metals_mix	51264701.27	3449082.405	6.727986938
UIJ66272.1	50S_ribosomal_protein_L7/L12_[Bacillus_cereus]	CPTF_zcontrol	53022352.33	5935116.26	11.19361175
UIJ66273.1	50S_ribosomal_protein_L10_[Bacillus_cereus]	CPTF_Al	19171706.33	668961.2196	3.489314973
UIJ66273.1	50S_ribosomal_protein_L10_[Bacillus_cereus]	CPTF_Cd	18904828.33	826708.1196	4.372999876
UIJ66273.1	50S_ribosomal_protein_L10_[Bacillus_cereus]	CPTF_Co	21324936.33	686250.0623	3.218063827
UIJ66273.1	50S_ribosomal_protein_L10_[Bacillus_cereus]	CPTF_Cu	19690265.33	136192.2852	0.691673184
UIJ66273.1	50S_ribosomal_protein_L10_[Bacillus_cereus]	CPTF_Fe	19584645.33	369701.1295	1.887709086
UIJ66273.1	50S_ribosomal_protein_L10_[Bacillus_cereus]	CPTF_Mn	19820346.33	344720.3582	1.739224696
UIJ66273.1	50S_ribosomal_protein_L10_[Bacillus_cereus]	CPTF_Ni	18935585	948439.6184	5.008768508
UIJ66273.1	50S_ribosomal_protein_L10_[Bacillus_cereus]	CPTF_U	17587689.33	1934758.467	11.00064045
UIJ66273.1	50S_ribosomal_protein_L10_[Bacillus_cereus]	CPTF_metals_mix	18108395.67	1073057.791	5.925747433
UIJ66273.1	50S_ribosomal_protein_L10_[Bacillus_cereus]	CPTF_zcontrol	19291739	1420879.733	7.365223702
UIJ66274.1	50S_ribosomal_protein_L1_[Bacillus_cereus]	CPTF_Al	16294108.67	1063852.443	6.529061913
UIJ66274.1	50S_ribosomal_protein_L1_[Bacillus_cereus]	CPTF_Cd	17755288.67	417839.6106	2.353324795
UIJ66274.1	50S_ribosomal_protein_L1_[Bacillus_cereus]	CPTF_Co	18965577.33	938136.6879	4.946523227
UIJ66274.1	50S_ribosomal_protein_L1_[Bacillus_cereus]	CPTF_Cu	16685643.67	1066965.646	6.394512953
UIJ66274.1	50S_ribosomal_protein_L1_[Bacillus_cereus]	CPTF_Fe	16454238.67	1562664.59	9.497033692
UIJ66274.1	50S_ribosomal_protein_L1_[Bacillus_cereus]	CPTF_Mn	17130142.67	3519115.78	20.54341197
UIJ66274.1	50S_ribosomal_protein_L1_[Bacillus_cereus]	CPTF_Ni	13957830.33	2178947.06	15.61092955
UIJ66274.1	50S_ribosomal_protein_L1_[Bacillus_cereus]	CPTF_U	12417329.67	400781.9925	3.227602096

UIJ66274.1	50S_ribosomal_protein_L1_[Bacillus_cereus]	CPTF_metals_mix	18252172.87	1035322.277	5.672323424
UIJ66274.1	50S_ribosomal_protein_L1_[Bacillus_cereus]	CPTF_zcontrol	16480142.67	1263894.264	7.669194916
UIJ66275.1	50S_ribosomal_protein_L11_[Bacillus_cereus]	CPTF_Al	7956293	470851.6721	5.917978035
UIJ66275.1	50S_ribosomal_protein_L11_[Bacillus_cereus]	CPTF_Cd	8322204.667	249611.3636	2.999341805
UIJ66275.1	50S_ribosomal_protein_L11_[Bacillus_cereus]	CPTF_Co	8682287	415074.1953	4.78070116
UIJ66275.1	50S_ribosomal_protein_L11_[Bacillus_cereus]	CPTF_Cu	7701743	222714.5572	2.891742262
UIJ66275.1	50S_ribosomal_protein_L11_[Bacillus_cereus]	CPTF_Fe	8073842	458428.533	5.677947786
UIJ66275.1	50S_ribosomal_protein_L11_[Bacillus_cereus]	CPTF_Mn	8299897.667	807608.6234	9.730344347
UIJ66275.1	50S_ribosomal_protein_L11_[Bacillus_cereus]	CPTF_Ni	7028627.967	293434.3712	4.174845683
UIJ66275.1	50S_ribosomal_protein_L11_[Bacillus_cereus]	CPTF_U	7571557.333	554381.7825	7.321899025
UIJ66275.1	50S_ribosomal_protein_L11_[Bacillus_cereus]	CPTF_metals_mix	6035185.667	334762.9767	5.546854649
UIJ66275.1	50S_ribosomal_protein_L11_[Bacillus_cereus]	CPTF_zcontrol	8254556.667	791243.7428	9.585538931
UIJ66276.1	transcription_termination/antitermination_protein_NusG_[Bacillus_cereus]	CPTF_Al	1086645.033	166094.0803	15.28503561
UIJ66276.1	transcription_termination/antitermination_protein_NusG_[Bacillus_cereus]	CPTF_Cd	1403628.067	115718.2578	8.244225127
UIJ66276.1	transcription_termination/antitermination_protein_NusG_[Bacillus_cereus]	CPTF_Co	1489860.2	52972.33844	3.555524098
UIJ66276.1	transcription_termination/antitermination_protein_NusG_[Bacillus_cereus]	CPTF_Cu	1434165.367	91426.40463	6.374885823
UIJ66276.1	transcription_termination/antitermination_protein_NusG_[Bacillus_cereus]	CPTF_Fe	1248574.4	76295.65041	6.110621074
UIJ66276.1	transcription_termination/antitermination_protein_NusG_[Bacillus_cereus]	CPTF_Mn	1298616.567	180500.006	13.89940731
UIJ66276.1	transcription_termination/antitermination_protein_NusG_[Bacillus_cereus]	CPTF_Ni	1431992.9	35618.30005	2.487323788
UIJ66276.1	transcription_termination/antitermination_protein_NusG_[Bacillus_cereus]	CPTF_U	1268200.167	582931.1708	45.96523373
UIJ66276.1	transcription_termination/antitermination_protein_NusG_[Bacillus_cereus]	CPTF_metals_mix	1692838.633	110431.8982	6.523474596
UIJ66276.1	transcription_termination/antitermination_protein_NusG_[Bacillus_cereus]	CPTF_zcontrol	1064999.833	75108.20064	7.052414309
UIJ66279.1	RNA_polymerase_sporulation_sigma_factor_SigH_[Bacillus_cereus]	CPTF_Al	2775525.2	323065.6914	11.63980393
UIJ66279.1	RNA_polymerase_sporulation_sigma_factor_SigH_[Bacillus_cereus]	CPTF_Cd	3022141.633	533239.3811	17.64442061
UIJ66279.1	RNA_polymerase_sporulation_sigma_factor_SigH_[Bacillus_cereus]	CPTF_Co	2949464.13	259599.9959	8.801598677
UIJ66279.1	RNA_polymerase_sporulation_sigma_factor_SigH_[Bacillus_cereus]	CPTF_Cu	2577818.2	433764.5459	16.82680904
UIJ66279.1	RNA_polymerase_sporulation_sigma_factor_SigH_[Bacillus_cereus]	CPTF_Fe	3121740.8	513948.5834	16.4635252
UIJ66279.1	RNA_polymerase_sporulation_sigma_factor_SigH_[Bacillus_cereus]	CPTF_Mn	3043177.2	398039.192	13.07972444
UIJ66279.1	RNA_polymerase_sporulation_sigma_factor_SigH_[Bacillus_cereus]	CPTF_Ni	2813332.533	348473.9012	12.3865166
UIJ66279.1	RNA_polymerase_sporulation_sigma_factor_SigH_[Bacillus_cereus]	CPTF_U	2804783.433	590751.2421	21.06227651
UIJ66279.1	RNA_polymerase_sporulation_sigma_factor_SigH_[Bacillus_cereus]	CPTF_metals_mix	2123301.233	656949.6409	30.94001127
UIJ66279.1	RNA_polymerase_sporulation_sigma_factor_SigH_[Bacillus_cereus]	CPTF_zcontrol	2880003	483459.146	16.78675842
UIJ66281.1	23S_rRNA_(guanosine(2251)-2'-O)-methyltransferase_RlmB_[Bacillus_cereus]	CPTF_Al	0	0	0
UIJ66281.1	23S_rRNA_(guanosine(2251)-2'-O)-methyltransferase_RlmB_[Bacillus_cereus]	CPTF_Cd	187899	23082.65676	12.28460862
UIJ66281.1	23S_rRNA_(guanosine(2251)-2'-O)-methyltransferase_RlmB_[Bacillus_cereus]	CPTF_Co	124658.7333	53456.92381	42.8826143
UIJ66281.1	23S_rRNA_(guanosine(2251)-2'-O)-methyltransferase_RlmB_[Bacillus_cereus]	CPTF_Cu	86330.4	149528.639	173.2050808
UIJ66281.1	23S_rRNA_(guanosine(2251)-2'-O)-methyltransferase_RlmB_[Bacillus_cereus]	CPTF_Fe	92179	82710.56324	89.72820625
UIJ66281.1	23S_rRNA_(guanosine(2251)-2'-O)-methyltransferase_RlmB_[Bacillus_cereus]	CPTF_Mn	78514.33333	135990.8145	173.2050808
UIJ66281.1	23S_rRNA_(guanosine(2251)-2'-O)-methyltransferase_RlmB_[Bacillus_cereus]	CPTF_Ni	89056.66667	78390.70221	88.02339582
UIJ66281.1	23S_rRNA_(guanosine(2251)-2'-O)-methyltransferase_RlmB_[Bacillus_cereus]	CPTF_U	60117.96667	52080.4741	86.63046504
UIJ66281.1	23S_rRNA_(guanosine(2251)-2'-O)-methyltransferase_RlmB_[Bacillus_cereus]	CPTF_metals_mix	707683.1333	22696.23403	3.207118125
UIJ66281.1	23S_rRNA_(guanosine(2251)-2'-O)-methyltransferase_RlmB_[Bacillus_cereus]	CPTF_zcontrol	74572.3	66576.41747	89.27767746
UIJ66283.1	cysteine--tRNA_ligase_[Bacillus_cereus]	CPTF_Al	1562262.533	321305.9889	20.56670899
UIJ66283.1	cysteine--tRNA_ligase_[Bacillus_cereus]	CPTF_Cd	1730147.567	513162.1744	29.66002347
UIJ66283.1	cysteine--tRNA_ligase_[Bacillus_cereus]	CPTF_Co	1982873.167	129374.5365	6.524599691
UIJ66283.1	cysteine--tRNA_ligase_[Bacillus_cereus]	CPTF_Cu	1837642.9	287324.8292	15.63550944
UIJ66283.1	cysteine--tRNA_ligase_[Bacillus_cereus]	CPTF_Fe	1710965.933	220684.2162	12.89822386
UIJ66283.1	cysteine--tRNA_ligase_[Bacillus_cereus]	CPTF_Mn	1651987.6	523526.2738	31.69069028
UIJ66283.1	cysteine--tRNA_ligase_[Bacillus_cereus]	CPTF_Ni	2026087.167	33337.50616	1.645413223
UIJ66283.1	cysteine--tRNA_ligase_[Bacillus_cereus]	CPTF_U	1921427.8	265380.0446	13.81160638
UIJ66283.1	cysteine--tRNA_ligase_[Bacillus_cereus]	CPTF_metals_mix	2866231.067	188771.5038	6.586053232
UIJ66283.1	cysteine--tRNA_ligase_[Bacillus_cereus]	CPTF_zcontrol	1001446.6	884.8106916	0.088353257
UIJ66285.1	glutamate--tRNA_ligase_[Bacillus_cereus]	CPTF_Al	5744637.567	64116.82014	1.11611602
UIJ66285.1	glutamate--tRNA_ligase_[Bacillus_cereus]	CPTF_Cd	5509804.6	451067.6774	8.186636553
UIJ66285.1	glutamate--tRNA_ligase_[Bacillus_cereus]	CPTF_Co	5307619.833	657236.5794	12.38288725
UIJ66285.1	glutamate--tRNA_ligase_[Bacillus_cereus]	CPTF_Cu	5500089.767	285654.3457	5.19363061
UIJ66285.1	glutamate--tRNA_ligase_[Bacillus_cereus]	CPTF_Fe	5265954.233	207807.802	3.946251577
UIJ66285.1	glutamate--tRNA_ligase_[Bacillus_cereus]	CPTF_Mn	5103693.7	338963.9701	6.641542186
UIJ66285.1	glutamate--tRNA_ligase_[Bacillus_cereus]	CPTF_Ni	4930607.5	482741.2103	9.790704498
UIJ66285.1	glutamate--tRNA_ligase_[Bacillus_cereus]	CPTF_U	4752505.2	411922.0341	8.667471507
UIJ66285.1	glutamate--tRNA_ligase_[Bacillus_cereus]	CPTF_metals_mix	6029713.067	449336.2459	7.452033636

UIJ66285.1	glutamate--tRNA_ligase_[Bacillus_cereus]	CPTF_zcontrol	5188578.533	112803.9299	2.174081575
UIJ66286.1	2-C-methyl-D-erythritol_2,4-cyclodiphosphate_synthase_[Bacillus_cereus]	CPTF_Al	49210.4	44289.34993	89.99997953
UIJ66286.1	2-C-methyl-D-erythritol_2,4-cyclodiphosphate_synthase_[Bacillus_cereus]	CPTF_Cd	35746	61913.88817	173.2050808
UIJ66286.1	2-C-methyl-D-erythritol_2,4-cyclodiphosphate_synthase_[Bacillus_cereus]	CPTF_Co	108470.3333	6587.019837	6.072646441
UIJ66286.1	2-C-methyl-D-erythritol_2,4-cyclodiphosphate_synthase_[Bacillus_cereus]	CPTF_Cu	72357	62839.12546	86.84595197
UIJ66286.1	2-C-methyl-D-erythritol_2,4-cyclodiphosphate_synthase_[Bacillus_cereus]	CPTF_Fe	56229.26667	48775.85055	86.74459661
UIJ66286.1	2-C-methyl-D-erythritol_2,4-cyclodiphosphate_synthase_[Bacillus_cereus]	CPTF_Mn	73438.33333	63716.27888	86.76160799
UIJ66286.1	2-C-methyl-D-erythritol_2,4-cyclodiphosphate_synthase_[Bacillus_cereus]	CPTF_Ni	33026.8	29662.43218	89.8132189
UIJ66286.1	2-C-methyl-D-erythritol_2,4-cyclodiphosphate_synthase_[Bacillus_cereus]	CPTF_U	0	0	0
UIJ66286.1	2-C-methyl-D-erythritol_2,4-cyclodiphosphate_synthase_[Bacillus_cereus]	CPTF_metals_mix	0	0	0
UIJ66286.1	2-C-methyl-D-erythritol_2,4-cyclodiphosphate_synthase_[Bacillus_cereus]	CPTF_zcontrol	0	0	0
UIJ66288.1	PIN/TRAM_domain-containing_protein_[Bacillus_cereus]	CPTF_Al	19423.96667	17792.12573	91.59882755
UIJ66288.1	PIN/TRAM_domain-containing_protein_[Bacillus_cereus]	CPTF_Cd	0	0	0
UIJ66288.1	PIN/TRAM_domain-containing_protein_[Bacillus_cereus]	CPTF_Co	5349.1	9264.912975	173.2050808
UIJ66288.1	PIN/TRAM_domain-containing_protein_[Bacillus_cereus]	CPTF_Cu	0	0	0
UIJ66288.1	PIN/TRAM_domain-containing_protein_[Bacillus_cereus]	CPTF_Fe	7170.2	12419.1507	173.2050808
UIJ66288.1	PIN/TRAM_domain-containing_protein_[Bacillus_cereus]	CPTF_Mn	0	0	0
UIJ66288.1	PIN/TRAM_domain-containing_protein_[Bacillus_cereus]	CPTF_Ni	13200.8	22864.4563	173.2050808
UIJ66288.1	PIN/TRAM_domain-containing_protein_[Bacillus_cereus]	CPTF_U	12053.8	20877.79402	173.2050808
UIJ66288.1	PIN/TRAM_domain-containing_protein_[Bacillus_cereus]	CPTF_metals_mix	87803.6	68543.12841	78.06414362
UIJ66288.1	PIN/TRAM_domain-containing_protein_[Bacillus_cereus]	CPTF_zcontrol	0	0	0
UIJ66289.1	DNA_integrity_scanning_diadenylate_cyclase_DisA_[Bacillus_cereus]	CPTF_Al	5240.666667	9077.100932	173.2050808
UIJ66289.1	DNA_integrity_scanning_diadenylate_cyclase_DisA_[Bacillus_cereus]	CPTF_Cd	17522.6	19214.0964	109.6532273
UIJ66289.1	DNA_integrity_scanning_diadenylate_cyclase_DisA_[Bacillus_cereus]	CPTF_Co	33975.86667	30423.44391	89.54427627
UIJ66289.1	DNA_integrity_scanning_diadenylate_cyclase_DisA_[Bacillus_cereus]	CPTF_Cu	31659.23333	28879.08483	91.21852233
UIJ66289.1	DNA_integrity_scanning_diadenylate_cyclase_DisA_[Bacillus_cereus]	CPTF_Fe	0	0	0
UIJ66289.1	DNA_integrity_scanning_diadenylate_cyclase_DisA_[Bacillus_cereus]	CPTF_Mn	0	0	0
UIJ66289.1	DNA_integrity_scanning_diadenylate_cyclase_DisA_[Bacillus_cereus]	CPTF_Ni	0	0	0
UIJ66289.1	DNA_integrity_scanning_diadenylate_cyclase_DisA_[Bacillus_cereus]	CPTF_U	0	0	0
UIJ66289.1	DNA_integrity_scanning_diadenylate_cyclase_DisA_[Bacillus_cereus]	CPTF_metals_mix	83366	21492.43714	25.78081849
UIJ66289.1	DNA_integrity_scanning_diadenylate_cyclase_DisA_[Bacillus_cereus]	CPTF_zcontrol	10397.56667	18009.11374	173.2050808
UIJ66290.1	DNA_repair_protein_RadA_[Bacillus_cereus]	CPTF_Al	0	0	0
UIJ66290.1	DNA_repair_protein_RadA_[Bacillus_cereus]	CPTF_Cd	0	0	0
UIJ66290.1	DNA_repair_protein_RadA_[Bacillus_cereus]	CPTF_Co	0	0	0
UIJ66290.1	DNA_repair_protein_RadA_[Bacillus_cereus]	CPTF_Cu	39770.1	37703.45478	94.80352019
UIJ66290.1	DNA_repair_protein_RadA_[Bacillus_cereus]	CPTF_Fe	0	0	0
UIJ66290.1	DNA_repair_protein_RadA_[Bacillus_cereus]	CPTF_Mn	11423.26667	19785.67826	173.2050808
UIJ66290.1	DNA_repair_protein_RadA_[Bacillus_cereus]	CPTF_Ni	0	0	0
UIJ66290.1	DNA_repair_protein_RadA_[Bacillus_cereus]	CPTF_U	19279.3	33392.72713	173.2050808
UIJ66290.1	DNA_repair_protein_RadA_[Bacillus_cereus]	CPTF_metals_mix	32785.76667	31250.66013	95.31776532
UIJ66290.1	DNA_repair_protein_RadA_[Bacillus_cereus]	CPTF_zcontrol	0	0	0
UIJ66291.1	ATP-dependent_protease_ATP-binding_subunit_ClpC_[Bacillus_cereus]	CPTF_Al	8182831.767	319786.7108	3.908020107
UIJ66291.1	ATP-dependent_protease_ATP-binding_subunit_ClpC_[Bacillus_cereus]	CPTF_Cd	9753838.633	626042.5668	6.418422432
UIJ66291.1	ATP-dependent_protease_ATP-binding_subunit_ClpC_[Bacillus_cereus]	CPTF_Co	8500956.867	507453.5332	5.96936958
UIJ66291.1	ATP-dependent_protease_ATP-binding_subunit_ClpC_[Bacillus_cereus]	CPTF_Cu	8180105.833	514973.6299	6.295439697
UIJ66291.1	ATP-dependent_protease_ATP-binding_subunit_ClpC_[Bacillus_cereus]	CPTF_Fe	8234696.033	412445.6076	5.008631842
UIJ66291.1	ATP-dependent_protease_ATP-binding_subunit_ClpC_[Bacillus_cereus]	CPTF_Mn	8453418.533	1195906.897	14.14702102
UIJ66291.1	ATP-dependent_protease_ATP-binding_subunit_ClpC_[Bacillus_cereus]	CPTF_Ni	7250469.733	187475.4021	2.585700086
UIJ66291.1	ATP-dependent_protease_ATP-binding_subunit_ClpC_[Bacillus_cereus]	CPTF_U	6988018.967	469417.1336	6.717456491
UIJ66291.1	ATP-dependent_protease_ATP-binding_subunit_ClpC_[Bacillus_cereus]	CPTF_metals_mix	16036268.47	1770137.264	11.03833643
UIJ66291.1	ATP-dependent_protease_ATP-binding_subunit_ClpC_[Bacillus_cereus]	CPTF_zcontrol	6850404.367	1156187.287	16.87765021
UIJ66292.1	protein_arginine_kinase_[Bacillus_cereus]	CPTF_Al	48264	83595.70018	173.2050808
UIJ66292.1	protein_arginine_kinase_[Bacillus_cereus]	CPTF_Cd	151404.3333	160197.8637	105.807978
UIJ66292.1	protein_arginine_kinase_[Bacillus_cereus]	CPTF_Co	158937.3333	27071.97552	17.03311296
UIJ66292.1	protein_arginine_kinase_[Bacillus_cereus]	CPTF_Cu	38631	66910.85475	173.2050808
UIJ66292.1	protein_arginine_kinase_[Bacillus_cereus]	CPTF_Fe	211057.6667	206448.2416	97.81603523
UIJ66292.1	protein_arginine_kinase_[Bacillus_cereus]	CPTF_Mn	98219.6	122948.1475	125.1767952
UIJ66292.1	protein_arginine_kinase_[Bacillus_cereus]	CPTF_Ni	67280.33333	72685.54947	108.0338724
UIJ66292.1	protein_arginine_kinase_[Bacillus_cereus]	CPTF_U	199164.3333	20150.72188	10.1176358
UIJ66292.1	protein_arginine_kinase_[Bacillus_cereus]	CPTF_metals_mix	82301	71350.99479	86.69517355
UIJ66292.1	protein_arginine_kinase_[Bacillus_cereus]	CPTF_zcontrol	64941.66667	112482.2662	173.2050808

UIJ66295.1	lysine--tRNA_ligase_[Bacillus_cereus]	CPTF_Al	5691016.4	176705.9045	3.104997281
UIJ66295.1	lysine--tRNA_ligase_[Bacillus_cereus]	CPTF_Cd	5567921.667	615859.0654	11.06084285
UIJ66295.1	lysine--tRNA_ligase_[Bacillus_cereus]	CPTF_Co	5806514.267	206571.3764	3.557579761
UIJ66295.1	lysine--tRNA_ligase_[Bacillus_cereus]	CPTF_Cu	5893340.833	474574.2779	8.052720712
UIJ66295.1	lysine--tRNA_ligase_[Bacillus_cereus]	CPTF_Fe	4628426.267	1102464.279	23.81941973
UIJ66295.1	lysine--tRNA_ligase_[Bacillus_cereus]	CPTF_Mn	5178586.433	884996.2702	17.08953363
UIJ66295.1	lysine--tRNA_ligase_[Bacillus_cereus]	CPTF_Ni	4680808.1	619319.3874	13.23103563
UIJ66295.1	lysine--tRNA_ligase_[Bacillus_cereus]	CPTF_U	4271273.133	723234.9147	16.93253726
UIJ66295.1	lysine--tRNA_ligase_[Bacillus_cereus]	CPTF_metals_mix	7656295.067	924975.9133	12.08124694
UIJ66295.1	lysine--tRNA_ligase_[Bacillus_cereus]	CPTF_zcontrol	4896405.567	192569.6362	3.932877569
UIJ66300.1	dihydropteroate_synthase_[Bacillus_cereus]	CPTF_Al	959817.6667	215835.4959	22.48713515
UIJ66300.1	dihydropteroate_synthase_[Bacillus_cereus]	CPTF_Cd	770548.7667	359969.3039	46.71596652
UIJ66300.1	dihydropteroate_synthase_[Bacillus_cereus]	CPTF_Co	1150699.2	60441.24951	5.252567266
UIJ66300.1	dihydropteroate_synthase_[Bacillus_cereus]	CPTF_Cu	1164383.667	55423.43222	4.759894338
UIJ66300.1	dihydropteroate_synthase_[Bacillus_cereus]	CPTF_Fe	824117.9667	206510.4695	25.05836274
UIJ66300.1	dihydropteroate_synthase_[Bacillus_cereus]	CPTF_Mn	651220.6667	124176.3583	19.06824593
UIJ66300.1	dihydropteroate_synthase_[Bacillus_cereus]	CPTF_Ni	996491.2333	25800.70174	2.589154915
UIJ66300.1	dihydropteroate_synthase_[Bacillus_cereus]	CPTF_U	791594.2667	131744.3521	16.64291388
UIJ66300.1	dihydropteroate_synthase_[Bacillus_cereus]	CPTF_metals_mix	925868.3333	89155.52126	9.629395244
UIJ66300.1	dihydropteroate_synthase_[Bacillus_cereus]	CPTF_zcontrol	1030593.4	121276.0423	11.76759353
UIJ66303.1	aminodeoxychorismate_synthase_component_I_[Bacillus_cereus]	CPTF_Al	11741.6	20337.04776	173.2050808
UIJ66303.1	aminodeoxychorismate_synthase_component_I_[Bacillus_cereus]	CPTF_Cd	0	0	0
UIJ66303.1	aminodeoxychorismate_synthase_component_I_[Bacillus_cereus]	CPTF_Co	0	0	0
UIJ66303.1	aminodeoxychorismate_synthase_component_I_[Bacillus_cereus]	CPTF_Cu	20705.56667	18530.50549	89.49528304
UIJ66303.1	aminodeoxychorismate_synthase_component_I_[Bacillus_cereus]	CPTF_Fe	21647.46667	37494.51212	173.2050808
UIJ66303.1	aminodeoxychorismate_synthase_component_I_[Bacillus_cereus]	CPTF_Mn	18001.43333	31179.39714	173.2050808
UIJ66303.1	aminodeoxychorismate_synthase_component_I_[Bacillus_cereus]	CPTF_Ni	74629.13333	70515.95835	94.488513
UIJ66303.1	aminodeoxychorismate_synthase_component_I_[Bacillus_cereus]	CPTF_U	0	0	0
UIJ66303.1	aminodeoxychorismate_synthase_component_I_[Bacillus_cereus]	CPTF_metals_mix	33989.86667	29675.295	87.3063001
UIJ66303.1	aminodeoxychorismate_synthase_component_I_[Bacillus_cereus]	CPTF_zcontrol	0	0	0
UIJ66304.1	cysteine_synthase_A_[Bacillus_cereus]	CPTF_Al	3284055.5	592477.913	18.04104446
UIJ66304.1	cysteine_synthase_A_[Bacillus_cereus]	CPTF_Cd	4128218.6	513825.3579	12.4466606
UIJ66304.1	cysteine_synthase_A_[Bacillus_cereus]	CPTF_Co	2878979.767	300822.5412	10.44892863
UIJ66304.1	cysteine_synthase_A_[Bacillus_cereus]	CPTF_Cu	3218627	206949.6096	6.429748138
UIJ66304.1	cysteine_synthase_A_[Bacillus_cereus]	CPTF_Fe	2774492.067	208995.1328	7.532734922
UIJ66304.1	cysteine_synthase_A_[Bacillus_cereus]	CPTF_Mn	4185839.7	2382946.612	56.9287594
UIJ66304.1	cysteine_synthase_A_[Bacillus_cereus]	CPTF_Ni	2032939.133	352349.7349	17.33203563
UIJ66304.1	cysteine_synthase_A_[Bacillus_cereus]	CPTF_U	1887341.033	403278.4117	21.36754326
UIJ66304.1	cysteine_synthase_A_[Bacillus_cereus]	CPTF_metals_mix	12053683.5	1383046.507	11.47405693
UIJ66304.1	cysteine_synthase_A_[Bacillus_cereus]	CPTF_zcontrol	2430207.667	399486.9203	16.43838614
UIJ66305.1	redox-regulated_molecular_chaperone_HslO_[Bacillus_cereus]	CPTF_Al	57541.4	14918.93312	25.92730299
UIJ66305.1	redox-regulated_molecular_chaperone_HslO_[Bacillus_cereus]	CPTF_Cd	104267.7667	52796.08646	50.6350986
UIJ66305.1	redox-regulated_molecular_chaperone_HslO_[Bacillus_cereus]	CPTF_Co	149848.5333	45923.44384	30.64657546
UIJ66305.1	redox-regulated_molecular_chaperone_HslO_[Bacillus_cereus]	CPTF_Cu	121822.8667	78945.88384	64.80383035
UIJ66305.1	redox-regulated_molecular_chaperone_HslO_[Bacillus_cereus]	CPTF_Fe	88215.7	77326.68472	87.65637491
UIJ66305.1	redox-regulated_molecular_chaperone_HslO_[Bacillus_cereus]	CPTF_Mn	185735.6333	65112.15595	35.05636198
UIJ66305.1	redox-regulated_molecular_chaperone_HslO_[Bacillus_cereus]	CPTF_Ni	54050.33333	93617.9235	173.2050808
UIJ66305.1	redox-regulated_molecular_chaperone_HslO_[Bacillus_cereus]	CPTF_U	63549.93333	71313.23222	112.2160614
UIJ66305.1	redox-regulated_molecular_chaperone_HslO_[Bacillus_cereus]	CPTF_metals_mix	626700.8333	44045.19866	7.028105966
UIJ66305.1	redox-regulated_molecular_chaperone_HslO_[Bacillus_cereus]	CPTF_zcontrol	161996.5333	51994.65057	32.09615015
UIJ66307.1	ATP-dependent_zinc_metalloprotease_FtsH_[Bacillus_cereus]	CPTF_Al	8134400.533	553768.7325	6.807738692
UIJ66307.1	ATP-dependent_zinc_metalloprotease_FtsH_[Bacillus_cereus]	CPTF_Cd	9075668.267	293261.1088	3.231289424
UIJ66307.1	ATP-dependent_zinc_metalloprotease_FtsH_[Bacillus_cereus]	CPTF_Co	7812981.5	438166.1076	5.608180534
UIJ66307.1	ATP-dependent_zinc_metalloprotease_FtsH_[Bacillus_cereus]	CPTF_Cu	7312826.4	304838.2607	4.168542285
UIJ66307.1	ATP-dependent_zinc_metalloprotease_FtsH_[Bacillus_cereus]	CPTF_Fe	8218777.233	439498.6193	5.347493998
UIJ66307.1	ATP-dependent_zinc_metalloprotease_FtsH_[Bacillus_cereus]	CPTF_Mn	7916627.367	554063.9824	6.998737679
UIJ66307.1	ATP-dependent_zinc_metalloprotease_FtsH_[Bacillus_cereus]	CPTF_Ni	7944416.933	521034.7351	6.558501895
UIJ66307.1	ATP-dependent_zinc_metalloprotease_FtsH_[Bacillus_cereus]	CPTF_U	8623286.933	759467.5491	8.807170107
UIJ66307.1	ATP-dependent_zinc_metalloprotease_FtsH_[Bacillus_cereus]	CPTF_metals_mix	7988001.6	667571.234	8.357174515
UIJ66307.1	ATP-dependent_zinc_metalloprotease_FtsH_[Bacillus_cereus]	CPTF_zcontrol	8197784.433	103116.7997	1.257861811
UIJ66308.1	hypoxanthine_phosphoribosyltransferase_[Bacillus_cereus]	CPTF_Al	840983.2667	162210.8892	19.28824218

UIJ66308.1	hypoxanthine_phosphoribosyltransferase_[Bacillus_cereus]	CPTF_Cd	1124617.8	357824.6467	31.81744471
UIJ66308.1	hypoxanthine_phosphoribosyltransferase_[Bacillus_cereus]	CPTF_Co	1126911.467	121994.2874	10.82554318
UIJ66308.1	hypoxanthine_phosphoribosyltransferase_[Bacillus_cereus]	CPTF_Cu	1158886.667	145303.1369	12.53816625
UIJ66308.1	hypoxanthine_phosphoribosyltransferase_[Bacillus_cereus]	CPTF_Fe	774138.3333	534114.2028	68.99467186
UIJ66308.1	hypoxanthine_phosphoribosyltransferase_[Bacillus_cereus]	CPTF_Mn	1265249.333	846428.0152	66.89811983
UIJ66308.1	hypoxanthine_phosphoribosyltransferase_[Bacillus_cereus]	CPTF_Ni	706015.6667	522852.8375	74.0568322
UIJ66308.1	hypoxanthine_phosphoribosyltransferase_[Bacillus_cereus]	CPTF_U	534102.5667	62551.38801	11.71149362
UIJ66308.1	hypoxanthine_phosphoribosyltransferase_[Bacillus_cereus]	CPTF_metals_mix	5240969.7	1059660.924	20.21879509
UIJ66308.1	hypoxanthine_phosphoribosyltransferase_[Bacillus_cereus]	CPTF_zcontrol	992715	247331.0468	24.91460759
UIJ66309.1	tRNA_lysidine(34)_synthetase_TilS_[Bacillus_cereus]	CPTF_Al	241797.6667	210101.7122	86.89153832
UIJ66309.1	tRNA_lysidine(34)_synthetase_TilS_[Bacillus_cereus]	CPTF_Cd	418465.3333	28482.54687	6.806429256
UIJ66309.1	tRNA_lysidine(34)_synthetase_TilS_[Bacillus_cereus]	CPTF_Co	300533.3333	260329.7827	86.62259849
UIJ66309.1	tRNA_lysidine(34)_synthetase_TilS_[Bacillus_cereus]	CPTF_Cu	271378.6667	235296.2233	86.70402364
UIJ66309.1	tRNA_lysidine(34)_synthetase_TilS_[Bacillus_cereus]	CPTF_Fe	450218.3333	40004.19331	8.885509618
UIJ66309.1	tRNA_lysidine(34)_synthetase_TilS_[Bacillus_cereus]	CPTF_Mn	315880.6667	296443.0082	93.84651848
UIJ66309.1	tRNA_lysidine(34)_synthetase_TilS_[Bacillus_cereus]	CPTF_Ni	383315	119311.1517	31.12613692
UIJ66309.1	tRNA_lysidine(34)_synthetase_TilS_[Bacillus_cereus]	CPTF_U	308247.3333	271344.7418	88.0282528
UIJ66309.1	tRNA_lysidine(34)_synthetase_TilS_[Bacillus_cereus]	CPTF_metals_mix	0	0	0
UIJ66309.1	tRNA_lysidine(34)_synthetase_TilS_[Bacillus_cereus]	CPTF_zcontrol	365957.3333	87052.17995	23.78752167
UIJ66311.1	S1_domain-containing_RNA-binding_protein_[Bacillus_cereus]	CPTF_Al	284173.6667	15359.80359	5.405076329
UIJ66311.1	S1_domain-containing_RNA-binding_protein_[Bacillus_cereus]	CPTF_Cd	414105	36582.43678	8.834096855
UIJ66311.1	S1_domain-containing_RNA-binding_protein_[Bacillus_cereus]	CPTF_Co	387656	54746.80626	14.12252261
UIJ66311.1	S1_domain-containing_RNA-binding_protein_[Bacillus_cereus]	CPTF_Cu	342384.6667	48028.84818	14.02774506
UIJ66311.1	S1_domain-containing_RNA-binding_protein_[Bacillus_cereus]	CPTF_Fe	383194	118482.1445	30.91962414
UIJ66311.1	S1_domain-containing_RNA-binding_protein_[Bacillus_cereus]	CPTF_Mn	389267	80591.27513	20.70334118
UIJ66311.1	S1_domain-containing_RNA-binding_protein_[Bacillus_cereus]	CPTF_Ni	395971.3333	60313.8694	15.23187775
UIJ66311.1	S1_domain-containing_RNA-binding_protein_[Bacillus_cereus]	CPTF_U	462557.3333	51980.50774	11.23763564
UIJ66311.1	S1_domain-containing_RNA-binding_protein_[Bacillus_cereus]	CPTF_metals_mix	1125466	49011.91157	4.354810503
UIJ66311.1	S1_domain-containing_RNA-binding_protein_[Bacillus_cereus]	CPTF_zcontrol	407973.6667	78542.63553	19.25188853
UIJ66316.1	nucleoside_triphosphate_pyrophosphohydrolase_[Bacillus_cereus]	CPTF_Al	90537.66667	78705.13666	86.93082068
UIJ66316.1	nucleoside_triphosphate_pyrophosphohydrolase_[Bacillus_cereus]	CPTF_Cd	128300.6267	84956.81797	66.21699378
UIJ66316.1	nucleoside_triphosphate_pyrophosphohydrolase_[Bacillus_cereus]	CPTF_Co	148126.8333	30073.58348	20.30258988
UIJ66316.1	nucleoside_triphosphate_pyrophosphohydrolase_[Bacillus_cereus]	CPTF_Cu	133319.1	6466.686952	4.850533008
UIJ66316.1	nucleoside_triphosphate_pyrophosphohydrolase_[Bacillus_cereus]	CPTF_Fe	156934.5	80396.12579	51.22909608
UIJ66316.1	nucleoside_triphosphate_pyrophosphohydrolase_[Bacillus_cereus]	CPTF_Mn	39723.66667	68803.40893	173.2050808
UIJ66316.1	nucleoside_triphosphate_pyrophosphohydrolase_[Bacillus_cereus]	CPTF_Ni	84861.9	43687.72253	51.48096204
UIJ66316.1	nucleoside_triphosphate_pyrophosphohydrolase_[Bacillus_cereus]	CPTF_U	98427.66667	85879.93642	87.25182597
UIJ66316.1	nucleoside_triphosphate_pyrophosphohydrolase_[Bacillus_cereus]	CPTF_metals_mix	138086.2	55392.54632	40.11446931
UIJ66316.1	nucleoside_triphosphate_pyrophosphohydrolase_[Bacillus_cereus]	CPTF_zcontrol	115504.1667	59944.5004	51.89812812
UIJ66319.1	transcription-repair_coupling_factor_[Bacillus_cereus]	CPTF_Al	1834092.067	296991.6311	16.19284203
UIJ66319.1	transcription-repair_coupling_factor_[Bacillus_cereus]	CPTF_Cd	1874258.833	155945.3681	8.320375252
UIJ66319.1	transcription-repair_coupling_factor_[Bacillus_cereus]	CPTF_Co	1695578.133	317311.1489	18.71403875
UIJ66319.1	transcription-repair_coupling_factor_[Bacillus_cereus]	CPTF_Cu	1591697.933	76834.48174	4.827202457
UIJ66319.1	transcription-repair_coupling_factor_[Bacillus_cereus]	CPTF_Fe	1839366.567	111147.2369	6.042690939
UIJ66319.1	transcription-repair_coupling_factor_[Bacillus_cereus]	CPTF_Mn	1586434	299557.2331	18.88242644
UIJ66319.1	transcription-repair_coupling_factor_[Bacillus_cereus]	CPTF_Ni	1451370.1	121506.1241	8.37182219
UIJ66319.1	transcription-repair_coupling_factor_[Bacillus_cereus]	CPTF_U	1542448.233	176452.9284	11.43979581
UIJ66319.1	transcription-repair_coupling_factor_[Bacillus_cereus]	CPTF_metals_mix	1909070.233	550487.0816	28.8353499
UIJ66319.1	transcription-repair_coupling_factor_[Bacillus_cereus]	CPTF_zcontrol	1694465.7	132949.1398	7.846080319
UIJ66322.1	ribose-phosphate_diphosphokinase_[Bacillus_cereus]	CPTF_Al	6615252.367	344644.561	5.209847515
UIJ66322.1	ribose-phosphate_diphosphokinase_[Bacillus_cereus]	CPTF_Cd	6639708.167	31347.61067	0.472123321
UIJ66322.1	ribose-phosphate_diphosphokinase_[Bacillus_cereus]	CPTF_Co	6715746.133	486664.9316	7.246624901
UIJ66322.1	ribose-phosphate_diphosphokinase_[Bacillus_cereus]	CPTF_Cu	6603156.8	291720.9958	4.417901992
UIJ66322.1	ribose-phosphate_diphosphokinase_[Bacillus_cereus]	CPTF_Fe	6767255	103297.3661	1.526429343
UIJ66322.1	ribose-phosphate_diphosphokinase_[Bacillus_cereus]	CPTF_Mn	6607561.767	146726.9465	2.220591372
UIJ66322.1	ribose-phosphate_diphosphokinase_[Bacillus_cereus]	CPTF_Ni	7012937.533	231618.8036	3.302735873
UIJ66322.1	ribose-phosphate_diphosphokinase_[Bacillus_cereus]	CPTF_U	7621899.667	287546.9724	3.772641795
UIJ66322.1	ribose-phosphate_diphosphokinase_[Bacillus_cereus]	CPTF_metals_mix	7481221.353	629244.9318	8.410992031
UIJ66322.1	ribose-phosphate_diphosphokinase_[Bacillus_cereus]	CPTF_zcontrol	7294535.667	197404.3558	2.70619495
UIJ66323.1	bifunctional_UDP-N-acetylglucosamine_diphosphorylase/glucosamine-1-phosphate_N-acetyltransferase_GlmU_[Bacillus_cereus]	CPTF_Al	71052.96667	2694.142465	3.79173818
UIJ66323.1	bifunctional_UDP-N-acetylglucosamine_diphosphorylase/glucosamine-1-phosphate_N-acetyltransferase_GlmU_[Bacillus_cereus]	CPTF_Cd	32220.7	5889.097089	18.27737165

UIJ66323.1	bifunctional_UDP-N-acetylglucosamine_diphosphorylase/glucosamine-1-phosphate_N-acetyltransferase_GlmU_[Bacillus_cereus]	CPTF_Co	63710.7	39533.25887	62.05120784
UIJ66323.1	bifunctional_UDP-N-acetylglucosamine_diphosphorylase/glucosamine-1-phosphate_N-acetyltransferase_GlmU_[Bacillus_cereus]	CPTF_Cu	13572.4	12924.83584	95.2288161
UIJ66323.1	bifunctional_UDP-N-acetylglucosamine_diphosphorylase/glucosamine-1-phosphate_N-acetyltransferase_GlmU_[Bacillus_cereus]	CPTF_Fe	66014.23333	35691.14069	54.06582625
UIJ66323.1	bifunctional_UDP-N-acetylglucosamine_diphosphorylase/glucosamine-1-phosphate_N-acetyltransferase_GlmU_[Bacillus_cereus]	CPTF_Mn	39173.36667	45473.82317	116.0835206
UIJ66323.1	bifunctional_UDP-N-acetylglucosamine_diphosphorylase/glucosamine-1-phosphate_N-acetyltransferase_GlmU_[Bacillus_cereus]	CPTF_Ni	54659.26667	29849.33374	54.60983207
UIJ66323.1	bifunctional_UDP-N-acetylglucosamine_diphosphorylase/glucosamine-1-phosphate_N-acetyltransferase_GlmU_[Bacillus_cereus]	CPTF_U	0	0	0
UIJ66323.1	bifunctional_UDP-N-acetylglucosamine_diphosphorylase/glucosamine-1-phosphate_N-acetyltransferase_GlmU_[Bacillus_cereus]	CPTF_metals_mix	38439.03333	7769.790737	20.21328338
UIJ66323.1	bifunctional_UDP-N-acetylglucosamine_diphosphorylase/glucosamine-1-phosphate_N-acetyltransferase_GlmU_[Bacillus_cereus]	CPTF_zcontrol	23856.46667	41320.61236	173.2050808
UIJ66325.1	pur_operon_repressor_[Bacillus_cereus]	CPTF_Al	1121699.267	142365.7118	12.69196799
UIJ66325.1	pur_operon_repressor_[Bacillus_cereus]	CPTF_Cd	1107250.667	48189.173	4.352146668
UIJ66325.1	pur_operon_repressor_[Bacillus_cereus]	CPTF_Co	1008675.633	7628.799468	0.756318406
UIJ66325.1	pur_operon_repressor_[Bacillus_cereus]	CPTF_Cu	998057.5	118211.741	11.84418143
UIJ66325.1	pur_operon_repressor_[Bacillus_cereus]	CPTF_Fe	1209790.067	31447.25815	2.599397946
UIJ66325.1	pur_operon_repressor_[Bacillus_cereus]	CPTF_Mn	997040.8667	139813.815	14.02287706
UIJ66325.1	pur_operon_repressor_[Bacillus_cereus]	CPTF_Ni	932294.9667	40088.92721	4.300026134
UIJ66325.1	pur_operon_repressor_[Bacillus_cereus]	CPTF_U	943608.1	272667.4387	28.89625881
UIJ66325.1	pur_operon_repressor_[Bacillus_cereus]	CPTF_metals_mix	862500.8	71616.87318	8.303397884
UIJ66325.1	pur_operon_repressor_[Bacillus_cereus]	CPTF_zcontrol	1162400.667	54215.29543	4.664079864
UIJ66329.1	16S_rRNA_(adenine(1518)-N(6)/adenine(1519)-N(6))-dimethyltransferase_RsmA_[Bacillus_cereus]	CPTF_Al	258777	21378.97558	8.261543948
UIJ66329.1	16S_rRNA_(adenine(1518)-N(6)/adenine(1519)-N(6))-dimethyltransferase_RsmA_[Bacillus_cereus]	CPTF_Cd	205779.3333	178241.2768	86.61767627
UIJ66329.1	16S_rRNA_(adenine(1518)-N(6)/adenine(1519)-N(6))-dimethyltransferase_RsmA_[Bacillus_cereus]	CPTF_Co	241005	72874.53434	30.23776865
UIJ66329.1	16S_rRNA_(adenine(1518)-N(6)/adenine(1519)-N(6))-dimethyltransferase_RsmA_[Bacillus_cereus]	CPTF_Cu	266804.6667	40761.115	15.27751201
UIJ66329.1	16S_rRNA_(adenine(1518)-N(6)/adenine(1519)-N(6))-dimethyltransferase_RsmA_[Bacillus_cereus]	CPTF_Fe	200809.6667	173978.2362	86.63837709
UIJ66329.1	16S_rRNA_(adenine(1518)-N(6)/adenine(1519)-N(6))-dimethyltransferase_RsmA_[Bacillus_cereus]	CPTF_Mn	90628	83991.45345	92.67715656
UIJ66329.1	16S_rRNA_(adenine(1518)-N(6)/adenine(1519)-N(6))-dimethyltransferase_RsmA_[Bacillus_cereus]	CPTF_Ni	83562.33333	144734.2069	173.2050808
UIJ66329.1	16S_rRNA_(adenine(1518)-N(6)/adenine(1519)-N(6))-dimethyltransferase_RsmA_[Bacillus_cereus]	CPTF_U	64276	111329.2977	173.2050808
UIJ66329.1	16S_rRNA_(adenine(1518)-N(6)/adenine(1519)-N(6))-dimethyltransferase_RsmA_[Bacillus_cereus]	CPTF_metals_mix	264180.3333	38876.75676	14.71599202
UIJ66329.1	16S_rRNA_(adenine(1518)-N(6)/adenine(1519)-N(6))-dimethyltransferase_RsmA_[Bacillus_cereus]	CPTF_zcontrol	159430.6667	23992.62364	15.04893892
UIJ66330.1	ribonuclease_M5_[Bacillus_cereus]	CPTF_Al	0	0	0
UIJ66330.1	ribonuclease_M5_[Bacillus_cereus]	CPTF_Cd	0	0	0
UIJ66330.1	ribonuclease_M5_[Bacillus_cereus]	CPTF_Co	76391.33333	66483.84956	87.03061808
UIJ66330.1	ribonuclease_M5_[Bacillus_cereus]	CPTF_Cu	0	0	0
UIJ66330.1	ribonuclease_M5_[Bacillus_cereus]	CPTF_Fe	82573	74620.11159	90.36865753
UIJ66330.1	ribonuclease_M5_[Bacillus_cereus]	CPTF_Mn	0	0	0
UIJ66330.1	ribonuclease_M5_[Bacillus_cereus]	CPTF_Ni	0	0	0
UIJ66330.1	ribonuclease_M5_[Bacillus_cereus]	CPTF_U	16360.23333	28336.75536	173.2050808
UIJ66330.1	ribonuclease_M5_[Bacillus_cereus]	CPTF_metals_mix	20168.76667	34933.32859	173.2050808
UIJ66330.1	ribonuclease_M5_[Bacillus_cereus]	CPTF_zcontrol	47170.36667	41972.69825	88.98107269
UIJ66332.1	methionine--tRNA_ligase_[Bacillus_cereus]	CPTF_Al	7851367.933	699116.3995	8.904389725
UIJ66332.1	methionine--tRNA_ligase_[Bacillus_cereus]	CPTF_Cd	8367586.133	687434.3743	8.215444255
UIJ66332.1	methionine--tRNA_ligase_[Bacillus_cereus]	CPTF_Co	7415239.667	307962.0288	4.153096092
UIJ66332.1	methionine--tRNA_ligase_[Bacillus_cereus]	CPTF_Cu	7440875.5	726099.9561	9.758259712
UIJ66332.1	methionine--tRNA_ligase_[Bacillus_cereus]	CPTF_Fe	7631062.7	498072.3599	6.526906927
UIJ66332.1	methionine--tRNA_ligase_[Bacillus_cereus]	CPTF_Mn	7871774.333	198254.8368	2.518553358
UIJ66332.1	methionine--tRNA_ligase_[Bacillus_cereus]	CPTF_Ni	7614233.967	398146.7914	5.228980265
UIJ66332.1	methionine--tRNA_ligase_[Bacillus_cereus]	CPTF_U	8072055.3	812762.7097	10.06884467
UIJ66332.1	methionine--tRNA_ligase_[Bacillus_cereus]	CPTF_metals_mix	8795839.7	522105.6128	5.935824556
UIJ66332.1	methionine--tRNA_ligase_[Bacillus_cereus]	CPTF_zcontrol	7857608.367	683488.4469	8.698428517
UIJ66333.1	AbrB/MazE/SpoVT_family_DNA-binding_domain-containing_protein_[Bacillus_cereus]	CPTF_Al	2821114.667	316973.4593	11.23575242
UIJ66333.1	AbrB/MazE/SpoVT_family_DNA-binding_domain-containing_protein_[Bacillus_cereus]	CPTF_Cd	3366685.667	294128.7599	8.736448515
UIJ66333.1	AbrB/MazE/SpoVT_family_DNA-binding_domain-containing_protein_[Bacillus_cereus]	CPTF_Co	3271199.667	404708.3501	12.37186327
UIJ66333.1	AbrB/MazE/SpoVT_family_DNA-binding_domain-containing_protein_[Bacillus_cereus]	CPTF_Cu	3055219.533	37878.28019	1.239789147
UIJ66333.1	AbrB/MazE/SpoVT_family_DNA-binding_domain-containing_protein_[Bacillus_cereus]	CPTF_Fe	2943365	307215.8456	10.43757215
UIJ66333.1	AbrB/MazE/SpoVT_family_DNA-binding_domain-containing_protein_[Bacillus_cereus]	CPTF_Mn	3729900.467	1644200.155	44.08160942
UIJ66333.1	AbrB/MazE/SpoVT_family_DNA-binding_domain-containing_protein_[Bacillus_cereus]	CPTF_Ni	2860892.233	59472.38883	2.07880563
UIJ66333.1	AbrB/MazE/SpoVT_family_DNA-binding_domain-containing_protein_[Bacillus_cereus]	CPTF_U	2827873.633	524393.4354	18.54373651
UIJ66333.1	AbrB/MazE/SpoVT_family_DNA-binding_domain-containing_protein_[Bacillus_cereus]	CPTF_metals_mix	8843962	613591.8779	6.937975061
UIJ66333.1	AbrB/MazE/SpoVT_family_DNA-binding_domain-containing_protein_[Bacillus_cereus]	CPTF_zcontrol	2993217.833	244276.788	8.161009375
UIJ66334.1	16S_rRNA_(cytidine(1402)-2'-O)-methyltransferase_[Bacillus_cereus]	CPTF_Al	0	0	0
UIJ66334.1	16S_rRNA_(cytidine(1402)-2'-O)-methyltransferase_[Bacillus_cereus]	CPTF_Cd	0	0	0
UIJ66334.1	16S_rRNA_(cytidine(1402)-2'-O)-methyltransferase_[Bacillus_cereus]	CPTF_Co	0	0	0

UIJ66334.1	16S_rRNA_(cytidine(1402)-2'-O)-methyltransferase_[Bacillus_cereus]	CPTF_Cu	0	0	0
UIJ66334.1	16S_rRNA_(cytidine(1402)-2'-O)-methyltransferase_[Bacillus_cereus]	CPTF_Fe	0	0	0
UIJ66334.1	16S_rRNA_(cytidine(1402)-2'-O)-methyltransferase_[Bacillus_cereus]	CPTF_Mn	0	0	0
UIJ66334.1	16S_rRNA_(cytidine(1402)-2'-O)-methyltransferase_[Bacillus_cereus]	CPTF_Ni	0	0	0
UIJ66334.1	16S_rRNA_(cytidine(1402)-2'-O)-methyltransferase_[Bacillus_cereus]	CPTF_U	11438.7	19812.40957	173.2050808
UIJ66334.1	16S_rRNA_(cytidine(1402)-2'-O)-methyltransferase_[Bacillus_cereus]	CPTF_metals_mix	157241.6667	10249.402	6.518248131
UIJ66334.1	16S_rRNA_(cytidine(1402)-2'-O)-methyltransferase_[Bacillus_cereus]	CPTF_zcontrol	0	0	0
UIJ66337.1	DNA_replication_initiation_control_protein_YabA_[Bacillus_cereus]	CPTF_Al	37476.66667	64911.49076	173.2050808
UIJ66337.1	DNA_replication_initiation_control_protein_YabA_[Bacillus_cereus]	CPTF_Cd	71625.33333	124058.7164	173.2050808
UIJ66337.1	DNA_replication_initiation_control_protein_YabA_[Bacillus_cereus]	CPTF_Co	52879	91589.11465	173.2050808
UIJ66337.1	DNA_replication_initiation_control_protein_YabA_[Bacillus_cereus]	CPTF_Cu	81754.66667	72983.20808	89.27099952
UIJ66337.1	DNA_replication_initiation_control_protein_YabA_[Bacillus_cereus]	CPTF_Fe	114469	102787.5188	89.79507015
UIJ66337.1	DNA_replication_initiation_control_protein_YabA_[Bacillus_cereus]	CPTF_Mn	0	0	0
UIJ66337.1	DNA_replication_initiation_control_protein_YabA_[Bacillus_cereus]	CPTF_Ni	47854.33333	82886.1367	173.2050808
UIJ66337.1	DNA_replication_initiation_control_protein_YabA_[Bacillus_cereus]	CPTF_U	0	0	0
UIJ66337.1	DNA_replication_initiation_control_protein_YabA_[Bacillus_cereus]	CPTF_metals_mix	96332.63333	143759.9533	149.2328698
UIJ66337.1	DNA_replication_initiation_control_protein_YabA_[Bacillus_cereus]	CPTF_zcontrol	0	0	0
UIJ66338.1	stage_0_sporulation_family_protein_[Bacillus_cereus]	CPTF_Al	185652.3333	141090.0481	75.99691615
UIJ66338.1	stage_0_sporulation_family_protein_[Bacillus_cereus]	CPTF_Cd	31533.43333	54617.50867	173.2050808
UIJ66338.1	stage_0_sporulation_family_protein_[Bacillus_cereus]	CPTF_Co	301725.2667	109627.5711	36.33357337
UIJ66338.1	stage_0_sporulation_family_protein_[Bacillus_cereus]	CPTF_Cu	166048.6	228008.9174	137.3145678
UIJ66338.1	stage_0_sporulation_family_protein_[Bacillus_cereus]	CPTF_Fe	53071	91921.66841	173.2050808
UIJ66338.1	stage_0_sporulation_family_protein_[Bacillus_cereus]	CPTF_Mn	215209.4	231962.3155	107.784472
UIJ66338.1	stage_0_sporulation_family_protein_[Bacillus_cereus]	CPTF_Ni	218132.7333	197996.9704	90.76903193
UIJ66338.1	stage_0_sporulation_family_protein_[Bacillus_cereus]	CPTF_U	283881.4333	185887.6082	65.48072058
UIJ66338.1	stage_0_sporulation_family_protein_[Bacillus_cereus]	CPTF_metals_mix	282512.3667	61407.35719	21.73616607
UIJ66338.1	stage_0_sporulation_family_protein_[Bacillus_cereus]	CPTF_zcontrol	306654.5333	117269.3936	38.24153268
UIJ66340.1	dTMP_kinase_[Bacillus_cereus]	CPTF_Al	237127.3333	147506.7101	62.20569683
UIJ66340.1	dTMP_kinase_[Bacillus_cereus]	CPTF_Cd	242198.3333	62525.58174	25.81585962
UIJ66340.1	dTMP_kinase_[Bacillus_cereus]	CPTF_Co	243372.0333	83259.65999	34.21085769
UIJ66340.1	dTMP_kinase_[Bacillus_cereus]	CPTF_Cu	290581.3333	115787.9718	39.84700959
UIJ66340.1	dTMP_kinase_[Bacillus_cereus]	CPTF_Fe	165489.2	75210.67814	45.44748427
UIJ66340.1	dTMP_kinase_[Bacillus_cereus]	CPTF_Mn	188501.6667	107985.4185	57.28618764
UIJ66340.1	dTMP_kinase_[Bacillus_cereus]	CPTF_Ni	227680.6667	131621.7267	57.80979501
UIJ66340.1	dTMP_kinase_[Bacillus_cereus]	CPTF_U	105311	93849.33065	89.11636073
UIJ66340.1	dTMP_kinase_[Bacillus_cereus]	CPTF_metals_mix	201921.9	28362.64393	14.04634363
UIJ66340.1	dTMP_kinase_[Bacillus_cereus]	CPTF_zcontrol	150483.3333	21939.79271	14.57954993
UIJ66351.1	DUF3797_domain-containing_protein_[Bacillus_cereus]	CPTF_Al	105066.9667	14971.80688	14.24977551
UIJ66351.1	DUF3797_domain-containing_protein_[Bacillus_cereus]	CPTF_Cd	115967.9	39084.80628	33.70312499
UIJ66351.1	DUF3797_domain-containing_protein_[Bacillus_cereus]	CPTF_Co	120480.3333	3969.288391	3.294552963
UIJ66351.1	DUF3797_domain-containing_protein_[Bacillus_cereus]	CPTF_Cu	66361.26667	16793.23807	25.30578289
UIJ66351.1	DUF3797_domain-containing_protein_[Bacillus_cereus]	CPTF_Fe	65052.6	18488.94949	28.4215381
UIJ66351.1	DUF3797_domain-containing_protein_[Bacillus_cereus]	CPTF_Mn	136199.8	160593.7638	117.9104256
UIJ66351.1	DUF3797_domain-containing_protein_[Bacillus_cereus]	CPTF_Ni	83993.36667	51760.29453	61.62426461
UIJ66351.1	DUF3797_domain-containing_protein_[Bacillus_cereus]	CPTF_U	12056.33333	20882.18189	173.2050808
UIJ66351.1	DUF3797_domain-containing_protein_[Bacillus_cereus]	CPTF_metals_mix	423839.3333	60659.16068	14.31182901
UIJ66351.1	DUF3797_domain-containing_protein_[Bacillus_cereus]	CPTF_zcontrol	53272.06667	15557.63306	29.20411021
UIJ66352.1	serine--tRNA_ligase_[Bacillus_cereus]	CPTF_Al	4219652.367	591502.5182	14.0178021
UIJ66352.1	serine--tRNA_ligase_[Bacillus_cereus]	CPTF_Cd	5311541.567	627100.7794	11.80637996
UIJ66352.1	serine--tRNA_ligase_[Bacillus_cereus]	CPTF_Co	3971320.367	840648.2536	21.16797881
UIJ66352.1	serine--tRNA_ligase_[Bacillus_cereus]	CPTF_Cu	2665341	527344.1977	19.78524315
UIJ66352.1	serine--tRNA_ligase_[Bacillus_cereus]	CPTF_Fe	3489213.133	497171.638	14.24881826
UIJ66352.1	serine--tRNA_ligase_[Bacillus_cereus]	CPTF_Mn	4844812.967	2954219.832	60.97696344
UIJ66352.1	serine--tRNA_ligase_[Bacillus_cereus]	CPTF_Ni	2511244.867	446221.1866	17.76892379
UIJ66352.1	serine--tRNA_ligase_[Bacillus_cereus]	CPTF_U	3495772	934703.9156	26.73812582
UIJ66352.1	serine--tRNA_ligase_[Bacillus_cereus]	CPTF_metals_mix	7320036.967	560392.8825	7.65560181
UIJ66352.1	serine--tRNA_ligase_[Bacillus_cereus]	CPTF_zcontrol	3629440.567	1107262.573	30.50780286
UIJ66353.1	pyridoxal_5'-phosphate_synthase_glutaminase_subunit_PdxT_[Bacillus_cereus]	CPTF_Al	1325418.667	96571.38027	7.286103832
UIJ66353.1	pyridoxal_5'-phosphate_synthase_glutaminase_subunit_PdxT_[Bacillus_cereus]	CPTF_Cd	1294807.267	136987.067	10.57972646
UIJ66353.1	pyridoxal_5'-phosphate_synthase_glutaminase_subunit_PdxT_[Bacillus_cereus]	CPTF_Co	1291589.333	128864.8128	9.977228017
UIJ66353.1	pyridoxal_5'-phosphate_synthase_glutaminase_subunit_PdxT_[Bacillus_cereus]	CPTF_Cu	1149460.667	142763.8859	12.42007578

UIJ66353.1	pyridoxal_5'-phosphate_synthase_glutaminase_subunit_PdxT_[Bacillus_cereus]	CPTF_Fe	1226164.667	27829.09	2.269604626
UIJ66353.1	pyridoxal_5'-phosphate_synthase_glutaminase_subunit_PdxT_[Bacillus_cereus]	CPTF_Mn	1274853	73079.75235	5.732406195
UIJ66353.1	pyridoxal_5'-phosphate_synthase_glutaminase_subunit_PdxT_[Bacillus_cereus]	CPTF_Ni	1036100.333	57798.35718	5.57845175
UIJ66353.1	pyridoxal_5'-phosphate_synthase_glutaminase_subunit_PdxT_[Bacillus_cereus]	CPTF_U	1350298.333	204951.3452	15.17822692
UIJ66353.1	pyridoxal_5'-phosphate_synthase_glutaminase_subunit_PdxT_[Bacillus_cereus]	CPTF_metals_mix	1589409	121413.8943	7.638933359
UIJ66353.1	pyridoxal_5'-phosphate_synthase_glutaminase_subunit_PdxT_[Bacillus_cereus]	CPTF_zcontrol	1309795	100297.0181	7.657459231
UIJ66354.1	pyridoxal_5'-phosphate_synthase_lyase_subunit_PdxS_[Bacillus_cereus]	CPTF_Al	8848102.233	227206.4672	2.567855357
UIJ66354.1	pyridoxal_5'-phosphate_synthase_lyase_subunit_PdxS_[Bacillus_cereus]	CPTF_Cd	9386300.033	289671.834	3.08611309
UIJ66354.1	pyridoxal_5'-phosphate_synthase_lyase_subunit_PdxS_[Bacillus_cereus]	CPTF_Co	9717228	382551.0615	3.936833235
UIJ66354.1	pyridoxal_5'-phosphate_synthase_lyase_subunit_PdxS_[Bacillus_cereus]	CPTF_Cu	8353839.433	798266.7175	9.555686627
UIJ66354.1	pyridoxal_5'-phosphate_synthase_lyase_subunit_PdxS_[Bacillus_cereus]	CPTF_Fe	8888696.167	1113758.998	12.53006039
UIJ66354.1	pyridoxal_5'-phosphate_synthase_lyase_subunit_PdxS_[Bacillus_cereus]	CPTF_Mn	9262090	2065032.876	22.29553887
UIJ66354.1	pyridoxal_5'-phosphate_synthase_lyase_subunit_PdxS_[Bacillus_cereus]	CPTF_Ni	8003792.367	1034503.074	12.92516131
UIJ66354.1	pyridoxal_5'-phosphate_synthase_lyase_subunit_PdxS_[Bacillus_cereus]	CPTF_U	7009757.667	631693.1531	9.011626124
UIJ66354.1	pyridoxal_5'-phosphate_synthase_lyase_subunit_PdxS_[Bacillus_cereus]	CPTF_metals_mix	8589581.36	648634.5163	7.551410123
UIJ66354.1	pyridoxal_5'-phosphate_synthase_lyase_subunit_PdxS_[Bacillus_cereus]	CPTF_zcontrol	9462863	1261796.16	13.33419029
UIJ66355.1	IMP_dehydrogenase_[Bacillus_cereus]	CPTF_Al	16332722.1	728837.3616	4.462436556
UIJ66355.1	IMP_dehydrogenase_[Bacillus_cereus]	CPTF_Cd	17200020.47	863341.8563	5.019423424
UIJ66355.1	IMP_dehydrogenase_[Bacillus_cereus]	CPTF_Co	18556296.83	2822766.687	15.21190738
UIJ66355.1	IMP_dehydrogenase_[Bacillus_cereus]	CPTF_Cu	20280221.8	334123.1039	1.647531803
UIJ66355.1	IMP_dehydrogenase_[Bacillus_cereus]	CPTF_Fe	17750705.53	786367.6897	4.430064417
UIJ66355.1	IMP_dehydrogenase_[Bacillus_cereus]	CPTF_Mn	16452828.2	1986610.874	12.0745859
UIJ66355.1	IMP_dehydrogenase_[Bacillus_cereus]	CPTF_Ni	17098306.4	802778.9991	4.695079035
UIJ66355.1	IMP_dehydrogenase_[Bacillus_cereus]	CPTF_U	13889937.9	1400446.773	10.08245525
UIJ66355.1	IMP_dehydrogenase_[Bacillus_cereus]	CPTF_metals_mix	25520618.93	1306271.289	5.118493764
UIJ66355.1	IMP_dehydrogenase_[Bacillus_cereus]	CPTF_zcontrol	15198650.07	699028.8509	4.599282488
UIJ66357.1	DNA_gyrase_subunit_A_[Bacillus_cereus]	CPTF_Al	2182166.967	164722.7886	7.548587761
UIJ66357.1	DNA_gyrase_subunit_A_[Bacillus_cereus]	CPTF_Cd	2094151.833	142763.904	6.817266147
UIJ66357.1	DNA_gyrase_subunit_A_[Bacillus_cereus]	CPTF_Co	2195111.167	288866.67	13.15954629
UIJ66357.1	DNA_gyrase_subunit_A_[Bacillus_cereus]	CPTF_Cu	2347751.667	412198.2779	17.55714984
UIJ66357.1	DNA_gyrase_subunit_A_[Bacillus_cereus]	CPTF_Fe	2327594.667	21162.95745	0.909220053
UIJ66357.1	DNA_gyrase_subunit_A_[Bacillus_cereus]	CPTF_Mn	2164834.867	181817.734	8.398688361
UIJ66357.1	DNA_gyrase_subunit_A_[Bacillus_cereus]	CPTF_Ni	2205093.333	81140.07642	3.679666307
UIJ66357.1	DNA_gyrase_subunit_A_[Bacillus_cereus]	CPTF_U	2125611.633	356614.4107	16.77702573
UIJ66357.1	DNA_gyrase_subunit_A_[Bacillus_cereus]	CPTF_metals_mix	2210630.333	93182.08353	4.215181622
UIJ66357.1	DNA_gyrase_subunit_A_[Bacillus_cereus]	CPTF_zcontrol	2015719.133	126395.6665	6.27049991
UIJ66358.1	DNA_topoisomerase_(ATP-hydrolyzing)_subunit_B_[Bacillus_cereus]	CPTF_Al	2912257.3	272240.7087	9.348099451
UIJ66358.1	DNA_topoisomerase_(ATP-hydrolyzing)_subunit_B_[Bacillus_cereus]	CPTF_Cd	3266374.567	748534.7055	22.91637687
UIJ66358.1	DNA_topoisomerase_(ATP-hydrolyzing)_subunit_B_[Bacillus_cereus]	CPTF_Co	2245246.833	701948.5206	31.26375729
UIJ66358.1	DNA_topoisomerase_(ATP-hydrolyzing)_subunit_B_[Bacillus_cereus]	CPTF_Cu	2820066.5	546528.5109	19.37998664
UIJ66358.1	DNA_topoisomerase_(ATP-hydrolyzing)_subunit_B_[Bacillus_cereus]	CPTF_Fe	2777623.333	135833.0704	4.890262434
UIJ66358.1	DNA_topoisomerase_(ATP-hydrolyzing)_subunit_B_[Bacillus_cereus]	CPTF_Mn	3006065.767	760770.7034	25.30785294
UIJ66358.1	DNA_topoisomerase_(ATP-hydrolyzing)_subunit_B_[Bacillus_cereus]	CPTF_Ni	1880596.8	578650.1637	30.76949635
UIJ66358.1	DNA_topoisomerase_(ATP-hydrolyzing)_subunit_B_[Bacillus_cereus]	CPTF_U	2533572.733	284877.2037	11.24409021
UIJ66358.1	DNA_topoisomerase_(ATP-hydrolyzing)_subunit_B_[Bacillus_cereus]	CPTF_metals_mix	4813635.667	290741.2944	6.039952222
UIJ66358.1	DNA_topoisomerase_(ATP-hydrolyzing)_subunit_B_[Bacillus_cereus]	CPTF_zcontrol	2639145.4	474339.7967	17.97323469
UIJ66361.1	DNA_polymerase_III_subunit_beta_[Bacillus_cereus]	CPTF_Al	621525	25806.62882	4.152146546
UIJ66361.1	DNA_polymerase_III_subunit_beta_[Bacillus_cereus]	CPTF_Cd	578768	26741.31199	4.620385369
UIJ66361.1	DNA_polymerase_III_subunit_beta_[Bacillus_cereus]	CPTF_Co	605212	74327.3773	12.28121341
UIJ66361.1	DNA_polymerase_III_subunit_beta_[Bacillus_cereus]	CPTF_Cu	556536.6667	36037.69563	6.475349746
UIJ66361.1	DNA_polymerase_III_subunit_beta_[Bacillus_cereus]	CPTF_Fe	736198	195685.7709	26.58058985
UIJ66361.1	DNA_polymerase_III_subunit_beta_[Bacillus_cereus]	CPTF_Mn	684817.3333	62052.61126	9.061191684
UIJ66361.1	DNA_polymerase_III_subunit_beta_[Bacillus_cereus]	CPTF_Ni	603527	61247.63617	10.14828436
UIJ66361.1	DNA_polymerase_III_subunit_beta_[Bacillus_cereus]	CPTF_U	675167.3333	124141.6608	18.38679905
UIJ66361.1	DNA_polymerase_III_subunit_beta_[Bacillus_cereus]	CPTF_metals_mix	597009.8	58397.80073	9.781715598
UIJ66361.1	DNA_polymerase_III_subunit_beta_[Bacillus_cereus]	CPTF_zcontrol	656550.3333	60757.31203	9.254021961
UIJ66362.1	chromosomal_replication_initiator_protein_DnaA_[Bacillus_cereus]	CPTF_Al	883243.4333	723916.1554	81.96111379
UIJ66362.1	chromosomal_replication_initiator_protein_DnaA_[Bacillus_cereus]	CPTF_Cd	1072238.2	520417.006	48.53557783
UIJ66362.1	chromosomal_replication_initiator_protein_DnaA_[Bacillus_cereus]	CPTF_Co	645777.3333	238821.8176	36.98206879
UIJ66362.1	chromosomal_replication_initiator_protein_DnaA_[Bacillus_cereus]	CPTF_Cu	638724.6667	194881.4795	30.51103075
UIJ66362.1	chromosomal_replication_initiator_protein_DnaA_[Bacillus_cereus]	CPTF_Fe	738364.9667	255915.1257	34.65970587

UIJ66362.1	chromosomal_replication_initiator_protein_DnaA_[Bacillus_cereus]	CPTF_Mn	879694.3667	291197.581	33.10213093
UIJ66362.1	chromosomal_replication_initiator_protein_DnaA_[Bacillus_cereus]	CPTF_Ni	682326.6667	205185.3521	30.07142504
UIJ66362.1	chromosomal_replication_initiator_protein_DnaA_[Bacillus_cereus]	CPTF_U	789810	266898.8683	33.79279425
UIJ66362.1	chromosomal_replication_initiator_protein_DnaA_[Bacillus_cereus]	CPTF_metals_mix	686887.1667	390503.7249	56.85121864
UIJ66362.1	chromosomal_replication_initiator_protein_DnaA_[Bacillus_cereus]	CPTF_zcontrol	477019.3333	413890.7345	86.76602929
UIJ66363.1	50S_ribosomal_protein_L34_[Bacillus_cereus]	CPTF_Al	196258	177052.4384	90.21412549
UIJ66363.1	50S_ribosomal_protein_L34_[Bacillus_cereus]	CPTF_Cd	260417.3333	226502.3241	86.97666979
UIJ66363.1	50S_ribosomal_protein_L34_[Bacillus_cereus]	CPTF_Co	51132	88563.22189	173.2050808
UIJ66363.1	50S_ribosomal_protein_L34_[Bacillus_cereus]	CPTF_Cu	87847.66667	152156.622	173.2050808
UIJ66363.1	50S_ribosomal_protein_L34_[Bacillus_cereus]	CPTF_Fe	110487	191369.0976	173.2050808
UIJ66363.1	50S_ribosomal_protein_L34_[Bacillus_cereus]	CPTF_Mn	155583.3333	269478.2381	173.2050808
UIJ66363.1	50S_ribosomal_protein_L34_[Bacillus_cereus]	CPTF_Ni	20594.7	35671.06677	173.2050808
UIJ66363.1	50S_ribosomal_protein_L34_[Bacillus_cereus]	CPTF_U	0	0	0
UIJ66363.1	50S_ribosomal_protein_L34_[Bacillus_cereus]	CPTF_metals_mix	225532	199628.1419	88.51433139
UIJ66363.1	50S_ribosomal_protein_L34_[Bacillus_cereus]	CPTF_zcontrol	0	0	0
UIJ66366.1	protein_jag_[Bacillus_cereus]	CPTF_Al	0	0	0
UIJ66366.1	protein_jag_[Bacillus_cereus]	CPTF_Cd	0	0	0
UIJ66366.1	protein_jag_[Bacillus_cereus]	CPTF_Co	20352.6	35251.73727	173.2050808
UIJ66366.1	protein_jag_[Bacillus_cereus]	CPTF_Cu	0	0	0
UIJ66366.1	protein_jag_[Bacillus_cereus]	CPTF_Fe	0	0	0
UIJ66366.1	protein_jag_[Bacillus_cereus]	CPTF_Mn	0	0	0
UIJ66366.1	protein_jag_[Bacillus_cereus]	CPTF_Ni	12332.53333	21360.57432	173.2050808
UIJ66366.1	protein_jag_[Bacillus_cereus]	CPTF_U	0	0	0
UIJ66366.1	protein_jag_[Bacillus_cereus]	CPTF_metals_mix	0	0	0
UIJ66366.1	protein_jag_[Bacillus_cereus]	CPTF_zcontrol	0	0	0
UIJ66367.1	tRNA_uridine-5-carboxymethylaminomethyl(34)_synthesis_GTPase_MnmE_[Bacillus_cereus]	CPTF_Al	0	0	0
UIJ66367.1	tRNA_uridine-5-carboxymethylaminomethyl(34)_synthesis_GTPase_MnmE_[Bacillus_cereus]	CPTF_Cd	0	0	0
UIJ66367.1	tRNA_uridine-5-carboxymethylaminomethyl(34)_synthesis_GTPase_MnmE_[Bacillus_cereus]	CPTF_Co	0	0	0
UIJ66367.1	tRNA_uridine-5-carboxymethylaminomethyl(34)_synthesis_GTPase_MnmE_[Bacillus_cereus]	CPTF_Cu	0	0	0
UIJ66367.1	tRNA_uridine-5-carboxymethylaminomethyl(34)_synthesis_GTPase_MnmE_[Bacillus_cereus]	CPTF_Fe	51425.66667	89071.86748	173.2050808
UIJ66367.1	tRNA_uridine-5-carboxymethylaminomethyl(34)_synthesis_GTPase_MnmE_[Bacillus_cereus]	CPTF_Mn	0	0	0
UIJ66367.1	tRNA_uridine-5-carboxymethylaminomethyl(34)_synthesis_GTPase_MnmE_[Bacillus_cereus]	CPTF_Ni	0	0	0
UIJ66367.1	tRNA_uridine-5-carboxymethylaminomethyl(34)_synthesis_GTPase_MnmE_[Bacillus_cereus]	CPTF_U	127456.3333	114171.6435	89.57706576
UIJ66367.1	tRNA_uridine-5-carboxymethylaminomethyl(34)_synthesis_GTPase_MnmE_[Bacillus_cereus]	CPTF_metals_mix	142058.5667	63072.19526	44.39872705
UIJ66367.1	tRNA_uridine-5-carboxymethylaminomethyl(34)_synthesis_GTPase_MnmE_[Bacillus_cereus]	CPTF_zcontrol	0	0	0
UIJ66368.1	tRNA_uridine-5-carboxymethylaminomethyl(34)_synthesis_enzyme_MnmG_[Bacillus_cereus]	CPTF_Al	649189.2	321404.0746	49.50853689
UIJ66368.1	tRNA_uridine-5-carboxymethylaminomethyl(34)_synthesis_enzyme_MnmG_[Bacillus_cereus]	CPTF_Cd	706336.7667	253498.3876	35.88916782
UIJ66368.1	tRNA_uridine-5-carboxymethylaminomethyl(34)_synthesis_enzyme_MnmG_[Bacillus_cereus]	CPTF_Co	826643.2	146811.8541	17.76000263
UIJ66368.1	tRNA_uridine-5-carboxymethylaminomethyl(34)_synthesis_enzyme_MnmG_[Bacillus_cereus]	CPTF_Cu	713402.3	201936.1319	28.30606684
UIJ66368.1	tRNA_uridine-5-carboxymethylaminomethyl(34)_synthesis_enzyme_MnmG_[Bacillus_cereus]	CPTF_Fe	609357.3667	211430.6387	34.69731397
UIJ66368.1	tRNA_uridine-5-carboxymethylaminomethyl(34)_synthesis_enzyme_MnmG_[Bacillus_cereus]	CPTF_Mn	467012.5667	259212.476	55.50438992
UIJ66368.1	tRNA_uridine-5-carboxymethylaminomethyl(34)_synthesis_enzyme_MnmG_[Bacillus_cereus]	CPTF_Ni	558241.7	302745.9799	54.23206111
UIJ66368.1	tRNA_uridine-5-carboxymethylaminomethyl(34)_synthesis_enzyme_MnmG_[Bacillus_cereus]	CPTF_U	427140.5333	339778.4724	79.54723232
UIJ66368.1	tRNA_uridine-5-carboxymethylaminomethyl(34)_synthesis_enzyme_MnmG_[Bacillus_cereus]	CPTF_metals_mix	1070454.5	73480.49936	6.864420614
UIJ66368.1	tRNA_uridine-5-carboxymethylaminomethyl(34)_synthesis_enzyme_MnmG_[Bacillus_cereus]	CPTF_zcontrol	582786.0667	234273.6329	40.19890768
UIJ66370.1	nucleoid_occlusion_protein_[Bacillus_cereus]	CPTF_Al	155264.9333	134857.9125	86.85664536
UIJ66370.1	nucleoid_occlusion_protein_[Bacillus_cereus]	CPTF_Cd	185537.2333	133531.8832	71.97039689
UIJ66370.1	nucleoid_occlusion_protein_[Bacillus_cereus]	CPTF_Co	45870.36667	33117.74708	72.19856627
UIJ66370.1	nucleoid_occlusion_protein_[Bacillus_cereus]	CPTF_Cu	97081.56667	104247.0768	107.3809173
UIJ66370.1	nucleoid_occlusion_protein_[Bacillus_cereus]	CPTF_Fe	109986.3	130897.3369	119.0124014
UIJ66370.1	nucleoid_occlusion_protein_[Bacillus_cereus]	CPTF_Mn	103109.4667	90006.45968	87.2921397
UIJ66370.1	nucleoid_occlusion_protein_[Bacillus_cereus]	CPTF_Ni	17190.86667	29775.45449	173.2050808
UIJ66370.1	nucleoid_occlusion_protein_[Bacillus_cereus]	CPTF_U	0	0	0
UIJ66370.1	nucleoid_occlusion_protein_[Bacillus_cereus]	CPTF_metals_mix	152077.6667	12805.24227	8.420199068
UIJ66370.1	nucleoid_occlusion_protein_[Bacillus_cereus]	CPTF_zcontrol	72487.03333	102627.1987	141.5800785
UIJ66371.1	sporulation_initiation_inhibitor_protein_Soj_[Bacillus_cereus]	CPTF_Al	292180	41596.43865	14.23657973
UIJ66371.1	sporulation_initiation_inhibitor_protein_Soj_[Bacillus_cereus]	CPTF_Cd	371946.5	132010.4025	35.49177167
UIJ66371.1	sporulation_initiation_inhibitor_protein_Soj_[Bacillus_cereus]	CPTF_Co	252740	8574.107825	3.392461749
UIJ66371.1	sporulation_initiation_inhibitor_protein_Soj_[Bacillus_cereus]	CPTF_Cu	263185.6667	51010.96451	19.38212105
UIJ66371.1	sporulation_initiation_inhibitor_protein_Soj_[Bacillus_cereus]	CPTF_Fe	378436.6	39792.73169	10.51503256
UIJ66371.1	sporulation_initiation_inhibitor_protein_Soj_[Bacillus_cereus]	CPTF_Mn	247984.3333	76338.25738	30.78350005

UIJ66371.1	sporulation_initiation_inhibitor_protein_Soj_[Bacillus_cereus]	CPTF_Ni	309928.3333	65026.06598	20.98100076
UIJ66371.1	sporulation_initiation_inhibitor_protein_Soj_[Bacillus_cereus]	CPTF_U	233742.6667	42946.18125	18.37327428
UIJ66371.1	sporulation_initiation_inhibitor_protein_Soj_[Bacillus_cereus]	CPTF_metals_mix	208911.2333	11002.74853	5.266709861
UIJ66371.1	sporulation_initiation_inhibitor_protein_Soj_[Bacillus_cereus]	CPTF_zcontrol	188893.6667	117245.2932	62.0694676
UIJ66372.1	stage_0_sporulation_protein_Spo0J_[Bacillus_cereus]	CPTF_Al	746391.3	211599.5244	28.34967723
UIJ66372.1	stage_0_sporulation_protein_Spo0J_[Bacillus_cereus]	CPTF_Cd	675882.9	449404.062	66.49140879
UIJ66372.1	stage_0_sporulation_protein_Spo0J_[Bacillus_cereus]	CPTF_Co	821314.5333	156358.9606	19.03764687
UIJ66372.1	stage_0_sporulation_protein_Spo0J_[Bacillus_cereus]	CPTF_Cu	798633.1333	127308.4517	15.94079263
UIJ66372.1	stage_0_sporulation_protein_Spo0J_[Bacillus_cereus]	CPTF_Fe	604285.2	136904.72	22.6556467
UIJ66372.1	stage_0_sporulation_protein_Spo0J_[Bacillus_cereus]	CPTF_Mn	539479.3333	349189.4944	64.72713092
UIJ66372.1	stage_0_sporulation_protein_Spo0J_[Bacillus_cereus]	CPTF_Ni	689757.3667	198309.2957	28.75058757
UIJ66372.1	stage_0_sporulation_protein_Spo0J_[Bacillus_cereus]	CPTF_U	430956.0667	403129.8886	93.54315202
UIJ66372.1	stage_0_sporulation_protein_Spo0J_[Bacillus_cereus]	CPTF_metals_mix	746558.8667	57840.88172	7.747665228
UIJ66372.1	stage_0_sporulation_protein_Spo0J_[Bacillus_cereus]	CPTF_zcontrol	562268.9333	390133.6958	69.38560406
UIJ66375.1	DUF951_domain-containing_protein_[Bacillus_cereus]	CPTF_Al	116759.5667	36132.23613	30.94584638
UIJ66375.1	DUF951_domain-containing_protein_[Bacillus_cereus]	CPTF_Cd	81377.4	92988.92953	114.2687399
UIJ66375.1	DUF951_domain-containing_protein_[Bacillus_cereus]	CPTF_Co	14108.83333	24437.21617	173.2050808
UIJ66375.1	DUF951_domain-containing_protein_[Bacillus_cereus]	CPTF_Cu	93362.63333	33278.80476	35.64467236
UIJ66375.1	DUF951_domain-containing_protein_[Bacillus_cereus]	CPTF_Fe	145356.5	77953.52426	53.62919736
UIJ66375.1	DUF951_domain-containing_protein_[Bacillus_cereus]	CPTF_Mn	0	0	0
UIJ66375.1	DUF951_domain-containing_protein_[Bacillus_cereus]	CPTF_Ni	79040.43333	34316.47817	43.41635885
UIJ66375.1	DUF951_domain-containing_protein_[Bacillus_cereus]	CPTF_U	71487.93333	64133.90592	89.71291088
UIJ66375.1	DUF951_domain-containing_protein_[Bacillus_cereus]	CPTF_metals_mix	223899	38366.7333	17.13573232
UIJ66375.1	DUF951_domain-containing_protein_[Bacillus_cereus]	CPTF_zcontrol	0	0	0
UIJ66376.1	redox-regulated_ATPase_YchF_[Bacillus_cereus]	CPTF_Al	1533698.867	219836.4263	14.3337412
UIJ66376.1	redox-regulated_ATPase_YchF_[Bacillus_cereus]	CPTF_Cd	1891035.767	347273.7336	18.36420758
UIJ66376.1	redox-regulated_ATPase_YchF_[Bacillus_cereus]	CPTF_Co	1776743.7	100217.2211	5.640499589
UIJ66376.1	redox-regulated_ATPase_YchF_[Bacillus_cereus]	CPTF_Cu	1784936.033	176557.2784	9.891518524
UIJ66376.1	redox-regulated_ATPase_YchF_[Bacillus_cereus]	CPTF_Fe	1625293.233	77228.82133	4.751685403
UIJ66376.1	redox-regulated_ATPase_YchF_[Bacillus_cereus]	CPTF_Mn	1488935.567	247127.7509	16.59761217
UIJ66376.1	redox-regulated_ATPase_YchF_[Bacillus_cereus]	CPTF_Ni	1629454.767	150248.5184	9.220784855
UIJ66376.1	redox-regulated_ATPase_YchF_[Bacillus_cereus]	CPTF_U	1358933.62	460176.1285	33.86303214
UIJ66376.1	redox-regulated_ATPase_YchF_[Bacillus_cereus]	CPTF_metals_mix	2591358.683	241170.6698	9.306726675
UIJ66376.1	redox-regulated_ATPase_YchF_[Bacillus_cereus]	CPTF_zcontrol	1448809.667	299018.458	20.6389055
UIJ66377.1	30S_ribosomal_protein_S6_[Bacillus_cereus]	CPTF_Al	6688097.333	964434.3328	14.42015995
UIJ66377.1	30S_ribosomal_protein_S6_[Bacillus_cereus]	CPTF_Cd	7060744.667	710838.983	10.06747895
UIJ66377.1	30S_ribosomal_protein_S6_[Bacillus_cereus]	CPTF_Co	7343430.6	357031.4446	4.861916236
UIJ66377.1	30S_ribosomal_protein_S6_[Bacillus_cereus]	CPTF_Cu	6838044.667	296160.0682	4.331063669
UIJ66377.1	30S_ribosomal_protein_S6_[Bacillus_cereus]	CPTF_Fe	6354383.367	360216.025	5.668780182
UIJ66377.1	30S_ribosomal_protein_S6_[Bacillus_cereus]	CPTF_Mn	6790459.333	968041.1252	14.25590049
UIJ66377.1	30S_ribosomal_protein_S6_[Bacillus_cereus]	CPTF_Ni	5656958.667	568466.858	10.04898376
UIJ66377.1	30S_ribosomal_protein_S6_[Bacillus_cereus]	CPTF_U	5534801	915494.8132	16.54069971
UIJ66377.1	30S_ribosomal_protein_S6_[Bacillus_cereus]	CPTF_metals_mix	8844911.233	1695261.613	19.16651923
UIJ66377.1	30S_ribosomal_protein_S6_[Bacillus_cereus]	CPTF_zcontrol	6004304.433	243519.1529	4.055742936
UIJ66378.1	single-stranded_DNA-binding_protein_[Bacillus_cereus]	CPTF_Al	122914.6	58558.26017	47.64141946
UIJ66378.1	single-stranded_DNA-binding_protein_[Bacillus_cereus]	CPTF_Cd	364644.5667	73756.2534	20.22688945
UIJ66378.1	single-stranded_DNA-binding_protein_[Bacillus_cereus]	CPTF_Co	84726.93333	73621.04	86.89213348
UIJ66378.1	single-stranded_DNA-binding_protein_[Bacillus_cereus]	CPTF_Cu	189019.0333	164149.4996	86.84284153
UIJ66378.1	single-stranded_DNA-binding_protein_[Bacillus_cereus]	CPTF_Fe	216542.7667	124659.1833	57.56792768
UIJ66378.1	single-stranded_DNA-binding_protein_[Bacillus_cereus]	CPTF_Mn	133611.9667	152959.0332	114.4800402
UIJ66378.1	single-stranded_DNA-binding_protein_[Bacillus_cereus]	CPTF_Ni	84385.4	74567.76367	88.3657169
UIJ66378.1	single-stranded_DNA-binding_protein_[Bacillus_cereus]	CPTF_U	58089.13333	2795.286909	4.812065095
UIJ66378.1	single-stranded_DNA-binding_protein_[Bacillus_cereus]	CPTF_metals_mix	345457.4333	106074.5979	30.70554797
UIJ66378.1	single-stranded_DNA-binding_protein_[Bacillus_cereus]	CPTF_zcontrol	126457.7333	138242.0193	109.3187547
UIJ66379.1	30S_ribosomal_protein_S18_[Bacillus_cereus]	CPTF_Al	2499308.667	551414.8614	22.06269553
UIJ66379.1	30S_ribosomal_protein_S18_[Bacillus_cereus]	CPTF_Cd	2521476.333	371737.3958	14.74284692
UIJ66379.1	30S_ribosomal_protein_S18_[Bacillus_cereus]	CPTF_Co	2408741.333	438871.6724	18.21995854
UIJ66379.1	30S_ribosomal_protein_S18_[Bacillus_cereus]	CPTF_Cu	2397748	455148.646	18.98233868
UIJ66379.1	30S_ribosomal_protein_S18_[Bacillus_cereus]	CPTF_Fe	2901388	311024.659	10.71985749
UIJ66379.1	30S_ribosomal_protein_S18_[Bacillus_cereus]	CPTF_Mn	2337037.333	300293.9852	12.8493448
UIJ66379.1	30S_ribosomal_protein_S18_[Bacillus_cereus]	CPTF_Ni	1956433.333	209995.9733	10.73361252

UIJ66379.1	30S_ribosomal_protein_S18_[Bacillus_cereus]	CPTF_U	1718633.833	813221.015	47.31787535
UIJ66379.1	30S_ribosomal_protein_S18_[Bacillus_cereus]	CPTF_metals_mix	2308485.333	472873.0813	20.4841276
UIJ66379.1	30S_ribosomal_protein_S18_[Bacillus_cereus]	CPTF_zcontrol	1961390.6	307896.6541	15.69787548
UIJ66382.1	50S_ribosomal_protein_L9_[Bacillus_cereus]	CPTF_Al	3687168	1139034.82	30.89186119
UIJ66382.1	50S_ribosomal_protein_L9_[Bacillus_cereus]	CPTF_Cd	4760512	152282.1732	3.19886124
UIJ66382.1	50S_ribosomal_protein_L9_[Bacillus_cereus]	CPTF_Co	4149316	146752.5611	3.536789224
UIJ66382.1	50S_ribosomal_protein_L9_[Bacillus_cereus]	CPTF_Cu	4228394.333	252403.2814	5.969246514
UIJ66382.1	50S_ribosomal_protein_L9_[Bacillus_cereus]	CPTF_Fe	4601995	325381.8489	7.07045203
UIJ66382.1	50S_ribosomal_protein_L9_[Bacillus_cereus]	CPTF_Mn	3949034.333	495667.6821	12.55161744
UIJ66382.1	50S_ribosomal_protein_L9_[Bacillus_cereus]	CPTF_Ni	2776134	166862.6344	6.010611679
UIJ66382.1	50S_ribosomal_protein_L9_[Bacillus_cereus]	CPTF_U	3885253.667	160584.8053	4.133187149
UIJ66382.1	50S_ribosomal_protein_L9_[Bacillus_cereus]	CPTF_metals_mix	4538687.667	487964.7946	10.75123098
UIJ66382.1	50S_ribosomal_protein_L9_[Bacillus_cereus]	CPTF_zcontrol	4078325.333	478821.7343	11.74064586
UIJ66384.1	adenylosuccinate_synthase_[Bacillus_cereus]	CPTF_Al	14836225.87	1775014.614	11.96405764
UIJ66384.1	adenylosuccinate_synthase_[Bacillus_cereus]	CPTF_Cd	14966457.77	1135056.751	7.584003968
UIJ66384.1	adenylosuccinate_synthase_[Bacillus_cereus]	CPTF_Co	13466306.6	1692784.998	12.57052174
UIJ66384.1	adenylosuccinate_synthase_[Bacillus_cereus]	CPTF_Cu	12465426.4	1247584.599	10.00835879
UIJ66384.1	adenylosuccinate_synthase_[Bacillus_cereus]	CPTF_Fe	14650506.27	444109.4297	3.031358928
UIJ66384.1	adenylosuccinate_synthase_[Bacillus_cereus]	CPTF_Mn	14599177.13	682885.5771	4.677562104
UIJ66384.1	adenylosuccinate_synthase_[Bacillus_cereus]	CPTF_Ni	10741233.23	2009669.999	18.70986278
UIJ66384.1	adenylosuccinate_synthase_[Bacillus_cereus]	CPTF_U	12935601.23	2209846.496	17.08344635
UIJ66384.1	adenylosuccinate_synthase_[Bacillus_cereus]	CPTF_metals_mix	10178007.9	1111149.877	10.91716462
UIJ66384.1	adenylosuccinate_synthase_[Bacillus_cereus]	CPTF_zcontrol	14478687.73	624005.7355	4.309822458
UIJ66385.1	cell_wall_metabolism_DNA-binding_response_regulator_WalR_[Bacillus_cereus]	CPTF_Al	380684.1333	305055.4152	80.13347248
UIJ66385.1	cell_wall_metabolism_DNA-binding_response_regulator_WalR_[Bacillus_cereus]	CPTF_Cd	278735.1	149751.8799	53.72551928
UIJ66385.1	cell_wall_metabolism_DNA-binding_response_regulator_WalR_[Bacillus_cereus]	CPTF_Co	562222	423533.4117	75.33205952
UIJ66385.1	cell_wall_metabolism_DNA-binding_response_regulator_WalR_[Bacillus_cereus]	CPTF_Cu	1176850.533	77678.61065	6.600550236
UIJ66385.1	cell_wall_metabolism_DNA-binding_response_regulator_WalR_[Bacillus_cereus]	CPTF_Fe	501243.4667	509135.6943	101.5745298
UIJ66385.1	cell_wall_metabolism_DNA-binding_response_regulator_WalR_[Bacillus_cereus]	CPTF_Mn	587764.8	409100.2324	69.60271053
UIJ66385.1	cell_wall_metabolism_DNA-binding_response_regulator_WalR_[Bacillus_cereus]	CPTF_Ni	545126.8667	455016.5308	83.46984136
UIJ66385.1	cell_wall_metabolism_DNA-binding_response_regulator_WalR_[Bacillus_cereus]	CPTF_U	853319.3333	350471.418	41.07154312
UIJ66385.1	cell_wall_metabolism_DNA-binding_response_regulator_WalR_[Bacillus_cereus]	CPTF_metals_mix	2568525.9	492097.8955	19.15876712
UIJ66385.1	cell_wall_metabolism_DNA-binding_response_regulator_WalR_[Bacillus_cereus]	CPTF_zcontrol	175282.8333	94204.32388	53.74418139
UIJ66398.1	UDP-glucose_4-epimerase_GalE_[Bacillus_cereus]	CPTF_Al	390119.3333	34128.54202	8.748231401
UIJ66398.1	UDP-glucose_4-epimerase_GalE_[Bacillus_cereus]	CPTF_Cd	437573	25707.24093	5.874960504
UIJ66398.1	UDP-glucose_4-epimerase_GalE_[Bacillus_cereus]	CPTF_Co	370276	15133.13606	4.086988101
UIJ66398.1	UDP-glucose_4-epimerase_GalE_[Bacillus_cereus]	CPTF_Cu	721086.3333	251152.932	34.82980059
UIJ66398.1	UDP-glucose_4-epimerase_GalE_[Bacillus_cereus]	CPTF_Fe	501359.6667	206873.8715	41.26256763
UIJ66398.1	UDP-glucose_4-epimerase_GalE_[Bacillus_cereus]	CPTF_Mn	379413	86004.45726	22.66776765
UIJ66398.1	UDP-glucose_4-epimerase_GalE_[Bacillus_cereus]	CPTF_Ni	424914	27601.12058	6.495695735
UIJ66398.1	UDP-glucose_4-epimerase_GalE_[Bacillus_cereus]	CPTF_U	440879.6667	156263.8865	35.44365919
UIJ66398.1	UDP-glucose_4-epimerase_GalE_[Bacillus_cereus]	CPTF_metals_mix	1191077	68147.29325	5.721485114
UIJ66398.1	UDP-glucose_4-epimerase_GalE_[Bacillus_cereus]	CPTF_zcontrol	423834.6667	20357.93875	4.803273624
UIJ66401.1	superoxide_dismutase_[Mn]_[Bacillus_cereus]	CPTF_Al	20539.3	35575.11115	173.2050808
UIJ66401.1	superoxide_dismutase_[Mn]_[Bacillus_cereus]	CPTF_Cd	60643.8	53435.93836	88.11442944
UIJ66401.1	superoxide_dismutase_[Mn]_[Bacillus_cereus]	CPTF_Co	18960.06667	17482.06786	92.20467505
UIJ66401.1	superoxide_dismutase_[Mn]_[Bacillus_cereus]	CPTF_Cu	53526.2	19861.29596	37.10574626
UIJ66401.1	superoxide_dismutase_[Mn]_[Bacillus_cereus]	CPTF_Fe	41176.33333	71319.5014	173.2050808
UIJ66401.1	superoxide_dismutase_[Mn]_[Bacillus_cereus]	CPTF_Mn	0	0	0
UIJ66401.1	superoxide_dismutase_[Mn]_[Bacillus_cereus]	CPTF_Ni	28924.16667	50098.12623	173.2050808
UIJ66401.1	superoxide_dismutase_[Mn]_[Bacillus_cereus]	CPTF_U	0	0	0
UIJ66401.1	superoxide_dismutase_[Mn]_[Bacillus_cereus]	CPTF_metals_mix	38403.26667	33268.26745	86.6287437
UIJ66401.1	superoxide_dismutase_[Mn]_[Bacillus_cereus]	CPTF_zcontrol	24815.06667	42980.95626	173.2050808
UIJ66409.1	peptide-methionine_(R)-S-oxide_reductase_MsrB_[Bacillus_cereus]	CPTF_Al	0	0	0
UIJ66409.1	peptide-methionine_(R)-S-oxide_reductase_MsrB_[Bacillus_cereus]	CPTF_Cd	0	0	0
UIJ66409.1	peptide-methionine_(R)-S-oxide_reductase_MsrB_[Bacillus_cereus]	CPTF_Co	0	0	0
UIJ66409.1	peptide-methionine_(R)-S-oxide_reductase_MsrB_[Bacillus_cereus]	CPTF_Cu	0	0	0
UIJ66409.1	peptide-methionine_(R)-S-oxide_reductase_MsrB_[Bacillus_cereus]	CPTF_Fe	0	0	0
UIJ66409.1	peptide-methionine_(R)-S-oxide_reductase_MsrB_[Bacillus_cereus]	CPTF_Mn	31298.86667	54211.22729	173.2050808
UIJ66409.1	peptide-methionine_(R)-S-oxide_reductase_MsrB_[Bacillus_cereus]	CPTF_Ni	0	0	0
UIJ66409.1	peptide-methionine_(R)-S-oxide_reductase_MsrB_[Bacillus_cereus]	CPTF_U	0	0	0

UIJ66409.1	peptide-methionine_(R)-S-oxide_reductase_MsrB_[Bacillus_cereus]	CPTF_metals_mix	69226.96667	66075.16572	95.44714857
UIJ66409.1	peptide-methionine_(R)-S-oxide_reductase_MsrB_[Bacillus_cereus]	CPTF_zcontrol	0	0	0
UIJ66412.1	glycosyltransferase_family_2_protein_[Bacillus_cereus]	CPTF_Al	0	0	0
UIJ66412.1	glycosyltransferase_family_2_protein_[Bacillus_cereus]	CPTF_Cd	9559.433333	16557.42422	173.2050808
UIJ66412.1	glycosyltransferase_family_2_protein_[Bacillus_cereus]	CPTF_Co	9961.4	17253.65091	173.2050808
UIJ66412.1	glycosyltransferase_family_2_protein_[Bacillus_cereus]	CPTF_Cu	9511.333333	16474.11258	173.2050808
UIJ66412.1	glycosyltransferase_family_2_protein_[Bacillus_cereus]	CPTF_Fe	7535.533333	13051.9266	173.2050808
UIJ66412.1	glycosyltransferase_family_2_protein_[Bacillus_cereus]	CPTF_Mn	0	0	0
UIJ66412.1	glycosyltransferase_family_2_protein_[Bacillus_cereus]	CPTF_Ni	0	0	0
UIJ66412.1	glycosyltransferase_family_2_protein_[Bacillus_cereus]	CPTF_U	0	0	0
UIJ66412.1	glycosyltransferase_family_2_protein_[Bacillus_cereus]	CPTF_metals_mix	11644.06667	20168.11507	173.2050808
UIJ66412.1	glycosyltransferase_family_2_protein_[Bacillus_cereus]	CPTF_zcontrol	0	0	0
UIJ66413.1	YfhO_family_protein_[Bacillus_cereus]	CPTF_Al	22288.6	38604.98763	173.2050808
UIJ66413.1	YfhO_family_protein_[Bacillus_cereus]	CPTF_Cd	40060.53333	4360.207432	10.88404739
UIJ66413.1	YfhO_family_protein_[Bacillus_cereus]	CPTF_Co	39468.56667	35811.8355	90.73507989
UIJ66413.1	YfhO_family_protein_[Bacillus_cereus]	CPTF_Cu	55759.4	12346.0291	22.14161038
UIJ66413.1	YfhO_family_protein_[Bacillus_cereus]	CPTF_Fe	23698.23333	41046.54418	173.2050808
UIJ66413.1	YfhO_family_protein_[Bacillus_cereus]	CPTF_Mn	28287.83333	24515.98104	86.66616759
UIJ66413.1	YfhO_family_protein_[Bacillus_cereus]	CPTF_Ni	23631.46667	40930.90092	173.2050808
UIJ66413.1	YfhO_family_protein_[Bacillus_cereus]	CPTF_U	42748.3	7754.107617	18.13898475
UIJ66413.1	YfhO_family_protein_[Bacillus_cereus]	CPTF_metals_mix	42711.93333	28334.2099	66.33792406
UIJ66413.1	YfhO_family_protein_[Bacillus_cereus]	CPTF_zcontrol	0	0	0
UIJ66423.1	glycosyltransferase_family_4_protein_[Bacillus_cereus]	CPTF_Al	0	0	0
UIJ66423.1	glycosyltransferase_family_4_protein_[Bacillus_cereus]	CPTF_Cd	0	0	0
UIJ66423.1	glycosyltransferase_family_4_protein_[Bacillus_cereus]	CPTF_Co	9435	16341.89937	173.2050808
UIJ66423.1	glycosyltransferase_family_4_protein_[Bacillus_cereus]	CPTF_Cu	0	0	0
UIJ66423.1	glycosyltransferase_family_4_protein_[Bacillus_cereus]	CPTF_Fe	0	0	0
UIJ66423.1	glycosyltransferase_family_4_protein_[Bacillus_cereus]	CPTF_Mn	0	0	0
UIJ66423.1	glycosyltransferase_family_4_protein_[Bacillus_cereus]	CPTF_Ni	0	0	0
UIJ66423.1	glycosyltransferase_family_4_protein_[Bacillus_cereus]	CPTF_U	0	0	0
UIJ66423.1	glycosyltransferase_family_4_protein_[Bacillus_cereus]	CPTF_metals_mix	258027.1	37442.83492	14.51120247
UIJ66423.1	glycosyltransferase_family_4_protein_[Bacillus_cereus]	CPTF_zcontrol	0	0	0
UIJ66430.1	pyridoxine/pyridoxal/pyridoxamine_kinase_[Bacillus_cereus]	CPTF_Al	115875.0667	26886.9535	23.20339851
UIJ66430.1	pyridoxine/pyridoxal/pyridoxamine_kinase_[Bacillus_cereus]	CPTF_Cd	110460	18481.46296	16.73136244
UIJ66430.1	pyridoxine/pyridoxal/pyridoxamine_kinase_[Bacillus_cereus]	CPTF_Co	124984.5667	51841.65885	41.47844829
UIJ66430.1	pyridoxine/pyridoxal/pyridoxamine_kinase_[Bacillus_cereus]	CPTF_Cu	110537.9	51016.13395	46.1526173
UIJ66430.1	pyridoxine/pyridoxal/pyridoxamine_kinase_[Bacillus_cereus]	CPTF_Fe	87154.23333	33652.85639	38.61299113
UIJ66430.1	pyridoxine/pyridoxal/pyridoxamine_kinase_[Bacillus_cereus]	CPTF_Mn	54587.86667	47605.60404	87.2091308
UIJ66430.1	pyridoxine/pyridoxal/pyridoxamine_kinase_[Bacillus_cereus]	CPTF_Ni	26627.73333	46120.58702	173.2050808
UIJ66430.1	pyridoxine/pyridoxal/pyridoxamine_kinase_[Bacillus_cereus]	CPTF_U	60651.66667	54770.9268	90.3040754
UIJ66430.1	pyridoxine/pyridoxal/pyridoxamine_kinase_[Bacillus_cereus]	CPTF_metals_mix	634824.8667	145901.8912	22.98301451
UIJ66430.1	pyridoxine/pyridoxal/pyridoxamine_kinase_[Bacillus_cereus]	CPTF_zcontrol	19947.16667	34549.50613	173.2050808
UIJ66432.1	FMN-dependent_NADH-azoreductase_[Bacillus_cereus]	CPTF_Al	4718118.767	529326.3057	11.2190119
UIJ66432.1	FMN-dependent_NADH-azoreductase_[Bacillus_cereus]	CPTF_Cd	4492870.333	343948.4932	7.655428883
UIJ66432.1	FMN-dependent_NADH-azoreductase_[Bacillus_cereus]	CPTF_Co	6116542.733	552454.6858	9.032139722
UIJ66432.1	FMN-dependent_NADH-azoreductase_[Bacillus_cereus]	CPTF_Cu	6823618.533	88180.17844	1.29227884
UIJ66432.1	FMN-dependent_NADH-azoreductase_[Bacillus_cereus]	CPTF_Fe	4341458.2	277414.9724	6.389903107
UIJ66432.1	FMN-dependent_NADH-azoreductase_[Bacillus_cereus]	CPTF_Mn	4685501.4	769106.3439	16.41460066
UIJ66432.1	FMN-dependent_NADH-azoreductase_[Bacillus_cereus]	CPTF_Ni	5119004.7	220489.2257	4.3072675
UIJ66432.1	FMN-dependent_NADH-azoreductase_[Bacillus_cereus]	CPTF_U	4723587.833	663630.0137	14.04927858
UIJ66432.1	FMN-dependent_NADH-azoreductase_[Bacillus_cereus]	CPTF_metals_mix	8839675.2	666303.068	7.537641971
UIJ66432.1	FMN-dependent_NADH-azoreductase_[Bacillus_cereus]	CPTF_zcontrol	4660773.4	281529.0851	6.040394178
UIJ66438.1	homoserine_dehydrogenase_[Bacillus_cereus]	CPTF_Al	916299.7	161824.804	17.66068503
UIJ66438.1	homoserine_dehydrogenase_[Bacillus_cereus]	CPTF_Cd	991299.2333	88803.6624	8.958310409
UIJ66438.1	homoserine_dehydrogenase_[Bacillus_cereus]	CPTF_Co	1040797.1	265589.2836	25.51787314
UIJ66438.1	homoserine_dehydrogenase_[Bacillus_cereus]	CPTF_Cu	773092.7667	210611.9679	27.24278081
UIJ66438.1	homoserine_dehydrogenase_[Bacillus_cereus]	CPTF_Fe	1084726.933	197707.0519	18.22643523
UIJ66438.1	homoserine_dehydrogenase_[Bacillus_cereus]	CPTF_Mn	933228.9667	155676.0131	16.68143818
UIJ66438.1	homoserine_dehydrogenase_[Bacillus_cereus]	CPTF_Ni	1120104.733	228475.7075	20.39770931
UIJ66438.1	homoserine_dehydrogenase_[Bacillus_cereus]	CPTF_U	896699.9667	372777.517	41.5721569
UIJ66438.1	homoserine_dehydrogenase_[Bacillus_cereus]	CPTF_metals_mix	1035361.7	178741.4285	17.26367013

UIJ66438.1	homoserine_dehydrogenase_[Bacillus_cereus]	CPTF_zcontrol	829765.6	89156.77987	10.74481515
UIJ66444.1	uracil-DNA_glycosylase_[Bacillus_cereus]	CPTF_Al	0	0	0
UIJ66444.1	uracil-DNA_glycosylase_[Bacillus_cereus]	CPTF_Cd	0	0	0
UIJ66444.1	uracil-DNA_glycosylase_[Bacillus_cereus]	CPTF_Co	0	0	0
UIJ66444.1	uracil-DNA_glycosylase_[Bacillus_cereus]	CPTF_Cu	0	0	0
UIJ66444.1	uracil-DNA_glycosylase_[Bacillus_cereus]	CPTF_Fe	0	0	0
UIJ66444.1	uracil-DNA_glycosylase_[Bacillus_cereus]	CPTF_Mn	23114	40034.62237	173.2050808
UIJ66444.1	uracil-DNA_glycosylase_[Bacillus_cereus]	CPTF_Ni	0	0	0
UIJ66444.1	uracil-DNA_glycosylase_[Bacillus_cereus]	CPTF_U	0	0	0
UIJ66444.1	uracil-DNA_glycosylase_[Bacillus_cereus]	CPTF_metals_mix	0	0	0
UIJ66444.1	uracil-DNA_glycosylase_[Bacillus_cereus]	CPTF_zcontrol	0	0	0
UIJ66452.1	heme-dependent_peroxidase_[Bacillus_cereus]	CPTF_Al	4397483.4	213579.9346	4.85686733
UIJ66452.1	heme-dependent_peroxidase_[Bacillus_cereus]	CPTF_Cd	4857314.167	138432.9824	2.849990296
UIJ66452.1	heme-dependent_peroxidase_[Bacillus_cereus]	CPTF_Co	4418925.1	243026.9786	5.499685401
UIJ66452.1	heme-dependent_peroxidase_[Bacillus_cereus]	CPTF_Cu	4692777.167	108708.3857	2.316504317
UIJ66452.1	heme-dependent_peroxidase_[Bacillus_cereus]	CPTF_Fe	4210378.7	148218.1546	3.52030459
UIJ66452.1	heme-dependent_peroxidase_[Bacillus_cereus]	CPTF_Mn	4469645.933	839129.0688	18.77394947
UIJ66452.1	heme-dependent_peroxidase_[Bacillus_cereus]	CPTF_Ni	3696408.633	512451.1222	13.86348678
UIJ66452.1	heme-dependent_peroxidase_[Bacillus_cereus]	CPTF_U	3797615.1	692527.2149	18.23584531
UIJ66452.1	heme-dependent_peroxidase_[Bacillus_cereus]	CPTF_metals_mix	7769389.4	260638.9986	3.354690892
UIJ66452.1	heme-dependent_peroxidase_[Bacillus_cereus]	CPTF_zcontrol	4693809.1	122442.9052	2.608604283
UIJ66453.1	phosphate_acetyltransferase_[Bacillus_cereus]	CPTF_Al	2624801.733	937925.7033	35.73320192
UIJ66453.1	phosphate_acetyltransferase_[Bacillus_cereus]	CPTF_Cd	2931360.967	126706.2075	4.322436197
UIJ66453.1	phosphate_acetyltransferase_[Bacillus_cereus]	CPTF_Co	2570038.633	210276.7762	8.181852734
UIJ66453.1	phosphate_acetyltransferase_[Bacillus_cereus]	CPTF_Cu	2808186.667	138709.9571	4.939484925
UIJ66453.1	phosphate_acetyltransferase_[Bacillus_cereus]	CPTF_Fe	2899621.333	455972.4365	15.72524078
UIJ66453.1	phosphate_acetyltransferase_[Bacillus_cereus]	CPTF_Mn	2319477.667	276216.3376	11.90855776
UIJ66453.1	phosphate_acetyltransferase_[Bacillus_cereus]	CPTF_Ni	2177583	322016.7078	14.78780408
UIJ66453.1	phosphate_acetyltransferase_[Bacillus_cereus]	CPTF_U	2107302.333	414986.5139	19.69278481
UIJ66453.1	phosphate_acetyltransferase_[Bacillus_cereus]	CPTF_metals_mix	3503342.467	550112.8062	15.702513
UIJ66453.1	phosphate_acetyltransferase_[Bacillus_cereus]	CPTF_zcontrol	2329250.233	357282.9654	15.33896875
UIJ66461.1	HD_domain-containing_protein_[Bacillus_cereus]	CPTF_Al	21241.13333	36790.72214	173.2050808
UIJ66461.1	HD_domain-containing_protein_[Bacillus_cereus]	CPTF_Cd	81076.86667	52706.56775	65.00814587
UIJ66461.1	HD_domain-containing_protein_[Bacillus_cereus]	CPTF_Co	30352.63333	52572.30308	173.2050808
UIJ66461.1	HD_domain-containing_protein_[Bacillus_cereus]	CPTF_Cu	31840.56667	55149.47921	173.2050808
UIJ66461.1	HD_domain-containing_protein_[Bacillus_cereus]	CPTF_Fe	60571.4	55341.62848	91.36593916
UIJ66461.1	HD_domain-containing_protein_[Bacillus_cereus]	CPTF_Mn	11923.1	20651.41498	173.2050808
UIJ66461.1	HD_domain-containing_protein_[Bacillus_cereus]	CPTF_Ni	18002.33333	31180.95599	173.2050808
UIJ66461.1	HD_domain-containing_protein_[Bacillus_cereus]	CPTF_U	0	0	0
UIJ66461.1	HD_domain-containing_protein_[Bacillus_cereus]	CPTF_metals_mix	106440.9667	44074.75227	41.40769635
UIJ66461.1	HD_domain-containing_protein_[Bacillus_cereus]	CPTF_zcontrol	29881.9	51756.96903	173.2050808
UIJ66462.1	4-oxalocrotonate_tautomerase_[Bacillus_cereus]	CPTF_Al	0	0	0
UIJ66462.1	4-oxalocrotonate_tautomerase_[Bacillus_cereus]	CPTF_Cd	0	0	0
UIJ66462.1	4-oxalocrotonate_tautomerase_[Bacillus_cereus]	CPTF_Co	0	0	0
UIJ66462.1	4-oxalocrotonate_tautomerase_[Bacillus_cereus]	CPTF_Cu	0	0	0
UIJ66462.1	4-oxalocrotonate_tautomerase_[Bacillus_cereus]	CPTF_Fe	0	0	0
UIJ66462.1	4-oxalocrotonate_tautomerase_[Bacillus_cereus]	CPTF_Mn	0	0	0
UIJ66462.1	4-oxalocrotonate_tautomerase_[Bacillus_cereus]	CPTF_Ni	41408.33333	71721.33719	173.2050808
UIJ66462.1	4-oxalocrotonate_tautomerase_[Bacillus_cereus]	CPTF_U	0	0	0
UIJ66462.1	4-oxalocrotonate_tautomerase_[Bacillus_cereus]	CPTF_metals_mix	309767.6667	191353.6746	61.77328855
UIJ66462.1	4-oxalocrotonate_tautomerase_[Bacillus_cereus]	CPTF_zcontrol	0	0	0
UIJ66464.1	YwhD_family_protein_[Bacillus_cereus]	CPTF_Al	11566.6	20033.93887	173.2050808
UIJ66464.1	YwhD_family_protein_[Bacillus_cereus]	CPTF_Cd	0	0	0
UIJ66464.1	YwhD_family_protein_[Bacillus_cereus]	CPTF_Co	42239.73333	45592.45445	107.9373634
UIJ66464.1	YwhD_family_protein_[Bacillus_cereus]	CPTF_Cu	11655.3	20187.57178	173.2050808
UIJ66464.1	YwhD_family_protein_[Bacillus_cereus]	CPTF_Fe	0	0	0
UIJ66464.1	YwhD_family_protein_[Bacillus_cereus]	CPTF_Mn	14985.4	25955.47417	173.2050808
UIJ66464.1	YwhD_family_protein_[Bacillus_cereus]	CPTF_Ni	21927.73333	37979.94823	173.2050808
UIJ66464.1	YwhD_family_protein_[Bacillus_cereus]	CPTF_U	0	0	0
UIJ66464.1	YwhD_family_protein_[Bacillus_cereus]	CPTF_metals_mix	350043.3333	53580.83489	15.30691483
UIJ66464.1	YwhD_family_protein_[Bacillus_cereus]	CPTF_zcontrol	0	0	0

UIJ66466.1	glycerophosphodiester_phosphodiesterase_[Bacillus_cereus]	CPTF_Al	67864.33333	58774.66711	86.60612169
UIJ66466.1	glycerophosphodiester_phosphodiesterase_[Bacillus_cereus]	CPTF_Cd	232705.3333	212922.647	91.49882557
UIJ66466.1	glycerophosphodiester_phosphodiesterase_[Bacillus_cereus]	CPTF_Co	40502.6	14513.30255	35.83301455
UIJ66466.1	glycerophosphodiester_phosphodiesterase_[Bacillus_cereus]	CPTF_Cu	97065.36667	143067.9419	147.3933977
UIJ66466.1	glycerophosphodiester_phosphodiesterase_[Bacillus_cereus]	CPTF_Fe	105086.2	73150.36359	69.60986656
UIJ66466.1	glycerophosphodiester_phosphodiesterase_[Bacillus_cereus]	CPTF_Mn	96887	167813.2066	173.2050808
UIJ66466.1	glycerophosphodiester_phosphodiesterase_[Bacillus_cereus]	CPTF_Ni	0	0	0
UIJ66466.1	glycerophosphodiester_phosphodiesterase_[Bacillus_cereus]	CPTF_U	13809.96667	23919.56392	173.2050808
UIJ66466.1	glycerophosphodiester_phosphodiesterase_[Bacillus_cereus]	CPTF_metals_mix	362334.3333	142824.8337	39.41796858
UIJ66466.1	glycerophosphodiester_phosphodiesterase_[Bacillus_cereus]	CPTF_zcontrol	79138.93333	137072.6534	173.2050808
UIJ66469.1	spermidine_synthase_[Bacillus_cereus]	CPTF_Al	2309114	154589.5194	6.694754757
UIJ66469.1	spermidine_synthase_[Bacillus_cereus]	CPTF_Cd	1915319.033	195224.6929	10.19280285
UIJ66469.1	spermidine_synthase_[Bacillus_cereus]	CPTF_Co	2305195.367	875176.783	37.96540613
UIJ66469.1	spermidine_synthase_[Bacillus_cereus]	CPTF_Cu	1571644	494146.4886	31.44137531
UIJ66469.1	spermidine_synthase_[Bacillus_cereus]	CPTF_Fe	1695935.7	703938.3166	41.50737063
UIJ66469.1	spermidine_synthase_[Bacillus_cereus]	CPTF_Mn	2080389.433	377518.9327	18.14655115
UIJ66469.1	spermidine_synthase_[Bacillus_cereus]	CPTF_Ni	2103863.333	385736.3893	18.33466952
UIJ66469.1	spermidine_synthase_[Bacillus_cereus]	CPTF_U	1064210.667	231536.8213	21.75667173
UIJ66469.1	spermidine_synthase_[Bacillus_cereus]	CPTF_metals_mix	1260342	594541.1493	47.1730014
UIJ66469.1	spermidine_synthase_[Bacillus_cereus]	CPTF_zcontrol	1474355.467	287552.4863	19.50360634
UIJ66470.1	agmatinase_[Bacillus_cereus]	CPTF_Al	2699703.3	406249.0764	15.04791569
UIJ66470.1	agmatinase_[Bacillus_cereus]	CPTF_Cd	3089742.067	25779.04988	0.834343105
UIJ66470.1	agmatinase_[Bacillus_cereus]	CPTF_Co	3122184.6	257820.9121	8.25770879
UIJ66470.1	agmatinase_[Bacillus_cereus]	CPTF_Cu	2682397	345523.0109	12.88112874
UIJ66470.1	agmatinase_[Bacillus_cereus]	CPTF_Fe	2974726.667	117591.0235	3.953002635
UIJ66470.1	agmatinase_[Bacillus_cereus]	CPTF_Mn	2592863.9	444281.5519	17.13478104
UIJ66470.1	agmatinase_[Bacillus_cereus]	CPTF_Ni	1883210.967	376819.8595	20.00943421
UIJ66470.1	agmatinase_[Bacillus_cereus]	CPTF_U	2572714.133	435241.3003	16.91759277
UIJ66470.1	agmatinase_[Bacillus_cereus]	CPTF_metals_mix	2477442.733	499928.3535	20.17920926
UIJ66470.1	agmatinase_[Bacillus_cereus]	CPTF_zcontrol	2782812.5	350453.1306	12.59348701
UIJ66475.1	arginine--tRNA_ligase_[Bacillus_cereus]	CPTF_Al	1829647.833	272262.26	14.88058276
UIJ66475.1	arginine--tRNA_ligase_[Bacillus_cereus]	CPTF_Cd	1701660.1	26339.34202	1.547861528
UIJ66475.1	arginine--tRNA_ligase_[Bacillus_cereus]	CPTF_Co	2038006.633	272556.6342	13.37368729
UIJ66475.1	arginine--tRNA_ligase_[Bacillus_cereus]	CPTF_Cu	1822010.233	139961.5852	7.681712353
UIJ66475.1	arginine--tRNA_ligase_[Bacillus_cereus]	CPTF_Fe	1938951.633	268844.2065	13.86544161
UIJ66475.1	arginine--tRNA_ligase_[Bacillus_cereus]	CPTF_Mn	1998672.833	209077.0042	10.46079182
UIJ66475.1	arginine--tRNA_ligase_[Bacillus_cereus]	CPTF_Ni	1892498.433	56435.5663	2.982066738
UIJ66475.1	arginine--tRNA_ligase_[Bacillus_cereus]	CPTF_U	1728213.833	443809.1574	25.68022248
UIJ66475.1	arginine--tRNA_ligase_[Bacillus_cereus]	CPTF_metals_mix	1614005.933	180894.787	11.20781425
UIJ66475.1	arginine--tRNA_ligase_[Bacillus_cereus]	CPTF_zcontrol	1823498.933	363727.116	19.9466591
UIJ66480.1	type_I_methionyl_aminopeptidase_[Bacillus_cereus]	CPTF_Al	914566.0333	185098.5601	20.23894977
UIJ66480.1	type_I_methionyl_aminopeptidase_[Bacillus_cereus]	CPTF_Cd	919633.3	32508.46632	3.534937928
UIJ66480.1	type_I_methionyl_aminopeptidase_[Bacillus_cereus]	CPTF_Co	856384.3333	74294.32148	8.675348041
UIJ66480.1	type_I_methionyl_aminopeptidase_[Bacillus_cereus]	CPTF_Cu	1011511.1	68538.28919	6.775831644
UIJ66480.1	type_I_methionyl_aminopeptidase_[Bacillus_cereus]	CPTF_Fe	934743.4333	127879.5822	13.68071469
UIJ66480.1	type_I_methionyl_aminopeptidase_[Bacillus_cereus]	CPTF_Mn	878228.3333	203784.3587	23.20402918
UIJ66480.1	type_I_methionyl_aminopeptidase_[Bacillus_cereus]	CPTF_Ni	995600.8333	122238.4087	12.27785319
UIJ66480.1	type_I_methionyl_aminopeptidase_[Bacillus_cereus]	CPTF_U	1149713.767	199038.796	17.31203033
UIJ66480.1	type_I_methionyl_aminopeptidase_[Bacillus_cereus]	CPTF_metals_mix	865657.7667	158229.5509	18.27853419
UIJ66480.1	type_I_methionyl_aminopeptidase_[Bacillus_cereus]	CPTF_zcontrol	851330.3333	184246.5896	21.64219721
UIJ66491.1	acetyl-CoA_C-acetyltransferase_[Bacillus_cereus]	CPTF_Al	742822.8333	351341.8335	47.29820056
UIJ66491.1	acetyl-CoA_C-acetyltransferase_[Bacillus_cereus]	CPTF_Cd	1173902.433	234971.6509	20.01628451
UIJ66491.1	acetyl-CoA_C-acetyltransferase_[Bacillus_cereus]	CPTF_Co	686625.3	159462.1253	23.22403869
UIJ66491.1	acetyl-CoA_C-acetyltransferase_[Bacillus_cereus]	CPTF_Cu	638754	76575.23484	11.98822001
UIJ66491.1	acetyl-CoA_C-acetyltransferase_[Bacillus_cereus]	CPTF_Fe	733402.2667	166491.9978	22.70132033
UIJ66491.1	acetyl-CoA_C-acetyltransferase_[Bacillus_cereus]	CPTF_Mn	695356.1667	182885.8541	26.3010329
UIJ66491.1	acetyl-CoA_C-acetyltransferase_[Bacillus_cereus]	CPTF_Ni	304713.0667	192248.2468	63.09156641
UIJ66491.1	acetyl-CoA_C-acetyltransferase_[Bacillus_cereus]	CPTF_U	480496	210269.6073	43.76094853
UIJ66491.1	acetyl-CoA_C-acetyltransferase_[Bacillus_cereus]	CPTF_metals_mix	1087607.067	137711.6534	12.66189395
UIJ66491.1	acetyl-CoA_C-acetyltransferase_[Bacillus_cereus]	CPTF_zcontrol	570312.1667	128552.515	22.54072813
UIJ66492.1	3-hydroxybutyryl-CoA_dehydrogenase_[Bacillus_cereus]	CPTF_Al	1353538	92091.55131	6.803765488

UIJ66492.1	3-hydroxybutyryl-CoA_dehydrogenase_[Bacillus_cereus]	CPTF_Cd	1295946.5	63532.35978	4.90239063
UIJ66492.1	3-hydroxybutyryl-CoA_dehydrogenase_[Bacillus_cereus]	CPTF_Co	1201552	104103.71	8.664103596
UIJ66492.1	3-hydroxybutyryl-CoA_dehydrogenase_[Bacillus_cereus]	CPTF_Cu	1299590.667	141357.0714	10.87704575
UIJ66492.1	3-hydroxybutyryl-CoA_dehydrogenase_[Bacillus_cereus]	CPTF_Fe	1267969.6	243431.4487	19.19852406
UIJ66492.1	3-hydroxybutyryl-CoA_dehydrogenase_[Bacillus_cereus]	CPTF_Mn	1255740.333	126542.8503	10.07715106
UIJ66492.1	3-hydroxybutyryl-CoA_dehydrogenase_[Bacillus_cereus]	CPTF_Ni	1392152.333	321086.8736	23.06406174
UIJ66492.1	3-hydroxybutyryl-CoA_dehydrogenase_[Bacillus_cereus]	CPTF_U	1360565.8	440469.7446	32.37401268
UIJ66492.1	3-hydroxybutyryl-CoA_dehydrogenase_[Bacillus_cereus]	CPTF_metals_mix	1158217.667	108245.4051	9.345860304
UIJ66492.1	3-hydroxybutyryl-CoA_dehydrogenase_[Bacillus_cereus]	CPTF_zcontrol	1325470.667	129499.6797	9.770090203
UIJ66493.1	acyl-CoA_dehydrogenase_[Bacillus_cereus]	CPTF_Al	224786	87497.86808	38.92496334
UIJ66493.1	acyl-CoA_dehydrogenase_[Bacillus_cereus]	CPTF_Cd	246874.3333	31351.85832	12.69952121
UIJ66493.1	acyl-CoA_dehydrogenase_[Bacillus_cereus]	CPTF_Co	114654.4667	80258.95969	70.00072655
UIJ66493.1	acyl-CoA_dehydrogenase_[Bacillus_cereus]	CPTF_Cu	115631.4	45427.16512	39.28618448
UIJ66493.1	acyl-CoA_dehydrogenase_[Bacillus_cereus]	CPTF_Fe	124371.2333	62605.01791	50.33721724
UIJ66493.1	acyl-CoA_dehydrogenase_[Bacillus_cereus]	CPTF_Mn	92169.03333	107569.8147	116.7092794
UIJ66493.1	acyl-CoA_dehydrogenase_[Bacillus_cereus]	CPTF_Ni	211693.3333	66485.37534	31.40645683
UIJ66493.1	acyl-CoA_dehydrogenase_[Bacillus_cereus]	CPTF_U	244375.6667	81278.79713	33.2597751
UIJ66493.1	acyl-CoA_dehydrogenase_[Bacillus_cereus]	CPTF_metals_mix	159192.5333	51309.51659	32.23110753
UIJ66493.1	acyl-CoA_dehydrogenase_[Bacillus_cereus]	CPTF_zcontrol	226306.6667	45994.84735	20.32412391
UIJ66494.1	acyl-CoA_dehydrogenase_AcdA_[Bacillus_cereus]	CPTF_Al	184107.3333	236027.7902	128.2011889
UIJ66494.1	acyl-CoA_dehydrogenase_AcdA_[Bacillus_cereus]	CPTF_Cd	431150	165334.8636	38.34741125
UIJ66494.1	acyl-CoA_dehydrogenase_AcdA_[Bacillus_cereus]	CPTF_Co	273791	49577.11984	18.1076514
UIJ66494.1	acyl-CoA_dehydrogenase_AcdA_[Bacillus_cereus]	CPTF_Cu	232555.9333	92417.09058	39.73972595
UIJ66494.1	acyl-CoA_dehydrogenase_AcdA_[Bacillus_cereus]	CPTF_Fe	203049.3333	351691.7618	173.2050808
UIJ66494.1	acyl-CoA_dehydrogenase_AcdA_[Bacillus_cereus]	CPTF_Mn	174140	151024.893	86.72613586
UIJ66494.1	acyl-CoA_dehydrogenase_AcdA_[Bacillus_cereus]	CPTF_Ni	282418.8333	138502.9552	49.0416852
UIJ66494.1	acyl-CoA_dehydrogenase_AcdA_[Bacillus_cereus]	CPTF_U	267200	234751.2666	87.85601295
UIJ66494.1	acyl-CoA_dehydrogenase_AcdA_[Bacillus_cereus]	CPTF_metals_mix	380221.9667	49623.68962	13.05124216
UIJ66494.1	acyl-CoA_dehydrogenase_AcdA_[Bacillus_cereus]	CPTF_zcontrol	141876.5333	137505.0967	96.91884451
UIJ66496.1	DNA-directed_RNA_polymerase_subunit_delta_[Bacillus_cereus]	CPTF_Al	1257041	374802.7042	29.81626726
UIJ66496.1	DNA-directed_RNA_polymerase_subunit_delta_[Bacillus_cereus]	CPTF_Cd	1470000	235796.5225	16.04057976
UIJ66496.1	DNA-directed_RNA_polymerase_subunit_delta_[Bacillus_cereus]	CPTF_Co	1069225	389338.9231	36.41318928
UIJ66496.1	DNA-directed_RNA_polymerase_subunit_delta_[Bacillus_cereus]	CPTF_Cu	830520.3333	75825.92342	9.129929801
UIJ66496.1	DNA-directed_RNA_polymerase_subunit_delta_[Bacillus_cereus]	CPTF_Fe	1486666.667	1302932.59	87.64120559
UIJ66496.1	DNA-directed_RNA_polymerase_subunit_delta_[Bacillus_cereus]	CPTF_Mn	1796044.033	1072507.153	59.71496984
UIJ66496.1	DNA-directed_RNA_polymerase_subunit_delta_[Bacillus_cereus]	CPTF_Ni	0	0	0
UIJ66496.1	DNA-directed_RNA_polymerase_subunit_delta_[Bacillus_cereus]	CPTF_U	0	0	0
UIJ66496.1	DNA-directed_RNA_polymerase_subunit_delta_[Bacillus_cereus]	CPTF_metals_mix	2538683.7	593040.9352	23.36017422
UIJ66496.1	DNA-directed_RNA_polymerase_subunit_delta_[Bacillus_cereus]	CPTF_zcontrol	1458086.667	706937.3257	48.48390304
UIJ66497.1	CTP_synthase_[Bacillus_cereus]	CPTF_Al	7107683.4	197455.5735	2.778058087
UIJ66497.1	CTP_synthase_[Bacillus_cereus]	CPTF_Cd	7335200.033	329119.3798	4.486849415
UIJ66497.1	CTP_synthase_[Bacillus_cereus]	CPTF_Co	7993005.3	591403.3257	7.399010804
UIJ66497.1	CTP_synthase_[Bacillus_cereus]	CPTF_Cu	6771122.333	485825.1756	7.174957883
UIJ66497.1	CTP_synthase_[Bacillus_cereus]	CPTF_Fe	7390078.833	189316.1636	2.561761083
UIJ66497.1	CTP_synthase_[Bacillus_cereus]	CPTF_Mn	7433602.2	952958.0128	12.81959926
UIJ66497.1	CTP_synthase_[Bacillus_cereus]	CPTF_Ni	7763217.933	621566.2746	8.006554498
UIJ66497.1	CTP_synthase_[Bacillus_cereus]	CPTF_U	8244783.7	336379.4776	4.07990664
UIJ66497.1	CTP_synthase_[Bacillus_cereus]	CPTF_metals_mix	4697151.233	413307.3726	8.799107205
UIJ66497.1	CTP_synthase_[Bacillus_cereus]	CPTF_zcontrol	8262506.1	125140.557	1.51455933
UIJ66499.1	sporulation_initiation_phosphotransferase_Spo0F_[Bacillus_cereus]	CPTF_Al	1253333.333	56862.40703	4.536894178
UIJ66499.1	sporulation_initiation_phosphotransferase_Spo0F_[Bacillus_cereus]	CPTF_Cd	1458042.333	447054.2891	30.66126949
UIJ66499.1	sporulation_initiation_phosphotransferase_Spo0F_[Bacillus_cereus]	CPTF_Co	1618772.667	495788.0384	30.62740363
UIJ66499.1	sporulation_initiation_phosphotransferase_Spo0F_[Bacillus_cereus]	CPTF_Cu	1679072.667	341266.031	20.32467312
UIJ66499.1	sporulation_initiation_phosphotransferase_Spo0F_[Bacillus_cereus]	CPTF_Fe	1778869.333	416545.9542	23.4163323
UIJ66499.1	sporulation_initiation_phosphotransferase_Spo0F_[Bacillus_cereus]	CPTF_Mn	1484003.667	431315.984	29.06434759
UIJ66499.1	sporulation_initiation_phosphotransferase_Spo0F_[Bacillus_cereus]	CPTF_Ni	1662989.333	361944.3166	21.76468059
UIJ66499.1	sporulation_initiation_phosphotransferase_Spo0F_[Bacillus_cereus]	CPTF_U	1253333.333	50332.22957	4.01586938
UIJ66499.1	sporulation_initiation_phosphotransferase_Spo0F_[Bacillus_cereus]	CPTF_metals_mix	2528254.767	630213.4375	24.92681694
UIJ66499.1	sporulation_initiation_phosphotransferase_Spo0F_[Bacillus_cereus]	CPTF_zcontrol	2119994.333	79707.81215	3.75981251
UIJ66500.1	fructose-bisphosphate_aldolase_[Bacillus_cereus]	CPTF_Al	26920943.17	2604015.009	9.672822354
UIJ66500.1	fructose-bisphosphate_aldolase_[Bacillus_cereus]	CPTF_Cd	25869773.13	2290515.242	8.854021371

UIJ66500.1	fructose-bisphosphate_aldolase_[Bacillus_cereus]	CPTF_Co	26557471.37	1300277.08	4.896087667
UIJ66500.1	fructose-bisphosphate_aldolase_[Bacillus_cereus]	CPTF_Cu	24623008.63	2611718.533	10.60682133
UIJ66500.1	fructose-bisphosphate_aldolase_[Bacillus_cereus]	CPTF_Fe	23145827.47	708235.7868	3.059885363
UIJ66500.1	fructose-bisphosphate_aldolase_[Bacillus_cereus]	CPTF_Mn	25808610.27	4466523.706	17.30633172
UIJ66500.1	fructose-bisphosphate_aldolase_[Bacillus_cereus]	CPTF_Ni	22447662.27	3988011.567	17.76582131
UIJ66500.1	fructose-bisphosphate_aldolase_[Bacillus_cereus]	CPTF_U	17751673.63	2811937.313	15.84040678
UIJ66500.1	fructose-bisphosphate_aldolase_[Bacillus_cereus]	CPTF_metals_mix	31496005.33	1398506.014	4.440264722
UIJ66500.1	fructose-bisphosphate_aldolase_[Bacillus_cereus]	CPTF_zcontrol	23858768.43	810178.488	3.395726356
UIJ66502.1	class_II_fructose-bisphosphatase_[Bacillus_cereus]	CPTF_Al	9600490.2	834337.8543	8.690575553
UIJ66502.1	class_II_fructose-bisphosphatase_[Bacillus_cereus]	CPTF_Cd	8103064.767	497799.8725	6.143353001
UIJ66502.1	class_II_fructose-bisphosphatase_[Bacillus_cereus]	CPTF_Co	8373359.567	664595.6695	7.937025327
UIJ66502.1	class_II_fructose-bisphosphatase_[Bacillus_cereus]	CPTF_Cu	8246881.8	342052.2562	4.147655617
UIJ66502.1	class_II_fructose-bisphosphatase_[Bacillus_cereus]	CPTF_Fe	8477001.2	602766.7717	7.110613264
UIJ66502.1	class_II_fructose-bisphosphatase_[Bacillus_cereus]	CPTF_Mn	8933372.333	1453666.24	16.27231225
UIJ66502.1	class_II_fructose-bisphosphatase_[Bacillus_cereus]	CPTF_Ni	8912700.967	289291.4288	3.245833445
UIJ66502.1	class_II_fructose-bisphosphatase_[Bacillus_cereus]	CPTF_U	8725849	288677.7758	3.308305883
UIJ66502.1	class_II_fructose-bisphosphatase_[Bacillus_cereus]	CPTF_metals_mix	7430899.157	977308.7474	13.15195815
UIJ66502.1	class_II_fructose-bisphosphatase_[Bacillus_cereus]	CPTF_zcontrol	9243665.933	319884.6033	3.460581609
UIJ66503.1	transcription_termination_factor_Rho_[Bacillus_cereus]	CPTF_Al	2171033.4	328719.7355	15.14116436
UIJ66503.1	transcription_termination_factor_Rho_[Bacillus_cereus]	CPTF_Cd	2527640.867	425955.4801	16.85189877
UIJ66503.1	transcription_termination_factor_Rho_[Bacillus_cereus]	CPTF_Co	2436019.833	52512.55959	2.155670445
UIJ66503.1	transcription_termination_factor_Rho_[Bacillus_cereus]	CPTF_Cu	2168772.4	174869.7603	8.063075697
UIJ66503.1	transcription_termination_factor_Rho_[Bacillus_cereus]	CPTF_Fe	2114708.467	156320.9769	7.392081666
UIJ66503.1	transcription_termination_factor_Rho_[Bacillus_cereus]	CPTF_Mn	2035028.933	319975.1622	15.72337164
UIJ66503.1	transcription_termination_factor_Rho_[Bacillus_cereus]	CPTF_Ni	1509037.5	193985.5569	12.85491957
UIJ66503.1	transcription_termination_factor_Rho_[Bacillus_cereus]	CPTF_U	1409618.667	279501.7975	19.82818503
UIJ66503.1	transcription_termination_factor_Rho_[Bacillus_cereus]	CPTF_metals_mix	2296586.9	19707.23868	0.858109862
UIJ66503.1	transcription_termination_factor_Rho_[Bacillus_cereus]	CPTF_zcontrol	1962922.233	321057.9117	16.35611978
UIJ66504.1	type_B_50S_ribosomal_protein_L31_[Bacillus_cereus]	CPTF_Al	780467	144677.3081	18.53727424
UIJ66504.1	type_B_50S_ribosomal_protein_L31_[Bacillus_cereus]	CPTF_Cd	1127226.367	369499.7442	32.77955122
UIJ66504.1	type_B_50S_ribosomal_protein_L31_[Bacillus_cereus]	CPTF_Co	787181.5	67960.54574	8.633402302
UIJ66504.1	type_B_50S_ribosomal_protein_L31_[Bacillus_cereus]	CPTF_Cu	787733	199382.2794	25.31089587
UIJ66504.1	type_B_50S_ribosomal_protein_L31_[Bacillus_cereus]	CPTF_Fe	848572.9	261216.5507	30.78304182
UIJ66504.1	type_B_50S_ribosomal_protein_L31_[Bacillus_cereus]	CPTF_Mn	1387542.567	1332698.326	96.04738321
UIJ66504.1	type_B_50S_ribosomal_protein_L31_[Bacillus_cereus]	CPTF_Ni	378335.2333	121730.0488	32.17518171
UIJ66504.1	type_B_50S_ribosomal_protein_L31_[Bacillus_cereus]	CPTF_U	622240.9	289327.5681	46.49767768
UIJ66504.1	type_B_50S_ribosomal_protein_L31_[Bacillus_cereus]	CPTF_metals_mix	6217740.667	1090609.746	17.54029003
UIJ66504.1	type_B_50S_ribosomal_protein_L31_[Bacillus_cereus]	CPTF_zcontrol	567365.2333	243690.3325	42.95122756
UIJ66506.1	peptide_chain_release_factor_N(5)-glutamine_methyltransferase_[Bacillus_cereus]	CPTF_Al	15876.3	27498.55824	173.2050808
UIJ66506.1	peptide_chain_release_factor_N(5)-glutamine_methyltransferase_[Bacillus_cereus]	CPTF_Cd	35192.5	5380.287839	15.28816606
UIJ66506.1	peptide_chain_release_factor_N(5)-glutamine_methyltransferase_[Bacillus_cereus]	CPTF_Co	19315.26667	22021.50121	114.0108578
UIJ66506.1	peptide_chain_release_factor_N(5)-glutamine_methyltransferase_[Bacillus_cereus]	CPTF_Cu	16634.13333	14771.81441	88.80423233
UIJ66506.1	peptide_chain_release_factor_N(5)-glutamine_methyltransferase_[Bacillus_cereus]	CPTF_Fe	15179.7	14195.19882	93.51435679
UIJ66506.1	peptide_chain_release_factor_N(5)-glutamine_methyltransferase_[Bacillus_cereus]	CPTF_Mn	7517.9	13021.38477	173.2050808
UIJ66506.1	peptide_chain_release_factor_N(5)-glutamine_methyltransferase_[Bacillus_cereus]	CPTF_Ni	0	0	0
UIJ66506.1	peptide_chain_release_factor_N(5)-glutamine_methyltransferase_[Bacillus_cereus]	CPTF_U	0	0	0
UIJ66506.1	peptide_chain_release_factor_N(5)-glutamine_methyltransferase_[Bacillus_cereus]	CPTF_metals_mix	29141.43333	26833.8967	92.08159527
UIJ66506.1	peptide_chain_release_factor_N(5)-glutamine_methyltransferase_[Bacillus_cereus]	CPTF_zcontrol	7009.8	12141.32975	173.2050808
UIJ66509.1	threonylcarbamoyl-AMP_synthase_[Bacillus_cereus]	CPTF_Al	169699	147098.381	86.68193741
UIJ66509.1	threonylcarbamoyl-AMP_synthase_[Bacillus_cereus]	CPTF_Cd	282123.3333	58368.87124	20.68913285
UIJ66509.1	threonylcarbamoyl-AMP_synthase_[Bacillus_cereus]	CPTF_Co	198880	28880.47278	14.52155711
UIJ66509.1	threonylcarbamoyl-AMP_synthase_[Bacillus_cereus]	CPTF_Cu	85695.93333	98194.1465	114.5843714
UIJ66509.1	threonylcarbamoyl-AMP_synthase_[Bacillus_cereus]	CPTF_Fe	133662.6667	116246.9085	86.97036461
UIJ66509.1	threonylcarbamoyl-AMP_synthase_[Bacillus_cereus]	CPTF_Mn	86620.66667	150031.3957	173.2050808
UIJ66509.1	threonylcarbamoyl-AMP_synthase_[Bacillus_cereus]	CPTF_Ni	253646	2264.977042	0.892967775
UIJ66509.1	threonylcarbamoyl-AMP_synthase_[Bacillus_cereus]	CPTF_U	191102	94891.23852	49.65475951
UIJ66509.1	threonylcarbamoyl-AMP_synthase_[Bacillus_cereus]	CPTF_metals_mix	0	0	0
UIJ66509.1	threonylcarbamoyl-AMP_synthase_[Bacillus_cereus]	CPTF_zcontrol	198724.3333	11733.6806	5.904501175
UIJ66512.1	flavin_reductase_family_protein_[Bacillus_cereus]	CPTF_Al	502389.6333	63834.23362	12.70612078
UIJ66512.1	flavin_reductase_family_protein_[Bacillus_cereus]	CPTF_Cd	727495.6667	109213.5729	15.01226439
UIJ66512.1	flavin_reductase_family_protein_[Bacillus_cereus]	CPTF_Co	493358.9333	21504.61301	4.358816991

UIJ66512.1	flavin_reductase_family_protein_[Bacillus_cereus]	CPTF_Cu	265924	48934.18828	18.40156897
UIJ66512.1	flavin_reductase_family_protein_[Bacillus_cereus]	CPTF_Fe	553750.6	81579.24701	14.73212797
UIJ66512.1	flavin_reductase_family_protein_[Bacillus_cereus]	CPTF_Mn	595878	209666.6905	35.18617745
UIJ66512.1	flavin_reductase_family_protein_[Bacillus_cereus]	CPTF_Ni	562157.7333	12547.7756	2.232073821
UIJ66512.1	flavin_reductase_family_protein_[Bacillus_cereus]	CPTF_U	256330.6667	35234.82746	13.74584942
UIJ66512.1	flavin_reductase_family_protein_[Bacillus_cereus]	CPTF_metals_mix	1021842.3	240119.0334	23.49863902
UIJ66512.1	flavin_reductase_family_protein_[Bacillus_cereus]	CPTF_zcontrol	430590	20788.63783	4.827942552
UIJ66514.1	PTS_glucose_transporter_subunit_IIA_[Bacillus_cereus]	CPTF_Al	4066836.333	42801.81512	1.05245974
UIJ66514.1	PTS_glucose_transporter_subunit_IIA_[Bacillus_cereus]	CPTF_Cd	4303282.333	121046.2061	2.812880883
UIJ66514.1	PTS_glucose_transporter_subunit_IIA_[Bacillus_cereus]	CPTF_Co	3856014	154460.3344	4.005699522
UIJ66514.1	PTS_glucose_transporter_subunit_IIA_[Bacillus_cereus]	CPTF_Cu	3965450.8	182005.2611	4.58977479
UIJ66514.1	PTS_glucose_transporter_subunit_IIA_[Bacillus_cereus]	CPTF_Fe	4052697.667	227180.1895	5.605653522
UIJ66514.1	PTS_glucose_transporter_subunit_IIA_[Bacillus_cereus]	CPTF_Mn	3971310	345419.1459	8.697864078
UIJ66514.1	PTS_glucose_transporter_subunit_IIA_[Bacillus_cereus]	CPTF_Ni	3859690.333	113441.8058	2.939142676
UIJ66514.1	PTS_glucose_transporter_subunit_IIA_[Bacillus_cereus]	CPTF_U	4430563.333	350630.9218	7.913912868
UIJ66514.1	PTS_glucose_transporter_subunit_IIA_[Bacillus_cereus]	CPTF_metals_mix	3766073	31620.74996	0.83962127
UIJ66514.1	PTS_glucose_transporter_subunit_IIA_[Bacillus_cereus]	CPTF_zcontrol	4097005	141299.1081	3.448839044
UIJ66515.1	low_molecular_weight_protein_arginine_phosphatase_[Bacillus_cereus]	CPTF_Al	0	0	0
UIJ66515.1	low_molecular_weight_protein_arginine_phosphatase_[Bacillus_cereus]	CPTF_Cd	0	0	0
UIJ66515.1	low_molecular_weight_protein_arginine_phosphatase_[Bacillus_cereus]	CPTF_Co	0	0	0
UIJ66515.1	low_molecular_weight_protein_arginine_phosphatase_[Bacillus_cereus]	CPTF_Cu	0	0	0
UIJ66515.1	low_molecular_weight_protein_arginine_phosphatase_[Bacillus_cereus]	CPTF_Fe	0	0	0
UIJ66515.1	low_molecular_weight_protein_arginine_phosphatase_[Bacillus_cereus]	CPTF_Mn	0	0	0
UIJ66515.1	low_molecular_weight_protein_arginine_phosphatase_[Bacillus_cereus]	CPTF_Ni	0	0	0
UIJ66515.1	low_molecular_weight_protein_arginine_phosphatase_[Bacillus_cereus]	CPTF_U	0	0	0
UIJ66515.1	low_molecular_weight_protein_arginine_phosphatase_[Bacillus_cereus]	CPTF_metals_mix	36925.3	12206.10349	33.0562067
UIJ66515.1	low_molecular_weight_protein_arginine_phosphatase_[Bacillus_cereus]	CPTF_zcontrol	0	0	0
UIJ66516.1	ribose_5-phosphate_isomerase_B_[Bacillus_cereus]	CPTF_Al	21842.06667	19099.10938	87.44186012
UIJ66516.1	ribose_5-phosphate_isomerase_B_[Bacillus_cereus]	CPTF_Cd	30797.2	30307.3424	98.40940863
UIJ66516.1	ribose_5-phosphate_isomerase_B_[Bacillus_cereus]	CPTF_Co	52546.56667	27280.83595	51.91744709
UIJ66516.1	ribose_5-phosphate_isomerase_B_[Bacillus_cereus]	CPTF_Cu	36957.63333	37030.81566	100.1980168
UIJ66516.1	ribose_5-phosphate_isomerase_B_[Bacillus_cereus]	CPTF_Fe	23501	40704.92603	173.2050808
UIJ66516.1	ribose_5-phosphate_isomerase_B_[Bacillus_cereus]	CPTF_Mn	0	0	0
UIJ66516.1	ribose_5-phosphate_isomerase_B_[Bacillus_cereus]	CPTF_Ni	0	0	0
UIJ66516.1	ribose_5-phosphate_isomerase_B_[Bacillus_cereus]	CPTF_U	11353.36667	19664.6079	173.2050808
UIJ66516.1	ribose_5-phosphate_isomerase_B_[Bacillus_cereus]	CPTF_metals_mix	496394.3	459660.825	92.5999402
UIJ66516.1	ribose_5-phosphate_isomerase_B_[Bacillus_cereus]	CPTF_zcontrol	0	0	0
UIJ66517.1	TIGR01440_family_protein_[Bacillus_cereus]	CPTF_Al	929218.9333	35718.07931	3.843881999
UIJ66517.1	TIGR01440_family_protein_[Bacillus_cereus]	CPTF_Cd	1013648.867	93562.67876	9.230284948
UIJ66517.1	TIGR01440_family_protein_[Bacillus_cereus]	CPTF_Co	896590.2	25392.00828	2.832064
UIJ66517.1	TIGR01440_family_protein_[Bacillus_cereus]	CPTF_Cu	984724.1667	195694.8467	19.87306225
UIJ66517.1	TIGR01440_family_protein_[Bacillus_cereus]	CPTF_Fe	747820.8333	658342.2334	88.03475433
UIJ66517.1	TIGR01440_family_protein_[Bacillus_cereus]	CPTF_Mn	1033184.233	103860.1594	10.05243363
UIJ66517.1	TIGR01440_family_protein_[Bacillus_cereus]	CPTF_Ni	1204057.667	242983.4438	20.1803826
UIJ66517.1	TIGR01440_family_protein_[Bacillus_cereus]	CPTF_U	1003465.667	126961.1143	12.6522629
UIJ66517.1	TIGR01440_family_protein_[Bacillus_cereus]	CPTF_metals_mix	756402.8	326821.3093	43.20731088
UIJ66517.1	TIGR01440_family_protein_[Bacillus_cereus]	CPTF_zcontrol	1199323.833	164069.5176	13.68016819
UIJ66518.1	serine_hydroxymethyltransferase_[Bacillus_cereus]	CPTF_Al	13711236.77	742581.5153	5.415860932
UIJ66518.1	serine_hydroxymethyltransferase_[Bacillus_cereus]	CPTF_Cd	14838611	415292.5538	2.7987293
UIJ66518.1	serine_hydroxymethyltransferase_[Bacillus_cereus]	CPTF_Co	13222142.5	1052157.828	7.957544155
UIJ66518.1	serine_hydroxymethyltransferase_[Bacillus_cereus]	CPTF_Cu	10702818.7	396208.0721	3.701903986
UIJ66518.1	serine_hydroxymethyltransferase_[Bacillus_cereus]	CPTF_Fe	13906093.53	576988.2722	4.149175833
UIJ66518.1	serine_hydroxymethyltransferase_[Bacillus_cereus]	CPTF_Mn	14817369.9	1728590.19	11.66597178
UIJ66518.1	serine_hydroxymethyltransferase_[Bacillus_cereus]	CPTF_Ni	12671688.53	682782.2935	5.388250285
UIJ66518.1	serine_hydroxymethyltransferase_[Bacillus_cereus]	CPTF_U	11443364.27	454587.1423	3.972495603
UIJ66518.1	serine_hydroxymethyltransferase_[Bacillus_cereus]	CPTF_metals_mix	13279319.03	1375429.414	10.35768032
UIJ66518.1	serine_hydroxymethyltransferase_[Bacillus_cereus]	CPTF_zcontrol	14308397.27	746154.8889	5.214804111
UIJ66519.1	uracil_phosphoribosyltransferase_[Bacillus_cereus]	CPTF_Al	3355074.833	111860.6473	3.334073095
UIJ66519.1	uracil_phosphoribosyltransferase_[Bacillus_cereus]	CPTF_Cd	4097131.333	265875.5319	6.489309477
UIJ66519.1	uracil_phosphoribosyltransferase_[Bacillus_cereus]	CPTF_Co	3250349.767	309145.8319	9.511155848
UIJ66519.1	uracil_phosphoribosyltransferase_[Bacillus_cereus]	CPTF_Cu	2978286.233	141300.0362	4.744340375

UIJ66519.1	uracil_phosphoribosyltransferase_[Bacillus_cereus]	CPTF_Fe	3438833.133	304254.5316	8.847609635
UIJ66519.1	uracil_phosphoribosyltransferase_[Bacillus_cereus]	CPTF_Mn	3589677.833	326239.0726	9.088254929
UIJ66519.1	uracil_phosphoribosyltransferase_[Bacillus_cereus]	CPTF_Ni	2642387.967	125059.1375	4.732807562
UIJ66519.1	uracil_phosphoribosyltransferase_[Bacillus_cereus]	CPTF_U	3260316.633	182911.6	5.610240372
UIJ66519.1	uracil_phosphoribosyltransferase_[Bacillus_cereus]	CPTF_metals_mix	3603300.267	350423.0811	9.725059117
UIJ66519.1	uracil_phosphoribosyltransferase_[Bacillus_cereus]	CPTF_zcontrol	3856455	302162.7467	7.835246275
UIJ66522.1	F0F1_ATP_synthase_subunit_A_[Bacillus_cereus]	CPTF_Al	647593.6667	153467.4167	23.6981034
UIJ66522.1	F0F1_ATP_synthase_subunit_A_[Bacillus_cereus]	CPTF_Cd	533924	47563.65744	8.908319806
UIJ66522.1	F0F1_ATP_synthase_subunit_A_[Bacillus_cereus]	CPTF_Co	660126	41652.602	6.309795705
UIJ66522.1	F0F1_ATP_synthase_subunit_A_[Bacillus_cereus]	CPTF_Cu	592511	72284.5889	12.19970412
UIJ66522.1	F0F1_ATP_synthase_subunit_A_[Bacillus_cereus]	CPTF_Fe	579243.6667	91692.73231	15.82973411
UIJ66522.1	F0F1_ATP_synthase_subunit_A_[Bacillus_cereus]	CPTF_Mn	680765.3333	127371.4525	18.71003799
UIJ66522.1	F0F1_ATP_synthase_subunit_A_[Bacillus_cereus]	CPTF_Ni	639450.6667	71901.92344	11.24432692
UIJ66522.1	F0F1_ATP_synthase_subunit_A_[Bacillus_cereus]	CPTF_U	666817.6667	368366.1399	55.24240858
UIJ66522.1	F0F1_ATP_synthase_subunit_A_[Bacillus_cereus]	CPTF_metals_mix	343510.8	298948.4757	87.02738769
UIJ66522.1	F0F1_ATP_synthase_subunit_A_[Bacillus_cereus]	CPTF_zcontrol	804633	83238.56722	10.34491094
UIJ66524.1	F0F1_ATP_synthase_subunit_B_[Bacillus_cereus]	CPTF_Al	3935961	221872.4835	5.637060009
UIJ66524.1	F0F1_ATP_synthase_subunit_B_[Bacillus_cereus]	CPTF_Cd	4415102.467	100884.1643	2.284979002
UIJ66524.1	F0F1_ATP_synthase_subunit_B_[Bacillus_cereus]	CPTF_Co	4031829.333	259154.631	6.427718277
UIJ66524.1	F0F1_ATP_synthase_subunit_B_[Bacillus_cereus]	CPTF_Cu	3909994.7	97311.61558	2.488791496
UIJ66524.1	F0F1_ATP_synthase_subunit_B_[Bacillus_cereus]	CPTF_Fe	3840389.2	500553.4376	13.03392473
UIJ66524.1	F0F1_ATP_synthase_subunit_B_[Bacillus_cereus]	CPTF_Mn	3966834	473137.5333	11.92733382
UIJ66524.1	F0F1_ATP_synthase_subunit_B_[Bacillus_cereus]	CPTF_Ni	3101444	684898.5114	22.08321386
UIJ66524.1	F0F1_ATP_synthase_subunit_B_[Bacillus_cereus]	CPTF_U	3748913.133	1096003.308	29.23522816
UIJ66524.1	F0F1_ATP_synthase_subunit_B_[Bacillus_cereus]	CPTF_metals_mix	5802280.867	359117.3402	6.189244342
UIJ66524.1	F0F1_ATP_synthase_subunit_B_[Bacillus_cereus]	CPTF_zcontrol	3850904.233	113645.8582	2.951147349
UIJ66525.1	F0F1_ATP_synthase_subunit_delta_[Bacillus_cereus]	CPTF_Al	4086951.567	143945.1593	3.522066679
UIJ66525.1	F0F1_ATP_synthase_subunit_delta_[Bacillus_cereus]	CPTF_Cd	4055549.667	177901.9179	4.386629002
UIJ66525.1	F0F1_ATP_synthase_subunit_delta_[Bacillus_cereus]	CPTF_Co	3935590.667	217472.1097	5.525780705
UIJ66525.1	F0F1_ATP_synthase_subunit_delta_[Bacillus_cereus]	CPTF_Cu	3563166.667	171436.5353	4.811353253
UIJ66525.1	F0F1_ATP_synthase_subunit_delta_[Bacillus_cereus]	CPTF_Fe	4178747.233	134016.5038	3.207097638
UIJ66525.1	F0F1_ATP_synthase_subunit_delta_[Bacillus_cereus]	CPTF_Mn	3918551.1	24164.99512	0.616681894
UIJ66525.1	F0F1_ATP_synthase_subunit_delta_[Bacillus_cereus]	CPTF_Ni	3911276.4	91956.8561	2.351070257
UIJ66525.1	F0F1_ATP_synthase_subunit_delta_[Bacillus_cereus]	CPTF_U	4038225.9	131743.1705	3.262402199
UIJ66525.1	F0F1_ATP_synthase_subunit_delta_[Bacillus_cereus]	CPTF_metals_mix	3315810.367	43896.86202	1.323865275
UIJ66525.1	F0F1_ATP_synthase_subunit_delta_[Bacillus_cereus]	CPTF_zcontrol	4014634.967	154981.5565	3.860414653
UIJ66526.1	F0F1_ATP_synthase_subunit_alpha_[Bacillus_cereus]	CPTF_Al	30496324.2	1319113.66	4.32548412
UIJ66526.1	F0F1_ATP_synthase_subunit_alpha_[Bacillus_cereus]	CPTF_Cd	29954432.43	719339.0684	2.401444494
UIJ66526.1	F0F1_ATP_synthase_subunit_alpha_[Bacillus_cereus]	CPTF_Co	30556778.73	1106215.228	3.620195826
UIJ66526.1	F0F1_ATP_synthase_subunit_alpha_[Bacillus_cereus]	CPTF_Cu	27274964.53	2485774.309	9.11375817
UIJ66526.1	F0F1_ATP_synthase_subunit_alpha_[Bacillus_cereus]	CPTF_Fe	29273195.2	1531200.348	5.230725029
UIJ66526.1	F0F1_ATP_synthase_subunit_alpha_[Bacillus_cereus]	CPTF_Mn	30921002.7	3834684.302	12.40155224
UIJ66526.1	F0F1_ATP_synthase_subunit_alpha_[Bacillus_cereus]	CPTF_Ni	25103674.03	2131799.729	8.491982993
UIJ66526.1	F0F1_ATP_synthase_subunit_alpha_[Bacillus_cereus]	CPTF_U	26207134.14	4779632.873	18.23790746
UIJ66526.1	F0F1_ATP_synthase_subunit_alpha_[Bacillus_cereus]	CPTF_metals_mix	36180778.97	2225263.115	6.15040134
UIJ66526.1	F0F1_ATP_synthase_subunit_alpha_[Bacillus_cereus]	CPTF_zcontrol	29149668.6	2480609.592	8.509906668
UIJ66527.1	F0F1_ATP_synthase_subunit_gamma_[Bacillus_cereus]	CPTF_Al	5037498	669434.6006	13.2890296
UIJ66527.1	F0F1_ATP_synthase_subunit_gamma_[Bacillus_cereus]	CPTF_Cd	5253341.5	1130984.924	21.52886737
UIJ66527.1	F0F1_ATP_synthase_subunit_gamma_[Bacillus_cereus]	CPTF_Co	5248761	802617.1383	15.2915543
UIJ66527.1	F0F1_ATP_synthase_subunit_gamma_[Bacillus_cereus]	CPTF_Cu	4628430	716700.5677	15.48474467
UIJ66527.1	F0F1_ATP_synthase_subunit_gamma_[Bacillus_cereus]	CPTF_Fe	4504683.433	204587.5326	4.541662819
UIJ66527.1	F0F1_ATP_synthase_subunit_gamma_[Bacillus_cereus]	CPTF_Mn	4064304.5	1179409.14	29.01871993
UIJ66527.1	F0F1_ATP_synthase_subunit_gamma_[Bacillus_cereus]	CPTF_Ni	4070315.467	420272.9914	10.32531741
UIJ66527.1	F0F1_ATP_synthase_subunit_gamma_[Bacillus_cereus]	CPTF_U	3316378.333	1261883.339	38.05004171
UIJ66527.1	F0F1_ATP_synthase_subunit_gamma_[Bacillus_cereus]	CPTF_metals_mix	5996659.4	813359.8314	13.56354892
UIJ66527.1	F0F1_ATP_synthase_subunit_gamma_[Bacillus_cereus]	CPTF_zcontrol	4509434.667	860457.204	19.08126556
UIJ66528.1	F0F1_ATP_synthase_subunit_beta_[Bacillus_cereus]	CPTF_Al	43088183.3	3347257.768	7.768389177
UIJ66528.1	F0F1_ATP_synthase_subunit_beta_[Bacillus_cereus]	CPTF_Cd	48687874.97	823463.3215	1.69131087
UIJ66528.1	F0F1_ATP_synthase_subunit_beta_[Bacillus_cereus]	CPTF_Co	40297751.33	2522138.33	6.258756995
UIJ66528.1	F0F1_ATP_synthase_subunit_beta_[Bacillus_cereus]	CPTF_Cu	35738998	525790.8432	1.471196375
UIJ66528.1	F0F1_ATP_synthase_subunit_beta_[Bacillus_cereus]	CPTF_Fe	43855842.67	4590638.497	10.46756422

UIJ66528.1	F0F1_ATP_synthase_subunit_beta_[Bacillus_cereus]	CPTF_Mn	44186959.77	8814630.653	19.94848865
UIJ66528.1	F0F1_ATP_synthase_subunit_beta_[Bacillus_cereus]	CPTF_Ni	30590140.47	2011775.164	6.576547649
UIJ66528.1	F0F1_ATP_synthase_subunit_beta_[Bacillus_cereus]	CPTF_U	36448389.2	3997577.316	10.96777499
UIJ66528.1	F0F1_ATP_synthase_subunit_beta_[Bacillus_cereus]	CPTF_metals_mix	46240284.37	4533619.719	9.80448062
UIJ66528.1	F0F1_ATP_synthase_subunit_beta_[Bacillus_cereus]	CPTF_zcontrol	41320107.8	4271913.917	10.33858367
UIJ66529.1	F0F1_ATP_synthase_subunit_epsilon_[Bacillus_cereus]	CPTF_Al	2575626.833	236463.6811	9.180820687
UIJ66529.1	F0F1_ATP_synthase_subunit_epsilon_[Bacillus_cereus]	CPTF_Cd	2618729.933	82109.5575	3.135472522
UIJ66529.1	F0F1_ATP_synthase_subunit_epsilon_[Bacillus_cereus]	CPTF_Co	2764866.733	160615.2492	5.80914976
UIJ66529.1	F0F1_ATP_synthase_subunit_epsilon_[Bacillus_cereus]	CPTF_Cu	2487595	332904.2524	13.38257443
UIJ66529.1	F0F1_ATP_synthase_subunit_epsilon_[Bacillus_cereus]	CPTF_Fe	2560492.2	219045.6214	8.554824788
UIJ66529.1	F0F1_ATP_synthase_subunit_epsilon_[Bacillus_cereus]	CPTF_Mn	2583237	350102.4427	13.55285801
UIJ66529.1	F0F1_ATP_synthase_subunit_epsilon_[Bacillus_cereus]	CPTF_Ni	2330502.1	292231.6078	12.53942692
UIJ66529.1	F0F1_ATP_synthase_subunit_epsilon_[Bacillus_cereus]	CPTF_U	2015495.033	250335.5967	12.42055141
UIJ66529.1	F0F1_ATP_synthase_subunit_epsilon_[Bacillus_cereus]	CPTF_metals_mix	2419095.633	58998.2203	2.4388544
UIJ66529.1	F0F1_ATP_synthase_subunit_epsilon_[Bacillus_cereus]	CPTF_zcontrol	2378438.867	227037.1088	9.545635668
UIJ66530.1	DUF975_family_protein_[Bacillus_cereus]	CPTF_Al	91012.06667	19950.945	21.92120861
UIJ66530.1	DUF975_family_protein_[Bacillus_cereus]	CPTF_Cd	35039.13333	32379.36594	92.40915189
UIJ66530.1	DUF975_family_protein_[Bacillus_cereus]	CPTF_Co	75680.96667	79000.03847	104.3856097
UIJ66530.1	DUF975_family_protein_[Bacillus_cereus]	CPTF_Cu	0	0	0
UIJ66530.1	DUF975_family_protein_[Bacillus_cereus]	CPTF_Fe	49702.93333	45934.9504	92.41899285
UIJ66530.1	DUF975_family_protein_[Bacillus_cereus]	CPTF_Mn	22072.1	38229.99863	173.2050808
UIJ66530.1	DUF975_family_protein_[Bacillus_cereus]	CPTF_Ni	33736.73333	29367.65831	87.04950187
UIJ66530.1	DUF975_family_protein_[Bacillus_cereus]	CPTF_U	31999.96667	55425.56811	173.2050808
UIJ66530.1	DUF975_family_protein_[Bacillus_cereus]	CPTF_metals_mix	18212.63333	31545.20627	173.2050808
UIJ66530.1	DUF975_family_protein_[Bacillus_cereus]	CPTF_zcontrol	58819.63333	27589.54627	46.90533535
UIJ66546.1	UDP-N-acetylglucosamine_1-carboxyvinyltransferase_[Bacillus_cereus]	CPTF_Al	345623.3333	331357.3152	95.8723799
UIJ66546.1	UDP-N-acetylglucosamine_1-carboxyvinyltransferase_[Bacillus_cereus]	CPTF_Cd	639625.6667	28538.53942	4.461756447
UIJ66546.1	UDP-N-acetylglucosamine_1-carboxyvinyltransferase_[Bacillus_cereus]	CPTF_Co	753549.4667	72691.27184	9.646516262
UIJ66546.1	UDP-N-acetylglucosamine_1-carboxyvinyltransferase_[Bacillus_cereus]	CPTF_Cu	707427.3333	85687.98265	12.11261972
UIJ66546.1	UDP-N-acetylglucosamine_1-carboxyvinyltransferase_[Bacillus_cereus]	CPTF_Fe	366020.3333	319511.3408	87.29333091
UIJ66546.1	UDP-N-acetylglucosamine_1-carboxyvinyltransferase_[Bacillus_cereus]	CPTF_Mn	604204.6667	42625.56301	7.054821878
UIJ66546.1	UDP-N-acetylglucosamine_1-carboxyvinyltransferase_[Bacillus_cereus]	CPTF_Ni	690338	71021.63862	10.2879515
UIJ66546.1	UDP-N-acetylglucosamine_1-carboxyvinyltransferase_[Bacillus_cereus]	CPTF_U	763260	122779.8912	16.0862473
UIJ66546.1	UDP-N-acetylglucosamine_1-carboxyvinyltransferase_[Bacillus_cereus]	CPTF_metals_mix	634877.4	90934.9003	14.32322214
UIJ66546.1	UDP-N-acetylglucosamine_1-carboxyvinyltransferase_[Bacillus_cereus]	CPTF_zcontrol	707592	37925.63571	5.359816916
UIJ66554.1	rod_shape-determining_protein_[Bacillus_cereus]	CPTF_Al	1784117.133	763898.1689	42.81659285
UIJ66554.1	rod_shape-determining_protein_[Bacillus_cereus]	CPTF_Cd	1218414.333	442023.2332	36.27856478
UIJ66554.1	rod_shape-determining_protein_[Bacillus_cereus]	CPTF_Co	1935071.967	1248458.81	64.5174356
UIJ66554.1	rod_shape-determining_protein_[Bacillus_cereus]	CPTF_Cu	2159044.067	588337.8866	27.24992489
UIJ66554.1	rod_shape-determining_protein_[Bacillus_cereus]	CPTF_Fe	1939866.333	1149452.206	59.25419632
UIJ66554.1	rod_shape-determining_protein_[Bacillus_cereus]	CPTF_Mn	1330104.6	352330.8955	26.48896151
UIJ66554.1	rod_shape-determining_protein_[Bacillus_cereus]	CPTF_Ni	1202086.467	241408.8444	20.08248584
UIJ66554.1	rod_shape-determining_protein_[Bacillus_cereus]	CPTF_U	1679119.867	816333.0999	48.61672571
UIJ66554.1	rod_shape-determining_protein_[Bacillus_cereus]	CPTF_metals_mix	3453758.9	158843.7601	4.599156013
UIJ66554.1	rod_shape-determining_protein_[Bacillus_cereus]	CPTF_zcontrol	1169599.7	307980.2244	26.33210529
UIJ66555.1	3-hydroxyacyl-ACP_dehydratase_FabZ_[Bacillus_cereus]	CPTF_Al	547907.8667	22326.02493	4.074777218
UIJ66555.1	3-hydroxyacyl-ACP_dehydratase_FabZ_[Bacillus_cereus]	CPTF_Cd	518290.3333	113393.3248	21.87834067
UIJ66555.1	3-hydroxyacyl-ACP_dehydratase_FabZ_[Bacillus_cereus]	CPTF_Co	625565.5667	122705.5506	19.61513823
UIJ66555.1	3-hydroxyacyl-ACP_dehydratase_FabZ_[Bacillus_cereus]	CPTF_Cu	493373	55766.27845	11.30306653
UIJ66555.1	3-hydroxyacyl-ACP_dehydratase_FabZ_[Bacillus_cereus]	CPTF_Fe	463426.2667	81254.7638	17.53348259
UIJ66555.1	3-hydroxyacyl-ACP_dehydratase_FabZ_[Bacillus_cereus]	CPTF_Mn	568175.3333	49411.79828	8.696575754
UIJ66555.1	3-hydroxyacyl-ACP_dehydratase_FabZ_[Bacillus_cereus]	CPTF_Ni	460075.8	157587.8143	34.25257628
UIJ66555.1	3-hydroxyacyl-ACP_dehydratase_FabZ_[Bacillus_cereus]	CPTF_U	335907.3333	127761.3755	38.03470863
UIJ66555.1	3-hydroxyacyl-ACP_dehydratase_FabZ_[Bacillus_cereus]	CPTF_metals_mix	316414.3333	26857.38074	8.488041758
UIJ66555.1	3-hydroxyacyl-ACP_dehydratase_FabZ_[Bacillus_cereus]	CPTF_zcontrol	514535.3333	71388.28614	13.87432145
UIJ66556.1	CpsD/CapB_family_tyrosine-protein_kinase_[Bacillus_cereus]	CPTF_Al	21307.7	36906.01899	173.2050808
UIJ66556.1	CpsD/CapB_family_tyrosine-protein_kinase_[Bacillus_cereus]	CPTF_Cd	50052.03333	43427.63098	86.76496854
UIJ66556.1	CpsD/CapB_family_tyrosine-protein_kinase_[Bacillus_cereus]	CPTF_Co	0	0	0
UIJ66556.1	CpsD/CapB_family_tyrosine-protein_kinase_[Bacillus_cereus]	CPTF_Cu	37123.06667	5402.604696	14.55322844
UIJ66556.1	CpsD/CapB_family_tyrosine-protein_kinase_[Bacillus_cereus]	CPTF_Fe	16924.46667	29314.03616	173.2050808
UIJ66556.1	CpsD/CapB_family_tyrosine-protein_kinase_[Bacillus_cereus]	CPTF_Mn	58431.66667	101206.6154	173.2050808

UIJ66556.1	CpsD/CapB_family_tyrosine-protein_kinase_[Bacillus_cereus]	CPTF_Ni	0	0	0
UIJ66556.1	CpsD/CapB_family_tyrosine-protein_kinase_[Bacillus_cereus]	CPTF_U	0	0	0
UIJ66556.1	CpsD/CapB_family_tyrosine-protein_kinase_[Bacillus_cereus]	CPTF_metals_mix	173953.1667	57372.5212	32.98159056
UIJ66556.1	CpsD/CapB_family_tyrosine-protein_kinase_[Bacillus_cereus]	CPTF_zcontrol	0	0	0
UIJ66559.1	tyrosine_protein_phosphatase_[Bacillus_cereus]	CPTF_Al	9644.1	16704.07119	173.2050808
UIJ66559.1	tyrosine_protein_phosphatase_[Bacillus_cereus]	CPTF_Cd	26962.73333	23375.46255	86.69544833
UIJ66559.1	tyrosine_protein_phosphatase_[Bacillus_cereus]	CPTF_Co	0	0	0
UIJ66559.1	tyrosine_protein_phosphatase_[Bacillus_cereus]	CPTF_Cu	0	0	0
UIJ66559.1	tyrosine_protein_phosphatase_[Bacillus_cereus]	CPTF_Fe	0	0	0
UIJ66559.1	tyrosine_protein_phosphatase_[Bacillus_cereus]	CPTF_Mn	0	0	0
UIJ66559.1	tyrosine_protein_phosphatase_[Bacillus_cereus]	CPTF_Ni	0	0	0
UIJ66559.1	tyrosine_protein_phosphatase_[Bacillus_cereus]	CPTF_U	0	0	0
UIJ66559.1	tyrosine_protein_phosphatase_[Bacillus_cereus]	CPTF_metals_mix	0	0	0
UIJ66559.1	tyrosine_protein_phosphatase_[Bacillus_cereus]	CPTF_zcontrol	0	0	0
UIJ66592.1	efflux_RND_transporter_periplasmic_adaptor_subunit_[Bacillus_cereus]	CPTF_Al	979074.0333	97339.12803	9.941957882
UIJ66592.1	efflux_RND_transporter_periplasmic_adaptor_subunit_[Bacillus_cereus]	CPTF_Cd	742014.2667	225400.6205	30.37685805
UIJ66592.1	efflux_RND_transporter_periplasmic_adaptor_subunit_[Bacillus_cereus]	CPTF_Co	866949.8	174526.0146	20.13104041
UIJ66592.1	efflux_RND_transporter_periplasmic_adaptor_subunit_[Bacillus_cereus]	CPTF_Cu	682930.5	56171.41748	8.225056208
UIJ66592.1	efflux_RND_transporter_periplasmic_adaptor_subunit_[Bacillus_cereus]	CPTF_Fe	716700.8	175359.8572	24.46765194
UIJ66592.1	efflux_RND_transporter_periplasmic_adaptor_subunit_[Bacillus_cereus]	CPTF_Mn	781136.9667	175621.0171	22.48274306
UIJ66592.1	efflux_RND_transporter_periplasmic_adaptor_subunit_[Bacillus_cereus]	CPTF_Ni	860218.7667	210283.1406	24.44530958
UIJ66592.1	efflux_RND_transporter_periplasmic_adaptor_subunit_[Bacillus_cereus]	CPTF_U	581023.6667	148762.1674	25.60346091
UIJ66592.1	efflux_RND_transporter_periplasmic_adaptor_subunit_[Bacillus_cereus]	CPTF_metals_mix	349899.7	35868.89935	10.25119466
UIJ66592.1	efflux_RND_transporter_periplasmic_adaptor_subunit_[Bacillus_cereus]	CPTF_zcontrol	739053.3333	65696.6602	8.889298951
UIJ66594.1	ABC_transporter_permease_[Bacillus_cereus]	CPTF_Al	82208.26667	8249.689463	10.03510936
UIJ66594.1	ABC_transporter_permease_[Bacillus_cereus]	CPTF_Cd	141144.2	69482.89202	49.22830128
UIJ66594.1	ABC_transporter_permease_[Bacillus_cereus]	CPTF_Co	112424.5	19488.00385	17.33430334
UIJ66594.1	ABC_transporter_permease_[Bacillus_cereus]	CPTF_Cu	78361.23333	10518.94039	13.42365344
UIJ66594.1	ABC_transporter_permease_[Bacillus_cereus]	CPTF_Fe	121696.9667	28387.78484	23.32661661
UIJ66594.1	ABC_transporter_permease_[Bacillus_cereus]	CPTF_Mn	73404.66667	63883.79182	87.0296055
UIJ66594.1	ABC_transporter_permease_[Bacillus_cereus]	CPTF_Ni	43867.03333	45588.70892	103.9247596
UIJ66594.1	ABC_transporter_permease_[Bacillus_cereus]	CPTF_U	0	0	0
UIJ66594.1	ABC_transporter_permease_[Bacillus_cereus]	CPTF_metals_mix	12020.13333	20819.48165	173.2050808
UIJ66594.1	ABC_transporter_permease_[Bacillus_cereus]	CPTF_zcontrol	102999.1667	54644.80491	53.05363788
UIJ66595.1	YIP1_family_protein_[Bacillus_cereus]	CPTF_Al	97143.06667	110424.6687	113.6722079
UIJ66595.1	YIP1_family_protein_[Bacillus_cereus]	CPTF_Cd	341097	58095.17272	17.0318627
UIJ66595.1	YIP1_family_protein_[Bacillus_cereus]	CPTF_Co	119110	121072.5377	101.6476683
UIJ66595.1	YIP1_family_protein_[Bacillus_cereus]	CPTF_Cu	30931.53333	35579.32318	115.0260571
UIJ66595.1	YIP1_family_protein_[Bacillus_cereus]	CPTF_Fe	181995.0333	124040.2853	68.15586287
UIJ66595.1	YIP1_family_protein_[Bacillus_cereus]	CPTF_Mn	151976	164564.8728	108.2834611
UIJ66595.1	YIP1_family_protein_[Bacillus_cereus]	CPTF_Ni	0	0	0
UIJ66595.1	YIP1_family_protein_[Bacillus_cereus]	CPTF_U	24417.46667	42292.29286	173.2050808
UIJ66595.1	YIP1_family_protein_[Bacillus_cereus]	CPTF_metals_mix	228889	42758.9054	18.68106611
UIJ66595.1	YIP1_family_protein_[Bacillus_cereus]	CPTF_zcontrol	15880.26667	27505.4287	173.2050808
UIJ66597.1	NCS2_family_permease_[Bacillus_cereus]	CPTF_Al	42948.33333	74388.69543	173.2050808
UIJ66597.1	NCS2_family_permease_[Bacillus_cereus]	CPTF_Cd	21046.36667	36453.37638	173.2050808
UIJ66597.1	NCS2_family_permease_[Bacillus_cereus]	CPTF_Co	81426.6	66906.67727	82.16808422
UIJ66597.1	NCS2_family_permease_[Bacillus_cereus]	CPTF_Cu	63993.46667	68535.68227	107.0979365
UIJ66597.1	NCS2_family_permease_[Bacillus_cereus]	CPTF_Fe	56624.53333	53947.96124	95.27312291
UIJ66597.1	NCS2_family_permease_[Bacillus_cereus]	CPTF_Mn	54036.66667	93594.25214	173.2050808
UIJ66597.1	NCS2_family_permease_[Bacillus_cereus]	CPTF_Ni	113148.2667	73670.91543	65.11006982
UIJ66597.1	NCS2_family_permease_[Bacillus_cereus]	CPTF_U	271337	367552.7158	135.4598583
UIJ66597.1	NCS2_family_permease_[Bacillus_cereus]	CPTF_metals_mix	0	0	0
UIJ66597.1	NCS2_family_permease_[Bacillus_cereus]	CPTF_zcontrol	244513.3333	156438.7198	63.97962749
UIJ66603.1	5-(carboxyamino)imidazole_ribonucleotide_mutase_[Bacillus_cereus]	CPTF_Al	2023974.833	112389.2231	5.552896275
UIJ66603.1	5-(carboxyamino)imidazole_ribonucleotide_mutase_[Bacillus_cereus]	CPTF_Cd	2268700.133	185692.648	8.184979814
UIJ66603.1	5-(carboxyamino)imidazole_ribonucleotide_mutase_[Bacillus_cereus]	CPTF_Co	2357952.5	146416.2295	6.209464757
UIJ66603.1	5-(carboxyamino)imidazole_ribonucleotide_mutase_[Bacillus_cereus]	CPTF_Cu	1758286	65219.68188	3.709276072
UIJ66603.1	5-(carboxyamino)imidazole_ribonucleotide_mutase_[Bacillus_cereus]	CPTF_Fe	2138514.6	58588.36549	2.739675731
UIJ66603.1	5-(carboxyamino)imidazole_ribonucleotide_mutase_[Bacillus_cereus]	CPTF_Mn	1990267.4	234205.5464	11.76754171
UIJ66603.1	5-(carboxyamino)imidazole_ribonucleotide_mutase_[Bacillus_cereus]	CPTF_Ni	1969324.733	247188.7163	12.55195307

UIJ66603.1	5-(carboxyamino)imidazole_ribonucleotide_mutase_[Bacillus_cereus]	CPTF_U	1275579.833	243814.3559	19.11400209
UIJ66603.1	5-(carboxyamino)imidazole_ribonucleotide_mutase_[Bacillus_cereus]	CPTF_metals_mix	1603848.733	47935.2416	2.988763255
UIJ66603.1	5-(carboxyamino)imidazole_ribonucleotide_mutase_[Bacillus_cereus]	CPTF_zcontrol	1852293.833	129350.2633	6.983247527
UIJ66604.1	5-(carboxyamino)imidazole_ribonucleotide_synthase_[Bacillus_cereus]	CPTF_Al	6238703.1	58247.63254	0.933649696
UIJ66604.1	5-(carboxyamino)imidazole_ribonucleotide_synthase_[Bacillus_cereus]	CPTF_Cd	6395932.7	91785.10303	1.435054234
UIJ66604.1	5-(carboxyamino)imidazole_ribonucleotide_synthase_[Bacillus_cereus]	CPTF_Co	6212078.267	170682.4535	2.747590197
UIJ66604.1	5-(carboxyamino)imidazole_ribonucleotide_synthase_[Bacillus_cereus]	CPTF_Cu	5146963.267	565802.6625	10.99294153
UIJ66604.1	5-(carboxyamino)imidazole_ribonucleotide_synthase_[Bacillus_cereus]	CPTF_Fe	6567495.333	434301.6029	6.612895494
UIJ66604.1	5-(carboxyamino)imidazole_ribonucleotide_synthase_[Bacillus_cereus]	CPTF_Mn	6018531.267	582246.0961	9.674222336
UIJ66604.1	5-(carboxyamino)imidazole_ribonucleotide_synthase_[Bacillus_cereus]	CPTF_Ni	4474533.333	943854.2139	21.09391401
UIJ66604.1	5-(carboxyamino)imidazole_ribonucleotide_synthase_[Bacillus_cereus]	CPTF_U	5999368.333	236100.3898	3.93542081
UIJ66604.1	5-(carboxyamino)imidazole_ribonucleotide_synthase_[Bacillus_cereus]	CPTF_metals_mix	3695394.8	29415.27941	0.79599829
UIJ66604.1	5-(carboxyamino)imidazole_ribonucleotide_synthase_[Bacillus_cereus]	CPTF_zcontrol	6515897.433	778368.9754	11.94569103
UIJ66605.1	adenylosuccinate_lyase_[Bacillus_cereus]	CPTF_Al	10683509.9	1374780.197	12.8682447
UIJ66605.1	adenylosuccinate_lyase_[Bacillus_cereus]	CPTF_Cd	12498368.17	1154047.429	9.233584844
UIJ66605.1	adenylosuccinate_lyase_[Bacillus_cereus]	CPTF_Co	11826830.7	736648.4122	6.228620591
UIJ66605.1	adenylosuccinate_lyase_[Bacillus_cereus]	CPTF_Cu	10235667.63	152789.1541	1.492713124
UIJ66605.1	adenylosuccinate_lyase_[Bacillus_cereus]	CPTF_Fe	11539897.9	2046422.082	17.7334505
UIJ66605.1	adenylosuccinate_lyase_[Bacillus_cereus]	CPTF_Mn	11487561.73	2942452.601	25.61424843
UIJ66605.1	adenylosuccinate_lyase_[Bacillus_cereus]	CPTF_Ni	8299076.833	677196.4003	8.159900359
UIJ66605.1	adenylosuccinate_lyase_[Bacillus_cereus]	CPTF_U	8797150.8	1402600.51	15.94380433
UIJ66605.1	adenylosuccinate_lyase_[Bacillus_cereus]	CPTF_metals_mix	11793545.23	887787.7519	7.527742798
UIJ66605.1	adenylosuccinate_lyase_[Bacillus_cereus]	CPTF_zcontrol	10912130.03	1411225.578	12.93263161
UIJ66606.1	phosphoribosylaminoimidazolesuccinocarboxamide_synthase_[Bacillus_cereus]	CPTF_Al	8072643.333	1532860.838	18.98833846
UIJ66606.1	phosphoribosylaminoimidazolesuccinocarboxamide_synthase_[Bacillus_cereus]	CPTF_Cd	8653143.167	251375.6772	2.90502159
UIJ66606.1	phosphoribosylaminoimidazolesuccinocarboxamide_synthase_[Bacillus_cereus]	CPTF_Co	8938962.267	218284.525	2.441944809
UIJ66606.1	phosphoribosylaminoimidazolesuccinocarboxamide_synthase_[Bacillus_cereus]	CPTF_Cu	6878455.7	531346.1938	7.724789066
UIJ66606.1	phosphoribosylaminoimidazolesuccinocarboxamide_synthase_[Bacillus_cereus]	CPTF_Fe	9080792	321762.7317	3.543333353
UIJ66606.1	phosphoribosylaminoimidazolesuccinocarboxamide_synthase_[Bacillus_cereus]	CPTF_Mn	8815755.7	492612.4388	5.587863997
UIJ66606.1	phosphoribosylaminoimidazolesuccinocarboxamide_synthase_[Bacillus_cereus]	CPTF_Ni	8776303.533	669894.4731	7.632991163
UIJ66606.1	phosphoribosylaminoimidazolesuccinocarboxamide_synthase_[Bacillus_cereus]	CPTF_U	9226735.5	277803.4376	3.010852945
UIJ66606.1	phosphoribosylaminoimidazolesuccinocarboxamide_synthase_[Bacillus_cereus]	CPTF_metals_mix	4966380.433	652859.7859	13.1455855
UIJ66606.1	phosphoribosylaminoimidazolesuccinocarboxamide_synthase_[Bacillus_cereus]	CPTF_zcontrol	8936598.067	319215.3278	3.572000502
UIJ66607.1	phosphoribosylformylglycinamidine_synthase_subunit_PurS_[Bacillus_cereus]	CPTF_Al	8182070.433	347614.5005	4.248490688
UIJ66607.1	phosphoribosylformylglycinamidine_synthase_subunit_PurS_[Bacillus_cereus]	CPTF_Cd	8195135.933	65095.2607	0.794315814
UIJ66607.1	phosphoribosylformylglycinamidine_synthase_subunit_PurS_[Bacillus_cereus]	CPTF_Co	9015586.1	668581.744	7.415843369
UIJ66607.1	phosphoribosylformylglycinamidine_synthase_subunit_PurS_[Bacillus_cereus]	CPTF_Cu	6611601.7	164992.536	2.495500236
UIJ66607.1	phosphoribosylformylglycinamidine_synthase_subunit_PurS_[Bacillus_cereus]	CPTF_Fe	8861141.167	583463.4627	6.584518311
UIJ66607.1	phosphoribosylformylglycinamidine_synthase_subunit_PurS_[Bacillus_cereus]	CPTF_Mn	8889422.033	1082721.096	12.1798818
UIJ66607.1	phosphoribosylformylglycinamidine_synthase_subunit_PurS_[Bacillus_cereus]	CPTF_Ni	6878977.667	614045.6785	8.926408956
UIJ66607.1	phosphoribosylformylglycinamidine_synthase_subunit_PurS_[Bacillus_cereus]	CPTF_U	7449712.667	824423.8722	11.06651906
UIJ66607.1	phosphoribosylformylglycinamidine_synthase_subunit_PurS_[Bacillus_cereus]	CPTF_metals_mix	5246867.2	312470.3101	5.955369142
UIJ66607.1	phosphoribosylformylglycinamidine_synthase_subunit_PurS_[Bacillus_cereus]	CPTF_zcontrol	8869553.233	867276.2306	9.778127576
UIJ66608.1	phosphoribosylformylglycinamidine_synthase_subunit_PurQ_[Bacillus_cereus]	CPTF_Al	4926441.333	473091.5502	9.603109389
UIJ66608.1	phosphoribosylformylglycinamidine_synthase_subunit_PurQ_[Bacillus_cereus]	CPTF_Cd	5917269.667	473073.1991	7.994788572
UIJ66608.1	phosphoribosylformylglycinamidine_synthase_subunit_PurQ_[Bacillus_cereus]	CPTF_Co	5101066	375213.4691	7.355589383
UIJ66608.1	phosphoribosylformylglycinamidine_synthase_subunit_PurQ_[Bacillus_cereus]	CPTF_Cu	3738383	133715.7211	3.576833115
UIJ66608.1	phosphoribosylformylglycinamidine_synthase_subunit_PurQ_[Bacillus_cereus]	CPTF_Fe	5373146	566087.4268	10.53549311
UIJ66608.1	phosphoribosylformylglycinamidine_synthase_subunit_PurQ_[Bacillus_cereus]	CPTF_Mn	5707693.333	1501986.441	26.3151216
UIJ66608.1	phosphoribosylformylglycinamidine_synthase_subunit_PurQ_[Bacillus_cereus]	CPTF_Ni	4333472	97287.79239	2.24503106
UIJ66608.1	phosphoribosylformylglycinamidine_synthase_subunit_PurQ_[Bacillus_cereus]	CPTF_U	4690674	378433.0714	8.067776003
UIJ66608.1	phosphoribosylformylglycinamidine_synthase_subunit_PurQ_[Bacillus_cereus]	CPTF_metals_mix	4693550.4	390694.9974	8.324082286
UIJ66608.1	phosphoribosylformylglycinamidine_synthase_subunit_PurQ_[Bacillus_cereus]	CPTF_zcontrol	4976585.667	376530.5359	7.566041482
UIJ66609.1	phosphoribosylformylglycinamidine_synthase_II_[Bacillus_cereus]	CPTF_Al	29380606.2	1104999.885	3.760983953
UIJ66609.1	phosphoribosylformylglycinamidine_synthase_II_[Bacillus_cereus]	CPTF_Cd	30872125.23	1743326.459	5.64692727
UIJ66609.1	phosphoribosylformylglycinamidine_synthase_II_[Bacillus_cereus]	CPTF_Co	31737636.47	659345.0203	2.077486208
UIJ66609.1	phosphoribosylformylglycinamidine_synthase_II_[Bacillus_cereus]	CPTF_Cu	25471744.67	1493726.181	5.864247622
UIJ66609.1	phosphoribosylformylglycinamidine_synthase_II_[Bacillus_cereus]	CPTF_Fe	31925845.47	1435725.084	4.497062061
UIJ66609.1	phosphoribosylformylglycinamidine_synthase_II_[Bacillus_cereus]	CPTF_Mn	30679134.8	543150.331	1.770422584
UIJ66609.1	phosphoribosylformylglycinamidine_synthase_II_[Bacillus_cereus]	CPTF_Ni	26862361.8	233625.7534	0.869714119
UIJ66609.1	phosphoribosylformylglycinamidine_synthase_II_[Bacillus_cereus]	CPTF_U	27172113.33	1268214.93	4.667340059

UIJ66609.1	phosphoribosylformylglycinamidine_synthase_II_[Bacillus_cereus]	CPTF_metals_mix	19640144.3	730235.0679	3.718073843
UIJ66609.1	phosphoribosylformylglycinamidine_synthase_II_[Bacillus_cereus]	CPTF_zcontrol	30778077	2723159.647	8.847725111
UIJ66610.1	amidophosphoribosyltransferase_[Bacillus_cereus]	CPTF_Al	4463054.9	422125.0358	9.458208452
UIJ66610.1	amidophosphoribosyltransferase_[Bacillus_cereus]	CPTF_Cd	4253382.733	217026.3004	5.102439964
UIJ66610.1	amidophosphoribosyltransferase_[Bacillus_cereus]	CPTF_Co	5280785.8	287601.46	5.446186815
UIJ66610.1	amidophosphoribosyltransferase_[Bacillus_cereus]	CPTF_Cu	3680358.1	132734.5718	3.606566756
UIJ66610.1	amidophosphoribosyltransferase_[Bacillus_cereus]	CPTF_Fe	4389273.933	198963.7208	4.532952917
UIJ66610.1	amidophosphoribosyltransferase_[Bacillus_cereus]	CPTF_Mn	4562538.7	513646.717	11.25791474
UIJ66610.1	amidophosphoribosyltransferase_[Bacillus_cereus]	CPTF_Ni	4202355.233	311759.4954	7.418684953
UIJ66610.1	amidophosphoribosyltransferase_[Bacillus_cereus]	CPTF_U	4183794.967	380429.3478	9.092925222
UIJ66610.1	amidophosphoribosyltransferase_[Bacillus_cereus]	CPTF_metals_mix	3969923.433	424271.946	10.6871569
UIJ66610.1	amidophosphoribosyltransferase_[Bacillus_cereus]	CPTF_zcontrol	4446673.367	147405.8507	3.314969159
UIJ66611.1	phosphoribosylformylglycinamidine_cyclo-ligase_[Bacillus_cereus]	CPTF_Al	2877786.933	321591.1061	11.17494497
UIJ66611.1	phosphoribosylformylglycinamidine_cyclo-ligase_[Bacillus_cereus]	CPTF_Cd	3584012.533	234985.6649	6.55649674
UIJ66611.1	phosphoribosylformylglycinamidine_cyclo-ligase_[Bacillus_cereus]	CPTF_Co	3174173.5	79184.26335	2.494641939
UIJ66611.1	phosphoribosylformylglycinamidine_cyclo-ligase_[Bacillus_cereus]	CPTF_Cu	2392172.167	506157.2447	21.158897
UIJ66611.1	phosphoribosylformylglycinamidine_cyclo-ligase_[Bacillus_cereus]	CPTF_Fe	3973204.5	453992.3523	11.42635252
UIJ66611.1	phosphoribosylformylglycinamidine_cyclo-ligase_[Bacillus_cereus]	CPTF_Mn	3610856.133	1693954.74	46.91282836
UIJ66611.1	phosphoribosylformylglycinamidine_cyclo-ligase_[Bacillus_cereus]	CPTF_Ni	2614013.167	178583.2269	6.83176463
UIJ66611.1	phosphoribosylformylglycinamidine_cyclo-ligase_[Bacillus_cereus]	CPTF_U	3122666.5	226651.3953	7.25826454
UIJ66611.1	phosphoribosylformylglycinamidine_cyclo-ligase_[Bacillus_cereus]	CPTF_metals_mix	4732055.7	1153405.639	24.37430394
UIJ66611.1	phosphoribosylformylglycinamidine_cyclo-ligase_[Bacillus_cereus]	CPTF_zcontrol	3079186.433	558766.7766	18.14657179
UIJ66612.1	phosphoribosylglycinamide_formyltransferase_[Bacillus_cereus]	CPTF_Al	52945.33333	91704.00736	173.2050808
UIJ66612.1	phosphoribosylglycinamide_formyltransferase_[Bacillus_cereus]	CPTF_Cd	0	0	0
UIJ66612.1	phosphoribosylglycinamide_formyltransferase_[Bacillus_cereus]	CPTF_Co	166330.3333	288092.5882	173.2050808
UIJ66612.1	phosphoribosylglycinamide_formyltransferase_[Bacillus_cereus]	CPTF_Cu	0	0	0
UIJ66612.1	phosphoribosylglycinamide_formyltransferase_[Bacillus_cereus]	CPTF_Fe	117433.3333	203400.4998	173.2050808
UIJ66612.1	phosphoribosylglycinamide_formyltransferase_[Bacillus_cereus]	CPTF_Mn	0	0	0
UIJ66612.1	phosphoribosylglycinamide_formyltransferase_[Bacillus_cereus]	CPTF_Ni	0	0	0
UIJ66612.1	phosphoribosylglycinamide_formyltransferase_[Bacillus_cereus]	CPTF_U	0	0	0
UIJ66612.1	phosphoribosylglycinamide_formyltransferase_[Bacillus_cereus]	CPTF_metals_mix	151021.8333	236049.1959	156.3013709
UIJ66612.1	phosphoribosylglycinamide_formyltransferase_[Bacillus_cereus]	CPTF_zcontrol	0	0	0
UIJ66613.1	bifunctional_phosphoribosylaminoimidazolecarboxamide_formyltransferase/IMP_cyclohydrolase_[Bacillus_cereus]	CPTF_Al	17545487.77	382940.9436	2.182560831
UIJ66613.1	bifunctional_phosphoribosylaminoimidazolecarboxamide_formyltransferase/IMP_cyclohydrolase_[Bacillus_cereus]	CPTF_Cd	18515274.4	1660491.768	8.968226625
UIJ66613.1	bifunctional_phosphoribosylaminoimidazolecarboxamide_formyltransferase/IMP_cyclohydrolase_[Bacillus_cereus]	CPTF_Co	17937258.1	1275853.675	7.112869021
UIJ66613.1	bifunctional_phosphoribosylaminoimidazolecarboxamide_formyltransferase/IMP_cyclohydrolase_[Bacillus_cereus]	CPTF_Cu	14166533.27	1371891.944	9.684034321
UIJ66613.1	bifunctional_phosphoribosylaminoimidazolecarboxamide_formyltransferase/IMP_cyclohydrolase_[Bacillus_cereus]	CPTF_Fe	19385506.8	1556872.446	8.031115525
UIJ66613.1	bifunctional_phosphoribosylaminoimidazolecarboxamide_formyltransferase/IMP_cyclohydrolase_[Bacillus_cereus]	CPTF_Mn	17807386.67	1826037.256	10.25438089
UIJ66613.1	bifunctional_phosphoribosylaminoimidazolecarboxamide_formyltransferase/IMP_cyclohydrolase_[Bacillus_cereus]	CPTF_Ni	15883657.27	1340899.648	8.442008194
UIJ66613.1	bifunctional_phosphoribosylaminoimidazolecarboxamide_formyltransferase/IMP_cyclohydrolase_[Bacillus_cereus]	CPTF_U	15636471.53	1628044.883	10.4118431
UIJ66613.1	bifunctional_phosphoribosylaminoimidazolecarboxamide_formyltransferase/IMP_cyclohydrolase_[Bacillus_cereus]	CPTF_metals_mix	10767119.97	792748.1522	7.362675949
UIJ66613.1	bifunctional_phosphoribosylaminoimidazolecarboxamide_formyltransferase/IMP_cyclohydrolase_[Bacillus_cereus]	CPTF_zcontrol	17420831.8	1472597.186	8.453081936
UIJ66614.1	phosphoribosylamine--glycine_ligase_[Bacillus_cereus]	CPTF_Al	4981294.233	420798.3845	8.447571349
UIJ66614.1	phosphoribosylamine--glycine_ligase_[Bacillus_cereus]	CPTF_Cd	5041546.1	252146.936	5.001381144
UIJ66614.1	phosphoribosylamine--glycine_ligase_[Bacillus_cereus]	CPTF_Co	4952097.767	583794.693	11.78883618
UIJ66614.1	phosphoribosylamine--glycine_ligase_[Bacillus_cereus]	CPTF_Cu	3774204.6	345952.7513	9.166242639
UIJ66614.1	phosphoribosylamine--glycine_ligase_[Bacillus_cereus]	CPTF_Fe	4716198.2	827409.6238	17.543996
UIJ66614.1	phosphoribosylamine--glycine_ligase_[Bacillus_cereus]	CPTF_Mn	5216415.633	1966591.041	37.700045
UIJ66614.1	phosphoribosylamine--glycine_ligase_[Bacillus_cereus]	CPTF_Ni	4003239.933	116846.9279	2.91880901
UIJ66614.1	phosphoribosylamine--glycine_ligase_[Bacillus_cereus]	CPTF_U	4014199.8	688518.0264	17.1520617
UIJ66614.1	phosphoribosylamine--glycine_ligase_[Bacillus_cereus]	CPTF_metals_mix	6166164.833	715037.7321	11.59615014
UIJ66614.1	phosphoribosylamine--glycine_ligase_[Bacillus_cereus]	CPTF_zcontrol	5565157.567	605570.5848	10.88146342
UIJ66616.1	SPFH_domain-containing_protein_[Bacillus_cereus]	CPTF_Al	250739.8667	121677.9591	48.52756792
UIJ66616.1	SPFH_domain-containing_protein_[Bacillus_cereus]	CPTF_Cd	306403.7667	89790.34282	29.30458192
UIJ66616.1	SPFH_domain-containing_protein_[Bacillus_cereus]	CPTF_Co	297914.5667	42658.90828	14.31917504
UIJ66616.1	SPFH_domain-containing_protein_[Bacillus_cereus]	CPTF_Cu	346535.1333	107829.7201	31.11653327
UIJ66616.1	SPFH_domain-containing_protein_[Bacillus_cereus]	CPTF_Fe	208234.7	6583.667201	3.161657111
UIJ66616.1	SPFH_domain-containing_protein_[Bacillus_cereus]	CPTF_Mn	217366.6667	44764.77759	20.59413169
UIJ66616.1	SPFH_domain-containing_protein_[Bacillus_cereus]	CPTF_Ni	109566.6333	151858.715	138.5994169
UIJ66616.1	SPFH_domain-containing_protein_[Bacillus_cereus]	CPTF_U	247626.3	172863.3608	69.80815881
UIJ66616.1	SPFH_domain-containing_protein_[Bacillus_cereus]	CPTF_metals_mix	485760.7333	104184.9913	21.44780016

UIJ66616.1	SPFH_domain-containing_protein_[Bacillus_cereus]	CPTF_zcontrol	182235.6667	59706.70483	32.7634573
UIJ66619.1	DNA_helicase_PcrA_[Bacillus_cereus]	CPTF_Al	359160.6667	288367.1764	80.28918618
UIJ66619.1	DNA_helicase_PcrA_[Bacillus_cereus]	CPTF_Cd	419278.3333	355019.509	84.67394586
UIJ66619.1	DNA_helicase_PcrA_[Bacillus_cereus]	CPTF_Co	491011.3333	452689.2769	92.19528068
UIJ66619.1	DNA_helicase_PcrA_[Bacillus_cereus]	CPTF_Cu	522624.6667	77841.2427	14.89429177
UIJ66619.1	DNA_helicase_PcrA_[Bacillus_cereus]	CPTF_Fe	323426.6667	124062.0848	38.35864435
UIJ66619.1	DNA_helicase_PcrA_[Bacillus_cereus]	CPTF_Mn	254274.1333	177171.6087	69.67740148
UIJ66619.1	DNA_helicase_PcrA_[Bacillus_cereus]	CPTF_Ni	397764.3333	160604.3165	40.37675152
UIJ66619.1	DNA_helicase_PcrA_[Bacillus_cereus]	CPTF_U	526503.6667	189719.8941	36.03391698
UIJ66619.1	DNA_helicase_PcrA_[Bacillus_cereus]	CPTF_metals_mix	491399.4333	73820.97052	15.02259985
UIJ66619.1	DNA_helicase_PcrA_[Bacillus_cereus]	CPTF_zcontrol	434277.3333	171645.3143	39.52435487
UIJ66620.1	NAD-dependent_DNA_ligase_LigA_[Bacillus_cereus]	CPTF_Al	495104.6667	213768.4332	43.1764125
UIJ66620.1	NAD-dependent_DNA_ligase_LigA_[Bacillus_cereus]	CPTF_Cd	474066.1333	265319.9625	55.96686703
UIJ66620.1	NAD-dependent_DNA_ligase_LigA_[Bacillus_cereus]	CPTF_Co	684847.5333	36981.26704	5.399927026
UIJ66620.1	NAD-dependent_DNA_ligase_LigA_[Bacillus_cereus]	CPTF_Cu	578959.9333	344692.6508	59.53652938
UIJ66620.1	NAD-dependent_DNA_ligase_LigA_[Bacillus_cereus]	CPTF_Fe	403715.2	180036.795	44.59500039
UIJ66620.1	NAD-dependent_DNA_ligase_LigA_[Bacillus_cereus]	CPTF_Mn	245818.9	267259.3064	108.7220333
UIJ66620.1	NAD-dependent_DNA_ligase_LigA_[Bacillus_cereus]	CPTF_Ni	433256.3667	236479.7969	54.58195543
UIJ66620.1	NAD-dependent_DNA_ligase_LigA_[Bacillus_cereus]	CPTF_U	368486.1333	36605.51991	9.934029153
UIJ66620.1	NAD-dependent_DNA_ligase_LigA_[Bacillus_cereus]	CPTF_metals_mix	827099.9	93075.24297	11.25320448
UIJ66620.1	NAD-dependent_DNA_ligase_LigA_[Bacillus_cereus]	CPTF_zcontrol	359002.8333	84647.50239	23.57850538
UIJ66623.1	L-glutamate_gamma-semialdehyde_dehydrogenase_[Bacillus_cereus]	CPTF_Al	32805186.6	2265855.158	6.907002803
UIJ66623.1	L-glutamate_gamma-semialdehyde_dehydrogenase_[Bacillus_cereus]	CPTF_Cd	36144526.67	1462614.944	4.046573795
UIJ66623.1	L-glutamate_gamma-semialdehyde_dehydrogenase_[Bacillus_cereus]	CPTF_Co	29216445.47	2783545.135	9.527323022
UIJ66623.1	L-glutamate_gamma-semialdehyde_dehydrogenase_[Bacillus_cereus]	CPTF_Cu	27682571.27	1459144.593	5.270986497
UIJ66623.1	L-glutamate_gamma-semialdehyde_dehydrogenase_[Bacillus_cereus]	CPTF_Fe	32748846.47	3974924.556	12.13760173
UIJ66623.1	L-glutamate_gamma-semialdehyde_dehydrogenase_[Bacillus_cereus]	CPTF_Mn	33643219.03	4273422.297	12.70218017
UIJ66623.1	L-glutamate_gamma-semialdehyde_dehydrogenase_[Bacillus_cereus]	CPTF_Ni	25312411.4	1473103.718	5.819689381
UIJ66623.1	L-glutamate_gamma-semialdehyde_dehydrogenase_[Bacillus_cereus]	CPTF_U	26355503.47	2506437.978	9.510112306
UIJ66623.1	L-glutamate_gamma-semialdehyde_dehydrogenase_[Bacillus_cereus]	CPTF_metals_mix	29031103.7	2603220.158	8.967003753
UIJ66623.1	L-glutamate_gamma-semialdehyde_dehydrogenase_[Bacillus_cereus]	CPTF_zcontrol	30325924.43	2377206.173	7.838858063
UIJ66627.1	MetQ/NlpA_family_ABC_transporter_substrate-binding_protein_[Bacillus_cereus]	CPTF_Al	272287.3333	60451.24701	22.20127035
UIJ66627.1	MetQ/NlpA_family_ABC_transporter_substrate-binding_protein_[Bacillus_cereus]	CPTF_Cd	310991.3333	88528.0646	28.4664089
UIJ66627.1	MetQ/NlpA_family_ABC_transporter_substrate-binding_protein_[Bacillus_cereus]	CPTF_Co	270816.1667	32352.84464	11.94642293
UIJ66627.1	MetQ/NlpA_family_ABC_transporter_substrate-binding_protein_[Bacillus_cereus]	CPTF_Cu	322638.6667	116975.6019	36.25591537
UIJ66627.1	MetQ/NlpA_family_ABC_transporter_substrate-binding_protein_[Bacillus_cereus]	CPTF_Fe	306252.4667	69622.62184	22.73373423
UIJ66627.1	MetQ/NlpA_family_ABC_transporter_substrate-binding_protein_[Bacillus_cereus]	CPTF_Mn	242399	77363.22104	31.91565189
UIJ66627.1	MetQ/NlpA_family_ABC_transporter_substrate-binding_protein_[Bacillus_cereus]	CPTF_Ni	326230	9828.889205	3.012871043
UIJ66627.1	MetQ/NlpA_family_ABC_transporter_substrate-binding_protein_[Bacillus_cereus]	CPTF_U	317828.3333	68934.30927	21.68916426
UIJ66627.1	MetQ/NlpA_family_ABC_transporter_substrate-binding_protein_[Bacillus_cereus]	CPTF_metals_mix	183580.6667	27077.75612	14.74978635
UIJ66627.1	MetQ/NlpA_family_ABC_transporter_substrate-binding_protein_[Bacillus_cereus]	CPTF_zcontrol	331537	49140.28939	14.82196237
UIJ66629.1	Asp-tRNA(Asn)/Glu-tRNA(Gln)_amidotransferase_subunit_GatC_[Bacillus_cereus]	CPTF_Al	575524.3333	500916.3057	87.03651205
UIJ66629.1	Asp-tRNA(Asn)/Glu-tRNA(Gln)_amidotransferase_subunit_GatC_[Bacillus_cereus]	CPTF_Cd	738755.6667	55184.12495	7.469875013
UIJ66629.1	Asp-tRNA(Asn)/Glu-tRNA(Gln)_amidotransferase_subunit_GatC_[Bacillus_cereus]	CPTF_Co	874414	120339.3858	13.76228947
UIJ66629.1	Asp-tRNA(Asn)/Glu-tRNA(Gln)_amidotransferase_subunit_GatC_[Bacillus_cereus]	CPTF_Cu	839855.3333	49996.76798	5.953021431
UIJ66629.1	Asp-tRNA(Asn)/Glu-tRNA(Gln)_amidotransferase_subunit_GatC_[Bacillus_cereus]	CPTF_Fe	261793.3333	453439.3544	173.2050808
UIJ66629.1	Asp-tRNA(Asn)/Glu-tRNA(Gln)_amidotransferase_subunit_GatC_[Bacillus_cereus]	CPTF_Mn	876790	140400.1373	16.01297201
UIJ66629.1	Asp-tRNA(Asn)/Glu-tRNA(Gln)_amidotransferase_subunit_GatC_[Bacillus_cereus]	CPTF_Ni	292159.6667	506035.3866	173.2050808
UIJ66629.1	Asp-tRNA(Asn)/Glu-tRNA(Gln)_amidotransferase_subunit_GatC_[Bacillus_cereus]	CPTF_U	613325	532484.0267	86.81922745
UIJ66629.1	Asp-tRNA(Asn)/Glu-tRNA(Gln)_amidotransferase_subunit_GatC_[Bacillus_cereus]	CPTF_metals_mix	486051.6667	8812.271973	1.813031942
UIJ66629.1	Asp-tRNA(Asn)/Glu-tRNA(Gln)_amidotransferase_subunit_GatC_[Bacillus_cereus]	CPTF_zcontrol	876645.6667	115847.8933	13.21490514
UIJ66630.1	Asp-tRNA(Asn)/Glu-tRNA(Gln)_amidotransferase_subunit_GatA_[Bacillus_cereus]	CPTF_Al	3139410.833	253591.5835	8.077680717
UIJ66630.1	Asp-tRNA(Asn)/Glu-tRNA(Gln)_amidotransferase_subunit_GatA_[Bacillus_cereus]	CPTF_Cd	3322562.4	49015.73885	1.475239076
UIJ66630.1	Asp-tRNA(Asn)/Glu-tRNA(Gln)_amidotransferase_subunit_GatA_[Bacillus_cereus]	CPTF_Co	3296014.233	288895.0074	8.764980578
UIJ66630.1	Asp-tRNA(Asn)/Glu-tRNA(Gln)_amidotransferase_subunit_GatA_[Bacillus_cereus]	CPTF_Cu	2977320.3	190122.3871	6.385688065
UIJ66630.1	Asp-tRNA(Asn)/Glu-tRNA(Gln)_amidotransferase_subunit_GatA_[Bacillus_cereus]	CPTF_Fe	2957055.1	100594.2166	3.401837748
UIJ66630.1	Asp-tRNA(Asn)/Glu-tRNA(Gln)_amidotransferase_subunit_GatA_[Bacillus_cereus]	CPTF_Mn	2990495	291596.1443	9.75076515
UIJ66630.1	Asp-tRNA(Asn)/Glu-tRNA(Gln)_amidotransferase_subunit_GatA_[Bacillus_cereus]	CPTF_Ni	2910844.033	19650.81214	0.675089834
UIJ66630.1	Asp-tRNA(Asn)/Glu-tRNA(Gln)_amidotransferase_subunit_GatA_[Bacillus_cereus]	CPTF_U	2820765	586784.3196	20.80231141
UIJ66630.1	Asp-tRNA(Asn)/Glu-tRNA(Gln)_amidotransferase_subunit_GatA_[Bacillus_cereus]	CPTF_metals_mix	4028035.467	385442.0928	9.568984583
UIJ66630.1	Asp-tRNA(Asn)/Glu-tRNA(Gln)_amidotransferase_subunit_GatA_[Bacillus_cereus]	CPTF_zcontrol	3406577.667	340123.7274	9.984323292

UIJ66631.1	Asp-tRNA(Asn)/Glu-tRNA(Gln)_amidotransferase_subunit_GatB_[Bacillus_cereus]	CPTF_Al	4253772.133	577487.4261	13.57589001
UIJ66631.1	Asp-tRNA(Asn)/Glu-tRNA(Gln)_amidotransferase_subunit_GatB_[Bacillus_cereus]	CPTF_Cd	5026564.733	197712.127	3.933344888
UIJ66631.1	Asp-tRNA(Asn)/Glu-tRNA(Gln)_amidotransferase_subunit_GatB_[Bacillus_cereus]	CPTF_Co	4908752.2	525397.9669	10.70328966
UIJ66631.1	Asp-tRNA(Asn)/Glu-tRNA(Gln)_amidotransferase_subunit_GatB_[Bacillus_cereus]	CPTF_Cu	4517111.867	220516.5708	4.88180451
UIJ66631.1	Asp-tRNA(Asn)/Glu-tRNA(Gln)_amidotransferase_subunit_GatB_[Bacillus_cereus]	CPTF_Fe	4576857.167	223642.7248	4.886382002
UIJ66631.1	Asp-tRNA(Asn)/Glu-tRNA(Gln)_amidotransferase_subunit_GatB_[Bacillus_cereus]	CPTF_Mn	3683660.667	467372.6126	12.68772167
UIJ66631.1	Asp-tRNA(Asn)/Glu-tRNA(Gln)_amidotransferase_subunit_GatB_[Bacillus_cereus]	CPTF_Ni	4573335.633	143968.8511	3.148005365
UIJ66631.1	Asp-tRNA(Asn)/Glu-tRNA(Gln)_amidotransferase_subunit_GatB_[Bacillus_cereus]	CPTF_U	3896650.033	538028.2704	13.80745681
UIJ66631.1	Asp-tRNA(Asn)/Glu-tRNA(Gln)_amidotransferase_subunit_GatB_[Bacillus_cereus]	CPTF_metals_mix	4500701.5	1134660.316	25.2107436
UIJ66631.1	Asp-tRNA(Asn)/Glu-tRNA(Gln)_amidotransferase_subunit_GatB_[Bacillus_cereus]	CPTF_zcontrol	4157895.5	457851.6073	11.01161891
UIJ66632.1	diacylglycerol_kinase_[Bacillus_cereus]	CPTF_Al	1111037.667	123909.9603	11.15263361
UIJ66632.1	diacylglycerol_kinase_[Bacillus_cereus]	CPTF_Cd	1200233.667	183976.5577	15.32839503
UIJ66632.1	diacylglycerol_kinase_[Bacillus_cereus]	CPTF_Co	1072562.033	195526.1154	18.22981882
UIJ66632.1	diacylglycerol_kinase_[Bacillus_cereus]	CPTF_Cu	907642.3333	311369.9115	34.30535356
UIJ66632.1	diacylglycerol_kinase_[Bacillus_cereus]	CPTF_Fe	1139884.333	221331.5919	19.41702201
UIJ66632.1	diacylglycerol_kinase_[Bacillus_cereus]	CPTF_Mn	978317.6667	127973.916	13.0810186
UIJ66632.1	diacylglycerol_kinase_[Bacillus_cereus]	CPTF_Ni	973038.6667	188819.2355	19.40511122
UIJ66632.1	diacylglycerol_kinase_[Bacillus_cereus]	CPTF_U	726529.7667	189465.4881	26.07814528
UIJ66632.1	diacylglycerol_kinase_[Bacillus_cereus]	CPTF_metals_mix	1554864.667	465555.7963	29.94188538
UIJ66632.1	diacylglycerol_kinase_[Bacillus_cereus]	CPTF_zcontrol	879574	236535.6668	26.89207125
UIJ66639.1	aminopeptidase_[Bacillus_cereus]	CPTF_Al	8909142.1	609698.66	6.843517066
UIJ66639.1	aminopeptidase_[Bacillus_cereus]	CPTF_Cd	9778112.933	411125.6309	4.204549832
UIJ66639.1	aminopeptidase_[Bacillus_cereus]	CPTF_Co	8707487.3	232298.0814	2.667796959
UIJ66639.1	aminopeptidase_[Bacillus_cereus]	CPTF_Cu	8042425.167	457893.3829	5.693473964
UIJ66639.1	aminopeptidase_[Bacillus_cereus]	CPTF_Fe	8890008.933	371406.0787	4.177791962
UIJ66639.1	aminopeptidase_[Bacillus_cereus]	CPTF_Mn	8630130.3	725785.2464	8.409899053
UIJ66639.1	aminopeptidase_[Bacillus_cereus]	CPTF_Ni	6251743.467	553855.6161	8.859218537
UIJ66639.1	aminopeptidase_[Bacillus_cereus]	CPTF_U	7858127.267	1117107.22	14.21594716
UIJ66639.1	aminopeptidase_[Bacillus_cereus]	CPTF_metals_mix	7502272.8	248933.1529	3.318103187
UIJ66639.1	aminopeptidase_[Bacillus_cereus]	CPTF_zcontrol	8648995.733	540973.7269	6.254757703
UIJ66642.1	AimR_family_lysis-lysogeny_pheromone_receptor_[Bacillus_cereus]	CPTF_Al	100216.4667	23727.312	23.67606122
UIJ66642.1	AimR_family_lysis-lysogeny_pheromone_receptor_[Bacillus_cereus]	CPTF_Cd	611262.0333	11546.58484	1.888974648
UIJ66642.1	AimR_family_lysis-lysogeny_pheromone_receptor_[Bacillus_cereus]	CPTF_Co	44938.33333	77835.47654	173.2050808
UIJ66642.1	AimR_family_lysis-lysogeny_pheromone_receptor_[Bacillus_cereus]	CPTF_Cu	42700.6	37543.01784	87.92152297
UIJ66642.1	AimR_family_lysis-lysogeny_pheromone_receptor_[Bacillus_cereus]	CPTF_Fe	126773.6667	125040.138	98.63257981
UIJ66642.1	AimR_family_lysis-lysogeny_pheromone_receptor_[Bacillus_cereus]	CPTF_Mn	26671.96667	46197.2014	173.2050808
UIJ66642.1	AimR_family_lysis-lysogeny_pheromone_receptor_[Bacillus_cereus]	CPTF_Ni	61780.53333	63657.43163	103.0380092
UIJ66642.1	AimR_family_lysis-lysogeny_pheromone_receptor_[Bacillus_cereus]	CPTF_U	29144	50478.88874	173.2050808
UIJ66642.1	AimR_family_lysis-lysogeny_pheromone_receptor_[Bacillus_cereus]	CPTF_metals_mix	0	0	0
UIJ66642.1	AimR_family_lysis-lysogeny_pheromone_receptor_[Bacillus_cereus]	CPTF_zcontrol	0	0	0
UIJ66686.1	DUF262_domain-containing_protein_[Bacillus_cereus]	CPTF_Al	27997.46667	2771.904295	9.900553963
UIJ66686.1	DUF262_domain-containing_protein_[Bacillus_cereus]	CPTF_Cd	23032.4	21117.43758	91.68578861
UIJ66686.1	DUF262_domain-containing_protein_[Bacillus_cereus]	CPTF_Co	23818.56667	2614.74338	10.97775285
UIJ66686.1	DUF262_domain-containing_protein_[Bacillus_cereus]	CPTF_Cu	18366.43333	16766.5997	91.28936141
UIJ66686.1	DUF262_domain-containing_protein_[Bacillus_cereus]	CPTF_Fe	18462.66667	16137.51357	87.40619035
UIJ66686.1	DUF262_domain-containing_protein_[Bacillus_cereus]	CPTF_Mn	9321.366667	16145.08066	173.2050808
UIJ66686.1	DUF262_domain-containing_protein_[Bacillus_cereus]	CPTF_Ni	29608.5	4399.038022	14.85734847
UIJ66686.1	DUF262_domain-containing_protein_[Bacillus_cereus]	CPTF_U	8461.133333	14655.11282	173.2050808
UIJ66686.1	DUF262_domain-containing_protein_[Bacillus_cereus]	CPTF_metals_mix	19647.4	3729.444571	18.98187328
UIJ66686.1	DUF262_domain-containing_protein_[Bacillus_cereus]	CPTF_zcontrol	27750.13333	6611.545761	23.82527565
UIJ66687.1	DUF3696_domain-containing_protein_[Bacillus_cereus]	CPTF_Al	253491.0667	77580.23307	30.60472075
UIJ66687.1	DUF3696_domain-containing_protein_[Bacillus_cereus]	CPTF_Cd	169845.4333	109470.7425	64.45315622
UIJ66687.1	DUF3696_domain-containing_protein_[Bacillus_cereus]	CPTF_Co	80823.93333	71749.17945	88.77219469
UIJ66687.1	DUF3696_domain-containing_protein_[Bacillus_cereus]	CPTF_Cu	124380.3667	78298.58346	62.95091867
UIJ66687.1	DUF3696_domain-containing_protein_[Bacillus_cereus]	CPTF_Fe	190362.2667	37338.42795	19.61440605
UIJ66687.1	DUF3696_domain-containing_protein_[Bacillus_cereus]	CPTF_Mn	114817	99471.89446	86.63516244
UIJ66687.1	DUF3696_domain-containing_protein_[Bacillus_cereus]	CPTF_Ni	138274	119809.1378	86.64617921
UIJ66687.1	DUF3696_domain-containing_protein_[Bacillus_cereus]	CPTF_U	159734.1	57489.12447	35.99051453
UIJ66687.1	DUF3696_domain-containing_protein_[Bacillus_cereus]	CPTF_metals_mix	166022.8	63224.83127	38.08201721
UIJ66687.1	DUF3696_domain-containing_protein_[Bacillus_cereus]	CPTF_zcontrol	196956.5667	116098.2324	58.94610897
UIJ66703.1	dipeptide_epimerase_[Bacillus_cereus]	CPTF_Al	0	0	0

UIJ66703.1	dipeptide_epimerase_[Bacillus_cereus]	CPTF_Cd	0	0	0
UIJ66703.1	dipeptide_epimerase_[Bacillus_cereus]	CPTF_Co	0	0	0
UIJ66703.1	dipeptide_epimerase_[Bacillus_cereus]	CPTF_Cu	0	0	0
UIJ66703.1	dipeptide_epimerase_[Bacillus_cereus]	CPTF_Fe	25273.16667	43774.40873	173.2050808
UIJ66703.1	dipeptide_epimerase_[Bacillus_cereus]	CPTF_Mn	20413.76667	35357.68104	173.2050808
UIJ66703.1	dipeptide_epimerase_[Bacillus_cereus]	CPTF_Ni	0	0	0
UIJ66703.1	dipeptide_epimerase_[Bacillus_cereus]	CPTF_U	0	0	0
UIJ66703.1	dipeptide_epimerase_[Bacillus_cereus]	CPTF_metals_mix	0	0	0
UIJ66703.1	dipeptide_epimerase_[Bacillus_cereus]	CPTF_zcontrol	13773.9	23857.09462	173.2050808
UIJ66706.1	M20_peptidase_aminoacylase_family_protein_[Bacillus_cereus]	CPTF_Al	6274.5	10867.75279	173.2050808
UIJ66706.1	M20_peptidase_aminoacylase_family_protein_[Bacillus_cereus]	CPTF_Cd	0	0	0
UIJ66706.1	M20_peptidase_aminoacylase_family_protein_[Bacillus_cereus]	CPTF_Co	2842.583333	4923.498758	173.2050808
UIJ66706.1	M20_peptidase_aminoacylase_family_protein_[Bacillus_cereus]	CPTF_Cu	0	0	0
UIJ66706.1	M20_peptidase_aminoacylase_family_protein_[Bacillus_cereus]	CPTF_Fe	0	0	0
UIJ66706.1	M20_peptidase_aminoacylase_family_protein_[Bacillus_cereus]	CPTF_Mn	0	0	0
UIJ66706.1	M20_peptidase_aminoacylase_family_protein_[Bacillus_cereus]	CPTF_Ni	0	0	0
UIJ66706.1	M20_peptidase_aminoacylase_family_protein_[Bacillus_cereus]	CPTF_U	0	0	0
UIJ66706.1	M20_peptidase_aminoacylase_family_protein_[Bacillus_cereus]	CPTF_metals_mix	77972.73333	44665.18788	57.28308598
UIJ66706.1	M20_peptidase_aminoacylase_family_protein_[Bacillus_cereus]	CPTF_zcontrol	0	0	0
UIJ66708.1	alkyl_hydroperoxide_reductase_subunit_F_[Bacillus_cereus]	CPTF_Al	2106747.3	159866.8036	7.588323649
UIJ66708.1	alkyl_hydroperoxide_reductase_subunit_F_[Bacillus_cereus]	CPTF_Cd	2195954.967	106808.395	4.863870005
UIJ66708.1	alkyl_hydroperoxide_reductase_subunit_F_[Bacillus_cereus]	CPTF_Co	2369633.433	165150.6452	6.969459617
UIJ66708.1	alkyl_hydroperoxide_reductase_subunit_F_[Bacillus_cereus]	CPTF_Cu	3230568.9	181471.0867	5.617310522
UIJ66708.1	alkyl_hydroperoxide_reductase_subunit_F_[Bacillus_cereus]	CPTF_Fe	2149698.633	436425.0568	20.30168555
UIJ66708.1	alkyl_hydroperoxide_reductase_subunit_F_[Bacillus_cereus]	CPTF_Mn	2151133.667	346722.4462	16.11812653
UIJ66708.1	alkyl_hydroperoxide_reductase_subunit_F_[Bacillus_cereus]	CPTF_Ni	2358616.667	244514.9142	10.36687808
UIJ66708.1	alkyl_hydroperoxide_reductase_subunit_F_[Bacillus_cereus]	CPTF_U	1452044.633	990316.9616	68.20155103
UIJ66708.1	alkyl_hydroperoxide_reductase_subunit_F_[Bacillus_cereus]	CPTF_metals_mix	7828124.033	1259687.46	16.09181784
UIJ66708.1	alkyl_hydroperoxide_reductase_subunit_F_[Bacillus_cereus]	CPTF_zcontrol	1943114.667	257826.7123	13.26873379
UIJ66709.1	peroxiredoxin_[Bacillus_cereus]	CPTF_Al	10099693.8	316193.234	3.130720992
UIJ66709.1	peroxiredoxin_[Bacillus_cereus]	CPTF_Cd	11635608	879963.8859	7.562680746
UIJ66709.1	peroxiredoxin_[Bacillus_cereus]	CPTF_Co	10314822.47	579266.1505	5.615861566
UIJ66709.1	peroxiredoxin_[Bacillus_cereus]	CPTF_Cu	12585841	1114202.961	8.852828837
UIJ66709.1	peroxiredoxin_[Bacillus_cereus]	CPTF_Fe	9608066.333	603325.5701	6.279365162
UIJ66709.1	peroxiredoxin_[Bacillus_cereus]	CPTF_Mn	11494477.33	3414430.36	29.70496405
UIJ66709.1	peroxiredoxin_[Bacillus_cereus]	CPTF_Ni	9295454	702215.2236	7.554394046
UIJ66709.1	peroxiredoxin_[Bacillus_cereus]	CPTF_U	7379712.267	1084525.372	14.69603872
UIJ66709.1	peroxiredoxin_[Bacillus_cereus]	CPTF_metals_mix	40165790.33	4105845.077	10.22224396
UIJ66709.1	peroxiredoxin_[Bacillus_cereus]	CPTF_zcontrol	9251119.633	334789.8503	3.618911695
UIJ66715.1	iron-hydroxamate_ABC_transporter_substrate-binding_protein_[Bacillus_cereus]	CPTF_Al	0	0	0
UIJ66715.1	iron-hydroxamate_ABC_transporter_substrate-binding_protein_[Bacillus_cereus]	CPTF_Cd	0	0	0
UIJ66715.1	iron-hydroxamate_ABC_transporter_substrate-binding_protein_[Bacillus_cereus]	CPTF_Co	0	0	0
UIJ66715.1	iron-hydroxamate_ABC_transporter_substrate-binding_protein_[Bacillus_cereus]	CPTF_Cu	15790.53333	27350.00601	173.2050808
UIJ66715.1	iron-hydroxamate_ABC_transporter_substrate-binding_protein_[Bacillus_cereus]	CPTF_Fe	0	0	0
UIJ66715.1	iron-hydroxamate_ABC_transporter_substrate-binding_protein_[Bacillus_cereus]	CPTF_Mn	0	0	0
UIJ66715.1	iron-hydroxamate_ABC_transporter_substrate-binding_protein_[Bacillus_cereus]	CPTF_Ni	0	0	0
UIJ66715.1	iron-hydroxamate_ABC_transporter_substrate-binding_protein_[Bacillus_cereus]	CPTF_U	0	0	0
UIJ66715.1	iron-hydroxamate_ABC_transporter_substrate-binding_protein_[Bacillus_cereus]	CPTF_metals_mix	1095552.3	61727.1789	5.634343418
UIJ66715.1	iron-hydroxamate_ABC_transporter_substrate-binding_protein_[Bacillus_cereus]	CPTF_zcontrol	0	0	0
UIJ66716.1	NAD(P)/FAD-dependent_oxidoreductase_[Bacillus_cereus]	CPTF_Al	0	0	0
UIJ66716.1	NAD(P)/FAD-dependent_oxidoreductase_[Bacillus_cereus]	CPTF_Cd	0	0	0
UIJ66716.1	NAD(P)/FAD-dependent_oxidoreductase_[Bacillus_cereus]	CPTF_Co	0	0	0
UIJ66716.1	NAD(P)/FAD-dependent_oxidoreductase_[Bacillus_cereus]	CPTF_Cu	0	0	0
UIJ66716.1	NAD(P)/FAD-dependent_oxidoreductase_[Bacillus_cereus]	CPTF_Fe	0	0	0
UIJ66716.1	NAD(P)/FAD-dependent_oxidoreductase_[Bacillus_cereus]	CPTF_Mn	0	0	0
UIJ66716.1	NAD(P)/FAD-dependent_oxidoreductase_[Bacillus_cereus]	CPTF_Ni	0	0	0
UIJ66716.1	NAD(P)/FAD-dependent_oxidoreductase_[Bacillus_cereus]	CPTF_U	0	0	0
UIJ66716.1	NAD(P)/FAD-dependent_oxidoreductase_[Bacillus_cereus]	CPTF_metals_mix	467724.3333	113907.0172	24.35345119
UIJ66716.1	NAD(P)/FAD-dependent_oxidoreductase_[Bacillus_cereus]	CPTF_zcontrol	0	0	0
UIJ66717.1	hypothetical_protein_LW858_28775_[Bacillus_cereus]	CPTF_Al	216076	44430.61999	20.56249653
UIJ66717.1	hypothetical_protein_LW858_28775_[Bacillus_cereus]	CPTF_Cd	236535.3333	27624.15636	11.67865958

UIJ66717.1	hypothetical_protein_LW858_28775_[Bacillus_cereus]	CPTF_Co	240445.6667	16345.84441	6.798144725
UIJ66717.1	hypothetical_protein_LW858_28775_[Bacillus_cereus]	CPTF_Cu	202348	17693.66415	8.744175454
UIJ66717.1	hypothetical_protein_LW858_28775_[Bacillus_cereus]	CPTF_Fe	194129	33865.01107	17.44459152
UIJ66717.1	hypothetical_protein_LW858_28775_[Bacillus_cereus]	CPTF_Mn	187847	58761.18681	31.28140817
UIJ66717.1	hypothetical_protein_LW858_28775_[Bacillus_cereus]	CPTF_Ni	179072	58305.85017	32.56000389
UIJ66717.1	hypothetical_protein_LW858_28775_[Bacillus_cereus]	CPTF_U	225467.3333	53282.64784	23.63209209
UIJ66717.1	hypothetical_protein_LW858_28775_[Bacillus_cereus]	CPTF_metals_mix	144903.3333	24148.23425	16.6650647
UIJ66717.1	hypothetical_protein_LW858_28775_[Bacillus_cereus]	CPTF_zcontrol	186150.6667	48342.2315	25.96941089
UIJ66726.1	nucleotidyltransferase_domain-containing_protein_[Bacillus_cereus]	CPTF_Al	534906	280635.6766	52.46448472
UIJ66726.1	nucleotidyltransferase_domain-containing_protein_[Bacillus_cereus]	CPTF_Cd	775321.6667	55462.36491	7.153465109
UIJ66726.1	nucleotidyltransferase_domain-containing_protein_[Bacillus_cereus]	CPTF_Co	346642.6667	263236.8889	75.93897526
UIJ66726.1	nucleotidyltransferase_domain-containing_protein_[Bacillus_cereus]	CPTF_Cu	553913	236952.1635	42.77786648
UIJ66726.1	nucleotidyltransferase_domain-containing_protein_[Bacillus_cereus]	CPTF_Fe	645950.6667	337077.8257	52.18321508
UIJ66726.1	nucleotidyltransferase_domain-containing_protein_[Bacillus_cereus]	CPTF_Mn	310799.3333	116060.9457	37.34272673
UIJ66726.1	nucleotidyltransferase_domain-containing_protein_[Bacillus_cereus]	CPTF_Ni	260158.6667	32877.28435	12.63739731
UIJ66726.1	nucleotidyltransferase_domain-containing_protein_[Bacillus_cereus]	CPTF_U	229281.3333	40653.76837	17.73095427
UIJ66726.1	nucleotidyltransferase_domain-containing_protein_[Bacillus_cereus]	CPTF_metals_mix	572319	81452.95248	14.23208953
UIJ66726.1	nucleotidyltransferase_domain-containing_protein_[Bacillus_cereus]	CPTF_zcontrol	232806.3333	65791.1224	28.26002259
UIJ66729.1	acyl-CoA_desaturase_[Bacillus_cereus]	CPTF_Al	46296.33333	80187.60154	173.2050808
UIJ66729.1	acyl-CoA_desaturase_[Bacillus_cereus]	CPTF_Cd	217190.3333	192254.8698	88.51907306
UIJ66729.1	acyl-CoA_desaturase_[Bacillus_cereus]	CPTF_Co	90353	156495.9866	173.2050808
UIJ66729.1	acyl-CoA_desaturase_[Bacillus_cereus]	CPTF_Cu	0	0	0
UIJ66729.1	acyl-CoA_desaturase_[Bacillus_cereus]	CPTF_Fe	89165.66667	154439.465	173.2050808
UIJ66729.1	acyl-CoA_desaturase_[Bacillus_cereus]	CPTF_Mn	182193.3333	204693.5427	112.3496338
UIJ66729.1	acyl-CoA_desaturase_[Bacillus_cereus]	CPTF_Ni	0	0	0
UIJ66729.1	acyl-CoA_desaturase_[Bacillus_cereus]	CPTF_U	0	0	0
UIJ66729.1	acyl-CoA_desaturase_[Bacillus_cereus]	CPTF_metals_mix	980613.3667	132620.0898	13.52419764
UIJ66729.1	acyl-CoA_desaturase_[Bacillus_cereus]	CPTF_zcontrol	133629	143342.4915	107.2689996
UIJ66731.1	ABC_transporter_substrate-binding_protein_[Bacillus_cereus]	CPTF_Al	177052.3333	306663.6369	173.2050808
UIJ66731.1	ABC_transporter_substrate-binding_protein_[Bacillus_cereus]	CPTF_Cd	103791.4333	52396.00869	50.48201669
UIJ66731.1	ABC_transporter_substrate-binding_protein_[Bacillus_cereus]	CPTF_Co	469770.6333	361963.1421	77.05103649
UIJ66731.1	ABC_transporter_substrate-binding_protein_[Bacillus_cereus]	CPTF_Cu	109854.6	140505.4389	127.9012794
UIJ66731.1	ABC_transporter_substrate-binding_protein_[Bacillus_cereus]	CPTF_Fe	31233.16667	54097.43155	173.2050808
UIJ66731.1	ABC_transporter_substrate-binding_protein_[Bacillus_cereus]	CPTF_Mn	10849.7	18792.23165	173.2050808
UIJ66731.1	ABC_transporter_substrate-binding_protein_[Bacillus_cereus]	CPTF_Ni	364544	315720.6125	86.60699737
UIJ66731.1	ABC_transporter_substrate-binding_protein_[Bacillus_cereus]	CPTF_U	245717	425594.3283	173.2050808
UIJ66731.1	ABC_transporter_substrate-binding_protein_[Bacillus_cereus]	CPTF_metals_mix	15678.5	27155.95859	173.2050808
UIJ66731.1	ABC_transporter_substrate-binding_protein_[Bacillus_cereus]	CPTF_zcontrol	0	0	0
UIJ66735.1	arginine_deiminase_[Bacillus_cereus]	CPTF_Al	14236388.17	871812.3357	6.123830887
UIJ66735.1	arginine_deiminase_[Bacillus_cereus]	CPTF_Cd	15543669.2	2307853.311	14.84754521
UIJ66735.1	arginine_deiminase_[Bacillus_cereus]	CPTF_Co	15556570.33	1402520.271	9.015613602
UIJ66735.1	arginine_deiminase_[Bacillus_cereus]	CPTF_Cu	17342452.1	1913192.395	11.03184477
UIJ66735.1	arginine_deiminase_[Bacillus_cereus]	CPTF_Fe	14445874.17	1889543.036	13.0801571
UIJ66735.1	arginine_deiminase_[Bacillus_cereus]	CPTF_Mn	13299391.87	946482.7966	7.116737412
UIJ66735.1	arginine_deiminase_[Bacillus_cereus]	CPTF_Ni	15424293.73	1302952.246	8.447402965
UIJ66735.1	arginine_deiminase_[Bacillus_cereus]	CPTF_U	16080089.1	1862686.551	11.5838074
UIJ66735.1	arginine_deiminase_[Bacillus_cereus]	CPTF_metals_mix	24769166.4	1359238.252	5.487622109
UIJ66735.1	arginine_deiminase_[Bacillus_cereus]	CPTF_zcontrol	14627208.09	2323531.711	15.88499799
UIJ66736.1	ornithine_carbamoyltransferase_[Bacillus_cereus]	CPTF_Al	71416	123696.1405	173.2050808
UIJ66736.1	ornithine_carbamoyltransferase_[Bacillus_cereus]	CPTF_Cd	124960.8333	147385.7541	117.9455595
UIJ66736.1	ornithine_carbamoyltransferase_[Bacillus_cereus]	CPTF_Co	155552.5333	112915.8573	72.59017575
UIJ66736.1	ornithine_carbamoyltransferase_[Bacillus_cereus]	CPTF_Cu	194975.3333	53417.04797	27.39682352
UIJ66736.1	ornithine_carbamoyltransferase_[Bacillus_cereus]	CPTF_Fe	51519	89233.52556	173.2050808
UIJ66736.1	ornithine_carbamoyltransferase_[Bacillus_cereus]	CPTF_Mn	43661.33333	75623.64766	173.2050808
UIJ66736.1	ornithine_carbamoyltransferase_[Bacillus_cereus]	CPTF_Ni	68475.66667	118603.3337	173.2050808
UIJ66736.1	ornithine_carbamoyltransferase_[Bacillus_cereus]	CPTF_U	23798.6	41220.38435	173.2050808
UIJ66736.1	ornithine_carbamoyltransferase_[Bacillus_cereus]	CPTF_metals_mix	450510.6333	68609.24715	15.22921815
UIJ66736.1	ornithine_carbamoyltransferase_[Bacillus_cereus]	CPTF_zcontrol	80505.63333	76909.94361	95.53361725
UIJ66738.1	carbamate_kinase_[Bacillus_cereus]	CPTF_Al	0	0	0
UIJ66738.1	carbamate_kinase_[Bacillus_cereus]	CPTF_Cd	0	0	0
UIJ66738.1	carbamate_kinase_[Bacillus_cereus]	CPTF_Co	0	0	0

UIJ66738.1	carbamate_kinase_[Bacillus_cereus]	CPTF_Cu	0	0	0
UIJ66738.1	carbamate_kinase_[Bacillus_cereus]	CPTF_Fe	0	0	0
UIJ66738.1	carbamate_kinase_[Bacillus_cereus]	CPTF_Mn	0	0	0
UIJ66738.1	carbamate_kinase_[Bacillus_cereus]	CPTF_Ni	0	0	0
UIJ66738.1	carbamate_kinase_[Bacillus_cereus]	CPTF_U	0	0	0
UIJ66738.1	carbamate_kinase_[Bacillus_cereus]	CPTF_metals_mix	13092.83333	22677.45255	173.2050808
UIJ66738.1	carbamate_kinase_[Bacillus_cereus]	CPTF_zcontrol	0	0	0
UIJ66742.1	alpha-glucosidase_[Bacillus_cereus]	CPTF_Al	227931.3667	173655.7138	76.18772103
UIJ66742.1	alpha-glucosidase_[Bacillus_cereus]	CPTF_Cd	200918.7	174005.3078	86.60483461
UIJ66742.1	alpha-glucosidase_[Bacillus_cereus]	CPTF_Co	161794.6	124077.3889	76.68821387
UIJ66742.1	alpha-glucosidase_[Bacillus_cereus]	CPTF_Cu	305378.7	18285.60356	5.987845113
UIJ66742.1	alpha-glucosidase_[Bacillus_cereus]	CPTF_Fe	127527.8667	126076.4628	98.8618928
UIJ66742.1	alpha-glucosidase_[Bacillus_cereus]	CPTF_Mn	49775.56667	70780.32461	142.1989328
UIJ66742.1	alpha-glucosidase_[Bacillus_cereus]	CPTF_Ni	296165.8333	7820.472866	2.640572269
UIJ66742.1	alpha-glucosidase_[Bacillus_cereus]	CPTF_U	189152.8333	125598.0587	66.40030523
UIJ66742.1	alpha-glucosidase_[Bacillus_cereus]	CPTF_metals_mix	16583.63333	17318.24229	104.4297226
UIJ66742.1	alpha-glucosidase_[Bacillus_cereus]	CPTF_zcontrol	180900.3333	159352.8569	88.08875803
UIJ66745.1	YhgE/Pip_domain-containing_protein_[Bacillus_cereus]	CPTF_Al	0	0	0
UIJ66745.1	YhgE/Pip_domain-containing_protein_[Bacillus_cereus]	CPTF_Cd	0	0	0
UIJ66745.1	YhgE/Pip_domain-containing_protein_[Bacillus_cereus]	CPTF_Co	0	0	0
UIJ66745.1	YhgE/Pip_domain-containing_protein_[Bacillus_cereus]	CPTF_Cu	6194.166667	10728.61138	173.2050808
UIJ66745.1	YhgE/Pip_domain-containing_protein_[Bacillus_cereus]	CPTF_Fe	0	0	0
UIJ66745.1	YhgE/Pip_domain-containing_protein_[Bacillus_cereus]	CPTF_Mn	0	0	0
UIJ66745.1	YhgE/Pip_domain-containing_protein_[Bacillus_cereus]	CPTF_Ni	0	0	0
UIJ66745.1	YhgE/Pip_domain-containing_protein_[Bacillus_cereus]	CPTF_U	0	0	0
UIJ66745.1	YhgE/Pip_domain-containing_protein_[Bacillus_cereus]	CPTF_metals_mix	0	0	0
UIJ66745.1	YhgE/Pip_domain-containing_protein_[Bacillus_cereus]	CPTF_zcontrol	0	0	0
UIJ66746.1	DNA_topoisomerase_III_[Bacillus_cereus]	CPTF_Al	535701.7333	73977.67915	13.80949035
UIJ66746.1	DNA_topoisomerase_III_[Bacillus_cereus]	CPTF_Cd	474813.1333	108824.7488	22.91949005
UIJ66746.1	DNA_topoisomerase_III_[Bacillus_cereus]	CPTF_Co	447841	27516.65988	6.144292256
UIJ66746.1	DNA_topoisomerase_III_[Bacillus_cereus]	CPTF_Cu	595967.4	89418.10339	15.00385816
UIJ66746.1	DNA_topoisomerase_III_[Bacillus_cereus]	CPTF_Fe	589019.3	95734.07568	16.25313053
UIJ66746.1	DNA_topoisomerase_III_[Bacillus_cereus]	CPTF_Mn	302307.3333	145923.8897	48.27004629
UIJ66746.1	DNA_topoisomerase_III_[Bacillus_cereus]	CPTF_Ni	524230.3333	31080.68269	5.928821877
UIJ66746.1	DNA_topoisomerase_III_[Bacillus_cereus]	CPTF_U	341914.3333	131192.5652	38.37001039
UIJ66746.1	DNA_topoisomerase_III_[Bacillus_cereus]	CPTF_metals_mix	533570.0333	26928.2619	5.046809269
UIJ66746.1	DNA_topoisomerase_III_[Bacillus_cereus]	CPTF_zcontrol	443271.2333	152194.8471	34.33447416
UIJ66811.1	ectonucleotide_pyrophosphatase/phosphodiesterase_[Bacillus_cereus]	CPTF_Al	1038421	162374.2582	15.63665009
UIJ66811.1	ectonucleotide_pyrophosphatase/phosphodiesterase_[Bacillus_cereus]	CPTF_Cd	650440.6667	581496.8122	89.40043911
UIJ66811.1	ectonucleotide_pyrophosphatase/phosphodiesterase_[Bacillus_cereus]	CPTF_Co	303638.3	499826.1542	164.6123543
UIJ66811.1	ectonucleotide_pyrophosphatase/phosphodiesterase_[Bacillus_cereus]	CPTF_Cu	629276	590005.1631	93.75936205
UIJ66811.1	ectonucleotide_pyrophosphatase/phosphodiesterase_[Bacillus_cereus]	CPTF_Fe	423687.6333	656566.3789	154.9647257
UIJ66811.1	ectonucleotide_pyrophosphatase/phosphodiesterase_[Bacillus_cereus]	CPTF_Mn	343333.3333	594670.7773	173.2050808
UIJ66811.1	ectonucleotide_pyrophosphatase/phosphodiesterase_[Bacillus_cereus]	CPTF_Ni	876666.6667	794753.6306	90.65630767
UIJ66811.1	ectonucleotide_pyrophosphatase/phosphodiesterase_[Bacillus_cereus]	CPTF_U	716666.6667	1241303.079	173.2050808
UIJ66811.1	ectonucleotide_pyrophosphatase/phosphodiesterase_[Bacillus_cereus]	CPTF_metals_mix	0	0	0
UIJ66811.1	ectonucleotide_pyrophosphatase/phosphodiesterase_[Bacillus_cereus]	CPTF_zcontrol	0	0	0
UIJ66817.1	TerD_family_protein_[Bacillus_cereus]	CPTF_Al	4644556	1102021.763	23.72717141
UIJ66817.1	TerD_family_protein_[Bacillus_cereus]	CPTF_Cd	4704187	769830.1692	16.36478672
UIJ66817.1	TerD_family_protein_[Bacillus_cereus]	CPTF_Co	4873682.667	319113.6788	6.547690948
UIJ66817.1	TerD_family_protein_[Bacillus_cereus]	CPTF_Cu	6734715.667	435758.7152	6.470335746
UIJ66817.1	TerD_family_protein_[Bacillus_cereus]	CPTF_Fe	4429524.667	1073076.236	24.22553923
UIJ66817.1	TerD_family_protein_[Bacillus_cereus]	CPTF_Mn	4735573	598981.6814	12.64855766
UIJ66817.1	TerD_family_protein_[Bacillus_cereus]	CPTF_Ni	4171597	722257.2704	17.31368755
UIJ66817.1	TerD_family_protein_[Bacillus_cereus]	CPTF_U	4007302	676151.6212	16.87298889
UIJ66817.1	TerD_family_protein_[Bacillus_cereus]	CPTF_metals_mix	11233026.07	578126.4094	5.146666677
UIJ66817.1	TerD_family_protein_[Bacillus_cereus]	CPTF_zcontrol	4453202.333	406420.3352	9.126473598
UIJ66818.1	TerD_family_protein_[Bacillus_cereus]	CPTF_Al	3912954.167	104400.3559	2.66806999
UIJ66818.1	TerD_family_protein_[Bacillus_cereus]	CPTF_Cd	4329895.4	286884.6263	6.625671057
UIJ66818.1	TerD_family_protein_[Bacillus_cereus]	CPTF_Co	4099252.5	122607.375	2.990969085
UIJ66818.1	TerD_family_protein_[Bacillus_cereus]	CPTF_Cu	5456363.667	287957.8061	5.27746726

UIJ66818.1	TerD_family_protein_[Bacillus_cereus]	CPTF_Fe	3778486.4	254291.1391	6.729973651
UIJ66818.1	TerD_family_protein_[Bacillus_cereus]	CPTF_Mn	4192354.4	610239.9958	14.55602121
UIJ66818.1	TerD_family_protein_[Bacillus_cereus]	CPTF_Ni	4054379.133	426419.0503	10.51749322
UIJ66818.1	TerD_family_protein_[Bacillus_cereus]	CPTF_U	3821622.8	342380.3046	8.959029254
UIJ66818.1	TerD_family_protein_[Bacillus_cereus]	CPTF_metals_mix	14963174.17	1977669.182	13.21690946
UIJ66818.1	TerD_family_protein_[Bacillus_cereus]	CPTF_zcontrol	3770628.233	157417.1425	4.174825327
UIJ66819.1	TerD_family_protein_[Bacillus_cereus]	CPTF_Al	2600898.433	473827.3694	18.2178344
UIJ66819.1	TerD_family_protein_[Bacillus_cereus]	CPTF_Cd	3073422.5	142938.475	4.650791586
UIJ66819.1	TerD_family_protein_[Bacillus_cereus]	CPTF_Co	2200406	255104.4423	11.59351694
UIJ66819.1	TerD_family_protein_[Bacillus_cereus]	CPTF_Cu	3160886.633	83037.19127	2.627022127
UIJ66819.1	TerD_family_protein_[Bacillus_cereus]	CPTF_Fe	2701104.733	336783.7525	12.46837075
UIJ66819.1	TerD_family_protein_[Bacillus_cereus]	CPTF_Mn	2279422.9	524792.6897	23.02305069
UIJ66819.1	TerD_family_protein_[Bacillus_cereus]	CPTF_Ni	1814175.667	184196.9255	10.1532023
UIJ66819.1	TerD_family_protein_[Bacillus_cereus]	CPTF_U	1841554.3	70342.5813	3.819739733
UIJ66819.1	TerD_family_protein_[Bacillus_cereus]	CPTF_metals_mix	6078481.367	382844.0949	6.298351048
UIJ66819.1	TerD_family_protein_[Bacillus_cereus]	CPTF_zcontrol	2105127.967	169571.1242	8.055145666
UIJ66821.1	YceG_family_protein_[Bacillus_cereus]	CPTF_Al	89439.66667	77644.22974	86.81185053
UIJ66821.1	YceG_family_protein_[Bacillus_cereus]	CPTF_Cd	0	0	0
UIJ66821.1	YceG_family_protein_[Bacillus_cereus]	CPTF_Co	46360.5	57997.12498	125.1003009
UIJ66821.1	YceG_family_protein_[Bacillus_cereus]	CPTF_Cu	34158.66667	59164.54619	173.2050808
UIJ66821.1	YceG_family_protein_[Bacillus_cereus]	CPTF_Fe	23597.96667	40872.87722	173.2050808
UIJ66821.1	YceG_family_protein_[Bacillus_cereus]	CPTF_Mn	23108.43333	40024.98062	173.2050808
UIJ66821.1	YceG_family_protein_[Bacillus_cereus]	CPTF_Ni	46885.66667	81208.35681	173.2050808
UIJ66821.1	YceG_family_protein_[Bacillus_cereus]	CPTF_U	44473.33333	77030.07292	173.2050808
UIJ66821.1	YceG_family_protein_[Bacillus_cereus]	CPTF_metals_mix	69156.5	50200.22426	72.58930723
UIJ66821.1	YceG_family_protein_[Bacillus_cereus]	CPTF_zcontrol	71416.66667	61852.36368	86.60774377
UIJ66822.1	toxic_anion_resistance_protein_[Bacillus_cereus]	CPTF_Al	336533.2333	86301.36946	25.64423389
UIJ66822.1	toxic_anion_resistance_protein_[Bacillus_cereus]	CPTF_Cd	225032.7333	131487.2506	58.43027752
UIJ66822.1	toxic_anion_resistance_protein_[Bacillus_cereus]	CPTF_Co	380044.2333	152584.5066	40.14914403
UIJ66822.1	toxic_anion_resistance_protein_[Bacillus_cereus]	CPTF_Cu	581994.3667	465350.5369	79.95791085
UIJ66822.1	toxic_anion_resistance_protein_[Bacillus_cereus]	CPTF_Fe	312522.8	174375.906	55.79621904
UIJ66822.1	toxic_anion_resistance_protein_[Bacillus_cereus]	CPTF_Mn	619481.7	304231.7143	49.11068628
UIJ66822.1	toxic_anion_resistance_protein_[Bacillus_cereus]	CPTF_Ni	475085.8333	193907.2599	40.81520564
UIJ66822.1	toxic_anion_resistance_protein_[Bacillus_cereus]	CPTF_U	359608.3333	368153.0066	102.3761055
UIJ66822.1	toxic_anion_resistance_protein_[Bacillus_cereus]	CPTF_metals_mix	1054344.8	205148.6632	19.45745482
UIJ66822.1	toxic_anion_resistance_protein_[Bacillus_cereus]	CPTF_zcontrol	301558.3333	268370.9953	88.99472031
UIJ66823.1	cation-translocating_P-type_ATPase_[Bacillus_cereus]	CPTF_Al	69116.1	61178.59483	88.51569291
UIJ66823.1	cation-translocating_P-type_ATPase_[Bacillus_cereus]	CPTF_Cd	131151.0667	179069.5931	136.5368942
UIJ66823.1	cation-translocating_P-type_ATPase_[Bacillus_cereus]	CPTF_Co	88260.1	14191.97591	16.07971882
UIJ66823.1	cation-translocating_P-type_ATPase_[Bacillus_cereus]	CPTF_Cu	44151.56667	38418.43903	87.01489421
UIJ66823.1	cation-translocating_P-type_ATPase_[Bacillus_cereus]	CPTF_Fe	76320	132190.1176	173.2050808
UIJ66823.1	cation-translocating_P-type_ATPase_[Bacillus_cereus]	CPTF_Mn	152974.2333	137535.2454	89.90745852
UIJ66823.1	cation-translocating_P-type_ATPase_[Bacillus_cereus]	CPTF_Ni	116461.3	23887.01369	20.51068783
UIJ66823.1	cation-translocating_P-type_ATPase_[Bacillus_cereus]	CPTF_U	0	0	0
UIJ66823.1	cation-translocating_P-type_ATPase_[Bacillus_cereus]	CPTF_metals_mix	90835.06667	61858.86007	68.1001978
UIJ66823.1	cation-translocating_P-type_ATPase_[Bacillus_cereus]	CPTF_zcontrol	50337	87186.2415	173.2050808
UIJ66826.1	DUF4075_domain-containing_protein_[Bacillus_cereus]	CPTF_Al	1639728.333	370522.3406	22.59656878
UIJ66826.1	DUF4075_domain-containing_protein_[Bacillus_cereus]	CPTF_Cd	2251307	52478.46394	2.33102211
UIJ66826.1	DUF4075_domain-containing_protein_[Bacillus_cereus]	CPTF_Co	1609081.333	69093.4003	4.293965685
UIJ66826.1	DUF4075_domain-containing_protein_[Bacillus_cereus]	CPTF_Cu	1235777.333	145483.6693	11.77264426
UIJ66826.1	DUF4075_domain-containing_protein_[Bacillus_cereus]	CPTF_Fe	1919451.333	194704.896	10.14377872
UIJ66826.1	DUF4075_domain-containing_protein_[Bacillus_cereus]	CPTF_Mn	1801153.5	418001.0319	23.20740747
UIJ66826.1	DUF4075_domain-containing_protein_[Bacillus_cereus]	CPTF_Ni	1443333.333	90737.71726	6.28667787
UIJ66826.1	DUF4075_domain-containing_protein_[Bacillus_cereus]	CPTF_U	1431785	179819.1382	12.55908801
UIJ66826.1	DUF4075_domain-containing_protein_[Bacillus_cereus]	CPTF_metals_mix	1941502.133	659549.0156	33.97106829
UIJ66826.1	DUF4075_domain-containing_protein_[Bacillus_cereus]	CPTF_zcontrol	1415809.333	152469.5429	10.76907316
UIJ66828.1	cadmium-translocating_P-type_ATPase_[Bacillus_cereus]	CPTF_Al	0	0	0
UIJ66828.1	cadmium-translocating_P-type_ATPase_[Bacillus_cereus]	CPTF_Cd	0	0	0
UIJ66828.1	cadmium-translocating_P-type_ATPase_[Bacillus_cereus]	CPTF_Co	22577.03333	21036.66432	93.1772745
UIJ66828.1	cadmium-translocating_P-type_ATPase_[Bacillus_cereus]	CPTF_Cu	116775.6667	202261.3878	173.2050808
UIJ66828.1	cadmium-translocating_P-type_ATPase_[Bacillus_cereus]	CPTF_Fe	5873.3	10172.85401	173.2050808

UIJ66828.1	cadmium-translocating_P-type_ATPase_[Bacillus_cereus]	CPTF_Mn	0	0	0
UIJ66828.1	cadmium-translocating_P-type_ATPase_[Bacillus_cereus]	CPTF_Ni	0	0	0
UIJ66828.1	cadmium-translocating_P-type_ATPase_[Bacillus_cereus]	CPTF_U	0	0	0
UIJ66828.1	cadmium-translocating_P-type_ATPase_[Bacillus_cereus]	CPTF_metals_mix	89243.03333	77315.70739	86.63500612
UIJ66828.1	cadmium-translocating_P-type_ATPase_[Bacillus_cereus]	CPTF_zcontrol	0	0	0
UIJ66832.1	GTP-binding_protein_[Bacillus_cereus]	CPTF_Al	545712.2333	89276.19592	16.35957387
UIJ66832.1	GTP-binding_protein_[Bacillus_cereus]	CPTF_Cd	516910.9	56037.40771	10.84082532
UIJ66832.1	GTP-binding_protein_[Bacillus_cereus]	CPTF_Co	520398.4	151501.706	29.11263871
UIJ66832.1	GTP-binding_protein_[Bacillus_cereus]	CPTF_Cu	532534.6333	65303.22888	12.2627196
UIJ66832.1	GTP-binding_protein_[Bacillus_cereus]	CPTF_Fe	495534	17603.3122	3.552392408
UIJ66832.1	GTP-binding_protein_[Bacillus_cereus]	CPTF_Mn	525342.7	127411.615	24.25304757
UIJ66832.1	GTP-binding_protein_[Bacillus_cereus]	CPTF_Ni	542646.3667	172157.2998	31.72550493
UIJ66832.1	GTP-binding_protein_[Bacillus_cereus]	CPTF_U	535796.0333	65522.25663	12.22895515
UIJ66832.1	GTP-binding_protein_[Bacillus_cereus]	CPTF_metals_mix	810353.2333	202950.1165	25.0446482
UIJ66832.1	GTP-binding_protein_[Bacillus_cereus]	CPTF_zcontrol	332241.2	16090.11913	4.842903025
UIJ66839.1	hypothetical_protein_LW858_00340_[Bacillus_cereus]	CPTF_Al	66987.66667	58013.44775	86.60317733
UIJ66839.1	hypothetical_protein_LW858_00340_[Bacillus_cereus]	CPTF_Cd	123672.4667	58727.06032	47.48596184
UIJ66839.1	hypothetical_protein_LW858_00340_[Bacillus_cereus]	CPTF_Co	90082.83333	39552.20393	43.90648303
UIJ66839.1	hypothetical_protein_LW858_00340_[Bacillus_cereus]	CPTF_Cu	143096.8	65198.14985	45.56226963
UIJ66839.1	hypothetical_protein_LW858_00340_[Bacillus_cereus]	CPTF_Fe	101710.4333	50432.30996	49.58420518
UIJ66839.1	hypothetical_protein_LW858_00340_[Bacillus_cereus]	CPTF_Mn	147999.3333	40110.81178	27.10202193
UIJ66839.1	hypothetical_protein_LW858_00340_[Bacillus_cereus]	CPTF_Ni	62390.36667	2716.643903	4.354268211
UIJ66839.1	hypothetical_protein_LW858_00340_[Bacillus_cereus]	CPTF_U	78131.33333	67708.67762	86.66008211
UIJ66839.1	hypothetical_protein_LW858_00340_[Bacillus_cereus]	CPTF_metals_mix	366584.5667	65258.27553	17.80169747
UIJ66839.1	hypothetical_protein_LW858_00340_[Bacillus_cereus]	CPTF_zcontrol	42253.2	37104.04743	87.81357963
UIJ66840.1	class_I_fumarate_hydratase_[Bacillus_cereus]	CPTF_Al	1668514.767	333964.9282	20.01570108
UIJ66840.1	class_I_fumarate_hydratase_[Bacillus_cereus]	CPTF_Cd	1680903.067	47571.46654	2.830113615
UIJ66840.1	class_I_fumarate_hydratase_[Bacillus_cereus]	CPTF_Co	1539262.4	708110.1669	46.00321342
UIJ66840.1	class_I_fumarate_hydratase_[Bacillus_cereus]	CPTF_Cu	1043152.967	162583.0942	15.58573856
UIJ66840.1	class_I_fumarate_hydratase_[Bacillus_cereus]	CPTF_Fe	1694934.567	283891.4661	16.74940565
UIJ66840.1	class_I_fumarate_hydratase_[Bacillus_cereus]	CPTF_Mn	1786409.9	429374.8956	24.03563122
UIJ66840.1	class_I_fumarate_hydratase_[Bacillus_cereus]	CPTF_Ni	1527509.733	242596.9818	15.8818616
UIJ66840.1	class_I_fumarate_hydratase_[Bacillus_cereus]	CPTF_U	1156590.6	189687.7995	16.40060013
UIJ66840.1	class_I_fumarate_hydratase_[Bacillus_cereus]	CPTF_metals_mix	962333.3333	173353.5916	18.01388205
UIJ66840.1	class_I_fumarate_hydratase_[Bacillus_cereus]	CPTF_zcontrol	1606733	362382.1375	22.55397365
UIJ66843.1	23S_rRNA_(uracil(1939)-C(5))-methyltransferase_RlmD_[Bacillus_cereus]	CPTF_Al	173523.3333	47221.08421	27.21310345
UIJ66843.1	23S_rRNA_(uracil(1939)-C(5))-methyltransferase_RlmD_[Bacillus_cereus]	CPTF_Cd	95943.76667	99383.23609	103.5848805
UIJ66843.1	23S_rRNA_(uracil(1939)-C(5))-methyltransferase_RlmD_[Bacillus_cereus]	CPTF_Co	218713.6667	61187.11827	27.97590073
UIJ66843.1	23S_rRNA_(uracil(1939)-C(5))-methyltransferase_RlmD_[Bacillus_cereus]	CPTF_Cu	141434.6667	16643.44941	11.76758839
UIJ66843.1	23S_rRNA_(uracil(1939)-C(5))-methyltransferase_RlmD_[Bacillus_cereus]	CPTF_Fe	206846.3333	114688.8954	55.44642419
UIJ66843.1	23S_rRNA_(uracil(1939)-C(5))-methyltransferase_RlmD_[Bacillus_cereus]	CPTF_Mn	186041.6	72328.88524	38.87780219
UIJ66843.1	23S_rRNA_(uracil(1939)-C(5))-methyltransferase_RlmD_[Bacillus_cereus]	CPTF_Ni	125767.8667	112243.4699	89.24654035
UIJ66843.1	23S_rRNA_(uracil(1939)-C(5))-methyltransferase_RlmD_[Bacillus_cereus]	CPTF_U	141339	32974.01779	23.32973757
UIJ66843.1	23S_rRNA_(uracil(1939)-C(5))-methyltransferase_RlmD_[Bacillus_cereus]	CPTF_metals_mix	31782.46667	55048.84706	173.2050808
UIJ66843.1	23S_rRNA_(uracil(1939)-C(5))-methyltransferase_RlmD_[Bacillus_cereus]	CPTF_zcontrol	227498.7	27694.0776	12.17329048
UIJ66862.1	formate_C-acetyltransferase_[Bacillus_cereus]	CPTF_Al	113440149.6	7625850.119	6.722355485
UIJ66862.1	formate_C-acetyltransferase_[Bacillus_cereus]	CPTF_Cd	106611551.4	12778657.97	11.98618518
UIJ66862.1	formate_C-acetyltransferase_[Bacillus_cereus]	CPTF_Co	104659521.7	1948157.041	1.861423603
UIJ66862.1	formate_C-acetyltransferase_[Bacillus_cereus]	CPTF_Cu	116170595	7701341.773	6.629338322
UIJ66862.1	formate_C-acetyltransferase_[Bacillus_cereus]	CPTF_Fe	104325119.1	10390670.62	9.959893365
UIJ66862.1	formate_C-acetyltransferase_[Bacillus_cereus]	CPTF_Mn	107436317.1	14301968.05	13.31204237
UIJ66862.1	formate_C-acetyltransferase_[Bacillus_cereus]	CPTF_Ni	97422180.3	8017569.245	8.229716498
UIJ66862.1	formate_C-acetyltransferase_[Bacillus_cereus]	CPTF_U	96714886.43	4959170.006	5.127618083
UIJ66862.1	formate_C-acetyltransferase_[Bacillus_cereus]	CPTF_metals_mix	152629521.5	4551399.48	2.981991581
UIJ66862.1	formate_C-acetyltransferase_[Bacillus_cereus]	CPTF_zcontrol	96909369.03	4948085.782	5.10589
UIJ66863.1	pyruvate_formate_lyase-activating_protein_[Bacillus_cereus]	CPTF_Al	5762473	469777.8672	8.152365612
UIJ66863.1	pyruvate_formate_lyase-activating_protein_[Bacillus_cereus]	CPTF_Cd	4330391	1177104.14	27.18239854
UIJ66863.1	pyruvate_formate_lyase-activating_protein_[Bacillus_cereus]	CPTF_Co	5336550.667	726177.329	13.60761612
UIJ66863.1	pyruvate_formate_lyase-activating_protein_[Bacillus_cereus]	CPTF_Cu	5413249.667	1043192.348	19.27109245
UIJ66863.1	pyruvate_formate_lyase-activating_protein_[Bacillus_cereus]	CPTF_Fe	5524215	596712.6225	10.80176319
UIJ66863.1	pyruvate_formate_lyase-activating_protein_[Bacillus_cereus]	CPTF_Mn	5831491	442614.8206	7.590079803

UIJ66863.1	pyruvate_formate_lyase-activating_protein_[Bacillus_cereus]	CPTF_Ni	5039630.667	738806.389	14.65993121
UIJ66863.1	pyruvate_formate_lyase-activating_protein_[Bacillus_cereus]	CPTF_U	5363985	684031.2059	12.75229528
UIJ66863.1	pyruvate_formate_lyase-activating_protein_[Bacillus_cereus]	CPTF_metals_mix	3304647.867	173911.6741	5.262638597
UIJ66863.1	pyruvate_formate_lyase-activating_protein_[Bacillus_cereus]	CPTF_zcontrol	5611297	1009196.077	17.98507683
UIJ66880.1	DUF402_domain-containing_protein_[Bacillus_cereus]	CPTF_Al	180308.6667	13347.74233	7.402718115
UIJ66880.1	DUF402_domain-containing_protein_[Bacillus_cereus]	CPTF_Cd	192564.3333	18401.50008	9.556027207
UIJ66880.1	DUF402_domain-containing_protein_[Bacillus_cereus]	CPTF_Co	341639.7	157216.0907	46.01809765
UIJ66880.1	DUF402_domain-containing_protein_[Bacillus_cereus]	CPTF_Cu	431523.7	255151.9691	59.12814733
UIJ66880.1	DUF402_domain-containing_protein_[Bacillus_cereus]	CPTF_Fe	306315.3	147596.2642	48.18442442
UIJ66880.1	DUF402_domain-containing_protein_[Bacillus_cereus]	CPTF_Mn	202503.3333	116630.7716	57.59449471
UIJ66880.1	DUF402_domain-containing_protein_[Bacillus_cereus]	CPTF_Ni	262030.6667	141396.0863	53.96165576
UIJ66880.1	DUF402_domain-containing_protein_[Bacillus_cereus]	CPTF_U	238962.8333	57549.14777	24.08288643
UIJ66880.1	DUF402_domain-containing_protein_[Bacillus_cereus]	CPTF_metals_mix	708430.8	81930.63035	11.56508587
UIJ66880.1	DUF402_domain-containing_protein_[Bacillus_cereus]	CPTF_zcontrol	394848.8	87910.76825	22.26441317
UIJ66883.1	glutamate_synthase_[Bacillus_cereus]	CPTF_Al	7454929.3	321415.3703	4.311447599
UIJ66883.1	glutamate_synthase_[Bacillus_cereus]	CPTF_Cd	7159049.4	421300.1814	5.884862052
UIJ66883.1	glutamate_synthase_[Bacillus_cereus]	CPTF_Co	6721135.9	732878.0769	10.90408062
UIJ66883.1	glutamate_synthase_[Bacillus_cereus]	CPTF_Cu	6732445.107	994494.9076	14.77167495
UIJ66883.1	glutamate_synthase_[Bacillus_cereus]	CPTF_Fe	7569778.033	922988.2745	12.19306921
UIJ66883.1	glutamate_synthase_[Bacillus_cereus]	CPTF_Mn	6688582.5	935574.8576	13.98764024
UIJ66883.1	glutamate_synthase_[Bacillus_cereus]	CPTF_Ni	6973779.667	188168.0937	2.698222523
UIJ66883.1	glutamate_synthase_[Bacillus_cereus]	CPTF_U	5450208.5	1764943.01	32.38303653
UIJ66883.1	glutamate_synthase_[Bacillus_cereus]	CPTF_metals_mix	2958781.6	473657.9736	16.00854803
UIJ66883.1	glutamate_synthase_[Bacillus_cereus]	CPTF_zcontrol	6016374.967	952370.0687	15.82963286
UIJ66884.1	glutamate-1-semialdehyde-2,1-aminomutase_[Bacillus_cereus]	CPTF_Al	2193829.7	152975.1262	6.972971795
UIJ66884.1	glutamate-1-semialdehyde-2,1-aminomutase_[Bacillus_cereus]	CPTF_Cd	2678835.433	122204.3099	4.561844614
UIJ66884.1	glutamate-1-semialdehyde-2,1-aminomutase_[Bacillus_cereus]	CPTF_Co	2289516.8	303889.6699	13.27309194
UIJ66884.1	glutamate-1-semialdehyde-2,1-aminomutase_[Bacillus_cereus]	CPTF_Cu	2464419.833	193096.6428	7.835379354
UIJ66884.1	glutamate-1-semialdehyde-2,1-aminomutase_[Bacillus_cereus]	CPTF_Fe	2397821.867	49847.31231	2.078858025
UIJ66884.1	glutamate-1-semialdehyde-2,1-aminomutase_[Bacillus_cereus]	CPTF_Mn	2127361.833	73266.44527	3.444004876
UIJ66884.1	glutamate-1-semialdehyde-2,1-aminomutase_[Bacillus_cereus]	CPTF_Ni	1840076.333	339362.555	18.44285201
UIJ66884.1	glutamate-1-semialdehyde-2,1-aminomutase_[Bacillus_cereus]	CPTF_U	2205214.433	244662.896	11.094744
UIJ66884.1	glutamate-1-semialdehyde-2,1-aminomutase_[Bacillus_cereus]	CPTF_metals_mix	3939323.567	212071.1115	5.383439768
UIJ66884.1	glutamate-1-semialdehyde-2,1-aminomutase_[Bacillus_cereus]	CPTF_zcontrol	1995505.333	150099.4362	7.521875973
UIJ66889.1	thioredoxin-dependent_thiol_peroxidase_[Bacillus_cereus]	CPTF_Al	237160.1333	93688.89826	39.50448878
UIJ66889.1	thioredoxin-dependent_thiol_peroxidase_[Bacillus_cereus]	CPTF_Cd	264805.1667	43027.03301	16.2485625
UIJ66889.1	thioredoxin-dependent_thiol_peroxidase_[Bacillus_cereus]	CPTF_Co	309784.3667	37959.58166	12.25354981
UIJ66889.1	thioredoxin-dependent_thiol_peroxidase_[Bacillus_cereus]	CPTF_Cu	267529.9333	70046.83815	26.18280403
UIJ66889.1	thioredoxin-dependent_thiol_peroxidase_[Bacillus_cereus]	CPTF_Fe	172905.9	45513.71496	26.32282355
UIJ66889.1	thioredoxin-dependent_thiol_peroxidase_[Bacillus_cereus]	CPTF_Mn	196446.7	124487.0065	63.36935489
UIJ66889.1	thioredoxin-dependent_thiol_peroxidase_[Bacillus_cereus]	CPTF_Ni	169354.8667	146674.7042	86.60790629
UIJ66889.1	thioredoxin-dependent_thiol_peroxidase_[Bacillus_cereus]	CPTF_U	37573	65078.34499	173.2050808
UIJ66889.1	thioredoxin-dependent_thiol_peroxidase_[Bacillus_cereus]	CPTF_metals_mix	382127.1	4014.351667	1.050527866
UIJ66889.1	thioredoxin-dependent_thiol_peroxidase_[Bacillus_cereus]	CPTF_zcontrol	91232.66667	79209.28938	86.82119275
UIJ66891.1	DUF3884_family_protein_[Bacillus_cereus]	CPTF_Al	189380	165788.4996	87.54277092
UIJ66891.1	DUF3884_family_protein_[Bacillus_cereus]	CPTF_Cd	207268.6667	183606.0699	88.58361124
UIJ66891.1	DUF3884_family_protein_[Bacillus_cereus]	CPTF_Co	185251.6667	170788.7205	92.19281186
UIJ66891.1	DUF3884_family_protein_[Bacillus_cereus]	CPTF_Cu	393923	12060.56864	3.061656375
UIJ66891.1	DUF3884_family_protein_[Bacillus_cereus]	CPTF_Fe	214829	212004.3365	98.68515726
UIJ66891.1	DUF3884_family_protein_[Bacillus_cereus]	CPTF_Mn	131636.6667	228001.3948	173.2050808
UIJ66891.1	DUF3884_family_protein_[Bacillus_cereus]	CPTF_Ni	920600.6667	137288.1362	14.9128869
UIJ66891.1	DUF3884_family_protein_[Bacillus_cereus]	CPTF_U	558893.3333	28488.02103	5.09721969
UIJ66891.1	DUF3884_family_protein_[Bacillus_cereus]	CPTF_metals_mix	0	0	0
UIJ66891.1	DUF3884_family_protein_[Bacillus_cereus]	CPTF_zcontrol	152539	264205.2981	173.2050808
UIJ66892.1	YebC/PmpR_family_DNA-binding_transcriptional_regulator_[Bacillus_cereus]	CPTF_Al	2297539.333	151068.2994	6.575221465
UIJ66892.1	YebC/PmpR_family_DNA-binding_transcriptional_regulator_[Bacillus_cereus]	CPTF_Cd	2302542	462546.4102	20.08851131
UIJ66892.1	YebC/PmpR_family_DNA-binding_transcriptional_regulator_[Bacillus_cereus]	CPTF_Co	2398251.333	79330.11923	3.307831758
UIJ66892.1	YebC/PmpR_family_DNA-binding_transcriptional_regulator_[Bacillus_cereus]	CPTF_Cu	2034976.333	258405.7402	12.69821845
UIJ66892.1	YebC/PmpR_family_DNA-binding_transcriptional_regulator_[Bacillus_cereus]	CPTF_Fe	1971160.333	217358.2445	11.02691855
UIJ66892.1	YebC/PmpR_family_DNA-binding_transcriptional_regulator_[Bacillus_cereus]	CPTF_Mn	2431017	286116.93	11.76943353
UIJ66892.1	YebC/PmpR_family_DNA-binding_transcriptional_regulator_[Bacillus_cereus]	CPTF_Ni	2048085.333	265133.0245	12.94540907

UIJ66892.1	YebC/PmpR_family_DNA-binding_transcriptional_regulator_[Bacillus_cereus]	CPTF_U	1672525.767	411983.0659	24.63238978
UIJ66892.1	YebC/PmpR_family_DNA-binding_transcriptional_regulator_[Bacillus_cereus]	CPTF_metals_mix	2300213.667	125317.9509	5.448100439
UIJ66892.1	YebC/PmpR_family_DNA-binding_transcriptional_regulator_[Bacillus_cereus]	CPTF_zcontrol	2365865.667	104776.0569	4.428656214
UIJ66895.1	DsbA_family_protein_[Bacillus_cereus]	CPTF_Al	399693.3333	109618.5617	27.42566677
UIJ66895.1	DsbA_family_protein_[Bacillus_cereus]	CPTF_Cd	588706.6667	33298.40123	5.656195711
UIJ66895.1	DsbA_family_protein_[Bacillus_cereus]	CPTF_Co	368514.6667	65923.61617	17.88900745
UIJ66895.1	DsbA_family_protein_[Bacillus_cereus]	CPTF_Cu	282378.3333	104836.3874	37.12621509
UIJ66895.1	DsbA_family_protein_[Bacillus_cereus]	CPTF_Fe	457736.6667	78138.31291	17.07058197
UIJ66895.1	DsbA_family_protein_[Bacillus_cereus]	CPTF_Mn	190838.1667	109299.681	57.27349143
UIJ66895.1	DsbA_family_protein_[Bacillus_cereus]	CPTF_Ni	0	0	0
UIJ66895.1	DsbA_family_protein_[Bacillus_cereus]	CPTF_U	187200	163958.5168	87.58467777
UIJ66895.1	DsbA_family_protein_[Bacillus_cereus]	CPTF_metals_mix	545518.3333	27477.81815	5.037010944
UIJ66895.1	DsbA_family_protein_[Bacillus_cereus]	CPTF_zcontrol	180638.3333	161098.2153	89.18274008
UIJ66898.1	tRNA_(uridine(34)/cytosine(34)/5-carboxymethylaminomethyluridine(34)-2'-O)-methyltransferase_TrmL_[Bacillus_cereus]	CPTF_Al	0	0	0
UIJ66898.1	tRNA_(uridine(34)/cytosine(34)/5-carboxymethylaminomethyluridine(34)-2'-O)-methyltransferase_TrmL_[Bacillus_cereus]	CPTF_Cd	0	0	0
UIJ66898.1	tRNA_(uridine(34)/cytosine(34)/5-carboxymethylaminomethyluridine(34)-2'-O)-methyltransferase_TrmL_[Bacillus_cereus]	CPTF_Co	0	0	0
UIJ66898.1	tRNA_(uridine(34)/cytosine(34)/5-carboxymethylaminomethyluridine(34)-2'-O)-methyltransferase_TrmL_[Bacillus_cereus]	CPTF_Cu	0	0	0
UIJ66898.1	tRNA_(uridine(34)/cytosine(34)/5-carboxymethylaminomethyluridine(34)-2'-O)-methyltransferase_TrmL_[Bacillus_cereus]	CPTF_Fe	0	0	0
UIJ66898.1	tRNA_(uridine(34)/cytosine(34)/5-carboxymethylaminomethyluridine(34)-2'-O)-methyltransferase_TrmL_[Bacillus_cereus]	CPTF_Mn	0	0	0
UIJ66898.1	tRNA_(uridine(34)/cytosine(34)/5-carboxymethylaminomethyluridine(34)-2'-O)-methyltransferase_TrmL_[Bacillus_cereus]	CPTF_Ni	0	0	0
UIJ66898.1	tRNA_(uridine(34)/cytosine(34)/5-carboxymethylaminomethyluridine(34)-2'-O)-methyltransferase_TrmL_[Bacillus_cereus]	CPTF_U	0	0	0
UIJ66898.1	tRNA_(uridine(34)/cytosine(34)/5-carboxymethylaminomethyluridine(34)-2'-O)-methyltransferase_TrmL_[Bacillus_cereus]	CPTF_metals_mix	51554.66667	89295.30203	173.2050808
UIJ66898.1	tRNA_(uridine(34)/cytosine(34)/5-carboxymethylaminomethyluridine(34)-2'-O)-methyltransferase_TrmL_[Bacillus_cereus]	CPTF_zcontrol	0	0	0
UIJ66906.1	flotillin_family_protein_[Bacillus_cereus]	CPTF_Al	1650657.433	34318.14528	2.079059203
UIJ66906.1	flotillin_family_protein_[Bacillus_cereus]	CPTF_Cd	1811235.333	106378.0977	5.873234457
UIJ66906.1	flotillin_family_protein_[Bacillus_cereus]	CPTF_Co	1465695.533	105293.0348	7.183827229
UIJ66906.1	flotillin_family_protein_[Bacillus_cereus]	CPTF_Cu	1131236.867	692711.3661	61.23486482
UIJ66906.1	flotillin_family_protein_[Bacillus_cereus]	CPTF_Fe	1569562.767	134406.8616	8.563331421
UIJ66906.1	flotillin_family_protein_[Bacillus_cereus]	CPTF_Mn	1651467.333	446406.059	27.0308743
UIJ66906.1	flotillin_family_protein_[Bacillus_cereus]	CPTF_Ni	915851.2333	268683.1027	29.33698104
UIJ66906.1	flotillin_family_protein_[Bacillus_cereus]	CPTF_U	803506.9333	383309.3917	47.7045531
UIJ66906.1	flotillin_family_protein_[Bacillus_cereus]	CPTF_metals_mix	1170350.233	223607.0659	19.10599576
UIJ66906.1	flotillin_family_protein_[Bacillus_cereus]	CPTF_zcontrol	923900.9	643650.3559	69.66660124
UIJ66931.1	GNAT_family_N-acetyltransferase_[Bacillus_cereus]	CPTF_Al	4656.7	8065.640996	173.2050808
UIJ66931.1	GNAT_family_N-acetyltransferase_[Bacillus_cereus]	CPTF_Cd	37408.26667	35408.44416	94.65406263
UIJ66931.1	GNAT_family_N-acetyltransferase_[Bacillus_cereus]	CPTF_Co	52297.7	23689.5057	45.29741403
UIJ66931.1	GNAT_family_N-acetyltransferase_[Bacillus_cereus]	CPTF_Cu	49643.8	24822.32254	50.00085115
UIJ66931.1	GNAT_family_N-acetyltransferase_[Bacillus_cereus]	CPTF_Fe	22151.76667	19204.9544	86.69716818
UIJ66931.1	GNAT_family_N-acetyltransferase_[Bacillus_cereus]	CPTF_Mn	30073.7	26235.21061	87.23639129
UIJ66931.1	GNAT_family_N-acetyltransferase_[Bacillus_cereus]	CPTF_Ni	15339.53333	26568.8511	173.2050808
UIJ66931.1	GNAT_family_N-acetyltransferase_[Bacillus_cereus]	CPTF_U	0	0	0
UIJ66931.1	GNAT_family_N-acetyltransferase_[Bacillus_cereus]	CPTF_metals_mix	600366	66187.4386	11.02451481
UIJ66931.1	GNAT_family_N-acetyltransferase_[Bacillus_cereus]	CPTF_zcontrol	0	0	0
UIJ66936.1	formate_dehydrogenase_subunit_alpha_[Bacillus_cereus]	CPTF_Al	0	0	0
UIJ66936.1	formate_dehydrogenase_subunit_alpha_[Bacillus_cereus]	CPTF_Cd	42683.66667	73930.27932	173.2050808
UIJ66936.1	formate_dehydrogenase_subunit_alpha_[Bacillus_cereus]	CPTF_Co	67718.66667	117292.1713	173.2050808
UIJ66936.1	formate_dehydrogenase_subunit_alpha_[Bacillus_cereus]	CPTF_Cu	94386.96667	82365.88251	87.26404229
UIJ66936.1	formate_dehydrogenase_subunit_alpha_[Bacillus_cereus]	CPTF_Fe	108415.8667	133431.986	123.0742235
UIJ66936.1	formate_dehydrogenase_subunit_alpha_[Bacillus_cereus]	CPTF_Mn	18026.36667	31222.58294	173.2050808
UIJ66936.1	formate_dehydrogenase_subunit_alpha_[Bacillus_cereus]	CPTF_Ni	0	0	0
UIJ66936.1	formate_dehydrogenase_subunit_alpha_[Bacillus_cereus]	CPTF_U	11648.76667	20176.25571	173.2050808
UIJ66936.1	formate_dehydrogenase_subunit_alpha_[Bacillus_cereus]	CPTF_metals_mix	12111.56667	20977.84883	173.2050808
UIJ66936.1	formate_dehydrogenase_subunit_alpha_[Bacillus_cereus]	CPTF_zcontrol	119109.3333	103708.6344	87.07011571
UIJ66938.1	alanine_dehydrogenase_[Bacillus_cereus]	CPTF_Al	212303	30947.7024	14.57713852
UIJ66938.1	alanine_dehydrogenase_[Bacillus_cereus]	CPTF_Cd	289658	9773.034994	3.37399105
UIJ66938.1	alanine_dehydrogenase_[Bacillus_cereus]	CPTF_Co	144162	27791.18312	19.27774526
UIJ66938.1	alanine_dehydrogenase_[Bacillus_cereus]	CPTF_Cu	191842	4781.972815	2.492662094
UIJ66938.1	alanine_dehydrogenase_[Bacillus_cereus]	CPTF_Fe	178755.3333	82081.32982	45.91825502
UIJ66938.1	alanine_dehydrogenase_[Bacillus_cereus]	CPTF_Mn	150045.1333	51450.86166	34.29025688
UIJ66938.1	alanine_dehydrogenase_[Bacillus_cereus]	CPTF_Ni	143873.3333	51708.52044	35.94030891
UIJ66938.1	alanine_dehydrogenase_[Bacillus_cereus]	CPTF_U	239036.3333	87740.67866	36.7060009

UIJ66938.1	alanine_dehydrogenase_[Bacillus_cereus]	CPTF_metals_mix	346365.3	77021.27241	22.23700596
UIJ66938.1	alanine_dehydrogenase_[Bacillus_cereus]	CPTF_zcontrol	102140.4667	38141.73064	37.3424284
UIJ66941.1	cadmium-translocating_P-type_ATPase_[Bacillus_cereus]	CPTF_Al	0	0	0
UIJ66941.1	cadmium-translocating_P-type_ATPase_[Bacillus_cereus]	CPTF_Cd	62068.66667	58104.7804	93.61370804
UIJ66941.1	cadmium-translocating_P-type_ATPase_[Bacillus_cereus]	CPTF_Co	0	0	0
UIJ66941.1	cadmium-translocating_P-type_ATPase_[Bacillus_cereus]	CPTF_Cu	9718.5	16832.93577	173.2050808
UIJ66941.1	cadmium-translocating_P-type_ATPase_[Bacillus_cereus]	CPTF_Fe	0	0	0
UIJ66941.1	cadmium-translocating_P-type_ATPase_[Bacillus_cereus]	CPTF_Mn	0	0	0
UIJ66941.1	cadmium-translocating_P-type_ATPase_[Bacillus_cereus]	CPTF_Ni	51079.4	49179.49919	96.28049505
UIJ66941.1	cadmium-translocating_P-type_ATPase_[Bacillus_cereus]	CPTF_U	0	0	0
UIJ66941.1	cadmium-translocating_P-type_ATPase_[Bacillus_cereus]	CPTF_metals_mix	1412729.633	350475.949	24.80842341
UIJ66941.1	cadmium-translocating_P-type_ATPase_[Bacillus_cereus]	CPTF_zcontrol	0	0	0
UIJ66942.1	nicotinate_phosphoribosyltransferase_[Bacillus_cereus]	CPTF_Al	563532.8667	165240.2979	29.32221129
UIJ66942.1	nicotinate_phosphoribosyltransferase_[Bacillus_cereus]	CPTF_Cd	1037282.633	139992.8507	13.49611438
UIJ66942.1	nicotinate_phosphoribosyltransferase_[Bacillus_cereus]	CPTF_Co	448816.6667	267154.2385	59.52413498
UIJ66942.1	nicotinate_phosphoribosyltransferase_[Bacillus_cereus]	CPTF_Cu	501542.2667	94586.97196	18.85922249
UIJ66942.1	nicotinate_phosphoribosyltransferase_[Bacillus_cereus]	CPTF_Fe	558595.1333	181301.3492	32.45666465
UIJ66942.1	nicotinate_phosphoribosyltransferase_[Bacillus_cereus]	CPTF_Mn	302180.5333	46528.60111	15.39761698
UIJ66942.1	nicotinate_phosphoribosyltransferase_[Bacillus_cereus]	CPTF_Ni	352350.7333	108079.4139	30.6738155
UIJ66942.1	nicotinate_phosphoribosyltransferase_[Bacillus_cereus]	CPTF_U	245360.1667	151487.1904	61.74074318
UIJ66942.1	nicotinate_phosphoribosyltransferase_[Bacillus_cereus]	CPTF_metals_mix	1021395	94010.09949	9.204088476
UIJ66942.1	nicotinate_phosphoribosyltransferase_[Bacillus_cereus]	CPTF_zcontrol	296881.4	134891.9547	45.43631049
UIJ66943.1	tetratricopeptide_repeat_protein_[Bacillus_cereus]	CPTF_Al	1026374.733	66791.76662	6.507541977
UIJ66943.1	tetratricopeptide_repeat_protein_[Bacillus_cereus]	CPTF_Cd	927373.3	70252.01427	7.575375986
UIJ66943.1	tetratricopeptide_repeat_protein_[Bacillus_cereus]	CPTF_Co	582819.2333	504751.2915	86.60511915
UIJ66943.1	tetratricopeptide_repeat_protein_[Bacillus_cereus]	CPTF_Cu	356611.8667	564182.483	158.2063122
UIJ66943.1	tetratricopeptide_repeat_protein_[Bacillus_cereus]	CPTF_Fe	977898.0667	119834.7154	12.25431561
UIJ66943.1	tetratricopeptide_repeat_protein_[Bacillus_cereus]	CPTF_Mn	766213.2667	333485.9972	43.52391322
UIJ66943.1	tetratricopeptide_repeat_protein_[Bacillus_cereus]	CPTF_Ni	920522.8667	37902.75599	4.117524655
UIJ66943.1	tetratricopeptide_repeat_protein_[Bacillus_cereus]	CPTF_U	600252.3333	547851.0315	91.2701211
UIJ66943.1	tetratricopeptide_repeat_protein_[Bacillus_cereus]	CPTF_metals_mix	539703.3333	39336.01444	7.288451268
UIJ66943.1	tetratricopeptide_repeat_protein_[Bacillus_cereus]	CPTF_zcontrol	913414.3667	36977.35249	4.048256065
UIJ66955.1	metalloregulator_ArsR/SmtB_family_transcription_factor_[Bacillus_cereus]	CPTF_Al	26329.36667	45603.8008	173.2050808
UIJ66955.1	metalloregulator_ArsR/SmtB_family_transcription_factor_[Bacillus_cereus]	CPTF_Cd	177592	224020.2235	126.1431954
UIJ66955.1	metalloregulator_ArsR/SmtB_family_transcription_factor_[Bacillus_cereus]	CPTF_Co	62229.93333	53908.39946	86.62776348
UIJ66955.1	metalloregulator_ArsR/SmtB_family_transcription_factor_[Bacillus_cereus]	CPTF_Cu	0	0	0
UIJ66955.1	metalloregulator_ArsR/SmtB_family_transcription_factor_[Bacillus_cereus]	CPTF_Fe	20785.5	36001.54206	173.2050808
UIJ66955.1	metalloregulator_ArsR/SmtB_family_transcription_factor_[Bacillus_cereus]	CPTF_Mn	17544.1	30387.27257	173.2050808
UIJ66955.1	metalloregulator_ArsR/SmtB_family_transcription_factor_[Bacillus_cereus]	CPTF_Ni	0	0	0
UIJ66955.1	metalloregulator_ArsR/SmtB_family_transcription_factor_[Bacillus_cereus]	CPTF_U	0	0	0
UIJ66955.1	metalloregulator_ArsR/SmtB_family_transcription_factor_[Bacillus_cereus]	CPTF_metals_mix	459950.5333	430788.043	93.65964636
UIJ66955.1	metalloregulator_ArsR/SmtB_family_transcription_factor_[Bacillus_cereus]	CPTF_zcontrol	0	0	0
UIJ66957.1	peptide_MFS_transporter_[Bacillus_cereus]	CPTF_Al	93040.33333	85736.34717	92.14965607
UIJ66957.1	peptide_MFS_transporter_[Bacillus_cereus]	CPTF_Cd	57646.9	69371.46374	120.338585
UIJ66957.1	peptide_MFS_transporter_[Bacillus_cereus]	CPTF_Co	125682.4	118095.237	93.96322554
UIJ66957.1	peptide_MFS_transporter_[Bacillus_cereus]	CPTF_Cu	221160.8333	126699.6348	57.28845968
UIJ66957.1	peptide_MFS_transporter_[Bacillus_cereus]	CPTF_Fe	89759	80102.24791	89.2414665
UIJ66957.1	peptide_MFS_transporter_[Bacillus_cereus]	CPTF_Mn	33482	57992.52514	173.2050808
UIJ66957.1	peptide_MFS_transporter_[Bacillus_cereus]	CPTF_Ni	117576.5667	51895.15757	44.13733028
UIJ66957.1	peptide_MFS_transporter_[Bacillus_cereus]	CPTF_U	63790.63333	56190.63146	88.08602223
UIJ66957.1	peptide_MFS_transporter_[Bacillus_cereus]	CPTF_metals_mix	452134.8	59909.80649	13.25043029
UIJ66957.1	peptide_MFS_transporter_[Bacillus_cereus]	CPTF_zcontrol	0	0	0
UIJ66964.1	glycine_C-acetyltransferase_[Bacillus_cereus]	CPTF_Al	8718480.567	242996.6074	2.787143994
UIJ66964.1	glycine_C-acetyltransferase_[Bacillus_cereus]	CPTF_Cd	8459969.533	99820.33257	1.179913618
UIJ66964.1	glycine_C-acetyltransferase_[Bacillus_cereus]	CPTF_Co	8105672.4	488276.1619	6.023882262
UIJ66964.1	glycine_C-acetyltransferase_[Bacillus_cereus]	CPTF_Cu	7727279.933	434866.2209	5.627675258
UIJ66964.1	glycine_C-acetyltransferase_[Bacillus_cereus]	CPTF_Fe	7546359.467	218355.3736	2.893519379
UIJ66964.1	glycine_C-acetyltransferase_[Bacillus_cereus]	CPTF_Mn	7821257.767	377164.9791	4.822305956
UIJ66964.1	glycine_C-acetyltransferase_[Bacillus_cereus]	CPTF_Ni	7634274.633	396103.7416	5.18849217
UIJ66964.1	glycine_C-acetyltransferase_[Bacillus_cereus]	CPTF_U	7110824	180667.5381	2.540739837
UIJ66964.1	glycine_C-acetyltransferase_[Bacillus_cereus]	CPTF_metals_mix	7137357.1	940677.1881	13.17962903

UIJ66964.1	glycine_C-acetyltransferase_[Bacillus_cereus]	CPTF_zcontrol	8751472.867	438175.6981	5.006879468
UIJ66965.1	L-threonine_3-dehydrogenase_[Bacillus_cereus]	CPTF_Al	147759.1333	193729.1196	131.1114347
UIJ66965.1	L-threonine_3-dehydrogenase_[Bacillus_cereus]	CPTF_Cd	198147.3667	172327.6282	86.96942639
UIJ66965.1	L-threonine_3-dehydrogenase_[Bacillus_cereus]	CPTF_Co	124058.8	35266.1362	28.42695254
UIJ66965.1	L-threonine_3-dehydrogenase_[Bacillus_cereus]	CPTF_Cu	152409.4	220355.5433	144.5813338
UIJ66965.1	L-threonine_3-dehydrogenase_[Bacillus_cereus]	CPTF_Fe	75255.03333	41554.29827	55.2179654
UIJ66965.1	L-threonine_3-dehydrogenase_[Bacillus_cereus]	CPTF_Mn	112902.3	137002.8311	121.3463597
UIJ66965.1	L-threonine_3-dehydrogenase_[Bacillus_cereus]	CPTF_Ni	145398.5667	125992.1097	86.65292419
UIJ66965.1	L-threonine_3-dehydrogenase_[Bacillus_cereus]	CPTF_U	109602.2667	189836.6945	173.2050808
UIJ66965.1	L-threonine_3-dehydrogenase_[Bacillus_cereus]	CPTF_metals_mix	213646.2333	166744.9818	78.04723687
UIJ66965.1	L-threonine_3-dehydrogenase_[Bacillus_cereus]	CPTF_zcontrol	375747.0333	170723.0432	45.43563304
UIJ66966.1	NUDIX_hydrolase_[Bacillus_cereus]	CPTF_Al	1280406.533	89886.83488	7.020179337
UIJ66966.1	NUDIX_hydrolase_[Bacillus_cereus]	CPTF_Cd	1174749.867	116047.1557	9.878456601
UIJ66966.1	NUDIX_hydrolase_[Bacillus_cereus]	CPTF_Co	1276554.967	29669.6557	2.324197272
UIJ66966.1	NUDIX_hydrolase_[Bacillus_cereus]	CPTF_Cu	1177403.6	117819.3818	10.00671153
UIJ66966.1	NUDIX_hydrolase_[Bacillus_cereus]	CPTF_Fe	1190856.633	17261.49936	1.449502726
UIJ66966.1	NUDIX_hydrolase_[Bacillus_cereus]	CPTF_Mn	1199829.1	164539.8238	13.71360503
UIJ66966.1	NUDIX_hydrolase_[Bacillus_cereus]	CPTF_Ni	1259485.7	58677.75093	4.65886599
UIJ66966.1	NUDIX_hydrolase_[Bacillus_cereus]	CPTF_U	1147041.033	68510.23611	5.972779884
UIJ66966.1	NUDIX_hydrolase_[Bacillus_cereus]	CPTF_metals_mix	883157.8667	28030.24197	3.173865402
UIJ66966.1	NUDIX_hydrolase_[Bacillus_cereus]	CPTF_zcontrol	1213055.3	89584.12537	7.384999297
UIJ66975.1	alpha,alpha-phosphotrehalase_[Bacillus_cereus]	CPTF_Al	80337.76667	56792.8196	70.69255464
UIJ66975.1	alpha,alpha-phosphotrehalase_[Bacillus_cereus]	CPTF_Cd	132412.2	84376.55466	63.72264388
UIJ66975.1	alpha,alpha-phosphotrehalase_[Bacillus_cereus]	CPTF_Co	142740.5667	68283.11384	47.83721645
UIJ66975.1	alpha,alpha-phosphotrehalase_[Bacillus_cereus]	CPTF_Cu	150220.4333	64256.89348	42.77506865
UIJ66975.1	alpha,alpha-phosphotrehalase_[Bacillus_cereus]	CPTF_Fe	91780.03333	113275.5809	123.4207232
UIJ66975.1	alpha,alpha-phosphotrehalase_[Bacillus_cereus]	CPTF_Mn	20871.8	18205.70498	87.22632923
UIJ66975.1	alpha,alpha-phosphotrehalase_[Bacillus_cereus]	CPTF_Ni	88582.26667	78219.35855	88.30137396
UIJ66975.1	alpha,alpha-phosphotrehalase_[Bacillus_cereus]	CPTF_U	51836.66667	45246.4717	87.28661507
UIJ66975.1	alpha,alpha-phosphotrehalase_[Bacillus_cereus]	CPTF_metals_mix	145602.3333	104488.2537	71.76276049
UIJ66975.1	alpha,alpha-phosphotrehalase_[Bacillus_cereus]	CPTF_zcontrol	78689.4	52606.00921	66.85272631
UIJ66981.1	phosphotransferase_[Bacillus_cereus]	CPTF_Al	456803.8333	63941.3175	13.99754399
UIJ66981.1	phosphotransferase_[Bacillus_cereus]	CPTF_Cd	528031.7667	28886.47487	5.470594138
UIJ66981.1	phosphotransferase_[Bacillus_cereus]	CPTF_Co	385578	87311.00084	22.64418635
UIJ66981.1	phosphotransferase_[Bacillus_cereus]	CPTF_Cu	437665.7	71464.09659	16.32846636
UIJ66981.1	phosphotransferase_[Bacillus_cereus]	CPTF_Fe	549453.0667	154619.7897	28.14067281
UIJ66981.1	phosphotransferase_[Bacillus_cereus]	CPTF_Mn	421702.8	89865.28449	21.31009908
UIJ66981.1	phosphotransferase_[Bacillus_cereus]	CPTF_Ni	610651.6667	170802.3774	27.97050867
UIJ66981.1	phosphotransferase_[Bacillus_cereus]	CPTF_U	445902.3333	122006.9824	27.36181743
UIJ66981.1	phosphotransferase_[Bacillus_cereus]	CPTF_metals_mix	247514.7667	52867.07848	21.35916139
UIJ66981.1	phosphotransferase_[Bacillus_cereus]	CPTF_zcontrol	460505.7333	92415.0284	20.06816022
UIJ66992.1	response_regulator_transcription_factor_[Bacillus_cereus]	CPTF_Al	551546.6667	264260.9644	47.91271172
UIJ66992.1	response_regulator_transcription_factor_[Bacillus_cereus]	CPTF_Cd	566813.1333	166726.4954	29.41471987
UIJ66992.1	response_regulator_transcription_factor_[Bacillus_cereus]	CPTF_Co	679373.6667	48965.23	7.207407706
UIJ66992.1	response_regulator_transcription_factor_[Bacillus_cereus]	CPTF_Cu	920373	96427.75554	10.47703002
UIJ66992.1	response_regulator_transcription_factor_[Bacillus_cereus]	CPTF_Fe	414300.6333	194513.7024	46.94989261
UIJ66992.1	response_regulator_transcription_factor_[Bacillus_cereus]	CPTF_Mn	614654.4667	164117.4292	26.70076248
UIJ66992.1	response_regulator_transcription_factor_[Bacillus_cereus]	CPTF_Ni	421057.3333	291513.0404	69.23357398
UIJ66992.1	response_regulator_transcription_factor_[Bacillus_cereus]	CPTF_U	306086.6667	65303.17059	21.33486287
UIJ66992.1	response_regulator_transcription_factor_[Bacillus_cereus]	CPTF_metals_mix	1015248.833	144246.0383	14.20794918
UIJ66992.1	response_regulator_transcription_factor_[Bacillus_cereus]	CPTF_zcontrol	416573.3333	309233.4732	74.23266168
UIJ66993.1	cell_wall-binding_repeat-containing_protein_[Bacillus_cereus]	CPTF_Al	10025654.87	526672.2609	5.253245478
UIJ66993.1	cell_wall-binding_repeat-containing_protein_[Bacillus_cereus]	CPTF_Cd	9320187.367	787752.5446	8.452110603
UIJ66993.1	cell_wall-binding_repeat-containing_protein_[Bacillus_cereus]	CPTF_Co	9320076.3	560327.0387	6.012043471
UIJ66993.1	cell_wall-binding_repeat-containing_protein_[Bacillus_cereus]	CPTF_Cu	9948521.933	834880.983	8.392010276
UIJ66993.1	cell_wall-binding_repeat-containing_protein_[Bacillus_cereus]	CPTF_Fe	9490989.2	716397.8102	7.548189079
UIJ66993.1	cell_wall-binding_repeat-containing_protein_[Bacillus_cereus]	CPTF_Mn	9699036.4	2011582.438	20.7400236
UIJ66993.1	cell_wall-binding_repeat-containing_protein_[Bacillus_cereus]	CPTF_Ni	8650385.633	972441.9507	11.2416023
UIJ66993.1	cell_wall-binding_repeat-containing_protein_[Bacillus_cereus]	CPTF_U	8096068.667	1181028.296	14.58767637
UIJ66993.1	cell_wall-binding_repeat-containing_protein_[Bacillus_cereus]	CPTF_metals_mix	10941579.63	677285.6118	6.190016748
UIJ66993.1	cell_wall-binding_repeat-containing_protein_[Bacillus_cereus]	CPTF_zcontrol	9338794.1	316450.8722	3.388562471

UIJ66994.1	DUF4352_domain-containing_protein_[Bacillus_cereus]	CPTF_Al	267638	297783.6802	111.2636024
UIJ66994.1	DUF4352_domain-containing_protein_[Bacillus_cereus]	CPTF_Cd	369814.6667	195283.7813	52.80585085
UIJ66994.1	DUF4352_domain-containing_protein_[Bacillus_cereus]	CPTF_Co	406573	252745.1604	62.16476755
UIJ66994.1	DUF4352_domain-containing_protein_[Bacillus_cereus]	CPTF_Cu	107130.6667	93863.57137	87.6159687
UIJ66994.1	DUF4352_domain-containing_protein_[Bacillus_cereus]	CPTF_Fe	369978	221040.073	59.74411262
UIJ66994.1	DUF4352_domain-containing_protein_[Bacillus_cereus]	CPTF_Mn	362224.6667	243029.1915	67.0934958
UIJ66994.1	DUF4352_domain-containing_protein_[Bacillus_cereus]	CPTF_Ni	199561.3333	11223.56763	5.624119383
UIJ66994.1	DUF4352_domain-containing_protein_[Bacillus_cereus]	CPTF_U	290103	121715.8921	41.95609564
UIJ66994.1	DUF4352_domain-containing_protein_[Bacillus_cereus]	CPTF_metals_mix	27838.83333	24697.37339	88.71554744
UIJ66994.1	DUF4352_domain-containing_protein_[Bacillus_cereus]	CPTF_zcontrol	215814.6667	38070.49911	17.64036694
UIJ66997.1	universal_stress_protein_[Bacillus_cereus]	CPTF_Al	0	0	0
UIJ66997.1	universal_stress_protein_[Bacillus_cereus]	CPTF_Cd	0	0	0
UIJ66997.1	universal_stress_protein_[Bacillus_cereus]	CPTF_Co	0	0	0
UIJ66997.1	universal_stress_protein_[Bacillus_cereus]	CPTF_Cu	77829.5	67531.30238	86.76825931
UIJ66997.1	universal_stress_protein_[Bacillus_cereus]	CPTF_Fe	0	0	0
UIJ66997.1	universal_stress_protein_[Bacillus_cereus]	CPTF_Mn	0	0	0
UIJ66997.1	universal_stress_protein_[Bacillus_cereus]	CPTF_Ni	0	0	0
UIJ66997.1	universal_stress_protein_[Bacillus_cereus]	CPTF_U	0	0	0
UIJ66997.1	universal_stress_protein_[Bacillus_cereus]	CPTF_metals_mix	78408.83333	29140.10049	37.16430821
UIJ66997.1	universal_stress_protein_[Bacillus_cereus]	CPTF_zcontrol	0	0	0
UIJ67002.1	ribose_operon_transcriptional_repressor_RbsR_[Bacillus_cereus]	CPTF_Al	27371.9	24630.74886	89.98552843
UIJ67002.1	ribose_operon_transcriptional_repressor_RbsR_[Bacillus_cereus]	CPTF_Cd	10529.6	18237.80218	173.2050808
UIJ67002.1	ribose_operon_transcriptional_repressor_RbsR_[Bacillus_cereus]	CPTF_Co	0	0	0
UIJ67002.1	ribose_operon_transcriptional_repressor_RbsR_[Bacillus_cereus]	CPTF_Cu	72050.93333	33467.40455	46.44964749
UIJ67002.1	ribose_operon_transcriptional_repressor_RbsR_[Bacillus_cereus]	CPTF_Fe	0	0	0
UIJ67002.1	ribose_operon_transcriptional_repressor_RbsR_[Bacillus_cereus]	CPTF_Mn	12062.03333	20892.05458	173.2050808
UIJ67002.1	ribose_operon_transcriptional_repressor_RbsR_[Bacillus_cereus]	CPTF_Ni	15079.83333	26119.0375	173.2050808
UIJ67002.1	ribose_operon_transcriptional_repressor_RbsR_[Bacillus_cereus]	CPTF_U	0	0	0
UIJ67002.1	ribose_operon_transcriptional_repressor_RbsR_[Bacillus_cereus]	CPTF_metals_mix	81536.73333	27502.72628	33.73047357
UIJ67002.1	ribose_operon_transcriptional_repressor_RbsR_[Bacillus_cereus]	CPTF_zcontrol	10035.46667	17381.93814	173.2050808
UIJ67003.1	D-ribose_pyranase_[Bacillus_cereus]	CPTF_Al	0	0	0
UIJ67003.1	D-ribose_pyranase_[Bacillus_cereus]	CPTF_Cd	0	0	0
UIJ67003.1	D-ribose_pyranase_[Bacillus_cereus]	CPTF_Co	0	0	0
UIJ67003.1	D-ribose_pyranase_[Bacillus_cereus]	CPTF_Cu	0	0	0
UIJ67003.1	D-ribose_pyranase_[Bacillus_cereus]	CPTF_Fe	0	0	0
UIJ67003.1	D-ribose_pyranase_[Bacillus_cereus]	CPTF_Mn	0	0	0
UIJ67003.1	D-ribose_pyranase_[Bacillus_cereus]	CPTF_Ni	0	0	0
UIJ67003.1	D-ribose_pyranase_[Bacillus_cereus]	CPTF_U	0	0	0
UIJ67003.1	D-ribose_pyranase_[Bacillus_cereus]	CPTF_metals_mix	176511.3333	34863.81867	19.75160349
UIJ67003.1	D-ribose_pyranase_[Bacillus_cereus]	CPTF_zcontrol	0	0	0
UIJ67006.1	ribose_ABC_transporter_substrate-binding_protein_RbsB_[Bacillus_cereus]	CPTF_Al	72883.66667	63437.56854	87.03948558
UIJ67006.1	ribose_ABC_transporter_substrate-binding_protein_RbsB_[Bacillus_cereus]	CPTF_Cd	75419	66842.14409	88.62772523
UIJ67006.1	ribose_ABC_transporter_substrate-binding_protein_RbsB_[Bacillus_cereus]	CPTF_Co	104307.1	21023.61635	20.15549886
UIJ67006.1	ribose_ABC_transporter_substrate-binding_protein_RbsB_[Bacillus_cereus]	CPTF_Cu	75307.33333	65615.61388	87.13044397
UIJ67006.1	ribose_ABC_transporter_substrate-binding_protein_RbsB_[Bacillus_cereus]	CPTF_Fe	0	0	0
UIJ67006.1	ribose_ABC_transporter_substrate-binding_protein_RbsB_[Bacillus_cereus]	CPTF_Mn	59984.43333	61353.50657	102.2823809
UIJ67006.1	ribose_ABC_transporter_substrate-binding_protein_RbsB_[Bacillus_cereus]	CPTF_Ni	114046.1667	26346.01789	23.1011867
UIJ67006.1	ribose_ABC_transporter_substrate-binding_protein_RbsB_[Bacillus_cereus]	CPTF_U	0	0	0
UIJ67006.1	ribose_ABC_transporter_substrate-binding_protein_RbsB_[Bacillus_cereus]	CPTF_metals_mix	24475.76667	42393.27142	173.2050808
UIJ67006.1	ribose_ABC_transporter_substrate-binding_protein_RbsB_[Bacillus_cereus]	CPTF_zcontrol	67148.93333	63777.56036	94.97926057
UIJ67007.1	fructose-6-phosphate_aldolase_[Bacillus_cereus]	CPTF_Al	1028163.667	114958.9912	11.18100113
UIJ67007.1	fructose-6-phosphate_aldolase_[Bacillus_cereus]	CPTF_Cd	1881394.2	558837.8342	29.70338881
UIJ67007.1	fructose-6-phosphate_aldolase_[Bacillus_cereus]	CPTF_Co	1088595.333	80908.31781	7.43235942
UIJ67007.1	fructose-6-phosphate_aldolase_[Bacillus_cereus]	CPTF_Cu	1230253	29264.71696	2.378755993
UIJ67007.1	fructose-6-phosphate_aldolase_[Bacillus_cereus]	CPTF_Fe	1462741	772779.8516	52.83094216
UIJ67007.1	fructose-6-phosphate_aldolase_[Bacillus_cereus]	CPTF_Mn	1858699	976905.9542	52.55858825
UIJ67007.1	fructose-6-phosphate_aldolase_[Bacillus_cereus]	CPTF_Ni	994530.6667	141464.222	14.2242192
UIJ67007.1	fructose-6-phosphate_aldolase_[Bacillus_cereus]	CPTF_U	811104.1667	124858.431	15.39363698
UIJ67007.1	fructose-6-phosphate_aldolase_[Bacillus_cereus]	CPTF_metals_mix	6382616.667	986643.319	15.45828883
UIJ67007.1	fructose-6-phosphate_aldolase_[Bacillus_cereus]	CPTF_zcontrol	950094.6667	193440.6527	20.3601451
UIJ67011.1	(R,R)-butanediol_dehydrogenase_[Bacillus_cereus]	CPTF_Al	2255008.133	39312.63569	1.743347845

UIJ67011.1	(R,R)-butanediol_dehydrogenase_[Bacillus_cereus]	CPTF_Cd	1941651.633	295922.6824	15.24077117
UIJ67011.1	(R,R)-butanediol_dehydrogenase_[Bacillus_cereus]	CPTF_Co	1884027.1	358239.4318	19.01455833
UIJ67011.1	(R,R)-butanediol_dehydrogenase_[Bacillus_cereus]	CPTF_Cu	2658893	232861.7875	8.757847252
UIJ67011.1	(R,R)-butanediol_dehydrogenase_[Bacillus_cereus]	CPTF_Fe	1899383.033	268323.9578	14.12690085
UIJ67011.1	(R,R)-butanediol_dehydrogenase_[Bacillus_cereus]	CPTF_Mn	2182563.567	418416.1402	19.170857
UIJ67011.1	(R,R)-butanediol_dehydrogenase_[Bacillus_cereus]	CPTF_Ni	2045981.167	245075.6213	11.97839087
UIJ67011.1	(R,R)-butanediol_dehydrogenase_[Bacillus_cereus]	CPTF_U	1999639.467	328449.9726	16.42545959
UIJ67011.1	(R,R)-butanediol_dehydrogenase_[Bacillus_cereus]	CPTF_metals_mix	6056616.667	510057.3259	8.421489322
UIJ67011.1	(R,R)-butanediol_dehydrogenase_[Bacillus_cereus]	CPTF_zcontrol	1408226.633	348236.4876	24.72872472
UIJ67016.1	AimR_family_lysis-lysogeny_pheromone_receptor_[Bacillus_cereus]	CPTF_Al	197308.6667	172365.5355	87.35831954
UIJ67016.1	AimR_family_lysis-lysogeny_pheromone_receptor_[Bacillus_cereus]	CPTF_Cd	152304.6667	132203.0787	86.80172551
UIJ67016.1	AimR_family_lysis-lysogeny_pheromone_receptor_[Bacillus_cereus]	CPTF_Co	299121.6667	51718.11756	17.28999378
UIJ67016.1	AimR_family_lysis-lysogeny_pheromone_receptor_[Bacillus_cereus]	CPTF_Cu	200287.6667	22847.60518	11.40739495
UIJ67016.1	AimR_family_lysis-lysogeny_pheromone_receptor_[Bacillus_cereus]	CPTF_Fe	182061.3333	172697.3029	94.85666161
UIJ67016.1	AimR_family_lysis-lysogeny_pheromone_receptor_[Bacillus_cereus]	CPTF_Mn	266405	38344.30196	14.3932366
UIJ67016.1	AimR_family_lysis-lysogeny_pheromone_receptor_[Bacillus_cereus]	CPTF_Ni	253809.6667	55607.39825	21.90909392
UIJ67016.1	AimR_family_lysis-lysogeny_pheromone_receptor_[Bacillus_cereus]	CPTF_U	345218.6667	50248.37957	14.55552217
UIJ67016.1	AimR_family_lysis-lysogeny_pheromone_receptor_[Bacillus_cereus]	CPTF_metals_mix	0	0	0
UIJ67016.1	AimR_family_lysis-lysogeny_pheromone_receptor_[Bacillus_cereus]	CPTF_zcontrol	325635.3333	14753.89658	4.530803347
UIJ67018.1	helix-turn-helix_domain-containing_protein_[Bacillus_cereus]	CPTF_Al	387743	53693.97824	13.84782659
UIJ67018.1	helix-turn-helix_domain-containing_protein_[Bacillus_cereus]	CPTF_Cd	412934.7333	19766.73629	4.786891171
UIJ67018.1	helix-turn-helix_domain-containing_protein_[Bacillus_cereus]	CPTF_Co	327648.3333	53896.44445	16.4494792
UIJ67018.1	helix-turn-helix_domain-containing_protein_[Bacillus_cereus]	CPTF_Cu	354309.6667	37487.18667	10.58034544
UIJ67018.1	helix-turn-helix_domain-containing_protein_[Bacillus_cereus]	CPTF_Fe	378742.4333	38106.90454	10.06142993
UIJ67018.1	helix-turn-helix_domain-containing_protein_[Bacillus_cereus]	CPTF_Mn	184712.5333	160118.2717	86.68511489
UIJ67018.1	helix-turn-helix_domain-containing_protein_[Bacillus_cereus]	CPTF_Ni	314237.3333	34400.7134	10.94736677
UIJ67018.1	helix-turn-helix_domain-containing_protein_[Bacillus_cereus]	CPTF_U	240939.3333	209369.0154	86.8969846
UIJ67018.1	helix-turn-helix_domain-containing_protein_[Bacillus_cereus]	CPTF_metals_mix	269452.9667	195615.0017	72.59708591
UIJ67018.1	helix-turn-helix_domain-containing_protein_[Bacillus_cereus]	CPTF_zcontrol	177247.6667	178784.3173	100.8669511
UIJ67063.1	cytochrome_aa3_quinol_oxidase_subunit_III_[Bacillus_cereus]	CPTF_Al	0	0	0
UIJ67063.1	cytochrome_aa3_quinol_oxidase_subunit_III_[Bacillus_cereus]	CPTF_Cd	0	0	0
UIJ67063.1	cytochrome_aa3_quinol_oxidase_subunit_III_[Bacillus_cereus]	CPTF_Co	44069	38229.33397	86.74881202
UIJ67063.1	cytochrome_aa3_quinol_oxidase_subunit_III_[Bacillus_cereus]	CPTF_Cu	51751.43333	44951.21142	86.85983851
UIJ67063.1	cytochrome_aa3_quinol_oxidase_subunit_III_[Bacillus_cereus]	CPTF_Fe	0	0	0
UIJ67063.1	cytochrome_aa3_quinol_oxidase_subunit_III_[Bacillus_cereus]	CPTF_Mn	15026.6	26026.83467	173.2050808
UIJ67063.1	cytochrome_aa3_quinol_oxidase_subunit_III_[Bacillus_cereus]	CPTF_Ni	12695.26667	21988.84688	173.2050808
UIJ67063.1	cytochrome_aa3_quinol_oxidase_subunit_III_[Bacillus_cereus]	CPTF_U	0	0	0
UIJ67063.1	cytochrome_aa3_quinol_oxidase_subunit_III_[Bacillus_cereus]	CPTF_metals_mix	89350.93333	31091.70062	34.7972869
UIJ67063.1	cytochrome_aa3_quinol_oxidase_subunit_III_[Bacillus_cereus]	CPTF_zcontrol	9922.1	17185.58132	173.2050808
UIJ67064.1	cytochrome_aa3_quinol_oxidase_subunit_I_[Bacillus_cereus]	CPTF_Al	1890453.967	250829.0894	13.26819345
UIJ67064.1	cytochrome_aa3_quinol_oxidase_subunit_I_[Bacillus_cereus]	CPTF_Cd	1802700.5	66901.52489	3.711183577
UIJ67064.1	cytochrome_aa3_quinol_oxidase_subunit_I_[Bacillus_cereus]	CPTF_Co	1580371.067	35181.17236	2.226133666
UIJ67064.1	cytochrome_aa3_quinol_oxidase_subunit_I_[Bacillus_cereus]	CPTF_Cu	1614458	99451.40211	6.16004889
UIJ67064.1	cytochrome_aa3_quinol_oxidase_subunit_I_[Bacillus_cereus]	CPTF_Fe	1883037.133	41076.81698	2.181413009
UIJ67064.1	cytochrome_aa3_quinol_oxidase_subunit_I_[Bacillus_cereus]	CPTF_Mn	1906266.833	349972.3212	18.35904162
UIJ67064.1	cytochrome_aa3_quinol_oxidase_subunit_I_[Bacillus_cereus]	CPTF_Ni	1795658.367	166254.3182	9.258683124
UIJ67064.1	cytochrome_aa3_quinol_oxidase_subunit_I_[Bacillus_cereus]	CPTF_U	1964033.333	250752.2298	12.76720845
UIJ67064.1	cytochrome_aa3_quinol_oxidase_subunit_I_[Bacillus_cereus]	CPTF_metals_mix	1203998.533	71365.24154	5.927352863
UIJ67064.1	cytochrome_aa3_quinol_oxidase_subunit_I_[Bacillus_cereus]	CPTF_zcontrol	2124714.533	103908.199	4.890454569
UIJ67065.1	cytochrome_aa3_quinol_oxidase_subunit_II_[Bacillus_cereus]	CPTF_Al	1713729.333	568814.5142	33.19161918
UIJ67065.1	cytochrome_aa3_quinol_oxidase_subunit_II_[Bacillus_cereus]	CPTF_Cd	2308098.4	59020.2204	2.557092904
UIJ67065.1	cytochrome_aa3_quinol_oxidase_subunit_II_[Bacillus_cereus]	CPTF_Co	2048907	172818.2384	8.43465508
UIJ67065.1	cytochrome_aa3_quinol_oxidase_subunit_II_[Bacillus_cereus]	CPTF_Cu	1909065.833	214877.8519	11.25565437
UIJ67065.1	cytochrome_aa3_quinol_oxidase_subunit_II_[Bacillus_cereus]	CPTF_Fe	1938826.667	476720.7125	24.58810376
UIJ67065.1	cytochrome_aa3_quinol_oxidase_subunit_II_[Bacillus_cereus]	CPTF_Mn	1846656	293274.0118	15.88135591
UIJ67065.1	cytochrome_aa3_quinol_oxidase_subunit_II_[Bacillus_cereus]	CPTF_Ni	1969702.5	106339.9223	5.398780899
UIJ67065.1	cytochrome_aa3_quinol_oxidase_subunit_II_[Bacillus_cereus]	CPTF_U	1436679.8	446993.1553	31.11292825
UIJ67065.1	cytochrome_aa3_quinol_oxidase_subunit_II_[Bacillus_cereus]	CPTF_metals_mix	1985966.833	391673.3884	19.72205083
UIJ67065.1	cytochrome_aa3_quinol_oxidase_subunit_II_[Bacillus_cereus]	CPTF_zcontrol	1864997.333	352047.7138	18.87658001
UIJ67098.1	potassium-transporting_ATPase_subunit_KdpA_[Bacillus_cereus]	CPTF_Al	235053.6667	154664.7268	65.79975074
UIJ67098.1	potassium-transporting_ATPase_subunit_KdpA_[Bacillus_cereus]	CPTF_Cd	91090.23333	43625.01393	47.89208715

UIJ67098.1	potassium-transporting_ATPase_subunit_KdpA_[Bacillus_cereus]	CPTF_Co	666952	131367.4426	19.69668621
UIJ67098.1	potassium-transporting_ATPase_subunit_KdpA_[Bacillus_cereus]	CPTF_Cu	273091	251510.3981	92.09765175
UIJ67098.1	potassium-transporting_ATPase_subunit_KdpA_[Bacillus_cereus]	CPTF_Fe	207606.3333	135060.9402	65.05627164
UIJ67098.1	potassium-transporting_ATPase_subunit_KdpA_[Bacillus_cereus]	CPTF_Mn	323788.3333	288749.5536	89.1784922
UIJ67098.1	potassium-transporting_ATPase_subunit_KdpA_[Bacillus_cereus]	CPTF_Ni	526162.3333	76240.55932	14.48993105
UIJ67098.1	potassium-transporting_ATPase_subunit_KdpA_[Bacillus_cereus]	CPTF_U	598364.6667	337287.8112	56.36827005
UIJ67098.1	potassium-transporting_ATPase_subunit_KdpA_[Bacillus_cereus]	CPTF_metals_mix	508105	48196.23036	9.485486339
UIJ67098.1	potassium-transporting_ATPase_subunit_KdpA_[Bacillus_cereus]	CPTF_zcontrol	390225.6667	228704.2549	58.60820402
UIJ67099.1	K(+)-transporting_ATPase_subunit_C_[Bacillus_cereus]	CPTF_Al	389256.7667	161972.73	41.61076798
UIJ67099.1	K(+)-transporting_ATPase_subunit_C_[Bacillus_cereus]	CPTF_Cd	302938.2667	132376.0858	43.69738009
UIJ67099.1	K(+)-transporting_ATPase_subunit_C_[Bacillus_cereus]	CPTF_Co	562803.7667	165286.6739	29.36843775
UIJ67099.1	K(+)-transporting_ATPase_subunit_C_[Bacillus_cereus]	CPTF_Cu	415900.0667	42369.51619	10.18742712
UIJ67099.1	K(+)-transporting_ATPase_subunit_C_[Bacillus_cereus]	CPTF_Fe	227007.0667	42693.45827	18.80710539
UIJ67099.1	K(+)-transporting_ATPase_subunit_C_[Bacillus_cereus]	CPTF_Mn	307114.8	172849.5804	56.28174884
UIJ67099.1	K(+)-transporting_ATPase_subunit_C_[Bacillus_cereus]	CPTF_Ni	360316.6333	114319.975	31.72764297
UIJ67099.1	K(+)-transporting_ATPase_subunit_C_[Bacillus_cereus]	CPTF_U	257375.8	81595.4235	31.70283434
UIJ67099.1	K(+)-transporting_ATPase_subunit_C_[Bacillus_cereus]	CPTF_metals_mix	350236.5667	83973.25868	23.97615403
UIJ67099.1	K(+)-transporting_ATPase_subunit_C_[Bacillus_cereus]	CPTF_zcontrol	211856.9667	87378.67891	41.24418483
UIJ67109.1	thioredoxin_family_protein_[Bacillus_cereus]	CPTF_Al	0	0	0
UIJ67109.1	thioredoxin_family_protein_[Bacillus_cereus]	CPTF_Cd	0	0	0
UIJ67109.1	thioredoxin_family_protein_[Bacillus_cereus]	CPTF_Co	0	0	0
UIJ67109.1	thioredoxin_family_protein_[Bacillus_cereus]	CPTF_Cu	0	0	0
UIJ67109.1	thioredoxin_family_protein_[Bacillus_cereus]	CPTF_Fe	0	0	0
UIJ67109.1	thioredoxin_family_protein_[Bacillus_cereus]	CPTF_Mn	15740.66667	27263.63441	173.2050808
UIJ67109.1	thioredoxin_family_protein_[Bacillus_cereus]	CPTF_Ni	0	0	0
UIJ67109.1	thioredoxin_family_protein_[Bacillus_cereus]	CPTF_U	0	0	0
UIJ67109.1	thioredoxin_family_protein_[Bacillus_cereus]	CPTF_metals_mix	19492.8	17986.07301	92.27034091
UIJ67109.1	thioredoxin_family_protein_[Bacillus_cereus]	CPTF_zcontrol	0	0	0
UIJ67113.1	PCYCGC_domain-containing_protein_[Bacillus_cereus]	CPTF_Al	247123.9667	166324.9562	67.30425964
UIJ67113.1	PCYCGC_domain-containing_protein_[Bacillus_cereus]	CPTF_Cd	295200.6667	107341.5645	36.36223647
UIJ67113.1	PCYCGC_domain-containing_protein_[Bacillus_cereus]	CPTF_Co	268793	182490.5229	67.89258757
UIJ67113.1	PCYCGC_domain-containing_protein_[Bacillus_cereus]	CPTF_Cu	160172.6667	55574.26223	34.69647062
UIJ67113.1	PCYCGC_domain-containing_protein_[Bacillus_cereus]	CPTF_Fe	309063.3333	110737.7114	35.83010324
UIJ67113.1	PCYCGC_domain-containing_protein_[Bacillus_cereus]	CPTF_Mn	251197.6667	112487.6848	44.78054526
UIJ67113.1	PCYCGC_domain-containing_protein_[Bacillus_cereus]	CPTF_Ni	269447.3333	60767.79346	22.55275371
UIJ67113.1	PCYCGC_domain-containing_protein_[Bacillus_cereus]	CPTF_U	392176.6667	123901.2944	31.59323462
UIJ67113.1	PCYCGC_domain-containing_protein_[Bacillus_cereus]	CPTF_metals_mix	261123.3333	29986.20011	11.48353911
UIJ67113.1	PCYCGC_domain-containing_protein_[Bacillus_cereus]	CPTF_zcontrol	305354.6667	130868.4094	42.85783833
UIJ67136.1	hypothetical_protein_LW858_02225_[Bacillus_cereus]	CPTF_Al	607751.6667	54858.4767	9.026462568
UIJ67136.1	hypothetical_protein_LW858_02225_[Bacillus_cereus]	CPTF_Cd	390277	340103.8014	87.14420819
UIJ67136.1	hypothetical_protein_LW858_02225_[Bacillus_cereus]	CPTF_Co	513270.3333	79243.52773	15.43894564
UIJ67136.1	hypothetical_protein_LW858_02225_[Bacillus_cereus]	CPTF_Cu	543840	98497.34114	18.11145579
UIJ67136.1	hypothetical_protein_LW858_02225_[Bacillus_cereus]	CPTF_Fe	300425	267482.2234	89.03460877
UIJ67136.1	hypothetical_protein_LW858_02225_[Bacillus_cereus]	CPTF_Mn	159090.3333	275552.5403	173.2050808
UIJ67136.1	hypothetical_protein_LW858_02225_[Bacillus_cereus]	CPTF_Ni	656033.6667	113142.6934	17.2464767
UIJ67136.1	hypothetical_protein_LW858_02225_[Bacillus_cereus]	CPTF_U	331106.3333	287754.3343	86.90692545
UIJ67136.1	hypothetical_protein_LW858_02225_[Bacillus_cereus]	CPTF_metals_mix	230485.3333	206313.2578	89.51253202
UIJ67136.1	hypothetical_protein_LW858_02225_[Bacillus_cereus]	CPTF_zcontrol	363629.6667	315184.8628	86.67743359
UIJ67137.1	LCP_family_protein_[Bacillus_cereus]	CPTF_Al	12905.16667	22352.40435	173.2050808
UIJ67137.1	LCP_family_protein_[Bacillus_cereus]	CPTF_Cd	138681.0333	173878.3335	125.3800388
UIJ67137.1	LCP_family_protein_[Bacillus_cereus]	CPTF_Co	183214.0667	87969.42253	48.01455703
UIJ67137.1	LCP_family_protein_[Bacillus_cereus]	CPTF_Cu	153966.6333	182221.5457	118.3513218
UIJ67137.1	LCP_family_protein_[Bacillus_cereus]	CPTF_Fe	181181.1667	166009.6397	91.62632231
UIJ67137.1	LCP_family_protein_[Bacillus_cereus]	CPTF_Mn	264699.7667	125489.1567	47.40811007
UIJ67137.1	LCP_family_protein_[Bacillus_cereus]	CPTF_Ni	199082.9667	158460.6669	79.59529113
UIJ67137.1	LCP_family_protein_[Bacillus_cereus]	CPTF_U	30036.36667	52024.51314	173.2050808
UIJ67137.1	LCP_family_protein_[Bacillus_cereus]	CPTF_metals_mix	24003.26667	41574.87741	173.2050808
UIJ67137.1	LCP_family_protein_[Bacillus_cereus]	CPTF_zcontrol	172649.3667	153704.2287	89.02681294
UIJ67203.1	NADP-dependent_glyceraldehyde-3-phosphate_dehydrogenase_[Bacillus_cereus]	CPTF_Al	2301403.433	395284.16	17.17578736
UIJ67203.1	NADP-dependent_glyceraldehyde-3-phosphate_dehydrogenase_[Bacillus_cereus]	CPTF_Cd	2711632.6	26646.96887	0.982690976
UIJ67203.1	NADP-dependent_glyceraldehyde-3-phosphate_dehydrogenase_[Bacillus_cereus]	CPTF_Co	2191085.833	240264.0934	10.9655263

UIJ67203.1	NADP-dependent_glyceraldehyde-3-phosphate_dehydrogenase_[Bacillus_cereus]	CPTF_Cu	2445052.667	312095.9906	12.76438724
UIJ67203.1	NADP-dependent_glyceraldehyde-3-phosphate_dehydrogenase_[Bacillus_cereus]	CPTF_Fe	2031315.1	384970.4323	18.95178312
UIJ67203.1	NADP-dependent_glyceraldehyde-3-phosphate_dehydrogenase_[Bacillus_cereus]	CPTF_Mn	1655161.8	525109.8042	31.72558745
UIJ67203.1	NADP-dependent_glyceraldehyde-3-phosphate_dehydrogenase_[Bacillus_cereus]	CPTF_Ni	1476506.967	137219.0219	9.293489637
UIJ67203.1	NADP-dependent_glyceraldehyde-3-phosphate_dehydrogenase_[Bacillus_cereus]	CPTF_U	1552257.233	82666.60524	5.325573846
UIJ67203.1	NADP-dependent_glyceraldehyde-3-phosphate_dehydrogenase_[Bacillus_cereus]	CPTF_metals_mix	5051964.4	560465.2911	11.09400714
UIJ67203.1	NADP-dependent_glyceraldehyde-3-phosphate_dehydrogenase_[Bacillus_cereus]	CPTF_zcontrol	1735753.1	177331.8459	10.2164211
UIJ67206.1	amino_acid_ABC_transporter_substrate-binding_protein_[Bacillus_cereus]	CPTF_Al	2791574.733	146792.1154	5.258398197
UIJ67206.1	amino_acid_ABC_transporter_substrate-binding_protein_[Bacillus_cereus]	CPTF_Cd	3176401	495869.3167	15.61104271
UIJ67206.1	amino_acid_ABC_transporter_substrate-binding_protein_[Bacillus_cereus]	CPTF_Co	3047099.7	92040.54935	3.020595268
UIJ67206.1	amino_acid_ABC_transporter_substrate-binding_protein_[Bacillus_cereus]	CPTF_Cu	3333819.733	8006.129703	0.240148849
UIJ67206.1	amino_acid_ABC_transporter_substrate-binding_protein_[Bacillus_cereus]	CPTF_Fe	2510537.267	161175.9019	6.419976473
UIJ67206.1	amino_acid_ABC_transporter_substrate-binding_protein_[Bacillus_cereus]	CPTF_Mn	2618088.167	77906.75067	2.975711501
UIJ67206.1	amino_acid_ABC_transporter_substrate-binding_protein_[Bacillus_cereus]	CPTF_Ni	2789523.267	107905.9949	3.86825936
UIJ67206.1	amino_acid_ABC_transporter_substrate-binding_protein_[Bacillus_cereus]	CPTF_U	2238372.7	583158.2618	26.05277762
UIJ67206.1	amino_acid_ABC_transporter_substrate-binding_protein_[Bacillus_cereus]	CPTF_metals_mix	4060513.967	331555.7875	8.16536503
UIJ67206.1	amino_acid_ABC_transporter_substrate-binding_protein_[Bacillus_cereus]	CPTF_zcontrol	2798221.8	126548.4221	4.522458587
UIJ67212.1	YlbF/YmcA_family_competence_regulator_[Bacillus_cereus]	CPTF_Al	3849891.167	140408.8525	3.647086278
UIJ67212.1	YlbF/YmcA_family_competence_regulator_[Bacillus_cereus]	CPTF_Cd	4039282.233	48874.19132	1.209972181
UIJ67212.1	YlbF/YmcA_family_competence_regulator_[Bacillus_cereus]	CPTF_Co	3611094.333	370478.8739	10.25946264
UIJ67212.1	YlbF/YmcA_family_competence_regulator_[Bacillus_cereus]	CPTF_Cu	3609830.667	268302.0692	7.432538919
UIJ67212.1	YlbF/YmcA_family_competence_regulator_[Bacillus_cereus]	CPTF_Fe	4002496.667	362886.3709	9.066500264
UIJ67212.1	YlbF/YmcA_family_competence_regulator_[Bacillus_cereus]	CPTF_Mn	4172487.7	133082.8628	3.189532777
UIJ67212.1	YlbF/YmcA_family_competence_regulator_[Bacillus_cereus]	CPTF_Ni	2377320.7	1627043.552	68.44022145
UIJ67212.1	YlbF/YmcA_family_competence_regulator_[Bacillus_cereus]	CPTF_U	3974433.367	183277.7636	4.611418703
UIJ67212.1	YlbF/YmcA_family_competence_regulator_[Bacillus_cereus]	CPTF_metals_mix	3923245.333	62673.95977	1.597502946
UIJ67212.1	YlbF/YmcA_family_competence_regulator_[Bacillus_cereus]	CPTF_zcontrol	4300609.733	282807.3334	6.575982265
UIJ67220.1	tetratricopeptide_repeat_protein_[Bacillus_cereus]	CPTF_Al	7898218.767	920694.6644	11.65699117
UIJ67220.1	tetratricopeptide_repeat_protein_[Bacillus_cereus]	CPTF_Cd	7139312.133	2068980.486	28.98010968
UIJ67220.1	tetratricopeptide_repeat_protein_[Bacillus_cereus]	CPTF_Co	8837551.1	975983.4228	11.04359581
UIJ67220.1	tetratricopeptide_repeat_protein_[Bacillus_cereus]	CPTF_Cu	8524106.3	233342.6192	2.737443797
UIJ67220.1	tetratricopeptide_repeat_protein_[Bacillus_cereus]	CPTF_Fe	6235651.667	1146066.96	18.37926525
UIJ67220.1	tetratricopeptide_repeat_protein_[Bacillus_cereus]	CPTF_Mn	6138460.233	1121514.757	18.27029441
UIJ67220.1	tetratricopeptide_repeat_protein_[Bacillus_cereus]	CPTF_Ni	6202945.933	2197717.457	35.43022107
UIJ67220.1	tetratricopeptide_repeat_protein_[Bacillus_cereus]	CPTF_U	5028654.5	302068.9984	6.006954712
UIJ67220.1	tetratricopeptide_repeat_protein_[Bacillus_cereus]	CPTF_metals_mix	9972504.3	543756.4944	5.452557132
UIJ67220.1	tetratricopeptide_repeat_protein_[Bacillus_cereus]	CPTF_zcontrol	7252727.5	2137376.057	29.46996225
UIJ67228.1	S-layer_homology_domain-containing_protein_[Bacillus_cereus]	CPTF_Al	0	0	0
UIJ67228.1	S-layer_homology_domain-containing_protein_[Bacillus_cereus]	CPTF_Cd	0	0	0
UIJ67228.1	S-layer_homology_domain-containing_protein_[Bacillus_cereus]	CPTF_Co	0	0	0
UIJ67228.1	S-layer_homology_domain-containing_protein_[Bacillus_cereus]	CPTF_Cu	0	0	0
UIJ67228.1	S-layer_homology_domain-containing_protein_[Bacillus_cereus]	CPTF_Fe	0	0	0
UIJ67228.1	S-layer_homology_domain-containing_protein_[Bacillus_cereus]	CPTF_Mn	0	0	0
UIJ67228.1	S-layer_homology_domain-containing_protein_[Bacillus_cereus]	CPTF_Ni	0	0	0
UIJ67228.1	S-layer_homology_domain-containing_protein_[Bacillus_cereus]	CPTF_U	0	0	0
UIJ67228.1	S-layer_homology_domain-containing_protein_[Bacillus_cereus]	CPTF_metals_mix	10522.13333	18224.86954	173.2050808
UIJ67228.1	S-layer_homology_domain-containing_protein_[Bacillus_cereus]	CPTF_zcontrol	0	0	0
UIJ67230.1	fatty_acid--CoA_ligase_[Bacillus_cereus]	CPTF_Al	101866.2333	29900.60614	29.35281414
UIJ67230.1	fatty_acid--CoA_ligase_[Bacillus_cereus]	CPTF_Cd	178682.2	63642.41103	35.61765583
UIJ67230.1	fatty_acid--CoA_ligase_[Bacillus_cereus]	CPTF_Co	132147.8667	41528.87528	31.42606561
UIJ67230.1	fatty_acid--CoA_ligase_[Bacillus_cereus]	CPTF_Cu	79764.96667	50796.45846	63.68266744
UIJ67230.1	fatty_acid--CoA_ligase_[Bacillus_cereus]	CPTF_Fe	155675.0333	30399.20364	19.5273468
UIJ67230.1	fatty_acid--CoA_ligase_[Bacillus_cereus]	CPTF_Mn	112264.8667	95895.21492	85.4187225
UIJ67230.1	fatty_acid--CoA_ligase_[Bacillus_cereus]	CPTF_Ni	25311.83333	22969.85019	90.74747721
UIJ67230.1	fatty_acid--CoA_ligase_[Bacillus_cereus]	CPTF_U	85865.83333	74667.47205	86.95830362
UIJ67230.1	fatty_acid--CoA_ligase_[Bacillus_cereus]	CPTF_metals_mix	97551.06667	12802.84051	13.12424451
UIJ67230.1	fatty_acid--CoA_ligase_[Bacillus_cereus]	CPTF_zcontrol	41602.73333	40803.20038	98.07817205
UIJ67238.1	enoyl-CoA_hydratase_[Bacillus_cereus]	CPTF_Al	756261	102060.9924	13.49547211
UIJ67238.1	enoyl-CoA_hydratase_[Bacillus_cereus]	CPTF_Cd	1049535.333	121796.3818	11.60479099
UIJ67238.1	enoyl-CoA_hydratase_[Bacillus_cereus]	CPTF_Co	855070.6667	164188.861	19.20178851
UIJ67238.1	enoyl-CoA_hydratase_[Bacillus_cereus]	CPTF_Cu	768287.3333	87655.38645	11.40919323

UIJ67238.1	enoyl-CoA_hydratase_[Bacillus_cereus]	CPTF_Fe	768293.6667	149602.9091	19.47209975
UIJ67238.1	enoyl-CoA_hydratase_[Bacillus_cereus]	CPTF_Mn	877583.3333	73372.2745	8.360718773
UIJ67238.1	enoyl-CoA_hydratase_[Bacillus_cereus]	CPTF_Ni	626437.0667	226176.5206	36.10522631
UIJ67238.1	enoyl-CoA_hydratase_[Bacillus_cereus]	CPTF_U	543767.3333	110222.6449	20.27018509
UIJ67238.1	enoyl-CoA_hydratase_[Bacillus_cereus]	CPTF_metals_mix	778629.3333	105106.754	13.4989461
UIJ67238.1	enoyl-CoA_hydratase_[Bacillus_cereus]	CPTF_zcontrol	464204.6333	124394.3235	26.79730329
UIJ67242.1	M42_family_metallopeptidase_[Bacillus_cereus]	CPTF_Al	1426863	141979.8559	9.950489706
UIJ67242.1	M42_family_metallopeptidase_[Bacillus_cereus]	CPTF_Cd	838937.3333	465295.8197	55.46252398
UIJ67242.1	M42_family_metallopeptidase_[Bacillus_cereus]	CPTF_Co	1174322	518986.5146	44.19456628
UIJ67242.1	M42_family_metallopeptidase_[Bacillus_cereus]	CPTF_Cu	1579757.667	152986.3936	9.684168451
UIJ67242.1	M42_family_metallopeptidase_[Bacillus_cereus]	CPTF_Fe	1450363	279923.2721	19.30022154
UIJ67242.1	M42_family_metallopeptidase_[Bacillus_cereus]	CPTF_Mn	1281275.333	310226.3706	24.21231117
UIJ67242.1	M42_family_metallopeptidase_[Bacillus_cereus]	CPTF_Ni	1168901.233	661077.8642	56.55549377
UIJ67242.1	M42_family_metallopeptidase_[Bacillus_cereus]	CPTF_U	1155092.667	742745.2688	64.30179069
UIJ67242.1	M42_family_metallopeptidase_[Bacillus_cereus]	CPTF_metals_mix	510586.6667	384005.9813	75.20877578
UIJ67242.1	M42_family_metallopeptidase_[Bacillus_cereus]	CPTF_zcontrol	924005	644904.3276	69.79446297
UIJ67247.1	ABC_transporter_substrate-binding_protein_[Bacillus_cereus]	CPTF_Al	2748614	250836.6717	9.125932986
UIJ67247.1	ABC_transporter_substrate-binding_protein_[Bacillus_cereus]	CPTF_Cd	2481475	417428.3527	16.82178353
UIJ67247.1	ABC_transporter_substrate-binding_protein_[Bacillus_cereus]	CPTF_Co	2861078	273734.0907	9.567515836
UIJ67247.1	ABC_transporter_substrate-binding_protein_[Bacillus_cereus]	CPTF_Cu	2854318.067	144852.2959	5.074847741
UIJ67247.1	ABC_transporter_substrate-binding_protein_[Bacillus_cereus]	CPTF_Fe	2466705	257512.8916	10.43954958
UIJ67247.1	ABC_transporter_substrate-binding_protein_[Bacillus_cereus]	CPTF_Mn	2359667	500444.3069	21.20825976
UIJ67247.1	ABC_transporter_substrate-binding_protein_[Bacillus_cereus]	CPTF_Ni	2594335.5	346910.3357	13.37183782
UIJ67247.1	ABC_transporter_substrate-binding_protein_[Bacillus_cereus]	CPTF_U	2416358.033	248995.9661	10.30459736
UIJ67247.1	ABC_transporter_substrate-binding_protein_[Bacillus_cereus]	CPTF_metals_mix	2275293.8	185173.6391	8.138449597
UIJ67247.1	ABC_transporter_substrate-binding_protein_[Bacillus_cereus]	CPTF_zcontrol	2472255.667	232296.6072	9.396140144
UIJ67250.1	ABC_transporter_ATP-binding_protein_[Bacillus_cereus]	CPTF_Al	0	0	0
UIJ67250.1	ABC_transporter_ATP-binding_protein_[Bacillus_cereus]	CPTF_Cd	44327.66667	40236.65698	90.77097895
UIJ67250.1	ABC_transporter_ATP-binding_protein_[Bacillus_cereus]	CPTF_Co	0	0	0
UIJ67250.1	ABC_transporter_ATP-binding_protein_[Bacillus_cereus]	CPTF_Cu	7183.733333	12442.59112	173.2050808
UIJ67250.1	ABC_transporter_ATP-binding_protein_[Bacillus_cereus]	CPTF_Fe	0	0	0
UIJ67250.1	ABC_transporter_ATP-binding_protein_[Bacillus_cereus]	CPTF_Mn	20243.53333	35062.82826	173.2050808
UIJ67250.1	ABC_transporter_ATP-binding_protein_[Bacillus_cereus]	CPTF_Ni	24254.76667	42010.48819	173.2050808
UIJ67250.1	ABC_transporter_ATP-binding_protein_[Bacillus_cereus]	CPTF_U	13344.8	23113.87162	173.2050808
UIJ67250.1	ABC_transporter_ATP-binding_protein_[Bacillus_cereus]	CPTF_metals_mix	0	0	0
UIJ67250.1	ABC_transporter_ATP-binding_protein_[Bacillus_cereus]	CPTF_zcontrol	0	0	0
UIJ67251.1	ABC_transporter_ATP-binding_protein_[Bacillus_cereus]	CPTF_Al	32861.33333	36937.90364	112.405371
UIJ67251.1	ABC_transporter_ATP-binding_protein_[Bacillus_cereus]	CPTF_Cd	20806.53333	18165.68986	87.30762385
UIJ67251.1	ABC_transporter_ATP-binding_protein_[Bacillus_cereus]	CPTF_Co	19500.66667	17908.49792	91.83531121
UIJ67251.1	ABC_transporter_ATP-binding_protein_[Bacillus_cereus]	CPTF_Cu	39828.33333	14328.23676	35.97498455
UIJ67251.1	ABC_transporter_ATP-binding_protein_[Bacillus_cereus]	CPTF_Fe	22898.76667	39661.8273	173.2050808
UIJ67251.1	ABC_transporter_ATP-binding_protein_[Bacillus_cereus]	CPTF_Mn	18956.1	32832.92831	173.2050808
UIJ67251.1	ABC_transporter_ATP-binding_protein_[Bacillus_cereus]	CPTF_Ni	32743.66667	28374.58705	86.6567185
UIJ67251.1	ABC_transporter_ATP-binding_protein_[Bacillus_cereus]	CPTF_U	18320	31731.17079	173.2050808
UIJ67251.1	ABC_transporter_ATP-binding_protein_[Bacillus_cereus]	CPTF_metals_mix	32520.96667	9499.468963	29.21029089
UIJ67251.1	ABC_transporter_ATP-binding_protein_[Bacillus_cereus]	CPTF_zcontrol	41678.33333	25326.80577	60.767319
UIJ67285.1	DNA_(cytosine-5-)-methyltransferase_[Bacillus_cereus]	CPTF_Al	0	0	0
UIJ67285.1	DNA_(cytosine-5-)-methyltransferase_[Bacillus_cereus]	CPTF_Cd	10619.86667	18394.14864	173.2050808
UIJ67285.1	DNA_(cytosine-5-)-methyltransferase_[Bacillus_cereus]	CPTF_Co	17995.66667	31169.40898	173.2050808
UIJ67285.1	DNA_(cytosine-5-)-methyltransferase_[Bacillus_cereus]	CPTF_Cu	0	0	0
UIJ67285.1	DNA_(cytosine-5-)-methyltransferase_[Bacillus_cereus]	CPTF_Fe	0	0	0
UIJ67285.1	DNA_(cytosine-5-)-methyltransferase_[Bacillus_cereus]	CPTF_Mn	0	0	0
UIJ67285.1	DNA_(cytosine-5-)-methyltransferase_[Bacillus_cereus]	CPTF_Ni	0	0	0
UIJ67285.1	DNA_(cytosine-5-)-methyltransferase_[Bacillus_cereus]	CPTF_U	24166.06667	41856.85529	173.2050808
UIJ67285.1	DNA_(cytosine-5-)-methyltransferase_[Bacillus_cereus]	CPTF_metals_mix	81776.03333	59245.55488	72.44855549
UIJ67285.1	DNA_(cytosine-5-)-methyltransferase_[Bacillus_cereus]	CPTF_zcontrol	0	0	0
UIJ67292.1	DUF262_domain-containing_protein_[Bacillus_cereus]	CPTF_Al	517476.3333	138027.1317	26.67312934
UIJ67292.1	DUF262_domain-containing_protein_[Bacillus_cereus]	CPTF_Cd	630288.6667	115104.1549	18.26213305
UIJ67292.1	DUF262_domain-containing_protein_[Bacillus_cereus]	CPTF_Co	412544.1333	135996.622	32.96535109
UIJ67292.1	DUF262_domain-containing_protein_[Bacillus_cereus]	CPTF_Cu	532230	31965.11267	6.005883297
UIJ67292.1	DUF262_domain-containing_protein_[Bacillus_cereus]	CPTF_Fe	562122.3333	380811.7976	67.74535987

UIJ67292.1	DUF262_domain-containing_protein_[Bacillus_cereus]	CPTF_Mn	452614.6667	70791.08476	15.64047521
UIJ67292.1	DUF262_domain-containing_protein_[Bacillus_cereus]	CPTF_Ni	604854.2	231361.609	38.25080639
UIJ67292.1	DUF262_domain-containing_protein_[Bacillus_cereus]	CPTF_U	599575.4333	284055.2508	47.37606563
UIJ67292.1	DUF262_domain-containing_protein_[Bacillus_cereus]	CPTF_metals_mix	863721.4	43786.33877	5.069497962
UIJ67292.1	DUF262_domain-containing_protein_[Bacillus_cereus]	CPTF_zcontrol	549202.0667	85915.41696	15.64368056
UIJ67293.1	HNH_endonuclease_[Bacillus_cereus]	CPTF_Al	10707.83333	18546.51137	173.2050808
UIJ67293.1	HNH_endonuclease_[Bacillus_cereus]	CPTF_Cd	0	0	0
UIJ67293.1	HNH_endonuclease_[Bacillus_cereus]	CPTF_Co	60060.73333	65225.42166	108.5991097
UIJ67293.1	HNH_endonuclease_[Bacillus_cereus]	CPTF_Cu	15901.23333	27541.74404	173.2050808
UIJ67293.1	HNH_endonuclease_[Bacillus_cereus]	CPTF_Fe	0	0	0
UIJ67293.1	HNH_endonuclease_[Bacillus_cereus]	CPTF_Mn	8258.4	14303.96839	173.2050808
UIJ67293.1	HNH_endonuclease_[Bacillus_cereus]	CPTF_Ni	15585.26667	26994.47372	173.2050808
UIJ67293.1	HNH_endonuclease_[Bacillus_cereus]	CPTF_U	9140.833333	15832.38776	173.2050808
UIJ67293.1	HNH_endonuclease_[Bacillus_cereus]	CPTF_metals_mix	268087	134351.4458	50.11486786
UIJ67293.1	HNH_endonuclease_[Bacillus_cereus]	CPTF_zcontrol	0	0	0
UIJ67318.1	dihydroxyacetone_kinase_subunit_DhaK_[Bacillus_cereus]	CPTF_Al	0	0	0
UIJ67318.1	dihydroxyacetone_kinase_subunit_DhaK_[Bacillus_cereus]	CPTF_Cd	18990.83333	17338.19466	91.29770325
UIJ67318.1	dihydroxyacetone_kinase_subunit_DhaK_[Bacillus_cereus]	CPTF_Co	0	0	0
UIJ67318.1	dihydroxyacetone_kinase_subunit_DhaK_[Bacillus_cereus]	CPTF_Cu	0	0	0
UIJ67318.1	dihydroxyacetone_kinase_subunit_DhaK_[Bacillus_cereus]	CPTF_Fe	0	0	0
UIJ67318.1	dihydroxyacetone_kinase_subunit_DhaK_[Bacillus_cereus]	CPTF_Mn	19712.76667	34143.51342	173.2050808
UIJ67318.1	dihydroxyacetone_kinase_subunit_DhaK_[Bacillus_cereus]	CPTF_Ni	0	0	0
UIJ67318.1	dihydroxyacetone_kinase_subunit_DhaK_[Bacillus_cereus]	CPTF_U	0	0	0
UIJ67318.1	dihydroxyacetone_kinase_subunit_DhaK_[Bacillus_cereus]	CPTF_metals_mix	70836.26667	20679.33283	29.19314329
UIJ67318.1	dihydroxyacetone_kinase_subunit_DhaK_[Bacillus_cereus]	CPTF_zcontrol	0	0	0
UIJ67337.1	heavy_metal-binding_domain-containing_protein_[Bacillus_cereus]	CPTF_Al	292985.1	150675.0628	51.42755137
UIJ67337.1	heavy_metal-binding_domain-containing_protein_[Bacillus_cereus]	CPTF_Cd	467113.9667	111296.3685	23.82638424
UIJ67337.1	heavy_metal-binding_domain-containing_protein_[Bacillus_cereus]	CPTF_Co	335244.0667	76223.35956	22.73667669
UIJ67337.1	heavy_metal-binding_domain-containing_protein_[Bacillus_cereus]	CPTF_Cu	486186.8333	200064.1533	41.14964445
UIJ67337.1	heavy_metal-binding_domain-containing_protein_[Bacillus_cereus]	CPTF_Fe	347811.6667	141785.4804	40.76501565
UIJ67337.1	heavy_metal-binding_domain-containing_protein_[Bacillus_cereus]	CPTF_Mn	371060.6667	38349.79588	10.33518218
UIJ67337.1	heavy_metal-binding_domain-containing_protein_[Bacillus_cereus]	CPTF_Ni	270584.9667	34633.68527	12.79956004
UIJ67337.1	heavy_metal-binding_domain-containing_protein_[Bacillus_cereus]	CPTF_U	431839.5	87608.18788	20.28721038
UIJ67337.1	heavy_metal-binding_domain-containing_protein_[Bacillus_cereus]	CPTF_metals_mix	2280059.667	341316.1216	14.96961359
UIJ67337.1	heavy_metal-binding_domain-containing_protein_[Bacillus_cereus]	CPTF_zcontrol	98179.6	30531.9783	31.09808789
UIJ67340.1	choloylglycine_hydrolase_[Bacillus_cereus]	CPTF_Al	705711.0667	82454.17749	11.68384363
UIJ67340.1	choloylglycine_hydrolase_[Bacillus_cereus]	CPTF_Cd	751582.8	114991.6997	15.29993763
UIJ67340.1	choloylglycine_hydrolase_[Bacillus_cereus]	CPTF_Co	656884.8	4762.102342	0.724952433
UIJ67340.1	choloylglycine_hydrolase_[Bacillus_cereus]	CPTF_Cu	636768.3	86410.18621	13.57011431
UIJ67340.1	choloylglycine_hydrolase_[Bacillus_cereus]	CPTF_Fe	617513.3333	65865.94705	10.66631982
UIJ67340.1	choloylglycine_hydrolase_[Bacillus_cereus]	CPTF_Mn	527764.6667	37059.08414	7.021895644
UIJ67340.1	choloylglycine_hydrolase_[Bacillus_cereus]	CPTF_Ni	538742.2667	45017.9596	8.356121727
UIJ67340.1	choloylglycine_hydrolase_[Bacillus_cereus]	CPTF_U	492945.3333	130854.7705	26.54549331
UIJ67340.1	choloylglycine_hydrolase_[Bacillus_cereus]	CPTF_metals_mix	688611	14672.82757	2.130786115
UIJ67340.1	choloylglycine_hydrolase_[Bacillus_cereus]	CPTF_zcontrol	579019.7333	36844.65734	6.363281806
UIJ67341.1	YflJ_family_protein_[Bacillus_cereus]	CPTF_Al	1767559	503322.0524	28.47554466
UIJ67341.1	YflJ_family_protein_[Bacillus_cereus]	CPTF_Cd	2131684.667	246914.7283	11.58307944
UIJ67341.1	YflJ_family_protein_[Bacillus_cereus]	CPTF_Co	1862450.333	203063.7987	10.90304504
UIJ67341.1	YflJ_family_protein_[Bacillus_cereus]	CPTF_Cu	1896491.333	478902.7129	25.25203804
UIJ67341.1	YflJ_family_protein_[Bacillus_cereus]	CPTF_Fe	2049538.333	254979.1331	12.4408082
UIJ67341.1	YflJ_family_protein_[Bacillus_cereus]	CPTF_Mn	1619934.333	779875.8698	48.14243725
UIJ67341.1	YflJ_family_protein_[Bacillus_cereus]	CPTF_Ni	1151194	449147.213	39.01577084
UIJ67341.1	YflJ_family_protein_[Bacillus_cereus]	CPTF_U	977459	152237.6181	15.57483414
UIJ67341.1	YflJ_family_protein_[Bacillus_cereus]	CPTF_metals_mix	2310589	156965.5958	6.793315288
UIJ67341.1	YflJ_family_protein_[Bacillus_cereus]	CPTF_zcontrol	1633870.333	90944.50841	5.566201097
UIJ67343.1	hypothetical_protein_LW858_03415_[Bacillus_cereus]	CPTF_Al	0	0	0
UIJ67343.1	hypothetical_protein_LW858_03415_[Bacillus_cereus]	CPTF_Cd	7732.766667	13393.54475	173.2050808
UIJ67343.1	hypothetical_protein_LW858_03415_[Bacillus_cereus]	CPTF_Co	18643.4	18656.96476	100.0727591
UIJ67343.1	hypothetical_protein_LW858_03415_[Bacillus_cereus]	CPTF_Cu	0	0	0
UIJ67343.1	hypothetical_protein_LW858_03415_[Bacillus_cereus]	CPTF_Fe	0	0	0
UIJ67343.1	hypothetical_protein_LW858_03415_[Bacillus_cereus]	CPTF_Mn	0	0	0

UIJ67343.1	hypothetical_protein_LW858_03415_[Bacillus_cereus]	CPTF_Ni	0	0	0
UIJ67343.1	hypothetical_protein_LW858_03415_[Bacillus_cereus]	CPTF_U	0	0	0
UIJ67343.1	hypothetical_protein_LW858_03415_[Bacillus_cereus]	CPTF_metals_mix	244801	55102.04462	22.50891321
UIJ67343.1	hypothetical_protein_LW858_03415_[Bacillus_cereus]	CPTF_zcontrol	0	0	0
UIJ67346.1	3'-5'_exoribonuclease_YhaM_[Bacillus_cereus]	CPTF_Al	1601974.567	238839.1783	14.90904932
UIJ67346.1	3'-5'_exoribonuclease_YhaM_[Bacillus_cereus]	CPTF_Cd	1599569.633	555727.7501	34.74232935
UIJ67346.1	3'-5'_exoribonuclease_YhaM_[Bacillus_cereus]	CPTF_Co	1399295.433	349582.706	24.98276616
UIJ67346.1	3'-5'_exoribonuclease_YhaM_[Bacillus_cereus]	CPTF_Cu	1689775.233	197102.1833	11.66440243
UIJ67346.1	3'-5'_exoribonuclease_YhaM_[Bacillus_cereus]	CPTF_Fe	1470798.3	257690.1738	17.52042913
UIJ67346.1	3'-5'_exoribonuclease_YhaM_[Bacillus_cereus]	CPTF_Mn	1549295.267	414950.8771	26.78320176
UIJ67346.1	3'-5'_exoribonuclease_YhaM_[Bacillus_cereus]	CPTF_Ni	1460734.667	394371.632	26.99817024
UIJ67346.1	3'-5'_exoribonuclease_YhaM_[Bacillus_cereus]	CPTF_U	1205345	135321.5828	11.22679256
UIJ67346.1	3'-5'_exoribonuclease_YhaM_[Bacillus_cereus]	CPTF_metals_mix	1330151.833	376787.8738	28.32668154
UIJ67346.1	3'-5'_exoribonuclease_YhaM_[Bacillus_cereus]	CPTF_zcontrol	1541223.1	282292.9929	18.31616674
UIJ67349.1	helix-turn-helix_domain-containing_protein_[Bacillus_cereus]	CPTF_Al	36705.9	31981.9274	87.13020903
UIJ67349.1	helix-turn-helix_domain-containing_protein_[Bacillus_cereus]	CPTF_Cd	40438.96667	36142.94228	89.37652283
UIJ67349.1	helix-turn-helix_domain-containing_protein_[Bacillus_cereus]	CPTF_Co	21110	36563.59255	173.2050808
UIJ67349.1	helix-turn-helix_domain-containing_protein_[Bacillus_cereus]	CPTF_Cu	18580.5	32182.37003	173.2050808
UIJ67349.1	helix-turn-helix_domain-containing_protein_[Bacillus_cereus]	CPTF_Fe	64444.2	68017.86554	105.5453641
UIJ67349.1	helix-turn-helix_domain-containing_protein_[Bacillus_cereus]	CPTF_Mn	18380.76667	31836.42175	173.2050808
UIJ67349.1	helix-turn-helix_domain-containing_protein_[Bacillus_cereus]	CPTF_Ni	24681.36667	42749.38107	173.2050808
UIJ67349.1	helix-turn-helix_domain-containing_protein_[Bacillus_cereus]	CPTF_U	9249.266667	16020.1998	173.2050808
UIJ67349.1	helix-turn-helix_domain-containing_protein_[Bacillus_cereus]	CPTF_metals_mix	70201.23333	38940.87157	55.47035248
UIJ67349.1	helix-turn-helix_domain-containing_protein_[Bacillus_cereus]	CPTF_zcontrol	0	0	0
UIJ67376.1	ring-cleaving_dioxygenase_[Bacillus_cereus]	CPTF_Al	19126.73333	18801.36732	98.29889397
UIJ67376.1	ring-cleaving_dioxygenase_[Bacillus_cereus]	CPTF_Cd	71828.23333	8411.510023	11.71059016
UIJ67376.1	ring-cleaving_dioxygenase_[Bacillus_cereus]	CPTF_Co	22005.26667	3447.707804	15.66764837
UIJ67376.1	ring-cleaving_dioxygenase_[Bacillus_cereus]	CPTF_Cu	27846.6	24119.4542	86.61543673
UIJ67376.1	ring-cleaving_dioxygenase_[Bacillus_cereus]	CPTF_Fe	52932.86667	34360.21358	64.91281456
UIJ67376.1	ring-cleaving_dioxygenase_[Bacillus_cereus]	CPTF_Mn	35440.73333	35192.09254	99.29843216
UIJ67376.1	ring-cleaving_dioxygenase_[Bacillus_cereus]	CPTF_Ni	18522.76667	17162.59531	92.65675924
UIJ67376.1	ring-cleaving_dioxygenase_[Bacillus_cereus]	CPTF_U	48668.83333	6743.146558	13.85516376
UIJ67376.1	ring-cleaving_dioxygenase_[Bacillus_cereus]	CPTF_metals_mix	51614.33333	27505.10096	53.28965654
UIJ67376.1	ring-cleaving_dioxygenase_[Bacillus_cereus]	CPTF_zcontrol	20068.33333	19133.57019	95.34209879
UIJ67377.1	ring-cleaving_dioxygenase_[Bacillus_cereus]	CPTF_Al	19785.43333	19704.94924	99.59321543
UIJ67377.1	ring-cleaving_dioxygenase_[Bacillus_cereus]	CPTF_Cd	27148.4	26238.95782	96.65010763
UIJ67377.1	ring-cleaving_dioxygenase_[Bacillus_cereus]	CPTF_Co	0	0	0
UIJ67377.1	ring-cleaving_dioxygenase_[Bacillus_cereus]	CPTF_Cu	51395.8	18561.2175	36.11426907
UIJ67377.1	ring-cleaving_dioxygenase_[Bacillus_cereus]	CPTF_Fe	14993.5	25969.50378	173.2050808
UIJ67377.1	ring-cleaving_dioxygenase_[Bacillus_cereus]	CPTF_Mn	20736.3	20278.04321	97.79007445
UIJ67377.1	ring-cleaving_dioxygenase_[Bacillus_cereus]	CPTF_Ni	13999.03333	24247.03699	173.2050808
UIJ67377.1	ring-cleaving_dioxygenase_[Bacillus_cereus]	CPTF_U	10202.1	17670.55554	173.2050808
UIJ67377.1	ring-cleaving_dioxygenase_[Bacillus_cereus]	CPTF_metals_mix	20187.1	6183.048919	30.62871298
UIJ67377.1	ring-cleaving_dioxygenase_[Bacillus_cereus]	CPTF_zcontrol	10512.96667	18208.9924	173.2050808
UIJ67383.1	glycerol_kinase_GlpK_[Bacillus_cereus]	CPTF_Al	110391.7667	51064.26905	46.25731664
UIJ67383.1	glycerol_kinase_GlpK_[Bacillus_cereus]	CPTF_Cd	163267.6667	53902.24723	33.01464909
UIJ67383.1	glycerol_kinase_GlpK_[Bacillus_cereus]	CPTF_Co	127663.5333	26045.16164	20.40141062
UIJ67383.1	glycerol_kinase_GlpK_[Bacillus_cereus]	CPTF_Cu	176354	42909.41061	24.33140763
UIJ67383.1	glycerol_kinase_GlpK_[Bacillus_cereus]	CPTF_Fe	163569.8	60776.80654	37.15649621
UIJ67383.1	glycerol_kinase_GlpK_[Bacillus_cereus]	CPTF_Mn	89049.93333	35585.50013	39.96128778
UIJ67383.1	glycerol_kinase_GlpK_[Bacillus_cereus]	CPTF_Ni	152934.6667	9396.318499	6.144008225
UIJ67383.1	glycerol_kinase_GlpK_[Bacillus_cereus]	CPTF_U	82841.1	22825.56905	27.55343549
UIJ67383.1	glycerol_kinase_GlpK_[Bacillus_cereus]	CPTF_metals_mix	224871.3333	13664.25081	6.076475206
UIJ67383.1	glycerol_kinase_GlpK_[Bacillus_cereus]	CPTF_zcontrol	95538	36263.57524	37.95722669
UIJ67386.1	UvrD-helicase_domain-containing_protein_[Bacillus_cereus]	CPTF_Al	120673.3333	107190.6617	88.8271325
UIJ67386.1	UvrD-helicase_domain-containing_protein_[Bacillus_cereus]	CPTF_Cd	0	0	0
UIJ67386.1	UvrD-helicase_domain-containing_protein_[Bacillus_cereus]	CPTF_Co	0	0	0
UIJ67386.1	UvrD-helicase_domain-containing_protein_[Bacillus_cereus]	CPTF_Cu	24205.06667	41924.40527	173.2050808
UIJ67386.1	UvrD-helicase_domain-containing_protein_[Bacillus_cereus]	CPTF_Fe	0	0	0
UIJ67386.1	UvrD-helicase_domain-containing_protein_[Bacillus_cereus]	CPTF_Mn	23922.4	41434.81224	173.2050808
UIJ67386.1	UvrD-helicase_domain-containing_protein_[Bacillus_cereus]	CPTF_Ni	0	0	0

UIJ67386.1	UvrD-helicase_domain-containing_protein_[Bacillus_cereus]	CPTF_U	0	0	0
UIJ67386.1	UvrD-helicase_domain-containing_protein_[Bacillus_cereus]	CPTF_metals_mix	0	0	0
UIJ67386.1	UvrD-helicase_domain-containing_protein_[Bacillus_cereus]	CPTF_zcontrol	0	0	0
UIJ67388.1	peptidylprolyl_isomerase_PrsA_[Bacillus_cereus]	CPTF_Al	1554604.667	122221.6977	7.861915014
UIJ67388.1	peptidylprolyl_isomerase_PrsA_[Bacillus_cereus]	CPTF_Cd	1634679	149440.0139	9.141856837
UIJ67388.1	peptidylprolyl_isomerase_PrsA_[Bacillus_cereus]	CPTF_Co	1591064.667	225700.1654	14.18548033
UIJ67388.1	peptidylprolyl_isomerase_PrsA_[Bacillus_cereus]	CPTF_Cu	1729999.4	261428.2141	15.1114627
UIJ67388.1	peptidylprolyl_isomerase_PrsA_[Bacillus_cereus]	CPTF_Fe	1641568.833	132568.4517	8.075716897
UIJ67388.1	peptidylprolyl_isomerase_PrsA_[Bacillus_cereus]	CPTF_Mn	1728061.567	105722.1586	6.117962498
UIJ67388.1	peptidylprolyl_isomerase_PrsA_[Bacillus_cereus]	CPTF_Ni	1575449.033	233895.7899	14.84629366
UIJ67388.1	peptidylprolyl_isomerase_PrsA_[Bacillus_cereus]	CPTF_U	1932523.033	143690.7888	7.435398511
UIJ67388.1	peptidylprolyl_isomerase_PrsA_[Bacillus_cereus]	CPTF_metals_mix	1842723.9	175768.5787	9.538519511
UIJ67388.1	peptidylprolyl_isomerase_PrsA_[Bacillus_cereus]	CPTF_zcontrol	1921770.567	108214.3667	5.630972217
UIJ67391.1	HTH-type_transcriptional_regulator_Hpr_[Bacillus_cereus]	CPTF_Al	1414910.667	202415.1589	14.3058614
UIJ67391.1	HTH-type_transcriptional_regulator_Hpr_[Bacillus_cereus]	CPTF_Cd	1061926.267	58346.52232	5.494404287
UIJ67391.1	HTH-type_transcriptional_regulator_Hpr_[Bacillus_cereus]	CPTF_Co	1465376.033	399694.8617	27.27592458
UIJ67391.1	HTH-type_transcriptional_regulator_Hpr_[Bacillus_cereus]	CPTF_Cu	1395616.933	103108.6521	7.388033895
UIJ67391.1	HTH-type_transcriptional_regulator_Hpr_[Bacillus_cereus]	CPTF_Fe	1293186.4	142651.893	11.03103876
UIJ67391.1	HTH-type_transcriptional_regulator_Hpr_[Bacillus_cereus]	CPTF_Mn	1186455	109435.6746	9.22375266
UIJ67391.1	HTH-type_transcriptional_regulator_Hpr_[Bacillus_cereus]	CPTF_Ni	1450460.133	64937.55129	4.477031102
UIJ67391.1	HTH-type_transcriptional_regulator_Hpr_[Bacillus_cereus]	CPTF_U	1270319.067	212991.5755	16.76677782
UIJ67391.1	HTH-type_transcriptional_regulator_Hpr_[Bacillus_cereus]	CPTF_metals_mix	1292686.233	239103.3815	18.49662937
UIJ67391.1	HTH-type_transcriptional_regulator_Hpr_[Bacillus_cereus]	CPTF_zcontrol	1328900.033	113403.8151	8.533660338
UIJ67392.1	YtxH_domain-containing_protein_[Bacillus_cereus]	CPTF_Al	856308.7	598046.3534	69.84004173
UIJ67392.1	YtxH_domain-containing_protein_[Bacillus_cereus]	CPTF_Cd	1493715.333	213892.3236	14.31948369
UIJ67392.1	YtxH_domain-containing_protein_[Bacillus_cereus]	CPTF_Co	633449.3	607515.7003	95.90597074
UIJ67392.1	YtxH_domain-containing_protein_[Bacillus_cereus]	CPTF_Cu	655172	392228.2748	59.86645871
UIJ67392.1	YtxH_domain-containing_protein_[Bacillus_cereus]	CPTF_Fe	785854.3333	570211.9626	72.55949842
UIJ67392.1	YtxH_domain-containing_protein_[Bacillus_cereus]	CPTF_Mn	951600.7	840346.5574	88.30873678
UIJ67392.1	YtxH_domain-containing_protein_[Bacillus_cereus]	CPTF_Ni	631799.5333	260861.2205	41.28860608
UIJ67392.1	YtxH_domain-containing_protein_[Bacillus_cereus]	CPTF_U	486570.9333	332713.2532	68.37918799
UIJ67392.1	YtxH_domain-containing_protein_[Bacillus_cereus]	CPTF_metals_mix	1976299.3	57204.78548	2.894540593
UIJ67392.1	YtxH_domain-containing_protein_[Bacillus_cereus]	CPTF_zcontrol	616028.9667	162311.6343	26.34805229
UIJ67393.1	HIT_family_protein_[Bacillus_cereus]	CPTF_Al	796799.6667	82231.70226	10.32024808
UIJ67393.1	HIT_family_protein_[Bacillus_cereus]	CPTF_Cd	924438.6667	35464.86115	3.83636713
UIJ67393.1	HIT_family_protein_[Bacillus_cereus]	CPTF_Co	643266.3333	44502.526	6.918211586
UIJ67393.1	HIT_family_protein_[Bacillus_cereus]	CPTF_Cu	626277.3333	134412.9169	21.46220368
UIJ67393.1	HIT_family_protein_[Bacillus_cereus]	CPTF_Fe	731452.8333	197579.1782	27.01188227
UIJ67393.1	HIT_family_protein_[Bacillus_cereus]	CPTF_Mn	603892.0333	210506.2357	34.85825678
UIJ67393.1	HIT_family_protein_[Bacillus_cereus]	CPTF_Ni	886861	64318.06579	7.25232768
UIJ67393.1	HIT_family_protein_[Bacillus_cereus]	CPTF_U	624582.4667	128461.2928	20.56754707
UIJ67393.1	HIT_family_protein_[Bacillus_cereus]	CPTF_metals_mix	691413.3333	165951.6098	24.00179484
UIJ67393.1	HIT_family_protein_[Bacillus_cereus]	CPTF_zcontrol	477570.3	220750.7777	46.22372407
UIJ67394.1	ABC_transporter_ATP-binding_protein_EcsA_[Bacillus_cereus]	CPTF_Al	123457.1667	37961.29531	30.74855542
UIJ67394.1	ABC_transporter_ATP-binding_protein_EcsA_[Bacillus_cereus]	CPTF_Cd	146441.9	38940.75727	26.59126744
UIJ67394.1	ABC_transporter_ATP-binding_protein_EcsA_[Bacillus_cereus]	CPTF_Co	132569	27865.28186	21.01945542
UIJ67394.1	ABC_transporter_ATP-binding_protein_EcsA_[Bacillus_cereus]	CPTF_Cu	119018.8	20266.88817	17.02830828
UIJ67394.1	ABC_transporter_ATP-binding_protein_EcsA_[Bacillus_cereus]	CPTF_Fe	145113.6667	15838.73165	10.91470708
UIJ67394.1	ABC_transporter_ATP-binding_protein_EcsA_[Bacillus_cereus]	CPTF_Mn	161277.4	66796.87844	41.41738299
UIJ67394.1	ABC_transporter_ATP-binding_protein_EcsA_[Bacillus_cereus]	CPTF_Ni	147482.6667	22871.24387	15.50775043
UIJ67394.1	ABC_transporter_ATP-binding_protein_EcsA_[Bacillus_cereus]	CPTF_U	54659	94672.16509	173.2050808
UIJ67394.1	ABC_transporter_ATP-binding_protein_EcsA_[Bacillus_cereus]	CPTF_metals_mix	389852.7333	49202.09199	12.62068668
UIJ67394.1	ABC_transporter_ATP-binding_protein_EcsA_[Bacillus_cereus]	CPTF_zcontrol	158710.6667	35988.19643	22.67534828
UIJ67406.1	heme-degrading_monooxygenase_HmoB_[Bacillus_cereus]	CPTF_Al	871457.2667	151076.852	17.33611707
UIJ67406.1	heme-degrading_monooxygenase_HmoB_[Bacillus_cereus]	CPTF_Cd	1485388.867	228353.0558	15.37328446
UIJ67406.1	heme-degrading_monooxygenase_HmoB_[Bacillus_cereus]	CPTF_Co	816979.0333	158669.0232	19.42143149
UIJ67406.1	heme-degrading_monooxygenase_HmoB_[Bacillus_cereus]	CPTF_Cu	813486.6667	300955.0567	36.99569631
UIJ67406.1	heme-degrading_monooxygenase_HmoB_[Bacillus_cereus]	CPTF_Fe	908811	139109.0053	15.30670351
UIJ67406.1	heme-degrading_monooxygenase_HmoB_[Bacillus_cereus]	CPTF_Mn	959143.8	546096.0748	56.9357874
UIJ67406.1	heme-degrading_monooxygenase_HmoB_[Bacillus_cereus]	CPTF_Ni	410562.9	252628.6077	61.5322543
UIJ67406.1	heme-degrading_monooxygenase_HmoB_[Bacillus_cereus]	CPTF_U	454356.4667	98319.49345	21.6392856

UIJ67406.1	heme-degrading_monooxygenase_HmoB_[Bacillus_cereus]	CPTF_metals_mix	1717486.167	159031.7014	9.259562289
UIJ67406.1	heme-degrading_monooxygenase_HmoB_[Bacillus_cereus]	CPTF_zcontrol	606243.5667	226602.945	37.37820201
UIJ67408.1	uroporphyrinogen_decarboxylase_[Bacillus_cereus]	CPTF_Al	1196434	25123.07839	2.099829861
UIJ67408.1	uroporphyrinogen_decarboxylase_[Bacillus_cereus]	CPTF_Cd	1244040.667	86132.11341	6.923576996
UIJ67408.1	uroporphyrinogen_decarboxylase_[Bacillus_cereus]	CPTF_Co	1053240.267	29028.70965	2.756133674
UIJ67408.1	uroporphyrinogen_decarboxylase_[Bacillus_cereus]	CPTF_Cu	1046786.333	100029.2605	9.555843187
UIJ67408.1	uroporphyrinogen_decarboxylase_[Bacillus_cereus]	CPTF_Fe	1200148.567	62628.67727	5.218410371
UIJ67408.1	uroporphyrinogen_decarboxylase_[Bacillus_cereus]	CPTF_Mn	1186666.667	98149.54576	8.271029137
UIJ67408.1	uroporphyrinogen_decarboxylase_[Bacillus_cereus]	CPTF_Ni	1203333.333	90737.71726	7.54053052
UIJ67408.1	uroporphyrinogen_decarboxylase_[Bacillus_cereus]	CPTF_U	1269831.367	17468.38034	1.37564568
UIJ67408.1	uroporphyrinogen_decarboxylase_[Bacillus_cereus]	CPTF_metals_mix	871207.1333	96086.93564	11.02917228
UIJ67408.1	uroporphyrinogen_decarboxylase_[Bacillus_cereus]	CPTF_zcontrol	1323333.333	5773.502692	0.436284838
UIJ67409.1	ferrochelatase_[Bacillus_cereus]	CPTF_Al	397775.7667	214576.2898	53.94403275
UIJ67409.1	ferrochelatase_[Bacillus_cereus]	CPTF_Cd	367677.8	61675.03278	16.77420633
UIJ67409.1	ferrochelatase_[Bacillus_cereus]	CPTF_Co	467098.2	210944.2582	45.16058041
UIJ67409.1	ferrochelatase_[Bacillus_cereus]	CPTF_Cu	451343.8667	180784.3631	40.05468478
UIJ67409.1	ferrochelatase_[Bacillus_cereus]	CPTF_Fe	320235.2	184640.0066	57.65762372
UIJ67409.1	ferrochelatase_[Bacillus_cereus]	CPTF_Mn	218260.1333	116784.6254	53.50708056
UIJ67409.1	ferrochelatase_[Bacillus_cereus]	CPTF_Ni	215615.1	30031.14794	13.92812838
UIJ67409.1	ferrochelatase_[Bacillus_cereus]	CPTF_U	72512.33333	96502.99355	133.0849376
UIJ67409.1	ferrochelatase_[Bacillus_cereus]	CPTF_metals_mix	644122.9333	173730.958	26.9717082
UIJ67409.1	ferrochelatase_[Bacillus_cereus]	CPTF_zcontrol	182229	26504.22847	14.54446245
UIJ67411.1	DUF4026_domain-containing_protein_[Bacillus_cereus]	CPTF_Al	23173.86667	21598.85501	93.20350084
UIJ67411.1	DUF4026_domain-containing_protein_[Bacillus_cereus]	CPTF_Cd	54218.9	15041.55784	27.74227776
UIJ67411.1	DUF4026_domain-containing_protein_[Bacillus_cereus]	CPTF_Co	0	0	0
UIJ67411.1	DUF4026_domain-containing_protein_[Bacillus_cereus]	CPTF_Cu	0	0	0
UIJ67411.1	DUF4026_domain-containing_protein_[Bacillus_cereus]	CPTF_Fe	29994.06667	16014.81886	53.39328955
UIJ67411.1	DUF4026_domain-containing_protein_[Bacillus_cereus]	CPTF_Mn	0	0	0
UIJ67411.1	DUF4026_domain-containing_protein_[Bacillus_cereus]	CPTF_Ni	0	0	0
UIJ67411.1	DUF4026_domain-containing_protein_[Bacillus_cereus]	CPTF_U	0	0	0
UIJ67411.1	DUF4026_domain-containing_protein_[Bacillus_cereus]	CPTF_metals_mix	121898.7	19983.97302	16.39391808
UIJ67411.1	DUF4026_domain-containing_protein_[Bacillus_cereus]	CPTF_zcontrol	0	0	0
UIJ67412.1	DUF6359_domain-containing_protein_[Bacillus_cereus]	CPTF_Al	0	0	0
UIJ67412.1	DUF6359_domain-containing_protein_[Bacillus_cereus]	CPTF_Cd	0	0	0
UIJ67412.1	DUF6359_domain-containing_protein_[Bacillus_cereus]	CPTF_Co	0	0	0
UIJ67412.1	DUF6359_domain-containing_protein_[Bacillus_cereus]	CPTF_Cu	0	0	0
UIJ67412.1	DUF6359_domain-containing_protein_[Bacillus_cereus]	CPTF_Fe	21682.43333	37555.07617	173.2050808
UIJ67412.1	DUF6359_domain-containing_protein_[Bacillus_cereus]	CPTF_Mn	0	0	0
UIJ67412.1	DUF6359_domain-containing_protein_[Bacillus_cereus]	CPTF_Ni	0	0	0
UIJ67412.1	DUF6359_domain-containing_protein_[Bacillus_cereus]	CPTF_U	21314	36916.93091	173.2050808
UIJ67412.1	DUF6359_domain-containing_protein_[Bacillus_cereus]	CPTF_metals_mix	170470	24408.58892	14.3184073
UIJ67412.1	DUF6359_domain-containing_protein_[Bacillus_cereus]	CPTF_zcontrol	0	0	0
UIJ67416.1	TetR/AcrR_family_transcriptional_regulator_[Bacillus_cereus]	CPTF_Al	76337.5	16686.39184	21.85870881
UIJ67416.1	TetR/AcrR_family_transcriptional_regulator_[Bacillus_cereus]	CPTF_Cd	46818.63333	41360.43713	88.34182927
UIJ67416.1	TetR/AcrR_family_transcriptional_regulator_[Bacillus_cereus]	CPTF_Co	66394.8	14724.90658	22.17780095
UIJ67416.1	TetR/AcrR_family_transcriptional_regulator_[Bacillus_cereus]	CPTF_Cu	42565.36667	39118.39886	91.90194264
UIJ67416.1	TetR/AcrR_family_transcriptional_regulator_[Bacillus_cereus]	CPTF_Fe	39663.16667	35881.9548	90.46669193
UIJ67416.1	TetR/AcrR_family_transcriptional_regulator_[Bacillus_cereus]	CPTF_Mn	13140.3	22759.66723	173.2050808
UIJ67416.1	TetR/AcrR_family_transcriptional_regulator_[Bacillus_cereus]	CPTF_Ni	34020.86667	33971.3587	99.85447765
UIJ67416.1	TetR/AcrR_family_transcriptional_regulator_[Bacillus_cereus]	CPTF_U	0	0	0
UIJ67416.1	TetR/AcrR_family_transcriptional_regulator_[Bacillus_cereus]	CPTF_metals_mix	65249.5	57461.82131	88.06476878
UIJ67416.1	TetR/AcrR_family_transcriptional_regulator_[Bacillus_cereus]	CPTF_zcontrol	0	0	0
UIJ67425.1	lipoate--protein_ligase_[Bacillus_cereus]	CPTF_Al	404955.8333	109043.2573	26.92719757
UIJ67425.1	lipoate--protein_ligase_[Bacillus_cereus]	CPTF_Cd	307013.8667	94435.02835	30.75920621
UIJ67425.1	lipoate--protein_ligase_[Bacillus_cereus]	CPTF_Co	557998.7	122699.9365	21.98928716
UIJ67425.1	lipoate--protein_ligase_[Bacillus_cereus]	CPTF_Cu	706443.4333	19484.49594	2.758111268
UIJ67425.1	lipoate--protein_ligase_[Bacillus_cereus]	CPTF_Fe	461410.6667	174550.4858	37.82974656
UIJ67425.1	lipoate--protein_ligase_[Bacillus_cereus]	CPTF_Mn	413214.6333	30233.40071	7.316633601
UIJ67425.1	lipoate--protein_ligase_[Bacillus_cereus]	CPTF_Ni	502898.8333	98028.93437	19.49277427
UIJ67425.1	lipoate--protein_ligase_[Bacillus_cereus]	CPTF_U	570150.1333	40543.78486	7.111071714
UIJ67425.1	lipoate--protein_ligase_[Bacillus_cereus]	CPTF_metals_mix	899048.1667	136491.8287	15.18181492

UIJ67425.1	lipoate--protein_ligase_[Bacillus_cereus]	CPTF_zcontrol	514396	49802.57406	9.681757646
UIJ67427.1	fatty_acid--CoA_ligase_family_protein_[Bacillus_cereus]	CPTF_Al	37677.66667	65259.63298	173.2050808
UIJ67427.1	fatty_acid--CoA_ligase_family_protein_[Bacillus_cereus]	CPTF_Cd	66346.23333	61943.48669	93.36398403
UIJ67427.1	fatty_acid--CoA_ligase_family_protein_[Bacillus_cereus]	CPTF_Co	53569.66667	92785.38441	173.2050808
UIJ67427.1	fatty_acid--CoA_ligase_family_protein_[Bacillus_cereus]	CPTF_Cu	33071.43333	57281.40281	173.2050808
UIJ67427.1	fatty_acid--CoA_ligase_family_protein_[Bacillus_cereus]	CPTF_Fe	42022.66667	72785.39374	173.2050808
UIJ67427.1	fatty_acid--CoA_ligase_family_protein_[Bacillus_cereus]	CPTF_Mn	0	0	0
UIJ67427.1	fatty_acid--CoA_ligase_family_protein_[Bacillus_cereus]	CPTF_Ni	37018.66667	64118.2115	173.2050808
UIJ67427.1	fatty_acid--CoA_ligase_family_protein_[Bacillus_cereus]	CPTF_U	0	0	0
UIJ67427.1	fatty_acid--CoA_ligase_family_protein_[Bacillus_cereus]	CPTF_metals_mix	0	0	0
UIJ67427.1	fatty_acid--CoA_ligase_family_protein_[Bacillus_cereus]	CPTF_zcontrol	0	0	0
UIJ67457.1	AraC_family_transcriptional_regulator_[Bacillus_cereus]	CPTF_Al	0	0	0
UIJ67457.1	AraC_family_transcriptional_regulator_[Bacillus_cereus]	CPTF_Cd	36177.33333	62660.97942	173.2050808
UIJ67457.1	AraC_family_transcriptional_regulator_[Bacillus_cereus]	CPTF_Co	31958.83333	55354.32308	173.2050808
UIJ67457.1	AraC_family_transcriptional_regulator_[Bacillus_cereus]	CPTF_Cu	5960.866667	10324.52392	173.2050808
UIJ67457.1	AraC_family_transcriptional_regulator_[Bacillus_cereus]	CPTF_Fe	33470.33333	57972.31788	173.2050808
UIJ67457.1	AraC_family_transcriptional_regulator_[Bacillus_cereus]	CPTF_Mn	0	0	0
UIJ67457.1	AraC_family_transcriptional_regulator_[Bacillus_cereus]	CPTF_Ni	0	0	0
UIJ67457.1	AraC_family_transcriptional_regulator_[Bacillus_cereus]	CPTF_U	0	0	0
UIJ67457.1	AraC_family_transcriptional_regulator_[Bacillus_cereus]	CPTF_metals_mix	74956.5	25494.92694	34.01296344
UIJ67457.1	AraC_family_transcriptional_regulator_[Bacillus_cereus]	CPTF_zcontrol	41768	72344.29813	173.2050808
UIJ67468.1	isocitrate_lyase_[Bacillus_cereus]	CPTF_Al	56872.3	98505.71314	173.2050808
UIJ67468.1	isocitrate_lyase_[Bacillus_cereus]	CPTF_Cd	69003.93333	66612.22047	96.53394706
UIJ67468.1	isocitrate_lyase_[Bacillus_cereus]	CPTF_Co	145861.8333	175794.1722	120.521022
UIJ67468.1	isocitrate_lyase_[Bacillus_cereus]	CPTF_Cu	66602	18838.6536	28.28541725
UIJ67468.1	isocitrate_lyase_[Bacillus_cereus]	CPTF_Fe	96890.33333	167818.9801	173.2050808
UIJ67468.1	isocitrate_lyase_[Bacillus_cereus]	CPTF_Mn	81101.5	97460.20119	120.1706518
UIJ67468.1	isocitrate_lyase_[Bacillus_cereus]	CPTF_Ni	15376.6	26633.05245	173.2050808
UIJ67468.1	isocitrate_lyase_[Bacillus_cereus]	CPTF_U	48434.33333	45133.42144	93.1847686
UIJ67468.1	isocitrate_lyase_[Bacillus_cereus]	CPTF_metals_mix	83369.7	62198.63811	74.60580776
UIJ67468.1	isocitrate_lyase_[Bacillus_cereus]	CPTF_zcontrol	28453.66667	49283.19633	173.2050808
UIJ67470.1	RNA_chaperone/antiterminator_CspA_[Bacillus_cereus]	CPTF_Al	1184801	463854.9378	39.15045124
UIJ67470.1	RNA_chaperone/antiterminator_CspA_[Bacillus_cereus]	CPTF_Cd	1652326.667	132088.0554	7.99406425
UIJ67470.1	RNA_chaperone/antiterminator_CspA_[Bacillus_cereus]	CPTF_Co	1961996.333	71468.25895	3.642629588
UIJ67470.1	RNA_chaperone/antiterminator_CspA_[Bacillus_cereus]	CPTF_Cu	1317758.667	90972.593	6.903585254
UIJ67470.1	RNA_chaperone/antiterminator_CspA_[Bacillus_cereus]	CPTF_Fe	1943301.667	431649.0648	22.21214916
UIJ67470.1	RNA_chaperone/antiterminator_CspA_[Bacillus_cereus]	CPTF_Mn	1134856.333	795833.5519	70.12637006
UIJ67470.1	RNA_chaperone/antiterminator_CspA_[Bacillus_cereus]	CPTF_Ni	1371482.333	389850.7758	28.42550475
UIJ67470.1	RNA_chaperone/antiterminator_CspA_[Bacillus_cereus]	CPTF_U	1227214.667	161229.8198	13.13786611
UIJ67470.1	RNA_chaperone/antiterminator_CspA_[Bacillus_cereus]	CPTF_metals_mix	3726525	702428.7735	18.8494314
UIJ67470.1	RNA_chaperone/antiterminator_CspA_[Bacillus_cereus]	CPTF_zcontrol	1367093	82699.51687	6.049297076
UIJ67477.1	helicase-exonuclease_AddAB_subunit_AddA_[Bacillus_cereus]	CPTF_Al	301480	53649.68731	17.79543827
UIJ67477.1	helicase-exonuclease_AddAB_subunit_AddA_[Bacillus_cereus]	CPTF_Cd	230448.6667	65047.72341	28.22655664
UIJ67477.1	helicase-exonuclease_AddAB_subunit_AddA_[Bacillus_cereus]	CPTF_Co	400088	244122.5906	61.01722385
UIJ67477.1	helicase-exonuclease_AddAB_subunit_AddA_[Bacillus_cereus]	CPTF_Cu	222381.6667	59711.39734	26.85086331
UIJ67477.1	helicase-exonuclease_AddAB_subunit_AddA_[Bacillus_cereus]	CPTF_Fe	314707.3333	238517.6407	75.79030274
UIJ67477.1	helicase-exonuclease_AddAB_subunit_AddA_[Bacillus_cereus]	CPTF_Mn	301863.3333	202007.0992	66.92005187
UIJ67477.1	helicase-exonuclease_AddAB_subunit_AddA_[Bacillus_cereus]	CPTF_Ni	185817.3333	27307.19151	14.69571811
UIJ67477.1	helicase-exonuclease_AddAB_subunit_AddA_[Bacillus_cereus]	CPTF_U	171817.3333	60360.46152	35.13060082
UIJ67477.1	helicase-exonuclease_AddAB_subunit_AddA_[Bacillus_cereus]	CPTF_metals_mix	163151.9333	84074.6708	51.53151978
UIJ67477.1	helicase-exonuclease_AddAB_subunit_AddA_[Bacillus_cereus]	CPTF_zcontrol	281451	186153.1363	66.14051339
UIJ67488.1	ornithine_aminotransferase_[Bacillus_cereus]	CPTF_Al	11548239.57	492978.162	4.268859848
UIJ67488.1	ornithine_aminotransferase_[Bacillus_cereus]	CPTF_Cd	11082377.97	570137.7506	5.144543458
UIJ67488.1	ornithine_aminotransferase_[Bacillus_cereus]	CPTF_Co	11513725.33	429977.9177	3.734481284
UIJ67488.1	ornithine_aminotransferase_[Bacillus_cereus]	CPTF_Cu	12395903.27	227624.3756	1.836287124
UIJ67488.1	ornithine_aminotransferase_[Bacillus_cereus]	CPTF_Fe	10684757.57	121217.845	1.134493171
UIJ67488.1	ornithine_aminotransferase_[Bacillus_cereus]	CPTF_Mn	11366105.63	1114930.669	9.809258375
UIJ67488.1	ornithine_aminotransferase_[Bacillus_cereus]	CPTF_Ni	10734398.1	1177269.264	10.96725921
UIJ67488.1	ornithine_aminotransferase_[Bacillus_cereus]	CPTF_U	9985574.567	413166.7789	4.137636509
UIJ67488.1	ornithine_aminotransferase_[Bacillus_cereus]	CPTF_metals_mix	13831839.4	622267.7539	4.498806962
UIJ67488.1	ornithine_aminotransferase_[Bacillus_cereus]	CPTF_zcontrol	10922378.7	521125.1835	4.771169338

UIJ67493.1	catalase_[Bacillus_cereus]	CPTF_Al	96368.53333	103379.0111	107.2746544
UIJ67493.1	catalase_[Bacillus_cereus]	CPTF_Cd	104933.4667	47478.90116	45.24667169
UIJ67493.1	catalase_[Bacillus_cereus]	CPTF_Co	122867.4	74927.87563	60.9827144
UIJ67493.1	catalase_[Bacillus_cereus]	CPTF_Cu	242655.9	229261.6178	94.48013331
UIJ67493.1	catalase_[Bacillus_cereus]	CPTF_Fe	15278.73333	26463.54241	173.2050808
UIJ67493.1	catalase_[Bacillus_cereus]	CPTF_Mn	0	0	0
UIJ67493.1	catalase_[Bacillus_cereus]	CPTF_Ni	21411.86667	37086.44095	173.2050808
UIJ67493.1	catalase_[Bacillus_cereus]	CPTF_U	47619.96667	42522.06826	89.29461996
UIJ67493.1	catalase_[Bacillus_cereus]	CPTF_metals_mix	138779.6667	36940.81219	26.61831742
UIJ67493.1	catalase_[Bacillus_cereus]	CPTF_zcontrol	27167.6	24640.56356	90.69834494
UIJ67496.1	YajQ_family_cyclic_di-GMP-binding_protein_[Bacillus_cereus]	CPTF_Al	2599214.233	341897.6354	13.15388439
UIJ67496.1	YajQ_family_cyclic_di-GMP-binding_protein_[Bacillus_cereus]	CPTF_Cd	2408007	942240.5427	39.1294769
UIJ67496.1	YajQ_family_cyclic_di-GMP-binding_protein_[Bacillus_cereus]	CPTF_Co	2211178.967	344318.0864	15.57169689
UIJ67496.1	YajQ_family_cyclic_di-GMP-binding_protein_[Bacillus_cereus]	CPTF_Cu	2699820.167	312827.8933	11.58699002
UIJ67496.1	YajQ_family_cyclic_di-GMP-binding_protein_[Bacillus_cereus]	CPTF_Fe	2286016.633	999159.8174	43.70746052
UIJ67496.1	YajQ_family_cyclic_di-GMP-binding_protein_[Bacillus_cereus]	CPTF_Mn	2395481	769314.2706	32.11523158
UIJ67496.1	YajQ_family_cyclic_di-GMP-binding_protein_[Bacillus_cereus]	CPTF_Ni	1044628.833	1128808.309	108.0583144
UIJ67496.1	YajQ_family_cyclic_di-GMP-binding_protein_[Bacillus_cereus]	CPTF_U	1662079.7	1405786.259	84.57995478
UIJ67496.1	YajQ_family_cyclic_di-GMP-binding_protein_[Bacillus_cereus]	CPTF_metals_mix	3619694	233985.2334	6.464226904
UIJ67496.1	YajQ_family_cyclic_di-GMP-binding_protein_[Bacillus_cereus]	CPTF_zcontrol	2598508.6	199265.6123	7.668460759
UIJ67497.1	S1-like_domain-containing_RNA-binding_protein_[Bacillus_cereus]	CPTF_Al	117457.2	115567.1045	98.3908219
UIJ67497.1	S1-like_domain-containing_RNA-binding_protein_[Bacillus_cereus]	CPTF_Cd	9643.133333	16702.39688	173.2050808
UIJ67497.1	S1-like_domain-containing_RNA-binding_protein_[Bacillus_cereus]	CPTF_Co	93032.3	59563.30263	64.02432556
UIJ67497.1	S1-like_domain-containing_RNA-binding_protein_[Bacillus_cereus]	CPTF_Cu	17912.5	31025.36009	173.2050808
UIJ67497.1	S1-like_domain-containing_RNA-binding_protein_[Bacillus_cereus]	CPTF_Fe	0	0	0
UIJ67497.1	S1-like_domain-containing_RNA-binding_protein_[Bacillus_cereus]	CPTF_Mn	9464.666667	16393.28354	173.2050808
UIJ67497.1	S1-like_domain-containing_RNA-binding_protein_[Bacillus_cereus]	CPTF_Ni	54209.73333	48870.86812	90.1514638
UIJ67497.1	S1-like_domain-containing_RNA-binding_protein_[Bacillus_cereus]	CPTF_U	41006.5	71025.34144	173.2050808
UIJ67497.1	S1-like_domain-containing_RNA-binding_protein_[Bacillus_cereus]	CPTF_metals_mix	120414.9	39376.22751	32.70046108
UIJ67497.1	S1-like_domain-containing_RNA-binding_protein_[Bacillus_cereus]	CPTF_zcontrol	28253.16667	24486.99989	86.66993042
UIJ67509.1	ATP-dependent_chaperone_ClpB_[Bacillus_cereus]	CPTF_Al	690076.6667	68778.53306	9.96679592
UIJ67509.1	ATP-dependent_chaperone_ClpB_[Bacillus_cereus]	CPTF_Cd	1008212.8	209441.9667	20.77358735
UIJ67509.1	ATP-dependent_chaperone_ClpB_[Bacillus_cereus]	CPTF_Co	856751.3333	185225.7434	21.61954539
UIJ67509.1	ATP-dependent_chaperone_ClpB_[Bacillus_cereus]	CPTF_Cu	920372.6333	109874.5699	11.93805269
UIJ67509.1	ATP-dependent_chaperone_ClpB_[Bacillus_cereus]	CPTF_Fe	905299.6667	63814.17626	7.048956121
UIJ67509.1	ATP-dependent_chaperone_ClpB_[Bacillus_cereus]	CPTF_Mn	651618.9	204068.6833	31.31718299
UIJ67509.1	ATP-dependent_chaperone_ClpB_[Bacillus_cereus]	CPTF_Ni	745211.6667	93186.56213	12.50471058
UIJ67509.1	ATP-dependent_chaperone_ClpB_[Bacillus_cereus]	CPTF_U	762172.5	132209.8595	17.34644841
UIJ67509.1	ATP-dependent_chaperone_ClpB_[Bacillus_cereus]	CPTF_metals_mix	1645118.547	60017.47199	3.648215633
UIJ67509.1	ATP-dependent_chaperone_ClpB_[Bacillus_cereus]	CPTF_zcontrol	1581801.6	1695446.806	107.1845424
UIJ67513.1	ComZ_family_protein_[Bacillus_cereus]	CPTF_Al	0	0	0
UIJ67513.1	ComZ_family_protein_[Bacillus_cereus]	CPTF_Cd	0	0	0
UIJ67513.1	ComZ_family_protein_[Bacillus_cereus]	CPTF_Co	0	0	0
UIJ67513.1	ComZ_family_protein_[Bacillus_cereus]	CPTF_Cu	26160.53333	45311.37289	173.2050808
UIJ67513.1	ComZ_family_protein_[Bacillus_cereus]	CPTF_Fe	0	0	0
UIJ67513.1	ComZ_family_protein_[Bacillus_cereus]	CPTF_Mn	0	0	0
UIJ67513.1	ComZ_family_protein_[Bacillus_cereus]	CPTF_Ni	8736.233333	15131.6	173.2050808
UIJ67513.1	ComZ_family_protein_[Bacillus_cereus]	CPTF_U	0	0	0
UIJ67513.1	ComZ_family_protein_[Bacillus_cereus]	CPTF_metals_mix	38959.5	36190.2474	92.8919709
UIJ67513.1	ComZ_family_protein_[Bacillus_cereus]	CPTF_zcontrol	0	0	0
UIJ67514.1	beta-ketoacyl-ACP_synthase_III_[Bacillus_cereus]	CPTF_Al	2572233.367	89194.16851	3.467576841
UIJ67514.1	beta-ketoacyl-ACP_synthase_III_[Bacillus_cereus]	CPTF_Cd	2444130.767	87971.32655	3.599288866
UIJ67514.1	beta-ketoacyl-ACP_synthase_III_[Bacillus_cereus]	CPTF_Co	2659667.733	63932.53996	2.403779207
UIJ67514.1	beta-ketoacyl-ACP_synthase_III_[Bacillus_cereus]	CPTF_Cu	2828888.667	91702.85405	3.241656525
UIJ67514.1	beta-ketoacyl-ACP_synthase_III_[Bacillus_cereus]	CPTF_Fe	2377442.733	245107.4515	10.30971001
UIJ67514.1	beta-ketoacyl-ACP_synthase_III_[Bacillus_cereus]	CPTF_Mn	2401263.033	268731.3238	11.19124894
UIJ67514.1	beta-ketoacyl-ACP_synthase_III_[Bacillus_cereus]	CPTF_Ni	2563141.4	56102.84562	2.188831471
UIJ67514.1	beta-ketoacyl-ACP_synthase_III_[Bacillus_cereus]	CPTF_U	2204189.467	161174.3406	7.312181779
UIJ67514.1	beta-ketoacyl-ACP_synthase_III_[Bacillus_cereus]	CPTF_metals_mix	2362385.867	97827.76557	4.141057858
UIJ67514.1	beta-ketoacyl-ACP_synthase_III_[Bacillus_cereus]	CPTF_zcontrol	2461736	220974.3816	8.976363898
UIJ67515.1	beta-ketoacyl-ACP_synthase_II_[Bacillus_cereus]	CPTF_Al	466377.3667	75476.3957	16.18354601

UIJ67515.1	beta-ketoacyl-ACP_synthase_II_[Bacillus_cereus]	CPTF_Cd	1310458.667	513330.3974	39.1718114
UIJ67515.1	beta-ketoacyl-ACP_synthase_II_[Bacillus_cereus]	CPTF_Co	842881.06	436085.925	51.73753993
UIJ67515.1	beta-ketoacyl-ACP_synthase_II_[Bacillus_cereus]	CPTF_Cu	1019417.8	662844.1561	65.02183463
UIJ67515.1	beta-ketoacyl-ACP_synthase_II_[Bacillus_cereus]	CPTF_Fe	1251761.333	747488.569	59.71494319
UIJ67515.1	beta-ketoacyl-ACP_synthase_II_[Bacillus_cereus]	CPTF_Mn	1179049.933	680602.3021	57.72463769
UIJ67515.1	beta-ketoacyl-ACP_synthase_II_[Bacillus_cereus]	CPTF_Ni	397703.3333	78032.90765	19.62088349
UIJ67515.1	beta-ketoacyl-ACP_synthase_II_[Bacillus_cereus]	CPTF_U	946898.3333	386583.3812	40.82628173
UIJ67515.1	beta-ketoacyl-ACP_synthase_II_[Bacillus_cereus]	CPTF_metals_mix	1770387.1	1148480.602	64.87172226
UIJ67515.1	beta-ketoacyl-ACP_synthase_II_[Bacillus_cereus]	CPTF_zcontrol	1428678.933	102146.5569	7.149720943
UIJ67518.1	tryptophan--tRNA_ligase_[Bacillus_cereus]	CPTF_Al	2013175.633	324618.5382	16.12470034
UIJ67518.1	tryptophan--tRNA_ligase_[Bacillus_cereus]	CPTF_Cd	2425644.267	366371.1644	15.10407645
UIJ67518.1	tryptophan--tRNA_ligase_[Bacillus_cereus]	CPTF_Co	2145668.633	721531.2213	33.62733695
UIJ67518.1	tryptophan--tRNA_ligase_[Bacillus_cereus]	CPTF_Cu	1676894.733	299491.6773	17.85989731
UIJ67518.1	tryptophan--tRNA_ligase_[Bacillus_cereus]	CPTF_Fe	2101624.933	322942.4669	15.36632259
UIJ67518.1	tryptophan--tRNA_ligase_[Bacillus_cereus]	CPTF_Mn	2115083.1	415237.3732	19.63220137
UIJ67518.1	tryptophan--tRNA_ligase_[Bacillus_cereus]	CPTF_Ni	2162498.467	664013.1873	30.7058339
UIJ67518.1	tryptophan--tRNA_ligase_[Bacillus_cereus]	CPTF_U	1411255.667	415018.9252	29.40777741
UIJ67518.1	tryptophan--tRNA_ligase_[Bacillus_cereus]	CPTF_metals_mix	1472040.167	239952.2246	16.30065742
UIJ67518.1	tryptophan--tRNA_ligase_[Bacillus_cereus]	CPTF_zcontrol	1643488.3	440597.3938	26.80867237
UIJ67520.1	ABC_transporter_permease_[Bacillus_cereus]	CPTF_Al	920895.6667	143787.0746	15.61382899
UIJ67520.1	ABC_transporter_permease_[Bacillus_cereus]	CPTF_Cd	802418.2667	88178.03759	10.98903667
UIJ67520.1	ABC_transporter_permease_[Bacillus_cereus]	CPTF_Co	1037846.667	200583.1563	19.32685846
UIJ67520.1	ABC_transporter_permease_[Bacillus_cereus]	CPTF_Cu	977234.9333	37971.05893	3.885560947
UIJ67520.1	ABC_transporter_permease_[Bacillus_cereus]	CPTF_Fe	740850.7333	46988.92156	6.342562604
UIJ67520.1	ABC_transporter_permease_[Bacillus_cereus]	CPTF_Mn	905119.6333	72764.60162	8.039224755
UIJ67520.1	ABC_transporter_permease_[Bacillus_cereus]	CPTF_Ni	803334.6667	189259.8804	23.5592821
UIJ67520.1	ABC_transporter_permease_[Bacillus_cereus]	CPTF_U	681086.3	206168.0345	30.27047153
UIJ67520.1	ABC_transporter_permease_[Bacillus_cereus]	CPTF_metals_mix	641880.1333	122003.906	19.00727249
UIJ67520.1	ABC_transporter_permease_[Bacillus_cereus]	CPTF_zcontrol	712768.6667	115281.8671	16.17381242
UIJ67521.1	ABC_transporter_permease_[Bacillus_cereus]	CPTF_Al	2794059.9	141765.0144	5.073800113
UIJ67521.1	ABC_transporter_permease_[Bacillus_cereus]	CPTF_Cd	2843788.467	98398.42879	3.460117725
UIJ67521.1	ABC_transporter_permease_[Bacillus_cereus]	CPTF_Co	2765156.333	138071.2007	4.993251162
UIJ67521.1	ABC_transporter_permease_[Bacillus_cereus]	CPTF_Cu	2557659.967	219531.2161	8.5832839
UIJ67521.1	ABC_transporter_permease_[Bacillus_cereus]	CPTF_Fe	2776243.7	119024.1737	4.287237956
UIJ67521.1	ABC_transporter_permease_[Bacillus_cereus]	CPTF_Mn	2827242.533	417059.1805	14.75144688
UIJ67521.1	ABC_transporter_permease_[Bacillus_cereus]	CPTF_Ni	2604235.8	286191.9386	10.9894787
UIJ67521.1	ABC_transporter_permease_[Bacillus_cereus]	CPTF_U	2658577.3	479105.3057	18.02111624
UIJ67521.1	ABC_transporter_permease_[Bacillus_cereus]	CPTF_metals_mix	2057007.733	167315.2462	8.133914301
UIJ67521.1	ABC_transporter_permease_[Bacillus_cereus]	CPTF_zcontrol	2726649.733	241303.4519	8.849814811
UIJ67522.1	ABC_transporter_ATP-binding_protein_[Bacillus_cereus]	CPTF_Al	3837562.467	1015698.617	26.46728557
UIJ67522.1	ABC_transporter_ATP-binding_protein_[Bacillus_cereus]	CPTF_Cd	5083541.167	1076958.569	21.18520405
UIJ67522.1	ABC_transporter_ATP-binding_protein_[Bacillus_cereus]	CPTF_Co	4430212.767	625901.2031	14.12801678
UIJ67522.1	ABC_transporter_ATP-binding_protein_[Bacillus_cereus]	CPTF_Cu	3295362.033	1020861.968	30.97875006
UIJ67522.1	ABC_transporter_ATP-binding_protein_[Bacillus_cereus]	CPTF_Fe	4054839.5	865683.4102	21.34938782
UIJ67522.1	ABC_transporter_ATP-binding_protein_[Bacillus_cereus]	CPTF_Mn	3015453.333	299349.3312	9.927175059
UIJ67522.1	ABC_transporter_ATP-binding_protein_[Bacillus_cereus]	CPTF_Ni	2683467.567	1133437.746	42.23780308
UIJ67522.1	ABC_transporter_ATP-binding_protein_[Bacillus_cereus]	CPTF_U	2980791.867	906848.0387	30.42305801
UIJ67522.1	ABC_transporter_ATP-binding_protein_[Bacillus_cereus]	CPTF_metals_mix	3438490.767	539158.2633	15.68008466
UIJ67522.1	ABC_transporter_ATP-binding_protein_[Bacillus_cereus]	CPTF_zcontrol	2970559.433	731361.283	24.62032151
UIJ67523.1	ATP-binding_cassette_domain-containing_protein_[Bacillus_cereus]	CPTF_Al	6422418.833	349396.6785	5.44026616
UIJ67523.1	ATP-binding_cassette_domain-containing_protein_[Bacillus_cereus]	CPTF_Cd	6648492.133	745660.4889	11.21548276
UIJ67523.1	ATP-binding_cassette_domain-containing_protein_[Bacillus_cereus]	CPTF_Co	5803713	426581.981	7.350156374
UIJ67523.1	ATP-binding_cassette_domain-containing_protein_[Bacillus_cereus]	CPTF_Cu	5623925.033	579861.2834	10.31061545
UIJ67523.1	ATP-binding_cassette_domain-containing_protein_[Bacillus_cereus]	CPTF_Fe	6886548.2	328145.7289	4.765024789
UIJ67523.1	ATP-binding_cassette_domain-containing_protein_[Bacillus_cereus]	CPTF_Mn	6148985.867	1291220.709	20.9989214
UIJ67523.1	ATP-binding_cassette_domain-containing_protein_[Bacillus_cereus]	CPTF_Ni	6272116.033	903801.6222	14.40983581
UIJ67523.1	ATP-binding_cassette_domain-containing_protein_[Bacillus_cereus]	CPTF_U	6952509.6	374048.0602	5.380043779
UIJ67523.1	ATP-binding_cassette_domain-containing_protein_[Bacillus_cereus]	CPTF_metals_mix	4676561.733	313073.2187	6.694516968
UIJ67523.1	ATP-binding_cassette_domain-containing_protein_[Bacillus_cereus]	CPTF_zcontrol	6349630.567	345367.4889	5.439174536
UIJ67531.1	oligoendopeptidase_F_[Bacillus_cereus]	CPTF_Al	13980889.53	558229.7245	3.992805488
UIJ67531.1	oligoendopeptidase_F_[Bacillus_cereus]	CPTF_Cd	12927149	304971.7595	2.359157147

UIJ67531.1	oligoendopeptidase_F_[Bacillus_cereus]	CPTF_Co	13362775	188766.2634	1.412627717
UIJ67531.1	oligoendopeptidase_F_[Bacillus_cereus]	CPTF_Cu	12592967.2	395144.9177	3.137822179
UIJ67531.1	oligoendopeptidase_F_[Bacillus_cereus]	CPTF_Fe	12503553.96	861051.3993	6.88645326
UIJ67531.1	oligoendopeptidase_F_[Bacillus_cereus]	CPTF_Mn	13424834.37	509862.4934	3.797905281
UIJ67531.1	oligoendopeptidase_F_[Bacillus_cereus]	CPTF_Ni	11837751.07	882297.6947	7.453254336
UIJ67531.1	oligoendopeptidase_F_[Bacillus_cereus]	CPTF_U	11806399.17	1639922.133	13.89011256
UIJ67531.1	oligoendopeptidase_F_[Bacillus_cereus]	CPTF_metals_mix	9834640.477	477563.4389	4.855931847
UIJ67531.1	oligoendopeptidase_F_[Bacillus_cereus]	CPTF_zcontrol	12595082.63	583819.9692	4.635300825
UIJ67546.1	AAA_family_ATPase_[Bacillus_cereus]	CPTF_Al	37432	64834.12583	173.2050808
UIJ67546.1	AAA_family_ATPase_[Bacillus_cereus]	CPTF_Cd	86545.33333	77559.61414	89.61732673
UIJ67546.1	AAA_family_ATPase_[Bacillus_cereus]	CPTF_Co	32450.43333	56205.79926	173.2050808
UIJ67546.1	AAA_family_ATPase_[Bacillus_cereus]	CPTF_Cu	78603.5	15946.09958	20.28675515
UIJ67546.1	AAA_family_ATPase_[Bacillus_cereus]	CPTF_Fe	106015.5667	32611.48728	30.76103661
UIJ67546.1	AAA_family_ATPase_[Bacillus_cereus]	CPTF_Mn	0	0	0
UIJ67546.1	AAA_family_ATPase_[Bacillus_cereus]	CPTF_Ni	0	0	0
UIJ67546.1	AAA_family_ATPase_[Bacillus_cereus]	CPTF_U	0	0	0
UIJ67546.1	AAA_family_ATPase_[Bacillus_cereus]	CPTF_metals_mix	83160.66667	73281.54754	88.1204426
UIJ67546.1	AAA_family_ATPase_[Bacillus_cereus]	CPTF_zcontrol	0	0	0
UIJ67587.1	NAD_kinase_[Bacillus_cereus]	CPTF_Al	108624.2333	43467.43379	40.01633195
UIJ67587.1	NAD_kinase_[Bacillus_cereus]	CPTF_Cd	0	0	0
UIJ67587.1	NAD_kinase_[Bacillus_cereus]	CPTF_Co	96189	86797.45309	90.2363608
UIJ67587.1	NAD_kinase_[Bacillus_cereus]	CPTF_Cu	219127	59115.25949	26.97762461
UIJ67587.1	NAD_kinase_[Bacillus_cereus]	CPTF_Fe	94197.33333	90652.90123	96.2372267
UIJ67587.1	NAD_kinase_[Bacillus_cereus]	CPTF_Mn	57494.66667	99583.68383	173.2050808
UIJ67587.1	NAD_kinase_[Bacillus_cereus]	CPTF_Ni	117253.1333	43956.91841	37.48890726
UIJ67587.1	NAD_kinase_[Bacillus_cereus]	CPTF_U	149254.6667	76771.83368	51.43680623
UIJ67587.1	NAD_kinase_[Bacillus_cereus]	CPTF_metals_mix	65755.66667	57009.22364	86.69857144
UIJ67587.1	NAD_kinase_[Bacillus_cereus]	CPTF_zcontrol	156214.6667	45794.31057	29.31498786
UIJ67605.1	enoyl-ACP_reductase_FabI_[Bacillus_cereus]	CPTF_Al	19696437.67	416458.0668	2.114382681
UIJ67605.1	enoyl-ACP_reductase_FabI_[Bacillus_cereus]	CPTF_Cd	21536307	592554.406	2.751420687
UIJ67605.1	enoyl-ACP_reductase_FabI_[Bacillus_cereus]	CPTF_Co	18725769.07	938684.6163	5.012796073
UIJ67605.1	enoyl-ACP_reductase_FabI_[Bacillus_cereus]	CPTF_Cu	17268582.67	1666208.335	9.648784545
UIJ67605.1	enoyl-ACP_reductase_FabI_[Bacillus_cereus]	CPTF_Fe	21142397.03	1726959.236	8.168228195
UIJ67605.1	enoyl-ACP_reductase_FabI_[Bacillus_cereus]	CPTF_Mn	20435840.63	299918.9139	1.467612315
UIJ67605.1	enoyl-ACP_reductase_FabI_[Bacillus_cereus]	CPTF_Ni	17795415.33	354614.403	1.992729006
UIJ67605.1	enoyl-ACP_reductase_FabI_[Bacillus_cereus]	CPTF_U	18842615.67	409733.7114	2.174505486
UIJ67605.1	enoyl-ACP_reductase_FabI_[Bacillus_cereus]	CPTF_metals_mix	16594219.27	1397834.091	8.423620713
UIJ67605.1	enoyl-ACP_reductase_FabI_[Bacillus_cereus]	CPTF_zcontrol	20020916.53	618968.384	3.091608633
UIJ67612.1	GNAT_family_N-acetyltransferase_[Bacillus_cereus]	CPTF_Al	0	0	0
UIJ67612.1	GNAT_family_N-acetyltransferase_[Bacillus_cereus]	CPTF_Cd	0	0	0
UIJ67612.1	GNAT_family_N-acetyltransferase_[Bacillus_cereus]	CPTF_Co	10024.06667	17362.19277	173.2050808
UIJ67612.1	GNAT_family_N-acetyltransferase_[Bacillus_cereus]	CPTF_Cu	47935.16667	26083.99674	54.41515814
UIJ67612.1	GNAT_family_N-acetyltransferase_[Bacillus_cereus]	CPTF_Fe	19399.4	33600.74644	173.2050808
UIJ67612.1	GNAT_family_N-acetyltransferase_[Bacillus_cereus]	CPTF_Mn	0	0	0
UIJ67612.1	GNAT_family_N-acetyltransferase_[Bacillus_cereus]	CPTF_Ni	0	0	0
UIJ67612.1	GNAT_family_N-acetyltransferase_[Bacillus_cereus]	CPTF_U	0	0	0
UIJ67612.1	GNAT_family_N-acetyltransferase_[Bacillus_cereus]	CPTF_metals_mix	0	0	0
UIJ67612.1	GNAT_family_N-acetyltransferase_[Bacillus_cereus]	CPTF_zcontrol	0	0	0
UIJ67613.1	YjcG_family_protein_[Bacillus_cereus]	CPTF_Al	7281787.033	416872.2787	5.724862273
UIJ67613.1	YjcG_family_protein_[Bacillus_cereus]	CPTF_Cd	6777481.667	208216.3492	3.072178715
UIJ67613.1	YjcG_family_protein_[Bacillus_cereus]	CPTF_Co	6845172.667	377178.0194	5.51013156
UIJ67613.1	YjcG_family_protein_[Bacillus_cereus]	CPTF_Cu	7337644.133	527312.0346	7.18639423
UIJ67613.1	YjcG_family_protein_[Bacillus_cereus]	CPTF_Fe	6496247.667	449426.323	6.918244902
UIJ67613.1	YjcG_family_protein_[Bacillus_cereus]	CPTF_Mn	7628256.667	941069.0006	12.33661952
UIJ67613.1	YjcG_family_protein_[Bacillus_cereus]	CPTF_Ni	7083835.667	515613.1102	7.278727718
UIJ67613.1	YjcG_family_protein_[Bacillus_cereus]	CPTF_U	4531694.2	712253.8294	15.71716444
UIJ67613.1	YjcG_family_protein_[Bacillus_cereus]	CPTF_metals_mix	9474777.667	1131812.236	11.94552818
UIJ67613.1	YjcG_family_protein_[Bacillus_cereus]	CPTF_zcontrol	7720455.333	541891.9098	7.018911274
UIJ67614.1	esterase_family_protein_[Bacillus_cereus]	CPTF_Al	886776.6667	170657.0249	19.24464539
UIJ67614.1	esterase_family_protein_[Bacillus_cereus]	CPTF_Cd	884993	92698.91301	10.4745363
UIJ67614.1	esterase_family_protein_[Bacillus_cereus]	CPTF_Co	645123.2	128032.5673	19.84621965

UIJ67614.1	esterase_family_protein_[Bacillus_cereus]	CPTF_Cu	949177.6667	121042.6676	12.75237207
UIJ67614.1	esterase_family_protein_[Bacillus_cereus]	CPTF_Fe	745197	155455.7133	20.86102243
UIJ67614.1	esterase_family_protein_[Bacillus_cereus]	CPTF_Mn	712915.3333	105260.2652	14.76476382
UIJ67614.1	esterase_family_protein_[Bacillus_cereus]	CPTF_Ni	664728.3333	304658.6213	45.83204989
UIJ67614.1	esterase_family_protein_[Bacillus_cereus]	CPTF_U	568710	227850.2688	40.06440345
UIJ67614.1	esterase_family_protein_[Bacillus_cereus]	CPTF_metals_mix	760261.1667	95245.9432	12.5280558
UIJ67614.1	esterase_family_protein_[Bacillus_cereus]	CPTF_zcontrol	677336	181515.8458	26.79849377
UIJ67617.1	sodium/proline_symporter_PutP_[Bacillus_cereus]	CPTF_Al	0	0	0
UIJ67617.1	sodium/proline_symporter_PutP_[Bacillus_cereus]	CPTF_Cd	24817.46667	42985.11318	173.2050808
UIJ67617.1	sodium/proline_symporter_PutP_[Bacillus_cereus]	CPTF_Co	65805.66667	113978.7581	173.2050808
UIJ67617.1	sodium/proline_symporter_PutP_[Bacillus_cereus]	CPTF_Cu	0	0	0
UIJ67617.1	sodium/proline_symporter_PutP_[Bacillus_cereus]	CPTF_Fe	0	0	0
UIJ67617.1	sodium/proline_symporter_PutP_[Bacillus_cereus]	CPTF_Mn	29198.93333	50574.03606	173.2050808
UIJ67617.1	sodium/proline_symporter_PutP_[Bacillus_cereus]	CPTF_Ni	0	0	0
UIJ67617.1	sodium/proline_symporter_PutP_[Bacillus_cereus]	CPTF_U	0	0	0
UIJ67617.1	sodium/proline_symporter_PutP_[Bacillus_cereus]	CPTF_metals_mix	22090.5	38261.86836	173.2050808
UIJ67617.1	sodium/proline_symporter_PutP_[Bacillus_cereus]	CPTF_zcontrol	0	0	0
UIJ67618.1	anthranilate_synthase_component_I_[Bacillus_cereus]	CPTF_Al	441193.3667	94859.43466	21.50064843
UIJ67618.1	anthranilate_synthase_component_I_[Bacillus_cereus]	CPTF_Cd	345719.3333	67038.19728	19.39093097
UIJ67618.1	anthranilate_synthase_component_I_[Bacillus_cereus]	CPTF_Co	653930.6667	260818.4033	39.88471815
UIJ67618.1	anthranilate_synthase_component_I_[Bacillus_cereus]	CPTF_Cu	323728.8667	63589.84342	19.6429327
UIJ67618.1	anthranilate_synthase_component_I_[Bacillus_cereus]	CPTF_Fe	648190.1667	410096.2139	63.26788571
UIJ67618.1	anthranilate_synthase_component_I_[Bacillus_cereus]	CPTF_Mn	691640.8	338892.5739	48.99834912
UIJ67618.1	anthranilate_synthase_component_I_[Bacillus_cereus]	CPTF_Ni	536156.6667	48470.65658	9.040390541
UIJ67618.1	anthranilate_synthase_component_I_[Bacillus_cereus]	CPTF_U	381665.0333	311619.0444	81.64726061
UIJ67618.1	anthranilate_synthase_component_I_[Bacillus_cereus]	CPTF_metals_mix	6327.566667	10959.66695	173.2050808
UIJ67618.1	anthranilate_synthase_component_I_[Bacillus_cereus]	CPTF_zcontrol	442873.8333	129143.2764	29.16028599
UIJ67619.1	aminodeoxychorismate/anthranilate_synthase_component_II_[Bacillus_cereus]	CPTF_Al	0	0	0
UIJ67619.1	aminodeoxychorismate/anthranilate_synthase_component_II_[Bacillus_cereus]	CPTF_Cd	67253	116485.613	173.2050808
UIJ67619.1	aminodeoxychorismate/anthranilate_synthase_component_II_[Bacillus_cereus]	CPTF_Co	149142.6	132365.5123	88.75097544
UIJ67619.1	aminodeoxychorismate/anthranilate_synthase_component_II_[Bacillus_cereus]	CPTF_Cu	0	0	0
UIJ67619.1	aminodeoxychorismate/anthranilate_synthase_component_II_[Bacillus_cereus]	CPTF_Fe	134117.4667	116389.1105	86.78147102
UIJ67619.1	aminodeoxychorismate/anthranilate_synthase_component_II_[Bacillus_cereus]	CPTF_Mn	57464.2	51825.53541	90.18751746
UIJ67619.1	aminodeoxychorismate/anthranilate_synthase_component_II_[Bacillus_cereus]	CPTF_Ni	142135.6667	123421.3508	86.83348357
UIJ67619.1	aminodeoxychorismate/anthranilate_synthase_component_II_[Bacillus_cereus]	CPTF_U	0	0	0
UIJ67619.1	aminodeoxychorismate/anthranilate_synthase_component_II_[Bacillus_cereus]	CPTF_metals_mix	0	0	0
UIJ67619.1	aminodeoxychorismate/anthranilate_synthase_component_II_[Bacillus_cereus]	CPTF_zcontrol	0	0	0
UIJ67620.1	anthranilate_phosphoribosyltransferase_[Bacillus_cereus]	CPTF_Al	675660.4	50049.89716	7.40755225
UIJ67620.1	anthranilate_phosphoribosyltransferase_[Bacillus_cereus]	CPTF_Cd	470503.3333	308023.5874	65.46682362
UIJ67620.1	anthranilate_phosphoribosyltransferase_[Bacillus_cereus]	CPTF_Co	576298.8667	191298.5044	33.1943225
UIJ67620.1	anthranilate_phosphoribosyltransferase_[Bacillus_cereus]	CPTF_Cu	404618.6667	32178.77497	7.952864666
UIJ67620.1	anthranilate_phosphoribosyltransferase_[Bacillus_cereus]	CPTF_Fe	735860.7	102072.512	13.87117317
UIJ67620.1	anthranilate_phosphoribosyltransferase_[Bacillus_cereus]	CPTF_Mn	665636.3333	360821.8996	54.20706196
UIJ67620.1	anthranilate_phosphoribosyltransferase_[Bacillus_cereus]	CPTF_Ni	818089.1333	100936.9968	12.33814174
UIJ67620.1	anthranilate_phosphoribosyltransferase_[Bacillus_cereus]	CPTF_U	580112.4667	203261.5554	35.03830155
UIJ67620.1	anthranilate_phosphoribosyltransferase_[Bacillus_cereus]	CPTF_metals_mix	0	0	0
UIJ67620.1	anthranilate_phosphoribosyltransferase_[Bacillus_cereus]	CPTF_zcontrol	426130.8667	263868.2713	61.92188643
UIJ67621.1	indole-3-glycerol_phosphate_synthase_TrpC_[Bacillus_cereus]	CPTF_Al	1539731.333	113352.3142	7.361824217
UIJ67621.1	indole-3-glycerol_phosphate_synthase_TrpC_[Bacillus_cereus]	CPTF_Cd	1716466	393747.0578	22.93940328
UIJ67621.1	indole-3-glycerol_phosphate_synthase_TrpC_[Bacillus_cereus]	CPTF_Co	1834825.333	275224.335	15.00002916
UIJ67621.1	indole-3-glycerol_phosphate_synthase_TrpC_[Bacillus_cereus]	CPTF_Cu	1185528	121601.0802	10.25712427
UIJ67621.1	indole-3-glycerol_phosphate_synthase_TrpC_[Bacillus_cereus]	CPTF_Fe	1906484.4	460671.4676	24.16340085
UIJ67621.1	indole-3-glycerol_phosphate_synthase_TrpC_[Bacillus_cereus]	CPTF_Mn	1816830.667	888241.02	48.88958758
UIJ67621.1	indole-3-glycerol_phosphate_synthase_TrpC_[Bacillus_cereus]	CPTF_Ni	1213637.333	112019.2522	9.23004337
UIJ67621.1	indole-3-glycerol_phosphate_synthase_TrpC_[Bacillus_cereus]	CPTF_U	1229406	475422.6783	38.67092549
UIJ67621.1	indole-3-glycerol_phosphate_synthase_TrpC_[Bacillus_cereus]	CPTF_metals_mix	73034.33333	126499.176	173.2050808
UIJ67621.1	indole-3-glycerol_phosphate_synthase_TrpC_[Bacillus_cereus]	CPTF_zcontrol	1426286.933	303003.0557	21.24418647
UIJ67623.1	tryptophan_synthase_subunit_beta_[Bacillus_cereus]	CPTF_Al	2756100.067	143646.108	5.211933692
UIJ67623.1	tryptophan_synthase_subunit_beta_[Bacillus_cereus]	CPTF_Cd	1787125.3	628475.7313	35.16685323
UIJ67623.1	tryptophan_synthase_subunit_beta_[Bacillus_cereus]	CPTF_Co	2635192.633	359929.7849	13.65857586
UIJ67623.1	tryptophan_synthase_subunit_beta_[Bacillus_cereus]	CPTF_Cu	1291000.833	556498.4616	43.1059723

UIJ67623.1	tryptophan_synthase_subunit_beta_[Bacillus_cereus]	CPTF_Fe	2342316.2	393521.9637	16.80054827
UIJ67623.1	tryptophan_synthase_subunit_beta_[Bacillus_cereus]	CPTF_Mn	3053137.733	1382766.735	45.29002148
UIJ67623.1	tryptophan_synthase_subunit_beta_[Bacillus_cereus]	CPTF_Ni	2057307.333	145212.2934	7.058366589
UIJ67623.1	tryptophan_synthase_subunit_beta_[Bacillus_cereus]	CPTF_U	1960443.333	1170433.632	59.70249751
UIJ67623.1	tryptophan_synthase_subunit_beta_[Bacillus_cereus]	CPTF_metals_mix	0	0	0
UIJ67623.1	tryptophan_synthase_subunit_beta_[Bacillus_cereus]	CPTF_zcontrol	1973408.387	355915.3623	18.03556551
UIJ67624.1	tryptophan_synthase_subunit_alpha_[Bacillus_cereus]	CPTF_Al	0	0	0
UIJ67624.1	tryptophan_synthase_subunit_alpha_[Bacillus_cereus]	CPTF_Cd	63496	109978.2981	173.2050808
UIJ67624.1	tryptophan_synthase_subunit_alpha_[Bacillus_cereus]	CPTF_Co	98476.33333	170566.0127	173.2050808
UIJ67624.1	tryptophan_synthase_subunit_alpha_[Bacillus_cereus]	CPTF_Cu	0	0	0
UIJ67624.1	tryptophan_synthase_subunit_alpha_[Bacillus_cereus]	CPTF_Fe	9747.833333	16883.7426	173.2050808
UIJ67624.1	tryptophan_synthase_subunit_alpha_[Bacillus_cereus]	CPTF_Mn	203992.6333	353325.6053	173.2050808
UIJ67624.1	tryptophan_synthase_subunit_alpha_[Bacillus_cereus]	CPTF_Ni	0	0	0
UIJ67624.1	tryptophan_synthase_subunit_alpha_[Bacillus_cereus]	CPTF_U	0	0	0
UIJ67624.1	tryptophan_synthase_subunit_alpha_[Bacillus_cereus]	CPTF_metals_mix	6303.766667	10918.44415	173.2050808
UIJ67624.1	tryptophan_synthase_subunit_alpha_[Bacillus_cereus]	CPTF_zcontrol	11311.03333	19591.28442	173.2050808
UIJ67632.1	CoA-disulfide_reductase_[Bacillus_cereus]	CPTF_Al	21971.86667	38056.3894	173.2050808
UIJ67632.1	CoA-disulfide_reductase_[Bacillus_cereus]	CPTF_Cd	52489.66667	90914.76954	173.2050808
UIJ67632.1	CoA-disulfide_reductase_[Bacillus_cereus]	CPTF_Co	0	0	0
UIJ67632.1	CoA-disulfide_reductase_[Bacillus_cereus]	CPTF_Cu	39142.33333	67796.51006	173.2050808
UIJ67632.1	CoA-disulfide_reductase_[Bacillus_cereus]	CPTF_Fe	0	0	0
UIJ67632.1	CoA-disulfide_reductase_[Bacillus_cereus]	CPTF_Mn	41091.66667	71172.85443	173.2050808
UIJ67632.1	CoA-disulfide_reductase_[Bacillus_cereus]	CPTF_Ni	0	0	0
UIJ67632.1	CoA-disulfide_reductase_[Bacillus_cereus]	CPTF_U	0	0	0
UIJ67632.1	CoA-disulfide_reductase_[Bacillus_cereus]	CPTF_metals_mix	99677.66667	88844.21819	89.13151879
UIJ67632.1	CoA-disulfide_reductase_[Bacillus_cereus]	CPTF_zcontrol	28655.93333	49633.53247	173.2050808
UIJ67637.1	2-oxoglutarate_dehydrogenase_complex_dihydrolipoyllysine-residue_succinyltransferase_[Bacillus_cereus]	CPTF_Al	12022233.97	666064.3943	5.540271435
UIJ67637.1	2-oxoglutarate_dehydrogenase_complex_dihydrolipoyllysine-residue_succinyltransferase_[Bacillus_cereus]	CPTF_Cd	13018343.4	400306.5395	3.074942235
UIJ67637.1	2-oxoglutarate_dehydrogenase_complex_dihydrolipoyllysine-residue_succinyltransferase_[Bacillus_cereus]	CPTF_Co	11203277.77	1528315.187	13.64167897
UIJ67637.1	2-oxoglutarate_dehydrogenase_complex_dihydrolipoyllysine-residue_succinyltransferase_[Bacillus_cereus]	CPTF_Cu	11945636.23	506465.8325	4.23975603
UIJ67637.1	2-oxoglutarate_dehydrogenase_complex_dihydrolipoyllysine-residue_succinyltransferase_[Bacillus_cereus]	CPTF_Fe	11961036.43	600197.7108	5.017940662
UIJ67637.1	2-oxoglutarate_dehydrogenase_complex_dihydrolipoyllysine-residue_succinyltransferase_[Bacillus_cereus]	CPTF_Mn	12601177.83	960561.7009	7.622792993
UIJ67637.1	2-oxoglutarate_dehydrogenase_complex_dihydrolipoyllysine-residue_succinyltransferase_[Bacillus_cereus]	CPTF_Ni	13074994.57	874050.8871	6.6849044
UIJ67637.1	2-oxoglutarate_dehydrogenase_complex_dihydrolipoyllysine-residue_succinyltransferase_[Bacillus_cereus]	CPTF_U	12984910.2	1243461.271	9.576202312
UIJ67637.1	2-oxoglutarate_dehydrogenase_complex_dihydrolipoyllysine-residue_succinyltransferase_[Bacillus_cereus]	CPTF_metals_mix	7006409.4	375179.703	5.354807028
UIJ67637.1	2-oxoglutarate_dehydrogenase_complex_dihydrolipoyllysine-residue_succinyltransferase_[Bacillus_cereus]	CPTF_zcontrol	13322419.33	693431.1982	5.204994535
UIJ67638.1	2-oxoglutarate_dehydrogenase_E1_component_[Bacillus_cereus]	CPTF_Al	6119088.367	406077.8835	6.636248068
UIJ67638.1	2-oxoglutarate_dehydrogenase_E1_component_[Bacillus_cereus]	CPTF_Cd	7204866.367	565807.8792	7.853134957
UIJ67638.1	2-oxoglutarate_dehydrogenase_E1_component_[Bacillus_cereus]	CPTF_Co	6250535.567	1176874.696	18.82838171
UIJ67638.1	2-oxoglutarate_dehydrogenase_E1_component_[Bacillus_cereus]	CPTF_Cu	7290901.5	736239.6201	10.09806017
UIJ67638.1	2-oxoglutarate_dehydrogenase_E1_component_[Bacillus_cereus]	CPTF_Fe	6204595.7	369961.3488	5.962698726
UIJ67638.1	2-oxoglutarate_dehydrogenase_E1_component_[Bacillus_cereus]	CPTF_Mn	6358231.267	537235.0176	8.449441285
UIJ67638.1	2-oxoglutarate_dehydrogenase_E1_component_[Bacillus_cereus]	CPTF_Ni	6497231.3	117945.3231	1.815316673
UIJ67638.1	2-oxoglutarate_dehydrogenase_E1_component_[Bacillus_cereus]	CPTF_U	6302276.6	1224192.506	19.4246077
UIJ67638.1	2-oxoglutarate_dehydrogenase_E1_component_[Bacillus_cereus]	CPTF_metals_mix	5967801.5	462768.0262	7.754413852
UIJ67638.1	2-oxoglutarate_dehydrogenase_E1_component_[Bacillus_cereus]	CPTF_zcontrol	6667679.567	470709.114	7.059564115
UIJ67651.1	CalY_family_protein_[Bacillus_cereus]	CPTF_Al	5249391.867	755578.0698	14.39363052
UIJ67651.1	CalY_family_protein_[Bacillus_cereus]	CPTF_Cd	5051204.333	155624.3856	3.080936255
UIJ67651.1	CalY_family_protein_[Bacillus_cereus]	CPTF_Co	4625414.667	434173.1163	9.386685251
UIJ67651.1	CalY_family_protein_[Bacillus_cereus]	CPTF_Cu	2720949.667	49070.01808	1.803415134
UIJ67651.1	CalY_family_protein_[Bacillus_cereus]	CPTF_Fe	4870405.133	571980.8575	11.74400983
UIJ67651.1	CalY_family_protein_[Bacillus_cereus]	CPTF_Mn	4907336.333	758854.2302	15.46366865
UIJ67651.1	CalY_family_protein_[Bacillus_cereus]	CPTF_Ni	4697069.333	564870.6763	12.02602381
UIJ67651.1	CalY_family_protein_[Bacillus_cereus]	CPTF_U	5127080	1328766.906	25.91664078
UIJ67651.1	CalY_family_protein_[Bacillus_cereus]	CPTF_metals_mix	1840579.367	333919.9639	18.14211166
UIJ67651.1	CalY_family_protein_[Bacillus_cereus]	CPTF_zcontrol	5267728.867	812365.5583	15.42155223
UIJ67653.1	biofilm_matrix_protein_CalY_[Bacillus_cereus]	CPTF_Al	35641497.17	6667987.424	18.70849418
UIJ67653.1	biofilm_matrix_protein_CalY_[Bacillus_cereus]	CPTF_Cd	36723092.27	3632298.305	9.89104697
UIJ67653.1	biofilm_matrix_protein_CalY_[Bacillus_cereus]	CPTF_Co	35041585.87	1373154.041	3.918641256
UIJ67653.1	biofilm_matrix_protein_CalY_[Bacillus_cereus]	CPTF_Cu	26581220.73	1358941.894	5.112413414
UIJ67653.1	biofilm_matrix_protein_CalY_[Bacillus_cereus]	CPTF_Fe	34895957.23	2346979.28	6.725648086

UIJ67653.1	biofilm_matrix_protein_CalY_[Bacillus_cereus]	CPTF_Mn	34710267.7	6114763.577	17.61658432
UIJ67653.1	biofilm_matrix_protein_CalY_[Bacillus_cereus]	CPTF_Ni	36316393.3	4417567.317	12.16411355
UIJ67653.1	biofilm_matrix_protein_CalY_[Bacillus_cereus]	CPTF_U	36494091.97	4300407.19	11.78384489
UIJ67653.1	biofilm_matrix_protein_CalY_[Bacillus_cereus]	CPTF_metals_mix	16470245.3	922379.263	5.600276415
UIJ67653.1	biofilm_matrix_protein_CalY_[Bacillus_cereus]	CPTF_zcontrol	37127526.63	2499244.516	6.731513631
UIJ67654.1	helix-turn-helix_domain-containing_protein_[Bacillus_cereus]	CPTF_Al	23808.63333	41237.76259	173.2050808
UIJ67654.1	helix-turn-helix_domain-containing_protein_[Bacillus_cereus]	CPTF_Cd	0	0	0
UIJ67654.1	helix-turn-helix_domain-containing_protein_[Bacillus_cereus]	CPTF_Co	0	0	0
UIJ67654.1	helix-turn-helix_domain-containing_protein_[Bacillus_cereus]	CPTF_Cu	21363.26667	37002.26328	173.2050808
UIJ67654.1	helix-turn-helix_domain-containing_protein_[Bacillus_cereus]	CPTF_Fe	0	0	0
UIJ67654.1	helix-turn-helix_domain-containing_protein_[Bacillus_cereus]	CPTF_Mn	0	0	0
UIJ67654.1	helix-turn-helix_domain-containing_protein_[Bacillus_cereus]	CPTF_Ni	0	0	0
UIJ67654.1	helix-turn-helix_domain-containing_protein_[Bacillus_cereus]	CPTF_U	0	0	0
UIJ67654.1	helix-turn-helix_domain-containing_protein_[Bacillus_cereus]	CPTF_metals_mix	173931.6667	55693.31091	32.02022494
UIJ67654.1	helix-turn-helix_domain-containing_protein_[Bacillus_cereus]	CPTF_zcontrol	0	0	0
UIJ67656.1	immune_inhibitor_A_[Bacillus_cereus]	CPTF_Al	5069264.9	962217.5331	18.98140168
UIJ67656.1	immune_inhibitor_A_[Bacillus_cereus]	CPTF_Cd	5729849.9	948896.0186	16.56057375
UIJ67656.1	immune_inhibitor_A_[Bacillus_cereus]	CPTF_Co	5390649.5	672241.3524	12.47050754
UIJ67656.1	immune_inhibitor_A_[Bacillus_cereus]	CPTF_Cu	2582947	179578.5181	6.952466236
UIJ67656.1	immune_inhibitor_A_[Bacillus_cereus]	CPTF_Fe	5577171.233	1211527.968	21.72298316
UIJ67656.1	immune_inhibitor_A_[Bacillus_cereus]	CPTF_Mn	5234831.333	762478.9746	14.56549268
UIJ67656.1	immune_inhibitor_A_[Bacillus_cereus]	CPTF_Ni	3696446.033	88208.38656	2.386302567
UIJ67656.1	immune_inhibitor_A_[Bacillus_cereus]	CPTF_U	4900171.667	1172490.152	23.92753217
UIJ67656.1	immune_inhibitor_A_[Bacillus_cereus]	CPTF_metals_mix	1560263.667	364307.2714	23.34908383
UIJ67656.1	immune_inhibitor_A_[Bacillus_cereus]	CPTF_zcontrol	5125825.533	834454.4114	16.27941501
UIJ67658.1	spermidine/putrescine_ABC_transporter_ATP-binding_protein_PotA_[Bacillus_cereus]	CPTF_Al	12346.5	21384.7653	173.2050808
UIJ67658.1	spermidine/putrescine_ABC_transporter_ATP-binding_protein_PotA_[Bacillus_cereus]	CPTF_Cd	61931.8	54704.40522	88.33007473
UIJ67658.1	spermidine/putrescine_ABC_transporter_ATP-binding_protein_PotA_[Bacillus_cereus]	CPTF_Co	56507.83333	49804.95244	88.13813856
UIJ67658.1	spermidine/putrescine_ABC_transporter_ATP-binding_protein_PotA_[Bacillus_cereus]	CPTF_Cu	24845.03333	43032.86005	173.2050808
UIJ67658.1	spermidine/putrescine_ABC_transporter_ATP-binding_protein_PotA_[Bacillus_cereus]	CPTF_Fe	38120.4	66026.4696	173.2050808
UIJ67658.1	spermidine/putrescine_ABC_transporter_ATP-binding_protein_PotA_[Bacillus_cereus]	CPTF_Mn	0	0	0
UIJ67658.1	spermidine/putrescine_ABC_transporter_ATP-binding_protein_PotA_[Bacillus_cereus]	CPTF_Ni	28680.46667	49676.02545	173.2050808
UIJ67658.1	spermidine/putrescine_ABC_transporter_ATP-binding_protein_PotA_[Bacillus_cereus]	CPTF_U	42350.66667	73353.5064	173.2050808
UIJ67658.1	spermidine/putrescine_ABC_transporter_ATP-binding_protein_PotA_[Bacillus_cereus]	CPTF_metals_mix	66127.03333	60284.01904	91.1639552
UIJ67658.1	spermidine/putrescine_ABC_transporter_ATP-binding_protein_PotA_[Bacillus_cereus]	CPTF_zcontrol	30697.03333	53168.82137	173.2050808
UIJ67661.1	spermidine/putrescine_ABC_transporter_substrate-binding_protein_PotD_[Bacillus_cereus	CPTF_Al	1032053.3	499922.9	48.4396397
UIJ67661.1	spermidine/putrescine_ABC_transporter_substrate-binding_protein_PotD_[Bacillus_cereus	CPTF_Cd	979452.8333	487521.3846	49.77487103
UIJ67661.1	spermidine/putrescine_ABC_transporter_substrate-binding_protein_PotD_[Bacillus_cereus	CPTF_Co	1142834.6	535388.5582	46.84742291
UIJ67661.1	spermidine/putrescine_ABC_transporter_substrate-binding_protein_PotD_[Bacillus_cereus	CPTF_Cu	774291.2667	314753.0745	40.65047458
UIJ67661.1	spermidine/putrescine_ABC_transporter_substrate-binding_protein_PotD_[Bacillus_cereus	CPTF_Fe	593589	232700.631	39.20231523
UIJ67661.1	spermidine/putrescine_ABC_transporter_substrate-binding_protein_PotD_[Bacillus_cereus	CPTF_Mn	517917.8667	119952.2648	23.16048016
UIJ67661.1	spermidine/putrescine_ABC_transporter_substrate-binding_protein_PotD_[Bacillus_cereus	CPTF_Ni	1192630.833	370953.6576	31.10381245
UIJ67661.1	spermidine/putrescine_ABC_transporter_substrate-binding_protein_PotD_[Bacillus_cereus	CPTF_U	905570.3333	466968.836	51.56626921
UIJ67661.1	spermidine/putrescine_ABC_transporter_substrate-binding_protein_PotD_[Bacillus_cereus	CPTF_metals_mix	1743743.833	382854.9006	21.95591424
UIJ67661.1	spermidine/putrescine_ABC_transporter_substrate-binding_protein_PotD_[Bacillus_cereus	CPTF_zcontrol	1152055	220060.173	19.10153361
UIJ67672.1	response_regulator_transcription_factor_[Bacillus_cereus]	CPTF_Al	0	0	0
UIJ67672.1	response_regulator_transcription_factor_[Bacillus_cereus]	CPTF_Cd	0	0	0
UIJ67672.1	response_regulator_transcription_factor_[Bacillus_cereus]	CPTF_Co	0	0	0
UIJ67672.1	response_regulator_transcription_factor_[Bacillus_cereus]	CPTF_Cu	0	0	0
UIJ67672.1	response_regulator_transcription_factor_[Bacillus_cereus]	CPTF_Fe	0	0	0
UIJ67672.1	response_regulator_transcription_factor_[Bacillus_cereus]	CPTF_Mn	0	0	0
UIJ67672.1	response_regulator_transcription_factor_[Bacillus_cereus]	CPTF_Ni	0	0	0
UIJ67672.1	response_regulator_transcription_factor_[Bacillus_cereus]	CPTF_U	0	0	0
UIJ67672.1	response_regulator_transcription_factor_[Bacillus_cereus]	CPTF_metals_mix	132890.4333	55708.26303	41.92044652
UIJ67672.1	response_regulator_transcription_factor_[Bacillus_cereus]	CPTF_zcontrol	0	0	0
UIJ67675.1	(Fe-S)-binding_protein_[Bacillus_cereus]	CPTF_Al	15421.06667	26710.07097	173.2050808
UIJ67675.1	(Fe-S)-binding_protein_[Bacillus_cereus]	CPTF_Cd	0	0	0
UIJ67675.1	(Fe-S)-binding_protein_[Bacillus_cereus]	CPTF_Co	189961.6667	38508.60021	20.27177424
UIJ67675.1	(Fe-S)-binding_protein_[Bacillus_cereus]	CPTF_Cu	65066.86667	57870.12171	88.93946286
UIJ67675.1	(Fe-S)-binding_protein_[Bacillus_cereus]	CPTF_Fe	8587.9	14874.67913	173.2050808
UIJ67675.1	(Fe-S)-binding_protein_[Bacillus_cereus]	CPTF_Mn	15302.06667	26503.95693	173.2050808

UIJ67675.1	(Fe-S)-binding_protein_[Bacillus_cereus]	CPTF_Ni	0	0	0
UIJ67675.1	(Fe-S)-binding_protein_[Bacillus_cereus]	CPTF_U	21474.96667	37195.73336	173.2050808
UIJ67675.1	(Fe-S)-binding_protein_[Bacillus_cereus]	CPTF_metals_mix	287216.6667	60469.63528	21.05366516
UIJ67675.1	(Fe-S)-binding_protein_[Bacillus_cereus]	CPTF_zcontrol	0	0	0
UIJ67676.1	LutB/LldF_family_L-lactate_oxidation_iron-sulfur_protein_[Bacillus_cereus]	CPTF_Al	552200.4	930258.2981	168.4638943
UIJ67676.1	LutB/LldF_family_L-lactate_oxidation_iron-sulfur_protein_[Bacillus_cereus]	CPTF_Cd	1060388	321277.002	30.2980609
UIJ67676.1	LutB/LldF_family_L-lactate_oxidation_iron-sulfur_protein_[Bacillus_cereus]	CPTF_Co	1230677.567	124306.0541	10.10061916
UIJ67676.1	LutB/LldF_family_L-lactate_oxidation_iron-sulfur_protein_[Bacillus_cereus]	CPTF_Cu	1263515.567	507495.7116	40.16537073
UIJ67676.1	LutB/LldF_family_L-lactate_oxidation_iron-sulfur_protein_[Bacillus_cereus]	CPTF_Fe	380934	336182.016	88.25203737
UIJ67676.1	LutB/LldF_family_L-lactate_oxidation_iron-sulfur_protein_[Bacillus_cereus]	CPTF_Mn	698666	228156.2308	32.65598023
UIJ67676.1	LutB/LldF_family_L-lactate_oxidation_iron-sulfur_protein_[Bacillus_cereus]	CPTF_Ni	775941	288944.2527	37.23791534
UIJ67676.1	LutB/LldF_family_L-lactate_oxidation_iron-sulfur_protein_[Bacillus_cereus]	CPTF_U	712243.6667	54969.61808	7.717810724
UIJ67676.1	LutB/LldF_family_L-lactate_oxidation_iron-sulfur_protein_[Bacillus_cereus]	CPTF_metals_mix	1605452.633	138717.9286	8.640424872
UIJ67676.1	LutB/LldF_family_L-lactate_oxidation_iron-sulfur_protein_[Bacillus_cereus]	CPTF_zcontrol	630641.6667	413451.8239	65.56049906
UIJ67677.1	lactate_utilization_protein_C_[Bacillus_cereus]	CPTF_Al	1259286	73516.43158	5.837945596
UIJ67677.1	lactate_utilization_protein_C_[Bacillus_cereus]	CPTF_Cd	1281018.767	233731.3665	18.24574101
UIJ67677.1	lactate_utilization_protein_C_[Bacillus_cereus]	CPTF_Co	1626854	95362.67461	5.861784438
UIJ67677.1	lactate_utilization_protein_C_[Bacillus_cereus]	CPTF_Cu	1361152	91189.69985	6.699450161
UIJ67677.1	lactate_utilization_protein_C_[Bacillus_cereus]	CPTF_Fe	1322655.9	139285.3676	10.53073348
UIJ67677.1	lactate_utilization_protein_C_[Bacillus_cereus]	CPTF_Mn	943218.0333	278544.4967	29.53129466
UIJ67677.1	lactate_utilization_protein_C_[Bacillus_cereus]	CPTF_Ni	1318977	87806.18413	6.657142932
UIJ67677.1	lactate_utilization_protein_C_[Bacillus_cereus]	CPTF_U	1142672	154377.0866	13.51018373
UIJ67677.1	lactate_utilization_protein_C_[Bacillus_cereus]	CPTF_metals_mix	1249006.333	468765.3446	37.53106226
UIJ67677.1	lactate_utilization_protein_C_[Bacillus_cereus]	CPTF_zcontrol	1219052.433	190988.1265	15.66693288
UIJ67684.1	MaoC_family_dehydratase_[Bacillus_cereus]	CPTF_Al	85834.93333	17663.76293	20.57875768
UIJ67684.1	MaoC_family_dehydratase_[Bacillus_cereus]	CPTF_Cd	80883.2	25077.56544	31.0046653
UIJ67684.1	MaoC_family_dehydratase_[Bacillus_cereus]	CPTF_Co	69104.36667	35298.72579	51.08031156
UIJ67684.1	MaoC_family_dehydratase_[Bacillus_cereus]	CPTF_Cu	86510.03333	7877.583522	9.10597675
UIJ67684.1	MaoC_family_dehydratase_[Bacillus_cereus]	CPTF_Fe	27282.76667	47255.13804	173.2050808
UIJ67684.1	MaoC_family_dehydratase_[Bacillus_cereus]	CPTF_Mn	51721.26667	28228.63767	54.57839587
UIJ67684.1	MaoC_family_dehydratase_[Bacillus_cereus]	CPTF_Ni	82822.53333	18296.91316	22.09170913
UIJ67684.1	MaoC_family_dehydratase_[Bacillus_cereus]	CPTF_U	84618.83333	21803.07855	25.76622448
UIJ67684.1	MaoC_family_dehydratase_[Bacillus_cereus]	CPTF_metals_mix	37858.23333	18844.33356	49.77605107
UIJ67684.1	MaoC_family_dehydratase_[Bacillus_cereus]	CPTF_zcontrol	34425.43333	29942.98097	86.979242
UIJ67685.1	polyhydroxyalkanoic_acid_inclusion_protein_PhaP_[Bacillus_cereus]	CPTF_Al	7384125.1	246573.9488	3.339243925
UIJ67685.1	polyhydroxyalkanoic_acid_inclusion_protein_PhaP_[Bacillus_cereus]	CPTF_Cd	7297660.267	139286.3738	1.908644259
UIJ67685.1	polyhydroxyalkanoic_acid_inclusion_protein_PhaP_[Bacillus_cereus]	CPTF_Co	7135724.9	448377.4604	6.283558667
UIJ67685.1	polyhydroxyalkanoic_acid_inclusion_protein_PhaP_[Bacillus_cereus]	CPTF_Cu	6359177.467	648077.1461	10.19121025
UIJ67685.1	polyhydroxyalkanoic_acid_inclusion_protein_PhaP_[Bacillus_cereus]	CPTF_Fe	7160958.733	259467.2927	3.623359698
UIJ67685.1	polyhydroxyalkanoic_acid_inclusion_protein_PhaP_[Bacillus_cereus]	CPTF_Mn	6752814.733	318165.0979	4.711592283
UIJ67685.1	polyhydroxyalkanoic_acid_inclusion_protein_PhaP_[Bacillus_cereus]	CPTF_Ni	5929544.667	640738.4642	10.80586285
UIJ67685.1	polyhydroxyalkanoic_acid_inclusion_protein_PhaP_[Bacillus_cereus]	CPTF_U	6737100.233	365381.6737	5.423426415
UIJ67685.1	polyhydroxyalkanoic_acid_inclusion_protein_PhaP_[Bacillus_cereus]	CPTF_metals_mix	4378781.967	498402.2439	11.38221194
UIJ67685.1	polyhydroxyalkanoic_acid_inclusion_protein_PhaP_[Bacillus_cereus]	CPTF_zcontrol	7023732	587095.8009	8.358744338
UIJ67687.1	polyhydroxyalkanoic_acid_synthase_subunit_PhaR_[Bacillus_cereus]	CPTF_Al	18290767	1675295.677	9.159242349
UIJ67687.1	polyhydroxyalkanoic_acid_synthase_subunit_PhaR_[Bacillus_cereus]	CPTF_Cd	18863186	933140.4767	4.946886898
UIJ67687.1	polyhydroxyalkanoic_acid_synthase_subunit_PhaR_[Bacillus_cereus]	CPTF_Co	15700996.77	1771128.294	11.28035576
UIJ67687.1	polyhydroxyalkanoic_acid_synthase_subunit_PhaR_[Bacillus_cereus]	CPTF_Cu	13147896.6	523118.9489	3.978727281
UIJ67687.1	polyhydroxyalkanoic_acid_synthase_subunit_PhaR_[Bacillus_cereus]	CPTF_Fe	17137372	3189105.166	18.60906775
UIJ67687.1	polyhydroxyalkanoic_acid_synthase_subunit_PhaR_[Bacillus_cereus]	CPTF_Mn	18132622.43	5553429.931	30.6267334
UIJ67687.1	polyhydroxyalkanoic_acid_synthase_subunit_PhaR_[Bacillus_cereus]	CPTF_Ni	9601068	1968572.123	20.50367858
UIJ67687.1	polyhydroxyalkanoic_acid_synthase_subunit_PhaR_[Bacillus_cereus]	CPTF_U	14421118.33	223829.5924	1.552095942
UIJ67687.1	polyhydroxyalkanoic_acid_synthase_subunit_PhaR_[Bacillus_cereus]	CPTF_metals_mix	11699327.13	1718732.193	14.69086362
UIJ67687.1	polyhydroxyalkanoic_acid_synthase_subunit_PhaR_[Bacillus_cereus]	CPTF_zcontrol	16154267.27	2894979.769	17.92083616
UIJ67688.1	acetoacetyl-CoA_reductase_[Bacillus_cereus]	CPTF_Al	28871161.37	879886.9846	3.047632804
UIJ67688.1	acetoacetyl-CoA_reductase_[Bacillus_cereus]	CPTF_Cd	29769704.17	201508.384	0.676890784
UIJ67688.1	acetoacetyl-CoA_reductase_[Bacillus_cereus]	CPTF_Co	27476558.9	1827765.479	6.652090188
UIJ67688.1	acetoacetyl-CoA_reductase_[Bacillus_cereus]	CPTF_Cu	22580005	1528219.026	6.76801899
UIJ67688.1	acetoacetyl-CoA_reductase_[Bacillus_cereus]	CPTF_Fe	28619795	2551743.457	8.916008856
UIJ67688.1	acetoacetyl-CoA_reductase_[Bacillus_cereus]	CPTF_Mn	30567975.53	6227981.314	20.37420276
UIJ67688.1	acetoacetyl-CoA_reductase_[Bacillus_cereus]	CPTF_Ni	23863028.1	2088776.036	8.753189358

UJJ67688.1	acetoacetyl-CoA_reductase_[Bacillus_cereus]	CPTF_U	21544907.57	752196.7212	3.491297045
UJJ67688.1	acetoacetyl-CoA_reductase_[Bacillus_cereus]	CPTF_metals_mix	20345201	2024946.901	9.952946157
UJJ67688.1	acetoacetyl-CoA_reductase_[Bacillus_cereus]	CPTF_zcontrol	29381714.57	1590350.53	5.412722006
UJJ67689.1	class_III_poly(R)-hydroxyalkanoic_acid_synthase_subunit_PhaC_[Bacillus_cereus]	CPTF_Al	16291408.9	240450.9799	1.475937296
UJJ67689.1	class_III_poly(R)-hydroxyalkanoic_acid_synthase_subunit_PhaC_[Bacillus_cereus]	CPTF_Cd	14361344.8	1752952.902	12.20604983
UJJ67689.1	class_III_poly(R)-hydroxyalkanoic_acid_synthase_subunit_PhaC_[Bacillus_cereus]	CPTF_Co	14347322.53	1189476.503	8.290581743
UJJ67689.1	class_III_poly(R)-hydroxyalkanoic_acid_synthase_subunit_PhaC_[Bacillus_cereus]	CPTF_Cu	12145667.7	1418396.386	11.67820841
UJJ67689.1	class_III_poly(R)-hydroxyalkanoic_acid_synthase_subunit_PhaC_[Bacillus_cereus]	CPTF_Fe	15743153.1	749315.8394	4.75963001
UJJ67689.1	class_III_poly(R)-hydroxyalkanoic_acid_synthase_subunit_PhaC_[Bacillus_cereus]	CPTF_Mn	16502581.2	571807.6159	3.464958657
UJJ67689.1	class_III_poly(R)-hydroxyalkanoic_acid_synthase_subunit_PhaC_[Bacillus_cereus]	CPTF_Ni	12397156.8	1606865.892	12.96156786
UJJ67689.1	class_III_poly(R)-hydroxyalkanoic_acid_synthase_subunit_PhaC_[Bacillus_cereus]	CPTF_U	13723420	1446080.145	10.5373161
UJJ67689.1	class_III_poly(R)-hydroxyalkanoic_acid_synthase_subunit_PhaC_[Bacillus_cereus]	CPTF_metals_mix	8390805.2	475377.271	5.665454741
UJJ67689.1	class_III_poly(R)-hydroxyalkanoic_acid_synthase_subunit_PhaC_[Bacillus_cereus]	CPTF_zcontrol	16390530.13	973321.4095	5.93831561
UJJ67708.1	MarR_family_transcriptional_regulator_[Bacillus_cereus]	CPTF_Al	123634.6667	42966.79948	34.7530354
UJJ67708.1	MarR_family_transcriptional_regulator_[Bacillus_cereus]	CPTF_Cd	128224.3333	112720.9659	87.9091846
UJJ67708.1	MarR_family_transcriptional_regulator_[Bacillus_cereus]	CPTF_Co	120521.5	31835.96479	26.41517471
UJJ67708.1	MarR_family_transcriptional_regulator_[Bacillus_cereus]	CPTF_Cu	66645.49667	61457.42708	92.21542362
UJJ67708.1	MarR_family_transcriptional_regulator_[Bacillus_cereus]	CPTF_Fe	96955.3	93259.81949	96.18846984
UJJ67708.1	MarR_family_transcriptional_regulator_[Bacillus_cereus]	CPTF_Mn	33191.73333	44150.75315	133.017317
UJJ67708.1	MarR_family_transcriptional_regulator_[Bacillus_cereus]	CPTF_Ni	86057.66667	149056.251	173.2050808
UJJ67708.1	MarR_family_transcriptional_regulator_[Bacillus_cereus]	CPTF_U	55903.66667	96827.991	173.2050808
UJJ67708.1	MarR_family_transcriptional_regulator_[Bacillus_cereus]	CPTF_metals_mix	16578.76667	28715.26619	173.2050808
UJJ67708.1	MarR_family_transcriptional_regulator_[Bacillus_cereus]	CPTF_zcontrol	11780.36667	20404.1936	173.2050808
UJJ67709.1	M3_family_oligoendopeptidase_[Bacillus_cereus]	CPTF_Al	5212984.333	558273.4727	10.70928737
UJJ67709.1	M3_family_oligoendopeptidase_[Bacillus_cereus]	CPTF_Cd	5229323.633	453964.057	8.681123771
UJJ67709.1	M3_family_oligoendopeptidase_[Bacillus_cereus]	CPTF_Co	5072547.5	467045.3951	9.207314373
UJJ67709.1	M3_family_oligoendopeptidase_[Bacillus_cereus]	CPTF_Cu	5201599.467	419644.9269	8.067613233
UJJ67709.1	M3_family_oligoendopeptidase_[Bacillus_cereus]	CPTF_Fe	4856084.5	515846.7545	10.62268901
UJJ67709.1	M3_family_oligoendopeptidase_[Bacillus_cereus]	CPTF_Mn	5060971.9	613628.0499	12.12470771
UJJ67709.1	M3_family_oligoendopeptidase_[Bacillus_cereus]	CPTF_Ni	4544523.933	193170.7217	4.250626128
UJJ67709.1	M3_family_oligoendopeptidase_[Bacillus_cereus]	CPTF_U	3797368.667	526833.3852	13.87364334
UJJ67709.1	M3_family_oligoendopeptidase_[Bacillus_cereus]	CPTF_metals_mix	6104184.4	250539.5759	4.104390684
UJJ67709.1	M3_family_oligoendopeptidase_[Bacillus_cereus]	CPTF_zcontrol	4677429.633	379122.0867	8.105350939
UJJ67710.1	DinB_family_protein_[Bacillus_cereus]	CPTF_Al	6125970.367	762375.3493	12.44497286
UJJ67710.1	DinB_family_protein_[Bacillus_cereus]	CPTF_Cd	5656736.333	231147.9714	4.086242628
UJJ67710.1	DinB_family_protein_[Bacillus_cereus]	CPTF_Co	5465380.633	328417.6485	6.009053542
UJJ67710.1	DinB_family_protein_[Bacillus_cereus]	CPTF_Cu	5211072.533	119604.8386	2.295205792
UJJ67710.1	DinB_family_protein_[Bacillus_cereus]	CPTF_Fe	5985460.667	656502.8029	10.96829199
UJJ67710.1	DinB_family_protein_[Bacillus_cereus]	CPTF_Mn	5978815.733	361504.7713	6.046427711
UJJ67710.1	DinB_family_protein_[Bacillus_cereus]	CPTF_Ni	6069785.7	407835.5295	6.719109201
UJJ67710.1	DinB_family_protein_[Bacillus_cereus]	CPTF_U	6033681.7	843196.325	13.97482279
UJJ67710.1	DinB_family_protein_[Bacillus_cereus]	CPTF_metals_mix	3784921.367	282831.4053	7.472583387
UJJ67710.1	DinB_family_protein_[Bacillus_cereus]	CPTF_zcontrol	5538612.9	282815.8765	5.106258221
UJJ67716.1	preQ(1)_synthase_[Bacillus_cereus]	CPTF_Al	70663.76667	61228.62855	86.64784151
UJJ67716.1	preQ(1)_synthase_[Bacillus_cereus]	CPTF_Cd	50358.36667	72917.06038	144.7963173
UJJ67716.1	preQ(1)_synthase_[Bacillus_cereus]	CPTF_Co	127177.7667	32191.18477	25.31195948
UJJ67716.1	preQ(1)_synthase_[Bacillus_cereus]	CPTF_Cu	37217.53333	64462.65867	173.2050808
UJJ67716.1	preQ(1)_synthase_[Bacillus_cereus]	CPTF_Fe	6933.366667	12008.94333	173.2050808
UJJ67716.1	preQ(1)_synthase_[Bacillus_cereus]	CPTF_Mn	31433.93333	30229.46767	96.16826298
UJJ67716.1	preQ(1)_synthase_[Bacillus_cereus]	CPTF_Ni	8372.066667	14500.84483	173.2050808
UJJ67716.1	preQ(1)_synthase_[Bacillus_cereus]	CPTF_U	18991.6	32894.41612	173.2050808
UJJ67716.1	preQ(1)_synthase_[Bacillus_cereus]	CPTF_metals_mix	490238.5667	57330.68988	11.69444711
UJJ67716.1	preQ(1)_synthase_[Bacillus_cereus]	CPTF_zcontrol	72271.4	62757.1018	86.83532047
UJJ67720.1	abortive_phage_infection_protein_[Bacillus_cereus]	CPTF_Al	0	0	0
UJJ67720.1	abortive_phage_infection_protein_[Bacillus_cereus]	CPTF_Cd	26534.53333	23294.13758	87.78800548
UJJ67720.1	abortive_phage_infection_protein_[Bacillus_cereus]	CPTF_Co	24483.7	6018.307948	24.58087605
UJJ67720.1	abortive_phage_infection_protein_[Bacillus_cereus]	CPTF_Cu	59539.3	8183.401911	13.74453833
UJJ67720.1	abortive_phage_infection_protein_[Bacillus_cereus]	CPTF_Fe	0	0	0
UJJ67720.1	abortive_phage_infection_protein_[Bacillus_cereus]	CPTF_Mn	0	0	0
UJJ67720.1	abortive_phage_infection_protein_[Bacillus_cereus]	CPTF_Ni	46117.96667	3274.163909	7.099540907
UJJ67720.1	abortive_phage_infection_protein_[Bacillus_cereus]	CPTF_U	11045.86667	19132.00228	173.2050808

UIJ67720.1	abortive_phage_infection_protein_[Bacillus_cereus]	CPTF_metals_mix	15981.5	27680.76998	173.2050808
UIJ67720.1	abortive_phage_infection_protein_[Bacillus_cereus]	CPTF_zcontrol	0	0	0
UIJ67723.1	class_1b_ribonucleoside-diphosphate_reductase_subunit_alpha_[Bacillus_cereus]	CPTF_Al	4882847.533	201794.3428	4.132718488
UIJ67723.1	class_1b_ribonucleoside-diphosphate_reductase_subunit_alpha_[Bacillus_cereus]	CPTF_Cd	4118208.267	794415.5919	19.29032095
UIJ67723.1	class_1b_ribonucleoside-diphosphate_reductase_subunit_alpha_[Bacillus_cereus]	CPTF_Co	4408719.6	166854.6429	3.784650829
UIJ67723.1	class_1b_ribonucleoside-diphosphate_reductase_subunit_alpha_[Bacillus_cereus]	CPTF_Cu	4482157.867	393687.0405	8.783426471
UIJ67723.1	class_1b_ribonucleoside-diphosphate_reductase_subunit_alpha_[Bacillus_cereus]	CPTF_Fe	4645628.333	423026.7914	9.105911214
UIJ67723.1	class_1b_ribonucleoside-diphosphate_reductase_subunit_alpha_[Bacillus_cereus]	CPTF_Mn	4680979.567	691206.3812	14.7662764
UIJ67723.1	class_1b_ribonucleoside-diphosphate_reductase_subunit_alpha_[Bacillus_cereus]	CPTF_Ni	4080371.667	957998.8955	23.47822634
UIJ67723.1	class_1b_ribonucleoside-diphosphate_reductase_subunit_alpha_[Bacillus_cereus]	CPTF_U	4303245.633	504443.7433	11.72240179
UIJ67723.1	class_1b_ribonucleoside-diphosphate_reductase_subunit_alpha_[Bacillus_cereus]	CPTF_metals_mix	4644574.5	124695.6058	2.684758438
UIJ67723.1	class_1b_ribonucleoside-diphosphate_reductase_subunit_alpha_[Bacillus_cereus]	CPTF_zcontrol	4781112.1	483089.1183	10.10411612
UIJ67724.1	class_1b_ribonucleoside-diphosphate_reductase_subunit_beta_[Bacillus_cereus]	CPTF_Al	770905.1	88127.86364	11.43173961
UIJ67724.1	class_1b_ribonucleoside-diphosphate_reductase_subunit_beta_[Bacillus_cereus]	CPTF_Cd	939286.4667	258478.0716	27.51855592
UIJ67724.1	class_1b_ribonucleoside-diphosphate_reductase_subunit_beta_[Bacillus_cereus]	CPTF_Co	703410.9	144436.2371	20.53369333
UIJ67724.1	class_1b_ribonucleoside-diphosphate_reductase_subunit_beta_[Bacillus_cereus]	CPTF_Cu	809593.5333	55143.07401	6.811204851
UIJ67724.1	class_1b_ribonucleoside-diphosphate_reductase_subunit_beta_[Bacillus_cereus]	CPTF_Fe	800743.0667	132058.7967	16.49203124
UIJ67724.1	class_1b_ribonucleoside-diphosphate_reductase_subunit_beta_[Bacillus_cereus]	CPTF_Mn	885195.5333	358296.2302	40.47650679
UIJ67724.1	class_1b_ribonucleoside-diphosphate_reductase_subunit_beta_[Bacillus_cereus]	CPTF_Ni	715442.1	33752.33258	4.717688906
UIJ67724.1	class_1b_ribonucleoside-diphosphate_reductase_subunit_beta_[Bacillus_cereus]	CPTF_U	798555.9667	183981.9133	23.03932611
UIJ67724.1	class_1b_ribonucleoside-diphosphate_reductase_subunit_beta_[Bacillus_cereus]	CPTF_metals_mix	1406936.867	96584.41867	6.86487226
UIJ67724.1	class_1b_ribonucleoside-diphosphate_reductase_subunit_beta_[Bacillus_cereus]	CPTF_zcontrol	711989.6667	11354.8492	1.594805336
UIJ67731.1	DUF3913_family_protein_[Bacillus_cereus]	CPTF_Al	0	0	0
UIJ67731.1	DUF3913_family_protein_[Bacillus_cereus]	CPTF_Cd	272964.3333	75250.15714	27.56776177
UIJ67731.1	DUF3913_family_protein_[Bacillus_cereus]	CPTF_Co	25998.53333	45030.78066	173.2050808
UIJ67731.1	DUF3913_family_protein_[Bacillus_cereus]	CPTF_Cu	0	0	0
UIJ67731.1	DUF3913_family_protein_[Bacillus_cereus]	CPTF_Fe	62005	107395.8103	173.2050808
UIJ67731.1	DUF3913_family_protein_[Bacillus_cereus]	CPTF_Mn	114924.3333	199054.7844	173.2050808
UIJ67731.1	DUF3913_family_protein_[Bacillus_cereus]	CPTF_Ni	0	0	0
UIJ67731.1	DUF3913_family_protein_[Bacillus_cereus]	CPTF_U	0	0	0
UIJ67731.1	DUF3913_family_protein_[Bacillus_cereus]	CPTF_metals_mix	1058979.333	212312.8948	20.04882325
UIJ67731.1	DUF3913_family_protein_[Bacillus_cereus]	CPTF_zcontrol	0	0	0
UIJ67737.1	D-alanyl-lipoteichoic_acid_biosynthesis_protein_DltD_[Bacillus_cereus]	CPTF_Al	0	0	0
UIJ67737.1	D-alanyl-lipoteichoic_acid_biosynthesis_protein_DltD_[Bacillus_cereus]	CPTF_Cd	0	0	0
UIJ67737.1	D-alanyl-lipoteichoic_acid_biosynthesis_protein_DltD_[Bacillus_cereus]	CPTF_Co	4592.466667	7954.385599	173.2050808
UIJ67737.1	D-alanyl-lipoteichoic_acid_biosynthesis_protein_DltD_[Bacillus_cereus]	CPTF_Cu	4518.266667	7825.867429	173.2050808
UIJ67737.1	D-alanyl-lipoteichoic_acid_biosynthesis_protein_DltD_[Bacillus_cereus]	CPTF_Fe	0	0	0
UIJ67737.1	D-alanyl-lipoteichoic_acid_biosynthesis_protein_DltD_[Bacillus_cereus]	CPTF_Mn	0	0	0
UIJ67737.1	D-alanyl-lipoteichoic_acid_biosynthesis_protein_DltD_[Bacillus_cereus]	CPTF_Ni	0	0	0
UIJ67737.1	D-alanyl-lipoteichoic_acid_biosynthesis_protein_DltD_[Bacillus_cereus]	CPTF_U	0	0	0
UIJ67737.1	D-alanyl-lipoteichoic_acid_biosynthesis_protein_DltD_[Bacillus_cereus]	CPTF_metals_mix	10091.23333	17478.52884	173.2050808
UIJ67737.1	D-alanyl-lipoteichoic_acid_biosynthesis_protein_DltD_[Bacillus_cereus]	CPTF_zcontrol	0	0	0
UIJ67738.1	D-alanine--poly(phosphoribitol)_ligase_subunit_DltC_[Bacillus_cereus]	CPTF_Al	48234.33333	16137.84848	33.45718156
UIJ67738.1	D-alanine--poly(phosphoribitol)_ligase_subunit_DltC_[Bacillus_cereus]	CPTF_Cd	102935.0333	23607.0503	22.93393176
UIJ67738.1	D-alanine--poly(phosphoribitol)_ligase_subunit_DltC_[Bacillus_cereus]	CPTF_Co	79292.03333	14446.34422	18.21916228
UIJ67738.1	D-alanine--poly(phosphoribitol)_ligase_subunit_DltC_[Bacillus_cereus]	CPTF_Cu	71313.23333	12935.94218	18.13960969
UIJ67738.1	D-alanine--poly(phosphoribitol)_ligase_subunit_DltC_[Bacillus_cereus]	CPTF_Fe	91559.86667	10442.16798	11.40474354
UIJ67738.1	D-alanine--poly(phosphoribitol)_ligase_subunit_DltC_[Bacillus_cereus]	CPTF_Mn	19839.4	34362.84879	173.2050808
UIJ67738.1	D-alanine--poly(phosphoribitol)_ligase_subunit_DltC_[Bacillus_cereus]	CPTF_Ni	77460.96667	25858.23495	33.38227764
UIJ67738.1	D-alanine--poly(phosphoribitol)_ligase_subunit_DltC_[Bacillus_cereus]	CPTF_U	0	0	0
UIJ67738.1	D-alanine--poly(phosphoribitol)_ligase_subunit_DltC_[Bacillus_cereus]	CPTF_metals_mix	128724.4333	84552.68257	65.68502993
UIJ67738.1	D-alanine--poly(phosphoribitol)_ligase_subunit_DltC_[Bacillus_cereus]	CPTF_zcontrol	47546.96667	44865.56209	94.36051391
UIJ67740.1	D-alanine--poly(phosphoribitol)_ligase_subunit_DltA_[Bacillus_cereus]	CPTF_Al	1322994	33794.58763	2.554402184
UIJ67740.1	D-alanine--poly(phosphoribitol)_ligase_subunit_DltA_[Bacillus_cereus]	CPTF_Cd	1359897.467	104476.5962	7.682681875
UIJ67740.1	D-alanine--poly(phosphoribitol)_ligase_subunit_DltA_[Bacillus_cereus]	CPTF_Co	1435052.067	62742.76843	4.372157893
UIJ67740.1	D-alanine--poly(phosphoribitol)_ligase_subunit_DltA_[Bacillus_cereus]	CPTF_Cu	1367889.667	65615.44639	4.796837639
UIJ67740.1	D-alanine--poly(phosphoribitol)_ligase_subunit_DltA_[Bacillus_cereus]	CPTF_Fe	1244711.267	33529.4816	2.693755773
UIJ67740.1	D-alanine--poly(phosphoribitol)_ligase_subunit_DltA_[Bacillus_cereus]	CPTF_Mn	1138881.333	121989.8273	10.71137297
UIJ67740.1	D-alanine--poly(phosphoribitol)_ligase_subunit_DltA_[Bacillus_cereus]	CPTF_Ni	1345467.867	86810.8234	6.452091912
UIJ67740.1	D-alanine--poly(phosphoribitol)_ligase_subunit_DltA_[Bacillus_cereus]	CPTF_U	1352198.4	36886.80247	2.727913483
UIJ67740.1	D-alanine--poly(phosphoribitol)_ligase_subunit_DltA_[Bacillus_cereus]	CPTF_metals_mix	1627772.267	94979.57175	5.834942252

UIJ67740.1	D-alanine--poly(phosphoribitol)_ligase_subunit_DltA_[Bacillus_cereus]	CPTF_zcontrol	1343423.333	39536.49297	2.942966077
UIJ67752.1	bacitracin_resistance_undecaprenyl-diphosphatase_[Bacillus_cereus]	CPTF_Al	380288.3333	45240.45647	11.89635666
UIJ67752.1	bacitracin_resistance_undecaprenyl-diphosphatase_[Bacillus_cereus]	CPTF_Cd	217691.3333	201711.7183	92.65950792
UIJ67752.1	bacitracin_resistance_undecaprenyl-diphosphatase_[Bacillus_cereus]	CPTF_Co	449322.3333	40708.88042	9.060061654
UIJ67752.1	bacitracin_resistance_undecaprenyl-diphosphatase_[Bacillus_cereus]	CPTF_Cu	554252.6667	86465.34817	15.60034861
UIJ67752.1	bacitracin_resistance_undecaprenyl-diphosphatase_[Bacillus_cereus]	CPTF_Fe	136324.6667	236121.249	173.2050808
UIJ67752.1	bacitracin_resistance_undecaprenyl-diphosphatase_[Bacillus_cereus]	CPTF_Mn	121306.3333	210108.7326	173.2050808
UIJ67752.1	bacitracin_resistance_undecaprenyl-diphosphatase_[Bacillus_cereus]	CPTF_Ni	382497	141976.0208	37.11820505
UIJ67752.1	bacitracin_resistance_undecaprenyl-diphosphatase_[Bacillus_cereus]	CPTF_U	77610.33333	134425.0405	173.2050808
UIJ67752.1	bacitracin_resistance_undecaprenyl-diphosphatase_[Bacillus_cereus]	CPTF_metals_mix	523785.6667	25387.61181	4.846946647
UIJ67752.1	bacitracin_resistance_undecaprenyl-diphosphatase_[Bacillus_cereus]	CPTF_zcontrol	250641.3333	217111.3923	86.62234172
UIJ67753.1	thioredoxin_family_protein_[Bacillus_cereus]	CPTF_Al	193349.4333	21038.27264	10.88095904
UIJ67753.1	thioredoxin_family_protein_[Bacillus_cereus]	CPTF_Cd	143144.2667	160505.4195	112.1284305
UIJ67753.1	thioredoxin_family_protein_[Bacillus_cereus]	CPTF_Co	102388.9333	74703.73908	72.96075527
UIJ67753.1	thioredoxin_family_protein_[Bacillus_cereus]	CPTF_Cu	225145.5667	6028.108971	2.677427346
UIJ67753.1	thioredoxin_family_protein_[Bacillus_cereus]	CPTF_Fe	140042.1667	67084.09082	47.90277987
UIJ67753.1	thioredoxin_family_protein_[Bacillus_cereus]	CPTF_Mn	146394.9333	55160.46136	37.67921478
UIJ67753.1	thioredoxin_family_protein_[Bacillus_cereus]	CPTF_Ni	156731.4	53868.40704	34.36988825
UIJ67753.1	thioredoxin_family_protein_[Bacillus_cereus]	CPTF_U	36160.66667	62632.1119	173.2050808
UIJ67753.1	thioredoxin_family_protein_[Bacillus_cereus]	CPTF_metals_mix	1449857.4	79402.61682	5.476581133
UIJ67753.1	thioredoxin_family_protein_[Bacillus_cereus]	CPTF_zcontrol	105145.9	95624.61485	90.94469195
UIJ67763.1	branched-chain-amino-acid_transaminase_[Bacillus_cereus]	CPTF_Al	27729278.6	4241657.005	15.29667276
UIJ67763.1	branched-chain-amino-acid_transaminase_[Bacillus_cereus]	CPTF_Cd	30725976	5161544.31	16.79863419
UIJ67763.1	branched-chain-amino-acid_transaminase_[Bacillus_cereus]	CPTF_Co	29559500.3	2545869.001	8.61269296
UIJ67763.1	branched-chain-amino-acid_transaminase_[Bacillus_cereus]	CPTF_Cu	30722426.27	430451.2371	1.401097795
UIJ67763.1	branched-chain-amino-acid_transaminase_[Bacillus_cereus]	CPTF_Fe	29677495.67	3823835.882	12.88463126
UIJ67763.1	branched-chain-amino-acid_transaminase_[Bacillus_cereus]	CPTF_Mn	27013824.37	4051696.67	14.99860447
UIJ67763.1	branched-chain-amino-acid_transaminase_[Bacillus_cereus]	CPTF_Ni	25524185.47	1238857.793	4.8536624
UIJ67763.1	branched-chain-amino-acid_transaminase_[Bacillus_cereus]	CPTF_U	24202741.9	730182.73	3.016942184
UIJ67763.1	branched-chain-amino-acid_transaminase_[Bacillus_cereus]	CPTF_metals_mix	37798559.03	6073737.039	16.06869996
UIJ67763.1	branched-chain-amino-acid_transaminase_[Bacillus_cereus]	CPTF_zcontrol	27487237.67	399958.3188	1.455069162
UIJ67764.1	acetolactate_synthase_large_subunit_[Bacillus_cereus]	CPTF_Al	5217765.767	938315.9636	17.98309862
UIJ67764.1	acetolactate_synthase_large_subunit_[Bacillus_cereus]	CPTF_Cd	5109143.367	148246.3075	2.901588325
UIJ67764.1	acetolactate_synthase_large_subunit_[Bacillus_cereus]	CPTF_Co	5490990.367	472451.5963	8.604123569
UIJ67764.1	acetolactate_synthase_large_subunit_[Bacillus_cereus]	CPTF_Cu	4887747.133	49165.55123	1.00589392
UIJ67764.1	acetolactate_synthase_large_subunit_[Bacillus_cereus]	CPTF_Fe	4699284.867	61372.84524	1.306003934
UIJ67764.1	acetolactate_synthase_large_subunit_[Bacillus_cereus]	CPTF_Mn	4847755.933	171110.0195	3.529674798
UIJ67764.1	acetolactate_synthase_large_subunit_[Bacillus_cereus]	CPTF_Ni	4719435.067	344041.4607	7.28988652
UIJ67764.1	acetolactate_synthase_large_subunit_[Bacillus_cereus]	CPTF_U	5735482.667	894102.0395	15.58895897
UIJ67764.1	acetolactate_synthase_large_subunit_[Bacillus_cereus]	CPTF_metals_mix	4475044.4	238086.0092	5.32030496
UIJ67764.1	acetolactate_synthase_large_subunit_[Bacillus_cereus]	CPTF_zcontrol	4984688.533	97882.6552	1.963666427
UIJ67765.1	acetolactate_synthase_small_subunit_[Bacillus_cereus]	CPTF_Al	1695286.667	102621.8219	6.053361001
UIJ67765.1	acetolactate_synthase_small_subunit_[Bacillus_cereus]	CPTF_Cd	1489915.8	317665.5596	21.32104107
UIJ67765.1	acetolactate_synthase_small_subunit_[Bacillus_cereus]	CPTF_Co	1663715.367	88009.82929	5.289957107
UIJ67765.1	acetolactate_synthase_small_subunit_[Bacillus_cereus]	CPTF_Cu	1895517.667	163032.43	8.600944896
UIJ67765.1	acetolactate_synthase_small_subunit_[Bacillus_cereus]	CPTF_Fe	1574480.967	134223.9136	8.524962605
UIJ67765.1	acetolactate_synthase_small_subunit_[Bacillus_cereus]	CPTF_Mn	1600663	127339.9875	7.955452681
UIJ67765.1	acetolactate_synthase_small_subunit_[Bacillus_cereus]	CPTF_Ni	1545012.667	123861.1708	8.016838533
UIJ67765.1	acetolactate_synthase_small_subunit_[Bacillus_cereus]	CPTF_U	945845.4333	644926.3344	68.18517187
UIJ67765.1	acetolactate_synthase_small_subunit_[Bacillus_cereus]	CPTF_metals_mix	1352451	18693.22415	1.382173857
UIJ67765.1	acetolactate_synthase_small_subunit_[Bacillus_cereus]	CPTF_zcontrol	1562969.367	163238.5641	10.44413074
UIJ67766.1	ketol-acid_reductoisomerase_[Bacillus_cereus]	CPTF_Al	12021683.6	605356.2759	5.035536586
UIJ67766.1	ketol-acid_reductoisomerase_[Bacillus_cereus]	CPTF_Cd	14482460.87	535062.9143	3.694557984
UIJ67766.1	ketol-acid_reductoisomerase_[Bacillus_cereus]	CPTF_Co	12604493.87	787550.402	6.248171567
UIJ67766.1	ketol-acid_reductoisomerase_[Bacillus_cereus]	CPTF_Cu	11591916.33	392944.8286	3.389817674
UIJ67766.1	ketol-acid_reductoisomerase_[Bacillus_cereus]	CPTF_Fe	11992627.13	696820.2302	5.810405197
UIJ67766.1	ketol-acid_reductoisomerase_[Bacillus_cereus]	CPTF_Mn	14673217.43	4894005.134	33.35331979
UIJ67766.1	ketol-acid_reductoisomerase_[Bacillus_cereus]	CPTF_Ni	9760922.367	949021.2318	9.722659357
UIJ67766.1	ketol-acid_reductoisomerase_[Bacillus_cereus]	CPTF_U	10635370.17	1340518.452	12.60434222
UIJ67766.1	ketol-acid_reductoisomerase_[Bacillus_cereus]	CPTF_metals_mix	17394460.7	570805.4719	3.281535897
UIJ67766.1	ketol-acid_reductoisomerase_[Bacillus_cereus]	CPTF_zcontrol	11412925.67	893576.7055	7.829514812

UIJ67767.1	2-isopropylmalate_synthase_[Bacillus_cereus]	CPTF_Al	7493170.433	429062.9868	5.72605402
UIJ67767.1	2-isopropylmalate_synthase_[Bacillus_cereus]	CPTF_Cd	7349765.867	905003.717	12.31336798
UIJ67767.1	2-isopropylmalate_synthase_[Bacillus_cereus]	CPTF_Co	8389691.067	911021.6151	10.85882195
UIJ67767.1	2-isopropylmalate_synthase_[Bacillus_cereus]	CPTF_Cu	7962623.933	342852.0167	4.305766787
UIJ67767.1	2-isopropylmalate_synthase_[Bacillus_cereus]	CPTF_Fe	7262835.267	559793.5153	7.707644394
UIJ67767.1	2-isopropylmalate_synthase_[Bacillus_cereus]	CPTF_Mn	7542420.167	460307.4731	6.102914754
UIJ67767.1	2-isopropylmalate_synthase_[Bacillus_cereus]	CPTF_Ni	7248644.967	285178.4762	3.934231536
UIJ67767.1	2-isopropylmalate_synthase_[Bacillus_cereus]	CPTF_U	6565843.8	627386.2553	9.555302782
UIJ67767.1	2-isopropylmalate_synthase_[Bacillus_cereus]	CPTF_metals_mix	7303478.833	237726.413	3.254975039
UIJ67767.1	2-isopropylmalate_synthase_[Bacillus_cereus]	CPTF_zcontrol	7583001.867	274936.5312	3.625695153
UIJ67768.1	3-isopropylmalate_dehydrogenase_[Bacillus_cereus]	CPTF_Al	6845273.9	416927.2956	6.090732112
UIJ67768.1	3-isopropylmalate_dehydrogenase_[Bacillus_cereus]	CPTF_Cd	6775615.667	371236.6491	5.47900984
UIJ67768.1	3-isopropylmalate_dehydrogenase_[Bacillus_cereus]	CPTF_Co	6712180	781629.5913	11.64494384
UIJ67768.1	3-isopropylmalate_dehydrogenase_[Bacillus_cereus]	CPTF_Cu	6248665.233	331228.3431	5.30078554
UIJ67768.1	3-isopropylmalate_dehydrogenase_[Bacillus_cereus]	CPTF_Fe	6512586.967	673065.8981	10.33484699
UIJ67768.1	3-isopropylmalate_dehydrogenase_[Bacillus_cereus]	CPTF_Mn	6790667.4	684410.0127	10.07868553
UIJ67768.1	3-isopropylmalate_dehydrogenase_[Bacillus_cereus]	CPTF_Ni	5232808	195160.5946	3.729557718
UIJ67768.1	3-isopropylmalate_dehydrogenase_[Bacillus_cereus]	CPTF_U	5085585.333	1010107.922	19.86217624
UIJ67768.1	3-isopropylmalate_dehydrogenase_[Bacillus_cereus]	CPTF_metals_mix	4481589.1	333702.6926	7.446079619
UIJ67768.1	3-isopropylmalate_dehydrogenase_[Bacillus_cereus]	CPTF_zcontrol	5950754.433	1123976.363	18.88796414
UIJ67769.1	3-isopropylmalate_dehydratase_large_subunit_[Bacillus_cereus]	CPTF_Al	439751	146193.3996	33.24458606
UIJ67769.1	3-isopropylmalate_dehydratase_large_subunit_[Bacillus_cereus]	CPTF_Cd	797510.3333	57582.06191	7.22022769
UIJ67769.1	3-isopropylmalate_dehydratase_large_subunit_[Bacillus_cereus]	CPTF_Co	531787.6667	237624.508	44.68409535
UIJ67769.1	3-isopropylmalate_dehydratase_large_subunit_[Bacillus_cereus]	CPTF_Cu	487626.6667	311467.6929	63.87421242
UIJ67769.1	3-isopropylmalate_dehydratase_large_subunit_[Bacillus_cereus]	CPTF_Fe	504856	134455.1006	26.63236658
UIJ67769.1	3-isopropylmalate_dehydratase_large_subunit_[Bacillus_cereus]	CPTF_Mn	549820.3333	178455.9887	32.45714606
UIJ67769.1	3-isopropylmalate_dehydratase_large_subunit_[Bacillus_cereus]	CPTF_Ni	118042.3333	102269.197	86.63772912
UIJ67769.1	3-isopropylmalate_dehydratase_large_subunit_[Bacillus_cereus]	CPTF_U	119928	207721.3893	173.2050808
UIJ67769.1	3-isopropylmalate_dehydratase_large_subunit_[Bacillus_cereus]	CPTF_metals_mix	0	0	0
UIJ67769.1	3-isopropylmalate_dehydratase_large_subunit_[Bacillus_cereus]	CPTF_zcontrol	311950	292415.6003	93.73797092
UIJ67775.1	1-(5-phosphoribosyl)-5-[(5-phosphoribosylamino)methylideneamino]imidazole-4-carboxamide_isomerase_[Bacillus_cereus]	CPTF_Al	217520.6667	45482.10682	20.90932669
UIJ67775.1	1-(5-phosphoribosyl)-5-[(5-phosphoribosylamino)methylideneamino]imidazole-4-carboxamide_isomerase_[Bacillus_cereus]	CPTF_Cd	53320.66667	92354.10376	173.2050808
UIJ67775.1	1-(5-phosphoribosyl)-5-[(5-phosphoribosylamino)methylideneamino]imidazole-4-carboxamide_isomerase_[Bacillus_cereus]	CPTF_Co	118183.6667	121509.962	102.8145136
UIJ67775.1	1-(5-phosphoribosyl)-5-[(5-phosphoribosylamino)methylideneamino]imidazole-4-carboxamide_isomerase_[Bacillus_cereus]	CPTF_Cu	148296.1	46788.67048	31.55084354
UIJ67775.1	1-(5-phosphoribosyl)-5-[(5-phosphoribosylamino)methylideneamino]imidazole-4-carboxamide_isomerase_[Bacillus_cereus]	CPTF_Fe	218862.3333	72154.67232	32.96806317
UIJ67775.1	1-(5-phosphoribosyl)-5-[(5-phosphoribosylamino)methylideneamino]imidazole-4-carboxamide_isomerase_[Bacillus_cereus]	CPTF_Mn	47832.33333	82848.03158	173.2050808
UIJ67775.1	1-(5-phosphoribosyl)-5-[(5-phosphoribosylamino)methylideneamino]imidazole-4-carboxamide_isomerase_[Bacillus_cereus]	CPTF_Ni	125349.3667	60529.62333	48.28873487
UIJ67775.1	1-(5-phosphoribosyl)-5-[(5-phosphoribosylamino)methylideneamino]imidazole-4-carboxamide_isomerase_[Bacillus_cereus]	CPTF_U	139645	121068.1898	86.69711755
UIJ67775.1	1-(5-phosphoribosyl)-5-[(5-phosphoribosylamino)methylideneamino]imidazole-4-carboxamide_isomerase_[Bacillus_cereus]	CPTF_metals_mix	65825.3	58747.86987	89.24816122
UIJ67775.1	1-(5-phosphoribosyl)-5-[(5-phosphoribosylamino)methylideneamino]imidazole-4-carboxamide_isomerase_[Bacillus_cereus]	CPTF_zcontrol	72167.9	79285.8296	109.8630133
UIJ67781.1	glycerate_dehydrogenase_[Bacillus_cereus]	CPTF_Al	1577160.233	420016.7347	26.63120245
UIJ67781.1	glycerate_dehydrogenase_[Bacillus_cereus]	CPTF_Cd	1777826.9	184157.01	10.35854559
UIJ67781.1	glycerate_dehydrogenase_[Bacillus_cereus]	CPTF_Co	2036649.667	216170.372	10.61401848
UIJ67781.1	glycerate_dehydrogenase_[Bacillus_cereus]	CPTF_Cu	1823751.333	558570.3458	30.62754969
UIJ67781.1	glycerate_dehydrogenase_[Bacillus_cereus]	CPTF_Fe	903338.1333	46252.00953	5.12012145
UIJ67781.1	glycerate_dehydrogenase_[Bacillus_cereus]	CPTF_Mn	1519703.5	513316.6743	33.77742265
UIJ67781.1	glycerate_dehydrogenase_[Bacillus_cereus]	CPTF_Ni	1257415.033	58210.50268	4.629378617
UIJ67781.1	glycerate_dehydrogenase_[Bacillus_cereus]	CPTF_U	1469807.833	477797.6065	32.5074881
UIJ67781.1	glycerate_dehydrogenase_[Bacillus_cereus]	CPTF_metals_mix	3519437.433	64926.11288	1.844786677
UIJ67781.1	glycerate_dehydrogenase_[Bacillus_cereus]	CPTF_zcontrol	1640467.133	107205.3688	6.535051304
UIJ67785.1	diaminopimelate_decarboxylase_[Bacillus_cereus]	CPTF_Al	95572.5	35000.85016	36.62230261
UIJ67785.1	diaminopimelate_decarboxylase_[Bacillus_cereus]	CPTF_Cd	44307.33333	76742.55248	173.2050808
UIJ67785.1	diaminopimelate_decarboxylase_[Bacillus_cereus]	CPTF_Co	111719.2	56918.46467	50.94779114
UIJ67785.1	diaminopimelate_decarboxylase_[Bacillus_cereus]	CPTF_Cu	138691.3333	30065.18036	21.67776431
UIJ67785.1	diaminopimelate_decarboxylase_[Bacillus_cereus]	CPTF_Fe	52567.1	52533.0332	99.93519369
UIJ67785.1	diaminopimelate_decarboxylase_[Bacillus_cereus]	CPTF_Mn	57673.86667	68554.24716	118.8653564
UIJ67785.1	diaminopimelate_decarboxylase_[Bacillus_cereus]	CPTF_Ni	90248.43333	105937.4533	117.3842574
UIJ67785.1	diaminopimelate_decarboxylase_[Bacillus_cereus]	CPTF_U	55266.46667	47938.82407	86.7412501
UIJ67785.1	diaminopimelate_decarboxylase_[Bacillus_cereus]	CPTF_metals_mix	678759	116871.4008	17.21839428
UIJ67785.1	diaminopimelate_decarboxylase_[Bacillus_cereus]	CPTF_zcontrol	47014.66667	7610.304197	16.1870853
UIJ67787.1	phosphoadenylyl-sulfate_reductase_[Bacillus_cereus]	CPTF_Al	105647.6667	97018.48463	91.832113

UIJ67787.1	phosphoadenylyl-sulfate_reductase_[Bacillus_cereus]	CPTF_Cd	0	0	0
UIJ67787.1	phosphoadenylyl-sulfate_reductase_[Bacillus_cereus]	CPTF_Co	110924.9667	111368.5693	100.3999123
UIJ67787.1	phosphoadenylyl-sulfate_reductase_[Bacillus_cereus]	CPTF_Cu	117116.0333	101567.3099	86.72365944
UIJ67787.1	phosphoadenylyl-sulfate_reductase_[Bacillus_cereus]	CPTF_Fe	104363.3	158455.2606	151.8304429
UIJ67787.1	phosphoadenylyl-sulfate_reductase_[Bacillus_cereus]	CPTF_Mn	28026.03333	48542.51367	173.2050808
UIJ67787.1	phosphoadenylyl-sulfate_reductase_[Bacillus_cereus]	CPTF_Ni	68465.4	94610.47288	138.1872784
UIJ67787.1	phosphoadenylyl-sulfate_reductase_[Bacillus_cereus]	CPTF_U	0	0	0
UIJ67787.1	phosphoadenylyl-sulfate_reductase_[Bacillus_cereus]	CPTF_metals_mix	0	0	0
UIJ67787.1	phosphoadenylyl-sulfate_reductase_[Bacillus_cereus]	CPTF_zcontrol	135038.3667	117410.622	86.94612122
UIJ67788.1	sulfate_adenylyltransferase_[Bacillus_cereus]	CPTF_Al	3530025.667	342734.8779	9.709132746
UIJ67788.1	sulfate_adenylyltransferase_[Bacillus_cereus]	CPTF_Cd	2852068.9	304639.9504	10.68136714
UIJ67788.1	sulfate_adenylyltransferase_[Bacillus_cereus]	CPTF_Co	3192544.233	391565.6848	12.26500422
UIJ67788.1	sulfate_adenylyltransferase_[Bacillus_cereus]	CPTF_Cu	3834829.867	309550.4254	8.072077149
UIJ67788.1	sulfate_adenylyltransferase_[Bacillus_cereus]	CPTF_Fe	2842685.833	305606.2335	10.75061584
UIJ67788.1	sulfate_adenylyltransferase_[Bacillus_cereus]	CPTF_Mn	3019295.067	467079.6135	15.46982336
UIJ67788.1	sulfate_adenylyltransferase_[Bacillus_cereus]	CPTF_Ni	2743882.833	843450.5919	30.73930788
UIJ67788.1	sulfate_adenylyltransferase_[Bacillus_cereus]	CPTF_U	2369517.533	435043.4054	18.35999942
UIJ67788.1	sulfate_adenylyltransferase_[Bacillus_cereus]	CPTF_metals_mix	1309492.767	252249.474	19.26314374
UIJ67788.1	sulfate_adenylyltransferase_[Bacillus_cereus]	CPTF_zcontrol	3030878.367	127705.2533	4.213473383
UIJ67789.1	adenylyl-sulfate_kinase_[Bacillus_cereus]	CPTF_Al	1673809.333	256305.6482	15.31271472
UIJ67789.1	adenylyl-sulfate_kinase_[Bacillus_cereus]	CPTF_Cd	1412665.6	190148.0269	13.46022915
UIJ67789.1	adenylyl-sulfate_kinase_[Bacillus_cereus]	CPTF_Co	1392925	93704.32097	6.727161977
UIJ67789.1	adenylyl-sulfate_kinase_[Bacillus_cereus]	CPTF_Cu	1756927.667	192041.3007	10.93051833
UIJ67789.1	adenylyl-sulfate_kinase_[Bacillus_cereus]	CPTF_Fe	1492028.333	68941.29752	4.62064265
UIJ67789.1	adenylyl-sulfate_kinase_[Bacillus_cereus]	CPTF_Mn	1652874.133	84699.52017	5.124378104
UIJ67789.1	adenylyl-sulfate_kinase_[Bacillus_cereus]	CPTF_Ni	1409135.533	435888.6371	30.9330527
UIJ67789.1	adenylyl-sulfate_kinase_[Bacillus_cereus]	CPTF_U	1332651.667	385623.1503	28.93653007
UIJ67789.1	adenylyl-sulfate_kinase_[Bacillus_cereus]	CPTF_metals_mix	703885.9	61459.23909	8.731420688
UIJ67789.1	adenylyl-sulfate_kinase_[Bacillus_cereus]	CPTF_zcontrol	1830086.667	156484.3806	8.550654098
UIJ67790.1	nitrite/sulfite_reductase_[Bacillus_cereus]	CPTF_Al	3533491.833	379686.816	10.74537126
UIJ67790.1	nitrite/sulfite_reductase_[Bacillus_cereus]	CPTF_Cd	2586170.833	321975.6007	12.44989683
UIJ67790.1	nitrite/sulfite_reductase_[Bacillus_cereus]	CPTF_Co	3455845.267	313947.2158	9.084527564
UIJ67790.1	nitrite/sulfite_reductase_[Bacillus_cereus]	CPTF_Cu	4475465.5	265842.4104	5.93999463
UIJ67790.1	nitrite/sulfite_reductase_[Bacillus_cereus]	CPTF_Fe	3095177.4	725043.6094	23.42494519
UIJ67790.1	nitrite/sulfite_reductase_[Bacillus_cereus]	CPTF_Mn	3174931.267	249130.2755	7.84679272
UIJ67790.1	nitrite/sulfite_reductase_[Bacillus_cereus]	CPTF_Ni	3212560.6	330828.4969	10.29796907
UIJ67790.1	nitrite/sulfite_reductase_[Bacillus_cereus]	CPTF_U	2960592.233	884280.518	29.8683658
UIJ67790.1	nitrite/sulfite_reductase_[Bacillus_cereus]	CPTF_metals_mix	741543.0333	152293.1973	20.53733774
UIJ67790.1	nitrite/sulfite_reductase_[Bacillus_cereus]	CPTF_zcontrol	3607898	331049.1756	9.17568001
UIJ67791.1	DUF3906_family_protein_[Bacillus_cereus]	CPTF_Al	42717.33333	73988.5917	173.2050808
UIJ67791.1	DUF3906_family_protein_[Bacillus_cereus]	CPTF_Cd	73929.7	66492.40244	89.94004093
UIJ67791.1	DUF3906_family_protein_[Bacillus_cereus]	CPTF_Co	142340.3333	10722.67049	7.533121663
UIJ67791.1	DUF3906_family_protein_[Bacillus_cereus]	CPTF_Cu	153580.6667	23962.26609	15.6023975
UIJ67791.1	DUF3906_family_protein_[Bacillus_cereus]	CPTF_Fe	80957.83333	80544.72809	99.48972789
UIJ67791.1	DUF3906_family_protein_[Bacillus_cereus]	CPTF_Mn	15705.56667	27202.83943	173.2050808
UIJ67791.1	DUF3906_family_protein_[Bacillus_cereus]	CPTF_Ni	53931.33333	93411.80945	173.2050808
UIJ67791.1	DUF3906_family_protein_[Bacillus_cereus]	CPTF_U	44804.66667	77603.95908	173.2050808
UIJ67791.1	DUF3906_family_protein_[Bacillus_cereus]	CPTF_metals_mix	0	0	0
UIJ67791.1	DUF3906_family_protein_[Bacillus_cereus]	CPTF_zcontrol	96962.26667	29289.60734	30.2072222
UIJ67793.1	NAD(P)-binding_protein_[Bacillus_cereus]	CPTF_Al	12169.86667	21078.82739	173.2050808
UIJ67793.1	NAD(P)-binding_protein_[Bacillus_cereus]	CPTF_Cd	7757.433333	13436.26867	173.2050808
UIJ67793.1	NAD(P)-binding_protein_[Bacillus_cereus]	CPTF_Co	78763.03333	75006.24172	95.23026037
UIJ67793.1	NAD(P)-binding_protein_[Bacillus_cereus]	CPTF_Cu	73769.76667	23308.77989	31.59665666
UIJ67793.1	NAD(P)-binding_protein_[Bacillus_cereus]	CPTF_Fe	0	0	0
UIJ67793.1	NAD(P)-binding_protein_[Bacillus_cereus]	CPTF_Mn	0	0	0
UIJ67793.1	NAD(P)-binding_protein_[Bacillus_cereus]	CPTF_Ni	13315.46667	23063.06479	173.2050808
UIJ67793.1	NAD(P)-binding_protein_[Bacillus_cereus]	CPTF_U	0	0	0
UIJ67793.1	NAD(P)-binding_protein_[Bacillus_cereus]	CPTF_metals_mix	0	0	0
UIJ67793.1	NAD(P)-binding_protein_[Bacillus_cereus]	CPTF_zcontrol	20753.4	17983.00055	86.6508647
UIJ67800.1	PspA/IM30_family_protein_[Bacillus_cereus]	CPTF_Al	0	0	0
UIJ67800.1	PspA/IM30_family_protein_[Bacillus_cereus]	CPTF_Cd	0	0	0

UIJ67800.1	PspA/IM30_family_protein_[Bacillus_cereus]	CPTF_Co	0	0	0
UIJ67800.1	PspA/IM30_family_protein_[Bacillus_cereus]	CPTF_Cu	0	0	0
UIJ67800.1	PspA/IM30_family_protein_[Bacillus_cereus]	CPTF_Fe	0	0	0
UIJ67800.1	PspA/IM30_family_protein_[Bacillus_cereus]	CPTF_Mn	0	0	0
UIJ67800.1	PspA/IM30_family_protein_[Bacillus_cereus]	CPTF_Ni	0	0	0
UIJ67800.1	PspA/IM30_family_protein_[Bacillus_cereus]	CPTF_U	196867.3333	340984.2237	173.2050808
UIJ67800.1	PspA/IM30_family_protein_[Bacillus_cereus]	CPTF_metals_mix	69281.76667	64232.3259	92.71173208
UIJ67800.1	PspA/IM30_family_protein_[Bacillus_cereus]	CPTF_zcontrol	0	0	0
UIJ67811.1	NO-inducible_flavohemoprotein_[Bacillus_cereus]	CPTF_Al	3385109.567	847291.0884	25.02994576
UIJ67811.1	NO-inducible_flavohemoprotein_[Bacillus_cereus]	CPTF_Cd	3907220.967	884501.2016	22.63760379
UIJ67811.1	NO-inducible_flavohemoprotein_[Bacillus_cereus]	CPTF_Co	2149332.833	259060.692	12.0530747
UIJ67811.1	NO-inducible_flavohemoprotein_[Bacillus_cereus]	CPTF_Cu	3047478.5	375889.281	12.33443586
UIJ67811.1	NO-inducible_flavohemoprotein_[Bacillus_cereus]	CPTF_Fe	3310056.967	421600.0553	12.73694258
UIJ67811.1	NO-inducible_flavohemoprotein_[Bacillus_cereus]	CPTF_Mn	3151749.9	782491.4946	24.82720772
UIJ67811.1	NO-inducible_flavohemoprotein_[Bacillus_cereus]	CPTF_Ni	2242636.333	512975.1814	22.87375683
UIJ67811.1	NO-inducible_flavohemoprotein_[Bacillus_cereus]	CPTF_U	2747238.667	515521.4821	18.76507813
UIJ67811.1	NO-inducible_flavohemoprotein_[Bacillus_cereus]	CPTF_metals_mix	2254869.2	327336.9737	14.51689409
UIJ67811.1	NO-inducible_flavohemoprotein_[Bacillus_cereus]	CPTF_zcontrol	2666429.233	107558.9895	4.033821267
UIJ67816.1	DUF47_domain-containing_protein_[Bacillus_cereus]	CPTF_Al	452744.7	246635.8524	54.47570173
UIJ67816.1	DUF47_domain-containing_protein_[Bacillus_cereus]	CPTF_Cd	1063633.933	135906.1434	12.77752986
UIJ67816.1	DUF47_domain-containing_protein_[Bacillus_cereus]	CPTF_Co	597308.1	153633.4382	25.72097017
UIJ67816.1	DUF47_domain-containing_protein_[Bacillus_cereus]	CPTF_Cu	372170.3333	111181.4304	29.87380252
UIJ67816.1	DUF47_domain-containing_protein_[Bacillus_cereus]	CPTF_Fe	673479.4	361886.0486	53.73379626
UIJ67816.1	DUF47_domain-containing_protein_[Bacillus_cereus]	CPTF_Mn	662896.9667	440709.5833	66.48236536
UIJ67816.1	DUF47_domain-containing_protein_[Bacillus_cereus]	CPTF_Ni	208504.8	66007.6923	31.6576368
UIJ67816.1	DUF47_domain-containing_protein_[Bacillus_cereus]	CPTF_U	185722.1333	65948.98573	35.50949181
UIJ67816.1	DUF47_domain-containing_protein_[Bacillus_cereus]	CPTF_metals_mix	1930538.867	118274.5731	6.126505671
UIJ67816.1	DUF47_domain-containing_protein_[Bacillus_cereus]	CPTF_zcontrol	514180.3333	237081.4248	46.10861392
UIJ67817.1	PBP1A_family_penicillin-binding_protein_[Bacillus_cereus]	CPTF_Al	0	0	0
UIJ67817.1	PBP1A_family_penicillin-binding_protein_[Bacillus_cereus]	CPTF_Cd	0	0	0
UIJ67817.1	PBP1A_family_penicillin-binding_protein_[Bacillus_cereus]	CPTF_Co	17695.2	30648.98545	173.2050808
UIJ67817.1	PBP1A_family_penicillin-binding_protein_[Bacillus_cereus]	CPTF_Cu	0	0	0
UIJ67817.1	PBP1A_family_penicillin-binding_protein_[Bacillus_cereus]	CPTF_Fe	0	0	0
UIJ67817.1	PBP1A_family_penicillin-binding_protein_[Bacillus_cereus]	CPTF_Mn	0	0	0
UIJ67817.1	PBP1A_family_penicillin-binding_protein_[Bacillus_cereus]	CPTF_Ni	0	0	0
UIJ67817.1	PBP1A_family_penicillin-binding_protein_[Bacillus_cereus]	CPTF_U	0	0	0
UIJ67817.1	PBP1A_family_penicillin-binding_protein_[Bacillus_cereus]	CPTF_metals_mix	0	0	0
UIJ67817.1	PBP1A_family_penicillin-binding_protein_[Bacillus_cereus]	CPTF_zcontrol	19410.3	33619.62579	173.2050808
UIJ67821.1	DUF3924_domain-containing_protein_[Bacillus_cereus]	CPTF_Al	505016.3333	81059.15729	16.05079914
UIJ67821.1	DUF3924_domain-containing_protein_[Bacillus_cereus]	CPTF_Cd	485395	27888.94141	5.745617777
UIJ67821.1	DUF3924_domain-containing_protein_[Bacillus_cereus]	CPTF_Co	416617.9667	44116.03063	10.589085
UIJ67821.1	DUF3924_domain-containing_protein_[Bacillus_cereus]	CPTF_Cu	295888.4667	10578.19394	3.575061259
UIJ67821.1	DUF3924_domain-containing_protein_[Bacillus_cereus]	CPTF_Fe	518425	73994.3383	14.27291089
UIJ67821.1	DUF3924_domain-containing_protein_[Bacillus_cereus]	CPTF_Mn	378125.1	65780.43403	17.39647382
UIJ67821.1	DUF3924_domain-containing_protein_[Bacillus_cereus]	CPTF_Ni	522178	62924.96103	12.05048107
UIJ67821.1	DUF3924_domain-containing_protein_[Bacillus_cereus]	CPTF_U	275074.8	220674.0264	80.22327977
UIJ67821.1	DUF3924_domain-containing_protein_[Bacillus_cereus]	CPTF_metals_mix	24952.3	22869.73686	91.65382293
UIJ67821.1	DUF3924_domain-containing_protein_[Bacillus_cereus]	CPTF_zcontrol	312018.7667	60803.5526	19.487146
UIJ67822.1	tyrosine-type_recombinase/integrase_[Bacillus_cereus]	CPTF_Al	0	0	0
UIJ67822.1	tyrosine-type_recombinase/integrase_[Bacillus_cereus]	CPTF_Cd	0	0	0
UIJ67822.1	tyrosine-type_recombinase/integrase_[Bacillus_cereus]	CPTF_Co	0	0	0
UIJ67822.1	tyrosine-type_recombinase/integrase_[Bacillus_cereus]	CPTF_Cu	0	0	0
UIJ67822.1	tyrosine-type_recombinase/integrase_[Bacillus_cereus]	CPTF_Fe	0	0	0
UIJ67822.1	tyrosine-type_recombinase/integrase_[Bacillus_cereus]	CPTF_Mn	0	0	0
UIJ67822.1	tyrosine-type_recombinase/integrase_[Bacillus_cereus]	CPTF_Ni	0	0	0
UIJ67822.1	tyrosine-type_recombinase/integrase_[Bacillus_cereus]	CPTF_U	0	0	0
UIJ67822.1	tyrosine-type_recombinase/integrase_[Bacillus_cereus]	CPTF_metals_mix	64585.2	69516.12012	107.6347524
UIJ67822.1	tyrosine-type_recombinase/integrase_[Bacillus_cereus]	CPTF_zcontrol	0	0	0
UIJ67824.1	purine-nucleoside_phosphorylase_[Bacillus_cereus]	CPTF_Al	1243130.933	111523.5937	8.971186435
UIJ67824.1	purine-nucleoside_phosphorylase_[Bacillus_cereus]	CPTF_Cd	1330333	79688.88126	5.990145419
UIJ67824.1	purine-nucleoside_phosphorylase_[Bacillus_cereus]	CPTF_Co	1207105.267	43505.96032	3.604156284

UIJ67824.1	purine-nucleoside_phosphorylase_[Bacillus_cereus]	CPTF_Cu	1683493.333	71949.74694	4.273836166
UIJ67824.1	purine-nucleoside_phosphorylase_[Bacillus_cereus]	CPTF_Fe	1116377.967	60514.27488	5.42059022
UIJ67824.1	purine-nucleoside_phosphorylase_[Bacillus_cereus]	CPTF_Mn	1194124.967	51095.86073	4.278937478
UIJ67824.1	purine-nucleoside_phosphorylase_[Bacillus_cereus]	CPTF_Ni	1268546.667	130206.6868	10.26424098
UIJ67824.1	purine-nucleoside_phosphorylase_[Bacillus_cereus]	CPTF_U	1095089.667	97222.34262	8.878025752
UIJ67824.1	purine-nucleoside_phosphorylase_[Bacillus_cereus]	CPTF_metals_mix	1490069.433	40921.13511	2.746256932
UIJ67824.1	purine-nucleoside_phosphorylase_[Bacillus_cereus]	CPTF_zcontrol	1246279	17882.75776	1.434892007
UIJ67828.1	YpuI_family_protein_[Bacillus_cereus]	CPTF_Al	160120.7	14393.51884	8.989168074
UIJ67828.1	YpuI_family_protein_[Bacillus_cereus]	CPTF_Cd	181902.6667	10141.75873	5.575376609
UIJ67828.1	YpuI_family_protein_[Bacillus_cereus]	CPTF_Co	137878.3	16051.97162	11.6421305
UIJ67828.1	YpuI_family_protein_[Bacillus_cereus]	CPTF_Cu	188542.6333	20300.34205	10.76697704
UIJ67828.1	YpuI_family_protein_[Bacillus_cereus]	CPTF_Fe	153876	34793.86631	22.61162645
UIJ67828.1	YpuI_family_protein_[Bacillus_cereus]	CPTF_Mn	80787.1	27396.01815	33.91137713
UIJ67828.1	YpuI_family_protein_[Bacillus_cereus]	CPTF_Ni	172129.8	51175.60559	29.73082267
UIJ67828.1	YpuI_family_protein_[Bacillus_cereus]	CPTF_U	69629.53333	65992.27253	94.77626716
UIJ67828.1	YpuI_family_protein_[Bacillus_cereus]	CPTF_metals_mix	144572.2	9053.39981	6.262199655
UIJ67828.1	YpuI_family_protein_[Bacillus_cereus]	CPTF_zcontrol	109432.1333	24622.35706	22.50011611
UIJ67833.1	23S_rRNA_pseudouridine(2605)_synthase_RluB_[Bacillus_cereus]	CPTF_Al	7412.766667	12839.28849	173.2050808
UIJ67833.1	23S_rRNA_pseudouridine(2605)_synthase_RluB_[Bacillus_cereus]	CPTF_Cd	0	0	0
UIJ67833.1	23S_rRNA_pseudouridine(2605)_synthase_RluB_[Bacillus_cereus]	CPTF_Co	0	0	0
UIJ67833.1	23S_rRNA_pseudouridine(2605)_synthase_RluB_[Bacillus_cereus]	CPTF_Cu	0	0	0
UIJ67833.1	23S_rRNA_pseudouridine(2605)_synthase_RluB_[Bacillus_cereus]	CPTF_Fe	0	0	0
UIJ67833.1	23S_rRNA_pseudouridine(2605)_synthase_RluB_[Bacillus_cereus]	CPTF_Mn	0	0	0
UIJ67833.1	23S_rRNA_pseudouridine(2605)_synthase_RluB_[Bacillus_cereus]	CPTF_Ni	0	0	0
UIJ67833.1	23S_rRNA_pseudouridine(2605)_synthase_RluB_[Bacillus_cereus]	CPTF_U	0	0	0
UIJ67833.1	23S_rRNA_pseudouridine(2605)_synthase_RluB_[Bacillus_cereus]	CPTF_metals_mix	32948.16667	57067.89868	173.2050808
UIJ67833.1	23S_rRNA_pseudouridine(2605)_synthase_RluB_[Bacillus_cereus]	CPTF_zcontrol	0	0	0
UIJ67834.1	thiol-disulfide_oxidoreductase_ResA_[Bacillus_cereus]	CPTF_Al	72650.53333	72130.25463	99.28386114
UIJ67834.1	thiol-disulfide_oxidoreductase_ResA_[Bacillus_cereus]	CPTF_Cd	53158.26667	67436.13904	126.8591759
UIJ67834.1	thiol-disulfide_oxidoreductase_ResA_[Bacillus_cereus]	CPTF_Co	58533	101382.1299	173.2050808
UIJ67834.1	thiol-disulfide_oxidoreductase_ResA_[Bacillus_cereus]	CPTF_Cu	57603.33333	99771.90002	173.2050808
UIJ67834.1	thiol-disulfide_oxidoreductase_ResA_[Bacillus_cereus]	CPTF_Fe	43832.3	38130.63011	86.99208142
UIJ67834.1	thiol-disulfide_oxidoreductase_ResA_[Bacillus_cereus]	CPTF_Mn	36251.33333	62789.15118	173.2050808
UIJ67834.1	thiol-disulfide_oxidoreductase_ResA_[Bacillus_cereus]	CPTF_Ni	38794.66667	67194.33373	173.2050808
UIJ67834.1	thiol-disulfide_oxidoreductase_ResA_[Bacillus_cereus]	CPTF_U	110219.1333	24267.56634	22.01756229
UIJ67834.1	thiol-disulfide_oxidoreductase_ResA_[Bacillus_cereus]	CPTF_metals_mix	6301.733333	10914.92231	173.2050808
UIJ67834.1	thiol-disulfide_oxidoreductase_ResA_[Bacillus_cereus]	CPTF_zcontrol	32713.26667	56661.03995	173.2050808
UIJ67836.1	cytochrome_c_biogenesis_protein_ResC_[Bacillus_cereus]	CPTF_Al	1465260.667	500260.6759	34.14141165
UIJ67836.1	cytochrome_c_biogenesis_protein_ResC_[Bacillus_cereus]	CPTF_Cd	870054.9667	729495.1282	83.84471742
UIJ67836.1	cytochrome_c_biogenesis_protein_ResC_[Bacillus_cereus]	CPTF_Co	972672.6667	186165.1671	19.13954956
UIJ67836.1	cytochrome_c_biogenesis_protein_ResC_[Bacillus_cereus]	CPTF_Cu	0	0	0
UIJ67836.1	cytochrome_c_biogenesis_protein_ResC_[Bacillus_cereus]	CPTF_Fe	1030000	902164.0649	87.58874417
UIJ67836.1	cytochrome_c_biogenesis_protein_ResC_[Bacillus_cereus]	CPTF_Mn	333333.3333	577350.2692	173.2050808
UIJ67836.1	cytochrome_c_biogenesis_protein_ResC_[Bacillus_cereus]	CPTF_Ni	776341	1011331.738	130.2690104
UIJ67836.1	cytochrome_c_biogenesis_protein_ResC_[Bacillus_cereus]	CPTF_U	1023333.333	962306.2576	94.03644211
UIJ67836.1	cytochrome_c_biogenesis_protein_ResC_[Bacillus_cereus]	CPTF_metals_mix	94863.73333	117053.6358	123.3913443
UIJ67836.1	cytochrome_c_biogenesis_protein_ResC_[Bacillus_cereus]	CPTF_zcontrol	1323333.333	299054.0642	22.5985439
UIJ67837.1	DNA-binding_response_regulator_ResD_[Bacillus_cereus]	CPTF_Al	792878.2	95247.81101	12.01291838
UIJ67837.1	DNA-binding_response_regulator_ResD_[Bacillus_cereus]	CPTF_Cd	821174.1	38272.64808	4.660722748
UIJ67837.1	DNA-binding_response_regulator_ResD_[Bacillus_cereus]	CPTF_Co	882560.0667	48749.62342	5.523660684
UIJ67837.1	DNA-binding_response_regulator_ResD_[Bacillus_cereus]	CPTF_Cu	883385.5667	70879.32495	8.02360007
UIJ67837.1	DNA-binding_response_regulator_ResD_[Bacillus_cereus]	CPTF_Fe	836764.1667	74987.37858	8.961590562
UIJ67837.1	DNA-binding_response_regulator_ResD_[Bacillus_cereus]	CPTF_Mn	800986.1333	123994.1862	15.48019136
UIJ67837.1	DNA-binding_response_regulator_ResD_[Bacillus_cereus]	CPTF_Ni	906411.9667	108933.3981	12.01808914
UIJ67837.1	DNA-binding_response_regulator_ResD_[Bacillus_cereus]	CPTF_U	967941.6	99059.19169	10.23400499
UIJ67837.1	DNA-binding_response_regulator_ResD_[Bacillus_cereus]	CPTF_metals_mix	808604.3333	117582.7176	14.54144045
UIJ67837.1	DNA-binding_response_regulator_ResD_[Bacillus_cereus]	CPTF_zcontrol	808266.8667	66285.38673	8.200928364
UIJ67849.1	NAD-specific_glutamate_dehydrogenase_[Bacillus_cereus]	CPTF_Al	2732600.7	734093.3642	26.86427491
UIJ67849.1	NAD-specific_glutamate_dehydrogenase_[Bacillus_cereus]	CPTF_Cd	2716063.167	265964.1597	9.792267093
UIJ67849.1	NAD-specific_glutamate_dehydrogenase_[Bacillus_cereus]	CPTF_Co	2579336.4	651487.376	25.25794526
UIJ67849.1	NAD-specific_glutamate_dehydrogenase_[Bacillus_cereus]	CPTF_Cu	2322978.233	283529.4535	12.20542876

UIJ67849.1	NAD-specific_glutamate_dehydrogenase_[Bacillus_cereus]	CPTF_Fe	2703236.967	53617.43233	1.983452912
UIJ67849.1	NAD-specific_glutamate_dehydrogenase_[Bacillus_cereus]	CPTF_Mn	2976309.2	575546.5681	19.33759329
UIJ67849.1	NAD-specific_glutamate_dehydrogenase_[Bacillus_cereus]	CPTF_Ni	2229005.5	185227.0497	8.30985162
UIJ67849.1	NAD-specific_glutamate_dehydrogenase_[Bacillus_cereus]	CPTF_U	2172476.067	291658.0388	13.42514393
UIJ67849.1	NAD-specific_glutamate_dehydrogenase_[Bacillus_cereus]	CPTF_metals_mix	3572890.267	252824.2591	7.076183151
UIJ67849.1	NAD-specific_glutamate_dehydrogenase_[Bacillus_cereus]	CPTF_zcontrol	2783460.333	120918.7544	4.344188165
UIJ67855.1	(d)CMP_kinase_[Bacillus_cereus]	CPTF_Al	62198.06667	80506.558	129.4357885
UIJ67855.1	(d)CMP_kinase_[Bacillus_cereus]	CPTF_Cd	279299.2333	50928.65969	18.23444307
UIJ67855.1	(d)CMP_kinase_[Bacillus_cereus]	CPTF_Co	203999.9333	48527.22473	23.78786303
UIJ67855.1	(d)CMP_kinase_[Bacillus_cereus]	CPTF_Cu	232700.1	121063.7282	52.0256451
UIJ67855.1	(d)CMP_kinase_[Bacillus_cereus]	CPTF_Fe	181522.8333	211182.2032	116.3391951
UIJ67855.1	(d)CMP_kinase_[Bacillus_cereus]	CPTF_Mn	261580.5333	118354.0638	45.24574606
UIJ67855.1	(d)CMP_kinase_[Bacillus_cereus]	CPTF_Ni	110303.0333	117762.7722	106.7629499
UIJ67855.1	(d)CMP_kinase_[Bacillus_cereus]	CPTF_U	0	0	0
UIJ67855.1	(d)CMP_kinase_[Bacillus_cereus]	CPTF_metals_mix	172429.1333	41610.63426	24.13202076
UIJ67855.1	(d)CMP_kinase_[Bacillus_cereus]	CPTF_zcontrol	176905.2333	103146.3108	58.30596915
UIJ67856.1	30S_ribosomal_protein_S1_[Bacillus_cereus]	CPTF_Al	12809581.93	1076536.996	8.404154026
UIJ67856.1	30S_ribosomal_protein_S1_[Bacillus_cereus]	CPTF_Cd	15380603.6	916007.8986	5.95560436
UIJ67856.1	30S_ribosomal_protein_S1_[Bacillus_cereus]	CPTF_Co	12442553.33	501905.2236	4.033779966
UIJ67856.1	30S_ribosomal_protein_S1_[Bacillus_cereus]	CPTF_Cu	11438377.73	867392.2288	7.583175246
UIJ67856.1	30S_ribosomal_protein_S1_[Bacillus_cereus]	CPTF_Fe	12473062.43	2044921.123	16.39469965
UIJ67856.1	30S_ribosomal_protein_S1_[Bacillus_cereus]	CPTF_Mn	14293041.27	5012800.554	35.07161604
UIJ67856.1	30S_ribosomal_protein_S1_[Bacillus_cereus]	CPTF_Ni	10518892	965854.726	9.182095661
UIJ67856.1	30S_ribosomal_protein_S1_[Bacillus_cereus]	CPTF_U	10821443.57	583882.4863	5.395606258
UIJ67856.1	30S_ribosomal_protein_S1_[Bacillus_cereus]	CPTF_metals_mix	24681317.76	1649810.44	6.684450386
UIJ67856.1	30S_ribosomal_protein_S1_[Bacillus_cereus]	CPTF_zcontrol	11702649.07	854793.9595	7.304277473
UIJ67861.1	ribosome-associated_GTPase_EngA_[Bacillus_cereus]	CPTF_Al	560016.1333	95155.17395	16.99150583
UIJ67861.1	ribosome-associated_GTPase_EngA_[Bacillus_cereus]	CPTF_Cd	421320.5333	119544.2731	28.37371163
UIJ67861.1	ribosome-associated_GTPase_EngA_[Bacillus_cereus]	CPTF_Co	662981.4	71549.8488	10.79213516
UIJ67861.1	ribosome-associated_GTPase_EngA_[Bacillus_cereus]	CPTF_Cu	737871.1	75716.12327	10.26142957
UIJ67861.1	ribosome-associated_GTPase_EngA_[Bacillus_cereus]	CPTF_Fe	481688.8333	248247.9436	51.53699368
UIJ67861.1	ribosome-associated_GTPase_EngA_[Bacillus_cereus]	CPTF_Mn	394723.5	57202.35602	14.49175335
UIJ67861.1	ribosome-associated_GTPase_EngA_[Bacillus_cereus]	CPTF_Ni	487517.1333	54562.23379	11.19185974
UIJ67861.1	ribosome-associated_GTPase_EngA_[Bacillus_cereus]	CPTF_U	470578.5333	66599.60751	14.15270838
UIJ67861.1	ribosome-associated_GTPase_EngA_[Bacillus_cereus]	CPTF_metals_mix	908758.6333	196070.6926	21.57566217
UIJ67861.1	ribosome-associated_GTPase_EngA_[Bacillus_cereus]	CPTF_zcontrol	464620.2667	55710.50782	11.99054622
UIJ67862.1	NAD(P)H-dependent_glycerol-3-phosphate_dehydrogenase_[Bacillus_cereus]	CPTF_Al	898758.0667	56163.89131	6.249055601
UIJ67862.1	NAD(P)H-dependent_glycerol-3-phosphate_dehydrogenase_[Bacillus_cereus]	CPTF_Cd	711574.1667	289951.5024	40.74789615
UIJ67862.1	NAD(P)H-dependent_glycerol-3-phosphate_dehydrogenase_[Bacillus_cereus]	CPTF_Co	905560.3667	40585.14943	4.481771831
UIJ67862.1	NAD(P)H-dependent_glycerol-3-phosphate_dehydrogenase_[Bacillus_cereus]	CPTF_Cu	861988.2333	66702.47297	7.738211543
UIJ67862.1	NAD(P)H-dependent_glycerol-3-phosphate_dehydrogenase_[Bacillus_cereus]	CPTF_Fe	869710.2	38965.37085	4.480270652
UIJ67862.1	NAD(P)H-dependent_glycerol-3-phosphate_dehydrogenase_[Bacillus_cereus]	CPTF_Mn	785200.6667	96146.61522	12.24484636
UIJ67862.1	NAD(P)H-dependent_glycerol-3-phosphate_dehydrogenase_[Bacillus_cereus]	CPTF_Ni	735038.9667	47177.07324	6.418309148
UIJ67862.1	NAD(P)H-dependent_glycerol-3-phosphate_dehydrogenase_[Bacillus_cereus]	CPTF_U	801407.3333	56904.02208	7.100511777
UIJ67862.1	NAD(P)H-dependent_glycerol-3-phosphate_dehydrogenase_[Bacillus_cereus]	CPTF_metals_mix	776330.0333	53586.57054	6.902550235
UIJ67862.1	NAD(P)H-dependent_glycerol-3-phosphate_dehydrogenase_[Bacillus_cereus]	CPTF_zcontrol	875054.2333	110509.9475	12.62892553
UIJ67867.1	HU_family_DNA-binding_protein_[Bacillus_cereus]	CPTF_Al	9094116.8	2627686.65	28.89435783
UIJ67867.1	HU_family_DNA-binding_protein_[Bacillus_cereus]	CPTF_Cd	16813520.7	2883979.328	17.15273903
UIJ67867.1	HU_family_DNA-binding_protein_[Bacillus_cereus]	CPTF_Co	8554098.353	2361631.156	27.60818334
UIJ67867.1	HU_family_DNA-binding_protein_[Bacillus_cereus]	CPTF_Cu	7196132.967	810710.372	11.26591707
UIJ67867.1	HU_family_DNA-binding_protein_[Bacillus_cereus]	CPTF_Fe	10974871.3	4837660.108	44.0794245
UIJ67867.1	HU_family_DNA-binding_protein_[Bacillus_cereus]	CPTF_Mn	16967978.1	15781063.91	93.0049757
UIJ67867.1	HU_family_DNA-binding_protein_[Bacillus_cereus]	CPTF_Ni	5175251.367	898109.9047	17.35393783
UIJ67867.1	HU_family_DNA-binding_protein_[Bacillus_cereus]	CPTF_U	6814155	933758.4898	13.70321764
UIJ67867.1	HU_family_DNA-binding_protein_[Bacillus_cereus]	CPTF_metals_mix	68983270.07	8677261.026	12.57879051
UIJ67867.1	HU_family_DNA-binding_protein_[Bacillus_cereus]	CPTF_zcontrol	7657031.533	1344457.722	17.5584718
UIJ67868.1	GTP_cyclohydrolase_I_FolE_[Bacillus_cereus]	CPTF_Al	1558873.567	40133.49036	2.574518628
UIJ67868.1	GTP_cyclohydrolase_I_FolE_[Bacillus_cereus]	CPTF_Cd	1602595.6	50718.32591	3.164761335
UIJ67868.1	GTP_cyclohydrolase_I_FolE_[Bacillus_cereus]	CPTF_Co	1395674.933	98944.20792	7.089344772
UIJ67868.1	GTP_cyclohydrolase_I_FolE_[Bacillus_cereus]	CPTF_Cu	1521810.967	42443.8265	2.789034081
UIJ67868.1	GTP_cyclohydrolase_I_FolE_[Bacillus_cereus]	CPTF_Fe	1467764.767	85321.79479	5.813042848

UIJ67868.1	GTP_cyclohydrolase_I_FolE_[Bacillus_cereus]	CPTF_Mn	1427920.367	104581.7133	7.324057823
UIJ67868.1	GTP_cyclohydrolase_I_FolE_[Bacillus_cereus]	CPTF_Ni	1558111.267	106438.9289	6.83127907
UIJ67868.1	GTP_cyclohydrolase_I_FolE_[Bacillus_cereus]	CPTF_U	1195072.067	260589.9418	21.80537468
UIJ67868.1	GTP_cyclohydrolase_I_FolE_[Bacillus_cereus]	CPTF_metals_mix	1660763.667	339688.4553	20.45375041
UIJ67868.1	GTP_cyclohydrolase_I_FolE_[Bacillus_cereus]	CPTF_zcontrol	1541774.5	24237.56471	1.572056401
UIJ67870.1	2-heptaprenyl-1,4-naphthoquinone_methyltransferase_[Bacillus_cereus]	CPTF_Al	59820.26667	52135.27702	87.15320062
UIJ67870.1	2-heptaprenyl-1,4-naphthoquinone_methyltransferase_[Bacillus_cereus]	CPTF_Cd	72626.36667	23470.35229	32.31657229
UIJ67870.1	2-heptaprenyl-1,4-naphthoquinone_methyltransferase_[Bacillus_cereus]	CPTF_Co	85785.1	30355.12959	35.38508388
UIJ67870.1	2-heptaprenyl-1,4-naphthoquinone_methyltransferase_[Bacillus_cereus]	CPTF_Cu	69514.16667	96806.7171	139.2618537
UIJ67870.1	2-heptaprenyl-1,4-naphthoquinone_methyltransferase_[Bacillus_cereus]	CPTF_Fe	84260	8085.402419	9.595777853
UIJ67870.1	2-heptaprenyl-1,4-naphthoquinone_methyltransferase_[Bacillus_cereus]	CPTF_Mn	16392.13333	28392.00778	173.2050808
UIJ67870.1	2-heptaprenyl-1,4-naphthoquinone_methyltransferase_[Bacillus_cereus]	CPTF_Ni	113904.7667	126891.4948	111.4013913
UIJ67870.1	2-heptaprenyl-1,4-naphthoquinone_methyltransferase_[Bacillus_cereus]	CPTF_U	11762.4	20373.07442	173.2050808
UIJ67870.1	2-heptaprenyl-1,4-naphthoquinone_methyltransferase_[Bacillus_cereus]	CPTF_metals_mix	220022	47389.4344	21.53849815
UIJ67870.1	2-heptaprenyl-1,4-naphthoquinone_methyltransferase_[Bacillus_cereus]	CPTF_zcontrol	30589.43333	52982.45271	173.2050808
UIJ67872.1	nucleoside-diphosphate_kinase_[Bacillus_cereus]	CPTF_Al	12880150	789450.2377	6.129200652
UIJ67872.1	nucleoside-diphosphate_kinase_[Bacillus_cereus]	CPTF_Cd	12742813.67	379061.8063	2.974710423
UIJ67872.1	nucleoside-diphosphate_kinase_[Bacillus_cereus]	CPTF_Co	15492842.33	480080.4559	3.098724208
UIJ67872.1	nucleoside-diphosphate_kinase_[Bacillus_cereus]	CPTF_Cu	11969233.33	573642.9662	4.792645863
UIJ67872.1	nucleoside-diphosphate_kinase_[Bacillus_cereus]	CPTF_Fe	12648597.67	668214.3919	5.282912853
UIJ67872.1	nucleoside-diphosphate_kinase_[Bacillus_cereus]	CPTF_Mn	12390011	1382883.055	11.16127383
UIJ67872.1	nucleoside-diphosphate_kinase_[Bacillus_cereus]	CPTF_Ni	10609794.33	1028086.154	9.689972511
UIJ67872.1	nucleoside-diphosphate_kinase_[Bacillus_cereus]	CPTF_U	11520820	520268.1838	4.515895429
UIJ67872.1	nucleoside-diphosphate_kinase_[Bacillus_cereus]	CPTF_metals_mix	14159494.4	357935.0041	2.527879838
UIJ67872.1	nucleoside-diphosphate_kinase_[Bacillus_cereus]	CPTF_zcontrol	12375809	364491.2806	2.945191548
UIJ67873.1	chorismate_synthase_[Bacillus_cereus]	CPTF_Al	44696.33333	77416.32025	173.2050808
UIJ67873.1	chorismate_synthase_[Bacillus_cereus]	CPTF_Cd	0	0	0
UIJ67873.1	chorismate_synthase_[Bacillus_cereus]	CPTF_Co	48632	84233.09487	173.2050808
UIJ67873.1	chorismate_synthase_[Bacillus_cereus]	CPTF_Cu	145306.3333	127344.1669	87.63841463
UIJ67873.1	chorismate_synthase_[Bacillus_cereus]	CPTF_Fe	62918.66667	108978.3274	173.2050808
UIJ67873.1	chorismate_synthase_[Bacillus_cereus]	CPTF_Mn	0	0	0
UIJ67873.1	chorismate_synthase_[Bacillus_cereus]	CPTF_Ni	72371.33333	125350.8263	173.2050808
UIJ67873.1	chorismate_synthase_[Bacillus_cereus]	CPTF_U	0	0	0
UIJ67873.1	chorismate_synthase_[Bacillus_cereus]	CPTF_metals_mix	191609.6667	41937.42869	21.88690655
UIJ67873.1	chorismate_synthase_[Bacillus_cereus]	CPTF_zcontrol	24246.16667	41995.59256	173.2050808
UIJ67874.1	3-dehydroquinate_synthase_[Bacillus_cereus]	CPTF_Al	0	0	0
UIJ67874.1	3-dehydroquinate_synthase_[Bacillus_cereus]	CPTF_Cd	391548.6667	370171.5825	94.54037621
UIJ67874.1	3-dehydroquinate_synthase_[Bacillus_cereus]	CPTF_Co	0	0	0
UIJ67874.1	3-dehydroquinate_synthase_[Bacillus_cereus]	CPTF_Cu	475717	415084.7744	87.25455983
UIJ67874.1	3-dehydroquinate_synthase_[Bacillus_cereus]	CPTF_Fe	410212	355399.8398	86.63808952
UIJ67874.1	3-dehydroquinate_synthase_[Bacillus_cereus]	CPTF_Mn	276903.3333	243365.1613	87.8881299
UIJ67874.1	3-dehydroquinate_synthase_[Bacillus_cereus]	CPTF_Ni	204541.6667	354276.5589	173.2050808
UIJ67874.1	3-dehydroquinate_synthase_[Bacillus_cereus]	CPTF_U	363173	316899.1216	87.25844751
UIJ67874.1	3-dehydroquinate_synthase_[Bacillus_cereus]	CPTF_metals_mix	838805.4667	262449.5765	31.28849143
UIJ67874.1	3-dehydroquinate_synthase_[Bacillus_cereus]	CPTF_zcontrol	213760.3333	370243.758	173.2050808
UIJ67875.1	histidinol-phosphate_transaminase_[Bacillus_cereus]	CPTF_Al	2797386.567	405706.2684	14.50304628
UIJ67875.1	histidinol-phosphate_transaminase_[Bacillus_cereus]	CPTF_Cd	2917109	136309.2007	4.672749654
UIJ67875.1	histidinol-phosphate_transaminase_[Bacillus_cereus]	CPTF_Co	2471567.3	123280.9398	4.987966131
UIJ67875.1	histidinol-phosphate_transaminase_[Bacillus_cereus]	CPTF_Cu	2433357.1	168476.9083	6.923640936
UIJ67875.1	histidinol-phosphate_transaminase_[Bacillus_cereus]	CPTF_Fe	2742034.3	170206.4393	6.20730526
UIJ67875.1	histidinol-phosphate_transaminase_[Bacillus_cereus]	CPTF_Mn	2276576.667	510020.7452	22.40296813
UIJ67875.1	histidinol-phosphate_transaminase_[Bacillus_cereus]	CPTF_Ni	2583745.133	157197.6213	6.084099368
UIJ67875.1	histidinol-phosphate_transaminase_[Bacillus_cereus]	CPTF_U	2767328.3	116434.8279	4.207481559
UIJ67875.1	histidinol-phosphate_transaminase_[Bacillus_cereus]	CPTF_metals_mix	2681747	524957.9352	19.57522224
UIJ67875.1	histidinol-phosphate_transaminase_[Bacillus_cereus]	CPTF_zcontrol	2681298.467	65970.38012	2.460389283
UIJ67876.1	tetratricopeptide_repeat_protein_[Bacillus_cereus]	CPTF_Al	0	0	0
UIJ67876.1	tetratricopeptide_repeat_protein_[Bacillus_cereus]	CPTF_Cd	0	0	0
UIJ67876.1	tetratricopeptide_repeat_protein_[Bacillus_cereus]	CPTF_Co	0	0	0
UIJ67876.1	tetratricopeptide_repeat_protein_[Bacillus_cereus]	CPTF_Cu	0	0	0
UIJ67876.1	tetratricopeptide_repeat_protein_[Bacillus_cereus]	CPTF_Fe	0	0	0
UIJ67876.1	tetratricopeptide_repeat_protein_[Bacillus_cereus]	CPTF_Mn	0	0	0

UIJ67876.1	tetratricopeptide_repeat_protein_[Bacillus_cereus]	CPTF_Ni	0	0	0
UIJ67876.1	tetratricopeptide_repeat_protein_[Bacillus_cereus]	CPTF_U	0	0	0
UIJ67876.1	tetratricopeptide_repeat_protein_[Bacillus_cereus]	CPTF_metals_mix	17302.7	18939.31541	109.4587284
UIJ67876.1	tetratricopeptide_repeat_protein_[Bacillus_cereus]	CPTF_zcontrol	0	0	0
UIJ67878.1	YpiF_family_protein_[Bacillus_cereus]	CPTF_Al	288087	18937.56951	6.573559207
UIJ67878.1	YpiF_family_protein_[Bacillus_cereus]	CPTF_Cd	230580	199721.0196	86.61680094
UIJ67878.1	YpiF_family_protein_[Bacillus_cereus]	CPTF_Co	186022	161192.7172	86.65250198
UIJ67878.1	YpiF_family_protein_[Bacillus_cereus]	CPTF_Cu	148569.6667	129548.4268	87.19709059
UIJ67878.1	YpiF_family_protein_[Bacillus_cereus]	CPTF_Fe	283635.3333	37141.91781	13.09495449
UIJ67878.1	YpiF_family_protein_[Bacillus_cereus]	CPTF_Mn	310896	11013.71422	3.542571864
UIJ67878.1	YpiF_family_protein_[Bacillus_cereus]	CPTF_Ni	299623.3333	17962.77811	5.995119909
UIJ67878.1	YpiF_family_protein_[Bacillus_cereus]	CPTF_U	310898.3333	31560.91229	10.15152186
UIJ67878.1	YpiF_family_protein_[Bacillus_cereus]	CPTF_metals_mix	66316.33333	114863.2587	173.2050808
UIJ67878.1	YpiF_family_protein_[Bacillus_cereus]	CPTF_zcontrol	290211.3333	17136.53793	5.904847938
UIJ67879.1	menaquinol-cytochrome_c_reductase_iron-sulfur_subunit_[Bacillus_cereus]	CPTF_Al	185398.1333	106819.8938	57.61648832
UIJ67879.1	menaquinol-cytochrome_c_reductase_iron-sulfur_subunit_[Bacillus_cereus]	CPTF_Cd	339111.4667	131099.6949	38.65976464
UIJ67879.1	menaquinol-cytochrome_c_reductase_iron-sulfur_subunit_[Bacillus_cereus]	CPTF_Co	265298	50487.77899	19.03059163
UIJ67879.1	menaquinol-cytochrome_c_reductase_iron-sulfur_subunit_[Bacillus_cereus]	CPTF_Cu	246269.5667	141377.0389	57.40743398
UIJ67879.1	menaquinol-cytochrome_c_reductase_iron-sulfur_subunit_[Bacillus_cereus]	CPTF_Fe	270686.3333	91465.38927	33.79017631
UIJ67879.1	menaquinol-cytochrome_c_reductase_iron-sulfur_subunit_[Bacillus_cereus]	CPTF_Mn	254559.6667	44331.49574	17.41497242
UIJ67879.1	menaquinol-cytochrome_c_reductase_iron-sulfur_subunit_[Bacillus_cereus]	CPTF_Ni	372964	66605.52379	17.85843239
UIJ67879.1	menaquinol-cytochrome_c_reductase_iron-sulfur_subunit_[Bacillus_cereus]	CPTF_U	280616	53811.51026	19.17620886
UIJ67879.1	menaquinol-cytochrome_c_reductase_iron-sulfur_subunit_[Bacillus_cereus]	CPTF_metals_mix	492401.6667	84345.38259	17.12938609
UIJ67879.1	menaquinol-cytochrome_c_reductase_iron-sulfur_subunit_[Bacillus_cereus]	CPTF_zcontrol	267996.3333	38145.94744	14.23375722
UIJ67885.1	zinc_metallopeptidase_[Bacillus_cereus]	CPTF_Al	0	0	0
UIJ67885.1	zinc_metallopeptidase_[Bacillus_cereus]	CPTF_Cd	0	0	0
UIJ67885.1	zinc_metallopeptidase_[Bacillus_cereus]	CPTF_Co	0	0	0
UIJ67885.1	zinc_metallopeptidase_[Bacillus_cereus]	CPTF_Cu	6442.5	11158.73733	173.2050808
UIJ67885.1	zinc_metallopeptidase_[Bacillus_cereus]	CPTF_Fe	0	0	0
UIJ67885.1	zinc_metallopeptidase_[Bacillus_cereus]	CPTF_Mn	0	0	0
UIJ67885.1	zinc_metallopeptidase_[Bacillus_cereus]	CPTF_Ni	0	0	0
UIJ67885.1	zinc_metallopeptidase_[Bacillus_cereus]	CPTF_U	0	0	0
UIJ67885.1	zinc_metallopeptidase_[Bacillus_cereus]	CPTF_metals_mix	27932.73333	11206.21101	40.11856224
UIJ67885.1	zinc_metallopeptidase_[Bacillus_cereus]	CPTF_zcontrol	0	0	0
UIJ67888.1	dihydrodipicolinate_reductase_[Bacillus_cereus]	CPTF_Al	1134399	787.4287269	0.069413736
UIJ67888.1	dihydrodipicolinate_reductase_[Bacillus_cereus]	CPTF_Cd	1207035	121911.2546	10.10005961
UIJ67888.1	dihydrodipicolinate_reductase_[Bacillus_cereus]	CPTF_Co	1042279.633	151752.6464	14.55968643
UIJ67888.1	dihydrodipicolinate_reductase_[Bacillus_cereus]	CPTF_Cu	1283078.067	132489.2302	10.32589003
UIJ67888.1	dihydrodipicolinate_reductase_[Bacillus_cereus]	CPTF_Fe	1092124.667	123248.9522	11.28524572
UIJ67888.1	dihydrodipicolinate_reductase_[Bacillus_cereus]	CPTF_Mn	1031928.233	43261.02458	4.192251281
UIJ67888.1	dihydrodipicolinate_reductase_[Bacillus_cereus]	CPTF_Ni	909330.3333	106297.6427	11.68966203
UIJ67888.1	dihydrodipicolinate_reductase_[Bacillus_cereus]	CPTF_U	1115059	218002.8103	19.55078702
UIJ67888.1	dihydrodipicolinate_reductase_[Bacillus_cereus]	CPTF_metals_mix	2170048.2	141045.03	6.4996266
UIJ67888.1	dihydrodipicolinate_reductase_[Bacillus_cereus]	CPTF_zcontrol	954465.3333	30460.70577	3.191389431
UIJ67889.1	methylglyoxal_synthase_[Bacillus_cereus]	CPTF_Al	0	0	0
UIJ67889.1	methylglyoxal_synthase_[Bacillus_cereus]	CPTF_Cd	0	0	0
UIJ67889.1	methylglyoxal_synthase_[Bacillus_cereus]	CPTF_Co	0	0	0
UIJ67889.1	methylglyoxal_synthase_[Bacillus_cereus]	CPTF_Cu	11887.46667	20589.69624	173.2050808
UIJ67889.1	methylglyoxal_synthase_[Bacillus_cereus]	CPTF_Fe	0	0	0
UIJ67889.1	methylglyoxal_synthase_[Bacillus_cereus]	CPTF_Mn	0	0	0
UIJ67889.1	methylglyoxal_synthase_[Bacillus_cereus]	CPTF_Ni	0	0	0
UIJ67889.1	methylglyoxal_synthase_[Bacillus_cereus]	CPTF_U	0	0	0
UIJ67889.1	methylglyoxal_synthase_[Bacillus_cereus]	CPTF_metals_mix	12572.83333	12812.89361	101.909357
UIJ67889.1	methylglyoxal_synthase_[Bacillus_cereus]	CPTF_zcontrol	0	0	0
UIJ67893.1	biotin--[acetyl-CoA-carboxylase]_ligase_[Bacillus_cereus]	CPTF_Al	72859.96667	20709.9444	28.42431221
UIJ67893.1	biotin--[acetyl-CoA-carboxylase]_ligase_[Bacillus_cereus]	CPTF_Cd	0	0	0
UIJ67893.1	biotin--[acetyl-CoA-carboxylase]_ligase_[Bacillus_cereus]	CPTF_Co	68403.86667	23513.03237	34.37383516
UIJ67893.1	biotin--[acetyl-CoA-carboxylase]_ligase_[Bacillus_cereus]	CPTF_Cu	39642.16667	42347.80086	106.8251421
UIJ67893.1	biotin--[acetyl-CoA-carboxylase]_ligase_[Bacillus_cereus]	CPTF_Fe	50261.2	63484.65187	126.3094631
UIJ67893.1	biotin--[acetyl-CoA-carboxylase]_ligase_[Bacillus_cereus]	CPTF_Mn	23030.2	39889.47651	173.2050808
UIJ67893.1	biotin--[acetyl-CoA-carboxylase]_ligase_[Bacillus_cereus]	CPTF_Ni	68912	12929.07026	18.76171096

UIJ67893.1	biotin--[acetyl-CoA-carboxylase]_ligase_[Bacillus_cereus]	CPTF_U	46913	19763.82096	42.12866574
UIJ67893.1	biotin--[acetyl-CoA-carboxylase]_ligase_[Bacillus_cereus]	CPTF_metals_mix	26248.13333	45463.10054	173.2050808
UIJ67893.1	biotin--[acetyl-CoA-carboxylase]_ligase_[Bacillus_cereus]	CPTF_zcontrol	0	0	0
UIJ67895.1	3-methyl-2-oxobutanoate_hydroxymethyltransferase_[Bacillus_cereus]	CPTF_Al	0	0	0
UIJ67895.1	3-methyl-2-oxobutanoate_hydroxymethyltransferase_[Bacillus_cereus]	CPTF_Cd	0	0	0
UIJ67895.1	3-methyl-2-oxobutanoate_hydroxymethyltransferase_[Bacillus_cereus]	CPTF_Co	72219.66667	62571.91073	86.641096
UIJ67895.1	3-methyl-2-oxobutanoate_hydroxymethyltransferase_[Bacillus_cereus]	CPTF_Cu	19433.2	33659.28975	173.2050808
UIJ67895.1	3-methyl-2-oxobutanoate_hydroxymethyltransferase_[Bacillus_cereus]	CPTF_Fe	0	0	0
UIJ67895.1	3-methyl-2-oxobutanoate_hydroxymethyltransferase_[Bacillus_cereus]	CPTF_Mn	34818.33333	60307.12237	173.2050808
UIJ67895.1	3-methyl-2-oxobutanoate_hydroxymethyltransferase_[Bacillus_cereus]	CPTF_Ni	0	0	0
UIJ67895.1	3-methyl-2-oxobutanoate_hydroxymethyltransferase_[Bacillus_cereus]	CPTF_U	0	0	0
UIJ67895.1	3-methyl-2-oxobutanoate_hydroxymethyltransferase_[Bacillus_cereus]	CPTF_metals_mix	56886.46667	26806.24794	47.1223641
UIJ67895.1	3-methyl-2-oxobutanoate_hydroxymethyltransferase_[Bacillus_cereus]	CPTF_zcontrol	0	0	0
UIJ67896.1	pantoate--beta-alanine_ligase_[Bacillus_cereus]	CPTF_Al	137112.6333	118963.2972	86.76319193
UIJ67896.1	pantoate--beta-alanine_ligase_[Bacillus_cereus]	CPTF_Cd	95020.83333	39891.98185	41.98235319
UIJ67896.1	pantoate--beta-alanine_ligase_[Bacillus_cereus]	CPTF_Co	66484.93333	63694.71838	95.8032372
UIJ67896.1	pantoate--beta-alanine_ligase_[Bacillus_cereus]	CPTF_Cu	49104.1	42698.9646	86.95600694
UIJ67896.1	pantoate--beta-alanine_ligase_[Bacillus_cereus]	CPTF_Fe	74596.46667	52174.49053	69.94230808
UIJ67896.1	pantoate--beta-alanine_ligase_[Bacillus_cereus]	CPTF_Mn	40331.76667	35789.06838	88.73667418
UIJ67896.1	pantoate--beta-alanine_ligase_[Bacillus_cereus]	CPTF_Ni	82574.66667	71575.96414	86.68029437
UIJ67896.1	pantoate--beta-alanine_ligase_[Bacillus_cereus]	CPTF_U	98856.33333	171224.192	173.2050808
UIJ67896.1	pantoate--beta-alanine_ligase_[Bacillus_cereus]	CPTF_metals_mix	19832.7	34351.24405	173.2050808
UIJ67896.1	pantoate--beta-alanine_ligase_[Bacillus_cereus]	CPTF_zcontrol	36335	62934.06609	173.2050808
UIJ67899.1	YpmA_family_protein_[Bacillus_cereus]	CPTF_Al	106037.5333	130035.5571	122.6316315
UIJ67899.1	YpmA_family_protein_[Bacillus_cereus]	CPTF_Cd	135978.3333	118223.2236	86.94269208
UIJ67899.1	YpmA_family_protein_[Bacillus_cereus]	CPTF_Co	95193.33333	164879.6899	173.2050808
UIJ67899.1	YpmA_family_protein_[Bacillus_cereus]	CPTF_Cu	160783	140546.5173	87.41379208
UIJ67899.1	YpmA_family_protein_[Bacillus_cereus]	CPTF_Fe	16217.8	28090.05359	173.2050808
UIJ67899.1	YpmA_family_protein_[Bacillus_cereus]	CPTF_Mn	80577.33333	69642.01921	86.4287962
UIJ67899.1	YpmA_family_protein_[Bacillus_cereus]	CPTF_Ni	208602	69972.99546	33.54377976
UIJ67899.1	YpmA_family_protein_[Bacillus_cereus]	CPTF_U	76726.33333	132893.9076	173.2050808
UIJ67899.1	YpmA_family_protein_[Bacillus_cereus]	CPTF_metals_mix	120602.3333	104485.7602	86.6365992
UIJ67899.1	YpmA_family_protein_[Bacillus_cereus]	CPTF_zcontrol	93755.66667	83360.38496	88.9123697
UIJ67901.1	aspartate_transaminase_AspB_[Bacillus_cereus]	CPTF_Al	795098.4333	100380.4905	12.62491363
UIJ67901.1	aspartate_transaminase_AspB_[Bacillus_cereus]	CPTF_Cd	811105	70377.4593	8.676738437
UIJ67901.1	aspartate_transaminase_AspB_[Bacillus_cereus]	CPTF_Co	904111.6333	192666.4003	21.31002337
UIJ67901.1	aspartate_transaminase_AspB_[Bacillus_cereus]	CPTF_Cu	947098.5667	91389.17954	9.649384209
UIJ67901.1	aspartate_transaminase_AspB_[Bacillus_cereus]	CPTF_Fe	672661.6667	156006.5935	23.19243109
UIJ67901.1	aspartate_transaminase_AspB_[Bacillus_cereus]	CPTF_Mn	863614.1667	724794.0667	83.92568055
UIJ67901.1	aspartate_transaminase_AspB_[Bacillus_cereus]	CPTF_Ni	504183.4	226986.6093	45.02064314
UIJ67901.1	aspartate_transaminase_AspB_[Bacillus_cereus]	CPTF_U	410080.7333	42599.5136	10.38807975
UIJ67901.1	aspartate_transaminase_AspB_[Bacillus_cereus]	CPTF_metals_mix	3630320.667	346754.1619	9.551612481
UIJ67901.1	aspartate_transaminase_AspB_[Bacillus_cereus]	CPTF_zcontrol	615831.2667	27341.21169	4.43972451
UIJ67905.1	PBP1A_family_penicillin-binding_protein_[Bacillus_cereus]	CPTF_Al	44743.33333	77497.72663	173.2050808
UIJ67905.1	PBP1A_family_penicillin-binding_protein_[Bacillus_cereus]	CPTF_Cd	55418	95986.79165	173.2050808
UIJ67905.1	PBP1A_family_penicillin-binding_protein_[Bacillus_cereus]	CPTF_Co	122244.5667	81996.44324	67.07573635
UIJ67905.1	PBP1A_family_penicillin-binding_protein_[Bacillus_cereus]	CPTF_Cu	112641.7667	56893.41236	50.50827419
UIJ67905.1	PBP1A_family_penicillin-binding_protein_[Bacillus_cereus]	CPTF_Fe	98920.33333	91396.81948	92.3943707
UIJ67905.1	PBP1A_family_penicillin-binding_protein_[Bacillus_cereus]	CPTF_Mn	112267.3333	109901.3114	97.89251078
UIJ67905.1	PBP1A_family_penicillin-binding_protein_[Bacillus_cereus]	CPTF_Ni	180916.3333	160242.0164	88.57244308
UIJ67905.1	PBP1A_family_penicillin-binding_protein_[Bacillus_cereus]	CPTF_U	99055.2	171568.6392	173.2050808
UIJ67905.1	PBP1A_family_penicillin-binding_protein_[Bacillus_cereus]	CPTF_metals_mix	39141.83333	36726.6639	93.82969772
UIJ67905.1	PBP1A_family_penicillin-binding_protein_[Bacillus_cereus]	CPTF_zcontrol	51720.33333	89582.24512	173.2050808
UIJ67914.1	cell_division_regulator_GpsB_[Bacillus_cereus]	CPTF_Al	357661.5	320113.0039	89.50166677
UIJ67914.1	cell_division_regulator_GpsB_[Bacillus_cereus]	CPTF_Cd	561845.2333	168205.2219	29.93799928
UIJ67914.1	cell_division_regulator_GpsB_[Bacillus_cereus]	CPTF_Co	159887.7333	104950.8447	65.64033557
UIJ67914.1	cell_division_regulator_GpsB_[Bacillus_cereus]	CPTF_Cu	175594.6333	99822.34434	56.84817494
UIJ67914.1	cell_division_regulator_GpsB_[Bacillus_cereus]	CPTF_Fe	213000.4333	156070.185	73.27223824
UIJ67914.1	cell_division_regulator_GpsB_[Bacillus_cereus]	CPTF_Mn	378631.6667	347175.4537	91.69213362
UIJ67914.1	cell_division_regulator_GpsB_[Bacillus_cereus]	CPTF_Ni	0	0	0
UIJ67914.1	cell_division_regulator_GpsB_[Bacillus_cereus]	CPTF_U	76815	133047.4828	173.2050808

UIJ67914.1	cell_division_regulator_GpsB_[Bacillus_cereus]	CPTF_metals_mix	869724.5	23093.10359	2.655220542
UIJ67914.1	cell_division_regulator_GpsB_[Bacillus_cereus]	CPTF_zcontrol	162182.6667	117490.2342	72.44315103
UIJ67915.1	class_I_SAM-dependent_RNA_methyltransferase_[Bacillus_cereus]	CPTF_Al	954465.7	154077.9172	16.14284486
UIJ67915.1	class_I_SAM-dependent_RNA_methyltransferase_[Bacillus_cereus]	CPTF_Cd	1177886.2	128484.6497	10.90806987
UIJ67915.1	class_I_SAM-dependent_RNA_methyltransferase_[Bacillus_cereus]	CPTF_Co	809235.4667	59045.79533	7.296491289
UIJ67915.1	class_I_SAM-dependent_RNA_methyltransferase_[Bacillus_cereus]	CPTF_Cu	551377.6667	173900.4858	31.53926906
UIJ67915.1	class_I_SAM-dependent_RNA_methyltransferase_[Bacillus_cereus]	CPTF_Fe	1159071.6	378713.3932	32.67385666
UIJ67915.1	class_I_SAM-dependent_RNA_methyltransferase_[Bacillus_cereus]	CPTF_Mn	882734.8667	137305.2036	15.55452365
UIJ67915.1	class_I_SAM-dependent_RNA_methyltransferase_[Bacillus_cereus]	CPTF_Ni	231065.1	275359.405	119.1696214
UIJ67915.1	class_I_SAM-dependent_RNA_methyltransferase_[Bacillus_cereus]	CPTF_U	74968.43333	39350.54339	52.48948343
UIJ67915.1	class_I_SAM-dependent_RNA_methyltransferase_[Bacillus_cereus]	CPTF_metals_mix	520320.5667	103827.7429	19.95457215
UIJ67915.1	class_I_SAM-dependent_RNA_methyltransferase_[Bacillus_cereus]	CPTF_zcontrol	749327.8	254035.5525	33.90179205
UIJ67917.1	ATP-dependent_DNA_helicase_[Bacillus_cereus]	CPTF_Al	263841.4	179556.6592	68.05477048
UIJ67917.1	ATP-dependent_DNA_helicase_[Bacillus_cereus]	CPTF_Cd	264153.2333	84437.17403	31.96522449
UIJ67917.1	ATP-dependent_DNA_helicase_[Bacillus_cereus]	CPTF_Co	258620.7	211390.7663	81.73775968
UIJ67917.1	ATP-dependent_DNA_helicase_[Bacillus_cereus]	CPTF_Cu	198346.3667	180761.0699	91.13404642
UIJ67917.1	ATP-dependent_DNA_helicase_[Bacillus_cereus]	CPTF_Fe	264906.4	68942.63918	26.02528258
UIJ67917.1	ATP-dependent_DNA_helicase_[Bacillus_cereus]	CPTF_Mn	181433.2	201998.9322	111.3351538
UIJ67917.1	ATP-dependent_DNA_helicase_[Bacillus_cereus]	CPTF_Ni	257140.2667	177923.686	69.19324161
UIJ67917.1	ATP-dependent_DNA_helicase_[Bacillus_cereus]	CPTF_U	407029.3333	145932.4968	35.85306632
UIJ67917.1	ATP-dependent_DNA_helicase_[Bacillus_cereus]	CPTF_metals_mix	77463.1	134170.0249	173.2050808
UIJ67917.1	ATP-dependent_DNA_helicase_[Bacillus_cereus]	CPTF_zcontrol	377675.3333	72696.70726	19.24846577
UIJ67918.1	carboxypeptidase_[Bacillus_cereus]	CPTF_Al	1021518.933	291168.914	28.50352592
UIJ67918.1	carboxypeptidase_[Bacillus_cereus]	CPTF_Cd	1150135.9	158834.8689	13.81009574
UIJ67918.1	carboxypeptidase_[Bacillus_cereus]	CPTF_Co	1102662.167	50165.3748	4.549478192
UIJ67918.1	carboxypeptidase_[Bacillus_cereus]	CPTF_Cu	944631.8	196917.6759	20.84597151
UIJ67918.1	carboxypeptidase_[Bacillus_cereus]	CPTF_Fe	954032.2667	216765.6689	22.72099975
UIJ67918.1	carboxypeptidase_[Bacillus_cereus]	CPTF_Mn	748019.2667	155782.4934	20.82600012
UIJ67918.1	carboxypeptidase_[Bacillus_cereus]	CPTF_Ni	334793.4	278049.3874	83.05103606
UIJ67918.1	carboxypeptidase_[Bacillus_cereus]	CPTF_U	345191.2667	325413.2787	94.2704263
UIJ67918.1	carboxypeptidase_[Bacillus_cereus]	CPTF_metals_mix	1331293.967	352361.6262	26.46760482
UIJ67918.1	carboxypeptidase_[Bacillus_cereus]	CPTF_zcontrol	593488.1667	123127.8885	20.74647742
UIJ67919.1	GNAT_family_N-acetyltransferase_[Bacillus_cereus]	CPTF_Al	48227.1	45322.32256	93.97687723
UIJ67919.1	GNAT_family_N-acetyltransferase_[Bacillus_cereus]	CPTF_Cd	16978.26667	29407.22049	173.2050808
UIJ67919.1	GNAT_family_N-acetyltransferase_[Bacillus_cereus]	CPTF_Co	51556.2	12913.87435	25.04815008
UIJ67919.1	GNAT_family_N-acetyltransferase_[Bacillus_cereus]	CPTF_Cu	54066.56667	20837.85052	38.54110184
UIJ67919.1	GNAT_family_N-acetyltransferase_[Bacillus_cereus]	CPTF_Fe	20326.93333	35207.2813	173.2050808
UIJ67919.1	GNAT_family_N-acetyltransferase_[Bacillus_cereus]	CPTF_Mn	33174.73333	28787.75717	86.77615244
UIJ67919.1	GNAT_family_N-acetyltransferase_[Bacillus_cereus]	CPTF_Ni	52548.5	12449.21503	23.69090464
UIJ67919.1	GNAT_family_N-acetyltransferase_[Bacillus_cereus]	CPTF_U	16839.5	29166.86957	173.2050808
UIJ67919.1	GNAT_family_N-acetyltransferase_[Bacillus_cereus]	CPTF_metals_mix	0	0	0
UIJ67919.1	GNAT_family_N-acetyltransferase_[Bacillus_cereus]	CPTF_zcontrol	65682.46667	19099.60936	29.07870293
UIJ67921.1	xanthine_phosphoribosyltransferase_[Bacillus_cereus]	CPTF_Al	220221.0333	72604.18027	32.96877649
UIJ67921.1	xanthine_phosphoribosyltransferase_[Bacillus_cereus]	CPTF_Cd	160816.7333	56790.60839	35.31386766
UIJ67921.1	xanthine_phosphoribosyltransferase_[Bacillus_cereus]	CPTF_Co	241467.6333	36239.64156	15.00807419
UIJ67921.1	xanthine_phosphoribosyltransferase_[Bacillus_cereus]	CPTF_Cu	171603.8333	73835.54755	43.0267472
UIJ67921.1	xanthine_phosphoribosyltransferase_[Bacillus_cereus]	CPTF_Fe	123187	14508.8942	11.77794264
UIJ67921.1	xanthine_phosphoribosyltransferase_[Bacillus_cereus]	CPTF_Mn	135758.2	32357.30877	23.83451517
UIJ67921.1	xanthine_phosphoribosyltransferase_[Bacillus_cereus]	CPTF_Ni	29865.63333	51728.79433	173.2050808
UIJ67921.1	xanthine_phosphoribosyltransferase_[Bacillus_cereus]	CPTF_U	33840	58612.59933	173.2050808
UIJ67921.1	xanthine_phosphoribosyltransferase_[Bacillus_cereus]	CPTF_metals_mix	135301.4	101491.1733	75.01117748
UIJ67921.1	xanthine_phosphoribosyltransferase_[Bacillus_cereus]	CPTF_zcontrol	199634.2	127745.9898	63.99003269
UIJ67923.1	dynamin_family_protein_[Bacillus_cereus]	CPTF_Al	89964.66667	82713.45066	91.939929
UIJ67923.1	dynamin_family_protein_[Bacillus_cereus]	CPTF_Cd	81595.33333	73740.2936	90.37317526
UIJ67923.1	dynamin_family_protein_[Bacillus_cereus]	CPTF_Co	122722	107276.3255	87.41409489
UIJ67923.1	dynamin_family_protein_[Bacillus_cereus]	CPTF_Cu	120640.6667	108849.7054	90.2263792
UIJ67923.1	dynamin_family_protein_[Bacillus_cereus]	CPTF_Fe	44343.66667	76805.48366	173.2050808
UIJ67923.1	dynamin_family_protein_[Bacillus_cereus]	CPTF_Mn	0	0	0
UIJ67923.1	dynamin_family_protein_[Bacillus_cereus]	CPTF_Ni	55097.33333	95431.38069	173.2050808
UIJ67923.1	dynamin_family_protein_[Bacillus_cereus]	CPTF_U	44927.33333	77816.42398	173.2050808
UIJ67923.1	dynamin_family_protein_[Bacillus_cereus]	CPTF_metals_mix	50593.66667	87630.80121	173.2050808

UIJ67923.1	dynamin_family_protein_[Bacillus_cereus]	CPTF_zcontrol	119229.6333	36100.58217	30.27819608
UIJ67925.1	GNAT_family_N-acetyltransferase_[Bacillus_cereus]	CPTF_Al	115990.3333	100754.0792	86.86420353
UIJ67925.1	GNAT_family_N-acetyltransferase_[Bacillus_cereus]	CPTF_Cd	39150.66667	67810.94382	173.2050808
UIJ67925.1	GNAT_family_N-acetyltransferase_[Bacillus_cereus]	CPTF_Co	152327	7051.780839	4.629370262
UIJ67925.1	GNAT_family_N-acetyltransferase_[Bacillus_cereus]	CPTF_Cu	172303.3333	34399.33612	19.96440548
UIJ67925.1	GNAT_family_N-acetyltransferase_[Bacillus_cereus]	CPTF_Fe	64317	111400.3118	173.2050808
UIJ67925.1	GNAT_family_N-acetyltransferase_[Bacillus_cereus]	CPTF_Mn	56180.3	65543.27409	116.6659382
UIJ67925.1	GNAT_family_N-acetyltransferase_[Bacillus_cereus]	CPTF_Ni	47670.66667	82568.0167	173.2050808
UIJ67925.1	GNAT_family_N-acetyltransferase_[Bacillus_cereus]	CPTF_U	117542.6333	43347.24163	36.87788882
UIJ67925.1	GNAT_family_N-acetyltransferase_[Bacillus_cereus]	CPTF_metals_mix	17863.06667	30939.73905	173.2050808
UIJ67925.1	GNAT_family_N-acetyltransferase_[Bacillus_cereus]	CPTF_zcontrol	108989.0667	15926.7912	14.61320083
UIJ67926.1	YpbS_family_protein_[Bacillus_cereus]	CPTF_Al	281215.0333	8245.682466	2.932162754
UIJ67926.1	YpbS_family_protein_[Bacillus_cereus]	CPTF_Cd	233436.5333	47457.39627	20.32989249
UIJ67926.1	YpbS_family_protein_[Bacillus_cereus]	CPTF_Co	297591.7	41640.60657	13.99252955
UIJ67926.1	YpbS_family_protein_[Bacillus_cereus]	CPTF_Cu	244580.0333	25173.30723	10.2924621
UIJ67926.1	YpbS_family_protein_[Bacillus_cereus]	CPTF_Fe	280605.2667	39652.47826	14.13105275
UIJ67926.1	YpbS_family_protein_[Bacillus_cereus]	CPTF_Mn	161461.9	82083.99443	50.8379961
UIJ67926.1	YpbS_family_protein_[Bacillus_cereus]	CPTF_Ni	267444.3333	46156.15646	17.25822936
UIJ67926.1	YpbS_family_protein_[Bacillus_cereus]	CPTF_U	66438	115073.9916	173.2050808
UIJ67926.1	YpbS_family_protein_[Bacillus_cereus]	CPTF_metals_mix	218532.5333	17603.44877	8.055298908
UIJ67926.1	YpbS_family_protein_[Bacillus_cereus]	CPTF_zcontrol	209981.9	106138.9962	50.54673578
UIJ67927.1	sulfurtransferase_[Bacillus_cereus]	CPTF_Al	2032376	318151.8297	15.65418159
UIJ67927.1	sulfurtransferase_[Bacillus_cereus]	CPTF_Cd	2044784.4	158832.5478	7.767691685
UIJ67927.1	sulfurtransferase_[Bacillus_cereus]	CPTF_Co	2273567.667	92884.07966	4.085388837
UIJ67927.1	sulfurtransferase_[Bacillus_cereus]	CPTF_Cu	2175359	223142.7595	10.2577441
UIJ67927.1	sulfurtransferase_[Bacillus_cereus]	CPTF_Fe	2120031.1	121036.5467	5.709187317
UIJ67927.1	sulfurtransferase_[Bacillus_cereus]	CPTF_Mn	1605501.333	462449.0001	28.80402467
UIJ67927.1	sulfurtransferase_[Bacillus_cereus]	CPTF_Ni	1667897.333	473391.7743	28.38254878
UIJ67927.1	sulfurtransferase_[Bacillus_cereus]	CPTF_U	1972024.067	205139.6509	10.40249226
UIJ67927.1	sulfurtransferase_[Bacillus_cereus]	CPTF_metals_mix	2098491.733	72559.37606	3.457691775
UIJ67927.1	sulfurtransferase_[Bacillus_cereus]	CPTF_zcontrol	1958385.7	13177.43631	0.672872372
UIJ67945.1	cysteine_hydrolase_[Bacillus_cereus]	CPTF_Al	387640.0667	23863.83086	6.156182735
UIJ67945.1	cysteine_hydrolase_[Bacillus_cereus]	CPTF_Cd	270239.8	187320.5196	69.316407
UIJ67945.1	cysteine_hydrolase_[Bacillus_cereus]	CPTF_Co	329975.6667	120336.8046	36.46838745
UIJ67945.1	cysteine_hydrolase_[Bacillus_cereus]	CPTF_Cu	117222	203034.4598	173.2050808
UIJ67945.1	cysteine_hydrolase_[Bacillus_cereus]	CPTF_Fe	226063	208103.347	92.0554655
UIJ67945.1	cysteine_hydrolase_[Bacillus_cereus]	CPTF_Mn	438597.6667	33348.24555	7.603379609
UIJ67945.1	cysteine_hydrolase_[Bacillus_cereus]	CPTF_Ni	142374	246599.0017	173.2050808
UIJ67945.1	cysteine_hydrolase_[Bacillus_cereus]	CPTF_U	422058	63255.95113	14.98750198
UIJ67945.1	cysteine_hydrolase_[Bacillus_cereus]	CPTF_metals_mix	51472.36667	18931.76514	36.78044428
UIJ67945.1	cysteine_hydrolase_[Bacillus_cereus]	CPTF_zcontrol	343757.8333	300599.0503	87.44500377
UIJ67949.1	aldose_1-epimerase_family_protein_[Bacillus_cereus]	CPTF_Al	90761.23333	32140.56553	35.41221769
UIJ67949.1	aldose_1-epimerase_family_protein_[Bacillus_cereus]	CPTF_Cd	81423.13333	86919.25688	106.7500762
UIJ67949.1	aldose_1-epimerase_family_protein_[Bacillus_cereus]	CPTF_Co	100377.0667	21728.61435	21.64699077
UIJ67949.1	aldose_1-epimerase_family_protein_[Bacillus_cereus]	CPTF_Cu	123444.4667	76484.97567	61.95901504
UIJ67949.1	aldose_1-epimerase_family_protein_[Bacillus_cereus]	CPTF_Fe	156557.8	46631.05391	29.78520004
UIJ67949.1	aldose_1-epimerase_family_protein_[Bacillus_cereus]	CPTF_Mn	51930	45505.11623	87.62779941
UIJ67949.1	aldose_1-epimerase_family_protein_[Bacillus_cereus]	CPTF_Ni	147516	51919.17609	35.19562358
UIJ67949.1	aldose_1-epimerase_family_protein_[Bacillus_cereus]	CPTF_U	59652.96667	103321.9691	173.2050808
UIJ67949.1	aldose_1-epimerase_family_protein_[Bacillus_cereus]	CPTF_metals_mix	124289.6	33602.97924	27.03603459
UIJ67949.1	aldose_1-epimerase_family_protein_[Bacillus_cereus]	CPTF_zcontrol	82546.86667	12278.14277	14.87414758
UIJ67954.1	cold_shock-like_protein_CspB_[Bacillus_cereus]	CPTF_Al	158315.3333	274210.201	173.2050808
UIJ67954.1	cold_shock-like_protein_CspB_[Bacillus_cereus]	CPTF_Cd	467841.3333	568111.0275	121.4324146
UIJ67954.1	cold_shock-like_protein_CspB_[Bacillus_cereus]	CPTF_Co	885375.6667	160199.5618	18.09396484
UIJ67954.1	cold_shock-like_protein_CspB_[Bacillus_cereus]	CPTF_Cu	204896.3333	354890.8596	173.2050808
UIJ67954.1	cold_shock-like_protein_CspB_[Bacillus_cereus]	CPTF_Fe	458192.3333	410465.7791	89.5837292
UIJ67954.1	cold_shock-like_protein_CspB_[Bacillus_cereus]	CPTF_Mn	151404	262239.4205	173.2050808
UIJ67954.1	cold_shock-like_protein_CspB_[Bacillus_cereus]	CPTF_Ni	287691	498295.4289	173.2050808
UIJ67954.1	cold_shock-like_protein_CspB_[Bacillus_cereus]	CPTF_U	197306.6667	191628.8472	97.12233777
UIJ67954.1	cold_shock-like_protein_CspB_[Bacillus_cereus]	CPTF_metals_mix	843605	23509.4853	2.786788283
UIJ67954.1	cold_shock-like_protein_CspB_[Bacillus_cereus]	CPTF_zcontrol	0	0	0

UIJ67967.1	oxygen-insensitive_NADPH_nitroreductase_[Bacillus_cereus]	CPTF_Al	68444.3	36440.6044	53.24125515
UIJ67967.1	oxygen-insensitive_NADPH_nitroreductase_[Bacillus_cereus]	CPTF_Cd	68216.6	60436.2513	88.59464016
UIJ67967.1	oxygen-insensitive_NADPH_nitroreductase_[Bacillus_cereus]	CPTF_Co	34919.36667	30577.86536	87.56706743
UIJ67967.1	oxygen-insensitive_NADPH_nitroreductase_[Bacillus_cereus]	CPTF_Cu	77140.93333	28271.44383	36.64908189
UIJ67967.1	oxygen-insensitive_NADPH_nitroreductase_[Bacillus_cereus]	CPTF_Fe	54570.73333	55350.83139	101.4295173
UIJ67967.1	oxygen-insensitive_NADPH_nitroreductase_[Bacillus_cereus]	CPTF_Mn	33842.33333	58616.64078	173.2050808
UIJ67967.1	oxygen-insensitive_NADPH_nitroreductase_[Bacillus_cereus]	CPTF_Ni	62573.26667	22573.31258	36.0750106
UIJ67967.1	oxygen-insensitive_NADPH_nitroreductase_[Bacillus_cereus]	CPTF_U	29606.06667	51279.21168	173.2050808
UIJ67967.1	oxygen-insensitive_NADPH_nitroreductase_[Bacillus_cereus]	CPTF_metals_mix	146202.0333	40620.44931	27.78377864
UIJ67967.1	oxygen-insensitive_NADPH_nitroreductase_[Bacillus_cereus]	CPTF_zcontrol	46280.46667	48178.03752	104.100155
UIJ67975.1	response_regulator_[Bacillus_cereus]	CPTF_Al	37891.2	35661.99984	94.11683936
UIJ67975.1	response_regulator_[Bacillus_cereus]	CPTF_Cd	0	0	0
UIJ67975.1	response_regulator_[Bacillus_cereus]	CPTF_Co	0	0	0
UIJ67975.1	response_regulator_[Bacillus_cereus]	CPTF_Cu	21452.3	37156.47354	173.2050808
UIJ67975.1	response_regulator_[Bacillus_cereus]	CPTF_Fe	20279.7	35125.47076	173.2050808
UIJ67975.1	response_regulator_[Bacillus_cereus]	CPTF_Mn	28928.76667	50106.09367	173.2050808
UIJ67975.1	response_regulator_[Bacillus_cereus]	CPTF_Ni	18161.96667	31457.44903	173.2050808
UIJ67975.1	response_regulator_[Bacillus_cereus]	CPTF_U	25937.43333	44924.95235	173.2050808
UIJ67975.1	response_regulator_[Bacillus_cereus]	CPTF_metals_mix	169938.2333	137789.4806	81.08209549
UIJ67975.1	response_regulator_[Bacillus_cereus]	CPTF_zcontrol	0	0	0
UIJ67978.1	transglycosylase_[Bacillus_cereus]	CPTF_Al	0	0	0
UIJ67978.1	transglycosylase_[Bacillus_cereus]	CPTF_Cd	0	0	0
UIJ67978.1	transglycosylase_[Bacillus_cereus]	CPTF_Co	0	0	0
UIJ67978.1	transglycosylase_[Bacillus_cereus]	CPTF_Cu	0	0	0
UIJ67978.1	transglycosylase_[Bacillus_cereus]	CPTF_Fe	0	0	0
UIJ67978.1	transglycosylase_[Bacillus_cereus]	CPTF_Mn	280574	485968.4233	173.2050808
UIJ67978.1	transglycosylase_[Bacillus_cereus]	CPTF_Ni	0	0	0
UIJ67978.1	transglycosylase_[Bacillus_cereus]	CPTF_U	0	0	0
UIJ67978.1	transglycosylase_[Bacillus_cereus]	CPTF_metals_mix	3010000	288444.102	9.582860533
UIJ67978.1	transglycosylase_[Bacillus_cereus]	CPTF_zcontrol	0	0	0
UIJ68002.1	chemotaxis_protein_[Bacillus_cereus]	CPTF_Al	0	0	0
UIJ68002.1	chemotaxis_protein_[Bacillus_cereus]	CPTF_Cd	0	0	0
UIJ68002.1	chemotaxis_protein_[Bacillus_cereus]	CPTF_Co	0	0	0
UIJ68002.1	chemotaxis_protein_[Bacillus_cereus]	CPTF_Cu	0	0	0
UIJ68002.1	chemotaxis_protein_[Bacillus_cereus]	CPTF_Fe	0	0	0
UIJ68002.1	chemotaxis_protein_[Bacillus_cereus]	CPTF_Mn	0	0	0
UIJ68002.1	chemotaxis_protein_[Bacillus_cereus]	CPTF_Ni	0	0	0
UIJ68002.1	chemotaxis_protein_[Bacillus_cereus]	CPTF_U	0	0	0
UIJ68002.1	chemotaxis_protein_[Bacillus_cereus]	CPTF_metals_mix	21169.03333	36665.84128	173.2050808
UIJ68002.1	chemotaxis_protein_[Bacillus_cereus]	CPTF_zcontrol	0	0	0
UIJ68046.1	hypothetical_protein_LW858_07360_[Bacillus_cereus]	CPTF_Al	3978061	1708280.967	42.94255334
UIJ68046.1	hypothetical_protein_LW858_07360_[Bacillus_cereus]	CPTF_Cd	5011027.333	273344.4421	5.454858333
UIJ68046.1	hypothetical_protein_LW858_07360_[Bacillus_cereus]	CPTF_Co	3709833	1961188.186	52.86459487
UIJ68046.1	hypothetical_protein_LW858_07360_[Bacillus_cereus]	CPTF_Cu	5338423.333	483837.8074	9.063309092
UIJ68046.1	hypothetical_protein_LW858_07360_[Bacillus_cereus]	CPTF_Fe	3325812	1690535.006	50.83074467
UIJ68046.1	hypothetical_protein_LW858_07360_[Bacillus_cereus]	CPTF_Mn	5261231.333	1596963.492	30.35341711
UIJ68046.1	hypothetical_protein_LW858_07360_[Bacillus_cereus]	CPTF_Ni	4853074.667	438171.8051	9.028746418
UIJ68046.1	hypothetical_protein_LW858_07360_[Bacillus_cereus]	CPTF_U	5411730.333	548675.4318	10.13863216
UIJ68046.1	hypothetical_protein_LW858_07360_[Bacillus_cereus]	CPTF_metals_mix	3434139.233	166839.0902	4.858250609
UIJ68046.1	hypothetical_protein_LW858_07360_[Bacillus_cereus]	CPTF_zcontrol	5498432.667	347233.4183	6.315134501
UIJ68071.1	peptidase_E_[Bacillus_cereus]	CPTF_Al	0	0	0
UIJ68071.1	peptidase_E_[Bacillus_cereus]	CPTF_Cd	14673.23333	25414.78564	173.2050808
UIJ68071.1	peptidase_E_[Bacillus_cereus]	CPTF_Co	11170.73333	19348.27769	173.2050808
UIJ68071.1	peptidase_E_[Bacillus_cereus]	CPTF_Cu	0	0	0
UIJ68071.1	peptidase_E_[Bacillus_cereus]	CPTF_Fe	0	0	0
UIJ68071.1	peptidase_E_[Bacillus_cereus]	CPTF_Mn	0	0	0
UIJ68071.1	peptidase_E_[Bacillus_cereus]	CPTF_Ni	0	0	0
UIJ68071.1	peptidase_E_[Bacillus_cereus]	CPTF_U	0	0	0
UIJ68071.1	peptidase_E_[Bacillus_cereus]	CPTF_metals_mix	60789.43333	15809.00325	26.00616979
UIJ68071.1	peptidase_E_[Bacillus_cereus]	CPTF_zcontrol	0	0	0
UIJ68081.1	DUF445_domain-containing_protein_[Bacillus_cereus]	CPTF_Al	21210.56667	18374.52267	86.62909839

UIJ68081.1	DUF445_domain-containing_protein_[Bacillus_cereus]	CPTF_Cd	33233.96667	5999.18889	18.05137783
UIJ68081.1	DUF445_domain-containing_protein_[Bacillus_cereus]	CPTF_Co	12859.46667	22273.24962	173.2050808
UIJ68081.1	DUF445_domain-containing_protein_[Bacillus_cereus]	CPTF_Cu	23803.83333	20963.83982	88.06917578
UIJ68081.1	DUF445_domain-containing_protein_[Bacillus_cereus]	CPTF_Fe	9976.9	17280.4977	173.2050808
UIJ68081.1	DUF445_domain-containing_protein_[Bacillus_cereus]	CPTF_Mn	0	0	0
UIJ68081.1	DUF445_domain-containing_protein_[Bacillus_cereus]	CPTF_Ni	23996.06667	20950.96796	87.31000898
UIJ68081.1	DUF445_domain-containing_protein_[Bacillus_cereus]	CPTF_U	11627.16667	20138.84341	173.2050808
UIJ68081.1	DUF445_domain-containing_protein_[Bacillus_cereus]	CPTF_metals_mix	37109.36667	19268.59655	51.92380867
UIJ68081.1	DUF445_domain-containing_protein_[Bacillus_cereus]	CPTF_zcontrol	0	0	0
UIJ68095.1	oxaloacetate-decarboxylating_malate_dehydrogenase_[Bacillus_cereus]	CPTF_Al	0	0	0
UIJ68095.1	oxaloacetate-decarboxylating_malate_dehydrogenase_[Bacillus_cereus]	CPTF_Cd	0	0	0
UIJ68095.1	oxaloacetate-decarboxylating_malate_dehydrogenase_[Bacillus_cereus]	CPTF_Co	27109.93333	46955.78192	173.2050808
UIJ68095.1	oxaloacetate-decarboxylating_malate_dehydrogenase_[Bacillus_cereus]	CPTF_Cu	18715.7	32416.5433	173.2050808
UIJ68095.1	oxaloacetate-decarboxylating_malate_dehydrogenase_[Bacillus_cereus]	CPTF_Fe	0	0	0
UIJ68095.1	oxaloacetate-decarboxylating_malate_dehydrogenase_[Bacillus_cereus]	CPTF_Mn	0	0	0
UIJ68095.1	oxaloacetate-decarboxylating_malate_dehydrogenase_[Bacillus_cereus]	CPTF_Ni	0	0	0
UIJ68095.1	oxaloacetate-decarboxylating_malate_dehydrogenase_[Bacillus_cereus]	CPTF_U	0	0	0
UIJ68095.1	oxaloacetate-decarboxylating_malate_dehydrogenase_[Bacillus_cereus]	CPTF_metals_mix	97029.43333	46978.36429	48.41661203
UIJ68095.1	oxaloacetate-decarboxylating_malate_dehydrogenase_[Bacillus_cereus]	CPTF_zcontrol	0	0	0
UIJ68100.1	aspartate--ammonia_ligase_[Bacillus_cereus]	CPTF_Al	6833914.967	537147.512	7.860026274
UIJ68100.1	aspartate--ammonia_ligase_[Bacillus_cereus]	CPTF_Cd	7175124.3	173872.3408	2.423265905
UIJ68100.1	aspartate--ammonia_ligase_[Bacillus_cereus]	CPTF_Co	6813373.867	119426.8551	1.752829912
UIJ68100.1	aspartate--ammonia_ligase_[Bacillus_cereus]	CPTF_Cu	7399815.367	824611.7485	11.14368005
UIJ68100.1	aspartate--ammonia_ligase_[Bacillus_cereus]	CPTF_Fe	6277530.367	474090.5945	7.552183212
UIJ68100.1	aspartate--ammonia_ligase_[Bacillus_cereus]	CPTF_Mn	6532603.8	471263.3907	7.214020705
UIJ68100.1	aspartate--ammonia_ligase_[Bacillus_cereus]	CPTF_Ni	5657835.367	322053.1059	5.692161137
UIJ68100.1	aspartate--ammonia_ligase_[Bacillus_cereus]	CPTF_U	5459837.833	1964690.229	35.9844063
UIJ68100.1	aspartate--ammonia_ligase_[Bacillus_cereus]	CPTF_metals_mix	8704109.533	579734.3497	6.660467076
UIJ68100.1	aspartate--ammonia_ligase_[Bacillus_cereus]	CPTF_zcontrol	6557164.667	468660.8734	7.147309809
UIJ68101.1	VOC_family_protein_[Bacillus_cereus]	CPTF_Al	16144.1	18485.36113	114.502271
UIJ68101.1	VOC_family_protein_[Bacillus_cereus]	CPTF_Cd	17211	15448.53508	89.75966
UIJ68101.1	VOC_family_protein_[Bacillus_cereus]	CPTF_Co	42831.2	37362.82991	87.23274135
UIJ68101.1	VOC_family_protein_[Bacillus_cereus]	CPTF_Cu	69765.93333	17797.43447	25.51020766
UIJ68101.1	VOC_family_protein_[Bacillus_cereus]	CPTF_Fe	33014.86667	13009.81712	39.40593566
UIJ68101.1	VOC_family_protein_[Bacillus_cereus]	CPTF_Mn	0	0	0
UIJ68101.1	VOC_family_protein_[Bacillus_cereus]	CPTF_Ni	44934.7	8996.917963	20.02220547
UIJ68101.1	VOC_family_protein_[Bacillus_cereus]	CPTF_U	0	0	0
UIJ68101.1	VOC_family_protein_[Bacillus_cereus]	CPTF_metals_mix	55604.73333	9813.214132	17.64816328
UIJ68101.1	VOC_family_protein_[Bacillus_cereus]	CPTF_zcontrol	27740.1	25287.92811	91.16019089
UIJ68102.1	aspartate_kinase_[Bacillus_cereus]	CPTF_Al	325260	100441.3715	30.88033312
UIJ68102.1	aspartate_kinase_[Bacillus_cereus]	CPTF_Cd	261245.6667	238957.7671	91.46860507
UIJ68102.1	aspartate_kinase_[Bacillus_cereus]	CPTF_Co	442677	37713.07354	8.519320756
UIJ68102.1	aspartate_kinase_[Bacillus_cereus]	CPTF_Cu	461353.3333	64442.31193	13.96810368
UIJ68102.1	aspartate_kinase_[Bacillus_cereus]	CPTF_Fe	341222	298772.0577	87.5594357
UIJ68102.1	aspartate_kinase_[Bacillus_cereus]	CPTF_Mn	421552	77744.17687	18.44236936
UIJ68102.1	aspartate_kinase_[Bacillus_cereus]	CPTF_Ni	379892.3333	129104.7248	33.98455654
UIJ68102.1	aspartate_kinase_[Bacillus_cereus]	CPTF_U	403857.3333	118453.5406	29.33054097
UIJ68102.1	aspartate_kinase_[Bacillus_cereus]	CPTF_metals_mix	259174.6667	229093.8069	88.39359564
UIJ68102.1	aspartate_kinase_[Bacillus_cereus]	CPTF_zcontrol	310902.3333	269336.3515	86.63053396
UIJ68109.1	UDP-galactose-lipid_carrier_transferase_[Bacillus_cereus]	CPTF_Al	487946.3667	51140.10702	10.480682
UIJ68109.1	UDP-galactose-lipid_carrier_transferase_[Bacillus_cereus]	CPTF_Cd	364832.2	214944.1924	58.91590502
UIJ68109.1	UDP-galactose-lipid_carrier_transferase_[Bacillus_cereus]	CPTF_Co	344360.0333	215398.3055	62.55032079
UIJ68109.1	UDP-galactose-lipid_carrier_transferase_[Bacillus_cereus]	CPTF_Cu	469827.5667	203133.11	43.23567292
UIJ68109.1	UDP-galactose-lipid_carrier_transferase_[Bacillus_cereus]	CPTF_Fe	409021.9667	239111.5704	58.4593469
UIJ68109.1	UDP-galactose-lipid_carrier_transferase_[Bacillus_cereus]	CPTF_Mn	246600.5	285913.1695	115.941845
UIJ68109.1	UDP-galactose-lipid_carrier_transferase_[Bacillus_cereus]	CPTF_Ni	220516.3	51984.74559	23.57410567
UIJ68109.1	UDP-galactose-lipid_carrier_transferase_[Bacillus_cereus]	CPTF_U	93361.96667	56447.49396	60.46090927
UIJ68109.1	UDP-galactose-lipid_carrier_transferase_[Bacillus_cereus]	CPTF_metals_mix	621081.9333	67861.78714	10.92638241
UIJ68109.1	UDP-galactose-lipid_carrier_transferase_[Bacillus_cereus]	CPTF_zcontrol	333936.1667	110306.3908	33.03217856
UIJ68114.1	GTPase_HflX_[Bacillus_cereus]	CPTF_Al	0	0	0
UIJ68114.1	GTPase_HflX_[Bacillus_cereus]	CPTF_Cd	0	0	0

UIJ68114.1	GTPase_HflX_[Bacillus_cereus]	CPTF_Co	5139.033333	8901.066835	173.2050808
UIJ68114.1	GTPase_HflX_[Bacillus_cereus]	CPTF_Cu	18191.5	22362.2268	122.9267889
UIJ68114.1	GTPase_HflX_[Bacillus_cereus]	CPTF_Fe	0	0	0
UIJ68114.1	GTPase_HflX_[Bacillus_cereus]	CPTF_Mn	0	0	0
UIJ68114.1	GTPase_HflX_[Bacillus_cereus]	CPTF_Ni	2197.073333	3805.442641	173.2050808
UIJ68114.1	GTPase_HflX_[Bacillus_cereus]	CPTF_U	0	0	0
UIJ68114.1	GTPase_HflX_[Bacillus_cereus]	CPTF_metals_mix	88355.8	77146.59465	87.31356022
UIJ68114.1	GTPase_HflX_[Bacillus_cereus]	CPTF_zcontrol	0	0	0
UIJ68116.1	cysteine_synthase_A_[Bacillus_cereus]	CPTF_Al	617280.6667	103986.8819	16.84596449
UIJ68116.1	cysteine_synthase_A_[Bacillus_cereus]	CPTF_Cd	794352.5333	186277.8367	23.45027288
UIJ68116.1	cysteine_synthase_A_[Bacillus_cereus]	CPTF_Co	457664.4	103898.5425	22.70190613
UIJ68116.1	cysteine_synthase_A_[Bacillus_cereus]	CPTF_Cu	419172.6667	88281.33099	21.06085106
UIJ68116.1	cysteine_synthase_A_[Bacillus_cereus]	CPTF_Fe	450714.6667	113826.8382	25.25474465
UIJ68116.1	cysteine_synthase_A_[Bacillus_cereus]	CPTF_Mn	508034.6667	147541.2363	29.04156862
UIJ68116.1	cysteine_synthase_A_[Bacillus_cereus]	CPTF_Ni	446565.8	198393.7453	44.42654258
UIJ68116.1	cysteine_synthase_A_[Bacillus_cereus]	CPTF_U	381799	139755.1	36.60436513
UIJ68116.1	cysteine_synthase_A_[Bacillus_cereus]	CPTF_metals_mix	997976.1333	258947.0891	25.94722262
UIJ68116.1	cysteine_synthase_A_[Bacillus_cereus]	CPTF_zcontrol	436450	166665.143	38.18653751
UIJ68153.1	rhodanese-related_sulfurtransferase_[Bacillus_cereus]	CPTF_Al	4373.533333	7575.181942	173.2050808
UIJ68153.1	rhodanese-related_sulfurtransferase_[Bacillus_cereus]	CPTF_Cd	0	0	0
UIJ68153.1	rhodanese-related_sulfurtransferase_[Bacillus_cereus]	CPTF_Co	0	0	0
UIJ68153.1	rhodanese-related_sulfurtransferase_[Bacillus_cereus]	CPTF_Cu	0	0	0
UIJ68153.1	rhodanese-related_sulfurtransferase_[Bacillus_cereus]	CPTF_Fe	0	0	0
UIJ68153.1	rhodanese-related_sulfurtransferase_[Bacillus_cereus]	CPTF_Mn	0	0	0
UIJ68153.1	rhodanese-related_sulfurtransferase_[Bacillus_cereus]	CPTF_Ni	0	0	0
UIJ68153.1	rhodanese-related_sulfurtransferase_[Bacillus_cereus]	CPTF_U	0	0	0
UIJ68153.1	rhodanese-related_sulfurtransferase_[Bacillus_cereus]	CPTF_metals_mix	8228.933333	14252.93063	173.2050808
UIJ68153.1	rhodanese-related_sulfurtransferase_[Bacillus_cereus]	CPTF_zcontrol	0	0	0
UIJ68155.1	2-dehydropantoate_2-reductase_[Bacillus_cereus]	CPTF_Al	0	0	0
UIJ68155.1	2-dehydropantoate_2-reductase_[Bacillus_cereus]	CPTF_Cd	84649	146616.3688	173.2050808
UIJ68155.1	2-dehydropantoate_2-reductase_[Bacillus_cereus]	CPTF_Co	27762.63333	48086.29149	173.2050808
UIJ68155.1	2-dehydropantoate_2-reductase_[Bacillus_cereus]	CPTF_Cu	54313.16667	94073.16419	173.2050808
UIJ68155.1	2-dehydropantoate_2-reductase_[Bacillus_cereus]	CPTF_Fe	18616.9	32245.41668	173.2050808
UIJ68155.1	2-dehydropantoate_2-reductase_[Bacillus_cereus]	CPTF_Mn	60861.86667	56134.53642	92.23268936
UIJ68155.1	2-dehydropantoate_2-reductase_[Bacillus_cereus]	CPTF_Ni	0	0	0
UIJ68155.1	2-dehydropantoate_2-reductase_[Bacillus_cereus]	CPTF_U	0	0	0
UIJ68155.1	2-dehydropantoate_2-reductase_[Bacillus_cereus]	CPTF_metals_mix	99454.46667	71536.32097	71.92871609
UIJ68155.1	2-dehydropantoate_2-reductase_[Bacillus_cereus]	CPTF_zcontrol	17299.4	29963.43974	173.2050808
UIJ68164.1	deoxyribose-phosphate_aldolase_[Bacillus_cereus]	CPTF_Al	420086	143230.7076	34.09556796
UIJ68164.1	deoxyribose-phosphate_aldolase_[Bacillus_cereus]	CPTF_Cd	541039.7667	270665.9432	50.02699614
UIJ68164.1	deoxyribose-phosphate_aldolase_[Bacillus_cereus]	CPTF_Co	443855.8	39573.88065	8.915931851
UIJ68164.1	deoxyribose-phosphate_aldolase_[Bacillus_cereus]	CPTF_Cu	284235.7333	62314.75938	21.92361905
UIJ68164.1	deoxyribose-phosphate_aldolase_[Bacillus_cereus]	CPTF_Fe	446141.7667	160445.2574	35.96284171
UIJ68164.1	deoxyribose-phosphate_aldolase_[Bacillus_cereus]	CPTF_Mn	292574.2	220619.8653	75.40646624
UIJ68164.1	deoxyribose-phosphate_aldolase_[Bacillus_cereus]	CPTF_Ni	86769.4	54884.48423	63.25327158
UIJ68164.1	deoxyribose-phosphate_aldolase_[Bacillus_cereus]	CPTF_U	252908.4333	33145.32955	13.10566402
UIJ68164.1	deoxyribose-phosphate_aldolase_[Bacillus_cereus]	CPTF_metals_mix	401528.4667	226138.961	56.31953391
UIJ68164.1	deoxyribose-phosphate_aldolase_[Bacillus_cereus]	CPTF_zcontrol	320522.3333	204738.8589	63.87662812
UIJ68168.1	aminopeptidase_P_family_protein_[Bacillus_cereus]	CPTF_Al	2101756.6	329928.5642	15.69775321
UIJ68168.1	aminopeptidase_P_family_protein_[Bacillus_cereus]	CPTF_Cd	2094333.367	270524.5528	12.91697669
UIJ68168.1	aminopeptidase_P_family_protein_[Bacillus_cereus]	CPTF_Co	1988400.933	156171.7635	7.854138515
UIJ68168.1	aminopeptidase_P_family_protein_[Bacillus_cereus]	CPTF_Cu	2396635.6	133427.3485	5.56727725
UIJ68168.1	aminopeptidase_P_family_protein_[Bacillus_cereus]	CPTF_Fe	1814600.233	323939.5433	17.85184072
UIJ68168.1	aminopeptidase_P_family_protein_[Bacillus_cereus]	CPTF_Mn	1901694.667	117099.1781	6.157622467
UIJ68168.1	aminopeptidase_P_family_protein_[Bacillus_cereus]	CPTF_Ni	1821610.967	164753.721	9.044396639
UIJ68168.1	aminopeptidase_P_family_protein_[Bacillus_cereus]	CPTF_U	2060564.1	580510.5143	28.17240747
UIJ68168.1	aminopeptidase_P_family_protein_[Bacillus_cereus]	CPTF_metals_mix	3460778.367	250816.2646	7.247394604
UIJ68168.1	aminopeptidase_P_family_protein_[Bacillus_cereus]	CPTF_zcontrol	2080115.233	134255.9221	6.454254069
UIJ68170.1	multicopper_oxidase_family_protein_[Bacillus_cereus]	CPTF_Al	12527863.37	1043822.739	8.332009284
UIJ68170.1	multicopper_oxidase_family_protein_[Bacillus_cereus]	CPTF_Cd	11461488.23	1765964.469	15.40781121
UIJ68170.1	multicopper_oxidase_family_protein_[Bacillus_cereus]	CPTF_Co	11563642.83	355390.0495	3.073339903

UIJ68170.1	multicopper_oxidase_family_protein_[Bacillus_cereus]	CPTF_Cu	17491493.67	1157730.575	6.618820535
UIJ68170.1	multicopper_oxidase_family_protein_[Bacillus_cereus]	CPTF_Fe	12244756.57	2385773.208	19.48403952
UIJ68170.1	multicopper_oxidase_family_protein_[Bacillus_cereus]	CPTF_Mn	14148341.37	2446708.664	17.29325439
UIJ68170.1	multicopper_oxidase_family_protein_[Bacillus_cereus]	CPTF_Ni	10769782.33	611432.3862	5.677295671
UIJ68170.1	multicopper_oxidase_family_protein_[Bacillus_cereus]	CPTF_U	10337357.47	1210143.852	11.70651064
UIJ68170.1	multicopper_oxidase_family_protein_[Bacillus_cereus]	CPTF_metals_mix	22250147.67	532737.2392	2.39430878
UIJ68170.1	multicopper_oxidase_family_protein_[Bacillus_cereus]	CPTF_zcontrol	12359070.63	1284396.394	10.392338
UIJ68172.1	TetR/AcrR_family_transcriptional_regulator_[Bacillus_cereus]	CPTF_Al	0	0	0
UIJ68172.1	TetR/AcrR_family_transcriptional_regulator_[Bacillus_cereus]	CPTF_Cd	0	0	0
UIJ68172.1	TetR/AcrR_family_transcriptional_regulator_[Bacillus_cereus]	CPTF_Co	31092.13333	27317.1773	87.85880661
UIJ68172.1	TetR/AcrR_family_transcriptional_regulator_[Bacillus_cereus]	CPTF_Cu	62737.33333	39152.52264	62.40705583
UIJ68172.1	TetR/AcrR_family_transcriptional_regulator_[Bacillus_cereus]	CPTF_Fe	25593.4	44329.06914	173.2050808
UIJ68172.1	TetR/AcrR_family_transcriptional_regulator_[Bacillus_cereus]	CPTF_Mn	25586.53333	44317.17572	173.2050808
UIJ68172.1	TetR/AcrR_family_transcriptional_regulator_[Bacillus_cereus]	CPTF_Ni	0	0	0
UIJ68172.1	TetR/AcrR_family_transcriptional_regulator_[Bacillus_cereus]	CPTF_U	0	0	0
UIJ68172.1	TetR/AcrR_family_transcriptional_regulator_[Bacillus_cereus]	CPTF_metals_mix	27908.43333	48338.82449	173.2050808
UIJ68172.1	TetR/AcrR_family_transcriptional_regulator_[Bacillus_cereus]	CPTF_zcontrol	0	0	0
UIJ68200.1	DUF4352_domain-containing_protein_[Bacillus_cereus]	CPTF_Al	552541.6333	177580.7325	32.13888725
UIJ68200.1	DUF4352_domain-containing_protein_[Bacillus_cereus]	CPTF_Cd	922893.6667	50679.64469	5.491385034
UIJ68200.1	DUF4352_domain-containing_protein_[Bacillus_cereus]	CPTF_Co	492210	207183.8956	42.09258154
UIJ68200.1	DUF4352_domain-containing_protein_[Bacillus_cereus]	CPTF_Cu	469900.6333	23766.46426	5.057763828
UIJ68200.1	DUF4352_domain-containing_protein_[Bacillus_cereus]	CPTF_Fe	649625.0333	160552.1913	24.71459427
UIJ68200.1	DUF4352_domain-containing_protein_[Bacillus_cereus]	CPTF_Mn	587261.6667	269339.1058	45.86355982
UIJ68200.1	DUF4352_domain-containing_protein_[Bacillus_cereus]	CPTF_Ni	0	0	0
UIJ68200.1	DUF4352_domain-containing_protein_[Bacillus_cereus]	CPTF_U	348139.4667	98114.09503	28.18241091
UIJ68200.1	DUF4352_domain-containing_protein_[Bacillus_cereus]	CPTF_metals_mix	826008	34744.97004	4.206372098
UIJ68200.1	DUF4352_domain-containing_protein_[Bacillus_cereus]	CPTF_zcontrol	452731.6667	170246.1385	37.60420378
UIJ68201.1	MarR_family_transcriptional_regulator_[Bacillus_cereus]	CPTF_Al	46163.1	43228.61923	93.64323287
UIJ68201.1	MarR_family_transcriptional_regulator_[Bacillus_cereus]	CPTF_Cd	82758.83333	44951.37704	54.31610769
UIJ68201.1	MarR_family_transcriptional_regulator_[Bacillus_cereus]	CPTF_Co	30931.13333	28873.32891	93.34714187
UIJ68201.1	MarR_family_transcriptional_regulator_[Bacillus_cereus]	CPTF_Cu	50000.16667	19224.64462	38.44916109
UIJ68201.1	MarR_family_transcriptional_regulator_[Bacillus_cereus]	CPTF_Fe	35931.9	31455.57869	87.54220815
UIJ68201.1	MarR_family_transcriptional_regulator_[Bacillus_cereus]	CPTF_Mn	25626.83333	44386.97737	173.2050808
UIJ68201.1	MarR_family_transcriptional_regulator_[Bacillus_cereus]	CPTF_Ni	0	0	0
UIJ68201.1	MarR_family_transcriptional_regulator_[Bacillus_cereus]	CPTF_U	0	0	0
UIJ68201.1	MarR_family_transcriptional_regulator_[Bacillus_cereus]	CPTF_metals_mix	63490.4	8465.924063	13.33417975
UIJ68201.1	MarR_family_transcriptional_regulator_[Bacillus_cereus]	CPTF_zcontrol	0	0	0
UIJ68212.1	DUF6376_family_protein_[Bacillus_cereus]	CPTF_Al	157018.6667	19066.27091	12.14267788
UIJ68212.1	DUF6376_family_protein_[Bacillus_cereus]	CPTF_Cd	153627	46444.5719	30.23203727
UIJ68212.1	DUF6376_family_protein_[Bacillus_cereus]	CPTF_Co	124289	10065.36005	8.098351466
UIJ68212.1	DUF6376_family_protein_[Bacillus_cereus]	CPTF_Cu	66365.9	61121.90733	92.09836276
UIJ68212.1	DUF6376_family_protein_[Bacillus_cereus]	CPTF_Fe	167583.3333	38809.33105	23.15822837
UIJ68212.1	DUF6376_family_protein_[Bacillus_cereus]	CPTF_Mn	94394.66667	82684.48032	87.5944407
UIJ68212.1	DUF6376_family_protein_[Bacillus_cereus]	CPTF_Ni	66809.5	63107.1167	94.45829815
UIJ68212.1	DUF6376_family_protein_[Bacillus_cereus]	CPTF_U	53671.33333	92961.47624	173.2050808
UIJ68212.1	DUF6376_family_protein_[Bacillus_cereus]	CPTF_metals_mix	0	0	0
UIJ68212.1	DUF6376_family_protein_[Bacillus_cereus]	CPTF_zcontrol	115980	5081.235184	4.381130526
UIJ68217.1	nitroreductase_family_protein_[Bacillus_cereus]	CPTF_Al	4397519.733	294936.5409	6.706883852
UIJ68217.1	nitroreductase_family_protein_[Bacillus_cereus]	CPTF_Cd	4644996.1	335261.6258	7.217694452
UIJ68217.1	nitroreductase_family_protein_[Bacillus_cereus]	CPTF_Co	3391859.833	639876.3153	18.86505772
UIJ68217.1	nitroreductase_family_protein_[Bacillus_cereus]	CPTF_Cu	4982125.233	351230.2756	7.049808248
UIJ68217.1	nitroreductase_family_protein_[Bacillus_cereus]	CPTF_Fe	3900043.2	164652.5061	4.221812366
UIJ68217.1	nitroreductase_family_protein_[Bacillus_cereus]	CPTF_Mn	3874017.967	385668.5312	9.955259229
UIJ68217.1	nitroreductase_family_protein_[Bacillus_cereus]	CPTF_Ni	3893374.833	204256.5672	5.246260018
UIJ68217.1	nitroreductase_family_protein_[Bacillus_cereus]	CPTF_U	3659364.533	423734.1417	11.57944604
UIJ68217.1	nitroreductase_family_protein_[Bacillus_cereus]	CPTF_metals_mix	6569502.333	266266.9249	4.053076038
UIJ68217.1	nitroreductase_family_protein_[Bacillus_cereus]	CPTF_zcontrol	4410755.533	571794.5308	12.96364141
UIJ68219.1	SH3_domain-containing_protein_[Bacillus_cereus]	CPTF_Al	3661930.1	276210.6854	7.542762363
UIJ68219.1	SH3_domain-containing_protein_[Bacillus_cereus]	CPTF_Cd	3730510.267	105417.7155	2.825825636
UIJ68219.1	SH3_domain-containing_protein_[Bacillus_cereus]	CPTF_Co	3832065.467	260897.9182	6.808284475
UIJ68219.1	SH3_domain-containing_protein_[Bacillus_cereus]	CPTF_Cu	6213599.333	263075.462	4.233865879

UIJ68219.1	SH3_domain-containing_protein_[Bacillus_cereus]	CPTF_Fe	4031777.033	683411.7956	16.95063467
UIJ68219.1	SH3_domain-containing_protein_[Bacillus_cereus]	CPTF_Mn	3638871.567	701509.3318	19.27821081
UIJ68219.1	SH3_domain-containing_protein_[Bacillus_cereus]	CPTF_Ni	3682922.933	423208.6084	11.49110682
UIJ68219.1	SH3_domain-containing_protein_[Bacillus_cereus]	CPTF_U	2854136.667	303931.8764	10.64882001
UIJ68219.1	SH3_domain-containing_protein_[Bacillus_cereus]	CPTF_metals_mix	6489752.7	159222.6944	2.453447793
UIJ68219.1	SH3_domain-containing_protein_[Bacillus_cereus]	CPTF_zcontrol	3715100.833	442332.2434	11.90633211
UIJ68225.1	3-ketoacyl-ACP_reductase_[Bacillus_cereus]	CPTF_Al	808923.6667	217111.3286	26.83953228
UIJ68225.1	3-ketoacyl-ACP_reductase_[Bacillus_cereus]	CPTF_Cd	1041271.333	113424.881	10.89292266
UIJ68225.1	3-ketoacyl-ACP_reductase_[Bacillus_cereus]	CPTF_Co	754849	154616.8022	20.48314327
UIJ68225.1	3-ketoacyl-ACP_reductase_[Bacillus_cereus]	CPTF_Cu	773830.6667	134062.9426	17.32458384
UIJ68225.1	3-ketoacyl-ACP_reductase_[Bacillus_cereus]	CPTF_Fe	739624.3333	49831.27265	6.737376045
UIJ68225.1	3-ketoacyl-ACP_reductase_[Bacillus_cereus]	CPTF_Mn	780715.3333	257750.216	33.01462198
UIJ68225.1	3-ketoacyl-ACP_reductase_[Bacillus_cereus]	CPTF_Ni	514785.3333	445864.1367	86.61166273
UIJ68225.1	3-ketoacyl-ACP_reductase_[Bacillus_cereus]	CPTF_U	599855.7667	128169.1384	21.36665938
UIJ68225.1	3-ketoacyl-ACP_reductase_[Bacillus_cereus]	CPTF_metals_mix	1163678	118739.701	10.20382795
UIJ68225.1	3-ketoacyl-ACP_reductase_[Bacillus_cereus]	CPTF_zcontrol	610338	22207.48261	3.638554803
UIJ68228.1	NUDIX_domain-containing_protein_[Bacillus_cereus]	CPTF_Al	29958.9	51890.33694	173.2050808
UIJ68228.1	NUDIX_domain-containing_protein_[Bacillus_cereus]	CPTF_Cd	109840.1333	21300.3329	19.39212221
UIJ68228.1	NUDIX_domain-containing_protein_[Bacillus_cereus]	CPTF_Co	16551.56667	28668.15441	173.2050808
UIJ68228.1	NUDIX_domain-containing_protein_[Bacillus_cereus]	CPTF_Cu	63220.3	48197.23413	76.23695891
UIJ68228.1	NUDIX_domain-containing_protein_[Bacillus_cereus]	CPTF_Fe	103153.8667	23663.61911	22.94011837
UIJ68228.1	NUDIX_domain-containing_protein_[Bacillus_cereus]	CPTF_Mn	56391.9	52878.31783	93.76934956
UIJ68228.1	NUDIX_domain-containing_protein_[Bacillus_cereus]	CPTF_Ni	115439.3333	100010.1459	86.63437583
UIJ68228.1	NUDIX_domain-containing_protein_[Bacillus_cereus]	CPTF_U	144323	12186.52465	8.443924148
UIJ68228.1	NUDIX_domain-containing_protein_[Bacillus_cereus]	CPTF_metals_mix	17109.16667	29633.94594	173.2050808
UIJ68228.1	NUDIX_domain-containing_protein_[Bacillus_cereus]	CPTF_zcontrol	44341.76667	51345.09666	115.7939805
UIJ68230.1	homoserine_dehydrogenase_[Bacillus_cereus]	CPTF_Al	636514.3667	81457.19635	12.79738536
UIJ68230.1	homoserine_dehydrogenase_[Bacillus_cereus]	CPTF_Cd	605141	175206.4787	28.95300081
UIJ68230.1	homoserine_dehydrogenase_[Bacillus_cereus]	CPTF_Co	625329.9667	133373.5069	21.3285008
UIJ68230.1	homoserine_dehydrogenase_[Bacillus_cereus]	CPTF_Cu	555220.3333	258071.688	46.48095044
UIJ68230.1	homoserine_dehydrogenase_[Bacillus_cereus]	CPTF_Fe	818093.7833	96063.57518	11.74236709
UIJ68230.1	homoserine_dehydrogenase_[Bacillus_cereus]	CPTF_Mn	726259.2333	408970.1438	56.31186841
UIJ68230.1	homoserine_dehydrogenase_[Bacillus_cereus]	CPTF_Ni	672415.1333	300423.8176	44.67832485
UIJ68230.1	homoserine_dehydrogenase_[Bacillus_cereus]	CPTF_U	753280.1667	439252.1788	58.31192672
UIJ68230.1	homoserine_dehydrogenase_[Bacillus_cereus]	CPTF_metals_mix	108656.0667	45521.27609	41.89483154
UIJ68230.1	homoserine_dehydrogenase_[Bacillus_cereus]	CPTF_zcontrol	683932.4	162328.6218	23.73460034
UIJ68231.1	threonine_synthase_[Bacillus_cereus]	CPTF_Al	1226328.033	409826.144	33.41896563
UIJ68231.1	threonine_synthase_[Bacillus_cereus]	CPTF_Cd	1184002.133	359590.2782	30.3707458
UIJ68231.1	threonine_synthase_[Bacillus_cereus]	CPTF_Co	1228439.667	564397.143	45.94422977
UIJ68231.1	threonine_synthase_[Bacillus_cereus]	CPTF_Cu	974140.3333	668281.3521	68.60216431
UIJ68231.1	threonine_synthase_[Bacillus_cereus]	CPTF_Fe	1286121.6	208707.7601	16.22768485
UIJ68231.1	threonine_synthase_[Bacillus_cereus]	CPTF_Mn	1343599.933	674179.4004	50.17709391
UIJ68231.1	threonine_synthase_[Bacillus_cereus]	CPTF_Ni	1282494.667	722780.323	56.35737456
UIJ68231.1	threonine_synthase_[Bacillus_cereus]	CPTF_U	976069.5	324831.8086	33.27957779
UIJ68231.1	threonine_synthase_[Bacillus_cereus]	CPTF_metals_mix	77191.26667	7992.659843	10.35435767
UIJ68231.1	threonine_synthase_[Bacillus_cereus]	CPTF_zcontrol	1094481.333	144096.0656	13.16569422
UIJ68239.1	YkyA_family_protein_[Bacillus_cereus]	CPTF_Al	1753333.333	502427.4409	28.65555746
UIJ68239.1	YkyA_family_protein_[Bacillus_cereus]	CPTF_Cd	2480000	426731.7659	17.20692604
UIJ68239.1	YkyA_family_protein_[Bacillus_cereus]	CPTF_Co	1221876.667	345854.9777	28.30522811
UIJ68239.1	YkyA_family_protein_[Bacillus_cereus]	CPTF_Cu	915975.6667	151547.3368	16.54490859
UIJ68239.1	YkyA_family_protein_[Bacillus_cereus]	CPTF_Fe	2050000	1054324.428	51.43045991
UIJ68239.1	YkyA_family_protein_[Bacillus_cereus]	CPTF_Mn	1543333.333	332014.0559	21.5127898
UIJ68239.1	YkyA_family_protein_[Bacillus_cereus]	CPTF_Ni	343315.3333	324692.3742	94.57555274
UIJ68239.1	YkyA_family_protein_[Bacillus_cereus]	CPTF_U	1007452.333	505167.1326	50.14303068
UIJ68239.1	YkyA_family_protein_[Bacillus_cereus]	CPTF_metals_mix	901895	374583.1256	41.53289746
UIJ68239.1	YkyA_family_protein_[Bacillus_cereus]	CPTF_zcontrol	1456666.667	256969.5183	17.64092803
UIJ68252.1	DUF1963_domain-containing_protein_[Bacillus_cereus]	CPTF_Al	0	0	0
UIJ68252.1	DUF1963_domain-containing_protein_[Bacillus_cereus]	CPTF_Cd	0	0	0
UIJ68252.1	DUF1963_domain-containing_protein_[Bacillus_cereus]	CPTF_Co	0	0	0
UIJ68252.1	DUF1963_domain-containing_protein_[Bacillus_cereus]	CPTF_Cu	0	0	0
UIJ68252.1	DUF1963_domain-containing_protein_[Bacillus_cereus]	CPTF_Fe	0	0	0

UIJ68252.1	DUF1963_domain-containing_protein_[Bacillus_cereus]	CPTF_Mn	0	0	0
UIJ68252.1	DUF1963_domain-containing_protein_[Bacillus_cereus]	CPTF_Ni	0	0	0
UIJ68252.1	DUF1963_domain-containing_protein_[Bacillus_cereus]	CPTF_U	0	0	0
UIJ68252.1	DUF1963_domain-containing_protein_[Bacillus_cereus]	CPTF_metals_mix	28915.9	50083.80795	173.2050808
UIJ68252.1	DUF1963_domain-containing_protein_[Bacillus_cereus]	CPTF_zcontrol	0	0	0
UIJ68256.1	ammonia-dependent_NAD(+)_synthetase_[Bacillus_cereus]	CPTF_Al	2057301.133	75421.21204	3.666026855
UIJ68256.1	ammonia-dependent_NAD(+)_synthetase_[Bacillus_cereus]	CPTF_Cd	2026334.3	235897.4714	11.64158705
UIJ68256.1	ammonia-dependent_NAD(+)_synthetase_[Bacillus_cereus]	CPTF_Co	1845303.767	74814.13812	4.054299323
UIJ68256.1	ammonia-dependent_NAD(+)_synthetase_[Bacillus_cereus]	CPTF_Cu	2322407.5	189753.4059	8.170547412
UIJ68256.1	ammonia-dependent_NAD(+)_synthetase_[Bacillus_cereus]	CPTF_Fe	2000752.5	355746.9449	17.78065727
UIJ68256.1	ammonia-dependent_NAD(+)_synthetase_[Bacillus_cereus]	CPTF_Mn	1846637.167	177495.2314	9.611808674
UIJ68256.1	ammonia-dependent_NAD(+)_synthetase_[Bacillus_cereus]	CPTF_Ni	1958163.333	360043.3627	18.38678912
UIJ68256.1	ammonia-dependent_NAD(+)_synthetase_[Bacillus_cereus]	CPTF_U	2153996.667	418268.1456	19.41823551
UIJ68256.1	ammonia-dependent_NAD(+)_synthetase_[Bacillus_cereus]	CPTF_metals_mix	2459338.367	66224.44695	2.692774929
UIJ68256.1	ammonia-dependent_NAD(+)_synthetase_[Bacillus_cereus]	CPTF_zcontrol	1674264.1	250979.9592	14.99046412
UIJ68257.1	AbrB/MazE/SpoVT_family_DNA-binding_domain-containing_protein_[Bacillus_cereus]	CPTF_Al	78363.43333	51567.05417	65.804996
UIJ68257.1	AbrB/MazE/SpoVT_family_DNA-binding_domain-containing_protein_[Bacillus_cereus]	CPTF_Cd	71369	19382.12851	27.15762938
UIJ68257.1	AbrB/MazE/SpoVT_family_DNA-binding_domain-containing_protein_[Bacillus_cereus]	CPTF_Co	126071.2	51991.44307	41.23974633
UIJ68257.1	AbrB/MazE/SpoVT_family_DNA-binding_domain-containing_protein_[Bacillus_cereus]	CPTF_Cu	127641.3333	23013.4074	18.02974538
UIJ68257.1	AbrB/MazE/SpoVT_family_DNA-binding_domain-containing_protein_[Bacillus_cereus]	CPTF_Fe	61675.13333	57327.07116	92.9500563
UIJ68257.1	AbrB/MazE/SpoVT_family_DNA-binding_domain-containing_protein_[Bacillus_cereus]	CPTF_Mn	95392.46667	43746.08837	45.85905983
UIJ68257.1	AbrB/MazE/SpoVT_family_DNA-binding_domain-containing_protein_[Bacillus_cereus]	CPTF_Ni	100130.9	29894.84253	29.85576134
UIJ68257.1	AbrB/MazE/SpoVT_family_DNA-binding_domain-containing_protein_[Bacillus_cereus]	CPTF_U	35475.16667	33593.00255	94.69441782
UIJ68257.1	AbrB/MazE/SpoVT_family_DNA-binding_domain-containing_protein_[Bacillus_cereus]	CPTF_metals_mix	116243.4333	27047.55655	23.26802966
UIJ68257.1	AbrB/MazE/SpoVT_family_DNA-binding_domain-containing_protein_[Bacillus_cereus]	CPTF_zcontrol	75026.56667	68351.0373	91.10244589
UIJ68280.1	DinB_family_protein_[Bacillus_cereus]	CPTF_Al	0	0	0
UIJ68280.1	DinB_family_protein_[Bacillus_cereus]	CPTF_Cd	18924.5	16433.75181	86.83849936
UIJ68280.1	DinB_family_protein_[Bacillus_cereus]	CPTF_Co	3596.333333	6229.032054	173.2050808
UIJ68280.1	DinB_family_protein_[Bacillus_cereus]	CPTF_Cu	18447.8	17775.59683	96.35618789
UIJ68280.1	DinB_family_protein_[Bacillus_cereus]	CPTF_Fe	20781	18189.14325	87.52775731
UIJ68280.1	DinB_family_protein_[Bacillus_cereus]	CPTF_Mn	0	0	0
UIJ68280.1	DinB_family_protein_[Bacillus_cereus]	CPTF_Ni	14319.5	24802.10154	173.2050808
UIJ68280.1	DinB_family_protein_[Bacillus_cereus]	CPTF_U	7176.833333	12430.63997	173.2050808
UIJ68280.1	DinB_family_protein_[Bacillus_cereus]	CPTF_metals_mix	0	0	0
UIJ68280.1	DinB_family_protein_[Bacillus_cereus]	CPTF_zcontrol	0	0	0
UIJ68285.1	DNA_starvation/stationary_phase_protection_protein_[Bacillus_cereus]	CPTF_Al	3627112.667	297124.1412	8.191753841
UIJ68285.1	DNA_starvation/stationary_phase_protection_protein_[Bacillus_cereus]	CPTF_Cd	3675773	236241.7248	6.426994399
UIJ68285.1	DNA_starvation/stationary_phase_protection_protein_[Bacillus_cereus]	CPTF_Co	3772344.333	437540.1079	11.59862593
UIJ68285.1	DNA_starvation/stationary_phase_protection_protein_[Bacillus_cereus]	CPTF_Cu	3589212.667	365829.5384	10.19247318
UIJ68285.1	DNA_starvation/stationary_phase_protection_protein_[Bacillus_cereus]	CPTF_Fe	3426687.333	114442.3526	3.339737229
UIJ68285.1	DNA_starvation/stationary_phase_protection_protein_[Bacillus_cereus]	CPTF_Mn	3623745.667	1203856.643	33.22133376
UIJ68285.1	DNA_starvation/stationary_phase_protection_protein_[Bacillus_cereus]	CPTF_Ni	2272035.667	794579.0385	34.97211994
UIJ68285.1	DNA_starvation/stationary_phase_protection_protein_[Bacillus_cereus]	CPTF_U	2587474.667	409765.9471	15.83652016
UIJ68285.1	DNA_starvation/stationary_phase_protection_protein_[Bacillus_cereus]	CPTF_metals_mix	5236330.667	352745.6066	6.736503653
UIJ68285.1	DNA_starvation/stationary_phase_protection_protein_[Bacillus_cereus]	CPTF_zcontrol	3356289.667	391553.6192	11.66626418
UIJ68286.1	DUF3939_domain-containing_protein_[Bacillus_cereus]	CPTF_Al	112534.5333	122036.6657	108.443748
UIJ68286.1	DUF3939_domain-containing_protein_[Bacillus_cereus]	CPTF_Cd	22584.56667	39117.61693	173.2050808
UIJ68286.1	DUF3939_domain-containing_protein_[Bacillus_cereus]	CPTF_Co	182957.6667	65512.53644	35.80748357
UIJ68286.1	DUF3939_domain-containing_protein_[Bacillus_cereus]	CPTF_Cu	193282.3333	71133.68987	36.80299624
UIJ68286.1	DUF3939_domain-containing_protein_[Bacillus_cereus]	CPTF_Fe	37608	65138.96677	173.2050808
UIJ68286.1	DUF3939_domain-containing_protein_[Bacillus_cereus]	CPTF_Mn	93323	80927.31187	86.71743501
UIJ68286.1	DUF3939_domain-containing_protein_[Bacillus_cereus]	CPTF_Ni	239704.6667	43425.57215	18.11628149
UIJ68286.1	DUF3939_domain-containing_protein_[Bacillus_cereus]	CPTF_U	160184	172020.1743	107.3891114
UIJ68286.1	DUF3939_domain-containing_protein_[Bacillus_cereus]	CPTF_metals_mix	240176.8333	69828.50389	29.07378822
UIJ68286.1	DUF3939_domain-containing_protein_[Bacillus_cereus]	CPTF_zcontrol	110420	96012.27996	86.95189274
UIJ68287.1	alpha/beta_hydrolase_[Bacillus_cereus]	CPTF_Al	0	0	0
UIJ68287.1	alpha/beta_hydrolase_[Bacillus_cereus]	CPTF_Cd	0	0	0
UIJ68287.1	alpha/beta_hydrolase_[Bacillus_cereus]	CPTF_Co	51459.63333	16948.41823	32.93536533
UIJ68287.1	alpha/beta_hydrolase_[Bacillus_cereus]	CPTF_Cu	0	0	0
UIJ68287.1	alpha/beta_hydrolase_[Bacillus_cereus]	CPTF_Fe	14101.36667	24424.28352	173.2050808
UIJ68287.1	alpha/beta_hydrolase_[Bacillus_cereus]	CPTF_Mn	0	0	0

UIJ68287.1	alpha/beta_hydrolase_[Bacillus_cereus]	CPTF_Ni	0	0	0
UIJ68287.1	alpha/beta_hydrolase_[Bacillus_cereus]	CPTF_U	0	0	0
UIJ68287.1	alpha/beta_hydrolase_[Bacillus_cereus]	CPTF_metals_mix	0	0	0
UIJ68287.1	alpha/beta_hydrolase_[Bacillus_cereus]	CPTF_zcontrol	0	0	0
UIJ68291.1	GNAT_family_N-acetyltransferase_[Bacillus_cereus]	CPTF_Al	332392.8333	137964.4298	41.50643936
UIJ68291.1	GNAT_family_N-acetyltransferase_[Bacillus_cereus]	CPTF_Cd	121418.6	37913.12232	31.22513546
UIJ68291.1	GNAT_family_N-acetyltransferase_[Bacillus_cereus]	CPTF_Co	407045.5	149514.6026	36.73166823
UIJ68291.1	GNAT_family_N-acetyltransferase_[Bacillus_cereus]	CPTF_Cu	414919.1667	60439.5348	14.56658059
UIJ68291.1	GNAT_family_N-acetyltransferase_[Bacillus_cereus]	CPTF_Fe	311347.8667	152454.1269	48.96584921
UIJ68291.1	GNAT_family_N-acetyltransferase_[Bacillus_cereus]	CPTF_Mn	241293.0667	250821.086	103.9487331
UIJ68291.1	GNAT_family_N-acetyltransferase_[Bacillus_cereus]	CPTF_Ni	523322.6667	143459.787	27.41325689
UIJ68291.1	GNAT_family_N-acetyltransferase_[Bacillus_cereus]	CPTF_U	534183.6667	75821.96194	14.19398733
UIJ68291.1	GNAT_family_N-acetyltransferase_[Bacillus_cereus]	CPTF_metals_mix	160701	53429.57948	33.24782016
UIJ68291.1	GNAT_family_N-acetyltransferase_[Bacillus_cereus]	CPTF_zcontrol	379955.6667	117908.5086	31.03217532
UIJ68298.1	NADPH_dehydrogenase_NamA_[Bacillus_cereus]	CPTF_Al	6595.233333	11423.27922	173.2050808
UIJ68298.1	NADPH_dehydrogenase_NamA_[Bacillus_cereus]	CPTF_Cd	0	0	0
UIJ68298.1	NADPH_dehydrogenase_NamA_[Bacillus_cereus]	CPTF_Co	7203.966667	12477.63628	173.2050808
UIJ68298.1	NADPH_dehydrogenase_NamA_[Bacillus_cereus]	CPTF_Cu	0	0	0
UIJ68298.1	NADPH_dehydrogenase_NamA_[Bacillus_cereus]	CPTF_Fe	0	0	0
UIJ68298.1	NADPH_dehydrogenase_NamA_[Bacillus_cereus]	CPTF_Mn	0	0	0
UIJ68298.1	NADPH_dehydrogenase_NamA_[Bacillus_cereus]	CPTF_Ni	0	0	0
UIJ68298.1	NADPH_dehydrogenase_NamA_[Bacillus_cereus]	CPTF_U	8475.766667	14680.4585	173.2050808
UIJ68298.1	NADPH_dehydrogenase_NamA_[Bacillus_cereus]	CPTF_metals_mix	112518.1667	20563.75112	18.27593866
UIJ68298.1	NADPH_dehydrogenase_NamA_[Bacillus_cereus]	CPTF_zcontrol	0	0	0
UIJ68301.1	peptide_ABC_transporter_substrate-binding_protein_[Bacillus_cereus]	CPTF_Al	625499.5667	459683.4976	73.49061807
UIJ68301.1	peptide_ABC_transporter_substrate-binding_protein_[Bacillus_cereus]	CPTF_Cd	187857.3333	113693.2439	60.52105704
UIJ68301.1	peptide_ABC_transporter_substrate-binding_protein_[Bacillus_cereus]	CPTF_Co	361313.7	421397.1061	116.6291525
UIJ68301.1	peptide_ABC_transporter_substrate-binding_protein_[Bacillus_cereus]	CPTF_Cu	797029.8667	241568.7602	30.30862083
UIJ68301.1	peptide_ABC_transporter_substrate-binding_protein_[Bacillus_cereus]	CPTF_Fe	498130.6667	635981.3768	127.6736044
UIJ68301.1	peptide_ABC_transporter_substrate-binding_protein_[Bacillus_cereus]	CPTF_Mn	581743.6667	819507.4111	140.870878
UIJ68301.1	peptide_ABC_transporter_substrate-binding_protein_[Bacillus_cereus]	CPTF_Ni	1724802.6	526423.1582	30.52077717
UIJ68301.1	peptide_ABC_transporter_substrate-binding_protein_[Bacillus_cereus]	CPTF_U	1285595.633	166721.8329	12.96845047
UIJ68301.1	peptide_ABC_transporter_substrate-binding_protein_[Bacillus_cereus]	CPTF_metals_mix	12080.1	11990.91782	99.26174302
UIJ68301.1	peptide_ABC_transporter_substrate-binding_protein_[Bacillus_cereus]	CPTF_zcontrol	501159.7	754930.9241	150.6367978
UIJ68304.1	phosphoserine_phosphatase_1_[Bacillus_cereus]	CPTF_Al	31123.53333	27576.07415	88.60200368
UIJ68304.1	phosphoserine_phosphatase_1_[Bacillus_cereus]	CPTF_Cd	21582.13333	37381.35147	173.2050808
UIJ68304.1	phosphoserine_phosphatase_1_[Bacillus_cereus]	CPTF_Co	29711.26667	27815.45102	93.61920288
UIJ68304.1	phosphoserine_phosphatase_1_[Bacillus_cereus]	CPTF_Cu	15883.03333	27510.22071	173.2050808
UIJ68304.1	phosphoserine_phosphatase_1_[Bacillus_cereus]	CPTF_Fe	17476.53333	30270.24367	173.2050808
UIJ68304.1	phosphoserine_phosphatase_1_[Bacillus_cereus]	CPTF_Mn	0	0	0
UIJ68304.1	phosphoserine_phosphatase_1_[Bacillus_cereus]	CPTF_Ni	6866.9	11893.81969	173.2050808
UIJ68304.1	phosphoserine_phosphatase_1_[Bacillus_cereus]	CPTF_U	0	0	0
UIJ68304.1	phosphoserine_phosphatase_1_[Bacillus_cereus]	CPTF_metals_mix	47318.16667	40988.7033	86.6236082
UIJ68304.1	phosphoserine_phosphatase_1_[Bacillus_cereus]	CPTF_zcontrol	14401.5	24944.12971	173.2050808
UIJ68307.1	NUDIX_hydrolase_[Bacillus_cereus]	CPTF_Al	0	0	0
UIJ68307.1	NUDIX_hydrolase_[Bacillus_cereus]	CPTF_Cd	0	0	0
UIJ68307.1	NUDIX_hydrolase_[Bacillus_cereus]	CPTF_Co	0	0	0
UIJ68307.1	NUDIX_hydrolase_[Bacillus_cereus]	CPTF_Cu	15496.16667	26840.14799	173.2050808
UIJ68307.1	NUDIX_hydrolase_[Bacillus_cereus]	CPTF_Fe	12344.23333	21380.83931	173.2050808
UIJ68307.1	NUDIX_hydrolase_[Bacillus_cereus]	CPTF_Mn	0	0	0
UIJ68307.1	NUDIX_hydrolase_[Bacillus_cereus]	CPTF_Ni	14178.76667	24558.34426	173.2050808
UIJ68307.1	NUDIX_hydrolase_[Bacillus_cereus]	CPTF_U	35456.3	41253.3588	116.3498696
UIJ68307.1	NUDIX_hydrolase_[Bacillus_cereus]	CPTF_metals_mix	0	0	0
UIJ68307.1	NUDIX_hydrolase_[Bacillus_cereus]	CPTF_zcontrol	17853.6	30923.3423	173.2050808
UIJ68320.1	cyclic_di-AMP_binding_protein_CbpA_[Bacillus_cereus]	CPTF_Al	816891.9333	402040.5299	49.21587709
UIJ68320.1	cyclic_di-AMP_binding_protein_CbpA_[Bacillus_cereus]	CPTF_Cd	396033.5667	194015.7229	48.98971683
UIJ68320.1	cyclic_di-AMP_binding_protein_CbpA_[Bacillus_cereus]	CPTF_Co	1020278.267	146659.4321	14.37445419
UIJ68320.1	cyclic_di-AMP_binding_protein_CbpA_[Bacillus_cereus]	CPTF_Cu	1282053.3	547737.9181	42.72349036
UIJ68320.1	cyclic_di-AMP_binding_protein_CbpA_[Bacillus_cereus]	CPTF_Fe	595621.9333	417976.753	70.17484239
UIJ68320.1	cyclic_di-AMP_binding_protein_CbpA_[Bacillus_cereus]	CPTF_Mn	705156.5333	369837.8046	52.44761795
UIJ68320.1	cyclic_di-AMP_binding_protein_CbpA_[Bacillus_cereus]	CPTF_Ni	504222.2667	201263.9159	39.9157136

UIJ68320.1	cyclic_di-AMP_binding_protein_CbpA_[Bacillus_cereus]	CPTF_U	565803.4	211076.5393	37.30563289
UIJ68320.1	cyclic_di-AMP_binding_protein_CbpA_[Bacillus_cereus]	CPTF_metals_mix	2692924.767	445795.6314	16.55432921
UIJ68320.1	cyclic_di-AMP_binding_protein_CbpA_[Bacillus_cereus]	CPTF_zcontrol	574377.8333	392751.5888	68.37861178
UIJ68322.1	chaperone_CsaA_[Bacillus_cereus]	CPTF_Al	41242.36667	41908.31151	101.6147105
UIJ68322.1	chaperone_CsaA_[Bacillus_cereus]	CPTF_Cd	190540.3333	24796.11837	13.01357982
UIJ68322.1	chaperone_CsaA_[Bacillus_cereus]	CPTF_Co	24887.06667	43105.66392	173.2050808
UIJ68322.1	chaperone_CsaA_[Bacillus_cereus]	CPTF_Cu	0	0	0
UIJ68322.1	chaperone_CsaA_[Bacillus_cereus]	CPTF_Fe	94421.66667	83622.04185	88.56234464
UIJ68322.1	chaperone_CsaA_[Bacillus_cereus]	CPTF_Mn	32679.23333	56602.09249	173.2050808
UIJ68322.1	chaperone_CsaA_[Bacillus_cereus]	CPTF_Ni	0	0	0
UIJ68322.1	chaperone_CsaA_[Bacillus_cereus]	CPTF_U	0	0	0
UIJ68322.1	chaperone_CsaA_[Bacillus_cereus]	CPTF_metals_mix	111190.3	22361.87747	20.11135636
UIJ68322.1	chaperone_CsaA_[Bacillus_cereus]	CPTF_zcontrol	0	0	0
UIJ68328.1	NUDIX_domain-containing_protein_[Bacillus_cereus]	CPTF_Al	402278.8333	102552.2111	25.49281807
UIJ68328.1	NUDIX_domain-containing_protein_[Bacillus_cereus]	CPTF_Cd	390534.7	104954.3978	26.87453838
UIJ68328.1	NUDIX_domain-containing_protein_[Bacillus_cereus]	CPTF_Co	294226.0667	161587.2939	54.91943515
UIJ68328.1	NUDIX_domain-containing_protein_[Bacillus_cereus]	CPTF_Cu	285235.6	61678.96284	21.62386562
UIJ68328.1	NUDIX_domain-containing_protein_[Bacillus_cereus]	CPTF_Fe	290916.6	144824.6094	49.78217447
UIJ68328.1	NUDIX_domain-containing_protein_[Bacillus_cereus]	CPTF_Mn	287253.1333	122907.3855	42.78713486
UIJ68328.1	NUDIX_domain-containing_protein_[Bacillus_cereus]	CPTF_Ni	349776.2667	51764.86084	14.79942059
UIJ68328.1	NUDIX_domain-containing_protein_[Bacillus_cereus]	CPTF_U	206400.4667	157839.5383	76.47247164
UIJ68328.1	NUDIX_domain-containing_protein_[Bacillus_cereus]	CPTF_metals_mix	132691.1667	31153.30655	23.4780561
UIJ68328.1	NUDIX_domain-containing_protein_[Bacillus_cereus]	CPTF_zcontrol	278899.9667	49572.92318	17.77444572
UIJ68331.1	SPFH/Band_7/PHB_domain_protein_[Bacillus_cereus]	CPTF_Al	35027.33333	60669.12099	173.2050808
UIJ68331.1	SPFH/Band_7/PHB_domain_protein_[Bacillus_cereus]	CPTF_Cd	0	0	0
UIJ68331.1	SPFH/Band_7/PHB_domain_protein_[Bacillus_cereus]	CPTF_Co	93500.66667	82644.3466	88.38904528
UIJ68331.1	SPFH/Band_7/PHB_domain_protein_[Bacillus_cereus]	CPTF_Cu	0	0	0
UIJ68331.1	SPFH/Band_7/PHB_domain_protein_[Bacillus_cereus]	CPTF_Fe	0	0	0
UIJ68331.1	SPFH/Band_7/PHB_domain_protein_[Bacillus_cereus]	CPTF_Mn	101620.6667	92016.08199	90.54859115
UIJ68331.1	SPFH/Band_7/PHB_domain_protein_[Bacillus_cereus]	CPTF_Ni	89857.66667	79334.49597	88.28906749
UIJ68331.1	SPFH/Band_7/PHB_domain_protein_[Bacillus_cereus]	CPTF_U	0	0	0
UIJ68331.1	SPFH/Band_7/PHB_domain_protein_[Bacillus_cereus]	CPTF_metals_mix	0	0	0
UIJ68331.1	SPFH/Band_7/PHB_domain_protein_[Bacillus_cereus]	CPTF_zcontrol	0	0	0
UIJ68339.1	glycosyl_transferase_[Bacillus_cereus]	CPTF_Al	1158390.1	162033.5525	13.98782262
UIJ68339.1	glycosyl_transferase_[Bacillus_cereus]	CPTF_Cd	1527484.6	383050.028	25.07717773
UIJ68339.1	glycosyl_transferase_[Bacillus_cereus]	CPTF_Co	1596908.333	275926.869	17.27881703
UIJ68339.1	glycosyl_transferase_[Bacillus_cereus]	CPTF_Cu	2932187.3	226594.615	7.727835635
UIJ68339.1	glycosyl_transferase_[Bacillus_cereus]	CPTF_Fe	1552469.033	149626.8504	9.637992592
UIJ68339.1	glycosyl_transferase_[Bacillus_cereus]	CPTF_Mn	1120446.2	148718.9611	13.27319072
UIJ68339.1	glycosyl_transferase_[Bacillus_cereus]	CPTF_Ni	1480889.933	743472.9187	50.20446841
UIJ68339.1	glycosyl_transferase_[Bacillus_cereus]	CPTF_U	771446.9667	524154.5055	67.94433424
UIJ68339.1	glycosyl_transferase_[Bacillus_cereus]	CPTF_metals_mix	1395683.6	160976.3745	11.53387305
UIJ68339.1	glycosyl_transferase_[Bacillus_cereus]	CPTF_zcontrol	635708.2667	276599.6487	43.51046907
UIJ68342.1	GNAT_family_N-acetyltransferase_[Bacillus_cereus]	CPTF_Al	781521.6667	82731.27272	10.58592183
UIJ68342.1	GNAT_family_N-acetyltransferase_[Bacillus_cereus]	CPTF_Cd	771752	59570.34244	7.718845231
UIJ68342.1	GNAT_family_N-acetyltransferase_[Bacillus_cereus]	CPTF_Co	638256	97425.73142	15.26436593
UIJ68342.1	GNAT_family_N-acetyltransferase_[Bacillus_cereus]	CPTF_Cu	773201.6667	86847.06851	11.23213674
UIJ68342.1	GNAT_family_N-acetyltransferase_[Bacillus_cereus]	CPTF_Fe	635252.3333	56134.08784	8.836502425
UIJ68342.1	GNAT_family_N-acetyltransferase_[Bacillus_cereus]	CPTF_Mn	834639	57254.8202	6.859830442
UIJ68342.1	GNAT_family_N-acetyltransferase_[Bacillus_cereus]	CPTF_Ni	683818.6667	44923.54445	6.569511282
UIJ68342.1	GNAT_family_N-acetyltransferase_[Bacillus_cereus]	CPTF_U	633724.3333	127120.1474	20.0592183
UIJ68342.1	GNAT_family_N-acetyltransferase_[Bacillus_cereus]	CPTF_metals_mix	543874.3333	93668.29125	17.22241435
UIJ68342.1	GNAT_family_N-acetyltransferase_[Bacillus_cereus]	CPTF_zcontrol	548119.6667	241945.825	44.14105892
UIJ68347.1	GNAT_family_N-acetyltransferase_[Bacillus_cereus]	CPTF_Al	152931.3333	12990.46044	8.494309278
UIJ68347.1	GNAT_family_N-acetyltransferase_[Bacillus_cereus]	CPTF_Cd	150207.3333	10252.76813	6.825744058
UIJ68347.1	GNAT_family_N-acetyltransferase_[Bacillus_cereus]	CPTF_Co	161606.3333	62417.6163	38.62324886
UIJ68347.1	GNAT_family_N-acetyltransferase_[Bacillus_cereus]	CPTF_Cu	202149.5667	47893.35095	23.69203741
UIJ68347.1	GNAT_family_N-acetyltransferase_[Bacillus_cereus]	CPTF_Fe	153055.6667	12635.09724	8.255229955
UIJ68347.1	GNAT_family_N-acetyltransferase_[Bacillus_cereus]	CPTF_Mn	108959.3333	50624.49115	46.46182167
UIJ68347.1	GNAT_family_N-acetyltransferase_[Bacillus_cereus]	CPTF_Ni	154441.6667	5206.639447	3.371266032
UIJ68347.1	GNAT_family_N-acetyltransferase_[Bacillus_cereus]	CPTF_U	127866	14863.87032	11.62456816

UIJ68347.1	GNAT_family_N-acetyltransferase_[Bacillus_cereus]	CPTF_metals_mix	83431.3	10802.29066	12.94752768
UIJ68347.1	GNAT_family_N-acetyltransferase_[Bacillus_cereus]	CPTF_zcontrol	99441.4	34859.29537	35.05511323
UIJ68350.1	WXG100_family_type_VII_secretion_target_[Bacillus_cereus]	CPTF_Al	32891336.67	1049399.735	3.190504982
UIJ68350.1	WXG100_family_type_VII_secretion_target_[Bacillus_cereus]	CPTF_Cd	31632190.13	1625590.517	5.139038777
UIJ68350.1	WXG100_family_type_VII_secretion_target_[Bacillus_cereus]	CPTF_Co	29235820.37	2910591.696	9.955567041
UIJ68350.1	WXG100_family_type_VII_secretion_target_[Bacillus_cereus]	CPTF_Cu	27072519.67	879960.5097	3.250382752
UIJ68350.1	WXG100_family_type_VII_secretion_target_[Bacillus_cereus]	CPTF_Fe	33318110.67	4696634.172	14.09634003
UIJ68350.1	WXG100_family_type_VII_secretion_target_[Bacillus_cereus]	CPTF_Mn	32188686.3	1386237.054	4.306597171
UIJ68350.1	WXG100_family_type_VII_secretion_target_[Bacillus_cereus]	CPTF_Ni	29117643.33	1776377.301	6.100690502
UIJ68350.1	WXG100_family_type_VII_secretion_target_[Bacillus_cereus]	CPTF_U	31557075.33	5119569.155	16.22320542
UIJ68350.1	WXG100_family_type_VII_secretion_target_[Bacillus_cereus]	CPTF_metals_mix	21242823.33	905610.333	4.263135454
UIJ68350.1	WXG100_family_type_VII_secretion_target_[Bacillus_cereus]	CPTF_zcontrol	31847917	356691.0816	1.119982452
UIJ68351.1	type_VII_secretion_protein_EsaA_[Bacillus_cereus]	CPTF_Al	308353.7667	230027.1198	74.59844655
UIJ68351.1	type_VII_secretion_protein_EsaA_[Bacillus_cereus]	CPTF_Cd	126676.1667	144937.3549	114.4156463
UIJ68351.1	type_VII_secretion_protein_EsaA_[Bacillus_cereus]	CPTF_Co	8974.066667	15543.53942	173.2050808
UIJ68351.1	type_VII_secretion_protein_EsaA_[Bacillus_cereus]	CPTF_Cu	37459.6	36796.37674	98.22949722
UIJ68351.1	type_VII_secretion_protein_EsaA_[Bacillus_cereus]	CPTF_Fe	52926.8	91671.90668	173.2050808
UIJ68351.1	type_VII_secretion_protein_EsaA_[Bacillus_cereus]	CPTF_Mn	24068.06667	41687.11431	173.2050808
UIJ68351.1	type_VII_secretion_protein_EsaA_[Bacillus_cereus]	CPTF_Ni	130270.7	152331.9143	116.9349011
UIJ68351.1	type_VII_secretion_protein_EsaA_[Bacillus_cereus]	CPTF_U	124447.5	215549.3929	173.2050808
UIJ68351.1	type_VII_secretion_protein_EsaA_[Bacillus_cereus]	CPTF_metals_mix	0	0	0
UIJ68351.1	type_VII_secretion_protein_EsaA_[Bacillus_cereus]	CPTF_zcontrol	215713	231682.3381	107.4030485
UIJ68355.1	type_VII_secretion_protein_EssC_[Bacillus_cereus]	CPTF_Al	105697.8333	39898.11021	37.74733025
UIJ68355.1	type_VII_secretion_protein_EssC_[Bacillus_cereus]	CPTF_Cd	90932.5	115735.5101	127.2762875
UIJ68355.1	type_VII_secretion_protein_EssC_[Bacillus_cereus]	CPTF_Co	111353.7667	58567.48431	52.59587176
UIJ68355.1	type_VII_secretion_protein_EssC_[Bacillus_cereus]	CPTF_Cu	90225.83333	74208.23923	82.24721954
UIJ68355.1	type_VII_secretion_protein_EssC_[Bacillus_cereus]	CPTF_Fe	53641.4	53573.62909	99.87365931
UIJ68355.1	type_VII_secretion_protein_EssC_[Bacillus_cereus]	CPTF_Mn	154838.5	138316.441	89.32948914
UIJ68355.1	type_VII_secretion_protein_EssC_[Bacillus_cereus]	CPTF_Ni	178844.1	137811.6589	77.05686621
UIJ68355.1	type_VII_secretion_protein_EssC_[Bacillus_cereus]	CPTF_U	99060.83333	64419.93477	65.03068125
UIJ68355.1	type_VII_secretion_protein_EssC_[Bacillus_cereus]	CPTF_metals_mix	24934.76667	21697.83499	87.01839996
UIJ68355.1	type_VII_secretion_protein_EssC_[Bacillus_cereus]	CPTF_zcontrol	163745.8333	68066.43234	41.56834464
UIJ68377.1	formate--tetrahydrofolate_ligase_[Bacillus_cereus]	CPTF_Al	9345438.467	792428.0649	8.479303221
UIJ68377.1	formate--tetrahydrofolate_ligase_[Bacillus_cereus]	CPTF_Cd	9568694.8	346445.0416	3.620609172
UIJ68377.1	formate--tetrahydrofolate_ligase_[Bacillus_cereus]	CPTF_Co	8716594.733	993187.923	11.39421934
UIJ68377.1	formate--tetrahydrofolate_ligase_[Bacillus_cereus]	CPTF_Cu	9194117.433	730117.8196	7.941140896
UIJ68377.1	formate--tetrahydrofolate_ligase_[Bacillus_cereus]	CPTF_Fe	8307506.1	547673.9164	6.592518979
UIJ68377.1	formate--tetrahydrofolate_ligase_[Bacillus_cereus]	CPTF_Mn	8467607.367	270954.2633	3.199891677
UIJ68377.1	formate--tetrahydrofolate_ligase_[Bacillus_cereus]	CPTF_Ni	8256042.733	796401.8496	9.646290303
UIJ68377.1	formate--tetrahydrofolate_ligase_[Bacillus_cereus]	CPTF_U	7839751.5	893632.7899	11.39873872
UIJ68377.1	formate--tetrahydrofolate_ligase_[Bacillus_cereus]	CPTF_metals_mix	10791581.6	709009.1889	6.570021107
UIJ68377.1	formate--tetrahydrofolate_ligase_[Bacillus_cereus]	CPTF_zcontrol	9037445.267	417909.6884	4.624201597
UIJ68379.1	DEAD/DEAH_box_helicase_[Bacillus_cereus]	CPTF_Al	1252817.167	229141.2188	18.29007655
UIJ68379.1	DEAD/DEAH_box_helicase_[Bacillus_cereus]	CPTF_Cd	752116	240672.3453	31.99936516
UIJ68379.1	DEAD/DEAH_box_helicase_[Bacillus_cereus]	CPTF_Co	463806.9333	343099.7391	73.97468956
UIJ68379.1	DEAD/DEAH_box_helicase_[Bacillus_cereus]	CPTF_Cu	742350.8667	601069.3904	80.9683692
UIJ68379.1	DEAD/DEAH_box_helicase_[Bacillus_cereus]	CPTF_Fe	1429200.167	131431.0947	9.196129262
UIJ68379.1	DEAD/DEAH_box_helicase_[Bacillus_cereus]	CPTF_Mn	1091719.633	866582.825	79.37778149
UIJ68379.1	DEAD/DEAH_box_helicase_[Bacillus_cereus]	CPTF_Ni	712752.4667	1077268.62	151.1420402
UIJ68379.1	DEAD/DEAH_box_helicase_[Bacillus_cereus]	CPTF_U	728332.5333	977521.2061	134.2135853
UIJ68379.1	DEAD/DEAH_box_helicase_[Bacillus_cereus]	CPTF_metals_mix	593329.3	177969.8831	29.99512802
UIJ68379.1	DEAD/DEAH_box_helicase_[Bacillus_cereus]	CPTF_zcontrol	896205.2667	674973.8887	75.31465322
UIJ68381.1	VOC_family_protein_[Bacillus_cereus]	CPTF_Al	216106.3333	33317.87258	15.4173513
UIJ68381.1	VOC_family_protein_[Bacillus_cereus]	CPTF_Cd	238908	9181.744442	3.843213472
UIJ68381.1	VOC_family_protein_[Bacillus_cereus]	CPTF_Co	203012.6667	68389.04211	33.68708132
UIJ68381.1	VOC_family_protein_[Bacillus_cereus]	CPTF_Cu	215919.3333	43077.2339	19.95061453
UIJ68381.1	VOC_family_protein_[Bacillus_cereus]	CPTF_Fe	221173.3333	21535.22144	9.736807377
UIJ68381.1	VOC_family_protein_[Bacillus_cereus]	CPTF_Mn	149557.1	75282.74593	50.33712604
UIJ68381.1	VOC_family_protein_[Bacillus_cereus]	CPTF_Ni	241353.3333	19630.12818	8.133356978
UIJ68381.1	VOC_family_protein_[Bacillus_cereus]	CPTF_U	207022	29053.9731	14.03424424
UIJ68381.1	VOC_family_protein_[Bacillus_cereus]	CPTF_metals_mix	162421.6667	12780.70046	7.868839623

UIJ68381.1	VOC_family_protein_[Bacillus_cereus]	CPTF_zcontrol	179438.3333	7511.923877	4.186354018
UIJ68387.1	MBL_fold_metallo-hydrolase_[Bacillus_cereus]	CPTF_Al	64792.96667	58012.65097	89.53541403
UIJ68387.1	MBL_fold_metallo-hydrolase_[Bacillus_cereus]	CPTF_Cd	57451.2	83221.77895	144.8564677
UIJ68387.1	MBL_fold_metallo-hydrolase_[Bacillus_cereus]	CPTF_Co	41033.33333	71071.81814	173.2050808
UIJ68387.1	MBL_fold_metallo-hydrolase_[Bacillus_cereus]	CPTF_Cu	0	0	0
UIJ68387.1	MBL_fold_metallo-hydrolase_[Bacillus_cereus]	CPTF_Fe	8252.433333	14293.63382	173.2050808
UIJ68387.1	MBL_fold_metallo-hydrolase_[Bacillus_cereus]	CPTF_Mn	0	0	0
UIJ68387.1	MBL_fold_metallo-hydrolase_[Bacillus_cereus]	CPTF_Ni	0	0	0
UIJ68387.1	MBL_fold_metallo-hydrolase_[Bacillus_cereus]	CPTF_U	0	0	0
UIJ68387.1	MBL_fold_metallo-hydrolase_[Bacillus_cereus]	CPTF_metals_mix	35816.63333	62036.22869	173.2050808
UIJ68387.1	MBL_fold_metallo-hydrolase_[Bacillus_cereus]	CPTF_zcontrol	43659	75619.60621	173.2050808
UIJ68392.1	nitrate_reductase_subunit_alpha_[Bacillus_cereus]	CPTF_Al	45678087.17	3404746.608	7.453785434
UIJ68392.1	nitrate_reductase_subunit_alpha_[Bacillus_cereus]	CPTF_Cd	36336217.63	554080.6328	1.524871516
UIJ68392.1	nitrate_reductase_subunit_alpha_[Bacillus_cereus]	CPTF_Co	39580787.63	3098812.528	7.829082526
UIJ68392.1	nitrate_reductase_subunit_alpha_[Bacillus_cereus]	CPTF_Cu	45685092.3	1680683.238	3.678843915
UIJ68392.1	nitrate_reductase_subunit_alpha_[Bacillus_cereus]	CPTF_Fe	42733287.1	2419915.496	5.6628349
UIJ68392.1	nitrate_reductase_subunit_alpha_[Bacillus_cereus]	CPTF_Mn	47794542.67	7078715.002	14.81071814
UIJ68392.1	nitrate_reductase_subunit_alpha_[Bacillus_cereus]	CPTF_Ni	37290298.63	2526641.283	6.775599487
UIJ68392.1	nitrate_reductase_subunit_alpha_[Bacillus_cereus]	CPTF_U	40433982.03	688787.5306	1.70348676
UIJ68392.1	nitrate_reductase_subunit_alpha_[Bacillus_cereus]	CPTF_metals_mix	34769238.3	1683031.79	4.840577107
UIJ68392.1	nitrate_reductase_subunit_alpha_[Bacillus_cereus]	CPTF_zcontrol	51229284.4	1693881.531	3.306471193
UIJ68393.1	nitrate_reductase_subunit_beta_[Bacillus_cereus]	CPTF_Al	5750995.467	128864.784	2.240738752
UIJ68393.1	nitrate_reductase_subunit_beta_[Bacillus_cereus]	CPTF_Cd	3364230.233	1024196.74	30.44371725
UIJ68393.1	nitrate_reductase_subunit_beta_[Bacillus_cereus]	CPTF_Co	4541032.8	929887.5104	20.47744536
UIJ68393.1	nitrate_reductase_subunit_beta_[Bacillus_cereus]	CPTF_Cu	5245437.467	885676.4702	16.88470172
UIJ68393.1	nitrate_reductase_subunit_beta_[Bacillus_cereus]	CPTF_Fe	4532902.5	993216.3865	21.91126737
UIJ68393.1	nitrate_reductase_subunit_beta_[Bacillus_cereus]	CPTF_Mn	5548515.567	1668864.585	30.0776769
UIJ68393.1	nitrate_reductase_subunit_beta_[Bacillus_cereus]	CPTF_Ni	2809536.433	734369.1852	26.13844677
UIJ68393.1	nitrate_reductase_subunit_beta_[Bacillus_cereus]	CPTF_U	3445414.8	281576.0415	8.172485982
UIJ68393.1	nitrate_reductase_subunit_beta_[Bacillus_cereus]	CPTF_metals_mix	1425803.867	565485.2055	39.66079898
UIJ68393.1	nitrate_reductase_subunit_beta_[Bacillus_cereus]	CPTF_zcontrol	5729326.267	762207.9997	13.30362357
UIJ68394.1	nitrate_reductase_molybdenum_cofactor_assembly_chaperone_[Bacillus_cereus]	CPTF_Al	2145825.667	115349.137	5.375512971
UIJ68394.1	nitrate_reductase_molybdenum_cofactor_assembly_chaperone_[Bacillus_cereus]	CPTF_Cd	1601319.333	73455.31108	4.587174435
UIJ68394.1	nitrate_reductase_molybdenum_cofactor_assembly_chaperone_[Bacillus_cereus]	CPTF_Co	1739145.667	219899.2929	12.6440986
UIJ68394.1	nitrate_reductase_molybdenum_cofactor_assembly_chaperone_[Bacillus_cereus]	CPTF_Cu	2174089	207269.6261	9.533631148
UIJ68394.1	nitrate_reductase_molybdenum_cofactor_assembly_chaperone_[Bacillus_cereus]	CPTF_Fe	1752555	157309.0534	8.97598383
UIJ68394.1	nitrate_reductase_molybdenum_cofactor_assembly_chaperone_[Bacillus_cereus]	CPTF_Mn	2047534.667	91574.14051	4.472409772
UIJ68394.1	nitrate_reductase_molybdenum_cofactor_assembly_chaperone_[Bacillus_cereus]	CPTF_Ni	1546200.4	270448.4055	17.49116127
UIJ68394.1	nitrate_reductase_molybdenum_cofactor_assembly_chaperone_[Bacillus_cereus]	CPTF_U	1913306.867	187982.9017	9.825026239
UIJ68394.1	nitrate_reductase_molybdenum_cofactor_assembly_chaperone_[Bacillus_cereus]	CPTF_metals_mix	1517095	108065.8329	7.123208033
UIJ68394.1	nitrate_reductase_molybdenum_cofactor_assembly_chaperone_[Bacillus_cereus]	CPTF_zcontrol	2405719	57890.81643	2.406383141
UIJ68395.1	respiratory_nitrate_reductase_subunit_gamma_[Bacillus_cereus]	CPTF_Al	1785921	131392.9774	7.357155072
UIJ68395.1	respiratory_nitrate_reductase_subunit_gamma_[Bacillus_cereus]	CPTF_Cd	1364710.033	226622.5276	16.60591057
UIJ68395.1	respiratory_nitrate_reductase_subunit_gamma_[Bacillus_cereus]	CPTF_Co	1851453.333	157948.7228	8.531066917
UIJ68395.1	respiratory_nitrate_reductase_subunit_gamma_[Bacillus_cereus]	CPTF_Cu	2426444	247136.1005	10.18511453
UIJ68395.1	respiratory_nitrate_reductase_subunit_gamma_[Bacillus_cereus]	CPTF_Fe	1608542.667	211212.7257	13.13068842
UIJ68395.1	respiratory_nitrate_reductase_subunit_gamma_[Bacillus_cereus]	CPTF_Mn	1685694.333	336704.4542	19.97422947
UIJ68395.1	respiratory_nitrate_reductase_subunit_gamma_[Bacillus_cereus]	CPTF_Ni	1030987.433	379502.636	36.80962772
UIJ68395.1	respiratory_nitrate_reductase_subunit_gamma_[Bacillus_cereus]	CPTF_U	1336459	264308.3406	19.77676387
UIJ68395.1	respiratory_nitrate_reductase_subunit_gamma_[Bacillus_cereus]	CPTF_metals_mix	1083485.333	108334.614	9.998715315
UIJ68395.1	respiratory_nitrate_reductase_subunit_gamma_[Bacillus_cereus]	CPTF_zcontrol	2152138.333	325170.1062	15.10916381
UIJ68397.1	GTP_3',8-cyclase_MoaA_[Bacillus_cereus]	CPTF_Al	17263.9	29901.95194	173.2050808
UIJ68397.1	GTP_3',8-cyclase_MoaA_[Bacillus_cereus]	CPTF_Cd	0	0	0
UIJ68397.1	GTP_3',8-cyclase_MoaA_[Bacillus_cereus]	CPTF_Co	32866.7	56926.79428	173.2050808
UIJ68397.1	GTP_3',8-cyclase_MoaA_[Bacillus_cereus]	CPTF_Cu	29798.76667	51612.97787	173.2050808
UIJ68397.1	GTP_3',8-cyclase_MoaA_[Bacillus_cereus]	CPTF_Fe	0	0	0
UIJ68397.1	GTP_3',8-cyclase_MoaA_[Bacillus_cereus]	CPTF_Mn	141269.1667	195119.9864	138.1193016
UIJ68397.1	GTP_3',8-cyclase_MoaA_[Bacillus_cereus]	CPTF_Ni	0	0	0
UIJ68397.1	GTP_3',8-cyclase_MoaA_[Bacillus_cereus]	CPTF_U	157320.6333	272487.33	173.2050808
UIJ68397.1	GTP_3',8-cyclase_MoaA_[Bacillus_cereus]	CPTF_metals_mix	38959.66667	67480.12211	173.2050808
UIJ68397.1	GTP_3',8-cyclase_MoaA_[Bacillus_cereus]	CPTF_zcontrol	24947.96667	43211.14581	173.2050808

UIJ68398.1	molybdopterin-synthase_adenylyltransferase_MoeB_[Bacillus_cereus]	CPTF_Al	4953847.933	176808.7058	3.569118556
UIJ68398.1	molybdopterin-synthase_adenylyltransferase_MoeB_[Bacillus_cereus]	CPTF_Cd	4423949.7	97595.51572	2.206072002
UIJ68398.1	molybdopterin-synthase_adenylyltransferase_MoeB_[Bacillus_cereus]	CPTF_Co	4315449.667	355359.8728	8.234596629
UIJ68398.1	molybdopterin-synthase_adenylyltransferase_MoeB_[Bacillus_cereus]	CPTF_Cu	4697125	465173.2068	9.903360179
UIJ68398.1	molybdopterin-synthase_adenylyltransferase_MoeB_[Bacillus_cereus]	CPTF_Fe	4820807.4	157384.524	3.264692218
UIJ68398.1	molybdopterin-synthase_adenylyltransferase_MoeB_[Bacillus_cereus]	CPTF_Mn	4741819.9	16173.51393	0.341082417
UIJ68398.1	molybdopterin-synthase_adenylyltransferase_MoeB_[Bacillus_cereus]	CPTF_Ni	4536677.3	176555.3144	3.891731828
UIJ68398.1	molybdopterin-synthase_adenylyltransferase_MoeB_[Bacillus_cereus]	CPTF_U	5467856.7	710448.3053	12.99317711
UIJ68398.1	molybdopterin-synthase_adenylyltransferase_MoeB_[Bacillus_cereus]	CPTF_metals_mix	3672717.667	524773.411	14.28842232
UIJ68398.1	molybdopterin-synthase_adenylyltransferase_MoeB_[Bacillus_cereus]	CPTF_zcontrol	4780263.8	403301.3025	8.43680013
UIJ68399.1	molybdopterin_molybdotransferase_MoeA_[Bacillus_cereus]	CPTF_Al	332772	10281.7641	3.089732339
UIJ68399.1	molybdopterin_molybdotransferase_MoeA_[Bacillus_cereus]	CPTF_Cd	301006.5667	277039.5678	92.03771562
UIJ68399.1	molybdopterin_molybdotransferase_MoeA_[Bacillus_cereus]	CPTF_Co	375992.7667	135711.6804	36.09422639
UIJ68399.1	molybdopterin_molybdotransferase_MoeA_[Bacillus_cereus]	CPTF_Cu	479381.7667	126086.2149	26.30183784
UIJ68399.1	molybdopterin_molybdotransferase_MoeA_[Bacillus_cereus]	CPTF_Fe	173815.9667	188126.7898	108.2333191
UIJ68399.1	molybdopterin_molybdotransferase_MoeA_[Bacillus_cereus]	CPTF_Mn	460760.5333	461025.1106	100.0574219
UIJ68399.1	molybdopterin_molybdotransferase_MoeA_[Bacillus_cereus]	CPTF_Ni	209711.7333	94125.77376	44.88340841
UIJ68399.1	molybdopterin_molybdotransferase_MoeA_[Bacillus_cereus]	CPTF_U	157774.3333	150058.196	95.10938365
UIJ68399.1	molybdopterin_molybdotransferase_MoeA_[Bacillus_cereus]	CPTF_metals_mix	1196648.7	189847.8341	15.86495971
UIJ68399.1	molybdopterin_molybdotransferase_MoeA_[Bacillus_cereus]	CPTF_zcontrol	206118.2667	147607.7973	71.61315669
UIJ68402.1	nitrate_transporter_NarK_[Bacillus_cereus]	CPTF_Al	4286666.667	635164.0208	14.81720111
UIJ68402.1	nitrate_transporter_NarK_[Bacillus_cereus]	CPTF_Cd	2996666.667	106926.7662	3.568190196
UIJ68402.1	nitrate_transporter_NarK_[Bacillus_cereus]	CPTF_Co	3913333.333	205020.3242	5.239020209
UIJ68402.1	nitrate_transporter_NarK_[Bacillus_cereus]	CPTF_Cu	4113333.333	695005.9952	16.89641804
UIJ68402.1	nitrate_transporter_NarK_[Bacillus_cereus]	CPTF_Fe	3960000	600000	15.15151515
UIJ68402.1	nitrate_transporter_NarK_[Bacillus_cereus]	CPTF_Mn	3910000	832105.7625	21.2814773
UIJ68402.1	nitrate_transporter_NarK_[Bacillus_cereus]	CPTF_Ni	3640000	283548.9376	7.789805977
UIJ68402.1	nitrate_transporter_NarK_[Bacillus_cereus]	CPTF_U	4686666.667	493186.9152	10.52319165
UIJ68402.1	nitrate_transporter_NarK_[Bacillus_cereus]	CPTF_metals_mix	2322118.667	104231.0317	4.488617797
UIJ68402.1	nitrate_transporter_NarK_[Bacillus_cereus]	CPTF_zcontrol	4903333.333	60277.13773	1.229309403
UIJ68404.1	precorrin-2_dehydrogenase_[Bacillus_cereus]	CPTF_Al	453907	94773.06496	20.8794015
UIJ68404.1	precorrin-2_dehydrogenase_[Bacillus_cereus]	CPTF_Cd	482109.6667	30529.35778	6.332450869
UIJ68404.1	precorrin-2_dehydrogenase_[Bacillus_cereus]	CPTF_Co	390675.6667	57592.9884	14.74189291
UIJ68404.1	precorrin-2_dehydrogenase_[Bacillus_cereus]	CPTF_Cu	296032	30300.60283	10.2355836
UIJ68404.1	precorrin-2_dehydrogenase_[Bacillus_cereus]	CPTF_Fe	459813	166248.1977	36.15561059
UIJ68404.1	precorrin-2_dehydrogenase_[Bacillus_cereus]	CPTF_Mn	475768.3333	228827.7367	48.09646222
UIJ68404.1	precorrin-2_dehydrogenase_[Bacillus_cereus]	CPTF_Ni	66409	115023.7621	173.2050808
UIJ68404.1	precorrin-2_dehydrogenase_[Bacillus_cereus]	CPTF_U	153123.7333	70898.48868	46.30143684
UIJ68404.1	precorrin-2_dehydrogenase_[Bacillus_cereus]	CPTF_metals_mix	417267	29668.02857	7.110082649
UIJ68404.1	precorrin-2_dehydrogenase_[Bacillus_cereus]	CPTF_zcontrol	471084.3333	216831.0353	46.02807183
UIJ68405.1	sirohydrochlorin_chelatase_[Bacillus_cereus]	CPTF_Al	1306717.967	593448.336	45.41518148
UIJ68405.1	sirohydrochlorin_chelatase_[Bacillus_cereus]	CPTF_Cd	758305	438375.1307	57.80986947
UIJ68405.1	sirohydrochlorin_chelatase_[Bacillus_cereus]	CPTF_Co	1166519.1	276416.8797	23.69587259
UIJ68405.1	sirohydrochlorin_chelatase_[Bacillus_cereus]	CPTF_Cu	1701877	206660.7622	12.143108
UIJ68405.1	sirohydrochlorin_chelatase_[Bacillus_cereus]	CPTF_Fe	1143757.667	266958.8904	23.34051156
UIJ68405.1	sirohydrochlorin_chelatase_[Bacillus_cereus]	CPTF_Mn	706396	85869.95914	12.15606531
UIJ68405.1	sirohydrochlorin_chelatase_[Bacillus_cereus]	CPTF_Ni	1463080.967	113330.3958	7.746009851
UIJ68405.1	sirohydrochlorin_chelatase_[Bacillus_cereus]	CPTF_U	1100869.333	113947.8154	10.35071211
UIJ68405.1	sirohydrochlorin_chelatase_[Bacillus_cereus]	CPTF_metals_mix	1066652.167	150731.7413	14.13129284
UIJ68405.1	sirohydrochlorin_chelatase_[Bacillus_cereus]	CPTF_zcontrol	1109518	349592.5727	31.50850844
UIJ68407.1	nitrite_reductase_small_subunit_NirD_[Bacillus_cereus]	CPTF_Al	0	0	0
UIJ68407.1	nitrite_reductase_small_subunit_NirD_[Bacillus_cereus]	CPTF_Cd	0	0	0
UIJ68407.1	nitrite_reductase_small_subunit_NirD_[Bacillus_cereus]	CPTF_Co	25542.73333	44241.3119	173.2050808
UIJ68407.1	nitrite_reductase_small_subunit_NirD_[Bacillus_cereus]	CPTF_Cu	0	0	0
UIJ68407.1	nitrite_reductase_small_subunit_NirD_[Bacillus_cereus]	CPTF_Fe	0	0	0
UIJ68407.1	nitrite_reductase_small_subunit_NirD_[Bacillus_cereus]	CPTF_Mn	0	0	0
UIJ68407.1	nitrite_reductase_small_subunit_NirD_[Bacillus_cereus]	CPTF_Ni	0	0	0
UIJ68407.1	nitrite_reductase_small_subunit_NirD_[Bacillus_cereus]	CPTF_U	0	0	0
UIJ68407.1	nitrite_reductase_small_subunit_NirD_[Bacillus_cereus]	CPTF_metals_mix	0	0	0
UIJ68407.1	nitrite_reductase_small_subunit_NirD_[Bacillus_cereus]	CPTF_zcontrol	0	0	0
UIJ68408.1	NADPH-nitrite_reductase_large_subunit_[Bacillus_cereus]	CPTF_Al	9660387.5	1133375.368	11.73219364

UIJ68408.1	NADPH-nitrite_reductase_large_subunit_[Bacillus_cereus]	CPTF_Cd	9198683.927	1282311.503	13.94016267
UIJ68408.1	NADPH-nitrite_reductase_large_subunit_[Bacillus_cereus]	CPTF_Co	10796758.9	994918.0287	9.214969399
UIJ68408.1	NADPH-nitrite_reductase_large_subunit_[Bacillus_cereus]	CPTF_Cu	9971700.1	774353.2078	7.765508389
UIJ68408.1	NADPH-nitrite_reductase_large_subunit_[Bacillus_cereus]	CPTF_Fe	7509469.333	1915538.381	25.50830553
UIJ68408.1	NADPH-nitrite_reductase_large_subunit_[Bacillus_cereus]	CPTF_Mn	8634602.367	2421408.549	28.04308116
UIJ68408.1	NADPH-nitrite_reductase_large_subunit_[Bacillus_cereus]	CPTF_Ni	9648376.667	1681352.879	17.42627736
UIJ68408.1	NADPH-nitrite_reductase_large_subunit_[Bacillus_cereus]	CPTF_U	10937328.2	1505880.345	13.76826513
UIJ68408.1	NADPH-nitrite_reductase_large_subunit_[Bacillus_cereus]	CPTF_metals_mix	13384435.83	481503.597	3.597488926
UIJ68408.1	NADPH-nitrite_reductase_large_subunit_[Bacillus_cereus]	CPTF_zcontrol	13263567.93	1247143.616	9.40277625
UIJ68409.1	iron-sulfur_cluster_repair_di-iron_protein_[Bacillus_cereus]	CPTF_Al	376102.1	136613.4128	36.3234911
UIJ68409.1	iron-sulfur_cluster_repair_di-iron_protein_[Bacillus_cereus]	CPTF_Cd	506926.6667	117423.4177	23.16378787
UIJ68409.1	iron-sulfur_cluster_repair_di-iron_protein_[Bacillus_cereus]	CPTF_Co	285684.6333	21064.45329	7.37332388
UIJ68409.1	iron-sulfur_cluster_repair_di-iron_protein_[Bacillus_cereus]	CPTF_Cu	705761.6667	372365.3297	52.76077567
UIJ68409.1	iron-sulfur_cluster_repair_di-iron_protein_[Bacillus_cereus]	CPTF_Fe	398294.6667	30789.46807	7.730323967
UIJ68409.1	iron-sulfur_cluster_repair_di-iron_protein_[Bacillus_cereus]	CPTF_Mn	282924.8	80037.33868	28.28926226
UIJ68409.1	iron-sulfur_cluster_repair_di-iron_protein_[Bacillus_cereus]	CPTF_Ni	358491.1667	105381.1887	29.39575601
UIJ68409.1	iron-sulfur_cluster_repair_di-iron_protein_[Bacillus_cereus]	CPTF_U	331625.9333	94544.53678	28.50939184
UIJ68409.1	iron-sulfur_cluster_repair_di-iron_protein_[Bacillus_cereus]	CPTF_metals_mix	80872.23333	87095.67289	107.6953972
UIJ68409.1	iron-sulfur_cluster_repair_di-iron_protein_[Bacillus_cereus]	CPTF_zcontrol	218755	72106.37462	32.96216069
UIJ68412.1	arylamine_N-acetyltransferase_[Bacillus_cereus]	CPTF_Al	911855.3333	131795.8593	14.45359308
UIJ68412.1	arylamine_N-acetyltransferase_[Bacillus_cereus]	CPTF_Cd	934922.3333	145098.2864	15.51982248
UIJ68412.1	arylamine_N-acetyltransferase_[Bacillus_cereus]	CPTF_Co	737881.3333	106748.4964	14.46689212
UIJ68412.1	arylamine_N-acetyltransferase_[Bacillus_cereus]	CPTF_Cu	750839.6667	56837.00155	7.569792071
UIJ68412.1	arylamine_N-acetyltransferase_[Bacillus_cereus]	CPTF_Fe	838396	167248.8734	19.94867263
UIJ68412.1	arylamine_N-acetyltransferase_[Bacillus_cereus]	CPTF_Mn	746479.3333	204706.5244	27.4229326
UIJ68412.1	arylamine_N-acetyltransferase_[Bacillus_cereus]	CPTF_Ni	875994.6667	59907.10085	6.838751779
UIJ68412.1	arylamine_N-acetyltransferase_[Bacillus_cereus]	CPTF_U	1086367.333	277044.4178	25.50190983
UIJ68412.1	arylamine_N-acetyltransferase_[Bacillus_cereus]	CPTF_metals_mix	282378.3333	37864.13198	13.40900753
UIJ68412.1	arylamine_N-acetyltransferase_[Bacillus_cereus]	CPTF_zcontrol	859800	139019.8588	16.16886006
UIJ68414.1	stage_V_sporulation_protein_S_[Bacillus_cereus]	CPTF_Al	661105	312515.2626	47.27165315
UIJ68414.1	stage_V_sporulation_protein_S_[Bacillus_cereus]	CPTF_Cd	790758.3333	188444.9166	23.83091124
UIJ68414.1	stage_V_sporulation_protein_S_[Bacillus_cereus]	CPTF_Co	589890	25120.82899	4.258561594
UIJ68414.1	stage_V_sporulation_protein_S_[Bacillus_cereus]	CPTF_Cu	640201.6667	97376.42197	15.21027311
UIJ68414.1	stage_V_sporulation_protein_S_[Bacillus_cereus]	CPTF_Fe	679788.2667	114510.2986	16.84499486
UIJ68414.1	stage_V_sporulation_protein_S_[Bacillus_cereus]	CPTF_Mn	602505.6667	24509.50208	4.067928891
UIJ68414.1	stage_V_sporulation_protein_S_[Bacillus_cereus]	CPTF_Ni	745575.6667	72556.06657	9.731549703
UIJ68414.1	stage_V_sporulation_protein_S_[Bacillus_cereus]	CPTF_U	384485	217181.9992	56.48646871
UIJ68414.1	stage_V_sporulation_protein_S_[Bacillus_cereus]	CPTF_metals_mix	390013.6667	103256.5096	26.47510033
UIJ68414.1	stage_V_sporulation_protein_S_[Bacillus_cereus]	CPTF_zcontrol	487851.3333	27352.80171	5.606790397
UIJ68423.1	ABC-F_family_ATP-binding_cassette_domain-containing_protein_[Bacillus_cereus]	CPTF_Al	225205.2667	52591.91287	23.35287875
UIJ68423.1	ABC-F_family_ATP-binding_cassette_domain-containing_protein_[Bacillus_cereus]	CPTF_Cd	384397.8667	100453.9834	26.13281502
UIJ68423.1	ABC-F_family_ATP-binding_cassette_domain-containing_protein_[Bacillus_cereus]	CPTF_Co	413072.4	62313.45423	15.08535894
UIJ68423.1	ABC-F_family_ATP-binding_cassette_domain-containing_protein_[Bacillus_cereus]	CPTF_Cu	421735.3667	86769.17602	20.57431813
UIJ68423.1	ABC-F_family_ATP-binding_cassette_domain-containing_protein_[Bacillus_cereus]	CPTF_Fe	233154.1333	26592.7894	11.4056693
UIJ68423.1	ABC-F_family_ATP-binding_cassette_domain-containing_protein_[Bacillus_cereus]	CPTF_Mn	168475.3	64385.64679	38.21666843
UIJ68423.1	ABC-F_family_ATP-binding_cassette_domain-containing_protein_[Bacillus_cereus]	CPTF_Ni	453822.2667	222771.1392	49.08774989
UIJ68423.1	ABC-F_family_ATP-binding_cassette_domain-containing_protein_[Bacillus_cereus]	CPTF_U	443696	398639.1629	89.84511082
UIJ68423.1	ABC-F_family_ATP-binding_cassette_domain-containing_protein_[Bacillus_cereus]	CPTF_metals_mix	276466.5667	49825.50349	18.02225278
UIJ68423.1	ABC-F_family_ATP-binding_cassette_domain-containing_protein_[Bacillus_cereus]	CPTF_zcontrol	202423.4667	96068.94563	47.45939155
UIJ68425.1	DUF4318_domain-containing_protein_[Bacillus_cereus]	CPTF_Al	0	0	0
UIJ68425.1	DUF4318_domain-containing_protein_[Bacillus_cereus]	CPTF_Cd	0	0	0
UIJ68425.1	DUF4318_domain-containing_protein_[Bacillus_cereus]	CPTF_Co	0	0	0
UIJ68425.1	DUF4318_domain-containing_protein_[Bacillus_cereus]	CPTF_Cu	0	0	0
UIJ68425.1	DUF4318_domain-containing_protein_[Bacillus_cereus]	CPTF_Fe	0	0	0
UIJ68425.1	DUF4318_domain-containing_protein_[Bacillus_cereus]	CPTF_Mn	0	0	0
UIJ68425.1	DUF4318_domain-containing_protein_[Bacillus_cereus]	CPTF_Ni	0	0	0
UIJ68425.1	DUF4318_domain-containing_protein_[Bacillus_cereus]	CPTF_U	0	0	0
UIJ68425.1	DUF4318_domain-containing_protein_[Bacillus_cereus]	CPTF_metals_mix	13663.26667	23665.47206	173.2050808
UIJ68425.1	DUF4318_domain-containing_protein_[Bacillus_cereus]	CPTF_zcontrol	0	0	0
UIJ68429.1	BrxA/BrxB_family_bacilliredoxin_[Bacillus_cereus]	CPTF_Al	1800133.667	293102.0298	16.28223699
UIJ68429.1	BrxA/BrxB_family_bacilliredoxin_[Bacillus_cereus]	CPTF_Cd	2089886.167	71506.44586	3.421547403

UIJ68429.1	BrxA/BrxB_family_bacilliredoxin_[Bacillus_cereus]	CPTF_Co	1906292.767	391074.3357	20.51491474
UIJ68429.1	BrxA/BrxB_family_bacilliredoxin_[Bacillus_cereus]	CPTF_Cu	1686917.233	42586.16834	2.524496608
UIJ68429.1	BrxA/BrxB_family_bacilliredoxin_[Bacillus_cereus]	CPTF_Fe	1846405.967	349846.4288	18.94742733
UIJ68429.1	BrxA/BrxB_family_bacilliredoxin_[Bacillus_cereus]	CPTF_Mn	2149766.1	542166.2757	25.21977976
UIJ68429.1	BrxA/BrxB_family_bacilliredoxin_[Bacillus_cereus]	CPTF_Ni	1391762.133	505562.0243	36.32531826
UIJ68429.1	BrxA/BrxB_family_bacilliredoxin_[Bacillus_cereus]	CPTF_U	1664031	495358.9291	29.76861183
UIJ68429.1	BrxA/BrxB_family_bacilliredoxin_[Bacillus_cereus]	CPTF_metals_mix	3283733.433	228947.6017	6.972173789
UIJ68429.1	BrxA/BrxB_family_bacilliredoxin_[Bacillus_cereus]	CPTF_zcontrol	1755114.267	257239.3882	14.65656072
UIJ68431.1	arginine--tRNA_ligase_[Bacillus_cereus]	CPTF_Al	410450.3667	315943.9349	76.97494277
UIJ68431.1	arginine--tRNA_ligase_[Bacillus_cereus]	CPTF_Cd	372167.7	175191.318	47.0732194
UIJ68431.1	arginine--tRNA_ligase_[Bacillus_cereus]	CPTF_Co	277271.1667	210604.5466	75.95616562
UIJ68431.1	arginine--tRNA_ligase_[Bacillus_cereus]	CPTF_Cu	67096.8	15130.89289	22.5508413
UIJ68431.1	arginine--tRNA_ligase_[Bacillus_cereus]	CPTF_Fe	345480.5333	374880.3617	108.5098365
UIJ68431.1	arginine--tRNA_ligase_[Bacillus_cereus]	CPTF_Mn	490675.9333	391152.7943	79.71713462
UIJ68431.1	arginine--tRNA_ligase_[Bacillus_cereus]	CPTF_Ni	471561.8	540587.2894	114.6376338
UIJ68431.1	arginine--tRNA_ligase_[Bacillus_cereus]	CPTF_U	406432.3	636172.3107	156.5260218
UIJ68431.1	arginine--tRNA_ligase_[Bacillus_cereus]	CPTF_metals_mix	1424862.1	249074.8059	17.48062538
UIJ68431.1	arginine--tRNA_ligase_[Bacillus_cereus]	CPTF_zcontrol	600309.2	295103.5669	49.15859475
UIJ68435.1	isoleucine--tRNA_ligase_[Bacillus_cereus]	CPTF_Al	133878.6667	92407.89374	69.02361372
UIJ68435.1	isoleucine--tRNA_ligase_[Bacillus_cereus]	CPTF_Cd	185891.3667	110013.3196	59.181511
UIJ68435.1	isoleucine--tRNA_ligase_[Bacillus_cereus]	CPTF_Co	335763.8	97772.98846	29.11957408
UIJ68435.1	isoleucine--tRNA_ligase_[Bacillus_cereus]	CPTF_Cu	161057.3	141137.8627	87.63208047
UIJ68435.1	isoleucine--tRNA_ligase_[Bacillus_cereus]	CPTF_Fe	239496.1667	109958.8514	45.91257259
UIJ68435.1	isoleucine--tRNA_ligase_[Bacillus_cereus]	CPTF_Mn	159635.3333	90886.8755	56.9340594
UIJ68435.1	isoleucine--tRNA_ligase_[Bacillus_cereus]	CPTF_Ni	88846.4	73400.41683	82.61495888
UIJ68435.1	isoleucine--tRNA_ligase_[Bacillus_cereus]	CPTF_U	149541.6667	133179.0636	89.05816455
UIJ68435.1	isoleucine--tRNA_ligase_[Bacillus_cereus]	CPTF_metals_mix	247929.7	55951.26628	22.56739159
UIJ68435.1	isoleucine--tRNA_ligase_[Bacillus_cereus]	CPTF_zcontrol	149752.3333	22120.50416	14.77139198
UIJ68437.1	aspartate--tRNA(Asn)_ligase_[Bacillus_cereus]	CPTF_Al	26540.53333	45969.55219	173.2050808
UIJ68437.1	aspartate--tRNA(Asn)_ligase_[Bacillus_cereus]	CPTF_Cd	65054.76667	57486.61246	88.36648781
UIJ68437.1	aspartate--tRNA(Asn)_ligase_[Bacillus_cereus]	CPTF_Co	150221.9333	29328.71034	19.52358733
UIJ68437.1	aspartate--tRNA(Asn)_ligase_[Bacillus_cereus]	CPTF_Cu	15794.56667	27356.99195	173.2050808
UIJ68437.1	aspartate--tRNA(Asn)_ligase_[Bacillus_cereus]	CPTF_Fe	38724.46667	38756.69009	100.083212
UIJ68437.1	aspartate--tRNA(Asn)_ligase_[Bacillus_cereus]	CPTF_Mn	22567.1	39087.36378	173.2050808
UIJ68437.1	aspartate--tRNA(Asn)_ligase_[Bacillus_cereus]	CPTF_Ni	103680	90050.80691	86.85455913
UIJ68437.1	aspartate--tRNA(Asn)_ligase_[Bacillus_cereus]	CPTF_U	35997.33333	62349.21027	173.2050808
UIJ68437.1	aspartate--tRNA(Asn)_ligase_[Bacillus_cereus]	CPTF_metals_mix	43401.93333	37592.62681	86.61509736
UIJ68437.1	aspartate--tRNA(Asn)_ligase_[Bacillus_cereus]	CPTF_zcontrol	0	0	0
UIJ68448.1	HAD_family_hydrolase_[Bacillus_cereus]	CPTF_Al	62737.36667	54348.53361	86.62864971
UIJ68448.1	HAD_family_hydrolase_[Bacillus_cereus]	CPTF_Cd	75252.46667	45130.30805	59.97186544
UIJ68448.1	HAD_family_hydrolase_[Bacillus_cereus]	CPTF_Co	66453.76667	57812.04246	86.99588504
UIJ68448.1	HAD_family_hydrolase_[Bacillus_cereus]	CPTF_Cu	39756	68859.41191	173.2050808
UIJ68448.1	HAD_family_hydrolase_[Bacillus_cereus]	CPTF_Fe	69353.3	66601.06188	96.03156862
UIJ68448.1	HAD_family_hydrolase_[Bacillus_cereus]	CPTF_Mn	44272.53333	23828.40566	53.8220966
UIJ68448.1	HAD_family_hydrolase_[Bacillus_cereus]	CPTF_Ni	109778.3333	95109.14337	86.63744519
UIJ68448.1	HAD_family_hydrolase_[Bacillus_cereus]	CPTF_U	72991.73333	11984.68519	16.41923631
UIJ68448.1	HAD_family_hydrolase_[Bacillus_cereus]	CPTF_metals_mix	228531.4333	82084.06841	35.91806484
UIJ68448.1	HAD_family_hydrolase_[Bacillus_cereus]	CPTF_zcontrol	20504.93333	35515.58634	173.2050808
UIJ68464.1	serine_hydrolase_[Bacillus_cereus]	CPTF_Al	6102185.667	330872.1209	5.422190326
UIJ68464.1	serine_hydrolase_[Bacillus_cereus]	CPTF_Cd	6112415	635816.3867	10.40204873
UIJ68464.1	serine_hydrolase_[Bacillus_cereus]	CPTF_Co	5331387.333	675806.311	12.67599348
UIJ68464.1	serine_hydrolase_[Bacillus_cereus]	CPTF_Cu	4919200.2	297308.7431	6.043843125
UIJ68464.1	serine_hydrolase_[Bacillus_cereus]	CPTF_Fe	5981813	323999.8207	5.416415068
UIJ68464.1	serine_hydrolase_[Bacillus_cereus]	CPTF_Mn	5566665.5	577808.9419	10.37980353
UIJ68464.1	serine_hydrolase_[Bacillus_cereus]	CPTF_Ni	5478949.333	354245.6561	6.465576419
UIJ68464.1	serine_hydrolase_[Bacillus_cereus]	CPTF_U	4439006.433	475628.3566	10.71474808
UIJ68464.1	serine_hydrolase_[Bacillus_cereus]	CPTF_metals_mix	5434258	261910.617	4.819620581
UIJ68464.1	serine_hydrolase_[Bacillus_cereus]	CPTF_zcontrol	5262844.667	206400.9865	3.921852146
UIJ68465.1	thymidylate_synthase_[Bacillus_cereus]	CPTF_Al	85509.63333	53047.8681	62.0373004
UIJ68465.1	thymidylate_synthase_[Bacillus_cereus]	CPTF_Cd	214584.2667	139019.3623	64.78544047
UIJ68465.1	thymidylate_synthase_[Bacillus_cereus]	CPTF_Co	113487.3	6673.744563	5.880609163

UIJ68465.1	thymidylate_synthase_[Bacillus_cereus]	CPTF_Cu	177142.1333	65444.92541	36.94486692
UIJ68465.1	thymidylate_synthase_[Bacillus_cereus]	CPTF_Fe	80876.16667	61537.74004	76.0888437
UIJ68465.1	thymidylate_synthase_[Bacillus_cereus]	CPTF_Mn	84113.43333	11836.97616	14.07263464
UIJ68465.1	thymidylate_synthase_[Bacillus_cereus]	CPTF_Ni	591534.8	806958.37	136.417734
UIJ68465.1	thymidylate_synthase_[Bacillus_cereus]	CPTF_U	491288.6	769723.6955	156.6744466
UIJ68465.1	thymidylate_synthase_[Bacillus_cereus]	CPTF_metals_mix	579237.6333	34940.19229	6.032099828
UIJ68465.1	thymidylate_synthase_[Bacillus_cereus]	CPTF_zcontrol	108592.6333	22179.34443	20.42435454
UIJ68467.1	penicillin-binding_protein_3_[Bacillus_cereus]	CPTF_Al	642800.5667	112517.2967	17.5042311
UIJ68467.1	penicillin-binding_protein_3_[Bacillus_cereus]	CPTF_Cd	1056493.333	398678.4924	37.73601591
UIJ68467.1	penicillin-binding_protein_3_[Bacillus_cereus]	CPTF_Co	767252.3667	99134.81925	12.9207577
UIJ68467.1	penicillin-binding_protein_3_[Bacillus_cereus]	CPTF_Cu	789389.9	88442.14719	11.20386101
UIJ68467.1	penicillin-binding_protein_3_[Bacillus_cereus]	CPTF_Fe	696359.3333	256253.5821	36.79904467
UIJ68467.1	penicillin-binding_protein_3_[Bacillus_cereus]	CPTF_Mn	863821.6667	434311.0295	50.27785783
UIJ68467.1	penicillin-binding_protein_3_[Bacillus_cereus]	CPTF_Ni	625108.0333	303069.2978	48.48270725
UIJ68467.1	penicillin-binding_protein_3_[Bacillus_cereus]	CPTF_U	371624.0667	9976.41523	2.684544981
UIJ68467.1	penicillin-binding_protein_3_[Bacillus_cereus]	CPTF_metals_mix	1573396.2	234862.4647	14.92710257
UIJ68467.1	penicillin-binding_protein_3_[Bacillus_cereus]	CPTF_zcontrol	528812.1	120818.8481	22.84721702
UIJ68468.1	FMN-dependent_NADH-azoreductase_[Bacillus_cereus]	CPTF_Al	29254.96667	50671.08864	173.2050808
UIJ68468.1	FMN-dependent_NADH-azoreductase_[Bacillus_cereus]	CPTF_Cd	36879.1	34479.35886	93.49295091
UIJ68468.1	FMN-dependent_NADH-azoreductase_[Bacillus_cereus]	CPTF_Co	0	0	0
UIJ68468.1	FMN-dependent_NADH-azoreductase_[Bacillus_cereus]	CPTF_Cu	23057.9	39937.45432	173.2050808
UIJ68468.1	FMN-dependent_NADH-azoreductase_[Bacillus_cereus]	CPTF_Fe	35570.33333	61609.62458	173.2050808
UIJ68468.1	FMN-dependent_NADH-azoreductase_[Bacillus_cereus]	CPTF_Mn	0	0	0
UIJ68468.1	FMN-dependent_NADH-azoreductase_[Bacillus_cereus]	CPTF_Ni	0	0	0
UIJ68468.1	FMN-dependent_NADH-azoreductase_[Bacillus_cereus]	CPTF_U	0	0	0
UIJ68468.1	FMN-dependent_NADH-azoreductase_[Bacillus_cereus]	CPTF_metals_mix	0	0	0
UIJ68468.1	FMN-dependent_NADH-azoreductase_[Bacillus_cereus]	CPTF_zcontrol	0	0	0
UIJ68471.1	twin-arginine_translocase_TatA/TatE_family_subunit_[Bacillus_cereus]	CPTF_Al	89131.66667	44284.08833	49.68390022
UIJ68471.1	twin-arginine_translocase_TatA/TatE_family_subunit_[Bacillus_cereus]	CPTF_Cd	105621.6	48675.97094	46.08524293
UIJ68471.1	twin-arginine_translocase_TatA/TatE_family_subunit_[Bacillus_cereus]	CPTF_Co	102270.3	28475.36075	27.84323577
UIJ68471.1	twin-arginine_translocase_TatA/TatE_family_subunit_[Bacillus_cereus]	CPTF_Cu	16884.53333	29244.8696	173.2050808
UIJ68471.1	twin-arginine_translocase_TatA/TatE_family_subunit_[Bacillus_cereus]	CPTF_Fe	76865.8	14600.79369	18.9951756
UIJ68471.1	twin-arginine_translocase_TatA/TatE_family_subunit_[Bacillus_cereus]	CPTF_Mn	172202.7667	75758.89183	43.99400387
UIJ68471.1	twin-arginine_translocase_TatA/TatE_family_subunit_[Bacillus_cereus]	CPTF_Ni	22560.76667	39076.39412	173.2050808
UIJ68471.1	twin-arginine_translocase_TatA/TatE_family_subunit_[Bacillus_cereus]	CPTF_U	191155.3333	28519.01549	14.91928841
UIJ68471.1	twin-arginine_translocase_TatA/TatE_family_subunit_[Bacillus_cereus]	CPTF_metals_mix	0	0	0
UIJ68471.1	twin-arginine_translocase_TatA/TatE_family_subunit_[Bacillus_cereus]	CPTF_zcontrol	136927.6	71435.19594	52.17004895
UIJ68474.1	DegV_family_protein_[Bacillus_cereus]	CPTF_Al	0	0	0
UIJ68474.1	DegV_family_protein_[Bacillus_cereus]	CPTF_Cd	284947.3333	250219.6353	87.81259062
UIJ68474.1	DegV_family_protein_[Bacillus_cereus]	CPTF_Co	0	0	0
UIJ68474.1	DegV_family_protein_[Bacillus_cereus]	CPTF_Cu	0	0	0
UIJ68474.1	DegV_family_protein_[Bacillus_cereus]	CPTF_Fe	114336.3333	198036.3385	173.2050808
UIJ68474.1	DegV_family_protein_[Bacillus_cereus]	CPTF_Mn	85001.66667	147227.2054	173.2050808
UIJ68474.1	DegV_family_protein_[Bacillus_cereus]	CPTF_Ni	0	0	0
UIJ68474.1	DegV_family_protein_[Bacillus_cereus]	CPTF_U	0	0	0
UIJ68474.1	DegV_family_protein_[Bacillus_cereus]	CPTF_metals_mix	494161.4333	136011.9512	27.52378919
UIJ68474.1	DegV_family_protein_[Bacillus_cereus]	CPTF_zcontrol	0	0	0
UIJ68476.1	SCO_family_protein_[Bacillus_cereus]	CPTF_Al	637697.6667	27230.93671	4.270195445
UIJ68476.1	SCO_family_protein_[Bacillus_cereus]	CPTF_Cd	650114.3333	23802.1563	3.661226199
UIJ68476.1	SCO_family_protein_[Bacillus_cereus]	CPTF_Co	536177	82504.68064	15.38758295
UIJ68476.1	SCO_family_protein_[Bacillus_cereus]	CPTF_Cu	473531	41457.23943	8.754915608
UIJ68476.1	SCO_family_protein_[Bacillus_cereus]	CPTF_Fe	665059	14223.72514	2.138716286
UIJ68476.1	SCO_family_protein_[Bacillus_cereus]	CPTF_Mn	496558	74659.96209	15.03549678
UIJ68476.1	SCO_family_protein_[Bacillus_cereus]	CPTF_Ni	587687.3333	26495.55203	4.50844361
UIJ68476.1	SCO_family_protein_[Bacillus_cereus]	CPTF_U	645430.3333	78609.28202	12.17935972
UIJ68476.1	SCO_family_protein_[Bacillus_cereus]	CPTF_metals_mix	260387	18657.89846	7.165449297
UIJ68476.1	SCO_family_protein_[Bacillus_cereus]	CPTF_zcontrol	638912.6667	78797.01996	12.3329876
UIJ68478.1	asparagine_synthase_(glutamine-hydrolyzing)_[Bacillus_cereus]	CPTF_Al	1983958.667	685104.2796	34.53218512
UIJ68478.1	asparagine_synthase_(glutamine-hydrolyzing)_[Bacillus_cereus]	CPTF_Cd	2458253.9	315364.8224	12.82881408
UIJ68478.1	asparagine_synthase_(glutamine-hydrolyzing)_[Bacillus_cereus]	CPTF_Co	2311963.267	173473.8416	7.503313054
UIJ68478.1	asparagine_synthase_(glutamine-hydrolyzing)_[Bacillus_cereus]	CPTF_Cu	2679360.233	315258.03	11.76616814

UJJ68478.1	asparagine_synthase_(glutamine-hydrolyzing)_[Bacillus_cereus]	CPTF_Fe	2159810.033	557171.7015	25.79725499
UJJ68478.1	asparagine_synthase_(glutamine-hydrolyzing)_[Bacillus_cereus]	CPTF_Mn	1762031.8	547221.9035	31.05630122
UJJ68478.1	asparagine_synthase_(glutamine-hydrolyzing)_[Bacillus_cereus]	CPTF_Ni	2402363.633	342484.9459	14.25616594
UJJ68478.1	asparagine_synthase_(glutamine-hydrolyzing)_[Bacillus_cereus]	CPTF_U	2052345.333	986372.3192	48.06073828
UJJ68478.1	asparagine_synthase_(glutamine-hydrolyzing)_[Bacillus_cereus]	CPTF_metals_mix	2316924.167	604414.8588	26.08695043
UJJ68478.1	asparagine_synthase_(glutamine-hydrolyzing)_[Bacillus_cereus]	CPTF_zcontrol	1501544.3	169296.4734	11.27482376
UJJ68481.1	iron-siderophore_ABC_transporter_substrate-binding_protein_[Bacillus_cereus]	CPTF_Al	0	0	0
UJJ68481.1	iron-siderophore_ABC_transporter_substrate-binding_protein_[Bacillus_cereus]	CPTF_Cd	0	0	0
UJJ68481.1	iron-siderophore_ABC_transporter_substrate-binding_protein_[Bacillus_cereus]	CPTF_Co	0	0	0
UJJ68481.1	iron-siderophore_ABC_transporter_substrate-binding_protein_[Bacillus_cereus]	CPTF_Cu	0	0	0
UJJ68481.1	iron-siderophore_ABC_transporter_substrate-binding_protein_[Bacillus_cereus]	CPTF_Fe	0	0	0
UJJ68481.1	iron-siderophore_ABC_transporter_substrate-binding_protein_[Bacillus_cereus]	CPTF_Mn	0	0	0
UJJ68481.1	iron-siderophore_ABC_transporter_substrate-binding_protein_[Bacillus_cereus]	CPTF_Ni	0	0	0
UJJ68481.1	iron-siderophore_ABC_transporter_substrate-binding_protein_[Bacillus_cereus]	CPTF_U	0	0	0
UJJ68481.1	iron-siderophore_ABC_transporter_substrate-binding_protein_[Bacillus_cereus]	CPTF_metals_mix	26889	31802.00242	118.2714211
UJJ68481.1	iron-siderophore_ABC_transporter_substrate-binding_protein_[Bacillus_cereus]	CPTF_zcontrol	0	0	0
UJJ68482.1	D-amino-acid_transaminase_[Bacillus_cereus]	CPTF_Al	747284.0333	138458.3163	18.52820482
UJJ68482.1	D-amino-acid_transaminase_[Bacillus_cereus]	CPTF_Cd	579254.4667	14570.01369	2.515304505
UJJ68482.1	D-amino-acid_transaminase_[Bacillus_cereus]	CPTF_Co	580507.3	154347.7863	26.58843159
UJJ68482.1	D-amino-acid_transaminase_[Bacillus_cereus]	CPTF_Cu	573154.6	93917.14137	16.38600499
UJJ68482.1	D-amino-acid_transaminase_[Bacillus_cereus]	CPTF_Fe	649271.3667	107909.4743	16.62008827
UJJ68482.1	D-amino-acid_transaminase_[Bacillus_cereus]	CPTF_Mn	473958.6333	110215.292	23.25420075
UJJ68482.1	D-amino-acid_transaminase_[Bacillus_cereus]	CPTF_Ni	623249.8667	55146.91102	8.848282842
UJJ68482.1	D-amino-acid_transaminase_[Bacillus_cereus]	CPTF_U	352299	168618.6058	47.86235721
UJJ68482.1	D-amino-acid_transaminase_[Bacillus_cereus]	CPTF_metals_mix	286441.3333	102950.7182	35.94129275
UJJ68482.1	D-amino-acid_transaminase_[Bacillus_cereus]	CPTF_zcontrol	450352.6667	25029.38686	5.557730354
UJJ68483.1	nitroreductase_family_protein_[Bacillus_cereus]	CPTF_Al	0	0	0
UJJ68483.1	nitroreductase_family_protein_[Bacillus_cereus]	CPTF_Cd	0	0	0
UJJ68483.1	nitroreductase_family_protein_[Bacillus_cereus]	CPTF_Co	0	0	0
UJJ68483.1	nitroreductase_family_protein_[Bacillus_cereus]	CPTF_Cu	0	0	0
UJJ68483.1	nitroreductase_family_protein_[Bacillus_cereus]	CPTF_Fe	8686.2	15044.93972	173.2050808
UJJ68483.1	nitroreductase_family_protein_[Bacillus_cereus]	CPTF_Mn	0	0	0
UJJ68483.1	nitroreductase_family_protein_[Bacillus_cereus]	CPTF_Ni	26487.6	45877.86897	173.2050808
UJJ68483.1	nitroreductase_family_protein_[Bacillus_cereus]	CPTF_U	0	0	0
UJJ68483.1	nitroreductase_family_protein_[Bacillus_cereus]	CPTF_metals_mix	0	0	0
UJJ68483.1	nitroreductase_family_protein_[Bacillus_cereus]	CPTF_zcontrol	0	0	0
UJJ68485.1	cysteine_hydrolase_[Bacillus_cereus]	CPTF_Al	355422.69	18153.71273	5.107640352
UJJ68485.1	cysteine_hydrolase_[Bacillus_cereus]	CPTF_Cd	411561.2333	46330.92019	11.2573577
UJJ68485.1	cysteine_hydrolase_[Bacillus_cereus]	CPTF_Co	355200.0667	42308.61891	11.91120804
UJJ68485.1	cysteine_hydrolase_[Bacillus_cereus]	CPTF_Cu	415902.9667	27329.31952	6.571080687
UJJ68485.1	cysteine_hydrolase_[Bacillus_cereus]	CPTF_Fe	402200.9	23959.2413	5.957033239
UJJ68485.1	cysteine_hydrolase_[Bacillus_cereus]	CPTF_Mn	362491	48247.76667	13.31005919
UJJ68485.1	cysteine_hydrolase_[Bacillus_cereus]	CPTF_Ni	394666.6333	21771.34651	5.51638894
UJJ68485.1	cysteine_hydrolase_[Bacillus_cereus]	CPTF_U	453326.4333	87556.89367	19.31431464
UJJ68485.1	cysteine_hydrolase_[Bacillus_cereus]	CPTF_metals_mix	585292.17	13154.34173	2.247482951
UJJ68485.1	cysteine_hydrolase_[Bacillus_cereus]	CPTF_zcontrol	379229.3333	24614.22683	6.490591488
UJJ68491.1	alcohol_dehydrogenase_AdhP_[Bacillus_cereus]	CPTF_Al	5556001.667	475081.5113	8.550780575
UJJ68491.1	alcohol_dehydrogenase_AdhP_[Bacillus_cereus]	CPTF_Cd	7023667	864118.543	12.30295433
UJJ68491.1	alcohol_dehydrogenase_AdhP_[Bacillus_cereus]	CPTF_Co	5588730.9	387743.4158	6.93795108
UJJ68491.1	alcohol_dehydrogenase_AdhP_[Bacillus_cereus]	CPTF_Cu	5963003.633	287068.404	4.814157791
UJJ68491.1	alcohol_dehydrogenase_AdhP_[Bacillus_cereus]	CPTF_Fe	5324767.067	48806.39711	0.916592153
UJJ68491.1	alcohol_dehydrogenase_AdhP_[Bacillus_cereus]	CPTF_Mn	6393816.1	2555343.72	39.96586201
UJJ68491.1	alcohol_dehydrogenase_AdhP_[Bacillus_cereus]	CPTF_Ni	5492694.633	321575.0273	5.854595035
UJJ68491.1	alcohol_dehydrogenase_AdhP_[Bacillus_cereus]	CPTF_U	4996152.333	48743.05096	0.975611785
UJJ68491.1	alcohol_dehydrogenase_AdhP_[Bacillus_cereus]	CPTF_metals_mix	38998061.13	9207600.016	23.61040459
UJJ68491.1	alcohol_dehydrogenase_AdhP_[Bacillus_cereus]	CPTF_zcontrol	4924017	365254.9237	7.417824181
UJJ68496.1	decarboxylating_6-phosphogluconate_dehydrogenase_[Bacillus_cereus]	CPTF_Al	743160.4333	140132.3699	18.85627431
UJJ68496.1	decarboxylating_6-phosphogluconate_dehydrogenase_[Bacillus_cereus]	CPTF_Cd	883274.3333	65240.96702	7.386263198
UJJ68496.1	decarboxylating_6-phosphogluconate_dehydrogenase_[Bacillus_cereus]	CPTF_Co	803536.7	98546.95911	12.26415161
UJJ68496.1	decarboxylating_6-phosphogluconate_dehydrogenase_[Bacillus_cereus]	CPTF_Cu	777760.1667	28609.7212	3.678476017
UJJ68496.1	decarboxylating_6-phosphogluconate_dehydrogenase_[Bacillus_cereus]	CPTF_Fe	757360	123922.9743	16.36249265

UIJ68496.1	decarboxylating_6-phosphogluconate_dehydrogenase_[Bacillus_cereus]	CPTF_Mn	795747.6667	254701.9535	32.00787941
UIJ68496.1	decarboxylating_6-phosphogluconate_dehydrogenase_[Bacillus_cereus]	CPTF_Ni	700369.5333	161520.5779	23.06219363
UIJ68496.1	decarboxylating_6-phosphogluconate_dehydrogenase_[Bacillus_cereus]	CPTF_U	441433.3333	393036.3484	89.03640001
UIJ68496.1	decarboxylating_6-phosphogluconate_dehydrogenase_[Bacillus_cereus]	CPTF_metals_mix	780155.1667	168661.8055	21.61900769
UIJ68496.1	decarboxylating_6-phosphogluconate_dehydrogenase_[Bacillus_cereus]	CPTF_zcontrol	1043527.6	169100.0804	16.20465816
UIJ68499.1	NAD(P)H-dependent_oxidoreductase_[Bacillus_cereus]	CPTF_Al	1891424.967	491270.6475	25.97357316
UIJ68499.1	NAD(P)H-dependent_oxidoreductase_[Bacillus_cereus]	CPTF_Cd	1423613.667	297595.9122	20.9042607
UIJ68499.1	NAD(P)H-dependent_oxidoreductase_[Bacillus_cereus]	CPTF_Co	2043292.567	231929.9973	11.35079729
UIJ68499.1	NAD(P)H-dependent_oxidoreductase_[Bacillus_cereus]	CPTF_Cu	1985409.967	231142.013	11.64202945
UIJ68499.1	NAD(P)H-dependent_oxidoreductase_[Bacillus_cereus]	CPTF_Fe	1241364.4	206944.2853	16.67071211
UIJ68499.1	NAD(P)H-dependent_oxidoreductase_[Bacillus_cereus]	CPTF_Mn	1646412.5	433717.6543	26.34319494
UIJ68499.1	NAD(P)H-dependent_oxidoreductase_[Bacillus_cereus]	CPTF_Ni	1651285.033	842875.4456	51.04360717
UIJ68499.1	NAD(P)H-dependent_oxidoreductase_[Bacillus_cereus]	CPTF_U	1308109.333	84802.36245	6.482819156
UIJ68499.1	NAD(P)H-dependent_oxidoreductase_[Bacillus_cereus]	CPTF_metals_mix	1572867.633	271436.4098	17.25742231
UIJ68499.1	NAD(P)H-dependent_oxidoreductase_[Bacillus_cereus]	CPTF_zcontrol	1716836.533	311855.6526	18.16455128
UIJ68503.1	ABC_transporter_permease/substrate-binding_protein_[Bacillus_cereus]	CPTF_Al	0	0	0
UIJ68503.1	ABC_transporter_permease/substrate-binding_protein_[Bacillus_cereus]	CPTF_Cd	320152	554519.5301	173.2050808
UIJ68503.1	ABC_transporter_permease/substrate-binding_protein_[Bacillus_cereus]	CPTF_Co	0	0	0
UIJ68503.1	ABC_transporter_permease/substrate-binding_protein_[Bacillus_cereus]	CPTF_Cu	0	0	0
UIJ68503.1	ABC_transporter_permease/substrate-binding_protein_[Bacillus_cereus]	CPTF_Fe	0	0	0
UIJ68503.1	ABC_transporter_permease/substrate-binding_protein_[Bacillus_cereus]	CPTF_Mn	285793.3333	495008.5738	173.2050808
UIJ68503.1	ABC_transporter_permease/substrate-binding_protein_[Bacillus_cereus]	CPTF_Ni	0	0	0
UIJ68503.1	ABC_transporter_permease/substrate-binding_protein_[Bacillus_cereus]	CPTF_U	279502.6667	484112.8195	173.2050808
UIJ68503.1	ABC_transporter_permease/substrate-binding_protein_[Bacillus_cereus]	CPTF_metals_mix	0	0	0
UIJ68503.1	ABC_transporter_permease/substrate-binding_protein_[Bacillus_cereus]	CPTF_zcontrol	0	0	0
UIJ68505.1	LemA_domain_protein_[Bacillus_cereus]	CPTF_Al	213002.1667	17691.30558	8.30569278
UIJ68505.1	LemA_domain_protein_[Bacillus_cereus]	CPTF_Cd	294506.6667	68424.47632	23.23359165
UIJ68505.1	LemA_domain_protein_[Bacillus_cereus]	CPTF_Co	233279.9667	48588.66466	20.8284772
UIJ68505.1	LemA_domain_protein_[Bacillus_cereus]	CPTF_Cu	175301.8	47901.78336	27.32532316
UIJ68505.1	LemA_domain_protein_[Bacillus_cereus]	CPTF_Fe	280990.3333	63082.73582	22.45014449
UIJ68505.1	LemA_domain_protein_[Bacillus_cereus]	CPTF_Mn	233311.3	88534.52274	37.94695016
UIJ68505.1	LemA_domain_protein_[Bacillus_cereus]	CPTF_Ni	245882.5667	76447.46837	31.09104863
UIJ68505.1	LemA_domain_protein_[Bacillus_cereus]	CPTF_U	0	0	0
UIJ68505.1	LemA_domain_protein_[Bacillus_cereus]	CPTF_metals_mix	136834.4	10279.60291	7.512440519
UIJ68505.1	LemA_domain_protein_[Bacillus_cereus]	CPTF_zcontrol	199032.3333	106060.3238	53.28798696
UIJ68510.1	aldehyde_dehydrogenase_family_protein_[Bacillus_cereus]	CPTF_Al	12890.56667	22327.11641	173.2050808
UIJ68510.1	aldehyde_dehydrogenase_family_protein_[Bacillus_cereus]	CPTF_Cd	45686.83333	43260.10712	94.68834665
UIJ68510.1	aldehyde_dehydrogenase_family_protein_[Bacillus_cereus]	CPTF_Co	109991.9333	159647.7502	145.1449623
UIJ68510.1	aldehyde_dehydrogenase_family_protein_[Bacillus_cereus]	CPTF_Cu	0	0	0
UIJ68510.1	aldehyde_dehydrogenase_family_protein_[Bacillus_cereus]	CPTF_Fe	28408.23333	49204.50349	173.2050808
UIJ68510.1	aldehyde_dehydrogenase_family_protein_[Bacillus_cereus]	CPTF_Mn	0	0	0
UIJ68510.1	aldehyde_dehydrogenase_family_protein_[Bacillus_cereus]	CPTF_Ni	0	0	0
UIJ68510.1	aldehyde_dehydrogenase_family_protein_[Bacillus_cereus]	CPTF_U	20270.76667	35109.99778	173.2050808
UIJ68510.1	aldehyde_dehydrogenase_family_protein_[Bacillus_cereus]	CPTF_metals_mix	54890.46667	9988.233898	18.1966642
UIJ68510.1	aldehyde_dehydrogenase_family_protein_[Bacillus_cereus]	CPTF_zcontrol	0	0	0
UIJ68511.1	YueI_family_protein_[Bacillus_cereus]	CPTF_Al	50767.46667	50027.20627	98.54186067
UIJ68511.1	YueI_family_protein_[Bacillus_cereus]	CPTF_Cd	60307.63333	68381.06152	113.3870751
UIJ68511.1	YueI_family_protein_[Bacillus_cereus]	CPTF_Co	91118.1	48525.80307	53.25594263
UIJ68511.1	YueI_family_protein_[Bacillus_cereus]	CPTF_Cu	73360.2	13717.79971	18.6992398
UIJ68511.1	YueI_family_protein_[Bacillus_cereus]	CPTF_Fe	82552.33333	5238.098653	6.345185461
UIJ68511.1	YueI_family_protein_[Bacillus_cereus]	CPTF_Mn	0	0	0
UIJ68511.1	YueI_family_protein_[Bacillus_cereus]	CPTF_Ni	18890.46667	32719.24805	173.2050808
UIJ68511.1	YueI_family_protein_[Bacillus_cereus]	CPTF_U	33379	57814.12391	173.2050808
UIJ68511.1	YueI_family_protein_[Bacillus_cereus]	CPTF_metals_mix	86362	23647.67201	27.38203378
UIJ68511.1	YueI_family_protein_[Bacillus_cereus]	CPTF_zcontrol	0	0	0
UIJ68519.1	lysine_2,3-aminomutase_[Bacillus_cereus]	CPTF_Al	2902560.333	269832.1125	9.296348103
UIJ68519.1	lysine_2,3-aminomutase_[Bacillus_cereus]	CPTF_Cd	2857391.4	384467.7269	13.45519997
UIJ68519.1	lysine_2,3-aminomutase_[Bacillus_cereus]	CPTF_Co	1859005.367	205129.0563	11.0343445
UIJ68519.1	lysine_2,3-aminomutase_[Bacillus_cereus]	CPTF_Cu	2978251.733	350881.4854	11.78145828
UIJ68519.1	lysine_2,3-aminomutase_[Bacillus_cereus]	CPTF_Fe	3169211	593420.0897	18.72453711
UIJ68519.1	lysine_2,3-aminomutase_[Bacillus_cereus]	CPTF_Mn	2846234.467	479207.5708	16.83654584

UIJ68519.1	lysine_2,3-aminomutase_[Bacillus_cereus]	CPTF_Ni	2002006.767	571969.5689	28.56981197
UIJ68519.1	lysine_2,3-aminomutase_[Bacillus_cereus]	CPTF_U	2097281	228101.3711	10.87605195
UIJ68519.1	lysine_2,3-aminomutase_[Bacillus_cereus]	CPTF_metals_mix	4011361.733	241365.3076	6.017041684
UIJ68519.1	lysine_2,3-aminomutase_[Bacillus_cereus]	CPTF_zcontrol	2322068.333	83110.07401	3.579139891
UIJ68520.1	YokU_family_protein_[Bacillus_cereus]	CPTF_Al	26803.83333	46425.60117	173.2050808
UIJ68520.1	YokU_family_protein_[Bacillus_cereus]	CPTF_Cd	0	0	0
UIJ68520.1	YokU_family_protein_[Bacillus_cereus]	CPTF_Co	0	0	0
UIJ68520.1	YokU_family_protein_[Bacillus_cereus]	CPTF_Cu	0	0	0
UIJ68520.1	YokU_family_protein_[Bacillus_cereus]	CPTF_Fe	0	0	0
UIJ68520.1	YokU_family_protein_[Bacillus_cereus]	CPTF_Mn	0	0	0
UIJ68520.1	YokU_family_protein_[Bacillus_cereus]	CPTF_Ni	0	0	0
UIJ68520.1	YokU_family_protein_[Bacillus_cereus]	CPTF_U	0	0	0
UIJ68520.1	YokU_family_protein_[Bacillus_cereus]	CPTF_metals_mix	191726.3333	14256.47885	7.435848069
UIJ68520.1	YokU_family_protein_[Bacillus_cereus]	CPTF_zcontrol	0	0	0
UIJ68527.1	sporulation_protein_[Bacillus_cereus]	CPTF_Al	134525.2	25513.36324	18.96548991
UIJ68527.1	sporulation_protein_[Bacillus_cereus]	CPTF_Cd	268242.3333	72703.37034	27.10361539
UIJ68527.1	sporulation_protein_[Bacillus_cereus]	CPTF_Co	138883.1333	102267.0756	73.63534591
UIJ68527.1	sporulation_protein_[Bacillus_cereus]	CPTF_Cu	210586.5	149317.3774	70.90548414
UIJ68527.1	sporulation_protein_[Bacillus_cereus]	CPTF_Fe	290841.9	177388.5977	60.99141758
UIJ68527.1	sporulation_protein_[Bacillus_cereus]	CPTF_Mn	281276.4	249139.8705	88.57475085
UIJ68527.1	sporulation_protein_[Bacillus_cereus]	CPTF_Ni	84323.73333	83958.90466	99.56734759
UIJ68527.1	sporulation_protein_[Bacillus_cereus]	CPTF_U	12585.96667	21799.53373	173.2050808
UIJ68527.1	sporulation_protein_[Bacillus_cereus]	CPTF_metals_mix	1568076.5	175796.8462	11.21098659
UIJ68527.1	sporulation_protein_[Bacillus_cereus]	CPTF_zcontrol	99901.86667	77931.33425	78.00788599
UIJ68530.1	thioredoxin_family_protein_[Bacillus_cereus]	CPTF_Al	22621.26667	39181.1832	173.2050808
UIJ68530.1	thioredoxin_family_protein_[Bacillus_cereus]	CPTF_Cd	34129.43333	29659.10584	86.90184085
UIJ68530.1	thioredoxin_family_protein_[Bacillus_cereus]	CPTF_Co	13595.1	23547.40393	173.2050808
UIJ68530.1	thioredoxin_family_protein_[Bacillus_cereus]	CPTF_Cu	0	0	0
UIJ68530.1	thioredoxin_family_protein_[Bacillus_cereus]	CPTF_Fe	0	0	0
UIJ68530.1	thioredoxin_family_protein_[Bacillus_cereus]	CPTF_Mn	31034.9	53754.02361	173.2050808
UIJ68530.1	thioredoxin_family_protein_[Bacillus_cereus]	CPTF_Ni	53041.56667	47694.23919	89.91860947
UIJ68530.1	thioredoxin_family_protein_[Bacillus_cereus]	CPTF_U	59304.33333	102718.1184	173.2050808
UIJ68530.1	thioredoxin_family_protein_[Bacillus_cereus]	CPTF_metals_mix	399706.3	62216.34681	15.56551568
UIJ68530.1	thioredoxin_family_protein_[Bacillus_cereus]	CPTF_zcontrol	0	0	0
UIJ68548.1	PBP1A_family_penicillin-binding_protein_[Bacillus_cereus]	CPTF_Al	39089.53333	36037.0558	92.19106171
UIJ68548.1	PBP1A_family_penicillin-binding_protein_[Bacillus_cereus]	CPTF_Cd	37859.5	33721.1926	89.06930256
UIJ68548.1	PBP1A_family_penicillin-binding_protein_[Bacillus_cereus]	CPTF_Co	50723.76667	7535.714024	14.85637704
UIJ68548.1	PBP1A_family_penicillin-binding_protein_[Bacillus_cereus]	CPTF_Cu	119616.2667	67543.86495	56.46712344
UIJ68548.1	PBP1A_family_penicillin-binding_protein_[Bacillus_cereus]	CPTF_Fe	50232.8	57724.50759	114.9139757
UIJ68548.1	PBP1A_family_penicillin-binding_protein_[Bacillus_cereus]	CPTF_Mn	32566.73333	29026.05592	89.12793194
UIJ68548.1	PBP1A_family_penicillin-binding_protein_[Bacillus_cereus]	CPTF_Ni	43954.3	38067.24317	86.60641432
UIJ68548.1	PBP1A_family_penicillin-binding_protein_[Bacillus_cereus]	CPTF_U	0	0	0
UIJ68548.1	PBP1A_family_penicillin-binding_protein_[Bacillus_cereus]	CPTF_metals_mix	245079.7	14473.21934	5.905515364
UIJ68548.1	PBP1A_family_penicillin-binding_protein_[Bacillus_cereus]	CPTF_zcontrol	9788.533333	16954.23706	173.2050808
UIJ68552.1	citrate_synthase_[Bacillus_cereus]	CPTF_Al	1793163.833	266724.1556	14.87450007
UIJ68552.1	citrate_synthase_[Bacillus_cereus]	CPTF_Cd	1629604	506605.1558	31.08762348
UIJ68552.1	citrate_synthase_[Bacillus_cereus]	CPTF_Co	1674203.633	205099.513	12.25057149
UIJ68552.1	citrate_synthase_[Bacillus_cereus]	CPTF_Cu	1322832.1	442517.6556	33.45229191
UIJ68552.1	citrate_synthase_[Bacillus_cereus]	CPTF_Fe	1625931.833	363205.1609	22.33827725
UIJ68552.1	citrate_synthase_[Bacillus_cereus]	CPTF_Mn	1448514.2	558713.1296	38.57146375
UIJ68552.1	citrate_synthase_[Bacillus_cereus]	CPTF_Ni	1344598.567	236450.7494	17.58522992
UIJ68552.1	citrate_synthase_[Bacillus_cereus]	CPTF_U	1126458.2	301232.1217	26.74152682
UIJ68552.1	citrate_synthase_[Bacillus_cereus]	CPTF_metals_mix	2794777	243647.9589	8.717974954
UIJ68552.1	citrate_synthase_[Bacillus_cereus]	CPTF_zcontrol	1176101	141255.5717	12.01049669
UIJ68553.1	2-methylcitrate_dehydratase_[Bacillus_cereus]	CPTF_Al	5530776.5	222634.4347	4.025373918
UIJ68553.1	2-methylcitrate_dehydratase_[Bacillus_cereus]	CPTF_Cd	5991756.7	264781.9551	4.419103918
UIJ68553.1	2-methylcitrate_dehydratase_[Bacillus_cereus]	CPTF_Co	5377158.1	344002.6143	6.397480006
UIJ68553.1	2-methylcitrate_dehydratase_[Bacillus_cereus]	CPTF_Cu	5468895.667	192164.9113	3.513779071
UIJ68553.1	2-methylcitrate_dehydratase_[Bacillus_cereus]	CPTF_Fe	6025119.933	290383.3396	4.819544553
UIJ68553.1	2-methylcitrate_dehydratase_[Bacillus_cereus]	CPTF_Mn	5194628.933	658125.1671	12.66933934
UIJ68553.1	2-methylcitrate_dehydratase_[Bacillus_cereus]	CPTF_Ni	4844557.033	544825.3816	11.24613412

UIJ68553.1	2-methylcitrate_dehydratase_[Bacillus_cereus]	CPTF_U	5240168	890532.7725	16.99435538
UIJ68553.1	2-methylcitrate_dehydratase_[Bacillus_cereus]	CPTF_metals_mix	4736533.9	219247.2529	4.628854296
UIJ68553.1	2-methylcitrate_dehydratase_[Bacillus_cereus]	CPTF_zcontrol	5030543.067	102363.4121	2.034838201
UIJ68554.1	methylisocitrate_lyase_[Bacillus_cereus]	CPTF_Al	1229960.633	97690.03446	7.942533428
UIJ68554.1	methylisocitrate_lyase_[Bacillus_cereus]	CPTF_Cd	1496824.733	190396.5847	12.72003198
UIJ68554.1	methylisocitrate_lyase_[Bacillus_cereus]	CPTF_Co	1033896.6	88111.07827	8.5222331
UIJ68554.1	methylisocitrate_lyase_[Bacillus_cereus]	CPTF_Cu	1258726.367	129095.7479	10.25606131
UIJ68554.1	methylisocitrate_lyase_[Bacillus_cereus]	CPTF_Fe	1414210	357229.5829	25.26000968
UIJ68554.1	methylisocitrate_lyase_[Bacillus_cereus]	CPTF_Mn	1436631.267	52864.82989	3.679777206
UIJ68554.1	methylisocitrate_lyase_[Bacillus_cereus]	CPTF_Ni	1288333.133	113574.476	8.815613992
UIJ68554.1	methylisocitrate_lyase_[Bacillus_cereus]	CPTF_U	1526947.9	163265.2662	10.69226175
UIJ68554.1	methylisocitrate_lyase_[Bacillus_cereus]	CPTF_metals_mix	1757876.3	167517.2539	9.529524569
UIJ68554.1	methylisocitrate_lyase_[Bacillus_cereus]	CPTF_zcontrol	1278482	170298.2787	13.32035013
UIJ68555.1	acyl-CoA_dehydrogenase_family_protein_[Bacillus_cereus]	CPTF_Al	0	0	0
UIJ68555.1	acyl-CoA_dehydrogenase_family_protein_[Bacillus_cereus]	CPTF_Cd	27962.63333	28016.00163	100.1908558
UIJ68555.1	acyl-CoA_dehydrogenase_family_protein_[Bacillus_cereus]	CPTF_Co	0	0	0
UIJ68555.1	acyl-CoA_dehydrogenase_family_protein_[Bacillus_cereus]	CPTF_Cu	0	0	0
UIJ68555.1	acyl-CoA_dehydrogenase_family_protein_[Bacillus_cereus]	CPTF_Fe	0	0	0
UIJ68555.1	acyl-CoA_dehydrogenase_family_protein_[Bacillus_cereus]	CPTF_Mn	0	0	0
UIJ68555.1	acyl-CoA_dehydrogenase_family_protein_[Bacillus_cereus]	CPTF_Ni	0	0	0
UIJ68555.1	acyl-CoA_dehydrogenase_family_protein_[Bacillus_cereus]	CPTF_U	11410	19762.69971	173.2050808
UIJ68555.1	acyl-CoA_dehydrogenase_family_protein_[Bacillus_cereus]	CPTF_metals_mix	200836.6	136463.3354	67.94744353
UIJ68555.1	acyl-CoA_dehydrogenase_family_protein_[Bacillus_cereus]	CPTF_zcontrol	0	0	0
UIJ68556.1	NAD(P)-dependent_oxidoreductase_[Bacillus_cereus]	CPTF_Al	315376.6667	32482.13208	10.29947219
UIJ68556.1	NAD(P)-dependent_oxidoreductase_[Bacillus_cereus]	CPTF_Cd	362651.3333	23610.37582	6.51048918
UIJ68556.1	NAD(P)-dependent_oxidoreductase_[Bacillus_cereus]	CPTF_Co	332134.6667	68921.97114	20.7512127
UIJ68556.1	NAD(P)-dependent_oxidoreductase_[Bacillus_cereus]	CPTF_Cu	376512.3333	102671.8492	27.26918619
UIJ68556.1	NAD(P)-dependent_oxidoreductase_[Bacillus_cereus]	CPTF_Fe	364027	6929.019628	1.903435632
UIJ68556.1	NAD(P)-dependent_oxidoreductase_[Bacillus_cereus]	CPTF_Mn	334948	76374.86621	22.80200694
UIJ68556.1	NAD(P)-dependent_oxidoreductase_[Bacillus_cereus]	CPTF_Ni	318685.6667	41959.22699	13.16633642
UIJ68556.1	NAD(P)-dependent_oxidoreductase_[Bacillus_cereus]	CPTF_U	400912.3333	56171.30875	14.01087073
UIJ68556.1	NAD(P)-dependent_oxidoreductase_[Bacillus_cereus]	CPTF_metals_mix	518879.4333	57007.5749	10.98667074
UIJ68556.1	NAD(P)-dependent_oxidoreductase_[Bacillus_cereus]	CPTF_zcontrol	299327.3333	41488.40506	13.86054678
UIJ68557.1	CoA-acylating_methylmalonate-semialdehyde_dehydrogenase_[Bacillus_cereus]	CPTF_Al	3203501.467	268338.3596	8.376408203
UIJ68557.1	CoA-acylating_methylmalonate-semialdehyde_dehydrogenase_[Bacillus_cereus]	CPTF_Cd	3243462.3	249627.3963	7.696324889
UIJ68557.1	CoA-acylating_methylmalonate-semialdehyde_dehydrogenase_[Bacillus_cereus]	CPTF_Co	2854542.767	672422.747	23.55623306
UIJ68557.1	CoA-acylating_methylmalonate-semialdehyde_dehydrogenase_[Bacillus_cereus]	CPTF_Cu	3248819.767	234051.8151	7.204210512
UIJ68557.1	CoA-acylating_methylmalonate-semialdehyde_dehydrogenase_[Bacillus_cereus]	CPTF_Fe	3205238.333	674154.9035	21.0329103
UIJ68557.1	CoA-acylating_methylmalonate-semialdehyde_dehydrogenase_[Bacillus_cereus]	CPTF_Mn	2988665	653195.0084	21.85574524
UIJ68557.1	CoA-acylating_methylmalonate-semialdehyde_dehydrogenase_[Bacillus_cereus]	CPTF_Ni	3204000.2	78388.32606	2.446576815
UIJ68557.1	CoA-acylating_methylmalonate-semialdehyde_dehydrogenase_[Bacillus_cereus]	CPTF_U	3393109.667	526804.4617	15.52571279
UIJ68557.1	CoA-acylating_methylmalonate-semialdehyde_dehydrogenase_[Bacillus_cereus]	CPTF_metals_mix	4417141.9	627474.1527	14.20543344
UIJ68557.1	CoA-acylating_methylmalonate-semialdehyde_dehydrogenase_[Bacillus_cereus]	CPTF_zcontrol	3156135.333	203856.3865	6.459050865
UIJ68559.1	enoyl-CoA_hydratase/isomerase_family_protein_[Bacillus_cereus]	CPTF_Al	3691610.633	155884.5929	4.222671576
UIJ68559.1	enoyl-CoA_hydratase/isomerase_family_protein_[Bacillus_cereus]	CPTF_Cd	4696836.333	241650.028	5.144953132
UIJ68559.1	enoyl-CoA_hydratase/isomerase_family_protein_[Bacillus_cereus]	CPTF_Co	3654454.633	227672.9002	6.230010303
UIJ68559.1	enoyl-CoA_hydratase/isomerase_family_protein_[Bacillus_cereus]	CPTF_Cu	3372583.3	172503.7951	5.114886119
UIJ68559.1	enoyl-CoA_hydratase/isomerase_family_protein_[Bacillus_cereus]	CPTF_Fe	4022324.333	102641.1334	2.5517866
UIJ68559.1	enoyl-CoA_hydratase/isomerase_family_protein_[Bacillus_cereus]	CPTF_Mn	4079835.6	119630.4711	2.93223754
UIJ68559.1	enoyl-CoA_hydratase/isomerase_family_protein_[Bacillus_cereus]	CPTF_Ni	3634785.5	172192.6215	4.737353044
UIJ68559.1	enoyl-CoA_hydratase/isomerase_family_protein_[Bacillus_cereus]	CPTF_U	3977895.367	495150.5297	12.44755038
UIJ68559.1	enoyl-CoA_hydratase/isomerase_family_protein_[Bacillus_cereus]	CPTF_metals_mix	2911113.5	68670.30009	2.358901503
UIJ68559.1	enoyl-CoA_hydratase/isomerase_family_protein_[Bacillus_cereus]	CPTF_zcontrol	3942525.333	149189.365	3.784106693
UIJ68562.1	exonuclease_SbcCD_subunit_D_[Bacillus_cereus]	CPTF_Al	68475.1	15428.9717	22.53223682
UIJ68562.1	exonuclease_SbcCD_subunit_D_[Bacillus_cereus]	CPTF_Cd	36466	32843.86917	90.06710133
UIJ68562.1	exonuclease_SbcCD_subunit_D_[Bacillus_cereus]	CPTF_Co	52194.6	17010.40507	32.59035431
UIJ68562.1	exonuclease_SbcCD_subunit_D_[Bacillus_cereus]	CPTF_Cu	27326.46667	23774.95345	87.00339396
UIJ68562.1	exonuclease_SbcCD_subunit_D_[Bacillus_cereus]	CPTF_Fe	17078.1	29580.1369	173.2050808
UIJ68562.1	exonuclease_SbcCD_subunit_D_[Bacillus_cereus]	CPTF_Mn	33967.23333	29691.08506	87.41096092
UIJ68562.1	exonuclease_SbcCD_subunit_D_[Bacillus_cereus]	CPTF_Ni	33216.23333	28787.2541	86.66622075
UIJ68562.1	exonuclease_SbcCD_subunit_D_[Bacillus_cereus]	CPTF_U	0	0	0

UIJ68562.1	exonuclease_SbcCD_subunit_D_[Bacillus_cereus]	CPTF_metals_mix	13473.36667	23336.55562	173.2050808
UIJ68562.1	exonuclease_SbcCD_subunit_D_[Bacillus_cereus]	CPTF_zcontrol	13961.76667	24182.48923	173.2050808
UIJ68563.1	SMC_family_ATPase_[Bacillus_cereus]	CPTF_Al	201723.1333	187243.1005	92.82182831
UIJ68563.1	SMC_family_ATPase_[Bacillus_cereus]	CPTF_Cd	219765.8333	325766.8163	148.2336046
UIJ68563.1	SMC_family_ATPase_[Bacillus_cereus]	CPTF_Co	265273.1667	249538.6792	94.06857178
UIJ68563.1	SMC_family_ATPase_[Bacillus_cereus]	CPTF_Cu	127279.8667	35153.51095	27.61906645
UIJ68563.1	SMC_family_ATPase_[Bacillus_cereus]	CPTF_Fe	108370.6667	28547.22593	26.34220754
UIJ68563.1	SMC_family_ATPase_[Bacillus_cereus]	CPTF_Mn	167908.3333	94479.99167	56.2687925
UIJ68563.1	SMC_family_ATPase_[Bacillus_cereus]	CPTF_Ni	72975.9	66338.10941	90.9041333
UIJ68563.1	SMC_family_ATPase_[Bacillus_cereus]	CPTF_U	76773.23333	72408.39695	94.31463781
UIJ68563.1	SMC_family_ATPase_[Bacillus_cereus]	CPTF_metals_mix	145506.1667	82072.66822	56.40494152
UIJ68563.1	SMC_family_ATPase_[Bacillus_cereus]	CPTF_zcontrol	128514.6667	17424.28249	13.55820541
UIJ68566.1	YkvS_family_protein_[Bacillus_cereus]	CPTF_Al	72706.66667	125931.6407	173.2050808
UIJ68566.1	YkvS_family_protein_[Bacillus_cereus]	CPTF_Cd	291315.6667	52720.09365	18.09723941
UIJ68566.1	YkvS_family_protein_[Bacillus_cereus]	CPTF_Co	130784.3333	132559.8096	101.3575604
UIJ68566.1	YkvS_family_protein_[Bacillus_cereus]	CPTF_Cu	0	0	0
UIJ68566.1	YkvS_family_protein_[Bacillus_cereus]	CPTF_Fe	121276.9333	137962.7961	113.7584802
UIJ68566.1	YkvS_family_protein_[Bacillus_cereus]	CPTF_Mn	75344.33333	130500.2134	173.2050808
UIJ68566.1	YkvS_family_protein_[Bacillus_cereus]	CPTF_Ni	0	0	0
UIJ68566.1	YkvS_family_protein_[Bacillus_cereus]	CPTF_U	0	0	0
UIJ68566.1	YkvS_family_protein_[Bacillus_cereus]	CPTF_metals_mix	454111.3333	15536.41671	3.421279227
UIJ68566.1	YkvS_family_protein_[Bacillus_cereus]	CPTF_zcontrol	0	0	0
UIJ68570.1	isochorismate_synthase_DhbC_[Bacillus_cereus]	CPTF_Al	0	0	0
UIJ68570.1	isochorismate_synthase_DhbC_[Bacillus_cereus]	CPTF_Cd	0	0	0
UIJ68570.1	isochorismate_synthase_DhbC_[Bacillus_cereus]	CPTF_Co	0	0	0
UIJ68570.1	isochorismate_synthase_DhbC_[Bacillus_cereus]	CPTF_Cu	10464.66667	18125.33435	173.2050808
UIJ68570.1	isochorismate_synthase_DhbC_[Bacillus_cereus]	CPTF_Fe	0	0	0
UIJ68570.1	isochorismate_synthase_DhbC_[Bacillus_cereus]	CPTF_Mn	0	0	0
UIJ68570.1	isochorismate_synthase_DhbC_[Bacillus_cereus]	CPTF_Ni	0	0	0
UIJ68570.1	isochorismate_synthase_DhbC_[Bacillus_cereus]	CPTF_U	0	0	0
UIJ68570.1	isochorismate_synthase_DhbC_[Bacillus_cereus]	CPTF_metals_mix	110590.3333	26197.63662	23.68890284
UIJ68570.1	isochorismate_synthase_DhbC_[Bacillus_cereus]	CPTF_zcontrol	0	0	0
UIJ68571.1	(2,3-dihydroxybenzoyl)adenylate_synthase_[Bacillus_cereus]	CPTF_Al	0	0	0
UIJ68571.1	(2,3-dihydroxybenzoyl)adenylate_synthase_[Bacillus_cereus]	CPTF_Cd	0	0	0
UIJ68571.1	(2,3-dihydroxybenzoyl)adenylate_synthase_[Bacillus_cereus]	CPTF_Co	0	0	0
UIJ68571.1	(2,3-dihydroxybenzoyl)adenylate_synthase_[Bacillus_cereus]	CPTF_Cu	0	0	0
UIJ68571.1	(2,3-dihydroxybenzoyl)adenylate_synthase_[Bacillus_cereus]	CPTF_Fe	0	0	0
UIJ68571.1	(2,3-dihydroxybenzoyl)adenylate_synthase_[Bacillus_cereus]	CPTF_Mn	0	0	0
UIJ68571.1	(2,3-dihydroxybenzoyl)adenylate_synthase_[Bacillus_cereus]	CPTF_Ni	0	0	0
UIJ68571.1	(2,3-dihydroxybenzoyl)adenylate_synthase_[Bacillus_cereus]	CPTF_U	0	0	0
UIJ68571.1	(2,3-dihydroxybenzoyl)adenylate_synthase_[Bacillus_cereus]	CPTF_metals_mix	90560.63333	32986.44841	36.42471038
UIJ68571.1	(2,3-dihydroxybenzoyl)adenylate_synthase_[Bacillus_cereus]	CPTF_zcontrol	0	0	0
UIJ68572.1	isochorismatase_family_protein_[Bacillus_cereus]	CPTF_Al	0	0	0
UIJ68572.1	isochorismatase_family_protein_[Bacillus_cereus]	CPTF_Cd	0	0	0
UIJ68572.1	isochorismatase_family_protein_[Bacillus_cereus]	CPTF_Co	0	0	0
UIJ68572.1	isochorismatase_family_protein_[Bacillus_cereus]	CPTF_Cu	63294.83333	60438.60436	95.4874216
UIJ68572.1	isochorismatase_family_protein_[Bacillus_cereus]	CPTF_Fe	0	0	0
UIJ68572.1	isochorismatase_family_protein_[Bacillus_cereus]	CPTF_Mn	0	0	0
UIJ68572.1	isochorismatase_family_protein_[Bacillus_cereus]	CPTF_Ni	0	0	0
UIJ68572.1	isochorismatase_family_protein_[Bacillus_cereus]	CPTF_U	0	0	0
UIJ68572.1	isochorismatase_family_protein_[Bacillus_cereus]	CPTF_metals_mix	777629	64817.83887	8.335316568
UIJ68572.1	isochorismatase_family_protein_[Bacillus_cereus]	CPTF_zcontrol	0	0	0
UIJ68573.1	amino_acid_adenylation_domain-containing_protein_[Bacillus_cereus]	CPTF_Al	0	0	0
UIJ68573.1	amino_acid_adenylation_domain-containing_protein_[Bacillus_cereus]	CPTF_Cd	0	0	0
UIJ68573.1	amino_acid_adenylation_domain-containing_protein_[Bacillus_cereus]	CPTF_Co	0	0	0
UIJ68573.1	amino_acid_adenylation_domain-containing_protein_[Bacillus_cereus]	CPTF_Cu	100859.8667	12614.17407	12.5066337
UIJ68573.1	amino_acid_adenylation_domain-containing_protein_[Bacillus_cereus]	CPTF_Fe	0	0	0
UIJ68573.1	amino_acid_adenylation_domain-containing_protein_[Bacillus_cereus]	CPTF_Mn	0	0	0
UIJ68573.1	amino_acid_adenylation_domain-containing_protein_[Bacillus_cereus]	CPTF_Ni	0	0	0
UIJ68573.1	amino_acid_adenylation_domain-containing_protein_[Bacillus_cereus]	CPTF_U	0	0	0
UIJ68573.1	amino_acid_adenylation_domain-containing_protein_[Bacillus_cereus]	CPTF_metals_mix	170116	44716.13106	26.2856704

UIJ68573.1	amino_acid_adenylation_domain-containing_protein_[Bacillus_cereus]	CPTF_zcontrol	21345.96667	36972.2988	173.2050808
UIJ68578.1	HU_family_DNA-binding_protein_[Bacillus_cereus]	CPTF_Al	855138	66364.07719	7.76062778
UIJ68578.1	HU_family_DNA-binding_protein_[Bacillus_cereus]	CPTF_Cd	963101	87302.09735	9.064687644
UIJ68578.1	HU_family_DNA-binding_protein_[Bacillus_cereus]	CPTF_Co	991507	33588.419	3.387612896
UIJ68578.1	HU_family_DNA-binding_protein_[Bacillus_cereus]	CPTF_Cu	926164.3333	101068.0906	10.91254402
UIJ68578.1	HU_family_DNA-binding_protein_[Bacillus_cereus]	CPTF_Fe	837837.6667	116143.3356	13.86227192
UIJ68578.1	HU_family_DNA-binding_protein_[Bacillus_cereus]	CPTF_Mn	968657	485569.5919	50.12812501
UIJ68578.1	HU_family_DNA-binding_protein_[Bacillus_cereus]	CPTF_Ni	902756.3333	233227.369	25.83502994
UIJ68578.1	HU_family_DNA-binding_protein_[Bacillus_cereus]	CPTF_U	555130	67561.94578	12.17047282
UIJ68578.1	HU_family_DNA-binding_protein_[Bacillus_cereus]	CPTF_metals_mix	946901.3333	87252.64497	9.214544525
UIJ68578.1	HU_family_DNA-binding_protein_[Bacillus_cereus]	CPTF_zcontrol	811927.3333	36333.83905	4.475011194
UIJ68586.1	23S_rRNA_pseudouridine(2604)_synthase_RluF_[Bacillus_cereus]	CPTF_Al	88809.46667	23915.43874	26.92892958
UIJ68586.1	23S_rRNA_pseudouridine(2604)_synthase_RluF_[Bacillus_cereus]	CPTF_Cd	75090.66667	20807.67745	27.71007154
UIJ68586.1	23S_rRNA_pseudouridine(2604)_synthase_RluF_[Bacillus_cereus]	CPTF_Co	84802.9	55742.66653	65.73202865
UIJ68586.1	23S_rRNA_pseudouridine(2604)_synthase_RluF_[Bacillus_cereus]	CPTF_Cu	131024.1333	59469.53395	45.38822921
UIJ68586.1	23S_rRNA_pseudouridine(2604)_synthase_RluF_[Bacillus_cereus]	CPTF_Fe	76755.96667	16826.55433	21.92214503
UIJ68586.1	23S_rRNA_pseudouridine(2604)_synthase_RluF_[Bacillus_cereus]	CPTF_Mn	59724.63333	11176.32298	18.71308764
UIJ68586.1	23S_rRNA_pseudouridine(2604)_synthase_RluF_[Bacillus_cereus]	CPTF_Ni	65705.13333	56904.18688	86.60538986
UIJ68586.1	23S_rRNA_pseudouridine(2604)_synthase_RluF_[Bacillus_cereus]	CPTF_U	65638.4	16877.33513	25.71259374
UIJ68586.1	23S_rRNA_pseudouridine(2604)_synthase_RluF_[Bacillus_cereus]	CPTF_metals_mix	157755.1333	24873.48427	15.76714731
UIJ68586.1	23S_rRNA_pseudouridine(2604)_synthase_RluF_[Bacillus_cereus]	CPTF_zcontrol	102960.1667	26541.48849	25.77840475
UIJ68587.1	threonine--tRNA_ligase_[Bacillus_cereus]	CPTF_Al	76509.3	17536.34483	22.92054016
UIJ68587.1	threonine--tRNA_ligase_[Bacillus_cereus]	CPTF_Cd	89822.2	43623.30696	48.56628647
UIJ68587.1	threonine--tRNA_ligase_[Bacillus_cereus]	CPTF_Co	76367.2	28584.65184	37.43053542
UIJ68587.1	threonine--tRNA_ligase_[Bacillus_cereus]	CPTF_Cu	100976.8667	80420.36238	79.64236268
UIJ68587.1	threonine--tRNA_ligase_[Bacillus_cereus]	CPTF_Fe	117461.2	61860.40653	52.664545
UIJ68587.1	threonine--tRNA_ligase_[Bacillus_cereus]	CPTF_Mn	93875.5	57749.06965	61.51665733
UIJ68587.1	threonine--tRNA_ligase_[Bacillus_cereus]	CPTF_Ni	104731.2667	96950.03968	92.5702923
UIJ68587.1	threonine--tRNA_ligase_[Bacillus_cereus]	CPTF_U	80471.46667	97444.42073	121.091891
UIJ68587.1	threonine--tRNA_ligase_[Bacillus_cereus]	CPTF_metals_mix	0	0	0
UIJ68587.1	threonine--tRNA_ligase_[Bacillus_cereus]	CPTF_zcontrol	77183.33333	39714.13005	51.45428208
UIJ68604.1	GNAT_family_N-acetyltransferase_[Bacillus_cereus]	CPTF_Al	0	0	0
UIJ68604.1	GNAT_family_N-acetyltransferase_[Bacillus_cereus]	CPTF_Cd	0	0	0
UIJ68604.1	GNAT_family_N-acetyltransferase_[Bacillus_cereus]	CPTF_Co	0	0	0
UIJ68604.1	GNAT_family_N-acetyltransferase_[Bacillus_cereus]	CPTF_Cu	0	0	0
UIJ68604.1	GNAT_family_N-acetyltransferase_[Bacillus_cereus]	CPTF_Fe	0	0	0
UIJ68604.1	GNAT_family_N-acetyltransferase_[Bacillus_cereus]	CPTF_Mn	0	0	0
UIJ68604.1	GNAT_family_N-acetyltransferase_[Bacillus_cereus]	CPTF_Ni	0	0	0
UIJ68604.1	GNAT_family_N-acetyltransferase_[Bacillus_cereus]	CPTF_U	0	0	0
UIJ68604.1	GNAT_family_N-acetyltransferase_[Bacillus_cereus]	CPTF_metals_mix	379456	12217.61892	3.219772231
UIJ68604.1	GNAT_family_N-acetyltransferase_[Bacillus_cereus]	CPTF_zcontrol	0	0	0
UIJ68609.1	protoporphyrinogen_oxidase_[Bacillus_cereus]	CPTF_Al	267213.0333	41462.62294	15.51669184
UIJ68609.1	protoporphyrinogen_oxidase_[Bacillus_cereus]	CPTF_Cd	149729.1333	85884.59355	57.35997507
UIJ68609.1	protoporphyrinogen_oxidase_[Bacillus_cereus]	CPTF_Co	332092.4	49622.57151	14.94239902
UIJ68609.1	protoporphyrinogen_oxidase_[Bacillus_cereus]	CPTF_Cu	387261.3333	39183.68615	10.11815092
UIJ68609.1	protoporphyrinogen_oxidase_[Bacillus_cereus]	CPTF_Fe	217343.6667	96037.40201	44.18688774
UIJ68609.1	protoporphyrinogen_oxidase_[Bacillus_cereus]	CPTF_Mn	165161	55091.14986	33.35602828
UIJ68609.1	protoporphyrinogen_oxidase_[Bacillus_cereus]	CPTF_Ni	203673.9333	96656.86515	47.45666938
UIJ68609.1	protoporphyrinogen_oxidase_[Bacillus_cereus]	CPTF_U	162223.2	116404.9975	71.75607279
UIJ68609.1	protoporphyrinogen_oxidase_[Bacillus_cereus]	CPTF_metals_mix	558743.6	106988.9209	19.14812463
UIJ68609.1	protoporphyrinogen_oxidase_[Bacillus_cereus]	CPTF_zcontrol	183399.0333	25911.6784	14.12857959
UIJ68613.1	cold-shock_protein_CspD_[Bacillus_cereus]	CPTF_Al	557060.5333	94750.55809	17.00902369
UIJ68613.1	cold-shock_protein_CspD_[Bacillus_cereus]	CPTF_Cd	1018535.2	230901.2182	22.66993013
UIJ68613.1	cold-shock_protein_CspD_[Bacillus_cereus]	CPTF_Co	864266.7	154244.1018	17.84681763
UIJ68613.1	cold-shock_protein_CspD_[Bacillus_cereus]	CPTF_Cu	779004	79038.13282	10.14604968
UIJ68613.1	cold-shock_protein_CspD_[Bacillus_cereus]	CPTF_Fe	812079.5333	327742.694	40.35844773
UIJ68613.1	cold-shock_protein_CspD_[Bacillus_cereus]	CPTF_Mn	631853.0667	447434.4592	70.81305494
UIJ68613.1	cold-shock_protein_CspD_[Bacillus_cereus]	CPTF_Ni	429927.3333	222576.0859	51.77062928
UIJ68613.1	cold-shock_protein_CspD_[Bacillus_cereus]	CPTF_U	602087.3333	224307.4553	37.25496998
UIJ68613.1	cold-shock_protein_CspD_[Bacillus_cereus]	CPTF_metals_mix	1055788.267	128291.9682	12.15129702
UIJ68613.1	cold-shock_protein_CspD_[Bacillus_cereus]	CPTF_zcontrol	304741.4667	68323.52377	22.42015979

UIJ68615.1	ATP-binding_protein_[Bacillus_cereus]	CPTF_Al	859787.6667	53450.92629	6.2167589
UIJ68615.1	ATP-binding_protein_[Bacillus_cereus]	CPTF_Cd	818452.3333	36013.00211	4.400134333
UIJ68615.1	ATP-binding_protein_[Bacillus_cereus]	CPTF_Co	834207	43628.75167	5.229967103
UIJ68615.1	ATP-binding_protein_[Bacillus_cereus]	CPTF_Cu	831242.6667	72166.46246	8.681756286
UIJ68615.1	ATP-binding_protein_[Bacillus_cereus]	CPTF_Fe	845597	32774.38317	3.875886878
UIJ68615.1	ATP-binding_protein_[Bacillus_cereus]	CPTF_Mn	773891.3333	162788.1486	21.03501378
UIJ68615.1	ATP-binding_protein_[Bacillus_cereus]	CPTF_Ni	883408	122092.2644	13.82059755
UIJ68615.1	ATP-binding_protein_[Bacillus_cereus]	CPTF_U	858694.3333	33380.96476	3.887409462
UIJ68615.1	ATP-binding_protein_[Bacillus_cereus]	CPTF_metals_mix	0	0	0
UIJ68615.1	ATP-binding_protein_[Bacillus_cereus]	CPTF_zcontrol	899680	82321.0682	9.150038702
UIJ68623.1	GNAT_family_N-acetyltransferase_[Bacillus_cereus]	CPTF_Al	12348.53333	21388.28713	173.2050808
UIJ68623.1	GNAT_family_N-acetyltransferase_[Bacillus_cereus]	CPTF_Cd	10971.76667	19003.65732	173.2050808
UIJ68623.1	GNAT_family_N-acetyltransferase_[Bacillus_cereus]	CPTF_Co	21248.3	18504.04664	87.08483335
UIJ68623.1	GNAT_family_N-acetyltransferase_[Bacillus_cereus]	CPTF_Cu	19863.23333	17215.09007	86.6681158
UIJ68623.1	GNAT_family_N-acetyltransferase_[Bacillus_cereus]	CPTF_Fe	0	0	0
UIJ68623.1	GNAT_family_N-acetyltransferase_[Bacillus_cereus]	CPTF_Mn	0	0	0
UIJ68623.1	GNAT_family_N-acetyltransferase_[Bacillus_cereus]	CPTF_Ni	9494.333333	16444.66772	173.2050808
UIJ68623.1	GNAT_family_N-acetyltransferase_[Bacillus_cereus]	CPTF_U	0	0	0
UIJ68623.1	GNAT_family_N-acetyltransferase_[Bacillus_cereus]	CPTF_metals_mix	31556.03333	30547.37608	96.80359935
UIJ68623.1	GNAT_family_N-acetyltransferase_[Bacillus_cereus]	CPTF_zcontrol	0	0	0
UIJ68630.1	DUF5519_family_protein_[Bacillus_cereus]	CPTF_Al	266375.3333	183930.1457	69.04924092
UIJ68630.1	DUF5519_family_protein_[Bacillus_cereus]	CPTF_Cd	93336.2	105493.6346	113.0254227
UIJ68630.1	DUF5519_family_protein_[Bacillus_cereus]	CPTF_Co	183955.2	139552.8606	75.86241685
UIJ68630.1	DUF5519_family_protein_[Bacillus_cereus]	CPTF_Cu	315360.3	92146.62523	29.21947538
UIJ68630.1	DUF5519_family_protein_[Bacillus_cereus]	CPTF_Fe	139507.5333	67477.54774	48.36838995
UIJ68630.1	DUF5519_family_protein_[Bacillus_cereus]	CPTF_Mn	138031.4333	161156.6547	116.7535907
UIJ68630.1	DUF5519_family_protein_[Bacillus_cereus]	CPTF_Ni	123927.4333	46472.64058	37.49988145
UIJ68630.1	DUF5519_family_protein_[Bacillus_cereus]	CPTF_U	22913.83333	39687.92353	173.2050808
UIJ68630.1	DUF5519_family_protein_[Bacillus_cereus]	CPTF_metals_mix	809718.7667	192611.3128	23.78743346
UIJ68630.1	DUF5519_family_protein_[Bacillus_cereus]	CPTF_zcontrol	77882.56667	62770.4908	80.59633046
UIJ68634.1	class_I_SAM-dependent_methyltransferase_[Bacillus_cereus]	CPTF_Al	236649	22964.13946	9.703881891
UIJ68634.1	class_I_SAM-dependent_methyltransferase_[Bacillus_cereus]	CPTF_Cd	250682.6333	41530.36285	16.56690864
UIJ68634.1	class_I_SAM-dependent_methyltransferase_[Bacillus_cereus]	CPTF_Co	154539.5667	82635.50831	53.47207197
UIJ68634.1	class_I_SAM-dependent_methyltransferase_[Bacillus_cereus]	CPTF_Cu	181990.6667	27046.82285	14.86165381
UIJ68634.1	class_I_SAM-dependent_methyltransferase_[Bacillus_cereus]	CPTF_Fe	245708.7333	20063.43365	8.165535418
UIJ68634.1	class_I_SAM-dependent_methyltransferase_[Bacillus_cereus]	CPTF_Mn	218777.6667	58420.424	26.70310224
UIJ68634.1	class_I_SAM-dependent_methyltransferase_[Bacillus_cereus]	CPTF_Ni	107644.8667	8066.241916	7.493382793
UIJ68634.1	class_I_SAM-dependent_methyltransferase_[Bacillus_cereus]	CPTF_U	117844.3333	65020.44261	55.17485718
UIJ68634.1	class_I_SAM-dependent_methyltransferase_[Bacillus_cereus]	CPTF_metals_mix	716935.3333	92873.36561	12.95421795
UIJ68634.1	class_I_SAM-dependent_methyltransferase_[Bacillus_cereus]	CPTF_zcontrol	178798.3333	57267.24322	32.02895808
UIJ68635.1	toxic_anion_resistance_protein_[Bacillus_cereus]	CPTF_Al	1795246.967	71189.21234	3.965427246
UIJ68635.1	toxic_anion_resistance_protein_[Bacillus_cereus]	CPTF_Cd	1675498.9	59642.8917	3.559709392
UIJ68635.1	toxic_anion_resistance_protein_[Bacillus_cereus]	CPTF_Co	1651839.433	71467.86173	4.326562273
UIJ68635.1	toxic_anion_resistance_protein_[Bacillus_cereus]	CPTF_Cu	1851748.067	137103.7919	7.404019716
UIJ68635.1	toxic_anion_resistance_protein_[Bacillus_cereus]	CPTF_Fe	1695422.867	67094.82391	3.957409401
UIJ68635.1	toxic_anion_resistance_protein_[Bacillus_cereus]	CPTF_Mn	1784479.7	100174.0891	5.613630075
UIJ68635.1	toxic_anion_resistance_protein_[Bacillus_cereus]	CPTF_Ni	1841486.333	157766.7715	8.567360432
UIJ68635.1	toxic_anion_resistance_protein_[Bacillus_cereus]	CPTF_U	1836437	50709.4932	2.761297731
UIJ68635.1	toxic_anion_resistance_protein_[Bacillus_cereus]	CPTF_metals_mix	2227245.867	226433.8055	10.16653837
UIJ68635.1	toxic_anion_resistance_protein_[Bacillus_cereus]	CPTF_zcontrol	1863481.233	19111.26444	1.025567851
UIJ68650.1	DEAD/DEAH_box_helicase_[Bacillus_cereus]	CPTF_Al	8058.9	13958.42425	173.2050808
UIJ68650.1	DEAD/DEAH_box_helicase_[Bacillus_cereus]	CPTF_Cd	0	0	0
UIJ68650.1	DEAD/DEAH_box_helicase_[Bacillus_cereus]	CPTF_Co	0	0	0
UIJ68650.1	DEAD/DEAH_box_helicase_[Bacillus_cereus]	CPTF_Cu	0	0	0
UIJ68650.1	DEAD/DEAH_box_helicase_[Bacillus_cereus]	CPTF_Fe	99086.9	171623.5452	173.2050808
UIJ68650.1	DEAD/DEAH_box_helicase_[Bacillus_cereus]	CPTF_Mn	74057.66667	128271.6414	173.2050808
UIJ68650.1	DEAD/DEAH_box_helicase_[Bacillus_cereus]	CPTF_Ni	0	0	0
UIJ68650.1	DEAD/DEAH_box_helicase_[Bacillus_cereus]	CPTF_U	0	0	0
UIJ68650.1	DEAD/DEAH_box_helicase_[Bacillus_cereus]	CPTF_metals_mix	16991.36667	19464.63218	114.5560128
UIJ68650.1	DEAD/DEAH_box_helicase_[Bacillus_cereus]	CPTF_zcontrol	0	0	0
UIJ68655.1	VOC_family_protein_[Bacillus_cereus]	CPTF_Al	0	0	0

UIJ68655.1	VOC_family_protein_[Bacillus_cereus]	CPTF_Cd	13888.16667	24055.01029	173.2050808
UIJ68655.1	VOC_family_protein_[Bacillus_cereus]	CPTF_Co	0	0	0
UIJ68655.1	VOC_family_protein_[Bacillus_cereus]	CPTF_Cu	0	0	0
UIJ68655.1	VOC_family_protein_[Bacillus_cereus]	CPTF_Fe	0	0	0
UIJ68655.1	VOC_family_protein_[Bacillus_cereus]	CPTF_Mn	0	0	0
UIJ68655.1	VOC_family_protein_[Bacillus_cereus]	CPTF_Ni	0	0	0
UIJ68655.1	VOC_family_protein_[Bacillus_cereus]	CPTF_U	0	0	0
UIJ68655.1	VOC_family_protein_[Bacillus_cereus]	CPTF_metals_mix	11883.03333	20582.01748	173.2050808
UIJ68655.1	VOC_family_protein_[Bacillus_cereus]	CPTF_zcontrol	0	0	0
UIJ68659.1	2,3-diphosphoglycerate-dependent_phosphoglycerate_mutase_[Bacillus_cereus]	CPTF_Al	581508.1333	206150.9458	35.45108554
UIJ68659.1	2,3-diphosphoglycerate-dependent_phosphoglycerate_mutase_[Bacillus_cereus]	CPTF_Cd	392823.8667	175962.1312	44.79415488
UIJ68659.1	2,3-diphosphoglycerate-dependent_phosphoglycerate_mutase_[Bacillus_cereus]	CPTF_Co	302704.4	40533.9095	13.39059145
UIJ68659.1	2,3-diphosphoglycerate-dependent_phosphoglycerate_mutase_[Bacillus_cereus]	CPTF_Cu	371923.4333	28431.34377	7.644407752
UIJ68659.1	2,3-diphosphoglycerate-dependent_phosphoglycerate_mutase_[Bacillus_cereus]	CPTF_Fe	521446.7667	181264.1659	34.7617777
UIJ68659.1	2,3-diphosphoglycerate-dependent_phosphoglycerate_mutase_[Bacillus_cereus]	CPTF_Mn	366120.9667	230205.1566	62.87680236
UIJ68659.1	2,3-diphosphoglycerate-dependent_phosphoglycerate_mutase_[Bacillus_cereus]	CPTF_Ni	576621.2	127102.6585	22.04266137
UIJ68659.1	2,3-diphosphoglycerate-dependent_phosphoglycerate_mutase_[Bacillus_cereus]	CPTF_U	285099.3667	274495.6025	96.28067774
UIJ68659.1	2,3-diphosphoglycerate-dependent_phosphoglycerate_mutase_[Bacillus_cereus]	CPTF_metals_mix	284771.5	30751.72506	10.7987369
UIJ68659.1	2,3-diphosphoglycerate-dependent_phosphoglycerate_mutase_[Bacillus_cereus]	CPTF_zcontrol	412136.2	321622.1157	78.03782238
UIJ68664.1	beta-Ala-His_dipeptidase_[Bacillus_cereus]	CPTF_Al	3714890.733	419349.1823	11.28833154
UIJ68664.1	beta-Ala-His_dipeptidase_[Bacillus_cereus]	CPTF_Cd	3703869.933	529810.0314	14.30422885
UIJ68664.1	beta-Ala-His_dipeptidase_[Bacillus_cereus]	CPTF_Co	3425918.467	625002.7714	18.24336386
UIJ68664.1	beta-Ala-His_dipeptidase_[Bacillus_cereus]	CPTF_Cu	3968166.867	305032.1857	7.686979806
UIJ68664.1	beta-Ala-His_dipeptidase_[Bacillus_cereus]	CPTF_Fe	3259233.9	275268.2288	8.445795462
UIJ68664.1	beta-Ala-His_dipeptidase_[Bacillus_cereus]	CPTF_Mn	2981769.633	623099.4272	20.89696737
UIJ68664.1	beta-Ala-His_dipeptidase_[Bacillus_cereus]	CPTF_Ni	3161789.333	371700.1109	11.75600496
UIJ68664.1	beta-Ala-His_dipeptidase_[Bacillus_cereus]	CPTF_U	2611231.267	140825.3663	5.393063729
UIJ68664.1	beta-Ala-His_dipeptidase_[Bacillus_cereus]	CPTF_metals_mix	3663745.8	297289.963	8.114371991
UIJ68664.1	beta-Ala-His_dipeptidase_[Bacillus_cereus]	CPTF_zcontrol	2968201.567	199189.9848	6.710797104
UIJ68670.1	hypothetical_protein_LW858_10810_[Bacillus_cereus]	CPTF_Al	7058.566667	12225.7961	173.2050808
UIJ68670.1	hypothetical_protein_LW858_10810_[Bacillus_cereus]	CPTF_Cd	17634.7	15272.0992	86.6025461
UIJ68670.1	hypothetical_protein_LW858_10810_[Bacillus_cereus]	CPTF_Co	10742.23333	18606.09392	173.2050808
UIJ68670.1	hypothetical_protein_LW858_10810_[Bacillus_cereus]	CPTF_Cu	4504.566667	7802.138333	173.2050808
UIJ68670.1	hypothetical_protein_LW858_10810_[Bacillus_cereus]	CPTF_Fe	0	0	0
UIJ68670.1	hypothetical_protein_LW858_10810_[Bacillus_cereus]	CPTF_Mn	0	0	0
UIJ68670.1	hypothetical_protein_LW858_10810_[Bacillus_cereus]	CPTF_Ni	11534.46667	19978.2823	173.2050808
UIJ68670.1	hypothetical_protein_LW858_10810_[Bacillus_cereus]	CPTF_U	0	0	0
UIJ68670.1	hypothetical_protein_LW858_10810_[Bacillus_cereus]	CPTF_metals_mix	0	0	0
UIJ68670.1	hypothetical_protein_LW858_10810_[Bacillus_cereus]	CPTF_zcontrol	0	0	0
UIJ68674.1	non-ribosomal_peptide_synthetase_[Bacillus_cereus]	CPTF_Al	69004.46667	88301.53661	127.9649577
UIJ68674.1	non-ribosomal_peptide_synthetase_[Bacillus_cereus]	CPTF_Cd	9265.033333	8232.930973	88.86024126
UIJ68674.1	non-ribosomal_peptide_synthetase_[Bacillus_cereus]	CPTF_Co	7961.52	7713.62557	96.88634293
UIJ68674.1	non-ribosomal_peptide_synthetase_[Bacillus_cereus]	CPTF_Cu	38000.33333	65818.50804	173.2050808
UIJ68674.1	non-ribosomal_peptide_synthetase_[Bacillus_cereus]	CPTF_Fe	35672.5	51823.50043	145.2757739
UIJ68674.1	non-ribosomal_peptide_synthetase_[Bacillus_cereus]	CPTF_Mn	96620.66667	83730.28829	86.65877723
UIJ68674.1	non-ribosomal_peptide_synthetase_[Bacillus_cereus]	CPTF_Ni	13398.83333	23207.4601	173.2050808
UIJ68674.1	non-ribosomal_peptide_synthetase_[Bacillus_cereus]	CPTF_U	109181.6667	111197.5364	101.8463445
UIJ68674.1	non-ribosomal_peptide_synthetase_[Bacillus_cereus]	CPTF_metals_mix	0	0	0
UIJ68674.1	non-ribosomal_peptide_synthetase_[Bacillus_cereus]	CPTF_zcontrol	17154	18209.16201	106.1511135
UIJ68675.1	amino_acid_adenylation_domain-containing_protein_[Bacillus_cereus]	CPTF_Al	336052.1333	146761.4315	43.67222133
UIJ68675.1	amino_acid_adenylation_domain-containing_protein_[Bacillus_cereus]	CPTF_Cd	244278.8	50670.60739	20.74294101
UIJ68675.1	amino_acid_adenylation_domain-containing_protein_[Bacillus_cereus]	CPTF_Co	296459.9	32006.38953	10.79619521
UIJ68675.1	amino_acid_adenylation_domain-containing_protein_[Bacillus_cereus]	CPTF_Cu	144467.1333	87954.27104	60.88185528
UIJ68675.1	amino_acid_adenylation_domain-containing_protein_[Bacillus_cereus]	CPTF_Fe	214620.7	49493.83528	23.06107252
UIJ68675.1	amino_acid_adenylation_domain-containing_protein_[Bacillus_cereus]	CPTF_Mn	207559.8333	184059.1428	88.67763086
UIJ68675.1	amino_acid_adenylation_domain-containing_protein_[Bacillus_cereus]	CPTF_Ni	198157.1667	114962.1883	58.01566011
UIJ68675.1	amino_acid_adenylation_domain-containing_protein_[Bacillus_cereus]	CPTF_U	178558.0333	36586.41681	20.48993043
UIJ68675.1	amino_acid_adenylation_domain-containing_protein_[Bacillus_cereus]	CPTF_metals_mix	0	0	0
UIJ68675.1	amino_acid_adenylation_domain-containing_protein_[Bacillus_cereus]	CPTF_zcontrol	219800.7667	167230.5059	76.08276734
UIJ68698.1	acyl-CoA_dehydrogenase_[Bacillus_cereus]	CPTF_Al	1292831.667	110684.2338	8.561380163
UIJ68698.1	acyl-CoA_dehydrogenase_[Bacillus_cereus]	CPTF_Cd	1298364	49512.70314	3.813468576

UIJ68698.1	acyl-CoA_dehydrogenase_[Bacillus_cereus]	CPTF_Co	1297974.333	88814.53941	6.842549743
UIJ68698.1	acyl-CoA_dehydrogenase_[Bacillus_cereus]	CPTF_Cu	1257728.6	144898.286	11.52063219
UIJ68698.1	acyl-CoA_dehydrogenase_[Bacillus_cereus]	CPTF_Fe	1342312.667	84418.37466	6.289024663
UIJ68698.1	acyl-CoA_dehydrogenase_[Bacillus_cereus]	CPTF_Mn	1708743.233	725314.7106	42.44726162
UIJ68698.1	acyl-CoA_dehydrogenase_[Bacillus_cereus]	CPTF_Ni	1121823.333	56404.11303	5.027896225
UIJ68698.1	acyl-CoA_dehydrogenase_[Bacillus_cereus]	CPTF_U	1196511	331815.1169	27.73189022
UIJ68698.1	acyl-CoA_dehydrogenase_[Bacillus_cereus]	CPTF_metals_mix	2214779	206652.9887	9.330636992
UIJ68698.1	acyl-CoA_dehydrogenase_[Bacillus_cereus]	CPTF_zcontrol	1293066.967	86015.74867	6.652072235
UIJ68699.1	acetyl-CoA_carboxylase_biotin_carboxylase_subunit_[Bacillus_cereus]	CPTF_Al	1406655.533	466331.2836	33.15177544
UIJ68699.1	acetyl-CoA_carboxylase_biotin_carboxylase_subunit_[Bacillus_cereus]	CPTF_Cd	1921060.267	111750.491	5.817125725
UIJ68699.1	acetyl-CoA_carboxylase_biotin_carboxylase_subunit_[Bacillus_cereus]	CPTF_Co	1370643.2	568110.0816	41.44842958
UIJ68699.1	acetyl-CoA_carboxylase_biotin_carboxylase_subunit_[Bacillus_cereus]	CPTF_Cu	1019980.133	80243.109	7.8671247
UIJ68699.1	acetyl-CoA_carboxylase_biotin_carboxylase_subunit_[Bacillus_cereus]	CPTF_Fe	1563631.433	408358.0175	26.11600207
UIJ68699.1	acetyl-CoA_carboxylase_biotin_carboxylase_subunit_[Bacillus_cereus]	CPTF_Mn	1235955.767	582041.2599	47.09240214
UIJ68699.1	acetyl-CoA_carboxylase_biotin_carboxylase_subunit_[Bacillus_cereus]	CPTF_Ni	726739	202150.2315	27.81607035
UIJ68699.1	acetyl-CoA_carboxylase_biotin_carboxylase_subunit_[Bacillus_cereus]	CPTF_U	902530.3	62335.30591	6.906727221
UIJ68699.1	acetyl-CoA_carboxylase_biotin_carboxylase_subunit_[Bacillus_cereus]	CPTF_metals_mix	2739534.3	280985.8768	10.25670227
UIJ68699.1	acetyl-CoA_carboxylase_biotin_carboxylase_subunit_[Bacillus_cereus]	CPTF_zcontrol	933184	207647.9014	22.25154968
UIJ68701.1	hydroxymethylglutaryl-CoA_lyase_[Bacillus_cereus]	CPTF_Al	89354.83333	154767.1112	173.2050808
UIJ68701.1	hydroxymethylglutaryl-CoA_lyase_[Bacillus_cereus]	CPTF_Cd	11008.96667	19068.08961	173.2050808
UIJ68701.1	hydroxymethylglutaryl-CoA_lyase_[Bacillus_cereus]	CPTF_Co	53298.73333	74792.22527	140.3264592
UIJ68701.1	hydroxymethylglutaryl-CoA_lyase_[Bacillus_cereus]	CPTF_Cu	65421.96667	72154.91778	110.2915755
UIJ68701.1	hydroxymethylglutaryl-CoA_lyase_[Bacillus_cereus]	CPTF_Fe	16615.46667	28778.83246	173.2050808
UIJ68701.1	hydroxymethylglutaryl-CoA_lyase_[Bacillus_cereus]	CPTF_Mn	0	0	0
UIJ68701.1	hydroxymethylglutaryl-CoA_lyase_[Bacillus_cereus]	CPTF_Ni	0	0	0
UIJ68701.1	hydroxymethylglutaryl-CoA_lyase_[Bacillus_cereus]	CPTF_U	36000.9	62355.38792	173.2050808
UIJ68701.1	hydroxymethylglutaryl-CoA_lyase_[Bacillus_cereus]	CPTF_metals_mix	183155.4	86417.18686	47.18244008
UIJ68701.1	hydroxymethylglutaryl-CoA_lyase_[Bacillus_cereus]	CPTF_zcontrol	17172.16667	29743.06514	173.2050808
UIJ68702.1	enoyl-CoA_hydratase_[Bacillus_cereus]	CPTF_Al	153127.2	85351.672	55.7390666
UIJ68702.1	enoyl-CoA_hydratase_[Bacillus_cereus]	CPTF_Cd	219918.3333	86158.34093	39.17742538
UIJ68702.1	enoyl-CoA_hydratase_[Bacillus_cereus]	CPTF_Co	177944.8667	85371.01515	47.97610448
UIJ68702.1	enoyl-CoA_hydratase_[Bacillus_cereus]	CPTF_Cu	94455.66667	83647.49062	88.55740854
UIJ68702.1	enoyl-CoA_hydratase_[Bacillus_cereus]	CPTF_Fe	146257.3333	142032.1753	97.11114794
UIJ68702.1	enoyl-CoA_hydratase_[Bacillus_cereus]	CPTF_Mn	81428.66667	72364.2074	88.86822094
UIJ68702.1	enoyl-CoA_hydratase_[Bacillus_cereus]	CPTF_Ni	0	0	0
UIJ68702.1	enoyl-CoA_hydratase_[Bacillus_cereus]	CPTF_U	0	0	0
UIJ68702.1	enoyl-CoA_hydratase_[Bacillus_cereus]	CPTF_metals_mix	601538.5	31334.5014	5.209060001
UIJ68702.1	enoyl-CoA_hydratase_[Bacillus_cereus]	CPTF_zcontrol	105784.6667	103884.2391	98.20349432
UIJ68703.1	acyl-CoA_carboxylase_subunit_beta_[Bacillus_cereus]	CPTF_Al	3175706.967	456191.8893	14.36504986
UIJ68703.1	acyl-CoA_carboxylase_subunit_beta_[Bacillus_cereus]	CPTF_Cd	3367017.867	87207.68382	2.590057056
UIJ68703.1	acyl-CoA_carboxylase_subunit_beta_[Bacillus_cereus]	CPTF_Co	2672654.033	527113.3619	19.72246895
UIJ68703.1	acyl-CoA_carboxylase_subunit_beta_[Bacillus_cereus]	CPTF_Cu	2943412.867	293731.7381	9.979291095
UIJ68703.1	acyl-CoA_carboxylase_subunit_beta_[Bacillus_cereus]	CPTF_Fe	3293228	429907.8761	13.0543004
UIJ68703.1	acyl-CoA_carboxylase_subunit_beta_[Bacillus_cereus]	CPTF_Mn	2921128.1	193394.7949	6.620551659
UIJ68703.1	acyl-CoA_carboxylase_subunit_beta_[Bacillus_cereus]	CPTF_Ni	2659772.333	250328.8056	9.411662887
UIJ68703.1	acyl-CoA_carboxylase_subunit_beta_[Bacillus_cereus]	CPTF_U	2450411.667	697304.9115	28.45664347
UIJ68703.1	acyl-CoA_carboxylase_subunit_beta_[Bacillus_cereus]	CPTF_metals_mix	3808775.533	65152.96649	1.710601371
UIJ68703.1	acyl-CoA_carboxylase_subunit_beta_[Bacillus_cereus]	CPTF_zcontrol	3128073.933	665703.3457	21.28157326
UIJ68704.1	AMP-binding_protein_[Bacillus_cereus]	CPTF_Al	1409144.267	471401.4234	33.45302781
UIJ68704.1	AMP-binding_protein_[Bacillus_cereus]	CPTF_Cd	1817365.333	292255.6105	16.08128014
UIJ68704.1	AMP-binding_protein_[Bacillus_cereus]	CPTF_Co	1763245.8	389578.3672	22.09438793
UIJ68704.1	AMP-binding_protein_[Bacillus_cereus]	CPTF_Cu	1578616.967	294002.8699	18.62407893
UIJ68704.1	AMP-binding_protein_[Bacillus_cereus]	CPTF_Fe	1449004.867	218723.4279	15.09473384
UIJ68704.1	AMP-binding_protein_[Bacillus_cereus]	CPTF_Mn	1507638.667	281494.0054	18.67118505
UIJ68704.1	AMP-binding_protein_[Bacillus_cereus]	CPTF_Ni	1417928	442759.0204	31.22577595
UIJ68704.1	AMP-binding_protein_[Bacillus_cereus]	CPTF_U	833821.3	589808.5754	70.73560911
UIJ68704.1	AMP-binding_protein_[Bacillus_cereus]	CPTF_metals_mix	1935113.7	186841.1352	9.655305276
UIJ68704.1	AMP-binding_protein_[Bacillus_cereus]	CPTF_zcontrol	1256084.367	120172.365	9.567220817
UIJ68731.1	glyoxalase/bleomycin_resistance/dioxygenase_family_protein_[Bacillus_cereus]	CPTF_Al	925940.7667	75850.42269	8.191714354
UIJ68731.1	glyoxalase/bleomycin_resistance/dioxygenase_family_protein_[Bacillus_cereus]	CPTF_Cd	336209.6667	82059.43569	24.4072208
UIJ68731.1	glyoxalase/bleomycin_resistance/dioxygenase_family_protein_[Bacillus_cereus]	CPTF_Co	1028816.667	39068.24813	3.797396504

UIJ68731.1	glyoxalase/bleomycin_resistance/dioxygenase_family_protein_[Bacillus_cereus]	CPTF_Cu	1349984.167	150223.7527	11.1278159
UIJ68731.1	glyoxalase/bleomycin_resistance/dioxygenase_family_protein_[Bacillus_cereus]	CPTF_Fe	674594.7	118439.5272	17.55713871
UIJ68731.1	glyoxalase/bleomycin_resistance/dioxygenase_family_protein_[Bacillus_cereus]	CPTF_Mn	553815	399911.0007	72.21021472
UIJ68731.1	glyoxalase/bleomycin_resistance/dioxygenase_family_protein_[Bacillus_cereus]	CPTF_Ni	922048.6667	285866.0156	31.00335438
UIJ68731.1	glyoxalase/bleomycin_resistance/dioxygenase_family_protein_[Bacillus_cereus]	CPTF_U	683064	238722.8365	34.9488242
UIJ68731.1	glyoxalase/bleomycin_resistance/dioxygenase_family_protein_[Bacillus_cereus]	CPTF_metals_mix	1268428.467	34237.56433	2.699211286
UIJ68731.1	glyoxalase/bleomycin_resistance/dioxygenase_family_protein_[Bacillus_cereus]	CPTF_zcontrol	900481	127897.638	14.20325782
UIJ68773.1	cytochrome_P450_[Bacillus_cereus]	CPTF_Al	334283	62344.55047	18.65023063
UIJ68773.1	cytochrome_P450_[Bacillus_cereus]	CPTF_Cd	271359.3333	51434.9964	18.95457059
UIJ68773.1	cytochrome_P450_[Bacillus_cereus]	CPTF_Co	352685.3333	209657.0903	59.44593396
UIJ68773.1	cytochrome_P450_[Bacillus_cereus]	CPTF_Cu	1098218.7	164960.7185	15.02075301
UIJ68773.1	cytochrome_P450_[Bacillus_cereus]	CPTF_Fe	214266.3333	18585.77796	8.674147576
UIJ68773.1	cytochrome_P450_[Bacillus_cereus]	CPTF_Mn	264361.3333	72687.17769	27.49538927
UIJ68773.1	cytochrome_P450_[Bacillus_cereus]	CPTF_Ni	328512.6667	40382.08981	12.29239963
UIJ68773.1	cytochrome_P450_[Bacillus_cereus]	CPTF_U	279499	242423.0045	86.73483786
UIJ68773.1	cytochrome_P450_[Bacillus_cereus]	CPTF_metals_mix	1783538.433	338128.5451	18.95829878
UIJ68773.1	cytochrome_P450_[Bacillus_cereus]	CPTF_zcontrol	295410.3333	111422.3748	37.71783254
UIJ68777.1	cytochrome_P450_[Bacillus_cereus]	CPTF_Al	91429.33333	158360.2506	173.2050808
UIJ68777.1	cytochrome_P450_[Bacillus_cereus]	CPTF_Cd	156926.8667	137370.2435	87.537747
UIJ68777.1	cytochrome_P450_[Bacillus_cereus]	CPTF_Co	193350.3333	168851.7483	87.3294322
UIJ68777.1	cytochrome_P450_[Bacillus_cereus]	CPTF_Cu	123015.6667	213069.3848	173.2050808
UIJ68777.1	cytochrome_P450_[Bacillus_cereus]	CPTF_Fe	306505.3333	47384.2133	15.45950695
UIJ68777.1	cytochrome_P450_[Bacillus_cereus]	CPTF_Mn	66363	114944.0877	173.2050808
UIJ68777.1	cytochrome_P450_[Bacillus_cereus]	CPTF_Ni	317369	274900.857	86.61868582
UIJ68777.1	cytochrome_P450_[Bacillus_cereus]	CPTF_U	103490	179249.9381	173.2050808
UIJ68777.1	cytochrome_P450_[Bacillus_cereus]	CPTF_metals_mix	0	0	0
UIJ68777.1	cytochrome_P450_[Bacillus_cereus]	CPTF_zcontrol	76555.66667	132598.3043	173.2050808
UIJ68794.1	penicillin-binding_protein_[Bacillus_cereus]	CPTF_Al	33597.33333	58192.28833	173.2050808
UIJ68794.1	penicillin-binding_protein_[Bacillus_cereus]	CPTF_Cd	37876.66667	65604.31109	173.2050808
UIJ68794.1	penicillin-binding_protein_[Bacillus_cereus]	CPTF_Co	56385.33333	97662.26214	173.2050808
UIJ68794.1	penicillin-binding_protein_[Bacillus_cereus]	CPTF_Cu	0	0	0
UIJ68794.1	penicillin-binding_protein_[Bacillus_cereus]	CPTF_Fe	52673.66667	91233.46689	173.2050808
UIJ68794.1	penicillin-binding_protein_[Bacillus_cereus]	CPTF_Mn	23151.6	40099.74748	173.2050808
UIJ68794.1	penicillin-binding_protein_[Bacillus_cereus]	CPTF_Ni	0	0	0
UIJ68794.1	penicillin-binding_protein_[Bacillus_cereus]	CPTF_U	0	0	0
UIJ68794.1	penicillin-binding_protein_[Bacillus_cereus]	CPTF_metals_mix	0	0	0
UIJ68794.1	penicillin-binding_protein_[Bacillus_cereus]	CPTF_zcontrol	18334.56667	31756.401	173.2050808
UIJ68796.1	beta-lactamase_family_protein_[Bacillus_cereus]	CPTF_Al	139909.6667	24830.78135	17.7477239
UIJ68796.1	beta-lactamase_family_protein_[Bacillus_cereus]	CPTF_Cd	108334.8333	25735.34055	23.75537005
UIJ68796.1	beta-lactamase_family_protein_[Bacillus_cereus]	CPTF_Co	130170	24274.34358	18.64818589
UIJ68796.1	beta-lactamase_family_protein_[Bacillus_cereus]	CPTF_Cu	137942.8333	52391.28652	37.98043382
UIJ68796.1	beta-lactamase_family_protein_[Bacillus_cereus]	CPTF_Fe	96358.33333	88216.12867	91.55007732
UIJ68796.1	beta-lactamase_family_protein_[Bacillus_cereus]	CPTF_Mn	95317.2	93190.7708	97.76910232
UIJ68796.1	beta-lactamase_family_protein_[Bacillus_cereus]	CPTF_Ni	126450	109932.8336	86.93778852
UIJ68796.1	beta-lactamase_family_protein_[Bacillus_cereus]	CPTF_U	101396.0333	26208.20948	25.84737156
UIJ68796.1	beta-lactamase_family_protein_[Bacillus_cereus]	CPTF_metals_mix	0	0	0
UIJ68796.1	beta-lactamase_family_protein_[Bacillus_cereus]	CPTF_zcontrol	102316.8	44936.11697	43.9186106
UIJ68800.1	ester_cyclase_[Bacillus_cereus]	CPTF_Al	697126.3667	313605.9356	44.98552208
UIJ68800.1	ester_cyclase_[Bacillus_cereus]	CPTF_Cd	1024669.667	115045.6116	11.22758049
UIJ68800.1	ester_cyclase_[Bacillus_cereus]	CPTF_Co	702498.3333	124194.7112	17.67900439
UIJ68800.1	ester_cyclase_[Bacillus_cereus]	CPTF_Cu	300257.4667	85387.85949	28.43821352
UIJ68800.1	ester_cyclase_[Bacillus_cereus]	CPTF_Fe	729042.5333	392042.309	53.77495703
UIJ68800.1	ester_cyclase_[Bacillus_cereus]	CPTF_Mn	785915	122800.4235	15.62515329
UIJ68800.1	ester_cyclase_[Bacillus_cereus]	CPTF_Ni	358663.3333	105473.0109	29.40724661
UIJ68800.1	ester_cyclase_[Bacillus_cereus]	CPTF_U	462868.3333	289122.2301	62.46316918
UIJ68800.1	ester_cyclase_[Bacillus_cereus]	CPTF_metals_mix	608349.4333	315395.0091	51.84438282
UIJ68800.1	ester_cyclase_[Bacillus_cereus]	CPTF_zcontrol	844833.6667	64663.69651	7.654015111
UIJ68837.1	nucleotide_excision_repair_endonuclease_[Bacillus_cereus]	CPTF_Al	485190.6667	105320.8102	21.70709732
UIJ68837.1	nucleotide_excision_repair_endonuclease_[Bacillus_cereus]	CPTF_Cd	566457.3333	100961.7299	17.82336002
UIJ68837.1	nucleotide_excision_repair_endonuclease_[Bacillus_cereus]	CPTF_Co	529190.6667	67160.93995	12.69125557
UIJ68837.1	nucleotide_excision_repair_endonuclease_[Bacillus_cereus]	CPTF_Cu	674994.3333	155995.0437	23.11057085

UIJ68837.1	nucleotide_excision_repair_endonuclease_[Bacillus_cereus]	CPTF_Fe	565742	129509.2821	22.89193344
UIJ68837.1	nucleotide_excision_repair_endonuclease_[Bacillus_cereus]	CPTF_Mn	384947	174445.7954	45.31683464
UIJ68837.1	nucleotide_excision_repair_endonuclease_[Bacillus_cereus]	CPTF_Ni	426877.3333	107312.2128	25.13888754
UIJ68837.1	nucleotide_excision_repair_endonuclease_[Bacillus_cereus]	CPTF_U	417727	78469.42672	18.7848587
UIJ68837.1	nucleotide_excision_repair_endonuclease_[Bacillus_cereus]	CPTF_metals_mix	445461.7333	34548.56502	7.755675165
UIJ68837.1	nucleotide_excision_repair_endonuclease_[Bacillus_cereus]	CPTF_zcontrol	526420.6667	155459.9096	29.53149818
UIJ68852.1	DinB_family_protein_[Bacillus_cereus]	CPTF_Al	1543582.93	198656.2969	12.86981691
UIJ68852.1	DinB_family_protein_[Bacillus_cereus]	CPTF_Cd	1671754	41093.36298	2.458098678
UIJ68852.1	DinB_family_protein_[Bacillus_cereus]	CPTF_Co	1531733.767	37652.17109	2.458140697
UIJ68852.1	DinB_family_protein_[Bacillus_cereus]	CPTF_Cu	1446949.633	195098.8745	13.48345996
UIJ68852.1	DinB_family_protein_[Bacillus_cereus]	CPTF_Fe	1566108.467	209705.8787	13.39025254
UIJ68852.1	DinB_family_protein_[Bacillus_cereus]	CPTF_Mn	1395869.567	456185.4524	32.68109452
UIJ68852.1	DinB_family_protein_[Bacillus_cereus]	CPTF_Ni	1691005.633	74335.9	4.395958152
UIJ68852.1	DinB_family_protein_[Bacillus_cereus]	CPTF_U	1833244	190678.1751	10.40113455
UIJ68852.1	DinB_family_protein_[Bacillus_cereus]	CPTF_metals_mix	1306816.033	103886.7928	7.949611124
UIJ68852.1	DinB_family_protein_[Bacillus_cereus]	CPTF_zcontrol	1602261	94363.5119	5.889397039
UIJ68858.1	DinB_family_protein_[Bacillus_cereus]	CPTF_Al	60347.83333	15040.60989	24.92319783
UIJ68858.1	DinB_family_protein_[Bacillus_cereus]	CPTF_Cd	86599.06667	28843.30025	33.30671029
UIJ68858.1	DinB_family_protein_[Bacillus_cereus]	CPTF_Co	65569.6	10634.67236	16.21890688
UIJ68858.1	DinB_family_protein_[Bacillus_cereus]	CPTF_Cu	44202.2	20712.53269	46.85860136
UIJ68858.1	DinB_family_protein_[Bacillus_cereus]	CPTF_Fe	62360.8	21858.59605	35.0518211
UIJ68858.1	DinB_family_protein_[Bacillus_cereus]	CPTF_Mn	36317.36667	36097.2511	99.393911
UIJ68858.1	DinB_family_protein_[Bacillus_cereus]	CPTF_Ni	40032.9	11073.91449	27.66203421
UIJ68858.1	DinB_family_protein_[Bacillus_cereus]	CPTF_U	7413.933333	12841.30922	173.2050808
UIJ68858.1	DinB_family_protein_[Bacillus_cereus]	CPTF_metals_mix	269492.9333	69409.64949	25.75564733
UIJ68858.1	DinB_family_protein_[Bacillus_cereus]	CPTF_zcontrol	38760.86667	34887.43909	90.00686024
UIJ68880.1	kynureninase_[Bacillus_cereus]	CPTF_Al	336733.1	148867.3003	44.20928633
UIJ68880.1	kynureninase_[Bacillus_cereus]	CPTF_Cd	503587.6667	21521.54774	4.273644724
UIJ68880.1	kynureninase_[Bacillus_cereus]	CPTF_Co	368291.09	41298.64001	11.21358652
UIJ68880.1	kynureninase_[Bacillus_cereus]	CPTF_Cu	322182.2667	142160.6508	44.12429409
UIJ68880.1	kynureninase_[Bacillus_cereus]	CPTF_Fe	397362.8633	31150.75093	7.839371467
UIJ68880.1	kynureninase_[Bacillus_cereus]	CPTF_Mn	207080.6	179283.1641	86.57651373
UIJ68880.1	kynureninase_[Bacillus_cereus]	CPTF_Ni	339983.7667	186074.9044	54.73052618
UIJ68880.1	kynureninase_[Bacillus_cereus]	CPTF_U	182922.2333	172224.6657	94.15184942
UIJ68880.1	kynureninase_[Bacillus_cereus]	CPTF_metals_mix	249389.76	60922.29795	24.42854829
UIJ68880.1	kynureninase_[Bacillus_cereus]	CPTF_zcontrol	284329.9667	123222.8309	43.33796834
UIJ68890.1	class_I_SAM-dependent_methyltransferase_[Bacillus_cereus]	CPTF_Al	5595.133333	9691.055208	173.2050808
UIJ68890.1	class_I_SAM-dependent_methyltransferase_[Bacillus_cereus]	CPTF_Cd	7222.6	12509.91016	173.2050808
UIJ68890.1	class_I_SAM-dependent_methyltransferase_[Bacillus_cereus]	CPTF_Co	15489.6	14349.84511	92.64180556
UIJ68890.1	class_I_SAM-dependent_methyltransferase_[Bacillus_cereus]	CPTF_Cu	25094.93333	25438.59621	101.3694512
UIJ68890.1	class_I_SAM-dependent_methyltransferase_[Bacillus_cereus]	CPTF_Fe	0	0	0
UIJ68890.1	class_I_SAM-dependent_methyltransferase_[Bacillus_cereus]	CPTF_Mn	0	0	0
UIJ68890.1	class_I_SAM-dependent_methyltransferase_[Bacillus_cereus]	CPTF_Ni	0	0	0
UIJ68890.1	class_I_SAM-dependent_methyltransferase_[Bacillus_cereus]	CPTF_U	0	0	0
UIJ68890.1	class_I_SAM-dependent_methyltransferase_[Bacillus_cereus]	CPTF_metals_mix	22131.83333	6061.491777	27.38811415
UIJ68890.1	class_I_SAM-dependent_methyltransferase_[Bacillus_cereus]	CPTF_zcontrol	0	0	0
UIJ68909.1	GNAT_family_N-acetyltransferase_[Bacillus_cereus]	CPTF_Al	0	0	0
UIJ68909.1	GNAT_family_N-acetyltransferase_[Bacillus_cereus]	CPTF_Cd	0	0	0
UIJ68909.1	GNAT_family_N-acetyltransferase_[Bacillus_cereus]	CPTF_Co	69758	120824.4002	173.2050808
UIJ68909.1	GNAT_family_N-acetyltransferase_[Bacillus_cereus]	CPTF_Cu	0	0	0
UIJ68909.1	GNAT_family_N-acetyltransferase_[Bacillus_cereus]	CPTF_Fe	0	0	0
UIJ68909.1	GNAT_family_N-acetyltransferase_[Bacillus_cereus]	CPTF_Mn	0	0	0
UIJ68909.1	GNAT_family_N-acetyltransferase_[Bacillus_cereus]	CPTF_Ni	0	0	0
UIJ68909.1	GNAT_family_N-acetyltransferase_[Bacillus_cereus]	CPTF_U	0	0	0
UIJ68909.1	GNAT_family_N-acetyltransferase_[Bacillus_cereus]	CPTF_metals_mix	33528	58072.19948	173.2050808
UIJ68909.1	GNAT_family_N-acetyltransferase_[Bacillus_cereus]	CPTF_zcontrol	0	0	0
UIJ68939.1	manganese-dependent_inorganic_pyrophosphatase_[Bacillus_cereus]	CPTF_Al	2128287.333	175338.5454	8.238480897
UIJ68939.1	manganese-dependent_inorganic_pyrophosphatase_[Bacillus_cereus]	CPTF_Cd	2485184.2	79446.17826	3.196792345
UIJ68939.1	manganese-dependent_inorganic_pyrophosphatase_[Bacillus_cereus]	CPTF_Co	2453034.6	147389.6097	6.008460284
UIJ68939.1	manganese-dependent_inorganic_pyrophosphatase_[Bacillus_cereus]	CPTF_Cu	1980458.133	40872.84467	2.06380756
UIJ68939.1	manganese-dependent_inorganic_pyrophosphatase_[Bacillus_cereus]	CPTF_Fe	2232861.6	342914.1972	15.35761093

UIJ68939.1	manganese-dependent_inorganic_pyrophosphatase_[Bacillus_cereus]	CPTF_Mn	2860817.7	848804.761	29.67000522
UIJ68939.1	manganese-dependent_inorganic_pyrophosphatase_[Bacillus_cereus]	CPTF_Ni	1894286.4	294641.463	15.55421941
UIJ68939.1	manganese-dependent_inorganic_pyrophosphatase_[Bacillus_cereus]	CPTF_U	1908976.8	115876.3421	6.070075976
UIJ68939.1	manganese-dependent_inorganic_pyrophosphatase_[Bacillus_cereus]	CPTF_metals_mix	5956121.9	665269.2734	11.16950399
UIJ68939.1	manganese-dependent_inorganic_pyrophosphatase_[Bacillus_cereus]	CPTF_zcontrol	2206838.9	323118.1233	14.64167245
UIJ68941.1	GNAT_family_N-acetyltransferase_[Bacillus_cereus]	CPTF_Al	20479.7	17737.5112	86.61021012
UIJ68941.1	GNAT_family_N-acetyltransferase_[Bacillus_cereus]	CPTF_Cd	34481.93333	6494.590522	18.83476329
UIJ68941.1	GNAT_family_N-acetyltransferase_[Bacillus_cereus]	CPTF_Co	27067.53333	37117.95233	137.130901
UIJ68941.1	GNAT_family_N-acetyltransferase_[Bacillus_cereus]	CPTF_Cu	50904.66667	88169.46901	173.2050808
UIJ68941.1	GNAT_family_N-acetyltransferase_[Bacillus_cereus]	CPTF_Fe	0	0	0
UIJ68941.1	GNAT_family_N-acetyltransferase_[Bacillus_cereus]	CPTF_Mn	0	0	0
UIJ68941.1	GNAT_family_N-acetyltransferase_[Bacillus_cereus]	CPTF_Ni	54847.66667	94998.94534	173.2050808
UIJ68941.1	GNAT_family_N-acetyltransferase_[Bacillus_cereus]	CPTF_U	28855.6	27388.99974	94.91745013
UIJ68941.1	GNAT_family_N-acetyltransferase_[Bacillus_cereus]	CPTF_metals_mix	16276.73333	28192.12911	173.2050808
UIJ68941.1	GNAT_family_N-acetyltransferase_[Bacillus_cereus]	CPTF_zcontrol	10218.23333	17698.4993	173.2050808
UIJ68981.1	HAD_hydrolase-like_protein_[Bacillus_cereus]	CPTF_Al	0	0	0
UIJ68981.1	HAD_hydrolase-like_protein_[Bacillus_cereus]	CPTF_Cd	0	0	0
UIJ68981.1	HAD_hydrolase-like_protein_[Bacillus_cereus]	CPTF_Co	0	0	0
UIJ68981.1	HAD_hydrolase-like_protein_[Bacillus_cereus]	CPTF_Cu	0	0	0
UIJ68981.1	HAD_hydrolase-like_protein_[Bacillus_cereus]	CPTF_Fe	0	0	0
UIJ68981.1	HAD_hydrolase-like_protein_[Bacillus_cereus]	CPTF_Mn	0	0	0
UIJ68981.1	HAD_hydrolase-like_protein_[Bacillus_cereus]	CPTF_Ni	0	0	0
UIJ68981.1	HAD_hydrolase-like_protein_[Bacillus_cereus]	CPTF_U	0	0	0
UIJ68981.1	HAD_hydrolase-like_protein_[Bacillus_cereus]	CPTF_metals_mix	0	0	0
UIJ68981.1	HAD_hydrolase-like_protein_[Bacillus_cereus]	CPTF_zcontrol	11115.76667	19253.07263	173.2050808
UIJ69000.1	cytosolic_protein_[Bacillus_cereus]	CPTF_Al	0	0	0
UIJ69000.1	cytosolic_protein_[Bacillus_cereus]	CPTF_Cd	0	0	0
UIJ69000.1	cytosolic_protein_[Bacillus_cereus]	CPTF_Co	17297.26667	29959.7447	173.2050808
UIJ69000.1	cytosolic_protein_[Bacillus_cereus]	CPTF_Cu	0	0	0
UIJ69000.1	cytosolic_protein_[Bacillus_cereus]	CPTF_Fe	0	0	0
UIJ69000.1	cytosolic_protein_[Bacillus_cereus]	CPTF_Mn	0	0	0
UIJ69000.1	cytosolic_protein_[Bacillus_cereus]	CPTF_Ni	0	0	0
UIJ69000.1	cytosolic_protein_[Bacillus_cereus]	CPTF_U	0	0	0
UIJ69000.1	cytosolic_protein_[Bacillus_cereus]	CPTF_metals_mix	100540.4	24194.27541	24.0642323
UIJ69000.1	cytosolic_protein_[Bacillus_cereus]	CPTF_zcontrol	0	0	0
UIJ69004.1	class_I_SAM-dependent_methyltransferase_[Bacillus_cereus]	CPTF_Al	37273.6	3677.479274	9.866176795
UIJ69004.1	class_I_SAM-dependent_methyltransferase_[Bacillus_cereus]	CPTF_Cd	47339.1	3820.726805	8.070974744
UIJ69004.1	class_I_SAM-dependent_methyltransferase_[Bacillus_cereus]	CPTF_Co	41783.76667	15863.22272	37.96503759
UIJ69004.1	class_I_SAM-dependent_methyltransferase_[Bacillus_cereus]	CPTF_Cu	61876.7	24764.72604	40.02270005
UIJ69004.1	class_I_SAM-dependent_methyltransferase_[Bacillus_cereus]	CPTF_Fe	57989.9	23282.88802	40.149902
UIJ69004.1	class_I_SAM-dependent_methyltransferase_[Bacillus_cereus]	CPTF_Mn	31938.4	55318.93151	173.2050808
UIJ69004.1	class_I_SAM-dependent_methyltransferase_[Bacillus_cereus]	CPTF_Ni	83877.63333	14321.82228	17.07466187
UIJ69004.1	class_I_SAM-dependent_methyltransferase_[Bacillus_cereus]	CPTF_U	39879.16667	35183.83936	88.22611479
UIJ69004.1	class_I_SAM-dependent_methyltransferase_[Bacillus_cereus]	CPTF_metals_mix	21732.26667	2216.805831	10.20052747
UIJ69004.1	class_I_SAM-dependent_methyltransferase_[Bacillus_cereus]	CPTF_zcontrol	38462.36667	35328.64107	91.85248889
UIJ69005.1	hypothetical_protein_LW858_12670_[Bacillus_cereus]	CPTF_Al	2923333.333	576570.3195	19.723044
UIJ69005.1	hypothetical_protein_LW858_12670_[Bacillus_cereus]	CPTF_Cd	2433333.333	353459.0971	14.52571632
UIJ69005.1	hypothetical_protein_LW858_12670_[Bacillus_cereus]	CPTF_Co	2793333.333	245831.9209	8.800665426
UIJ69005.1	hypothetical_protein_LW858_12670_[Bacillus_cereus]	CPTF_Cu	2930000	149331.8452	5.096650008
UIJ69005.1	hypothetical_protein_LW858_12670_[Bacillus_cereus]	CPTF_Fe	1916666.667	1663199.727	86.77563795
UIJ69005.1	hypothetical_protein_LW858_12670_[Bacillus_cereus]	CPTF_Mn	2560000	1319242.207	51.5328987
UIJ69005.1	hypothetical_protein_LW858_12670_[Bacillus_cereus]	CPTF_Ni	3610000	272213.1518	7.54053052
UIJ69005.1	hypothetical_protein_LW858_12670_[Bacillus_cereus]	CPTF_U	3493333.333	280237.9941	8.022079984
UIJ69005.1	hypothetical_protein_LW858_12670_[Bacillus_cereus]	CPTF_metals_mix	0	0	0
UIJ69005.1	hypothetical_protein_LW858_12670_[Bacillus_cereus]	CPTF_zcontrol	3340000	228691.9325	6.847063848
UIJ69011.1	LL-diaminopimelate_aminotransferase_[Bacillus_cereus]	CPTF_Al	352460.4	116543.8095	33.06578825
UIJ69011.1	LL-diaminopimelate_aminotransferase_[Bacillus_cereus]	CPTF_Cd	223177.3333	31076.14835	13.9244196
UIJ69011.1	LL-diaminopimelate_aminotransferase_[Bacillus_cereus]	CPTF_Co	272844.5333	40825.45293	14.96289936
UIJ69011.1	LL-diaminopimelate_aminotransferase_[Bacillus_cereus]	CPTF_Cu	435899.5667	120626.204	27.67293505
UIJ69011.1	LL-diaminopimelate_aminotransferase_[Bacillus_cereus]	CPTF_Fe	279164.0667	16962.54821	6.076193263
UIJ69011.1	LL-diaminopimelate_aminotransferase_[Bacillus_cereus]	CPTF_Mn	251253.3333	114838.1845	45.70613372

UIJ69011.1	LL-diaminopimelate_aminotransferase_[Bacillus_cereus]	CPTF_Ni	313563.8667	65483.30526	20.88356224
UIJ69011.1	LL-diaminopimelate_aminotransferase_[Bacillus_cereus]	CPTF_U	321396	19767.76019	6.150593098
UIJ69011.1	LL-diaminopimelate_aminotransferase_[Bacillus_cereus]	CPTF_metals_mix	374467.3667	40933.49245	10.93112407
UIJ69011.1	LL-diaminopimelate_aminotransferase_[Bacillus_cereus]	CPTF_zcontrol	272973.9667	56078.85368	20.54366369
UIJ69012.1	beta-hydroxyacyl-ACP_dehydratase_[Bacillus_cereus]	CPTF_Al	269318.4667	23763.12468	8.823429368
UIJ69012.1	beta-hydroxyacyl-ACP_dehydratase_[Bacillus_cereus]	CPTF_Cd	163852.4667	99647.88041	60.81561202
UIJ69012.1	beta-hydroxyacyl-ACP_dehydratase_[Bacillus_cereus]	CPTF_Co	234814.1	62512.36537	26.6220663
UIJ69012.1	beta-hydroxyacyl-ACP_dehydratase_[Bacillus_cereus]	CPTF_Cu	272135.3333	53445.37006	19.63926161
UIJ69012.1	beta-hydroxyacyl-ACP_dehydratase_[Bacillus_cereus]	CPTF_Fe	261265.3333	100970.2799	38.64664271
UIJ69012.1	beta-hydroxyacyl-ACP_dehydratase_[Bacillus_cereus]	CPTF_Mn	97803.66667	169400.9198	173.2050808
UIJ69012.1	beta-hydroxyacyl-ACP_dehydratase_[Bacillus_cereus]	CPTF_Ni	134412.9333	133230.0874	99.119991
UIJ69012.1	beta-hydroxyacyl-ACP_dehydratase_[Bacillus_cereus]	CPTF_U	71175.86667	123280.2173	173.2050808
UIJ69012.1	beta-hydroxyacyl-ACP_dehydratase_[Bacillus_cereus]	CPTF_metals_mix	21221.13333	36756.08113	173.2050808
UIJ69012.1	beta-hydroxyacyl-ACP_dehydratase_[Bacillus_cereus]	CPTF_zcontrol	118165.1667	122747.8199	103.8781761
UIJ69025.1	FMNH2-dependent_alkanesulfonate_monooxygenase_[Bacillus_cereus]	CPTF_Al	62634.1	7104.200505	11.34238459
UIJ69025.1	FMNH2-dependent_alkanesulfonate_monooxygenase_[Bacillus_cereus]	CPTF_Cd	59927.56667	9533.133944	15.90776078
UIJ69025.1	FMNH2-dependent_alkanesulfonate_monooxygenase_[Bacillus_cereus]	CPTF_Co	44766.56667	38772.5392	86.61048207
UIJ69025.1	FMNH2-dependent_alkanesulfonate_monooxygenase_[Bacillus_cereus]	CPTF_Cu	158880.8333	14218.89244	8.94940701
UIJ69025.1	FMNH2-dependent_alkanesulfonate_monooxygenase_[Bacillus_cereus]	CPTF_Fe	81381.6	60221.10599	73.99842961
UIJ69025.1	FMNH2-dependent_alkanesulfonate_monooxygenase_[Bacillus_cereus]	CPTF_Mn	43996.71	67781.90807	154.0613106
UIJ69025.1	FMNH2-dependent_alkanesulfonate_monooxygenase_[Bacillus_cereus]	CPTF_Ni	143738.6667	15367.28103	10.69112535
UIJ69025.1	FMNH2-dependent_alkanesulfonate_monooxygenase_[Bacillus_cereus]	CPTF_U	115831.8333	60953.67955	52.62256307
UIJ69025.1	FMNH2-dependent_alkanesulfonate_monooxygenase_[Bacillus_cereus]	CPTF_metals_mix	0	0	0
UIJ69025.1	FMNH2-dependent_alkanesulfonate_monooxygenase_[Bacillus_cereus]	CPTF_zcontrol	110483.3	44716.32679	40.47338086
UIJ69034.1	hypothetical_protein_LW858_12820_[Bacillus_cereus]	CPTF_Al	0	0	0
UIJ69034.1	hypothetical_protein_LW858_12820_[Bacillus_cereus]	CPTF_Cd	0	0	0
UIJ69034.1	hypothetical_protein_LW858_12820_[Bacillus_cereus]	CPTF_Co	0	0	0
UIJ69034.1	hypothetical_protein_LW858_12820_[Bacillus_cereus]	CPTF_Cu	0	0	0
UIJ69034.1	hypothetical_protein_LW858_12820_[Bacillus_cereus]	CPTF_Fe	12298.03333	21300.81857	173.2050808
UIJ69034.1	hypothetical_protein_LW858_12820_[Bacillus_cereus]	CPTF_Mn	0	0	0
UIJ69034.1	hypothetical_protein_LW858_12820_[Bacillus_cereus]	CPTF_Ni	0	0	0
UIJ69034.1	hypothetical_protein_LW858_12820_[Bacillus_cereus]	CPTF_U	0	0	0
UIJ69034.1	hypothetical_protein_LW858_12820_[Bacillus_cereus]	CPTF_metals_mix	5286	9155.620569	173.2050808
UIJ69034.1	hypothetical_protein_LW858_12820_[Bacillus_cereus]	CPTF_zcontrol	0	0	0
UIJ69040.1	nucleotidyltransferase_domain-containing_protein_[Bacillus_cereus]	CPTF_Al	8779.266667	15206.13592	173.2050808
UIJ69040.1	nucleotidyltransferase_domain-containing_protein_[Bacillus_cereus]	CPTF_Cd	9322.666667	16147.33233	173.2050808
UIJ69040.1	nucleotidyltransferase_domain-containing_protein_[Bacillus_cereus]	CPTF_Co	5348.4	9263.700539	173.2050808
UIJ69040.1	nucleotidyltransferase_domain-containing_protein_[Bacillus_cereus]	CPTF_Cu	0	0	0
UIJ69040.1	nucleotidyltransferase_domain-containing_protein_[Bacillus_cereus]	CPTF_Fe	9693.366667	16789.40356	173.2050808
UIJ69040.1	nucleotidyltransferase_domain-containing_protein_[Bacillus_cereus]	CPTF_Mn	0	0	0
UIJ69040.1	nucleotidyltransferase_domain-containing_protein_[Bacillus_cereus]	CPTF_Ni	0	0	0
UIJ69040.1	nucleotidyltransferase_domain-containing_protein_[Bacillus_cereus]	CPTF_U	0	0	0
UIJ69040.1	nucleotidyltransferase_domain-containing_protein_[Bacillus_cereus]	CPTF_metals_mix	0	0	0
UIJ69040.1	nucleotidyltransferase_domain-containing_protein_[Bacillus_cereus]	CPTF_zcontrol	0	0	0
UIJ69042.1	pyridoxal-phosphate_dependent_enzyme_[Bacillus_cereus]	CPTF_Al	26360.46667	45657.66758	173.2050808
UIJ69042.1	pyridoxal-phosphate_dependent_enzyme_[Bacillus_cereus]	CPTF_Cd	10479.8	18151.54605	173.2050808
UIJ69042.1	pyridoxal-phosphate_dependent_enzyme_[Bacillus_cereus]	CPTF_Co	92631.56667	116811.5328	126.103376
UIJ69042.1	pyridoxal-phosphate_dependent_enzyme_[Bacillus_cereus]	CPTF_Cu	63896.56667	74042.15831	115.8781483
UIJ69042.1	pyridoxal-phosphate_dependent_enzyme_[Bacillus_cereus]	CPTF_Fe	0	0	0
UIJ69042.1	pyridoxal-phosphate_dependent_enzyme_[Bacillus_cereus]	CPTF_Mn	53955.33333	93453.37867	173.2050808
UIJ69042.1	pyridoxal-phosphate_dependent_enzyme_[Bacillus_cereus]	CPTF_Ni	0	0	0
UIJ69042.1	pyridoxal-phosphate_dependent_enzyme_[Bacillus_cereus]	CPTF_U	0	0	0
UIJ69042.1	pyridoxal-phosphate_dependent_enzyme_[Bacillus_cereus]	CPTF_metals_mix	16364.6	28344.31865	173.2050808
UIJ69042.1	pyridoxal-phosphate_dependent_enzyme_[Bacillus_cereus]	CPTF_zcontrol	0	0	0
UIJ69043.1	VOC_family_protein_[Bacillus_cereus]	CPTF_Al	5489383.833	403231.2388	7.345655743
UIJ69043.1	VOC_family_protein_[Bacillus_cereus]	CPTF_Cd	3802538.767	263834.0406	6.93836557
UIJ69043.1	VOC_family_protein_[Bacillus_cereus]	CPTF_Co	5289890.843	471645.7346	8.915982362
UIJ69043.1	VOC_family_protein_[Bacillus_cereus]	CPTF_Cu	7349014.6	796122.7902	10.83305495
UIJ69043.1	VOC_family_protein_[Bacillus_cereus]	CPTF_Fe	4448102.033	345902.2926	7.776401935
UIJ69043.1	VOC_family_protein_[Bacillus_cereus]	CPTF_Mn	5170607.767	450881.5434	8.720087923
UIJ69043.1	VOC_family_protein_[Bacillus_cereus]	CPTF_Ni	5606090.633	477289.5616	8.513768199

UJJ69043.1	VOC_family_protein_[Bacillus_cereus]	CPTF_U	5051190.333	1069211.187	21.16750936
UJJ69043.1	VOC_family_protein_[Bacillus_cereus]	CPTF_metals_mix	6990246.1	446219.5906	6.383460385
UJJ69043.1	VOC_family_protein_[Bacillus_cereus]	CPTF_zcontrol	5412017.967	493044.2126	9.11017324
UJJ69044.1	MBL_fold_metallo-hydrolase_[Bacillus_cereus]	CPTF_Al	687861.4	46843.06038	6.809956247
UJJ69044.1	MBL_fold_metallo-hydrolase_[Bacillus_cereus]	CPTF_Cd	554424.6	100877.7832	18.19504098
UJJ69044.1	MBL_fold_metallo-hydrolase_[Bacillus_cereus]	CPTF_Co	734574.3	82465.59569	11.22631103
UJJ69044.1	MBL_fold_metallo-hydrolase_[Bacillus_cereus]	CPTF_Cu	1114499.967	156959.3823	14.08339049
UJJ69044.1	MBL_fold_metallo-hydrolase_[Bacillus_cereus]	CPTF_Fe	537961.9	36541.92132	6.792659725
UJJ69044.1	MBL_fold_metallo-hydrolase_[Bacillus_cereus]	CPTF_Mn	660947.6667	92441.57321	13.98621674
UJJ69044.1	MBL_fold_metallo-hydrolase_[Bacillus_cereus]	CPTF_Ni	669871.6667	76715.9104	11.45232949
UJJ69044.1	MBL_fold_metallo-hydrolase_[Bacillus_cereus]	CPTF_U	609407.3333	294481.1523	48.32254819
UJJ69044.1	MBL_fold_metallo-hydrolase_[Bacillus_cereus]	CPTF_metals_mix	2279685.067	161584.6137	7.08802352
UJJ69044.1	MBL_fold_metallo-hydrolase_[Bacillus_cereus]	CPTF_zcontrol	657294	162238.9404	24.68285734
UJJ69045.1	polysaccharide_deacetylase_[Bacillus_cereus]	CPTF_Al	15033.33333	26038.49714	173.2050808
UJJ69045.1	polysaccharide_deacetylase_[Bacillus_cereus]	CPTF_Cd	121406	110349.8103	90.89320984
UJJ69045.1	polysaccharide_deacetylase_[Bacillus_cereus]	CPTF_Co	11293.86667	19561.55088	173.2050808
UJJ69045.1	polysaccharide_deacetylase_[Bacillus_cereus]	CPTF_Cu	0	0	0
UJJ69045.1	polysaccharide_deacetylase_[Bacillus_cereus]	CPTF_Fe	73731.66667	127706.9928	173.2050808
UJJ69045.1	polysaccharide_deacetylase_[Bacillus_cereus]	CPTF_Mn	78721.16667	136349.0603	173.2050808
UJJ69045.1	polysaccharide_deacetylase_[Bacillus_cereus]	CPTF_Ni	16866.2	29213.11533	173.2050808
UJJ69045.1	polysaccharide_deacetylase_[Bacillus_cereus]	CPTF_U	0	0	0
UJJ69045.1	polysaccharide_deacetylase_[Bacillus_cereus]	CPTF_metals_mix	198542.3667	118032.6887	59.44962314
UJJ69045.1	polysaccharide_deacetylase_[Bacillus_cereus]	CPTF_zcontrol	0	0	0
UJJ69049.1	ATP-binding_cassette_domain-containing_protein_[Bacillus_cereus]	CPTF_Al	1264360.367	450847.1017	35.65811722
UJJ69049.1	ATP-binding_cassette_domain-containing_protein_[Bacillus_cereus]	CPTF_Cd	1189771.533	399605.1089	33.58670952
UJJ69049.1	ATP-binding_cassette_domain-containing_protein_[Bacillus_cereus]	CPTF_Co	1422548.033	199352.5262	14.01376415
UJJ69049.1	ATP-binding_cassette_domain-containing_protein_[Bacillus_cereus]	CPTF_Cu	1411204.8	159182.1417	11.2798753
UJJ69049.1	ATP-binding_cassette_domain-containing_protein_[Bacillus_cereus]	CPTF_Fe	1334143.933	235064.9781	17.61916179
UJJ69049.1	ATP-binding_cassette_domain-containing_protein_[Bacillus_cereus]	CPTF_Mn	1085539.067	124309.8638	11.45144082
UJJ69049.1	ATP-binding_cassette_domain-containing_protein_[Bacillus_cereus]	CPTF_Ni	1116838.867	143684.8211	12.86531347
UJJ69049.1	ATP-binding_cassette_domain-containing_protein_[Bacillus_cereus]	CPTF_U	1029516.7	239914.9106	23.30364438
UJJ69049.1	ATP-binding_cassette_domain-containing_protein_[Bacillus_cereus]	CPTF_metals_mix	1890475.333	208103.6745	11.00800793
UJJ69049.1	ATP-binding_cassette_domain-containing_protein_[Bacillus_cereus]	CPTF_zcontrol	1298809.2	131697.4159	10.13985857
UJJ69050.1	hypothetical_protein_LW858_12905_[Bacillus_cereus]	CPTF_Al	18247.8	16002.31097	87.69446712
UJJ69050.1	hypothetical_protein_LW858_12905_[Bacillus_cereus]	CPTF_Cd	26443.53333	22958.40951	86.82050623
UJJ69050.1	hypothetical_protein_LW858_12905_[Bacillus_cereus]	CPTF_Co	24027.46667	6208.841668	25.84060049
UJJ69050.1	hypothetical_protein_LW858_12905_[Bacillus_cereus]	CPTF_Cu	44098.33333	17436.04733	39.53901657
UJJ69050.1	hypothetical_protein_LW858_12905_[Bacillus_cereus]	CPTF_Fe	17182.13333	14974.35384	87.15072542
UJJ69050.1	hypothetical_protein_LW858_12905_[Bacillus_cereus]	CPTF_Mn	10453.03333	18105.18483	173.2050808
UJJ69050.1	hypothetical_protein_LW858_12905_[Bacillus_cereus]	CPTF_Ni	20469.5	20767.81562	101.4573664
UJJ69050.1	hypothetical_protein_LW858_12905_[Bacillus_cereus]	CPTF_U	0	0	0
UJJ69050.1	hypothetical_protein_LW858_12905_[Bacillus_cereus]	CPTF_metals_mix	56689.33333	19541.914	34.47194182
UJJ69050.1	hypothetical_protein_LW858_12905_[Bacillus_cereus]	CPTF_zcontrol	8202.333333	14206.85807	173.2050808
UJJ69053.1	GNAT_family_N-acetyltransferase_[Bacillus_cereus]	CPTF_Al	0	0	0
UJJ69053.1	GNAT_family_N-acetyltransferase_[Bacillus_cereus]	CPTF_Cd	62653.9	54651.04826	87.22688972
UJJ69053.1	GNAT_family_N-acetyltransferase_[Bacillus_cereus]	CPTF_Co	121944	110270.0494	90.42679377
UJJ69053.1	GNAT_family_N-acetyltransferase_[Bacillus_cereus]	CPTF_Cu	13382.66667	23179.45861	173.2050808
UJJ69053.1	GNAT_family_N-acetyltransferase_[Bacillus_cereus]	CPTF_Fe	32528.2	56340.49508	173.2050808
UJJ69053.1	GNAT_family_N-acetyltransferase_[Bacillus_cereus]	CPTF_Mn	41714.33333	72251.34474	173.2050808
UJJ69053.1	GNAT_family_N-acetyltransferase_[Bacillus_cereus]	CPTF_Ni	40022.66667	69321.29212	173.2050808
UJJ69053.1	GNAT_family_N-acetyltransferase_[Bacillus_cereus]	CPTF_U	0	0	0
UJJ69053.1	GNAT_family_N-acetyltransferase_[Bacillus_cereus]	CPTF_metals_mix	52263.56667	54839.94758	104.9295926
UJJ69053.1	GNAT_family_N-acetyltransferase_[Bacillus_cereus]	CPTF_zcontrol	36920	63947.31582	173.2050808
UJJ69054.1	3-phosphoshikimate_1-carboxyvinyltransferase_[Bacillus_cereus]	CPTF_Al	2801465.567	103971.1253	3.711311912
UJJ69054.1	3-phosphoshikimate_1-carboxyvinyltransferase_[Bacillus_cereus]	CPTF_Cd	3966182.133	1090304.794	27.49003343
UJJ69054.1	3-phosphoshikimate_1-carboxyvinyltransferase_[Bacillus_cereus]	CPTF_Co	3557709.1	1127110.382	31.68079092
UJJ69054.1	3-phosphoshikimate_1-carboxyvinyltransferase_[Bacillus_cereus]	CPTF_Cu	2828070.2	112615.6673	3.982067605
UJJ69054.1	3-phosphoshikimate_1-carboxyvinyltransferase_[Bacillus_cereus]	CPTF_Fe	3013277.8	466907.4752	15.49500266
UJJ69054.1	3-phosphoshikimate_1-carboxyvinyltransferase_[Bacillus_cereus]	CPTF_Mn	3124873.867	698151.416	22.34174708
UJJ69054.1	3-phosphoshikimate_1-carboxyvinyltransferase_[Bacillus_cereus]	CPTF_Ni	2682254.733	824335.8523	30.73294427
UJJ69054.1	3-phosphoshikimate_1-carboxyvinyltransferase_[Bacillus_cereus]	CPTF_U	2540567.333	173456.4532	6.827469238

UIJ69054.1	3-phosphoshikimate_1-carboxyvinyltransferase_[Bacillus_cereus]	CPTF_metals_mix	4574688.467	150954.8048	3.299783272
UIJ69054.1	3-phosphoshikimate_1-carboxyvinyltransferase_[Bacillus_cereus]	CPTF_zcontrol	2879315.133	162318.0111	5.637382627
UIJ69055.1	prephenate_dehydrogenase_[Bacillus_cereus]	CPTF_Al	308176.1	127477.1765	41.36504307
UIJ69055.1	prephenate_dehydrogenase_[Bacillus_cereus]	CPTF_Cd	297232.7	55899.20761	18.80654706
UIJ69055.1	prephenate_dehydrogenase_[Bacillus_cereus]	CPTF_Co	427696.8667	136390.1711	31.88944827
UIJ69055.1	prephenate_dehydrogenase_[Bacillus_cereus]	CPTF_Cu	386766.0333	46530.15288	12.03056858
UIJ69055.1	prephenate_dehydrogenase_[Bacillus_cereus]	CPTF_Fe	344894.6667	88763.22319	25.73632815
UIJ69055.1	prephenate_dehydrogenase_[Bacillus_cereus]	CPTF_Mn	301025.6667	77000.14087	25.57926097
UIJ69055.1	prephenate_dehydrogenase_[Bacillus_cereus]	CPTF_Ni	227094.3333	229322.0505	100.9809655
UIJ69055.1	prephenate_dehydrogenase_[Bacillus_cereus]	CPTF_U	307119.6667	154074.5008	50.16757879
UIJ69055.1	prephenate_dehydrogenase_[Bacillus_cereus]	CPTF_metals_mix	205772.6667	48368.6296	23.5058574
UIJ69055.1	prephenate_dehydrogenase_[Bacillus_cereus]	CPTF_zcontrol	430330.9667	37393.02369	8.689363904
UIJ69056.1	histidinol-phosphate_transaminase_[Bacillus_cereus]	CPTF_Al	13350.9	23124.43713	173.2050808
UIJ69056.1	histidinol-phosphate_transaminase_[Bacillus_cereus]	CPTF_Cd	12352.73333	21395.56175	173.2050808
UIJ69056.1	histidinol-phosphate_transaminase_[Bacillus_cereus]	CPTF_Co	324144.6333	74657.60322	23.03218858
UIJ69056.1	histidinol-phosphate_transaminase_[Bacillus_cereus]	CPTF_Cu	77864.83333	134865.8475	173.2050808
UIJ69056.1	histidinol-phosphate_transaminase_[Bacillus_cereus]	CPTF_Fe	55220.16667	95644.13427	173.2050808
UIJ69056.1	histidinol-phosphate_transaminase_[Bacillus_cereus]	CPTF_Mn	63567.06667	61836.07157	97.27689952
UIJ69056.1	histidinol-phosphate_transaminase_[Bacillus_cereus]	CPTF_Ni	23484.83333	23088.56603	98.31266716
UIJ69056.1	histidinol-phosphate_transaminase_[Bacillus_cereus]	CPTF_U	27574.36667	47760.20405	173.2050808
UIJ69056.1	histidinol-phosphate_transaminase_[Bacillus_cereus]	CPTF_metals_mix	589337.5	67657.7784	11.48031109
UIJ69056.1	histidinol-phosphate_transaminase_[Bacillus_cereus]	CPTF_zcontrol	80395.1	139248.3979	173.2050808
UIJ69057.1	chorismate_synthase_[Bacillus_cereus]	CPTF_Al	179341.6667	195646.9899	109.0917652
UIJ69057.1	chorismate_synthase_[Bacillus_cereus]	CPTF_Cd	505260.6667	64195.66743	12.70545516
UIJ69057.1	chorismate_synthase_[Bacillus_cereus]	CPTF_Co	641267.2333	179007.1812	27.9145997
UIJ69057.1	chorismate_synthase_[Bacillus_cereus]	CPTF_Cu	600885	83180.1656	13.84294259
UIJ69057.1	chorismate_synthase_[Bacillus_cereus]	CPTF_Fe	293041.7333	260131.7772	88.76953268
UIJ69057.1	chorismate_synthase_[Bacillus_cereus]	CPTF_Mn	379869.4333	165845.5498	43.65856668
UIJ69057.1	chorismate_synthase_[Bacillus_cereus]	CPTF_Ni	540593.6667	98681.14204	18.25421719
UIJ69057.1	chorismate_synthase_[Bacillus_cereus]	CPTF_U	278383.5667	199728.7366	71.74587891
UIJ69057.1	chorismate_synthase_[Bacillus_cereus]	CPTF_metals_mix	927406.1167	158933.1786	17.13738736
UIJ69057.1	chorismate_synthase_[Bacillus_cereus]	CPTF_zcontrol	270456.6	159973.3661	59.14936671
UIJ69058.1	bifunctional_3-deoxy-7-phosphoheptulonate_synthase/chorismate_mutase_[Bacillus_cereus]	CPTF_Al	10445937.07	711834.6976	6.814464734
UIJ69058.1	bifunctional_3-deoxy-7-phosphoheptulonate_synthase/chorismate_mutase_[Bacillus_cereus]	CPTF_Cd	12173122.5	1168705.84	9.600707129
UIJ69058.1	bifunctional_3-deoxy-7-phosphoheptulonate_synthase/chorismate_mutase_[Bacillus_cereus]	CPTF_Co	13584587.7	1214916.613	8.943345501
UIJ69058.1	bifunctional_3-deoxy-7-phosphoheptulonate_synthase/chorismate_mutase_[Bacillus_cereus]	CPTF_Cu	11118556.97	477678.077	4.296223677
UIJ69058.1	bifunctional_3-deoxy-7-phosphoheptulonate_synthase/chorismate_mutase_[Bacillus_cereus]	CPTF_Fe	10760603.67	909522.943	8.452341255
UIJ69058.1	bifunctional_3-deoxy-7-phosphoheptulonate_synthase/chorismate_mutase_[Bacillus_cereus]	CPTF_Mn	10774987.93	2389755.387	22.17872912
UIJ69058.1	bifunctional_3-deoxy-7-phosphoheptulonate_synthase/chorismate_mutase_[Bacillus_cereus]	CPTF_Ni	8734311.9	359017.2518	4.110423991
UIJ69058.1	bifunctional_3-deoxy-7-phosphoheptulonate_synthase/chorismate_mutase_[Bacillus_cereus]	CPTF_U	9778829.467	398281.6374	4.072896851
UIJ69058.1	bifunctional_3-deoxy-7-phosphoheptulonate_synthase/chorismate_mutase_[Bacillus_cereus]	CPTF_metals_mix	15989759.7	1078435.315	6.744537346
UIJ69058.1	bifunctional_3-deoxy-7-phosphoheptulonate_synthase/chorismate_mutase_[Bacillus_cereus]	CPTF_zcontrol	10279374.47	852695.58	8.295208846
UIJ69065.1	SH3_domain-containing_protein_[Bacillus_cereus]	CPTF_Al	0	0	0
UIJ69065.1	SH3_domain-containing_protein_[Bacillus_cereus]	CPTF_Cd	69706	120734.3336	173.2050808
UIJ69065.1	SH3_domain-containing_protein_[Bacillus_cereus]	CPTF_Co	0	0	0
UIJ69065.1	SH3_domain-containing_protein_[Bacillus_cereus]	CPTF_Cu	0	0	0
UIJ69065.1	SH3_domain-containing_protein_[Bacillus_cereus]	CPTF_Fe	0	0	0
UIJ69065.1	SH3_domain-containing_protein_[Bacillus_cereus]	CPTF_Mn	0	0	0
UIJ69065.1	SH3_domain-containing_protein_[Bacillus_cereus]	CPTF_Ni	0	0	0
UIJ69065.1	SH3_domain-containing_protein_[Bacillus_cereus]	CPTF_U	0	0	0
UIJ69065.1	SH3_domain-containing_protein_[Bacillus_cereus]	CPTF_metals_mix	71779.76667	47622.81763	66.34574037
UIJ69065.1	SH3_domain-containing_protein_[Bacillus_cereus]	CPTF_zcontrol	0	0	0
UIJ69070.1	DUF2332_domain-containing_protein_[Bacillus_cereus]	CPTF_Al	2177257	102094.4775	4.689133048
UIJ69070.1	DUF2332_domain-containing_protein_[Bacillus_cereus]	CPTF_Cd	1997822.633	159339.0633	7.975636109
UIJ69070.1	DUF2332_domain-containing_protein_[Bacillus_cereus]	CPTF_Co	1634682.333	47845.85594	2.926920721
UIJ69070.1	DUF2332_domain-containing_protein_[Bacillus_cereus]	CPTF_Cu	1989832.4	195460.0762	9.822941679
UIJ69070.1	DUF2332_domain-containing_protein_[Bacillus_cereus]	CPTF_Fe	1824054.567	286203.22	15.69049662
UIJ69070.1	DUF2332_domain-containing_protein_[Bacillus_cereus]	CPTF_Mn	1794594.667	249762.7264	13.91750076
UIJ69070.1	DUF2332_domain-containing_protein_[Bacillus_cereus]	CPTF_Ni	1821642.533	28937.55421	1.588541862
UIJ69070.1	DUF2332_domain-containing_protein_[Bacillus_cereus]	CPTF_U	1767269.533	521583.4615	29.51352081
UIJ69070.1	DUF2332_domain-containing_protein_[Bacillus_cereus]	CPTF_metals_mix	1025036.2	114205.1142	11.14156887

UIJ69070.1	DUF2332_domain-containing_protein_[Bacillus_cereus]	CPTF_zcontrol	1569063.2	39755.38017	2.533701649
UIJ69071.1	malate:quinone_oxidoreductase_[Bacillus_cereus]	CPTF_Al	2111459.667	459489.9203	21.76171904
UIJ69071.1	malate:quinone_oxidoreductase_[Bacillus_cereus]	CPTF_Cd	1695123.867	395685.1377	23.34255009
UIJ69071.1	malate:quinone_oxidoreductase_[Bacillus_cereus]	CPTF_Co	1978376.473	452615.6992	22.87813797
UIJ69071.1	malate:quinone_oxidoreductase_[Bacillus_cereus]	CPTF_Cu	1623959.9	79119.31497	4.8719993
UIJ69071.1	malate:quinone_oxidoreductase_[Bacillus_cereus]	CPTF_Fe	1848253.267	517082.6405	27.97682816
UIJ69071.1	malate:quinone_oxidoreductase_[Bacillus_cereus]	CPTF_Mn	1424047.133	136097.7163	9.557107566
UIJ69071.1	malate:quinone_oxidoreductase_[Bacillus_cereus]	CPTF_Ni	1674695.667	37770.71649	2.255377932
UIJ69071.1	malate:quinone_oxidoreductase_[Bacillus_cereus]	CPTF_U	1483520.5	380143.2914	25.62440434
UIJ69071.1	malate:quinone_oxidoreductase_[Bacillus_cereus]	CPTF_metals_mix	2637182.367	26490.80906	1.004511838
UIJ69071.1	malate:quinone_oxidoreductase_[Bacillus_cereus]	CPTF_zcontrol	1560263.933	227563.5237	14.58493777
UIJ69081.1	heme-degrading_monooxygenase_HmoA_[Bacillus_cereus]	CPTF_Al	199360.3333	38450.93518	19.28715434
UIJ69081.1	heme-degrading_monooxygenase_HmoA_[Bacillus_cereus]	CPTF_Cd	309970.5	54925.168	17.71948234
UIJ69081.1	heme-degrading_monooxygenase_HmoA_[Bacillus_cereus]	CPTF_Co	347949.7333	113498.7836	32.61930466
UIJ69081.1	heme-degrading_monooxygenase_HmoA_[Bacillus_cereus]	CPTF_Cu	2316818.9	125237.6846	5.405588005
UIJ69081.1	heme-degrading_monooxygenase_HmoA_[Bacillus_cereus]	CPTF_Fe	201642.4	93847.95094	46.54177442
UIJ69081.1	heme-degrading_monooxygenase_HmoA_[Bacillus_cereus]	CPTF_Mn	196181.3	38550.59344	19.65049342
UIJ69081.1	heme-degrading_monooxygenase_HmoA_[Bacillus_cereus]	CPTF_Ni	198482.0333	22235.04127	11.20254609
UIJ69081.1	heme-degrading_monooxygenase_HmoA_[Bacillus_cereus]	CPTF_U	229183.3333	72859.12658	31.79076136
UIJ69081.1	heme-degrading_monooxygenase_HmoA_[Bacillus_cereus]	CPTF_metals_mix	4089762.8	141660.5523	3.463784068
UIJ69081.1	heme-degrading_monooxygenase_HmoA_[Bacillus_cereus]	CPTF_zcontrol	170489.3333	1144.07794	0.671055437
UIJ69085.1	glutamate-5-semialdehyde_dehydrogenase_[Bacillus_cereus]	CPTF_Al	0	0	0
UIJ69085.1	glutamate-5-semialdehyde_dehydrogenase_[Bacillus_cereus]	CPTF_Cd	309919.9	272966.5123	88.07647147
UIJ69085.1	glutamate-5-semialdehyde_dehydrogenase_[Bacillus_cereus]	CPTF_Co	141473	245038.4239	173.2050808
UIJ69085.1	glutamate-5-semialdehyde_dehydrogenase_[Bacillus_cereus]	CPTF_Cu	245159.3333	218304.016	89.04576997
UIJ69085.1	glutamate-5-semialdehyde_dehydrogenase_[Bacillus_cereus]	CPTF_Fe	158066	209789.6468	132.7228163
UIJ69085.1	glutamate-5-semialdehyde_dehydrogenase_[Bacillus_cereus]	CPTF_Mn	85877.4	100223.44	116.7052566
UIJ69085.1	glutamate-5-semialdehyde_dehydrogenase_[Bacillus_cereus]	CPTF_Ni	161607.3333	175401.4958	108.5356043
UIJ69085.1	glutamate-5-semialdehyde_dehydrogenase_[Bacillus_cereus]	CPTF_U	46555	80635.62535	173.2050808
UIJ69085.1	glutamate-5-semialdehyde_dehydrogenase_[Bacillus_cereus]	CPTF_metals_mix	30088.6	26078.31765	86.67175492
UIJ69085.1	glutamate-5-semialdehyde_dehydrogenase_[Bacillus_cereus]	CPTF_zcontrol	59034.33333	102250.4647	173.2050808
UIJ69088.1	M20_family_metallopeptidase_[Bacillus_cereus]	CPTF_Al	411528	393933.2177	95.72452365
UIJ69088.1	M20_family_metallopeptidase_[Bacillus_cereus]	CPTF_Cd	497229.3333	630086.786	126.7195525
UIJ69088.1	M20_family_metallopeptidase_[Bacillus_cereus]	CPTF_Co	565631.9	591015.3862	104.4876334
UIJ69088.1	M20_family_metallopeptidase_[Bacillus_cereus]	CPTF_Cu	376302.8	433309.3812	115.1491249
UIJ69088.1	M20_family_metallopeptidase_[Bacillus_cereus]	CPTF_Fe	569185.8333	385426.49	67.71540461
UIJ69088.1	M20_family_metallopeptidase_[Bacillus_cereus]	CPTF_Mn	234896	281453.7941	119.8205989
UIJ69088.1	M20_family_metallopeptidase_[Bacillus_cereus]	CPTF_Ni	483798.3333	196901.9314	40.69917523
UIJ69088.1	M20_family_metallopeptidase_[Bacillus_cereus]	CPTF_U	205980	263150.8422	127.7555307
UIJ69088.1	M20_family_metallopeptidase_[Bacillus_cereus]	CPTF_metals_mix	217498.2667	234063.5242	107.6162711
UIJ69088.1	M20_family_metallopeptidase_[Bacillus_cereus]	CPTF_zcontrol	265331.3333	32125.18673	12.1075737
UIJ69092.1	immune_inhibitor_A_[Bacillus_cereus]	CPTF_Al	1535332.667	342230.2892	22.29030207
UIJ69092.1	immune_inhibitor_A_[Bacillus_cereus]	CPTF_Cd	2269849.3	952455.6269	41.96118337
UIJ69092.1	immune_inhibitor_A_[Bacillus_cereus]	CPTF_Co	2243560.567	749012.5538	33.3849937
UIJ69092.1	immune_inhibitor_A_[Bacillus_cereus]	CPTF_Cu	734355.4	148659.3934	20.24352151
UIJ69092.1	immune_inhibitor_A_[Bacillus_cereus]	CPTF_Fe	1642620.9	710541.7498	43.25658768
UIJ69092.1	immune_inhibitor_A_[Bacillus_cereus]	CPTF_Mn	1552903.767	241435.7184	15.54737155
UIJ69092.1	immune_inhibitor_A_[Bacillus_cereus]	CPTF_Ni	1765825.433	574815.9168	32.55225041
UIJ69092.1	immune_inhibitor_A_[Bacillus_cereus]	CPTF_U	1431490.8	785048.9902	54.84135771
UIJ69092.1	immune_inhibitor_A_[Bacillus_cereus]	CPTF_metals_mix	225117.4	61030.74014	27.11062767
UIJ69092.1	immune_inhibitor_A_[Bacillus_cereus]	CPTF_zcontrol	1628162.733	289287.121	17.76770313
UIJ69098.1	HAD-IA_family_hydrolase_[Bacillus_cereus]	CPTF_Al	610748.1333	302795.2282	49.57775746
UIJ69098.1	HAD-IA_family_hydrolase_[Bacillus_cereus]	CPTF_Cd	750433.4333	509073.8176	67.83730509
UIJ69098.1	HAD-IA_family_hydrolase_[Bacillus_cereus]	CPTF_Co	654338.6667	66103.36505	10.1023168
UIJ69098.1	HAD-IA_family_hydrolase_[Bacillus_cereus]	CPTF_Cu	573274	245902.5801	42.89442398
UIJ69098.1	HAD-IA_family_hydrolase_[Bacillus_cereus]	CPTF_Fe	588600	178942.4665	30.40137045
UIJ69098.1	HAD-IA_family_hydrolase_[Bacillus_cereus]	CPTF_Mn	446644	311792.8834	69.80791937
UIJ69098.1	HAD-IA_family_hydrolase_[Bacillus_cereus]	CPTF_Ni	266589.0667	84756.34163	31.79287984
UIJ69098.1	HAD-IA_family_hydrolase_[Bacillus_cereus]	CPTF_U	286919.7	224742.9515	78.3295645
UIJ69098.1	HAD-IA_family_hydrolase_[Bacillus_cereus]	CPTF_metals_mix	317673	218092.7067	68.65320838
UIJ69098.1	HAD-IA_family_hydrolase_[Bacillus_cereus]	CPTF_zcontrol	348733.3333	302467.7581	86.73325122

UIJ69099.1	hypothetical_protein_LW858_13180_[Bacillus_cereus]	CPTF_Al	11313.66667	19595.84549	173.2050808
UIJ69099.1	hypothetical_protein_LW858_13180_[Bacillus_cereus]	CPTF_Cd	28959.46667	25908.88918	89.46604396
UIJ69099.1	hypothetical_protein_LW858_13180_[Bacillus_cereus]	CPTF_Co	23376.6	4266.314722	18.25036456
UIJ69099.1	hypothetical_protein_LW858_13180_[Bacillus_cereus]	CPTF_Cu	9096.633333	15755.83111	173.2050808
UIJ69099.1	hypothetical_protein_LW858_13180_[Bacillus_cereus]	CPTF_Fe	13533.53333	23440.76734	173.2050808
UIJ69099.1	hypothetical_protein_LW858_13180_[Bacillus_cereus]	CPTF_Mn	6173.8	10693.33528	173.2050808
UIJ69099.1	hypothetical_protein_LW858_13180_[Bacillus_cereus]	CPTF_Ni	0	0	0
UIJ69099.1	hypothetical_protein_LW858_13180_[Bacillus_cereus]	CPTF_U	0	0	0
UIJ69099.1	hypothetical_protein_LW858_13180_[Bacillus_cereus]	CPTF_metals_mix	67014.96667	24415.12981	36.43235389
UIJ69099.1	hypothetical_protein_LW858_13180_[Bacillus_cereus]	CPTF_zcontrol	0	0	0
UIJ69100.1	GNAT_family_N-acetyltransferase_[Bacillus_cereus]	CPTF_Al	455303.7	219395.6616	48.18666344
UIJ69100.1	GNAT_family_N-acetyltransferase_[Bacillus_cereus]	CPTF_Cd	494757	300590.6225	60.75520357
UIJ69100.1	GNAT_family_N-acetyltransferase_[Bacillus_cereus]	CPTF_Co	312878.6	277421.806	88.66755539
UIJ69100.1	GNAT_family_N-acetyltransferase_[Bacillus_cereus]	CPTF_Cu	112733.2667	155157.7945	137.6326608
UIJ69100.1	GNAT_family_N-acetyltransferase_[Bacillus_cereus]	CPTF_Fe	0	0	0
UIJ69100.1	GNAT_family_N-acetyltransferase_[Bacillus_cereus]	CPTF_Mn	464528.2667	196359.777	42.27079191
UIJ69100.1	GNAT_family_N-acetyltransferase_[Bacillus_cereus]	CPTF_Ni	9219.933333	15969.39298	173.2050808
UIJ69100.1	GNAT_family_N-acetyltransferase_[Bacillus_cereus]	CPTF_U	134566.3333	233075.7263	173.2050808
UIJ69100.1	GNAT_family_N-acetyltransferase_[Bacillus_cereus]	CPTF_metals_mix	0	0	0
UIJ69100.1	GNAT_family_N-acetyltransferase_[Bacillus_cereus]	CPTF_zcontrol	148736.3667	226255.978	152.1188013
UIJ69101.1	glyoxalase/bleomycin_resistance/dioxygenase_family_protein_[Bacillus_cereus]	CPTF_Al	493948	236310.2533	47.84111957
UIJ69101.1	glyoxalase/bleomycin_resistance/dioxygenase_family_protein_[Bacillus_cereus]	CPTF_Cd	403813	45004.25954	11.14482682
UIJ69101.1	glyoxalase/bleomycin_resistance/dioxygenase_family_protein_[Bacillus_cereus]	CPTF_Co	518884	100887.2357	19.44311941
UIJ69101.1	glyoxalase/bleomycin_resistance/dioxygenase_family_protein_[Bacillus_cereus]	CPTF_Cu	510017	79332.07552	15.55479043
UIJ69101.1	glyoxalase/bleomycin_resistance/dioxygenase_family_protein_[Bacillus_cereus]	CPTF_Fe	393069.6667	98491.68617	25.05705592
UIJ69101.1	glyoxalase/bleomycin_resistance/dioxygenase_family_protein_[Bacillus_cereus]	CPTF_Mn	482242	206702.0001	42.8627121
UIJ69101.1	glyoxalase/bleomycin_resistance/dioxygenase_family_protein_[Bacillus_cereus]	CPTF_Ni	575541.6667	28089.48033	4.880529414
UIJ69101.1	glyoxalase/bleomycin_resistance/dioxygenase_family_protein_[Bacillus_cereus]	CPTF_U	572526	349308.4031	61.01179739
UIJ69101.1	glyoxalase/bleomycin_resistance/dioxygenase_family_protein_[Bacillus_cereus]	CPTF_metals_mix	355135.3333	41223.85831	11.60792927
UIJ69101.1	glyoxalase/bleomycin_resistance/dioxygenase_family_protein_[Bacillus_cereus]	CPTF_zcontrol	642869	12591.68619	1.958670613
UIJ69138.1	hypothetical_protein_LW858_13400_[Bacillus_cereus]	CPTF_Al	2197304.2	872945.4493	39.72801987
UIJ69138.1	hypothetical_protein_LW858_13400_[Bacillus_cereus]	CPTF_Cd	1646518.933	418204.0381	25.3992851
UIJ69138.1	hypothetical_protein_LW858_13400_[Bacillus_cereus]	CPTF_Co	2203167.433	631764.3545	28.67527655
UIJ69138.1	hypothetical_protein_LW858_13400_[Bacillus_cereus]	CPTF_Cu	2539907.133	658154.5064	25.91254215
UIJ69138.1	hypothetical_protein_LW858_13400_[Bacillus_cereus]	CPTF_Fe	2259478.9	423189.3944	18.72951301
UIJ69138.1	hypothetical_protein_LW858_13400_[Bacillus_cereus]	CPTF_Mn	1831818.6	828671.6951	45.23765045
UIJ69138.1	hypothetical_protein_LW858_13400_[Bacillus_cereus]	CPTF_Ni	1776646.933	729164.0652	41.04158522
UIJ69138.1	hypothetical_protein_LW858_13400_[Bacillus_cereus]	CPTF_U	1423832.167	781714.4009	54.90214501
UIJ69138.1	hypothetical_protein_LW858_13400_[Bacillus_cereus]	CPTF_metals_mix	1874481.067	627462.7461	33.47394419
UIJ69138.1	hypothetical_protein_LW858_13400_[Bacillus_cereus]	CPTF_zcontrol	2578969.7	78068.93738	3.027136665
UIJ69139.1	cytoplasmic_protein_[Bacillus_cereus]	CPTF_Al	690727.3333	144641.9914	20.94053391
UIJ69139.1	cytoplasmic_protein_[Bacillus_cereus]	CPTF_Cd	739242.7333	98030.72428	13.26096556
UIJ69139.1	cytoplasmic_protein_[Bacillus_cereus]	CPTF_Co	702426.7667	121630.1101	17.31569978
UIJ69139.1	cytoplasmic_protein_[Bacillus_cereus]	CPTF_Cu	672710.8667	82371.65823	12.2447343
UIJ69139.1	cytoplasmic_protein_[Bacillus_cereus]	CPTF_Fe	667391.0667	185083.7136	27.732423
UIJ69139.1	cytoplasmic_protein_[Bacillus_cereus]	CPTF_Mn	569227.1667	61273.7734	10.76437967
UIJ69139.1	cytoplasmic_protein_[Bacillus_cereus]	CPTF_Ni	535876.3667	73774.60018	13.76709345
UIJ69139.1	cytoplasmic_protein_[Bacillus_cereus]	CPTF_U	595811	17814.32814	2.98992938
UIJ69139.1	cytoplasmic_protein_[Bacillus_cereus]	CPTF_metals_mix	548068.3667	129951.6161	23.71084047
UIJ69139.1	cytoplasmic_protein_[Bacillus_cereus]	CPTF_zcontrol	570823.5333	58319.71606	10.21676799
UIJ69140.1	DUF3958_family_protein_[Bacillus_cereus]	CPTF_Al	352119	41872.62446	11.89161177
UIJ69140.1	DUF3958_family_protein_[Bacillus_cereus]	CPTF_Cd	102541.3333	177606.7992	173.2050808
UIJ69140.1	DUF3958_family_protein_[Bacillus_cereus]	CPTF_Co	211030.6667	183461.0665	86.93573753
UIJ69140.1	DUF3958_family_protein_[Bacillus_cereus]	CPTF_Cu	276606.3333	12774.56447	4.618319586
UIJ69140.1	DUF3958_family_protein_[Bacillus_cereus]	CPTF_Fe	116628.6667	202006.7763	173.2050808
UIJ69140.1	DUF3958_family_protein_[Bacillus_cereus]	CPTF_Mn	221306.6667	193394.0843	87.38737393
UIJ69140.1	DUF3958_family_protein_[Bacillus_cereus]	CPTF_Ni	268102.6667	19122.85398	7.132660864
UIJ69140.1	DUF3958_family_protein_[Bacillus_cereus]	CPTF_U	82831	143467.5004	173.2050808
UIJ69140.1	DUF3958_family_protein_[Bacillus_cereus]	CPTF_metals_mix	0	0	0
UIJ69140.1	DUF3958_family_protein_[Bacillus_cereus]	CPTF_zcontrol	251878	218259.7949	86.65298078
UIJ69142.1	antibiotic_biosynthesis_monooxygenase_[Bacillus_cereus]	CPTF_Al	299299.3333	47635.08628	15.91553371

UIJ69142.1	antibiotic_biosynthesis_monooxygenase_[Bacillus_cereus]	CPTF_Cd	243246	42102.059	17.30842809
UIJ69142.1	antibiotic_biosynthesis_monooxygenase_[Bacillus_cereus]	CPTF_Co	296218.6667	22886.11003	7.72608637
UIJ69142.1	antibiotic_biosynthesis_monooxygenase_[Bacillus_cereus]	CPTF_Cu	274650.3333	5643.560608	2.054816588
UIJ69142.1	antibiotic_biosynthesis_monooxygenase_[Bacillus_cereus]	CPTF_Fe	271789.6667	14886.52187	5.47722143
UIJ69142.1	antibiotic_biosynthesis_monooxygenase_[Bacillus_cereus]	CPTF_Mn	274884	49816.6453	18.12278827
UIJ69142.1	antibiotic_biosynthesis_monooxygenase_[Bacillus_cereus]	CPTF_Ni	204486.3333	178384.8711	87.23559575
UIJ69142.1	antibiotic_biosynthesis_monooxygenase_[Bacillus_cereus]	CPTF_U	76477.66667	132463.2043	173.2050808
UIJ69142.1	antibiotic_biosynthesis_monooxygenase_[Bacillus_cereus]	CPTF_metals_mix	140871.3333	122747.2241	87.13428145
UIJ69142.1	antibiotic_biosynthesis_monooxygenase_[Bacillus_cereus]	CPTF_zcontrol	306212.6667	37077.00911	12.10825454
UIJ69148.1	DUF4240_domain-containing_protein_[Bacillus_cereus]	CPTF_Al	0	0	0
UIJ69148.1	DUF4240_domain-containing_protein_[Bacillus_cereus]	CPTF_Cd	0	0	0
UIJ69148.1	DUF4240_domain-containing_protein_[Bacillus_cereus]	CPTF_Co	29851.4	51704.14148	173.2050808
UIJ69148.1	DUF4240_domain-containing_protein_[Bacillus_cereus]	CPTF_Cu	0	0	0
UIJ69148.1	DUF4240_domain-containing_protein_[Bacillus_cereus]	CPTF_Fe	0	0	0
UIJ69148.1	DUF4240_domain-containing_protein_[Bacillus_cereus]	CPTF_Mn	0	0	0
UIJ69148.1	DUF4240_domain-containing_protein_[Bacillus_cereus]	CPTF_Ni	0	0	0
UIJ69148.1	DUF4240_domain-containing_protein_[Bacillus_cereus]	CPTF_U	0	0	0
UIJ69148.1	DUF4240_domain-containing_protein_[Bacillus_cereus]	CPTF_metals_mix	0	0	0
UIJ69148.1	DUF4240_domain-containing_protein_[Bacillus_cereus]	CPTF_zcontrol	0	0	0
UIJ69162.1	DUF2785_domain-containing_protein_[Bacillus_cereus]	CPTF_Al	0	0	0
UIJ69162.1	DUF2785_domain-containing_protein_[Bacillus_cereus]	CPTF_Cd	0	0	0
UIJ69162.1	DUF2785_domain-containing_protein_[Bacillus_cereus]	CPTF_Co	7992.466667	13843.35834	173.2050808
UIJ69162.1	DUF2785_domain-containing_protein_[Bacillus_cereus]	CPTF_Cu	16634.96667	28812.60745	173.2050808
UIJ69162.1	DUF2785_domain-containing_protein_[Bacillus_cereus]	CPTF_Fe	22295.1	38616.24596	173.2050808
UIJ69162.1	DUF2785_domain-containing_protein_[Bacillus_cereus]	CPTF_Mn	0	0	0
UIJ69162.1	DUF2785_domain-containing_protein_[Bacillus_cereus]	CPTF_Ni	26153.63333	27619.79841	105.6059709
UIJ69162.1	DUF2785_domain-containing_protein_[Bacillus_cereus]	CPTF_U	0	0	0
UIJ69162.1	DUF2785_domain-containing_protein_[Bacillus_cereus]	CPTF_metals_mix	12217.6	21161.50395	173.2050808
UIJ69162.1	DUF2785_domain-containing_protein_[Bacillus_cereus]	CPTF_zcontrol	0	0	0
UIJ69164.1	metallophosphatase_family_protein_[Bacillus_cereus]	CPTF_Al	49010.56667	45980.80767	93.81815147
UIJ69164.1	metallophosphatase_family_protein_[Bacillus_cereus]	CPTF_Cd	75285	65897.40427	87.53058945
UIJ69164.1	metallophosphatase_family_protein_[Bacillus_cereus]	CPTF_Co	35038.3	32785.63404	93.57084689
UIJ69164.1	metallophosphatase_family_protein_[Bacillus_cereus]	CPTF_Cu	0	0	0
UIJ69164.1	metallophosphatase_family_protein_[Bacillus_cereus]	CPTF_Fe	44274.33333	76685.3948	173.2050808
UIJ69164.1	metallophosphatase_family_protein_[Bacillus_cereus]	CPTF_Mn	55334.33333	54046.19696	97.67208478
UIJ69164.1	metallophosphatase_family_protein_[Bacillus_cereus]	CPTF_Ni	0	0	0
UIJ69164.1	metallophosphatase_family_protein_[Bacillus_cereus]	CPTF_U	23321.66667	40394.31158	173.2050808
UIJ69164.1	metallophosphatase_family_protein_[Bacillus_cereus]	CPTF_metals_mix	54077.5	47113.31124	87.1218367
UIJ69164.1	metallophosphatase_family_protein_[Bacillus_cereus]	CPTF_zcontrol	62706.33333	57675.06593	91.97646054
UIJ69182.1	DUF979_domain-containing_protein_[Bacillus_cereus]	CPTF_Al	0	0	0
UIJ69182.1	DUF979_domain-containing_protein_[Bacillus_cereus]	CPTF_Cd	0	0	0
UIJ69182.1	DUF979_domain-containing_protein_[Bacillus_cereus]	CPTF_Co	0	0	0
UIJ69182.1	DUF979_domain-containing_protein_[Bacillus_cereus]	CPTF_Cu	0	0	0
UIJ69182.1	DUF979_domain-containing_protein_[Bacillus_cereus]	CPTF_Fe	0	0	0
UIJ69182.1	DUF979_domain-containing_protein_[Bacillus_cereus]	CPTF_Mn	0	0	0
UIJ69182.1	DUF979_domain-containing_protein_[Bacillus_cereus]	CPTF_Ni	220343.3333	381645.8484	173.2050808
UIJ69182.1	DUF979_domain-containing_protein_[Bacillus_cereus]	CPTF_U	0	0	0
UIJ69182.1	DUF979_domain-containing_protein_[Bacillus_cereus]	CPTF_metals_mix	0	0	0
UIJ69182.1	DUF979_domain-containing_protein_[Bacillus_cereus]	CPTF_zcontrol	232236.6667	402245.706	173.2050808
UIJ69188.1	signal_peptidase_I_[Bacillus_cereus]	CPTF_Al	0	0	0
UIJ69188.1	signal_peptidase_I_[Bacillus_cereus]	CPTF_Cd	0	0	0
UIJ69188.1	signal_peptidase_I_[Bacillus_cereus]	CPTF_Co	0	0	0
UIJ69188.1	signal_peptidase_I_[Bacillus_cereus]	CPTF_Cu	84188.6	8169.905301	9.704289299
UIJ69188.1	signal_peptidase_I_[Bacillus_cereus]	CPTF_Fe	12563.8	21761.13994	173.2050808
UIJ69188.1	signal_peptidase_I_[Bacillus_cereus]	CPTF_Mn	0	0	0
UIJ69188.1	signal_peptidase_I_[Bacillus_cereus]	CPTF_Ni	33960.93333	31136.63198	91.6836757
UIJ69188.1	signal_peptidase_I_[Bacillus_cereus]	CPTF_U	0	0	0
UIJ69188.1	signal_peptidase_I_[Bacillus_cereus]	CPTF_metals_mix	164071.9333	20803.81679	12.6796926
UIJ69188.1	signal_peptidase_I_[Bacillus_cereus]	CPTF_zcontrol	0	0	0
UIJ69189.1	copper_homeostasis_protein_CutC_[Bacillus_cereus]	CPTF_Al	0	0	0
UIJ69189.1	copper_homeostasis_protein_CutC_[Bacillus_cereus]	CPTF_Cd	0	0	0

UIJ69189.1	copper_homeostasis_protein_CutC_[Bacillus_cereus]	CPTF_Co	0	0	0
UIJ69189.1	copper_homeostasis_protein_CutC_[Bacillus_cereus]	CPTF_Cu	0	0	0
UIJ69189.1	copper_homeostasis_protein_CutC_[Bacillus_cereus]	CPTF_Fe	0	0	0
UIJ69189.1	copper_homeostasis_protein_CutC_[Bacillus_cereus]	CPTF_Mn	0	0	0
UIJ69189.1	copper_homeostasis_protein_CutC_[Bacillus_cereus]	CPTF_Ni	0	0	0
UIJ69189.1	copper_homeostasis_protein_CutC_[Bacillus_cereus]	CPTF_U	0	0	0
UIJ69189.1	copper_homeostasis_protein_CutC_[Bacillus_cereus]	CPTF_metals_mix	559440.2667	213202.6028	38.1099852
UIJ69189.1	copper_homeostasis_protein_CutC_[Bacillus_cereus]	CPTF_zcontrol	0	0	0
UIJ69192.1	acyl-CoA/acyl-ACP_dehydrogenase_[Bacillus_cereus]	CPTF_Al	229074.3333	198923.1685	86.83782491
UIJ69192.1	acyl-CoA/acyl-ACP_dehydrogenase_[Bacillus_cereus]	CPTF_Cd	280475.6667	71466.10633	25.48032319
UIJ69192.1	acyl-CoA/acyl-ACP_dehydrogenase_[Bacillus_cereus]	CPTF_Co	276245.5333	103448.5046	37.44802797
UIJ69192.1	acyl-CoA/acyl-ACP_dehydrogenase_[Bacillus_cereus]	CPTF_Cu	340703.5667	15262.48233	4.479695495
UIJ69192.1	acyl-CoA/acyl-ACP_dehydrogenase_[Bacillus_cereus]	CPTF_Fe	185564	165515.5626	89.19594457
UIJ69192.1	acyl-CoA/acyl-ACP_dehydrogenase_[Bacillus_cereus]	CPTF_Mn	156416	156485.0456	100.0441423
UIJ69192.1	acyl-CoA/acyl-ACP_dehydrogenase_[Bacillus_cereus]	CPTF_Ni	230620.0333	47749.88834	20.70500453
UIJ69192.1	acyl-CoA/acyl-ACP_dehydrogenase_[Bacillus_cereus]	CPTF_U	206707.8333	181522.5509	87.816
UIJ69192.1	acyl-CoA/acyl-ACP_dehydrogenase_[Bacillus_cereus]	CPTF_metals_mix	403787.0667	43568.4277	10.78995126
UIJ69192.1	acyl-CoA/acyl-ACP_dehydrogenase_[Bacillus_cereus]	CPTF_zcontrol	248941.3333	41648.09722	16.73008522
UIJ69213.1	LacI_family_transcriptional_regulator_[Bacillus_cereus]	CPTF_Al	22199.1	38449.96908	173.2050808
UIJ69213.1	LacI_family_transcriptional_regulator_[Bacillus_cereus]	CPTF_Cd	0	0	0
UIJ69213.1	LacI_family_transcriptional_regulator_[Bacillus_cereus]	CPTF_Co	22611.9	39164.95966	173.2050808
UIJ69213.1	LacI_family_transcriptional_regulator_[Bacillus_cereus]	CPTF_Cu	25620.23333	44375.54584	173.2050808
UIJ69213.1	LacI_family_transcriptional_regulator_[Bacillus_cereus]	CPTF_Fe	40780.3	36901.61709	90.48883184
UIJ69213.1	LacI_family_transcriptional_regulator_[Bacillus_cereus]	CPTF_Mn	0	0	0
UIJ69213.1	LacI_family_transcriptional_regulator_[Bacillus_cereus]	CPTF_Ni	17381.73333	30106.04526	173.2050808
UIJ69213.1	LacI_family_transcriptional_regulator_[Bacillus_cereus]	CPTF_U	0	0	0
UIJ69213.1	LacI_family_transcriptional_regulator_[Bacillus_cereus]	CPTF_metals_mix	0	0	0
UIJ69213.1	LacI_family_transcriptional_regulator_[Bacillus_cereus]	CPTF_zcontrol	0	0	0
UIJ69218.1	DinB_family_protein_[Bacillus_cereus]	CPTF_Al	28885.26667	25019.87646	86.61812524
UIJ69218.1	DinB_family_protein_[Bacillus_cereus]	CPTF_Cd	48355.56667	12492.75359	25.8351922
UIJ69218.1	DinB_family_protein_[Bacillus_cereus]	CPTF_Co	47332.9	12298.38116	25.98273328
UIJ69218.1	DinB_family_protein_[Bacillus_cereus]	CPTF_Cu	60732.43333	20174.9669	33.2194279
UIJ69218.1	DinB_family_protein_[Bacillus_cereus]	CPTF_Fe	52068.03333	7095.353098	13.62708104
UIJ69218.1	DinB_family_protein_[Bacillus_cereus]	CPTF_Mn	56763.06667	49972.38609	88.03679757
UIJ69218.1	DinB_family_protein_[Bacillus_cereus]	CPTF_Ni	13341.73333	23108.55999	173.2050808
UIJ69218.1	DinB_family_protein_[Bacillus_cereus]	CPTF_U	12109.23333	20973.80737	173.2050808
UIJ69218.1	DinB_family_protein_[Bacillus_cereus]	CPTF_metals_mix	7615.133333	13189.79784	173.2050808
UIJ69218.1	DinB_family_protein_[Bacillus_cereus]	CPTF_zcontrol	33258.36667	29057.06343	87.3676802
UIJ69225.1	VOC_family_protein_[Bacillus_cereus]	CPTF_Al	59282.66667	11507.26402	19.41084075
UIJ69225.1	VOC_family_protein_[Bacillus_cereus]	CPTF_Cd	101471.4	32041.60729	31.57698355
UIJ69225.1	VOC_family_protein_[Bacillus_cereus]	CPTF_Co	72969.53333	32417.40652	44.42594743
UIJ69225.1	VOC_family_protein_[Bacillus_cereus]	CPTF_Cu	98035.33333	36706.23016	37.44183746
UIJ69225.1	VOC_family_protein_[Bacillus_cereus]	CPTF_Fe	75612.7	27094.86323	35.83374648
UIJ69225.1	VOC_family_protein_[Bacillus_cereus]	CPTF_Mn	57345.46667	32876.44293	57.33050029
UIJ69225.1	VOC_family_protein_[Bacillus_cereus]	CPTF_Ni	101665.3667	18725.91109	18.41916446
UIJ69225.1	VOC_family_protein_[Bacillus_cereus]	CPTF_U	46207.1	18078.79149	39.1255705
UIJ69225.1	VOC_family_protein_[Bacillus_cereus]	CPTF_metals_mix	863227.2333	149655.2344	17.33671375
UIJ69225.1	VOC_family_protein_[Bacillus_cereus]	CPTF_zcontrol	35082	3211.214649	9.153453762
UIJ69245.1	TerD_family_protein_[Bacillus_cereus]	CPTF_Al	175987.9	69501.50705	39.49220773
UIJ69245.1	TerD_family_protein_[Bacillus_cereus]	CPTF_Cd	159160.2333	85813.06065	53.91614404
UIJ69245.1	TerD_family_protein_[Bacillus_cereus]	CPTF_Co	172104.8	53722.64791	31.2150782
UIJ69245.1	TerD_family_protein_[Bacillus_cereus]	CPTF_Cu	115672.0333	61685.14168	53.32761939
UIJ69245.1	TerD_family_protein_[Bacillus_cereus]	CPTF_Fe	196381.0333	82610.5878	42.06647984
UIJ69245.1	TerD_family_protein_[Bacillus_cereus]	CPTF_Mn	118008.0667	46501.19465	39.40509828
UIJ69245.1	TerD_family_protein_[Bacillus_cereus]	CPTF_Ni	184783.0333	102463.516	55.45071652
UIJ69245.1	TerD_family_protein_[Bacillus_cereus]	CPTF_U	224759.5	84618.28989	37.64837077
UIJ69245.1	TerD_family_protein_[Bacillus_cereus]	CPTF_metals_mix	34333.83333	34287.34493	99.86459887
UIJ69245.1	TerD_family_protein_[Bacillus_cereus]	CPTF_zcontrol	188176.9	164770.0809	87.56126862
UIJ69251.1	D-cysteine_desulfhydrase_[Bacillus_cereus]	CPTF_Al	43965.8	39939.64468	90.84252916
UIJ69251.1	D-cysteine_desulfhydrase_[Bacillus_cereus]	CPTF_Cd	128222	30648.1396	23.90240333
UIJ69251.1	D-cysteine_desulfhydrase_[Bacillus_cereus]	CPTF_Co	38238.8	33853.13137	88.53084137

UIJ69251.1	D-cysteine_desulfhydrase_[Bacillus_cereus]	CPTF_Cu	39775.33333	34450.98294	86.61393897
UIJ69251.1	D-cysteine_desulfhydrase_[Bacillus_cereus]	CPTF_Fe	62839.8	23795.61494	37.86710801
UIJ69251.1	D-cysteine_desulfhydrase_[Bacillus_cereus]	CPTF_Mn	54995.3	33183.17366	60.33819919
UIJ69251.1	D-cysteine_desulfhydrase_[Bacillus_cereus]	CPTF_Ni	0	0	0
UIJ69251.1	D-cysteine_desulfhydrase_[Bacillus_cereus]	CPTF_U	9777.766667	16935.58865	173.2050808
UIJ69251.1	D-cysteine_desulfhydrase_[Bacillus_cereus]	CPTF_metals_mix	152384	25115.20498	16.48152364
UIJ69251.1	D-cysteine_desulfhydrase_[Bacillus_cereus]	CPTF_zcontrol	30033.43333	26544.18483	88.38211914
UIJ69256.1	ABC_transporter_ATP-binding_protein_[Bacillus_cereus]	CPTF_Al	395442.2	343042.5698	86.74910513
UIJ69256.1	ABC_transporter_ATP-binding_protein_[Bacillus_cereus]	CPTF_Cd	804208.0333	88546.08479	11.01034572
UIJ69256.1	ABC_transporter_ATP-binding_protein_[Bacillus_cereus]	CPTF_Co	404064.9	364322.9009	90.16445152
UIJ69256.1	ABC_transporter_ATP-binding_protein_[Bacillus_cereus]	CPTF_Cu	209209.7667	83265.46842	39.79999105
UIJ69256.1	ABC_transporter_ATP-binding_protein_[Bacillus_cereus]	CPTF_Fe	566070.6667	211347.2258	37.33583778
UIJ69256.1	ABC_transporter_ATP-binding_protein_[Bacillus_cereus]	CPTF_Mn	388209.2333	336431.0152	86.66229092
UIJ69256.1	ABC_transporter_ATP-binding_protein_[Bacillus_cereus]	CPTF_Ni	46269.13333	40074.69491	86.6121581
UIJ69256.1	ABC_transporter_ATP-binding_protein_[Bacillus_cereus]	CPTF_U	0	0	0
UIJ69256.1	ABC_transporter_ATP-binding_protein_[Bacillus_cereus]	CPTF_metals_mix	1186394.667	169918.3537	14.32224524
UIJ69256.1	ABC_transporter_ATP-binding_protein_[Bacillus_cereus]	CPTF_zcontrol	374138.8667	292586.9735	78.20277431
UIJ69257.1	ABC_transporter_permease_[Bacillus_cereus]	CPTF_Al	930716.3667	72952.69254	7.838337774
UIJ69257.1	ABC_transporter_permease_[Bacillus_cereus]	CPTF_Cd	1117014.133	160593.5522	14.37703852
UIJ69257.1	ABC_transporter_permease_[Bacillus_cereus]	CPTF_Co	969405.9	224045.3081	23.1116097
UIJ69257.1	ABC_transporter_permease_[Bacillus_cereus]	CPTF_Cu	976782.7667	99813.05314	10.21855182
UIJ69257.1	ABC_transporter_permease_[Bacillus_cereus]	CPTF_Fe	995604.4667	150052.0155	15.07144861
UIJ69257.1	ABC_transporter_permease_[Bacillus_cereus]	CPTF_Mn	869461	131400.3835	15.11285537
UIJ69257.1	ABC_transporter_permease_[Bacillus_cereus]	CPTF_Ni	1029530.5	19431.85013	1.887447738
UIJ69257.1	ABC_transporter_permease_[Bacillus_cereus]	CPTF_U	1223884.033	75733.19125	6.187938496
UIJ69257.1	ABC_transporter_permease_[Bacillus_cereus]	CPTF_metals_mix	1429570.2	47886.27405	3.349697276
UIJ69257.1	ABC_transporter_permease_[Bacillus_cereus]	CPTF_zcontrol	937974.3333	125978.0054	13.4308585
UIJ69259.1	DinB_family_protein_[Bacillus_cereus]	CPTF_Al	129224	114970.8487	88.970198
UIJ69259.1	DinB_family_protein_[Bacillus_cereus]	CPTF_Cd	222522.6667	20852.34745	9.370886915
UIJ69259.1	DinB_family_protein_[Bacillus_cereus]	CPTF_Co	152193	139290.523	91.52229275
UIJ69259.1	DinB_family_protein_[Bacillus_cereus]	CPTF_Cu	149560.3333	25240.53506	16.87649024
UIJ69259.1	DinB_family_protein_[Bacillus_cereus]	CPTF_Fe	126494.3333	110060.2501	87.00804789
UIJ69259.1	DinB_family_protein_[Bacillus_cereus]	CPTF_Mn	257431.3333	185956.0326	72.2351977
UIJ69259.1	DinB_family_protein_[Bacillus_cereus]	CPTF_Ni	44936	77831.43509	173.2050808
UIJ69259.1	DinB_family_protein_[Bacillus_cereus]	CPTF_U	106234.3333	101907.5559	95.9271383
UIJ69259.1	DinB_family_protein_[Bacillus_cereus]	CPTF_metals_mix	83118.66667	72207.49298	86.87277585
UIJ69259.1	DinB_family_protein_[Bacillus_cereus]	CPTF_zcontrol	347879.6667	148374.6058	42.6511291
UIJ69267.1	LD-carboxypeptidase_[Bacillus_cereus]	CPTF_Al	0	0	0
UIJ69267.1	LD-carboxypeptidase_[Bacillus_cereus]	CPTF_Cd	0	0	0
UIJ69267.1	LD-carboxypeptidase_[Bacillus_cereus]	CPTF_Co	5789.433333	10027.59268	173.2050808
UIJ69267.1	LD-carboxypeptidase_[Bacillus_cereus]	CPTF_Cu	0	0	0
UIJ69267.1	LD-carboxypeptidase_[Bacillus_cereus]	CPTF_Fe	0	0	0
UIJ69267.1	LD-carboxypeptidase_[Bacillus_cereus]	CPTF_Mn	0	0	0
UIJ69267.1	LD-carboxypeptidase_[Bacillus_cereus]	CPTF_Ni	0	0	0
UIJ69267.1	LD-carboxypeptidase_[Bacillus_cereus]	CPTF_U	0	0	0
UIJ69267.1	LD-carboxypeptidase_[Bacillus_cereus]	CPTF_metals_mix	238821.5	51117.13969	21.40391032
UIJ69267.1	LD-carboxypeptidase_[Bacillus_cereus]	CPTF_zcontrol	0	0	0
UIJ69268.1	DUF3224_domain-containing_protein_[Bacillus_cereus]	CPTF_Al	153726.6667	166072.9836	108.0313437
UIJ69268.1	DUF3224_domain-containing_protein_[Bacillus_cereus]	CPTF_Cd	180601.3333	18389.74204	10.18250624
UIJ69268.1	DUF3224_domain-containing_protein_[Bacillus_cereus]	CPTF_Co	0	0	0
UIJ69268.1	DUF3224_domain-containing_protein_[Bacillus_cereus]	CPTF_Cu	51247	88762.40774	173.2050808
UIJ69268.1	DUF3224_domain-containing_protein_[Bacillus_cereus]	CPTF_Fe	116021.3333	100804.0277	86.88404522
UIJ69268.1	DUF3224_domain-containing_protein_[Bacillus_cereus]	CPTF_Mn	82825.33333	74292.99685	89.69839766
UIJ69268.1	DUF3224_domain-containing_protein_[Bacillus_cereus]	CPTF_Ni	0	0	0
UIJ69268.1	DUF3224_domain-containing_protein_[Bacillus_cereus]	CPTF_U	0	0	0
UIJ69268.1	DUF3224_domain-containing_protein_[Bacillus_cereus]	CPTF_metals_mix	157451.5333	153153.6089	97.27031912
UIJ69268.1	DUF3224_domain-containing_protein_[Bacillus_cereus]	CPTF_zcontrol	179193.6667	15704.96091	8.76423883
UIJ69269.1	DUF1015_family_protein_[Bacillus_cereus]	CPTF_Al	1050440.1	166434.8306	15.84429523
UIJ69269.1	DUF1015_family_protein_[Bacillus_cereus]	CPTF_Cd	1264126.333	241618.9634	19.11351398
UIJ69269.1	DUF1015_family_protein_[Bacillus_cereus]	CPTF_Co	1015598.067	128515.0836	12.65412842
UIJ69269.1	DUF1015_family_protein_[Bacillus_cereus]	CPTF_Cu	1071690.767	167102.955	15.59246008

UIJ69269.1	DUF1015_family_protein_[Bacillus_cereus]	CPTF_Fe	982390.3333	343047.1307	34.91963622
UIJ69269.1	DUF1015_family_protein_[Bacillus_cereus]	CPTF_Mn	1232798	114504.9345	9.28821547
UIJ69269.1	DUF1015_family_protein_[Bacillus_cereus]	CPTF_Ni	603852.3333	225311.2035	37.31230155
UIJ69269.1	DUF1015_family_protein_[Bacillus_cereus]	CPTF_U	648674.4	536930.5611	82.77350874
UIJ69269.1	DUF1015_family_protein_[Bacillus_cereus]	CPTF_metals_mix	1565959.633	131192.3574	8.377761123
UIJ69269.1	DUF1015_family_protein_[Bacillus_cereus]	CPTF_zcontrol	1037749.533	454940.5974	43.8391522
UIJ69270.1	3-phosphoglycerate_dehydrogenase_family_protein_[Bacillus_cereus]	CPTF_Al	2667652.333	175982.7756	6.596915698
UIJ69270.1	3-phosphoglycerate_dehydrogenase_family_protein_[Bacillus_cereus]	CPTF_Cd	2695360.5	344302.3908	12.7738902
UIJ69270.1	3-phosphoglycerate_dehydrogenase_family_protein_[Bacillus_cereus]	CPTF_Co	2712273.667	298615.46	11.00978355
UIJ69270.1	3-phosphoglycerate_dehydrogenase_family_protein_[Bacillus_cereus]	CPTF_Cu	2864194.5	277412.6141	9.685536861
UIJ69270.1	3-phosphoglycerate_dehydrogenase_family_protein_[Bacillus_cereus]	CPTF_Fe	2515670	264973.1098	10.53290415
UIJ69270.1	3-phosphoglycerate_dehydrogenase_family_protein_[Bacillus_cereus]	CPTF_Mn	2433380.333	385580.5647	15.84547057
UIJ69270.1	3-phosphoglycerate_dehydrogenase_family_protein_[Bacillus_cereus]	CPTF_Ni	2572666.633	59696.29719	2.320405466
UIJ69270.1	3-phosphoglycerate_dehydrogenase_family_protein_[Bacillus_cereus]	CPTF_U	2611942.667	338776.458	12.97028692
UIJ69270.1	3-phosphoglycerate_dehydrogenase_family_protein_[Bacillus_cereus]	CPTF_metals_mix	2545050.467	485883.0229	19.09129227
UIJ69270.1	3-phosphoglycerate_dehydrogenase_family_protein_[Bacillus_cereus]	CPTF_zcontrol	2503799.333	155667.4971	6.217251318
UIJ69271.1	3-phosphoserine/phosphohydroxythreonine_transaminase_[Bacillus_cereus]	CPTF_Al	2356241.667	165824.8145	7.0376828
UIJ69271.1	3-phosphoserine/phosphohydroxythreonine_transaminase_[Bacillus_cereus]	CPTF_Cd	2246336.667	411615.3112	18.32384777
UIJ69271.1	3-phosphoserine/phosphohydroxythreonine_transaminase_[Bacillus_cereus]	CPTF_Co	1512136.667	448033.993	29.62919972
UIJ69271.1	3-phosphoserine/phosphohydroxythreonine_transaminase_[Bacillus_cereus]	CPTF_Cu	1906559	698991.4275	36.66245983
UIJ69271.1	3-phosphoserine/phosphohydroxythreonine_transaminase_[Bacillus_cereus]	CPTF_Fe	2613385	610256.6822	23.3511971
UIJ69271.1	3-phosphoserine/phosphohydroxythreonine_transaminase_[Bacillus_cereus]	CPTF_Mn	1498893.333	624367.1257	41.65520734
UIJ69271.1	3-phosphoserine/phosphohydroxythreonine_transaminase_[Bacillus_cereus]	CPTF_Ni	2088305.333	608354.4237	29.13148829
UIJ69271.1	3-phosphoserine/phosphohydroxythreonine_transaminase_[Bacillus_cereus]	CPTF_U	1638395.667	342519.195	20.90576788
UIJ69271.1	3-phosphoserine/phosphohydroxythreonine_transaminase_[Bacillus_cereus]	CPTF_metals_mix	1612664.567	246031.7713	15.25622727
UIJ69271.1	3-phosphoserine/phosphohydroxythreonine_transaminase_[Bacillus_cereus]	CPTF_zcontrol	1581890.667	571342.7832	36.1177163
UIJ69321.1	peroxide-responsive_transcriptional_repressor_PerR_[Bacillus_cereus]	CPTF_Al	318257.4333	59654.51548	18.74410752
UIJ69321.1	peroxide-responsive_transcriptional_repressor_PerR_[Bacillus_cereus]	CPTF_Cd	279010.6333	166906.6608	59.82089598
UIJ69321.1	peroxide-responsive_transcriptional_repressor_PerR_[Bacillus_cereus]	CPTF_Co	365026.6333	87135.66536	23.87104321
UIJ69321.1	peroxide-responsive_transcriptional_repressor_PerR_[Bacillus_cereus]	CPTF_Cu	413008.8	56124.04856	13.58906846
UIJ69321.1	peroxide-responsive_transcriptional_repressor_PerR_[Bacillus_cereus]	CPTF_Fe	204212.6	149348.6601	73.13391049
UIJ69321.1	peroxide-responsive_transcriptional_repressor_PerR_[Bacillus_cereus]	CPTF_Mn	170170.9333	127215.4228	74.75743377
UIJ69321.1	peroxide-responsive_transcriptional_repressor_PerR_[Bacillus_cereus]	CPTF_Ni	327613.0667	78351.35826	23.91582212
UIJ69321.1	peroxide-responsive_transcriptional_repressor_PerR_[Bacillus_cereus]	CPTF_U	263476.6667	24229.91872	9.196229416
UIJ69321.1	peroxide-responsive_transcriptional_repressor_PerR_[Bacillus_cereus]	CPTF_metals_mix	272164.8333	89971.13536	33.05759023
UIJ69321.1	peroxide-responsive_transcriptional_repressor_PerR_[Bacillus_cereus]	CPTF_zcontrol	261843.6667	53052.65136	20.26119327
UIJ69323.1	hypothetical_protein_LW858_01035_[Bacillus_cereus]	CPTF_Al	136035.3333	118265.661	86.93745818
UIJ69323.1	hypothetical_protein_LW858_01035_[Bacillus_cereus]	CPTF_Cd	67427.33333	116787.5672	173.2050808
UIJ69323.1	hypothetical_protein_LW858_01035_[Bacillus_cereus]	CPTF_Co	0	0	0
UIJ69323.1	hypothetical_protein_LW858_01035_[Bacillus_cereus]	CPTF_Cu	0	0	0
UIJ69323.1	hypothetical_protein_LW858_01035_[Bacillus_cereus]	CPTF_Fe	53364.33333	92429.73665	173.2050808
UIJ69323.1	hypothetical_protein_LW858_01035_[Bacillus_cereus]	CPTF_Mn	0	0	0
UIJ69323.1	hypothetical_protein_LW858_01035_[Bacillus_cereus]	CPTF_Ni	0	0	0
UIJ69323.1	hypothetical_protein_LW858_01035_[Bacillus_cereus]	CPTF_U	0	0	0
UIJ69323.1	hypothetical_protein_LW858_01035_[Bacillus_cereus]	CPTF_metals_mix	14775.16667	25591.33936	173.2050808
UIJ69323.1	hypothetical_protein_LW858_01035_[Bacillus_cereus]	CPTF_zcontrol	42843	74206.25275	173.2050808
UIJ69328.1	potassium-transporting_ATPase_subunit_KdpB_[Bacillus_cereus]	CPTF_Al	1452409.333	226375.8037	15.58622618
UIJ69328.1	potassium-transporting_ATPase_subunit_KdpB_[Bacillus_cereus]	CPTF_Cd	1333117.667	421840.0848	31.64312464
UIJ69328.1	potassium-transporting_ATPase_subunit_KdpB_[Bacillus_cereus]	CPTF_Co	2499341.767	750310.2987	30.0203161
UIJ69328.1	potassium-transporting_ATPase_subunit_KdpB_[Bacillus_cereus]	CPTF_Cu	1465988.433	53712.22658	3.663891567
UIJ69328.1	potassium-transporting_ATPase_subunit_KdpB_[Bacillus_cereus]	CPTF_Fe	1333691.333	152228.3906	11.41406462
UIJ69328.1	potassium-transporting_ATPase_subunit_KdpB_[Bacillus_cereus]	CPTF_Mn	1200997.533	67590.41504	5.627856275
UIJ69328.1	potassium-transporting_ATPase_subunit_KdpB_[Bacillus_cereus]	CPTF_Ni	1439449.7	159062.1055	11.05020242
UIJ69328.1	potassium-transporting_ATPase_subunit_KdpB_[Bacillus_cereus]	CPTF_U	1521818.3	278675.4922	18.31200822
UIJ69328.1	potassium-transporting_ATPase_subunit_KdpB_[Bacillus_cereus]	CPTF_metals_mix	1952701.733	124257.8457	6.363380722
UIJ69328.1	potassium-transporting_ATPase_subunit_KdpB_[Bacillus_cereus]	CPTF_zcontrol	1179485.2	102500.0355	8.690234988
UIJ69348.1	nitronate_monooxygenase_[Bacillus_cereus]	CPTF_Al	197017.4667	15913.58391	8.07724522
UIJ69348.1	nitronate_monooxygenase_[Bacillus_cereus]	CPTF_Cd	199867.3667	100357.778	50.21218803
UIJ69348.1	nitronate_monooxygenase_[Bacillus_cereus]	CPTF_Co	176684.8333	28648.56908	16.21450384
UIJ69348.1	nitronate_monooxygenase_[Bacillus_cereus]	CPTF_Cu	124813.5333	53062.58401	42.51348599
UIJ69348.1	nitronate_monooxygenase_[Bacillus_cereus]	CPTF_Fe	130248.7667	108408.2009	83.2316525

UIJ69348.1	nitronate_monooxygenase_[Bacillus_cereus]	CPTF_Mn	86304.7	26040.09089	30.17227439
UIJ69348.1	nitronate_monooxygenase_[Bacillus_cereus]	CPTF_Ni	119698.5667	37339.32787	31.19446532
UIJ69348.1	nitronate_monooxygenase_[Bacillus_cereus]	CPTF_U	12930.36667	22396.05203	173.2050808
UIJ69348.1	nitronate_monooxygenase_[Bacillus_cereus]	CPTF_metals_mix	380195.9333	142231.9749	37.41017786
UIJ69348.1	nitronate_monooxygenase_[Bacillus_cereus]	CPTF_zcontrol	18904.1	32742.86167	173.2050808
UIJ69349.1	3-isopropylmalate_dehydratase_small_subunit_[Bacillus_cereus]	CPTF_Al	27838.6	48217.86961	173.2050808
UIJ69349.1	3-isopropylmalate_dehydratase_small_subunit_[Bacillus_cereus]	CPTF_Cd	60852.8	12320.44096	20.24630084
UIJ69349.1	3-isopropylmalate_dehydratase_small_subunit_[Bacillus_cereus]	CPTF_Co	85863	78531.72524	91.46166013
UIJ69349.1	3-isopropylmalate_dehydratase_small_subunit_[Bacillus_cereus]	CPTF_Cu	61006.03333	55420.15969	90.84373571
UIJ69349.1	3-isopropylmalate_dehydratase_small_subunit_[Bacillus_cereus]	CPTF_Fe	18030.1	31229.04927	173.2050808
UIJ69349.1	3-isopropylmalate_dehydratase_small_subunit_[Bacillus_cereus]	CPTF_Mn	94527.6	117713.0321	124.5276852
UIJ69349.1	3-isopropylmalate_dehydratase_small_subunit_[Bacillus_cereus]	CPTF_Ni	29040.86667	50300.25656	173.2050808
UIJ69349.1	3-isopropylmalate_dehydratase_small_subunit_[Bacillus_cereus]	CPTF_U	0	0	0
UIJ69349.1	3-isopropylmalate_dehydratase_small_subunit_[Bacillus_cereus]	CPTF_metals_mix	193628.5	30761.32914	15.88677759
UIJ69349.1	3-isopropylmalate_dehydratase_small_subunit_[Bacillus_cereus]	CPTF_zcontrol	8797.566667	15237.83245	173.2050808
UIJ69357.1	aspartate_aminotransferase_family_protein_[Bacillus_cereus]	CPTF_Al	4640209.967	166125.9861	3.580139419
UIJ69357.1	aspartate_aminotransferase_family_protein_[Bacillus_cereus]	CPTF_Cd	5098643.467	165476.0798	3.245492275
UIJ69357.1	aspartate_aminotransferase_family_protein_[Bacillus_cereus]	CPTF_Co	3597879.467	651572.2746	18.10989725
UIJ69357.1	aspartate_aminotransferase_family_protein_[Bacillus_cereus]	CPTF_Cu	3282375.1	256486.1422	7.814041185
UIJ69357.1	aspartate_aminotransferase_family_protein_[Bacillus_cereus]	CPTF_Fe	4572271.633	552293.3352	12.07918907
UIJ69357.1	aspartate_aminotransferase_family_protein_[Bacillus_cereus]	CPTF_Mn	3817305.4	849974.1777	22.26633944
UIJ69357.1	aspartate_aminotransferase_family_protein_[Bacillus_cereus]	CPTF_Ni	3683171.9	522249.9953	14.17935436
UIJ69357.1	aspartate_aminotransferase_family_protein_[Bacillus_cereus]	CPTF_U	4242822.133	862028.525	20.31733827
UIJ69357.1	aspartate_aminotransferase_family_protein_[Bacillus_cereus]	CPTF_metals_mix	2252188.767	185940.51	8.255991361
UIJ69357.1	aspartate_aminotransferase_family_protein_[Bacillus_cereus]	CPTF_zcontrol	4102640.967	295176.3839	7.19478956
UIJ69363.1	acetolactate_synthase_large_subunit_[Bacillus_cereus]	CPTF_Al	783333.3333	683690.9633	87.27969745
UIJ69363.1	acetolactate_synthase_large_subunit_[Bacillus_cereus]	CPTF_Cd	1150000	104403.0651	9.078527399
UIJ69363.1	acetolactate_synthase_large_subunit_[Bacillus_cereus]	CPTF_Co	413333.3333	715914.3338	173.2050808
UIJ69363.1	acetolactate_synthase_large_subunit_[Bacillus_cereus]	CPTF_Cu	1186666.667	150443.788	12.6778473
UIJ69363.1	acetolactate_synthase_large_subunit_[Bacillus_cereus]	CPTF_Fe	1340000	80000	5.970149254
UIJ69363.1	acetolactate_synthase_large_subunit_[Bacillus_cereus]	CPTF_Mn	1039559	180661.9037	17.37870613
UIJ69363.1	acetolactate_synthase_large_subunit_[Bacillus_cereus]	CPTF_Ni	1350000	180831.4132	13.3949195
UIJ69363.1	acetolactate_synthase_large_subunit_[Bacillus_cereus]	CPTF_U	370000	640858.7988	173.2050808
UIJ69363.1	acetolactate_synthase_large_subunit_[Bacillus_cereus]	CPTF_metals_mix	566079	9564.627384	1.689627664
UIJ69363.1	acetolactate_synthase_large_subunit_[Bacillus_cereus]	CPTF_zcontrol	1230000	115325.6259	9.37606715
UIJ69368.1	GNAT_family_N-acetyltransferase_[Bacillus_cereus]	CPTF_Al	0	0	0
UIJ69368.1	GNAT_family_N-acetyltransferase_[Bacillus_cereus]	CPTF_Cd	0	0	0
UIJ69368.1	GNAT_family_N-acetyltransferase_[Bacillus_cereus]	CPTF_Co	0	0	0
UIJ69368.1	GNAT_family_N-acetyltransferase_[Bacillus_cereus]	CPTF_Cu	10908.13333	18893.44115	173.2050808
UIJ69368.1	GNAT_family_N-acetyltransferase_[Bacillus_cereus]	CPTF_Fe	0	0	0
UIJ69368.1	GNAT_family_N-acetyltransferase_[Bacillus_cereus]	CPTF_Mn	0	0	0
UIJ69368.1	GNAT_family_N-acetyltransferase_[Bacillus_cereus]	CPTF_Ni	15284.1	26472.83775	173.2050808
UIJ69368.1	GNAT_family_N-acetyltransferase_[Bacillus_cereus]	CPTF_U	0	0	0
UIJ69368.1	GNAT_family_N-acetyltransferase_[Bacillus_cereus]	CPTF_metals_mix	30527.63333	18647.1097	61.08272296
UIJ69368.1	GNAT_family_N-acetyltransferase_[Bacillus_cereus]	CPTF_zcontrol	0	0	0
UIJ69381.1	sensor_histidine_kinase_[Bacillus_cereus]	CPTF_Al	13616.96667	23585.27811	173.2050808
UIJ69381.1	sensor_histidine_kinase_[Bacillus_cereus]	CPTF_Cd	88703.5	41179.93452	46.42424991
UIJ69381.1	sensor_histidine_kinase_[Bacillus_cereus]	CPTF_Co	63204.43333	58032.36229	91.81691732
UIJ69381.1	sensor_histidine_kinase_[Bacillus_cereus]	CPTF_Cu	94220.2	128976.1919	136.8880472
UIJ69381.1	sensor_histidine_kinase_[Bacillus_cereus]	CPTF_Fe	43547.66667	17249.16539	39.60984986
UIJ69381.1	sensor_histidine_kinase_[Bacillus_cereus]	CPTF_Mn	57839.9	69436.9741	120.0503011
UIJ69381.1	sensor_histidine_kinase_[Bacillus_cereus]	CPTF_Ni	0	0	0
UIJ69381.1	sensor_histidine_kinase_[Bacillus_cereus]	CPTF_U	0	0	0
UIJ69381.1	sensor_histidine_kinase_[Bacillus_cereus]	CPTF_metals_mix	150709.3667	100310.4724	66.55888391
UIJ69381.1	sensor_histidine_kinase_[Bacillus_cereus]	CPTF_zcontrol	45209.03333	51620.148	114.1810479
UIJ69388.1	HAD-IA_family_hydrolase_[Bacillus_cereus]	CPTF_Al	0	0	0
UIJ69388.1	HAD-IA_family_hydrolase_[Bacillus_cereus]	CPTF_Cd	34014.2	29709.20864	87.34354664
UIJ69388.1	HAD-IA_family_hydrolase_[Bacillus_cereus]	CPTF_Co	5099.033333	8831.784803	173.2050808
UIJ69388.1	HAD-IA_family_hydrolase_[Bacillus_cereus]	CPTF_Cu	4297.533333	7443.546081	173.2050808
UIJ69388.1	HAD-IA_family_hydrolase_[Bacillus_cereus]	CPTF_Fe	0	0	0
UIJ69388.1	HAD-IA_family_hydrolase_[Bacillus_cereus]	CPTF_Mn	0	0	0

UIJ69388.1	HAD-IA_family_hydrolase_[Bacillus_cereus]	CPTF_Ni	0	0	0
UIJ69388.1	HAD-IA_family_hydrolase_[Bacillus_cereus]	CPTF_U	0	0	0
UIJ69388.1	HAD-IA_family_hydrolase_[Bacillus_cereus]	CPTF_metals_mix	41875.76667	36697.00662	87.63303825
UIJ69388.1	HAD-IA_family_hydrolase_[Bacillus_cereus]	CPTF_zcontrol	0	0	0
UIJ69391.1	AAC(3)_family_N-acetyltransferase_[Bacillus_cereus]	CPTF_Al	550516.2333	30466.50305	5.534169785
UIJ69391.1	AAC(3)_family_N-acetyltransferase_[Bacillus_cereus]	CPTF_Cd	532968	11803.82027	2.214733393
UIJ69391.1	AAC(3)_family_N-acetyltransferase_[Bacillus_cereus]	CPTF_Co	774997.7	389481.4294	50.25581745
UIJ69391.1	AAC(3)_family_N-acetyltransferase_[Bacillus_cereus]	CPTF_Cu	497375.4333	53544.18776	10.76534629
UIJ69391.1	AAC(3)_family_N-acetyltransferase_[Bacillus_cereus]	CPTF_Fe	551475.3333	13059.76173	2.36814975
UIJ69391.1	AAC(3)_family_N-acetyltransferase_[Bacillus_cereus]	CPTF_Mn	983301.7333	567708.8158	57.73495526
UIJ69391.1	AAC(3)_family_N-acetyltransferase_[Bacillus_cereus]	CPTF_Ni	817719.8333	416523.7654	50.93722183
UIJ69391.1	AAC(3)_family_N-acetyltransferase_[Bacillus_cereus]	CPTF_U	1070148	667475.2864	62.3722407
UIJ69391.1	AAC(3)_family_N-acetyltransferase_[Bacillus_cereus]	CPTF_metals_mix	486154.3333	9943.938874	2.045428415
UIJ69391.1	AAC(3)_family_N-acetyltransferase_[Bacillus_cereus]	CPTF_zcontrol	576932.8333	90946.25866	15.76375159
UIJ69412.1	GyrI-like_domain-containing_protein_[Bacillus_cereus]	CPTF_Al	0	0	0
UIJ69412.1	GyrI-like_domain-containing_protein_[Bacillus_cereus]	CPTF_Cd	6581.4	11399.31918	173.2050808
UIJ69412.1	GyrI-like_domain-containing_protein_[Bacillus_cereus]	CPTF_Co	0	0	0
UIJ69412.1	GyrI-like_domain-containing_protein_[Bacillus_cereus]	CPTF_Cu	13614.56667	23581.12119	173.2050808
UIJ69412.1	GyrI-like_domain-containing_protein_[Bacillus_cereus]	CPTF_Fe	18565.13333	16235.53609	87.45176132
UIJ69412.1	GyrI-like_domain-containing_protein_[Bacillus_cereus]	CPTF_Mn	0	0	0
UIJ69412.1	GyrI-like_domain-containing_protein_[Bacillus_cereus]	CPTF_Ni	0	0	0
UIJ69412.1	GyrI-like_domain-containing_protein_[Bacillus_cereus]	CPTF_U	0	0	0
UIJ69412.1	GyrI-like_domain-containing_protein_[Bacillus_cereus]	CPTF_metals_mix	7690.066667	6688.18416	86.97173185
UIJ69412.1	GyrI-like_domain-containing_protein_[Bacillus_cereus]	CPTF_zcontrol	0	0	0
UIJ69423.1	acyl_carrier_protein_[Bacillus_cereus]	CPTF_Al	0	0	0
UIJ69423.1	acyl_carrier_protein_[Bacillus_cereus]	CPTF_Cd	0	0	0
UIJ69423.1	acyl_carrier_protein_[Bacillus_cereus]	CPTF_Co	0	0	0
UIJ69423.1	acyl_carrier_protein_[Bacillus_cereus]	CPTF_Cu	0	0	0
UIJ69423.1	acyl_carrier_protein_[Bacillus_cereus]	CPTF_Fe	0	0	0
UIJ69423.1	acyl_carrier_protein_[Bacillus_cereus]	CPTF_Mn	0	0	0
UIJ69423.1	acyl_carrier_protein_[Bacillus_cereus]	CPTF_Ni	0	0	0
UIJ69423.1	acyl_carrier_protein_[Bacillus_cereus]	CPTF_U	0	0	0
UIJ69423.1	acyl_carrier_protein_[Bacillus_cereus]	CPTF_metals_mix	1309261.333	651036.2096	49.72545916
UIJ69423.1	acyl_carrier_protein_[Bacillus_cereus]	CPTF_zcontrol	0	0	0
UIJ69429.1	cytochrome_c_oxidase_subunit_I_[Bacillus_cereus]	CPTF_Al	258441	90764.17751	35.11988327
UIJ69429.1	cytochrome_c_oxidase_subunit_I_[Bacillus_cereus]	CPTF_Cd	387550.6667	50309.16607	12.98131326
UIJ69429.1	cytochrome_c_oxidase_subunit_I_[Bacillus_cereus]	CPTF_Co	198521.6667	56676.3752	28.54921387
UIJ69429.1	cytochrome_c_oxidase_subunit_I_[Bacillus_cereus]	CPTF_Cu	127071.3333	17157.81155	13.50250375
UIJ69429.1	cytochrome_c_oxidase_subunit_I_[Bacillus_cereus]	CPTF_Fe	300180.3333	82878.7513	27.60965396
UIJ69429.1	cytochrome_c_oxidase_subunit_I_[Bacillus_cereus]	CPTF_Mn	251982.1667	177872.7093	70.58940386
UIJ69429.1	cytochrome_c_oxidase_subunit_I_[Bacillus_cereus]	CPTF_Ni	0	0	0
UIJ69429.1	cytochrome_c_oxidase_subunit_I_[Bacillus_cereus]	CPTF_U	133707.3333	119323.6737	89.24243026
UIJ69429.1	cytochrome_c_oxidase_subunit_I_[Bacillus_cereus]	CPTF_metals_mix	191041.3333	16242.90923	8.50230102
UIJ69429.1	cytochrome_c_oxidase_subunit_I_[Bacillus_cereus]	CPTF_zcontrol	177834.8	98014.62893	55.11555046
UIJ69433.1	carbon-nitrogen_family_hydrolase_[Bacillus_cereus]	CPTF_Al	0	0	0
UIJ69433.1	carbon-nitrogen_family_hydrolase_[Bacillus_cereus]	CPTF_Cd	29371.56667	50873.04576	173.2050808
UIJ69433.1	carbon-nitrogen_family_hydrolase_[Bacillus_cereus]	CPTF_Co	0	0	0
UIJ69433.1	carbon-nitrogen_family_hydrolase_[Bacillus_cereus]	CPTF_Cu	47593.1	41381.48878	86.94850467
UIJ69433.1	carbon-nitrogen_family_hydrolase_[Bacillus_cereus]	CPTF_Fe	0	0	0
UIJ69433.1	carbon-nitrogen_family_hydrolase_[Bacillus_cereus]	CPTF_Mn	24096.23333	41735.9004	173.2050808
UIJ69433.1	carbon-nitrogen_family_hydrolase_[Bacillus_cereus]	CPTF_Ni	0	0	0
UIJ69433.1	carbon-nitrogen_family_hydrolase_[Bacillus_cereus]	CPTF_U	0	0	0
UIJ69433.1	carbon-nitrogen_family_hydrolase_[Bacillus_cereus]	CPTF_metals_mix	0	0	0
UIJ69433.1	carbon-nitrogen_family_hydrolase_[Bacillus_cereus]	CPTF_zcontrol	102455.7	88737.01265	86.61012775
UIJ69434.1	GntR_family_transcriptional_regulator_[Bacillus_cereus]	CPTF_Al	96668.86667	150120.385	155.2934157
UIJ69434.1	GntR_family_transcriptional_regulator_[Bacillus_cereus]	CPTF_Cd	370179.8	12495.68769	3.375572543
UIJ69434.1	GntR_family_transcriptional_regulator_[Bacillus_cereus]	CPTF_Co	92092.36667	79698.46783	86.54188259
UIJ69434.1	GntR_family_transcriptional_regulator_[Bacillus_cereus]	CPTF_Cu	384180.7	43378.8776	11.29126934
UIJ69434.1	GntR_family_transcriptional_regulator_[Bacillus_cereus]	CPTF_Fe	318112.9	41080.40007	12.91378001
UIJ69434.1	GntR_family_transcriptional_regulator_[Bacillus_cereus]	CPTF_Mn	182729.4	176598.0593	96.64457896
UIJ69434.1	GntR_family_transcriptional_regulator_[Bacillus_cereus]	CPTF_Ni	38701.93333	28662.47524	74.05954372

UIJ69434.1	GntR_family_transcriptional_regulator_[Bacillus_cereus]	CPTF_U	0	0	0
UIJ69434.1	GntR_family_transcriptional_regulator_[Bacillus_cereus]	CPTF_metals_mix	346376.5667	24632.58187	7.111503559
UIJ69434.1	GntR_family_transcriptional_regulator_[Bacillus_cereus]	CPTF_zcontrol	166981.6667	157566.971	94.36183875
UIJ69438.1	L-serine_ammonia-lyase,_iron-sulfur-dependent_subunit_beta_[Bacillus_cereus]	CPTF_Al	88268.7	49886.85796	56.51704167
UIJ69438.1	L-serine_ammonia-lyase,_iron-sulfur-dependent_subunit_beta_[Bacillus_cereus]	CPTF_Cd	102861.2333	58277.20753	56.65614307
UIJ69438.1	L-serine_ammonia-lyase,_iron-sulfur-dependent_subunit_beta_[Bacillus_cereus]	CPTF_Co	128264.6667	24119.72415	18.80465196
UIJ69438.1	L-serine_ammonia-lyase,_iron-sulfur-dependent_subunit_beta_[Bacillus_cereus]	CPTF_Cu	123284.9667	48306.53427	39.18282624
UIJ69438.1	L-serine_ammonia-lyase,_iron-sulfur-dependent_subunit_beta_[Bacillus_cereus]	CPTF_Fe	96577.26667	62693.7812	64.91567153
UIJ69438.1	L-serine_ammonia-lyase,_iron-sulfur-dependent_subunit_beta_[Bacillus_cereus]	CPTF_Mn	68634.9	59807.59242	87.13874781
UIJ69438.1	L-serine_ammonia-lyase,_iron-sulfur-dependent_subunit_beta_[Bacillus_cereus]	CPTF_Ni	164719.1667	18641.93822	11.31740683
UIJ69438.1	L-serine_ammonia-lyase,_iron-sulfur-dependent_subunit_beta_[Bacillus_cereus]	CPTF_U	47113.73333	56333.22515	119.5685868
UIJ69438.1	L-serine_ammonia-lyase,_iron-sulfur-dependent_subunit_beta_[Bacillus_cereus]	CPTF_metals_mix	269295.6667	30217.57532	11.22096605
UIJ69438.1	L-serine_ammonia-lyase,_iron-sulfur-dependent_subunit_beta_[Bacillus_cereus]	CPTF_zcontrol	75157.36667	50322.82942	66.95661603
UIJ69439.1	BrxA/BrxB_family_bacilliredoxin_[Bacillus_cereus]	CPTF_Al	660914.4333	155366.4154	23.5077958
UIJ69439.1	BrxA/BrxB_family_bacilliredoxin_[Bacillus_cereus]	CPTF_Cd	773002.9667	89816.5663	11.61917485
UIJ69439.1	BrxA/BrxB_family_bacilliredoxin_[Bacillus_cereus]	CPTF_Co	768544.8	77500.94448	10.08411539
UIJ69439.1	BrxA/BrxB_family_bacilliredoxin_[Bacillus_cereus]	CPTF_Cu	613073.4	83704.88994	13.65332274
UIJ69439.1	BrxA/BrxB_family_bacilliredoxin_[Bacillus_cereus]	CPTF_Fe	558110.5333	20703.63778	3.709594524
UIJ69439.1	BrxA/BrxB_family_bacilliredoxin_[Bacillus_cereus]	CPTF_Mn	632144.7333	110475.8922	17.47636045
UIJ69439.1	BrxA/BrxB_family_bacilliredoxin_[Bacillus_cereus]	CPTF_Ni	525732.9333	82883.25431	15.76527721
UIJ69439.1	BrxA/BrxB_family_bacilliredoxin_[Bacillus_cereus]	CPTF_U	254849.9667	130589.8272	51.24184591
UIJ69439.1	BrxA/BrxB_family_bacilliredoxin_[Bacillus_cereus]	CPTF_metals_mix	822142.2667	45600.79332	5.546581798
UIJ69439.1	BrxA/BrxB_family_bacilliredoxin_[Bacillus_cereus]	CPTF_zcontrol	585386.3333	88704.24749	15.15311213
UIJ69446.1	flavodoxin-dependent_(E)-4-hydroxy-3-methylbut-2-enyl-diphosphate_synthase_[Bacillus_cereus]	CPTF_Al	305220	277013.3137	90.75857209
UIJ69446.1	flavodoxin-dependent_(E)-4-hydroxy-3-methylbut-2-enyl-diphosphate_synthase_[Bacillus_cereus]	CPTF_Cd	235717.3333	204881.2435	86.91819165
UIJ69446.1	flavodoxin-dependent_(E)-4-hydroxy-3-methylbut-2-enyl-diphosphate_synthase_[Bacillus_cereus]	CPTF_Co	130118	225370.987	173.2050808
UIJ69446.1	flavodoxin-dependent_(E)-4-hydroxy-3-methylbut-2-enyl-diphosphate_synthase_[Bacillus_cereus]	CPTF_Cu	293497.3333	321279.496	109.4658995
UIJ69446.1	flavodoxin-dependent_(E)-4-hydroxy-3-methylbut-2-enyl-diphosphate_synthase_[Bacillus_cereus]	CPTF_Fe	126688.3333	219430.6301	173.2050808
UIJ69446.1	flavodoxin-dependent_(E)-4-hydroxy-3-methylbut-2-enyl-diphosphate_synthase_[Bacillus_cereus]	CPTF_Mn	138533.0333	167373.6732	120.8186013
UIJ69446.1	flavodoxin-dependent_(E)-4-hydroxy-3-methylbut-2-enyl-diphosphate_synthase_[Bacillus_cereus]	CPTF_Ni	295617.3333	123933.8049	41.92372739
UIJ69446.1	flavodoxin-dependent_(E)-4-hydroxy-3-methylbut-2-enyl-diphosphate_synthase_[Bacillus_cereus]	CPTF_U	278994.6667	247881.059	88.8479561
UIJ69446.1	flavodoxin-dependent_(E)-4-hydroxy-3-methylbut-2-enyl-diphosphate_synthase_[Bacillus_cereus]	CPTF_metals_mix	1918872.367	227874.2281	11.87542392
UIJ69446.1	flavodoxin-dependent_(E)-4-hydroxy-3-methylbut-2-enyl-diphosphate_synthase_[Bacillus_cereus]	CPTF_zcontrol	0	0	0
UIJ69448.1	tRNA_2-thiouridine(34)_synthase_MnmA_[Bacillus_cereus]	CPTF_Al	0	0	0
UIJ69448.1	tRNA_2-thiouridine(34)_synthase_MnmA_[Bacillus_cereus]	CPTF_Cd	151851.1	114976.9198	75.71688303
UIJ69448.1	tRNA_2-thiouridine(34)_synthase_MnmA_[Bacillus_cereus]	CPTF_Co	54851.83333	48413.34647	88.2620389
UIJ69448.1	tRNA_2-thiouridine(34)_synthase_MnmA_[Bacillus_cereus]	CPTF_Cu	14551.46667	25203.87959	173.2050808
UIJ69448.1	tRNA_2-thiouridine(34)_synthase_MnmA_[Bacillus_cereus]	CPTF_Fe	52173.73333	45195.21789	86.62446599
UIJ69448.1	tRNA_2-thiouridine(34)_synthase_MnmA_[Bacillus_cereus]	CPTF_Mn	42525	73655.46059	173.2050808
UIJ69448.1	tRNA_2-thiouridine(34)_synthase_MnmA_[Bacillus_cereus]	CPTF_Ni	42753.53333	38419.73179	89.86329034
UIJ69448.1	tRNA_2-thiouridine(34)_synthase_MnmA_[Bacillus_cereus]	CPTF_U	0	0	0
UIJ69448.1	tRNA_2-thiouridine(34)_synthase_MnmA_[Bacillus_cereus]	CPTF_metals_mix	359303.3333	16129.96507	4.48923335
UIJ69448.1	tRNA_2-thiouridine(34)_synthase_MnmA_[Bacillus_cereus]	CPTF_zcontrol	37389.36667	34506.20216	92.28881159
UIJ69450.1	DNA_polymerase/3'-5'_exonuclease_PolX_[Bacillus_cereus]	CPTF_Al	456570.6667	170996.117	37.45227837
UIJ69450.1	DNA_polymerase/3'-5'_exonuclease_PolX_[Bacillus_cereus]	CPTF_Cd	347248.3333	247019.9886	71.13640725
UIJ69450.1	DNA_polymerase/3'-5'_exonuclease_PolX_[Bacillus_cereus]	CPTF_Co	433125.6667	275708.7693	63.65560633
UIJ69450.1	DNA_polymerase/3'-5'_exonuclease_PolX_[Bacillus_cereus]	CPTF_Cu	571148.4667	43807.86591	7.670136307
UIJ69450.1	DNA_polymerase/3'-5'_exonuclease_PolX_[Bacillus_cereus]	CPTF_Fe	372223.3333	99868.30154	26.83020988
UIJ69450.1	DNA_polymerase/3'-5'_exonuclease_PolX_[Bacillus_cereus]	CPTF_Mn	221705.6667	97873.73525	44.14579777
UIJ69450.1	DNA_polymerase/3'-5'_exonuclease_PolX_[Bacillus_cereus]	CPTF_Ni	366188.9333	104162.7312	28.44507895
UIJ69450.1	DNA_polymerase/3'-5'_exonuclease_PolX_[Bacillus_cereus]	CPTF_U	163229.6333	282722.0182	173.2050808
UIJ69450.1	DNA_polymerase/3'-5'_exonuclease_PolX_[Bacillus_cereus]	CPTF_metals_mix	335982.9	77963.71936	23.20466886
UIJ69450.1	DNA_polymerase/3'-5'_exonuclease_PolX_[Bacillus_cereus]	CPTF_zcontrol	365703.3333	138037.2456	37.74568976
UIJ69451.1	S-adenosylmethionine_decarboxylase_proenzyme_[Bacillus_cereus]	CPTF_Al	66850.1	24619.85236	36.82844507
UIJ69451.1	S-adenosylmethionine_decarboxylase_proenzyme_[Bacillus_cereus]	CPTF_Cd	70278.33333	12127.65893	17.25661147
UIJ69451.1	S-adenosylmethionine_decarboxylase_proenzyme_[Bacillus_cereus]	CPTF_Co	334021.6333	72903.6451	21.82602497
UIJ69451.1	S-adenosylmethionine_decarboxylase_proenzyme_[Bacillus_cereus]	CPTF_Cu	103674.9333	179570.252	173.2050808
UIJ69451.1	S-adenosylmethionine_decarboxylase_proenzyme_[Bacillus_cereus]	CPTF_Fe	40031.1	36434.81957	91.01628375
UIJ69451.1	S-adenosylmethionine_decarboxylase_proenzyme_[Bacillus_cereus]	CPTF_Mn	166687.6333	95021.45821	57.00570361
UIJ69451.1	S-adenosylmethionine_decarboxylase_proenzyme_[Bacillus_cereus]	CPTF_Ni	185012.9667	91746.83178	49.58940632
UIJ69451.1	S-adenosylmethionine_decarboxylase_proenzyme_[Bacillus_cereus]	CPTF_U	315636	20700.73769	6.558420995

UIJ69451.1	S-adenosylmethionine_decarboxylase_proenzyme_[Bacillus_cereus]	CPTF_metals_mix	84740.66667	146775.1401	173.2050808
UIJ69451.1	S-adenosylmethionine_decarboxylase_proenzyme_[Bacillus_cereus]	CPTF_zcontrol	32381.66667	56086.6919	173.2050808
UIJ69452.1	NAD-dependent_malic_enzyme_[Bacillus_cereus]	CPTF_Al	558209.6	91487.25214	16.38940859
UIJ69452.1	NAD-dependent_malic_enzyme_[Bacillus_cereus]	CPTF_Cd	947360.4333	240779.268	25.41580369
UIJ69452.1	NAD-dependent_malic_enzyme_[Bacillus_cereus]	CPTF_Co	588433.2667	146391.3789	24.87816159
UIJ69452.1	NAD-dependent_malic_enzyme_[Bacillus_cereus]	CPTF_Cu	602724	96100.79098	15.94441087
UIJ69452.1	NAD-dependent_malic_enzyme_[Bacillus_cereus]	CPTF_Fe	647377.3333	128918.2237	19.91392301
UIJ69452.1	NAD-dependent_malic_enzyme_[Bacillus_cereus]	CPTF_Mn	787263.2667	271772.5152	34.52117312
UIJ69452.1	NAD-dependent_malic_enzyme_[Bacillus_cereus]	CPTF_Ni	355245.5333	127287.4255	35.83083065
UIJ69452.1	NAD-dependent_malic_enzyme_[Bacillus_cereus]	CPTF_U	233163.1	74740.0361	32.05483033
UIJ69452.1	NAD-dependent_malic_enzyme_[Bacillus_cereus]	CPTF_metals_mix	1721338.167	247473.7608	14.37682412
UIJ69452.1	NAD-dependent_malic_enzyme_[Bacillus_cereus]	CPTF_zcontrol	438594.2333	153305.9305	34.9539321
UIJ69455.1	acetyltransferase_[Bacillus_cereus]	CPTF_Al	126579.7333	46477.18431	36.71771387
UIJ69455.1	acetyltransferase_[Bacillus_cereus]	CPTF_Cd	182416.8	159780.8818	87.59110003
UIJ69455.1	acetyltransferase_[Bacillus_cereus]	CPTF_Co	72904.13333	67786.91444	92.98089331
UIJ69455.1	acetyltransferase_[Bacillus_cereus]	CPTF_Cu	62841	108843.8048	173.2050808
UIJ69455.1	acetyltransferase_[Bacillus_cereus]	CPTF_Fe	0	0	0
UIJ69455.1	acetyltransferase_[Bacillus_cereus]	CPTF_Mn	42201.33333	73094.85348	173.2050808
UIJ69455.1	acetyltransferase_[Bacillus_cereus]	CPTF_Ni	0	0	0
UIJ69455.1	acetyltransferase_[Bacillus_cereus]	CPTF_U	43696	75683.69209	173.2050808
UIJ69455.1	acetyltransferase_[Bacillus_cereus]	CPTF_metals_mix	53195	92136.44271	173.2050808
UIJ69455.1	acetyltransferase_[Bacillus_cereus]	CPTF_zcontrol	175987.1667	72073.42902	40.95379816
UIJ69464.1	glycogen_synthase_GlgA_[Bacillus_cereus]	CPTF_Al	4030632.467	207217.3561	5.141063043
UIJ69464.1	glycogen_synthase_GlgA_[Bacillus_cereus]	CPTF_Cd	3708979.633	309669.3742	8.349179688
UIJ69464.1	glycogen_synthase_GlgA_[Bacillus_cereus]	CPTF_Co	3769974.867	1161823.789	30.81781259
UIJ69464.1	glycogen_synthase_GlgA_[Bacillus_cereus]	CPTF_Cu	3410093.5	1302951.998	38.20868837
UIJ69464.1	glycogen_synthase_GlgA_[Bacillus_cereus]	CPTF_Fe	3549398.633	1367297.474	38.52194739
UIJ69464.1	glycogen_synthase_GlgA_[Bacillus_cereus]	CPTF_Mn	2293817.8	160486.9616	6.996499964
UIJ69464.1	glycogen_synthase_GlgA_[Bacillus_cereus]	CPTF_Ni	4000735.4	1305701.376	32.63653418
UIJ69464.1	glycogen_synthase_GlgA_[Bacillus_cereus]	CPTF_U	2461222.667	1566003.785	63.62706661
UIJ69464.1	glycogen_synthase_GlgA_[Bacillus_cereus]	CPTF_metals_mix	1563141.267	113696.371	7.273582588
UIJ69464.1	glycogen_synthase_GlgA_[Bacillus_cereus]	CPTF_zcontrol	4285373.833	201813.3448	4.709352152
UIJ69465.1	UTP--glucose-1-phosphate_uridylyltransferase_GalU_[Bacillus_cereus]	CPTF_Al	5916698	373985.7472	6.320852395
UIJ69465.1	UTP--glucose-1-phosphate_uridylyltransferase_GalU_[Bacillus_cereus]	CPTF_Cd	5990580.367	182856.7378	3.052404385
UIJ69465.1	UTP--glucose-1-phosphate_uridylyltransferase_GalU_[Bacillus_cereus]	CPTF_Co	5538925.633	254419.7847	4.593305661
UIJ69465.1	UTP--glucose-1-phosphate_uridylyltransferase_GalU_[Bacillus_cereus]	CPTF_Cu	5877148	136455.446	2.321797002
UIJ69465.1	UTP--glucose-1-phosphate_uridylyltransferase_GalU_[Bacillus_cereus]	CPTF_Fe	5585719.8	384226.7105	6.878732271
UIJ69465.1	UTP--glucose-1-phosphate_uridylyltransferase_GalU_[Bacillus_cereus]	CPTF_Mn	5527005.567	446808.7237	8.084101207
UIJ69465.1	UTP--glucose-1-phosphate_uridylyltransferase_GalU_[Bacillus_cereus]	CPTF_Ni	5147965.667	169606.7045	3.294635502
UIJ69465.1	UTP--glucose-1-phosphate_uridylyltransferase_GalU_[Bacillus_cereus]	CPTF_U	5343702.333	257051.8117	4.810369211
UIJ69465.1	UTP--glucose-1-phosphate_uridylyltransferase_GalU_[Bacillus_cereus]	CPTF_metals_mix	5387441.467	215895.154	4.007378185
UIJ69465.1	UTP--glucose-1-phosphate_uridylyltransferase_GalU_[Bacillus_cereus]	CPTF_zcontrol	5659727.933	221348.5581	3.910939901
UIJ69468.1	YutD_family_protein_[Bacillus_cereus]	CPTF_Al	565402.6333	81981.89213	14.49973652
UIJ69468.1	YutD_family_protein_[Bacillus_cereus]	CPTF_Cd	503087.3333	2741.970338	0.545028697
UIJ69468.1	YutD_family_protein_[Bacillus_cereus]	CPTF_Co	598050.9333	97574.90156	16.31548353
UIJ69468.1	YutD_family_protein_[Bacillus_cereus]	CPTF_Cu	611203	56333.47727	9.216819496
UIJ69468.1	YutD_family_protein_[Bacillus_cereus]	CPTF_Fe	636342.2	84151.16048	13.22419926
UIJ69468.1	YutD_family_protein_[Bacillus_cereus]	CPTF_Mn	668856	46457.85414	6.94586789
UIJ69468.1	YutD_family_protein_[Bacillus_cereus]	CPTF_Ni	726425.7667	179551.4813	24.71711351
UIJ69468.1	YutD_family_protein_[Bacillus_cereus]	CPTF_U	728123.6667	210418.7462	28.89876484
UIJ69468.1	YutD_family_protein_[Bacillus_cereus]	CPTF_metals_mix	634250.3333	40991.40038	6.462968677
UIJ69468.1	YutD_family_protein_[Bacillus_cereus]	CPTF_zcontrol	618428.3	140173.7184	22.66612288
UIJ69475.1	TIGR00730_family_Rossman_fold_protein_[Bacillus_cereus]	CPTF_Al	171416.1	76313.45587	44.51942138
UIJ69475.1	TIGR00730_family_Rossman_fold_protein_[Bacillus_cereus]	CPTF_Cd	113026.6667	26284.55614	23.2551812
UIJ69475.1	TIGR00730_family_Rossman_fold_protein_[Bacillus_cereus]	CPTF_Co	125499.6667	962.819611	0.767188979
UIJ69475.1	TIGR00730_family_Rossman_fold_protein_[Bacillus_cereus]	CPTF_Cu	106506.9667	43306.98277	40.66117375
UIJ69475.1	TIGR00730_family_Rossman_fold_protein_[Bacillus_cereus]	CPTF_Fe	135615.7667	58236.06771	42.94195959
UIJ69475.1	TIGR00730_family_Rossman_fold_protein_[Bacillus_cereus]	CPTF_Mn	223485	125059.9698	55.95899938
UIJ69475.1	TIGR00730_family_Rossman_fold_protein_[Bacillus_cereus]	CPTF_Ni	175826.8667	53342.01856	30.33780876
UIJ69475.1	TIGR00730_family_Rossman_fold_protein_[Bacillus_cereus]	CPTF_U	92942.2	76894.28426	82.73344536
UIJ69475.1	TIGR00730_family_Rossman_fold_protein_[Bacillus_cereus]	CPTF_metals_mix	121352.5333	28694.88604	23.64588958

UIJ69475.1	TIGR00730_family_Rossman_fold_protein_[Bacillus_cereus]	CPTF_zcontrol	107950.2	68402.85292	63.36519332
UIJ69478.1	carboxylesterase_[Bacillus_cereus]	CPTF_Al	82449.66667	71734.24517	87.00368124
UIJ69478.1	carboxylesterase_[Bacillus_cereus]	CPTF_Cd	114625.5	19726.65051	17.20965275
UIJ69478.1	carboxylesterase_[Bacillus_cereus]	CPTF_Co	138292.3333	21817.60299	15.77643711
UIJ69478.1	carboxylesterase_[Bacillus_cereus]	CPTF_Cu	155779.3333	40134.86152	25.76391917
UIJ69478.1	carboxylesterase_[Bacillus_cereus]	CPTF_Fe	98437.76667	72671.25322	73.82456519
UIJ69478.1	carboxylesterase_[Bacillus_cereus]	CPTF_Mn	46619	80746.4766	173.2050808
UIJ69478.1	carboxylesterase_[Bacillus_cereus]	CPTF_Ni	107779.3667	55920.00982	51.88378031
UIJ69478.1	carboxylesterase_[Bacillus_cereus]	CPTF_U	96938	84001.85072	86.65523398
UIJ69478.1	carboxylesterase_[Bacillus_cereus]	CPTF_metals_mix	257687.7333	145661.0122	56.52617233
UIJ69478.1	carboxylesterase_[Bacillus_cereus]	CPTF_zcontrol	81112	71830.19878	88.55680883
UIJ69479.1	S41_family_peptidase_[Bacillus_cereus]	CPTF_Al	0	0	0
UIJ69479.1	S41_family_peptidase_[Bacillus_cereus]	CPTF_Cd	43200	74824.59489	173.2050808
UIJ69479.1	S41_family_peptidase_[Bacillus_cereus]	CPTF_Co	0	0	0
UIJ69479.1	S41_family_peptidase_[Bacillus_cereus]	CPTF_Cu	37756.66667	65396.46499	173.2050808
UIJ69479.1	S41_family_peptidase_[Bacillus_cereus]	CPTF_Fe	0	0	0
UIJ69479.1	S41_family_peptidase_[Bacillus_cereus]	CPTF_Mn	0	0	0
UIJ69479.1	S41_family_peptidase_[Bacillus_cereus]	CPTF_Ni	0	0	0
UIJ69479.1	S41_family_peptidase_[Bacillus_cereus]	CPTF_U	0	0	0
UIJ69479.1	S41_family_peptidase_[Bacillus_cereus]	CPTF_metals_mix	53879.76667	76797.30891	142.5345982
UIJ69479.1	S41_family_peptidase_[Bacillus_cereus]	CPTF_zcontrol	37326.33333	64651.10579	173.2050808
UIJ69480.1	cell_division_ATP-binding_protein_FtsE_[Bacillus_cereus]	CPTF_Al	1273995	105066.7314	8.247028552
UIJ69480.1	cell_division_ATP-binding_protein_FtsE_[Bacillus_cereus]	CPTF_Cd	1323201	36395.40691	2.750557694
UIJ69480.1	cell_division_ATP-binding_protein_FtsE_[Bacillus_cereus]	CPTF_Co	1314815.667	27388.23971	2.083047868
UIJ69480.1	cell_division_ATP-binding_protein_FtsE_[Bacillus_cereus]	CPTF_Cu	1358846.233	78072.87582	5.745526896
UIJ69480.1	cell_division_ATP-binding_protein_FtsE_[Bacillus_cereus]	CPTF_Fe	1306976.667	150171.1901	11.48996719
UIJ69480.1	cell_division_ATP-binding_protein_FtsE_[Bacillus_cereus]	CPTF_Mn	1233179.333	105906.6289	8.588096319
UIJ69480.1	cell_division_ATP-binding_protein_FtsE_[Bacillus_cereus]	CPTF_Ni	1327890.333	125208.6222	9.429138769
UIJ69480.1	cell_division_ATP-binding_protein_FtsE_[Bacillus_cereus]	CPTF_U	1540303	267125.5224	17.34240097
UIJ69480.1	cell_division_ATP-binding_protein_FtsE_[Bacillus_cereus]	CPTF_metals_mix	1814699.633	298456.0172	16.44657946
UIJ69480.1	cell_division_ATP-binding_protein_FtsE_[Bacillus_cereus]	CPTF_zcontrol	1325069	126826.6175	9.571321755
UIJ69483.1	cold_shock_protein_CspC_[Bacillus_cereus]	CPTF_Al	373081	28108.94712	7.534274626
UIJ69483.1	cold_shock_protein_CspC_[Bacillus_cereus]	CPTF_Cd	471726.3333	38597.6676	8.182216017
UIJ69483.1	cold_shock_protein_CspC_[Bacillus_cereus]	CPTF_Co	381663.3333	49535.58152	12.97886834
UIJ69483.1	cold_shock_protein_CspC_[Bacillus_cereus]	CPTF_Cu	294546.6667	117171.876	39.78041146
UIJ69483.1	cold_shock_protein_CspC_[Bacillus_cereus]	CPTF_Fe	419714.3333	220240.1883	52.47383062
UIJ69483.1	cold_shock_protein_CspC_[Bacillus_cereus]	CPTF_Mn	270801.7	149871.718	55.34371388
UIJ69483.1	cold_shock_protein_CspC_[Bacillus_cereus]	CPTF_Ni	224248.3333	90468.40803	40.34295671
UIJ69483.1	cold_shock_protein_CspC_[Bacillus_cereus]	CPTF_U	313840.3333	86571.73979	27.58464435
UIJ69483.1	cold_shock_protein_CspC_[Bacillus_cereus]	CPTF_metals_mix	266256.3333	83746.57143	31.45336315
UIJ69483.1	cold_shock_protein_CspC_[Bacillus_cereus]	CPTF_zcontrol	327149	58905.50846	18.00571252
UIJ69487.1	glutamine-hydrolyzing_GMP_synthase_[Bacillus_cereus]	CPTF_Al	8171965.933	444718.8792	5.442006035
UIJ69487.1	glutamine-hydrolyzing_GMP_synthase_[Bacillus_cereus]	CPTF_Cd	9923635.567	325268.8493	3.277718606
UIJ69487.1	glutamine-hydrolyzing_GMP_synthase_[Bacillus_cereus]	CPTF_Co	8253282.667	401937.8706	4.870036406
UIJ69487.1	glutamine-hydrolyzing_GMP_synthase_[Bacillus_cereus]	CPTF_Cu	7727854.167	555917.4446	7.193684464
UIJ69487.1	glutamine-hydrolyzing_GMP_synthase_[Bacillus_cereus]	CPTF_Fe	9411350.167	650501.1619	6.911879277
UIJ69487.1	glutamine-hydrolyzing_GMP_synthase_[Bacillus_cereus]	CPTF_Mn	8325437.467	620055.3169	7.447720547
UIJ69487.1	glutamine-hydrolyzing_GMP_synthase_[Bacillus_cereus]	CPTF_Ni	7430158.867	718873.9099	9.675081284
UIJ69487.1	glutamine-hydrolyzing_GMP_synthase_[Bacillus_cereus]	CPTF_U	7908575.367	568554.5278	7.189089077
UIJ69487.1	glutamine-hydrolyzing_GMP_synthase_[Bacillus_cereus]	CPTF_metals_mix	10104140.83	1051461.102	10.40623958
UIJ69487.1	glutamine-hydrolyzing_GMP_synthase_[Bacillus_cereus]	CPTF_zcontrol	8645139.667	883610.4781	10.22089304
UIJ69494.1	DUF3981_domain-containing_protein_[Bacillus_cereus]	CPTF_Al	32409.56667	56135.01612	173.2050808
UIJ69494.1	DUF3981_domain-containing_protein_[Bacillus_cereus]	CPTF_Cd	121776	23448.59322	19.25551276
UIJ69494.1	DUF3981_domain-containing_protein_[Bacillus_cereus]	CPTF_Co	19380.36667	33567.77974	173.2050808
UIJ69494.1	DUF3981_domain-containing_protein_[Bacillus_cereus]	CPTF_Cu	0	0	0
UIJ69494.1	DUF3981_domain-containing_protein_[Bacillus_cereus]	CPTF_Fe	42178.13333	45824.03185	108.644049
UIJ69494.1	DUF3981_domain-containing_protein_[Bacillus_cereus]	CPTF_Mn	54005.66667	93540.55856	173.2050808
UIJ69494.1	DUF3981_domain-containing_protein_[Bacillus_cereus]	CPTF_Ni	0	0	0
UIJ69494.1	DUF3981_domain-containing_protein_[Bacillus_cereus]	CPTF_U	0	0	0
UIJ69494.1	DUF3981_domain-containing_protein_[Bacillus_cereus]	CPTF_metals_mix	234392	42613.99338	18.18065181
UIJ69494.1	DUF3981_domain-containing_protein_[Bacillus_cereus]	CPTF_zcontrol	0	0	0

UIJ69496.1	50S_ribosomal_protein_L18_[Bacillus_cereus]	CPTF_Al	2540532.667	129471.5145	5.096234982
UIJ69496.1	50S_ribosomal_protein_L18_[Bacillus_cereus]	CPTF_Cd	2304431.333	488826.3632	21.21245082
UIJ69496.1	50S_ribosomal_protein_L18_[Bacillus_cereus]	CPTF_Co	2539127	206536.9298	8.134170909
UIJ69496.1	50S_ribosomal_protein_L18_[Bacillus_cereus]	CPTF_Cu	2121395	134288.3455	6.330190533
UIJ69496.1	50S_ribosomal_protein_L18_[Bacillus_cereus]	CPTF_Fe	2105220.333	74695.74557	3.548120089
UIJ69496.1	50S_ribosomal_protein_L18_[Bacillus_cereus]	CPTF_Mn	2106983.333	102287.3914	4.85468441
UIJ69496.1	50S_ribosomal_protein_L18_[Bacillus_cereus]	CPTF_Ni	2295001.333	409244.0158	17.8319729
UIJ69496.1	50S_ribosomal_protein_L18_[Bacillus_cereus]	CPTF_U	1576862.333	38951.84638	2.470212241
UIJ69496.1	50S_ribosomal_protein_L18_[Bacillus_cereus]	CPTF_metals_mix	1834970.867	206479.4507	11.25246479
UIJ69496.1	50S_ribosomal_protein_L18_[Bacillus_cereus]	CPTF_zcontrol	1632253.667	122467.2049	7.50295174
UIJ69497.1	septation_regulator_SpoVG_[Bacillus_cereus]	CPTF_Al	3090967.667	814923.3672	26.36466812
UIJ69497.1	septation_regulator_SpoVG_[Bacillus_cereus]	CPTF_Cd	4780255.233	121649.7087	2.544837101
UIJ69497.1	septation_regulator_SpoVG_[Bacillus_cereus]	CPTF_Co	2531944	719133.6835	28.40243242
UIJ69497.1	septation_regulator_SpoVG_[Bacillus_cereus]	CPTF_Cu	6016618.333	3081645.446	51.21889533
UIJ69497.1	septation_regulator_SpoVG_[Bacillus_cereus]	CPTF_Fe	4200421.267	1627235.565	38.73981827
UIJ69497.1	septation_regulator_SpoVG_[Bacillus_cereus]	CPTF_Mn	2980790.333	2331757.442	78.22614747
UIJ69497.1	septation_regulator_SpoVG_[Bacillus_cereus]	CPTF_Ni	3795628.667	982084.9815	25.87410592
UIJ69497.1	septation_regulator_SpoVG_[Bacillus_cereus]	CPTF_U	1762568.333	244591.5892	13.87699896
UIJ69497.1	septation_regulator_SpoVG_[Bacillus_cereus]	CPTF_metals_mix	701843	136177.5252	19.40284724
UIJ69497.1	septation_regulator_SpoVG_[Bacillus_cereus]	CPTF_zcontrol	4796506.333	292127.3271	6.090418875
UIJ69499.1	YbaB/EbfC_family_nucleoid-associated_protein_[Bacillus_cereus]	CPTF_Al	1048279.333	148451.3502	14.16143059
UIJ69499.1	YbaB/EbfC_family_nucleoid-associated_protein_[Bacillus_cereus]	CPTF_Cd	1032272.733	182940.7449	17.72213282
UIJ69499.1	YbaB/EbfC_family_nucleoid-associated_protein_[Bacillus_cereus]	CPTF_Co	950625.3333	233713.9172	24.58528181
UIJ69499.1	YbaB/EbfC_family_nucleoid-associated_protein_[Bacillus_cereus]	CPTF_Cu	909712.7	113951.0901	12.52605247
UIJ69499.1	YbaB/EbfC_family_nucleoid-associated_protein_[Bacillus_cereus]	CPTF_Fe	1108453.333	108887.8562	9.823404645
UIJ69499.1	YbaB/EbfC_family_nucleoid-associated_protein_[Bacillus_cereus]	CPTF_Mn	1149277.567	327976.2402	28.53760046
UIJ69499.1	YbaB/EbfC_family_nucleoid-associated_protein_[Bacillus_cereus]	CPTF_Ni	1070847.833	134463.6891	12.55675035
UIJ69499.1	YbaB/EbfC_family_nucleoid-associated_protein_[Bacillus_cereus]	CPTF_U	1097455.367	143933.7318	13.11522419
UIJ69499.1	YbaB/EbfC_family_nucleoid-associated_protein_[Bacillus_cereus]	CPTF_metals_mix	1625653.5	42787.37132	2.632010531
UIJ69499.1	YbaB/EbfC_family_nucleoid-associated_protein_[Bacillus_cereus]	CPTF_zcontrol	999051.3333	22293.23557	2.231440451
UIJ69500.1	D-alanyl-D-alanine_carboxypeptidase_DacA_[Bacillus_cereus]	CPTF_Al	145275.1	70403.30082	48.46205635
UIJ69500.1	D-alanyl-D-alanine_carboxypeptidase_DacA_[Bacillus_cereus]	CPTF_Cd	187800.6333	67625.91702	36.00941904
UIJ69500.1	D-alanyl-D-alanine_carboxypeptidase_DacA_[Bacillus_cereus]	CPTF_Co	197630.4	177829.9149	89.98105295
UIJ69500.1	D-alanyl-D-alanine_carboxypeptidase_DacA_[Bacillus_cereus]	CPTF_Cu	64749.16667	86177.17506	133.0938752
UIJ69500.1	D-alanyl-D-alanine_carboxypeptidase_DacA_[Bacillus_cereus]	CPTF_Fe	266950.2667	73056.41079	27.36704919
UIJ69500.1	D-alanyl-D-alanine_carboxypeptidase_DacA_[Bacillus_cereus]	CPTF_Mn	152321.6667	174310.3067	114.4356614
UIJ69500.1	D-alanyl-D-alanine_carboxypeptidase_DacA_[Bacillus_cereus]	CPTF_Ni	253961.5333	147826.6298	58.20827581
UIJ69500.1	D-alanyl-D-alanine_carboxypeptidase_DacA_[Bacillus_cereus]	CPTF_U	237048.8667	63747.5353	26.89214937
UIJ69500.1	D-alanyl-D-alanine_carboxypeptidase_DacA_[Bacillus_cereus]	CPTF_metals_mix	76450.66667	106199.7434	138.9127761
UIJ69500.1	D-alanyl-D-alanine_carboxypeptidase_DacA_[Bacillus_cereus]	CPTF_zcontrol	321732.7667	27285.86425	8.480909338
UIJ69501.1	GMP_reductase_[Bacillus_cereus]	CPTF_Al	7837316.9	1070229.463	13.65555938
UIJ69501.1	GMP_reductase_[Bacillus_cereus]	CPTF_Cd	6907095.833	294591.9716	4.265062752
UIJ69501.1	GMP_reductase_[Bacillus_cereus]	CPTF_Co	7448408.167	812282.6178	10.90545254
UIJ69501.1	GMP_reductase_[Bacillus_cereus]	CPTF_Cu	4998642.767	359099.4159	7.183938375
UIJ69501.1	GMP_reductase_[Bacillus_cereus]	CPTF_Fe	6440876.7	254735.3958	3.954980163
UIJ69501.1	GMP_reductase_[Bacillus_cereus]	CPTF_Mn	7121593.333	1703634.982	23.92210425
UIJ69501.1	GMP_reductase_[Bacillus_cereus]	CPTF_Ni	5034035.267	536415.7654	10.65578084
UIJ69501.1	GMP_reductase_[Bacillus_cereus]	CPTF_U	5743104.167	1836161.028	31.971578
UIJ69501.1	GMP_reductase_[Bacillus_cereus]	CPTF_metals_mix	3403639.967	306893.6218	9.016629986
UIJ69501.1	GMP_reductase_[Bacillus_cereus]	CPTF_zcontrol	8803326.733	1398576.605	15.88691011
UIJ69502.1	ATP-binding_cassette_domain-containing_protein_[Bacillus_cereus]	CPTF_Al	1050629	42612.13927	4.055869319
UIJ69502.1	ATP-binding_cassette_domain-containing_protein_[Bacillus_cereus]	CPTF_Cd	1066345.667	67228.28647	6.304549132
UIJ69502.1	ATP-binding_cassette_domain-containing_protein_[Bacillus_cereus]	CPTF_Co	1109786	93409.46046	8.416889424
UIJ69502.1	ATP-binding_cassette_domain-containing_protein_[Bacillus_cereus]	CPTF_Cu	1131436	116171.1609	10.2675857
UIJ69502.1	ATP-binding_cassette_domain-containing_protein_[Bacillus_cereus]	CPTF_Fe	1097608	59499.0957	5.420796468
UIJ69502.1	ATP-binding_cassette_domain-containing_protein_[Bacillus_cereus]	CPTF_Mn	984455	227186.595	23.07739765
UIJ69502.1	ATP-binding_cassette_domain-containing_protein_[Bacillus_cereus]	CPTF_Ni	1017295.667	74995.78666	7.372073736
UIJ69502.1	ATP-binding_cassette_domain-containing_protein_[Bacillus_cereus]	CPTF_U	1260874	82651.09165	6.555063523
UIJ69502.1	ATP-binding_cassette_domain-containing_protein_[Bacillus_cereus]	CPTF_metals_mix	1038406.667	210626.4931	20.28362296
UIJ69502.1	ATP-binding_cassette_domain-containing_protein_[Bacillus_cereus]	CPTF_zcontrol	985468	60313.03731	6.120243103
UIJ69506.1	peptide_chain_release_factor_1_[Bacillus_cereus]	CPTF_Al	1455079.067	154463.8557	10.6154957

UIJ69506.1	peptide_chain_release_factor_1_[Bacillus_cereus]	CPTF_Cd	1629989.2	168214.105	10.31995212
UIJ69506.1	peptide_chain_release_factor_1_[Bacillus_cereus]	CPTF_Co	1649472.8	248234.9468	15.04935073
UIJ69506.1	peptide_chain_release_factor_1_[Bacillus_cereus]	CPTF_Cu	1561136.7	133978.2825	8.582098064
UIJ69506.1	peptide_chain_release_factor_1_[Bacillus_cereus]	CPTF_Fe	1358958.867	64388.14176	4.73804935
UIJ69506.1	peptide_chain_release_factor_1_[Bacillus_cereus]	CPTF_Mn	1591719.967	461578.7293	28.99873966
UIJ69506.1	peptide_chain_release_factor_1_[Bacillus_cereus]	CPTF_Ni	1489171.067	95728.87377	6.42833291
UIJ69506.1	peptide_chain_release_factor_1_[Bacillus_cereus]	CPTF_U	1319079.033	272856.1477	20.68535249
UIJ69506.1	peptide_chain_release_factor_1_[Bacillus_cereus]	CPTF_metals_mix	3556402.033	209530.632	5.891646391
UIJ69506.1	peptide_chain_release_factor_1_[Bacillus_cereus]	CPTF_zcontrol	1334282.767	59725.83458	4.476250167
UIJ69522.1	CalY_family_protein_(plasmid)_[Bacillus_cereus]	CPTF_Al	101577.8333	92800.68053	91.35918486
UIJ69522.1	CalY_family_protein_(plasmid)_[Bacillus_cereus]	CPTF_Cd	145300.3333	37084.45376	25.52262125
UIJ69522.1	CalY_family_protein_(plasmid)_[Bacillus_cereus]	CPTF_Co	207909.6	77863.19157	37.45050328
UIJ69522.1	CalY_family_protein_(plasmid)_[Bacillus_cereus]	CPTF_Cu	17552.7	30402.16821	173.2050808
UIJ69522.1	CalY_family_protein_(plasmid)_[Bacillus_cereus]	CPTF_Fe	61348	106257.8529	173.2050808
UIJ69522.1	CalY_family_protein_(plasmid)_[Bacillus_cereus]	CPTF_Mn	0	0	0
UIJ69522.1	CalY_family_protein_(plasmid)_[Bacillus_cereus]	CPTF_Ni	62280.66667	107873.279	173.2050808
UIJ69522.1	CalY_family_protein_(plasmid)_[Bacillus_cereus]	CPTF_U	0	0	0
UIJ69522.1	CalY_family_protein_(plasmid)_[Bacillus_cereus]	CPTF_metals_mix	0	0	0
UIJ69522.1	CalY_family_protein_(plasmid)_[Bacillus_cereus]	CPTF_zcontrol	205429	115791.3134	56.36561214
UIJ69527.1	type_II_toxin-antitoxin_system_death-on-curing_family_toxin_(plasmid)_[Bacillus_cereus]	CPTF_Al	2163640.333	452446.2252	20.91134179
UIJ69527.1	type_II_toxin-antitoxin_system_death-on-curing_family_toxin_(plasmid)_[Bacillus_cereus]	CPTF_Cd	2440164	87094.43582	3.569204194
UIJ69527.1	type_II_toxin-antitoxin_system_death-on-curing_family_toxin_(plasmid)_[Bacillus_cereus]	CPTF_Co	2273672.267	221195.2845	9.728547412
UIJ69527.1	type_II_toxin-antitoxin_system_death-on-curing_family_toxin_(plasmid)_[Bacillus_cereus]	CPTF_Cu	2132080.067	443250.6203	20.78958606
UIJ69527.1	type_II_toxin-antitoxin_system_death-on-curing_family_toxin_(plasmid)_[Bacillus_cereus]	CPTF_Fe	2216644.667	266528.3338	12.02395395
UIJ69527.1	type_II_toxin-antitoxin_system_death-on-curing_family_toxin_(plasmid)_[Bacillus_cereus]	CPTF_Mn	2580612	260154.8406	10.08112962
UIJ69527.1	type_II_toxin-antitoxin_system_death-on-curing_family_toxin_(plasmid)_[Bacillus_cereus]	CPTF_Ni	1719615.333	331478.4939	19.27631648
UIJ69527.1	type_II_toxin-antitoxin_system_death-on-curing_family_toxin_(plasmid)_[Bacillus_cereus]	CPTF_U	2352574	583742.1347	24.81291278
UIJ69527.1	type_II_toxin-antitoxin_system_death-on-curing_family_toxin_(plasmid)_[Bacillus_cereus]	CPTF_metals_mix	3498494.867	528103.0506	15.09515008
UIJ69527.1	type_II_toxin-antitoxin_system_death-on-curing_family_toxin_(plasmid)_[Bacillus_cereus]	CPTF_zcontrol	1725575.333	207016.2498	11.99694072
UIJ69534.1	hypothetical_protein_LW858_30370_(plasmid)_[Bacillus_cereus]	CPTF_Al	108455.8333	52603.59745	48.50232195
UIJ69534.1	hypothetical_protein_LW858_30370_(plasmid)_[Bacillus_cereus]	CPTF_Cd	43939.66667	76105.73513	173.2050808
UIJ69534.1	hypothetical_protein_LW858_30370_(plasmid)_[Bacillus_cereus]	CPTF_Co	112435	98678.25469	87.76471267
UIJ69534.1	hypothetical_protein_LW858_30370_(plasmid)_[Bacillus_cereus]	CPTF_Cu	65465.66667	113389.8608	173.2050808
UIJ69534.1	hypothetical_protein_LW858_30370_(plasmid)_[Bacillus_cereus]	CPTF_Fe	0	0	0
UIJ69534.1	hypothetical_protein_LW858_30370_(plasmid)_[Bacillus_cereus]	CPTF_Mn	48200	83484.84892	173.2050808
UIJ69534.1	hypothetical_protein_LW858_30370_(plasmid)_[Bacillus_cereus]	CPTF_Ni	111533.3333	99171.91525	88.91683973
UIJ69534.1	hypothetical_protein_LW858_30370_(plasmid)_[Bacillus_cereus]	CPTF_U	0	0	0
UIJ69534.1	hypothetical_protein_LW858_30370_(plasmid)_[Bacillus_cereus]	CPTF_metals_mix	363134.2333	34672.28271	9.548062266
UIJ69534.1	hypothetical_protein_LW858_30370_(plasmid)_[Bacillus_cereus]	CPTF_zcontrol	0	0	0
UIJ69541.1	N-acetylmuramoyl-L-alanine_amidase_(plasmid)_[Bacillus_cereus]	CPTF_Al	189036.6	111123.8915	58.78432615
UIJ69541.1	N-acetylmuramoyl-L-alanine_amidase_(plasmid)_[Bacillus_cereus]	CPTF_Cd	290731.5667	117241.2612	40.32629223
UIJ69541.1	N-acetylmuramoyl-L-alanine_amidase_(plasmid)_[Bacillus_cereus]	CPTF_Co	250400.3333	147394.5705	58.86356802
UIJ69541.1	N-acetylmuramoyl-L-alanine_amidase_(plasmid)_[Bacillus_cereus]	CPTF_Cu	508547	20708.81445	4.072153498
UIJ69541.1	N-acetylmuramoyl-L-alanine_amidase_(plasmid)_[Bacillus_cereus]	CPTF_Fe	361679.4	45689.88084	12.63270201
UIJ69541.1	N-acetylmuramoyl-L-alanine_amidase_(plasmid)_[Bacillus_cereus]	CPTF_Mn	59710.1	66730.37586	111.757267
UIJ69541.1	N-acetylmuramoyl-L-alanine_amidase_(plasmid)_[Bacillus_cereus]	CPTF_Ni	123219	15961.92557	12.95411063
UIJ69541.1	N-acetylmuramoyl-L-alanine_amidase_(plasmid)_[Bacillus_cereus]	CPTF_U	144598.3333	13756.51211	9.513603504
UIJ69541.1	N-acetylmuramoyl-L-alanine_amidase_(plasmid)_[Bacillus_cereus]	CPTF_metals_mix	523561	57371.25394	10.95789296
UIJ69541.1	N-acetylmuramoyl-L-alanine_amidase_(plasmid)_[Bacillus_cereus]	CPTF_zcontrol	170100.5	188729.4352	110.9517228
UIJ69542.1	polysaccharide_deacetylase_(plasmid)_[Bacillus_cereus]	CPTF_Al	0	0	0
UIJ69542.1	polysaccharide_deacetylase_(plasmid)_[Bacillus_cereus]	CPTF_Cd	0	0	0
UIJ69542.1	polysaccharide_deacetylase_(plasmid)_[Bacillus_cereus]	CPTF_Co	0	0	0
UIJ69542.1	polysaccharide_deacetylase_(plasmid)_[Bacillus_cereus]	CPTF_Cu	0	0	0
UIJ69542.1	polysaccharide_deacetylase_(plasmid)_[Bacillus_cereus]	CPTF_Fe	0	0	0
UIJ69542.1	polysaccharide_deacetylase_(plasmid)_[Bacillus_cereus]	CPTF_Mn	0	0	0
UIJ69542.1	polysaccharide_deacetylase_(plasmid)_[Bacillus_cereus]	CPTF_Ni	0	0	0
UIJ69542.1	polysaccharide_deacetylase_(plasmid)_[Bacillus_cereus]	CPTF_U	0	0	0
UIJ69542.1	polysaccharide_deacetylase_(plasmid)_[Bacillus_cereus]	CPTF_metals_mix	84777.56667	129920.9285	153.2491833
UIJ69542.1	polysaccharide_deacetylase_(plasmid)_[Bacillus_cereus]	CPTF_zcontrol	0	0	0
UIJ69547.1	YkvS_family_protein_(plasmid)_[Bacillus_cereus]	CPTF_Al	0	0	0
UIJ69547.1	YkvS_family_protein_(plasmid)_[Bacillus_cereus]	CPTF_Cd	0	0	0

UIJ69547.1	YkvS_family_protein_(plasmid)_[Bacillus_cereus]	CPTF_Co	0	0	0
UIJ69547.1	YkvS_family_protein_(plasmid)_[Bacillus_cereus]	CPTF_Cu	0	0	0
UIJ69547.1	YkvS_family_protein_(plasmid)_[Bacillus_cereus]	CPTF_Fe	0	0	0
UIJ69547.1	YkvS_family_protein_(plasmid)_[Bacillus_cereus]	CPTF_Mn	0	0	0
UIJ69547.1	YkvS_family_protein_(plasmid)_[Bacillus_cereus]	CPTF_Ni	0	0	0
UIJ69547.1	YkvS_family_protein_(plasmid)_[Bacillus_cereus]	CPTF_U	0	0	0
UIJ69547.1	YkvS_family_protein_(plasmid)_[Bacillus_cereus]	CPTF_metals_mix	104194.3333	180469.8792	173.2050808
UIJ69547.1	YkvS_family_protein_(plasmid)_[Bacillus_cereus]	CPTF_zcontrol	0	0	0
UIJ69570.1	metalloregulator_ArsR/SmtB_family_transcription_factor_(plasmid)_[Bacillus_cereus]	CPTF_Al	335248	40363.07755	12.03976684
UIJ69570.1	metalloregulator_ArsR/SmtB_family_transcription_factor_(plasmid)_[Bacillus_cereus]	CPTF_Cd	344331	44928.14624	13.04795277
UIJ69570.1	metalloregulator_ArsR/SmtB_family_transcription_factor_(plasmid)_[Bacillus_cereus]	CPTF_Co	302149.6667	35273.68199	11.67424157
UIJ69570.1	metalloregulator_ArsR/SmtB_family_transcription_factor_(plasmid)_[Bacillus_cereus]	CPTF_Cu	304587.6667	13952.08047	4.580645244
UIJ69570.1	metalloregulator_ArsR/SmtB_family_transcription_factor_(plasmid)_[Bacillus_cereus]	CPTF_Fe	319926.3333	33418.13571	10.44557207
UIJ69570.1	metalloregulator_ArsR/SmtB_family_transcription_factor_(plasmid)_[Bacillus_cereus]	CPTF_Mn	365850	11128.16512	3.041728884
UIJ69570.1	metalloregulator_ArsR/SmtB_family_transcription_factor_(plasmid)_[Bacillus_cereus]	CPTF_Ni	284671	19110.98572	6.71335883
UIJ69570.1	metalloregulator_ArsR/SmtB_family_transcription_factor_(plasmid)_[Bacillus_cereus]	CPTF_U	273851.3333	56770.0056	20.73022794
UIJ69570.1	metalloregulator_ArsR/SmtB_family_transcription_factor_(plasmid)_[Bacillus_cereus]	CPTF_metals_mix	282345.6667	45880.43602	16.24973975
UIJ69570.1	metalloregulator_ArsR/SmtB_family_transcription_factor_(plasmid)_[Bacillus_cereus]	CPTF_zcontrol	301865	29033.16006	9.617928564
UIJ69571.1	HU_family_DNA-binding_protein_(plasmid)_[Bacillus_cereus]	CPTF_Al	124079.6667	214912.2869	173.2050808
UIJ69571.1	HU_family_DNA-binding_protein_(plasmid)_[Bacillus_cereus]	CPTF_Cd	174296.2667	230908.9611	132.4807269
UIJ69571.1	HU_family_DNA-binding_protein_(plasmid)_[Bacillus_cereus]	CPTF_Co	32929.73333	30219.32353	91.76911099
UIJ69571.1	HU_family_DNA-binding_protein_(plasmid)_[Bacillus_cereus]	CPTF_Cu	54375.7	52521.80097	96.59057441
UIJ69571.1	HU_family_DNA-binding_protein_(plasmid)_[Bacillus_cereus]	CPTF_Fe	47157.36667	16242.33139	34.44282949
UIJ69571.1	HU_family_DNA-binding_protein_(plasmid)_[Bacillus_cereus]	CPTF_Mn	227055.3333	393271.3735	173.2050808
UIJ69571.1	HU_family_DNA-binding_protein_(plasmid)_[Bacillus_cereus]	CPTF_Ni	30354.43333	52575.42077	173.2050808
UIJ69571.1	HU_family_DNA-binding_protein_(plasmid)_[Bacillus_cereus]	CPTF_U	21905.63333	37941.66991	173.2050808
UIJ69571.1	HU_family_DNA-binding_protein_(plasmid)_[Bacillus_cereus]	CPTF_metals_mix	3619400.933	124211.5687	3.431826731
UIJ69571.1	HU_family_DNA-binding_protein_(plasmid)_[Bacillus_cereus]	CPTF_zcontrol	129908	225007.2563	173.2050808
UIJ69575.1	CalY_family_protein_(plasmid)_[Bacillus_cereus]	CPTF_Al	6040362.133	764380.5785	12.65454888
UIJ69575.1	CalY_family_protein_(plasmid)_[Bacillus_cereus]	CPTF_Cd	6643432.867	229307.7916	3.451646103
UIJ69575.1	CalY_family_protein_(plasmid)_[Bacillus_cereus]	CPTF_Co	6347293.767	772721.7005	12.17403399
UIJ69575.1	CalY_family_protein_(plasmid)_[Bacillus_cereus]	CPTF_Cu	1365406.033	81360.97812	5.958738729
UIJ69575.1	CalY_family_protein_(plasmid)_[Bacillus_cereus]	CPTF_Fe	4466107.867	1998277.106	44.74314472
UIJ69575.1	CalY_family_protein_(plasmid)_[Bacillus_cereus]	CPTF_Mn	5789519.7	355711.9021	6.144065839
UIJ69575.1	CalY_family_protein_(plasmid)_[Bacillus_cereus]	CPTF_Ni	3824641.933	1146540.723	29.97772715
UIJ69575.1	CalY_family_protein_(plasmid)_[Bacillus_cereus]	CPTF_U	4813761.367	2246650.349	46.67141094
UIJ69575.1	CalY_family_protein_(plasmid)_[Bacillus_cereus]	CPTF_metals_mix	447628.3333	110456.4997	24.67594016
UIJ69575.1	CalY_family_protein_(plasmid)_[Bacillus_cereus]	CPTF_zcontrol	6738669.067	214864.3459	3.188527939
UIJ69581.1	MerR_family_transcriptional_regulator_(plasmid)_[Bacillus_cereus]	CPTF_Al	100072.5333	7172.162816	7.166964377
UIJ69581.1	MerR_family_transcriptional_regulator_(plasmid)_[Bacillus_cereus]	CPTF_Cd	84575.03333	2253.955173	2.665036104
UIJ69581.1	MerR_family_transcriptional_regulator_(plasmid)_[Bacillus_cereus]	CPTF_Co	106491.7667	12440.52238	11.6821448
UIJ69581.1	MerR_family_transcriptional_regulator_(plasmid)_[Bacillus_cereus]	CPTF_Cu	109139.3	11101.44746	10.17181479
UIJ69581.1	MerR_family_transcriptional_regulator_(plasmid)_[Bacillus_cereus]	CPTF_Fe	64388.66667	12685.3907	19.70127874
UIJ69581.1	MerR_family_transcriptional_regulator_(plasmid)_[Bacillus_cereus]	CPTF_Mn	57297.03333	49732.63597	86.79792491
UIJ69581.1	MerR_family_transcriptional_regulator_(plasmid)_[Bacillus_cereus]	CPTF_Ni	110273.3333	4985.834166	4.521341666
UIJ69581.1	MerR_family_transcriptional_regulator_(plasmid)_[Bacillus_cereus]	CPTF_U	60227.93333	52269.17611	86.78560465
UIJ69581.1	MerR_family_transcriptional_regulator_(plasmid)_[Bacillus_cereus]	CPTF_metals_mix	99749.46667	5171.145546	5.184133528
UIJ69581.1	MerR_family_transcriptional_regulator_(plasmid)_[Bacillus_cereus]	CPTF_zcontrol	83848.36667	5339.320033	6.367828313
UIJ69582.1	cell_division_protein_FtsZ_(plasmid)_[Bacillus_cereus]	CPTF_Al	1033837.033	283493.5063	27.4214888
UIJ69582.1	cell_division_protein_FtsZ_(plasmid)_[Bacillus_cereus]	CPTF_Cd	1182933.933	201555.4996	17.03861002
UIJ69582.1	cell_division_protein_FtsZ_(plasmid)_[Bacillus_cereus]	CPTF_Co	987613.6333	117437.9024	11.89107748
UIJ69582.1	cell_division_protein_FtsZ_(plasmid)_[Bacillus_cereus]	CPTF_Cu	795407.6	106105.5513	13.33977087
UIJ69582.1	cell_division_protein_FtsZ_(plasmid)_[Bacillus_cereus]	CPTF_Fe	1189224.7	62790.58529	5.279959732
UIJ69582.1	cell_division_protein_FtsZ_(plasmid)_[Bacillus_cereus]	CPTF_Mn	1211796.6	335255.6618	27.6660012
UIJ69582.1	cell_division_protein_FtsZ_(plasmid)_[Bacillus_cereus]	CPTF_Ni	1197878.733	351342.8978	29.33042286
UIJ69582.1	cell_division_protein_FtsZ_(plasmid)_[Bacillus_cereus]	CPTF_U	957421.0667	546308.1496	57.0603853
UIJ69582.1	cell_division_protein_FtsZ_(plasmid)_[Bacillus_cereus]	CPTF_metals_mix	916315	304006.1873	33.17703926
UIJ69582.1	cell_division_protein_FtsZ_(plasmid)_[Bacillus_cereus]	CPTF_zcontrol	1410597.533	249285.9634	17.67236632
UIJ69620.1	urease_subunit_alpha_(plasmid)_[Bacillus_cereus]	CPTF_Al	0	0	0
UIJ69620.1	urease_subunit_alpha_(plasmid)_[Bacillus_cereus]	CPTF_Cd	0	0	0
UIJ69620.1	urease_subunit_alpha_(plasmid)_[Bacillus_cereus]	CPTF_Co	19857.7	34394.54532	173.2050808

UIJ69620.1	urease_subunit_alpha_(plasmid)_[Bacillus_cereus]	CPTF_Cu	63879.3	58115.16425	90.97652017
UIJ69620.1	urease_subunit_alpha_(plasmid)_[Bacillus_cereus]	CPTF_Fe	0	0	0
UIJ69620.1	urease_subunit_alpha_(plasmid)_[Bacillus_cereus]	CPTF_Mn	0	0	0
UIJ69620.1	urease_subunit_alpha_(plasmid)_[Bacillus_cereus]	CPTF_Ni	0	0	0
UIJ69620.1	urease_subunit_alpha_(plasmid)_[Bacillus_cereus]	CPTF_U	0	0	0
UIJ69620.1	urease_subunit_alpha_(plasmid)_[Bacillus_cereus]	CPTF_metals_mix	0	0	0
UIJ69620.1	urease_subunit_alpha_(plasmid)_[Bacillus_cereus]	CPTF_zcontrol	0	0	0
UIJ69621.1	urease_accessory_protein_UreE_(plasmid)_[Bacillus_cereus]	CPTF_Al	101090.8	32306.89771	31.95829661
UIJ69621.1	urease_accessory_protein_UreE_(plasmid)_[Bacillus_cereus]	CPTF_Cd	85133	11819.62126	13.88371285
UIJ69621.1	urease_accessory_protein_UreE_(plasmid)_[Bacillus_cereus]	CPTF_Co	88020.4	28242.22912	32.08600406
UIJ69621.1	urease_accessory_protein_UreE_(plasmid)_[Bacillus_cereus]	CPTF_Cu	74959.33333	68332.0798	91.15886809
UIJ69621.1	urease_accessory_protein_UreE_(plasmid)_[Bacillus_cereus]	CPTF_Fe	64443.1	20152.13203	31.27120208
UIJ69621.1	urease_accessory_protein_UreE_(plasmid)_[Bacillus_cereus]	CPTF_Mn	14195.23333	24586.86536	173.2050808
UIJ69621.1	urease_accessory_protein_UreE_(plasmid)_[Bacillus_cereus]	CPTF_Ni	52478.2	45969.52357	87.59737104
UIJ69621.1	urease_accessory_protein_UreE_(plasmid)_[Bacillus_cereus]	CPTF_U	79147.83333	11006.98584	13.90686944
UIJ69621.1	urease_accessory_protein_UreE_(plasmid)_[Bacillus_cereus]	CPTF_metals_mix	22477.13333	38931.53694	173.2050808
UIJ69621.1	urease_accessory_protein_UreE_(plasmid)_[Bacillus_cereus]	CPTF_zcontrol	45876.16667	44501.40752	97.00332603
UIJ69623.1	urease_accessory_protein_UreG_(plasmid)_[Bacillus_cereus]	CPTF_Al	311924.5	61922.47306	19.85175036
UIJ69623.1	urease_accessory_protein_UreG_(plasmid)_[Bacillus_cereus]	CPTF_Cd	389053	22322.98898	5.737775825
UIJ69623.1	urease_accessory_protein_UreG_(plasmid)_[Bacillus_cereus]	CPTF_Co	319414.8	27241.08672	8.528435978
UIJ69623.1	urease_accessory_protein_UreG_(plasmid)_[Bacillus_cereus]	CPTF_Cu	267015.2333	53350.08533	19.98016542
UIJ69623.1	urease_accessory_protein_UreG_(plasmid)_[Bacillus_cereus]	CPTF_Fe	286758.3333	40288.6772	14.04969708
UIJ69623.1	urease_accessory_protein_UreG_(plasmid)_[Bacillus_cereus]	CPTF_Mn	295866.3333	31414.36761	10.61775676
UIJ69623.1	urease_accessory_protein_UreG_(plasmid)_[Bacillus_cereus]	CPTF_Ni	215506.9	36439.82016	16.90888791
UIJ69623.1	urease_accessory_protein_UreG_(plasmid)_[Bacillus_cereus]	CPTF_U	271427.0333	112723.1992	41.52983503
UIJ69623.1	urease_accessory_protein_UreG_(plasmid)_[Bacillus_cereus]	CPTF_metals_mix	333344.7	42427.45352	12.72780204
UIJ69623.1	urease_accessory_protein_UreG_(plasmid)_[Bacillus_cereus]	CPTF_zcontrol	258688.5	92711.76949	35.83915384
UIJ69677.1	peptidylprolyl_isomerase_(plasmid)_[Bacillus_cereus]	CPTF_Al	1052870.667	346776.9746	32.93633165
UIJ69677.1	peptidylprolyl_isomerase_(plasmid)_[Bacillus_cereus]	CPTF_Cd	1171131.367	345608.4344	29.51064622
UIJ69677.1	peptidylprolyl_isomerase_(plasmid)_[Bacillus_cereus]	CPTF_Co	1149227.467	362629.3475	31.55418383
UIJ69677.1	peptidylprolyl_isomerase_(plasmid)_[Bacillus_cereus]	CPTF_Cu	1157026.3	54889.5982	4.744023382
UIJ69677.1	peptidylprolyl_isomerase_(plasmid)_[Bacillus_cereus]	CPTF_Fe	1276836.4	231757.535	18.15091855
UIJ69677.1	peptidylprolyl_isomerase_(plasmid)_[Bacillus_cereus]	CPTF_Mn	825127.9667	514028.7121	62.29684762
UIJ69677.1	peptidylprolyl_isomerase_(plasmid)_[Bacillus_cereus]	CPTF_Ni	1149822.333	222774.6805	19.3747046
UIJ69677.1	peptidylprolyl_isomerase_(plasmid)_[Bacillus_cereus]	CPTF_U	715347	67991.44794	9.504680657
UIJ69677.1	peptidylprolyl_isomerase_(plasmid)_[Bacillus_cereus]	CPTF_metals_mix	2163717.1	230367.0904	10.64682118
UIJ69677.1	peptidylprolyl_isomerase_(plasmid)_[Bacillus_cereus]	CPTF_zcontrol	1312134	582596.2028	44.40066356
UIJ69708.1	hypothetical_protein_LW858_29515_(plasmid)_[Bacillus_cereus]	CPTF_Al	0	0	0
UIJ69708.1	hypothetical_protein_LW858_29515_(plasmid)_[Bacillus_cereus]	CPTF_Cd	0	0	0
UIJ69708.1	hypothetical_protein_LW858_29515_(plasmid)_[Bacillus_cereus]	CPTF_Co	11467	19861.42661	173.2050808
UIJ69708.1	hypothetical_protein_LW858_29515_(plasmid)_[Bacillus_cereus]	CPTF_Cu	77609	134422.7311	173.2050808
UIJ69708.1	hypothetical_protein_LW858_29515_(plasmid)_[Bacillus_cereus]	CPTF_Fe	0	0	0
UIJ69708.1	hypothetical_protein_LW858_29515_(plasmid)_[Bacillus_cereus]	CPTF_Mn	0	0	0
UIJ69708.1	hypothetical_protein_LW858_29515_(plasmid)_[Bacillus_cereus]	CPTF_Ni	0	0	0
UIJ69708.1	hypothetical_protein_LW858_29515_(plasmid)_[Bacillus_cereus]	CPTF_U	0	0	0
UIJ69708.1	hypothetical_protein_LW858_29515_(plasmid)_[Bacillus_cereus]	CPTF_metals_mix	67300.86667	59394.1931	88.25175075
UIJ69708.1	hypothetical_protein_LW858_29515_(plasmid)_[Bacillus_cereus]	CPTF_zcontrol	0	0	0
UIJ69728.1	hypothetical_protein_LW858_29720_(plasmid)_[Bacillus_cereus]	CPTF_Al	94618.66667	82683.59792	87.38613725
UIJ69728.1	hypothetical_protein_LW858_29720_(plasmid)_[Bacillus_cereus]	CPTF_Cd	48891.66667	84682.85073	173.2050808
UIJ69728.1	hypothetical_protein_LW858_29720_(plasmid)_[Bacillus_cereus]	CPTF_Co	119009	106170.9711	89.21255627
UIJ69728.1	hypothetical_protein_LW858_29720_(plasmid)_[Bacillus_cereus]	CPTF_Cu	108630	94771.41196	87.2423934
UIJ69728.1	hypothetical_protein_LW858_29720_(plasmid)_[Bacillus_cereus]	CPTF_Fe	125616.2	71023.76584	56.54029165
UIJ69728.1	hypothetical_protein_LW858_29720_(plasmid)_[Bacillus_cereus]	CPTF_Mn	78235.6	90090.54452	115.1528774
UIJ69728.1	hypothetical_protein_LW858_29720_(plasmid)_[Bacillus_cereus]	CPTF_Ni	95270	85018.01482	89.23902049
UIJ69728.1	hypothetical_protein_LW858_29720_(plasmid)_[Bacillus_cereus]	CPTF_U	79627.33333	69427.44818	87.19047251
UIJ69728.1	hypothetical_protein_LW858_29720_(plasmid)_[Bacillus_cereus]	CPTF_metals_mix	281547.3333	45916.0992	16.30848307
UIJ69728.1	hypothetical_protein_LW858_29720_(plasmid)_[Bacillus_cereus]	CPTF_zcontrol	56049.56667	53879.5629	96.12842008
UIJ69785.1	hypothetical_protein_LW858_30150_(plasmid)_[Bacillus_cereus]	CPTF_Al	0	0	0
UIJ69785.1	hypothetical_protein_LW858_30150_(plasmid)_[Bacillus_cereus]	CPTF_Cd	0	0	0
UIJ69785.1	hypothetical_protein_LW858_30150_(plasmid)_[Bacillus_cereus]	CPTF_Co	0	0	0
UIJ69785.1	hypothetical_protein_LW858_30150_(plasmid)_[Bacillus_cereus]	CPTF_Cu	0	0	0

UIJ69785.1	hypothetical_protein_LW858_30150_(plasmid)_[Bacillus_cereus]	CPTF_Fe	0	0	0
UIJ69785.1	hypothetical_protein_LW858_30150_(plasmid)_[Bacillus_cereus]	CPTF_Mn	9589.7	16609.84763	173.2050808
UIJ69785.1	hypothetical_protein_LW858_30150_(plasmid)_[Bacillus_cereus]	CPTF_Ni	10854.33333	18800.25682	173.2050808
UIJ69785.1	hypothetical_protein_LW858_30150_(plasmid)_[Bacillus_cereus]	CPTF_U	14270.26667	24716.8269	173.2050808
UIJ69785.1	hypothetical_protein_LW858_30150_(plasmid)_[Bacillus_cereus]	CPTF_metals_mix	67021.46667	37906.91008	56.55935623
UIJ69785.1	hypothetical_protein_LW858_30150_(plasmid)_[Bacillus_cereus]	CPTF_zcontrol	9686.8	16778.02976	173.2050808
UIJ69803.1	metalloregulator_ArsR/SmtB_family_transcription_factor_(plasmid)_[Bacillus_cereus]	CPTF_Al	84393	73312.20287	86.87000446
UIJ69803.1	metalloregulator_ArsR/SmtB_family_transcription_factor_(plasmid)_[Bacillus_cereus]	CPTF_Cd	124667.6667	17028.222	13.65889204
UIJ69803.1	metalloregulator_ArsR/SmtB_family_transcription_factor_(plasmid)_[Bacillus_cereus]	CPTF_Co	56360.43333	65514.07806	116.2412604
UIJ69803.1	metalloregulator_ArsR/SmtB_family_transcription_factor_(plasmid)_[Bacillus_cereus]	CPTF_Cu	0	0	0
UIJ69803.1	metalloregulator_ArsR/SmtB_family_transcription_factor_(plasmid)_[Bacillus_cereus]	CPTF_Fe	45251.66667	78378.18579	173.2050808
UIJ69803.1	metalloregulator_ArsR/SmtB_family_transcription_factor_(plasmid)_[Bacillus_cereus]	CPTF_Mn	68005.33333	117788.6925	173.2050808
UIJ69803.1	metalloregulator_ArsR/SmtB_family_transcription_factor_(plasmid)_[Bacillus_cereus]	CPTF_Ni	0	0	0
UIJ69803.1	metalloregulator_ArsR/SmtB_family_transcription_factor_(plasmid)_[Bacillus_cereus]	CPTF_U	0	0	0
UIJ69803.1	metalloregulator_ArsR/SmtB_family_transcription_factor_(plasmid)_[Bacillus_cereus]	CPTF_metals_mix	134616.6667	23967.67136	17.8043863
UIJ69803.1	metalloregulator_ArsR/SmtB_family_transcription_factor_(plasmid)_[Bacillus_cereus]	CPTF_zcontrol	34911.66667	60468.78044	173.2050808
UIJ69838.1	Rpn_family_recombination-promoting_nuclease/putative_transposase_(plasmid)_[Bacillus_cereus]	CPTF_Al	1483333.333	180092.5688	12.14107205
UIJ69838.1	Rpn_family_recombination-promoting_nuclease/putative_transposase_(plasmid)_[Bacillus_cereus]	CPTF_Cd	1890176.333	235613.8104	12.4651762
UIJ69838.1	Rpn_family_recombination-promoting_nuclease/putative_transposase_(plasmid)_[Bacillus_cereus]	CPTF_Co	1654486.333	300993.4394	18.19256124
UIJ69838.1	Rpn_family_recombination-promoting_nuclease/putative_transposase_(plasmid)_[Bacillus_cereus]	CPTF_Cu	1597687.333	146544.687	9.172300732
UIJ69838.1	Rpn_family_recombination-promoting_nuclease/putative_transposase_(plasmid)_[Bacillus_cereus]	CPTF_Fe	1702747	140945.1479	8.277515562
UIJ69838.1	Rpn_family_recombination-promoting_nuclease/putative_transposase_(plasmid)_[Bacillus_cereus]	CPTF_Mn	1684030.667	192365.2225	11.42290496
UIJ69838.1	Rpn_family_recombination-promoting_nuclease/putative_transposase_(plasmid)_[Bacillus_cereus]	CPTF_Ni	1616666.667	63508.52961	3.928362656
UIJ69838.1	Rpn_family_recombination-promoting_nuclease/putative_transposase_(plasmid)_[Bacillus_cereus]	CPTF_U	1722349.333	15686.87672	0.910783685
UIJ69838.1	Rpn_family_recombination-promoting_nuclease/putative_transposase_(plasmid)_[Bacillus_cereus]	CPTF_metals_mix	2395107.333	155157.5249	6.478103205
UIJ69838.1	Rpn_family_recombination-promoting_nuclease/putative_transposase_(plasmid)_[Bacillus_cereus]	CPTF_zcontrol	1616666.667	55075.70547	3.406744668
UIJ69840.1	hypothetical_protein_LW858_31855_(plasmid)_[Bacillus_cereus]	CPTF_Al	41774.7	39039.93027	93.45352635
UIJ69840.1	hypothetical_protein_LW858_31855_(plasmid)_[Bacillus_cereus]	CPTF_Cd	0	0	0
UIJ69840.1	hypothetical_protein_LW858_31855_(plasmid)_[Bacillus_cereus]	CPTF_Co	25005.36667	22547.2057	90.16946641
UIJ69840.1	hypothetical_protein_LW858_31855_(plasmid)_[Bacillus_cereus]	CPTF_Cu	19134.3	33141.57977	173.2050808
UIJ69840.1	hypothetical_protein_LW858_31855_(plasmid)_[Bacillus_cereus]	CPTF_Fe	0	0	0
UIJ69840.1	hypothetical_protein_LW858_31855_(plasmid)_[Bacillus_cereus]	CPTF_Mn	0	0	0
UIJ69840.1	hypothetical_protein_LW858_31855_(plasmid)_[Bacillus_cereus]	CPTF_Ni	0	0	0
UIJ69840.1	hypothetical_protein_LW858_31855_(plasmid)_[Bacillus_cereus]	CPTF_U	0	0	0
UIJ69840.1	hypothetical_protein_LW858_31855_(plasmid)_[Bacillus_cereus]	CPTF_metals_mix	44885	21766.28431	48.4934484
UIJ69840.1	hypothetical_protein_LW858_31855_(plasmid)_[Bacillus_cereus]	CPTF_zcontrol	0	0	0
UIJ69842.1	SWIM_zinc_finger_family_protein_(plasmid)_[Bacillus_cereus]	CPTF_Al	208329	33538.01132	16.09858028
UIJ69842.1	SWIM_zinc_finger_family_protein_(plasmid)_[Bacillus_cereus]	CPTF_Cd	101472.2	73304.25756	72.24072954
UIJ69842.1	SWIM_zinc_finger_family_protein_(plasmid)_[Bacillus_cereus]	CPTF_Co	178832.3333	36438.8315	20.37597498
UIJ69842.1	SWIM_zinc_finger_family_protein_(plasmid)_[Bacillus_cereus]	CPTF_Cu	128347.9	83223.96987	64.84248661
UIJ69842.1	SWIM_zinc_finger_family_protein_(plasmid)_[Bacillus_cereus]	CPTF_Fe	224294	5423.471859	2.418019144
UIJ69842.1	SWIM_zinc_finger_family_protein_(plasmid)_[Bacillus_cereus]	CPTF_Mn	238128.1333	159878.0291	67.13949622
UIJ69842.1	SWIM_zinc_finger_family_protein_(plasmid)_[Bacillus_cereus]	CPTF_Ni	242898	72068.69898	29.67035504
UIJ69842.1	SWIM_zinc_finger_family_protein_(plasmid)_[Bacillus_cereus]	CPTF_U	201482.3333	176704.5752	87.70226764
UIJ69842.1	SWIM_zinc_finger_family_protein_(plasmid)_[Bacillus_cereus]	CPTF_metals_mix	47570	46621.19143	98.00544761
UIJ69842.1	SWIM_zinc_finger_family_protein_(plasmid)_[Bacillus_cereus]	CPTF_zcontrol	248136.3333	92706.58471	37.3611488
UIJ69851.1	Rpn_family_recombination-promoting_nuclease/putative_transposase_(plasmid)_[Bacillus_cereus]	CPTF_Al	423054.6333	213669.8249	50.50643772
UIJ69851.1	Rpn_family_recombination-promoting_nuclease/putative_transposase_(plasmid)_[Bacillus_cereus]	CPTF_Cd	205492.0667	111413.7094	54.21801005
UIJ69851.1	Rpn_family_recombination-promoting_nuclease/putative_transposase_(plasmid)_[Bacillus_cereus]	CPTF_Co	239962.9333	63660.92762	26.52948384
UIJ69851.1	Rpn_family_recombination-promoting_nuclease/putative_transposase_(plasmid)_[Bacillus_cereus]	CPTF_Cu	267109.6	4339.412665	1.62458132
UIJ69851.1	Rpn_family_recombination-promoting_nuclease/putative_transposase_(plasmid)_[Bacillus_cereus]	CPTF_Fe	401439.6333	135176.8247	33.67301418
UIJ69851.1	Rpn_family_recombination-promoting_nuclease/putative_transposase_(plasmid)_[Bacillus_cereus]	CPTF_Mn	697489.8	182027.9477	26.09757845
UIJ69851.1	Rpn_family_recombination-promoting_nuclease/putative_transposase_(plasmid)_[Bacillus_cereus]	CPTF_Ni	216427.4667	149164.3546	68.92117572
UIJ69851.1	Rpn_family_recombination-promoting_nuclease/putative_transposase_(plasmid)_[Bacillus_cereus]	CPTF_U	407601.1667	320920.8978	78.73404789
UIJ69851.1	Rpn_family_recombination-promoting_nuclease/putative_transposase_(plasmid)_[Bacillus_cereus]	CPTF_metals_mix	1260977.233	76929.16821	6.100757902
UIJ69851.1	Rpn_family_recombination-promoting_nuclease/putative_transposase_(plasmid)_[Bacillus_cereus]	CPTF_zcontrol	575503.1667	244035.2482	42.40380632
UIJ69857.1	Rpn_family_recombination-promoting_nuclease/putative_transposase_(plasmid)_[Bacillus_cereus]	CPTF_Al	911610.2667	283999.6377	31.15362431
UIJ69857.1	Rpn_family_recombination-promoting_nuclease/putative_transposase_(plasmid)_[Bacillus_cereus]	CPTF_Cd	964610.0667	130878.7443	13.5680467
UIJ69857.1	Rpn_family_recombination-promoting_nuclease/putative_transposase_(plasmid)_[Bacillus_cereus]	CPTF_Co	981751.8333	94664.89307	9.642446273
UIJ69857.1	Rpn_family_recombination-promoting_nuclease/putative_transposase_(plasmid)_[Bacillus_cereus]	CPTF_Cu	930537.4	104060.1286	11.18279916
UIJ69857.1	Rpn_family_recombination-promoting_nuclease/putative_transposase_(plasmid)_[Bacillus_cereus]	CPTF_Fe	920344.7667	101349.7338	11.01214865

UIJ69857.1	Rpn_family_recombination-promoting_nuclease/putative_transposase_(plasmid)_[Bacillus_cereus]	CPTF_Mn	1010494.2	76179.90287	7.538875816
UIJ69857.1	Rpn_family_recombination-promoting_nuclease/putative_transposase_(plasmid)_[Bacillus_cereus]	CPTF_Ni	1000324.767	69530.00926	6.950743556
UIJ69857.1	Rpn_family_recombination-promoting_nuclease/putative_transposase_(plasmid)_[Bacillus_cereus]	CPTF_U	882351.3333	273978.6395	31.05096905
UIJ69857.1	Rpn_family_recombination-promoting_nuclease/putative_transposase_(plasmid)_[Bacillus_cereus]	CPTF_metals_mix	1049192.667	182081.6209	17.3544504
UIJ69857.1	Rpn_family_recombination-promoting_nuclease/putative_transposase_(plasmid)_[Bacillus_cereus]	CPTF_zcontrol	994652.7667	25837.38217	2.597628342
UIJ69862.1	hypothetical_protein_LW858_31475_(plasmid)_[Bacillus_cereus]	CPTF_Al	30609.8	28167.15066	92.02004147
UIJ69862.1	hypothetical_protein_LW858_31475_(plasmid)_[Bacillus_cereus]	CPTF_Cd	0	0	0
UIJ69862.1	hypothetical_protein_LW858_31475_(plasmid)_[Bacillus_cereus]	CPTF_Co	39202.3	41990.14364	107.1114288
UIJ69862.1	hypothetical_protein_LW858_31475_(plasmid)_[Bacillus_cereus]	CPTF_Cu	0	0	0
UIJ69862.1	hypothetical_protein_LW858_31475_(plasmid)_[Bacillus_cereus]	CPTF_Fe	0	0	0
UIJ69862.1	hypothetical_protein_LW858_31475_(plasmid)_[Bacillus_cereus]	CPTF_Mn	0	0	0
UIJ69862.1	hypothetical_protein_LW858_31475_(plasmid)_[Bacillus_cereus]	CPTF_Ni	0	0	0
UIJ69862.1	hypothetical_protein_LW858_31475_(plasmid)_[Bacillus_cereus]	CPTF_U	0	0	0
UIJ69862.1	hypothetical_protein_LW858_31475_(plasmid)_[Bacillus_cereus]	CPTF_metals_mix	0	0	0
UIJ69862.1	hypothetical_protein_LW858_31475_(plasmid)_[Bacillus_cereus]	CPTF_zcontrol	0	0	0
UIJ69878.1	ParM/StbA_family_protein_(plasmid)_[Bacillus_cereus]	CPTF_Al	732206.3333	330376.7672	45.12071969
UIJ69878.1	ParM/StbA_family_protein_(plasmid)_[Bacillus_cereus]	CPTF_Cd	628258.4667	76775.6323	12.2203896
UIJ69878.1	ParM/StbA_family_protein_(plasmid)_[Bacillus_cereus]	CPTF_Co	816253.0333	297379.5887	36.43227976
UIJ69878.1	ParM/StbA_family_protein_(plasmid)_[Bacillus_cereus]	CPTF_Cu	776913.6667	286008.8743	36.81346932
UIJ69878.1	ParM/StbA_family_protein_(plasmid)_[Bacillus_cereus]	CPTF_Fe	615298	251689.1083	40.90523751
UIJ69878.1	ParM/StbA_family_protein_(plasmid)_[Bacillus_cereus]	CPTF_Mn	797386.3333	392564.875	49.23145264
UIJ69878.1	ParM/StbA_family_protein_(plasmid)_[Bacillus_cereus]	CPTF_Ni	231032.1333	200185.7002	86.64842302
UIJ69878.1	ParM/StbA_family_protein_(plasmid)_[Bacillus_cereus]	CPTF_U	275261.6333	154363.6728	56.07889152
UIJ69878.1	ParM/StbA_family_protein_(plasmid)_[Bacillus_cereus]	CPTF_metals_mix	946431	60227.63343	6.363658146
UIJ69878.1	ParM/StbA_family_protein_(plasmid)_[Bacillus_cereus]	CPTF_zcontrol	453573	183688.9531	40.49821155
UIJ69880.1	helix-turn-helix_domain-containing_protein_(plasmid)_[Bacillus_cereus]	CPTF_Al	0	0	0
UIJ69880.1	helix-turn-helix_domain-containing_protein_(plasmid)_[Bacillus_cereus]	CPTF_Cd	0	0	0
UIJ69880.1	helix-turn-helix_domain-containing_protein_(plasmid)_[Bacillus_cereus]	CPTF_Co	0	0	0
UIJ69880.1	helix-turn-helix_domain-containing_protein_(plasmid)_[Bacillus_cereus]	CPTF_Cu	14402.43333	24945.74629	173.2050808
UIJ69880.1	helix-turn-helix_domain-containing_protein_(plasmid)_[Bacillus_cereus]	CPTF_Fe	0	0	0
UIJ69880.1	helix-turn-helix_domain-containing_protein_(plasmid)_[Bacillus_cereus]	CPTF_Mn	0	0	0
UIJ69880.1	helix-turn-helix_domain-containing_protein_(plasmid)_[Bacillus_cereus]	CPTF_Ni	12395.23333	21469.17391	173.2050808
UIJ69880.1	helix-turn-helix_domain-containing_protein_(plasmid)_[Bacillus_cereus]	CPTF_U	0	0	0
UIJ69880.1	helix-turn-helix_domain-containing_protein_(plasmid)_[Bacillus_cereus]	CPTF_metals_mix	0	0	0
UIJ69880.1	helix-turn-helix_domain-containing_protein_(plasmid)_[Bacillus_cereus]	CPTF_zcontrol	0	0	0
UIJ69885.1	hypothetical_protein_LW858_31605_(plasmid)_[Bacillus_cereus]	CPTF_Al	141452.3333	26818.0276	18.95905636
UIJ69885.1	hypothetical_protein_LW858_31605_(plasmid)_[Bacillus_cereus]	CPTF_Cd	168601.6667	85163.37053	50.51158284
UIJ69885.1	hypothetical_protein_LW858_31605_(plasmid)_[Bacillus_cereus]	CPTF_Co	175501.0667	29453.5141	16.78252712
UIJ69885.1	hypothetical_protein_LW858_31605_(plasmid)_[Bacillus_cereus]	CPTF_Cu	329655	307730.7288	93.34932848
UIJ69885.1	hypothetical_protein_LW858_31605_(plasmid)_[Bacillus_cereus]	CPTF_Fe	27217.56667	47142.20833	173.2050808
UIJ69885.1	hypothetical_protein_LW858_31605_(plasmid)_[Bacillus_cereus]	CPTF_Mn	410988.2	283388.2259	68.95288622
UIJ69885.1	hypothetical_protein_LW858_31605_(plasmid)_[Bacillus_cereus]	CPTF_Ni	238992	230734.7746	96.54497833
UIJ69885.1	hypothetical_protein_LW858_31605_(plasmid)_[Bacillus_cereus]	CPTF_U	179240.3333	310453.3641	173.2050808
UIJ69885.1	hypothetical_protein_LW858_31605_(plasmid)_[Bacillus_cereus]	CPTF_metals_mix	855561.2667	234761.0029	27.43941458
UIJ69885.1	hypothetical_protein_LW858_31605_(plasmid)_[Bacillus_cereus]	CPTF_zcontrol	82784.43333	41784.9727	50.47443223
UIJ69895.1	hypothetical_protein_LW858_31670_(plasmid)_[Bacillus_cereus]	CPTF_Al	188882.3333	163723.6504	86.68023502
UIJ69895.1	hypothetical_protein_LW858_31670_(plasmid)_[Bacillus_cereus]	CPTF_Cd	552626.3333	378128.8754	68.42396979
UIJ69895.1	hypothetical_protein_LW858_31670_(plasmid)_[Bacillus_cereus]	CPTF_Co	296385.5667	370167.3923	124.8938659
UIJ69895.1	hypothetical_protein_LW858_31670_(plasmid)_[Bacillus_cereus]	CPTF_Cu	362185.7333	467136.4257	128.9770366
UIJ69895.1	hypothetical_protein_LW858_31670_(plasmid)_[Bacillus_cereus]	CPTF_Fe	689960.6667	425312.6473	61.64302806
UIJ69895.1	hypothetical_protein_LW858_31670_(plasmid)_[Bacillus_cereus]	CPTF_Mn	754822.9667	589585.0187	78.10904606
UIJ69895.1	hypothetical_protein_LW858_31670_(plasmid)_[Bacillus_cereus]	CPTF_Ni	0	0	0
UIJ69895.1	hypothetical_protein_LW858_31670_(plasmid)_[Bacillus_cereus]	CPTF_U	248898	431103.9819	173.2050808
UIJ69895.1	hypothetical_protein_LW858_31670_(plasmid)_[Bacillus_cereus]	CPTF_metals_mix	930710.7667	627992.5288	67.47450994
UIJ69895.1	hypothetical_protein_LW858_31670_(plasmid)_[Bacillus_cereus]	CPTF_zcontrol	351928.8333	321843.6759	91.45135193
UIJ69917.1	helix-turn-helix_domain-containing_protein_(plasmid)_[Bacillus_cereus]	CPTF_Al	25510.9	44186.17495	173.2050808
UIJ69917.1	helix-turn-helix_domain-containing_protein_(plasmid)_[Bacillus_cereus]	CPTF_Cd	0	0	0
UIJ69917.1	helix-turn-helix_domain-containing_protein_(plasmid)_[Bacillus_cereus]	CPTF_Co	82284.56667	73175.43397	88.92971906
UIJ69917.1	helix-turn-helix_domain-containing_protein_(plasmid)_[Bacillus_cereus]	CPTF_Cu	67939.43333	51292.79544	75.49782641
UIJ69917.1	helix-turn-helix_domain-containing_protein_(plasmid)_[Bacillus_cereus]	CPTF_Fe	46282.96667	40888.4914	88.34457759
UIJ69917.1	helix-turn-helix_domain-containing_protein_(plasmid)_[Bacillus_cereus]	CPTF_Mn	0	0	0

UIJ69917.1	helix-turn-helix_domain-containing_protein_(plasmid)_[Bacillus_cereus]	CPTF_Ni	45765.53333	61104.45793	133.5163244
UIJ69917.1	helix-turn-helix_domain-containing_protein_(plasmid)_[Bacillus_cereus]	CPTF_U	0	0	0
UIJ69917.1	helix-turn-helix_domain-containing_protein_(plasmid)_[Bacillus_cereus]	CPTF_metals_mix	17746.16667	30737.26231	173.2050808
UIJ69917.1	helix-turn-helix_domain-containing_protein_(plasmid)_[Bacillus_cereus]	CPTF_zcontrol	63748.66667	69974.9198	109.7668759
UIJ69922.1	Rpn_family_recombination-promoting_nuclease/putative_transposase_(plasmid)_[Bacillus_cereus]	CPTF_Al	214511	25082.25171	11.69275781
UIJ69922.1	Rpn_family_recombination-promoting_nuclease/putative_transposase_(plasmid)_[Bacillus_cereus]	CPTF_Cd	149576.1667	119794.1573	80.08906768
UIJ69922.1	Rpn_family_recombination-promoting_nuclease/putative_transposase_(plasmid)_[Bacillus_cereus]	CPTF_Co	58742.66667	101745.2832	173.2050808
UIJ69922.1	Rpn_family_recombination-promoting_nuclease/putative_transposase_(plasmid)_[Bacillus_cereus]	CPTF_Cu	148854.3333	257823.2683	173.2050808
UIJ69922.1	Rpn_family_recombination-promoting_nuclease/putative_transposase_(plasmid)_[Bacillus_cereus]	CPTF_Fe	11293.4	19560.74259	173.2050808
UIJ69922.1	Rpn_family_recombination-promoting_nuclease/putative_transposase_(plasmid)_[Bacillus_cereus]	CPTF_Mn	232676.1667	211847.9023	91.0483894
UIJ69922.1	Rpn_family_recombination-promoting_nuclease/putative_transposase_(plasmid)_[Bacillus_cereus]	CPTF_Ni	314119	287610.5053	91.56100245
UIJ69922.1	Rpn_family_recombination-promoting_nuclease/putative_transposase_(plasmid)_[Bacillus_cereus]	CPTF_U	238184.3333	210659.9984	88.44410356
UIJ69922.1	Rpn_family_recombination-promoting_nuclease/putative_transposase_(plasmid)_[Bacillus_cereus]	CPTF_metals_mix	581625	34635.34803	5.954927664
UIJ69922.1	Rpn_family_recombination-promoting_nuclease/putative_transposase_(plasmid)_[Bacillus_cereus]	CPTF_zcontrol	0	0	0
UIJ69928.1	transposase_(plasmid)_[Bacillus_cereus]	CPTF_Al	17313.8	29988.38127	173.2050808
UIJ69928.1	transposase_(plasmid)_[Bacillus_cereus]	CPTF_Cd	50640.43333	44125.47454	87.1348676
UIJ69928.1	transposase_(plasmid)_[Bacillus_cereus]	CPTF_Co	0	0	0
UIJ69928.1	transposase_(plasmid)_[Bacillus_cereus]	CPTF_Cu	0	0	0
UIJ69928.1	transposase_(plasmid)_[Bacillus_cereus]	CPTF_Fe	0	0	0
UIJ69928.1	transposase_(plasmid)_[Bacillus_cereus]	CPTF_Mn	0	0	0
UIJ69928.1	transposase_(plasmid)_[Bacillus_cereus]	CPTF_Ni	0	0	0
UIJ69928.1	transposase_(plasmid)_[Bacillus_cereus]	CPTF_U	0	0	0
UIJ69928.1	transposase_(plasmid)_[Bacillus_cereus]	CPTF_metals_mix	23380.06667	40495.46335	173.2050808
UIJ69928.1	transposase_(plasmid)_[Bacillus_cereus]	CPTF_zcontrol	20979	36336.69389	173.2050808
UIJ69930.1	MMPL_family_transporter_(plasmid)_[Bacillus_cereus]	CPTF_Al	808356.1	31264.76796	3.867697412
UIJ69930.1	MMPL_family_transporter_(plasmid)_[Bacillus_cereus]	CPTF_Cd	1063607.333	32119.78195	3.019890982
UIJ69930.1	MMPL_family_transporter_(plasmid)_[Bacillus_cereus]	CPTF_Co	938468.8333	40734.44047	4.340521392
UIJ69930.1	MMPL_family_transporter_(plasmid)_[Bacillus_cereus]	CPTF_Cu	603615.6333	42271.84037	7.003105625
UIJ69930.1	MMPL_family_transporter_(plasmid)_[Bacillus_cereus]	CPTF_Fe	731546.2	238136.9567	32.55255193
UIJ69930.1	MMPL_family_transporter_(plasmid)_[Bacillus_cereus]	CPTF_Mn	707106.0667	117887.6003	16.67184117
UIJ69930.1	MMPL_family_transporter_(plasmid)_[Bacillus_cereus]	CPTF_Ni	832656.7333	104550.0145	12.55619637
UIJ69930.1	MMPL_family_transporter_(plasmid)_[Bacillus_cereus]	CPTF_U	739184.5333	201883.9455	27.31171127
UIJ69930.1	MMPL_family_transporter_(plasmid)_[Bacillus_cereus]	CPTF_metals_mix	417937.3	147517.3306	35.29652189
UIJ69930.1	MMPL_family_transporter_(plasmid)_[Bacillus_cereus]	CPTF_zcontrol	653956.0667	143537.7965	21.94914976
UIJ69954.1	efflux_RND_transporter_periplasmic_adaptor_subunit_(plasmid)_[Bacillus_cereus]	CPTF_Al	118611.8	31730.2119	26.75131134
UIJ69954.1	efflux_RND_transporter_periplasmic_adaptor_subunit_(plasmid)_[Bacillus_cereus]	CPTF_Cd	119850.7	39144.98709	32.66145887
UIJ69954.1	efflux_RND_transporter_periplasmic_adaptor_subunit_(plasmid)_[Bacillus_cereus]	CPTF_Co	150283.1667	72851.29912	48.47602079
UIJ69954.1	efflux_RND_transporter_periplasmic_adaptor_subunit_(plasmid)_[Bacillus_cereus]	CPTF_Cu	72397.43333	32105.31273	44.34592671
UIJ69954.1	efflux_RND_transporter_periplasmic_adaptor_subunit_(plasmid)_[Bacillus_cereus]	CPTF_Fe	85994.4	51984.58005	60.45112245
UIJ69954.1	efflux_RND_transporter_periplasmic_adaptor_subunit_(plasmid)_[Bacillus_cereus]	CPTF_Mn	85206.76667	25393.18053	29.80183561
UIJ69954.1	efflux_RND_transporter_periplasmic_adaptor_subunit_(plasmid)_[Bacillus_cereus]	CPTF_Ni	111588.4333	24739.20883	22.17004764
UIJ69954.1	efflux_RND_transporter_periplasmic_adaptor_subunit_(plasmid)_[Bacillus_cereus]	CPTF_U	79984.43333	26390.79349	32.99491213
UIJ69954.1	efflux_RND_transporter_periplasmic_adaptor_subunit_(plasmid)_[Bacillus_cereus]	CPTF_metals_mix	25865.03333	15842.02744	61.24881894
UIJ69954.1	efflux_RND_transporter_periplasmic_adaptor_subunit_(plasmid)_[Bacillus_cereus]	CPTF_zcontrol	80260.26667	13566.1988	16.90275819
UIJ69961.1	DUF2089_domain-containing_protein_(plasmid)_[Bacillus_cereus]	CPTF_Al	0	0	0
UIJ69961.1	DUF2089_domain-containing_protein_(plasmid)_[Bacillus_cereus]	CPTF_Cd	0	0	0
UIJ69961.1	DUF2089_domain-containing_protein_(plasmid)_[Bacillus_cereus]	CPTF_Co	0	0	0
UIJ69961.1	DUF2089_domain-containing_protein_(plasmid)_[Bacillus_cereus]	CPTF_Cu	17054.5	4046.613422	23.72754066
UIJ69961.1	DUF2089_domain-containing_protein_(plasmid)_[Bacillus_cereus]	CPTF_Fe	0	0	0
UIJ69961.1	DUF2089_domain-containing_protein_(plasmid)_[Bacillus_cereus]	CPTF_Mn	0	0	0
UIJ69961.1	DUF2089_domain-containing_protein_(plasmid)_[Bacillus_cereus]	CPTF_Ni	3696.366667	6402.29487	173.2050808
UIJ69961.1	DUF2089_domain-containing_protein_(plasmid)_[Bacillus_cereus]	CPTF_U	0	0	0
UIJ69961.1	DUF2089_domain-containing_protein_(plasmid)_[Bacillus_cereus]	CPTF_metals_mix	8421.643333	10941.53676	129.9216356
UIJ69961.1	DUF2089_domain-containing_protein_(plasmid)_[Bacillus_cereus]	CPTF_zcontrol	0	0	0
UIJ69965.1	ParM/StbA_family_protein_(plasmid)_[Bacillus_cereus]	CPTF_Al	1530745.267	209045.9221	13.65648006
UIJ69965.1	ParM/StbA_family_protein_(plasmid)_[Bacillus_cereus]	CPTF_Cd	1604748.133	151166.2954	9.419939005
UIJ69965.1	ParM/StbA_family_protein_(plasmid)_[Bacillus_cereus]	CPTF_Co	963880.8667	92739.3196	9.62145041
UIJ69965.1	ParM/StbA_family_protein_(plasmid)_[Bacillus_cereus]	CPTF_Cu	1687492.3	606855.9244	35.96199665
UIJ69965.1	ParM/StbA_family_protein_(plasmid)_[Bacillus_cereus]	CPTF_Fe	1287279.667	373097.5807	28.98341288
UIJ69965.1	ParM/StbA_family_protein_(plasmid)_[Bacillus_cereus]	CPTF_Mn	1543666.5	233707.0314	15.13973591
UIJ69965.1	ParM/StbA_family_protein_(plasmid)_[Bacillus_cereus]	CPTF_Ni	868558.7667	109504.8596	12.60765118

UIJ69965.1	ParM/StbA_family_protein_(plasmid)_[Bacillus_cereus]	CPTF_U	843415.3	7022.412753	0.832616239
UIJ69965.1	ParM/StbA_family_protein_(plasmid)_[Bacillus_cereus]	CPTF_metals_mix	719310.8667	20143.53306	2.800393265
UIJ69965.1	ParM/StbA_family_protein_(plasmid)_[Bacillus_cereus]	CPTF_zcontrol	847339.9333	89170.95304	10.52363397
UIJ69972.1	peptidylprolyl_isomerase_(plasmid)_[Bacillus_cereus]	CPTF_Al	7661499.7	547111.6649	7.141051834
UIJ69972.1	peptidylprolyl_isomerase_(plasmid)_[Bacillus_cereus]	CPTF_Cd	8372708.667	1451507.616	17.33617726
UIJ69972.1	peptidylprolyl_isomerase_(plasmid)_[Bacillus_cereus]	CPTF_Co	7209297.933	1005576.547	13.94832834
UIJ69972.1	peptidylprolyl_isomerase_(plasmid)_[Bacillus_cereus]	CPTF_Cu	8143979.733	1237432.209	15.19444117
UIJ69972.1	peptidylprolyl_isomerase_(plasmid)_[Bacillus_cereus]	CPTF_Fe	7279122.967	527732.597	7.249947548
UIJ69972.1	peptidylprolyl_isomerase_(plasmid)_[Bacillus_cereus]	CPTF_Mn	9267591.933	2327682.55	25.1163686
UIJ69972.1	peptidylprolyl_isomerase_(plasmid)_[Bacillus_cereus]	CPTF_Ni	6426473.4	472472.1397	7.351966005
UIJ69972.1	peptidylprolyl_isomerase_(plasmid)_[Bacillus_cereus]	CPTF_U	6226495.533	1280889.233	20.57159162
UIJ69972.1	peptidylprolyl_isomerase_(plasmid)_[Bacillus_cereus]	CPTF_metals_mix	14085989.47	1567701.789	11.1295113
UIJ69972.1	peptidylprolyl_isomerase_(plasmid)_[Bacillus_cereus]	CPTF_zcontrol	7437823.133	480504.6957	6.460286661
UIJ69986.1	ABC_transporter_ATP-binding_protein_(plasmid)_[Bacillus_cereus]	CPTF_Al	504838.3333	45267.36882	8.966705941
UIJ69986.1	ABC_transporter_ATP-binding_protein_(plasmid)_[Bacillus_cereus]	CPTF_Cd	519633.6333	5167.040778	0.994362267
UIJ69986.1	ABC_transporter_ATP-binding_protein_(plasmid)_[Bacillus_cereus]	CPTF_Co	571141.9667	27488.17849	4.812845158
UIJ69986.1	ABC_transporter_ATP-binding_protein_(plasmid)_[Bacillus_cereus]	CPTF_Cu	541720.8333	68370.37874	12.62096167
UIJ69986.1	ABC_transporter_ATP-binding_protein_(plasmid)_[Bacillus_cereus]	CPTF_Fe	549228.1667	54302.07271	9.886978856
UIJ69986.1	ABC_transporter_ATP-binding_protein_(plasmid)_[Bacillus_cereus]	CPTF_Mn	550252.5	173117.6668	31.46149573
UIJ69986.1	ABC_transporter_ATP-binding_protein_(plasmid)_[Bacillus_cereus]	CPTF_Ni	595260.3667	101280.8525	17.01454661
UIJ69986.1	ABC_transporter_ATP-binding_protein_(plasmid)_[Bacillus_cereus]	CPTF_U	677989	133254.0112	19.65430283
UIJ69986.1	ABC_transporter_ATP-binding_protein_(plasmid)_[Bacillus_cereus]	CPTF_metals_mix	429353.1	176079.607	41.01044268
UIJ69986.1	ABC_transporter_ATP-binding_protein_(plasmid)_[Bacillus_cereus]	CPTF_zcontrol	673154.3333	22228.89186	3.302198435
UIJ69989.1	efflux_RND_transporter_periplasmic_adaptor_subunit_(plasmid)_[Bacillus_cereus]	CPTF_Al	27913.5	10910.40357	39.08647634
UIJ69989.1	efflux_RND_transporter_periplasmic_adaptor_subunit_(plasmid)_[Bacillus_cereus]	CPTF_Cd	0	0	0
UIJ69989.1	efflux_RND_transporter_periplasmic_adaptor_subunit_(plasmid)_[Bacillus_cereus]	CPTF_Co	94794.46333	38955.71127	41.09492254
UIJ69989.1	efflux_RND_transporter_periplasmic_adaptor_subunit_(plasmid)_[Bacillus_cereus]	CPTF_Cu	21023.8	8509.823002	40.47709264
UIJ69989.1	efflux_RND_transporter_periplasmic_adaptor_subunit_(plasmid)_[Bacillus_cereus]	CPTF_Fe	6248.466667	10822.66174	173.2050808
UIJ69989.1	efflux_RND_transporter_periplasmic_adaptor_subunit_(plasmid)_[Bacillus_cereus]	CPTF_Mn	7344.3	12720.70075	173.2050808
UIJ69989.1	efflux_RND_transporter_periplasmic_adaptor_subunit_(plasmid)_[Bacillus_cereus]	CPTF_Ni	61000.83333	64025.44718	104.9583156
UIJ69989.1	efflux_RND_transporter_periplasmic_adaptor_subunit_(plasmid)_[Bacillus_cereus]	CPTF_U	16456	14423.71171	87.6501684
UIJ69989.1	efflux_RND_transporter_periplasmic_adaptor_subunit_(plasmid)_[Bacillus_cereus]	CPTF_metals_mix	10208.66667	8870.543544	86.89228314
UIJ69989.1	efflux_RND_transporter_periplasmic_adaptor_subunit_(plasmid)_[Bacillus_cereus]	CPTF_zcontrol	26656.96667	6697.898521	25.12625913
UIJ69993.1	Rpn_family_recombination-promoting_nuclease/putative_transposase_(plasmid)_[Bacillus_cereus]	CPTF_Al	198118.9667	128858.0505	65.04074429
UIJ69993.1	Rpn_family_recombination-promoting_nuclease/putative_transposase_(plasmid)_[Bacillus_cereus]	CPTF_Cd	146193.3333	131992.1567	90.28602995
UIJ69993.1	Rpn_family_recombination-promoting_nuclease/putative_transposase_(plasmid)_[Bacillus_cereus]	CPTF_Co	269096.4667	77265.95249	28.71310554
UIJ69993.1	Rpn_family_recombination-promoting_nuclease/putative_transposase_(plasmid)_[Bacillus_cereus]	CPTF_Cu	311401.9667	39370.52972	12.64299328
UIJ69993.1	Rpn_family_recombination-promoting_nuclease/putative_transposase_(plasmid)_[Bacillus_cereus]	CPTF_Fe	146053.9	138197.5873	94.62094976
UIJ69993.1	Rpn_family_recombination-promoting_nuclease/putative_transposase_(plasmid)_[Bacillus_cereus]	CPTF_Mn	83853.5	80925.17821	96.50781209
UIJ69993.1	Rpn_family_recombination-promoting_nuclease/putative_transposase_(plasmid)_[Bacillus_cereus]	CPTF_Ni	263692.1	35128.26007	13.32169605
UIJ69993.1	Rpn_family_recombination-promoting_nuclease/putative_transposase_(plasmid)_[Bacillus_cereus]	CPTF_U	208819.6667	123420.7235	59.10397496
UIJ69993.1	Rpn_family_recombination-promoting_nuclease/putative_transposase_(plasmid)_[Bacillus_cereus]	CPTF_metals_mix	281947.8333	203027.4691	72.00887723
UIJ69993.1	Rpn_family_recombination-promoting_nuclease/putative_transposase_(plasmid)_[Bacillus_cereus]	CPTF_zcontrol	161095.4	139953.2598	86.87601246
UIJ70000.1	HU_family_DNA-binding_protein_(plasmid)_[Bacillus_cereus]	CPTF_Al	720051.3	71806.26877	9.972382353
UIJ70000.1	HU_family_DNA-binding_protein_(plasmid)_[Bacillus_cereus]	CPTF_Cd	804826.9	76607.91271	9.518557681
UIJ70000.1	HU_family_DNA-binding_protein_(plasmid)_[Bacillus_cereus]	CPTF_Co	937619.1667	86566.15159	9.232549276
UIJ70000.1	HU_family_DNA-binding_protein_(plasmid)_[Bacillus_cereus]	CPTF_Cu	804090.4333	58584.96473	7.285867646
UIJ70000.1	HU_family_DNA-binding_protein_(plasmid)_[Bacillus_cereus]	CPTF_Fe	721742.0333	61008.03109	8.452885972
UIJ70000.1	HU_family_DNA-binding_protein_(plasmid)_[Bacillus_cereus]	CPTF_Mn	770318.6333	96974.60796	12.58889553
UIJ70000.1	HU_family_DNA-binding_protein_(plasmid)_[Bacillus_cereus]	CPTF_Ni	811978.8333	65949.31423	8.122048448
UIJ70000.1	HU_family_DNA-binding_protein_(plasmid)_[Bacillus_cereus]	CPTF_U	596459	73551.61841	12.33137876
UIJ70000.1	HU_family_DNA-binding_protein_(plasmid)_[Bacillus_cereus]	CPTF_metals_mix	1653892.933	139601.3234	8.440771505
UIJ70000.1	HU_family_DNA-binding_protein_(plasmid)_[Bacillus_cereus]	CPTF_zcontrol	648020	41397.93913	6.388373681
UIJ70014.1	tetratricopeptide_repeat_protein_(plasmid)_[Bacillus_cereus]	CPTF_Al	2278974.333	753771.5534	33.07503478
UIJ70014.1	tetratricopeptide_repeat_protein_(plasmid)_[Bacillus_cereus]	CPTF_Cd	1774602.333	483572.1542	27.24960658
UIJ70014.1	tetratricopeptide_repeat_protein_(plasmid)_[Bacillus_cereus]	CPTF_Co	2379886	445543.1085	18.72119541
UIJ70014.1	tetratricopeptide_repeat_protein_(plasmid)_[Bacillus_cereus]	CPTF_Cu	1907118	527943.1239	27.6827718
UIJ70014.1	tetratricopeptide_repeat_protein_(plasmid)_[Bacillus_cereus]	CPTF_Fe	1866762.667	72634.60328	3.890939356
UIJ70014.1	tetratricopeptide_repeat_protein_(plasmid)_[Bacillus_cereus]	CPTF_Mn	2554711.833	356382.8014	13.9500196
UIJ70014.1	tetratricopeptide_repeat_protein_(plasmid)_[Bacillus_cereus]	CPTF_Ni	2238110.667	211659.9559	9.457081773
UIJ70014.1	tetratricopeptide_repeat_protein_(plasmid)_[Bacillus_cereus]	CPTF_U	1584564	1028205.451	64.8888559

UIJ70014.1	tetratricopeptide_repeat_protein_(plasmid)_[Bacillus_cereus]	CPTF_metals_mix	942728.8667	146807.0661	15.57256506
UIJ70014.1	tetratricopeptide_repeat_protein_(plasmid)_[Bacillus_cereus]	CPTF_zcontrol	2295345.333	993757.7223	43.29447547
UIJ70017.1	hypothetical_protein_LW858_32375_(plasmid)_[Bacillus_cereus]	CPTF_Al	142923.6667	127868.3285	89.46616852
UIJ70017.1	hypothetical_protein_LW858_32375_(plasmid)_[Bacillus_cereus]	CPTF_Cd	206223.6667	48537.99668	23.53657922
UIJ70017.1	hypothetical_protein_LW858_32375_(plasmid)_[Bacillus_cereus]	CPTF_Co	98376	85630.78139	87.04438215
UIJ70017.1	hypothetical_protein_LW858_32375_(plasmid)_[Bacillus_cereus]	CPTF_Cu	179445.6667	26711.79225	14.88572711
UIJ70017.1	hypothetical_protein_LW858_32375_(plasmid)_[Bacillus_cereus]	CPTF_Fe	187411.6667	12750.41538	6.803426705
UIJ70017.1	hypothetical_protein_LW858_32375_(plasmid)_[Bacillus_cereus]	CPTF_Mn	86232	74679.97033	86.60354663
UIJ70017.1	hypothetical_protein_LW858_32375_(plasmid)_[Bacillus_cereus]	CPTF_Ni	51918	89924.61383	173.2050808
UIJ70017.1	hypothetical_protein_LW858_32375_(plasmid)_[Bacillus_cereus]	CPTF_U	157071.6667	25323.91483	16.12252252
UIJ70017.1	hypothetical_protein_LW858_32375_(plasmid)_[Bacillus_cereus]	CPTF_metals_mix	222059.4333	51469.04964	23.17805142
UIJ70017.1	hypothetical_protein_LW858_32375_(plasmid)_[Bacillus_cereus]	CPTF_zcontrol	182199.6667	19804.96191	10.86992214
UIJ70018.1	ParM/StbA_family_protein_(plasmid)_[Bacillus_cereus]	CPTF_Al	2335074.4	306439.3888	13.12332441
UIJ70018.1	ParM/StbA_family_protein_(plasmid)_[Bacillus_cereus]	CPTF_Cd	1998424.767	104155.6288	5.211886409
UIJ70018.1	ParM/StbA_family_protein_(plasmid)_[Bacillus_cereus]	CPTF_Co	2242216.1	87051.82308	3.882401124
UIJ70018.1	ParM/StbA_family_protein_(plasmid)_[Bacillus_cereus]	CPTF_Cu	2669852.5	374749.8801	14.03635145
UIJ70018.1	ParM/StbA_family_protein_(plasmid)_[Bacillus_cereus]	CPTF_Fe	2068966.067	121186.9374	5.857367087
UIJ70018.1	ParM/StbA_family_protein_(plasmid)_[Bacillus_cereus]	CPTF_Mn	1630693.5	173922.1639	10.66553365
UIJ70018.1	ParM/StbA_family_protein_(plasmid)_[Bacillus_cereus]	CPTF_Ni	2216731.033	239254.2231	10.79311019
UIJ70018.1	ParM/StbA_family_protein_(plasmid)_[Bacillus_cereus]	CPTF_U	1800254.433	575117.264	31.94644342
UIJ70018.1	ParM/StbA_family_protein_(plasmid)_[Bacillus_cereus]	CPTF_metals_mix	2421000.433	54456.3974	2.249334475
UIJ70018.1	ParM/StbA_family_protein_(plasmid)_[Bacillus_cereus]	CPTF_zcontrol	1661466.967	621634.5492	37.41480039
UIJ70027.1	hypothetical_protein_LW858_32425_(plasmid)_[Bacillus_cereus]	CPTF_Al	1260000	1091237.829	86.60617693
UIJ70027.1	hypothetical_protein_LW858_32425_(plasmid)_[Bacillus_cereus]	CPTF_Cd	570000	987268.9603	173.2050808
UIJ70027.1	hypothetical_protein_LW858_32425_(plasmid)_[Bacillus_cereus]	CPTF_Co	543333.3333	941080.9388	173.2050808
UIJ70027.1	hypothetical_protein_LW858_32425_(plasmid)_[Bacillus_cereus]	CPTF_Cu	0	0	0
UIJ70027.1	hypothetical_protein_LW858_32425_(plasmid)_[Bacillus_cereus]	CPTF_Fe	540000	935307.4361	173.2050808
UIJ70027.1	hypothetical_protein_LW858_32425_(plasmid)_[Bacillus_cereus]	CPTF_Mn	680000	1177794.549	173.2050808
UIJ70027.1	hypothetical_protein_LW858_32425_(plasmid)_[Bacillus_cereus]	CPTF_Ni	0	0	0
UIJ70027.1	hypothetical_protein_LW858_32425_(plasmid)_[Bacillus_cereus]	CPTF_U	197017.6667	341244.6087	173.2050808
UIJ70027.1	hypothetical_protein_LW858_32425_(plasmid)_[Bacillus_cereus]	CPTF_metals_mix	1433333.333	150111.07	10.47286535
UIJ70027.1	hypothetical_protein_LW858_32425_(plasmid)_[Bacillus_cereus]	CPTF_zcontrol	640000	1108512.517	173.2050808
UIJ70029.1	hypothetical_protein_LW858_32435_(plasmid)_[Bacillus_cereus]	CPTF_Al	359159.4	45993.41873	12.80585131
UIJ70029.1	hypothetical_protein_LW858_32435_(plasmid)_[Bacillus_cereus]	CPTF_Cd	14651.93333	25377.89296	173.2050808
UIJ70029.1	hypothetical_protein_LW858_32435_(plasmid)_[Bacillus_cereus]	CPTF_Co	103543.2	137360.7081	132.6602887
UIJ70029.1	hypothetical_protein_LW858_32435_(plasmid)_[Bacillus_cereus]	CPTF_Cu	133464.4	95521.82155	71.57101186
UIJ70029.1	hypothetical_protein_LW858_32435_(plasmid)_[Bacillus_cereus]	CPTF_Fe	113005	195730.4015	173.2050808
UIJ70029.1	hypothetical_protein_LW858_32435_(plasmid)_[Bacillus_cereus]	CPTF_Mn	136387.9333	125980.5615	92.36928698
UIJ70029.1	hypothetical_protein_LW858_32435_(plasmid)_[Bacillus_cereus]	CPTF_Ni	125914.5333	147672.84	117.2802186
UIJ70029.1	hypothetical_protein_LW858_32435_(plasmid)_[Bacillus_cereus]	CPTF_U	0	0	0
UIJ70029.1	hypothetical_protein_LW858_32435_(plasmid)_[Bacillus_cereus]	CPTF_metals_mix	49469.33333	76648.748	154.9419465
UIJ70029.1	hypothetical_protein_LW858_32435_(plasmid)_[Bacillus_cereus]	CPTF_zcontrol	47820.43333	82827.42017	173.2050808
UIJ70064.1	HU_family_DNA-binding_protein_(plasmid)_[Bacillus_cereus]	CPTF_Al	869577.2	44767.13649	5.148149755
UIJ70064.1	HU_family_DNA-binding_protein_(plasmid)_[Bacillus_cereus]	CPTF_Cd	763461.2667	147415.7141	19.30886616
UIJ70064.1	HU_family_DNA-binding_protein_(plasmid)_[Bacillus_cereus]	CPTF_Co	938693.4667	26464.70827	2.819313142
UIJ70064.1	HU_family_DNA-binding_protein_(plasmid)_[Bacillus_cereus]	CPTF_Cu	911101.3333	80406.37627	8.825184788
UIJ70064.1	HU_family_DNA-binding_protein_(plasmid)_[Bacillus_cereus]	CPTF_Fe	827153.7	39142.00757	4.73213232
UIJ70064.1	HU_family_DNA-binding_protein_(plasmid)_[Bacillus_cereus]	CPTF_Mn	729414.6	65155.42106	8.932563327
UIJ70064.1	HU_family_DNA-binding_protein_(plasmid)_[Bacillus_cereus]	CPTF_Ni	974560	94379.72539	9.684342204
UIJ70064.1	HU_family_DNA-binding_protein_(plasmid)_[Bacillus_cereus]	CPTF_U	956002.0667	176332.4064	18.44477251
UIJ70064.1	HU_family_DNA-binding_protein_(plasmid)_[Bacillus_cereus]	CPTF_metals_mix	891319.8667	35248.54776	3.954646258
UIJ70064.1	HU_family_DNA-binding_protein_(plasmid)_[Bacillus_cereus]	CPTF_zcontrol	897780.0667	74416.648	8.288961937
UIJ70065.1	hypothetical_protein_LW858_32620_(plasmid)_[Bacillus_cereus]	CPTF_Al	0	0	0
UIJ70065.1	hypothetical_protein_LW858_32620_(plasmid)_[Bacillus_cereus]	CPTF_Cd	0	0	0
UIJ70065.1	hypothetical_protein_LW858_32620_(plasmid)_[Bacillus_cereus]	CPTF_Co	0	0	0
UIJ70065.1	hypothetical_protein_LW858_32620_(plasmid)_[Bacillus_cereus]	CPTF_Cu	0	0	0
UIJ70065.1	hypothetical_protein_LW858_32620_(plasmid)_[Bacillus_cereus]	CPTF_Fe	0	0	0
UIJ70065.1	hypothetical_protein_LW858_32620_(plasmid)_[Bacillus_cereus]	CPTF_Mn	0	0	0
UIJ70065.1	hypothetical_protein_LW858_32620_(plasmid)_[Bacillus_cereus]	CPTF_Ni	0	0	0
UIJ70065.1	hypothetical_protein_LW858_32620_(plasmid)_[Bacillus_cereus]	CPTF_U	0	0	0
UIJ70065.1	hypothetical_protein_LW858_32620_(plasmid)_[Bacillus_cereus]	CPTF_metals_mix	48150	83398.24638	173.2050808

UIJ70065.1	hypothetical_protein_LW858_32620_(plasmid)_[Bacillus_cereus]	CPTF_zcontrol	0	0	0
UIJ70084.1	DUF2325_domain-containing_protein_(plasmid)_[Bacillus_cereus]	CPTF_Al	367006.3333	47718.84203	13.00218489
UIJ70084.1	DUF2325_domain-containing_protein_(plasmid)_[Bacillus_cereus]	CPTF_Cd	739350.6667	36872.48991	4.987145014
UIJ70084.1	DUF2325_domain-containing_protein_(plasmid)_[Bacillus_cereus]	CPTF_Co	393982.3667	110052.5449	27.93336815
UIJ70084.1	DUF2325_domain-containing_protein_(plasmid)_[Bacillus_cereus]	CPTF_Cu	319737	69425.90546	21.71344119
UIJ70084.1	DUF2325_domain-containing_protein_(plasmid)_[Bacillus_cereus]	CPTF_Fe	344991	54251.913	15.72560241
UIJ70084.1	DUF2325_domain-containing_protein_(plasmid)_[Bacillus_cereus]	CPTF_Mn	168596.1667	214359.8729	127.1439779
UIJ70084.1	DUF2325_domain-containing_protein_(plasmid)_[Bacillus_cereus]	CPTF_Ni	203839.6	176843.8469	86.75637459
UIJ70084.1	DUF2325_domain-containing_protein_(plasmid)_[Bacillus_cereus]	CPTF_U	149124.3333	133361.2017	89.42953756
UIJ70084.1	DUF2325_domain-containing_protein_(plasmid)_[Bacillus_cereus]	CPTF_metals_mix	685225.8	259106.6188	37.81331917
UIJ70084.1	DUF2325_domain-containing_protein_(plasmid)_[Bacillus_cereus]	CPTF_zcontrol	287756.3333	83731.51827	29.09806269
UIJ70088.1	tyrosine-type_recombinase/integrase_(plasmid)_[Bacillus_cereus]	CPTF_Al	26320.33333	28427.11647	108.0043938
UIJ70088.1	tyrosine-type_recombinase/integrase_(plasmid)_[Bacillus_cereus]	CPTF_Cd	0	0	0
UIJ70088.1	tyrosine-type_recombinase/integrase_(plasmid)_[Bacillus_cereus]	CPTF_Co	47163.56667	29585.1632	62.72885046
UIJ70088.1	tyrosine-type_recombinase/integrase_(plasmid)_[Bacillus_cereus]	CPTF_Cu	73912.4	83189.88668	112.552003
UIJ70088.1	tyrosine-type_recombinase/integrase_(plasmid)_[Bacillus_cereus]	CPTF_Fe	12214.43333	21156.01912	173.2050808
UIJ70088.1	tyrosine-type_recombinase/integrase_(plasmid)_[Bacillus_cereus]	CPTF_Mn	0	0	0
UIJ70088.1	tyrosine-type_recombinase/integrase_(plasmid)_[Bacillus_cereus]	CPTF_Ni	8328.333333	14425.09648	173.2050808
UIJ70088.1	tyrosine-type_recombinase/integrase_(plasmid)_[Bacillus_cereus]	CPTF_U	7840.166667	13579.56701	173.2050808
UIJ70088.1	tyrosine-type_recombinase/integrase_(plasmid)_[Bacillus_cereus]	CPTF_metals_mix	35852.33333	62098.0629	173.2050808
UIJ70088.1	tyrosine-type_recombinase/integrase_(plasmid)_[Bacillus_cereus]	CPTF_zcontrol	30621.43333	53037.87833	173.2050808
UIJ70108.1	ParM/StbA_family_protein_(plasmid)_[Bacillus_cereus]	CPTF_Al	1609361.333	84556.04512	5.254012469
UIJ70108.1	ParM/StbA_family_protein_(plasmid)_[Bacillus_cereus]	CPTF_Cd	1566455.333	61096.38821	3.900295585
UIJ70108.1	ParM/StbA_family_protein_(plasmid)_[Bacillus_cereus]	CPTF_Co	1502566.433	145356.4245	9.673876724
UIJ70108.1	ParM/StbA_family_protein_(plasmid)_[Bacillus_cereus]	CPTF_Cu	1603746.333	219950.0118	13.71476319
UIJ70108.1	ParM/StbA_family_protein_(plasmid)_[Bacillus_cereus]	CPTF_Fe	1529635.333	528502.3757	34.55087394
UIJ70108.1	ParM/StbA_family_protein_(plasmid)_[Bacillus_cereus]	CPTF_Mn	1784294.133	181399.8987	10.16647958
UIJ70108.1	ParM/StbA_family_protein_(plasmid)_[Bacillus_cereus]	CPTF_Ni	2044156	150239.6764	7.349716773
UIJ70108.1	ParM/StbA_family_protein_(plasmid)_[Bacillus_cereus]	CPTF_U	1335510.333	579420.5176	43.38570082
UIJ70108.1	ParM/StbA_family_protein_(plasmid)_[Bacillus_cereus]	CPTF_metals_mix	1279965.667	249502.9317	19.49293939
UIJ70108.1	ParM/StbA_family_protein_(plasmid)_[Bacillus_cereus]	CPTF_zcontrol	1939685.333	135027.9392	6.961332174
UIJ70135.1	hypothetical_protein_LW858_33235_(plasmid)_[Bacillus_cereus]	CPTF_Al	368712.3333	61024.76776	16.55078017
UIJ70135.1	hypothetical_protein_LW858_33235_(plasmid)_[Bacillus_cereus]	CPTF_Cd	91998.33333	159345.7875	173.2050808
UIJ70135.1	hypothetical_protein_LW858_33235_(plasmid)_[Bacillus_cereus]	CPTF_Co	399597.6667	54688.63202	13.68592376
UIJ70135.1	hypothetical_protein_LW858_33235_(plasmid)_[Bacillus_cereus]	CPTF_Cu	173979	186861.4043	107.4045743
UIJ70135.1	hypothetical_protein_LW858_33235_(plasmid)_[Bacillus_cereus]	CPTF_Fe	246513.3333	224095.6483	90.90609635
UIJ70135.1	hypothetical_protein_LW858_33235_(plasmid)_[Bacillus_cereus]	CPTF_Mn	294013.6667	68501.21068	23.29864848
UIJ70135.1	hypothetical_protein_LW858_33235_(plasmid)_[Bacillus_cereus]	CPTF_Ni	280482.6667	162294.4199	57.86254881
UIJ70135.1	hypothetical_protein_LW858_33235_(plasmid)_[Bacillus_cereus]	CPTF_U	379064.6667	198418.344	52.34419387
UIJ70135.1	hypothetical_protein_LW858_33235_(plasmid)_[Bacillus_cereus]	CPTF_metals_mix	226584	96463.71586	42.57304834
UIJ70135.1	hypothetical_protein_LW858_33235_(plasmid)_[Bacillus_cereus]	CPTF_zcontrol	342541	26887.66238	7.849472727
UIJ70137.1	hypothetical_protein_LW858_33040_(plasmid)_[Bacillus_cereus]	CPTF_Al	938442.1	231007.7937	24.61609445
UIJ70137.1	hypothetical_protein_LW858_33040_(plasmid)_[Bacillus_cereus]	CPTF_Cd	939878.4667	280952.4851	29.89242706
UIJ70137.1	hypothetical_protein_LW858_33040_(plasmid)_[Bacillus_cereus]	CPTF_Co	1485283.8	153449.174	10.33130329
UIJ70137.1	hypothetical_protein_LW858_33040_(plasmid)_[Bacillus_cereus]	CPTF_Cu	1476209.633	360989.7844	24.45382934
UIJ70137.1	hypothetical_protein_LW858_33040_(plasmid)_[Bacillus_cereus]	CPTF_Fe	814734.5333	141134.2032	17.32272261
UIJ70137.1	hypothetical_protein_LW858_33040_(plasmid)_[Bacillus_cereus]	CPTF_Mn	737814.7667	176563.0647	23.93054092
UIJ70137.1	hypothetical_protein_LW858_33040_(plasmid)_[Bacillus_cereus]	CPTF_Ni	911767.8667	183234.0498	20.09656805
UIJ70137.1	hypothetical_protein_LW858_33040_(plasmid)_[Bacillus_cereus]	CPTF_U	769109.4667	207503.2455	26.97967642
UIJ70137.1	hypothetical_protein_LW858_33040_(plasmid)_[Bacillus_cereus]	CPTF_metals_mix	1920207.7	19440.58797	1.012421103
UIJ70137.1	hypothetical_protein_LW858_33040_(plasmid)_[Bacillus_cereus]	CPTF_zcontrol	812924.7667	11707.44357	1.440163229
UIJ70138.1	helix-turn-helix_domain-containing_protein_(plasmid)_[Bacillus_cereus]	CPTF_Al	504859.6667	236700.7127	46.88445688
UIJ70138.1	helix-turn-helix_domain-containing_protein_(plasmid)_[Bacillus_cereus]	CPTF_Cd	380004.3333	60999.53155	16.05232525
UIJ70138.1	helix-turn-helix_domain-containing_protein_(plasmid)_[Bacillus_cereus]	CPTF_Co	594302.3333	388351.9069	65.34584926
UIJ70138.1	helix-turn-helix_domain-containing_protein_(plasmid)_[Bacillus_cereus]	CPTF_Cu	764066.3333	120252.4651	15.73848498
UIJ70138.1	helix-turn-helix_domain-containing_protein_(plasmid)_[Bacillus_cereus]	CPTF_Fe	470442	151784.1938	32.26416727
UIJ70138.1	helix-turn-helix_domain-containing_protein_(plasmid)_[Bacillus_cereus]	CPTF_Mn	674040.6667	232129.2841	34.43846871
UIJ70138.1	helix-turn-helix_domain-containing_protein_(plasmid)_[Bacillus_cereus]	CPTF_Ni	693993.3333	326897.9461	47.10390293
UIJ70138.1	helix-turn-helix_domain-containing_protein_(plasmid)_[Bacillus_cereus]	CPTF_U	356824.4	240280.3867	67.33855273
UIJ70138.1	helix-turn-helix_domain-containing_protein_(plasmid)_[Bacillus_cereus]	CPTF_metals_mix	594119.8333	210268.1115	35.3915321
UIJ70138.1	helix-turn-helix_domain-containing_protein_(plasmid)_[Bacillus_cereus]	CPTF_zcontrol	363710.6667	20725.16032	5.698254745

UIJ70149.1	Rpn_family_recombination-promoting_nuclease/putative_transposase_(plasmid)_[Bacillus_cereus]	CPTF_Al	1476123.867	16958.89284	1.148880065
UIJ70149.1	Rpn_family_recombination-promoting_nuclease/putative_transposase_(plasmid)_[Bacillus_cereus]	CPTF_Cd	1499199.567	124320.0124	8.292425851
UIJ70149.1	Rpn_family_recombination-promoting_nuclease/putative_transposase_(plasmid)_[Bacillus_cereus]	CPTF_Co	1276682.333	168300.1054	13.18261411
UIJ70149.1	Rpn_family_recombination-promoting_nuclease/putative_transposase_(plasmid)_[Bacillus_cereus]	CPTF_Cu	1320772.5	94032.75918	7.119527336
UIJ70149.1	Rpn_family_recombination-promoting_nuclease/putative_transposase_(plasmid)_[Bacillus_cereus]	CPTF_Fe	1497707.633	280221.4655	18.71002452
UIJ70149.1	Rpn_family_recombination-promoting_nuclease/putative_transposase_(plasmid)_[Bacillus_cereus]	CPTF_Mn	1377679.5	198903.0095	14.43753859
UIJ70149.1	Rpn_family_recombination-promoting_nuclease/putative_transposase_(plasmid)_[Bacillus_cereus]	CPTF_Ni	1033063.2	158144.2211	15.30828134
UIJ70149.1	Rpn_family_recombination-promoting_nuclease/putative_transposase_(plasmid)_[Bacillus_cereus]	CPTF_U	1358568.033	215223.067	15.84190572
UIJ70149.1	Rpn_family_recombination-promoting_nuclease/putative_transposase_(plasmid)_[Bacillus_cereus]	CPTF_metals_mix	1072146.5	66733.18101	6.224259559
UIJ70149.1	Rpn_family_recombination-promoting_nuclease/putative_transposase_(plasmid)_[Bacillus_cereus]	CPTF_zcontrol	1419968.633	71838.02522	5.059127612
UIJ70161.1	S1_family_peptidase_(plasmid)_[Bacillus_cereus]	CPTF_Al	663476.6333	56100.28175	8.455502264
UIJ70161.1	S1_family_peptidase_(plasmid)_[Bacillus_cereus]	CPTF_Cd	325102.3	287890.4711	88.55380941
UIJ70161.1	S1_family_peptidase_(plasmid)_[Bacillus_cereus]	CPTF_Co	458635.4667	313257.839	68.30214009
UIJ70161.1	S1_family_peptidase_(plasmid)_[Bacillus_cereus]	CPTF_Cu	222600.4	249892.6483	112.2606466
UIJ70161.1	S1_family_peptidase_(plasmid)_[Bacillus_cereus]	CPTF_Fe	539298.9667	414549.7345	76.86826049
UIJ70161.1	S1_family_peptidase_(plasmid)_[Bacillus_cereus]	CPTF_Mn	684659.7667	19039.18705	2.780824575
UIJ70161.1	S1_family_peptidase_(plasmid)_[Bacillus_cereus]	CPTF_Ni	307308.6333	377785.1961	122.9334796
UIJ70161.1	S1_family_peptidase_(plasmid)_[Bacillus_cereus]	CPTF_U	280338.0333	355598.5093	126.8463309
UIJ70161.1	S1_family_peptidase_(plasmid)_[Bacillus_cereus]	CPTF_metals_mix	279673.5667	106627.3526	38.12564552
UIJ70161.1	S1_family_peptidase_(plasmid)_[Bacillus_cereus]	CPTF_zcontrol	273069.8	361812.5224	132.4981827
UIJ70166.1	spermidine_N1-acetyltransferase_(plasmid)_[Bacillus_cereus]	CPTF_Al	314208.6667	37217.54777	11.8448508
UIJ70166.1	spermidine_N1-acetyltransferase_(plasmid)_[Bacillus_cereus]	CPTF_Cd	190805.6667	168579.4348	88.35137745
UIJ70166.1	spermidine_N1-acetyltransferase_(plasmid)_[Bacillus_cereus]	CPTF_Co	317088	32386.23881	10.21364379
UIJ70166.1	spermidine_N1-acetyltransferase_(plasmid)_[Bacillus_cereus]	CPTF_Cu	229941.6667	30549.65069	13.28582642
UIJ70166.1	spermidine_N1-acetyltransferase_(plasmid)_[Bacillus_cereus]	CPTF_Fe	182878	158530.4666	86.68646124
UIJ70166.1	spermidine_N1-acetyltransferase_(plasmid)_[Bacillus_cereus]	CPTF_Mn	288271.3333	70034.304	24.29457803
UIJ70166.1	spermidine_N1-acetyltransferase_(plasmid)_[Bacillus_cereus]	CPTF_Ni	209924.3333	185738.4762	88.47877389
UIJ70166.1	spermidine_N1-acetyltransferase_(plasmid)_[Bacillus_cereus]	CPTF_U	220746.3333	192202.7635	87.06951574
UIJ70166.1	spermidine_N1-acetyltransferase_(plasmid)_[Bacillus_cereus]	CPTF_metals_mix	0	0	0
UIJ70166.1	spermidine_N1-acetyltransferase_(plasmid)_[Bacillus_cereus]	CPTF_zcontrol	259632.3333	224969.8193	86.64938469
UIJ70167.1	5'-nucleotidase,_lipoprotein_e(P4)_family_(plasmid)_[Bacillus_cereus]	CPTF_Al	171169	296473.4047	173.2050808
UIJ70167.1	5'-nucleotidase,_lipoprotein_e(P4)_family_(plasmid)_[Bacillus_cereus]	CPTF_Cd	176097.3333	305009.5284	173.2050808
UIJ70167.1	5'-nucleotidase,_lipoprotein_e(P4)_family_(plasmid)_[Bacillus_cereus]	CPTF_Co	0	0	0
UIJ70167.1	5'-nucleotidase,_lipoprotein_e(P4)_family_(plasmid)_[Bacillus_cereus]	CPTF_Cu	0	0	0
UIJ70167.1	5'-nucleotidase,_lipoprotein_e(P4)_family_(plasmid)_[Bacillus_cereus]	CPTF_Fe	0	0	0
UIJ70167.1	5'-nucleotidase,_lipoprotein_e(P4)_family_(plasmid)_[Bacillus_cereus]	CPTF_Mn	0	0	0
UIJ70167.1	5'-nucleotidase,_lipoprotein_e(P4)_family_(plasmid)_[Bacillus_cereus]	CPTF_Ni	105591	182888.9768	173.2050808
UIJ70167.1	5'-nucleotidase,_lipoprotein_e(P4)_family_(plasmid)_[Bacillus_cereus]	CPTF_U	0	0	0
UIJ70167.1	5'-nucleotidase,_lipoprotein_e(P4)_family_(plasmid)_[Bacillus_cereus]	CPTF_metals_mix	226269.2333	201464.5862	89.03755197
UIJ70167.1	5'-nucleotidase,_lipoprotein_e(P4)_family_(plasmid)_[Bacillus_cereus]	CPTF_zcontrol	0	0	0
UIJ70168.1	hypothetical_protein_LW858_33620_(plasmid)_[Bacillus_cereus]	CPTF_Al	0	0	0
UIJ70168.1	hypothetical_protein_LW858_33620_(plasmid)_[Bacillus_cereus]	CPTF_Cd	0	0	0
UIJ70168.1	hypothetical_protein_LW858_33620_(plasmid)_[Bacillus_cereus]	CPTF_Co	0	0	0
UIJ70168.1	hypothetical_protein_LW858_33620_(plasmid)_[Bacillus_cereus]	CPTF_Cu	0	0	0
UIJ70168.1	hypothetical_protein_LW858_33620_(plasmid)_[Bacillus_cereus]	CPTF_Fe	0	0	0
UIJ70168.1	hypothetical_protein_LW858_33620_(plasmid)_[Bacillus_cereus]	CPTF_Mn	0	0	0
UIJ70168.1	hypothetical_protein_LW858_33620_(plasmid)_[Bacillus_cereus]	CPTF_Ni	0	0	0
UIJ70168.1	hypothetical_protein_LW858_33620_(plasmid)_[Bacillus_cereus]	CPTF_U	3463333.333	552931.5811	15.9653007
UIJ70168.1	hypothetical_protein_LW858_33620_(plasmid)_[Bacillus_cereus]	CPTF_metals_mix	282904.3333	49625.79618	17.54154685
UIJ70168.1	hypothetical_protein_LW858_33620_(plasmid)_[Bacillus_cereus]	CPTF_zcontrol	0	0	0
UIJ70184.1	ParM/StbA_family_protein_(plasmid)_[Bacillus_cereus]	CPTF_Al	6161133	121671.6772	1.974826337
UIJ70184.1	ParM/StbA_family_protein_(plasmid)_[Bacillus_cereus]	CPTF_Cd	6616132.167	264995.0146	4.005285987
UIJ70184.1	ParM/StbA_family_protein_(plasmid)_[Bacillus_cereus]	CPTF_Co	5064329	54791.88213	1.081917903
UIJ70184.1	ParM/StbA_family_protein_(plasmid)_[Bacillus_cereus]	CPTF_Cu	5768636.2	727071.4743	12.60387116
UIJ70184.1	ParM/StbA_family_protein_(plasmid)_[Bacillus_cereus]	CPTF_Fe	6166224.333	31621.09445	0.512811288
UIJ70184.1	ParM/StbA_family_protein_(plasmid)_[Bacillus_cereus]	CPTF_Mn	6525412.667	125628.229	1.925215085
UIJ70184.1	ParM/StbA_family_protein_(plasmid)_[Bacillus_cereus]	CPTF_Ni	5075480	421635.3415	8.307299831
UIJ70184.1	ParM/StbA_family_protein_(plasmid)_[Bacillus_cereus]	CPTF_U	5519945.933	360169.1577	6.52486749
UIJ70184.1	ParM/StbA_family_protein_(plasmid)_[Bacillus_cereus]	CPTF_metals_mix	5487244.6	338631.239	6.171243742
UIJ70184.1	ParM/StbA_family_protein_(plasmid)_[Bacillus_cereus]	CPTF_zcontrol	5643750	209323.7491	3.708947935
UIJ70187.1	AimR_family_lysis-lysogeny_pheromone_receptor_(plasmid)_[Bacillus_cereus]	CPTF_Al	404946.6667	18084.69304	4.465944414

UIJ70187.1	AimR_family_lysis-lysogeny_pheromone_receptor_(plasmid)_[Bacillus_cereus]	CPTF_Cd	392581.3333	72874.7076	18.56295789
UIJ70187.1	AimR_family_lysis-lysogeny_pheromone_receptor_(plasmid)_[Bacillus_cereus]	CPTF_Co	397584	120921.8946	30.41417527
UIJ70187.1	AimR_family_lysis-lysogeny_pheromone_receptor_(plasmid)_[Bacillus_cereus]	CPTF_Cu	267162	131729.4471	49.30695498
UIJ70187.1	AimR_family_lysis-lysogeny_pheromone_receptor_(plasmid)_[Bacillus_cereus]	CPTF_Fe	383376.3333	24064.50451	6.276992714
UIJ70187.1	AimR_family_lysis-lysogeny_pheromone_receptor_(plasmid)_[Bacillus_cereus]	CPTF_Mn	329059.6667	64949.26968	19.73783975
UIJ70187.1	AimR_family_lysis-lysogeny_pheromone_receptor_(plasmid)_[Bacillus_cereus]	CPTF_Ni	392045.3333	64155.95499	16.36442256
UIJ70187.1	AimR_family_lysis-lysogeny_pheromone_receptor_(plasmid)_[Bacillus_cereus]	CPTF_U	388549	26115.73717	6.721349732
UIJ70187.1	AimR_family_lysis-lysogeny_pheromone_receptor_(plasmid)_[Bacillus_cereus]	CPTF_metals_mix	36830.66667	63792.58594	173.2050808
UIJ70187.1	AimR_family_lysis-lysogeny_pheromone_receptor_(plasmid)_[Bacillus_cereus]	CPTF_zcontrol	358379	14476.53318	4.039447954
UIJ70189.1	helix-turn-helix_domain-containing_protein_(plasmid)_[Bacillus_cereus]	CPTF_Al	41685.1	41657.67713	99.93421422
UIJ70189.1	helix-turn-helix_domain-containing_protein_(plasmid)_[Bacillus_cereus]	CPTF_Cd	22244.11	31187.35708	140.2050119
UIJ70189.1	helix-turn-helix_domain-containing_protein_(plasmid)_[Bacillus_cereus]	CPTF_Co	23894.46667	25353.08494	106.1044186
UIJ70189.1	helix-turn-helix_domain-containing_protein_(plasmid)_[Bacillus_cereus]	CPTF_Cu	25432.03333	7128.507112	28.02963891
UIJ70189.1	helix-turn-helix_domain-containing_protein_(plasmid)_[Bacillus_cereus]	CPTF_Fe	14211.26667	12872.11212	90.57681079
UIJ70189.1	helix-turn-helix_domain-containing_protein_(plasmid)_[Bacillus_cereus]	CPTF_Mn	5225	9049.96547	173.2050808
UIJ70189.1	helix-turn-helix_domain-containing_protein_(plasmid)_[Bacillus_cereus]	CPTF_Ni	23248.3	2589.235171	11.1373097
UIJ70189.1	helix-turn-helix_domain-containing_protein_(plasmid)_[Bacillus_cereus]	CPTF_U	0	0	0
UIJ70189.1	helix-turn-helix_domain-containing_protein_(plasmid)_[Bacillus_cereus]	CPTF_metals_mix	30016.13333	26738.62131	89.080832
UIJ70189.1	helix-turn-helix_domain-containing_protein_(plasmid)_[Bacillus_cereus]	CPTF_zcontrol	15822.53333	13814.34137	87.30802509
UIJ70191.1	Cys-Gln_thioester_bond-forming_surface_protein_(plasmid)_[Bacillus_cereus]	CPTF_Al	527479.3333	362647.2117	68.75098015
UIJ70191.1	Cys-Gln_thioester_bond-forming_surface_protein_(plasmid)_[Bacillus_cereus]	CPTF_Cd	120717.6667	209089.132	173.2050808
UIJ70191.1	Cys-Gln_thioester_bond-forming_surface_protein_(plasmid)_[Bacillus_cereus]	CPTF_Co	0	0	0
UIJ70191.1	Cys-Gln_thioester_bond-forming_surface_protein_(plasmid)_[Bacillus_cereus]	CPTF_Cu	0	0	0
UIJ70191.1	Cys-Gln_thioester_bond-forming_surface_protein_(plasmid)_[Bacillus_cereus]	CPTF_Fe	239281.3333	258837.2979	108.1727915
UIJ70191.1	Cys-Gln_thioester_bond-forming_surface_protein_(plasmid)_[Bacillus_cereus]	CPTF_Mn	0	0	0
UIJ70191.1	Cys-Gln_thioester_bond-forming_surface_protein_(plasmid)_[Bacillus_cereus]	CPTF_Ni	0	0	0
UIJ70191.1	Cys-Gln_thioester_bond-forming_surface_protein_(plasmid)_[Bacillus_cereus]	CPTF_U	161585	142061.8846	87.91774272
UIJ70191.1	Cys-Gln_thioester_bond-forming_surface_protein_(plasmid)_[Bacillus_cereus]	CPTF_metals_mix	23792.36667	41209.5879	173.2050808
UIJ70191.1	Cys-Gln_thioester_bond-forming_surface_protein_(plasmid)_[Bacillus_cereus]	CPTF_zcontrol	0	0	0
UIJ70202.1	hypothetical_protein_LW858_33665_(plasmid)_[Bacillus_cereus]	CPTF_Al	990111.5667	236064.4211	23.84220416
UIJ70202.1	hypothetical_protein_LW858_33665_(plasmid)_[Bacillus_cereus]	CPTF_Cd	968041.1333	100558.1097	10.38779306
UIJ70202.1	hypothetical_protein_LW858_33665_(plasmid)_[Bacillus_cereus]	CPTF_Co	753270.3	147050.5357	19.52161604
UIJ70202.1	hypothetical_protein_LW858_33665_(plasmid)_[Bacillus_cereus]	CPTF_Cu	828533.2	447662.2546	54.03069601
UIJ70202.1	hypothetical_protein_LW858_33665_(plasmid)_[Bacillus_cereus]	CPTF_Fe	949627.0333	216799.9505	22.8300104
UIJ70202.1	hypothetical_protein_LW858_33665_(plasmid)_[Bacillus_cereus]	CPTF_Mn	710287.3333	451789.9599	63.60664744
UIJ70202.1	hypothetical_protein_LW858_33665_(plasmid)_[Bacillus_cereus]	CPTF_Ni	612336.2667	284860.9154	46.52034036
UIJ70202.1	hypothetical_protein_LW858_33665_(plasmid)_[Bacillus_cereus]	CPTF_U	685702.6667	405713.2628	59.16752006
UIJ70202.1	hypothetical_protein_LW858_33665_(plasmid)_[Bacillus_cereus]	CPTF_metals_mix	1156684.733	85137.79784	7.36050156
UIJ70202.1	hypothetical_protein_LW858_33665_(plasmid)_[Bacillus_cereus]	CPTF_zcontrol	567570.6667	287151.092	50.59301139
UIJ70204.1	replication/maintenance_protein_RepL_(plasmid)_[Bacillus_cereus]	CPTF_Al	0	0	0
UIJ70204.1	replication/maintenance_protein_RepL_(plasmid)_[Bacillus_cereus]	CPTF_Cd	0	0	0
UIJ70204.1	replication/maintenance_protein_RepL_(plasmid)_[Bacillus_cereus]	CPTF_Co	0	0	0
UIJ70204.1	replication/maintenance_protein_RepL_(plasmid)_[Bacillus_cereus]	CPTF_Cu	0	0	0
UIJ70204.1	replication/maintenance_protein_RepL_(plasmid)_[Bacillus_cereus]	CPTF_Fe	59319	102743.5219	173.2050808
UIJ70204.1	replication/maintenance_protein_RepL_(plasmid)_[Bacillus_cereus]	CPTF_Mn	0	0	0
UIJ70204.1	replication/maintenance_protein_RepL_(plasmid)_[Bacillus_cereus]	CPTF_Ni	0	0	0
UIJ70204.1	replication/maintenance_protein_RepL_(plasmid)_[Bacillus_cereus]	CPTF_U	0	0	0
UIJ70204.1	replication/maintenance_protein_RepL_(plasmid)_[Bacillus_cereus]	CPTF_metals_mix	41655.66667	72149.73109	173.2050808
UIJ70204.1	replication/maintenance_protein_RepL_(plasmid)_[Bacillus_cereus]	CPTF_zcontrol	0	0	0
UIJ70209.1	replication/maintenance_protein_RepL_(plasmid)_[Bacillus_cereus]	CPTF_Al	58014.93333	22788.19186	39.27987252
UIJ70209.1	replication/maintenance_protein_RepL_(plasmid)_[Bacillus_cereus]	CPTF_Cd	41338.13333	38368.65595	92.81661473
UIJ70209.1	replication/maintenance_protein_RepL_(plasmid)_[Bacillus_cereus]	CPTF_Co	54638	94635.79202	173.2050808
UIJ70209.1	replication/maintenance_protein_RepL_(plasmid)_[Bacillus_cereus]	CPTF_Cu	135118.0333	61026.915	45.16563296
UIJ70209.1	replication/maintenance_protein_RepL_(plasmid)_[Bacillus_cereus]	CPTF_Fe	38417.66667	66541.35057	173.2050808
UIJ70209.1	replication/maintenance_protein_RepL_(plasmid)_[Bacillus_cereus]	CPTF_Mn	0	0	0
UIJ70209.1	replication/maintenance_protein_RepL_(plasmid)_[Bacillus_cereus]	CPTF_Ni	48790.83333	46160.44982	94.60885718
UIJ70209.1	replication/maintenance_protein_RepL_(plasmid)_[Bacillus_cereus]	CPTF_U	16080.46667	27852.18528	173.2050808
UIJ70209.1	replication/maintenance_protein_RepL_(plasmid)_[Bacillus_cereus]	CPTF_metals_mix	89770.13333	1128.349389	1.25693184
UIJ70209.1	replication/maintenance_protein_RepL_(plasmid)_[Bacillus_cereus]	CPTF_zcontrol	0	0	0
UIJ70213.1	helix-turn-helix_domain-containing_protein_(plasmid)_[Bacillus_cereus]	CPTF_Al	722750.8667	73173.89863	10.12435986
UIJ70213.1	helix-turn-helix_domain-containing_protein_(plasmid)_[Bacillus_cereus]	CPTF_Cd	507692.1667	241535.4836	47.57518423

UIJ70213.1	helix-turn-helix_domain-containing_protein_(plasmid)_[Bacillus_cereus]	CPTF_Co	875929.2333	30133.5835	3.440184704
UIJ70213.1	helix-turn-helix_domain-containing_protein_(plasmid)_[Bacillus_cereus]	CPTF_Cu	910470.1	79260.16492	8.705411075
UIJ70213.1	helix-turn-helix_domain-containing_protein_(plasmid)_[Bacillus_cereus]	CPTF_Fe	787811.4	28728.01627	3.646560112
UIJ70213.1	helix-turn-helix_domain-containing_protein_(plasmid)_[Bacillus_cereus]	CPTF_Mn	739383.6667	35931.61024	4.859670542
UIJ70213.1	helix-turn-helix_domain-containing_protein_(plasmid)_[Bacillus_cereus]	CPTF_Ni	689937.2	140606.9129	20.37966831
UIJ70213.1	helix-turn-helix_domain-containing_protein_(plasmid)_[Bacillus_cereus]	CPTF_U	657822	159696.2854	24.27651939
UIJ70213.1	helix-turn-helix_domain-containing_protein_(plasmid)_[Bacillus_cereus]	CPTF_metals_mix	997881.1333	90480.91344	9.067303752
UIJ70213.1	helix-turn-helix_domain-containing_protein_(plasmid)_[Bacillus_cereus]	CPTF_zcontrol	834918.8333	47995.10922	5.748476056
UIJ70214.1	plasmid_recombination_protein_(plasmid)_[Bacillus_cereus]	CPTF_Al	484399.4667	133760.6968	27.61371679
UIJ70214.1	plasmid_recombination_protein_(plasmid)_[Bacillus_cereus]	CPTF_Cd	865885.3	433268.6315	50.0376472
UIJ70214.1	plasmid_recombination_protein_(plasmid)_[Bacillus_cereus]	CPTF_Co	1288684.8	608477.6297	47.21694783
UIJ70214.1	plasmid_recombination_protein_(plasmid)_[Bacillus_cereus]	CPTF_Cu	1879290.633	555633.3265	29.56612015
UIJ70214.1	plasmid_recombination_protein_(plasmid)_[Bacillus_cereus]	CPTF_Fe	587663.7667	291841.1576	49.6612475
UIJ70214.1	plasmid_recombination_protein_(plasmid)_[Bacillus_cereus]	CPTF_Mn	700296.1667	135815.6577	19.39403129
UIJ70214.1	plasmid_recombination_protein_(plasmid)_[Bacillus_cereus]	CPTF_Ni	965880.6333	280349.6447	29.02528894
UIJ70214.1	plasmid_recombination_protein_(plasmid)_[Bacillus_cereus]	CPTF_U	560048.2667	443120.009	79.12175349
UIJ70214.1	plasmid_recombination_protein_(plasmid)_[Bacillus_cereus]	CPTF_metals_mix	2011293.267	395332.3368	19.65562871
UIJ70214.1	plasmid_recombination_protein_(plasmid)_[Bacillus_cereus]	CPTF_zcontrol	707074.2667	642279.9559	90.83627932
UIJ70217.1	helix-turn-helix_transcriptional_regulator_(plasmid)_[Bacillus_cereus]	CPTF_Al	247143	8104.737565	3.279371686
UIJ70217.1	helix-turn-helix_transcriptional_regulator_(plasmid)_[Bacillus_cereus]	CPTF_Cd	201157	24540.79469	12.19982138
UIJ70217.1	helix-turn-helix_transcriptional_regulator_(plasmid)_[Bacillus_cereus]	CPTF_Co	203253	58237.92049	28.65292049
UIJ70217.1	helix-turn-helix_transcriptional_regulator_(plasmid)_[Bacillus_cereus]	CPTF_Cu	204201	25255.25664	12.3678418
UIJ70217.1	helix-turn-helix_transcriptional_regulator_(plasmid)_[Bacillus_cereus]	CPTF_Fe	227223.6667	99323.85255	43.71193107
UIJ70217.1	helix-turn-helix_transcriptional_regulator_(plasmid)_[Bacillus_cereus]	CPTF_Mn	140991.1667	97929.08113	69.45760039
UIJ70217.1	helix-turn-helix_transcriptional_regulator_(plasmid)_[Bacillus_cereus]	CPTF_Ni	58716.56667	62599.66017	106.6132843
UIJ70217.1	helix-turn-helix_transcriptional_regulator_(plasmid)_[Bacillus_cereus]	CPTF_U	196631	51095.53729	25.9854943
UIJ70217.1	helix-turn-helix_transcriptional_regulator_(plasmid)_[Bacillus_cereus]	CPTF_metals_mix	93016	83674.46421	89.95706568
UIJ70217.1	helix-turn-helix_transcriptional_regulator_(plasmid)_[Bacillus_cereus]	CPTF_zcontrol	196928	12242.56824	6.216773763
UIJ70219.1	hypothetical_protein_LW858_33720_(plasmid)_[Bacillus_cereus]	CPTF_Al	356259.6667	88024.25544	24.70789249
UIJ70219.1	hypothetical_protein_LW858_33720_(plasmid)_[Bacillus_cereus]	CPTF_Cd	335397.8667	120197.802	35.83737821
UIJ70219.1	hypothetical_protein_LW858_33720_(plasmid)_[Bacillus_cereus]	CPTF_Co	584270.7667	19748.18588	3.379971582
UIJ70219.1	hypothetical_protein_LW858_33720_(plasmid)_[Bacillus_cereus]	CPTF_Cu	496249.4	66621.52201	13.42500807
UIJ70219.1	hypothetical_protein_LW858_33720_(plasmid)_[Bacillus_cereus]	CPTF_Fe	220800.6333	50322.13612	22.79075715
UIJ70219.1	hypothetical_protein_LW858_33720_(plasmid)_[Bacillus_cereus]	CPTF_Mn	296791.6333	185911.7522	62.64049633
UIJ70219.1	hypothetical_protein_LW858_33720_(plasmid)_[Bacillus_cereus]	CPTF_Ni	274980.9333	174027.7246	63.28719686
UIJ70219.1	hypothetical_protein_LW858_33720_(plasmid)_[Bacillus_cereus]	CPTF_U	237282	205542.5402	86.62373895
UIJ70219.1	hypothetical_protein_LW858_33720_(plasmid)_[Bacillus_cereus]	CPTF_metals_mix	341824.5	38010.27328	11.11982122
UIJ70219.1	hypothetical_protein_LW858_33720_(plasmid)_[Bacillus_cereus]	CPTF_zcontrol	384395.6667	335776.127	87.35169413
