# Supplementary material for: Mixed heavy metal stress induces global iron starvation response
Source: ISME J. 2022 Dec 26;17(3):382–92. doi: 10.1038/s41396-022-01351-3 (PMC9938188; doi:10.1038/s41396-022-01351-3)
Supplement: Supplementary file 3 — Table S7 [file 41396_2022_1351_MOESM3_ESM.pdf]

Genbank	Protein (NCBI)	COMM	U	Al	Mn	Fe	Co	Cu	Cd	Ni
UIJ70168.1	Hypothetical protein lw858 33620 (plasmid)									
UIJ67978.1	Transglycosylase									
UIJ69423.1	Acyl carrier protein									
UIJ66240.1	Translation initiation factor if-1									
UIJ66811.1	Ectonucleotide pyrophosphatase/phosphodiesterase									
UIJ64381.1	Sdr family oxidoreductase									
UIJ68572.1	Isochorismatase family protein									
UIJ69446.1	Flavodoxin-dependent (e)-4-hydroxy-3-methylbut-2-enyl-diphosphate synthase									
UIJ65389.1	Hypothetical protein lw858 20935									
UIJ65324.1	Trna threonylcarbamoyladenosine dehydratase									
UIJ65937.1	Ferritin									
UIJ69189.1	Copper homeostasis protein cutc									
UIJ67731.1	Duf3913 family protein									
UIJ64815.1	Methionyl-trna formyltransferase									
UIJ65126.1	Elongation factor p									
UIJ64819.1	Dna-directed rna polymerase subunit omega									
UIJ66239.1	50s ribosomal protein l36									
UIJ65025.1	Fixh family protein									
UIJ70191.1	Cys-gln thioester bond-forming surface protein (plasmid)									
UIJ68566.1	Ykvs family protein									
UIJ66464.1	Ywhd family protein									
UIJ64511.1	Flavodoxin									
UIJ64690.1	Iron-hydroxamate abc transporter substrate-binding protein									
UIJ64802.1	Phosphate acyltransferase plsx									
UIJ66462.1	4-oxalocrotonate tautomerase									
UIJ65464.1	Hypothetical protein lw858 21360									
UIJ66423.1	Glycosyltransferase family 4 protein									
UIJ64881.1	Yugn-like family protein									
UIJ66035.1	Degv family protein									
UIJ67343.1	Hypothetical protein lw858 03415									
UIJ69267.1	Ld-carboxypeptidase									
UIJ65424.1	Glutamate racemase									
UIJ67675.1	(fe-s)-binding protein									
UIJ64895.1	Pyridoxamine 5'-phosphate oxidase family protein									
UIJ65521.1	Acetyl-coa carboxylase, carboxyltransferase subunit beta									
UIJ66957.1	Peptide mfs transporter									
UIJ64909.1	Gapa-binding peptide sr1p									
UIJ66132.1	Trna (adenosine(37)-n6)-threonylcarbamoyltransferase complex dimerization subunit type 1 tsab									
UIJ68520.1	Yoku family protein									
UIJ66828.1	Cadmium-translocating p-type atpase									
UIJ69534.1	Hypothetical protein lw858 30370 (plasmid)									
UIJ66218.1	Ybbr-like domain-containing protein									
UIJ65729.1	Yegs/rv2252/bmru family lipid kinase									
UIJ67003.1	D-ribose pyranase									
UIJ67412.1	Duf6359 domain-containing protein									
UIJ69494.1	Duf3981 domain-containing protein									
UIJ69045.1	Polysaccharide deacetylase									
UIJ64950.1	Aminotransferase a									
UIJ64425.1	Fmn-binding negative transcriptional regulator									
UIJ66334.1	16s rrna (cytidine(1402)-2'-o)-methyltransferase									
UIJ65000.1	Duf1002 domain-containing protein									
UIJ64683.1	Copper chaperone copz									
UIJ68437.1	Aspartate--trna(asn) ligase									
UIJ68322.1	Chaperone csaa									
UIJ67975.1	Response regulator									
UIJ65452.1	Tetr family transcriptional regulator									
UIJ65637.1	Cyclic pyranopterin monophosphate synthase moac									
UIJ66367.1	Trna uridine-5-carboxymethylaminomethyl(34) synthesis gtpase mnme									
UIJ66516.1	Ribose 5-phosphate isomerase b									
UIJ64422.1	Ydiu family protein									
UIJ64524.1	3-oxoacyl-acp reductase fabg									
UIJ66375.1	Duf951 domain-containing protein									
UIJ65239.1	50s ribosomal protein l11 methyltransferase									
UIJ68298.1	Nadph dehydrogenase nama									
UIJ66001.1	Pyrophosphatase ppax									
UIJ66286.1	2-c-methyl-d-erythritol 2,4-cyclodiphosphate synthase									
UIJ68570.1	Isochorismate synthase dhbc									
UIJ64868.1	Duf177 domain-containing protein									
UIJ65340.1	Trna preq1(34) s-adenosylmethionine ribosyltransferase-isomerase quea									
UIJ65097.1	Glycerophosphodiester phosphodiesterase									
UIJ65355.1	Prephenate dehydratase									
UIJ69000.1	Cytosolic protein									
UIJ65137.1	Lipoate--protein ligase family protein									
UIJ65422.1	Ribonuclease ph									
UIJ64720.1	Dna mismatch repair endonuclease mutl									
UIJ64864.1	Bacillithiol biosynthesis cysteine-adding enzyme bshc									
UIJ67546.1	Aaa family atpase									
UIJ68095.1	Oxaloacetate-decarboxylating malate dehydrogenase									
UIJ67411.1	Duf4026 domain-containing protein									
UIJ64612.1	Alkene reductase									
UIJ68571.1	(2,3-dihydroxybenzoyl)adenylate synthase									
UIJ68511.1	Yuei family protein									
UIJ66556.1	Cpsd/capb family tyrosine-protein kinase									
UIJ65872.1	Methionine abc transporter atp-binding protein									
UIJ65219.1	Trna (adenine(22)-n(1))-methyltransferase trmk									
UIJ64759.1	Bifunctional riboflavin kinase/fad synthetase									
UIJ65175.1	Helix-turn-helix domain-containing protein									
UIJ66288.1	Pin/tram domain-containing protein									
UIJ67318.1	Dihydroxyacetone kinase subunit dhak									
UIJ66706.1	M20 peptidase aminoacylase family protein									
UIJ65204.1	Penicillin-binding protein 2									
UIJ65626.1	Srpbcc domain-containing protein									
UIJ64893.1	Peptidyl-prolyl cis-trans isomerase									
UIJ64998.1	Gnat family n-acetyltransferase ribt									
UIJ67893.1	Biotin--[acetyl-coa-carboxylase] ligase									
UIJ68071.1	Peptidase e									
UIJ65445.1	Succinate dehydrogenase cytochrome b558									
UIJ64682.1	Heavy metal translocating p-type atpase									

log2-fold change
6
n.s.
-6

UIJ67285.1	Dna (cytosine-5-)-methyltransferase
UIJ65786.1	Aminotransferase
UIJ65584.1	Gfo/idh/moca family oxidoreductase
UIJ68510.1	Aldehyde dehydrogenase family protein
UIJ67895.1	3-methyl-2-oxobutanoate hydroxymethyltransferase
UIJ66413.1	Yfho family protein
UIJ69840.1	Hypothetical protein lw858 31855 (plasmid)
UIJ67720.1	Abortive phage infection protein
UIJ65300.1	Yrrs family protein
UIJ69099.1	Hypothetical protein lw858 13180
UIJ64789.1	Signal peptidase i
UIJ66515.1	Low molecular weight protein arginine phosphatase
UIJ65712.1	Yitt family protein
UIJ64667.1	Trna (adenosine(37)-n6)-dimethylallyltransferase miaa
UIJ68081.1	Duf445 domain-containing protein
UIJ65998.1	Thioredoxin-disulfide reductase
UIJ67416.1	Tetr/acrr family transcriptional regulator
UIJ65608.1	Thioredoxin family protein
UIJ67885.1	Zinc metallopeptidase
UIJ64894.1	Phoh family protein
UIJ68527.1	Sporulation protein
UIJ70204.1	Replication/maintenance protein repl (plasmid)
UIJ65045.1	Bifunctional diaminohydroxyphosphoribosylaminopyrimidine deaminase/5-amino-6-(5-phosphoribosylamino)uracil reductase ribd
UIJ64665.1	Tyrosine-type recombinase/integrase
UIJ64338.1	Oxidoreductase
UIJ69081.1	Heme-degrading monooxygenase hmoa
UIJ64738.1	Insulinase family protein
UIJ68630.1	Duf5519 family protein
UIJ66504.1	Type b 50s ribosomal protein l31
UIJ69885.1	Hypothetical protein lw858 31605 (plasmid)
UIJ69571.1	Hu family dna-binding protein (plasmid)
UIJ66385.1	Cell wall metabolism dna-binding response regulator walr
UIJ65092.1	Butyrate kinase
UIJ68491.1	Alcohol dehydrogenase adhp
UIJ65018.1	Marr family transcriptional regulator
UIJ65705.1	S-ribosylhomocysteine lyase luxs
UIJ67007.1	Fructose-6-phosphate aldolase
UIJ65247.1	Elongation factor 4
UIJ67785.1	Diaminopimelate decarboxylase
UIJ67901.1	Aspartate transaminase aspb
UIJ68465.1	Thymidylate synthase
UIJ67915.1	Class i sam-dependent rna methyltransferase
UIJ69961.1	Duf2089 domain-containing protein (plasmid)
UIJ67337.1	Heavy metal-binding domain-containing protein
UIJ65365.1	50s ribosomal protein l21
UIJ69838.1	Rpn family recombination-promoting nuclease/putative transposase (plasmid)
UIJ65589.1	Catabolite control protein a
UIJ67011.1	(r,r)-butanediol dehydrogenase
UIJ66709.1	Peroxiredoxin
UIJ65974.1	Triose-phosphate isomerase
UIJ65250.1	30s ribosomal protein s20
UIJ65296.1	O-acetylserine dependent cystathionine beta-synthase
UIJ65769.1	Cold-shock protein cspd
UIJ67816.1	Duf47 domain-containing protein
UIJ65976.1	Type i glyceraldehyde-3-phosphate dehydrogenase
UIJ66708.1	Alkyl hydroperoxide reductase subunit f
UIJ65503.1	Glyceraldehyde-3-phosphate dehydrogenase
UIJ66136.1	Rna-binding transcriptional accessory protein
UIJ65067.1	N-acetyl-gamma-glutamyl-phosphate reductase
UIJ66128.1	Redox-sensing transcriptional repressor rex
UIJ64800.1	3-oxoacyl-[acyl-carrier-protein] reductase
UIJ65485.1	Phenylalanine--trna ligase subunit alpha
UIJ65676.1	Phosphoenolpyruvate carboxykinase (atp)
UIJ65448.1	Thioredoxin
UIJ65089.1	3-methyl-2-oxobutanoate dehydrogenase subunit beta
UIJ65297.1	5'-methylthioadenosine/s-adenosylhomocysteine nucleosidase
UIJ65800.1	Hotdog fold thioesterase
UIJ65779.1	L-lactate dehydrogenase
UIJ66554.1	Rod shape-determining protein
UIJ68467.1	Penicillin-binding protein 3
UIJ66351.1	Duf3797 domain-containing protein
UIJ67406.1	Heme-degrading monooxygenase hmob
UIJ69452.1	Nad-dependent malic enzyme
UIJ64517.1	Gnat family n-acetyltransferase
UIJ64570.1	Trypsin-like peptidase domain-containing protein
UIJ67203.1	Nadp-dependent glyceraldehyde-3-phosphate dehydrogenase
UIJ65877.1	Arsenate reductase family protein
UIJ67392.1	Ytxh domain-containing protein
UIJ66398.1	Udp-glucose 4-epimerase gale
UIJ64954.1	Extracellular solute-binding protein
UIJ66311.1	S1 domain-containing rna-binding protein
UIJ65519.1	6-phosphofructokinase
UIJ66616.1	Spfh domain-containing protein
UIJ67099.1	K(+)-transporting atpase subunit c
UIJ68939.1	Manganese-dependent inorganic pyrophosphatase
UIJ67470.1	Rna chaperone/antiterminator cspa
UIJ68409.1	Iron-sulfur cluster repair di-iron protein
UIJ65028.1	Transcriptional repressor
UIJ69506.1	Peptide chain release factor 1
UIJ64795.1	Signal recognition particle protein
UIJ67409.1	Ferrochelatase
UIJ68609.1	Protoporphyrinogen oxidase
UIJ65326.1	Histidine--trna ligase
UIJ65064.1	Acetylornithine transaminase
UIJ67394.1	Abc transporter atp-binding protein ecsa
UIJ70084.1	Duf2325 domain-containing protein (plasmid)
UIJ65651.1	Leucine--trna ligase
UIJ65526.1	Bifunctional oligoribonuclease/pap phosphatase nrna
UIJ66257.1	50s ribosomal protein l22
UIJ69954.1	Efflux rnd transporter periplasmic adaptor subunit (plasmid)

UIJ64763.1	Translation initiation factor if-2
UIJ66283.1	Cysteine--trna ligase
UIJ66832.1	Gtp-binding protein
UIJ70137.1	Hypothetical protein lw858 33040 (plasmid)
UIJ64779.1	Atp-dependent protease atpase subunit hslu
UIJ66291.1	Atp-dependent protease atp-binding subunit clpc
UIJ66620.1	Nad-dependent dna ligase liga
UIJ66512.1	Flavin reductase family protein
UIJ67888.1	Dihydrodipicolinate reductase
UIJ67918.1	Carboxypeptidase
UIJ65512.1	Malate dehydrogenase
UIJ65048.1	6,7-dimethyl-8-ribityllumazine synthase
UIJ64890.1	Pyruvate carboxylase
UIJ66148.1	D-alanine--d-alanine ligase
UIJ67781.1	Glycerate dehydrogenase
UIJ65242.1	Nucleotide exchange factor grpe
UIJ67856.1	30s ribosomal protein s1
UIJ64311.1	Lactonase family protein
UIJ65823.1	Iron-sulfur cluster assembly accessory protein
UIJ64783.1	Type i dna topoisomerase
UIJ65518.1	Pyruvate kinase
UIJ64832.1	Carbamoyl-phosphate synthase large subunit
UIJ65967.1	Ribonuclease r
UIJ69527.1	Type ii toxin-antitoxin system death-on-curing family toxin (plasmid)
UIJ65675.1	Methionine adenosyltransferase
UIJ64794.1	30s ribosomal protein s16
UIJ66305.1	Redox-regulated molecular chaperone hslo
UIJ65554.1	Thiol peroxidase
UIJ64988.1	Phosphocarrier protein hpr
UIJ64366.1	Hydroxylamine reductase
UIJ66883.1	Glutamate synthase
UIJ66244.1	50s ribosomal protein l15
UIJ68731.1	Glyoxalase/bleomycin resistance/dioxygenase family protein
UIJ65058.1	Bifunctional 2',3'-cyclic-nucleotide 2'-phosphodiesterase/3'-nucleotidase
UIJ66880.1	Duf402 domain-containing protein
UIJ68402.1	Nitrate transporter nark
UIJ66592.1	Efflux rnd transporter periplasmic adaptor subunit
UIJ69055.1	Prephenate dehydrogenase
UIJ67661.1	Spermidine/putrescine abc transporter substrate-binding protein potd
UIJ67653.1	Biofilm matrix protein caly
UIJ64616.1	Urocanate hydratase
UIJ65776.1	1,4-alpha-glucan branching protein glgb
UIJ67788.1	Sulfate adenylyltransferase
UIJ66254.1	50s ribosomal protein l29
UIJ68476.1	Sco family protein
UIJ69501.1	Gmp reductase
UIJ67789.1	Adenylyl-sulfate kinase
UIJ68800.1	Ester cyclase
UIJ65773.1	Glycogen phosphorylase
UIJ69464.1	Glycogen synthase glga
UIJ65926.1	Neutral protease nprb
UIJ64775.1	Ump kinase
UIJ68393.1	Nitrate reductase subunit beta
UIJ68412.1	Arylamine n-acetyltransferase
UIJ65936.1	Mazg-like family protein
UIJ67515.1	Beta-ketoacyl-acp synthase ii
UIJ65511.1	Maoc family dehydratase n-terminal domain-containing protein
UIJ68109.1	Udp-galactose-lipid carrier transferase
UIJ66965.1	L-threonine 3-dehydrogenase
UIJ67098.1	Potassium-transporting atpase subunit kdpa
UIJ65860.1	Lipoyl synthase
UIJ69497.1	Septation regulator spovg
UIJ64423.1	Hypothetical protein lw858 15595
UIJ67914.1	Cell division regulator gpsb
UIJ67790.1	Nitrite/sulfite reductase
UIJ67656.1	Immune inhibitor a
UIJ68231.1	Threonine synthase
UIJ66994.1	Duf4352 domain-containing protein
UIJ69522.1	Caly family protein (plasmid)
UIJ66530.1	Duf975 family protein
UIJ67791.1	Duf3906 family protein
UIJ68796.1	Beta-lactamase family protein
UIJ69025.1	Fmnh2-dependent alkanesulfonate monooxygenase
UIJ67923.1	Dynamin family protein
UIJ68212.1	Duf6376 family protein
UIJ68471.1	Twin-arginine translocase tata/tate family subunit
UIJ65306.1	U32 family peptidase
UIJ67855.1	(d)cmp kinase
UIJ69429.1	Cytochrome c oxidase subunit i
UIJ67618.1	Anthranilate synthase component i
UIJ69455.1	Acetyltransferase
UIJ68505.1	Lema domain protein
UIJ69268.1	Duf3224 domain-containing protein
UIJ65395.1	Xre family transcriptional regulator
UIJ66509.1	Threonylcarbamoyl-amp synthase
UIJ66597.1	Ncs2 family permease
UIJ69895.1	Hypothetical protein lw858 31670 (plasmid)
UIJ64615.1	Imidazolonepropionase
UIJ66309.1	Trna lysidine(34) synthetase tils
UIJ67620.1	Anthranilate phosphoribosyltransferase
UIJ65186.1	Bifunctional homocysteine s-methyltransferase/methylenetetrahydrofolate reductase
UIJ68615.1	Atp-binding protein
UIJ64771.1	1-deoxy-d-xylulose-5-phosphate reductoisomerase
UIJ67836.1	Cytochrome c biogenesis protein resc
UIJ66496.1	Dna-directed rna polymerase subunit delta
UIJ67623.1	Tryptophan synthase subunit beta
UIJ69005.1	Hypothetical protein lw858 12670
